# Pharmacokinetics of Dalbavancin in Complicated *Staphylococcus aureus* Bacteremia: A Secondary Analysis of the DOTS Randomized Clinical Trial

**DOI:** 10.1001/jamanetworkopen.2026.11652

**Published:** 2026-04-18

**Authors:** Thomas P. Lodise, Nicholas A. Turner, Toshimitsu Hamasaki, Nick Fishbane, Varduhi Ghazaryan, Alison Wall, Lizhao Ge, Qihang Wu, Lijuan Zeng, Todd Riccobene, Rinal Patel, Smitha Zaharoff, Urania Rappo, Scott Evans, Vance G. Fowler, Henry F. Chambers, Thomas L. Holland

**Affiliations:** 1 Department of Pharmacy Practice, Albany College of Pharmacy and Health Sciences, Albany, New York; 2 Division of Infectious Diseases, Department of Medicine, Duke University School of Medicine, Duke University, Durham, North Carolina; 3 The Biostatistics Center, Milken Institute School of Public Health, George Washington University, Rockville, Maryland; 4 Department of Biostatistics and Bioinformatics, Milken Institute School of Public Health, George Washington University, Rockville, Maryland; 5 The Emmes Company, Rockville, Maryland; 6 Division of Microbiology and Infectious Diseases, National Institutes of Health, Bethesda, Maryland; 7 AbbVie, Florham Park, New Jersey; 8 Duke Clinical Research Institute, Durham, North Carolina; 9 Anti-Infectives, Internal Medicine, Allergan, Madison New Jersey; 10 Now with BiomX, Inc, Ness Ziona, Israel; 11 Division of Infectious Diseases, Department of Medicine, University of California, San Francisco

## Abstract

**Importance:**

Dalbavancin is an alternative to prolonged intravenous therapy for complicated *Staphylococcus aureus* bacteremia, yet its pharmacokinetics (PK) in patients with this condition are uncertain.

**Objective:**

To characterize dalbavancin PK and evaluate associations of total and unbound exposures of dalbavancin with clinical success in patients with complicated *S aureus* bacteremia.

**Design, Setting, and Participants:**

This study was an exploratory prespecified secondary analysis of PK and exposure response within Dalbavancin as an Option for Treatment of *S aureus* Bacteremia (DOTS), a multicenter, randomized, open-label, assessor-blinded clinical trial conducted from April 2021 to December 2023. Data analysis was conducted from January 2024 to December 2025. Adults with complicated *S aureus* bacteremia who achieved bloodstream clearance were randomized to dalbavancin or standard therapy; PK analyses included dalbavancin recipients with at least 1 postdose concentration measurement.

**Intervention:**

Dalbavancin 1500 mg intravenously on days 1 and 8 (1125 mg for patients with severe kidney impairment not receiving dialysis).

**Main Outcomes and Measures:**

Individual exposure metrics, including day 22 concentration and area under the concentration-time curve (AUC) from days 0 to 22, were derived using nonlinear mixed-effects population PK modeling. Exposure metrics were then assessed for association with clinical success at day 70 (exposure-response analysis).

**Results:**

A total of 97 patients (mean [SD] age, 54.5 [15.8] years; 69 male [71.1%]) contributing 640 PK samples were included. Clearance was estimated at 0.066 L/h (95% CI, 0.062 to 0.069 L/h), and the central volume of distribution was estimated at 5.67 L (95% CI, 5.37 to 5.99 L). Interindividual variability was 22.6% (95% CI, 18.9% to 25.6%) for clearance and 19.7% (95% CI, 13.8% to 25.0%) for the central volume of distribution. Clearance increased with creatinine clearance according to a power function (exponent, 0.21; 95% CI, 0.16 to 0.30). Distribution volumes increased with body weight following power relationships, including the central volume (exponent, 0.57; 95% CI, 0.37 to 0.86), second peripheral volume (0.82; 95% CI, 0.37 to 1.46), and third peripheral volume (0.56; 95% CI, 0.30 to 0.82). Albumin was inversely associated with the second peripheral volume (exponent, −0.81; 95% CI, −1.79 to −0.32) and unbound-fraction scaling factor (exponent, −0.78; 95% CI, −0.98 to −0.54), and age was positively associated with the third peripheral volume via a power relationship (exponent, 0.63; 95% CI, 0.44 to 0.83). Among 93 evaluable patients, 72 individuals (77.4%) achieved clinical success at day 70. Patients with a day 22 concentration greater than 32 μg/mL (30 patients [32.3% of evaluable patients receiving dalbavancin]) had higher clinical success compared with 63 patients with a day 22 concentration of 32 μg/mL or less (29 patients [96.7%] vs 43 patients [68.3%]; adjusted difference, 25.3 percentage points; 95% CI, 3.5-47.0 percentage points) and experienced similar rates of serious adverse events (8 patients [26.7%] vs 27 patients [42.9%]; unadjusted difference, −16.2 percentage points, 95% CI −36.2 to 3.8 percentage points).

**Conclusions and Relevance:**

In this study, dalbavancin pharmacokinetics were predictably influenced by kidney function, body weight, albumin levels, and age, and higher total day 22 concentrations were associated with greater clinical success without increased serious adverse events. These findings are exploratory and support further evaluation of exposure-guided dosing strategies.

**Trial Registration:**

Clinical Trials.gov Identifier: NCT04775953

## Key Points

**Question:**

What are the pharmacokinetics of a 2-dose dalbavancin regimen used for consolidation therapy in complicated *Staphylococcus aureus* bacteremia, and is dalbavancin exposure associated with clinical success?

**Findings:**

In this prespecified secondary analysis that included 97 adults with complicated *S. aureus* bacteremia from a randomized clinical trial, kidney function, weight, albumin level, and age influenced dalbavancin pharmacokinetics. Total day 22 concentrations greater than 32 μg/mL were associated with increased rates of day 70 clinical success, without corresponding increases in serious adverse events.

**Meaning:**

Although 2 doses of dalbavancin were noninferior to standard therapy in complicated *S. aureus* bacteremia in the parent trial, these findings suggest that future studies should assess whether patients with day 22 concentrations of 32 μg/mL or less may benefit from an additional dose.

## Teaser

This prespecified secondary analysis of a randomized clinical trial investigates the pharmacokinetics of a 2-dose dalbavancin regimen and its association with clinical success among patients with complicated *Staphylococcus aureus* bacteremia.

## Introduction

*Staphylococcus aureus* is a leading cause of bloodstream infection–related mortality worldwide.^1,2^ Conventional treatment for complicated *S aureus* bacteremia requires intravenous access for prolonged antibiotics, which risks thrombosis, secondary infection, and discomfort.^3^ Dalbavancin, a long-acting lipoglycopeptide active against methicillin-resistant *S aureus* (MRSA), offers a simplified alternative due to its 14-day terminal half-life.^4^ Although dalbavancin is approved for acute bacterial skin and skin structure infections, population pharmacokinetic analyses suggest that two 1500-mg doses 1 week apart provide sustained levels for 4 to 6 weeks, supporting its use as consolidation therapy for *S aureus* bacteremia.^5,6,7,8,9^ In the Dalbavancin as an Option for Treatment of *S aureus* Bacteremia (DOTS) randomized clinical trial, this dalbavancin dosing strategy was noninferior to standard therapy.^10^

The modeling that informed dalbavancin dose selection in DOTS was based on a pharmacokinetic and pharmacodynamic exposure target derived from a neutropenic murine thigh infection model, assuming 93% protein binding.^5,6^ The relevance of these targets to patients with complicated *S aureus* bacteremia remains uncertain.^7,11^ Direct characterization of dalbavancin PK in this population is needed to better understand drug disposition and its relationship to clinical outcomes.^12^ The objectives of this secondary analysis of the DOTS trial were to characterize the PK of total and unbound dalbavancin in adults with complicated *S aureus* bacteremia, identify patient factors associated with variability in dalbavancin disposition, and evaluate whether dalbavancin exposure was associated with clinical success.

## Methods

### Study Design and Participants

This is a prespecified secondary analysis of DOTS, a multicenter, open-label, assessor-blinded superiority randomized clinical trial (NCT04775953) (see trial protocol and statistical analysis plan in Supplement 1).^10,13^ A central institutional review board (WCG) approved the protocol with assent from each participating center’s local institutional review board. All participants provided written informed consent. This study is reported following the Consolidated Standards of Reporting Trials (CONSORT) reporting guideline. The trial evaluated the efficacy and safety of a 2-dose dalbavancin regimen compared with standard intravenous antibiotic therapy in adult patients with complicated *S aureus* bacteremia after bloodstream clearance. Eligible patients were randomly assigned in a 1:1 ratio to receive dalbavancin 1500 mg intravenously on day 1 and day 8 after randomization (with dose adjustment to 1125 mg for patients with creatinine clearance <30 mL/min who were not receiving dialysis) or standard therapy.^10^ This prespecified secondary PK analysis included participants randomized to dalbavancin who had at least 1 measurable postdose plasma concentration measurement. In this study, *PK analysis* refers to the development of a population pharmacokinetic model to characterize dalbavancin disposition and generate individual exposure estimates for subsequent exposure-response evaluation. Race and ethnicity were self-reported by participants within prespecified categories (race: American Indian or Alaska Native, Asian, Black or African-American, and White; ethnicity: Hispanic or Latino and not Hispanic or Latino). Race and ethnicity are reported to help physician readers assess whether the study population reflects the patient populations they care for.

### PK Sampling and Processing

Plasma samples for PK analysis were collected on study day 1 (collected before the dose, 10 minutes after infusion, then at 6, 12, and 24 hours after infusion), on study day 8 before the second dose, and at days 22, 42, and 70. Total and unbound dalbavancin concentrations were determined centrally using a validated liquid chromatography–tandem mass spectrometry assay, with lower limits of quantification of 0.5 μg/mL and 0.05 μg/mL for total and unbound concentrations, respectively.^12^

### Population PK Modeling

A population PK modeling approach was used to describe dalbavancin disposition and estimate individual exposure metrics for exposure-response analyses. Nonlinear mixed-effects modeling was applied to characterize total and unbound dalbavancin concentration-time data (eAppendix in Supplement 2).

The structural (fixed-effects) component of the model described typical population PK parameters, including central clearance (CL), central volume of distribution (V_1_), peripheral volumes (V_2_ and V_3_), and intercompartmental clearances, using a 3-compartment model with zero-order input and first-order elimination.^6^ Total and unbound concentrations were linked through a structural relationship describing the fraction unbound (fu). In the base model, fu was assumed constant; alternative models allowing concentration-dependent binding were evaluated to assess potential nonlinearity in protein binding.

The random-effects component accounted for variability across individuals. Between-individual variability in pharmacokinetic parameters (eg, CL and distribution volumes) was modeled exponentially, assuming log-normal parameter distributions. Residual unexplained variability representing within-individual error and assay variability was evaluated using proportional and combined error models. Below–quantification-limit observations were handled using the M3 likelihood–based approach.^14^ The model is termed nonlinear because (1) concentration-time profiles are governed by differential equations describing multicompartment disposition and (2) parameters enter the model multiplicatively through exponential random-effects terms and, where applicable, nonlinear relationships between total and unbound concentrations.

Covariate model development began with exploratory analyses to identify predictors of CL, volumes, and fu, followed by forward inclusion and backward elimination to derive the final model. Model performance was assessed using diagnostic plots, prediction-corrected visual predictive checks,^15^ bootstrap resampling, η shrinkage, and condition numbers. Akaike information criterion (AIC) was used to formally compare competing models by balancing goodness of fit with model complexity, thereby reducing the risk of overfitting when evaluating additional compartments or nonlinear binding structures.

Individual empirical Bayes estimates were obtained from the final model and used to derive exposure metrics, including day 22 concentration in micrograms per milliliter and area under the concentration-time curve (AUC) from day 0 to day 22 in micrograms-hours per milliliter. Exposures were simulated through day 22 to coincide with the prespecified sampling window. Given dalbavancin’s linear pharmacokinetics, later exposures were assumed to be proportional to earlier values, so distal time windows were not analyzed.

### Exposure-Response Analysis

Exposure-response analysis assessed associations of total and unbound exposure metrics with clinical success and safety at day 70 after randomization among participants who were alive and free of infectious complications through day 22. This was done to identify potential failures from suboptimal exposure. Clinical success, adjudicated by a committee blinded to treatment assignment and PK data, was defined as the absence of clinical failure, infectious complications, and all-cause mortality. Indeterminate clinical efficacy (due to withdrawal, loss to follow-up, or insufficient data) was classified as clinical failure (worst-case imputation). Safety was defined as serious adverse events or adverse events leading to study drug discontinuation.

Each exposure was assessed as a continuous variable and in evenly distributed tertiles, ensuring at least 30 participants per category for stable model estimation. Exploratory dichotomization was used to identify exposure thresholds associated with clinical success at day 70. At each candidate dichotomization threshold, univariate log-binomial generalized linear models estimated effect sizes for clinical success, with 95% CIs and AIC values recorded.^16^ Thresholds were ranked by larger effect size, narrower CIs, and lower AIC. Top-ranked thresholds were used for subsequent exposure-response analyses.

### Statistical Analysis

Bivariate analyses were first conducted to evaluate associations of total and unbound exposures with clinical success at day 70, with exposures expressed as continuous, categorical, and dichotomization-derived binary variables. Safety analyses were limited to dichotomization-derived exposure variables. Fisher exact tests compared categorical and dichotomization-derived exposure variables across outcome groups, and 95% CIs for tertile-based analyses were calculated with the Wilson score method. Mean total and unbound exposure metrics were compared between outcome groups using 2-sample *t* tests. Stratified analyses assessed effect modification by duration of bacteremia (<2 vs ≥2 days), presence of deep-seated infection (yes vs no), and strain type (MRSA vs methicillin-susceptible *S aureus*) for dichotomization-derived total and unbound exposure variables. Multivariable models were then constructed, adjusting for selected covariates; generalized linear models with a binomial distribution were used to estimate differences in proportions of patients with clinical success. Candidate covariates included strain type, age, sex, weight, race, creatinine clearance,^17^ albumin level, medical history, whether participants were persons who inject drugs, underlying infection site, dialysis, immunosuppression, prerandomization antibiotic use, and duration of bacteremia. Covariates with bivariate associations at *P* < .10 were considered, with strain type forced at entry. Collinearity was assessed, and when it was present, only the covariate with the stronger association was retained. Final results are reported as adjusted and unadjusted differences with 95% CIs.

Inverse probability weighting (IPW) and tipping point analysis were performed post hoc to evaluate the robustness of findings under alternative assumptions regarding missing efficacy outcomes at day 70.^18^ In sequential tipping point analyses, an increasing number of indeterminate cases were reclassified as clinical successes. IPW was applied to estimate the probability of clinical success for participants with missing outcomes based on age, body mass index (calculated as weight in kilograms divided by height in meters squared), and status as persons who inject drugs. Data were analyzed from January 2024 to December 2025. All tests were 2-sided. All CIs are 2-sided, with a 95% confidence level. The software used for PK work was NONMEM version 7.3 (ICON Development Solutions) and Pumas version 2.5.1 (PumasAI, Inc).

## Results

### Study Population and PK Sampling Modeling

Of 100 patients randomized to dalbavancin, 97 patients (mean [SD] age, 54.5 [15.8] years; 69 male [71.1%]; 5 Asian [5.2%], 1 American Indian or Alaska Native [1.0%], 20 Black or African American [20.6%], and 67 White [69.1%]; 11 Hispanic or Latino [11.3%]) contributed at least 1 measurable postdose plasma concentration and were included in the PK population (eTable 1 in Supplement 3). Across all participants, 640 PK samples were available (median [range], 6 [1-8] samples per patient). A total of 11 patients received modified regimens, including 3 patients receiving 1125 mg on both days, 3 patients receiving 1125 mg then 1500 mg, 2 patients receiving 1500 mg then 1125 mg, and 3 patients receiving a single 1500-mg dose. The population had a median (range) weight of 84 (49-152) kg. The median (range) baseline creatinine clearance was 101 (4-372) mL/min, with 12 patients (12.4%) receiving hemodialysis (eTable 1 in Supplement 3).

### Observed Concentration-Time Data

Total and unbound dalbavancin concentration-time profiles by dosing group are shown in eFigure 1 in Supplement 3. Across all detectable pairs of total and unbound PK plasma samples, 408 of 442 samples (92.3%) had an unbound fraction of less than 1%, reflecting extensive protein binding,^12^ with a slight increase in free fraction at higher concentrations (eFigure 2 in Supplement 3). Exploratory analyses demonstrated a modest inverse relationship between albumin concentration and the fu, whereas no systematic relationship was observed with creatinine clearance (eFigures 3 and 4 in Supplement 3). By day 42, most unbound samples were below quantification limits (54 of 74 samples [73.0%] at day 42 and 51 of 52 samples [98.1]% at day 70) (eFigure 5 in Supplement 3). Total concentrations remained measurable in all postdose samples.

### Population PK Model

We estimated CL at 0.066 L/h (95% CI, 0.062 to 0.069 L) and V_1_ at 5.67 L (95% CI, 5.37 to 5.99 L). Interindividual variability was 22.6% (95% CI, 18.9% to 25.6%) for CL and 19.7% (95% CI, 13.8% to 25.0%) for V_1_. A 3-compartment model with zero-order input and first-order elimination best described total dalbavancin concentrations and outperformed simpler structures by AIC, diagnostic plots, and visual predictive checks (eAppendix in Supplement 2). Total and unbound concentrations were linked by a power function, C_U_(t) = A × C_T_(t)^K^, where A (ug/mL) scales the magnitude of the unbound concentration when total concentration equals 1 and where K (unitless) governs the curvature of the relationship. Covariate analyses identified clinically plausible effects: higher creatinine clearance was associated with increased clearance according to a power function (exponent, 0.21; 95% CI, 0.16 to 0.30). Distribution volumes increased with body weight following power relationships, including V1 (central compartment; exponent, 0.57; 95% CI, 0.37 to 0.86), V2 (second peripheral compartment; exponent, 0.82; 95% CI, 0.37 to 1.46) and V3 (third peripheral compartment; exponent, 0.56; 95% CI, 0.30 to 0.82). Albumin was inversely associated with V2 (exponent, −0.81; 95% CI, −1.79 to −0.32) and the unbound scaling factor (exponent, −0.78; 95% CI, −0.98 to −0.54). Age was positively associated with V3 via a power relationship (exponent, 0.63; 95% CI, 0.44 to 0.83). Model performance was robust; observed vs predicted plots for population and individual fits showed no systematic bias, while weighted residuals were approximately normal without time- or concentration-dependent trends. Prediction-corrected visual predictive checks captured the 5th, 50th, and 95th percentiles of observed concentrations; and nonparametric bootstrap resampling (1000 replicates) confirmed parameter stability.

### Individual Exposure Estimates

Exposure metrics from the population PK model (eg, day 22 concentration and AUC for days 0-22) were subsequently used as independent variables to evaluate associations with clinical outcomes (exposure-response analysis). Using empirical Bayes estimates derived from the final population PK model, individual exposure metrics were calculated for each participant. Among patients receiving two 1500-mg doses, the total mean (SD) day 22 concentration was 29.0 (10.6) μg/mL and total mean (SD) AUC for days 0 to 22 was 32 593 (7198) μg * h/mL. Unbound exposure metrics closely paralleled total exposures but demonstrated slightly greater relative variability (eAppendix in Supplement 2).

### Exposure-Response Analyses

Of 97 patients in the PK population, 93 patients (mean [SD], age, 54.1 [15.9] years; 66 men [71.0%]; 5 Asian [5.4%], 1 American Indian or Alaska Native [1.1%], 20 Black or African American [21.5%], and 64 White [68.8%]; 9 Hispanic or Latino [9.7%]) were evaluable through day 70 (Table 1). A total of 4 patients were unevaluable due to death or withdrawal before day 22 (3 deaths within 15 days of enrollment and 1 voluntary withdrawal). Among 93 evaluable patients, 72 individuals (77.4%) achieved clinical success at day 70. Baseline characteristics by clinical outcome at day 70 are summarized in Table 1.

**Table 1.** Baseline Characteristics by Clinical Outcome at Day 70

Baseline characteristic	Patients, No. (%)			SMD
	Overall (N = 93)	Clinical success (n = 72)	Clinical failure (n = 21)^a^	
Age, mean (SD), y	54.1 (15.9)	55.2 (15.7)	50.3 (16.2)	0.30
Sex				
Male	66 (71.0)	51 (70.8)	15 (71.4)	0.01
Female	27 (29.0)	21 (29.2)	6 (28.6)	0.01
Race				
Asian	5 (5.4)	4 (5.6)	1 (4.8)	0.04
American Indian or Alaska Native	1 (1.1)	1 (1.4)	0	0.17
Black or African American	20 (21.5)	15 (20.8)	5 (23.8)	0.07
White	64 (68.8)	49 (68.1)	15 (71.4)	0.07
Unknown	3 (3.2)	3 (4.2)	0	0.29
Ethnicity				
Hispanic or Latino	9 (9.7)	9 (12.5)	0	0.53
Not Hispanic or Latino	81 (87.1)	61 (84.7)	20 (95.2)	0.36
Not reported	2 (2.2)	1 (1.4)	1 (4.8)	0.20
Unknown	1 (1.1)	1 (1.4)	0	0.17
Weight, mean (SD), kg	88.1 (22.0)	89.5 (22.5)	83.3 (19.7)	0.30
BMI, mean (SD)	29.2 (6.8)	29.7 (7.1)	27.6 (5.6)	0.33
Creatinine clearance, mean (SD), mL/min	115.9 (70.7)	118.4 (73.0)	107.3 (63.1)	0.16
Albumin, mean (SD), g/dL	2.8 (0.6)	2.8 (0.6)	2.7 (0.7)	0.14
Qualifying pathogen				
MRSA	32 (34.4)	28 (38.9)	4 (19.0)	0.45
MSSA	61 (65.6)	44 (61.1)	17 (81.0)	0.45
Underlying site of infection				
Endovascular	28 (30.1)	21 (29.2)	7 (33.3)	0.09
Bone and joint	25 (26.9)	16 (22.2)	9 (42.9)	0.45
Skin	37 (39.8)	30 (41.7)	7 (33.3)	0.17
Pulmonary	8 (8.6)	7 (9.7)	1 (4.8)	0.19
Other or unknown	21 (22.6)	16 (22.2)	5 (23.8)	0.04
Persons who inject drugs status	14 (15.1)	8 (11.1)	6 (28.6)	0.45
Immunosuppression	33 (35.5)	25 (34.7)	8 (38.1)	0.07
Received dialysis	10 (10.8)	7 (9.7)	3 (14.3)	0.14
Prerandomization antibiotic				
β-Lactam	89 (95.7)	68 (94.4)	21 (100)	0.34
Vancomycin	78 (83.9)	62 (86.1)	16 (76.2)	0.26
Daptomycin	14 (15.1)	14 (19.4)	0	0.69
Other	13 (14.0)	13 (18.1)	0	0.66
Medical history				
Heart failure	21 (22.6)	15 (20.8)	6 (28.6)	0.18
Chronic kidney disease	18 (19.4)	14 (19.4)	4 (19.0)	0.01
Diabetes	41 (44.1)	32 (44.4)	9 (42.9)	0.03
Liver disease	11 (11.8)	6 (8.3)	5 (23.8)	0.43
Cancer	19 (20.4)	17 (23.6)	2 (9.5)	0.39
Duration of initial bacteremia, d				
<2	70 (75.3)	56 (77.8)	14 (66.7)	0.25
≥2	23 (24.7)	16 (22.2)	7 (33.3)	0.25

Abbreviations: BMI, body mass index (calculated as weight in kilograms divided by height in meters squared); MRSA, methicillin resistant *Staphylococcus aureus*; MSSA, methicillin susceptible *Staphylococcus aureus*; SMD, standardized mean difference.

SI conversion factor: To convert albumin to grams per liter, multiply by 10.0.

^a^ By day 70, 21 participants were classified as having clinical failures: 1 experienced all 3 outcomes, including death; 4 had both lack of clinical efficacy and an infectious complication; and 16 had a single event (7 infectious complications and 9 lack of clinical efficacy). All infectious complications occurred on or after day 40.

**Figure 1.**
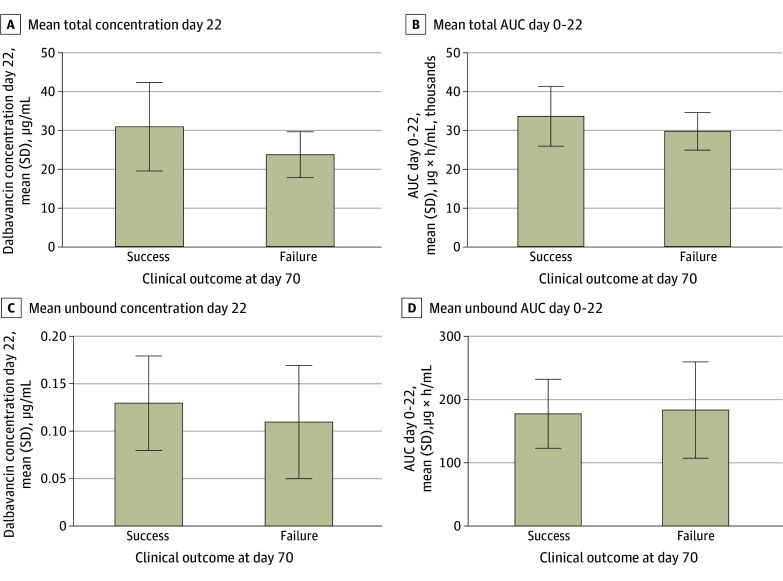
Association of Total and Unbound Dalbavancin Exposure Metrics With Clinical Success at Day 70

**Figure 2.**
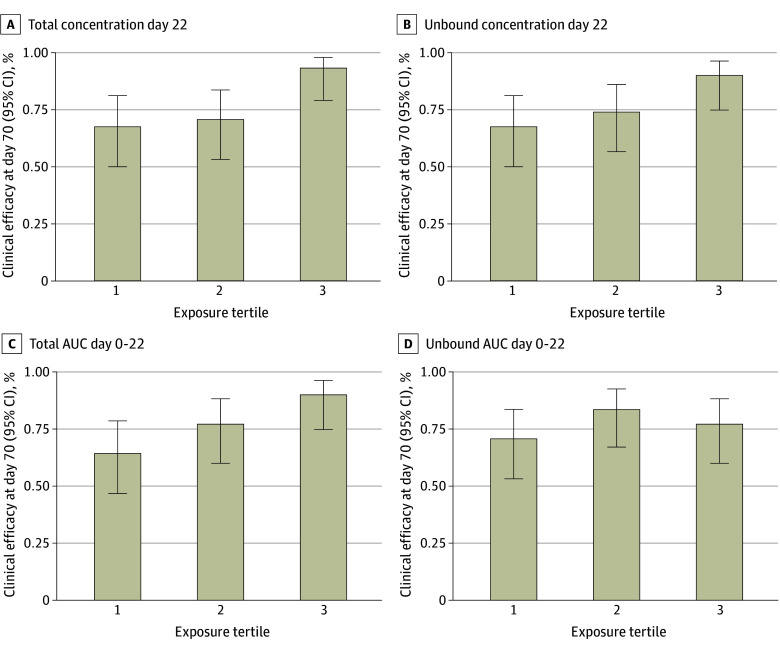
Association of Tertiles of Total and Unbound Dalbavancin Exposure With Clinical Success at Day 70 Tertiles of dalbavancin exposure were defined for total day 22 concentration (tertile 1: <23.7 μg/mL; tertile 2: 23.7 to <32 μg/mL; and tertile 3: 32 to <61.5 μg/mL), unbound day 22 concentration (tertile 1: <0.09 μg/mL; tertile 2: 0.09 to <0.138 μg/mL; and tertile 3: 0.138 to <0.301 μg/mL), total AUC for days 0 to 22 (tertile 1: <28 806.3 μg * h/mL; tertile 2: 28 806.3 to <35 183.8 μg * h/mL; and tertile 3: 35 183.8 to <51 488.9 μg * h/mL), and unbound AUC for days 0 to 22 (tertile 1: <146.2 μg * h/mL; tertile 2: 146.2 to <192.6 μg * h/mL; and tertile 3: 192.6 to <342.8 μg * h/mL).

Mean (SD) total dalbavancin exposures were consistently higher among patients with clinical success at day 70, whereas the pattern was less pronounced for unbound exposures (Figure 1). Associations across tertile categories are displayed in Figure 2. Patients in the highest total exposure tertile had higher clinical success at day 70 than those in lower tertiles, with similar but attenuated trends for unbound exposures. Dichotomization analyses identified exposure thresholds associated with improved clinical success (Table 2). For total concentration at day 22, the optimal cut point was 32 μg/mL. The 30 patients with a total day 22 concentration greater than 32 μg/mL (32.3% of evaluable patients) had a higher clinical success at day 70 than 63 patients with day 22 concentrations of 32 μg/mL or less (29 patients [96.7%] vs 43 patients [68.3%]). In adjusted generalized linear models, the adjusted difference was 25.3 percentage points (95% CI, 3.5 to 47.0 percentage points), with a similar association at day 42 (eTable 2 in Supplement 3). For total AUC day 0 to 22, a threshold of 37 414 μg * h/mL best distinguished higher from lower clinical success rates, with patients who had values above this cut point achieving a higher success rate, although the difference was not significant (adjusted difference, 20.8 percentage points; 95% CI, −2.9 to 44.5 percentage points). Most other dichotomized total and unbound AUC measures had higher success at day 70, although differences were nonsignficant (Table 2). There was no evidence of effect modification across prespecified subgroups of interest (Table 3). Among 3 patients without PK data, 1 discontinued on day 8, 1 experienced clinical failure at day 70, and 1 experienced clinical success at both points. Post hoc estimates based on typical PK parameters and covariates suggested that the participant with clinical failure had an estimated day 22 concentration of 32 μg/mL or less while the participant with clinical success had an estimated day 22 concentration greater than 32 μg/mL, consistent with primary exposure-efficacy relationships. No differences in day 70 safety end points were observed between dichotomization-derived exposure variables, with similar rates of serious adverse events in patients with a day 22 concentration greater than 32 μg/mL (8 patients [26.7%]) vs those with 32 μg/mL or less (27 patients [42.9%]; unadjusted difference, −16.2 percentage points; 95% CI, −36.2 to 3.8 percentage points) (eTable 2 in Supplement 3). Similar trends were seen for day 42 outcomes (eFigures 6-7 and eTables 3-5 in Supplement 3).

**Table 2.** Association of Total and Unbound Dichotomization Exposure Metrics With Clinical Efficacy at Day 70

Dichotomization exposure measure^a^	Patients, No.	Clinical efficacy at day 70, No. (%)	Difference (95% CI), percentage points	
			Unadjusted	Adjusted^b^
Day 22 concentration dichotomization point, μg/mL				
Total				
≤32.0	63	43 (68.3)	28.4 (15.2 to 41.6)	25.3 (3.5 to 47.0)
>32.0	30	29 (96.7)		
Unbound				
≤0.137	61	43 (70.5)	20.1 (4.9 to 35.4)	16.9 (0.4 to 33.4)
>0.137	32	29 (90.6)		
AUC day 0-22 dichotomization point, μg × h/mL				
Total				
≤37 414	71	51 (71.8)	23.6 (10.0 to 37.2)	20.8 (−2.9 to 44.5)
>37 414	22	21 (95.5)		
Unbound				
≤174	47	34 (72.3)	10.3 (−6.6 to 27.1)	10.3 (−5.5 to 26.0)
>174	46	38 (82.6)		

Abbreviation: AUC, area under the concentration–time curve.

^a^ Dichotomization is of exposure metrics into groups above and below specified dichotomization points.

^b^ Adjusted models included baseline *Staphylococcus aureus* strain type (forced in all models). Additional candidate covariates evaluated for the day 70 clinical efficacy model included primary site of infection (bone and joint), persons who inject drugs status, prerandomization daptomycin use, and other prerandomization antibiotic use. Primary site of infection, persons who inject drugs status, and daptomycin use were removed due to collinearity with strain type.

**Table 3.** Clinical Efficacy at Day 70 Across Dichotomized Dalbavancin Exposure Metrics by Clinical Subgroup

Dichotomization exposure metric^a^	Clinical efficacy at day 70, No./No. (% )		Difference (95% CI), percentage points
	Below	Above	
Total day 22 concentration (32 μg/mL)			
Bacteremia ≤2 d	34/48 (70.8)	22/22 (100)	29.2 (16.3 to 42.0)
Bacteremia >2 d	9/15 (60.0)	7/8 (87.5)	27.5 (−6.3 to 61.3)
No deep-seated infection^b^	18/24 (75.0)	18/19 (94.7)	19.7 (−0.3 to 39.8)
Deep-seated infection^b^	25/39 (64.1)	11/11 (100)	35.9 (20.8 to 51.0)
MSSA	27/43 (62.8)	17/18 (94.4)	31.7 (13.8 to 49.6)
MRSA	16/20 (80.0)	12/12 (100)	20.0 (2.5 to 37.5)
Unbound day 22 concentration (0.137 μg/mL)			
Bacteremia ≤2 d	34/47 (72.3)	22/23 (95.7)	23.3 (8.1 to 38.6)
Bacteremia >2 d	9/14 (64.3)	7/9 (77.8)	13.5 (−23.5 to 50.5)
No deep-seated infection^b^	19/25 (76.0)	17/18 (94.4)	18.4 (−1.4 to 38.3)
Deep-seated infection^b^	24/36 (66.7)	12/14 (85.7)	19.1 (−4.5 to 43.0)
MSSA	29/44 (65.9)	15/17 (88.2)	22.3 (1.6 to 43.1)
MRSA	14/17 (82.4)	14/15 (93.3)	11.0 (−11.1 to 33.1)
Total AUC day 0-22 (37 414 μg * h/mL)			
Bacteremia ≤2 d	40/53 (75.5)	16/17 (94.1)	18.7 (2.5 to 34.8)
Bacteremia >2 d	11/18 (61.1)	5/5 (100)	38.9 (16.4 to 61.4)
No deep-seated infection^b^	25/32 (78.1)	11/11 (100)	21.9 (7.6 to 36.2)
Deep-seated infection^b^	26/39 (66.7)	10/11 (90.9)	24.2 (1.7 to 46.8)
MSSA	32/48 (66.7)	12/13 (92.3)	25.6 (6.0 to 45.3)
MRSA	19/23 (82.6)	9/9 (100)	17.4 (1.9 to 32.9)
Unbound AUC day 0-22 (37 414 μg * h/mL)			
Bacteremia ≤2 d	27/37 (73.0)	29/34 (85.3)	10.3 (−8.2 to 28.8)
Bacteremia >2 d	7/11 (63.6)	9/12 (75.0)	11.4 (−26.2 to 48.9)
No deep-seated infection^b^	18/22 (81.8)	18/21 (85.7)	3.9 (−18.1 to 25.9)
Deep-seated infection^b^	16/25 (64.0)	20/25 (80.0)	16 (−8.5 to 40.5)
MSSA	24/34 (70.6)	20/27 (74.1)	3.5 (−19.1 to 26.0)
MRSA	10/13 (76.9)	18/19 (94.7)	17.8 (−7.2 to 42.8)

Abbreviations: AUC, area under the concentration–time curve; MRSA, methicillin-resistant *Staphylococcus aureus*; MSSA, methicillin-susceptible *Staphylococcus aureus*.

^a^ Dichotomization is of exposure metrics into groups above and below specified dichotomization points.

^b^ Deep-seated infection was defined as the presence of at least 1 of the following: endocarditis, osteoarticular infection, cardiac device infection, septic thrombophlebitis, or a deep noncutaneous abscess.

A total of 9 participants with indeterminate day 70 efficacy outcomes were classified as having clinical failure; all 9 had total day 22 concentrations of 32 μg/mL or less. Baseline characteristics among patients with nonmissing vs missing day 70 clinical efficacy data are shown in eTable 6 in Supplement 3. Across tipping point analyses, in which 1 through 9 indeterminate cases were sequentially reclassified as successes, the direction and magnitude of exposure-response relationships remained consistent with primary findings (eFigure 8 in Supplement 3). Changes in absolute risk differences were minimal, and overall inferences were unchanged across all assumptions. Complementary IPW analyses estimating the probability of success for participants with missing outcomes produced results nearly identical to those of complete-case analyses, further supporting the robustness of the findings (eTable 7 in Supplement 3).

## Discussion

In this exploratory prespecified dalbavancin PK secondary analysis of the DOTS randomized clinical trial, 2 key observations emerged.^10^ First, dalbavancin protein binding was considerably higher than previously estimated.^19^ Second, higher total exposures, particularly day 22 concentrations greater than 32 μg/mL, were associated with greater clinical success without an observed increase in toxic effects. This suggests that some patients may benefit from a third dose, either empirically or guided by therapeutic drug monitoring.

Dalbavancin PK results in DOTS were broadly consistent with those of prior studies,^6,20,21^ but the paired measurement of total and unbound concentrations revealed substantially higher protein binding (>99%) than previously appreciated.^19^ Unbound concentrations were quantified using ultracentrifugation with Tween-80 presaturated plasticware to minimize nonspecific adsorption,^22^ thereby avoiding limitations of radiolabeled equilibrium dialysis, including membrane binding artifacts.^23,24^ Because radiolabeled assays cannot reliably quantify protein binding beyond the radiochemical purity of the labeled compound, typically approximately 97% to 99%, they may underestimate binding for highly protein-bound drugs, such as dalbavancin.^23,24^ Using our approach, protein binding exceeded 99%, consistent with prior analytical work.^12,25,26^

Across the observed concentration range, fu remained low, with only modest nonlinear behavior at higher total concentrations. Albumin concentration, rather than kidney function, was the primary determinant of variability in fu.^27,28^ Importantly, hypoalbuminemia was common in the DOTS cohort, allowing protein binding to be evaluated across a clinically relevant range of albumin concentrations. While hypoalbuminemia can increase fu and lead to higher CL for some drugs,^27^ this was not observed in our study. This likely reflects dalbavancin’s very high protein binding and the modest effect of albumin on fu. Kidney impairment influenced clearance but did not meaningfully alter fu. The near-parallel decline of total and unbound plasma concentrations supports rapid equilibrium between bound and unbound drug, consistent with established pharmacologic principles.^29,30^ Unbound concentrations declined rapidly, with 73.0% of participants exhibiting unbound concentrations below quantification by day 42. Notably, infectious complications occurred after day 40, temporally corresponding to declining unbound exposure; however, causality cannot be inferred.

Exploratory exposure-response analyses demonstrated a consistent association between dalbavancin plasma concentrations and clinical success. Patients with higher total exposures experienced substantially higher rates of clinical success, whereas associations with unbound exposure metrics were comparatively attenuated. Notably, total day 22 concentration greater than 32 μg/mL was associated with clinical success rates exceeding 96%, compared with approximately 68% among patients with values below the threshold, without evidence of increased toxic effects. These trends were consistent across strain type, prior duration of bacteremia, and presence of deep-seated infection. Tipping point and IPW analyses addressing missing outcomes yielded results nearly identical to those of primary models, supporting the robustness of the findings. Although baseline kidney function and albumin levels appeared similar between outcome groups, variability in exposure can arise from extremes of creatinine clearance and body weight, as well as residual interpatient variability in clearance and distribution.^31^ Because exposure was not randomized, the lower concentrations observed among treatment failures likely reflect a combination of these covariate effects and inherent between-individual pharmacokinetic variability.^31^

Preclinical pharmacokinetic and pharmacodynamic targets for concentration-dependent drugs like dalbavancin are often expressed in terms of the AUC.^5,6,31,32^ In this cohort, however, AUC at 0 to 22 days and day 22 concentration were highly correlated, reflecting the prolonged half-life of dalbavancin. Whereas AUC at 0 to 22 days reflects cumulative early exposure, day 22 concentration represents the persistence of systemic concentrations at the end of the dosing interval. Given dalbavancin’s long half-life^6^ and the observation that infectious complications occurred after day 40, late exposure may be particularly relevant for preventing delayed relapse. In this context, day 22 concentration serves as a clinically intuitive marker of exposure durability during the period most relevant to late complications. In contrast, earlier times primarily reflect early exposure and would address a different mechanism than sustained antimicrobial coverage.

Unbound drug is generally considered pharmacologically active,^30,31^ yet total and unbound concentrations were strongly correlated in this population because protein binding was near constant and exceeded 99% in 92.3% of detectable pairs of total and unbound PK plasma samples. Consequently, the measurable dynamic range of unbound concentrations was narrow, and many later samples approached the lower limit of quantification, which may attenuate exposure-response discrimination. Exposure thresholds identified were empirically derived from clinical outcome modeling rather than prespecified microbiologic break points.^33,34^ Taken together, these findings support the use of total day 22 concentration as a practical and stable surrogate for sustained systemic exposure in this population.

From a clinical perspective, the parent DOTS trial demonstrated noninferiority of the 2-dose dalbavancin regimen vs standard therapy for completion of treatment in complicated *S aureus* bacteremia, supporting the use of the 2-dose regimen in practice.^10^ Even patients with dalbavancin levels below the day 22 concentration threshold experienced success rates numerically comparable to those of standard therapy. However, exploratory exposure-response analyses suggest that some patients may benefit from more sustained exposure. Day 22 was evaluated because it reflects sustained exposure after completion of the 2-dose regimen and coincided with the prespecified pharmacokinetic assessment window. Importantly, the 32 μg/mL threshold identified in this study requires external validation before clinical application.^35^ These findings should be viewed as hypothesis generating and may inform future studies evaluating alternative dalbavancin dosing strategies. For example, a supplemental dose administered between days 22 and 40, guided by low day 22 concentrations or applied empirically in patients at risk for lower exposure, could be prospectively evaluated.^35,36^

### Limitations

This study has several limitations. The population is not representative of all patients with *S aureus* bacteremia. Participants were enrolled after bloodstream clearance and clinical stabilization. Accordingly, overall mortality was low and relatively few patients had advanced chronic kidney disease or severe hypoalbuminemia, limiting generalizability to these populations.

These were exploratory, retrospective analyses of prospectively collected PK data evaluating only 1 renally adjusted dosing regimen, without randomization to exposure strata, so residual confounding cannot be excluded. Analyses were limited to patients with PK data who remained outcome free through day 21 to focus on late failures potentially related to suboptimal exposure. Threshold selection using dichotomization can yield spurious cut points, and multiple comparisons increase the risk of type I error.^37,38^ Nonetheless, higher continuous and categorical exposure metrics were associated with clinical success, and dichotomization thresholds identified overlapping patient sets, as expected for a drug with linear pharmacokinetics. Consistent effects across analytic approaches support a plausible exposure-response relationship. A total of 9 participants with indeterminate efficacy outcomes (classified as having failures) had total day 22 concentrations below the dichotomization threshold, which could have introduced bias. However, tipping point and IPW analyses produced minimal changes in absolute risk differences, supporting the robustness of the findings.

## Conclusions

In a field that increasingly favors abbreviated therapy for acute infections, findings in this secondary analysis of a randomized clinical trial highlight that treatment of *S aureus* bloodstream infections may require a tailored approach, with sustained therapeutic concentrations beyond bacteremia clearance and source control to optimize outcomes. Key priorities moving forward are to define which patients with *S aureus* bacteremia may benefit from additional dalbavancin doses, determine the optimal dosing window, and validate dose-intensified or therapeutic drug monitoring–guided strategies in rigorously controlled trials.

## Notes

Supplement 1.
Trial Protocol and SAP

Supplement 2.
eAppendix.

Supplement 3. eFigure 1. Total and Unbound Plasma Dalbavancin Concentration-Time Profiles by Dosage Group eFigure 2: Association Between Observed Total and Unbound Dalbavancin Concentrations eFigure 3. Associations of Serum Albumin and Total Dalbavancin Concentration With Fraction Unbound eFigure 4. Association Between Creatinine Clearance and Fraction Unbound of Dalbavancin eFigure 5. Distribution of Observed Total and Unbound Dalbavancin Concentrations by Unbound Quantification Status eFigure 6: Association Between Total and Unbound Mean (SD) Exposure Metrics and Clinical Success at Day 42 eFigure 7. Association Between Tertiles of Total and Unbound Dalbavancin Exposure and Clinical Success at Day 42 eFigure 8. Unadjusted Risk Differences for Clinical Success Across Assumptions for Missing Day 70 Outcomes Using the Total Day 22 Concentration Cut Point eTable 1. Baseline Characteristics of Pharmacokinetic (PK) Analysis Population eTable 2. Day 70 Safety Outcomes by Exposure Groups Defined by Total and Unbound Dalbavancin Concentrations eTable 3: Baseline Characteristics by Clinical Outcome at Day 42 (Exposure-Efficacy Population) eTable 4. Association Between Total and Unbound Dichotomization Exposure Metrics and Clinical Efficacy at Day 42 eTable 5. Clinical Efficacy at Day 42 Across Exposure Thresholds by Clinical Subgroups eTable 6. Comparison of Baseline Characteristics Among Participants With Nonmissing vs Missing Day 70 Clinical Efficacy Data eTable 7. Exposure-Outcome Differences With Inverse Probability Weighting to Address Missing Outcome Data

Supplement 4. **Nonauthor Collaborators.** Antibacterial Resistance Leadership Group

Supplement 5.
Data Sharing Statement.
