# Error in Figure Label

**DOI:** 10.1001/jamanetworkopen.2026.18518

**Published:** 2026-05-21

In the Original Investigation titled “Pharmacokinetics of Dalbavancin in Complicated *Staphylococcus aureus* Bacteremia: A Secondary Analysis of the DOTS Randomized Clinical Trial,”^1^ published April 18, 2026, the y-axis labels in Figure 1, panel C were missing a decimal place; they should have been 0 to 0.20 μ/mL instead of 0 to 20 μ/mL. The article has been corrected.^1^
