# Supplementary material for: Pharmacokinetics of Dalbavancin in Complicated Staphylococcus aureus Bacteremia: A Secondary Analysis of the DOTS Randomized Clinical Trial
Source: JAMA Netw Open. 2026 Apr 18;9(4):e2611652. doi: 10.1001/jamanetworkopen.2026.11652 (PMC13092111; doi:10.1001/jamanetworkopen.2026.11652)
Supplement: Supplement 1. — Trial Protocol and SAP [file jamanetwopen-e2611652-s001.pdf]

Dalbavancin as an Option for Treatment of *S. aureus* Bacteremia (DOTS): A Phase 2b, Multicenter, Randomized, Open-Label, Assessor-Blinded Superiority Study to Compare the Efficacy and Safety of Dalbavancin to Standard of Care Antibiotic Therapy for the Completion of Treatment of Patients with Complicated *S. aureus* Bacteremia

DMID Protocol Number: 20-0002

DMID Funding Mechanism: UM1AI104681

Pharmaceutical Support: Allergan, a subsidiary of AbbVie

IND Sponsor: National Institutes of Health (NIH)/National Institute of Allergy and Infectious Diseases (NIAID)/Division of Microbiology and Infectious Diseases (DMID)

Lead Principal Investigator: Thomas L. Holland, M.D.

DMID Clinical Project Manager: [redacted]

Draft or Version Number: v1.0

6 August 2020

STATEMENT OF ASSURANCE

Each Institution will hold a current Federal Wide Assurance (FWA) issued by the Office of Human Research Protections (OHRP) for federally-funded human subjects research. Each FWA will designate at least one Institutional Review Board (IRB)/Independent Ethics Committee (IEC) registered with OHRP, for which the research will be reviewed and approved by the IRB/IEC and will be subject to continuing review [45 CFR 46.103(b)]. The IRB/IEC designated under an FWA may include an institution's IRB/IEC, an independent IRB/IEC, or an IRB/IEC of another institution after establishing a written agreement with that other institution.

STATEMENT OF COMPLIANCE

The study trial will be carried out in accordance with Good Clinical Practice (GCP) and as required by the following:

- United States Code of Federal Regulations (CFR) 45 CFR Part 46: Protection of Human Subjects
- Food and Drug Administration (FDA) Regulations, as applicable: 21 CFR Part 50 (Protection of Human Subjects), 21 CFR Part 54 (Financial Disclosure by Clinical Investigators), 21 CFR Part 56 (Institutional Review Boards), 21 CFR Part 11, and 21 CFR Part 312 (Investigational New Drug Application), 21 CFR 812 (Investigational Device Exemptions)
- International Conference on Harmonisation: Good Clinical Practice (ICH E6); 62 Federal Register 25691 (1997); and future revisions
- Belmont Report: Ethical Principles and Guidelines for the Protection of Human Subjects of Research, Report of the National Commission for the Protection of Human Subjects of Biomedical and Behavioral Research
- National Institutes of Health (NIH) Office of Extramural Research, Research Involving Human Subjects, as applicable
- National Institute of Allergy and Infectious Diseases (NIAID) Clinical Terms of Award, as applicable
- Applicable Federal, State, and Local Regulations and Guidance

SIGNATURE PAGE

The signature below provides the necessary assurance that this trial will be conducted according to all stipulations of the protocol, including all statements regarding confidentiality, and according to local legal and regulatory requirements and applicable US federal regulations and ICH E6 Good Clinical Practice (GCP) guidelines.

I agree to conduct the study in compliance with GCP and applicable regulatory requirements.

I agree to conduct the study in accordance with the current protocol and will not make changes to the protocol without obtaining the sponsor's approval and IRB/IEC approval, except when necessary to protect the safety, rights, or welfare of subjects.

Site Investigator Signature:

Signed: ____________________________ Date: ______________

Name

Title

TABLE OF CONTENTS

STATEMENT OF ASSURANCE 2
STATEMENT OF COMPLIANCE 3
SIGNATURE PAGE 4
TABLE OF CONTENTS 5
LIST OF TABLES 10
LIST OF FIGURES 11
LIST OF ABBREVIATIONS 12
PROTOCOL SUMMARY 15
1 KEY ROLES 20
2 BACKGROUND AND SCIENTIFIC RATIONALE 21
2.1 Background 21
2.2 Scientific Rationale 23
2.2.1 Purpose of Study 23
2.2.2 Study Population 24
2.2.3 Selection of Dose 24
2.3 Potential Risks and Benefits 27
2.3.1 Potential Risks 28
2.3.2 Potential Benefits 30
3 STUDY DESIGN, OBJECTIVES AND ENDPOINTS OR OUTCOME MEASURES 31
3.1 Study Design Description 31
3.2 Study Objectives 31
3.2.1 Primary 31
3.2.2 Secondary 31
3.2.3 Exploratory 32
3.3 Study Endpoints or Outcome Measures 32
3.3.1 Primary 32
3.3.2 Secondary 34

3.3.3 Exploratory 35
4 STUDY INTERVENTION/INVESTIGATIONAL PRODUCT 36
4.1 Study Product Description 36
4.1.1 Formulation, Packaging, and Labeling 36
4.1.2 Product Storage and Stability 37
4.2 Acquisition/Distribution 37
4.3 Dosage/Regimen, Preparation, Dispensing and Administration of Study Intervention/Investigational Product 37
4.4 Pre-determined Modification of Study Intervention/Investigational Product for an Individual Subject 39
4.5 Accountability Procedures for the Study Intervention/Investigational Product(s) 40
5 SELECTION OF SUBJECTS AND STUDY ENROLLMENT AND WITHDRAWAL....41
5.1 Eligibility Criteria 41
5.1.1 Subject Inclusion Criteria 41
5.1.2 Subject Exclusion Criteria 42
5.2 Withdrawal from the Study, Discontinuation of Study Product, or Study Termination43
5.2.1 Withdrawal from the Study or Discontinuation of the Study Product 43
5.2.2 Subject Replacement 45
5.2.3 Study Termination 45
6 STUDY PROCEDURES 46
6.1 Induction Period: Visit 0 (Pre-screening, Day -10 to Day 1) 46
6.2 Screening: Visit 1 (Day -1 to Day 1) 46
6.3 Open-label Treatment Period, Planned Study Visits 47
6.3.1 Baseline (Randomization): Visit 2 (Day 1) 47
6.3.2 Visit 3 (Day 8 ± 1 day) 48
6.3.3 Visit 4 (Day 22 ± 2 days) 48
6.3.4 Visit 5 (Day 42 ± 3 days) 49
6.3.5 Test of Cure: Visit 6 (Day 70 ± 7 days) 49
6.3.6 Visit 7 (Day 180 ± 14 days – only for subjects with vertebral osteomyelitis)50
6.3.7 Final Study Visit 51

6.3.8 Early Termination Visit 51
6.4 Unscheduled Study Visits 51
6.5 Protocol Deviations 52
7 DESCRIPTION OF CLINICAL AND LABORATORY EVALUATIONS 53
7.1 Clinical Evaluations 53
7.1.1 Research Procedures 53
7.1.2 Assessment of Concomitant Medications/Treatments Other Than Study Product 54
7.1.3 Assessment of Subject Compliance with Study Intervention/Investigational Product/Investigational Device 55
7.1.4 Non-Research Standard of Care 55
7.2 Laboratory Evaluations 55
7.2.1 Clinical Laboratory Evaluations 55
7.2.2 Research Assays 56
8 ASSESSMENT OF SAFETY 57
8.1 Assessing and Recording Safety Parameters 57
8.1.1 Adverse Events (AEs) 57
8.1.2 Serious Adverse Events (SAEs) 60
8.2 Specification of Safety Parameters 61
8.2.1 Adverse Events of Special Interest (AESIs) 61
8.3 Reporting Procedures 61
8.3.1 Reporting Serious Adverse Events 61
8.3.2 Regulatory Reporting for Studies Conducted Under DMID Sponsored IND62
8.3.3 Reporting of Pregnancy 62
8.4 Type and Duration of Follow-up of Subjects after Adverse Events 63
8.5 Halting Rules 63
8.5.1 Study Halting Criteria 63
8.5.2 Individual Halting Rules 64
8.6 Safety Oversight 64
8.6.1 Data and Safety Monitoring Board (DSMB) 64

9 HUMAN SUBJECTS PROTECTION 66
9.1 Institutional Review Board/Independent Ethics Committee 66
9.2 Informed Consent Process 66
9.2.1 Other Informed Consent Procedures 68
9.3 Exclusion of Women, Minorities, and Children (Special Populations)....69
9.4 Subject Confidentiality 69
9.5 Certificate of Confidentiality 69
9.6 Costs, Subject Compensation, and Research Related Injuries 70
10 STATISTICAL CONSIDERATIONS 71
10.1 Study Hypotheses 71
10.2 Sample Size Considerations 72
10.3 Treatment Assignment Procedures 72
10.3.1 Randomization Procedures 72
10.3.2 Masking Procedures 73
10.4 Planned Interim Analyses 73
10.4.1 Interim Safety Review 73
10.4.2 Interim Efficacy Review 73
10.5 Final Analysis Plan 74
10.5.1 Study Populations 74
10.5.2 Patient Disposition 74
10.5.3 Demographics and Other Baseline Characteristics 75
10.5.4 Extent of Exposure and Treatment Compliance 75
10.5.5 Efficacy Analysis 75
10.5.6 Safety Analyses 77
11 SOURCE DOCUMENTS AND ACCESS TO SOURCE DATA/DOCUMENTS....81
12 QUALITY CONTROL AND QUALITY ASSURANCE 82
13 DATA HANDLING AND RECORD KEEPING 83
13.1 Data Management Responsibilities 83
13.2 Data Coordinating Center/Biostatistician Responsibilities 83

13.3 Data Capture Methods 83
13.4 Types of Data 83
13.5 Study Records Retention 84
14 CLINICAL MONITORING 85
15 PUBLICATION POLICY 86
16 LITERATURE REFERENCES 87
17 APPENDICES 90
Appendix A. Schedule of Events 91
Appendix B. Definitions 94
Appendix C. ARLG Bloodstream Infection Quality of Life Measure 98

LIST OF TABLES

Table 1: Treatment Arms 18
Table 2: Activity of Dalbavancin Against Gram-Positive Pathogens Collected in 2014 US Surveillance 22
Table 3: Activity of Dalbavancin Against Gram –Positive Pathogens Collected in 2014 EU Surveillance 22
Table 4: Substitution Patterns for Dalbavancin API Homologs 36
Table 5: Acceptable SOC Antibiotics....39

LIST OF FIGURES

Figure 1: Schematic of Study Design 19
Figure 2: Simulated Mean Plasma PK Profile for Dalbavancin (Source: Allergen data on file)..26
Figure 3: Dalbavancin Concentration Time Course for a Typical Subject Under the Proposed Regimen (Source: Allergen data on file) 27

LIST OF ABBREVIATIONS

ADL	Activity of Daily Living
AE	Adverse Event/Adverse Experience
AESI	Adverse Events of Special Interest
AUC	Area Under Curve
CNS	Central Nervous System
CE	Clinically Evaluable
CFR	Code of Federal Regulations
CI	Confidence Interval
CMS	Clinical Material Services
CRF	Case Report Form
CTCAE	Common Terminology Criteria for Adverse Events
DCC	Data Coordinating Center
DHHS	Department of Health and Human Services
DILI	Drug-Induced Liver Injury
DMID	Division of Microbiology and Infectious Diseases, NIAID, NIH, DHHS
DOOR	Desirability of Outcome Ranking
DSMB	Data and Safety Monitoring Board
eCRF	Electronic Case Report Form
EDC	Electronic Data Capture
FDA	Food and Drug Administration
FWA	Federal Wide Assurance
GCP	Good Clinical Practice
HEOR	Health Economics and Outcomes Research

HLGT	High Level Group Term
HIPAA	Health Insurance Portability and Accountability Act
ICD	Implantable Cardioverter Defibrillator
ICF	Informed Consent Form
ICH	International Conference on Harmonisation
IE	Infective Endocarditis
IEC	Independent or Institutional Ethics Committee
IND	Investigational New Drug Application
IRB	Institutional Review Board
ITT	Intent to Treat
MedDRA®	Medical Dictionary for Regulatory Activities
MIC	Minimum Inhibitory Concentration
mITT	Modified Intent to Treat
MM	Medical Monitor
MOP	Manual of Procedures
MRSA	Methicillin-resistant *Staphylococcus aureus*
MSSA	Methicillin-sensitive *Staphylococcus aureus*
N	Number (typically refers to subjects)
NIAID	National Institute of Allergy and Infectious Diseases, NIH, DHHS
NIH	National Institutes of Health
OHRP	Office for Human Research Protections
PCS	Potentially Clinically Significant
PID	Patient Identification
PD	Pharmacodynamic

PHI	Protected Health Information
PI	Principal Investigator
PK	Pharmacokinetics
PWID	Persons Who Inject Drugs
QA	Quality Assurance
QC	Quality Control
QoL	Quality of Life
SAE	Serious Adverse Event/Serious Adverse Experience
SD	Standard Deviation
TOC	Test of Cure
ULN	Upper Limit Normal
US	United States

PROTOCOL SUMMARY

**Title:**	Dalbavancin as an Option for Treatment of *S. aureus* Bacteremia (DOTS): A Phase 2b, Multicenter, Randomized, Open-Label, Assessor-Blinded Superiority Study to Compare the Efficacy and Safety of Dalbavancin to Standard of Care Antibiotic Therapy for the Completion of Treatment of Patients with Complicated *S. aureus* Bacteremia
**Design of the Study:**	Multicenter, randomized, open-label, assessor-blinded, superiority, active-controlled, parallel-group study
**Study Phase:**	2b
**Study Population:**	200 adult subjects (≥ 18 years old) diagnosed with complicated *S. aureus* bacteremia including definite or possible right-sided infective endocarditis (IE) treated with effective antibiotic therapy for at least 72 hours (maximum 10 days) and with subsequent clearance of bacteremia prior to randomization to study treatment
**Number of Sites:**	Approximately 20
**Description of Study Product or Intervention:**	• Dalbavancin 1500 mg intravenously (IV) over 30 (± 10) minutes on Day 1 and 1500 mg intravenously (IV) over 30 (± 10) minutes on Day 8 (if creatinine clearance ≥ 30 mL/min or if on regular hemodialysis or peritoneal dialysis)<br>• Dalbavancin 1125 mg IV over 30 (±10) minutes on Day 1 and Dalbavancin 1125 mg IV over 30 (±10) minutes on Day 8 (if creatinine clearance < 30 mL/min and not receiving regular hemodialysis or peritoneal dialysis)

Study Objectives:

Primary:

- To compare the Desirability of Outcome Ranking (DOOR) at Day 70 of dalbavancin to that of standard of care antibiotic therapy used to consolidate therapy for the treatment of subjects with complicated *S. aureus* bacteremia in the intent-to-treat (ITT) population.

Secondary:

- To compare the clinical outcomes of dalbavancin with the standard of care antibiotic therapy at day 70 in the modified intent-to-treat (mITT) population.
- To compare the safety of dalbavancin with that of the standard of care treatment in the mITT.
- To compare each individual component of the DOOR outcome by treatment arm, in the ITT population.

Exploratory:

- To compare the clinical outcomes of dalbavancin with the standard of care antibiotic therapy at day 70 in the clinically evaluable (CE) population.
- To compare the DOOR endpoint of subjects on dalbavancin with that of subjects receiving standard of care antimicrobial therapy at day 42 in the ITT, mITT, and CE populations.
- To compare the clinical and microbiologic outcomes of dalbavancin with the standard of care antibiotic therapy at day 42 in ITT, mITT and CE populations.
- To compare clinical and microbiologic outcomes of dalbavancin with standard of care antibiotic therapy between clinically important subgroups, including a) those with MSSA versus MRSA; b) persons who inject drugs (PWID) vs non-PWID; c) those who received infectious disease consultation vs those who did not; d) underlying site of infection (endovascular, bone and joint, skin, pulmonary); e)

divided by duration of initial bacteremia in the ITT, mITT, and CE populations

- To compare Quality of Life (QoL) of subjects on dalbavancin with that of subjects receiving standard of care antibiotic therapy at Baseline, Day 42, and Day 70, in the ITT, mITT, and CE populations
- To characterize the population pharmacokinetic profile for dalbavancin administered via a 2-dose regimen (1500 mg on day 1 and day 8, renally adjusted when appropriate) in patients with *Staphylococcus aureus* bacteremia
- To assess patient-level and clinical covariates associated with dalbavancin pharmacokinetics in patients with *Staphylococcus aureus* bacteremia
- Examine the association between individualized plasma concentration profiles and clinical and microbiologic outcomes at day 42 and TOC
- Examine the association between individualized plasma concentration profiles and occurrence of adverse drug events, including AST/ALT elevations >3X upper limit of normal
- Examine the association between individualized plasma concentration profiles and late recurrence risk among the subset of patients with osteomyelitis and a 6 month follow-up visit

**Duration of Individual Subject Participation:**	Approximately 70 ± 7 days, with a late post-treatment follow-up visit for the subset of patients with osteomyelitis at 6 months
**Estimated Time to Last Subject/Last Study Day:**	Approximately 30 months, from site activation to the last subject's last study day.

Table 1: Treatment Arms

Dalbavancin	100 subjects	Dalbavancin 1500 mg IV over 30 (± 10) minutes on Day 1 and 1500 mg IV over 30 (± 10) minutes on Day 8, renally dose-adjusted to 1125 mg for subjects with CrCl <30 and not on dialysis
Standard of Care[a]	100 subjects	• Methicillin-sensitive *Staphylococcus aureus* (MSSA): nafcillin (2 g IV q4h × 4-6 weeks) OR oxacillin (2 g IV Q4h x 4-6 weeks OR cefazolin (2 g IV q8h × 4-6 weeks)<br>• Methicillin-resistant *Staphylococcus aureus* (MRSA): vancomycin (dose per local standard of care × 4-6 weeks) OR daptomycin (6-10 mg/kg IV daily × 4-6 weeks)

[a]Renally dose-adjusted as appropriate, per local standard of care

Figure 1: Schematic of Study Design

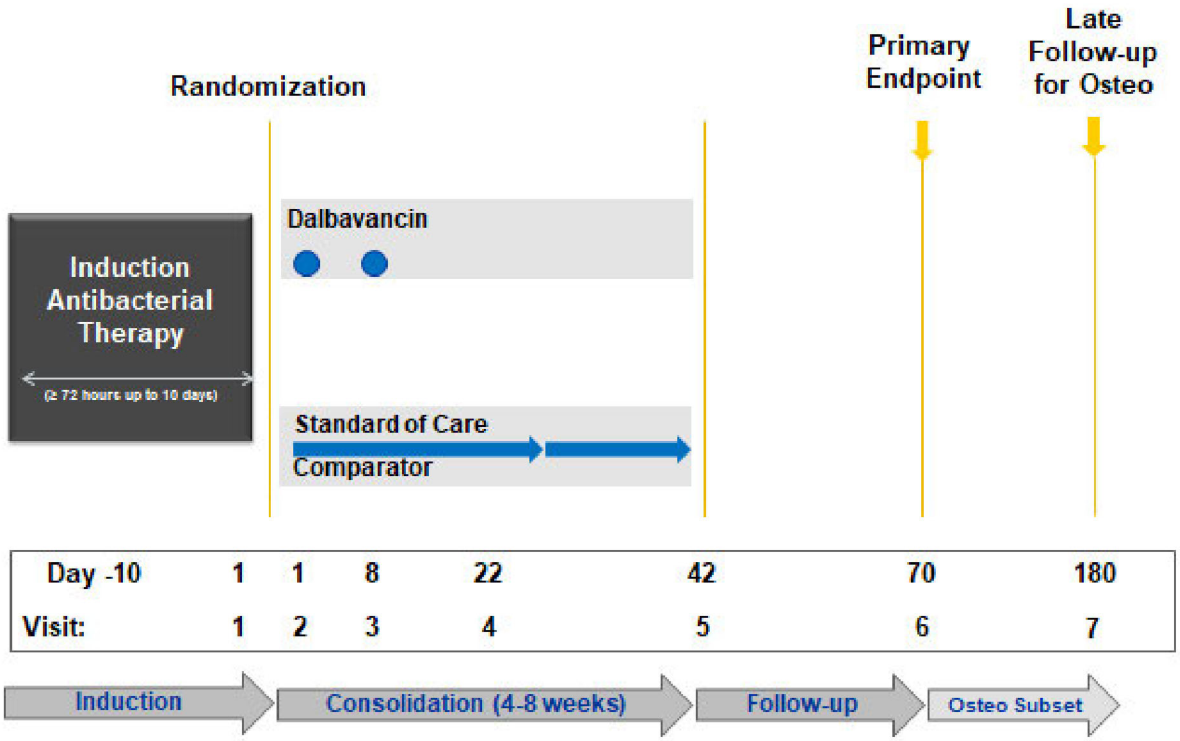

1 KEY ROLES

**Lead Principal Investigator:**	Thomas L. Holland, MD<br>Associate Professor of Medicine, Duke University<br>[redacted]<br>Duke University Medical Center<br>Durham, NC, 27710<br>[redacted]
**DMID Clinical Project Manager:**	[redacted]
**Statistical and Data Coordinating Center:**	The Emmes Company, LLC<br>401 N. Washington St. Suite 700<br>Rockville, MD 20850<br>[redacted]

2 BACKGROUND AND SCIENTIFIC RATIONALE

2.1 Background

Staphylococcus aureus bacteremia is a life-threatening infection with case fatality rates ranging from 15-50% (van Hal 2012). *S. aureus* bacteremia is also a leading cause of IE in industrialized nations (Tong 2015) and is associated with in-hospital mortality rates ranging from 16% to 25% (Thuny 2005, Murdoch 2009, Sy 2010, Selton-Suty 2012). Standard treatment of complicated *S. aureus* bacteremia and IE requires prolonged IV antibiotic therapy (4-6 weeks), typically necessitating placement of a central IV catheter, prolonged hospitalization, and home nursing or admission to a long-term care facility. Treatment is associated with high cost and healthcare burden, as well as complications such as catheter related bloodstream infections and catheter associated thrombosis (Keller 2018). Safe and effective alternative treatment strategies are needed.

Dalbavancin is a lipoglycopeptide that has potent activity against Gram-positive pathogens including MRSA (Table 2, Table 3). It is currently approved for treatment of acute bacterial skin and skin structure infection in the US and EU as a single dose or 2-dose regimen. A 2-dose, once weekly regimen can provide systemic therapy for 6 weeks, eliminating the need for a centrally placed catheter or prolonged IV access for antibiotic administration.

Table 2: Activity of Dalbavancin Against Gram-Positive Pathogens Collected in 2014 US Surveillance

		Dalbavancin MIC (µg/mL)			
Organism	N	Range	50%	90%	% Susceptible[a]
S aureus: all	1625	0.008 - 0.12	0.06	0.06	100
Methicillin-susceptible	875	0.008 - 0.12	0.06	0.06	100
Methicillin-resistant	750	0.008 - 0.12	0.06	0.06	100
Viridans group streptococci	220	≤ 0.002 - 0.12	0.015	0.03	100
Penicillin-nonsusceptible	46	0.004 - 0.06	0.015	0.03	100
S pyogenes	108	≤ 0.002 - 0.06	0.008	0.03	100
S dysgalactiae	75	≤ 0.004 - 0.12	0.015	0.03	100
E faecalis	151	0.02 - > 0.25	0.06	0.06	97.6

Bacterial pathogens frequently implicated as causative agents of IE include *S aureus* (including MRSA), *Streptococcus pyogenes,* viridans group streptococci, Group C and G streptococci, and *Enterococcus* spp.

IE = infective endocarditis; MRSA = methicillin-resistant *S aureus*; MIC = minimum inhibitory concentration

[a] Susceptible at ≤ 0.25 µg/mL

Source: Dalbavancin International (Two Continents) Surveillance Report for 2014, JMI Laboratories; Protocol 14-DUR-01, May 2015.

Table 3: Activity of Dalbavancin Against Gram –Positive Pathogens Collected in 2014 EU Surveillance

		Dalbavancin MIC (µg/mL)			
Organism	N	Range	50%	90%	% Susceptible[a]
S aureus: all	1625	0.004 - 0.12	0.06	0.06	100
Methicillin-susceptible	1209	0.015 - 0.12	0.06	0.06	100
Methicillin-resistant	416	0.004 - 0.12	0.03	0.06	100
Viridans group streptococci	213	≤ 0.002 - 0.12	0.015	0.03	100
Penicillin-nonsusceptible	57	≤ 0.002 - 0.06	0.015	0.03	100
S pyogenes	106	0.004 - 0.12	0.008	0.03	100
S dysgalactiae	94	≤ 0.002 - 0.12	0.015	0.03	100
E faecalis	305	0.03 - > 0.25	0.06	0.06	98.4

Bacterial pathogens frequently implicated as causative agents of IE include *S aureus* (including MRSA), *Streptococcus pyogenes,* viridans group streptococci, Group C and G streptococci, and *Enterococcus* spp.

IE = infective endocarditis; MRSA = methicillin-resistant *S aureus*; MIC = minimum inhibitory concentration

[a] Susceptible at ≤ 0.25 µg/mL

Source: Dalbavancin International (Two Continents) Surveillance Report for 2014, JMI Laboratories; Protocol 14-DUR-01, May 2015.

Dalbavancin appeared effective in rat and rabbit models of *S. aureus* IE (Candiani 1999, Lefort 2004). In the rat model of staphylococcal IE, dalbavancin was as effective as vancomycin and

teicoplanin at reducing the bacterial load in the heart, but with a lower dose and less frequent dosing intervals compared to standard of care (Candiani 1999). In a rabbit model of IE, dalbavancin given once daily (10 mg/kg for 4 days) or as a single dose of 40 mg/kg was effective against a strain of *S. aureus* with reduced susceptibility to vancomycin and teicoplanin (Lefort, 2004). Additionally, 5 Phase 2/3 clinical studies that evaluated the efficacy and safety of dalbavancin administered as a single dose or 2-dose regimen found that 100% of patients (55 of 55 evaluable subjects) with *S. aureus* bacteremia achieved clearance of bacteremia (Raad 2005, Dunne 2016, Boucher 2014, Selzter 2003, Jauregui 2005). Thus, dalbavancin may be a safe and effective alternative therapy for *S. aureus* bacteremia/IE, including highly antibiotic-resistant strains.

The safety profile of dalbavancin has been characterized for a total cumulative dose of 1500 mg, whether administered as a single dose or in split weekly doses (1000 mg on Day 1 followed by 500 mg on Day 8). Adverse reactions have been evaluated for 2473 patients treated with dalbavancin. Overall, the most common adverse reactions were nausea (4.7%), headache (3.8%), and diarrhea (3.4%). The median duration of adverse reactions was 3 days for patients receiving dalbavancin and 4 days for patients receiving a comparator. The safety database for the 3000 mg total dose includes 12 subjects from a Phase 1 study: 6 received a total of 3500 mg dalbavancin over 6 weeks (with a total of 2 mild adverse events), and 6 received a total of 4500 mg dalbavancin over 8 weeks (with total of 4 mild adverse events and 1 moderate adverse event). Elevations in ALT >3x the upper limit of normal have been reported, though at a frequency not significantly different than comparator arms in trials conducted to date. Per product insert, 4.9% (121/2473) subjects receiving dalbavancin experienced SAEs, with 2.6% (64/2473) discontinuing the medication as a result.

2.2 Scientific Rationale

2.2.1 Purpose of Study

The current standard of care for the antibiotic treatment of complicated bacteremia or IE uses a stepwise approach. The initial phase of treatment involves the initiation of empirical antibiotic therapy, definitive diagnosis (as per the modified Duke criteria), and the assessment of the need for early valve replacement, if applicable. Subsequent identification of the causative pathogen, including antibiotic susceptibility and MICs supports the choice of definitive antibiotic therapy and determination of the required duration of antibiotic treatment. Guidelines for the use of outpatient parenteral antibiotic therapy in the treatment of complicated bacteremia or IE similarly advocate that antibiotic therapy can be divided into an initial phase during which life threatening complications of complicated bacteremia or IE are likely to occur (approximately 14 days) and a completion phase of therapy (2 weeks to 6 weeks) (Holland 2018).

The proposed clinical study design of dalbavancin in the treatment of complicated bacteremia or IE is consistent with this standard of care. Specifically, prior to study eligibility, patients will receive

pre-randomization antibiotic therapy pending a definitive diagnosis of complicated *S. aureus* bacteremia or IE, as well as the resolution of bacteremia. Eligible patients will then be randomized into the study to complete their antibiotic therapy with either a 2-dose regimen of dalbavancin or the current standard of care with daily IV administration of antibiotic therapy for a total duration of 4 to 6 weeks (Baddour 2015) for most patients, and up to 8 weeks for patients with vertebral osteomyelitis/discitis.

The proposed clinical study design offers a number of advantages. First, it will support enrollment of patients with a confirmed diagnosis of complicated bacteremia or IE. Prior studies have been limited by the inability to confidently determine complicated vs uncomplicated status prior to randomization (Corey 2009). Second, the proposed study design addresses a true area of need in *S. aureus* bacteremia management and reflects the likely pattern of "real world" dalbavancin use by clinicians for the completion of systemic antibiotic therapy for complicated bacteremia or IE without the need for indwelling IV access to support daily therapy. This takes full advantage of the unusual PK profile of dalbavancin and the introduction of this therapy into clinical practice would potentially have a major impact on patient well-being and quality of life (QoL). In this study, presence or absence of these potential QoL effects will be assessed using an ARLG Bloodstream Infection QoL Measure developed specifically for this purpose (King 2020). Additionally, two previously validated measures (EQ-5D-5L, https://euroqol.org/eq-5d-instruments/sample-demo/ and the PROMIS Global Health short form, http://www.healthmeasures.net/administrator/components/com_instruments/uploads/Global%20Health%20Scale%20v1.2%2008.22.2016.pdf) will also be collected. Third, adequate treatment of complicated bacteremia or IE requires prolonged systemic antibiotic therapy to prevent relapse. Introduction of the 2-dose dalbavancin regimen may decrease the risk of relapse. Finally, the proposed design of this clinical study is consistent with antibiotic stewardship principles, reserving dalbavancin therapy for patients with fully characterized infections and pathogens.

2.2.2 Study Population

Approximately 200 eligible subjects will be enrolled to the study in the United States and Canada. Eligible subjects are adults who have been diagnosed with complicated bacteremia or right-sided IE due to *S. aureus*, have been treated with appropriate empiric/targeted antibiotic therapy, and in whom the blood cultures have become negative after at least 72 hours of initial antibiotic therapy (maximum 10 days). Subjects with uncomplicated bacteremia due to *S. aureus* will be excluded. More details regarding subject inclusion and exclusion criteria can be found in Section 5.1.

2.2.3 Selection of Dose

The dalbavancin dosing regimen consists of 1500 mg on Day 1 and 1500 mg on Day 8 for subjects with normal renal function or who are receiving dialysis, administered over 30 minutes by IV infusion. Patients with CrCl <30 who are not receiving dialysis will receive a reduced dose (1125mg

on Day 1 and 1125 mg on Day 8). Based on a comparison to the updated nonclinical pharmacokinetic (PK)/pharmacodynamics (PD) target of the area under the unbound drug concentration-time curve [fAUC]/MIC (Lepak 2015), this regimen is expected to provide sufficient therapeutic concentrations of free drug against *S. aureus* through Day 42.

Consistent with prior nonclinical investigation (Andes 2007), Lepak et al (Lepak 2015) found fAUC/MIC to be the most relevant PK/PD index in a neutropenic murine thigh infection model, with mean free drug daily area under the curve (AUC)/minimum inhibitory concentration (MIC)s for net stasis, 1log kill, and 2log kill of 27.1, 53.3, and 111.1, respectively. To justify the proposed dosing regimen, a target attainment analysis was conducted using the updated population PK model (Carrothers 2020). In the PK simulation, the proposed regimen was simulated using the Bayesian post hoc estimates of each of the 703 patients in the merged Phase 2/3 population PK dataset. As with previous target attainment analyses, free drug levels were assumed to be 7% of total drug concentrations. As a conservative assumption, the mean daily AUC for target attainment was calculated based on dalbavancin levels on Day 42. For the MIC, the dalbavancin *S. aureus* MIC_{90} of 0.06 mg/L was used. Results of this simulation analysis showed target attainments of > 99%, > 99%, and 90% for the net stasis, 1log kill, and 2log kill targets, respectively. For an even more conservative estimate, the US breakpoint for susceptibility of *S. aureus* to dalbavancin of 0.25 mg/L was also used: the 90% target attainment was achieved through Day 42 (stasis), Day 36 (1-log kill), and Day 28 (2-log kill) after clearance of bacteremia.

Simulations designed to evaluate plasma concentration-time profiles suggest that a 2-dose regimen of dalbavancin of 1500 mg given on Days 1 and 8 will provide plasma concentrations above the MIC_{99} of *S. aureus* for an average of 49 days after the start of therapy Figure 2.

Figure 2: Simulated Mean Plasma PK Profile for Dalbavancin (Source: Allergen data on file)

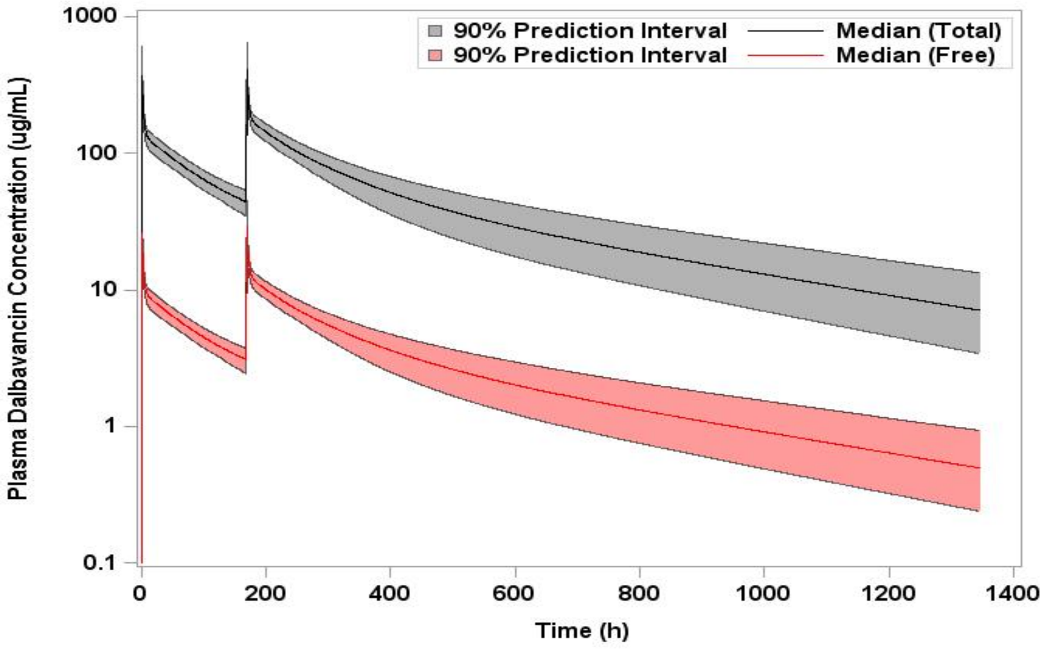

Figure 3 shows the time course of total dalbavancin concentration over the 6-week course of therapy for a typical patient. For this typical patient, Day 42 total drug concentration was 6.7 µg/mL [free drug = 7% × 6.7 = 0.47 µg/mL], which is greater than 7-fold the MIC_{90} value of 0.06 µg/mL for *S. aureus*, and > 15-fold the MIC_{90} value of 0.03 µg/mL for beta-hemolytic streptococci (Dalbavancin International [Two Continents] Surveillance Report for 2014) even at the end of a 6-week course of therapy.

Figure 3: Dalbavancin Concentration Time Course for a Typical Subject Under the Proposed Regimen (Source: Allergen data on file)

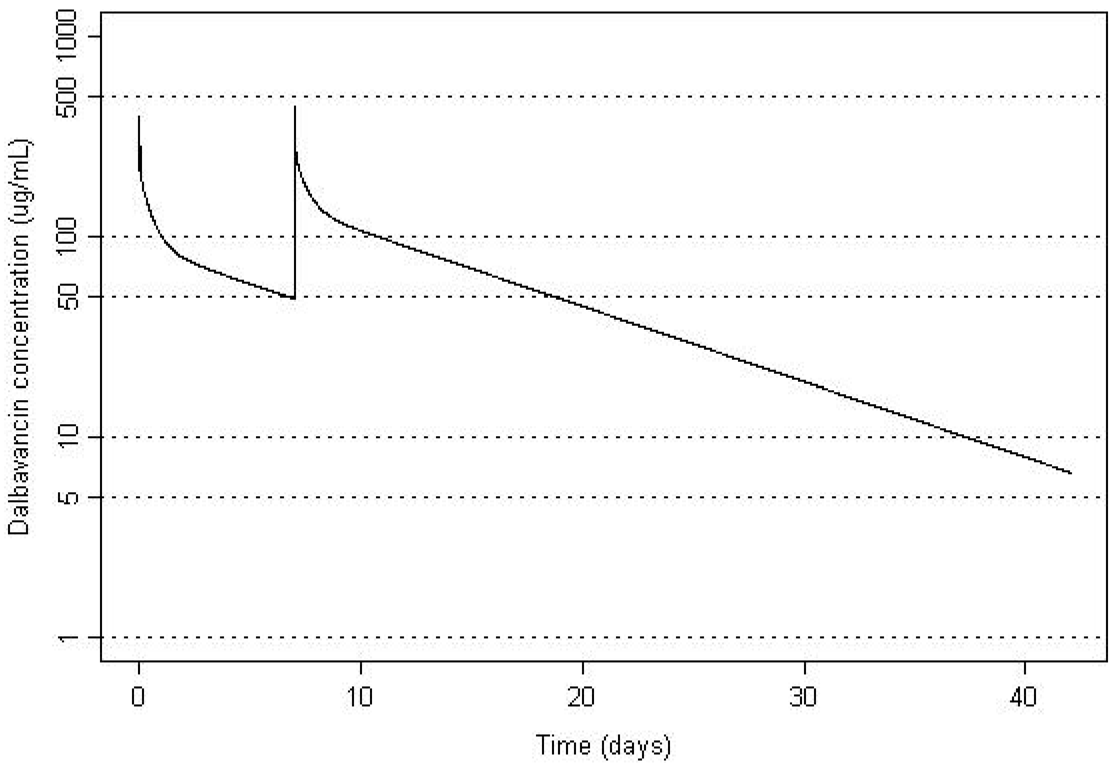

2.3 Potential Risks and Benefits

Dalbavancin is FDA-approved for the treatment of acute bacterial skin and skin structure infections caused by Gram positive organisms, though at lower doses than proposed for treatment of bacteremia here and has been the subject of one prior randomized controlled clinical trial for osteomyelitis using the same dosing regimen proposed in this study. Risk information is derived from both the product insert and previously conducted trials.

For those randomized to standard of care arms, the antistaphylococcal beta-lactams, cefazolin, vancomycin and daptomycin are all commonly used for treatment of *S. aureus* bacteremia and are associated with potential risks as well – outlined below from product inserts. These include risks associated with an indwelling vascular catheter, primarily secondary infections or thromboses. These also include the need for frequent blood draws to monitor for antibiotic toxicities.

2.3.1 Potential Risks

2.3.1.1 Dalbavancin

Hypersensitivity Reactions: Hypersensitivity reactions (both anaphylactic and limited cutaneous reactions) have been reported with glycopeptide antibiotics, including dalbavancin. Subjects will be excluded from this study if they report any history of anaphylactic reaction to glycopeptide antibiotics or dalbavancin.

Infusion-related Reactions: Rapid infusion of dalbavancin has been reported to cause flushing, urticarial, pruritic, or rash reactions resembling the "Red-Man Syndrome" described in association with vancomycin. Stopping or slowing the infusion typically resolves these reactions.

Central Nervous System Reactions: Headache has been infrequently reported with dalbavancin receipt (<4%).

Gastrointestinal: Nausea (<4%) and diarrhea (<4%) have been infrequently reported with dalbavancin receipt (<4%).

C. difficile infection: *C. difficile*-associated diarrhea has been reported with nearly all antibacterial agents, including dalbavancin.

Hepatic: Elevations of AST/ALT have been reported in clinical trials.

2.3.1.2 Cefazolin

Hypersensitivity reactions: Anaphylaxis and a range of cutaneous drug eruptions have been reported with cefazolin.

Localized reactions: Phlebitis has been rarely reported with cefazolin.

Nephrotoxicity: Elevations in BUN and creatinine, infrequently even acute renal failure, have been reported.

Gastrointestinal: Nausea, diarrhea and anorexia have been reported.

C. difficile diarrhea: *C. difficile* diarrhea has been reported with nearly all antibiotics.

Hematologic: Neutropenia and thrombocytopenia have both been infrequently reported and are generally reversible upon discontinuation.

Hepatic: Transient elevations in AST/ALT have been reported.

2.3.1.3 Nafcillin

Hypersensitivity reactions: Anaphylaxis and a range of cutaneous drug eruptions have been reported with nafcillin.

Localized reactions: Phlebitis, sometimes even with skin sloughing, has been rarely reported with nafcillin.

Nephrotoxicity: Elevations in BUN and creatinine, infrequently even acute renal failure or acute interstitial nephritis, have been reported.

Gastrointestinal: Nausea, diarrhea and anorexia have been reported.

C. difficile diarrhea: *C. difficile* diarrhea has been reported with nearly all antibiotics.

Hematologic: Neutropenia or even agranulocytosis has been reported with nafcillin and is generally reversible upon discontinuation.

Hepatic: Transient elevations in AST/ALT have been reported.

2.3.1.4 Oxacillin

Hypersensitivity reactions: Anaphylaxis and a range of cutaneous drug eruptions have been reported with oxacillin.

Nephrotoxicity: Elevations in BUN and creatinine, infrequently even acute renal failure or acute interstitial nephritis, have been reported.

Gastrointestinal: Nausea, diarrhea and anorexia have been reported.

C. difficile diarrhea: *C. difficile* diarrhea has been reported with nearly all antibiotics.

Hematologic: Neutropenia or even agranulocytosis has been reported with oxacillin and is generally reversible upon discontinuation.

Hepatic: Transient elevations in AST/ALT have been reported.

2.3.1.5 Vancomycin

Infusion reactions: Rapid infusion of vancomycin has been reported to cause flushing, urticarial, pruritic, or rash reactions known as the "Red-Man Syndrome". Stopping or slowing the infusion typically resolves these reactions.

Phlebitis and localized cutaneous reactions: Vancomycin can be irritating to soft tissues, including causing thrombophlebitis at infusion sites. Inadvertent extravasation may result in localized pain, tenderness, or even necrosis.

Hypersensitivity reactions: Hypersensitivity reactions including anaphylaxis have been reported with vancomycin.

Nephrotoxicity: When administered systemically, vancomycin can cause acute kidney injury. Risk of injury increases with supratherapeutic levels.

Ototoxicity: Hearing loss and tinnitus have been reported with vancomycin, reported mostly in patients with supratherapeutic levels, underlying hearing loss, or concomitant receipt of other ototoxic agents.

C. difficile diarrhea: *C. difficile* associated diarrhea has been reported with the use of nearly all antibiotics.

Hematologic: Vancomycin has occasionally been associated with neutropenia or rarely thrombocytopenia. Cytopenias are usually reversible upon discontinuation.

2.3.1.6 Daptomycin

Hypersensitivity reactions: Anaphylaxis and a range of cutaneous drug eruptions have been reported with daptomycin.

Myopathy/rhabdomyolysis: Daptomycin has been reported to cause a myositis characterized by elevated creatine kinase levels, muscle pain, and muscle weakness. Risk may be increased upon co-administration with statins. Myopathy is generally reversible upon discontinuation.

Eosinophilic pneumonia: Daptomycin has been rarely reported to cause eosinophilic pneumonia which may present with fever, dyspnea and pulmonary opacities.

Neuropathy: There are rare reports of peripheral neuropathy with daptomycin.

C. difficile diarrhea: *C. difficile* diarrhea has been reported with nearly all antibiotics.

2.3.1.7 Risks Associated with Intravenous Access

The risks associated with peripheral IV placement are minimal and include minor bruising/bleeding, localized discomfort at the site, and rarely infection or a superficial clot.

The risks associated with peripherally inserted central venous catheters (PICC lines) or central lines include bleeding, discomfort at the site, deep venous thrombosis (blood clot), infection, rarely nerve injury or an irregular heartbeat.

2.3.2 Potential Benefits

Dalbavancin may or may not improve the clinical outcome of an individual subject who participates in this trial. There is potential benefit to society from their participation in this study resulting from insights gained about outcomes with dalbavancin among patients with *S. aureus* bacteremia. Since standard of care for complicated *S. aureus* bacteremia currently requires dosing via indwelling central venous access, with frequent dosing and prolonged duration of therapy, the potential benefit is the possible demonstration that a dalbavancin treatment strategy may avoid prolonged hospital stays and the need for central venous access.

3 STUDY DESIGN, OBJECTIVES AND ENDPOINTS OR OUTCOME MEASURES

3.1 Study Design Description

This clinical study will be a Phase 2b, multicenter, randomized, open-label, assessor-blinded, superiority study. The study will compare dalbavancin to standard of care antibiotic therapy for the completion of therapy in patients with complicated bacteremia or right-sided native valve IE caused by *S. aureus* who have cleared their baseline bacteremia.

Approximately 200 subjects will be randomized 1:1 to receive either dalbavancin or a standard of care antibiotic regimen that is based upon the identification and antibiotic susceptibility pattern of the baseline organism. Those randomized to the dalbavancin treatment group will receive 2 doses of dalbavancin IV 1 week apart (1500 mg on Day 1 and Day 8 after randomization, with renal dose adjustment if appropriate). Those subjects randomized to the standard of care antibiotic therapy treatment group will receive an antibiotic regimen considered to be standard of care based on the methicillin susceptibility pattern of the pathogen isolated at Baseline for a duration of 4 to 6 weeks.

Figure 1 provides a schematic of the study design. Study procedures are presented in Section 6. Detailed descriptions of each study visit can be found in Section 6.3.

3.2 Study Objectives

3.2.1 Primary

- To compare the Desirability of Outcome Ranking (DOOR) at Day 70 of dalbavancin to that of standard of care antibiotic therapy used to consolidate therapy for the treatment of subjects with complicated *S. aureus* bacteremia in the intent-to-treat (ITT) population.

3.2.2 Secondary

- To compare the clinical outcomes of dalbavancin with the standard of care antibiotic therapy at day 70 in the mITT population (see Section 10.5.1 for definitions of study populations).
- To compare the safety of dalbavancin with that of the standard of care treatment in the mITT population.
- To compare each individual component of the DOOR outcome by treatment arm, in the ITT population.

3.2.3 Exploratory

1. To compare the clinical outcomes of dalbavancin with the standard of care antibiotic therapy at day 70 in the CE population (see Section 10.5.1 for definition).
2. To compare the DOOR endpoint of subjects on dalbavancin with that of subjects receiving standard of care antimicrobial therapy at day 42, in the ITT, mITT and CE populations.
3. To compare the clinical and microbiologic outcomes of dalbavancin with the standard of care antibiotic therapy at day 42 in ITT, mITT and CE populations.
4. To compare clinical and microbiologic outcomes of dalbavancin with standard of care antibiotic therapy between clinically important subgroups, including a) those with MSSA versus MRSA; b) persons who inject drugs (PWID) vs non-PWID; c) those who received infectious disease consultation vs those who did not; d) underlying site of infection (endovascular, bone and joint, skin, pulmonary); e) divided by duration of initial bacteremia, in the ITT, mITT, and CE populations
5. To compare Quality of Life (QoL) of subjects on dalbavancin with that of subjects receiving standard of care antibiotic therapy at Baseline, Day 42, and Day 70, in the ITT, mITT, and CE populations
6. To characterize the population pharmacokinetic profile for dalbavancin administered via a 2-dose regimen (1500 mg on day 1 and 1500 mg on day 8; renally adjusted when appropriate) in patients with *Staphylococcus aureus* bacteremia
7. To assess patient-level and clinical covariates associated with dalbavancin pharmacokinetics in patients with *S. aureus* bacteremia
8. Examine the association between individualized plasma concentration profiles and clinical and microbiologic outcomes at day 42 and TOC.
9. Examine the association between individualized plasma concentration profiles and occurrence of adverse drug events, including AST/ALT elevations >3X upper limit of normal.
10. Examine the association between individualized plasma concentration profiles and late recurrence risk among the subset of patients with osteomyelitis and a 6-month follow-up visit

3.3 Study Endpoints or Outcome Measures

3.3.1 Primary

The **primary outcome** measure is the DOOR endpoint at day 70. The clinical components of the DOOR endpoint (success/failure and infectious complications) will be completed by an independent adjudication committee, blinded to treatment assignment. Day 70 was selected in this study as it

occurs approximately 4 weeks after treatment completion for most patients, allowing time for detection of relapse.

There are 5 possible rankings in the DOOR endpoint:

Rank	Alive	How many of: 1) Clinical Failure 2) Infectious Complication 3) SAE, or AE leading to study drug discontinuation	QoL
1	Yes	0 of 3	Tiebreaker based on QoL score
2	Yes	1 of 3	
3	Yes	2 of 3	
4	Yes	3 of 3	
5	No (Death)	Any	

Rank 1: Alive without any of the following: (1) evidence of clinical failure; (2) an infectious complication; or (3) any SAE, or an AE leading to study drug discontinuation

Rank 2: Alive but with one of the following: (1) evidence of clinical failure; (2) an infectious complication; or (3) any SAE, or an AE leading to study drug discontinuation

Rank 3: Alive but with two of the following: (1) evidence of clinical failure; (2) an infectious complication; or (3) any SAE, or an AE leading to study drug discontinuation

Rank 4: Alive but with all of the following: (1) evidence of clinical failure; (2) an infectious complication; or (3) any SAE, or an AE leading to study drug discontinuation

Rank 5: Death

Clinical failure, infectious complications, and adverse events are each binary (yes/no) components of the DOOR endpoint.

For the primary endpoint, quality of life will be measured as a change from baseline QoL to day 70 QoL score, as assessed by the ARLG Bloodstream Infection QoL Measure (Appendix C).

Definitions for the DOOR endpoint:

Clinical Success: Resolution of clinical signs and symptoms of *S. aureus* bacteremia such that no additional antibiotic therapy is required or anticipated for its treatment.

Note that it is possible to achieve this overall Clinical Success status at Day 70 even if Infectious Complications have occurred prior to that time. For example, a patient who has a new metastatic focus on infection diagnosed after randomization, but who subsequently completes treatment and is felt to be cured at Day 70 would be considered a Clinical Success (and the Infectious Complication would result in a lower DOOR). That is, Clinical Success reflects the patient's overall status at the time of that assessment.

Clinical Failure: Absence of clinical success

Infectious Complications: occurrence of any of the following, between randomization and day 70:

- Endocarditis
- New evidence of metastatic foci of infection – e.g. osteomyelitis, visceral abscess, septic joint
- Relapse – isolation of baseline *S. aureus* pathogen from a blood culture drawn after randomization
- Readmission for subsequent care of indication under study
- Need for additional unplanned source control procedures – e.g. abscess debridement or drainage, cardiac valve replacement
- Change in antibiotic therapy due to inadequate clinical response. For any changes to study drug in the standard of care group, or when new antibiotics are started in either treatment group, the site PI will record the reason for the antibiotic change.

3.3.2 Secondary

The **secondary efficacy outcome** is as follows**.**

- Clinical efficacy, defined as none of: 1) Clinical failure; 2) Infectious complications; 3) Mortality

The **secondary safety outcome** is as follows.

- Proportion of patients who have either 1) an SAE; or 2) an AE leading to study drug discontinuation

Each component of the DOOR will also be examined separately:

- clinical success
- infectious complications
- SAEs

- AEs leading to study drug discontinuation
- mortality

3.3.3 Exploratory

1. Clinical efficacy by treatment group in the CE population at day 70
2. DOOR endpoint by treatment group at day 42 in the ITT, mITT, and CE populations
3. Clinical and microbiologic outcomes by treatment group at day 42 in the ITT, mITT, and CE populations
4. Clinical and microbiologic outcomes by treatment group, within each subgroup of clinical interest, at day 42 and day 70 in the ITT, mITT, and CE populations. A microbiologic success will be defined as no post-randomization growth (e.g., no positive cultures) of the baseline pathogen from blood cultures or another sterile body site
5. QoL at Baseline, Day 42, and Day 70, which will be assessed using the ARLG Bloodstream Infection QoL Measure (Appendix C) as well as two additional comparator measures (EQ-5D-5L, https://euroqol.org/eq-5d-instruments/sample-demo/ and PROMIS Global Health short form, http://www.healthmeasures.net/administrator/components/com_instruments/uploads/Global%20Health%20Scale%20v1.2%2008.22.2016.pdf) in the ITT, mITT, and CE populations
6. Population mean PK parameter estimates and the magnitude of the associated inter individual variability for the 2-dose dalbavancin regimen in patients with *S. aureus* bacteremia.
7. Individual post hoc PK parameter estimates and calculated exposure measures for the 2-dose dalbavancin regimen in patients with *S. aureus* bacteremia.
8. Clinical and microbiologic response at day 42 and Day 70 according to individual plasma dalbavancin concentration curves.
9. Occurrence of grade 3 or higher adverse drug events, adverse events of special interest (AESIs), and occurrence of AST/ALT elevations >3x upper limit of normal (ULN) from first dose of dalbavancin through follow-up period.
10. Late recurrence within the osteomyelitis population will be defined by the presence of the following up to 6 months after randomization: progressive imaging changes along with isolation of *S. aureus* from blood, bone biopsy, associated fluid aspiration, or operative tissue culture.

4 STUDY INTERVENTION/INVESTIGATIONAL PRODUCT

4.1 Study Product Description

Dalbavancin is a lyophilized, white to off-white to pale yellow solid. It is a lipoglycopeptide synthesized from a fermentation product of *Nonomuraea* species. Dalbavancin is a mixture of five closely related active homologs (A_0, A_1, B_0, B_1, and B_2), shown in Table 4, below. The homolog B_0 is the major component of dalbavancin. The homologs share the same core structure and differ in the fatty acid side chain of the N-acylaminogluronic acid moiety (R_1) structure and/or the presence of an additional methyl group (R_2) on the terminal amino group, shown in Table 4 below. The B_0 INN chemical name is 5,31-dichloro-38-de(methoxycarbonyl)-7-demethyl-19-deoxy-56-O [2-deoxy-2-[(10-methylundecanoyl)amino]-β-D-glucopyranuronosyl]-38-[[3-(dimethylamino)propyl] carbamoyl]-42-O-α-D-mannopyranosyl-15-N-methyl(ristomycin A aglycone) hydrochloride. Please refer to the package insert for further details.

Table 4: Substitution Patterns for Dalbavancin API Homologs

Dalbavancin	R_1	R_2	Molecular Formula	Molecular Weight*
A_0	$CH(CH_3)_2$	H	$C_{87}H_{98}N_{10}O_{28}Cl_2 \cdot 1.6\ HCl$	1802.7
A_1	$CH_2CH_2CH_3$	H	$C_{87}H_{98}N_{10}O_{28}Cl_2 \cdot 1.6\ HCl$	1802.7
B_0	$CH_2CH(CH_3)_2$	H	$C_{88}H_{100}N_{10}O_{28}Cl_2 \cdot 1.6\ HCl$	1816.7
B_1	$CH_2CH_2CH_2CH_3$	H	$C_{88}H_{100}N_{10}O_{28}Cl_2 \cdot 1.6\ HCl$	1816.7
B_2	$CH_2CH(CH_3)_2$	CH_3	$C_{89}H_{102}N_{10}O_{28}Cl_2 \cdot 1.6\ HCl$	1830.7

*Anhydrous free base

4.1.1 Formulation, Packaging, and Labeling

Dalbavancin

Dalbavancin is supplied in clear glass vials as a sterile, lyophilized, preservative-free, white to off-white to pale yellow solid. Each vial contains dalbavancin HCl equivalent to 500 mg of dalbavancin.

Investigational dalbavancin vials will be labeled according to manufacturer or regulatory specifications and include the statement "*Caution: New Drug – Limited by Federal Law to Investigational Use*." The dispensed study product (IV bags) will be labeled with the cautionary statement "For Investigational Use Only."

The SOC antibiotics (Cefazolin, nafcillin, oxacillin, vancomycin, and daptomycin) will be prepared and labeled in accordance with the clinical site's pharmacy standard operating procedures (SOPs).

4.1.2 Product Storage and Stability

Dalbavancin

Unreconstituted dalbavancin for injection should be stored at 25°C (77°F); excursions permitted to 15°C to 30°C (59°F to 86°F) [see USP Controlled Room Temperature].

Reconstituted vials may be stored either refrigerated at 2°C to 8°C (36°F to 46°F), or at controlled room temperature 20°C to 25°C (68°F to 77°F). Do not freeze.

Once diluted into an intravenous bag , dalbavancin may be stored either refrigerated at 2°C to 8°C (36°F to 46°F), or at controlled room temperature 20°C to 25°C (68°F to 77°F). Do not freeze.

The total time from reconstitution to dilution to administration should not exceed 48 hours.

SOC Antibiotics: Cefazolin, nafcillin, oxacillin, vancomycin, and daptomycin

Store per manufacturer's instructions.

4.2 Acquisition/Distribution

Dalbavancin for injection will be supplied by Allergan, a subsidiary of AbbVie and will be obtained from the DMID Clinical Materials Services (CMS), Fisher BioServices, upon request by the study sites and approval by DMID.

SOC antibiotics (cefazolin, nafcillin, oxacillin, vancomycin, and daptomycin) specified for use in the study will be obtained locally by each participating site.

Any diluents or other vehicles for preparation of dalbavancin and SOC antibiotics for use in the study will also be obtained locally by each participating site.

4.3 Dosage/Regimen, Preparation, Dispensing and Administration of Study Intervention/Investigational Product

Dalbavancin Group

For subjects randomized to the dalbavancin treatment group, the dosage of dalbavancin administered will be determined based on individual estimated serum creatinine clearance (CrCl) levels as follows:

Subjects with CrCl ≥ 30 mL/min and subjects receiving regular hemodialysis or peritoneal dialysis will receive 1500 mg IV dalbavancin over 30 (± 10) minutes on Day 1 and on Day 8.

Subjects with CrCl < 30 mL/min who are not receiving regular hemodialysis or peritoneal dialysis will receive 1125 mg IV dalbavancin over 30 (± 10) minutes on Day 1 and on Day 8.

A repeat serum creatinine will be required within the 72 hours prior to the second (Day 8) dalbavancin dose. Whether creatinine clearance needs to be reassessed on Day 8 will be left to the clinical judgment of the study investigator. For example, a subject with stable renal function up to 72 hours before the second dalbavancin dose and no anticipated change to renal function may not require a repeat creatinine clearance measurement on Day 8. In contrast, if the subject had either fluctuating renal function or renal function near the threshold for dose adjustment, it would be entirely appropriate to repeat a creatinine measurement prior to the Day 8 dose.

If CrCl crosses the 30ml/min threshold in either direction after Day 1 but before the Day 8 dose, then the dose should be adjusted accordingly. That is, if a subject has CrCl ≥ 30 mL/min on Day 1 and received 1500mg IV dalbavancin, but the CrCl is < 30 mL/min on day 8, then that subject will receive 1125mg IV for the Day 8 dose. If the CrCl improves to ≥ 30 mL/min between Day 1 and Day 8, then the dose will receive 1500mg IV dalbavancin for the Day 8 dose.

Standard of Care Group

Subjects randomized to the standard of care antibiotic therapy treatment group will receive an antibiotic considered standard of care, generally for a duration of 4 to 6 weeks (but up to a maximum of 8 weeks, which may be standard practice for osteomyelitis at some sites) and based on the results of antibiotic susceptibility testing for baseline pathogen. The site PI (or licensed sub-investigator listed on the Form FDA 1572), in agreement with the patient's treating clinician, will designate which antibiotic is considered the study drug for patients randomized to the standard of care group; this will generally be the antibiotic already being used to treat their infection. For example, a patient randomized to the standard of care group who is receiving vancomycin for treatment of bacteremia, would most likely continue to be treated with vancomycin and this would be designated as their study drug. If the treating clinician is changing the antibiotic regimen at the time of randomization, for example to an antibiotic that would be easier to administer in the outpatient setting, then that new antibiotic would be designated as the study drug.

Any changes to study drug (discontinuation or dose adjustment) will be captured and the reason for this change will be recorded.

For subjects randomized to standard of care antibiotic therapy, the counting of days for the duration of therapy may begin on the first day on which blood cultures are negative during the Induction Period.

Subjects randomized to the standard of care antibiotic therapy treatment group will receive the following antibiotic(s) shown in Table 5 for the specified duration based on baseline pathogen:

Table 5: Acceptable SOC Antibiotics

Baseline Pathogen	Standard of Care Therapy (doses may be adjusted for renal function)
MSSA[a]	nafcillin (2 g IV q4h × 4-6 weeks[b])<br>OR<br>oxacillin (2 g IV Q4h x 4-6 weeks[b])<br>OR<br>cefazolin (2 g IV q8h × 4-6 weeks[b])
MRSA	vancomycin (dose per local standard of care × 4-6 weeks[b])<br>[c] OR<br>daptomycin (6-10 mg/kg IV daily × 4-6 weeks[b])

IV = intravenous; qxh = every x hours; MRSA = methicillin--resistant *Staphylococcus aureus*; MSSA = methicillin--susceptible *Staphylococcus aureus*

[a] Vancomycin or daptomycin are also appropriate for patients with MSSA and anaphylactoid-type hypersensitivity to beta-lactams.

[b] Duration of antibiotics reflects usual standard of care for complicated SAB. It may be extended to a maximum of 8 weeks at the discretion of the treating clinician.

[c] Patients on vancomycin will have dose adjustment and monitoring based on local standard of care

Complete instructions for dosage, preparation, labeling, storage, stability, and administration for dalbavancin and SOC antibiotics are provided in the protocol-specific Manual of Procedures (MOP).

4.4 Pre-determined Modification of Study Intervention/Investigational Product for an Individual Subject

Dalbavancin dose will be modified per package insert for renal function only. Any contraindication to receipt of the second dose, including an allergic reaction, pregnancy or other adverse event (see individual halting rules in Section 8.5.2), should be discussed with the DMID Medical Officer.

Subjects with impaired renal function will have their dosage of standard of care antibiotic treatment adjusted as needed, based on local standard of care.

Any alteration in study drug therapy because of unusual clinical circumstances must be discussed with the DMID Medical Officer.

4.5 Accountability Procedures for the Study Intervention/Investigational Product(s)

Dalbavancin will be stored and shipped from the DMID contract Clinical Material Services (CMS) to the Clinical Sites. Once received, dalbavancin will be stored in and dispensed by the Investigational Pharmacy.

The Food and Drug Administration (FDA) requires accounting for the disposition of all investigational products. The Investigator is responsible for ensuring that a current record of product disposition is maintained and product is dispensed only at an official study site by authorized personnel as required by applicable regulations and guidelines. Records of product disposition, as required by federal law, consist of the date received, date administered, quantity administered, and the subject number to whom the drug was administered.

The Investigational Pharmacist will be responsible for maintaining accurate records of the shipment and dispensing of the investigational product. The pharmacy records must be available for inspection by the DMID monitoring contractors, and is subject to inspection by a regulatory agency (e.g., FDA) at any time. An assigned Study Monitor will review the pharmacy records.

Unused unreconstituted investigational product (dalbavancin) vials will be stored at 25°C (77°F); excursions permitted to 15°C to 30°C (59°F to 86°F) [see USP controlled room temperature] in the Investigational Pharmacy until clinical trial accountability is completed. At study termination, all unused investigational product will be disposed in accordance with the MOP following complete drug accountability and monitoring.

5 SELECTION OF SUBJECTS AND STUDY ENROLLMENT AND WITHDRAWAL

Subject Inclusion and Exclusion Criteria must be confirmed by a study investigator listed on the Form FDA 1572.

No exemptions are granted on Subject Inclusion/Exclusion Criteria in DMID-sponsored studies. Clarifications regarding applicability of specific inclusion and exclusion criteria may be discussed with a protocol clinician. Questions about eligibility will also be directed toward the DMID Medical Officer.

5.1 Eligibility Criteria

5.1.1 Subject Inclusion Criteria

A subject must meet all of the following criteria to be considered eligible for inclusion in the study:

1. Written informed consent obtained from the patient or legally authorized representative before the initiation of any study-specific procedures.
2. Patients ≥ 18 years old.
3. A diagnosis of complicated *S. aureus* (either MSSA or MRSA) bloodstream infection (see definition of uncomplicated bacteremia in Exclusion criterion #1).
4. Treated with effective antibiotic therapy for at least 72 hours (maximum 10 days).
5. Subsequent defervescence for at least 24 hours and clearance of bacteremia from the qualifying pathogen (at Screening), with negative blood culture incubated for at least 48 hours.[1]
6. Provider willing to treat with either dalbavancin for two doses, or standard of care intravenous monotherapy for at least 4 and *no more than* 8 weeks from randomization.

[1] Two negative blood cultures incubated for 48 hours are preferred. However, if only a single blood culture set is drawn, no growth at 48 hours will be considered adequate to demonstrate clearance. If more than one culture set is drawn, *all* must show no growth at 48 hours to be considered evidence of clearance (e.g., 1 of 2 positive cultures would still be considered as ongoing bacteremia).

7. Patients must be willing and able, if discharged, to return to the hospital or designated clinic for scheduled treatment, laboratory tests, or other procedures as required by the protocol.
8. According to the site PI or sub-investigator assessment, patients must be expected to survive with appropriate antibiotic therapy and appropriate supportive care throughout the study.

5.1.2 Subject Exclusion Criteria

1. Uncomplicated bacteremia[2]Appendix B.
2. Infectious CNS events, including septic emboli, ischemic or hemorrhagic stroke, epidural abscess, or meningitis (prior/unrelated CNS events are **not** exclusion criteria).
3. Known or suspected left-sided endocarditis or presence of a perivalvular abscess.
4. Planned right-sided valve replacement surgery in the first 3 days following randomization.
5. Presence of prosthetic heart valve, cardiac device[3] UNLESS removal is planned within 4 days post-randomization.
6. Presence of intravascular graft or intravascular material (excluding cardiac stents) UNLESS removal is planned within 4 days post-randomization
7. Infected prosthetic joint or extravascular hardware UNLESS removal is planned within 4 days post-randomization OR hardware was placed >60 days before bacteremia and clinically appears uninfected.
8. Polymicrobial bacteremia unless the non-S. aureus organism is a contaminant [see Definitions (Appendix B)].[4]

---

[2] Uncomplicated *Staphylococcus aureus* bacteremia is defined as all of the following: exclusion of endocarditis by echocardiography; catheter-associated bacteremia and removal of catheter; no implanted prostheses; follow-up blood cultures drawn within 48 hours after initial set that do not grow screening pathogen and all follow-up blood cultures thereafter do not grow the screening pathogen; defervescence within 72 hours of initiating effective therapy; and no evidence of metastatic sites of infection.
[3] Implantable cardioverter defibrillator (ICD), permanent pacemaker, valve support ring, ventricular assist device (VAD)
[4] *Note:* If a gram-negative bacteremia or fungemia develops after the qualifying *S. aureus* blood culture, AND the patient does not have right-sided endocarditis, AND the infection can be treated with an antibiotic without efficacy against the patient's *S. aureus* isolate (e.g. aztreonam), then the patient may remain eligible. Discussion with the DMID Medical Officer is strongly encouraged.

9. Significant hepatic insufficiency (Child-Pugh class C or AST/ALT values >5x ULN at the time of randomization).
10. Immunosuppression.[5]
11. History of hypersensitivity reaction to dalbavancin or other drugs of the glycopeptide class of antibiotics.
12. Treatment with either dalbavancin or oritavancin in the 60 days prior to enrollment.
13. Infection with S. aureus not susceptible to dalbavancin (dalbavancin mean inhibitory concentration [MIC] > 0.25 µg/mL) or vancomycin (vancomycin MIC > 2 µg/mL).
14. Planned treatment with concomitant systemic antibacterial therapy with potential efficacy against the patient's qualifying S. aureus isolate, other than that allowed in the protocol.
15. Pregnant/ nursing females.
16. Females of childbearing potential must have a negative pregnancy test[6] within 48h of randomization and use effective contraception for trial duration (Appendix B).
17. Other medical or psychiatric condition that may, in the judgment of the investigator, increase the risk of study participation or interfere with interpretation of study results.
18. Unwilling or unable to follow study procedures.
19. Treatment with an investigational drug within 30 days preceding the first dose of study medication.

5.2 Withdrawal from the Study, Discontinuation of Study Product, or Study Termination

5.2.1 Withdrawal from the Study or Discontinuation of the Study Product

Subjects may voluntarily withdraw their consent for study participation at any time without penalty or loss of benefits to which they are otherwise entitled.

An investigator may also discontinue a subject from receiving the study product for any reason. Follow-up safety evaluations for discontinued subjects will be conducted, if the subject agrees. If a

[5] On chemotherapy or immunotherapy for active hematologic malignancy expected to cause ANC < 500 cells/mm^3 lasting > 7 days during the study period, recent bone marrow transplant (in the past 90 days), solid organ transplantation within prior 3 months or receipt of augmented immunosuppression for rejection within 3 months,
chronic high dose oral steroids (equivalent of ≥ 20 mg prednisolone per day for or equivalent, for >2 weeks within the last month), chronic granulomatous disease, HIV infection with a CD4 cell count < 100 cells/mm^3 based on last known measurement or patient-reported value

[6] If the serum pregnancy test results cannot be obtained before randomization, a urine pregnancy test may be used for enrollment

subject withdraws or is withdrawn prior to completion of the study, the reason for this decision must be recorded in the case report forms (CRFs).

The reasons to withdraw from the study might include, but are not limited to, the following:

- Subject no longer meets eligibility criteria
- Subject withdraws consent
- Subject lost to follow-up
- Subject becomes non-compliant
- Medical disease or condition, or new clinical finding(s) for which continued participation, in the opinion of the investigator might compromise the safety of the subject, interfere with the subject's successful completion of this study, or interfere with the evaluation of responses
- Study or site prematurely terminated by the sponsor for any reason

The reasons to discontinue study product might include, but are not limited to, the following:

- Subject meets individual halting criteria (see Section 8.5.2)
- Subject becomes pregnant, if applicable
- Occurrence of an AE that, in the opinion of the investigator, warrants the subject's permanent discontinuation from IV study drug
- Subject has an insufficient therapeutic response to study drug (i.e., lack of efficacy for SAB). A patient who does not show signs of improvement despite treatment with study drug for an appropriate length of time or a patient who shows signs of clinical worsening at any time may be prematurely discontinued from study drug therapy and treated with salvage therapy as directed by their treating clinician. These patients would still continue to be followed in the study unless withdrawn for another reason.

A subject who is prematurely discontinued from study drug or withdrawn from the study should have the assessments for Early Termination (ET) as detailed in the Schedule of Events (Appendix A). A clear description of reason for early withdrawal or discontinuation from investigational product must be documented. The reasons for early withdrawal or premature discontinuation from study drug will be reflected on the relevant disposition page of the electronic case report form (eCRF).

The investigator should be explicit regarding study follow-up (e.g. safety and efficacy follow-up) that might be carried out despite the fact the subject will not receive further study product. If the subject consents, every attempt will be made to follow all AEs through resolution. The procedures

that collect safety data for the purposes of research must be inclusive in the original informed consent.

The investigator will inform the subject that already collected data will be retained and analyzed even if the subject withdraws from this study.

5.2.2 Subject Replacement

Subjects who withdraw, or are withdrawn from this study, or are lost to follow-up after signing the informed consent form (ICF) and administration of the study product will not be replaced. Subjects who withdraw, or are withdrawn from this study, or are lost to follow-up after randomization but before administration of the study product will not be replaced.

5.2.3 Study Termination

The sponsor reserves the right to terminate the study in its entirety or at a specific study center before study completion.

If the study is prematurely terminated by the sponsor, any regulatory authority, or the investigator for any reason, the investigator will promptly inform the currently enrolled study subjects and assure appropriate therapy or follow-up for the subjects, as necessary. The investigator will provide a detailed written explanation of the termination to the IRB/IEC.

6 STUDY PROCEDURES

The study schedule is outlined below and in the Schedule of Events (Appendix A). The descriptions of the procedures to be performed at each visit are provided below.

6.1 Induction Period: Visit 0 (Pre-screening, Day -10 to Day 1)

During the pre-screening period (Visit 0), chart review may be performed to determine if a patient is potentially eligible for the study. No study-related procedures will be requested or performed prior to obtaining informed consent:

- Confirm the subject is potentially eligible for the study based on chart review of inclusion/exclusion criteria

6.2 Screening: Visit 1 (Day -1 to Day 1)

- Obtain informed consent
- Obtain medical and surgical history, to include targeted/pertinent medical and surgical history only
- Collect medication history (from 30 days prior to ICF signing; extended review specifically for dalbavancin or oritavancin receipt in the prior 60 days given long half-lives of these two drugs)
- Review and record relevant medical events that occurred during the induction period. Examples include source control procedures [see Definitions (Appendix B)], vascular access procedures [see Definitions (Appendix B)], and complications of pre-randomization antibiotic therapy.Collect blood samples for hematology, serum chemistry laboratory assessments, and coagulation lab tests (PT/PTT and /or INR), if not already complete per standard of care within 48 hours prior to randomization (see Section 7.2.1)
- Record most recent vital signs (blood pressure, respiration rate, pulse rate, and temperature); these must be performed within 48 hours prior to randomization
- Perform physical examination (see Section 7.1.1)
- Perform pregnancy test for women of childbearing potential (see definition in Appendix B) within 48 hours of randomization; ensure test is negative before randomization. If the serum test results cannot be obtained before randomization, a urine pregnancy test may be used for enrollment.

- Collect results of echocardiography to evaluate for endocarditis; perform an echocardiogram if it has not already been done as part of standard of care for this episode of bacteremia/endocarditis. Either a transthoracic or transesophageal echocardiogram is acceptable.
- Review concomitant medications
- Review and record any concomitant nondrug interventions (e.g., procedures performed with a goal of source control).

6.3 Open-label Treatment Period, Planned Study Visits

6.3.1 Baseline (Randomization): Visit 2 (Day 1)

After a subject signs the ICF at the Screening Visit, any additional evaluations required to confirm eligibility will be performed (see Section 6.2). Subjects that do not meet all eligibility criteria after signing informed consent, for example due to an abnormal laboratory result, will be considered screen failures. Subject eligibility should be confirmed (or reconfirmed) within a maximum of 24 hours prior to randomization.

Once eligibility is confirmed, the subject may be randomized. Enrollment of subjects will be done online using the enrollment module of Advantage eClinical. Subjects will be randomized to receive either a 2-dose regimen of dalbavancin, or to completion of 4 to 6 weeks (maximum of 8) of standard of care antibiotic therapy in a 1:1 allocation ratio based on the randomization schedule further described in Section 10.3.1.

The Baseline Visit (Visit 2) will be conducted within a maximum of 10 days of effective antibiotic therapy for the qualifying bloodstream infection, after having confirmed defervescence for at least 24 hours and clearance of bacteremia from the qualifying pathogen, with negative blood culture incubated for at least 48 hours. Study procedures will be reviewed with the patient and the caregiver, if applicable.

At Baseline (Visit 2), the following procedures will be performed:

- Review medical and surgical history.
- Review concomitant medications (medications post first dose of investigational product).
- Assess QoLs.
- Randomization.
- Measure vital signs (blood pressure, respiration rate, pulse rate, and temperature).
- Perform targeted physical examination, focused on changes from screening.

- Administer IV investigational product or standard of care antibiotic therapy according to randomization as outlined in Section 4.3. The provider should designate which antibiotic is assigned as the study drug for patients randomized to standard of care group.
- Document study drug administration.
- For subjects receiving dalbavancin, collect blood samples for pharmacokinetic parameters prior to first dose, at end of infusion (+/-10 minutes), 6 (+/- 2) hours post end of dose, 12 hours (+/- 4) hours post end of dose, 24 (+/- 6) hours post end of dose, with documented draw time and date for each sample.
- Review and record AEs after study product administration
- Review and record any concomitant nondrug interventions (e.g., procedures performed with a goal of source control).

6.3.2 Visit 3 (Day 8 ± 1 day)

At Visit 3 the following procedures will be performed:

- Review and record AEs
- Measure vital signs and perform targeted physical exam focused on changes from baseline, for patients who remain in the hospital or are receiving care at a location where research personnel can provide an in-person assessment (e.g. at an infusion center). Patients in the standard of care group who have been discharged from the hospital may have their symptoms assessed by telephone. If assessed by telephone, then all assessments for this visit will be done by phone and absence of vital signs/physical exam will not be a protocol deviation.
- For subjects receiving dalbavancin, collect blood samples for pharmacokinetic parameters prior to 2nd dose of dalbavancin on day 8 (+/- 1 day) with documented draw time and date.
- Review concomitant medications
- Document study drug administration
- Collect QoL data

6.3.3 Visit 4 (Day 22 ± 2 days)

At Visit 4 the following procedures will be performed:

- Review and record AEs
- Measure vital signs (blood pressure, respiration rate, pulse rate, and temperature) and perform targeted physical examination, focused on changes from prior examinations.

- Collect blood samples for hematology and serum chemistry
- For subjects who received dalbavancin, collect blood samples for pharmacokinetic parameters if feasible on day 22 (+/- 2 days) with documented time of draw and date.
- Review concomitant medications
- Review and record any concomitant nondrug interventions (e.g. procedures performed with a goal of source control).
- Administer IV standard of care antibiotic therapy according to randomization as outlined in Section 4.3 and document compliance.
- Collect QoL data

6.3.4 Visit 5 (Day 42 ± 3 days)

At Visit 5, the following procedures will be performed:

- Review and record AEs
- Update medical history
- Measure vital signs (blood pressure, respiration rate, pulse rate, and temperature) and perform targeted physical examination, focused on changes from prior examinations.
- Collect blood samples for hematology and serum chemistry
- For subjects who received dalbavancin, collect blood samples for pharmacokinetic parameters if feasible on day 42 (+/- 3 days) with documented time of draw and date.
- Review concomitant medications
- Review and record any concomitant nondrug interventions (e.g., procedures performed with a goal of source control).
- Administer IV standard of care antibiotic therapy according to randomization as outlined in Section 4.3 and document, if ongoing
- Complete investigator assessment of efficacy
- Collect QoL data

6.3.5 Test of Cure: Visit 6 (Day 70 ± 7 days)

It is preferred that this visit be performed in-person, however a telephone or telemedicine visit is an acceptable alternative. If performed by telephone, then the subject should be asked about new symptoms (e.g. fever, pain) in lieu of a physical exam.

At Visit 6 (Day 70), the following procedures will be performed:

- Review and record AEs
- Update medical history
- Measure vital signs (blood pressure, respiration rate, pulse rate, and temperature) for patients with in-person visit.
- Perform targeted physical examination, focused on changes from prior examinations
- For subjects who received dalbavancin, collect blood samples for pharmacokinetic parameters if feasible on day 70 (± 7 days) with documented time of draw and date.
- Review concomitant medications
- Review and record any concomitant nondrug interventions (e.g., procedures performed with a goal of source control).
- Complete investigator assessment of efficacy
- Collect QoL data

6.3.6 Visit 7 (Day 180 ± 14 days – only for subjects with vertebral osteomyelitis)

Visit 7 will only be performed for subjects with vertebral osteomyelitis in order to determine long-term recurrence risk. As with visit 6, an in-person visit will be preferred however a telephone alternative will also be considered acceptable. If performed by telephone, the subject should be asked about specific symptoms (fever, back pain), resumption/continuation/repeat treatment with antibiotics for *S. aureus* osteomyelitis, or surgical intervention for osteomyelitis.

At Visit 7 (Day 180), the following procedures will be performed:

- Measure vital signs (blood pressure, respiration rate, pulse rate, and temperature) for patients with in-person visit.
- Perform targeted physical examination, focused on changes from prior examinations (especially point tenderness to spine or presence of neurologic deficits)
- Complete investigator assessment of efficacy
- Collect QoL data
- Review concomitant medications and concomitant nondrug interventions that are related to the treatment of osteomyelitis

6.3.7 Final Study Visit

- For subjects without vertebral osteomyelitis, the final study visit will be Visit 6 (Day 70 ± 7 days) as above. For subjects with vertebral osteomyelitis, the final study visit will be Visit 7 (Day 180 ± 14 days) as above.

6.3.8 Early Termination Visit

In circumstances where a subject withdraws from the study or discontinues the study product early, an Early Termination Visit will be performed, which include the following procedures:

- Review and record AEs
- Update medical history
- Measure vital signs (blood pressure, respiration rate, pulse rate, and temperature) and perform targeted physical examination, focused on changes from prior examinations.
- If clinically indicated, blood cultures should be collected at time of treatment discontinuation or for determination of treatment failure
- Review concomitant medications
- Review and record any concomitant nondrug interventions (e.g., procedures performed with a goal of source control).
- Complete investigator assessment of efficacy
- For subjects who received dalbavancin, collect blood samples for pharmacokinetic parameters if feasible with documented time of draw and date.
- Collect QoL data

6.4 Unscheduled Study Visits

An unscheduled study visit may be initiated by the subject/subject's LAR or investigator, if a subject is not improving on therapy, has a grade 3 and above AE or an SAE, or for any other reason. In addition, the reason for the visit will be documented, including who initiated the visit, what complaints the subject/subject's LAR has, and/or what concerns the primary medical team or site investigator have. Clinical outcome and any safety assessments will be documented.

Assessments to be completed at unscheduled study visits should include:

- Review and record AEs
- Update medical history

- Measure vital signs (blood pressure, respiration rate, pulse rate, and temperature) and perform targeted physical examination, focused on changes from prior examinations.
- Review concomitant medications
- Review and record any concomitant nondrug interventions (e.g., procedures performed with a goal of source control).

6.5 Protocol Deviations

A protocol deviation is any noncompliance with the clinical trial protocol, GCP, or protocol-specific Manual of Procedures (MOP) requirements. The noncompliance may be either on the part of the subject, the investigator, or the study site staff. As a result of deviations, corrective actions should be developed by the site and implemented promptly. It is the responsibility of the site Principal Investigator and other study personnel to use continuous vigilance to identify and report protocol deviations. All individual protocol deviations will be addressed in subject study records. All protocol deviations, either individual, product, or site-specific will be collected and the record stored in a sponsor-determined location. Protocol deviations must be sent to the IRB/IEC on which it relies per its guidelines as well as to the sites' local IRB/IEC if required. The site Principal Investigator and other study personnel are responsible for knowing and adhering to the requirements of their local IRB/IEC as well as the requirements of the IRB/IEC on which it relies.

Inability to draw pharmacokinetic samples will *not* be considered a violation of protocol, as pharmacokinetic sampling is being conducted for exploratory purposes only and does not alter in any way the planned treatment course for subjects.

7 DESCRIPTION OF CLINICAL AND LABORATORY EVALUATIONS

7.1 Clinical Evaluations

7.1.1 Research Procedures

- Medical and surgical history: includes targeted/pertinent medical and surgical history only.
- Medication history: from 30 days prior to signing the ICF, and 60 days for any prior lipoglycopeptides (oritavancin or dalbavancin).
- Vital Signs: Vital sign measurements will be documented at every in-person visit. The parameters are blood pressure (BP), respiration rate, pulse rate, and temperature. It is recommended that heart rate and BP readings be taken after the patient has been sitting for at least 5 minutes.
- Physical Examination: A physical examination (including general appearance, examination of head, eyes, ears, nose, throat, neck, skin, heart, lungs, abdomen, neurologic system, musculoskeletal system, extremities, height, and body weight) will be done at Screening (Visit 1). Physical examinations should be performed by a professionally trained physician or health professional licensed to perform physical examinations. Body weight and height will be measured at Screening (Visit 1). If height or weight is not obtainable (e.g., patient is immobilized), the last known or stated height and weight may be used. At subsequent visits, a targeted physical exam should focus on changes from prior exams and on the evaluation of newly reported symptoms.
- Echocardiograms: A transthoracic echocardiogram will be performed, or if clinically indicated, a transesophageal echocardiogram will be performed, unless one has been performed as standard of care for this episode of bacteremia/endocarditis. The overall interpretation and determination of the clinical significance of echocardiography findings will be the responsibility of the investigator, and the findings of all echocardiograms will be recorded in the patient's eCRF.
- Quality of Life instruments: The following three QoL measures will be administered:
  - The ARLG Bloodstream Infection QoL Measure (Appendix C) is a 41-item questionnaire covering multiple domains of QoL.
  - The EQ-5D-5L (https://euroqol.org/eq-5d-instruments/sample-demo/) is an instrument that comprises five dimensions: mobility, self-care, usual activities, pain/discomfort, and anxiety/depression, as well as an overall self-rated health on a vertical visual analogue scale.

- The PROMIS Global Health short form (http://www.healthmeasures.net/administrator/components/com_instruments/uploads/Global%20Health%20Scale%20v1.2%2008.22.2016.pdf) is a 10-item instrument that assesses general health and five primary domains (physical function, fatigue, pain, emotional distress, and social health).

7.1.2 Assessment of Concomitant Medications/Treatments Other Than Study Product

Medication history during the 30 days prior to ICF signing will be recorded at Screening (Visit 1) in the eCRF. Thereafter, any changes in concomitant medications or new medications added will be recorded in the eCRF.

Any medication taken by the subject, other than study drugs, is considered concomitant medication. All concomitant medications from Screening (Visit 1) through Day 70 ± 7 days (Visit 6) or, for subjects with vertebral osteomyelitis, Day 180 ± 14 days (Visit 7) must be recorded in the subject's medical record and on the eCRFs.

At each visit the investigator will obtain information on any therapeutic interventions (e.g., drug and nondrug therapy or surgery) provided. The use of any other investigational drug is prohibited and subjects may not participate in any other studies involving marketed products concomitantly while in this study.

The use of other (non-antibacterial) medications should be limited to those essential for the care of the subject. All medications required by the subject to manage underlying illnesses, other than infection under study, and any drugs that may be required for emergency treatments must be recorded on the eCRF.

Concomitant systemic antibacterials (other than dalbavancin or comparator study drug) for adjunctive therapy of the subject's *S. aureus* bacteremia are prohibited during the study, up to Day 70. This includes concomitant treatment with an aminoglycoside.

Patients who require additional therapy due to inadequate clinical response will be assessed as having lack of efficacy of study drug.

- Where possible, antibiotic treatment of intercurrent infections should be done with antibiotics that are not active against the patient's *S. aureus* isolate. Discussion with the DMID Medical Officer is encouraged before or within 24 hours of initiation of concomitant antibiotics for another indication. Exceptions include: Vancomycin oral 125 mg up to 500mg every 6 hours may be used in both treatment groups for the treatment of *Clostridium difficile* infections and may be continued as required throughout the duration of the study. The sponsor will not provide oral vancomycin.

- Metronidazole IV or oral 500 mg every 8 hours may be used in both treatment groups for the treatment of *C. difficile* infections and may be continued as required throughout the duration of the study. The sponsor will not provide metronidazole.
- Other antibacterials that do not achieve therapeutic levels in the serum (e.g., nitrofurantoin) may be considered. Consultation with the DMID Medical Officer is advised before use of these antibiotics.

7.1.3 Assessment of Subject Compliance with Study Intervention/Investigational Product/Investigational Device

Dalbavancin will be administered under the supervision of investigative site personnel, and infusion date, start, and stop time will be documented in the eCRF, as well as any infusion interruptions.

7.1.4 Non-Research Standard of Care

If clinically indicated, any surgical or non-surgical procedures required to achieve infection source control should be performed for patients enrolled in either treatment group. The necessity and timing of any such procedures will be decided upon by the patient's treating physicians. Procedures frequently required for source control in *Staphylococcus aureus* bacteremia may include debridement of osteomyelitis, removal of indwelling vascular access device, prosthetic devices or materials, incision and drainage of any abscesses, or heart valve replacement.

7.2 Laboratory Evaluations

7.2.1 Clinical Laboratory Evaluations

The following laboratory evaluations will be evaluated during the study, per the schedule of events (Appendix A)

- Hematology: absolute and differential white blood cell (WBC) count, erythrocyte count, hemoglobin, hematocrit, platelet count, and red blood cell (RBC) indices (mean corpuscular volume, mean corpuscular hemoglobin, and mean corpuscular hemoglobin concentration).
- Coagulation lab tests (PT/PTT and /or INR)
- Chemistry: sodium, potassium, calcium, chloride, bicarbonate, glucose, blood urea nitrogen, creatinine, total protein, alkaline phosphatase, albumin, total bilirubin, AST, and ALT.
- If clinically indicated, blood cultures should be collected at the time of treatment discontinuation or for determination of treatment failure. Blood cultures should be repeated every 24-48 hours upon knowledge of a positive result from any visit until clearance of bacteremia is confirmed. When blood cultures are required, 2 sets of blood samples (1 aerobic and 1 anaerobic bottle) should be obtained from 2 separate venipuncture sites.

- Culture, organism identification, and susceptibility testing will be conducted at the local laboratory. All pathogens will be tested for susceptibility; microbiological specimens and isolates will be collected, processed, and stored in accordance with local procedures.
- Pregnancy test (urine or serum)
- Vancomycin levels in patients on vancomycin, based on local standard of care.

7.2.2 Research Assays

- Dalbavancin Concentration Measurements: Venous blood samples for PK analyses will be collected at Visit 2 (Day 1), Visit 3 (Day 8 ± 1 day),Visit 4 (Day 22 ± 2 days), Visit 5 (Day 42 ± 3 days), Visit 6 (Day 70 ± 7 days), or any early termination (ET) visit as outlined in Section 6 and Appendix A. The actual date and time of each blood sample collection will be recorded in the subjects' source document and the eCRF. Blood collected will be processed for plasma separation at the study site's laboratory; subsequent PK analyses will be performed on the plasma samples at a central laboratory. PK sample collection, labeling, processing, storage, and shipment instructions will be provided in the MOP.

7.2.2.1 Laboratory Specimen Preparation, Handling, and Storage

Plasma PK specimens will be processed and stored in a -70ºC freezer until time of shipment to the central PK laboratory for analysis. Detailed instructions for the preparation, handling, and storage of plasma PK specimens are detailed in the study MOP including aliquots of specimens, temperature requirements, where they will be stored, and how they will be labeled.

7.2.2.2 Laboratory Specimen Shipping

Plasma PK samples will be shipped on dry ice to the central PK laboratory. Samples will be shipped in compliance with the International Air Transport Association (IATA) regulations. Instructions for the shipment of specimens are outlined in the study MOP.

8 ASSESSMENT OF SAFETY

8.1 Assessing and Recording Safety Parameters

This study will assist in determining the safety of dalbavancin compared to that of standard of care for the treatment of complicated *S. aureus* bacteremia. Safety will be assessed by the frequency of SAEs, AESIs, and the frequency of AEs leading to study drug discontinuation in each treatment arm.

8.1.1 Adverse Events (AEs)

ICH E6 defines an AE as any untoward medical occurrence in a patient or clinical investigation subject administered a pharmaceutical product regardless of its causal relationship to the study treatment. FDA defines an AE as any untoward medical occurrence associated with the use of a drug in humans, whether or not considered drug related.

An AE can therefore be any unfavorable and unintended sign (including an abnormal laboratory finding), symptom, or disease temporally associated with the use of medicinal (investigational) product. The occurrence of an AE may come to the attention of study personnel during study visits and interviews of a study recipient presenting for medical care, or upon review by a study monitor.

All AEs of grade 3 or higher including lab abnormalities (per CTCAE table, version 5.0, https://ctep.cancer.gov/protocolDevelopment/electronic_applications/docs/CTCAE_v5_Quick_Reference_8.5x11.pdf) will be captured on the appropriate data collection form and eCRF. AESIs of all grades will also be captured (see Section 8.2.1). Information to be collected for AEs includes event description, date of onset, assessment of severity, relationship to study product and alternate etiology (assessed only by those with the training and authority to make a diagnosis and listed on the Form FDA 1572 as an investigator), date of resolution, seriousness and outcome. AEs and AESIs that meet reporting criteria and occur during the trial collection and reporting period will be documented appropriately regardless of relationship and will be followed through resolution. Resolution of an AE is defined as the return to pre-treatment status or stabilization of the condition with the expectation that it will remain chronic.

For the purpose of the site's data collection responsibilities, any untoward event that was reported from the time of first study drug dose until the final protocol-defined study visit is to be considered an AE.

Examples of AEs are as follows:

- Changes in the general condition of the patient from baseline
- Subjective symptoms offered by or elicited from the patient
- Objective signs observed by the investigator or other study center personnel

- All diseases that occur after signing the ICF, including any change in severity or frequency of pre-existing disease
- All clinically significant abnormalities in laboratory values or clinically significant physical findings that occur during the study collection and reporting period

Please note that hospital admissions and/or medical/surgical procedures scheduled prior to consenting but occurring during the study should not be captured as AEs, but should be listed in the medical history if related to a pre-existing condition.

Any medical condition that is present at the time that the subject is screened will be considered as baseline and not reported as an AE. However, if the severity of any pre-existing medical condition increases, it should be recorded in source documents as an AE. In this trial, only Grade 3 and higher AEs and AESIs will be captured in the eCRF.

Any abnormal test result that is determined to be an error does not require reporting as an AE. Additional diagnostic testing or medical/surgical interventions that occur as a result of an AE due to an abnormal laboratory test finding should be noted in the eCRF, if it meets protocol-specified reporting criteria.

The following events are captured as efficacy endpoints and are therefore excluded from AE reporting:

- An event would be considered as adequately captured in the study endpoint if it is accurately and fully represented by a protocol-defined reason for clinical failure (other than mortality) or relapse. Events represented by the study endpoints include all of the following:
    - Worsening of signs and symptoms of complicated bacteremia or IE
    - Persistent bacteremia
    - Relapse of baseline bacteremia

8.1.1.1 Adverse Events Grading

All AEs (laboratory and clinical symptoms) will be graded for severity (per CTCAE table, version 5.0, https://ctep.cancer.gov/protocolDevelopment/electronic_applications/docs/CTCAE_v5_Quick_Reference_8.5x11.pdf) and assessed for relationship to study product as outlined below. AEs characterized as intermittent require documentation of onset and duration of each episode. The start and stop date of each reported AE will be recorded on the appropriate data collection form and eCRF. Changes in the severity of an AE will be documented in the source documents at the site to allow an assessment of the duration of the event at each level of intensity.

Severity of Event:

AEs will be assessed by the investigator (those with the training and authority to make a diagnosis and listed on the Form FDA 1572) using the CTCAE, version 5.0 (https://ctep.cancer.gov/protocolDevelopment/electronic_applications/docs/CTCAE_v5_Quick_Reference_8.5x11.pdf). The investigator will provide an assessment of the severity of each AE by recording a severity rating on the appropriate source documentation. *Severity,* which is a description of the intensity of manifestation of the AE, is distinct from *seriousness,* which implies a patient outcome or AE-required treatment measure associated with a threat to life or functionality (Section 8.1.2). Severity will be assessed according to CTCAE, version 5.0 (https://ctep.cancer.gov/protocolDevelopment/electronic_applications/docs/CTCAE_v5_Quick_Reference_8.5x11.pdf), which follows the following general guideline:

- Grade 1 Mild; asymptomatic or mild symptoms; clinical or diagnostic observations only; intervention not indicated.
- Grade 2 Moderate; minimal, local or noninvasive intervention indicated; limiting age-appropriate instrumental activity of daily living (ADL).
- Grade 3 Severe or medically significant but not immediately life-threatening; hospitalization or prolongation of hospitalization indicated; disabling; limiting self-care ADL.
- Grade 4 Life-threatening consequences; urgent intervention indicated.
- Grade 5 Death related to AE.

Relationship to Study Product: The assessment of the AE's relationship to study product will be done by a licensed study investigator indicated on the Form FDA 1572 and the assessment will be part of the documentation process. Whether the AE is related or not, is not a factor in determining what is or is not reported in this trial. If there is any doubt as to whether a clinical observation is an AE, the event should be reported.

In a clinical trial, the study product must always be suspect. The relationship to study product will be assessed for AEs using the terms related or not related:

- Related – There is a reasonable possibility that the study product caused the AE. Reasonable possibility means that there is evidence to suggest a causal relationship between the study product and the AE.
- Not Related – There is not a reasonable possibility that the administration of the study product caused the event.

8.1.2 Serious Adverse Events (SAEs)

An AE or suspected adverse reaction is considered a serious adverse event (SAE) if, in the view of either the site principal investigator or sponsor, it results in any of the following outcomes:

- Death,
- a life-threatening adverse event[1],
- inpatient hospitalization or prolongation of existing hospitalization,
- a persistent or significant incapacity or substantial disruption of the ability to conduct normal life functions, or
- a congenital anomaly/birth defect.
- Important medical events that may not result in death, be life-threatening, or require hospitalizations may be considered serious when, based upon appropriate medical judgment they may jeopardize the patient or subject and may require medical or surgical intervention to prevent one of the outcomes listed in this definition. Examples of such medical events include allergic bronchospasm requiring intensive treatment in an emergency room or at home, blood dyscrasias or convulsions that do not result in inpatient hospitalization, or the development of drug dependency or drug abuse.

[1] Life-threatening adverse event. An AE is considered "life-threatening" if, in the view of either the site principal investigator or sponsor, its occurrence places the patient or subject at immediate risk of death. It does not include an AE that, had it occurred in a more severe form, might have caused death.

SAEs will be:

- Assessed for severity and relationship to study product and alternate etiology (if not related to study product) by a licensed study physician listed on the Form FDA 1572 or by the Institution as the site Principal Investigator or Sub-Investigator.
- Recorded on the appropriate SAE data collection form and eCRF.
- Followed through resolution by a licensed study physician (for IND studies, a physician listed on the Form FDA 1572 as the site Principal Investigator or Sub-Investigator).
- Reviewed and evaluated by DMID, the DSMB (periodic review unless related), and the IRB/IEC.

8.2 Specification of Safety Parameters

Safety will be assessed by the frequency and severity of AEs, AESIs, and SAEs.

Safety will be assessed using descriptive statistics of AEs, vital signs, and laboratory tests by treatment group. For each safety parameter, the last assessment made before the first dose of investigational product will be used as the baseline for all analyses of that safety parameter.

8.2.1 Adverse Events of Special Interest (AESIs)

Adverse events of special interest (AESIs) are AEs that are common and known to occur following administration of study product. AESIs will be collected after first dose of study product is given and until final study visit. AESIs will be recorded on the eCRF and entered into the EDC. AESIs will include any CTCAE grades of allergic reaction, catheter-related infection, vascular access complication, infusion site extravasation, infusion-related reaction, alanine aminotransferase increases, aspartate aminotransferase increases, and acute kidney injury.

8.3 Reporting Procedures

8.3.1 Reporting Serious Adverse Events

SAEs will be followed until resolution even if this extends beyond the study-reporting period. Resolution of an AE is defined as the return to pretreatment status or stabilization of the condition with the expectation that it will remain chronic.

Any AE that meets a protocol-defined serious criterion must be submitted immediately (within 24 hours of site awareness) on an SAE form to the DMID Pharmacovigilance Group, at the following address:

DMID Pharmacovigilance Group

Clinical Research Operations and Management Support (CROMS)

6500 Rock Spring Dr. Suite 650

Bethesda, MD 20817, USA

SAE Hot Line: 1-800-537-9979 (US) or 1-301-897-1709 (outside US)

SAE FAX Number: 1-800-275-7619 (US) or 1-301-897-1710 (outside US)

SAE Email Address: PVG@dmidcroms.com

In addition to the SAE form, select SAE data fields must also be entered into the data coordinating center (DCC) system. Please see the protocol-specific MOP for details regarding this procedure.

Other supporting documentation of the event may be requested by the DMID Pharmacovigilance Group and should be provided as soon as possible.

The DMID Medical Monitor and DMID Clinical Project Manager will be notified of the SAE by the DMID Pharmacovigilance Group. The DMID Medical Monitor will review and assess the SAE for regulatory reporting and potential impact on study subject safety and protocol conduct.

At any time after completion of the study, if the site principal investigator or appropriate sub-investigator becomes aware of an SAE that is suspected to be related to study product, the site principal investigator or appropriate sub-investigator will report the event to the DMID Pharmacovigilance Group.

8.3.2 Regulatory Reporting for Studies Conducted Under DMID Sponsored IND

Following notification from the site Principal Investigator or appropriate sub-investigator, DMID, as the IND sponsor, will report any suspected unexpected serious adverse event. DMID will report an AE as a suspected unexpected adverse event only if there is evidence to suggest a causal relationship between the study intervention and the AE. DMID will submit an IND safety report to the FDA and will notify all participating site Principal Investigators (i.e., all Principal Investigators to whom the sponsor is providing drug under its IND(s) or under any Principal Investigator's IND(s) of potential serious risks from clinical studies or any other source, as soon as possible. DMID will report to the FDA any unexpected fatal or life-threatening suspected adverse reaction as soon as possible, but in no case later than 7 calendar days after the sponsor's initial receipt of the information. If the event is not fatal or life-threatening the IND safety report will be submitted within 15 calendar days after the sponsor determines that the information qualifies for reporting as specified in 21 CFR Part 312.32. Relevant follow up information to an IND safety report will be submitted as soon as the information is available. Upon request from FDA, DMID will submit to the FDA any additional data or information that the agency deems necessary, as soon as possible, but in no case later than 15 calendar days after receiving the request.

All SAEs designated as "not related" to study product(s), will be reported to the FDA at least annually in a summary format.

8.3.3 Reporting of Pregnancy

Female subjects of childbearing age who become pregnant during the study prior to day 8 will not receive the second dose of dalbavancin (if randomized to the dalbavancin group) and will be followed for pregnancy outcome. Pregnancy occurring during a clinical investigation, although not considered a SAE, must be reported within the same timelines as a SAE. The positive pregnancy test

will be recorded in the database within 5 days of site awareness, on the Pregnancy Report form. The report will include pregnancy outcome (e.g., any premature terminations, elective or therapeutic, any spontaneous abortions or stillbirths), as well as the health status of the mother and child, including date of delivery and infant's sex and weight. Any subject with a positive pregnancy test, who has received study product(s), will be followed through eight (8) weeks post-live delivery or elective or natural termination of the pregnancy, whichever occurs first. If the database is locked at time of pregnancy, a supplemental report will be generated and completed after birth, which will be appended to the database. Any occurring AEs or SAEs that occur to the mother or fetus will be recorded in the eCRF in the database and on the SAE Report form.

The site is responsible for notifying their local IRB of any pregnancies in accordance with local policies.

8.4 Type and Duration of Follow-up of Subjects after Adverse Events

AEs will be assessed, and AEs that are CTCAE grade 3 and higher, as well as all AESIs, will be followed from initial recognition of the AE until resolution.

SAEs will be followed up through resolution even if duration of follow-up goes beyond the protocol-defined follow-up period (test of cure).

Resolution of an AE is defined as the return to pre-treatment status or stabilization of the condition with the expectation that it will remain chronic.

8.5 Halting Rules

8.5.1 Study Halting Criteria

The halting rules outlined below will be used to evaluate whether it is safe to proceed with dosing or whether the study should be suspended for further safety evaluation.

If any of the halting rules below are met, further enrollment will be halted pending DSMB review of safety data and recommendations. The continuation of the administration of dalbavancin (the second dose) or switch to SOC will be at the site PI discretion.

- More than one subject **death** (e.g., 2 or more deaths) during participation in the study that is **suspected to be related** to the treatment with **dalbavancin**. Note that mortality among patients with *S. aureus* bacteremia is not uncommon, and deaths that are not directly and solely related to dalbavancin will not by themselves trigger this halting rule.
- Five or more subjects in the study are suspected with development of drug induced liver injury (DILI) that are assessed as **related** to **dalbavancin.**

- Five or more subjects in the study experience a Grade 4 **related** to **dalbavancin** AEs (laboratory or systemic) that are coded in the same HLGT per MedDRA classification.

8.5.2 Individual Halting Rules

A subject will be discontinued from further dosing in either treatment group if any of the following criteria are met:

- An individual infusion must be stopped if a drug-related hypersensitivity (grade 2 or higher) to dalbavancin or standard of care study drug is suspected, including anaphylaxis. Note that dalbavancin infusion can cause reactions that resemble "Red-Man Syndrome," including flushing of the upper body, urticaria, pruritus, and/or rash. Stopping or slowing the infusion may result in cessation of these reactions and this reaction is not by itself a reason to discontinue dalbavancin for an individual subject.
- New onset of illness or condition that meets exclusion criteria, at the investigator's discretion
- The treatment of any subject may be stopped for SAEs, clinically significant adverse events, including severe laboratory abnormalities that indicate to the Investigator that continued dosing is not in the best interest of the patient.

8.6 Safety Oversight

8.6.1 Data and Safety Monitoring Board (DSMB)

Safety oversight will be conducted by a DSMB that is an independent group with expertise to interpret data from this study and will monitor subject safety and advise DMID. The DSMB members will be separate and independent of study personnel participating in the study and should not have scientific, financial, or other conflict of interest related to this study. DSMBs must consist of at least three voting members, including a biostatistician experienced in statistical methods for clinical trials and a clinician with relevant expertise.

The DSMB will operate under the rules of a DMID-approved charter that defines the data elements to be assessed and the procedures for data reviews and will be written at the organizational meeting of the DSMB. Procedures for DSMB reviews/meetings will be defined in the charter. Reports may include enrollment and demographic information, medical history, concomitant medications, physical assessments, clinical laboratory values, dosing compliance, and solicited and unsolicited AE/SAEs. The DSMB will review SAEs on a regular basis and ad hoc during this trial. The DMID Medical Monitor will be responsible for reviewing SAEs in real time.

As defined in the charter, the DSMB will review data at specified times during the course of the study for subject and overall study progress and will conduct ad hoc reviews as appropriate when a halting rule is met or for immediate concerns regarding observations during this study.

The DSMB will conduct the following meetings:

- Organizational meeting
- Data review meeting for safety
- Every year
- An Interim analysis for efficacy after at least approximately 50% of subjects have completed the study
- Ad hoc meetings will occur when a halting rule is met, or when DMID or the DSMB chair has immediate concerns regarding observations during the trial
- Final review meeting will occur 6-8 months after the clinical database is locked to review cumulative safety and efficacy data. The final CSR, if available, will be provided for this review. The DSMB may be asked to provide recommendations in response to DMID's questions.

Additional data may be requested by the DSMB, and interim statistical reports may be generated as deemed necessary and appropriate by DMID. The DSMB may receive data in aggregate and presented by treatment arm. The DSMB may also be provided with expected and observed rates of the expected AEs. The DSMB will review grouped data in the closed session only. As an outcome of each review/meeting, the DSMB will make a recommendation as to the advisability of proceeding with study treatments), and to continue, modify, or terminate this trial.

9 HUMAN SUBJECTS PROTECTION

9.1 Institutional Review Board/Independent Ethics Committee

Each site principal investigator will obtain IRB approval for this protocol to be conducted at his/her research site(s) and send supporting documentation to the DMID before initiating recruitment of subjects. The investigator will submit applicable information to the IRB/IEC on which it relies for the review, to conduct the review in accordance with 45 CFR 46, ICH E6 GCP, and as applicable, 21 CFR 56 (Institutional Review Boards) and 21 CFR 50 (Protection of Human Subjects), other federal, state, and local regulations. The IRB/IEC must be registered with OHRP as applicable to the research. DMID must receive the documentation that verifies IRB/IEC-approval for this protocol, associated informed consent documents, and upon request any recruitment material and handouts or surveys intended for the subjects, prior to the recruitment and enrollment of subjects.

Any amendments to the protocol or consent materials will be approved by the IRB/IEC before they are implemented. IRB/IEC review and approval will occur at least annually throughout the enrollment and follow-up of subjects and may cease if annual review is no longer required by applicable regulations and the IRB/IEC. The investigator will notify the IRB/IEC of deviations from the protocol and reportable SAEs, as applicable to the IRB/IEC policy.

Each institution engaged in this research will hold a current FWA issued by the Office of Human Research Protection (OHRP) for federally funded research.

A single IRB of record, (WIRB Copernicus Group), will be accountable for compliance with regulatory requirements for this multi-centered study, at participating sites. A formal Reliance Agreement will be required between the single IRB and participating sites. The formal Reliance Agreement will set forth the specific responsibilities of the IRB and each participating site. Participating sites will then rely on the IRB of record to satisfy the regulatory requirements relevant to the IRB review. The participating sites will maintain essential required documentation of IRB reviews, approvals, and correspondence, and must provide copies of any agreements and essential documentation to the DMID or regulatory authorities upon request.

9.2 Informed Consent Process

Informed consent is a process that is initiated prior to an individual agreeing to participate in a trial and continuing throughout the individual's trial participation. Before any study procedures are performed, informed consent will be obtained and documented. Subjects will receive a concise and focused presentation of key information about the clinical trial, verbally and with a written consent

form. The explanation will be organized, and presented in lay terminology and language that facilitates understanding why one might or might not want to participate.

An investigator or designee will describe the protocol to potential subjects face-to-face. The key information about the purpose of the study, the procedures and experimental aspects of the study, risks and discomforts (including the PK sampling plan for subjects receiving dalbavancin), any expected benefits to the subject, and alternative treatment will be presented first to the subject.

Subjects will also receive an explanation that the trial involves research, and a detailed summary of the proposed study procedures and study interventions/products. This will include aspects of the trial that are experimental, the probability for random assignment to treatment groups, any expected benefits, all possible risks (including a statement that the particular treatment or procedure may involve risks to the subject or to the embryo or fetus, if the subject is or may become pregnant, that are currently unforeseeable), the expected duration of the subject's participation in the trial, alternative procedures that may be available and the important potential benefits and risks of these available alternative procedures.

Subjects will be informed that they will be notified in a timely manner if information becomes available that may be relevant to their willingness to continue participation in the trial. Subjects will receive an explanation as to whether any compensation and any medical treatments are available if injury occurs, and, if so, what they consist of, or where further information may be obtained. Subjects will be informed of the anticipated financial expenses, if any, to the subject for participating in the trial, as well as any anticipated prorated payments, if any, to the subject for participating in the trial. They will be informed of whom to contact (e.g., the investigator) for answers to any questions relating to the research project.

Information will also include the foreseeable circumstances and/or reasons under which the subject's participation in the trial may be terminated. The subjects will be informed that participation is voluntary and that they are free to withdraw from the study for any reason at any time without penalty or loss of benefits to which the subject is otherwise entitled.

The extent of the confidentiality of the subjects' records will be defined, and subjects will be informed that applicable data protection legislation will be followed. Subjects will be informed that the monitor(s), auditors(s), IRB, NIAID, and regulatory authority(ies) will be granted direct access to the subject's original medical records for verification of clinical trial procedures and/or data without violating the confidentiality of the subject, to the extent permitted by the applicable laws and regulations, and that, by signing a written informed consent form, the subject is authorizing such access.

Subjects will be informed that records identifying the subject will be kept confidential, and, to the extent permitted by the applicable laws and/or regulations, will not be made publicly available and, if the results of the trial are published, the subject's identity will remain confidential. Subjects will

be informed whether private information collected from this research and/or specimens will be used for additional research, even if identifiers are removed.

Subjects will be allowed sufficient time to consider participation in this research trial, and have the opportunity to discuss this trial with their family, friends or legally authorized representative, or think about it prior to agreeing to participate.

Informed consent forms will be IRB-approved and subjects will be asked to read and review the consent form. Subjects must sign the informed consent form prior to starting any study procedures being done specifically for this trial.

Once signed, a copy of the informed consent form will be given to the subject(s) for their records. The subject(s) may withdraw consent at any time throughout the course of the trial. The rights and welfare of the subject(s) will be protected by emphasizing to them that the quality of their medical care will not be adversely affected if they decline to participate in this study.

Study personnel may employ recruitment efforts prior to obtaining study consent if a patient-specific screening consent is on record or if the IRB has agreed that chart review is allowed without a fully executed screening consent. In cases where there is not a patient-specific screening consent on record, site Clinical staff may pre-screen via chart review and refer potential subjects to the Research staff. Research staff would obtain written consent per the standard informed consent process before conducting protocol-specific screening activities.

New information will be communicated by the site principal investigator to subjects who consent to participate in this trial in accordance with IRB requirements. The informed consent document will be updated and subjects will be re-consented per IRB requirements, if necessary. Subjects will be given a copy of all informed consent forms that they sign.

9.2.1 Other Informed Consent Procedures

Use of a Legally Authorized Representative (LAR)

Potential subjects for this study are adults but may be unable to provide legally effective informed consent due to their health status (*i.e.*, dementia, intubated, sedated). The subjects may be enrolled in the study if consent is obtained from the LAR. The investigator will be familiar with the IRB/IEC policy on which he/she relies regarding the priority list of LAR and whether enrollment in a study is permitted by an advanced directive *(e.g.*, living will, durable power of attorney for proxy consent). Additionally, subjects will be informed about the study to the extent compatible with the person’s understanding, and enrollment declined if the subject refuses participation.

If a LAR originally provides legally effective informed consent and the subject’s condition improves, the subject will also be informed about the study as soon as is feasible and will be re-consented. The subject may continue in the study only if the subject’s consent is provided.

9.3 Exclusion of Women, Minorities, and Children (Special Populations)

Children will be excluded from this trial. Management algorithms for *S. aureus* bacteremia are different in children and their participation would thus not be appropriate.

9.4 Subject Confidentiality

Subject confidentiality is strictly held in trust by the participating investigators, their staff, and the sponsor(s) and their agents. This confidentiality includes documentation, investigation data, subject's clinical information, and all other information generated during participation in the study. No information concerning the study or the data generated from the study will be released to any unauthorized third party without prior written approval of the DMID and the subject. Subject confidentiality will be maintained when study results are published or discussed in conferences. The study monitor or other authorized representatives of the sponsor or governmental regulatory agencies may inspect all documents and records required to be maintained by the investigator, including but not limited to, medical records (office, clinic, or hospital) and pharmacy records for the subjects in this study. The clinical study site will permit access to such records.

All records will be kept locked and all computer entry and networking programs will be carried out with coded numbers only and with password protected systems. All non-clinical specimens, evaluation forms, reports, and other records that leave the site will be identified only by a coded number.

9.5 Certificate of Confidentiality

To protect privacy, we have received a Certificate of Confidentiality. With this Certificate, the researchers cannot be forced to release information that may identify the research subject, even by a court subpoena, in any federal, state, or local civil, criminal, administrative, legislative, or other proceedings. The researchers will use the Certificate to resist any demands for information that would identify the subject, except as explained below.

The Certificate cannot be used to resist a demand for information from personnel of the United States Government that is used for auditing or evaluation of federally funded projects, like this study, or for information that must be released in order to meet the requirements of the Federal Food and Drug Administration (FDA).

A Certificate of Confidentiality does not prevent the subject from voluntarily releasing information about themselves or their involvement in this research. If any person or agency obtains a written consent to receive research information, then the researchers may not use the Certificate to withhold that information.

The Certificate of Confidentiality does not prevent the researchers from reporting without the

subject's consent, information that would identify the subject as a participant in the research project regarding matters that must be legally reported including: child and elder abuse, sexual abuse, or wanting to harm themselves or others.

The release of individual private information or specimens for other research will only occur if consent was obtained from the individual to whom the information, document, or biospecimen pertains.

9.6 Costs, Subject Compensation, and Research Related Injuries

There is no cost to subjects for the research tests, procedures, and study product while taking part in this trial. Procedures and treatment for clinical care may be billed to the subject, subject's insurance or third party. Subjects may be compensated for their participation in this trial. Compensation will be in accordance with the local IRB's policies and procedures, and subject to IRB approval.

If it is determined by the site principal investigator that an injury occurred to a subject as a direct result of the tests or treatments that are done for this trial, then referrals to appropriate health care facilities will be provided to the subject. Study personnel will try to reduce, control, and treat any complications from this trial. Immediate medical treatment may be provided by the participating site. No financial compensation will be provided to the subject by the NIAID, NIH to the subject for any injury suffered due to participation in this trial.

10 STATISTICAL CONSIDERATIONS

This is a phase 2b, multicenter, open-label, randomized, assessor-blinded, superiority study comparing a 2-dose dalbavancin regimen to standard of care therapy for the treatment of complicated bacteremia caused by *S. aureus*. Participants will be followed for 70 days, except for the subset with vertebral osteomyelitis who will be followed for 180 days.

On the basis of the intention-to-treat (ITT) principle, the primary outcome will be analyzed on the ITT set (defined as all participants as randomized regardless of whether they received the randomized treatment). A sensitivity analysis will be conducted using the modified ITT (mITT) population - defined as all participants who received at least one dose of study drug. Additionally, as one of the secondary assessments, we will compare the individual components of the DOOR outcome by treatment arm within the ITT population.

The secondary efficacy assessment, a non-inferiority analysis of overall clinical success rates by treatment group, will be conducted on the mITT population.

The secondary safety assessment will analyze rates of SAEs or of AEs leading to study drug discontinuation on the mITT population.

For all tests, P values will be two-sided with alpha < 0.05 level of significance. All reported confidence intervals will be two-sided 95%.

The outcomes could be missing for subjects who withdraw from the trial. The reasons for withdrawal will be reported and compared qualitatively by groups. The effect that any missing data might have on results will be assessed via sensitivity analysis. If the pattern of missing data is different to that envisaged at the design stage, further sensitivity analyses will be provided that are tailored to the missing data pattern observed.

The statistical analysis plan, which includes more technical and detailed elaboration of the principal features stated in the protocol, will be prepared separately.

10.1 Study Hypotheses

The primary objective is to compare the Desirability of Outcome Ranking (DOOR) at day 70 for dalbavancin versus standard of care antibiotic therapy for the treatment of complicated *S. aureus* bacteremia, conducted as a superiority assessment within the intention to treat population. We hypothesize that dalbavancin will have a higher DOOR relative to standard of care (e.g., the probability of a randomly selected patient having a better DOOR if assigned to receive dalbavancin versus standard of care (plus half the probability of a tied DOOR) is >50%). The null hypothesis would be no significant difference in DOOR between dalbavancin versus standard of care.

The key secondary objective will assess traditional clinical efficacy examining composite occurrence of clinical failure, infectious complications, or mortality as a non-inferiority comparison within the intention to treat population. We hypothesize that dalbavancin will have a non-inferior clinical efficacy rate relative to standard of care within a 20% absolute margin. The null hypothesis would be that dalbavancin has an inferior clinical efficacy relative to standard of care. The selection of a 20% non-inferiority margin is in line with previously published SAB trials, including the registrational trial of daptomycin versus standard of care (Fowler et al 2006). The protocol for an upcoming trial of ceftobiprole versus daptomycin includes a similar margin constructed around the particular context of SAB, noting that a non-inferiority margin of 20% includes more than half of the anticipated benefit of active-control treatment relative to untreated subjects (Hamed et al 2020). Additional secondary objectives will include a safety assessment and evaluation of the individual components of the primary DOOR outcome. Safety will be assessed by comparing the occurrence of serious (grade 3 or worse) adverse drug events between the dalbavancin and standard of care arms within the safety population. Each of the components of the DOOR outcome will be compared between the dalbavancin arm and standard of care arm within the ITT population.

10.2 Sample Size Considerations

The study is powered for a superiority comparison based on the primary objective, a comparison of DOOR outcomes. The probability of a subject from the dalbavancin arm having a superior DOOR ranking relative to a subject from the standard of care arm will be calculated along with a 95% confidence interval. Superiority will be considered to have been achieved if the 95% confidence interval for probability of having a superior DOOR ranking with dalbavancin does not cross 50%. If the confidence interval crosses 50% however, the null hypothesis cannot be rejected.

Sample size was calculated on the basis of the primary hypothesis. Assuming a 65% probability of a better DOOR in the dalbavancin treatment group versus the standard of care treatment group, with a 90% power and alpha=0.025 (by one-sided Wilcoxon rank sum test), 78 participants would be required in each treatment group. To allow for some inflation assuming around 12% of missing data or other study imperfections, using the method in Lachin (1981), we plan to recruit 100 per arm (200 subjects in total). Sample size was calculated using nQuery (MTT1-1 Module) (Version 8, Statistical Solution Ltd).

10.3 Treatment Assignment Procedures

10.3.1 Randomization Procedures

Once consented and upon entry of demographic data and confirmation of eligibility for the trial, the subject will be enrolled. Enrollment of subjects will be done online using the enrollment module of

Advantage eClinical. Subjects will be randomized 1:1 to dalbavancin or standard of care. Randomization will be stratified based on screening pathogen, MSSA vs. MRSA.

The list of randomized treatment assignments will be prepared by statisticians at the DCC (The Emmes Company). Emmes will assign each subject a treatment code and treatment assignment from the list after demographic and eligibility data have been entered.

Instructions for use of the enrollment module are included in the Advantage eClinical User's Guide. Manual back-up procedures and instructions are provided for use in case the site temporarily loses access to the internet or the online enrollment system is unavailable.

10.3.2 Masking Procedures

Study subjects and treating physicians will not be masked to treatment, as this is an open label study. Treatment group will be masked for study adjudicators.

10.4 Planned Interim Analyses

The DSMB will review interim reports of efficacy by treatment strategy. There will be one formal interim analysis of efficacy after approximately 50% of subjects have completed the trial. The statistical methods for the interim analysis will be fully specified in advance in a statistical analysis plan (SAP), to be prepared by a statistician, and summarized briefly in Section 10.4.2 below.

10.4.1 Interim Safety Review

The DSMB will evaluate safety at pre-specified intervals and at least yearly; however, ongoing review and summary of subject safety will occur to allow for early detection of a safety signal that may result from an AE or lack of efficacy of study drug. The DSMB will advise DMID on whether to continue, modify, or terminate the trial based on a risk-benefit assessment.

10.4.2 Interim Efficacy Review

A single interim analysis will be performed after approximately 50% of subjects have completed the trial. The interim efficacy analysis will consist of a quantitative evaluation of potential effect sizes and associated precision using a predicted intervals and predicted interval plots (PIPS) approach. Briefly, predicted intervals for both primary DOOR and secondary clinical failure outcomes will be modeled under a range of assumptions including: 1) the trends in outcomes observed at interim analysis continue to end of study, 2) the alternative hypothesis is true, 3) the null hypothesis is true, and 4) best and worst case scenarios for remaining outcomes. By relying on prediction intervals, no statistical hypothesis testing is required and no power is lost at interim analysis.

10.5 Final Analysis Plan

Results from primary and secondary endpoint analyses may be distributed by the SDCC to key study team members (protocol PIs, protocol statisticians, and other necessary study team members) after database lock and generation of all the CSR Tables, Listings, and Figures. These analyses may be used by the company collaborator for planning subsequent trials or by the lead principal investigator for manuscript development while the CSR is being finalized.

10.5.1 Study Populations

Five populations will be considered in the statistical analysis of the study:

1. *Screened Population*: The screened population will consist of all patients who undergo the Screening Visit (Visit 1) and receive a Patient Identification (PID) number.
2. *Intent-to-treat/Randomized Population*: The intent-to-treat (ITT) population will consist of all randomized patients regardless whether or not they received study treatment.
3. *Safety Population*: The safety population will consist of all randomized patients who received at least 1 dose of study drug. Patients will be analyzed based on the treatment received.
4. *Modified Intent-to-Treat Population*: The modified intent-to-treat (mITT) population will consist of all patients in the ITT population who received at least one dose of study drug.
5. *Clinically Evaluable Population*: The CE population will consist of all patients in the mITT population who met criteria for clinical evaluability. Patients will be considered clinically evaluable if they have a primary outcome assessment and do not have missing data or major protocol violations that prevent the adjudication committee from evaluating their outcomes.

An independent, blinded adjudication committee will be used to review the data from each patient to establish the baseline diagnosis, final diagnosis, and final outcome (including DOOR outcome and its individual components), including reasons for treatment failure. This committee will consist of 3-4 infectious disease experts with no relevant conflicts of interest.

10.5.2 Patient Disposition

The number of subjects in four of the study populations (ITT, Safety, mITT, and CE) will be summarized by treatment group and study center; the screened population will only be summarized by study center.

Screen failures (i.e., patients screened but not randomized) and the associated reasons for failure will be tabulated overall. The number and percentage of subjects who complete the treatment period (up to Day 42) and of subjects who prematurely discontinue during the same period will be presented for each treatment group and pooled across treatment groups for the ITT population. The reasons for premature discontinuation as recorded on the termination pages of the eCRF will be summarized

(number and percentage) by treatment group for all randomized subjects, along with the number of subjects completing each visit.

10.5.3 Demographics and Other Baseline Characteristics

Demographic parameters (i.e., age, race, ethnicity, sex, weight, height, body mass index) and other baseline characteristics will be summarized by treatment group for the Safety and ITT populations. Continuous variables will be summarized by number of subjects and mean, standard deviation (SD), median, minimum, and maximum values. Categorical variables will be summarized by number and percentage of subjects. The number and percentage of subjects with abnormalities in medical and surgical histories in each system organ class and preferred term will be summarized by treatment group for the ITT population.

Prior medication is defined as any medication taken before the date of the first dose of investigational product. Concomitant medication is defined as any medication started on or after the date of the first dose of investigational product.

Both prior and concomitant medication use will be summarized by the number and proportion of subjects in each treatment group receiving each medication within each therapeutic class for the safety population. If a subject took a specific medication multiple times or took multiple medications within a specific therapeutic class, that subject would be counted only once for the coded drug name or therapeutic class.

10.5.4 Extent of Exposure and Treatment Compliance

Exposure to investigational product for the safety population will be summarized for treatment duration, calculated as the number of doses of dalbavancin received for patients in the dalbavancin group, and by start and stop dates for standard of care antibiotics. Descriptive statistics (n, mean, standard deviation, minimum, median, and maximum) will be presented by treatment group.

10.5.5 Efficacy Analysis

10.5.5.1 Primary Efficacy Analysis

The primary efficacy endpoint is the DOOR outcome at Day 70 post study entry (test of cure) in the ITT population. The clinical components of the DOOR outcome (survival, clinical success/failure, and infectious complications) will be determined by the blinded clinical adjudication committee (on a rolling basis). The DOOR probability is calculated, using the equation:

$$\text{DOOR probability} = \Pr[DOOR_{\mathrm{D}} > DOOR_{\mathrm{SOC}}] + \tfrac{1}{2}\Pr[DOOR_{D} = DOOR_{\mathrm{SOC}}],$$

where $DOOR_{\mathrm{D}}$ and $DOOR_{\mathrm{SOC}}$ are the DOOR outcomes for dalbavancin and standard care groups, respectively, and $\Pr[DOOR_{\mathrm{D}} > DOOR_{\mathrm{SOC}}]$ is the probability of a DOOR from dalbavancin exceeding a DOOR from standard care and $\Pr[DOOR_{D} = DOOR_{\mathrm{SOC}}]$ is the proportion of two DOOR outcomes

being same. Pathogen (MSSA versus MRSA), which is considered in the permuted block randomization as a strata, will be incorporated into the calculation of the DOOR probability and its corresponding 95% confidence interval (the stratified analysis). In addition, the DOOR probability and 95% confidence interval without strata will be calculated.

As a secondary analysis of the primary outcome will be

i. DOOR distribution by groups
ii. Cumulative difference in DOOR categories for dalbavancin vs. Standard Care
iii. Point estimate and confidence interval of difference in mean partial credit vs. Standard Care
iv. Expected DOOR distribution for Standard Care and the expected numbers gained loss in each category with treatment

The same analysis above will be repeatedly conducted on the DOOR outcome at Day 42.

As an explanatory analysis of the primary outcome, in addition to pathogen (MSSA vs. MRSA), baseline covariate-adjustment analysis and subgroup analysis will be conducted.

10.5.5.2 Secondary Efficacy Outcomes

As the secondary efficacy outcomes, each component of the DOOR will also be examined separately: clinical success, infectious complications, SAEs, AEs leading to study drug discontinuation, mortality. Descriptive statistics, including number and percentage for the categorical variables, will be provided by groups.

Clinical failure at Days 42 and 70 will be analyzed using generalized estimating equations (GEE) assuming an unstructured correlation structure. The difference in proportions of clinical failure between the two groups at Days 42 and 70 will be calculated with the corresponding 2-sided 95% confidence interval. As a sensitivity analysis, generalized linear mixed model (GLMM) approach will be used to analyze clinical failure.

10.5.5.3 Additional Efficacy Parameters

1. QoL score – QoL will be assessed using the ARLG Bloodstream Infection QoL Measure for the primary and exploratory endpoints (Appendix C). Two additional comparator measures (EQ-5D-5L, https://euroqol.org/eq-5d-instruments/sample-demo/ and PROMIS Global Health short form, http://www.healthmeasures.net/administrator/components/com_instruments/uploads/Global%20Health%20Scale%20v1.2%2008.22.2016.pdf) will also be collected and will be used in exploratory analyses only.
2. Desirability of Outcome Ranking (DOOR) (Evans 2015) endpoint results at Day 42.

For the additional efficacy endpoints, descriptive statistics will be provided by treatment group. Continuous variables will be summarized by number of patients and mean, SD, median, minimum,

and maximum values by treatment group. Categorical variables will be summarized by number and percentage of patients by treatment group. For QoL measures, the descriptive statistics will be presented by item as well.

Descriptive statistics for the DOOR endpoint will be provided by treatment group and summarized by number of patients and mean, SD, median, minimum, and maximum values; p-values will be determined using the Wilcoxon-rank sum test for continuous variables.

10.5.6 Safety Analyses

Safety analyses will be based on the safety population. Safety will be assessed using descriptive statistics of AEs, vital signs, and laboratory tests by treatment group. For each safety parameter, the last assessment made before the first dose of investigational product will be used as the baseline for all analyses of that safety parameter.

10.5.6.1 Adverse Events

An AE (classified by preferred term) that occurs during the treatment period will be considered a treatment-emergent AE if it was not present before the first dose of investigational product or was present before the first dose of investigational product and increased in severity during the treatment period.

The number and percentage of patients reporting treatment-emergent AEs in each treatment group will be tabulated by system organ class and preferred term; by system organ class, preferred term, and severity; and by system organ class, preferred term, and causal relationship to the investigational product. If more than one AE is coded to the same preferred term for the same subject, the subject will be counted only once for that preferred term using the most severe and most related occurrence for the summarization by severity and by causal relationship to the investigational product.

The distribution of treatment-emergent AEs and AESIs by severity and causal relationship to the investigational product will be summarized by treatment group.

The incidence of common ($\geq$ 2% of patients in any treatment group) treatment-emergent AEs, on-therapy SAEs, AESIs, and AEs leading to premature discontinuation of the investigational product will be summarized by preferred term and treatment group and will be sorted by decreasing frequency for the investigational product. In addition, the incidence of fatal on-therapy SAEs (i.e., events that caused death) will be summarized separately by treatment group and preferred term. An SAE will be defined as an on-therapy SAE if it occurred during or after the first infusion of investigational product.

Listings will be presented for subjects with SAEs, AESIs, subjects with AEs leading to discontinuation, and subjects who die (if any).

10.5.6.2 Clinical Laboratory Parameters, and Vital Signs

Descriptive statistics for clinical laboratory values and changes from the baseline values at each assessment time point will be presented by treatment group for each clinical laboratory parameter.

Descriptive statistics for vital signs (e.g., pulse rate, systolic and diastolic BP) and changes from baseline values at each visit and at end of study will be presented by treatment group.

The number and percentage of subjects with potentially clinically significant (PCS) post-baseline clinical laboratory values will be tabulated by treatment group. The criteria for PCS laboratory values will be detailed in the statistical analysis plan. The percentages will be calculated relative to the number of subjects with available non-PCS baseline values and at least 1 post-baseline assessment. The numerator will be the total number of subjects with available non-PCS baseline values and at least 1 PCS post-baseline value. A supportive listing of subjects with PCS post-baseline values will be provided, including the PID number, study center number, and baseline and post-baseline values. A listing of all AEs that occur in subjects who have PCS laboratory values or vital signs will also be provided.

10.5.6.3 Exploratory Dalbavancin Plasma Pharmacokinetic Analyses

A range of exploratory analyses related to dalbavancin pharmacokinetics will be conducted among the subset of subjects receiving dalbavancin (n=100). The methodology used to evaluate PK parameters will be described in detail in the PK Plan and is reviewed briefly below.

<u>Pharmacokinetic Analysis</u>

Dalbavancin concentration-time data will be visualized using box and whisker plots, with investigation of any outliers for erroneous time or concentration data entry. Queries will be generated to resolve potential erroneous time or concentration data point entries due to transcription or measurement errors. Individual concentration-time plots will be generated on linear and semi-log scales to inform potentially optimal models for analysis.

<u>Non-Compartmental Pharmacokinetic Analysis</u>

Non-compartmental analysis (NCA) will serve as the initial approach to generate base PK parameter estimates for dalbavancin concentration-time data. These analyses will be conducted using an appropriate statistical package (e.g., Phoenix WinNonlin v8.2 or higher). This descriptive analysis will allow for comparison to previously published data. The following PK exposures for concentration-time data will be calculated as appropriate and if possible depending upon samples collected: plasma concentration prior to dose, maximum plasma concentration (C_{max}) after the first dose on Day 1, time to Cmax (T_{max}), plasma concentration on Day 8 [C_{8day}], plasma concentration on day 22 (C_{22day}), concentration on day 42 (C_{42day}), concentration on day 70 (C_{70day}), area under the plasma concentration-time curve (AUC) from days 0-8 ($AUC_{0\text{-}8days}$), AUC from days 0-22 ($AUC_{0\text{-}}$

22days), AUC from days 0-42 ($AUC_{0\text{-}42day}$), days 0-70 ($AUC_{0\text{-}70days}$), AUC from days 8-22 ($AUC_{8\text{-}22days}$), AUC from days 22-42 ($AUC_{22\text{-}42day}$), AUC from days 42-70 ($AUC_{42\text{-}70day}$), AUC to the last quantifiable sample ($AUC_{0\text{–}last}$), and AUC to infinity (AUC0–∞).

Population Pharmacokinetic Analysis

Population Pharmacokinetic Analysis. Population pharmacokinetic analysis provides a platform to identify patient covariates which can help explain a portion of the interindividual variability in selected PK parameters. The non-linear mixed effects modeling software NONMEM Version 7.3 or higher (ICON Development Solutions, Ellicott City, MD) will be used to develop the population PK model for dalbavancin concentrations in plasma. The first-order conditional estimation method with interaction (FOCEI) will be utilized; other estimation methods such as expectation-maximization (e.g., SAEM) will also be considered.

Structural PK model base development to model the plasma dalbavancin concentration-time data will be initiated using a linear three-compartment model with zero-order infusion as has been used previously (https://www.ncbi.nlm.nih.gov/pubmed/31087630). Other model modifications will be considered as necessary. Between-subject variability associated with model parameters and differing residual error structures will be tested. Model development will be guided by goodness of fit plots, plausibility of parameter estimates, reduction in inter-individual variability for structural and residual error parameters, as well as objective function and shrinkage values.

Upon selection of an appropriate base structural PK model, covariate effects (e.g., age, gender, body size descriptors, creatinine clearance, albumin and IV drug use status) will be evaluated using stepwise forward selection followed by stepwise backward elimination processes. Model validation will be assessed through visual predictive checks as well as bootstrapping. A listing of the individual PK parameters derived using the final population PK model will be provided for each patient. The steady-state volume of distribution (V_{ss}) will be calculated as the sum of the central (V_c) and peripheral volume terms (V_{p1} and V_{p2}). The alpha-phase half-life ($T_{1/2,\alpha}$) beta-phase half-life ($T_{1/2,\beta}$) and gamma-phase half-life ($T_{1/2,\gamma}$) will be calculated for each patient using the individual post-hoc PK parameters. Summary statistics (mean, standard deviation, median, minimum and maximum) will be calculated using CL, V_c, V_{p1}, V_{p2}, V_{ss}, $T_{1/2,\alpha}$, $T_{1/2,\beta}$, and $T_{1/2,\gamma}$ values.

Exposure-Response Relationship Analysis

Using the final population PK model, individual post-hoc PK parameters, and the individual patient dosing histories, simulations will be performed to generate plasma dalbavancin concentration-time profiles and calculate plasma dalbavancin exposure measures for each study subject. The following exposure variables will be calculated for each patient: C_{max}, C_{8day}, C_{22day}, C_{42day}, C_{70day}, $AUC_{0\text{-}8days}$, $AUC_{0\text{-}22days}$, $AUC_{0\text{-}42day}$, $AUC_{0\text{-}70days}$, $AUC_{8\text{-}22days}$, $AUC_{22\text{-}42day}$, $AUC_{42\text{-}70day}$, and AUC to infinity ($AUC0–\infty$). The associations between each of the simulated dalbavancin exposures, as appropriate, and each outcome of interest will be explored using standard exposure-response methodologies. Only simulated exposures that occurred prior to the outcome of interest will be considered. The outcomes evaluated will include (1) DOOR outcomes at day 42, mortality at day 42, infectious complications at day 42, adverse drug effects (grade 3 or higher) at day 42, late recurrence at 6 months (within the subset having vertebral osteomyelitis), and occurrence of AST/ALT elevation >3x upper limit of normal during treatment. Multivariable models will be used to estimate the effect of individual exposures measures on outcomes while accounting for relevant covariates.

11 SOURCE DOCUMENTS AND ACCESS TO SOURCE DATA/DOCUMENTS

Each participating site will maintain appropriate medical and research records in compliance with ICH E6, Section 4.9 and regulatory and institutional requirements for the protection of confidentiality of subjects. Each site will permit authorized representatives of the DMID, its designees, and appropriate regulatory agencies to examine (and when required by applicable law, to copy) clinical records for the purposes of quality assurance reviews, audits, and evaluation of the study safety and progress. These representatives will be permitted access to all source data and source documents, which include, but are not limited to, hospital records, clinical and office charts, laboratory notes, memoranda, subjects' memory aid or evaluation checklists, pharmacy dispensing records, recorded data from automated instruments, copies or transcriptions certified after verification as being accurate and complete, microfiches, photographic negatives, microfilm or magnetic media, x-rays, and subject files and records kept at the pharmacy, at the laboratories, and medico-technical departments involved in the clinical trial.

12 QUALITY CONTROL AND QUALITY ASSURANCE

Following a written DMID-accepted site quality management plan, each participating site(s) and its subcontractors are responsible for conducting routine quality assurance (QA) and quality control (QC) activities to internally monitor study progress and protocol compliance. The site principal investigator will provide direct access to all study-related sites, source data/data collection forms, and reports for the purpose of monitoring and auditing by the sponsor, and inspection by local and regulatory authorities. The site principal investigator will ensure all study personnel are appropriately trained and applicable documentations are maintained on site.

The DCC will implement quality control procedures beginning with the data entry system and generate data quality control checks that will be run on the database. Any missing data or data anomalies will be communicated to the participating site(s) for clarification and resolution.

13 DATA HANDLING AND RECORD KEEPING

13.1 Data Management Responsibilities

The investigator is responsible to ensure the accuracy, completeness, legibility, and timeliness of the data reported. All source documents should be completed in a neat, legible manner to ensure accurate interpretation of data. Black or blue permanent ink is required to ensure clarity of reproduced copies. When making changes or corrections, cross out the original entry with a single line, and initial and date the change. DO NOT ERASE, OVERWRITE, OR USE CORRECTION FLUID OR TAPE ON THE ORIGINAL.

Copies of the electronic CRF (eCRF) will be provided for use as source data collection forms and maintained for recording data for each subject enrolled in the study. Data reported in the eCRF derived from source data collection forms should be consistent or the discrepancies should be explained.

The sponsor and/or its designee will provide guidance to the site principal investigators and other study personnel on making corrections to the data collection forms and eCRF.

13.2 Data Coordinating Center/Biostatistician Responsibilities

Data collection is the responsibility of the study personnel at the participating clinical study site under the supervision of the site principal investigator. During the study, the site principal investigator must maintain complete and accurate documentation for the study.

The data coordinating center for this study, the Emmes Company, will be responsible for data management, quality review, analysis, and reporting of the study data.

13.3 Data Capture Methods

Clinical data (including, but not limited to, AE/SAEs, concomitant medications, medical history, physical assessments, and clinical laboratory values) will be collected on data collection forms by study personnel then entered into eCRFs via a 21 CFR Part 11-compliant internet data entry system provided by the study data coordinating center. The data system includes password protection and internal quality checks, such as automatic range checks, to identify data that appear inconsistent, incomplete, or inaccurate.

13.4 Types of Data

Data for this trial will include clinical, safety, and outcome measures (e.g., clinical laboratory values).

13.5 Study Records Retention

Study records and reports including, but not limited to, eCRFs, source documents, ICFs, laboratory test results, and study drug disposition records will be retained for 2 years after a marketing application is approved for the study product for the indication for which it is being investigated; or, if no application is to be filed or if the application is not approved for the study product, until 2 years after the investigation is discontinued and the FDA has been notified. These documents will be retained for a longer period, however, if required by local regulations. ICFs for future use will be maintained as long as the sample/specimen exists.

No records will be destroyed without the written consent of the sponsor. It is the responsibility of the sponsor to inform the site principal investigator when these documents no longer need to be retained.

14 CLINICAL MONITORING

Site monitoring is conducted to ensure that the human subjects' protections, study and laboratory procedures, study intervention administration, and data collection processes are of high quality and meet sponsor, ICH/GCP guidelines and applicable regulations, and that this trial is conducted in accordance with the protocol, protocol-specific MOP and applicable sponsor standard operating procedures. DMID, the sponsoring agency, or its designee will conduct site-monitoring visits as detailed in the clinical monitoring plan.

Site visits will be made at standard intervals as defined by DMID and may be made more frequently as directed by DMID. Monitoring visits will include, but are not limited to, review of regulatory files, accountability records, eCRFs, informed consent forms, medical and laboratory reports, and protocol and GCP compliance. Site monitors will have access to each participating site, study personnel, and all study documentation according to the DMID-approved site monitoring plan. Study monitors will meet with site principal investigators to discuss any problems and actions to be taken, and will document site visit findings and discussions.

15 PUBLICATION POLICY

Following completion of the study, the lead Principal Investigator is expected to publish the results of this research in a scientific journal. All investigators funded by the NIH must submit or have submitted for them to the National Library of Medicine's PubMed Central (http://www.ncbi.nlm.nih.gov/pmc/) an electronic version of their final, peer-reviewed manuscripts upon acceptance for publication, to be made publicly available no later than 12 months after the official date of publication. The NIH Public Access Policy ensures the public has access to the published results of NIH funded research. It requires investigators to submit final peer-reviewed journal manuscripts that arise from NIH funds to the digital archive PubMed Central upon acceptance for publication. Further, the policy stipulates that these papers must be accessible to the public on PubMed Central no later than 12 months after publication.

Refer to:

- NIH Public Access Policy, http://publicaccess.nih.gov/
- NIH Office of Extramural Research (OER) Grants and Funding, http://grants.nih.gov/grants/oer.htm

As of January 2018, all clinical trials supported by the NIH must be registered on ClinicalTrials.gov, no later than 21 days after the enrollment of the first subject. Results of all clinical trials supported by the NIH, generally, need to be submitted no later than 12 months following the primary completion date. A delay of up to 2 years is available for trials that meet certain criteria and have applied for certification of delayed posting.

As part of the result posting a copy of this protocol (and its amendments) and a copy of the Statistical Analysis Plan will be posted on ClincialTrials.gov.

For this trial the responsible party is NIH/NIAID/DMID which will register the trial and post results.

The responsible party does not plan to request certification of delayed posting.

Refer to:

- Public Law 110-85, Section 801, Clinical Trial Databases
- 42CFR11
- NIH NOT-OD-16-149

16 LITERATURE REFERENCES

1. van Hal SJ, Jensen SO, Vaska VL, Espedido BA, Paterson DL, Gosbell IB. Predictors of mortality in Staphylococcus aureus Bacteremia. *Clin Microbiol Rev.* 2012;25(2):362-386.

2. Tong SY, Davis JS, Eichenberger E, Holland TL, Fowler VG, Jr. Staphylococcus aureus infections: epidemiology, pathophysiology, clinical manifestations, and management. *Clin Microbiol Rev.* 2015;28(3):603-661.

3. Thuny F, Di Salvo G, Belliard O, et al. Risk of embolism and death in infective endocarditis: prognostic value of echocardiography: a prospective multicenter study. *Circulation.* 2005;112(1):69-75.

4. Murdoch DR, Corey GR, Hoen B, et al. Clinical presentation, etiology, and outcome of infective endocarditis in the 21st century: the International Collaboration on Endocarditis-Prospective Cohort Study. *Arch Intern Med.* 2009;169(5):463-473.

5. Selton-Suty C, Celard M, Le Moing V, et al. Preeminence of Staphylococcus aureus in infective endocarditis: a 1-year population-based survey. *Clin Infect Dis.* 2012;54(9):1230-1239.

6. Keller SC, Williams D, Rock C, Deol S, Trexler P, Cosgrove SE. A new frontier: Central line-associated bloodstream infection surveillance in home infusion therapy. *Am J Infect Control.* 2018;46(12):1419-1421.

7. Lefort A, Pavie J, Garry L, Chau F, Fantin B. Activities of dalbavancin in vitro and in a rabbit model of experimental endocarditis due to Staphylococcus aureus with or without reduced susceptibility to vancomycin and teicoplanin. *Antimicrob Agents Chemother.* 2004;48(3):1061-1064.

8. Candiani G, Abbondi M, Borgonovi M, Romano G, Parenti F. In-vitro and in-vivo antibacterial activity of BI 397, a new semi-synthetic glycopeptide antibiotic. *J Antimicrob Chemother.* 1999;44(2):179-192.

9. Raad I, Darouiche R, Vazquez J, et al. Efficacy and safety of weekly dalbavancin therapy for catheter-related bloodstream infection caused by gram-positive pathogens. *Clin Infect Dis.* 2005;40(3):374-380.

10. Dunne MW, Puttagunta S, Giordano P, Krievins D, Zelasky M, Baldassarre J. A Randomized Clinical Trial of Single-Dose Versus Weekly Dalbavancin for Treatment of Acute Bacterial Skin and Skin Structure Infection. *Clinical infectious diseases : an official publication of the Infectious Diseases Society of America.* 2016;62(5):545-551.

11. Seltzer E, Dorr MB, Goldstein BP, Perry M, Dowell JA, Henkel T. Once-weekly dalbavancin versus standard-of-care antimicrobial regimens for treatment of skin and soft-tissue infections. *Clinical infectious diseases : an official publication of the Infectious Diseases Society of America.* 2003;37(10):1298-1303.

12. Jauregui LE, Babazadeh S, Seltzer E, et al. Randomized, double-blind comparison of once-weekly dalbavancin versus twice-daily linezolid therapy for the treatment of complicated skin and skin structure infections. *Clinical infectious diseases : an official publication of the Infectious Diseases Society of America.* 2005;41(10):1407-1415.

13. Boucher HW, Wilcox M, Talbot GH, Puttagunta S, Das AF, Dunne MW. Once-weekly dalbavancin versus daily conventional therapy for skin infection. *N Engl J Med.* 2014;370(23):2169-2179.

14. Holland TL, Raad I, Boucher HW, et al. Effect of Algorithm-Based Therapy vs Usual Care on Clinical Success and Serious Adverse Events in Patients with Staphylococcal Bacteremia: A Randomized Clinical Trial. *Jama.* 2018;320(12):1249-1258.

15. Baddour LM, Wilson WR, Bayer AS, et al. Infective Endocarditis in Adults: Diagnosis, Antimicrobial Therapy, and Management of Complications: A Scientific Statement for Healthcare Professionals from the American Heart Association. *Circulation.* 2015;132(15):1435-1486.

16. Corey GR. Staphylococcus aureus bloodstream infections: definitions and treatment. *Clin Infect Dis.* 2009;48 Suppl 4:S254-259.

17. Lepak A, Marchillo K, VanHecker J, Andes D. Impact of Glycopeptide Resistance in Staphylococcus aureus on the Dalbavancin In Vivo Pharmacodynamic Target. *Antimicrob Agents Chemother.* 2015;59(12):7833-7836.

18. Andes D, Craig WA. In vivo pharmacodynamic activity of the glycopeptide dalbavancin. *Antimicrob Agents Chemother.* 2007;51(5):1633-1642.

19. Marbury T, Dowell JA, Seltzer E, Buckwalter M. Pharmacokinetics of dalbavancin in patients with renal or hepatic impairment. *J Clin Pharmacol.* 2009;49(4):465-476.

20. Dorr MB, Jabes D, Cavaleri M, et al. Human pharmacokinetics and rationale for once-weekly dosing of dalbavancin, a semi-synthetic glycopeptide. *J Antimicrob Chemother.* 2005;55 Suppl 2:ii25-30.

21. Carrothers TJ, Chittenden JT, Critchley I. Dalbavancin Population Pharmacokinetic Modeling and Target Attainment Analysis. *Clin Pharmacol Drug Dev.* 2020;9(1):21-

22. Evans SR, Rubin D, Follmann D, et al. Desirability of Outcome Ranking (DOOR) and Response Adjusted for Duration of Antibiotic Risk (RADAR). *Clin Infect Dis.* 2015;61(5):800-806.

23. Dalvance (dalbavancin) [product insert]. Madison, NJ: Allergan USA, Inc.; 2018.

24. Fowler VG, Jr., Boucher HW, Corey GR, et al. Daptomycin versus standard therapy for bacteremia and endocarditis caused by Staphylococcus aureus. *N Engl J Med.* 2006;355(7):653-665.

25. Hamed K, Engelhardt M, Jones ME, et al. Ceftobiprole versus daptomycin in Staphylococcus aureus bacteremia: a novel protocol for a double-blind, Phase III trial. *Future Microbiol.* 2020;15(1):35-48.

26. Lachin JL. Introduction to Sample Size Determination and Power Analysis for Clinical Trials. Controlled Clinical Trials 198; 2:93-113

27. King HA, Doernberg SB, Miller J, et al. Patients' experiences with Staphylococcus aureus and Gram-negative bacterial bloodstream infections: A qualitative descriptive study and concept elicitation phase to inform measurement of patient-reported quality of life. *Clin Infect Dis* 2020; Accepted, available epub ahead of print at: https://doi.org/10.1093/cid/ciaa611

17 APPENDICES

Appendix A. Schedule of Events

	**Induction Period**	**Screening/ Enrollment**	**Open label Treatment Period**				**Post-treatment Follow-up Period**		
	Visit 0 (Pre-Screening, Day -10 to Day 1)	Visit 1 (Day –1 to Day 1)	Visit 2 (Baseline, Day 1)	Visit 3 (Day 8 ± 1 day)	Visit 4 (Day 22 ± 2 days)	Visit 5 (Day 42 ± 3 days)	Visit 6 (TOC, Day 70 ± 7 days)[a]	ET[b]	Visit 7 (Day 180 ± 14 days, vertebral Osteomyelitis group)[a]
Informed Consent		X							
Dalbavancin[c]			X	X					
Standard of care antibiotic therapy[c]	X	X	X (Duration 28-56 days)						
Medical history[d]		X	X			X	X	X	
Medication history[e]		X							
Randomization			X						
AEs/AESIs/SAEs			X	X	X	X	X	X	
Hematology and serum chemistry blood sampling[f]		X		X[g]	X	X			
Coagulation lab tests[f]		X							

	**Induction Period**	**Screening/ Enrollment**	**Open label Treatment Period**				**Post-treatment Follow-up Period**		
	Visit 0 (Pre-Screening, Day -10 to Day 1)	Visit 1 (Day –1 to Day 1)	Visit 2 (Baseline, Day 1)	Visit 3 (Day 8 ± 1 day)	Visit 4 (Day 22 ± 2 days)	Visit 5 (Day 42 ± 3 days)	Visit 6 (TOC, Day 70 ± 7 days)[a]	ET[b]	Visit 7 (Day 180 ± 14 days, vertebral Osteomyelitis group)[a]
Pregnancy test[h]		X							
PK sampling[i]			X	X	X	X	X	X	
Vital signs[j]		X	X	X[k]	X	X	X	X	X
Physical examination[l]		X	X	X	X	X	X	X	X
Echocardiogram[m]		X							
Investigator assessment of efficacy						X	X	X	X
Concomitant medications[n]		X	X	X	X	X	X	X	X
Concomitant nondrug interventions		X	X	X	X	X	X	X	X
QoL assessment[o]			X	X	X	X	X	X	X

AEs = adverse events; AESIs = adverse events of special interest; eCRF = electronic case report form; ET = Early Termination; PK = pharmacokinetic; SAE = serious adverse events

[a]Telephone visit permissible if in-person visit is not possible; in person visit still preferred.

[b]Patients who prematurely discontinue therapy should have an ET Visit within 72 hours.

[c]All subjects will be receiving standard of care prior to randomization; after randomization, subjects will receive either dalbavancin or standard of care based on their assigned treatment group.

[d]Includes targeted/pertinent medical and surgical history only

[e]A complete medication history will be completed through 30 days prior to ICF signing; an extended 60 day review will be conducted for dalbavancin and oritavancin given the long half-lives of both drugs.

[f]Visit 1 hematology, coagulation lab tests (PT, PTT, and/or INR) and serum chemistry will be done in order to qualify the patient for the study, if not already collected per standard of care within 48 hours prior to randomization.

[g]A serum creatinine assessment will be required within the 72 hours prior to the 2nd (Day 8) dalbavancin dose. Whether a serum creatinine must be repeated on Day 8 will be at the discretion of the site investigator based upon stability of

the serum creatinine in the preceding 72 hours and whether the serum creatinine is near the threshold where dose adjustment would be necessary (e.g., near 30 mL/min).

[h]Women of childbearing potential only, if not already performed (see Appendix B, Definitions); ensure test is negative within 48 hours before randomization. If the serum test results cannot be obtained before randomization, a urine pregnancy test may be used for enrollment.

[i]Dalbavancin PK samples will be drawn only for subjects receiving dalbvacancin. PK samples will be drawn at Day 1 prior to dose, at end of infusion ± 10 minutes, 6 ± 2 hours post end of dose, 12 ± 4 hours post end of dose, 24 ± 6 hours post end of dose), Day 8 (prior to 2nd dose), Day 22 ± 2 days (at time of clinic visit), day 42 ± 3 days, day 70 ± 7 days, and with any ET visit. Each sample must be accompanied by draw time and date.

[j]Vital signs include blood pressure, respiration rate, pulse rate, and temperature.

[k]Day 8 vital signs not required for subjects receiving SOC antibiotics if discharge occurs prior to day 8.

[l]Targeted physical exam includes general appearance, examination of head, eyes, ears, nose, throat, neck, skin, heart, lungs, abdomen, neurologic system, musculoskeletal system, extremities, height, and body weight. If height or weight is not obtainable (eg, patient is immobilized), use the last known or stated height and weight. Subsequent physical exams will focus on changes from prior exams and on the evaluation of newly reported symptoms.

[m]Transthoracic echocardiogram or, if clinically indicated, transesophageal echocardiogram to be performed (local laboratory), unless one has been performed as standard of care for this episode of bacteremia/endocarditis

[n]All concomitant medications from Screening (Visit 1) through Day 42 (± 3 days) (Visit 5) must be recorded in the patient's medical record and on the eCRFs. Between the Day 42 Visit and Day 70~~84~~ Visit, all concomitant medications for an AE or any antibacterial therapy should be recorded in the patient's medical record and on the eCRF.

[o]QoL assessments include the ARLG Bloodstream Infection QoL Measure (Appendix C), the EQ-5D-5L (https://euroqol.org/eq-5d-instruments/sample-demo/), and the PROMIS Global Health Short Form (http://www.healthmeasures.net/administrator/components/com_instruments/uploads/Global%20Health%20Scale%20v1.2%2008.22.2016.pdf).

Appendix B. Definitions

Childbearing potential: a woman is considered of childbearing potential unless post-menopausal [≥1 year of spontaneous amenorrhea] or permanently surgically sterilized [bilateral oophorectomy, salpingectomy, hysterectomy].

Effective contraception: Includes non-male sexual relationships, abstinence from sexual intercourse with a male partner, monogamous relationship with a vasectomized partner who has been vasectomized ≥180 days before the subject received the first dose of study drug, barrier methods such as condoms or diaphragms, effective intrauterine devices (IUDs), NuvaRing®, or licensed hormonal methods such as implants, injectables, or oral contraceptives.

Complicated *S. aureus* Bacteremia:

Positive follow-up blood cultures: blood cultures positive for *S. aureus* drawn at least 24 hours after the initial qualifying blood culture

Persistent fever: oral temperature ≥38.0C for >72 hours after the initial positive blood culture for *S. aureus*

Endocarditis:

These criteria have been adjusted to be specific to *S. aureus* bacteremia, i.e. microbiological criteria related to organisms other than *S. aureus* have been removed. In addition, references to prosthetic valve infections have been removed, as these patients will be excluded from the study.

According to modified Duke Criteria, diagnosis of IE can be definite, possible, or rejected. A diagnosis of IE is **definite** if either the following pathological or clinical criteria are met:

Pathologic criteria:

- pathologic lesions: vegetation or intracardiac abscess demonstrating active endocarditis on histology, or
- microorganism: demonstrated by culture or histology of a vegetation or intracardiac abscess

One of these combinations of clinical criteria (see definitions below):

- two major clinical criteria
- one major and three minor criteria
- five minor criteria

Diagnosis of IE is **possible** if one of the following combinations of clinical criteria (see definitions below) are met:

- one major and one minor criteria
- three minor criteria are fulfilled

Diagnosis of IE is **rejected** if one of the following criteria are met:

- a firm alternate diagnosis is made
- resolution of clinical manifestations after ≤ 4 days of antibacterial treatment
- no pathological evidence of IE is found at surgery or autopsy after antibacterial treatment therapy for ≤ 4 days
- clinical criteria for possible or definite IE are not met

Major criteria for the diagnosis of infective endocarditis:

1. Positive blood culture with *S. aureus* from two separate blood cultures

2. Evidence of endocardial involvement with positive echocardiogram defined as

- oscillating intracardiac mass on valve or supporting structures, in the path of regurgitant jets in the absence of an alternative anatomic explanation
- abscess
- new valvular regurgitation (worsening or changing of preexisting murmur not sufficient)

Minor criteria for the diagnosis of infective endocarditis:

1. Predisposing factor: intravenous drug use or presence of a predisposing heart condition (a valve lesion associated with significant regurgitation or turbulence of blood flow)

2. Fever ≥38°C (100.4 °F)

3. Vascular phenomena: major arterial emboli, septic pulmonary infarcts, mycotic aneurysm, intracranial hemorrhage, conjunctival hemorrhages or Janeway lesions

4. Immunological phenomena: glomerulonephritis, Osler's nodes, Roth's spots, Rheumatoid factor

5. A single positive blood culture with *S. aureus*

Visceral Abscess (e.g. liver, spleen, kidney, etc.) – either of the following:

- Abscess visualized on radiographic exam, or
- Isolation of *S. aureus* from culture of abscess contents

Pleuropulmonary Infection – one of the following:

- Pulmonary infiltrate consistent with pneumonia in patients with *S. aureus* bacteremia
- *S. aureus* in pleural fluid, needle aspirate biopsy, or growth from bronchoalveolar lavage (BAL)/protected specimen brush (PSB)
- Clinical evidence of pneumonia (eg. Increased O2, increased respiratory rate, cough, mechanical ventilation, purulent sputum, etc)

Osteomyelitis - Either of the following:

- Radiographic evidence of bone lesion consistent with osteomyelitis; or
- Culture of bone yields staphylococcus.

Pyomyositis – Either of the following:

- Radiographic evidence consistent with pyomyositis; or
- Culture of abscess contents yields *S. aureus*.

Septic Arthritis – Either of the following:

- Staphylococcus in culture of synovial fluid; or
- Positive Gram stain of synovial fluid for Gram positive cocci AND synovial fluid cell count ≥20,000 WBC/mL without alternate explanation

Septic Thrombophlebitis – Either of the following:

- Palpable venous cord
- Evidence of thrombosis on radiologic exam

Relapsing Infection: Relapsing infection is defined as a staphylococcus that:

- Represents the same bacterial strain as the Baseline Infecting Pathogen (based on bacterial speciation, antibiotic susceptibility testing, and/or genotyping tests, as appropriate);

- Is documented by a culture yielding *S. aureus* obtained after randomization, unless from a procedure (e.g. abscess drainage, catheter removal) that was planned prior to randomization

Uncomplicated *Staphylococcus aureus* Bacteremia: defined as all of the following: exclusion of endocarditis by echocardiography; catheter-associated bacteremia and removal of catheter; no implanted prostheses; follow-up blood cultures drawn within 48 hours after initial set that do not grow screening pathogen and all follow-up blood cultures thereafter do not grow the screening pathogen; defervescence within 72 hours of initiating effective therapy; and no evidence of metastatic sites of infection.

Source Control Procedure: a procedure intended to treat *S. aureus* infection. Examples include, but are not limited to, surgical debridement or amputations, drainage of infected spaces, and removal of prosthetic material

Vascular Access Procedures: insertion or removal of vascular catheters

Microbiologic success: no post-randomization growth of the baseline pathogen from blood cultures or another sterile body site

Blood culture contaminant: the following organisms may be considered a contaminant if grown from only one blood culture (either alone or in addition to *S. aureus* isolated from the same blood culture): *Cutibacterium* species, *Micrococcus* species, viridans-group streptococcus, coagulase-negative staphylococci, *Corynebacterium* species, *Bacillus* species other than *B. anthracis*, or enterococcus species.

Appendix C. ARLG Bloodstream Infection Quality of Life Measure

**Please respond to each item by marking one box per row.**							
**Global**			**Excellent**	**Very Good**	**Good**	**Fair**	**Poor**
Global01	1	In general, would you say your health is:	☐	☐	☐	☐	☐
Global02	2	In general, would you say your quality of life is:	☐	☐	☐	☐	☐
**Thinking of your bloodstream infection, please answer the following questions to best capture your experiences.**							
**Fatigue**			**Not at all**	**A little bit**	**Somewhat**	**Quite a bit**	**Very much**
HI7	3	During the past 7 days, I feel fatigued	☐	☐	☐	☐	☐
AN3	4	During the past 7 days, I have trouble starting things because I am tired	☐	☐	☐	☐	☐
FATEXP41	5	In the past 7 days, how run-down did you feel on average?	☐	☐	☐	☐	☐
FATEXP40	6	In the past 7 days, how fatigued were you on average?	☐	☐	☐	☐	☐
**Gastrointestinal Nausea and Vomiting**			**Never**	**Rarely**	**Sometimes**	**Often**	**Always**
GISX49	7	In the past 7 days, how often did you have nausea—that	☐	☐	☐	☐	☐

		is, a feeling like you could vomit? (If never, skip to 10)					
GISX52	8	In the past 7 days, how often did you know that you would have nausea before it happened?	☐	☐	☐	☐	☐
GISX55	9	In the past 7 days, how often did you have a poor appetite?	☐	☐	☐	☐	☐
			**Never**	**One day**	**2-6 days**	**Once a day**	**More than once a day**
GISX59	10	In the past 7 days, how often did you throw up or vomit?	☐	☐	☐	☐	☐
**Pain Intensity**			**Had no pain**	**Mild**	**Moderate**	**Severe**	**Very severe**
PAINQU6	11	In the past 7 days, how intense was your pain at its worst?	☐	☐	☐	☐	☐
PAINQU8	12	In the past 7 days, how intense was your average pain?	☐	☐	☐	☐	☐
			**No pain**	**Mild**	**Moderate**	**Severe**	**Very severe**
PAINQU21	13	What is your level of pain right now?	☐	☐	☐	☐	☐
**Sleep Disturbance**			**Very poor**	**Poor**	**Fair**	**Good**	**Very Good**

Sleep109	14	In the past 7 days, my sleep quality was	☐	☐	☐	☐	☐
			**Not at all**	**A little bit**	**Somewhat**	**Quite a bit**	**Very much**
Sleep116	15	In the past 7 days, my sleep was refreshing	☐	☐	☐	☐	☐
Sleep20	16	In the past 7 days, I had a problem with my sleep	☐	☐	☐	☐	☐
Sleep44	17	In the past 7 days, I had difficulty falling asleep	☐	☐	☐	☐	☐
**Emotional Distress - Depression**			**Never**	**Rarely**	**Sometimes**	**Often**	**Always**
EDDEP04	18	In the past 7 days, I felt worthless	☐	☐	☐	☐	☐
EDDEP06	19	In the past 7 days, I felt helpless	☐	☐	☐	☐	☐
EDDEP29	20	In the past 7 days, I felt depressed	☐	☐	☐	☐	☐
EDDEP41	21	In the past 7 days, I felt hopeless	☐	☐	☐	☐	☐
**Emotional Distress - Anxiety**			**Never**	**Rarely**	**Sometimes**	**Often**	**Always**
EDANX01	22	In the past 7 days, I felt fearful	☐	☐	☐	☐	☐
EDANX40	23	In the past 7 days, I found it hard to focus on anything other than my anxiety	☐	☐	☐	☐	☐

EDANX41	24	In the past 7 days, my worries overwhelmed me	☐	☐	☐	☐	☐
EDANX53	25	In the past 7 days, I felt uneasy	☐	☐	☐	☐	☐
**Cognitive Function - Abilities**			**Not at all**	**A little bit**	**Somewhat**	**Quite a bit**	**Very much**
PC43_2r	26	In the past 7 days, my mind has been as sharp as usual	☐	☐	☐	☐	☐
PC44_2r	27	In the past 7 days, my memory has been as good as usual	☐	☐	☐	☐	☐
PC45_2r	28	In the past 7 days, my thinking has been as fast as usual	☐	☐	☐	☐	☐
PC47_2r	29	In the past 7 days, I have been able to keep track of what I am doing, even if I am interrupted	☐	☐	☐	☐	☐
**Physical Function**			**Without any difficulty**	**With a little difficulty**	**With some difficulty**	**With much difficulty**	**Unable to do**
PFA11	30	Are you able to do chores such as vacuuming or yard work?	☐	☐	☐	☐	☐
PFA21	31	Are you able to go up and down stairs at a normal pace?	☐	☐	☐	☐	☐

PFA23	32	Are you able to go for a walk of at least 15 minutes?	☐	☐	☐	☐	☐
PFA53	33	Are you able to run errands and shop?	☐	☐	☐	☐	☐
			**Not at all**	**Very little**	**Somewhat**	**Quite a lot**	**Cannot do**
PFC12	34	Does your health now limit you in doing two hours of physical labor?	☐	☐	☐	☐	☐
PFB1	35	Does your health now limit you in doing moderate work around the house like vacuuming, sweeping floors or carrying in groceries?	☐	☐	☐	☐	☐
**Ability to Participate in Social Roles and Activities**			**Never**	**Rarely**	**Sometimes**	**Often**	**Always**
SRPPER11_CaPS	36	I have trouble doing all of my regular leisure activities with others	☐	☐	☐	☐	☐
SRPPER18_CaPS	37	I have trouble doing all of the family activities that I want to do	☐	☐	☐	☐	☐
SRPPER23_CaPS	38	I have trouble doing all of my usual work (include work at home)	☐	☐	☐	☐	☐

SRPPER46 _CaPS	39	I have trouble doing all of the activities with friends that I want to do	☐	☐	☐	☐	☐
			**Excellent**	**Very Good**	**Good**	**Fair**	**Poor**
	40	Because of your bloodstream infection, would you say your health is..	☐	☐	☐	☐	☐
	41	Because of your bloodstream infection, would you say your quality of life is..	☐	☐	☐	☐	☐

Dalbavancin as an Option for Treatment of *S. aureus* Bacteremia (DOTS): A Phase 2b, Multicenter, Randomized, Open-Label, Assessor-Blinded Superiority Study to Compare the Efficacy and Safety of Dalbavancin to Standard of Care Antibiotic Therapy for the Completion of Treatment of Patients with Complicated *S. aureus* Bacteremia

DMID Protocol Number: 20-0002

DMID Funding Mechanism: UM1AI104681

Pharmaceutical Support: Allergan, a subsidiary of AbbVie

IND Sponsor: National Institutes of Health (NIH)/National Institute of Allergy and Infectious Diseases (NIAID)/Division of Microbiology and Infectious Diseases (DMID)

Lead Principal Investigator: Thomas L. Holland, M.D.

[redacted]

Draft or Version Number: v6.0

29 Sep 2023

STATEMENT OF ASSURANCE

Each Institution will hold a current Federal Wide Assurance (FWA) issued by the Office of Human Research Protections (OHRP) for federally-funded human subjects research. Each FWA will designate at least one Institutional Review Board (IRB)/Independent Ethics Committee (IEC) registered with OHRP, for which the research will be reviewed and approved by the IRB/IEC and will be subject to continuing review [45 CFR 46.103(b)]. The IRB/IEC designated under an FWA may include an institution's IRB/IEC, an independent IRB/IEC, or an IRB/IEC of another institution after establishing a written agreement with that other institution.

STATEMENT OF COMPLIANCE

The study trial will be carried out in accordance with Good Clinical Practice (GCP) and as required by the following:

- United States Code of Federal Regulations (CFR) 45 CFR Part 46: Protection of Human Subjects
- Food and Drug Administration (FDA) Regulations, as applicable: 21 CFR Part 50 (Protection of Human Subjects), 21 CFR Part 54 (Financial Disclosure by Clinical Investigators), 21 CFR Part 56 (Institutional Review Boards), 21 CFR Part 11, and 21 CFR Part 312 (Investigational New Drug Application), 21 CFR 812 (Investigational Device Exemptions)
- International Conference on Harmonisation: Good Clinical Practice (ICH E6); 62 Federal Register 25691 (1997); and future revisions
- Belmont Report: Ethical Principles and Guidelines for the Protection of Human Subjects of Research, Report of the National Commission for the Protection of Human Subjects of Biomedical and Behavioral Research
- National Institutes of Health (NIH) Office of Extramural Research, Research Involving Human Subjects, as applicable
- National Institute of Allergy and Infectious Diseases (NIAID) Clinical Terms of Award, as applicable
- Applicable Federal, State, and Local Regulations and Guidance

SIGNATURE PAGE

The signature below provides the necessary assurance that this trial will be conducted according to all stipulations of the protocol, including all statements regarding confidentiality, and according to local legal and regulatory requirements and applicable US federal regulations and ICH E6 Good Clinical Practice (GCP) guidelines.

I agree to conduct the study in compliance with GCP and applicable regulatory requirements.

I agree to conduct the study in accordance with the current protocol and will not make changes to the protocol without obtaining the sponsor's approval and IRB/IEC approval, except when necessary to protect the safety, rights, or welfare of subjects.

Site Investigator Signature:

Signed: ______________________________ Date: ______________

Name

Title

TABLE OF CONTENTS

STATEMENT OF ASSURANCE 2
STATEMENT OF COMPLIANCE 3
SIGNATURE PAGE 4
TABLE OF CONTENTS 5
LIST OF TABLES 10
LIST OF FIGURES 11
LIST OF ABBREVIATIONS 12
PROTOCOL SUMMARY 15
1 KEY ROLES 21
2 BACKGROUND AND SCIENTIFIC RATIONALE 22
2.1 Background 22
2.2 Scientific Rationale 24
2.2.1 Purpose of Study 24
2.2.2 Study Population 25
2.2.3 Selection of Dose 25
2.3 Potential Risks and Benefits 28
2.3.1 Potential Risks 29
2.3.1.1 Dalbavancin 29
2.3.1.2 Cefazolin 29
2.3.1.3 Nafcillin 29
2.3.1.4 Oxacillin 30
2.3.1.5 Vancomycin 30
2.3.1.6 Daptomycin 31
2.3.1.7 Risks Associated with Intravenous Access 31
2.3.2 Potential Benefits 31
3 STUDY DESIGN, OBJECTIVES AND ENDPOINTS OR OUTCOME MEASURES 32
3.1 Study Design Description 32

3.2 Study Objectives 32
3.2.1 Primary 32
3.2.2 Secondary 32
3.2.3 Exploratory 33
3.3 Study Endpoints or Outcome Measures 34
3.3.1 Primary 34
3.3.2 Secondary 35
3.3.3 Exploratory 36
4 STUDY INTERVENTION/INVESTIGATIONAL PRODUCT 37
4.1 Study Product Description 37
4.1.1 Formulation, Packaging, and Labeling 37
4.1.2 Product Storage and Stability 38
4.2 Acquisition/Distribution 38
4.3 Dosage/Regimen, Preparation, Dispensing and Administration of Study Intervention/Investigational Product 38
4.4 Pre-determined Modification of Study Intervention/Investigational Product for an Individual Subject 41
4.5 Accountability Procedures for the Study Intervention/Investigational Product(s) 41
5 SELECTION OF SUBJECTS AND STUDY ENROLLMENT AND WITHDRAWAL 42
5.1 Eligibility Criteria 42
5.1.1 Subject Inclusion Criteria 42
5.1.2 Subject Exclusion Criteria 43
5.2 Withdrawal from the Study, Discontinuation of Study Product, or Study Termination44
5.2.1 Withdrawal from the Study or Discontinuation of the Study Product 44
5.2.2 Subject Replacement 46
5.2.3 Study Termination 46
6 STUDY PROCEDURES 47
6.1 Induction Period: Visit 0 (Pre-screening, Day -10 to Day 1) 47
6.2 Screening: Visit 1 (Day -1 to Day 1) 47
6.3 Open-label Treatment Period, Planned Study Visits 48

6.3.1 Baseline (Randomization): Visit 2 (Day 1) 48
6.3.2 Visit 3 (Day 8 ± 1 day)....................49
6.3.3 Visit 4 (Day 22 ± 2 days) 50
6.3.4 Visit 5 (Day 42 ± 3 days) 50
6.3.5 Test of Cure: Visit 6 (Day 70 ± 7 days) 51
6.3.6 Visit 7 (Day 180 ± 14 days – only for subjects with osteomyelitis)....................51
6.3.7 Final Study Visit 52
6.3.8 Early Termination Visit 52
6.4 Unscheduled Study Visits....................53
6.5 Protocol Deviations 53
7 DESCRIPTION OF CLINICAL AND LABORATORY EVALUATIONS 54
7.1 Clinical Evaluations....................54
7.1.1 Research Procedures 54
7.1.2 Assessment of Concomitant Medications/Treatments Other Than Study Product 55
7.1.3 Assessment of Subject Compliance with Study Intervention/Investigational Product/Investigational Device 56
7.1.4 Non-Research Standard of Care....................56
7.2 Laboratory Evaluations....................57
7.2.1 Clinical Laboratory Evaluations 57
7.2.2 Research Assays....................57
7.2.2.1 Laboratory Specimen Preparation, Handling, and Storage....................58
7.2.2.2 Laboratory Specimen Shipping 58
8 ASSESSMENT OF SAFETY....................59
8.1 Assessing and Recording Safety Parameters....................59
8.1.1 Adverse Events (AEs)....................59
8.1.1.1 Adverse Events Grading 60
8.1.2 Serious Adverse Events (SAEs)....................61
8.2 Specification of Safety Parameters....................62
8.2.1 Adverse Events of Special Interest (AESIs) 63

8.3 Reporting Procedures 63
8.3.1 Reporting Serious Adverse Events 63
8.3.2 Regulatory Reporting for Studies Conducted Under DMID Sponsored IND64
8.3.3 Reporting of Pregnancy 65
8.4 Type and Duration of Follow-up of Subjects after Adverse Events 65
8.5 Halting Rules 65
8.5.1 Study Halting Criteria 65
8.5.2 Individual Halting Rules 66
8.6 Safety Oversight 66
8.6.1 Data and Safety Monitoring Board (DSMB) 66
9 HUMAN SUBJECTS PROTECTION 68
9.1 Institutional Review Board/Independent Ethics Committee 68
9.2 Informed Consent Process 68
9.2.1 Other Informed Consent Procedures 70
9.3 Exclusion of Women, Minorities, and Children (Special Populations) 71
9.4 Subject Confidentiality 71
9.5 Certificate of Confidentiality 71
9.6 Costs, Subject Compensation, and Research Related Injuries 72
10 STATISTICAL CONSIDERATIONS 73
10.1 Study Hypotheses 73
10.2 Sample Size Considerations 74
10.3 Treatment Assignment Procedures 74
10.3.1 Randomization Procedures 74
10.3.2 Masking Procedures 75
10.4 Planned Interim Analyses 75
10.4.1 Interim Safety Review 75
10.4.2 Interim Futility Review 75
10.5 Final Analysis Plan 76
10.5.1 Study Populations 76

10.5.2 Patient Disposition 76
10.5.3 Demographics and Other Baseline Characteristics 77
10.5.4 Extent of Exposure and Treatment Compliance 77
10.5.5 Efficacy Analysis 77
10.5.5.1 Primary Efficacy Analysis 77
10.5.5.2 Secondary Efficacy Outcomes 78
10.5.5.3 Additional Efficacy Parameters 78
10.5.6 Safety Analyses 79
10.5.6.1 Adverse Events 79
10.5.6.2 Clinical Laboratory Parameters, and Vital Signs 79
10.5.6.3 Exploratory Dalbavancin Plasma Pharmacokinetic Analyses 80
11 SOURCE DOCUMENTS AND ACCESS TO SOURCE DATA/DOCUMENTS 83
12 QUALITY CONTROL AND QUALITY ASSURANCE 84
13 DATA HANDLING AND RECORD KEEPING 85
13.1 Data Management Responsibilities 85
13.2 Data Coordinating Center/Biostatistician Responsibilities 85
13.3 Data Capture Methods 85
13.4 Types of Data 85
13.5 Study Records Retention 86
14 CLINICAL MONITORING 87
15 PUBLICATION POLICY 88
16 LITERATURE REFERENCES 89
17 APPENDICES 92
Appendix A. Schedule of Events 93
Appendix B. Definitions 96
Appendix C. ARLG Bloodstream Infection Quality of Life Measure 100

LIST OF TABLES

Table 1: Treatment Arms 19
Table 2: Activity of Dalbavancin Against Gram-Positive Pathogens Collected in 2014 US Surveillance 23
Table 3: Activity of Dalbavancin Against Gram –Positive Pathogens Collected in 2014 EU Surveillance 23
Table 4: Substitution Patterns for Dalbavancin API Homologs 37
Table 5: Acceptable SOC Antibiotics 40

LIST OF FIGURES

Figure 1: Schematic of Study Design 20
Figure 2: Simulated Mean Plasma PK Profile for Dalbavancin (Source: Allergen data on file)....................27
Figure 3: Dalbavancin Concentration Time Course for a Typical Subject Under the Proposed Regimen (Source: Allergen data on file)....................28

LIST OF ABBREVIATIONS

ADL	Activity of Daily Living
AE	Adverse Event/Adverse Experience
AESI	Adverse Events of Special Interest
AUC	Area Under Curve
CNS	Central Nervous System
CE	Clinically Evaluable
CFR	Code of Federal Regulations
CI	Confidence Interval
CMS	Clinical Material Services
CRF	Case Report Form
CTCAE	Common Terminology Criteria for Adverse Events
DCC	Data Coordinating Center
DHHS	Department of Health and Human Services
DILI	Drug-Induced Liver Injury
DMID	Division of Microbiology and Infectious Diseases, NIAID, NIH, DHHS
DOOR	Desirability of Outcome Ranking
DSMB	Data and Safety Monitoring Board
eCRF	Electronic Case Report Form
EDC	Electronic Data Capture
FDA	Food and Drug Administration
FWA	Federal Wide Assurance
GCP	Good Clinical Practice
HEOR	Health Economics and Outcomes Research
HLGT	High Level Group Term

HIPAA	Health Insurance Portability and Accountability Act
ICD	Implantable Cardioverter Defibrillator
ICF	Informed Consent Form
ICH	International Conference on Harmonisation
IE	Infective Endocarditis
IEC	Independent or Institutional Ethics Committee
IND	Investigational New Drug Application
IRB	Institutional Review Board
ITT	Intent to Treat
MedDRA®	Medical Dictionary for Regulatory Activities
MIC	Minimum Inhibitory Concentration
mITT	Modified Intent to Treat
MM	Medical Monitor
MOP	Manual of Procedures
MRSA	Methicillin-resistant *Staphylococcus aureus*
MSSA	Methicillin-sensitive *Staphylococcus aureus*
N	Number (typically refers to subjects)
NIAID	National Institute of Allergy and Infectious Diseases, NIH, DHHS
NIH	National Institutes of Health
OHRP	Office for Human Research Protections
PCS	Potentially Clinically Significant
PID	Patient Identification
PD	Pharmacodynamic
PHI	Protected Health Information
PI	Principal Investigator

PK	Pharmacokinetics
PWID	Persons Who Inject Drugs
QA	Quality Assurance
QC	Quality Control
QoL	Quality of Life
SAE	Serious Adverse Event/Serious Adverse Experience
SUSAR	Suspected Unexpected Serious Adverse Reaction
SD	Standard Deviation
SOC	Standard of Care
TOC	Test of Cure
ULN	Upper Limit of Normal
US	United States

PROTOCOL SUMMARY

**Title:**	Dalbavancin as an Option for Treatment of *S. aureus* Bacteremia (DOTS): A Phase 2b, Multicenter, Randomized, Open-Label, Assessor-Blinded Superiority Study to Compare the Efficacy and Safety of Dalbavancin to Standard of Care Antibiotic Therapy for the Completion of Treatment of Patients with Complicated *S. aureus* Bacteremia
**Design of the Study:**	Multicenter, randomized, open-label, assessor-blinded, superiority, active-controlled, parallel-group study
**Study Phase:**	2b
**Study Population:**	200 adult subjects (≥ 18 years old) diagnosed with complicated *S. aureus* bacteremia including definite or possible right-sided infective endocarditis (IE) treated with effective antibiotic therapy for at least 72 hours (maximum 10 days) and with subsequent clearance of bacteremia prior to randomization to study treatment
**Number of Sites:**	Approximately 20
**Description of Study Product or Intervention:**	• Dalbavancin 1500 mg intravenously (IV) over 30 (± 10) minutes on Day 1 and 1500 mg intravenously (IV) over 30 (± 10) minutes on Day 8 (if creatinine clearance ≥ 30 mL/min or if on regular hemodialysis or peritoneal dialysis)<br>• Dalbavancin 1125 mg IV over 30 (±10) minutes on Day 1 and Dalbavancin 1125 mg IV over 30 (±10) minutes on Day 8 (if creatinine clearance < 30 mL/min and not receiving regular hemodialysis or peritoneal dialysis)
**Study Objectives:**	Primary:

- To compare the Desirability of Outcome Ranking (DOOR) at Day 70 of dalbavancin to that of standard of care antibiotic therapy used to consolidate therapy for the treatment of subjects with complicated *S. aureus* bacteremia in the intent-to-treat (ITT) population.

Secondary:

- To compare the clinical outcomes of dalbavancin with the standard of care antibiotic therapy at day 70 in the modified intent-to-treat (mITT) population.
- To compare the safety of dalbavancin with that of the standard of care treatment in the mITT.
- To compare each individual component of the DOOR outcome by treatment arm, in the ITT population.

Exploratory:

- To compare the clinical outcomes of dalbavancin with the standard of care antibiotic therapy at day 70 in the clinically evaluable (CE) population.
- To compare the DOOR endpoint of subjects on dalbavancin with that of subjects receiving standard of care antimicrobial therapy at day 42 in the ITT, mITT, and CE populations.
- To compare the clinical and microbiologic outcomes of dalbavancin with the standard of care antibiotic therapy at day 42 in ITT, mITT and CE populations.
- To compare clinical and microbiologic outcomes of dalbavancin with standard of care antibiotic therapy between clinically important subgroups, including a) those with MSSA versus MRSA; b) persons who inject drugs (PWID) vs non-PWID; c) those who received infectious disease consultation vs those who did not; d) underlying site of infection (endovascular, bone and joint, skin, pulmonary); e)

subjects with immune-suppression[1]; f) divided by duration of initial bacteremia in the ITT, mITT, and CE populations

- To compare Quality of Life (QoL) of subjects on dalbavancin with that of subjects receiving standard of care antibiotic therapy at Baseline, Day 42, and Day 70, in the ITT, mITT, and CE populations
- To characterize the population pharmacokinetic profile for dalbavancin administered via a 2-dose regimen (1500 mg on day 1 and day 8, renally adjusted when appropriate) in patients with *Staphylococcus aureus* bacteremia
- To assess patient-level and clinical covariates associated with dalbavancin pharmacokinetics in patients with *Staphylococcus aureus* bacteremia
- Examine the association between individualized plasma concentration profiles and clinical and microbiologic outcomes at day 42 and TOC
- Examine the association between individualized plasma concentration profiles and occurrence of adverse drug events, including AST/ALT elevations >3X upper limit of normal
- Examine the association between individualized plasma concentration profiles and late recurrence risk among the subset of patients with osteomyelitis and a 6 month follow-up visit

Duration of Individual Subject Participation: Approximately 70 ± 7 days, with a late post-treatment follow-up visit for the subset of patients with osteomyelitis at 6 months

[1] Defined as: On chemotherapy or immunotherapy for active hematologic malignancy expected to cause ANC < 500 cells/mm^3 lasting > 7 days during the study period, chronic high dose oral steroids (equivalent of ≥ 20 mg prednisolone per day for or equivalent, for >2 weeks within the last month), HIV infection with a CD4 cell count < 100 cells/mm^3 based on last known measurement or patient-reported value

**Estimated Time to Last Subject/Last Study Day:**	Approximately 30 months, from site activation to the last subject's last study day.

Table 1: Treatment Arms

Dalbavancin	100 subjects	Dalbavancin 1500 mg IV over 30 (± 10) minutes on Day 1 and 1500 mg IV over 30 (± 10) minutes on Day 8, renally dose-adjusted to 1125 mg for subjects with CrCl <30 and not on dialysis
Standard of Care[a]	100 subjects	• Methicillin-sensitive *Staphylococcus aureus* (MSSA): nafcillin (2 g IV q4h × 4-6 weeks)[b] OR oxacillin (2 g IV Q4h x 4-6 weeks)[b] OR cefazolin (2 g IV q8h × 4-6 weeks) [b,c]<br>• Methicillin-resistant *Staphylococcus aureus* (MRSA): vancomycin (dose per local standard of care × 4-6 weeks) OR daptomycin (6-10 mg/kg IV daily × 4-6 weeks)[c]

[a]Renally dose-adjusted as appropriate, per local standard of care

[b]As applicable per site standard of care, beta-lactams may be administered at an equivalent dose via continuous IV infusion (e.g., nafcillin 12g/24h IV continuous)

[c]If there are extenuating circumstances in which preferred standard of care antibiotics cannot be used, for example complex allergy history, then an alternative antibiotic may be used after discussion with the protocol PIs and DMID Medical Officer

Figure 1: Schematic of Study Design

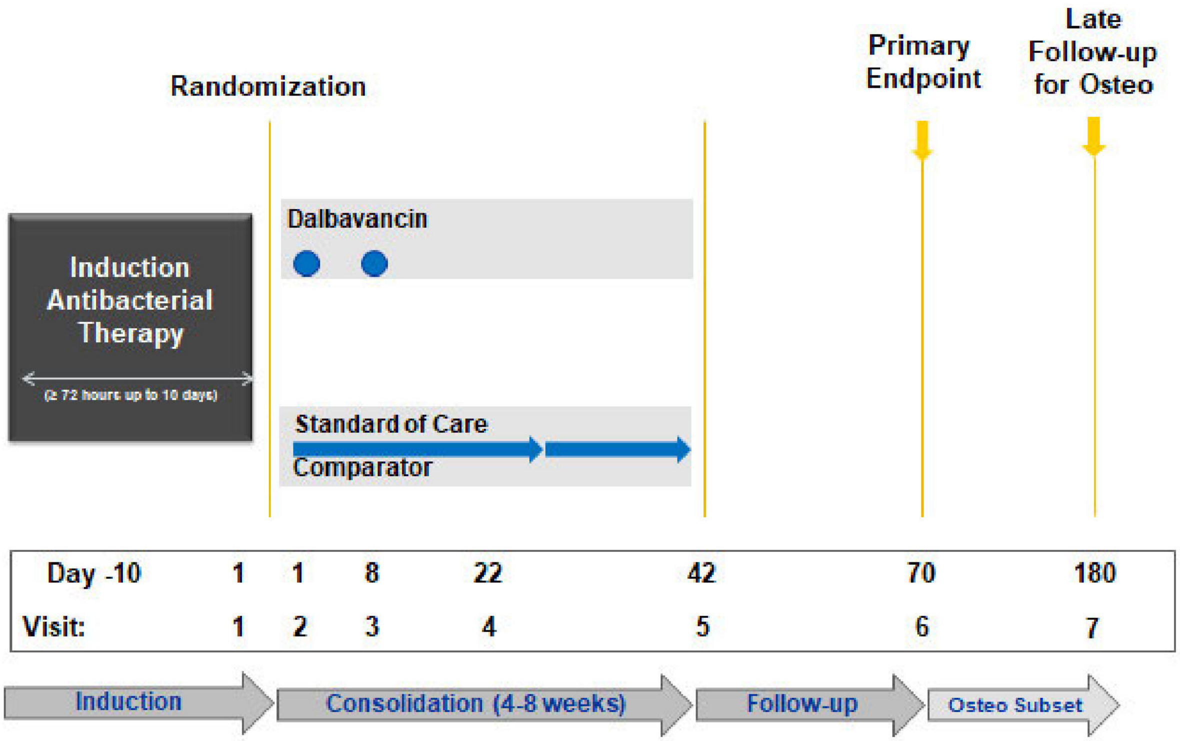

1 KEY ROLES

**Lead Principal Investigator:**	Thomas L. Holland, MD<br>Associate Professor of Medicine, Duke University<br>[redacted]<br>Duke University Medical Center<br>Durham, NC, 27710<br>[redacted]
**DMID Clinical Project Manager:**	[redacted]
**Statistical and Data Coordinating Center:**	The Emmes Company, LLC<br>401 N. Washington St. Suite 700<br>Rockville, MD 20850<br>[redacted]

2 BACKGROUND AND SCIENTIFIC RATIONALE

2.1 Background

Staphylococcus aureus bacteremia is a life-threatening infection with case fatality rates ranging from 15-50% (van Hal 2012). *S. aureus* bacteremia is also a leading cause of IE in industrialized nations (Tong 2015) and is associated with in-hospital mortality rates ranging from 16% to 25% (Thuny 2005, Murdoch 2009, Sy 2010, Selton-Suty 2012). Standard treatment of complicated *S. aureus* bacteremia and IE requires prolonged IV antibiotic therapy (4-6 weeks), typically necessitating placement of a central IV catheter, prolonged hospitalization, and home nursing or admission to a long-term care facility. Treatment is associated with high cost and healthcare burden, as well as complications such as catheter related bloodstream infections and catheter associated thrombosis (Keller 2018). Safe and effective alternative treatment strategies are needed.

Dalbavancin is a lipoglycopeptide that has potent activity against Gram-positive pathogens including MRSA (Table 2, Table 3). It is currently approved for treatment of acute bacterial skin and skin structure infection in the US and EU as a single dose or 2-dose regimen. A 2-dose, once weekly regimen can provide systemic therapy for 6 weeks, eliminating the need for a centrally placed catheter or prolonged IV access for antibiotic administration.

Table 2: Activity of Dalbavancin Against Gram-Positive Pathogens Collected in 2014 US Surveillance

		Dalbavancin MIC (µg/mL)			
Organism	N	Range	50%	90%	% Susceptible[a]
S aureus: all	1625	0.008 - 0.12	0.06	0.06	100
Methicillin-susceptible	875	0.008 - 0.12	0.06	0.06	100
Methicillin-resistant	750	0.008 - 0.12	0.06	0.06	100
Viridans group streptococci	220	≤ 0.002 - 0.12	0.015	0.03	100
Penicillin-nonsusceptible	46	0.004 - 0.06	0.015	0.03	100
S pyogenes	108	≤ 0.002 - 0.06	0.008	0.03	100
S dysgalactiae	75	≤ 0.004 - 0.12	0.015	0.03	100
E faecalis	151	0.02 - > 0.25	0.06	0.06	97.6

Bacterial pathogens frequently implicated as causative agents of IE include *S aureus* (including MRSA), *Streptococcus pyogenes,* viridans group streptococci, Group C and G streptococci, and *Enterococcus* spp.

IE = infective endocarditis; MRSA = methicillin-resistant *S aureus*; MIC = minimum inhibitory concentration

[a] Susceptible at ≤ 0.25 µg/mL

Source: Dalbavancin International (Two Continents) Surveillance Report for 2014, JMI Laboratories; Protocol 14-DUR-01, May 2015.

Table 3: Activity of Dalbavancin Against Gram –Positive Pathogens Collected in 2014 EU Surveillance

		Dalbavancin MIC (µg/mL)			
Organism	N	Range	50%	90%	% Susceptible[a]
S aureus: all	1625	0.004 - 0.12	0.06	0.06	100
Methicillin-susceptible	1209	0.015 - 0.12	0.06	0.06	100
Methicillin-resistant	416	0.004 - 0.12	0.03	0.06	100
Viridans group streptococci	213	≤ 0.002 - 0.12	0.015	0.03	100
Penicillin-nonsusceptible	57	≤ 0.002 - 0.06	0.015	0.03	100
S pyogenes	106	0.004 - 0.12	0.008	0.03	100
S dysgalactiae	94	≤ 0.002 - 0.12	0.015	0.03	100
E faecalis	305	0.03 - > 0.25	0.06	0.06	98.4

Bacterial pathogens frequently implicated as causative agents of IE include *S aureus* (including MRSA), *Streptococcus pyogenes,* viridans group streptococci, Group C and G streptococci, and *Enterococcus* spp.

IE = infective endocarditis; MRSA = methicillin-resistant *S aureus*; MIC = minimum inhibitory concentration

[a] Susceptible at ≤ 0.25 µg/mL

Source: Dalbavancin International (Two Continents) Surveillance Report for 2014, JMI Laboratories; Protocol 14-DUR-01, May 2015.

Dalbavancin appeared effective in rat and rabbit models of *S. aureus* IE (Candiani 1999, Lefort 2004). In the rat model of staphylococcal IE, dalbavancin was as effective as vancomycin and teicoplanin at reducing the bacterial load in the heart, but with a lower dose and less frequent dosing

intervals compared to standard of care (Candiani 1999). In a rabbit model of IE, dalbavancin given once daily (10 mg/kg for 4 days) or as a single dose of 40 mg/kg was effective against a strain of *S. aureus* with reduced susceptibility to vancomycin and teicoplanin (Lefort, 2004). Additionally, 5 Phase 2/3 clinical studies that evaluated the efficacy and safety of dalbavancin administered as a single dose or 2-dose regimen found that 100% of patients (55 of 55 evaluable subjects) with *S. aureus* bacteremia achieved clearance of bacteremia (Raad 2005, Dunne 2016, Boucher 2014, Selzter 2003, Jauregui 2005). Thus, dalbavancin may be a safe and effective alternative therapy for *S. aureus* bacteremia/IE, including highly antibiotic-resistant strains.

The safety profile of dalbavancin has been characterized for a total cumulative dose of 1500 mg, whether administered as a single dose or in split weekly doses (1000 mg on Day 1 followed by 500 mg on Day 8). Adverse reactions have been evaluated for 2473 patients treated with dalbavancin. Overall, the most common adverse reactions were nausea (4.7%), headache (3.8%), and diarrhea (3.4%). The median duration of adverse reactions was 3 days for patients receiving dalbavancin and 4 days for patients receiving a comparator. The safety database for the 3000 mg total dose includes 12 subjects from a Phase 1 study: 6 received a total of 3500 mg dalbavancin over 6 weeks (with a total of 2 mild adverse events), and 6 received a total of 4500 mg dalbavancin over 8 weeks (with total of 4 mild adverse events and 1 moderate adverse event). Elevations in ALT >3x the upper limit of normal have been reported, though at a frequency not significantly different than comparator arms in trials conducted to date. Per product insert, 4.9% (121/2473) subjects receiving dalbavancin experienced SAEs, with 2.6% (64/2473) discontinuing the medication as a result.

2.2 Scientific Rationale

2.2.1 Purpose of Study

The current standard of care for the antibiotic treatment of complicated bacteremia or IE uses a stepwise approach. The initial phase of treatment involves the initiation of empirical antibiotic therapy, definitive diagnosis (as per the modified Duke criteria), and the assessment of the need for early valve replacement, if applicable. Subsequent identification of the causative pathogen, including antibiotic susceptibility and MICs supports the choice of definitive antibiotic therapy and determination of the required duration of antibiotic treatment. Guidelines for the use of outpatient parenteral antibiotic therapy in the treatment of complicated bacteremia or IE similarly advocate that antibiotic therapy can be divided into an initial phase during which life threatening complications of complicated bacteremia or IE are likely to occur (approximately 14 days) and a completion phase of therapy (2 weeks to 6 weeks) (Holland 2018).

The proposed clinical study design of dalbavancin in the treatment of complicated bacteremia or IE is consistent with this standard of care. Specifically, prior to study eligibility, patients will receive pre-randomization antibiotic therapy pending a definitive diagnosis of complicated *S. aureus*

bacteremia or IE, as well as the resolution of bacteremia. Eligible patients will then be randomized into the study to complete their antibiotic therapy with either a 2-dose regimen of dalbavancin or the current standard of care with daily IV administration of antibiotic therapy for a total duration of 4 to 6 weeks (Baddour 2015) for most patients, and up to 8 weeks for patients with vertebral osteomyelitis/discitis.

The proposed clinical study design offers a number of advantages. First, it will support enrollment of patients with a confirmed diagnosis of complicated bacteremia or IE. Prior studies have been limited by the inability to confidently determine complicated vs uncomplicated status prior to randomization (Corey 2009). Second, the proposed study design addresses a true area of need in *S. aureus* bacteremia management and reflects the likely pattern of “real world” dalbavancin use by clinicians for the completion of systemic antibiotic therapy for complicated bacteremia or IE without the need for indwelling IV access to support daily therapy. This takes full advantage of the unusual PK profile of dalbavancin and the introduction of this therapy into clinical practice would potentially have a major impact on patient well-being and quality of life (QoL). In this study, presence or absence of these potential QoL effects will be assessed using an ARLG Bloodstream Infection QoL Measure developed specifically for this purpose (King 2020). Additionally, two previously validated measures (EQ-5D-5L, https://euroqol.org/eq-5d-instruments/sample-demo/ and the PROMIS Global Health short form, http://www.healthmeasures.net/administrator/components/com_instruments/uploads/Global%20Health%20Scale%20v1.2%2008.22.2016.pdf) will also be collected. Third, adequate treatment of complicated bacteremia or IE requires prolonged systemic antibiotic therapy to prevent relapse. Introduction of the 2-dose dalbavancin regimen may decrease the risk of relapse. Finally, the proposed design of this clinical study is consistent with antibiotic stewardship principles, reserving dalbavancin therapy for patients with fully characterized infections and pathogens.

2.2.2 Study Population

Approximately 200 eligible subjects will be enrolled to the study in the United States and Canada. Eligible subjects are adults who have been diagnosed with complicated bacteremia or right-sided IE due to *S. aureus*, have been treated with appropriate empiric/targeted antibiotic therapy, and in whom the blood cultures have become negative after at least 72 hours of initial antibiotic therapy (maximum 10 days). Subjects with uncomplicated bacteremia due to *S. aureus* will be excluded. More details regarding subject inclusion and exclusion criteria can be found in Section 5.1.

2.2.3 Selection of Dose

The dalbavancin dosing regimen consists of 1500 mg on Day 1 and 1500 mg on Day 8 for subjects with normal renal function or who are receiving dialysis, administered over 30 minutes by IV infusion. Patients with CrCl <30 who are not receiving dialysis will receive a reduced dose (1125mg on Day 1 and 1125 mg on Day 8). Based on a comparison to the updated nonclinical

pharmacokinetic (PK)/pharmacodynamics (PD) target of the area under the unbound drug concentration-time curve [fAUC]/MIC (Lepak 2015), this regimen is expected to provide sufficient therapeutic concentrations of free drug against *S. aureus* through Day 42.

Consistent with prior nonclinical investigation (Andes 2007), Lepak et al (Lepak 2015) found fAUC/MIC to be the most relevant PK/PD index in a neutropenic murine thigh infection model, with mean free drug daily area under the curve (AUC)/minimum inhibitory concentration (MIC)s for net stasis, 1log kill, and 2log kill of 27.1, 53.3, and 111.1, respectively. To justify the proposed dosing regimen, a target attainment analysis was conducted using the updated population PK model (Carrothers 2020). In the PK simulation, the proposed regimen was simulated using the Bayesian post hoc estimates of each of the 703 patients in the merged Phase 2/3 population PK dataset. As with previous target attainment analyses, free drug levels were assumed to be 7% of total drug concentrations. As a conservative assumption, the mean daily AUC for target attainment was calculated based on dalbavancin levels on Day 42. For the MIC, the dalbavancin *S. aureus* MIC_{90} of 0.06 mg/L was used. Results of this simulation analysis showed target attainments of > 99%, > 99%, and 90% for the net stasis, 1log kill, and 2log kill targets, respectively. For an even more conservative estimate, the US breakpoint for susceptibility of *S. aureus* to dalbavancin of 0.25 mg/L was also used: the 90% target attainment was achieved through Day 42 (stasis), Day 36 (1-log kill), and Day 28 (2-log kill) after clearance of bacteremia.

Simulations designed to evaluate plasma concentration-time profiles suggest that a 2-dose regimen of dalbavancin of 1500 mg given on Days 1 and 8 will provide plasma concentrations above the MIC_{99} of *S. aureus* for an average of 49 days after the start of therapy Figure 2.

Figure 2: Simulated Mean Plasma PK Profile for Dalbavancin (Source: Allergen data on file)

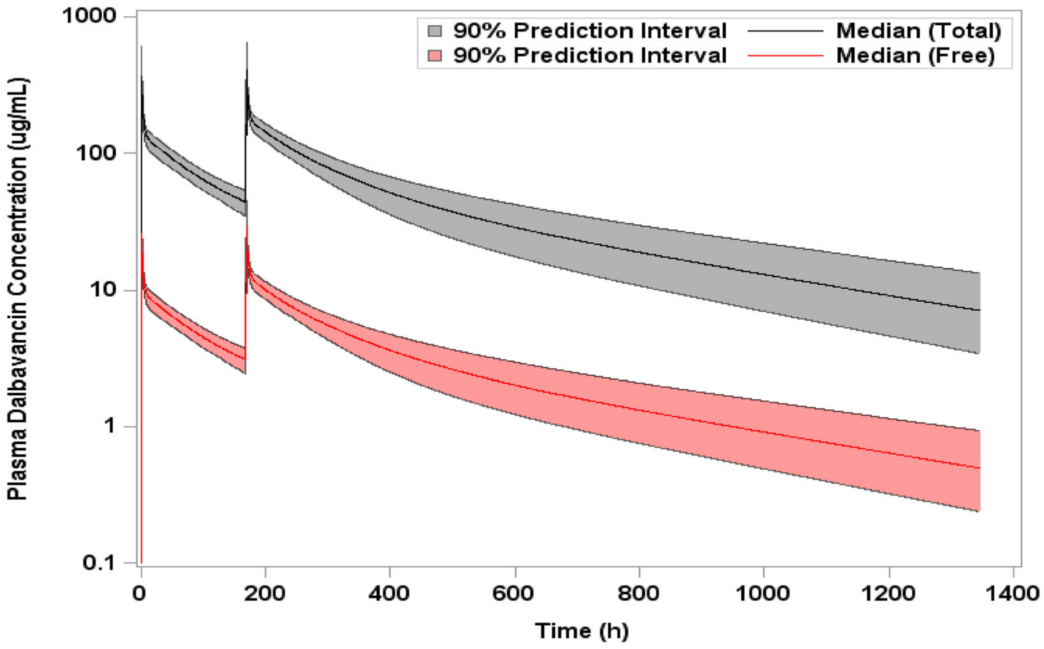

Figure 3 shows the time course of total dalbavancin concentration over the 6-week course of therapy for a typical patient. For this typical patient, Day 42 total drug concentration was 6.7 µg/mL [free drug = 7% × 6.7 = 0.47 µg/mL], which is greater than 7-fold the MIC_{90} value of 0.06 µg/mL for *S. aureus*, and > 15-fold the MIC_{90} value of 0.03 µg/mL for beta-hemolytic streptococci (Dalbavancin International [Two Continents] Surveillance Report for 2014) even at the end of a 6-week course of therapy.

Figure 3: Dalbavancin Concentration Time Course for a Typical Subject Under the Proposed Regimen (Source: Allergen data on file)

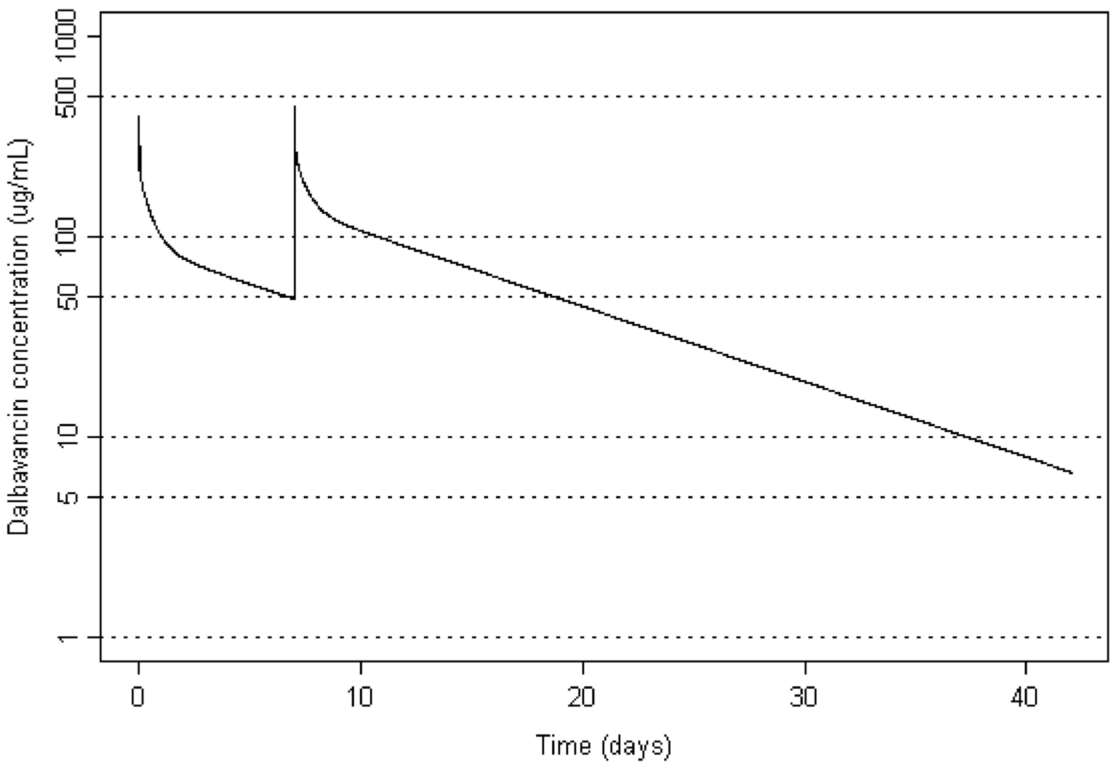

2.3 Potential Risks and Benefits

Dalbavancin is FDA-approved for the treatment of acute bacterial skin and skin structure infections caused by Gram positive organisms, though at lower doses than proposed for treatment of bacteremia here and has been the subject of one prior randomized controlled clinical trial for osteomyelitis using the same dosing regimen proposed in this study. Risk information is derived from both the product insert and previously conducted trials.

For those randomized to standard of care arms, the antistaphylococcal beta-lactams, cefazolin, vancomycin and daptomycin are all commonly used for treatment of *S. aureus* bacteremia and are associated with potential risks as well – outlined below from product inserts. These include risks associated with an indwelling vascular catheter, primarily secondary infections or thromboses. These also include the need for frequent blood draws to monitor for antibiotic toxicities.

2.3.1 Potential Risks

2.3.1.1 Dalbavancin

Hypersensitivity Reactions: Hypersensitivity reactions (both anaphylactic and limited cutaneous reactions) have been reported with glycopeptide antibiotics, including dalbavancin. Subjects will be excluded from this study if they report any history of anaphylactic reaction to glycopeptide antibiotics or dalbavancin.

Infusion-related Reactions: Rapid infusion of dalbavancin has been reported to cause flushing, urticarial, pruritic, or rash reactions resembling the "Red-Man Syndrome" described in association with vancomycin. Stopping or slowing the infusion typically resolves these reactions.

Central Nervous System Reactions: Headache has been infrequently reported with dalbavancin receipt (<4%).

Gastrointestinal: Nausea (4.7%) and diarrhea (<4%) have been infrequently reported with dalbavancin receipt (<4%).

C. difficile Infection: *C. difficile*-associated diarrhea has been reported with nearly all antibacterial agents, including dalbavancin.

Hepatic: Elevations of AST/ALT have been reported in clinical trials.

2.3.1.2 Cefazolin

Hypersensitivity Reactions: Anaphylaxis and a range of cutaneous drug eruptions have been reported with cefazolin.

Localized Reactions: Phlebitis has been rarely reported with cefazolin.

Nephrotoxicity: Elevations in BUN and creatinine, infrequently even acute renal failure, have been reported.

Gastrointestinal: Nausea, diarrhea and anorexia have been reported.

C. difficile Diarrhea: *C. difficile* diarrhea has been reported with nearly all antibiotics.

Hematologic: Neutropenia and thrombocytopenia have both been infrequently reported and are generally reversible upon discontinuation.

Hepatic: Transient elevations in AST/ALT have been reported.

2.3.1.3 Nafcillin

Hypersensitivity Reactions: Anaphylaxis and a range of cutaneous drug eruptions have been reported with nafcillin.

Localized Reactions: Phlebitis, sometimes even with skin sloughing, has been rarely reported with nafcillin.

Nephrotoxicity: Elevations in BUN and creatinine, infrequently even acute renal failure or acute interstitial nephritis, have been reported.

Gastrointestinal: Nausea, diarrhea and anorexia have been reported.

C. difficile Diarrhea: *C. difficile* diarrhea has been reported with nearly all antibiotics.

Hematologic: Neutropenia or even agranulocytosis has been reported with nafcillin and is generally reversible upon discontinuation.

Hepatic: Transient elevations in AST/ALT have been reported.

2.3.1.4 Oxacillin

Hypersensitivity Reactions: Anaphylaxis and a range of cutaneous drug eruptions have been reported with oxacillin.

Nephrotoxicity: Elevations in BUN and creatinine, infrequently even acute renal failure or acute interstitial nephritis, have been reported.

Gastrointestinal: Nausea, diarrhea and anorexia have been reported.

C. difficile Diarrhea: *C. difficile* diarrhea has been reported with nearly all antibiotics.

Hematologic: Neutropenia or even agranulocytosis has been reported with oxacillin and is generally reversible upon discontinuation.

Hepatic: Transient elevations in AST/ALT have been reported.

2.3.1.5 Vancomycin

Infusion Reactions: Rapid infusion of vancomycin has been reported to cause flushing, urticarial, pruritic, or rash reactions known as the “Red-Man Syndrome”. Stopping or slowing the infusion typically resolves these reactions.

Phlebitis and Localized Cutaneous Reactions: Vancomycin can be irritating to soft tissues, including causing thrombophlebitis at infusion sites. Inadvertent extravasation may result in localized pain, tenderness, or even necrosis.

Hypersensitivity Reactions: Hypersensitivity reactions including anaphylaxis have been reported with vancomycin.

Nephrotoxicity: When administered systemically, vancomycin can cause acute kidney injury. Risk of injury increases with supratherapeutic levels.

Ototoxicity: Hearing loss and tinnitus have been reported with vancomycin, reported mostly in patients with supratherapeutic levels, underlying hearing loss, or concomitant receipt of other ototoxic agents.

C. difficile Diarrhea: *C. difficile* associated diarrhea has been reported with the use of nearly all antibiotics.

Hematologic: Vancomycin has occasionally been associated with neutropenia or rarely thrombocytopenia. Cytopenias are usually reversible upon discontinuation.

2.3.1.6 Daptomycin

Hypersensitivity Reactions: Anaphylaxis and a range of cutaneous drug eruptions have been reported with daptomycin.

Myopathy/Rhabdomyolysis: Daptomycin has been reported to cause a myositis characterized by elevated creatine kinase levels, muscle pain, and muscle weakness. Risk may be increased upon co-administration with statins. Myopathy is generally reversible upon discontinuation.

Eosinophilic Pneumonia: Daptomycin has been rarely reported to cause eosinophilic pneumonia which may present with fever, dyspnea and pulmonary opacities.

Neuropathy: There are rare reports of peripheral neuropathy with daptomycin.

C. difficile Diarrhea: *C. difficile* diarrhea has been reported with nearly all antibiotics.

2.3.1.7 Risks Associated with Intravenous Access

The risks associated with peripheral IV placement are minimal and include minor bruising/bleeding, localized discomfort at the site, and rarely infection or a superficial clot.

The risks associated with peripherally inserted central venous catheters (PICC lines) or central lines include bleeding, discomfort at the site, deep venous thrombosis (blood clot), infection, rarely nerve injury or an irregular heartbeat.

2.3.2 Potential Benefits

Dalbavancin may or may not improve the clinical outcome of an individual subject who participates in this trial. There is potential benefit to society from their participation in this study resulting from insights gained about outcomes with dalbavancin among patients with *S. aureus* bacteremia. Since standard of care for complicated *S. aureus* bacteremia currently requires dosing via indwelling central venous access, with frequent dosing and prolonged duration of therapy, the potential benefit is the possible demonstration that a dalbavancin treatment strategy may avoid prolonged hospital stays and the need for central venous access.

3 STUDY DESIGN, OBJECTIVES AND ENDPOINTS OR OUTCOME MEASURES

3.1 Study Design Description

This clinical study will be a Phase 2b, multicenter, randomized, open-label, assessor-blinded, superiority study. The study will compare dalbavancin to standard of care antibiotic therapy for the completion of therapy in patients with complicated bacteremia or right-sided native valve IE caused by *S. aureus* who have cleared their baseline bacteremia.

Approximately 200 subjects will be randomized 1:1 to receive either dalbavancin or a standard of care antibiotic regimen that is based upon the identification and antibiotic susceptibility pattern of the baseline organism. Those randomized to the dalbavancin treatment group will receive 2 doses of dalbavancin IV 1 week apart (1500 mg on Day 1 and Day 8 after randomization, with renal dose adjustment if appropriate). Those subjects randomized to the standard of care antibiotic therapy treatment group will receive an antibiotic regimen considered to be standard of care based on the methicillin susceptibility pattern of the pathogen isolated at Baseline for a duration of 4 to 6 weeks.

Figure 1 provides a schematic of the study design. Study procedures are presented in Section 6. Detailed descriptions of each study visit can be found in Section 6.3.

3.2 Study Objectives

3.2.1 Primary

- To compare the Desirability of Outcome Ranking (DOOR) at Day 70 of dalbavancin to that of standard of care antibiotic therapy used to consolidate therapy for the treatment of subjects with complicated *S. aureus* bacteremia in the intent-to-treat (ITT) population.

3.2.2 Secondary

- To compare the clinical outcomes of dalbavancin with the standard of care antibiotic therapy at day 70 in the mITT population (see Section 10.5.1 for definitions of study populations).
- To compare the safety of dalbavancin with that of the standard of care treatment in the mITT population.
- To compare each individual component of the DOOR outcome by treatment arm, in the ITT population.

3.2.3 Exploratory

1. To compare the clinical outcomes of dalbavancin with the standard of care antibiotic therapy at day 70 in the CE population (see Section 10.5.1 for definition).
2. To compare the DOOR endpoint of subjects on dalbavancin with that of subjects receiving standard of care antimicrobial therapy at day 42, in the ITT, mITT and CE populations.
3. To compare the clinical and microbiologic outcomes of dalbavancin with the standard of care antibiotic therapy at day 42 in ITT, mITT and CE populations.
4. To compare clinical and microbiologic outcomes of dalbavancin with standard of care antibiotic therapy between clinically important subgroups, including a) those with MSSA versus MRSA; b) persons who inject drugs (PWID) vs non-PWID; c) those who received infectious disease consultation vs those who did not; d) underlying site of infection (endovascular, bone and joint, skin, pulmonary); e) subjects with immune-suppression[2]; f) divided by duration of initial bacteremia, in the ITT, mITT, and CE populations.
5. To compare Quality of Life (QoL) of subjects on dalbavancin with that of subjects receiving standard of care antibiotic therapy at Baseline, Day 42, and Day 70, in the ITT, mITT, and CE populations.
6. To characterize the population pharmacokinetic profile for dalbavancin administered via a 2-dose regimen (1500 mg on day 1 and 1500 mg on day 8; renally adjusted when appropriate) in patients with *S. aureus* bacteremia.
7. To assess patient-level and clinical covariates associated with dalbavancin pharmacokinetics in patients with *S. aureus* bacteremia.
8. Examine the association between individualized plasma concentration profiles and clinical and microbiologic outcomes at day 42 and TOC.
9. Examine the association between individualized plasma concentration profiles and occurrence of adverse drug events, including AST/ALT elevations >3X upper limit of normal.
10. Examine the association between individualized plasma concentration profiles and late recurrence risk among the subset of patients with osteomyelitis and a 6-month follow-up visit.

[2] Defined as: On chemotherapy or immunotherapy for active hematologic malignancy expected to cause ANC < 500 cells/mm^3 lasting > 7 days during the study period, chronic high dose oral steroids (equivalent of ≥ 20 mg prednisolone per day for or equivalent, for >2 weeks within the last month), HIV infection with a CD4 cell count < 100 cells/mm^3 based on last known measurement or patient-reported value

3.3 Study Endpoints or Outcome Measures

3.3.1 Primary

The **primary outcome** measure is the DOOR endpoint at day 70. The clinical components of the DOOR endpoint (success/failure and infectious complications) will be completed by an independent adjudication committee, blinded to treatment assignment. Day 70 was selected in this study as it occurs approximately 4 weeks after treatment completion for most patients, allowing time for detection of relapse.

There are 5 possible rankings in the DOOR endpoint:

Rank	Alive	How many of: 1) Clinical Failure 2) Infectious Complication 3) SAE, or AE leading to study drug discontinuation	QoL
1	Yes	0 of 3	Tiebreaker based on QoL score
2	Yes	1 of 3	
3	Yes	2 of 3	
4	Yes	3 of 3	
5	No (Death)	Any	

Rank 1: Alive without any of the following: (1) evidence of clinical failure; (2) an infectious complication; or (3) any SAE, or an AE leading to study drug discontinuation

Rank 2: Alive but with one of the following: (1) evidence of clinical failure; (2) an infectious complication; or (3) any SAE, or an AE leading to study drug discontinuation

Rank 3: Alive but with two of the following: (1) evidence of clinical failure; (2) an infectious complication; or (3) any SAE, or an AE leading to study drug discontinuation

Rank 4: Alive but with all of the following: (1) evidence of clinical failure; (2) an infectious complication; or (3) any SAE, or an AE leading to study drug discontinuation

Rank 5: Death

Clinical failure, infectious complications, and adverse events are each binary (yes/no) components of the DOOR endpoint.

For the primary endpoint, quality of life will be measured as a change from baseline QoL to day 70 QoL score, as assessed by the ARLG Bloodstream Infection QoL Measure (Appendix C).

<u>Definitions for the DOOR endpoint:</u>

Clinical Success: Resolution of clinical signs and symptoms of *S. aureus* bacteremia such that no additional antibiotic therapy is required or anticipated for its treatment.

Note that it is possible to achieve this overall Clinical Success status at Day 70 even if Infectious Complications have occurred prior to that time. For example, a patient who has a new metastatic focus of infection diagnosed after randomization, but who subsequently completes treatment and is felt to be cured at Day 70 would be considered a Clinical Success (and the Infectious Complication would result in a lower DOOR). That is, Clinical Success reflects the patient's overall status at the time of that assessment.

Clinical Failure: Absence of clinical success

Infectious Complications: occurrence of any of the following, between randomization and day 70:

- Endocarditis
- New evidence of metastatic foci of infection – e.g. osteomyelitis, visceral abscess, septic joint
- Relapse – isolation of baseline *S. aureus* pathogen from a blood culture drawn after randomization
- Readmission for subsequent care of indication under study
- Need for additional unplanned source control procedures – e.g. abscess debridement or drainage, cardiac valve replacement
- Change in antibiotic therapy due to inadequate clinical response. For any changes to study drug in the standard of care group, or when new antibiotics are started in either treatment group, the site PI will record the reason for the antibiotic change.

3.3.2 Secondary

The **secondary efficacy outcome** is as follows**.**

- Clinical efficacy, defined as none of: 1) Clinical failure; 2) Infectious complications; 3) All-cause mortality

The **secondary safety outcome** is as follows.

- Proportion of patients who have either 1) an SAE; or 2) an AE leading to study drug discontinuation

Each component of the DOOR will also be examined separately:

- clinical success
- infectious complications
- SAEs
- AEs leading to study drug discontinuation
- all-cause mortality

3.3.3 Exploratory

1. Clinical efficacy by treatment group in the CE population at day 70
2. DOOR endpoint by treatment group at day 42 in the ITT, mITT, and CE populations
3. Clinical and microbiologic outcomes by treatment group at day 42 in the ITT, mITT, and CE populations
4. Clinical and microbiologic outcomes by treatment group, within each subgroup of clinical interest, at day 42 and day 70 in the ITT, mITT, and CE populations. A microbiologic success will be defined as no post-randomization growth (e.g., no positive cultures) of the baseline pathogen from blood cultures or another sterile body site
5. QoL at Baseline, Day 42, and Day 70, which will be assessed using the ARLG Bloodstream Infection QoL Measure (Appendix C) as well as two additional comparator measures (EQ-5D-5L, https://euroqol.org/eq-5d-instruments/sample-demo/ and PROMIS Global Health short form, http://www.healthmeasures.net/administrator/components/com_instruments/uploads/Global%20Health%20Scale%20v1.2%2008.22.2016.pdf) in the ITT, mITT, and CE populations
6. Population mean PK parameter estimates and the magnitude of the associated inter individual variability for the 2-dose dalbavancin regimen in patients with *S. aureus* bacteremia.
7. Individual post hoc PK parameter estimates and calculated exposure measures for the 2-dose dalbavancin regimen in patients with *S. aureus* bacteremia.
8. Clinical and microbiologic response at day 42 and Day 70 according to individual plasma dalbavancin concentration curves.
9. Occurrence of grade 3 or higher adverse drug events, adverse events of special interest (AESIs), and occurrence of AST/ALT elevations >3x upper limit of normal (ULN) from first dose of dalbavancin through follow-up period.
10. Late recurrence within the osteomyelitis population will be defined by the presence of the following up to 6 months after randomization: progressive imaging changes along with isolation of *S. aureus* from blood, bone biopsy, associated fluid aspiration, or operative tissue culture.

4 STUDY INTERVENTION/INVESTIGATIONAL PRODUCT

4.1 Study Product Description

Dalbavancin is a lyophilized, white to off-white to pale yellow solid. It is a lipoglycopeptide synthesized from a fermentation product of *Nonomuraea* species. Dalbavancin is a mixture of five closely related active homologs (A_0, A_1, B_0, B_1, and B_2), shown in Table 4, below. The homolog B_0 is the major component of dalbavancin. The homologs share the same core structure and differ in the fatty acid side chain of the N-acylaminogluronic acid moiety (R_1) structure and/or the presence of an additional methyl group (R_2) on the terminal amino group, shown in Table 4 below. The B_0 INN chemical name is 5,31-dichloro-38-de(methoxycarbonyl)-7-demethyl-19-deoxy-56-O [2-deoxy-2-[(10-methylundecanoyl)amino]-β-D-glucopyranuronosyl]-38-[[3-(dimethylamino)propyl] carbamoyl]-42-O-α-D-mannopyranosyl-15-N-methyl(ristomycin A aglycone) hydrochloride. Please refer to the package insert and/or Investigator's Brochure for further details.

Table 4: Substitution Patterns for Dalbavancin API Homologs

Dalbavancin	R_1	R_2	Molecular Formula	Molecular Weight*
A_0	$CH(CH_3)_2$	H	$C_{87}H_{98}N_{10}O_{28}Cl_2 \cdot 1.6\ HCl$	1802.7
A_1	$CH_2CH_2CH_3$	H	$C_{87}H_{98}N_{10}O_{28}Cl_2 \cdot 1.6\ HCl$	1802.7
B_0	$CH_2CH(CH_3)_2$	H	$C_{88}H_{100}N_{10}O_{28}Cl_2 \cdot 1.6\ HCl$	1816.7
B_1	$CH_2CH_2CH_2CH_3$	H	$C_{88}H_{100}N_{10}O_{28}Cl_2 \cdot 1.6\ HCl$	1816.7
B_2	$CH_2CH(CH_3)_2$	CH_3	$C_{89}H_{102}N_{10}O_{28}Cl_2 \cdot 1.6\ HCl$	1830.7

*Anhydrous free base

4.1.1 Formulation, Packaging, and Labeling

Dalbavancin

Dalbavancin is supplied in clear glass vials as a sterile, lyophilized, preservative-free, white to off-white to pale yellow solid. Each vial contains dalbavancin HCl equivalent to 500 mg of dalbavancin.

Investigational dalbavancin vials will be labeled according to manufacturer or regulatory specifications and include the statement "*Caution: New Drug – Limited by Federal Law to Investigational Use*." The dispensed study product (IV bags) will be labeled with the cautionary statement "For Investigational Use Only."

The standard of care (SOC) antibiotics (Cefazolin, nafcillin, oxacillin, vancomycin, and daptomycin) will be prepared and labeled in accordance with the clinical site pharmacy's standard operating procedures (SOPs).

4.1.2 Product Storage and Stability

Dalbavancin

Unreconstituted dalbavancin for injection should be stored at 25°C (77°F); excursions permitted to 15°C to 30°C (59°F to 86°F) [see USP Controlled Room Temperature].

Reconstituted vials may be stored either refrigerated at 2°C to 8°C (36°F to 46°F), or at controlled room temperature 20°C to 25°C (68°F to 77°F). Do not freeze.

Once diluted into an intravenous bag , dalbavancin may be stored either refrigerated at 2°C to 8°C (36°F to 46°F), or at controlled room temperature 20°C to 25°C (68°F to 77°F). Do not freeze.

The total time from reconstitution to dilution to administration should not exceed 48 hours.

SOC Antibiotics: Cefazolin, nafcillin, oxacillin, vancomycin, and daptomycin

Store per manufacturer's instructions.

4.2 Acquisition/Distribution

Dalbavancin for injection will be supplied by Allergan, a subsidiary of AbbVie and will be obtained from the DMID Clinical Materials Services (CMS), Fisher BioServices, upon request by the study sites and approval by DMID.

SOC antibiotics (cefazolin, nafcillin, oxacillin, vancomycin, and daptomycin) specified for use in the study will be obtained locally by each participating site.

Any diluents or other vehicles for preparation of dalbavancin and SOC antibiotics for use in the study will also be obtained locally by each participating site.

4.3 Dosage/Regimen, Preparation, Dispensing and Administration of Study Intervention/Investigational Product

Dalbavancin Group

For subjects randomized to the dalbavancin treatment group, the dosage of dalbavancin administered will be determined based on individual estimated serum creatinine clearance (CrCl) levels and presence of regular hemodialysis or peritoneal dialysis as follows:

Subjects with CrCl ≥ 30 mL/min and subjects receiving regular hemodialysis or peritoneal dialysis will receive 1500 mg IV dalbavancin over 30 (± 10) minutes on Day 1 and on Day 8.

Subjects with CrCl < 30 mL/min who are not receiving regular hemodialysis or peritoneal dialysis will receive 1125 mg IV dalbavancin over 30 (± 10) minutes on Day 1 and on Day 8.

A repeat serum creatinine will be required within the 72 hours prior to the second (Day 8) dalbavancin dose. Whether creatinine clearance needs to be reassessed on Day 8 will be left to the clinical judgment of the study investigator. For example, a subject with stable renal function up to 72 hours before the second dalbavancin dose and no anticipated change to renal function may not require a repeat creatinine clearance measurement on Day 8. In contrast, if the subject had either fluctuating renal function or renal function near the threshold for dose adjustment, it would be entirely appropriate to repeat a creatinine measurement prior to the Day 8 dose.

If CrCl crosses the 30ml/min threshold in either direction after Day 1 but before the Day 8 dose, then the dose should be adjusted accordingly. That is, if a subject has CrCl ≥ 30 mL/min on Day 1 and received 1500mg IV dalbavancin, but the CrCl is < 30 mL/min on day 8then that subject will receive 1125mg IV for the Day 8 dose. If the CrCl improves to ≥ 30 mL/min between Day 1 and Day 8, then the dose will receive 1500mg IV dalbavancin for the Day 8 dose.

Standard of Care Group

Subjects randomized to the standard of care antibiotic therapy treatment group will receive an antibiotic considered standard of care, generally for a duration of 4 to 6 weeks (but up to a maximum of 8 weeks, which may be standard practice for vertebral osteomyelitis at some sites) and based on the results of antibiotic susceptibility testing for baseline pathogen. The site PI (or licensed sub-investigator listed on the Form FDA 1572), in agreement with the patient’s treating clinician, will designate which antibiotic is considered the study drug for patients randomized to the standard of care group; this will generally be the antibiotic already being used to treat their infection. For example, a patient randomized to the standard of care group who is receiving vancomycin for treatment of bacteremia, would most likely continue to be treated with vancomycin and this would be designated as their study drug. If the treating clinician is changing the antibiotic regimen at the time of randomization, for example to an antibiotic that would be easier to administer in the outpatient setting, then that new antibiotic would be designated as the study drug.

Any changes to study drug (discontinuation or dose adjustment) will be captured and the reason for this change will be recorded, with the exception that vancomycin dose adjustments do not need to be captured.

For subjects randomized to standard of care antibiotic therapy, the counting of days for the duration of therapy may begin on the first day on which blood cultures are negative during the Induction Period.

Subjects randomized to the standard of care antibiotic therapy treatment group will receive the following antibiotic(s) shown in Table 5 for the specified duration based on baseline pathogen:

Table 5: Acceptable SOC Antibiotics

Baseline Pathogen	Standard of Care Therapy (doses may be adjusted for renal function)[b]
MSSA[a]	nafcillin (2 g IV q4h × 4-6 weeks[c])[e]<br>OR<br>oxacillin (2 g IV Q4h x 4-6 weeks[c])[e]<br>OR<br>cefazolin (2 g IV q8h × 4-6 weeks[c])[e]
MRSA	vancomycin (dose per local standard of care × 4-6 weeks[c])[d] OR<br>daptomycin (6-10 mg/kg IV daily × 4-6 weeks[c])

IV = intravenous; qxh = every x hours; MRSA = methicillin--resistant *Staphylococcus aureus*; MSSA = methicillin--susceptible *Staphylococcus aureus*

[a] Vancomycin or daptomycin are also appropriate for patients with MSSA and anaphylactoid-type hypersensitivity to beta-lactams.

[b] If there are extenuating circumstances in which preferred standard of care antibiotics cannot be used, for example complex allergy history, then an alternative antibiotic may be used after discussion with the protocol PIs and DMID Medical Officer.

[c] Duration of antibiotics reflects usual standard of care for complicated SAB. It may be extended to a maximum of 8 weeks at the discretion of the treating clinician.

[d] Patients on vancomycin will have dose adjustment and monitoring based on local standard of care

[e] As applicable per site standard of care, beta-lactams may be administered at an equivalent dose via continuous IV infusion (e.g., nafcillin 12g/24h IV continuous).

Complete instructions for dosage, preparation, labeling, storage, stability, and administration for dalbavancin and SOC antibiotics are provided in the protocol-specific Manual of Procedures (MOP).

Regarding vancomycin dosing, dose adjustments and monitoring will be performed in accordance with local standard of care. Vancomycin monitoring may be performed by trough levels, area under the curve (AUC) based protocols, or other methods guided by sites' local standards. Because vancomycin dosing and monitoring is determined by these local standards of care, we will not require specific doses or levels to be recorded.

4.4 Pre-determined Modification of Study Intervention/Investigational Product for an Individual Subject

Dalbavancin dose will be modified per package insert for renal function only. Any contraindication to receipt of the second dose, including an allergic reaction, pregnancy or other adverse event (see individual halting rules in Section 8.5.2), should be discussed with the DMID Medical Officer.

Subjects with impaired renal function will have their dosage of standard of care antibiotic treatment adjusted as needed, based on local standard of care.

Any alteration in study drug therapy because of unusual clinical circumstances must be discussed with the DMID Medical Officer.

4.5 Accountability Procedures for the Study Intervention/Investigational Product(s)

Dalbavancin will be stored and shipped from the DMID contract Clinical Material Services (CMS) to the Clinical Sites. Once received, dalbavancin will be stored in and dispensed by the Investigational Pharmacy.

The Food and Drug Administration (FDA) requires accounting for the disposition of all investigational products. The Investigator is responsible for ensuring that a current record of product disposition is maintained and product is dispensed only at an official study site by authorized personnel as required by applicable regulations and guidelines. Records of product disposition, as required by federal law, consist of the date received, date administered, quantity administered, and the subject number to whom the drug was administered.

The Investigational Pharmacist will be responsible for maintaining accurate records of the shipment and dispensing of the investigational product. The pharmacy records must be available for inspection by the DMID monitoring contractors, and is subject to inspection by a regulatory agency (e.g., FDA) at any time. An assigned Study Monitor will review the pharmacy records.

Unused unreconstituted investigational product (dalbavancin) vials will be stored at 25ºC (77ºF); excursions permitted to 15ºC to 30ºC (59ºF to 86ºF) [see USP controlled room temperature] in the Investigational Pharmacy until clinical trial accountability is completed. At study termination, all unused investigational product will be disposed in accordance with the MOP following complete drug accountability and monitoring.

5 SELECTION OF SUBJECTS AND STUDY ENROLLMENT AND WITHDRAWAL

Subject Inclusion and Exclusion Criteria must be confirmed by a study investigator listed on the Form FDA 1572.

No exemptions are granted on Subject Inclusion/Exclusion Criteria in DMID-sponsored studies. Clarifications regarding applicability of specific inclusion and exclusion criteria may be discussed with a protocol clinician. Questions about eligibility will also be directed toward the DMID Medical Officer.

5.1 Eligibility Criteria

5.1.1 Subject Inclusion Criteria

A subject must meet all of the following criteria to be considered eligible for inclusion in the study:

1. Written informed consent obtained from the patient or legally authorized representative before the initiation of any study-specific procedures.
2. Patients ≥ 18 years old.
3. A diagnosis of complicated *S. aureus* (either MSSA or MRSA) bloodstream infection (see definition of uncomplicated bacteremia in Exclusion criterion #1).
4. Treated with effective antibiotic therapy for at least 72 hours (maximum 10 days)[3].
5. Subsequent defervescence for at least 24 hours and clearance of bacteremia from the qualifying pathogen (at Screening), with negative blood culture incubated for at least 48 hours.[4]
6. Provider willing to treat with either dalbavancin for two doses, or standard of care intravenous monotherapy for at least 4 and *no more than* 8 weeks from randomization.

[3] Ten consecutive days prior to randomization is the maximum allowed treatment duration. If a subject has received intermittent or incomplete therapy earlier in the treatment course for this episode of *S. aureus* bacteremia, then discuss with the protocol PI and DMID Medical Officer prior to enrollment.
[4] Two negative blood cultures incubated for 48 hours are preferred. However, if only a single blood culture set is drawn, no growth at 48 hours will be considered adequate to demonstrate clearance. If more than one culture set is drawn, *all* must show no growth at 48 hours to be considered evidence of clearance (e.g., 1 of 2 positive cultures would still be considered as ongoing bacteremia).

7. Patients must be willing and able, if discharged, to return to the hospital or designated clinic for scheduled treatment, laboratory tests, or other procedures as required by the protocol.
8. According to the site PI or sub-investigator assessment, patients must be expected to survive with appropriate antibiotic therapy and appropriate supportive care throughout the study.

5.1.2 Subject Exclusion Criteria

1. Uncomplicated bacteremia[5] (Appendix B).
2. Infectious CNS events, including septic emboli, ischemic or hemorrhagic stroke, epidural abscess, or meningitis (prior/unrelated CNS events are **not** exclusion criteria).
3. Known or suspected left-sided endocarditis or presence of a perivalvular abscess.
4. Planned right-sided valve replacement surgery in the first 3 days following randomization.
5. Presence of prosthetic heart valve, cardiac device[6] UNLESS removal is planned within 4 days post-randomization.
6. Presence of intravascular graft or intravascular material[7] UNLESS removal is planned within 4 days post-randomization.
7. Infected prosthetic joint or extravascular hardware UNLESS removal is planned within 4 days post-randomization OR hardware was placed >60 days before bacteremia and clinically appears uninfected.
8. Polymicrobial bacteremia unless the non-*S. aureus* organism is a contaminant [see Definitions (Appendix B)].[8]

---

[5] Uncomplicated *Staphylococcus aureus* bacteremia is defined as all of the following: exclusion of endocarditis by echocardiography; catheter-associated bacteremia and removal of catheter; no implanted prostheses; follow-up blood cultures drawn within 48 hours after initial set that do not grow screening pathogen and all follow-up blood cultures thereafter do not grow the screening pathogen; defervescence within 72 hours of initiating effective therapy; and no evidence of metastatic sites of infection. Any patient not meeting these strict criteria is considered to have *complicated* bacteremia and is eligible.

[6] Implantable cardioverter defibrillator (ICD), permanent pacemaker, valve support ring, ventricular assist device (VAD)

[7] Excluding cardiac stents, inferior vena cava filters in place for >6 weeks, vascular stents in place for >6 weeks, non-hemodialysis grafts in place >90 days, and hemodialysis grafts not used within the past 12 months and not previously infected. A fistula constructed from native veins or a biologic vascular graft (without synthetic graft material) does not count as intravascular graft/material.

[8] *Note:* If a gram-negative bacteremia or fungemia develops after the qualifying *S. aureus* blood culture, AND the patient does not have right-sided endocarditis, AND the infection can be treated with an antibiotic without efficacy against the patient's *S. aureus* isolate (e.g. aztreonam), then the patient may remain eligible. Discussion with the DMID Medical Officer is strongly encouraged.

9. Significant hepatic insufficiency (Child-Pugh class C or AST/ALT values >5x ULN at the time of randomization).
10. Immunosuppression.[9]
11. History of hypersensitivity reaction to dalbavancin or other drugs of the glycopeptide class of antibiotics.
12. Treatment with either dalbavancin or oritavancin in the 60 days prior to enrollment.
13. Infection with *S. aureus* not susceptible to dalbavancin (dalbavancin mean inhibitory concentration [MIC] > 0.25 µg/mL) or vancomycin (vancomycin MIC > 2 µg/mL).
14. Planned treatment with concomitant systemic antibacterial therapy with potential efficacy against the patient's qualifying *S. aureus* isolate, other than that allowed in the protocol.
15. Pregnant/ nursing females.
16. Females of childbearing potential must have a negative pregnancy test[10] within 48h of randomization and use effective contraception for trial duration (Appendix B).
17. Other medical or psychiatric condition that may, in the judgment of the investigator, increase the risk of study participation or interfere with interpretation of study results.
18. Unwilling or unable to follow study procedures.
19. Treatment with an investigational drug within 30 days preceding the first dose of study medication.

5.2 Withdrawal from the Study, Discontinuation of Study Product, or Study Termination

5.2.1 Withdrawal from the Study or Discontinuation of the Study Product

Subjects may voluntarily withdraw their consent for study participation at any time without penalty or loss of benefits to which they are otherwise entitled.

An investigator may also discontinue a subject from receiving the study product (i.e., dalbavancin or SOC antibiotic) for any reason. Follow-up safety evaluations for discontinued subjects will be

[9] On chemotherapy or immunotherapy for active hematologic malignancy expected to cause > 7 days of ANC < 100 cells/mm^3, recent bone marrow transplant (in the past 90 days), solid organ transplantation within prior 3 months or receipt of augmented immunosuppression for rejection within 3 months, chronic granulomatous disease, HIV infection with a CD4 cell count < 50 cells/mm^3 based on last known measurement or patient-reported value.

[10] If the serum pregnancy test results cannot be obtained before randomization, a urine pregnancy test may be used for enrollment.

conducted, if the subject agrees. If a subject withdraws or is withdrawn prior to completion of the study, the reason for this decision must be recorded in the case report forms (CRFs).

The reasons to withdraw from the study might include, but are not limited to, the following:

- Subject no longer meets eligibility criteria
- Subject withdraws consent
- Subject lost to follow-up
- Subject becomes non-compliant
- Medical disease or condition, or new clinical finding(s) for which continued participation, in the opinion of the investigator might compromise the safety of the subject, interfere with the subject's successful completion of this study, or interfere with the evaluation of responses
- Study or site prematurely terminated by the sponsor for any reason

The reasons to discontinue study product (i.e., dalbavancin or SOC antibiotic) might include, but are not limited to, the following:

- Subject meets individual halting criteria (see Section 8.5.2)
- Subject becomes pregnant, if applicable
- Occurrence of an AE that, in the opinion of the investigator, warrants the subject's permanent discontinuation from IV study drug (i.e., dalbavancin or SOC antibiotic)
- Subject has an insufficient therapeutic response to study drug (i.e., lack of efficacy for SAB). A patient who does not show signs of improvement despite treatment with study drug for an appropriate length of time or a patient who shows signs of clinical worsening at any time may be prematurely discontinued from study drug therapy and treated with salvage therapy as directed by their treating clinician. These patients would still continue to be followed in the study unless withdrawn for another reason.

A subject who is prematurely discontinued from study drug or withdrawn from the study should have the assessments for Early Termination (ET) as detailed in the Schedule of Events (Appendix A). A clear description of reason for early withdrawal or discontinuation from study product (i.e., dalbavancin or SOC antibiotic) must be documented. The reasons for early withdrawal or premature discontinuation from study drug will be reflected on the relevant disposition page of the electronic case report form (eCRF).

The investigator should be explicit regarding study follow-up (e.g. safety and efficacy follow-up) that might be carried out despite the fact the subject will not receive further study product. If the

subject consents, every attempt will be made to follow all AEs through resolution or through the end of the study, whichever occurs first. The procedures that collect safety data for the purposes of research must be inclusive in the original informed consent.

The investigator will inform the subject that already collected data will be retained and analyzed even if the subject withdraws from this study.

5.2.2 Subject Replacement

Subjects who withdraw, or are withdrawn from this study, or are lost to follow-up after signing the informed consent form (ICF) and administration of the study product will not be replaced. Subjects who withdraw, or are withdrawn from this study, or are lost to follow-up after randomization but before administration of the study product will not be replaced.

5.2.3 Study Termination

The sponsor reserves the right to terminate the study in its entirety or at a specific study center before study completion.

If the study is prematurely terminated by the sponsor, any regulatory authority, or the investigator for any reason, the investigator will promptly inform the currently enrolled study subjects and assure appropriate therapy or follow-up for the subjects, as necessary. The investigator will provide a detailed written explanation of the termination to the IRB/IEC.

6 STUDY PROCEDURES

The study schedule is outlined below and in the Schedule of Events (Appendix A). The descriptions of the procedures to be performed at each visit are provided below.

6.1 Induction Period: Visit 0 (Pre-screening, Day -10 to Day 1)

During the pre-screening period (Visit 0), chart review may be performed to determine if a patient is potentially eligible for the study. No study-related procedures will be requested or performed prior to obtaining informed consent:

- Confirm the subject is potentially eligible for the study based on chart review of inclusion/exclusion criteria

6.2 Screening: Visit 1 (Day -1 to Day 1)

- Obtain informed consent
- Obtain medical and surgical history, to include targeted/pertinent medical and surgical history only
- Collect medication history (from 30 days prior to ICF signing; extended review specifically for dalbavancin or oritavancin receipt in the prior 60 days given long half-lives of these two drugs)
- Review and record relevant medical events that occurred during the induction period. Examples include source control procedures [see Definitions (Appendix B)], vascular access procedures [see Definitions (Appendix B)], and complications of pre-randomization antibiotic therapy.
- Collect blood samples for hematology, serum chemistry laboratory assessments, and coagulation lab tests (PT/PTT and /or INR), if not already complete per standard of care within 48 hours prior to randomization (see Section 7.2.1)
- Record most recent vital signs (blood pressure, respiration rate, pulse rate, and temperature); these must be performed within 48 hours prior to randomization
- Perform physical examination (see Section 7.1.1)
- Perform pregnancy test for women of childbearing potential (see definition in Appendix B) within 48 hours of randomization; ensure test is negative before randomization. If the serum test results cannot be obtained before randomization, a urine pregnancy test may be used for enrollment.

- Collect results of echocardiography to evaluate for endocarditis; perform an echocardiogram if it has not already been done as part of standard of care for this episode of bacteremia/endocarditis. Either a transthoracic or transesophageal echocardiogram is acceptable.
- Review concomitant medications
- Review and record any concomitant nondrug interventions (e.g., procedures performed with a goal of source control).

6.3 Open-label Treatment Period, Planned Study Visits

6.3.1 Baseline (Randomization): Visit 2 (Day 1)

After a subject signs the ICF at the Screening Visit, any additional evaluations required to confirm eligibility will be performed (see Section 6.2). Subjects that do not meet all eligibility criteria after signing informed consent, for example due to an abnormal laboratory result, will be considered screen failures. Subject eligibility should be confirmed (or reconfirmed) within a maximum of 24 hours prior to randomization.

Once eligibility is confirmed, the subject may be randomized. Enrollment of subjects will be done online using the enrollment module of Advantage eClinical. Subjects will be randomized to receive either a 2-dose regimen of dalbavancin, or to completion of 4 to 6 weeks (maximum of 8) of standard of care antibiotic therapy in a 1:1 allocation ratio based on the randomization schedule further described in Section 10.3.1.

The Baseline Visit (Visit 2) will be conducted within a maximum of 10 days of effective antibiotic therapy for the qualifying bloodstream infection, after having confirmed defervescence for at least 24 hours and clearance of bacteremia from the qualifying pathogen, with negative blood culture incubated for at least 48 hours. Study procedures will be reviewed with the patient and the caregiver, if applicable.

At Baseline (Visit 2), the following procedures will be performed:

- Review medical and surgical history.
- Review concomitant medications (medications post first dose of study product).
- Collect QoL data.
- Randomization.
- Measure vital signs (blood pressure, respiration rate, pulse rate, and temperature).
- Perform targeted physical examination, focused on changes from screening.

- Administer IV dalbavancin or standard of care antibiotic therapy according to randomization as outlined in Section 4.3. The provider should designate which antibiotic is assigned as the study drug for patients randomized to standard of care group.
- Document study drug administration.
- For subjects receiving dalbavancin, collect blood samples for pharmacokinetic parameters prior to first dose, at end of infusion (+10 minutes), 6 (+/- 2) hours post end of dose, 12 hours (+/- 4) hours post end of dose, 24 (+/- 6) hours post end of dose, with documented draw time and date for each sample.
- Review and record AEs/SAEs/AESIs after study product (i.e., dalbavancin or SOC antibiotic) administration.
- Review and record any concomitant nondrug interventions (e.g., procedures performed with a goal of source control).

6.3.2 Visit 3 (Day 8 ± 1 day)

At Visit 3 the following procedures will be performed:

- Review and record AEs/SAEs/AESIs.
- Measure vital signs and perform targeted physical exam focused on changes from baseline, for patients who remain in the hospital or are receiving care at a location where research personnel can provide an in-person assessment (e.g. at an infusion center). Patients in the standard of care group who have been discharged from the hospital may have their symptoms assessed by telephone. If assessed by telephone, then all assessments for this visit will be done by phone and absence of vital signs/physical exam will not be a protocol deviation.
- For subjects receiving dalbavancin, collect blood samples for pharmacokinetic parameters prior to 2nd dose of dalbavancin on day 8 (+/- 1 day) with documented draw time and date.
- For subjects receiving dalbavancin, a serum creatinine assessment is required within 72 hours prior to the administration of the second dalbavancin dose. Whether a serum creatinine must be repeated on Day 8 will be at the discretion of the site investigator based upon stability of the serum creatinine in the preceding 72 hours and whether the serum creatinine is near the threshold where dose adjustment would be necessary (e.g., near 30 mL/min).
- Review concomitant medications.
- Review and record any concomitant nondrug interventions (e.g. procedures performed with a goal of source control).

- Document study drug administration.
- Collect QoL data.

6.3.3 Visit 4 (Day 22 ± 2 days)

At Visit 4 the following procedures will be performed:

- Review and record AEs/SAEs/AESIs.
- Measure vital signs (blood pressure, respiration rate, pulse rate, and temperature) and perform targeted physical examination, focused on changes from prior examinations.
- Collect blood samples for hematology and serum chemistry.
- For subjects who received dalbavancin, collect blood samples for pharmacokinetic parameters if feasible on day 22 (+/- 2 days) with documented time of draw and date.
- Review concomitant medications.
- Review and record any concomitant nondrug interventions (e.g. procedures performed with a goal of source control).
- Administer IV standard of care antibiotic therapy according to randomization as outlined in Section 4.3 and document compliance.
- Collect QoL data.

6.3.4 Visit 5 (Day 42 ± 3 days)

At Visit 5, the following procedures will be performed:

- Review and record AEs/SAEs/AESIs.
- Update medical history.
- Measure vital signs (blood pressure, respiration rate, pulse rate, and temperature) and perform targeted physical examination, focused on changes from prior examinations.
- Collect blood samples for hematology and serum chemistry.
- For subjects who received dalbavancin, collect blood samples for pharmacokinetic parameters if feasible on day 42 (+/- 3 days) with documented time of draw and date.
- Review concomitant medications.

- Review and record any concomitant nondrug interventions (e.g., procedures performed with a goal of source control).
- Administer IV standard of care antibiotic therapy according to randomization as outlined in Section 4.3 and document, if ongoing.
- Complete investigator assessment of efficacy.
- Collect QoL data.

6.3.5 Test of Cure: Visit 6 (Day 70 ± 7 days)

It is preferred that this visit be performed in-person, however a telephone visit is an acceptable alternative. If performed by telephone, then the subject should be asked about new symptoms (e.g. fever, pain) in lieu of a physical exam.

At Visit 6 (Day 70), the following procedures will be performed:

- Review and record AEs/SAEs/AESIs.
- Update medical history.
- Measure vital signs (blood pressure, respiration rate, pulse rate, and temperature) for patients with in-person visit.
- Perform targeted physical examination, focused on changes from prior examinations.
- For subjects who received dalbavancin, collect blood samples for pharmacokinetic parameters if feasible on day 70 (± 7 days) with documented time of draw and date.
- Review concomitant medications.
- Review and record any concomitant nondrug interventions (e.g., procedures performed with a goal of source control).
- Complete investigator assessment of efficacy.
- Collect QoL data.

6.3.6 Visit 7 (Day 180 ± 14 days – only for subjects with osteomyelitis)

Visit 7 will only be performed for subjects with osteomyelitis present at baseline assessment in order to determine long-term recurrence risk. As with visit 6, an in-person visit will be preferred however a telephone alternative will also be considered acceptable. If performed by telephone, the subject should be asked about specific symptoms (fever, back pain), resumption/continuation/repeat treatment with antibiotics for *S. aureus* osteomyelitis, or surgical intervention for osteomyelitis.

At Visit 7 (Day 180), the following procedures will be performed:

- Measure vital signs (blood pressure, respiration rate, pulse rate, and temperature) for patients with in-person visit.
- Perform targeted physical examination, focused on changes from prior examinations (especially point tenderness to spine or presence of neurologic deficits).
- Complete investigator assessment of efficacy.
- Collect QoL data.
- Review concomitant medications and concomitant nondrug interventions that are related to the treatment of osteomyelitis.

6.3.7 Final Study Visit

- For subjects without osteomyelitis, the final study visit will be Visit 6 (Day 70 ± 7 days) as above. For subjects with osteomyelitis, the final study visit will be Visit 7 (Day 180 ± 14 days) as above.

6.3.8 Early Termination Visit

In circumstances where a subject withdraws from the study or discontinues the study product (i.e., dalbavancin or SOC antibiotic) early, an Early Termination Visit will be performed, which include the following procedures:

- Review and record AEs/SAEs/AESIs.
- Update medical history.
- Measure vital signs (blood pressure, respiration rate, pulse rate, and temperature) and perform targeted physical examination, focused on changes from prior examinations.
- If clinically indicated, blood cultures should be collected at time of treatment discontinuation or for determination of treatment failure.
- Review concomitant medications.
- Review and record any concomitant nondrug interventions (e.g., procedures performed with a goal of source control).
- Complete investigator assessment of efficacy.
- For subjects who received dalbavancin, collect blood samples for pharmacokinetic parameters if feasible with documented time of draw and date.

- Collect QoL data.

6.4 Unscheduled Study Visits

An unscheduled study visit may be initiated by the subject/subject's LAR or investigator, if a subject is not improving on therapy, has a grade 3 and above AE or an SAE, or for any other reason. In addition, the reason for the visit will be documented, including who initiated the visit, what complaints the subject/subject's LAR has, and/or what concerns the primary medical team or site investigator have. Clinical outcome and any safety assessments will be documented.

Assessments to be completed at unscheduled study visits should include:

- Review and record AEs/SAEs/AESIs.
- Update medical history.
- Measure vital signs (blood pressure, respiration rate, pulse rate, and temperature) and perform targeted physical examination, focused on changes from prior examinations.
- Review concomitant medications.
- Review and record any concomitant nondrug interventions (e.g., procedures performed with a goal of source control).

6.5 Protocol Deviations

A protocol deviation is any noncompliance with the clinical trial protocol, GCP, or protocol-specific Manual of Procedures (MOP) requirements. The noncompliance may be either on the part of the subject, the investigator, or the study site staff. As a result of deviations, corrective actions should be developed by the site and implemented promptly. It is the responsibility of the site Principal Investigator and other study personnel to use continuous vigilance to identify and report protocol deviations. All individual protocol deviations will be addressed in subject study records. All protocol deviations, either individual, product, or site-specific will be collected and the record stored in a sponsor-determined location. Protocol deviations must be sent to the IRB/IEC on which it relies per its guidelines as well as to the sites' local IRB/IEC if required. The site Principal Investigator and other study personnel are responsible for knowing and adhering to the requirements of their local IRB/IEC as well as the requirements of the IRB/IEC on which it relies.

Inability to draw pharmacokinetic samples will *not* be considered a violation of protocol, as pharmacokinetic sampling is being conducted for exploratory purposes only and does not alter in any way the planned treatment course for subjects.

7 DESCRIPTION OF CLINICAL AND LABORATORY EVALUATIONS

7.1 Clinical Evaluations

7.1.1 Research Procedures

- Medical and Surgical History: includes targeted/pertinent medical and surgical history only.
- Medication History: from 30 days prior to signing the ICF, and 60 days for any prior lipoglycopeptides (oritavancin or dalbavancin) as reported by patient.[11]
- Vital Signs: Vital sign measurements will be documented at every in-person visit. The parameters are blood pressure (BP), respiration rate, pulse rate, and temperature. It is recommended that heart rate and BP readings be taken after the patient has been sitting for at least 5 minutes.
- Physical Examination: A physical examination (including general appearance, examination of head, eyes, ears, nose, throat, neck, skin, heart, lungs, abdomen, neurologic system, musculoskeletal system, extremities, height, and body weight) will be done at Screening (Visit 1). Physical examinations should be performed by a professionally trained physician or health professional licensed to perform physical examinations. Body weight and height will be measured at Screening (Visit 1). If height or weight is not obtainable (e.g., patient is immobilized), the last known or stated height and weight may be used. At subsequent visits, a targeted physical exam should focus on changes from prior exams and on the evaluation of newly reported symptoms.
- Echocardiograms: A transthoracic echocardiogram will be performed, or if clinically indicated, a transesophageal echocardiogram will be performed, unless one has been performed as standard of care for this episode of bacteremia/endocarditis. The overall interpretation and determination of the clinical significance of echocardiography findings will be the responsibility of the investigator, and the findings of all echocardiograms will be recorded in the patient's eCRF.
- Quality of Life Instruments: The following three QoL measures will be administered:

[11] As patients may receive medication prescriptions from multiple sources, patient-reported medications are intended to serve as the primary source for concomitant medications. For sites that have the ability to cross-query reported medications against prescription or medication administration records, this can be used as a secondary source but is not required.

- The ARLG Bloodstream Infection QoL Measure (Appendix C) is a 41-item questionnaire covering multiple domains of QoL.
- The EQ-5D-5L (https://euroqol.org/eq-5d-instruments/sample-demo/) is an instrument that comprises five dimensions: mobility, self-care, usual activities, pain/discomfort, and anxiety/depression, as well as an overall self-rated health on a vertical visual analogue scale.
- The PROMIS Global Health short form (http://www.healthmeasures.net/administrator/components/com_instruments/uploads/Global%20Health%20Scale%20v1.2%2008.22.2016.pdf) is a 10-item instrument that assesses general health and five primary domains (physical function, fatigue, pain, emotional distress, and social health).

7.1.2 Assessment of Concomitant Medications/Treatments Other Than Study Product

Patient-reported medication history during the 30 days prior to ICF signing will be recorded at Screening (Visit 1) in the eCRF.[12] Thereafter, any changes in concomitant medications or new medications added will be recorded in the eCRF.

While most systemic medications taken by the subject, other than study drug, are considered concomitant medications and should be recorded, the following specific exceptions do *not* need to be recorded:

1. Topical medications (eye drops, ear drops, intranasal drops or sprays, dermatologic treatments, topical lidocaine)
2. Vitamins and supplements (e.g., vitamins, minerals, herbal supplements, dietary supplements, iron/ferrous sulfate, magnesium, calcium, electrolyte replacements)
3. Symptomatic care medications (e.g., antipyretics such as ibuprofen or acetaminophen, antihistamines, decongestants, non-steroidal anti-inflammatory drugs [NSAIDs])
4. Gastrointestinal agents (prokinetic agents, laxatives, stool softeners, gastrointestinal stimulants, antacids)
5. Nicotine replacement products (e.g., patches, lozenges, gums, nasal sprays)
6. Heparin flushes (e.g., as used for vascular access flushes)

All relevant concomitant systemic medications from Screening (Visit 1) through Day 70 ± 7 days (Visit 6) must be recorded in the subject's medical record and on the eCRFs. Specific dosages or

[12] As patients may receive medication prescriptions from multiple sources, patient-reported medications are intended to serve as the primary source for concomitant medications. For sites that have the ability to cross-query reported medications against prescription or medication administration records, this can be used as a secondary source but is not required.

dose changes for concomitant medications do not need to be recorded in the eCRFs. After Visit 6, for subjects with osteomyelitis, who are followed until Day 180 ± 14 days (Visit 7), the only concomitant medications that must be recorded are new antibiotics that are prescribed for the treatment of osteomyelitis. At each visit the investigator will obtain information on any therapeutic interventions (e.g., drug and nondrug therapy or surgery) provided. Subjects may not participate in any other antibiotic treatment trials or interventional studies involving non-FDA approved investigational products concomitantly while in this study.

Concomitant systemic antibacterials (other than dalbavancin or comparator study drug) for adjunctive therapy of the subject's *S. aureus* bacteremia are prohibited during the study, up to Day 70. This includes concomitant treatment with an aminoglycoside.

Patients who require additional therapy due to inadequate clinical response will be assessed as having lack of efficacy of study drug.

- Where possible, antibiotic treatment of intercurrent infections should be done with antibiotics that are not active against the patient's *S. aureus* isolate. Discussion with the DMID Medical Officer is encouraged before or within 24 hours of initiation of concomitant antibiotics for another indication. Exceptions include: Vancomycin oral 125 mg up to 500mg every 6 hours may be used in both treatment groups for the treatment of *Clostridium difficile* infections and may be continued as required throughout the duration of the study. The sponsor will not provide oral vancomycin.
- Metronidazole IV or oral 500 mg every 8 hours may be used in both treatment groups for the treatment of *C. difficile* infections and may be continued as required throughout the duration of the study. The sponsor will not provide metronidazole.
- Other antibacterials that do not achieve therapeutic levels in the serum (e.g., nitrofurantoin) may be considered. Consultation with the DMID Medical Officer is advised before use of these antibiotics.

7.1.3 Assessment of Subject Compliance with Study Intervention/Investigational Product/Investigational Device

Dalbavancin will be administered under the supervision of investigative site personnel, and infusion date, start, and stop time will be documented in the eCRF, as well as any infusion interruptions.

7.1.4 Non-Research Standard of Care

If clinically indicated, any surgical or non-surgical procedures required to achieve infection source control should be performed for patients enrolled in either treatment group. The necessity and timing of any such procedures will be decided upon by the patient's treating physicians. Procedures frequently required for source control in *Staphylococcus aureus* bacteremia may include

debridement of osteomyelitis, removal of indwelling vascular access device, prosthetic devices or materials, incision and drainage of any abscesses, or heart valve replacement.

7.2 Laboratory Evaluations

7.2.1 Clinical Laboratory Evaluations

The following laboratory evaluations will be evaluated during the study, per the schedule of events (Appendix A)

- Hematology: absolute and differential white blood cell (WBC) count, erythrocyte count, hemoglobin, hematocrit, and platelet count..
- Coagulation lab tests (PT/PTT and/or INR).
- Chemistry: sodium, potassium, calcium, chloride, bicarbonate, glucose, blood urea nitrogen, creatinine, total protein, alkaline phosphatase, albumin, total bilirubin, AST, and ALT.
- If clinically indicated, blood cultures should be collected at the time of treatment discontinuation or for determination of treatment failure. Blood cultures should be repeated every 24-48 hours upon knowledge of a positive result from any visit until clearance of bacteremia is confirmed. When blood cultures are required, 2 sets of blood samples (1 aerobic and 1 anaerobic bottle) should be obtained from 2 separate venipuncture sites.
- Culture, organism identification, and susceptibility testing will be conducted at the local laboratory. All pathogens will be tested for susceptibility; microbiological specimens and isolates will be collected, processed, and stored in accordance with local procedures.
- Pregnancy test (urine or serum).

7.2.2 Research Assays

Dalbavancin Concentration Measurements: Venous blood samples for PK analyses will be collected at Visit 2 (Day 1), Visit 3 (Day 8 ± 1 day),Visit 4 (Day 22 ± 2 days), Visit 5 (Day 42 ± 3 days), Visit 6 (Day 70 ± 7 days), or any early termination (ET) visit as outlined in Section 6 and Appendix A. The actual date and time of each blood sample collection will be recorded in the subjects' source document and the eCRF. Blood collected will be processed for plasma separation at the study site's laboratory; subsequent PK analyses will be performed on the plasma samples at a central laboratory. PK sample collection, labeling, processing, storage, and shipment instructions will be provided in the MOP.

7.2.2.1 Laboratory Specimen Preparation, Handling, and Storage

Plasma PK specimens will be processed and stored in a -70°C or below freezer until time of shipment to the central PK laboratory for analysis. Detailed instructions for the preparation, handling, and storage of plasma PK specimens are detailed in the study MOP including aliquots of specimens, temperature requirements, where they will be stored, and how they will be labeled.

7.2.2.2 Laboratory Specimen Shipping

Plasma PK samples will be shipped on dry ice to the central PK laboratory. Samples will be shipped in compliance with the International Air Transport Association (IATA) regulations. Instructions for the shipment of specimens are outlined in the study MOP.

8 ASSESSMENT OF SAFETY

8.1 Assessing and Recording Safety Parameters

This study will assist in determining the safety of dalbavancin compared to that of standard of care for the treatment of complicated *S. aureus* bacteremia. Safety will be assessed by the frequency of SAEs, AESIs, and the frequency of AEs leading to study drug discontinuation in each treatment arm.

8.1.1 Adverse Events (AEs)

ICH E6 defines an AE as any untoward medical occurrence in a patient or clinical investigation subject administered a pharmaceutical product regardless of its causal relationship to the study treatment. FDA defines an AE as any untoward medical occurrence associated with the use of a drug in humans, whether or not considered drug related.

An AE can therefore be any unfavorable and unintended sign (including an abnormal laboratory finding), symptom, or disease temporally associated with the use of medicinal product. The occurrence of an AE may come to the attention of study personnel during study visits and interviews of a study recipient presenting for medical care, or upon review by a study monitor.

All AEs of grade 3 or higher including lab abnormalities (per CTCAE table, version 5.0, https://ctep.cancer.gov/protocolDevelopment/electronic_applications/docs/CTCAE_v5_Quick_Reference_8.5x11.pdf) will be captured on the appropriate data collection form and eCRF. All AEs, regardless of grade, leading to study drug discontinuation will be captured on the appropriate data collection form and eCRF. AESIs of all grades will also be captured (see Section 8.2.1). Information to be collected for AEs includes event description, date of onset, assessment of severity, relationship to study product (i.e., dalbavancin or SOC antibiotic) and alternate etiology (assessed only by those with the training and authority to make a diagnosis and listed on the Form FDA 1572 as an investigator), date of resolution, seriousness and outcome. AEs and AESIs that meet reporting criteria and occur during the trial collection and reporting period will be documented appropriately regardless of relationship and will be followed through resolution or through the end of the study, whichever occurs first. Resolution of an AE is defined as the return to pre-treatment status or stabilization of the condition with the expectation that it will remain chronic.

For the purpose of the site's data collection responsibilities, any untoward event that was reported from the time of first study drug dose (i.e., dalbavancin or SOC antibiotic) after randomization at the Baseline Visit (Visit 2) until study Visit 6 is to be considered an AE.

Examples of AEs are as follows:

- Changes in the general condition of the patient from baseline

- Subjective symptoms offered by or elicited from the patient
- Objective signs observed by the investigator or other study center personnel
- All diseases that occur after administration of study drug at the Baseline Visit, including any change in severity or frequency of pre-existing disease
- All clinically significant abnormalities in laboratory values or clinically significant physical findings that occur during the study collection and reporting period. However, a single abnormal laboratory result does not automatically indicate progression of a chronic condition. For example, a transient spike in blood glucose level for a subject with diabetes is not reported as an AE.

Please note that hospital admissions and/or medical/surgical procedures scheduled prior to study drug administration but occurring during the study should not be captured as AEs, but should be listed in the medical history if related to a pre-existing condition.

Any medical condition that is present at the time of signing of ICF when the subject is screened will be considered as baseline and not reported as an AE. However, if the severity of any pre-existing medical condition increases, it should be recorded in source documents as an AE. In this trial, only Grade 3 and higher AEs, AEs of any grade leading study drug discontinuation, and AESIs will be captured in the eCRF.

Any abnormal test result that is determined to be an error does not require reporting as an AE. Additional diagnostic testing or medical/surgical interventions that occur as a result of an AE due to an abnormal laboratory test finding should be noted in the eCRF, if it meets protocol-specified reporting criteria.

8.1.1.1 Adverse Events Grading

All AEs (laboratory and clinical symptoms) will be graded for severity (per CTCAE table, version 5.0, https://ctep.cancer.gov/protocolDevelopment/electronic_applications/docs/CTCAE_v5_Quick_Reference_8.5x11.pdf) and assessed for relationship to study product (i.e., dalbavancin or SOC antibiotic) as outlined below. AEs characterized as intermittent require documentation of onset and duration of each episode. The start and stop date of each reported AE will be recorded on the appropriate data collection form and eCRF. Changes in the severity of an AE will be documented in the source documents at the site to allow an assessment of the duration of the event at each level of intensity.

Severity of Event:

AEs will be assessed by the investigator (those with the training and authority to make a diagnosis and listed on the Form FDA 1572) using the CTCAE, version 5.0

(https://ctep.cancer.gov/protocolDevelopment/electronic_applications/docs/CTCAE_v5_Quick_Reference_8.5x11.pdf). The investigator will provide an assessment of the severity of each AE by recording a severity rating on the appropriate source documentation. *Severity,* which is a description of the intensity of manifestation of the AE, is distinct from *seriousness,* which implies a patient outcome or AE-required treatment measure associated with a threat to life or functionality (Section 8.1.2). Severity will be assessed according to CTCAE, version 5.0 (https://ctep.cancer.gov/protocolDevelopment/electronic_applications/docs/CTCAE_v5_Quick_Reference_8.5x11.pdf), which follows the following general guideline:

- Grade 1 Mild; asymptomatic or mild symptoms; clinical or diagnostic observations only; intervention not indicated.
- Grade 2 Moderate; minimal, local or noninvasive intervention indicated; limiting age-appropriate instrumental activity of daily living (ADL).
- Grade 3 Severe or medically significant but not immediately life-threatening; hospitalization or prolongation of hospitalization indicated; disabling; limiting self-care ADL.
- Grade 4 Life-threatening consequences; urgent intervention indicated.
- Grade 5 Death related to AE.

Relationship to Study Product: The assessment of the AE's relationship to study product (i.e., dalbavancin or SOC antibiotic) will be done by a licensed study investigator indicated on the Form FDA 1572 and the assessment will be part of the documentation process. Whether the AE is related or not, is not a factor in determining what is or is not reported in this trial. If there is any doubt as to whether a clinical observation is an AE, the event should be reported.

In a clinical trial, the study product must always be suspect. The relationship to study product (i.e., dalbavancin or SOC antibiotic) will be assessed for AEs using the terms related or not related:

- Related – There is a reasonable possibility that the study product caused the AE. Reasonable possibility means that there is evidence to suggest a causal relationship between the study product and the AE.
- Not Related – There is not a reasonable possibility that the administration of the study product caused the event.

8.1.2 Serious Adverse Events (SAEs)

An AE or suspected adverse reaction is considered a serious adverse event (SAE) if, in the view of either the site principal investigator or sponsor, it results in any of the following outcomes:

- Death,

- A life-threatening adverse event[1],
- Inpatient hospitalization or prolongation of existing hospitalization,
- A persistent or significant incapacity or substantial disruption of the ability to conduct normal life functions, or
- A congenital anomaly/birth defect.
- Important medical events that may not result in death, be life-threatening, or require hospitalizations may be considered serious when, based upon appropriate medical judgment they may jeopardize the patient or subject and may require medical or surgical intervention to prevent one of the outcomes listed in this definition. Examples of such medical events include allergic bronchospasm requiring intensive treatment in an emergency room or at home, blood dyscrasias or convulsions that do not result in inpatient hospitalization, or the development of drug dependency or drug abuse.

[1] Life-threatening adverse event. An AE is considered "life-threatening" if, in the view of either the site principal investigator or sponsor, its occurrence places the patient or subject at immediate risk of death. It does not include an AE that, had it occurred in a more severe form, might have caused death.

SAEs will be:

- Assessed for severity and relationship to study product (i.e., dalbavancin or SOC antibiotic) and alternate etiology (if not related to study product) by a licensed study physician listed on the Form FDA 1572 or by the Institution as the site Principal Investigator or Sub-Investigator.
- Recorded on the appropriate SAE data collection form and eCRF.
- Followed through resolution by a licensed study physician (for IND studies, a physician listed on the Form FDA 1572 as the site Principal Investigator or Sub-Investigator).
- Reviewed and evaluated by DMID, the DSMB (periodic review unless related), and the IRB/IEC.

8.2 Specification of Safety Parameters

Safety will be assessed by the frequency and severity of AEs, AESIs, and SAEs.

Safety will be assessed using descriptive statistics of AEs, vital signs, and laboratory tests by treatment group. For each safety parameter, the last assessment made before the first dose of study product will be used as the baseline for all analyses of that safety parameter.

8.2.1 Adverse Events of Special Interest (AESIs)

Adverse events of special interest (AESIs) are AEs that are common and known to occur following administration of study product (i.e., dalbavancin or SOC antibiotic). AESIs that are related to study product (i.e. dalbavancin or SOC antibiotic) will be collected after first dose of study product (i.e., dalbavancin or SOC antibiotic) is given and until final study visit. AESIs will be recorded on the eCRF and entered into the EDC. AESIs will include any CTCAE grades of allergic reaction, catheter-related infection, vascular access complication, infusion site extravasation, infusion-related reaction, alanine aminotransferase increases, aspartate aminotransferase increases, and acute kidney injury.

If an intravascular catheter related complication is related to the catheter or vascular access device itself and not to study medication being administered (e.g., catheter-associated thrombosis or catheter-associated infection), this should still be recorded as an adverse event of special interest but can be marked as not related to study drug.

8.3 Reporting Procedures

8.3.1 Reporting Serious Adverse Events

SAEs will be followed until resolution even if this extends beyond the study-reporting period. Resolution of an AE is defined as the return to pretreatment status or stabilization of the condition with the expectation that it will remain chronic.

Any AE that meets a protocol-defined serious criterion that is judged to be <u>related</u> to either study product (i.e., dalbavancin or SOC antibiotic) must be submitted immediately (within 24 hours of site awareness) on an SAE form to the DMID Pharmacovigilance Group, at the following address:

DMID Pharmacovigilance Group

Clinical Research Operations and Management Support (CROMS)

6500 Rock Spring Dr. Suite 650

Bethesda, MD 20817, USA

SAE Hot Line: 1-800-537-9979 (US) or 1-301-897-1709 (outside US)

SAE FAX Number: 1-800-275-7619 (US) or 1-301-897-1710 (outside US)

SAE Email Address: PVG@dmidcroms.com

In addition to the SAE form, select SAE data fields must also be entered into the data coordinating center (DCC) database system, regardless of relationship to study product (i.e., dalbavancin or SOC antibiotic). Please see the protocol-specific MOP for details regarding this procedure.

Other supporting documentation of the event may be requested by the DMID Pharmacovigilance Group and should be provided as soon as possible.

The DMID Medical Monitor and DMID Clinical Project Manager will be notified of the related SAE by the DMID Pharmacovigilance Group. The DMID Medical Monitor will review and assess the SAE for regulatory reporting and potential impact on study subject safety and protocol conduct.

SAEs that that are judged to be **not related** to study product (i.e., dalbavancin or SOC antibiotic) will be captured on the appropriate data collection form and eCRF, but do not require separate reporting to the DMID Pharmacovigilance Group.

At any time after completion of the study, if the site principal investigator or appropriate sub-investigator becomes aware of an SAE that is suspected to be related to dalbavancin that occurred during the subject's participation in the study, the site principal investigator or appropriate sub-investigator will report the event to the DMID Pharmacovigilance Group.

8.3.2 Regulatory Reporting for Studies Conducted Under DMID Sponsored IND

Following notification from the site Principal Investigator or appropriate sub-investigator, DMID, as the IND sponsor, will report any suspected unexpected serious adverse event. DMID will report an SAE as a Suspected Unexpected Serious Adverse Reaction (SUSAR) only if there is evidence to suggest a causal relationship between the study intervention and the SAE and based on whether that event is listed in the investigator brochure. DMID will submit an IND safety report to the FDA and will notify all participating site Principal Investigators (i.e., all Principal Investigators to whom the sponsor is providing drug under its IND(s) or under any Principal Investigator's IND(s) of potential serious risks from clinical studies or any other source, as soon as possible. DMID will report to the FDA any unexpected fatal or life-threatening suspected adverse reaction as soon as possible, but in no case later than 7 calendar days after the sponsor's initial receipt of the information. If the event is not fatal or life-threatening the IND safety report will be submitted within 15 calendar days after the sponsor determines that the information qualifies for reporting as specified in 21 CFR Part 312.32. Relevant follow up information to an IND safety report will be submitted as soon as the information is available. Upon request from FDA, DMID will submit to the FDA any additional data or information that the agency deems necessary, as soon as possible, but in no case later than 15 calendar days after receiving the request.

All SAEs designated as "not related" to study product(s), will be reported to the FDA at least annually in a summary format.

8.3.3 Reporting of Pregnancy

Female subjects of childbearing age who become pregnant during the study prior to day 8 will not receive the second dose of dalbavancin (if randomized to the dalbavancin group) and will be followed for pregnancy outcome. Pregnancy occurring during a subject's participation in the study must be recorded in the database within 5 days of site awareness, on the Pregnancy Report form. The report will include pregnancy outcome (e.g., any premature terminations, elective or therapeutic, any spontaneous abortions or stillbirths), as well as the health status of the mother and child, including date of delivery and infant's sex and weight. Any subject with a positive pregnancy test, who has received study product(s), will be followed through eight (8) weeks post-live delivery or elective or natural termination of the pregnancy, whichever occurs first. If the database is locked at time of pregnancy, a supplemental report will be generated and completed after birth, which will be appended to the database. Any occurring AEs or SAEs that occur to the mother or fetus will be recorded in the eCRF in the database and on the SAE Report form.

The site is responsible for notifying the IRB/IEC on which it relies of any pregnancies in accordance with their policies as well as notifying their local IRB/IEC of any pregnancies in accordance with local policies.

8.4 Type and Duration of Follow-up of Subjects after Adverse Events

AEs will be assessed, and AEs that are CTCAE grade 3 and higher, as well as all AESIs, will be followed from initial recognition of the AE until resolution or through the end of the study, whichever occurs first.

SAEs will be followed up through resolution even if duration of follow-up goes beyond the protocol-defined follow-up period (test of cure).

Resolution of an AE is defined as the return to pre-treatment status or stabilization of the condition with the expectation that it will remain chronic.

8.5 Halting Rules

8.5.1 Study Halting Criteria

The halting rules outlined below will be used to evaluate whether it is safe to proceed with dosing or whether the study should be suspended for further safety evaluation.

If any of the halting rules below are met, further enrollment will be halted pending DSMB review of safety data and recommendations. The continuation of the administration of dalbavancin (the second dose) or switch to SOC will be at the site PI discretion.

- More than one subject **death** (e.g., 2 or more deaths) during participation in the study that is **suspected to be related** to the treatment with **dalbavancin**. Note that mortality among patients with *S. aureus* bacteremia is not uncommon, and deaths that are not directly and solely related to dalbavancin will not by themselves trigger this halting rule.
- Five or more subjects in the study are suspected with development of drug induced liver injury (DILI) that are assessed as **related** to **dalbavancin.**
- Five or more subjects in the study experience a Grade 4 **related** to **dalbavancin** AEs (laboratory or systemic) that are coded in the same HLGT per MedDRA classification.

8.5.2 Individual Halting Rules

A subject will be discontinued from further dosing in either treatment group if any of the following criteria are met:

- An individual infusion must be stopped if a drug-related hypersensitivity (grade 2 or higher) to dalbavancin or standard of care study drug is suspected, including anaphylaxis. Note that dalbavancin infusion can cause reactions that resemble "Red-Man Syndrome," including flushing of the upper body, urticaria, pruritus, and/or rash. Stopping or slowing the infusion may result in cessation of these reactions and this reaction is not by itself a reason to discontinue dalbavancin for an individual subject.
- New onset of illness or condition that meets exclusion criteria, at the investigator's discretion
- The treatment of any subject may be stopped for SAEs, clinically significant adverse events, including severe laboratory abnormalities that indicate to the Investigator that continued dosing is not in the best interest of the patient.

8.6 Safety Oversight

8.6.1 Data and Safety Monitoring Board (DSMB)

Safety oversight will be conducted by a DSMB that is an independent group with expertise to interpret data from this study and will monitor subject safety and advise DMID. The DSMB members will be separate and independent of study personnel participating in the study and should not have scientific, financial, or other conflict of interest related to this study. DSMBs must consist of at least three voting members, including a biostatistician experienced in statistical methods for clinical trials and a clinician with relevant expertise.

The DSMB will operate under the rules of a DMID-approved charter that defines the data elements to be assessed and the procedures for data reviews and will be written at the organizational meeting of the DSMB. Procedures for DSMB reviews/meetings will be defined in the charter. Reports may include enrollment and demographic information, medical history, concomitant medications, physical assessments, clinical laboratory values, dosing compliance, and solicited and unsolicited AE/SAEs. The DSMB will review SAEs on a regular basis and ad hoc during this trial. The DMID Medical Monitor will be responsible for reviewing SAEs in real time.

As defined in the charter, the DSMB will review data at specified times during the course of the study for subject and overall study progress and will conduct ad hoc reviews as appropriate when a halting rule is met or for immediate concerns regarding observations during this study.

The DSMB will conduct the following meetings:

- Organizational meeting
- Data review meeting for safety
- Every year
- An Interim analysis for futility after at least approximately 50% of subjects have completed the study
- Ad hoc meetings will occur when a halting rule is met, or when DMID or the DSMB chair has immediate concerns regarding observations during the trial
- Final review meeting will occur 6-8 months after the clinical database is locked to review cumulative safety and efficacy data. The final CSR, if available, will be provided for this review. The DSMB may be asked to provide recommendations in response to DMID’s questions.

Additional data may be requested by the DSMB, and interim statistical reports may be generated as deemed necessary and appropriate by DMID. The DSMB may receive data in aggregate and presented by treatment arm. The DSMB may also be provided with expected and observed rates of the expected AEs. The DSMB will review grouped data in the closed session only. As an outcome of each review/meeting, the DSMB will make a recommendation as to the advisability of proceeding with study treatments), and to continue, modify, or terminate this trial.

9 HUMAN SUBJECTS PROTECTION

9.1 Institutional Review Board/Independent Ethics Committee

Each site principal investigator will obtain IRB approval for this protocol to be conducted at his/her research site(s) and send supporting documentation to the DMID before initiating recruitment of subjects. The investigator will submit applicable information to the IRB/IEC on which it relies for the review, to conduct the review in accordance with 45 CFR 46, ICH E6 GCP, and as applicable, 21 CFR 56 (Institutional Review Boards) and 21 CFR 50 (Protection of Human Subjects), other federal, state, and local regulations. The IRB/IEC must be registered with OHRP as applicable to the research. DMID must receive the documentation that verifies IRB/IEC-approval for this protocol, associated informed consent documents, and upon request any recruitment material and handouts or surveys intended for the subjects, prior to the recruitment and enrollment of subjects.

Any amendments to the protocol or consent materials will be approved by the IRB/IEC before they are implemented. IRB/IEC review and approval will occur at least annually throughout the enrollment and follow-up of subjects and may cease if annual review is no longer required by applicable regulations and the IRB/IEC. The investigator will notify the IRB/IEC of deviations from the protocol and reportable SAEs, as applicable to the IRB/IEC policy.

Each institution engaged in this research will hold a current FWA issued by the Office of Human Research Protection (OHRP) for federally funded research.

A single IRB of record, (WIRB Copernicus Group), will be accountable for compliance with regulatory requirements for this multi-centered study, at participating sites. A formal Reliance Agreement will be required between the single IRB and participating sites. The formal Reliance Agreement will set forth the specific responsibilities of the IRB and each participating site. Participating sites will then rely on the IRB of record to satisfy the regulatory requirements relevant to the IRB review. The participating sites will maintain essential required documentation of IRB reviews, approvals, and correspondence, and must provide copies of any agreements and essential documentation to the DMID or regulatory authorities upon request.

9.2 Informed Consent Process

Informed consent is a process that is initiated prior to an individual agreeing to participate in a trial and continuing throughout the individual's trial participation. Before any study procedures are performed, informed consent will be obtained and documented. Subjects will receive a concise and focused presentation of key information about the clinical trial, verbally and with a written consent

form. The explanation will be organized, and presented in lay terminology and language that facilitates understanding why one might or might not want to participate.

An investigator or designee will describe the protocol to potential subjects face-to-face. The key information about the purpose of the study, the procedures and experimental aspects of the study, risks and discomforts (including the PK sampling plan for subjects receiving dalbavancin), any expected benefits to the subject, and alternative treatment will be presented first to the subject.

Subjects will also receive an explanation that the trial involves research, and a detailed summary of the proposed study procedures and study interventions/products. This will include aspects of the trial that are experimental, the probability for random assignment to treatment groups, any expected benefits, all possible risks (including a statement that the particular treatment or procedure may involve risks to the subject or to the embryo or fetus, if the subject is or may become pregnant, that are currently unforeseeable), the expected duration of the subject's participation in the trial, alternative procedures that may be available and the important potential benefits and risks of these available alternative procedures.

Subjects will be informed that they will be notified in a timely manner if information becomes available that may be relevant to their willingness to continue participation in the trial. Subjects will receive an explanation as to whether any compensation and any medical treatments are available if injury occurs, and, if so, what they consist of, or where further information may be obtained. Subjects will be informed of the anticipated financial expenses, if any, to the subject for participating in the trial, as well as any anticipated prorated payments, if any, to the subject for participating in the trial. They will be informed of whom to contact (e.g., the investigator) for answers to any questions relating to the research project.

Information will also include the foreseeable circumstances and/or reasons under which the subject's participation in the trial may be terminated. The subjects will be informed that participation is voluntary and that they are free to withdraw from the study for any reason at any time without penalty or loss of benefits to which the subject is otherwise entitled.

The extent of the confidentiality of the subjects' records will be defined, and subjects will be informed that applicable data protection legislation will be followed. Subjects will be informed that the monitor(s), auditors(s), IRB, NIAID, and regulatory authority(ies) will be granted direct access to the subject's original medical records for verification of clinical trial procedures and/or data without violating the confidentiality of the subject, to the extent permitted by the applicable laws and regulations, and that, by signing a written informed consent form, the subject is authorizing such access.

Subjects will be informed that records identifying the subject will be kept confidential, and, to the extent permitted by the applicable laws and/or regulations, will not be made publicly available and, if the results of the trial are published, the subject's identity will remain confidential. Subjects will

be informed whether private information collected from this research and/or specimens will be used for additional research, even if identifiers are removed.

Subjects will be allowed sufficient time to consider participation in this research trial, and have the opportunity to discuss this trial with their family, friends or legally authorized representative, or think about it prior to agreeing to participate.

Informed consent forms will be IRB-approved and subjects will be asked to read and review the consent form. Subjects must sign the informed consent form prior to starting any study procedures being done specifically for this trial.

Once signed, a copy of the informed consent form will be given to the subject(s) for their records. The subject(s) may withdraw consent at any time throughout the course of the trial. The rights and welfare of the subject(s) will be protected by emphasizing to them that the quality of their medical care will not be adversely affected if they decline to participate in this study.

Study personnel may employ recruitment efforts prior to obtaining study consent if a patient-specific screening consent is on record or if the IRB has agreed that chart review is allowed without a fully executed screening consent. In cases where there is not a patient-specific screening consent on record, site Clinical staff may pre-screen via chart review and refer potential subjects to the Research staff. Research staff would obtain written consent per the standard informed consent process before conducting protocol-specific screening activities.

New information will be communicated by the site principal investigator to subjects who consent to participate in this trial in accordance with IRB requirements. The informed consent document will be updated and subjects will be re-consented per IRB requirements, if necessary. Subjects will be given a copy of all informed consent forms that they sign.

9.2.1 Other Informed Consent Procedures

Use of a Legally Authorized Representative (LAR)

Potential subjects for this study are adults but may be unable to provide legally effective informed consent due to their health status (*i.e.*, dementia, intubated, sedated). The subjects may be enrolled in the study if consent is obtained from the LAR. The investigator will be familiar with the IRB/IEC policy on which he/she relies regarding the priority list of LAR and whether enrollment in a study is permitted by an advanced directive *(e.g.*, living will, durable power of attorney for proxy consent). Additionally, subjects will be informed about the study to the extent compatible with the person's understanding, and enrollment declined if the subject refuses participation.

If a LAR originally provides legally effective informed consent and the subject's condition improves, the subject will also be informed about the study as soon as is feasible and will be re-consented. The subject may continue in the study only if the subject's consent is provided.

9.3 Exclusion of Women, Minorities, and Children (Special Populations)

Children will be excluded from this trial. Management algorithms for *S. aureus* bacteremia are different in children and their participation would thus not be appropriate.

9.4 Subject Confidentiality

Subject confidentiality is strictly held in trust by the participating investigators, their staff, and the sponsor(s) and their agents. This confidentiality includes documentation, investigation data, subject's clinical information, and all other information generated during participation in the study. No information concerning the study or the data generated from the study will be released to any unauthorized third party without prior written approval of the DMID and the subject. Subject confidentiality will be maintained when study results are published or discussed in conferences. The study monitor or other authorized representatives of the sponsor or governmental regulatory agencies may inspect all documents and records required to be maintained by the investigator, including but not limited to, medical records (office, clinic, or hospital) and pharmacy records for the subjects in this study. The clinical study site will permit access to such records.

All records will be kept locked and all computer entry and networking programs will be carried out with coded numbers only and with password protected systems. All non-clinical specimens, evaluation forms, reports, and other records that leave the site will be identified only by a coded number.

9.5 Certificate of Confidentiality

To protect privacy, we have received a Certificate of Confidentiality. With this Certificate, the researchers cannot be forced to release information that may identify the research subject, even by a court subpoena, in any federal, state, or local civil, criminal, administrative, legislative, or other proceedings. The researchers will use the Certificate to resist any demands for information that would identify the subject, except as explained below.

The Certificate cannot be used to resist a demand for information from personnel of the United States Government that is used for auditing or evaluation of federally funded projects, like this study, or for information that must be released in order to meet the requirements of the Federal Food and Drug Administration (FDA).

A Certificate of Confidentiality does not prevent the subject from voluntarily releasing information about themselves or their involvement in this research. If any person or agency obtains a written consent to receive research information, then the researchers may not use the Certificate to withhold that information.

The Certificate of Confidentiality does not prevent the researchers from reporting without the

subject's consent, information that would identify the subject as a participant in the research project regarding matters that must be legally reported including: child and elder abuse, sexual abuse, or wanting to harm themselves or others.

The release of individual private information or specimens for other research will only occur if consent was obtained from the individual to whom the information, document, or biospecimen pertains.

9.6 Costs, Subject Compensation, and Research Related Injuries

There is no cost to subjects for the research tests, procedures, and study product while taking part in this trial. Procedures and treatment for clinical care may be billed to the subject, subject's insurance or third party. Subjects may be compensated for their participation in this trial. Compensation will be in accordance with the local IRB's policies and procedures, and subject to IRB approval.

If it is determined by the site principal investigator that an injury occurred to a subject as a direct result of the tests or treatments that are done for this trial, then referrals to appropriate health care facilities will be provided to the subject. Study personnel will try to reduce, control, and treat any complications from this trial. Immediate medical treatment may be provided by the participating site. No financial compensation will be provided to the subject by the NIAID, NIH to the subject for any injury suffered due to participation in this trial.

10 STATISTICAL CONSIDERATIONS

This is a phase 2b, multicenter, open-label, randomized, assessor-blinded, superiority study comparing a 2-dose dalbavancin regimen to standard of care therapy for the treatment of complicated bacteremia caused by *S. aureus*. Participants will be followed for 70 days, except for the subset with osteomyelitis who will be followed for 180 days.

On the basis of the intention-to-treat (ITT) principle, the primary outcome will be analyzed on the ITT set (defined as all participants as randomized regardless of whether they received the randomized treatment). A sensitivity analysis will be conducted using the modified ITT (mITT) population - defined as all participants who received at least one dose of study drug. Additionally, as one of the secondary assessments, we will compare the individual components of the DOOR outcome by treatment arm within the ITT population.

The secondary efficacy assessment, a non-inferiority analysis of overall clinical success rates by treatment group, will be conducted on the mITT population.

The secondary safety assessment will analyze rates of SAEs or of AEs leading to study drug discontinuation on the mITT population.

For all tests, *P* values will be two-sided with alpha < 0.05 level of significance. All reported confidence intervals will be two-sided 95%.

The outcomes could be missing for subjects who withdraw from the trial. The reasons for withdrawal will be reported and compared qualitatively by groups. The effect that any missing data might have on results will be assessed via sensitivity analysis. If the pattern of missing data is different to that envisaged at the design stage, further sensitivity analyses will be provided that are tailored to the missing data pattern observed.

The statistical analysis plan, which includes more technical and detailed elaboration of the principal features stated in the protocol, will be prepared separately.

10.1 Study Hypotheses

The primary objective is to compare the Desirability of Outcome Ranking (DOOR) at day 70 for dalbavancin versus standard of care antibiotic therapy for the treatment of complicated *S. aureus* bacteremia, conducted as a superiority assessment within the intention to treat population. We hypothesize that dalbavancin will have a higher DOOR relative to standard of care (e.g., the probability of a randomly selected patient having a better DOOR if assigned to receive dalbavancin versus standard of care (plus half the probability of a tied DOOR) is >50%). The null hypothesis would be no significant difference in DOOR between dalbavancin versus standard of care.

The key secondary objective will assess traditional clinical efficacy examining composite occurrence of clinical failure, infectious complications, or mortality as a non-inferiority comparison within the intention to treat population. We hypothesize that dalbavancin will have a non-inferior clinical efficacy rate relative to standard of care within a 20% absolute margin. The null hypothesis would be that dalbavancin has an inferior clinical efficacy relative to standard of care. The selection of a 20% non-inferiority margin is in line with previously published SAB trials, including the registrational trial of daptomycin versus standard of care (Fowler et al 2006). The protocol for an upcoming trial of ceftobiprole versus daptomycin includes a similar margin constructed around the particular context of SAB, noting that a non-inferiority margin of 20% includes more than half of the anticipated benefit of active-control treatment relative to untreated subjects (Hamed et al 2020). Additional secondary objectives will include a safety assessment and evaluation of the individual components of the primary DOOR outcome. Safety will be assessed by comparing the occurrence of serious (grade 3 or worse) adverse drug events between the dalbavancin and standard of care arms within the safety population. Each of the components of the DOOR outcome will be compared between the dalbavancin arm and standard of care arm within the ITT population.

10.2 Sample Size Considerations

The study is powered for a superiority comparison based on the primary objective, a comparison of DOOR outcomes. The probability of a subject from the dalbavancin arm having a superior DOOR ranking relative to a subject from the standard of care arm will be calculated along with a 95% confidence interval. Superiority will be considered to have been achieved if the 95% confidence interval for probability of having a superior DOOR ranking with dalbavancin does not cross 50%. If the confidence interval crosses 50% however, the null hypothesis cannot be rejected.

Sample size was calculated on the basis of the primary hypothesis. Assuming a 65% probability of a better DOOR in the dalbavancin treatment group versus the standard of care treatment group, with a 90% power and alpha=0.025 (by one-sided Wilcoxon rank sum test), 78 participants would be required in each treatment group. To allow for some inflation assuming around 12% of missing data or other study imperfections, using the method in Lachin (1981), we plan to recruit 100 per arm (200 subjects in total). Sample size was calculated using nQuery (MTT1-1 Module) (Version 8, Statistical Solution Ltd).

10.3 Treatment Assignment Procedures

10.3.1 Randomization Procedures

Once consented and upon entry of demographic data and confirmation of eligibility for the trial, the subject will be enrolled. Enrollment of subjects will be done online using the enrollment module of

Advantage eClinical. Subjects will be randomized 1:1 to dalbavancin or standard of care. Randomization will be stratified based on screening pathogen, MSSA vs. MRSA.

The list of randomized treatment assignments will be prepared by statisticians at the DCC (The Emmes Company). Emmes will assign each subject a treatment code and treatment assignment from the list after demographic and eligibility data have been entered.

Instructions for use of the enrollment module are included in the Advantage eClinical User's Guide. Manual back-up procedures and instructions are provided for use in case the site temporarily loses access to the internet or the online enrollment system is unavailable.

10.3.2 Masking Procedures

Study subjects and treating physicians will not be masked to treatment, as this is an open label study. Treatment group will be masked for study adjudicators.

10.4 Planned Interim Analyses

The DSMB will review interim reports of efficacy by treatment strategy. There will be one formal interim analysis of futility after approximately 50% of subjects have completed the trial. The statistical methods for the interim analysis will be fully specified in advance in a statistical analysis plan (SAP), to be prepared by a statistician, and summarized briefly in Section 10.4.2 below.

10.4.1 Interim Safety Review

The DSMB will evaluate safety at pre-specified intervals and at least yearly; however, ongoing review and summary of subject safety will occur to allow for early detection of a safety signal that may result from an AE or lack of efficacy of study drug. The DSMB will advise DMID on whether to continue, modify, or terminate the trial based on a risk-benefit assessment.

10.4.2 Interim Futility Review

A single interim analysis will be performed after approximately 50% of subjects have completed the trial. The interim futility analysis will consist of a quantitative evaluation of potential effect sizes and associated precision using a predicted intervals and predicted interval plots (PIPS) approach. Briefly, predicted intervals for both primary DOOR and secondary clinical failure outcomes will be modeled under a range of assumptions including: 1) the trends in outcomes observed at interim analysis continue to end of study, 2) the alternative hypothesis is true, 3) the null hypothesis is true, and 4) best and worst case scenarios for remaining outcomes. By relying on prediction intervals, no statistical hypothesis testing is required and no power is lost at interim analysis.

10.5 Final Analysis Plan

Results from primary and secondary endpoint analyses may be distributed by the SDCC to key study team members (protocol PIs, protocol statisticians, and other necessary study team members) after database lock and prior to the generation of all the CSR Tables, Listings, and Figures. These analyses may be used by the company collaborator for planning subsequent trials or by the lead principal investigator for manuscript and abstract development while the CSR is being finalized.

10.5.1 Study Populations

Five populations will be considered in the statistical analysis of the study:

1. *Screened Population*: The screened population will consist of all patients who undergo the Screening Visit (Visit 1) and receive a Patient Identification (PID) number.
2. *Intent-to-Treat/Randomized Population*: The intent-to-treat (ITT) population will consist of all randomized patients regardless whether or not they received study treatment.
3. *Safety Population*: The safety population will consist of all randomized patients who received at least 1 dose of study drug. Patients will be analyzed based on the treatment received.
4. *Modified Intent-to-Treat Population*: The modified intent-to-treat (mITT) population will consist of all patients in the ITT population who received at least one dose of study drug.
5. *Clinically Evaluable Population*: The CE population will consist of all patients in the mITT population who met criteria for clinical evaluability. Patients will be considered clinically evaluable if they have a primary outcome assessment and do not have missing data or major protocol violations that prevent the adjudication committee from evaluating their outcomes.

An independent, blinded adjudication committee will be used to review the data from each patient to establish the baseline diagnosis, final diagnosis, and final outcome (including DOOR outcome and its individual components), including reasons for treatment failure. This committee will consist of 3-4 infectious disease experts with no relevant conflicts of interest.

10.5.2 Patient Disposition

The number of subjects in four of the study populations (ITT, Safety, mITT, and CE) will be summarized by treatment group and study center; the screened population will only be summarized by study center.

Screen failures (i.e., patients screened but not randomized) and the associated reasons for failure will be tabulated overall. The number and percentage of subjects who complete the treatment period (up to Day 42) and of subjects who prematurely discontinue during the same period will be presented for each treatment group and pooled across treatment groups for the ITT population. The reasons for premature discontinuation as recorded on the termination pages of the eCRF will be summarized (number and percentage) by treatment group for all randomized subjects, along with the number of subjects completing each visit.

10.5.3 Demographics and Other Baseline Characteristics

Demographic parameters (i.e., age, race, ethnicity, sex, weight, height, body mass index) and other baseline characteristics will be summarized by treatment group for the Safety and ITT populations. Continuous variables will be summarized by number of subjects and mean, standard deviation (SD), median, minimum, and maximum values. Categorical variables will be summarized by number and percentage of subjects. The number and percentage of subjects with abnormalities in medical and surgical histories in each system organ class and preferred term will be summarized by treatment group for the ITT population.

Prior medication is defined as any medication taken before the date of the first dose of study product. Concomitant medication is defined as any medication started on or after the date of the first dose of study product.

Both prior and concomitant medication use will be summarized by the number and proportion of subjects in each treatment group receiving each medication within each therapeutic class for the safety population. If a subject took a specific medication multiple times or took multiple medications within a specific therapeutic class, that subject would be counted only once for the coded drug name or therapeutic class.

10.5.4 Extent of Exposure and Treatment Compliance

Exposure to study product for the safety population will be summarized for treatment duration, calculated as the number of doses of dalbavancin received for patients in the dalbavancin group, and by start and stop dates for standard of care antibiotics. Descriptive statistics (n, mean, standard deviation, minimum, median, and maximum) will be presented by treatment group.

10.5.5 Efficacy Analysis

10.5.5.1 Primary Efficacy Analysis

The primary efficacy endpoint is the DOOR outcome at Day 70 post study entry (test of cure) in the ITT population. The clinical components of the DOOR outcome (survival, clinical success/failure, and infectious complications) will be determined by the blinded clinical adjudication committee (on a rolling basis). The DOOR probability is calculated, using the equation:

$$\text{DOOR probability} = \Pr[DOOR_{\mathrm{D}} > DOOR_{\mathrm{SOC}}] + \tfrac{1}{2}\Pr[DOOR_{D} = DOOR_{\mathrm{SOC}}],$$

where $DOOR_{\mathrm{D}}$ and $DOOR_{\mathrm{SOC}}$ are the DOOR outcomes for dalbavancin and standard care groups, respectively, and $\Pr[DOOR_{\mathrm{D}} > DOOR_{\mathrm{SOC}}]$ is the probability of a DOOR from dalbavancin exceeding a DOOR from standard care and $\Pr[DOOR_{D} = DOOR_{\mathrm{SOC}}]$ is the proportion of two DOOR outcomes being same. Pathogen (MSSA versus MRSA), which is considered in the permuted block randomization as a strata, will be incorporated into the calculation of the DOOR probability and its

corresponding 95% confidence interval (the stratified analysis). In addition, the DOOR probability and 95% confidence interval without strata will be calculated.

As a secondary analysis of the primary outcome will be:

i. DOOR distribution by groups
ii. Cumulative difference in DOOR categories for dalbavancin vs. Standard Care
iii. Point estimate and confidence interval of difference in mean partial credit vs. Standard Care
iv. Expected DOOR distribution for Standard Care and the expected numbers gained loss in each category with treatment

The same analysis above will be repeatedly conducted on the DOOR outcome at Day 42.

As an explanatory analysis of the primary outcome, in addition to pathogen (MSSA vs. MRSA), baseline covariate-adjustment analysis and subgroup analysis will be conducted.

10.5.5.2 Secondary Efficacy Outcomes

As the secondary efficacy outcomes, each component of the DOOR will also be examined separately: clinical success, infectious complications, SAEs, AEs leading to study drug discontinuation, all-cause mortality. Descriptive statistics, including number and percentage for the categorical variables, will be provided by groups.

Clinical failure at Day 70 will be analyzed using generalized estimating equations (GEE) assuming an unstructured correlation structure, including clinical failure at Day 42 (explanatory analysis time point). The difference in proportions of clinical failure between the two groups at Day 70 will be calculated with the corresponding 2-sided 95% confidence interval. As a sensitivity analysis, generalized linear mixed model (GLMM) approach will be used to analyze clinical failure.

10.5.5.3 Additional Efficacy Parameters

1. QoL score – QoL will be assessed using the ARLG Bloodstream Infection QoL Measure for the primary and exploratory endpoints (Appendix C). Two additional comparator measures (EQ-5D-5L, https://euroqol.org/eq-5d-instruments/sample-demo/ and PROMIS Global Health short form, http://www.healthmeasures.net/administrator/components/com_instruments/uploads/Global%20Health%20Scale%20v1.2%2008.22.2016.pdf) will also be collected and will be used in exploratory analyses only.
2. Desirability of Outcome Ranking (DOOR) (Evans 2015) endpoint results at Day 42.

For the additional efficacy endpoints, descriptive statistics will be provided by treatment group. Continuous variables will be summarized by number of patients and mean, SD, median, minimum, and maximum values by treatment group. Categorical variables will be summarized by number and percentage of patients by treatment group. For QoL measures, the descriptive statistics will be presented by item as well.

Descriptive statistics for the DOOR endpoint will be provided by treatment group and summarized by number of patients and mean, SD, median, minimum, and maximum values; p-values will be determined using the Wilcoxon-rank sum test for continuous variables.

10.5.6 Safety Analyses

Safety analyses will be based on the safety population. Safety will be assessed using descriptive statistics of AEs, vital signs, and laboratory tests by treatment group. For each safety parameter, the last assessment made before the first dose of study product will be used as the baseline for all analyses of that safety parameter.

10.5.6.1 Adverse Events

An AE (classified by preferred term) that occurs during the treatment period will be considered a treatment-emergent AE if it was not present before the first dose of study product or was present before the first dose of study product and increased in severity during the treatment period.

The number and percentage of patients reporting treatment-emergent AEs in each treatment group will be tabulated by system organ class and preferred term; by system organ class, preferred term, and severity; and by system organ class, preferred term, and causal relationship to the study product. If more than one AE is coded to the same preferred term for the same subject, the subject will be counted only once for that preferred term using the most severe and most related occurrence for the summarization by severity and by causal relationship to the study product.

The distribution of treatment-emergent AEs and AESIs by severity and causal relationship to the study product will be summarized by treatment group.

The incidence of common (≥ 2% of patients in any treatment group) treatment-emergent AEs, on-therapy SAEs, AESIs, and AEs leading to premature discontinuation of the study product will be summarized by preferred term and treatment group and will be sorted by decreasing frequency for the study product. In addition, the incidence of fatal on-therapy SAEs (i.e., events that caused death) will be summarized separately by treatment group and preferred term. An SAE will be defined as an on-therapy SAE if it occurred during or after the first infusion of study product.

Listings will be presented for subjects with SAEs, AESIs, subjects with AEs leading to discontinuation, and subjects who die (if any).

10.5.6.2 Clinical Laboratory Parameters, and Vital Signs

Descriptive statistics for clinical laboratory values and changes from the baseline values at each assessment time point will be presented by treatment group for each clinical laboratory parameter.

Descriptive statistics for vital signs (e.g., pulse rate, systolic and diastolic BP) and changes from baseline values at each visit and at end of study will be presented by treatment group.

The number and percentage of subjects with potentially clinically significant (PCS) post-baseline clinical laboratory values will be tabulated by treatment group. The criteria for PCS laboratory values will be detailed in the statistical analysis plan. The percentages will be calculated relative to the number of subjects with available non-PCS baseline values and at least 1 post-baseline assessment. The numerator will be the total number of subjects with available non-PCS baseline values and at least 1 PCS post-baseline value. A supportive listing of subjects with PCS post-baseline values will be provided, including the PID number, study center number, and baseline and post-baseline values. A listing of all AEs that occur in subjects who have PCS laboratory values or vital signs will also be provided.

10.5.6.3 Exploratory Dalbavancin Plasma Pharmacokinetic Analyses

A range of exploratory analyses related to dalbavancin pharmacokinetics will be conducted among the subset of subjects receiving dalbavancin (n=100). The methodology used to evaluate PK parameters will be described in detail in the PK Plan and is reviewed briefly below.

Pharmacokinetic Analysis

Dalbavancin concentration-time data will be visualized using box and whisker plots, with investigation of any outliers for erroneous time or concentration data entry. Queries will be generated to resolve potential erroneous time or concentration data point entries due to transcription or measurement errors. Individual concentration-time plots will be generated on linear and semi-log scales to inform potentially optimal models for analysis.

Non-Compartmental Pharmacokinetic Analysis

Non-compartmental analysis (NCA) will serve as the initial approach to generate base PK parameter estimates for dalbavancin concentration-time data. These analyses will be conducted using an appropriate statistical package (e.g., Phoenix WinNonlin v8.2 or higher). This descriptive analysis will allow for comparison to previously published data. The following PK exposures for concentration-time data will be calculated as appropriate and if possible depending upon samples collected: plasma concentration prior to dose, maximum plasma concentration (C_{max}) after the first dose on Day 1, time to C_{max} (T_{max}), plasma concentration on Day 8 [C_{8day}], plasma concentration on day 22 (C_{22day}), concentration on day 42 (C_{42day}), concentration on day 70 (C_{70day}), area under the plasma concentration-time curve (AUC) from days 0-8 ($AUC_{0\text{-}8days}$), AUC from days 0-22 ($AUC_{0\text{-}22days}$), AUC from days 0-42 ($AUC_{0\text{-}42day}$), days 0-70 ($AUC_{0\text{-}70days}$), AUC from days 8-22 ($AUC_{8\text{-}22days}$), AUC from days 22-42 ($AUC_{22\text{-}42day}$), AUC from days 42-70 ($AUC_{42\text{-}70day}$), AUC to the last quantifiable sample ($AUC_{0\text{–}last}$), and AUC to infinity (AUC0–∞).

Population Pharmacokinetic Analysis

Population Pharmacokinetic Analysis. Population pharmacokinetic analysis provides a platform to identify patient covariates which can help explain a portion of the interindividual variability in

selected PK parameters. The non-linear mixed effects modeling software NONMEM Version 7.3 or higher (ICON Development Solutions, Ellicott City, MD) will be used to develop the population PK model for dalbavancin concentrations in plasma. The first-order conditional estimation method with interaction (FOCEI) will be utilized; other estimation methods such as expectation-maximization (e.g., SAEM) will also be considered.

Structural PK model base development to model the plasma dalbavancin concentration-time data will be initiated using a linear three-compartment model with zero-order infusion as has been used previously (https://www.ncbi.nlm.nih.gov/pubmed/31087630). Other model modifications will be considered as necessary. Between-subject variability associated with model parameters and differing residual error structures will be tested. Model development will be guided by goodness of fit plots, plausibility of parameter estimates, reduction in inter-individual variability for structural and residual error parameters, as well as objective function and shrinkage values.

Upon selection of an appropriate base structural PK model, covariate effects (e.g., age, gender, body size descriptors, creatinine clearance, albumin and IV drug use status) will be evaluated using stepwise forward selection followed by stepwise backward elimination processes. Model validation will be assessed through visual predictive checks as well as bootstrapping. A listing of the individual PK parameters derived using the final population PK model will be provided for each patient. The steady-state volume of distribution (V_{ss}) will be calculated as the sum of the central (V_c) and peripheral volume terms (V_{p1} and V_{p2}). The alpha-phase half-life ($T_{1/2,\alpha}$) beta-phase half-life ($T_{1/2,\beta}$) and gamma-phase half-life ($T_{1/2,\gamma}$) will be calculated for each patient using the individual post-hoc PK parameters. Summary statistics (mean, standard deviation, median, minimum and maximum) will be calculated using CL, V_c, V_{p1}, V_{p2}, V_{ss}, $T_{1/2,\alpha}$, $T_{1/2,\beta}$, and $T_{1/2,\gamma}$ values.

Exposure-Response Relationship Analysis

Using the final population PK model, individual post-hoc PK parameters, and the individual patient dosing histories, simulations will be performed to generate plasma dalbavancin concentration-time profiles and calculate plasma dalbavancin exposure measures for each study subject. The following exposure variables will be calculated for each patient: C_{max}, C_{8day}, C_{22day}, C_{42day}, C_{70day}, $AUC_{0-8days}$, $AUC_{0-22days}$, $AUC_{0-42day}$, $AUC_{0-70days}$, $AUC_{8-22days}$, $AUC_{22-42day}$, $AUC_{42-70day}$, and AUC to infinity ($AUC0–\infty$). The associations between each of the simulated dalbavancin exposures, as appropriate, and each outcome of interest will be explored using standard exposure-response methodologies. Only simulated exposures that occurred prior to the outcome of interest will be considered. The outcomes evaluated will include (1) DOOR outcomes at day 42, mortality at day 42, infectious complications at day 42, adverse drug effects (grade 3 or higher) at day 42, late recurrence at 6 months (within the subset having osteomyelitis), and occurrence of AST/ALT elevation >3x upper limit of normal during treatment. Multivariable models will be used to estimate the effect of individual exposures measures on outcomes while accounting for relevant covariates.

11 SOURCE DOCUMENTS AND ACCESS TO SOURCE DATA/DOCUMENTS

Each participating site will maintain appropriate medical and research records in compliance with ICH E6, Section 4.9 and regulatory and institutional requirements for the protection of confidentiality of subjects. Each site will permit authorized representatives of the DMID, its designees, and appropriate regulatory agencies to examine (and when required by applicable law, to copy) clinical records for the purposes of quality assurance reviews, audits, and evaluation of the study safety and progress. These representatives will be permitted access to all source data and source documents, which include, but are not limited to, hospital records, clinical and office charts, laboratory notes, memoranda, subjects' memory aid or evaluation checklists, pharmacy dispensing records, recorded data from automated instruments, copies or transcriptions certified after verification as being accurate and complete, microfiches, photographic negatives, microfilm or magnetic media, x-rays, and subject files and records kept at the pharmacy, at the laboratories, and medico-technical departments involved in the clinical trial.

12 QUALITY CONTROL AND QUALITY ASSURANCE

Following a written DMID-accepted site quality management plan, each participating site(s) and its subcontractors are responsible for conducting routine quality assurance (QA) and quality control (QC) activities to internally monitor study progress and protocol compliance. The site principal investigator will provide direct access to all study-related sites, source data/data collection forms, and reports for the purpose of monitoring and auditing by the sponsor, and inspection by local and regulatory authorities. The site principal investigator will ensure all study personnel are appropriately trained and applicable documentations are maintained on site.

The DCC will implement quality control procedures beginning with the data entry system and generate data quality control checks that will be run on the database. Any missing data or data anomalies will be communicated to the participating site(s) for clarification and resolution.

13 DATA HANDLING AND RECORD KEEPING

13.1 Data Management Responsibilities

The investigator is responsible to ensure the accuracy, completeness, legibility, and timeliness of the data reported. All source documents should be completed in a neat, legible manner to ensure accurate interpretation of data. Black or blue permanent ink is required to ensure clarity of reproduced copies. When making changes or corrections, cross out the original entry with a single line, and initial and date the change. DO NOT ERASE, OVERWRITE, OR USE CORRECTION FLUID OR TAPE ON THE ORIGINAL.

Copies of the electronic CRF (eCRF) will be provided for use as source data collection forms and maintained for recording data for each subject enrolled in the study. Data reported in the eCRF derived from source data collection forms should be consistent or the discrepancies should be explained.

The sponsor and/or its designee will provide guidance to the site principal investigators and other study personnel on making corrections to the data collection forms and eCRF.

13.2 Data Coordinating Center/Biostatistician Responsibilities

Data collection is the responsibility of the study personnel at the participating clinical study site under the supervision of the site principal investigator. During the study, the site principal investigator must maintain complete and accurate documentation for the study.

The data coordinating center for this study, the Emmes Company, will be responsible for data management, quality review, analysis, and reporting of the study data.

13.3 Data Capture Methods

Clinical data (including, but not limited to, AE/SAEs, concomitant medications, medical history, physical assessments, and clinical laboratory values) will be collected on data collection forms by study personnel then entered into eCRFs via a 21 CFR Part 11-compliant internet data entry system provided by the study data coordinating center. The data system includes password protection and internal quality checks, such as automatic range checks, to identify data that appear inconsistent, incomplete, or inaccurate.

13.4 Types of Data

Data for this trial will include clinical, safety, and outcome measures (e.g., clinical laboratory values).

13.5 Study Records Retention

Study records and reports including, but not limited to, eCRFs, source documents, ICFs, laboratory test results, and study drug disposition records will be retained for 2 years after a marketing application is approved for the study product for the indication for which it is being investigated; or, if no application is to be filed or if the application is not approved for the study product, until 2 years after the investigation is discontinued and the FDA has been notified. These documents will be retained for a longer period, however, if required by local regulations. ICFs for future use will be maintained as long as the sample/specimen exists.

No records will be destroyed without the written consent of the sponsor. It is the responsibility of the sponsor to inform the site principal investigator when these documents no longer need to be retained.

14 CLINICAL MONITORING

Site monitoring is conducted to ensure that the human subjects' protections, study and laboratory procedures, study intervention administration, and data collection processes are of high quality and meet sponsor, ICH/GCP guidelines and applicable regulations, and that this trial is conducted in accordance with the protocol, protocol-specific MOP and applicable sponsor standard operating procedures. DMID, the sponsoring agency, or its designee will conduct site-monitoring visits as detailed in the clinical monitoring plan.

Site visits will be made at standard intervals as defined by DMID and may be made more frequently as directed by DMID. Monitoring visits will include, but are not limited to, review of regulatory files, accountability records, eCRFs, informed consent forms, medical and laboratory reports, and protocol and GCP compliance. Site monitors will have access to each participating site, study personnel, and all study documentation according to the DMID-approved site monitoring plan. Study monitors will meet with site principal investigators to discuss any problems and actions to be taken, and will document site visit findings and discussions.

15 PUBLICATION POLICY

Following completion of the study, the lead Principal Investigator is expected to publish the results of this research in a scientific journal. All investigators funded by the NIH must submit or have submitted for them to the National Library of Medicine's PubMed Central (http://www.ncbi.nlm.nih.gov/pmc/) an electronic version of their final, peer-reviewed manuscripts upon acceptance for publication, to be made publicly available no later than 12 months after the official date of publication. The NIH Public Access Policy ensures the public has access to the published results of NIH funded research. It requires investigators to submit final peer-reviewed journal manuscripts that arise from NIH funds to the digital archive PubMed Central upon acceptance for publication. Further, the policy stipulates that these papers must be accessible to the public on PubMed Central no later than 12 months after publication.

Refer to:

- NIH Public Access Policy, http://publicaccess.nih.gov/
- NIH Office of Extramural Research (OER) Grants and Funding, http://grants.nih.gov/grants/oer.htm

As of January 2018, all clinical trials supported by the NIH must be registered on ClinicalTrials.gov, no later than 21 days after the enrollment of the first subject. Results of all clinical trials supported by the NIH, generally, need to be submitted no later than 12 months following the primary completion date. A delay of up to 2 years is available for trials that meet certain criteria and have applied for certification of delayed posting.

As part of the result posting a copy of this protocol (and its amendments) and a copy of the Statistical Analysis Plan will be posted on ClincialTrials.gov.

For this trial the responsible party is NIH/NIAID/DMID which will register the trial and post results.

The responsible party does not plan to request certification of delayed posting.

Refer to:

- Public Law 110-85, Section 801, Clinical Trial Databases
- 42CFR11
- NIH NOT-OD-16-149

16 LITERATURE REFERENCES

1. Van Hal SJ, Jensen SO, Vaska VL, Espedido BA, Paterson DL, Gosbell IB. Predictors of mortality in Staphylococcus aureus Bacteremia. *Clin Microbiol Rev.* 2012;25(2):362-386.

2. Tong SY, Davis JS, Eichenberger E, Holland TL, Fowler VG, Jr. Staphylococcus aureus infections: epidemiology, pathophysiology, clinical manifestations, and management. *Clin Microbiol Rev.* 2015;28(3):603-661.

3. Thuny F, Di Salvo G, Belliard O, et al. Risk of embolism and death in infective endocarditis: prognostic value of echocardiography: a prospective multicenter study. *Circulation.* 2005;112(1):69-75.

4. Murdoch DR, Corey GR, Hoen B, et al. Clinical presentation, etiology, and outcome of infective endocarditis in the 21st century: the International Collaboration on Endocarditis-Prospective Cohort Study. *Arch Intern Med.* 2009;169(5):463-473.

5. Selton-Suty C, Celard M, Le Moing V, et al. Preeminence of Staphylococcus aureus in infective endocarditis: a 1-year population-based survey. *Clin Infect Dis.* 2012;54(9):1230-1239.

6. Keller SC, Williams D, Rock C, Deol S, Trexler P, Cosgrove SE. A new frontier: Central line-associated bloodstream infection surveillance in home infusion therapy. *Am J Infect Control.* 2018;46(12):1419-1421.

7. Lefort A, Pavie J, Garry L, Chau F, Fantin B. Activities of dalbavancin in vitro and in a rabbit model of experimental endocarditis due to Staphylococcus aureus with or without reduced susceptibility to vancomycin and teicoplanin. *Antimicrob Agents Chemother.* 2004;48(3):1061-1064.

8. Candiani G, Abbondi M, Borgonovi M, Romano G, Parenti F. In-vitro and in-vivo antibacterial activity of BI 397, a new semi-synthetic glycopeptide antibiotic. *J Antimicrob Chemother.* 1999;44(2):179-192.

9. Raad I, Darouiche R, Vazquez J, et al. Efficacy and safety of weekly dalbavancin therapy for catheter-related bloodstream infection caused by gram-positive pathogens. *Clin Infect Dis.* 2005;40(3):374-380.

10. Dunne MW, Puttagunta S, Giordano P, Krievins D, Zelasky M, Baldassarre J. A Randomized Clinical Trial of Single-Dose Versus Weekly Dalbavancin for Treatment of Acute Bacterial Skin and Skin Structure Infection. *Clinical infectious diseases : an official publication of the Infectious Diseases Society of America.* 2016;62(5):545-551.

11. Seltzer E, Dorr MB, Goldstein BP, Perry M, Dowell JA, Henkel T. Once-weekly dalbavancin versus standard-of-care antimicrobial regimens for treatment of skin and soft-tissue infections. *Clinical infectious diseases : an official publication of the Infectious Diseases Society of America.* 2003;37(10):1298-1303.

12. Jauregui LE, Babazadeh S, Seltzer E, et al. Randomized, double-blind comparison of once-weekly dalbavancin versus twice-daily linezolid therapy for the treatment of complicated skin and skin structure infections. *Clinical infectious diseases : an official publication of the Infectious Diseases Society of America.* 2005;41(10):1407-1415.

13. Boucher HW, Wilcox M, Talbot GH, Puttagunta S, Das AF, Dunne MW. Once-weekly dalbavancin versus daily conventional therapy for skin infection. *N Engl J Med.* 2014;370(23):2169-2179.

14. Holland TL, Raad I, Boucher HW, et al. Effect of Algorithm-Based Therapy vs Usual Care on Clinical Success and Serious Adverse Events in Patients with Staphylococcal Bacteremia: A Randomized Clinical Trial. *Jama.* 2018;320(12):1249-1258.

15. Baddour LM, Wilson WR, Bayer AS, et al. Infective Endocarditis in Adults: Diagnosis, Antimicrobial Therapy, and Management of Complications: A Scientific Statement for Healthcare Professionals from the American Heart Association. *Circulation.* 2015;132(15):1435-1486.

16. Corey GR. Staphylococcus aureus bloodstream infections: definitions and treatment. *Clin Infect Dis.* 2009;48 Suppl 4:S254-259.

17. Lepak A, Marchillo K, VanHecker J, Andes D. Impact of Glycopeptide Resistance in Staphylococcus aureus on the Dalbavancin In Vivo Pharmacodynamic Target. *Antimicrob Agents Chemother.* 2015;59(12):7833-7836.

18. Andes D, Craig WA. In vivo pharmacodynamic activity of the glycopeptide dalbavancin. *Antimicrob Agents Chemother.* 2007;51(5):1633-1642.

19. Marbury T, Dowell JA, Seltzer E, Buckwalter M. Pharmacokinetics of dalbavancin in patients with renal or hepatic impairment. *J Clin Pharmacol.* 2009;49(4):465-476.

20. Dorr MB, Jabes D, Cavaleri M, et al. Human pharmacokinetics and rationale for once-weekly dosing of dalbavancin, a semi-synthetic glycopeptide. *J Antimicrob Chemother.* 2005;55 Suppl 2:ii25-30.

21. Carrothers TJ, Chittenden JT, Critchley I. Dalbavancin Population Pharmacokinetic Modeling and Target Attainment Analysis. *Clin Pharmacol Drug Dev.* 2020;9(1):21-

22. Evans SR, Rubin D, Follmann D, et al. Desirability of Outcome Ranking (DOOR) and Response Adjusted for Duration of Antibiotic Risk (RADAR). *Clin Infect Dis.* 2015;61(5):800-806.

23. Dalvance (dalbavancin) [product insert]. Madison, NJ: Allergan USA, Inc.; 2018.

24. Fowler VG, Jr., Boucher HW, Corey GR, et al. Daptomycin versus standard therapy for bacteremia and endocarditis caused by Staphylococcus aureus. *N Engl J Med.* 2006;355(7):653-665.

25. Hamed K, Engelhardt M, Jones ME, et al. Ceftobiprole versus daptomycin in Staphylococcus aureus bacteremia: a novel protocol for a double-blind, Phase III trial. *Future Microbiol.* 2020;15(1):35-48.

26. Lachin JL. Introduction to Sample Size Determination and Power Analysis for Clinical Trials. Controlled Clinical Trials 198; 2:93-113

27. King HA, Doernberg SB, Miller J, et al. Patients' experiences with Staphylococcus aureus and Gram-negative bacterial bloodstream infections: A qualitative descriptive study and concept elicitation phase to inform measurement of patient-reported quality of life. *Clin Infect Dis* 2020; Accepted, available epub ahead of print at: https://doi.org/10.1093/cid/ciaa611

28. DALVANCE™ Dalbavancin for Injection [Investigator's Brochure]. Madison, NJ: Allergan USA, Inc.; 2020 August.

17 APPENDICES

Appendix A. Schedule of Events

	**Induction Period**	**Screening/ Enrollment**	**Open Label Treatment Period**				**Post-treatment Follow-up Period**		
	Visit 0 (Pre-Screening, Day -10 to Day 1)	Visit 1 (Day –1 to Day 1)	Visit 2 (Baseline, Day 1)	Visit 3 (Day 8 ± 1 day)	Visit 4 (Day 22 ± 2 days)	Visit 5 (Day 42 ± 3 days)	Visit 6 (TOC, Day 70 ± 7 days)[a]	ET[b]	Visit 7 (Day 180 ± 14 days, Osteomyelitis group)[a]
Informed Consent		X							
Dalbavancin[c]			X	X					
Standard of care antibiotic therapy[c]	X	X	X (Duration 28-56 days)						
Medical history[d]		X	X			X	X	X	
Medication history[e]		X							
Randomization			X						
AEs/AESIs/SAEs			X	X	X	X	X	X	
Hematology and serum chemistry blood sampling[f]		X		X[g]	X	X			
Coagulation lab tests[f]		X							

	Induction Period	Screening/ Enrollment	Open Label Treatment Period				Post-treatment Follow-up Period		
	Visit 0 (Pre-Screening, Day -10 to Day 1)	Visit 1 (Day –1 to Day 1)	Visit 2 (Baseline, Day 1)	Visit 3 (Day 8 ± 1 day)	Visit 4 (Day 22 ± 2 days)	Visit 5 (Day 42 ± 3 days)	Visit 6 (TOC, Day 70 ± 7 days)[a]	ET[b]	Visit 7 (Day 180 ± 14 days, Osteomyelitis group)[a]
Pregnancy test[h]		X							
PK sampling[i]			X	X	X	X	X	X	
Vital signs[j]		X	X	X[k]	X	X	X	X	X
Physical examination[l]		X	X	X	X	X	X	X	X
Echocardiogram[m]		X							
Investigator assessment of efficacy						X	X	X	X
Concomitant medications[n]		X	X	X	X	X	X	X	X
Concomitant nondrug interventions		X	X	X	X	X	X	X	X
QoL assessment[o]			X	X	X	X	X	X	X

AEs = adverse events; AESIs = adverse events of special interest; eCRF = electronic case report form; ET = Early Termination; PK = pharmacokinetic; SAE = serious adverse events

[a]Telephone visit permissible if in-person visit is not possible; in person visit still preferred.

[b]Patients who prematurely discontinue therapy should have an ET Visit within 72 hours.

[c]All subjects will be receiving standard of care prior to randomization; after randomization, subjects will receive either dalbavancin or standard of care based on their assigned treatment group.

[d]Includes targeted/pertinent medical and surgical history only

[e]A complete medication history will be completed through 30 days prior to ICF signing; an extended 60 day review will be conducted for dalbavancin and oritavancin given the long half-lives of both drugs.

[f]Visit 1 hematology, coagulation lab tests (PT, PTT, and/or INR) and serum chemistry will be done in order to qualify the patient for the study, if not already collected per standard of care within 48 hours prior to randomization.

[g]A serum creatinine assessment will be required within the 72 hours prior to the 2nd (Day 8) dalbavancin dose. Whether a serum creatinine must be repeated on Day 8 will be at the discretion of the site investigator based upon stability of the serum creatinine in the preceding 72 hours and whether the serum creatinine is near the threshold where dose adjustment would be necessary (e.g., near 30 mL/min).

[h]Women of childbearing potential only, if not already performed (see Appendix B, Definitions); ensure test is negative within 48 hours before randomization. If the serum test results cannot be obtained before randomization, a urine pregnancy test may be used for enrollment.

[i]Dalbavancin PK samples will be drawn only for subjects receiving dalbvacancin. PK samples will be drawn at Day 1 prior to dose, at end of infusion ± 10 minutes, 6 ± 2 hours post end of dose, 12 ± 4 hours post end of dose, 24 ± 6 hours post end of dose), Day 8 (prior to 2nd dose), Day 22 ± 2 days (at time of clinic visit), day 42 ± 3 days, day 70 ± 7 days, and with any ET visit. Each sample must be accompanied by draw time and date.

[j]Vital signs include blood pressure, respiration rate, pulse rate, and temperature.

[k]Day 8 vital signs not required for subjects receiving SOC antibiotics if discharge occurs prior to day 8.

[l]A physical examination (including general appearance, examination of head, eyes, ears, nose, throat, neck, skin, heart, lungs, abdomen, neurologic system, musculoskeletal system, extremities, height, and body weight) will be done at Screening (Visit 1). If height or weight is not obtainable (eg, patient is immobilized), use the last known or stated height and weight. At subsequent visits, targeted physical exams will focus on changes from prior exams and on the evaluation of newly reported symptoms.

[m]Transthoracic echocardiogram or, if clinically indicated, transesophageal echocardiogram to be performed (local laboratory), unless one has been performed as standard of care for this episode of bacteremia/endocarditis

[n]All concomitant medications from Screening (Visit 1) through Day 42 (± 3 days) (Visit 5) must be recorded in the patient's medical record and on the eCRFs. Between the Day 42 Visit and Day 70 Visit, all concomitant medications for an AE or any antibacterial therapy should be recorded in the patient's medical record and on the eCRF.

[o]QoL assessments include the ARLG Bloodstream Infection QoL Measure (Appendix C), the EQ-5D-5L (https://euroqol.org/eq-5d-instruments/sample-demo/), and the PROMIS Global Health Short Form (http://www.healthmeasures.net/administrator/components/com_instruments/uploads/Global%20Health%20Scale%20v1.2%2008.22.2016.pdf).

Appendix B. Definitions

Childbearing Potential: a woman is considered of childbearing potential unless post-menopausal [≥1 year of spontaneous amenorrhea] or permanently surgically sterilized [bilateral oophorectomy, salpingectomy, hysterectomy, tubal ligation].

Effective Contraception: Must include at least one of the following: non-male sexual relationships only, abstinence from sexual intercourse with a male partner, monogamous relationship with a vasectomized partner who has been vasectomized ≥180 days before the subject received the first dose of study drug, barrier methods such as condoms or diaphragms, effective intrauterine devices (IUDs), NuvaRing®, or licensed hormonal methods such as implants, injectables, or oral contraceptives.

Uncomplicated *Staphylococcus aureus* Bacteremia: defined as all of the following: exclusion of endocarditis by echocardiography; catheter-associated bacteremia and removal of catheter; no implanted prostheses; follow-up blood cultures drawn within 48 hours after initial set that do not grow screening pathogen and all follow-up blood cultures thereafter do not grow the screening pathogen; defervescence within 72 hours of initiating effective therapy; and no evidence of metastatic sites of infection.

Complicated *S. aureus* Bacteremia:

The following are examples of complicated *S. aureus* bacteremia. This is not an exhaustive list. If a patient's infection does not meet the definition of uncomplicated bacteremia above, then that patient has complicated bacteremia.

Positive follow-up blood cultures: blood cultures positive for *S. aureus* drawn at least 24 hours after the initial qualifying blood culture

Persistent fever: oral temperature ≥38.0C for >72 hours after the initial positive blood culture for *S. aureus*

Endocarditis:

These criteria have been adjusted to be specific to *S. aureus* bacteremia, i.e. microbiological criteria related to organisms other than *S. aureus* have been removed. In addition, references to prosthetic valve infections have been removed, as these patients will be excluded from the study.

According to modified Duke Criteria, diagnosis of IE can be definite, possible, or rejected. A diagnosis of IE is **definite** if either the following pathological or clinical criteria are met:

Pathologic criteria:

- pathologic lesions: vegetation or intracardiac abscess demonstrating active endocarditis on histology, or
- microorganism: demonstrated by culture or histology of a vegetation or intracardiac abscess

One of these combinations of clinical criteria (see definitions below):

- two major clinical criteria
- one major and three minor criteria
- five minor criteria

Diagnosis of IE is **possible** if one of the following combinations of clinical criteria (see definitions below) are met:

- one major and one minor criteria
- three minor criteria are fulfilled

Diagnosis of IE is **rejected** if one of the following criteria are met:

- a firm alternate diagnosis is made
- resolution of clinical manifestations after ≤ 4 days of antibacterial treatment
- no pathological evidence of IE is found at surgery or autopsy after antibacterial treatment therapy for ≤ 4 days
- clinical criteria for possible or definite IE are not met

Major criteria for the diagnosis of infective endocarditis:

1. Positive blood culture with *S. aureus* from two separate blood cultures
2. Evidence of endocardial involvement with positive echocardiogram defined as

- oscillating intracardiac mass on valve or supporting structures, in the path of regurgitant jets in the absence of an alternative anatomic explanation
- abscess
- new valvular regurgitation (worsening or changing of preexisting murmur not sufficient)

Minor criteria for the diagnosis of infective endocarditis:

1. Predisposing factor: intravenous drug use or presence of a predisposing heart condition (a valve lesion associated with significant regurgitation or turbulence of blood flow)
2. Fever ≥38°C (100.4 °F)
3. Vascular phenomena: major arterial emboli, septic pulmonary infarcts, mycotic aneurysm, intracranial hemorrhage, conjunctival hemorrhages or Janeway lesions
4. Immunological phenomena: glomerulonephritis, Osler's nodes, Roth's spots, Rheumatoid factor
5. A single positive blood culture with *S. aureus*

Visceral Abscess (e.g. liver, spleen, kidney, etc.) – either of the following:

- Abscess visualized on radiographic exam, or
- Isolation of *S. aureus* from culture of abscess contents

Pleuropulmonary Infection – one of the following:

- Pulmonary infiltrate consistent with pneumonia in patients with *S. aureus* bacteremia
- *S. aureus* in pleural fluid, needle aspirate biopsy, or growth from bronchoalveolar lavage (BAL)/protected specimen brush (PSB)
- Clinical evidence of pneumonia (e.g. increased O_2, increased respiratory rate, cough, mechanical ventilation, purulent sputum, etc.)

Osteomyelitis - Either of the following:

- Radiographic evidence of bone lesion consistent with osteomyelitis/discitis; or
- Culture of bone yields *S. aureus*.

Pyomyositis – Either of the following:

- Radiographic evidence consistent with pyomyositis; or
- Culture of abscess contents yields *S. aureus*.

Septic Arthritis – Either of the following:

- *S. aureus* in culture of synovial fluid; or
- Positive Gram stain of synovial fluid for Gram positive cocci AND synovial fluid cell count ≥20,000 WBC/mL without alternate explanation

Septic Thrombophlebitis – Either of the following:

- Palpable venous cord
- Evidence of thrombosis on radiologic exam

Relapsing Bacteremia: Relapsing bacteremia is defined as a *S. aureus* that:

- Represents the same bacterial strain as the Baseline Infecting Pathogen (based on bacterial speciation, antibiotic susceptibility testing, and/or genotyping tests, as appropriate);
- Is documented by a blood culture yielding *S. aureus* obtained after randomization

Source Control Procedure: a procedure intended to treat *S. aureus* infection. Examples include, but are not limited to, surgical debridement or amputations, drainage of infected spaces, and removal of prosthetic material

Vascular Access Procedures: insertion or removal of vascular catheters

Microbiologic Success: no post-randomization growth of the baseline pathogen from blood cultures or another sterile body site

Blood Culture Contaminant: the following organisms may be considered a contaminant if grown from only one blood culture (either alone or in addition to *S. aureus* isolated from the same blood culture): *Cutibacterium* species, *Micrococcus* species, viridans-group streptococcus, coagulase-negative staphylococci, *Corynebacterium* species, *Bacillus* species other than *B. anthracis*, or enterococcus species.

Appendix C. ARLG Bloodstream Infection Quality of Life Measure

**Please respond to each item by marking one box per row.**							
**Global**			**Excellent**	**Very Good**	**Good**	**Fair**	**Poor**
Global01	1	In general, would you say your health is:	☐	☐	☐	☐	☐
Global02	2	In general, would you say your quality of life is:	☐	☐	☐	☐	☐
**Thinking of your bloodstream infection, please answer the following questions to best capture your experiences.**							
**Fatigue**			**Not at all**	**A little bit**	**Somewhat**	**Quite a bit**	**Very much**
HI7	3	During the past 7 days, I feel fatigued	☐	☐	☐	☐	☐
AN3	4	During the past 7 days, I have trouble starting things because I am tired	☐	☐	☐	☐	☐
FATEXP41	5	In the past 7 days, how run-down did you feel on average?	☐	☐	☐	☐	☐
FATEXP40	6	In the past 7 days, how fatigued were you on average?	☐	☐	☐	☐	☐
**Gastrointestinal Nausea and Vomiting**			**Never**	**Rarely**	**Sometimes**	**Often**	**Always**
GISX49	7	In the past 7 days, how often did you have nausea—that is, a feeling	☐	☐	☐	☐	☐

		like you could vomit? (If never, skip to 9)					
GISX52	8	In the past 7 days, how often did you know that you would have nausea before it happened?	☐	☐	☐	☐	☐
GISX55	9	In the past 7 days, how often did you have a poor appetite?	☐	☐	☐	☐	☐
			**Never**	**One day**	**2-6 days**	**Once a day**	**More than once a day**
GISX59	10	In the past 7 days, how often did you throw up or vomit?	☐	☐	☐	☐	☐
**Pain Intensity**			**Had no pain**	**Mild**	**Moderate**	**Severe**	**Very severe**
PAINQU6	11	In the past 7 days, how intense was your pain at its worst?	☐	☐	☐	☐	☐
PAINQU8	12	In the past 7 days, how intense was your average pain?	☐	☐	☐	☐	☐
			**No pain**	**Mild**	**Moderate**	**Severe**	**Very severe**
PAINQU21	13	What is your level of pain right now?	☐	☐	☐	☐	☐
**Sleep Disturbance**			**Very poor**	**Poor**	**Fair**	**Good**	**Very Good**
Sleep109	14	In the past 7 days, my sleep quality was	☐	☐	☐	☐	☐

			**Not at all**	**A little bit**	**Somewhat**	**Quite a bit**	**Very much**
Sleep116	15	In the past 7 days, my sleep was refreshing	☐	☐	☐	☐	☐
Sleep20	16	In the past 7 days, I had a problem with my sleep	☐	☐	☐	☐	☐
Sleep44	17	In the past 7 days, I had difficulty falling asleep	☐	☐	☐	☐	☐
**Emotional Distress - Depression**			**Never**	**Rarely**	**Sometimes**	**Often**	**Always**
EDDEP04	18	In the past 7 days, I felt worthless	☐	☐	☐	☐	☐
EDDEP06	19	In the past 7 days, I felt helpless	☐	☐	☐	☐	☐
EDDEP29	20	In the past 7 days, I felt depressed	☐	☐	☐	☐	☐
EDDEP41	21	In the past 7 days, I felt hopeless	☐	☐	☐	☐	☐
**Emotional Distress - Anxiety**			**Never**	**Rarely**	**Sometimes**	**Often**	**Always**
EDANX01	22	In the past 7 days, I felt fearful	☐	☐	☐	☐	☐
EDANX40	23	In the past 7 days, I found it hard to focus on anything other than my anxiety	☐	☐	☐	☐	☐
EDANX41	24	In the past 7 days, my worries overwhelmed me	☐	☐	☐	☐	☐
EDANX53	25	In the past 7 days, I felt uneasy	☐	☐	☐	☐	☐

**Cognitive Function - Abilities**			**Not at all**	**A little bit**	**Somewhat**	**Quite a bit**	**Very much**
PC43_2r	26	In the past 7 days, my mind has been as sharp as usual	☐	☐	☐	☐	☐
PC44_2r	27	In the past 7 days, my memory has been as good as usual	☐	☐	☐	☐	☐
PC45_2r	28	In the past 7 days, my thinking has been as fast as usual	☐	☐	☐	☐	☐
PC47_2r	29	In the past 7 days, I have been able to keep track of what I am doing, even if I am interrupted	☐	☐	☐	☐	☐
**Physical Function**			**Without any difficulty**	**With a little difficulty**	**With some difficulty**	**With much difficulty**	**Unable to do**
PFA11	30	Are you able to do chores such as vacuuming or yard work?	☐	☐	☐	☐	☐
PFA21	31	Are you able to go up and down stairs at a normal pace?	☐	☐	☐	☐	☐
PFA23	32	Are you able to go for a walk of at least 15 minutes?	☐	☐	☐	☐	☐
PFA53	33	Are you able to run errands and shop?	☐	☐	☐	☐	☐
			**Not at all**	**Very little**	**Somewhat**	**Quite a lot**	**Cannot do**

PFC12	34	Does your health now limit you in doing two hours of physical labor?	☐	☐	☐	☐	☐
PFB1	35	Does your health now limit you in doing moderate work around the house like vacuuming, sweeping floors or carrying in groceries?	☐	☐	☐	☐	☐
**Ability to Participate in Social Roles and Activities**			**Never**	**Rarely**	**Sometimes**	**Often**	**Always**
SRPPER11 _CaPS	36	I have trouble doing all of my regular leisure activities with others	☐	☐	☐	☐	☐
SRPPER18 _CaPS	37	I have trouble doing all of the family activities that I want to do	☐	☐	☐	☐	☐
SRPPER23 _CaPS	38	I have trouble doing all of my usual work (include work at home)	☐	☐	☐	☐	☐
SRPPER46 CaPS	39	I have trouble doing all of the activities with friends that I want to do	☐	☐	☐	☐	☐
			**Excellent**	**Very Good**	**Good**	**Fair**	**Poor**
	40	Because of your bloodstream infection, would you say your health is..	☐	☐	☐	☐	☐

	41	Because of your bloodstream infection, would you say your quality of life is..	☐	☐	☐	☐	☐

20-0002 DOTS Protocol Summary of Changes v1.0 to v2.0

Section	Previous Version (v1.0) Language	Change (v2.0) Language	Rationale
All (Header)	Version 1.0	Version 2.0	Update
All (Header)	6 August 2020	19 October 2020	Update
Title Page	DMID Clinical Project Manager: [redacted]	DMID Clinical Project Manager: [redacted]	Update
Title Page	Draft or Version Number: v1.0	Draft or Version Number: v2.0	Update
Title Page	6 August 2020	19 October 2020	Update
**Protocol Summary** Study Objectives:	• Examine the association between individualized plasma concentration profiles and late recurrence risk among the subset of patients with osteomyelitis and a 6 month follow-up visit	• Examine the association between individualized plasma concentration profiles and late recurrence risk among the subset of patients with vertebral osteomyelitis and a 6 month follow-up visit	Clarification
**Protocol Summary** Duration of Individual Subject Participation:	Approximately 70 ± 7 days, with a late post-treatment follow-up visit for the subset of patients with osteomyelitis at 6 months	Approximately 70 ± 7 days, with a late post-treatment follow-up visit for the subset of patients with vertebral osteomyelitis at 6 months	Clarification
**Table 1: Treatment Arms** Standard of Care[a]	• Methicillin-sensitive Staphylococcus aureus (MSSA): nafcillin (2 g IV q4h × 4-6 weeks) OR oxacillin (2 g IV Q4h x 4-6 weeks) OR cefazolin (2 g IV q8h × 4-6 weeks)	• Methicillin-sensitive Staphylococcus aureus (MSSA): nafcillin (2 g IV q4h × 4-6 weeks)[b] OR oxacillin (2 g IV Q4h x 4-6 weeks)[b] OR cefazolin (2 g IV q8h × 4-6 weeks)	Clarification
**Table 1: Treatment Arms** Footnote	None	[b]As applicable per site standard of care, nafcillin and oxacillin may be administered at an equivalent dose via continuous IV infusion (e.g., 12g/24h IV continuous)	Clarification
**1 Key Roles** DMID Clinical	[redacted]	[redacted]	Update

Section	Previous Version (v1.0) Language	Change (v2.0) Language	Rationale
Project Manager:	Phone: [redacted]<br>Email: [redacted]	Phone: [redacted]<br>Email: [redacted]	
**2.3.1.1 Dalbavancin**	Gastrointestinal: Nausea (<4%) and diarrhea (<4%) have been infrequently reported with dalbavancin receipt (<4%).	Gastrointestinal: Nausea (4.7%) and diarrhea (<4%) have been infrequently reported with dalbavancin receipt (<4%).	Updated to match package insert and Investigator's Brochure
**3.2.3 Exploratory**	10. Examine the association between individualized plasma concentration profiles and late recurrence risk among the subset of patients with osteomyelitis and a 6-month follow-up visit	10. Examine the association between individualized plasma concentration profiles and late recurrence risk among the subset of patients with vertebral osteomyelitis and a 6-month follow-up visit	Clarification
**3.3.1 Primary**	For example, a patient who has a new metastatic focus on infection diagnosed after randomization, but who subsequently completes treatment and is felt to be cured at Day 70 would be considered a Clinical Success (and the Infectious Complication would result in a lower DOOR).	For example, a patient who has a new metastatic focus of infection diagnosed after randomization, but who subsequently completes treatment and is felt to be cured at Day 70 would be considered a Clinical Success (and the Infectious Complication would result in a lower DOOR).	Typographical error
**3.3.2 Secondary**	The **secondary efficacy outcome** is as follows.<br>• Clinical efficacy, defined as none of: 1) Clinical failure; 2) Infectious complications; 3) Mortality<br><br>The **secondary safety outcome** is as follows.<br>• Proportion of patients who have either 1) an SAE; or 2) an AE leading to study drug discontinuation	The **secondary efficacy outcome** is as follows.<br>• Clinical efficacy, defined as none of: 1) Clinical failure; 2) Infectious complications; 3) All-cause mortality<br><br>The **secondary safety outcome** is as follows.<br>• Proportion of patients who have either 1) an SAE; or 2) an AE leading to study drug discontinuation	Clarification based on FDA feedback

Section	Previous Version (v1.0) Language	Change (v2.0) Language	Rationale
	Each component of the DOOR will also be examined separately:<br>• clinical success<br>• infectious complications<br>• SAEs<br>• AEs leading to study drug discontinuation<br>• mortality	Each component of the DOOR will also be examined separately:<br>• clinical success<br>• infectious complications<br>• SAEs<br>• AEs leading to study drug discontinuation<br>• all-cause mortality	
**3.3.3 Exploratory**	10. Late recurrence within the osteomyelitis population will be defined by the presence of the following up to 6 months after randomization: progressive imaging changes along with isolation of S. aureus from blood, bone biopsy, associated fluid aspiration, or operative tissue culture.	10. Late recurrence within the vertebral osteomyelitis population will be defined by the presence of the following up to 6 months after randomization: progressive imaging changes along with isolation of S. aureus from blood, bone biopsy, associated fluid aspiration, or operative tissue culture.	Clarification
**4.1 Study Product Description**	Please refer to the package insert for further details.	Please refer to the package insert and/or Investigator's Brochure for further details.	Update
**4.1.1 Formulation, Packaging, and Labeling**	The SOC antibiotics (Cefazolin, nafcillin, oxacillin, vancomycin, and daptomycin) will be prepared and labeled in accordance with the clinical site's pharmacy standard operating procedures (SOPs).	The SOC antibiotics (Cefazolin, nafcillin, oxacillin, vancomycin, and daptomycin) will be prepared and labeled in accordance with the clinical site pharmacy's standard operating procedures (SOPs).	Grammar
**4.3 Dosage/Regimen, Preparation, Dispensing and Administrati**	Standard of Care Group<br>Subjects randomized to the standard of care antibiotic therapy treatment group will receive an antibiotic considered standard of care, generally for a duration of 4 to 6 weeks (but up to a maximum	Standard of Care Group<br>Subjects randomized to the standard of care antibiotic therapy treatment group will receive an antibiotic considered standard of care, generally for a duration of 4 to 6 weeks (but up to a maximum	Clarification

Section	Previous Version (v1.0) Language	Change (v2.0) Language	Rationale
**on of Study Intervention/ Study Product**	of 8 weeks, which may be standard practice for osteomyelitis at some sites) and based on the results of antibiotic susceptibility testing for baseline pathogen.	of 8 weeks, which may be standard practice for vertebral osteomyelitis at some sites) and based on the results of antibiotic susceptibility testing for baseline pathogen.	
**Table 5: Acceptable SOC Antibiotics** MSSA[a]	nafcillin (2 g IV q4h × 4-6 weeks[b])<br>OR<br>oxacillin (2 g IV Q4h x 4-6 weeks[b])<br>OR<br>cefazolin (2 g IV q8h × 4-6 weeks[b])	nafcillin (2 g IV q4h × 4-6 weeks[b])[d]<br>OR<br>oxacillin (2 g IV Q4h x 4-6 weeks[b])[d]<br>OR<br>cefazolin (2 g IV q8h × 4-6 weeks[b])	Clarification
**Table 5: Acceptable SOC Antibiotics** Footnote	None	[d]As applicable per site standard of care, nafcillin and oxacillin may be administered at an equivalent dose via continuous IV infusion (e.g., 12g/24h IV continuous).	Clarification
**5.1.2 Subject Exclusion Criteria**	1. Uncomplicated bacteremia[2]Appendix B.	2. Uncomplicated bacteremia[2](Appendix B).	Formatting
**6.2 Screening: Visit 1 (Day - 1 to Day 1)**	• Review and record relevant medical events that occurred during the induction period. Examples include source control procedures [see Definitions (Appendix B)], vascular access procedures [see Definitions (Appendix B)], and complications of pre-randomization antibiotic therapy.Collect blood samples for hematology, serum chemistry laboratory assessments, and coagulation lab tests (PT/PTT and /or INR), if not already complete per standard of care within 48 hours	• Review and record relevant medical events that occurred during the induction period. Examples include source control procedures [see Definitions (Appendix B)], vascular access procedures [see Definitions (Appendix B)], and complications of pre-randomization antibiotic therapy.<br>• Collect blood samples for hematology, serum chemistry laboratory assessments, and coagulation lab tests (PT/PTT and	Formatting

Section	Previous Version (v1.0) Language	Change (v2.0) Language	Rationale
	prior to randomization (see Section 7.2.1)	/or INR), if not already complete per standard of care within 48 hours prior to randomization (see Section 7.2.1)	
**6.3.1 Baseline (Randomization): Visit 2 (Day 1)**	• Assess QoLs.	• Collect QoL data.	Consistency
**6.3.1 Baseline (Randomization): Visit 2 (Day 1)**	• For subjects receiving dalbavancin, collect blood samples for pharmacokinetic parameters prior to first dose, at end of infusion (+/-10 minutes), 6 (+/- 2) hours post end of dose, 12 hours (+/- 4) hours post end of dose, 24 (+/- 6) hours post end of dose, with documented draw time and date for each sample.	• For subjects receiving dalbavancin, collect blood samples for pharmacokinetic parameters prior to first dose, at end of infusion (+ 10 minutes), 6 (+/- 2) hours post end of dose, 12 hours (+/- 4) hours post end of dose, 24 (+/- 6) hours post end of dose, with documented draw time and date for each sample.	Updated PK collection window for the end of infusion time point because the PK sample should not be collected prior to completion of infusion.
**6.3.1 Baseline (Randomization): Visit 2 (Day 1)**	• Review and record AEs after study product administration	• Review and record AEs/SAEs/AESIs after study product administration	Consistency
**6.3.2 Visit 3 (Day 8 ± 1 day)**	• Review and record AEs	• Review and record AEs/SAEs/AESIs	Consistency
**6.3.2 Visit 3 (Day 8 ± 1 day)**	• None	• For subjects receiving dalbavancin, a serum creatinine assessment is required within 72 hours prior to the administration of the second dalbavancin dose. Whether a serum creatinine must be repeated on Day 8 will be at the discretion of the site investigator based upon stability of the serum creatinine in the preceding 72 hours and whether the	Consistency with Appendix A

Section	Previous Version (v1.0) Language	Change (v2.0) Language	Rationale
		serum creatinine is near the threshold where dose adjustment would be necessary (e.g., near 30 mL/min).	
**6.3.2 Visit 3 (Day 8 ± 1 day)**	• None	• Review and record any concomitant nondrug interventions (e.g. procedures performed with a goal of source control).	Consistency with Appendix A
**6.3.3 Visit 4 (Day 22 ± 2 days)**	• Review and record AEs	• Review and record AEs/SAEs/AESIs	Consistency
**6.3.4 Visit 5 (Day 42 ± 3 days)**	• Review and record AEs	• Review and record AEs/SAEs/AESIs	Consistency
**6.3.5 Test of Cure: Visit 6 (Day 70 ± 7 days)**	It is preferred that this visit be performed in-person, however a telephone or telemedicine visit is an acceptable alternative.	It is preferred that this visit be performed in-person, however a telephone visit is an acceptable alternative.	Consistency
**6.3.5 Test of Cure: Visit 6 (Day 70 ± 7 days)**	• Review and record AEs	• Review and record AEs/SAEs/AESIs	Consistency
**6.3.8 Early Termination Visit**	• Review and record AEs	• Review and record AEs/SAEs/AESIs	Consistency
**6.4 Unscheduled Study Visits**	• Review and record AEs	• Review and record AEs/SAEs/AESIs	Consistency
**7.1.2 Assessment of Concomitant Medications/ Treatments**	Any medication taken by the subject, other than study drugs, is considered concomitant medication. All concomitant medications from Screening (Visit 1) through Day 70	Any systemic medication taken by the subject, other than study drugs, is considered a concomitant medication. Topical medications including eye drops, ear drops, or dermatologic treatments do not need	Clarification

Section	Previous Version (v1.0) Language	Change (v2.0) Language	Rationale
**Other Than Study Product**	± 7 days (Visit 6) or, for subjects with vertebral osteomyelitis, Day 180 ± 14 days (Visit 7) must be recorded in the subject's medical record and on the eCRFs.<br><br>At each visit the investigator will obtain information on any therapeutic interventions (e.g., drug and nondrug therapy or surgery) provided. The use of any other investigational drug is prohibited and subjects may not participate in any other studies involving marketed products concomitantly while in this study.<br><br>The use of other (non-antibacterial) medications should be limited to those essential for the care of the subject. All medications required by the subject to manage underlying illnesses, other than infection under study, and any drugs that may be required for emergency treatments must be recorded on the eCRF.	to be recorded in the eCRFs. All concomitant systemic medications from Screening (Visit 1) through Day 70 ± 7 days (Visit 6) must be recorded in the subject's medical record and on the eCRFs. After Visit 6, for subjects with vertebral osteomyelitis, who are followed until Day 180 ± 14 days (Visit 7), the only concomitant medications that must be recorded are new antibiotics that are prescribed for the treatment of osteomyelitis.<br><br>At each visit the investigator will obtain information on any therapeutic interventions (e.g., drug and nondrug therapy or surgery) provided. Subjects may not participate in any other antibiotic treatment trials or interventional studies involving non-FDA approved investigational products concomitantly while in this study.	
**7.2.2 Research Assays**	• Dalbavancin Concentration Measurements: Venous blood samples for PK analyses will be collected at Visit 2 (Day 1), Visit 3 (Day 8 ± 1 day),Visit 4 (Day 22 ± 2 days), Visit 5 (Day 42 ± 3 days), Visit 6 (Day 70 ± 7 days), or any early termination (ET) visit as outlined in Section 6 and Appendix	Dalbavancin Concentration Measurements: Venous blood samples for PK analyses will be collected at Visit 2 (Day 1), Visit 3 (Day 8 ± 1 day),Visit 4 (Day 22 ± 2 days), Visit 5 (Day 42 ± 3 days), Visit 6 (Day 70 ± 7 days), or any early termination (ET) visit as outlined in Section 6 and Appendix A. The actual date and time of each	Formatting

Section	Previous Version (v1.0) Language	Change (v2.0) Language	Rationale
	A. The actual date and time of each blood sample collection will be recorded in the subjects' source document and the eCRF. Blood collected will be processed for plasma separation at the study site's laboratory; subsequent PK analyses will be performed on the plasma samples at a central laboratory. PK sample collection, labeling, processing, storage, and shipment instructions will be provided in the MOP.	blood sample collection will be recorded in the subjects' source document and the eCRF. Blood collected will be processed for plasma separation at the study site's laboratory; subsequent PK analyses will be performed on the plasma samples at a central laboratory. PK sample collection, labeling, processing, storage, and shipment instructions will be provided in the MOP.	
**8.1.1 Adverse Events (AEs)**	None	All AEs, regardless of grade, leading to study drug discontinuation will be captured on the appropriate data collection form and eCRF.	Clarification
**8.1.1 Adverse Events (AEs)**	For the purpose of the site's data collection responsibilities, any untoward event that was reported from the time of first study drug dose until the final protocol-defined study visit is to be considered an AE.	For the purpose of the site's data collection responsibilities, any untoward event that was reported from the time of first study drug dose after randomization at the Baseline Visit (Visit 2) until study Visit 6 is to be considered an AE.	Clarification
**8.1.1 Adverse Events (AEs)**	• All diseases that occur after signing the ICF, including any change in severity or frequency of pre-existing disease	• All diseases that occur after administration of study drug at the Baseline Visit, including any change in severity or frequency of pre-existing disease	Clarification
**8.1.1 Adverse Events (AEs)**	In this trial, only Grade 3 and higher AEs and AESIs will be captured in the eCRF.	In this trial, only Grade 3 and higher AEs, AEs of any grade leading study drug discontinuation, and AESIs will be captured in the eCRF.	Clarification

Section	Previous Version (v1.0) Language	Change (v2.0) Language	Rationale
**8.3.3 Reporting of Pregnancy**	The site is responsible for notifying their local IRB of any pregnancies in accordance with local policies.	The site is responsible for notifying the IRB/IEC on which it relies of any pregnancies in accordance with their policies as well as notifying their local IRB/IEC of any pregnancies in accordance with local policies.	Clarification
**10.5.5.2 Secondary Efficacy Outcomes**	As the secondary efficacy outcomes, each component of the DOOR will also be examined separately: clinical success, infectious complications, SAEs, AEs leading to study drug discontinuation, mortality.<br><br>Clinical failure at Days 42 and 70 will be analyzed using generalized estimating equations (GEE) assuming an unstructured correlation structure. The difference in proportions of clinical failure between the two groups at Days 42 and 70 will be calculated with the corresponding 2-sided 95% confidence interval.	As the secondary efficacy outcomes, each component of the DOOR will also be examined separately: clinical success, infectious complications, SAEs, AEs leading to study drug discontinuation, all-cause mortality.<br><br>Clinical failure at Day 70 will be analyzed using generalized estimating equations (GEE) assuming an unstructured correlation structure, including clinical failure at Day 42 (explanatory analysis time point). The difference in proportions of clinical failure between the two groups at Day 70 will be calculated with the corresponding 2-sided 95% confidence interval.	Clarification based on FDA feedback
**16 Literature References**	None	28. DALVANCE™ Dalbavancin for Injection [Investigator's Brochure]. Madison, NJ: Allergan USA, Inc.; 2020 August.	Update
**Appendix A.** Schedule of Events	Visit 7 (Day 180 ± 14 days, vertebral Osteomyelitis group)[a]	Visit 7 (Day 180 ± 14 days, Vertebral Osteomyelitis group)[a]	Grammar
**Appendix A.**	[1]Targeted physical exam includes general appearance, examination of head, eyes, ears, nose, throat, neck, skin,	[1]A physical examination (including general appearance, examination of head, eyes, ears, nose, throat, neck, skin,	Consistency

Section	Previous Version (v1.0) Language	Change (v2.0) Language	Rationale
Schedule of Events, Footnote	heart, lungs, abdomen, neurologic system, musculoskeletal system, extremities, height, and body weight. If height or weight is not obtainable (eg, patient is immobilized), use the last known or stated height and weight. Subsequent physical exams will focus on changes from prior exams and on the evaluation of newly reported symptoms.	heart, lungs, abdomen, neurologic system, musculoskeletal system, extremities, height, and body weight) will be done at Screening (Visit 1). If height or weight is not obtainable (eg, patient is immobilized), use the last known or stated height and weight. At subsequent visits, targeted physical exams will focus on changes from prior exams and on the evaluation of newly reported symptoms.	
**Appendix A.** Schedule of Events, Footnote	[n]All concomitant medications from Screening (Visit 1) through Day 42 (± 3 days) (Visit 5) must be recorded in the patient's medical record and on the eCRFs. Between the Day 42 Visit and Day 70~~84~~ Visit, all concomitant medications for an AE or any antibacterial therapy should be recorded in the patient's medical record and on the eCRF.	[n]All concomitant medications from Screening (Visit 1) through Day 42 (± 3 days) (Visit 5) must be recorded in the patient's medical record and on the eCRFs. Between the Day 42 Visit and Day 70 Visit, all concomitant medications for an AE or any antibacterial therapy should be recorded in the patient's medical record and on the eCRF.	Typographical error
**Appendix B.** Definitions	**Effective contraception:** Includes non-male sexual relationships, abstinence from sexual intercourse with a male partner, monogamous relationship with a vasectomized partner who has been vasectomized ≥180 days before the subject received the first dose of study drug, barrier methods such as condoms or diaphragms, effective intrauterine devices (IUDs), NuvaRing®, or licensed hormonal methods such as implants, injectables, or oral contraceptives.	**Effective contraception:** Must include at least one of the following: non-male sexual relationships only, abstinence from sexual intercourse with a male partner, monogamous relationship with a vasectomized partner who has been vasectomized ≥180 days before the subject received the first dose of study drug, barrier methods such as condoms or diaphragms, effective intrauterine devices (IUDs), NuvaRing®, or licensed hormonal methods such as implants, injectables, or oral contraceptives.	Clarification

Section	Previous Version (v1.0) Language	Change (v2.0) Language	Rationale
**Appendix B.** Definitions	Pleuropulmonary Infection – one of the following:<br>• Pulmonary infiltrate consistent with pneumonia in patients with *S. aureus* bacteremia<br>• *S. aureus* in pleural fluid, needle aspirate biopsy, or growth from bronchoalveolar lavage (BAL)/protected specimen brush (PSB)<br>• Clinical evidence of pneumonia (eg. Increased O2, increased respiratory rate, cough, mechanical ventilation, purulent sputum, etc)	Pleuropulmonary Infection – one of the following:<br>• Pulmonary infiltrate consistent with pneumonia in patients with *S. aureus* bacteremia<br>• *S. aureus* in pleural fluid, needle aspirate biopsy, or growth from bronchoalveolar lavage (BAL)/protected specimen brush (PSB)<br>• Clinical evidence of pneumonia (e.g. increased O_2, increased respiratory rate, cough, mechanical ventilation, purulent sputum, etc.)	Grammar
**Appendix B.** Definitions	Osteomyelitis - Either of the following:<br>• Radiographic evidence of bone lesion consistent with osteomyelitis/discitis; or<br>• Culture of bone yields staphylococcus.	Osteomyelitis - Either of the following:<br>• Radiographic evidence of bone lesion consistent with osteomyelitis/discitis; or<br>• Culture of bone yields *S. aureus*.	Clarification
**Appendix B.** Definitions	Septic Arthritis – Either of the following:<br>• Staphylococcus in culture of synovial fluid; or<br>• Positive Gram stain of synovial fluid for Gram positive cocci AND synovial fluid cell count ≥20,000 WBC/mL without alternate explanation	Septic Arthritis – Either of the following:<br>• *S. aureus* in culture of synovial fluid; or<br>• Positive Gram stain of synovial fluid for Gram positive cocci AND synovial fluid cell count ≥20,000 WBC/mL without alternate explanation	Clarification
**Appendix B.** Definitions	**Relapsing Infection**: Relapsing infection is defined as a staphylococcus that:	**Relapsing Bacteremia**: Relapsing bacteremia is defined as a *S. aureus* that:	Clarification

Section	Previous Version (v1.0) Language	Change (v2.0) Language	Rationale
	• Represents the same bacterial strain as the Baseline Infecting Pathogen (based on bacterial speciation, antibiotic susceptibility testing, and/or genotyping tests, as appropriate);<br>• Is documented by a culture yielding *S. aureus* obtained after randomization, unless from a procedure (e.g., abscess drainage, catheter removal) that was planned prior to randomization	• Represents the same bacterial strain as the Baseline Infecting Pathogen (based on bacterial speciation, antibiotic susceptibility testing, and/or genotyping tests, as appropriate);<br>• Is documented by a blood culture yielding *S. aureus* obtained after randomization	

20-0002 DOTS Protocol Summary of Changes v2.0 to v3.0

Section	Previous Version (v2.0) Language	Change (v3.0) Language	Rationale
Global	N/A	Typographical and formatting errors corrected throughout document.	Correction
All (Header)	Version 2.0	Version 3.0	Update
All (Header)	19 October 2020	14 October 2021	Update
Title Page	DMID Clinical Project Manager: [redacted]	DMID Clinical Project Manager: [redacted]	Update
Title Page	Draft or Version Number: v2.0	Draft or Version Number: v3.0	Update
Title Page	19 October 2020	14 October 2021	Update
**List of Abbreviations**	None	SOC: Standard of Care	Update
**List of Abbreviations**	ULN: Upper Limit Normal	ULN: Upper Limit of Normal	Grammar
**Protocol Summary** Study Objectives Exploratory:	• To compare clinical and microbiologic outcomes of dalbavancin with standard of care antibiotic therapy between clinically important subgroups, including a) those with MSSA versus MRSA; b) persons who inject drugs (PWID) vs non-PWID; c) those who received infectious disease consultation vs those who did not; d) underlying site of infection (endovascular, bone and joint, skin, pulmonary); e) divided by duration of initial bacteremia in the ITT, mITT, and CE populations	• To compare clinical and microbiologic outcomes of dalbavancin with standard of care antibiotic therapy between clinically important subgroups, including a) those with MSSA versus MRSA; b) persons who inject drugs (PWID) vs non-PWID; c) those who received infectious disease consultation vs those who did not; d) underlying site of infection (endovascular, bone and joint, skin, pulmonary); e) subjects with immune-suppression[1] ; f) divided by duration of initial bacteremia in the ITT, mITT, and CE populations	Update
**Protocol Summary** Study Objectives Exploratory:	• Examine the association between individualized plasma concentration profiles and late recurrence risk among the subset of patients with vertebral osteomyelitis and a 6 month follow-up visit	• Examine the association between individualized plasma concentration profiles and late recurrence risk among the subset of patients with osteomyelitis and a 6 month follow-up visit	Update

Section	Previous Version (v2.0) Language	Change (v3.0) Language	Rationale
**Protocol Summary**<br>Duration of Individual Subject Participation:	• Approximately 70 ± 7 days, with a late post-treatment follow-up visit for the subset of patients with vertebral osteomyelitis at 6 months	• Approximately 70 ± 7 days, with a late post-treatment follow-up visit for the subset of patients with osteomyelitis at 6 months	Update
**Protocol Summary**<br>Study Objectives Exploratory:<br>Footnote	None	[1] Defined as: On chemotherapy or immunotherapy for active hematologic malignancy expected to cause ANC < 500 cells/mm³ lasting > 7 days during the study period, chronic high dose oral steroids (equivalent of ≥ 20 mg prednisolone per day for or equivalent, for >2 weeks within the last month), HIV infection with a CD4 cell count < 100 cells/mm³ based on last known measurement or patient-reported value	Update
**Table 1: Treatment Arms**<br>Footnote	None	[c]If there are extenuating circumstances in which preferred standard of care antibiotics cannot be used, for example complex allergy history, then an alternative antibiotic may be used after discussion with the protocol PIs and DMID Medical Officer	Clarification
**1 Key Roles**<br>DMID Clinical Project Manager:	[redacted]<br>Phone: [redacted]<br>Email: [redacted]	[redacted]<br>Phone: [redacted]<br>Email: [redacted]	Update

Section	Previous Version (v2.0) Language	Change (v3.0) Language	Rationale
**3.2.3 Exploratory**	4. To compare clinical and microbiologic outcomes of dalbavancin with standard of care antibiotic therapy between clinically important subgroups, including a) those with MSSA versus MRSA; b) persons who inject drugs (PWID) vs non-PWID; c) those who received infectious disease consultation vs those who did not; d) underlying site of infection (endovascular, bone and joint, skin, pulmonary); e) divided by duration of initial bacteremia, in the ITT, mITT, and CE populations	4. To compare clinical and microbiologic outcomes of dalbavancin with standard of care antibiotic therapy between clinically important subgroups, including a) those with MSSA versus MRSA; b) persons who inject drugs (PWID) vs non-PWID; c) those who received infectious disease consultation vs those who did not; d) underlying site of infection (endovascular, bone and joint, skin, pulmonary); e) subjects with immune-suppression[2] ; f) divided by duration of initial bacteremia, in the ITT, mITT, and CE populations	Update
**3.2.3 Exploratory**	6. To characterize the population pharmacokinetic profile for dalbavancin administered via a 2-dose regimen (1500 mg on day 1 and 1500 mg on day 8; renally adjusted when appropriate) in patients with *Staphylococcus aureus* bacteremia	6. To characterize the population pharmacokinetic profile for dalbavancin administered via a 2-dose regimen (1500 mg on day 1 and 1500 mg on day 8; renally adjusted when appropriate) in patients with *S. aureus* bacteremia	Update
**3.2.3 Exploratory**	10. Examine the association between individualized plasma concentration profiles and late recurrence risk among the subset of patients with vertebral osteomyelitis and a 6-month follow-up visit	10. Examine the association between individualized plasma concentration profiles and late recurrence risk among the subset of patients with osteomyelitis and a 6-month follow-up visit	Update
**3.3.3 Exploratory** Footnote	None	[2] Defined as: On chemotherapy or immunotherapy for active hematologic malignancy expected to cause ANC < 500 cells/mm^3 lasting > 7 days during the study period, chronic high dose oral steroids (equivalent of ≥ 20 mg prednisolone per day for or equivalent, for >2 weeks within the last month), HIV infection with a CD4 cell count < 100 cells/mm^3 based on last known measurement or patient-reported value	Update

Section	Previous Version (v2.0) Language	Change (v3.0) Language	Rationale
**3.3.3 Exploratory**	10. Late recurrence within the vertebral osteomyelitis population will be defined by the presence of the following up to 6 months after randomization: progressive imaging changes along with isolation of *S. aureus* from blood, bone biopsy, associated fluid aspiration, or operative tissue culture.	10. Late recurrence within the osteomyelitis population will be defined by the presence of the following up to 6 months after randomization: progressive imaging changes along with isolation of *S. aureus* from blood, bone biopsy, associated fluid aspiration, or operative tissue culture.	Update
**4.1.1 Formulation, Packaging, and Labeling**	The SOC antibiotics (Cefazolin, nafcillin, oxacillin, vancomycin, and daptomycin) will be prepared and labeled in accordance with the clinical site pharmacy's standard operating procedures (SOPs).	The standard of care (SOC) antibiotics (Cefazolin, nafcillin, oxacillin, vancomycin, and daptomycin) will be prepared and labeled in accordance with the clinical site pharmacy's standard operating procedures (SOPs).	Clarification
**Table 1: Acceptable SOC Antibiotics** Baseline Pathogen	Standard of Care Therapy (doses may be adjusted for renal function)	Standard of Care Therapy (doses may be adjusted for renal function)[b]	Clarification
**Table 5: Acceptable SOC Antibiotics** MSSA[a]	nafcillin (2 g IV q4h × 4-6 weeks[b])[d]<br>OR<br>oxacillin (2 g IV Q4h x 4-6 weeks[b])[d]<br>OR<br>cefazolin (2 g IV q8h × 4-6 weeks[b])	nafcillin (2 g IV q4h × 4-6 weeks[c])[e]<br>OR<br>oxacillin (2 g IV Q4h x 4-6 weeks[c])[e]<br>OR<br>cefazolin (2 g IV q8h × 4-6 weeks[c])	Clarification and update
**Table 2: Acceptable SOC Antibiotics** MRSA	vancomycin (dose per local standard of care × 4-6 weeks[b])<br>[c] OR<br>daptomycin (6-10 mg/kg IV daily × 4-6 weeks[b])	vancomycin (dose per local standard of care × 4-6 weeks[c])<br>[d] OR<br>daptomycin (6-10 mg/kg IV daily × 4-6 weeks[c])	Clarification and update

Section	Previous Version (v2.0) Language	Change (v3.0) Language	Rationale
**Table 5: Acceptable SOC Antibiotics** Footnote	[a] Vancomycin or daptomycin are also appropriate for patients with MSSA and anaphylactoid-type hypersensitivity to beta-lactams.<br>[b] Duration of antibiotics reflects usual standard of care for complicated SAB. It may be extended to a maximum of 8 weeks at the discretion of the treating clinician.<br>[c] Patients on vancomycin will have dose adjustment and monitoring based on local standard of care<br>[d] As applicable per site standard of care, nafcillin and oxacillin may be administered at an equivalent dose via continuous IV infusion (e.g., 12g/24h IV continuous).	[a] Vancomycin or daptomycin are also appropriate for patients with MSSA and anaphylactoid-type hypersensitivity to beta-lactams.<br>[b] If there are extenuating circumstances in which preferred standard of care antibiotics cannot be used, for example complex allergy history, then an alternative antibiotic may be used after discussion with the protocol PIs and DMID Medical Officer.<br>[c] Duration of antibiotics reflects usual standard of care for complicated SAB. It may be extended to a maximum of 8 weeks at the discretion of the treating clinician.<br>[d] Patients on vancomycin will have dose adjustment and monitoring based on local standard of care<br>[e] As applicable per site standard of care, nafcillin and oxacillin may be administered at an equivalent dose via continuous IV infusion (e.g., 12g/24h IV continuous).	Clarification and update
**5.1.1 Subject Inclusion Criteria**	4. Treated with effective antibiotic therapy for at least 72 hours (maximum 10 days).	4. Treated with effective antibiotic therapy for at least 72 hours (maximum 10 days)[3].	Clarification
**5.1.1 Subject Inclusion Criteria** Footnote	None	[3] Ten consecutive days prior to randomization is the maximum allowed treatment duration. If a subject has received intermittent or incomplete therapy earlier in the treatment course for this episode of *S. aureus* bacteremia, then discuss with the protocol PI and DMID Medical Officer prior to enrollment.	Clarification
**5.1.2 Subject Exclusion Criteria**	6. Presence of intravascular graft or intravascular material (excluding cardiac stents)	6. Presence of intravascular graft or intravascular material[7] UNLESS removal is planned within 4 days post-randomization.	Clarification

Section	Previous Version (v2.0) Language	Change (v3.0) Language	Rationale
	UNLESS removal is planned within 4 days post-randomization.		
**5.1.2 Subject Exclusion Criteria** Footnote	[1] Uncomplicated *Staphylococcus aureus* bacteremia is defined as all of the following: exclusion of endocarditis by echocardiography; catheter-associated bacteremia and removal of catheter; no implanted prostheses; follow-up blood cultures drawn within 48 hours after initial set that do not grow screening pathogen and all follow-up blood cultures thereafter do not grow the screening pathogen; defervescence within 72 hours of initiating effective therapy; and no evidence of metastatic sites of infection.	[5] Uncomplicated *Staphylococcus aureus* bacteremia is defined as all of the following: exclusion of endocarditis by echocardiography; catheter-associated bacteremia and removal of catheter; no implanted prostheses; follow-up blood cultures drawn within 48 hours after initial set that do not grow screening pathogen and all follow-up blood cultures thereafter do not grow the screening pathogen; defervescence within 72 hours of initiating effective therapy; and no evidence of metastatic sites of infection. Any patient not meeting these strict criteria is considered to have *complicated* bacteremia and is eligible	Clarification
**5.1.2 Subject Exclusion Criteria** Footnote	None	[7]Excluding cardiac stents, inferior vena cava filters in place for >6 weeks, vascular stents in place for >6 weeks, non-hemodialysis grafts in place >90 days, and hemodialysis grafts not used within the past 12 months and not previously infected. A fistula constructed from native veins (without synthetic graft material) does not count as intravascular graft/material.	Clarification
**5.1.2 Subject Exclusion Criteria** Footnote	[9] On chemotherapy or immunotherapy for active hematologic malignancy expected to cause ANC < 500 cells/mm^3 lasting > 7 days during the study period, recent bone marrow transplant (in the past 90 days), solid organ transplantation within prior 3 months or receipt of augmented immunosuppression for rejection within 3 months, chronic high dose oral steroids (equivalent of ≥ 20 mg prednisolone per day for or equivalent, for >2 weeks within the last	[9]On chemotherapy or immunotherapy for active hematologic malignancy expected to cause > 7 days of ANC < 100 cells/mm^3, recent bone marrow transplant (in the past 90 days), solid organ transplantation within prior 3 months or receipt of augmented immunosuppression for rejection within 3 months, chronic granulomatous disease, HIV infection with a CD4 cell count < 50 cells/mm^3 based on last known measurement or patient-reported value.	Update

Section	Previous Version (v2.0) Language	Change (v3.0) Language	Rationale
	month), chronic granulomatous disease, HIV infection with a CD4 cell count < 100 cells/mm^3 based on last known measurement or patient-reported value.		
**5.2.1 Withdrawal from the Study or Discontinuation of the Study Product**	An investigator may also discontinue a subject from receiving the study product for any reason.	An investigator may also discontinue a subject from receiving the study product (i.e., dalbavancin or SOC antibiotic) for any reason.	Clarification
**5.2.1 Withdrawal from the Study or Discontinuation of the Study Product**	The reasons to discontinue study product might include, but are not limited to, the following:	The reasons to discontinue study product (i.e., dalbavancin or SOC antibiotic) might include, but are not limited to, the following:	Clarification
**5.2.1 Withdrawal from the Study or Discontinuation of the Study Product**	• Occurrence of an AE that, in the opinion of the investigator, warrants the subject's permanent discontinuation from IV study drug	• Occurrence of an AE that, in the opinion of the investigator, warrants the subject's permanent discontinuation from IV study drug (i.e., dalbavancin or SOC antibiotic)	Clarification
**5.2.1 Withdrawal from the Study or Discontinuation of the Study Product**	A clear description of reason for early withdrawal or discontinuation from investigational product must be documented.	A clear description of reason for early withdrawal or discontinuation from study product (i.e., dalbavancin or SOC antibiotic) must be documented.	Clarification
**6.3.1 Baseline (Randomization): Visit 2 (Day1)**	• Review concomitant medications (medications post first dose of investigational product).	• Review concomitant medications (medications post first dose of study product).	Clarification
**6.3.1 Baseline (Randomization): Visit 2 (Day1)**	• Administer IV investigational product or standard of care antibiotic therapy according to randomization as outlined in Section 4.3. The provider should designate which antibiotic is assigned as the study drug for patients randomized to standard of care group.	• Administer IV dalbavancin or standard of care antibiotic therapy according to randomization as outlined in Section 4.3. The provider should designate which antibiotic is assigned as the study drug for patients randomized to standard of care group.	Clarification

Section	Previous Version (v2.0) Language	Change (v3.0) Language	Rationale
**6.3.1 Baseline (Randomization): Visit 2 (Day1)**	• Review and record AEs/SAEs/AESIs after study product administration.	• Review and record AEs/SAEs/AESIs after study product (i.e., dalbavancin or SOC antibiotic) administration.	Clarification
**6.3.6 Visit 7 Subtitle**	Visit 7 (Day 180 ± 14 days – only for subjects with vertebral osteomyelitis)	Visit 7 (Day 180 ± 14 days – only for subjects with osteomyelitis)	Update
**6.3.6 Visit 7**	Visit 7 will only be performed for subjects with vertebral osteomyelitis in order to determine long-term recurrence risk.	Visit 7 will only be performed for subjects with osteomyelitis in order to determine long-term recurrence risk.	Update
**6.3.7 Final Study Visit**	• For subjects without vertebral osteomyelitis, the final study visit will be Visit 6 (Day 70 ± 7 days) as above. For subjects with vertebral osteomyelitis, the final study visit will be Visit 7 (Day 180 ± 14 days) as above.	• For subjects without osteomyelitis, the final study visit will be Visit 6 (Day 70 ± 7 days) as above. For subjects with osteomyelitis, the final study visit will be Visit 7 (Day 180 ± 14 days) as above.	Update
**6.3.8 Early Termination Visit**	In circumstances where a subject withdraws from the study or discontinues the study product early, an Early Termination Visit will be performed, which include the following procedures:	In circumstances where a subject withdraws from the study or discontinues the study product (i.e., dalbavancin or SOC antibiotic) early, an Early Termination Visit will be performed, which include the following procedures:	Clarification
**7.1.2 Assessment of Concomitant Medications/Treat ments Other Than Study Product**	After Visit 6, for subjects with vertebral osteomyelitis, who are followed until Day 180 ± 14 days (Visit 7), the only concomitant medications that must be recorded are new antibiotics that are prescribed for the treatment of osteomyelitis.	After Visit 6, for subjects with osteomyelitis, who are followed until Day 180 ± 14 days (Visit 7), the only concomitant medications that must be recorded are new antibiotics that are prescribed for the treatment of osteomyelitis.	Update
**8.1.1 Adverse Events (AEs)**	An AE can therefore be any unfavorable and unintended sign (including an abnormal laboratory finding), symptom, or disease temporally associated with the use of medicinal (investigational) product.	An AE can therefore be any unfavorable and unintended sign (including an abnormal laboratory finding), symptom, or disease temporally associated with the use of medicinal product.	Clarification
**8.1.1 Adverse Events (AEs)**	Information to be collected for AEs includes event description, date of onset, assessment of	Information to be collected for AEs includes event description, date of onset, assessment of	Clarification

Section	Previous Version (v2.0) Language	Change (v3.0) Language	Rationale
	severity, relationship to study product and alternate etiology (assessed only by those with the training and authority to make a diagnosis and listed on the Form FDA 1572 as an investigator), date of resolution, seriousness and outcome.	severity, relationship to study product (i.e., dalbavancin or SOC antibiotic) and alternate etiology (assessed only by those with the training and authority to make a diagnosis and listed on the Form FDA 1572 as an investigator), date of resolution, seriousness and outcome.	
**8.1.1. Adverse Events (AEs)**	For the purpose of the site's data collection responsibilities, any untoward event that was reported from the time of first study drug dose after randomization at the Baseline Visit (Visit 2) until study Visit 6 is to be considered an AE.	For the purpose of the site's data collection responsibilities, any untoward event that was reported from the time of first study drug dose (i.e., dalbavancin or SOC antibiotic) after randomization at the Baseline Visit (Visit 2) until study Visit 6 is to be considered an AE.	Clarification
**8.1.1 Adverse Events (AEs)**	• All clinically significant abnormalities in laboratory values or clinically significant physical findings that occur during the study collection and reporting period	• All clinically significant abnormalities in laboratory values or clinically significant physical findings that occur during the study collection and reporting period. However, a single abnormal laboratory result does not automatically indicate progression of a chronic condition. For example, a transient spike in blood glucose level for a subject with diabetes is not reported as an AE.	Clarification
**8.1.1 Adverse Events (AEs)**	Any medical condition that is present at the time that the subject is screened will be considered as baseline and not reported as an AE.	Any medical condition that is present at the time of signing of ICF when the subject is screened will be considered as baseline and not reported as an AE.	Clarification
**8.1.1.1 Adverse Events Grading**	All AEs (laboratory and clinical symptoms) will be graded for severity (per CTCAE table, version 5.0, https://ctep.cancer.gov/protocolDevelopment/electronic_applications/docs/CTCAE_v5_Quick_Reference_8.5x11.pdf) and assessed for relationship to study product as outlined below.	All AEs (laboratory and clinical symptoms) will be graded for severity (per CTCAE table, version 5.0, https://ctep.cancer.gov/protocolDevelopment/electronic_applications/docs/CTCAE_v5_Quick_Reference_8.5x11.pdf) and assessed for	Clarification

Section	Previous Version (v2.0) Language	Change (v3.0) Language	Rationale
		relationship to study product (i.e., dalbavancin or SOC antibiotic) as outlined below.	
**8.1.1.1 Adverse Events Grading** Relationship to Study Product:	The assessment of the AE's relationship to study product will be done by a licensed study investigator indicated on the Form FDA 1572 and the assessment will be part of the documentation process.	The assessment of the AE's relationship to study product (i.e., dalbavancin or SOC antibiotic) will be done by a licensed study investigator indicated on the Form FDA 1572 and the assessment will be part of the documentation process.	Clarification
**8.1.1.1 Adverse Events Grading** Relationship to Study Product:	In a clinical trial, the study product must always be suspect. The relationship to study product will be assessed for AEs using the terms related or not related:	In a clinical trial, the study product must always be suspect. The relationship to study product (i.e., dalbavancin or SOC antibiotic) will be assessed for AEs using the terms related or not related:	Clarification
**8.1.2 Serious Adverse Events (SAEs)**	• Assessed for severity and relationship to study product and alternate etiology (if not related to study product) by a licensed study physician listed on the Form FDA 1572 or by the Institution as the site Principal Investigator or Sub-Investigator.	• Assessed for severity and relationship to study product (i.e., dalbavancin or SOC antibiotic) and alternate etiology (if not related to study product) by a licensed study physician listed on the Form FDA 1572 or by the Institution as the site Principal Investigator or Sub-Investigator.	Clarification
**8.2 Specification of Safety Parameters**	For each safety parameter, the last assessment made before the first dose of investigational product will be used as the baseline for all analyses of that safety parameter.	For each safety parameter, the last assessment made before the first dose of study product will be used as the baseline for all analyses of that safety parameter.	Clarification
**8.2.1 Adverse Events of Special Interests (AESIs)**	Adverse events of special interest (AESIs) are AEs that are common and known to occur following administration of study product. AESIs will be collected after first dose of study product is given and until final study visit.	Adverse events of special interest (AESIs) are AEs that are common and known to occur following administration of study product (i.e., dalbavancin or SOC antibiotic). AESIs will be collected after first dose of study product (i.e., dalbavancin or SOC antibiotic) is given and until final study visit.	Clarification

Section	Previous Version (v2.0) Language	Change (v3.0) Language	Rationale
**8.3.1 Reporting Serious Adverse Events**	Any AE that meets a protocol-defined serious criterion must be submitted immediately (within 24 hours of site awareness) on an SAE form to the DMID Pharmacovigilance Group, at the following address:	Any AE that meets a protocol-defined serious criterion that is judged to be related to either study product (i.e., dalbavancin or SOC antibiotic) must be submitted immediately (within 24 hours of site awareness) on an SAE form to the DMID Pharmacovigilance Group, at the following address:	Update
**8.3.1 Reporting of Serious Adverse Events**	In addition to the SAE form, select SAE data fields must also be entered into the data coordinating center (DCC) system.	In addition to the SAE form, select SAE data fields must also be entered into the data coordinating center (DCC) database system, regardless of relationship to study product (i.e., dalbavancin or SOC antibiotic).	Clarification
**8.3.1 Reporting of Serious Adverse Events**	The DMID Medical Monitor and DMID Clinical Project Manager will be notified of the SAE by the DMID Pharmacovigilance Group.	The DMID Medical Monitor and DMID Clinical Project Manager will be notified of the related SAE by the DMID Pharmacovigilance Group.	Update
**8.3.1 Reporting of Serious Adverse Events**	None	SAEs that are judged to be **not related** to study product (i.e., dalbavancin or SOC antibiotic) will be captured on the appropriate data collection form and eCRF, but do not require separate reporting to the DMID Pharmacovigilance Group.	Update
**8.3.1 Reporting of Serious Adverse Events**	At any time after completion of the study, if the site principal investigator or appropriate sub-investigator becomes aware of an SAE that is suspected to be related to study product, the site principal investigator or appropriate sub-investigator will report the event to the DMID Pharmacovigilance Group.	At any time after completion of the study, if the site principal investigator or appropriate sub-investigator becomes aware of an SAE that is suspected to be related to dalbavancin that occurred during the subject's participation in the study, the site principal investigator or appropriate sub-investigator will report the event to the DMID Pharmacovigilance Group.	Update

Section	Previous Version (v2.0) Language	Change (v3.0) Language	Rationale
**8.3.3 Reporting of Pregnancy**	Pregnancy occurring during a clinical investigation, although not considered a SAE, must be reported within the same timelines as a SAE. The positive pregnancy test will be recorded in the database within 5 days of site awareness, on the Pregnancy Report form.	Pregnancy occurring during a subject's participation in the study must be recorded in the database within 5 days of site awareness, on the Pregnancy Report form.	Update
**8.6.1 Data and Safety Monitoring Board (DSMB)**	• An Interim analysis for efficacy after at least approximately 50% of subjects have completed the study	• An Interim analysis for futility after at least approximately 50% of subjects have completed the study	Update
**10 Statistical Considerations**	This is a phase 2b, multicenter, open-label, randomized, assessor-blinded, superiority study comparing a 2-dose dalbavancin regimen to standard of care therapy for the treatment of complicated bacteremia caused by *S. aureus*. Participants will be followed for 70 days, except for the subset with vertebral osteomyelitis who will be followed for 180 days.	This is a phase 2b, multicenter, open-label, randomized, assessor-blinded, superiority study comparing a 2-dose dalbavancin regimen to standard of care therapy for the treatment of complicated bacteremia caused by *S. aureus*. Participants will be followed for 70 days, except for the subset with osteomyelitis who will be followed for 180 days.	Update
**10.4 Planned Interim Analysis**	The DSMB will review interim reports of efficacy by treatment strategy. There will be one formal interim analysis of efficacy after approximately 50% of subjects have completed the trial.	The DSMB will review interim reports of efficacy by treatment strategy. There will be one formal interim analysis of futility after approximately 50% of subjects have completed the trial.	Update
**10.4.2 Subtitle**	Interim Efficacy Review	Interim Futility Review	Update
**10.4.2 Interim Futility Review**	The interim efficacy analysis will consist of a quantitative evaluation of potential effect sizes and associated precision using a predicted intervals and predicted interval plots (PIPS) approach.	The interim futility analysis will consist of a quantitative evaluation of potential effect sizes and associated precision using a predicted intervals and predicted interval plots (PIPS) approach.	Update
**10.5.3 Demographics and Other Baseline Characteristics**	Prior medication is defined as any medication taken before the date of the first dose of investigational product. Concomitant medication is defined as any medication started	Prior medication is defined as any medication taken before the date of the first dose of study product. Concomitant medication is defined as	Consistency

Section	Previous Version (v2.0) Language	Change (v3.0) Language	Rationale
	on or after the date of the first dose of investigational product.	any medication started on or after the date of the first dose of study product.	
**10.5.4 Extent of Exposure and Treatment Compliance**	Exposure to investigational product for the safety population will be summarized for treatment duration, calculated as the number of doses of dalbavancin received for patients in the dalbavancin group, and by start and stop dates for standard of care antibiotics.	Exposure to study product for the safety population will be summarized for treatment duration, calculated as the number of doses of dalbavancin received for patients in the dalbavancin group, and by start and stop dates for standard of care antibiotics.	Consistency
**10.5.6 Safety Analyses**	For each safety parameter, the last assessment made before the first dose of investigational product will be used as the baseline for all analyses of that safety parameter.	For each safety parameter, the last assessment made before the first dose of study product will be used as the baseline for all analyses of that safety parameter.	Consistency
**10.5.6.1 Adverse Events**	An AE (classified by preferred term) that occurs during the treatment period will be considered a treatment-emergent AE if it was not present before the first dose of investigational product or was present before the first dose of investigational product and increased in severity during the treatment period.	An AE (classified by preferred term) that occurs during the treatment period will be considered a treatment-emergent AE if it was not present before the first dose of study product or was present before the first dose of study product and increased in severity during the treatment period.	Consistency
**10.5.6.1 Adverse Events**	The number and percentage of patients reporting treatment-emergent AEs in each treatment group will be tabulated by system organ class and preferred term; by system organ class, preferred term, and severity; and by system organ class, preferred term, and causal relationship to the investigational product. If more than one AE is coded to the same preferred term for the same subject, the subject will be counted only once for that preferred term using the most severe and most related occurrence for the summarization by severity and by causal relationship to the investigational product.	The number and percentage of patients reporting treatment-emergent AEs in each treatment group will be tabulated by system organ class and preferred term; by system organ class, preferred term, and severity; and by system organ class, preferred term, and causal relationship to the study product. If more than one AE is coded to the same preferred term for the same subject, the subject will be counted only once for that preferred term using the most severe and most related occurrence for the summarization by severity and by causal relationship to the study product.	Consistency

Section	Previous Version (v2.0) Language	Change (v3.0) Language	Rationale
**10.5.6.1 Adverse Events**	The distribution of treatment-emergent AEs and AESIs by severity and causal relationship to the investigational product will be summarized by treatment group	The distribution of treatment-emergent AEs and AESIs by severity and causal relationship to the study product will be summarized by treatment group	Consistency
**10.5.6.1 Adverse Events**	The incidence of common (≥ 2% of patients in any treatment group) treatment-emergent AEs, on-therapy SAEs, AESIs, and AEs leading to premature discontinuation of the investigational product will be summarized by preferred term and treatment group and will be sorted by decreasing frequency for the investigational product. In addition, the incidence of fatal on-therapy SAEs (i.e., events that caused death) will be summarized separately by treatment group and preferred term. An SAE will be defined as an on-therapy SAE if it occurred during or after the first infusion of investigational product.	The incidence of common (≥ 2% of patients in any treatment group) treatment-emergent AEs, on-therapy SAEs, AESIs, and AEs leading to premature discontinuation of the study product will be summarized by preferred term and treatment group and will be sorted by decreasing frequency for the study product. In addition, the incidence of fatal on-therapy SAEs (i.e., events that caused death) will be summarized separately by treatment group and preferred term. An SAE will be defined as an on-therapy SAE if it occurred during or after the first infusion of study product.	Consistency
**10.5.6.3 Exploratory Dalbavancin Plasma Pharmacokinetic Analysis** Exposure-Response Relationship Analysis	The outcomes evaluated will include (1) DOOR outcomes at day 42, mortality at day 42, infectious complications at day 42, adverse drug effects (grade 3 or higher) at day 42, late recurrence at 6 months (within the subset having vertebral osteomyelitis), and occurrence of AST/ALT elevation >3x upper limit of normal during treatment.	The outcomes evaluated will include (1) DOOR outcomes at day 42, mortality at day 42, infectious complications at day 42, adverse drug effects (grade 3 or higher) at day 42, late recurrence at 6 months (within the subset having osteomyelitis), and occurrence of AST/ALT elevation >3x upper limit of normal during treatment.	Update
**17 Appendices Appendix A.** Schedule of Events	Visit 7 (Day 180 ± 14 days, Vertebral Osteomyelitis group)[a]	Visit 7 (Day 180 ± 14 days, Osteomyelitis group)[a]	Update
**17 Appendices Appendix B.** Definitions	**Uncomplicated *Staphylococcus aureus* Bacteremia:** defined as all of the following: exclusion of endocarditis by echocardiography; catheter-associated bacteremia and removal of	**Uncomplicated *Staphylococcus aureus* Bacteremia:** defined as all of the following: exclusion of endocarditis by echocardiography; catheter-associated bacteremia and removal of	Moved to a different location within this section for readability

Section	Previous Version (v2.0) Language	Change (v3.0) Language	Rationale
	catheter; no implanted prostheses; follow-up blood cultures drawn within 48 hours after initial set that do not grow screening pathogen and all follow-up blood cultures thereafter do not grow the screening pathogen; defervescence within 72 hours of initiating effective therapy; and no evidence of metastatic sites of infection.	catheter; no implanted prostheses; follow-up blood cultures drawn within 48 hours after initial set that do not grow screening pathogen and all follow-up blood cultures thereafter do not grow the screening pathogen; defervescence within 72 hours of initiating effective therapy; and no evidence of metastatic sites of infection.	
**17 Appendices Appendix B.** Definitions, Complicated *S. aureus* Bacteremia:	None	The following are examples of complicated *S. aureus* bacteremia. This is not an exhaustive list. If a patient's infection does not meet the definition of uncomplicated bacteremia above, then that patient has complicated bacteremia.	Clarification

20-0002 DOTS Protocol Summary of Changes v3.0 to v4.0

Section	Previous Version (v3.0) Language	Change (v4.0) Language	Rationale
Global	N/A	Typographical and formatting errors corrected throughout document.	Correction
All (Header)	Version 3.0	Version 4.0	Update
All (Header)	14 October 2021	13 Jul 2022	Update
Title Page	Draft or Version Number: v3.0	Draft or Version Number: v4.0	Update
Title Page	14 October 2021	13 Jul 2022	Update
**List of Abbreviations**	None	SUSAR: Suspected Unexpected Serious Adverse Reaction	Update
**Table 1: Treatment Arms** Standard of Care	• Methicillin-sensitive Staphylococcus aureus (MSSA): nafcillin (2 g IV q4h × 4-6 weeks)[b] OR oxacillin (2 g IV Q4h x 4-6 weeks)[b] OR cefazolin (2 g IV q8h × 4-6 weeks) [c]	• Methicillin-sensitive Staphylococcus aureus (MSSA): nafcillin (2 g IV q4h × 4-6 weeks)[b] OR oxacillin (2 g IV Q4h x 4-6 weeks)[b] OR cefazolin (2 g IV q8h × 4-6 weeks) [b,c]	Update
**Table 1: Treatment Arms** Footnote	[b]As applicable per site standard of care, nafcillin and oxacillin may be administered at an equivalent dose via continuous IV infusion (e.g., 12g/24h IV continuous)	[b]As applicable per site standard of care, beta-lactams may be administered at an equivalent dose via continuous IV infusion (e.g., nafcillin 12g/24h IV continuous)	Update

Section	Previous Version (v3.0) Language	Change (v4.0) Language	Rationale
**4.3 Dosage/Regimen, Preparation, Dispensing and Administration of Study Intervention/Investigational Product**	For subjects randomized to the dalbavancin treatment group, the dosage of dalbavancin administered will be determined based on individual estimated serum creatinine clearance (CrCl) levels as follows:	For subjects randomized to the dalbavancin treatment group, the dosage of dalbavancin administered will be determined based on individual estimated serum creatinine clearance (CrCl) levels and presence of regular hemodialysis or peritoneal dialysis as follows:	Update
**4.3 Dosage/Regimen, Preparation, Dispensing and Administration of Study Intervention/Investigational Product**	Any changes to study drug (discontinuation or dose adjustment) will be captured and the reason for this change will be recorded.	Any changes to study drug (discontinuation or dose adjustment) will be captured and the reason for this change will be recorded, with the exception that vancomycin dose adjustments do not need to be captured.	Update
**Table 5: Acceptable SOC Antibiotics** MSSA	nafcillin (2 g IV q4h × 4-6 weeks[c])[e]<br>OR<br>oxacillin (2 g IV Q4h x 4-6 weeks[c])[e]<br>OR<br>cefazolin (2 g IV q8h × 4-6 weeks[c])	nafcillin (2 g IV q4h × 4-6 weeks[c])[e]<br>OR<br>oxacillin (2 g IV Q4h x 4-6 weeks[c])[e]<br>OR<br>cefazolin (2 g IV q8h × 4-6 weeks[c])[e]	Update
**Table 5: Acceptable SOC Antibiotics** Footnote	[e] As applicable per site standard of care, nafcillin and oxacillin may be administered at an equivalent dose via continuous IV infusion (e.g., 12g/24h IV continuous).	[e] As applicable per site standard of care, beta-lactams may be administered at an equivalent dose via continuous IV infusion (e.g., nafcillin 12g/24h IV continuous).	Update

Section	Previous Version (v3.0) Language	Change (v4.0) Language	Rationale
**4.3 Dosage/Regimen, Preparation, Dispensing and Administration of Study Intervention/Investigational Product**	None	Regarding vancomycin dosing, dose adjustments and monitoring will be performed in accordance with local standard of care. Vancomycin monitoring may be performed by trough levels, or area under the curve (AUC) based protocols, or other methods guided by sites' local standards. Because vancomycin dosing and monitoring is determined by these local standards of care, we will not require specific doses or levels to be recorded.	Update
**5.1.2 Subject Exclusion Criteria** Footnote	[7] Excluding cardiac stents, inferior vena cava filters in place for >6 weeks, vascular stents in place for >6 weeks, non-hemodialysis grafts in place >90 days, and hemodialysis grafts not used within the past 12 months and not previously infected. A fistula constructed from native veins or a native vascular graft (without synthetic graft material) does not count as intravascular graft/material.	[7] Excluding cardiac stents, inferior vena cava filters in place for >6 weeks, vascular stents in place for >6 weeks, non-hemodialysis grafts in place >90 days, and hemodialysis grafts not used within the past 12 months and not previously infected. A fistula constructed from native veins or a biologic vascular graft (without synthetic graft material) does not count as intravascular graft/material.	Update
**5.2.1 Withdrawal from the Study or Discontinuation of the Study Product**	If the subject consents, every attempt will be made to follow all AEs through.	If the subject consents, every attempt will be made to follow all AEs through resolution or through the end of the study, whichever occurs first.	Update
**6.3.6 Visit 7 (Day 180 ± 14 days – only for subjects with osteomyelitis)**	Visit 7 will only be performed for subjects with osteomyelitis in order to determine long-term recurrence risk. Any subject developing osteomyelitis during the study period, from baseline through day 70, will be followed to day 180 (e.g., osteomyelitis does not need to have been present at baseline to follow the subject out to Visit 7).	Visit 7 will only be performed for subjects with osteomyelitis present at baseline assessment in order to determine long-term recurrence risk.	Update

Section	Previous Version (v3.0) Language	Change (v4.0) Language	Rationale
**7.1.1 Research Procedures**	Medication History: from 30 days prior to signing the ICF, and 60 days for any prior lipoglycopeptides (oritavancin or dalbavancin).	Medication History: from 30 days prior to signing the ICF, and 60 days for any prior lipoglycopeptides (oritavancin or dalbavancin) as reported by patient.[11]	Update
**7.1.1 Research Procedures** Footnote	None	[11] As patients may receive medication prescriptions from multiple sources, patient-reported medications are intended to serve as the primary source for concomitant medications. For sites that have the ability to cross-query reported medications against prescription or medication administration records, this can be used as a secondary source but is not required.	Update
**7.1.2 Assessment of Concomitant Medications/Treatments Other Than Study Product**	Medication history during the 30 days prior to ICF signing will be recorded at Screening (Visit 1) in the eCRF. Thereafter, any changes in concomitant medications or new medications added will be recorded in the eCRF.	Patient-reported medication history during the 30 days prior to ICF signing will be recorded at Screening (Visit 1) in the eCRF.[12] Thereafter, any changes in concomitant medications or new medications added will be recorded in the eCRF.	Clarification

Section	Previous Version (v3.0) Language	Change (v4.0) Language	Rationale
**7.1.2 Assessment of Concomitant Medications/Treatments Other Than Study Product**	Any systemic medication taken by the subject, other than study drugs, is considered a concomitant medication. Topical medications including eye drops, ear drops, or dermatologic treatments do not need to be recorded in the eCRFs.	While most systemic medications taken by the subject, other than study drug, are considered concomitant medications and should be recorded, the following specific exceptions do not need to be recorded:<br>1. Topical medications (eye drops, ear drops, intranasal drops or sprays, dermatologic treatments, topical lidocaine)<br>2. Vitamins and supplements (e.g., vitamins, minerals, herbal supplements, dietary supplements, iron/ferrous sulfate, magnesium, calcium, electrolyte replacements)<br>3. Symptomatic care medications (e.g., antipyretics such as ibuprofen or acetaminophen, antihistamines, decongestants, non-steroidal anti-inflammatory drugs [NSAIDs])<br>4. Gastrointestinal agents (prokinetic agents, laxatives, stool softeners, gastrointestinal stimulants, antacids)<br>5. Nicotine replacement products (e.g., patches, lozenges, gums, nasal sprays)<br>6. Heparin flushes (e.g., as used for vascular access flushes)	Update

Section	Previous Version (v3.0) Language	Change (v4.0) Language	Rationale
**7.1.2 Assessment of Concomitant Medications/Treatments Other Than Study Product**	All concomitant systemic medications from Screening (Visit 1) through Day 70 ± 7 days (Visit 6) must be recorded in the subject's medical record and on the eCRFs. After Visit 6, for subjects with osteomyelitis, who are followed until Day 180 ± 14 days (Visit 7), the only concomitant medications that must be recorded are new antibiotics that are prescribed for the treatment of osteomyelitis.	All relevant concomitant systemic medications from Screening (Visit 1) through Day 70 ± 7 days (Visit 6) must be recorded in the subject's medical record and on the eCRFs. Specific dosages or dose changes for concomitant medications do not need to be recorded in the eCRFs. After Visit 6, for subjects with osteomyelitis, who are followed until Day 180 ± 14 days (Visit 7), the only concomitant medications that must be recorded are new antibiotics that are prescribed for the treatment of osteomyelitis.	Update
**7.1.2 Assessment of Concomitant Medications/Treatments Other Than Study Product** Footnote	None	[12] As patients may receive medication prescriptions from multiple sources, patient-reported medications are intended to serve as the primary source for concomitant medications. For sites that have the ability to cross-query reported medications against prescription or medication administration records, this can be used as a secondary source but is not required.	Update
**7.2.1 Clinical Laboratory Evaluations**	Hematology: absolute and differential white blood cell (WBC) count, erythrocyte count, hemoglobin, hematocrit, platelet count, and red blood cell (RBC) indices (mean corpuscular volume, mean corpuscular hemoglobin, and mean corpuscular hemoglobin concentration).	Hematology: absolute and differential white blood cell (WBC) count, erythrocyte count, hemoglobin, hematocrit, and platelet count.	Update
**7.2.1 Clinical Laboratory Evaluations**	Vancomycin levels in patients on vancomycin, based on local standard of care.	Vancomycin levels in patients on vancomycin, based on local standard of care.	
**7.2.2.1 Laboratory Specimen Preparation, Handling, and Storage**	Plasma PK specimens will be processed and stored in a -70ºC freezer until time of shipment to the central PK laboratory for analysis.	Plasma PK specimens will be processed and stored in a -70ºC or below freezer until time of shipment to the central PK laboratory for analysis.	Clarification

Section	Previous Version (v3.0) Language	Change (v4.0) Language	Rationale
**8.1.1 Adverse Events (AEs)**	AEs and AESIs that meet reporting criteria and occur during the trial collection and reporting period will be documented appropriately regardless of relationship and will be followed through resolution.	AEs and AESIs that meet reporting criteria and occur during the trial collection and reporting period will be documented appropriately regardless of relationship and will be followed through resolution or through the end of the study, whichever occurs first.	Update
**8.1.1 Adverse Events (AEs)**	Please note that hospital admissions and/or medical/surgical procedures scheduled prior to consenting but occurring during the study should not be captured as AEs, but should be listed in the medical history if related to a pre-existing condition.	Please note that hospital admissions and/or medical/surgical procedures scheduled prior to study drug administration but occurring during the study should not be captured as AEs, but should be listed in the medical history if related to a pre-existing condition.	Update
**8.1.1 Adverse Events (AEs)**	An event would be considered as adequately captured in the study endpoint if it is accurately and fully represented by a protocol-defined reason for clinical failure (other than mortality) or relapse. Events represented by the study endpoints include all of the following:<br>• Worsening of signs and symptoms of complicated bacteremia or IE<br>• Persistent bacteremia<br>• Relapse of baseline bacteremia	An event would be considered as adequately captured in the study endpoint if it is accurately and fully represented by a protocol-defined reason for clinical failure (other than mortality) or relapse. Events represented by the study endpoints include all of the following:<br>• Worsening of signs and symptoms of complicated bacteremia, infectious endocarditis, metastatic infection, or progression of infection.<br>• Persistent bacteremia<br>• Relapse of baseline bacteremia or associated sites of metastatic infection	Update
**8.2.1 Adverse Events of Special Interest (AESIs)**	AESIs will be collected after first dose of study product (i.e., dalbavancin or SOC antibiotic) is given and until final study visit. AESIs will be recorded on the eCRF and entered into the EDC.	AESIs that are related to study product (i.e. dalbavancin or SOC antibiotic) will be collected after first dose of study product (i.e., dalbavancin or SOC antibiotic) is given and until final study visit. AESIs will be recorded on the eCRF and entered into the EDC.	Update

Section	Previous Version (v3.0) Language	Change (v4.0) Language	Rationale
**8.2.1 Adverse Events of Special Interest (AESIs)**	None	If an intravascular catheter-associated catheter related complication is related to the catheter or vascular access device itself and not to study medication being administered (e.g., catheter-associated thrombosis or catheter-associated infection), this should still be recorded as an adverse event of special interest but can be marked as not related to study drug.	Update
**8.3.2 Regulatory Reporting for Studies Conducted Under DMID Sponsored IND**	DMID will report an AE as a suspected unexpected only if there is evidence to suggest a causal relationship between the study intervention and the AE.	DMID will report an SAE as a Suspected Unexpected Serious Adverse event Reaction (SUSAR) only if there is evidence to suggest a causal relationship between the study intervention and the SAE and based on whether thate event is listed in the investigator brochure.	Update
**8.4 Type and Duration of Follow-up of Subjects after Adverse Events**	AEs will be assessed, and AEs that are CTCAE grade 3 and higher, as well as all AESIs, will be followed from initial recognition of the AE until resolution.	AEs will be assessed, and AEs that are CTCAE grade 3 and higher, as well as all AESIs, will be followed from initial recognition of the AE until resolution or through the end of the study, whichever occurs first.	Update
**Appendix B. Definitions**	Childbearing Potential: a woman is considered of childbearing potential unless post-menopausal [≥1 year of spontaneous amenorrhea] or permanently surgically sterilized [bilateral oophorectomy, salpingectomy, hysterectomy].	Childbearing Potential: a woman is considered of childbearing potential unless post-menopausal [≥1 year of spontaneous amenorrhea] or permanently surgically sterilized [bilateral oophorectomy, salpingectomy, hysterectomy, tubal ligation].	Update
**Appendix C. ARLG Bloodstream Infection Quality of Life Measure**	In the past 7 days, how often did you have nausea—that is, a feeling like you could vomit? (If never, skip to 10)	In the past 7 days, how often did you have nausea—that is, a feeling like you could vomit? (If never, skip to 9)	Update

20-0002 DOTS Protocol Summary of Changes v4.0 to v5.0

Section	Previous Version (v4.0) Language	Change (v5.0) Language	Rationale
Global	N/A	Typographical and formatting errors corrected throughout document.	Correction
All (Header)	Version 4.0	Version 5.0	Update
All (Header)	13 Jul 2022	13 Sep 2022	Update
Title Page	Draft or Version Number: v4.0	Draft or Version Number: v5.0	Update
Title Page	13 Jul 2022	13 Sep 2022	Update
**8.1.1 Adverse Events (AEs)**	The following events are captured as efficacy endpoints and are therefore excluded from AE reporting:<br>An event would be considered as adequately captured in the study endpoint if it is accurately and fully represented by a protocol-defined reason for clinical failure (other than mortality) or relapse. Events represented by the study endpoints include all of the following:<br>Worsening of signs and symptoms of complicated bacteremia, infectious endocarditis, metastatic infection, or progression of infection<br>Persistent bacteremia<br>Relapse of baseline bacteremia or associated sites of metastatic infection	None	Update

20-0002 DOTS Protocol Summary of Changes v5.0 to v6.0

Section	Previous Version (v5.0) Language	Change (v6.0) Language	Rationale
All (Header)	Version 5.0	Version 6.0	Update
All (Header)	13 Sep 2022	29 Sep 2023	Update
Title Page	Draft or Version Number: v5.0	Draft or Version Number: v6.0	Update
Title Page	13 Sep 2022	29 Sep 2023	Update
**10.5 Final Analysis Plan**	Results from primary and secondary endpoint analyses may be distributed by the SDCC to key study team members (protocol PIs, protocol statisticians, and other necessary study team members) after database lock and generation of all the CSR Tables, Listings, and Figures. These analyses may be used by the company collaborator for planning subsequent trials or by the lead principal investigator for manuscript development while the CSR is being finalized.	Results from primary and secondary endpoint analyses may be distributed by the SDCC to key study team members (protocol PIs, protocol statisticians, and other necessary study team members) after database lock and prior to the generation of all the CSR Tables, Listings, and Figures. These analyses may be used by the company collaborator for planning subsequent trials or by the lead principal investigator for manuscript and abstract development while the CSR is being finalized.	Update

CLINICAL RESEARCH IN INFECTIOUS DISEASES

STATISTICAL ANALYSIS PLAN
for
DMID Protocol: 20-0002
Study Title:

Dalbavancin as an Option for Treatment of *S. aureus* Bacteremia (DOTS): A Phase 2b, Multicenter, Randomized, Open-Label, Assessor-Blinded Superiority Study to Compare the Efficacy and Safety of Dalbavancin to Standard of Care Antibiotic Therapy for the Completion of Treatment of Patients with Complicated *S. aureus* Bacteremia

NCT04775953

Version 1.0

DATE: 03OCT2022

THIS COMMUNICATION IS PRIVILEGED AND CONFIDENTIAL

STUDY TITLE

**Protocol Number Code:**	**DMID Protocol: 20-0002**
**Development Phase:**	Phase 2b
**Products:**	Dalbavancin<br>Standard of Care Antibiotics: Cefazolin, nafcillin, oxacillin, vancomycin, and daptomycin
**Form/Route:**	IV
**Indication Studied:**	Complicated *S. aureus* Bacteremia
**Sponsor:**	Division of Microbiology and Infectious Diseases<br>National Institute of Allergy and Infectious Diseases<br>National Institutes of Health
**Clinical Trial Initiation Date:**	15MAR2021
**Clinical Trial Completion Date:**	TBD
**Date of the Analysis Plan:**	03OCT2022
**Version Number:**	Version 1.0

This study was performed in compliance with Good Clinical Practice.

Information contained in this publication is the property of Division of Microbiology and Infectious Diseases and is confidential. This information may not be disclosed to third parties without written authorization from Division of Microbiology and Infectious Diseases. This report may not be reproduced, stored in a retrieval system or transmitted in any form or by any means - electronic, mechanical, recording or otherwise - without the prior authorization from Division of Microbiology and Infectious Diseases. This document must be returned to Division of Microbiology and Infectious Diseases upon request.

TABLE OF CONTENTS

STUDY TITLE 2
TABLE OF CONTENTS 3
LIST OF ABBREVIATIONS 7
1. PREFACE 9
2. INTRODUCTION 10
2.1. Purpose of the Analyses 10
3. STUDY OBJECTIVES AND ENDPOINTS 11
3.1. Study Objectives 11
3.1.1. Primary 11
3.1.2. Secondary 11
3.1.3. Exploratory 11
3.2. Endpoints 12
3.2.1. Primary 12
3.2.2. Secondary 12
3.2.3. Exploratory 13
3.3. Study Definitions and Derived Variables 13
4. INVESTIGATIONAL PLAN 15
4.1. Overall Study Design and Plan 15
4.2. Discussion of Study Design, Including the Choice of Control Groups 15
4.3. Selection of Study Population 16
4.3.1. Inclusion Criteria 16
4.3.2. Exclusion Criteria 16
4.3.3. Reasons for Withdrawal 16
4.4. Treatments 18
4.4.1. Treatments Administered 18
4.4.2. Identity of Investigational Product(s) 18
4.4.3. Method of Assigning Subjects to Treatment Groups (Randomization) 18
4.4.4. Selection of Doses in the Study 18
4.4.5. Blinding 19
4.4.6. Prior and Concomitant Therapy 19
4.4.7. Treatment Compliance 19

Table of Contents (continued)

4.5. Efficacy and Safety Variables 20
5. SAMPLE SIZE CONSIDERATIONS 21
6. GENERAL STATISTICAL CONSIDERATIONS 22
6.1. General Principles 22
6.2. Timing of Analyses 22
6.3. Analysis Populations 22
6.3.1. Screened Analysis Population 23
6.3.2. Intent-to-Treat Analysis Population 23
6.3.3. Safety Population 23
6.3.4. Modified Intent-to-Treat Population 23
6.3.5. Clinically Evaluable Analysis Population 23
6.4. Covariates and Subgroups 24
6.5. Missing Data 24
6.5.1. DOOR Categories 24
6.5.2. Quality of Life Score (QoL) 25
6.5.3. Desirability of Outcome Ranking at Day 70 or Day 42 26
6.6. Interim Analyses and Data Monitoring 27
6.7. Multicenter Studies 27
6.8. Multiple Comparisons/Multiplicity 27
7. STUDY SUBJECTS 28
7.1. Disposition of Subjects 28
7.2. Protocol Deviations 28
8. EFFICACY EVALUATION 29
8.1. Primary Efficacy Analysis 29
8.1.1. Analysis of DOOR at Day 70 Using ITT Analysis Population 29
8.1.1.1. ITT Analysis of DOOR using IPW 29
8.1.1.2. ITT Analysis of DOOR using Multiple Imputation 31
8.2. Secondary Efficacy Analyses 33
8.2.1. Analysis of Clinical Efficacy at Day 70 using IPW for the ITT and mITT Analysis Populations 33
8.2.2. Sensitivity Analysis of Clinical Efficacy at Day 70 using Multiple Imputation for the ITT and mITT Analysis Populations 34

Table of Contents (continued)

8.2.3. Analysis of DOOR Components Using ITT Analysis Population 36
8.2.4. Analysis of Clinical Failure at Day 70 Using ITT Population 36
8.3. Exploratory Efficacy Analyses 37
8.3.1. Analysis of Clinical Efficacy at Day 42 37
8.3.2. Additional Analyses of DOOR 37
8.3.2.1. Analysis of DOOR at Day 42 Using ITT and mITT Analysis Populations 37
8.3.2.2. Subgroup Analyses of DOOR 37
8.3.3. Analysis of DOOR Components at Day 42 and Day 70 Using mITT Analysis Populations 38
8.3.3.1. Analysis of DOOR Categories at Day 42 and Day 70 Using ITT and mITT Analysis Populations 38
8.3.3.2. Distribution of DOOR by Treatment Group Using ITT and mITT Analysis Populations 38
8.3.3.3. Cumulative Difference in DOOR Categories Using ITT and mITT Analysis Populations 38
8.3.3.4. Analysis of Difference in Mean Partial Credit Using ITT and mITT Analysis Populations 38
8.3.3.5. Analysis of Expected DOOR Category Distribution and Expected Numbers Gained Loss Using ITT and mITT Analysis Populations 39
8.3.4. Analysis of Microbiological Success at Day 42 and Day 70 using the ITT and mITT Analysis Populations 39
8.3.5. Analysis of QoL Score using ITT and mITT Analysis Populations 39
8.3.6. Bivariate Analysis of DOOR Probability vs Difference in Mean of Change in QoL Score from Baseline 39
8.3.7. Analysis of Late Recurrence Within ITT Population with Osteomyelitis 40
8.4. Supplemental Efficacy Analyses 40
8.4.1. Analysis of DOOR at Day 42 and 70 Using CE Analysis Populations 41
8.4.2. Analysis of Clinical Efficacy at Day 70 Using CE Analysis Population 41
8.4.3. Analysis of DOOR at Day 42 and Day 70 Using CE Analysis Population 41
8.4.3.1. Distribution of DOOR by Treatment Group Using CE Analysis Population 42
8.4.3.2. Cumulative Difference in DOOR Using CE Analysis Population 42
8.4.3.3. Analysis of Difference in Mean Partial Credit Using CE Analysis Population 42
8.4.3.4. Analysis of Expected DOOR Distribution and Expected Numbers Gained Loss 42

PRIVILEGED AND CONFIDENTIAL COMMUNICATION

Table of Contents (continued)

8.4.4. Analysis of Microbiological Success at Day 42 and Day 70 using the CE Analysis Populations 42
8.5. Interim Analyses 42
8.6. Imputation of Missing Data 43
8.6.1. Multiple Imputation of Missing DOOR Day 42 and Day 70 43
8.6.2. Multiple Imputation (MI) of Missing Values of Clinical Efficacy and Microbiologic Success on Day 42 and Day 70 44
9. SAFETY EVALUATION 47
9.1. Demographic and Other Baseline Characteristics 47
9.1.1. Prior and Concurrent Medical Conditions 47
9.1.2. Prior or Concomitant Medications and Nondrug Interventions 47
9.2. Measurements of Treatment Compliance 48
9.3. Adverse Events 48
9.3.1. Treatment-Emergent Adverse Events 48
9.4. Deaths, Serious Adverse Events, and Other Significant Adverse Events 49
9.5. Pregnancies 49
9.6. Clinical Laboratory Evaluations 49
9.7. Vital Signs and Physical Evaluations 50
9.8. Concomitant Medications and Nondrug Interventions 50
10. PHARMACOKINETICS 51
11. OTHER ANALYSES 52
12. REPORTING CONVENTIONS 53
13. TECHNICAL DETAILS 54
14. SUMMARY OF CHANGES IN THE CONDUCT OF THE STUDY OR PLANNED ANALYSES 55
15. REFERENCES 56
16. LISTING OF TABLES, FIGURES, AND LISTINGS 57
APPENDICES 58
APPENDIX 1. TABLE MOCK-UPS 59
APPENDIX 2. FIGURE MOCK-UPS 137
APPENDIX 3. LISTINGS MOCK-UPS 152
APPENDIX 4. NCA TEMPLATE 174

LIST OF ABBREVIATIONS

ABSSSI	Acute Bacterial Skin and Skin Structure Infections
AE	Adverse Event
AESI	Adverse Event of Special Interest
ALT	Alanine Aminotransferase
ARLG	Antibacterial Resistance Leadership Group
AST	Aspartate Aminotransferase
BP	Blood Pressure
BUN	Blood Urea Nitrogen
CE	Clinically Evaluable
CI	Confidence Interval
CRF	Case Report Form
CrCl	Creatinine Clearance
DCC	Data Coordinating Center
DMID	Division of Microbiology and Infectious Diseases
DSMB	Data and Safety Monitoring Board
DOOR	Desirability of Outcome Ranking
DOTS	Dalbavancin as an Option for Treatment of *S. aureus* Bacteremia
eCRF	Electronic Clinical Report Form
EDC	Electronic Data Capture
ET	Early Termination
F	Fahrenheit
FDA	Food and Drug Administration
GEE	Generalized Estimating Equations
GLMM	Generalized Linear Mixed Model
HLGT	High Level Group Term
ICH	International Council for Harmonisation
IE	Infective Endocarditis
IPW	Inverse Probability Weighting
IRB	Institutional Review Board
ITT	Intent-to-Treat
L	Liter
MAR	Missing at Random
MCAR	Missing Completely at Random

List of Abbreviations (continued)

MedDRA	Medical Dictionary for Regulatory Activities
mEq	Milliequivalent
mg	Milligram
mITT	Modified Intent-to-Treat
mL	Milliliter
MNAR	Missing Not at Random
MRSA	Methicillin-resistant *Staphylococcus aureus*
MSSA	Methicillin-sensitive *Staphylococcus aureus*
MVUE	Minimum Variance Unbiased Estimator
NIAID	National Institute of Allergy and Infectious Diseases
NIH	National Institutes of Health
PCS	Potentially Clinically Significant
PD	Pharmacodynamics
PI	Principal Investigator
PID	Patient Identification
PIPS	Predicted Interval Plots
PK	Pharmacokinetics
PT	Preferred Term
PTime	Prothrombin Time
PTT	Partial Thromboplastin Time
PWID	Person Who Injects Drugs
SAE	Serious Adverse Event
SD	Standard Deviation
SDCC	Statistical and Data Coordinating Center
SMC	Safety Monitoring Committee
SOC	System Organ Class
SOP	Standard Operating Procedures
TEE	Transesophageal Echocardiography
TOC	Test of Cure
ULN	Upper Limit of Normal
WBC	White Blood Cell
WHO	World Health Organization

1. PREFACE

The Statistical Analysis Plan (SAP) for "Dalbavancin as an Option for Treatment of *Staphylococcus aureus* (*S. aureus)* Bacteremia (DOTS): A Phase 2b, Multicenter, Randomized, Open-Label, Assessor-Blinded Superiority Study to Compare the Efficacy and Safety of Dalbavancin to Standard of Care Antibiotic Therapy for the Completion of Treatment of Patients with Complicated *S. aureus* Bacteremia" (DMID Protocol 20-0002) describes and expands upon the statistical information presented in the protocol.

This document describes all planned analyses and provides reasons and justifications for these analyses. It also includes sample tables, listings, and figures planned for the final analyses. Regarding the final analyses and Clinical Study Report (CSR), this SAP follows the International Council for Harmonisation of Technical Requirements for Registration of Pharmaceuticals for Human Use (ICH) Guidelines, as indicated in Topic E3 (Structure and Content of Clinical Study Reports), and more generally is consistent with Topic E6 (Good Clinical Practice: Integrated Addendum to ICH E6(R2)), Topic E8 (General Considerations for Clinical Trials), Topic E9 (Statistical Principles for Clinical Trials), Topic E9 (R1: Addendum on Estimands and Sensitivity Analysis in Clinical Trials to the Guidelines on Statistical Principles for Clinical Trials) and Topic E10 (Choice of Control Group and Related Issues in Clinical Trials). The structure and content of the SAP provides sufficient detail to meet the requirements identified by the Food and Drug Administration (FDA) and ICH, while all work planned and reported for this SAP will follow internationally accepted guidelines published by the American Statistical Association and the Royal Statistical Society for statistical practice.

This document contains four sections: (1) a review of the study design, (2) general statistical considerations, (3) comprehensive statistical analysis methods for efficacy and safety outcomes, and (4) a list of proposed tables and figures. Within the table, figure, and listing mock-ups (Appendices 1, 2, and 3), references to CSR sections are included. Any deviation from this SAP will be described and justified in protocol amendments and/or in the CSR, as appropriate. The reader of this SAP is encouraged to also review the study protocol for details on conduct of the study and the operational aspects of clinical assessments.

PRIVILEGED AND CONFIDENTIAL COMMUNICATION

2. INTRODUCTION

This is a Phase 2b, multicenter, randomized, open-label, assessor-blinded superiority clinical trial of dalbavancin vs. standard of care antibiotic therapy for treating complicated *S. aureus* bacteremia. The study is designed and powered for the primary analysis of a comparison of the efficacy of dalbavancin to standard of care therapy for the completion of therapy in patients with complicated bacteremia or right-sided native valve Infective Endocarditis (IE) caused by *S. aureus* who have cleared their baseline bacteremia using a superiority approach.

Approximately 200 subjects will be randomized 1:1 to receive either dalbavancin or a standard of care antibiotic regimen that is based upon the identification and antibiotic susceptibility pattern of the baseline organism. Those randomized to the dalbavancin treatment group will receive 2 doses of dalbavancin intravenous (IV) 1 week apart (1500 mg on Day 1 and Day 8 after randomization, with renal dose adjustment if appropriate). Those subjects randomized to the standard of care antibiotic therapy treatment group will receive an antibiotic regimen considered to be standard of care based on the methicillin susceptibility pattern of the pathogen isolated at Baseline for a duration of 4 to 6 weeks. The duration of standard of care antibiotics may be extended to a maximum of 8 weeks at the discretion of the treating clinician.

2.1. Purpose of the Analyses

Analysis of Desirability of Outcome Ranking (DOOR) at Day 70 will be used to assess the efficacy of dalbavancin versus standard of care therapy. Superiority of dalbavancin versus standard of care on Day 70 based on DOOR will be the primary analysis. With DOOR being a composite endpoint, the advantage of dalbavancin on the DOOR analysis does not necessarily imply an advantage on all DOOR components. Thus, examination of the effects on the overall clinical outcome and each component of DOOR is standardly conducted via a secondary or sensitivity analysis. Hence, the secondary outcome measures which include a comparison of clinical outcomes, safety outcomes, and comparison of each individual DOOR component will be conducted. Note that DOOR categories will be calculated using cumulative data from Day 1 through Day 70, except that clinical success or failure is specific to the date of assessment. For example, suppose a participant had experienced an SAE and clinical failure at Day 42 but at Day 70 they were considered to be cured. In this case, the SAE would still contribute to the DOOR at Day 70; however, the change from clinical failure at Day 42 to clinical success at Day 70 would result in a lower (better) DOOR at Day 70, reflective of the improvement in clinical outcome.

3. STUDY OBJECTIVES AND ENDPOINTS

3.1. Study Objectives

3.1.1. Primary

To compare DOOR at Day 70 of dalbavancin to that of standard of care antibiotic therapy used to consolidate therapy for the treatment of subjects with complicated *S. aureus* bacteremia in the intent-to-treat (ITT) population.

3.1.2. Secondary

1. To compare the clinical outcomes of dalbavancin with the standard of care antibiotic therapy at Day 70 in the modified intent-to-treat (mITT) population (see Section 6.3 for definitions of study populations).
2. To compare the safety of dalbavancin with that of the standard of care treatment in the mITT population.
3. To compare each individual component of DOOR by treatment arm, in the ITT population.

3.1.3. Exploratory

1. To compare the clinical outcomes of dalbavancin with the standard of care antibiotic therapy at Day 70 in the Clinically Evaluable (CE) population (see Section 6.3 for definition).
2. To compare the DOOR endpoint of subjects on dalbavancin with that of subjects receiving standard of care antimicrobial therapy at Day 42, in the ITT, mITT and CE populations.
3. To compare the clinical and microbiologic outcomes of dalbavancin with the standard of care antibiotic therapy at Day 42 in ITT, mITT and CE populations.
4. To compare clinical and microbiologic outcomes of dalbavancin with standard of care antibiotic therapy between clinically important subgroups, including a) those with MSSA versus MRSA; b) persons who inject drugs (PWID) vs non-PWID; c) those who received infectious disease consultation vs those who did not; d) underlying site of infection (endovascular, bone and joint, skin/skin structure pulmonary); e) subjects with immune-suppression (not severe enough to trigger exclusion)[1]; f) divided by duration of initial bacteremia, in the ITT, mITT, and CE populations.
5. To compare Quality of Life (QoL) of subjects on dalbavancin with that of subjects receiving standard of care antibiotic therapy at Baseline, Day 42, and Day 70, in the ITT, mITT, and CE populations.

1 Defined as: On chemotherapy or immunotherapy for active hematologic malignancy expected to cause ANC < 500 cells/mm3 lasting > 7 days during the study period, chronic high dose oral steroids (equivalent of ≥ 20 mg prednisolone per day for or equivalent, for >2 weeks within the last month), HIV infection with a CD4 cell count < 100 cells/mm3 based on last known measurement or patient-reported value

6. To characterize the population pharmacokinetic (PK) profile for dalbavancin administered via a 2-dose regimen (1500 mg on day 1 and 1500 mg on day 8; renally adjusted when appropriate) in patients with *S. aureus* bacteremia.
7. To assess patient-level and clinical covariates associated with dalbavancin pharmacokinetics in patients with *S. aureus* bacteremia.
8. Examine the association between individualized plasma concentration profiles and clinical and microbiologic outcomes at Day 42 and Test of Cure (TOC).
9. Examine the association between individualized plasma concentration profiles and occurrence of adverse drug events, including AST/ALT elevations >3X upper limit of normal.
10. Examine the association between individualized plasma concentration profiles and late recurrence risk among the subset of patients with osteomyelitis and a 6-month follow-up visit.

3.2. Endpoints

3.2.1. Primary

The primary outcome measure is the DOOR endpoint at Day 70. The clinical components of the DOOR endpoint (success/failure and infectious complications) will be completed by an independent adjudication committee, blinded to treatment assignment. Day 70 was selected in this study as it occurs at least 4 weeks after treatment completion for most patients, allowing time for detection of relapse.

Clinical failure, infectious complications, and adverse events are each binary (yes/no) components of DOOR.

For the primary analysis of DOOR, QoL will be used as a tie-breaker and will be calculated as change from baseline QoL to Day 70 QoL score, as assessed by questions from the PROMIS physical function item bank (PROMIS Item Bank v2.0, short form 6b) on the Antibacterial Resistance Leadership Group (ARLG) Bloodstream Infection QoL Measure (Appendix C of the protocol).

3.2.2. Secondary

The **secondary efficacy outcome** is as follows.

Clinical efficacy, defined as none of 1) Clinical failure; 2) Infectious complications; 3) All-cause mortality

The **secondary safety outcome** is as follows.

Safety, defined as proportion of patients who have either 1) an SAE; or 2) an AE leading to study drug discontinuation

Each component of DOOR will also be examined separately:

- clinical failure
- infectious complications
- SAEs
- AEs leading to study drug discontinuation
- all-cause mortality

3.2.3. Exploratory

1. Clinical efficacy by treatment group in the CE population at Day 70.
2. DOOR by treatment group at Day 42 in the ITT, mITT, and CE populations.
3. Clinical and microbiologic outcomes by treatment group at Day 42 in the ITT, mITT, and CE populations. A microbiologic success will be defined as no post-randomization growth (e.g., no positive cultures) of the baseline pathogen from blood cultures or another sterile body site.
4. Clinical and microbiologic outcomes by treatment group, within each subgroup of clinical interest, at Day 42 and Day 70 in the ITT, mITT, and CE populations.
5. QoL at Baseline, Day 42, and Day 70, which will be assessed by the score obtained from questions from the PROMIS physical function item bank (PROMIS Item Bank v2.0, short form 6b), included in the ARLG Bloodstream Infection QoL Measure (Appendix C of the protocol), as well as two additional comparator measures (EQ-5D-5L - https://euroqol.org/eq-5d-instruments/sample-demo/ and PROMIS Global Health short form - http://www.healthmeasures.net/administrator/components/com_instruments/uploads/Global%20Health%20Scale%20v1.2%2008.22.2016.pdf) in the ITT, mITT, and CE populations.
6. Population mean PK parameter estimates and the magnitude of the associated inter individual variability for the 2-dose dalbavancin regimen in patients with *S. aureus* bacteremia.
7. Individual post hoc PK parameter estimates and calculated exposure measures for the 2-dose dalbavancin regimen in patients with *S. aureus* bacteremia.
8. Clinical and microbiologic response at Day 42 and Day 70 according to individual plasma dalbavancin concentration curves.
9. Occurrence of grade 3 or higher adverse drug events, adverse events of special interest (AESIs), and occurrence of AST/ALT elevations >3x upper limit of normal (ULN) from first dose of dalbavancin through follow-up period.
10. Late recurrence within the osteomyelitis population will be defined by the presence of the following up to 6 months after randomization: progressive imaging changes along with isolation of *S. aureus* from blood, bone biopsy, associated fluid aspiration, or operative tissue.

3.3. Study Definitions and Derived Variables

DOOR:

There are 5 possibilities for the DOOR:

Rank 1: Alive without any of the following: (1) evidence of clinical failure; (2) an infectious complication; or (3) any SAE, or an AE leading to study drug discontinuation

Rank 2: Alive but with one of the following: (1) evidence of clinical failure; (2) an infectious complication; or (3) any SAE, or an AE leading to study drug discontinuation

Rank 3: Alive but with two of the following: (1) evidence of clinical failure; (2) an infectious complication; or (3) any SAE, or an AE leading to study drug discontinuation

Rank 4: Alive but with all of the following: (1) evidence of clinical failure; (2) an infectious complication; or (3) any SAE, or an AE leading to study drug discontinuation

Rank 5: Death

Note that if an infectious complication is also an SAE, or an AE leading to study drug discontinuation the event will count twice towards the DOOR. For example, if a participant is alive at Day 70 point and the only observed event between Day 1 and Day 70 is a serious infectious complication, the participant will have Rank 3 at Day 70. Additionally, DOOR is calculated using cumulative data from Day 1 through Day 70, except that clinical success or failure is specific to the date of assessment.

Additional details for deriving DOOR, including handling of missing data, are provided in Section 6.5.

Clinical Success: Resolution of clinical signs and symptoms of *S. aureus* bacteremia such that no additional antibiotic therapy is required or anticipated for its treatment.

Note that it is possible to achieve this overall Clinical Success status at Day 70 even if infectious complications have occurred prior to that time. For example, a patient who has a new metastatic focus of infection diagnosed after randomization, but who subsequently completes treatment and is felt to be cured at Day 70 would be considered a Clinical Success (and the Infectious Complication would result in a lower DOOR). That is, Clinical Success reflects the patient's overall status at the time of that assessment.

Determination of clinical success/failure and infectious complications at Day 42 and Day 70 will be determined by the adjudication committee after review of all relevant data up to Day 42 and Day 70, respectively.

Clinical Failure: Absence of clinical success

Infectious Complications: Occurrence of any of the following, between randomization and Day 70:

- Endocarditis
- New evidence of metastatic foci of infection – e.g., osteomyelitis, visceral abscess, septic joint
- Relapse – isolation of baseline *S. aureus* pathogen from a blood culture drawn after randomization
- Readmission for subsequent care of indication under study
- Need for additional unplanned source control procedures – e.g., abscess debridement or drainage, cardiac valve replacement
- Change in antibiotic therapy due to inadequate clinical response. For any changes to study drug in the standard of care group, or when new antibiotics are started in either treatment group, the site PI will record the reason for the antibiotic change.

Microbiologic Success: No post-randomization growth (e.g., no positive cultures) of the baseline pathogen from blood cultures or another sterile body site. Subjects who discontinue from the study before Day 42 and have no post-randomization growth will have missing microbiologic success.

PRIVILEGED AND CONFIDENTIAL COMMUNICATION

4. INVESTIGATIONAL PLAN

4.1. Overall Study Design and Plan

This is a Phase 2b, multicenter, randomized, open-label, assessor-blinded, superiority study to compare dalbavancin to standard of care antibiotic therapy for the completion of therapy in patients with complicated bacteremia or right-sided native valve IE caused by *S. aureus* who have cleared their baseline bacteremia.

Approximately 200 subjects will be randomized 1:1 to receive either dalbavancin or a standard of care antibiotic regimen that is based upon the identification and antibiotic susceptibility pattern of the baseline organism. Those randomized to the dalbavancin treatment group will receive 2 doses of dalbavancin IV 1 week apart (1500 mg on Day 1 and Day 8 after randomization, with renal dose adjustment if appropriate). Those subjects randomized to the standard of care antibiotic therapy treatment group will receive an antibiotic regimen considered to be standard of care based on the methicillin susceptibility pattern of the pathogen isolated at Baseline for a duration of 4 to 6 weeks. The duration of standard of care antibiotics may be extended to a maximum of 8 weeks at the discretion of the treating clinician.

Figure 1 of the protocol provides a schematic of the study design. Study procedures are presented in Section 6 of the study protocol. Detailed descriptions of each study visit can be found in Section 6.3 of the protocol.

The primary outcome measure is the superiority of dalbavancin versus standard of care therapy on Day 70 based on DOOR using the ITT population. Secondary endpoints include comparison of clinical efficacy and clinical safety with clinical efficacy measured by clinical failure, infectious complications, and all-cause mortality while clinical safety is measured by the proportion of SAEs and AEs leading to study drug discontinuation.

4.2. Discussion of Study Design, Including the Choice of Control Groups

The current standard of care for the antibiotic treatment of complicated bacteremia or IE uses a stepwise approach. The initial phase of treatment involves the initiation of empirical antibiotic therapy, definitive diagnosis (as per the modified Duke criteria), and the assessment of the need for early valve replacement, if applicable. Subsequent identification of the causative pathogen, including antibiotic susceptibility and MICs supports the choice of definitive antibiotic therapy and determination of the required duration of antibiotic treatment. Guidelines for the use of outpatient parenteral antibiotic therapy in the treatment of complicated bacteremia or IE similarly advocate that antibiotic therapy can be divided into an initial phase during which life-threatening complications of complicated bacteremia or IE are likely to occur (approximately 14 days) and a completion phase of therapy (2 weeks to 6 weeks) [9].

The proposed clinical study design of dalbavancin in the treatment of complicated bacteremia or IE is consistent with this standard of care. Specifically, prior to study eligibility, patients will receive pre-randomization antibiotic therapy pending a definitive diagnosis of complicated *S. aureus* bacteremia or IE, as well as the resolution of bacteremia. Eligible patients will then be randomized into the study to complete their antibiotic therapy with either a 2-dose regimen of dalbavancin or the current standard of care with daily IV administration of antibiotic therapy for a total duration of 4 to 6 weeks [10] for most patients, and up to 8 weeks for patients with osteomyelitis/discitis.

The proposed clinical study design offers several advantages. First, it will support enrollment of patients with a confirmed diagnosis of complicated bacteremia or IE. Prior studies have been limited by the inability to confidently determine complicated vs uncomplicated status prior to randomization [13]. Second, the proposed study design addresses a true area of need in *S. aureus* bacteremia management and reflects the likely pattern of "real world" dalbavancin use by clinicians for the completion of systemic antibiotic therapy for complicated bacteremia or IE without the need for indwelling IV access to support daily therapy. This takes full advantage of the unusual PK profile of dalbavancin and the introduction of this therapy into clinical practice would potentially have a major impact on patient well-being and QoL. In this study, presence or absence of these potential QoL effects will be assessed using an ARLG Bloodstream Infection QoL Measure developed specifically for this purpose [14 and 15]. Additionally, two previously validated measures (EQ-5D-5L, https://euroqol.org/eq-5d-instruments/sample-demo/ and the PROMIS Global Health short form, http://www.healthmeasures.net/administrator/components/com_instruments/uploads/Global%20Health%20Scale%20v1.2%2008.22.2016.pdf) will also be collected. Third, adequate treatment of complicated bacteremia or IE requires prolonged systemic antibiotic therapy to prevent relapse. Introduction of the 2-dose dalbavancin regimen may decrease the risk of relapse. Finally, the proposed design of this clinical study is consistent with antibiotic stewardship principles, reserving dalbavancin therapy for patients with fully characterized infections and pathogens.

4.3. Selection of Study Population

The study will enroll approximately 200 eligible subjects to the study in the United States and Canada. Eligible subjects are adults 18 years of age or older who have been diagnosed with complicated bacteremia or right-sided IE due to *S. aureus*, have been treated with appropriate empiric/targeted antibiotic therapy, and in whom the blood cultures have tested negative after at least 72 hours of initial antibiotic therapy (maximum 10 days). Subjects with uncomplicated bacteremia due to *S. aureus* will be excluded.

Subject Inclusion and Exclusion Criteria must be confirmed by a study investigator listed on the Form FDA 1572. No exemptions are granted on Subject Inclusion/Exclusion Criteria in DMID-sponsored studies. Clarifications regarding applicability of specific inclusion and exclusion criteria may be discussed with a protocol clinician. Questions about eligibility will also be directed toward the DMID Medical Officer.

4.3.1. Inclusion Criteria

For a list of inclusion criteria, see the most recent version of the Protocol.

4.3.2. Exclusion Criteria

For a list of exclusion criteria, see the most recent version of the Protocol.

4.3.3. Reasons for Withdrawal

Subject Withdrawal:

Subjects may voluntarily withdraw their consent for study participation at any time without penalty or loss of benefits to which they are otherwise entitled.

If a subject withdraws or is withdrawn prior to completion of the study, the reason for this decision must be recorded in the case report forms (CRFs).

PRIVILEGED AND CONFIDENTIAL COMMUNICATION

The reasons to withdraw from the study might include, but are not limited to, the following:

- Subject no longer meets eligibility criteria
- Subject withdraws consent
- Subject lost to follow-up
- Subject becomes non-compliant
- Medical disease or condition, or new clinical finding(s) for which continued participation, in the opinion of the investigator might compromise the safety of the subject, interfere with the subject's successful completion of this study, or interfere with the evaluation of responses
- Study or site prematurely terminated by the sponsor for any reason

Discontinuation of Treatment:

An investigator may also discontinue a subject from receiving the study product for any reason. Follow-up safety evaluations for discontinued subjects will be conducted if the subject agrees.

The reasons to discontinue study product might include, but are not limited to, the following:

- Subject meets individual halting criteria (see Protocol)
- Subject becomes pregnant, if applicable
- Occurrence of an AE that, in the opinion of the investigator, warrants the subject’s permanent discontinuation from IV study drug
- Subject has an insufficient therapeutic response to study drug (i.e., lack of efficacy for SAB). A patient who does not show signs of improvement despite treatment with study drug for an appropriate length of time or a patient who shows signs of clinical worsening at any time may be prematurely discontinued from study drug therapy and treated with salvage therapy as directed by their treating clinician. These patients would continue to be followed in the study unless withdrawn for another reason.

A subject who is prematurely discontinued from study drug or withdrawn from the study should have the assessments for Early Termination (ET) as detailed in the Schedule of Events (Table 1). A clear description of reason for early withdrawal or discontinuation from investigational product must be documented. The reasons for early withdrawal or premature discontinuation from study drug will be reflected on the relevant disposition page of the electronic case report form (eCRF).

The investigator should be explicit regarding study follow-up (e.g., safety and efficacy follow-up) that might be carried out despite the fact the subject will not receive further study product. If the subject consents, every attempt will be made to follow all AEs through resolution. The procedures that collect safety data for the purposes of research must be inclusive in the original informed consent.

The investigator will inform the subject that already collected data will be retained and analyzed even if the subject withdraws from this study.

Subjects who withdraw, or are withdrawn from this study, or are lost to follow-up after signing the informed consent form (ICF) and administration of the study product will not be replaced. Subjects who withdraw, or are withdrawn from this study, or are lost to follow-up after randomization but before administration of the study product will not be replaced.

4.4. Treatments

4.4.1. Treatments Administered

Subjects will be randomized to either receive dalbavancin or standard of care antibiotics. Dalbavancin 1500 mg will be administered IV over 30 (± 10) minutes on Day 1 and Day 8, renally dose-adjusted to 1125 mg for subjects with Creatinine Clearance (CrCl) <30 or not on dialysis.

Standard of care antibiotics will be administered based on the methicillin susceptibility pattern of the pathogen isolated at baseline. Subjects with methicillin-sensitive S. aureus (MSSA) will receive nafcillin (2 g IV q4h × 4-6 weeks) OR oxacillin (2 g IV Q4h x 4-6 weeks) OR cefazolin (2 g IV q8h × 4-6 weeks) while subjects with methicillin-resistant S. aureus (MRSA) will receive vancomycin (dose per local standard of care × 4-6 weeks) OR daptomycin (6-10 mg/kg IV daily × 4-6 weeks).

4.4.2. Identity of Investigational Product(s)

Dalbavancin is a lyophilized, white to off-white to pale yellow solid. It is a lipoglycopeptide synthesized from a fermentation product of *Nonomuraea* species. Dalbavancin is supplied in clear glass vials as a sterile, lyophilized, preservative-free, white to off-white to pale yellow solid. Each vial contains dalbavancin HCl equivalent to 500 mg of dalbavancin.

Investigational dalbavancin vials will be labeled according to manufacturer or regulatory specifications and include the statement “Caution: New Drug – Limited by Federal Law to Investigational Use.” The dispensed study product (IV bags) will be labeled with the cautionary statement “For Investigational Use Only.”

The standard of care antibiotics (Cefazolin, nafcillin, oxacillin, vancomycin, and daptomycin) will be prepared and labeled in accordance with the clinical site pharmacy’s standard operating procedures (SOPs).

4.4.3. Method of Assigning Subjects to Treatment Groups (Randomization)

Once consented and upon entry of demographic data and confirmation of eligibility for the trial, the subject will be enrolled. Enrollment of subjects will be done online using the enrollment module of Advantage eClinical. Subjects will be randomized 1:1 to dalbavancin or standard of care. Randomization will be stratified based on screening pathogen, MSSA vs. MRSA.

The list of randomized treatment assignments will be prepared by statisticians at the Statistical and Data Coordinating Center (SDCC) (The Emmes Company). Emmes will assign each subject a treatment code and treatment assignment from the list after demographic and eligibility data have been entered.

4.4.4. Selection of Doses in the Study

The dalbavancin dosing regimen consists of 1500 mg on Day 1 and 1500 mg on Day 8 for subjects with normal renal function (i.e., CrCl >= 30 mL/min) or who are receiving regular hemodialysis or peritoneal dialysis, administered over 30 minutes by IV infusion. Patients with CrCl <30 mL/min who are not receiving regular hemodialysis or peritoneal dialysis will receive a reduced dose (1125mg on Day 1 and 1125 mg on Day 8). Based on a comparison to the updated nonclinical pharmacokinetic /pharmacodynamics (PD) target of the area under the unbound drug concentration-time curve [fAUC]/MIC ([11]), this regimen is expected to provide sufficient therapeutic concentrations of free drug against *S. aureus* through Day 42.

4.4.5. Blinding

Study subjects and treating physicians will not be masked to treatment, as this is an open label study. Treatment group will be masked for study adjudicators.

4.4.6. Prior and Concomitant Therapy

Medication history during the 30 days prior to ICF signing will be recorded at Screening (Visit 1) in the eCRF. Thereafter, any changes in concomitant medications or new medications added will be recorded in the eCRF.

Any systemic medication taken by the subject, other than study drugs, is considered a concomitant medication. Topical medications including eye drops, ear drops, or dermatologic treatments do not need to be recorded in the eCRFs. All concomitant systemic medications from Screening (Visit 1) through Day 70 ± 7 days (Visit 6) must be recorded in the subject's medical record and on the eCRFs. After Visit 6, for subjects with osteomyelitis, who are followed until Day 180 ± 14 days (Visit 7), the only concomitant medications that must be recorded are new antibiotics that are prescribed for the treatment of osteomyelitis.

At each visit the investigator will obtain information on any therapeutic interventions (e.g., drug and nondrug therapy or surgery) provided. Subjects may not participate in any other antibiotic treatment trials or interventional studies involving non-FDA approved investigational products concomitantly while in this study.

Concomitant systemic antibacterials (other than dalbavancin or comparator study drug) for adjunctive therapy of the subject's *S. aureus* bacteremia are prohibited during the study, up to Day 70. This includes concomitant treatment with an aminoglycoside.

Patients who require additional therapy due to inadequate clinical response will be assessed as having lack of efficacy of study drug.

- Where possible, antibiotic treatment of intercurrent infections should be done with antibiotics that are not active against the patient's *S. aureus* isolate. Discussion with the DMID Medical Officer is encouraged before or within 24 hours of initiation of concomitant antibiotics for another indication. Exceptions include Vancomycin oral 125 mg up to 500mg every 6 hours may be used in both treatment groups for the treatment of *Clostridioides difficile (C. difficile)* infections and may be continued as required throughout the duration of the study. The sponsor will not provide oral vancomycin.
- Metronidazole IV or oral 500 mg every 8 hours may be used in both treatment groups for the treatment of *C. difficile* infections and may be continued as required throughout the duration of the study. The sponsor will not provide metronidazole.
- Other antibacterials that do not achieve therapeutic levels in the serum (e.g., nitrofurantoin) may be considered. Consultation with the DMID Medical Officer is advised before use of these antibiotics.

4.4.7. Treatment Compliance

Dalbavancin will be administered under the supervision of investigative site personnel, and infusion date, start, and stop time will be documented in the eCRF, as well as any infusion interruptions.

PRIVILEGED AND CONFIDENTIAL COMMUNICATION

4.5. Efficacy and Safety Variables

The primary efficacy variable for this study is DOOR at Day 70. Secondary analyses will analyze DOOR components measured by clinical failure, infectious complications, and AE leading to study drug discontinuation. Quality of life score will be used as a tie-breaker for DOOR analysis.

Safety will be assessed by the frequency of SAEs, AESIs, and the frequency of AEs leading to study drug discontinuation in each treatment group.

5. SAMPLE SIZE CONSIDERATIONS

The study is powered for a superiority comparison based on the primary objective, a comparison of DOOR. The probability of a subject from the dalbavancin arm having a superior DOOR relative to a subject from the standard of care arm will be calculated along with a 95% confidence interval. Superiority will be considered to have been achieved if the 95% confidence interval for probability of having a superior DOOR with dalbavancin does not cross 50%. If the confidence interval crosses 50% however, the null hypothesis cannot be rejected.

Sample size was calculated based on the primary hypothesis. Assuming a 65% probability of a better DOOR in the dalbavancin treatment group versus the standard of care treatment group, with a 90% power and alpha=0.025 (by one-sided Wilcoxon rank sum test), 78 participants would be required in each treatment group. To allow for some inflation assuming around 12% of missing data or other study imperfections, using the method described in Lachin, et al, [12] we plan to recruit 100 per arm (200 subjects in total). Sample size was calculated using nQuery (MTT1-1 Module) (Version 8, Statistical Solution Ltd).

6. GENERAL STATISTICAL CONSIDERATIONS

6.1. General Principles

All continuous variables will be summarized using the following descriptive statistics: n (non-missing sample size), mean, standard deviation, median, maximum, and minimum. The frequency and percentages (based on the non-missing sample size) of observed levels will be reported for all categorical measures. In general, all data will be listed, sorted by treatment and subject, and when appropriate by visit number within subject. All summary tables will be structured with a column for each treatment group in the following order:

- Dalbavancin
- Standard of Care

All summary tables will be annotated with the total population size relevant to that table/treatment, including any missing observations.

6.2. Timing of Analyses

There will be one planned interim analysis for futility after approximately 50% of subjects have completed the trial. The interim analysis for futility will be performed by the SDCC and will consist of a quantitative evaluation of potential effect sizes and associated precision using a predicted intervals and predicted interval plots (PIPS) approach [7 and 8]. The results of the interim analysis will be presented in the closed session of the Data and Safety Monitoring Board (DSMB).

The DSMB will evaluate safety annually; however, ongoing review and summary of subject safety will occur to allow for early detection of a safety signal that may result from an AE or lack of efficacy of study drug. The DSMB will advise DMID on whether to continue, modify, or terminate the trial based on a risk-benefit assessment.

The final analysis will be performed after database lock.

6.3. Analysis Populations

The primary analysis will be done using the ITT analysis population. Other analyses might use mITT and/or CE analysis populations. Analyses using ITT and mITT will include imputations for missing values using multiple imputation or adjusting for missing data using IPW for DOOR, clinical efficacy, and microbiologic success.

Reasons for exclusion from the screened analysis population are summarized in Table 8 while reasons for exclusions from the rest of the analysis populations (ITT, Safety, mITT, CE) are summarized in Table 4 by treatment group. Individual subject listing of exclusion reasons is also provided in Listing 5. Excluded subjects might satisfy multiple criteria justifying their exclusion but will have only one reason indicated in Table 4 and Listing 5. The exclusion reason indicated will be determined by first exclusion reason met based on the following rules in the order they are listed for each analysis population.

Screened Population Exclusions:

- Subject did not complete the Screening Visit (Visit 1)

ITT Population Exclusions:

- Subject not randomized to receive study product

Safety Population Exclusions:

- Subject not treated with at least one dose of study product

Modified ITT Population Exclusions:

- Subject was excluded from ITT population
- Subject not treated with at least one dose of study product

Clinically Evaluable at Day 42 Population Exclusions:

- Subject was excluded from mITT population
- Subject has missing data or major protocol violation preventing the adjudication committee from evaluating their outcomes at Day 42.

Clinically Evaluable at Day 70 Population Exclusions:

- Subject was excluded from mITT population
- Subject has missing data or major protocol violation preventing the adjudication committee from evaluating their outcomes at Day 70.

6.3.1. Screened Analysis Population

The screened population will consist of all patients who undergo the Screening Visit (Visit 1), signed informed consent, and receive a Patient Identification (PID) number. This population will include both enrolled and not enrolled subjects.

6.3.2. Intent-to-Treat Analysis Population

The intent-to-treat (ITT) population will consist of all randomized patients regardless of whether or not they received study treatment. This analysis population will be used for primary, secondary, and some exploratory analyses. Patients will be analyzed based on the treatment they were randomized to.

6.3.3. Safety Population

The safety population will consist of all randomized patients who received at least 1 dose of study drug. This population will be used for all safety analyses, and patients will be analyzed based on the treatment received.

6.3.4. Modified Intent-to-Treat Population

The modified intent-to-treat (mITT) population will consist of all patients in the ITT population who received at least one dose of study drug. This analysis population will be used for secondary and some exploratory analyses. Patients will be analyzed based on the treatment received.

6.3.5. Clinically Evaluable Analysis Population

The CE populations will consist of all patients in the mITT population who met criteria for clinical evaluability. Patients will be considered clinically evaluable at Day 70 if they have a primary outcome assessment of DOOR at Day 70 and do not have missing data or major protocol violations that prevent the

adjudication committee from evaluating their outcomes at Day 70. Similarly, clinically evaluable at Day 42 if they have a primary outcome assessment of DOOR at Day 42 and do not have missing data or major protocol violations that prevent the adjudication committee from evaluating their outcomes at Day 42. These analysis populations will be used for exploratory analyses and patients will be analyzed based on the treatment received.

6.4. Covariates and Subgroups

Subgroup analyses comparing clinical and microbiologic outcomes will be conducted and will include a) those with MSSA versus MRSA; b) persons who inject drugs (PWID) vs non-PWID; c) those who received infectious disease consultation vs those who did not; d) underlying site of infection (endovascular, bone and joint, skin/skin structure pulmonary); e) subjects with immune-suppression[2] (Yes vs. No); f) duration of initial bacteremia (<2, 2-4, >4 days). Note that, if the number of subjects in a subgroup category is less than 5 for at least one of the treatment groups, that subgroup category will be combined with the next subgroup category with the least number of subjects to form a combined subgroup category with that has at least 5 subjects for at least one of the study groups. However, if a subgroup only has two categories (Yes and No for example), the subgroup category with less than 10 subjects will be excluded from the analyses.

6.5. Missing Data

While all efforts will be made to minimize missing data, some missing data are expected. Whenever possible, subjects terminating from the study early will be given an early termination visit during which the available components of DOOR and related measures can be recorded. The analyses of DOOR, clinical efficacy, and microbiological success for ITT and mITT analyses populations will use Inverse Probability Weighing (IPW) and multiple imputation with linear models to impute values using available information (treatment, randomization strata variables, and available visit information), assuming a missing at random (MAR) model.

The effect that any missing data might have on results will be assessed via sensitivity analysis. If the pattern of missing data are different to that envisaged at the design stage, further sensitivity analyses will be provided that are tailored to the missing data pattern observed.

6.5.1. DOOR Categories

Subjects will be grouped into the five categories based their clinical outcomes. The clinical components of DOOR which include clinical failure, infectious complication, SAE or AE leading to study drug discontinuation will be determined by an independent adjudication committee. DOOR at Day 70 will be defined as follows:

1. If a subject died at any point prior or on to Day 70, then the DOOR at Day 70 will be **Rank 5**.

2 Defined as: On chemotherapy or immunotherapy for active hematologic malignancy expected to cause ANC < 500 cells/mm3 lasting > 7 days during the study period, chronic high dose oral steroids (equivalent of ≥ 20 mg prednisolone per day for or equivalent, for >2 weeks within the last month), HIV infection with a CD4 cell count < 100 cells/mm3 based on last known measurement or patient-reported value

2. If a subject is alive but has at least one of clinical failure at Day 70, infectious complication, any SAE (except for death), or an AE leading to study drug discontinuation prior or on Day 70, then the DOOR at Day 70 will be **2, 3 or 4**, depending on how many events the subject experienced.
   - Rank 2: Alive but with one of the following: (1) evidence of clinical failure at the specified time point; (2) an infectious complication by the specified time-point; or (3) any SAE, or an AE leading to study drug discontinuation by the specified time-point
   - Rank 3: Alive but with two of the following: (1) evidence of clinical failure at the specified time point; (2) an infectious complication by the specified time-point; or (3) any SAE, or an AE leading to study drug discontinuation by the specified time-point
   - Rank 4: Alive but with all of the following: (1) evidence of clinical failure at the specified time point; (2) an infectious complication by the specified time-point; or (3) any SAE, or an AE leading to study drug discontinuation by the specified time-point
3. Even if the adjudication committee does not have sufficient evidence to determine clinical failure, if a subject is alive but with any event of infectious complication, any SAE (except for death), or an AE leading to study drug discontinuation by Day 70, then DOOR at Day 70 will be **Rank 3 or 4**, depending on how many events the subject experienced by Day 70.
   - Rank 3: Alive but with one of the following: (2) an infectious complication; or (3) any SAE, or an AE leading to study drug discontinuation
   - Rank 4: Alive but with both of the following: (2) an infectious complication; or (3) any SAE, or an AE leading to study drug discontinuation
4. If a subject is alive (a) with no event of infectious complication, any SAE (except for death), or an AE leading to study drug discontinuation, (b) but the adjudication committee does not have sufficient evidence to determine clinical failure, then the DOOR will be **Rank 2**.
5. If a subject is withdrawn from the study before any assessment (no assessments on any components of DOOR after randomization), then the DOOR will be **missing**.

A similar algorithm will be used to determine DOOR at Day 42.

6.5.2. Quality of Life Score (QoL)

Three types of instruments will be used to assess qualify of life: questions from the PROMIS physical function item bank (PROMIS Item Bank v2.0, short form 6b) on the ARLG Bloodstream Infection QoL Measure, the full PROMIS Global Health short form, and the EQ-5D-5L. For the QoL assessments that utilize PROMIS questions the HealthMeasures Scoring Service (https://assessmentcenter.net/ac_scoringservice) will be used to obtain the final QoL score for each subject for a given item of the quality-of-life instrument and for each assessed timepoint. HeathMeasures is a free publicly available software which requires the user to submit subject responses. No data are stored or saved by this service, and no identifying information is submitted. Even so, as an extra precaution each subject will be assigned a dummy identifier with their corresponding responses. The patient ID assigned by eClinical will not be shared.

A change in QoL from baseline at Day 70 (or Day 42) is calculated by taking the QoL score at Day 70 (or Day 42) minus QoL score at baseline.

- **PROMIS Global Health short form**
  - A value of 5 represents an excellent QoL outcome while a value of 1 represents a poor QoL outcome using the PROMIS Global Health short form.
  - Higher QoL scores represent a better QoL outcome.
  - Higher change in QoL scores from baseline represents a better QoL outcome.
- **ARLG Bloodstream Infection QoL Measure**
  - A value of 5 represents an excellent QoL outcome while a value of 1 represents a poor QoL outcome using the ARLG Bloodstream Infection QoL Measure.
  - Higher QoL scores represent a better QoL outcome.
  - Higher change in QoL scores from baseline represents a better QoL outcome.
- **EQ-5D-5L**
  - A value of 1 represents an excellent QoL outcome while a value of 5 represents a poor QoL outcome using the EQ-5D-5L instrument.
  - Lower QoL scores represent a better QoL outcome
  - Lower change in QoL scores from baseline represents better outcomes.

A subject could have missing data for QoL if a subject missed a visit and/or responses to all the items of the QoL form are missing.

The change in the ARLG Bloodstream Infection QoL from baseline to Day 70 (or Day 42) will be utilized as a tie breaker for DOOR calculations as described in Section 6.5.3 below. Please note that for tie breaking, the standardized score is obtained from the questions arising from the PROMIS physical function item bank (PROMIS Item Bank v2.0, short form 6b) item bank on the ARLG Bloodstream Infection QoL.

6.5.3. Desirability of Outcome Ranking at Day 70 or Day 42

The QoL-adjusted DOOR considers QoL as a tie breaker when calculating the DOOR probability. This is accomplished by using the standardized score from the questions from the PROMIS physical function item bank (PROMIS Item Bank v2.0, short form 6b) item bank on the ARLG Bloodstream Infection QoL (higher QoL is better) as a tie-breaker for subjects with the same DOOR. However, if both subjects have a DOOR of 5, the change in QoL will not be used as a tie-breaker. If change in QoL cannot be calculated due to missing data, we proceed as follows:

- If two subjects have the same DOOR at Day 70 but the QoL is missing for one of the subjects at that timepoint then the subject with missing QoL will be ranked below the subject with non-missing QoL.

- If two subjects have the same DOOR at Day 70 but the QoL is missing for both of the subjects at that timepoint then they will have the same rank and will be indistinguishable for the primary endpoint analysis.

The tie-breaking algorithm to obtain the QoL-Adjusted DOOR from DOOR and change in QoL is implemented as follows. First, subjects are ranked based on their DOOR and then by their change in QoL. Next, the QoL-Adjusted DOOR is calculated from the rank based on the subjects DOOR and the change in QoL. If no two subjects have the same DOOR and change in QoL, then the QoL-Adjusted DOOR is the same as the rank. For subjects with tied DOOR and tied change in QoL the QoL-Adjusted DOOR is calculated as the mean of their ranks. The QoL-Adjusted DOOR, obtained after the tie-breaking algorithm, will be denoted as R_i. This algorithm is exemplified below.

Subject	DOOR	Change in QOL
A	1	10
B	1	-10
C	2	5
D	1	-5
E	2	-5
F	2	5
G	5	NA
H	5	NA

→

DOOR	Subject	Change in QOL	QoL-Adjusted DOOR
1	A	10	1
1	D	-5	2
1	B	-10	3
2	C	5	4.5
2	F	5	4.5
2	E	-5	6
5	G	NA	7.5
5	H	NA	7.5

Since subjects A, D, and B have a DOOR of 1, the change in QOL is used as a tie-breaker among these subjects and the QoL-Adjusted DOOR is 1, 2, and 3 for subjects A, D, and B, respectively. Similarly, since subjects C, F, and E all have a DOOR of 2, the change in QOL is used as a tie-breaker among these 3 subjects. Notice that since subjects C and F have the same change in QOL their QoL-Adjusted DOOR is equal to the mean of their rank.

DOOR at Day 70 without tie-breaking is defined by ranking all subjects (pooling together both treatment group) according to their DOOR at Day 70 (lower is more desirable) ignoring their change in QoL score.

The QoL-Adjusted DOOR at Day 42 is calculated similarly using the change in QoL at Day 42.

6.6. Interim Analyses and Data Monitoring

A single interim analysis will be performed after approximately 50% of subjects have completed the trial. The interim analysis for futility will consist of a quantitative evaluation of potential effect sizes and associated precision using a predicted intervals and PIPS approach. Briefly, predicted intervals for both primary DOOR and secondary clinical failure outcomes will be modeled under a range of assumptions including: 1) the trends observed at interim analysis continue to end of study, 2) the null hypothesis is true (i.e., the DOOR distributions are identical between treatment groups), and 3) the worst-case scenario as an alternative outcome. By relying on prediction intervals, no statistical hypothesis testing is required, and no power is lost at interim analysis. Details of interim analysis, including best and worst case scenarios for alternative outcomes are provided in Section 8.5.

6.7. Multicenter Studies

This is a multicenter study, but randomization is not stratified by site. Data will be pooled across all clinical sites and analyses will not adjust for potential site effects.

6.8. Multiple Comparisons/Multiplicity

Only one hypothesis test will be performed for the primary analysis. Secondary and exploratory analyses will not be corrected for multiplicity.

7. STUDY SUBJECTS

7.1. Disposition of Subjects

Reasons for screening failures will be summarized in Table 8. The completion status and reasons for early termination or treatment discontinuation by Day 42 will be summarized (Table 3 and Listing 2) for each treatment group and pooled across treatment groups for the ITT population. A subject could be discontinued early due to an adverse event (AE) (serious or non-serious), loss to follow-up, non-compliance with study, voluntary withdrawal, withdrawal at the investigator request, termination of the site by the sponsor, termination of the study by the sponsor, death, lack of eligibility at enrollment, inadequate clinical response, or becoming ineligible after enrollment. Number and percentage of subjects completing each visit will be presented in Table 3 by treatment group for all randomized subjects.

Subject disposition and eligibility for analysis will be summarized in a CONSORT flow diagram (Figure 1).

7.2. Protocol Deviations

A summary of subject-specific protocol deviations will be presented by the reason for the deviation, the deviation category, and treatment group for all subjects (Table 2 and Listing 3). Non-subject specific protocol deviations will be in Listing 4. All subject-specific protocol deviations and non-subject specific protocol deviations will be presented. Major protocol deviations preventing the adjudication committee from evaluating the outcome will be determined by the adjudication committee.

PRIVILEGED AND CONFIDENTIAL COMMUNICATION

8. EFFICACY EVALUATION

All efficacy variables will be listed by subject. Data will be summarized by treatment group. Continuous efficacy variables will be summarized with the number of observations, mean, median, standard deviation, minimum, and maximum. Categorical efficacy variables will be summarized by number and percent in each category.

All statistical tests are two-sided and performed at the α=0.05 significance level; all confidence intervals are two-sided with 95% confidence level.

8.1. Primary Efficacy Analysis

The primary efficacy endpoint is DOOR assessed at Day 70 post study entry (TOC) performed on the ITT analysis population.

8.1.1. Analysis of DOOR at Day 70 Using ITT Analysis Population

DOOR at Day 70 is defined in Section 6.5.3 with and without tie-breaking. The primary analysis in this section is based on the QoL-Adjusted DOOR, which is calculated after using QoL as a tie-breaker.

The null and alternative hypotheses corresponding to the primary analysis of this study are:

H_0: $\Pr[DOOR_D > DOOR_C] + ½ \Pr[DOOR_D = DOOR_C] \leq 50\%$ (i.e., no difference in DOOR at Day 70).

H_1: $\Pr[DOOR_D > DOOR_C] + ½ \Pr[DOOR_D = DOOR_C] > 50\%$ (i.e., difference in DOOR at Day 70).

where $DOOR_D$ and $DOOR_C$ are the QoL-Adjusted DOOR for dalbavancin and control, or standard of care, groups, respectively, and $\Pr[DOOR_D > DOOR_C]$ is the probability of a DOOR from dalbavancin exceeding a DOOR from standard care and $\Pr[DOOR_D = DOOR_C]$ is the proportion of two DOOR being the same.

$\Pr[DOOR_D > DOOR_C] + ½ \Pr[DOOR_D = DOOR_C]$ will be referred to as the DOOR probability throughout this SAP.

The superiority of Dalbavancin vs Standard of Care is concluded if the lower bound of the 95% CI for the DOOR probability is larger than 50%.

Due to the presence of missing data, IPW and multiple imputation (MI) will be used to handle missing data. For the primary analysis of DOOR, IPW method will be used. MI described in Section 8.1.1.2 will be used for sensitivity analysis.

8.1.1.1. ITT Analysis of DOOR using IPW

ITT analysis requires that all randomized subjects be included in the analysis. However, missing data are prone to happen in clinical trials due to missing scheduled visits or loss to follow up for example. In this case, analysis that is only based on complete data may be biased if the excluded subjects are systematically different from those included. IPW is one approach commonly used to reduce this bias under a missing at random (MAR) assumption. This is achieved by weighting complete cases with the inverse of their probability of being a complete case. While subjects missing the DOOR are excluded from the analysis per se, they may still inform the fitting of the logistic regression model used to provide predictions of the probability of completeness which are used to calculate the weights.

Specifically, let Y_i represent the outcome of interest (QoL-Adjusted DOOR; see Section 6.5.3), X_i represent the covariates of interest, Z_i represent any other variables measured in the data but not used in the analysis model. For the IPW approach, we first define the missingness model to estimate the weights (w_i) using a logistic regression model with outcome L and covariates taken from set (X, Z) where L_i is defined as 1 if DOOR data are complete (not missing) and 0 otherwise. Through this model, we obtain the fitted probabilities of each subject being complete, denoted as π_i. Note that as described in Section 6.5.3, the QoL-Adjusted DOOR is calculated by accounting for tied DOORs using QoL as a tie-breaker for primary analysis.

The DOOR probability can then be calculated using the QoL-Adjusted DOOR at Day 70 as the outcome following the algorithm below:

1. Name QoL-Adjusted DOOR from the group that received standard of care antibiotics "sample 1" and the QoL-Adjusted DOOR from the group that received dalbavancin "sample 2". Rename weights from sample 1 as w_{1i} and weights from sample 2 as w_{2i}.
2. For each observation in sample 2. If an observation in sample 2 has a smaller QoL-Adjusted DOOR than an observation in sample 1, then that observation in sample 2 gets an indicator of value of 1. Else if the observation in sample 2 is equal to the observation in sample 1, the observation in sample 2 gets an indicator value of ½.
3. Let π_{1i} be the fitted probability of being complete for each observation in sample 1 and π_{2i} be the fitted probability for being complete for each observation in sample 2 obtained from the logistic regression model with an indicator for having non-missing QoL-Adjusted DOOR as the outcome.
4. For each pair in step 2, create the weight w_j as the inverse of the probability of both values in the pair being non-missing, i.e., $w_j = 1/(\pi_{1i} \times \pi_{2i})$ with j being the index for observations in sample 2
5. For each pair in step 2, create the weighted indicator value as $w\_ind_j = w_j \times indicator_j$
6. Repeat step 2 through 5 for all observations in sample 2.
7. The DOOR probability can then be obtained by $DOOR\_prob\_IPW$ = weighted average of all the indicator values in step 5, i.e., $DOOR\_prob\_IPW = \frac{\sum_j w\_ind_j}{\sum w_j}$,

The DOOR probability (i.e. Pr(Desirable DOOR in dalbavancin) + 0.5 Pr(Equal DOOR)) using IPW is given by the value in *DOOR_prob_IPW.*

To estimate the 95% confidence intervals for the DOOR probability, the approach discussed in Halperin et al. after incorporating IPW weights [2]. The superiority of Dalbavancin vs Standard of Care is concluded if the lower bound of the 95% CI for the DOOR probability is larger than 50%.

Pseudocode for Missingness model to estimate fitted probabilities and IPW weights:

Define complete as 1 for complete and 0 for missing and trt=1 for Standard of care and 2 for Dalbavancin.

```
proc logistic data = dat;
  model complete (event='1') = [trt baseline_pathogen age infection_site bacteremia_duration
];
  output out = out1 p = probs xbeta = logit;
run;
proc transpose data=out1 out=out2;by patid;id trt; var probs;run;
data out2;
      set out2;
      wts=1/(probs_dalba * probs_soc);
run;
```

8.1.1.2. ITT Analysis of DOOR using Multiple Imputation

As a sensitivity analysis, multiple imputation with a linear model to impute missing DOOR at Day 70 will be used. Details of multiple imputation methods are described in Section 8.6.1.

For each of the 20 complete multiple imputation datasets, a Mann-Whitney U statistic will be computed using randomization to dalbavancin versus randomization to standard of care therapy to define the binary grouping and DOOR at Day 70 as the outcome. The U statistics are asymptotically normally distributed, and so they can be combined into a single test statistic using Rubin's Rules [1].

Defining the following:

n_1: number of subjects in ITT population randomized to standard of care

n_2: number of subjects in ITT population randomized to dalbavancin

m: number of imputed datasets ($m = 20$)

Q_i: U statistic computed from the ith multiply imputed dataset

$\bar{Q} = \frac{1}{m}\sum_{i=1}^{m} Q_i$

Q_0: the expected value of a U statistic under the null hypothesis ($Q_0 = \frac{n_1 n_2}{2}$)

U_i: The variance from the ith multiply imputed dataset (this is not the U statistic). Correcting for ties, the formula for the variance of the Mann-Whitney U statistic, as described in Halperin et al. [2], is:

$$U_i = Var(Q_i) = \frac{1}{n_1 n_2}[n_1 + n_2 + 1 - (n_1 + n_2 - 2)\theta]\zeta(1-\zeta)$$

Where

$$\zeta = \frac{1}{n_1 n_2} Q_i$$

$$\theta = \frac{[(n_1 + n_2 - 2)\zeta - (n_2 - 1)A - (n_1 - 1)B]}{(m + n - 2)\zeta(1-\zeta)}$$

To obtain an estimator $\hat{\theta}$ of θ, use the following fomulas for A and B, respectively:

$$A = A_1 - \frac{1}{n_2 - 1}\sum_{i=1}^{D-1} p_{1i}\left[q_{2i}\sum_{j=i+1}^{D} p_{2j} - \left(\sum_{j=i+1}^{D} p_{2j}\right)^2\right] - \frac{1}{4(n_2 - 1)}\sum_{i=1}^{D} p_{1i}p_{2i}q_{2i}$$

Where

$$A_1 = \sum_{i=1}^{D-1} p_{1i}\left[\sum_{j=i+1}^{D} p_{2j} + \frac{p_{2i}}{2}\right]^2 + \frac{p_{1D}p_{2D}^2}{4}$$

And

$$B = B_1 - \frac{1}{n_1 - 1}\sum_{j=2}^{D} p_{2j}\left[q_{1j}\sum_{i=1}^{j-1} p_{1i} - \left(\sum_{i=1}^{j-1} p_{1i}\right)^2\right] - \frac{1}{4(n_1 - 1)}\sum_{j=1}^{D} p_{2i}p_{1i}q_{1i}$$

Where

$$B_1 = \sum_{j=2}^{D} p_{2j} \left[\sum_{i=1}^{j-1} p_{1i} + \frac{p_{1j}}{2}\right]^2 + \frac{p_{11}^2 p_{21}}{4}$$

In the equations for A and B above, D is the number of distinct values of DOOR in the dataset; p_{1i}, for i = 1, 2, …, D, represents the proportion of subjects randomized to standard of care with the ith value of DOOR; p_{2j}, for j = 1, 2, … D, represents the proportion of subjects randomized to dalbavancin with the jth value of DOOR; and q = 1 – p in general.

Additionally, the equation for an unbiased estimate of $\zeta(1-\zeta)$ is given by:

$$\frac{(n_1 n_2 - n_1 - n_2 + 2)\zeta - n_1 n_2 \zeta^2}{(n_1 - 1)(n_2 - 1)} + \frac{A}{n_1 - 1} + \frac{B}{n_2 - 1}$$

After substituting the values of A, B and $\zeta(1-\zeta)$ in the equation θ to obtain an estimate $\hat{\theta}$ of θ, define θ as follows: If $\hat{\theta} < 0$ then $\theta = 0$, if $\hat{\theta} > 1$ then $\theta = 1$, otherwise $\hat{\theta} = \theta$.

$\bar{U} = \frac{1}{m}\sum_{i=1}^{m} U_i$ (The within imputation variance. This is not the mean of the U statistics.)

$B = \frac{1}{m-1}\sum_{i=1}^{m}(Q_i - \bar{Q})^2$

$T = \bar{U} + \frac{m+1}{m} B$

$W = \frac{(\bar{Q} - Q_0)^2}{T}$

$r = \frac{m+1}{m}\frac{B}{\bar{U}}$

$$\nu = (m-1)\left(1 + \frac{1}{r}\right)^2$$

As a sensitivity analysis, a 95% CI for U will be computed using the overall test statistic W through the inversion of the F-test. Dividing the bounds of this CI by $n_1 n_2$ will yield the bounds for the 95% CI of the DOOR probability. Thus, the CI for DOOR probability is given by:

$$95\%\ \text{CI:}\ \left(\frac{\bar{Q} - \sqrt{T \times F_{0.95,1,\nu}}}{n_1 n_2}, \frac{\bar{Q} + \sqrt{T \times F_{0.95,1,\nu}}}{n_1 n_2}\right)$$

A point estimate of the DOOR probability will be obtained by dividing $\bar{Q}$ by $n_1 n_2$. Results will be shown in Table 20 for ITT population with and without QoL as a tie-breaker. This analysis will be repeated in the mITT population and results will be reported in Table 21 with and without QoL as a tie-breaker. A listing of DOOR and its components is provided in Listing 9.

8.2. Secondary Efficacy Analyses

8.2.1. Analysis of Clinical Efficacy at Day 70 using IPW for the ITT and mITT Analysis Populations

Let Y_i represent the outcome of interest (clinical efficacy), X represent the covariates of interest, Z represent any other variables measured in the data but not used in the analysis model. For the IPW approach, we first define the missingness model to estimate the weights (w_i) using a logistic regression model with outcome R and covariates taken from set (X, Z) where R_i is defined as 1 if is complete (not missing clinical success) and 0 otherwise. Through this model, we obtain the fitted probabilities of being complete, denoted as π_i. Weights w_i are then obtained by the inverse of the fitted probabilities of being complete ($w_i = 1/\pi_i$).

To estimate the proportions of clinical efficacy, we will fit a linear regression model with treatment as a covariate adjusting for IPW weights ($Y_i = \beta_0 + \beta_1 trt + \epsilon_i$, where $\epsilon_i \sim N(0, w_i\sigma^2)$

The difference in proportions of clinical efficacy for dalbavancin compared to standard of care ($p_d - p_s$) will then be obtained by β_1. The rate of clinical efficacy for dalbavancin will be estimated as $p_d = \beta_0 + \beta_1$ and the rate of clinical efficacy for standard of care will be obtained by $p_s = \beta_0$. The two-sided 95% CI for the proportions of clinical efficacy and difference in proportions in clinical efficacy will use estimates from the linear regression with IPW.

Proportions of clinical efficacy for each treatment group along with their 95% CI, a point estimate of the difference in proportions of clinical efficacy at Day 70 along with 95% CI obtained from linear regression with multiple imputation model as described above will be provided in Table 36 for Day 70 using the ITT analysis population. These analyses will be repeated for the mITT analysis population and results will also be provided in Table 36.

The null hypothesis will be rejected and non-inferiority of dalbavancin versus standard of care with respect to clinical efficacy at Day 70 using a non-inferiority margin of 20% will be concluded if the lower bound of the 95% CI for the difference in proportions of clinical efficacy for dalbavancin relative to standard of care is greater than -20%.

Subgroup analyses of clinical efficacy at Day 70 will be performed for the ITT and mITT populations using IPW for different clinically important subgroups including a) those with MSSA versus MRSA; b) persons who inject drugs (PWID) vs non-PWID; c) those who received infectious disease consultation vs those who did not; d) underlying site of infection (endovascular, bone and joint, skin, pulmonary); e) subjects with immune-suppression; f) divided by duration of initial bacteremia, in the ITT, mITT, and CE populations and results will be reported in Table 39 for ITT and Table 40 for mITT analysis population. Proportions and difference in proportions of clinical efficacy along with their 95% CI estimated from linear regression model following multiple imputation will be reported.

Pseudocode:

Missingness model to estimate fitted probabilities and IPW weights:

Define complete as 1 for complete and 0 for missing and trt=1 for standard of care and 2 for Dalbavancin.

```
proc logistic data = dat;
  model complete (event='1') = [age sex trt and othercovariates that affect
completeness of the data];
  output out = out1 p = probs xbeta = logit;
run;
data out1;
      set out1;
      wts=1/probs;
run;
```

Final model:

```
proc glm data=out1;
      class trt(ref='1');
      model clinsuccess= trt solution;
      weight wts;
      lsmeans TRTPN/ pdiff=all tdiff cl stderr;
run;
```

8.2.2. Sensitivity Analysis of Clinical Efficacy at Day 70 using Multiple Imputation for the ITT and mITT Analysis Populations

Clinical efficacy at Day 70 is defined as absence of clinical failure, infectious complications, and all-cause mortality. A subject will be defined as not having clinical efficacy of at least one of these three components occurred. This endpoint will be analyzed using a non-inferiority approach. The corresponding hypotheses are:

Null hypothesis: $\pi_{\text{dalbavancin}} - \pi_{\text{standard of care}} \leq -20\%$,

Alternative hypothesis(non-inferiority): $\pi_{\text{dalbavancin}} - \pi_{\text{standard of care}} > -20\%$,

where π represents the probability of clinical efficacy at study Day 70. 20% is the non-inferiority margin used for this study and is also used for all secondary non-inferiority analyses of clinical efficacy.

The non-inferiority of dalbavancin versus standard of care with respect to clinical efficacy using a non-inferiority margin of 20%, will be determined for the ITT analysis population using a two-sided 95% CI of the difference in proportions of clinical efficacy as constructed using multiple imputation of clinical efficacy with linear regression. A lower bound of the CI greater than -20% will result in the conclusion of non-inferiority of dalbavancin. The imputation model will utilize available information collected at baseline and any completed study visits.

The secondary analysis will use multiple imputation with a linear regression model without rounding to impute missing values of clinical efficacy at Day 70 using the ITT population [3 and 4].

Although the linear regression without rounding can sometimes yield implausible imputed values of treatment success, Horton et.al [4] showed that this method yields an unbiased estimate of the binomial proportion.

- Let $Y_1, Y_2, \dots, Y_N$ be independent and identically distributed (iid) Bernouilli random variables
- Let $p = E(Y_i)$ be the probability of success

- Assume that only n out of the N Bernoulli data points are observed; the rest are missing. For simplicity, assume Let $Y_1, Y_2, \ldots, Y_n$ are observed and Let $Y_{n+1}, \ldots, Y_N$ are missing. Further assume that data are Missing Completely at Random (MCAR).

For estimating p, the minimum variance unbiased estimate (MVUE) of p denoted by $\hat{p}$ which is simply the mean of observed data, i.e.,

$$\hat{p} = \frac{1}{n}\sum_{i=1}^{n} Y_i$$

Rubin and Schenker [5] proposed using a full normal imputation method to impute missing values $Y_{n+1}, \ldots, Y_N$ which assumes that the Y_i are iid from normal distribution with mean p and variance σ^2. This method follows the following algorithm to generate the missing values.

This full normal imputation method without rounding incorrectly assumes a normal distribution and can sometimes yield implausible imputed values (above 1 or below 0). However, it produces an unbiased estimate of the probability of success p.

Allison [3] showed that this approach can be extended to allow covariates in the model. Hence, using simulation studies, Allison showed that multiple imputation using linear regression performed well in estimating regression coefficients in different missing data scenarios (MCAR, MAR) even when compared to logistic regression. The added benefit of using the linear regression model is that it directly provides proportion differences along with their 95% CI after applying PROC MIANALYZE to the model fits from the M multiply imputed datasets. This approach will follow the three steps described below:

Step 1: A multiple regression model: $Y_i = \beta_0 + \beta_1 X_1 + \beta_2 X_2 + \beta_3 X_3 + \cdots + \beta_c X_c + \epsilon_i \quad, \epsilon_i \sim N(0, \sigma^2)$ where Y_i represents the indicator for clinical efficacy and X_c are the covariates described in Section 8.6.2 to be used in the multiple imputation model to generate *M* multiply imputed datasets.

Step 2: A linear regression model $Y_i = \beta_0 + \beta_1 trt + \epsilon_i$ will be fit on each of the *M* multiply imputed datasets with *trt* defined as 0 for standard of care and 1 for dalbavancin.

Step 3: The final clinical efficacy estimates will be obtained by combining *M* estimates of clinical efficacy estimates using PROC MIANALYZE as described in in Section 8.6.2. The rate of clinical efficacy for dalbavancin will be estimated as $p_d = \beta_0 + \beta_1$ and the rate of clinical efficacy for standard of care will be obtained by $p_s = \beta_0$. The difference in proportions of clinical efficacy for dalbavancin compared to standard of care $(p_d - p_s)$ will then be obtained by β_1. The two-sided 95% CI for the proportions of clinical efficacy and difference in proportions in clinical efficacy will use estimates from the linear regression with multiple imputation.

Proportions of clinical efficacy for each treatment group along with their 95% CI, a point estimate of the difference in proportions of clinical efficacy at Day 70 along with 95% CI obtained from linear regression with multiple imputation model as described above will be provided in Table 35 for Day 70 using the ITT analysis population. These analyses will be repeated for the mITT analysis population and results will also be provided in Table 35.

The null hypothesis will be rejected and non-inferiority of dalbavancin versus standard of care with respect to clinical efficacy at Day 70 using a non-inferiority margin of 20% will be concluded if the lower bound of the 95% CI for the difference in proportions of clinical efficacy for dalbavancin relative to standard of care is greater than -20%.

An individual listing of observed values of clinical efficacy components is provided in Listing 9.

PRIVILEGED AND CONFIDENTIAL COMMUNICATION

8.2.3. Analysis of DOOR Components Using ITT Analysis Population

Results for the analysis of DOOR components at Day 70 will be presented. Proportions of subjects with clinical failure, infectious complications, SAEs & AEs leading to study drug discontinuation, and all-cause mortality at Day 70 will be reported in Table 42 by treatment group. Note that this analysis will consider subjects in the ITT population who have non-missing values for the corresponding DOOR component. The DOOR component will be summarized by the DOOR probability and 95% confidence interval (computed as the probability that a randomly selected patient will have a better DOOR if assigned to the intervention arm using the Wilcoxon-Mann-Whitney statistic corrected for ties). A summary of DOOR by component will be presented at Day 70 for the ITT population in Table 47. The DOOR probability will also be presented graphically using forest plots in Figure 2. These analyses will be repeated for Day 42, and analogous results will be reported in Table 45, Table 48, and Figure 5, respectively.

8.2.4. Analysis of Clinical Failure at Day 70 Using ITT Population

Clinical failure at Day 70 will be analyzed using weighted generalized estimating equations (GEE) assuming an unstructured correlation structure, including clinical failure at Day 42. The difference in proportions of clinical failure between the two groups at Day 70 will be calculated with the corresponding 95% confidence interval (Table 49). The GEE model will use subject random effects with an unstructured correlation structure to generate the average difference in proportions of clinical failure for dalbavancin compared to standard of care at Day 70 and IPW weights will be obtained using a similar approach as that described in Section 8.2.1 using the following pseudocode:

```
Weighted GEE:
proc gee data=temp descending;
class id trt clinfailure time/param=ref
missmodel trt time trt*time / type=obslevel; /* missingness model */
model clinfailure= trt time trt*time; /* marginal model */
repeated subject=id/ corr=un;
    estimate 'trt 1 at time=2' intercept 1 trt 1 time 2 trt*time 2;
    estimate 'trt 0 at time=2' intercept 1 time 2;
    estimate 'trt 1 vs 0 at time=2' trt 1 trt*time 2;
run;
```

where temp is a dataset with one row per timepoint per subject (one row for time=1 and another row for time=2 per subject). Clinfailure is a binary variable with a value of 1 indicating clinical failure and a value of 0 indicating clinical success, trt is a binary variable with a value of 1 indicating dalbavancin and a value of 0 indicating standard of care, id indicates each subject identifier, and time is binary variable with a value of 1 indicating Day 42 and a value of 2 indicating a Day 70 timepoint.

A linear model was used in this scenario since the outcome of interest is difference in proportions instead of odds ratios. The difference in proportions will be given by the sum of coefficient for the treatment and the coefficient for treatment*time interaction and will be interpreted as 'on average, the proportion clinical failure at Day 70 is increased (or decreased if the sign if negative) by xx amount when taking dalbavancin compared to standard of care'.

As a sensitivity analysis, generalized linear mixed model (GLMM) approach will be used to analyze clinical failure (Table 49) to model individual treatment differences for dalbavancin compared to standard of care using the following pseudocode:

```
proc mixed data=temp;
     class id;
     weight wts;
     model clinfailure=trt time trt*time;
     repeated/ subject=id type=un;
    estimate 'trt 1 at time=2' intercept 1 trt 1 time 1 trt*time 1;
    estimate 'trt 0 at time=2' intercept 1 time 1;
    estimate 'trt 1 vs 0 at time=2' trt 1 trt*time 1;

run;
```

Note that wts will be calculated from a similar missingness logistic regression model as that used for the weighted GEE model above.

Similarly, to GEE, the difference in proportions using GLMM will be given by the sum of coefficient for the treatment and the coefficient for treatment*time interaction. This treatment difference will be interpreted as 'for a specific individual, the proportion clinical failure at Day 70 is increased (or decreased if the sign if negative) by xx amount when taking dalbavancin compared to standard of care'.

8.3. Exploratory Efficacy Analyses

8.3.1. Analysis of Clinical Efficacy at Day 42

Clinical efficacy at Day 42 will be analyzed using a similar approach as that described in Section 8.2.1 using IPW and as described in Section 8.2.2 using multiple imputation for the ITT and mITT analysis populations, and results will be reported in Table 36.

Subgroup analyses of clinical efficacy at Day 42 will be performed for the ITT and mITT populations using IPW for different clinically important subgroups including a) those with MSSA versus MRSA; b) persons who inject drugs (PWID) vs non-PWID; c) those who received infectious disease consultation vs those who did not; d) underlying site of infection (endovascular, bone and joint, skin, pulmonary); e) divided by duration of initial bacteremia and results will be reported in Table 38 for ITT and Table 39 for mITT analysis population. Proportions and difference in proportions of clinical efficacy along with their 95% CI estimated from linear regression model following multiple imputation will be reported.

8.3.2. Additional Analyses of DOOR

8.3.2.1. Analysis of DOOR at Day 42 Using ITT and mITT Analysis Populations

The analysis of DOOR at Day 42 for the ITT and mITT analysis populations will be performed in an analogous manner as those described in Section 8.1.1.1 for the primary analysis using IPW and as described in Section 8.1.1.2 using multiple imputation and results will be reported in Table 22 for the ITT population and Table 23 for the mITT population.

8.3.2.2. Subgroup Analyses of DOOR

Subgroup analysis of DOOR will be performed using subjects in each of the following subgroups a) those with MSSA versus MRSA; b) persons who inject drugs (PWID) vs non-PWID; c) those who received infectious disease consultation vs those who did not; d) underlying site of infection (endovascular, bone and joint, skin, pulmonary); e) subjects with immune-suppression; f) divided by duration of initial bacteremia, in the ITT, mITT, and CE populations for Day 42 and Day 70. Analyses for the ITT and mITT will use a similar approach as that described in Section 8.1.1.1 using IPW and results will be reported in Table 25 for ITT

population and Table 26 for mITT population. Subgroup analysis of DOOR for the CE population will use the approach described in Section 8.3.3.2 for complete data and results will be reported in Table 27. Forest plots for DOOR probabilities are also presented for the ITT analysis population in Figure 20 for Day 70 and Figure 21 for Day 42.

8.3.3. Analysis of DOOR Components at Day 42 and Day 70 Using mITT Analysis Populations

Results for the analysis of DOOR components at Day 70 will be presented. Proportions of subjects with clinical failure, infectious complications, SAEs, AEs leading to study drug discontinuation, and all-cause mortality at Day 70 will be reported in Table 42 for each treatment group using mITT population. The DOOR component will be summarized by the DOOR probability and 95% confidence interval. The DOOR probability will also be presented graphically using forest plots in Figure 3 for the mITT population. This analysis will be repeated for Day 42, and results with be reported in Table 45 and Figure 6.

8.3.3.1. Analysis of DOOR Categories at Day 42 and Day 70 Using ITT and mITT Analysis Populations

The number and percent of subjects along with 95% CI for the percentages computed using the Wilson method in each DOOR category will be presented in Table 28, Table 29, and Table 30 for the ITT, mITT, and CE analysis populations, respectively. The percentage of subjects in each DOOR category will also be presented graphically in Figure 8, Figure 9, Figure 10 for Day 70 and Figure 11, Figure 12, Figure 13 for Day 42. The percentage of subjects in each DOOR category is also presented per subgroup for Day 70 using the ITT analysis populations starting with Figure 14 through Figure 19.

8.3.3.2. Distribution of DOOR by Treatment Group Using ITT and mITT Analysis Populations

The distribution of DOOR by treatment group will be presented by number and percentage of subjects at Day 42 and Day 70 using the ITT and mITT in Table 55. Subjects with missing DOOR will be excluded from analysis. The P-value computed using the Wilcoxon rank-sum test will also be provided. These summaries are also provided by Subgroup Categories for the ITT analysis population in Table 56 for Day 42 and Table 57 for Day 70.

8.3.3.3. Cumulative Difference in DOOR Categories Using ITT and mITT Analysis Populations

Cumulative DOOR probability as well as 95% CI will be provided in Table 31 for Day 42 and Day 70 Using the ITT analysis population. The Wilcoxon-Mann-Whitney statistic corrected for ties will be used as an estimate of the cumulative DOOR probability. Analogous results will be presented for the mITT population in Table 32. These results will also be presented graphically in Figure 22.

8.3.3.4. Analysis of Difference in Mean Partial Credit Using ITT and mITT Analysis Populations

The partial credit will be calculated assigning a partial credit score to each of the DOOR categories. QoL is not considered for analysis of partial credit score. Four scenarios will be considered. Scenario A will assign 100, 0,0, 0, 0 partial credit score to the 5 DOOR categories with DOOR category 1 taking a partial credit of 100 while the next 4 DOOR categories take a partial credit of 0. Similarly, scenario B will assign 100, 100, 0, 0, 0 while scenario C assigns 100, 100, 100, 0, 0 partial credit scores to the 5 DOOR categories, and scenario D assigns 100, 100, 100, 100, 0 partial credit scores to the 5 DOOR categories. For each scenario, the mean and standard deviation of these partial credit scores will be provided for each treatment group and corresponding scenario. The difference in mean partial credit score will also be summarized in Figure 23.

8.3.3.5. Analysis of Expected DOOR Category Distribution and Expected Numbers Gained Loss Using ITT and mITT Analysis Populations

The expected number for the DOOR category will be calculated by multiplying the proportion of subjects in that DOOR category by 1000 separately for each treatment group. The gained loss for each DOOR category will be calculated by taking the difference in expected number for Dalbavancin minus the expected number for standard of care. The total gained loss will be obtained by summing up all the gained losses for all the DOOR categories (i.e., add up all the differences in expected numbers for dalbavancin minus standard of care) and results will be reported in Table 34 for Day 42 and 70 for ITT and mITT analysis populations.

8.3.4. Analysis of Microbiological Success at Day 42 and Day 70 using the ITT and mITT Analysis Populations

Microbiological success at Day 42 is defined as the absence of a post-randomization growth (i.e., no positive cultures) of the baseline pathogen from blood cultures drawn post randomization or from another sterile body site until Day 42. Subjects who will still be in the study at Day 42 and have no post randomization cultures will be considered as having microbiological success. Subjects who are lost to follow up by Day 42 and have no post-randomization blood cultures will have missing microbiologic success. Due to the potential of having missing values of microbiologic success in the ITT and mITT, microbiologic success at Day 42 will be analyzed using a similar approach as that described in Section 8.2.1 using IPW and in Section 8.2.2 using multiple imputation for the ITT and mITT analysis populations and results will be reported in Table 50. These analyses will also be repeated for Day 70 and results will be reported in Table 50 for ITT and mITT analysis populations. Subgroup analyses of microbiologic success will be performed for the ITT and mITT populations using IPW for different clinically important subgroups including a) those with MSSA versus MRSA; b) persons who inject drugs (PWID) vs non-PWID; c) those who received infectious disease consultation vs those who did not; d) underlying site of infection (endovascular, bone and joint, skin, pulmonary); e) subjects with immune-suppression; f) divided by duration of initial bacteremia and results will be reported in Table 52 for ITT and Table 53 for mITT analysis population. Proportion and difference in proportions of microbiologic success along with their 95% CI estimated from linear regression model with IPW will be reported.

An individual listing of observed values of microbiologic success is provided in Listing 9 and a listing of culture results used to define microbiologic success is provided in Listing 10.

8.3.5. Analysis of QoL Score using ITT and mITT Analysis Populations

Summary statistics (number of subjects, mean, standard deviation, minimum, maximum) for QoL scores and change from baseline of QoL scores obtained from the ARLG Bloodstream Infection QoL Measure at Day 42 and Day 70 will be provided for each analysis population in Table 58. Similarly, summary statistics for QoL scores from the EQ-5D-5L instrument and PROMIS Global Health Short Form will be provided in Table 59 and Table 60, respectively. A listing of QoL data from the three instruments is provided in Listing 11.

Additionally, descriptive statistics of QoL measures will also be presented by item.

8.3.6. Bivariate Analysis of DOOR Probability vs Difference in Mean of Change in QoL Score from Baseline

In addition to the DOOR analyses described in Section 8.1.1 and Section 8.4.1, a two-dimensional analysis of the DOOR probability and difference in the mean change in QoL from baseline in the two treatment groups using the ARLG Bloodstream Infection QoL Measure will also be performed at Day 70. Only subjects with non-missing DOOR and change in QoL from baseline will be included in these analyses. The horizontal axis

is the DOOR probability (probability of a more desirable DOOR category when assigned to Dalbavancin vs. Standard of care) based on the DOOR categories without using mean change in QoL from baseline as a tie-breaker. The vertical axis is the difference in the means of the observed difference in mean change in QoL from baseline (dalbavancin minus standard of care). A result in the upper right quadrant represents more desirable results for dalbavancin group, while a result in the lower left represents more desirable standard of care. The other two quadrants represent tradeoffs for clinical outcomes and QoL score.

1000 bootstrap samples will be generated by resampling with replacement from the empirical distribution of DOOR and change in QoL from baseline at Day 70. The DOOR probability and mean difference in the mean change in QoL from baseline will be estimated using the bootstrap samples and plotted as a scatter plot. A 95% joint region of DOOR probability and difference in mean change in QoL from baseline will be constructed first using a parametric method. The parametric method will estimate the confidence region using the ellipse method from the CAR R package which uses a bivariate normal distribution. The 90%, 95%, and 99% confidence regions will be reported. These results will be reported in Figure 24 for Day 70 and Figure 25 for Day 42 using the ITT analysis population. Subjects in the ITT analysis population with missing values of DOOR categories and change in QoL from baseline will be excluded from this analysis.

8.3.7. Analysis of Late Recurrence Within ITT Population with Osteomyelitis

The osteomyelitis population includes all randomized subjects diagnosed with osteomyelitis. Subjects in this population will have an extra follow-up visit approximately 6 months after randomization to evaluate long term recurrence risk. Late recurrence within the osteomyelitis population will be defined by the presence of the following up to 6 months after randomization: progressive imaging changes along with isolation of *S. aureus* from blood, bone biopsy, associated fluid aspiration, or operative tissue.

Protocol amendment version 3.0 expanded Visit 7 (Day 180) to include all subjects with osteomyelitis, instead of only those with vertebral osteomyelitis at baseline (version 2.0). When possible, any subjects who were not originally eligible for Visit 7 under protocol version 2.0, but who were eligible under protocol version 3.0 were re-consented and data was collected. Thus, in addition to the planned analysis, which includes all subjects for whom Visit 7 consent and data were obtained, an additional sensitivity analysis will be conducted that excludes subjects enrolled under protocol version 2.0 who were not eligible prior to protocol version 3.0. That is, the planned analyses will be performed in the following groups:

1. All subjects for whom Visit 7 consent and data was obtained (either at the time of enrollment or retroactively following protocol version 3.0), and
2. Subjects who enrolled after protocol version 3.0 or later (i.e., excluding subjects those enrolled initially under protocol version 2.0 that re-consented under protocol version 3.0).

Note that no eligible subjects were enrolled under protocol version 1.0. If no data are available for subjects who were re-consented the sensitivity analysis will be excluded.

Number and percentage of subjects in each of the osteomyelitis populations above will be presented in Table 63 by treatment group. Difference in proportions along with their 95% CI will also be reported.

8.4. Supplemental Efficacy Analyses

All efficacy analyses performed using the CE analysis population will be considered supplemental.

PRIVILEGED AND CONFIDENTIAL COMMUNICATION

8.4.1. Analysis of DOOR at Day 42 and 70 Using CE Analysis Populations

Additional analyses of DOOR at Day 42 and Day 70 will be performed using only subjects in the CE population with complete data. These analyses will test the null hypotheses described above using the Mann-Whitney U Test, estimate Pr(Desirable DOOR in dalbavancin) + 0.5 Pr(Equal DOOR) using U divided by the number of pairwise comparisons (defined as ζ below), and will compute confidence intervals using the method described in Halperin et al. [2]. The methods described in Section 8.1.1.2 will be implemented for this analysis.

Results from the analysis using CE analysis population with complete data will be reported in Table 24 for both Day 42 and Day 70.

8.4.2. Analysis of Clinical Efficacy at Day 70 Using CE Analysis Population

The CE analysis populations at Day 70 will have no missing values of clinical efficacy, therefore the analysis of clinical efficacy at Day 70 will be performed using a linear regression without multiple imputation with treatment group as a covariate ($Y_i = \beta_0 + \beta_1 trt_i + \epsilon_i$) for subjects in the CE analysis population at Day 70. The rate of clinical efficacy in the dalbavancin arm will then be estimated by $p_d = \beta_0 + \beta_1$ and the rate of clinical efficacy in the standard of care arm will be provided by $p_s = \beta_0$.

The estimate for the difference in proportions of clinical efficacy will be provided by β_1 and its 95% CI will be calculated first by using the 95% CI for β_1 from the linear regression model above. As a sensitivity analysis, the 95% CI for the difference in proportions of clinical efficacy will be recalculated using the Miettinen–Nurminen method from PROC FREQ with RISKDIFF (CL= MN) in SAS.

Proportions of clinical efficacy for each treatment group, a point estimate of the difference in proportions of clinical improvement at Day 70 along with their 95% CIs obtained methods described above will be provided in Table 37 using the CE analysis population at Day 70. If the lower bound of the 95% CI for the difference in proportions of clinical efficacy is greater than -20%, it will be annotated by a footnote b.

Results from the subgroup analysis of clinical efficacy at Day 70 using CE population will be reported in Table 40.

Proportions and difference in proportions of clinical efficacy along with their 95% CI estimated from linear regression model without imputation will be reported for the CE analysis population.

Clinical efficacy will also be analyzed for the CE analysis population at Day 42 using a similar approach as that used for Day 70, and results will be reported in Table 37. Results from the subgroup analysis of clinical efficacy at Day 42 using CE population will also be reported in Table 40.

8.4.3. Analysis of DOOR at Day 42 and Day 70 Using CE Analysis Population

Analyses described in Section 8.3.3 will be performed using the CE analysis population. Proportions of subjects with clinical failure, infectious complications, SAEs, AEs leading to study drug discontinuation, and all-cause mortality at Day 70 will be reported in Table 43 using the CE population. The DOOR component will be summarized by the DOOR probability and 95% confidence interval. The DOOR probability will also be presented graphically using forest plots in Figure 4 for the CE population. This analysis will be repeated for Day 42 and results with be reported in Table 46 and Figure 7.

PRIVILEGED AND CONFIDENTIAL COMMUNICATION

8.4.3.1. Distribution of DOOR by Treatment Group Using CE Analysis Population

The distribution of DOOR by treatment group will be presented by number and percentage of subjects at Day 42 and Day 70 using the CE analysis population in Table 55. The P-value computed using the Wilcoxon rank-sum test will also be provided.

8.4.3.2. Cumulative Difference in DOOR Using CE Analysis Population

Cumulative DOOR probability as well as 95% CI will be provided in Table 33 for Day 42 and Day 70 Using the CE analysis population. The Wilcoxon-Mann-Whitney statistic corrected for ties will be used as an estimate of the cumulative DOOR probability. These results will also be presented graphically in Figure 22.

8.4.3.3. Analysis of Difference in Mean Partial Credit Using CE Analysis Population

Analysis of mean partial credit scores described in Section 8.3.3.4 will be repeated using the CE analysis population and results will provided in Figure 22.

8.4.3.4. Analysis of Expected DOOR Distribution and Expected Numbers Gained Loss

Analysis of expected DOOR distribution and expected gained loss described in Section 8.3.3.4 will be repeated for the CE analysis population and results will be reported in Table 34.

8.4.4. Analysis of Microbiological Success at Day 42 and Day 70 using the CE Analysis Populations

Analysis of microbiological success described in Section 8.3.4 will be repeated using the CE analysis population at Day 42 and Day 70. Results will be presented in Table 51. Subgroup analysis of microbiological success at Day 42 and Day 70 will also be performed using the CE population and results will be presented in Table 54.

8.5. Interim Analyses

Predictive intervals and predictive interval plots will be generated for DOOR and clinical efficacy under a range of assumptions including: 1) the trends observed at interim analysis continue to end of study, 2) the null hypothesis is true (i.e., the DOOR distributions are identical between treatment groups), and 3) and the worst-case scenario as an alternative outcome. These predicted interval plots provide a prediction of the trial results were the trial to continue as planned under varying assumptions regarding future data (e.g., current trend continues, null hypothesis is true, and worst-case scenario). For example, using then current trend, the remaining subjects will be simulated assuming outcomes continue to occur at the rates observed at the time of interim analysis. For each assumption, 10,000 complete datasets (N=200 subjects) will be simulated and used to calculate the probability of rejecting the null hypothesis for each scenario. By relying on prediction intervals, no statistical hypothesis testing is required, and no power is lost at interim analysis [7 and 8]. Note interim results will only be presented in the DSMB closed session.

Suppose the following distribution of DOOR is observed at the interim analysis, i.e. $\hat{p}_{D1}$ is the proportion of subjects in the Dalbavancin group observed to have a DOOR of 1 (alive with no events).

DOOR	Dalbavancin	Standard of Care
1 – Alive with no events	$\hat{p}_{D1}$	$\hat{p}_{C1}$
2 – Alive with 1 event	$\hat{p}_{D2}$	$\hat{p}_{C2}$
3 – Alive with 2 events	$\hat{p}_{D3}$	$\hat{p}_{C3}$
4 – Alive with 3 events	$\hat{p}_{D4}$	$\hat{p}_{C4}$
5 – Death	$\hat{p}_{D5}$	$\hat{p}_{C5}$

Let p_{Dk} and p_{Ck} denote the proportions of subjects in the Dalbavancin and Standard of Care groups with DOOR = *k,* for *k* = 1, 2, …, 5, used to simulate the predicted data.

- Under scenario 1, the trend observed at the interim continues, we assume that $p_{Dk} = \hat{p}_{Dk}$ and $p_{Ck} = \hat{p}_{Ck}$ for *k* = 1, 2, …, 5.
- Under scenario 2, the null hypothesis is true, we assume $p_{Dk} = p_{Ck} = \bar{p}_k = \frac{n_d}{n_d + n_c}\hat{p}_{Dk} + \frac{n_c}{n_d + n_c}\hat{p}_{Ck}$; where n_d is the number of subjects in the Dalbavancin group and n_c is the number of subjects in the standard of care arm.
- Under scenario 3, the worst-case scenario as an alternative outcome, we assume that $p_{Dk} = \hat{p}_{Ck}$ and $p_{Ck} = \hat{p}_{Dk}$ if the Standard of Care group is better at the interim analysis (as determined by DOOR probability).

Summary of PIPs statistics are presented in Table 61 and Figure 26 for DOOR at Day 70 and in Table 62 and Figure 27 for clinical efficacy at Day 70 using the ITT analysis population. Other tables and figures from the main analyses will also be presented in the interim report and are indicated via implementation notes in this SAP.

8.6. Imputation of Missing Data

8.6.1. Multiple Imputation of Missing DOOR Day 42 and Day 70

Several analyses depend on multiple imputation of DOOR at Day 70 and Day 42 for ITT and mITT analysis populations. First, a table showing the number and percentage of missing data for DOOR on Day 42 and Day 70 will be presented in Table 18 for ITT and Table 19 for mITT analysis population. In order to use the multiple imputation model to adjust for bias caused by missing data, we assume that data are missing at random (MAR).

Multiple imputations of each of these missing endpoints will be performed independently, and each subject will have their missing endpoints imputed independently of other subject's imputations using a subject-specific imputation model.

Before performing multiple imputation, an ordered list of variables to include in the subject-specific imputation model is constructed. Ordering is specified so that exact imputation results from final data are prespecified may be replicated in SAS (using seeds described below). The complete ordered list of variables for the imputation models for DOOR at Day 70 is below.

- Indicator of dalbavancin as study treatment (binary indicator, standard of care is the reference group)
- Baseline pathogen (binary indicator, MRSA is the reference group)
- Age at enrollment
- Site of infection
- Duration of bacteremia
- Observed DOOR category at Day 70

For DOOR at Day 42, the complete list of model variables is identical to the above with Day 70 being replaced by Day 42.

The actual list of model variables for each subject-specific imputation model will follow the ordering above but omit variables with missing values. The below pseudo-code / SAS code outlines the creation of 20 multiple imputation datasets. Note that the seeds used in the actual analysis must follow the specification given in the pseudo-code and subjects must be processed in the order described in the pseudo-code. The pseudo-code is in terms of Day 70 endpoints, but the general logic is also applicable to the Day 42 endpoints (with references to "D70" replaced with references to "D42").

```
DEFINE i=index variable for subjects having DOOR imputed.
      Subjects requiring imputation are sorted in ascending order
      by PATID.
DEFINE N=number of subjects requiring imputation
DEFINE g&i=analysis dataset containing predictors and DOOR for Subjects with complete
       DOOR at D70 as well as subject i (only one subject with missing data are
       included).  Note that subjects with complete DOOR that are missing a value for
       one or more variables in the subject-specific imputation model are excluded.
DEFINE imp_g&i = g&i, with 20 imputed values for the missing DOOR
      added by PROC MI
DEFINE &&modelVars_&i  = list of observed variables in subject i, to
      be used for imputation of DOOR.
%do i=1 %to &N;
PROC MI data=g&i out=imp_g&i seed=500&i NIMPUTE=20 noprint;
      var &&modelVars_&i DOOR;
      monotone reg(DOOR_D70 = &&modelVars_&i);
run;
%end;
imp_g&i will be subset to contain only rows for the subjects with imputed DOOR and
merged together and with subjects with complete data to create the twenty complete
multiply imputed datasets
```

8.6.2. Multiple Imputation (MI) of Missing Values of Clinical Efficacy and Microbiologic Success on Day 42 and Day 70

Secondary and exploratory analyses of clinical efficacy and microbiologic success for ITT and mITT populations depend on multiple imputation. First, a table showing the number and percentage of missing data for clinical efficacy and microbiologic success on Day 42 and Day 70 will be presented in Table 18 for ITT and Table 19 for mITT analysis population. In order to use the MI model to adjust for bias caused by missing data, we assume that data are missing at random (MAR).

For missing clinical efficacy on Day 42 and Day 70, multiple imputations of missing clinical efficacy on Day 42 and Day 70 will be performed independently, and each subject will have their missing clinical efficacy imputed independently of other subjects' imputations using a subject-specific imputation model. The pseudocode shown below details how missing data for clinical efficacy for Day 70 (Day 42) will be imputed using *m* multiply imputed datasets from linear models. The following covariates will be used non-missing for the MI model: treatment group, baseline pathogen, age, gender. The number of imputed, *m*, datasets will be chosen based on the average percent of missing data. Default value will be *m*=20 since sample size calculation assumed close to 20% drop-out rate.

As a first step to multiple imputation, an ordered list of variables to include in the subject-specific imputation model is constructed. Ordering is specified so that exact imputation results from final data are prespecified may be replicated in SAS (using seeds described below). The complete ordered list of variables for the imputation models for clinical improvement is below:

- Indicator of dalbavancin as study treatment (binary indicator, standard of care is the reference group
- Baseline pathogen (binary indicator, MRSA is the reference group)
- Age at enrollment
- Site of infection
- Duration of bacteremia
- Observed DOOR

The actual list of MI model variables for each subject-specific imputation model will follow the ordering above but omit variables with missing values. The below pseudo-code / SAS code outlines the creation of 20 multiple imputation datasets. Note that the seeds used in the actual analysis must follow the specification given in the pseudo-code, and subjects must be processed in the order described in the pseudo-code. The pseudo-code is for Day 70, but the general logic is also applicable to Day 42.

****

*Outcome variables: clinefficacy_D70

DEFINE i=index variable for subjects having clinical efficacy imputed.

Subjects requiring imputation are sorted in ascending order by PATID.

DEFINE N=number of subjects requiring imputation

DEFINE g&i=analysis dataset containing predictors and clinical efficacy for subjects with non-missing efficacy at Day 70 as well as subject i (only one subject with missing clinical efficacy is included). Note that subjects with complete clinical efficacy at Day 70 that are missing a value for one or more variables in the subject-specific imputation model are excluded.

DEFINE imp_g&i = g&i, with 20 imputed values for the missing clinical efficacy

added by PROC MI

DEFINE &&modelVars_&i = list of observed variables in subject i to be used for imputation of clinical efficacy

Step 1: Imputation model: This model will generate 20 datasets with each dataset containing original complete data along with imputed values for subjects with missing endpoint.

```
%do i=1 %to &N;
PROC MI data= g&i out= imp_g&i seed= 22131&i NIMPUTE=20 noprint;
     Var &&modelVars_&i clinefficacy_D70;
     monotone reg(clinefficacy_D70 = &&modelVars_&i;
run;
%end;
```

imp_g&i will be subset to contain only rows for the subjects with imputed clinical improvement and merged together and with ATP-5 data to create the twenty complete multiply imputed datasets

Step 2: Analysis model: This model will fit regression models to the 20 complete datasets to obtain parameter estimates for treatment success, clinical cure, microbiological success.

```
proc reg data= imp_g outest= out_clineff_D70 covout noprint;
model clinefficacy_D70= trt /clb alpha=0.05;
by _imputation_;
run;
```

Step 3: Combine estimates from models in step 2 to obtain overall estimates summarized over 20 imputed datasets;

```
proc mianalyze data= out_clineff_D70 alpha = 0.05;
modeleffects intercept trt;
ods output ParameterEstimates=parms_trts;
run;
```

****

The multiple imputation model for microbiologic success will use a similar algorithm and imputation variables as the clinical efficacy model described above.

9. SAFETY EVALUATION

9.1. Demographic and Other Baseline Characteristics

Summaries of race, ethnicity, sex, age, weight, height, body mass index (BMI), baseline pathogen, baseline QoL, will be presented for the ITT population by site (Table 9 and Table 13) and by treatment group (Table 11 and Table 15). Similar tables will be presented for the Safety population by site in Table 10 and Table 14 and by treatment group in Table 12 and Table 16. Age, weight, height, baseline QoL, and BMI will be summarized as continuous variables. The baseline QoL standardized score is obtained from the questions arising from the PROMIS physical function item bank (PROMIS Item Bank v2.0, short form 6b) item bank on the ARLG Bloodstream Infection QoL. Ethnicity will be categorized as Hispanic or Latino, or not Hispanic and not Latino. In accordance with NIH reporting policy, subjects may self-designate as belonging to more than one race or may refuse to identify a race, the latter reflected in the case report form (CRF) as "No" to each racial option. For subjects that were previously enrolled in this study, only information associated with their second enrollment will be reported and used for analysis.

Summaries of subject's medical history will be presented by MedDRA® V24.1 or higher system organ class (SOC) and treatment group (Table 17).

Individual subject listings will be presented for all demographics and baseline characteristics (Listing 6) and pre-existing medical conditions (Listing 7).

9.1.1. Prior and Concurrent Medical Conditions

Number and percentage of subjects' pre-existing and concurrent medical conditions will be presented by MedDRA system organ class and treatment group for the ITT population (Table 17).

Individual subject listings will be presented for all pre-existing medical conditions (Listing 7).

9.1.2. Prior or Concomitant Medications and Nondrug Interventions

Prior medication is defined as any medication taken before the date of the first dose of investigational product. Concomitant medication is defined as any medication started on or after the date of the first dose of investigational product. Concomitant medications will be coded to the Anatomical Therapeutic Classification using the WHO Drug Dictionary.

Both prior and concomitant medication use will be summarized by the number and proportion of subjects in each treatment group receiving each medication within each therapeutic class (ATC1 and ATC2) for the safety population in Table 108. If a subject took a specific medication multiple times or took multiple medications within a specific therapeutic class, that subject would be counted only once for the coded drug name or therapeutic class. A summary of subjects taking nondrug interventions by SOC is provided in Table 109.

Individual subject listings will be presented for all concomitant medications (Listing 18). A listing of nondrug interventions is provided in Listing 19.

9.2. Measurements of Treatment Compliance

Dates of first treatment will be summarized by site and treatment group in Table 5. Exposure to investigational product for the safety population will be summarized for treatment duration calculated as the number of doses of dalbavancin received for patients in the dalbavancin group. Number and percentage of subjects in the dalbavancin group receiving 1 or 2 doses of dalbavancin will be reported in Table 6. For subjects in the standard of care group, the duration of antibiotics will be calculated using the start and stop dates for standard of care antibiotics. Descriptive statistics for the duration of standard of care antibiotics (n, mean, standard deviation, minimum, median, and maximum) will be presented in Table 7. A listing of individual subjects' treatment duration is provided in Listing 8. A listing of all subjects who took at least one dose of study product is provided in Listing 1.

9.3. Adverse Events

Safety analyses will be based on the safety population. Safety will be assessed using descriptive statistics of AEs, SAEs, AESIs, AEs leading to study discontinuation, vital signs, and laboratory tests by treatment group. For each safety parameter, the last assessment made before the first dose of investigational product will be used as the baseline for all analyses of that safety parameter.

When calculating the incidence of adverse events (i.e., on a per subject basis), each subject will only be counted once and any repetitions of adverse events within a subject will be ignored; the denominator will be the number of subjects in the safety population. All adverse events reported will be included in the summaries and analyses. Safety analyses will be based on the safety population. Safety analyses encompass the component events to the DOOR calculation.

9.3.1. Treatment-Emergent Adverse Events

An AE (classified by preferred term) that occurs during the treatment period will be considered a treatment-emergent AE if it was not present before the first dose of investigational product or was present before the first dose of investigational product and increased in severity during the treatment period.

A summary of all treatment-emergent AEs in each treatment group will be tabulated by MedDRA SOC, preferred term (PT), and relationship to the investigational product in Table 66 and by MedDRA system organ class, high level group term (HLGT), and relationship to the investigational product in Table 68. A listing of all treatment-emergent AEs will be presented in Listing 12.

A summary of all treatment-emergent AEs will be tabulated by MedDRA SOC, PT, maximum severity, and causal relationship to the investigational product in Table 67. If more than one AE is coded to the same PT for the same subject, the subject will be counted only once for that PT using the most severe and most related occurrence for the summarization by severity and by causal relationship to the investigational product. Similarly, a summary of all treatment-emergent AEs will be tabulated by MedDRA, SOC, HLGT, maximum severity, and causal relationship to the investigational product in Table 69. If more than one AE is coded to the same HLGT for the same subject, the subject will be counted only once for that HLGT using the most severe and most related occurrence for the summarization by severity and by causal relationship to the investigational product.

The distribution of treatment-emergent AEs by severity and causal relationship to the investigational product will be summarized by treatment group in Table 70.

The distribution of AESIs by severity and causal relationship to the investigational product will be summarized by treatment group in Table 71.

The incidence of common (≥ 2% of patients in any treatment group) treatment-emergent AEs, on-therapy SAEs, AESIs, and AEs leading to premature discontinuation of the investigational product will be summarized by PT and treatment group and will be sorted by decreasing frequency for the investigational product Table 72. In addition, the incidence of fatal on-therapy SAEs (i.e., events that caused death) will be summarized separately by treatment group and PT in Table 73 and by treatment group and HLGT in Table 74. An SAE will be defined as an on-therapy SAE if it occurred during or after the first infusion of investigational product.

Bar charts of all adverse events will be presented by MedDRA SOC, severity and treatment group in Figure 28 and by MedDRA HLGT, severity, and treatment group in Figure 29. Forest plots of differences in risks of experiencing adverse events will be presented by SOC in Figure 30 and by HLGT in Figure 31. The 95% confidence intervals for difference in proportions will be computed using the Miettinen-Nurminen method.

9.4. Deaths, Serious Adverse Events, and Other Significant Adverse Events

Detailed narratives will be given for any deaths, SAEs, AESIs, and AEs leading to study product discontinuation that occurred during the study. Listings will include Subject IDs, AE description, AE onset date/end date, relationship to treatment, alternate etiology if not related, outcome, and duration of event (days).

Listings for SAEs, AESIs, subjects with AEs leading to discontinuation, and subjects who die (if any) will be presented in Table 75, Table 76, Table 77, and Table 78, respectively. A listing of all treatment-emergent AEs is presented in Table 79.

The number and percentage of subjects reporting SAEs, AEs Leading Discontinuation, Grade 3 or Higher AEs, AESIs, and ALT/AST Elevations will be reported by treatment group in Table 64 for the safety population by study arm and in Table 65 for standard of care antibiotics.

9.5. Pregnancies

For any subject in the Safety population who became pregnant during the study, every attempt will be made to follow these subjects to completion of pregnancy to document the outcome, including information regarding any complications with pregnancy and/or delivery. Listing 21, Listing 22, Listing 23, Listing 24, and Listing 25 will present any study pregnancies and their outcomes.

9.6. Clinical Laboratory Evaluations

Descriptive statistics for clinical laboratory values and changes from the baseline values at each assessment time point will be presented by treatment group for each clinical laboratory parameter starting with Table 83 and ending with Table 96 for serum chemistry parameters and starting with Table 98 and ending with Table 105 for hematology parameters. Forest plots of difference in risks of experiencing abnormal clinical laboratory events by clinical laboratory parameters will be presented in Figure 32 for hematology parameters and in Figure 33 for serum chemistry parameters. The 95% confidence intervals for difference in proportions will be computed using the Miettinen-Nurminen method.

The number and percentage of subjects with potentially clinically significant (PCS) post-baseline clinical laboratory values will be tabulated by treatment group in Table 82 for serum chemistry parameters and Table 97 for hematology parameters. The criteria for PCS laboratory values will be detailed in the statistical analysis plan. The percentages will be calculated relative to the number of subjects with available non-PCS

PRIVILEGED AND CONFIDENTIAL COMMUNICATION

baseline values and at least 1 post-baseline assessment. The numerator will be the total number of subjects with available non-PCS baseline values and at least 1 PCS post-baseline value. A supportive listing of subjects with PCS post-baseline values will be provided, including the PID number, study center number, and baseline and post-baseline values. A listing of PCS chemistry and hematology laboratory results is provided in Table 80 and Table 81, respectively. A listing of all AEs that occur in subjects who have PCS laboratory values or vital signs will also be provided in Listing 13. Individual laboratory results are provided in Listing 14 for chemistry laboratory parameters and in Listing 15 for hematology laboratory parameters.

9.7. Vital Signs and Physical Evaluations

Vital signs including pulse rate, systolic blood pressure (BP), diastolic BP, respiratory rate, and temperature will be collected at each visit from Visit 1 through Visit 7 and at the early termination visit. Descriptive statistics for vital sign values and changes from baseline will be presented at each timepoint by treatment group in Table 106 for vital signs values and Table 107 for changes from baseline. Individual vital signs measurements will be provided in Listing 16.

Targeted physical examinations will be performed, if indicated, based on a subject's medical history. A listing of physical exam findings will be presented in Listing 17. Echocardiogram will be performed if it has not already been done as part of standard of care for this episode of bacteremia/endocarditis. Either a transthoracic or transesophageal echocardiogram is acceptable, and results of this echocardiogram will be provided in Listing 20.

9.8. Concomitant Medications and Nondrug Interventions

Concomitant medications will be collected for the 30 days prior to ICF signing through Day 70 Visit. Concomitant medications will be coded to the Anatomical Therapeutic Classification using the WHO Drug Dictionary. The use of prior and concomitant medications taken during the study will be recorded on the CRFs. A by-subject listing of concomitant medication use will be presented (Listing 18). A listing of non-drug interventions will be presented in Listing 19. The use of concomitant medications during the study will be summarized by ATC1, ATC2 code, and study treatment for the Safety population (Table 108). A summary of subjects taking nondrug interventions by SOC is provided in Table 109.

PRIVILEGED AND CONFIDENTIAL COMMUNICATION

10. PHARMACOKINETICS

Analysis of PK endpoints will be provided in separate analysis plan document.

PRIVILEGED AND CONFIDENTIAL COMMUNICATION

11. OTHER ANALYSES

No other analyses are planned.

12. REPORTING CONVENTIONS

For the primary and secondary analyses percentages and probabilities will be reported to one decimal place. P-values ≥0.001 and ≤0.999 will be reported to 3 decimal places; p-values less than 0.001 will be reported as "<0.001"; p-values greater than 0.999 will be reported as "> 0.999". The mean, standard deviation, and any other statistics other than quantiles, will be reported to one decimal place greater than the original data. Quantiles, such as median, or minimum and maximum will use the same number of decimal places as the original data. Proportions will be presented as two decimal places; values <0.01 will be presented as "<0.01". Percentages will be reported to the nearest whole number; values < 1% will be presented as "<1" and values > 99% but below 100% will be presented as ">99". Estimated parameters, not on the same scale as raw observations (e.g., regression coefficients) will be reported to 3 significant figures.

13. TECHNICAL DETAILS

SAS version 9.4 or above or R version 3.2 or above will be used to perform analyses and to generate all tables, figures and listings.

PRIVILEGED AND CONFIDENTIAL COMMUNICATION

14. SUMMARY OF CHANGES IN THE CONDUCT OF THE STUDY OR PLANNED ANALYSES

No changes in the conduct of the study or planned analysis.

15. REFERENCES

1. Marshall, A., Altman, D.G., Holder, R.L. and Royston, P., 2009. Combining estimates of interest in prognostic modelling studies after multiple imputation: current practice and guidelines. BMC medical research methodology, 9(1), p.1.
2. Halperin, M., Hamdy, M.I., and Thall, P.F. Distribution-free confidence intervals for a parameter of Wilcoxon-Mann-Whitney type for ordered categories and progressive censoring. Biometrics. 1989 Jun;45(2):509-21. PMID: 2765635.
3. Allison, P. (2005). Imputation of categorical variables with PROC MI.
4. Horton, N.J., Lipsitz, S.R., and Parzen, M. (2003) A potential for bias when rounding in multiple imputation. American Statistician 57: 229-232.
5. Rubin, D. B., and Schenker, N. (1986). Multiple Imputation for Interval Estimation from Simple Random Samples with Ignorable Nonresponse. Journal of the American Statistical Association, 81, 366–374.
6. Miettinen, O., and Nurminen, M.M. Comparative analysis of two rates. Statistics in Medicine 4 2 (1985): 213-26.
7. Evans, S., Li, L. and Wei, L. Data monitoring in clinical trials using prediction. Drug Inform Journal 41, 73-742 (2007).
8. Li, L., Evans, S., Uno, H., and Wei, L. A graphical tool for data monitoring in clinical trials. Statistical Biopharmaceutical Research. 2009; 1:348-355.
9. Holland TL, Raad I, Boucher HW, et al. Effect of Algorithm-Based Therapy vs Usual Care on Clinical Success and Serious Adverse Events in Patients with Staphylococcal Bacteremia: A Randomized Clinical Trial. JAMA. 2018;320(12):1249-1258.
10. Baddour LM, Wilson WR, Bayer AS, et al. Infective Endocarditis in Adults: Diagnosis, Antimicrobial Therapy, and Management of Complications: A Scientific Statement for Healthcare Professionals from the American Heart Association. Circulation.2015;132(15):1435-1486
11. Lepak A, Marchillo K, VanHecker J, and Andes D. Impact of Glycopeptide Resistance in *Staphylococcus aureus* on the Dalbavancin In Vivo Pharmacodynamic Target. Antimicrob Agents Chemother. 2015;59(12):7833-7836.
12. Lachin JL. Introduction to Sample Size Determination and Power Analysis for Clinical Trials. Controlled Clinical Trials 198; 2:93-113
13. Corey GR. Staphylococcus aureus bloodstream infections: definitions and treatment. Clin Infect Dis. 2009;48 Suppl 4: S254-259.
14. King HA, Doernberg SB, Miller J, et al. Patients' experiences with Staphylococcus aureus and Gram-negative bacterial bloodstream infections: A qualitative descriptive study and concept elicitation phase to inform measurement of patient-reported quality of life. Clin infect Dis 2021;73(2):237-247.
15. King HA, Doernberg SB, Grover K, et al. Patients experiences with Staphylococcus aureus and Gram-negative bacterial bloodstream infections: results from cognitive interviews to inform assessment of health-related quality of life. Open Forum Infect Dis 2021;9(2): ofab622

PRIVILEGED AND CONFIDENTIAL COMMUNICATION

16. LISTING OF TABLES, FIGURES, AND LISTINGS

Table, figure, and listing shells are presented in Appendices 1, 2, and 3.

APPENDICES

APPENDIX 1. TABLE MOCK-UPS

LIST OF TABLES

Table 1: Schedule of Study Procedures 65

Table 2: Distribution of Protocol Deviations by Category, Type, and Treatment Group – ITT Population....67

Table 3: Subject Disposition by Treatment Group 68

Table 4: Analysis Populations by Treatment Group – ITT Population 69

Table 5: Dates of First Treatment by Site and Treatment Group 70

Table 6: Treatment Compliance for the Dalbavancin Treatment Group – Safety Population....71

Table 7: Treatment Compliance in the Standard of Care Treatment Group Measured by Number of Days – Safety Population 72

Table 8: Ineligibility Summary of Screen Failures....73

Table 9: Summary of Categorical Demographic and Baseline Characteristics by Site – ITT Population....74

Table 10: Summary of Categorical Demographic and Baseline Characteristics by Site – Safety Population....74

Table 11: Summary of Categorical Demographic and Baseline Characteristics by Treatment Group – ITT Population 75

Table 12: Summary of Categorical Demographic and Baseline Characteristics by Treatment Group – Safety Population 76

Table 13: Summary of Continuous Demographic and Baseline Characteristics by Site – ITT Population....77

Table 14: Summary of Continuous Demographic and Baseline Characteristics by Site – Safety Population....77

Table 15: Summary of Continuous Demographic and Baseline Characteristics by Treatment Group – ITT Population 78

Table 16: Summary of Continuous Demographic and Baseline Characteristics by Treatment Group – Safety Population 79

Table 17: Summary of Subjects with Pre-Existing Medical Conditions by MedDRA System Organ Class and Treatment Group – ITT Population....80

Table 18: Percentage of Subjects with Missing Data by Study Endpoint, Timepoint, and Treatment Group – ITT Analysis Population 81

Table 19: Percentage of Subjects with Missing Data by Study Endpoint, Timepoint, and Treatment Group – mITT Analysis Population 81

PRIVILEGED AND CONFIDENTIAL COMMUNICATION

Table 20: Analysis of DOOR at Day 70 – ITT Analysis Population.........82
Table 21: Analysis of DOOR at Day 70 – mITT Analysis Population 82
Table 22: Analysis of DOOR at Day 42 – ITT Analysis Population.........83
Table 23: Analysis of DOOR at Day 42 – mITT Analysis Population 83
Table 24: Analysis of DOOR at Day 42 and Day 70 – CE Analysis Population 84
Table 25: Subgroup Analysis of DOOR with Tie-Breaking by Timepoint – ITT Analysis Population 85
Table 26: Subgroup Analysis of DOOR with Tie-Breaking by Timepoint – mITT Analysis Population 86
Table 27: Subgroup Analysis of DOOR with Tie-Breaking by Timepoint – CE Analysis Population 87
Table 28: Analysis of DOOR Categories – ITT Analysis Population 88
Table 29: Analysis of DOOR Categories – mITT Analysis Population 88
Table 30: Analysis of DOOR Categories – CE Analysis Population 88
Table 31: Cumulative Proportions of DOOR - ITT Analysis Population 89
Table 32: Cumulative Proportions of DOOR – mITT Analysis Population.........89
Table 33: Cumulative Proportions of DOOR – CE Analysis Population.........89
Table 34: Summary of Expected Numbers Gained Loss at Day 42 and Day 70 by Analysis Population 90
Table 35: Analysis of Clinical Efficacy at Day 70 Using ITT and mITT Analysis Populations 91
Table 36: Analysis of Clinical Efficacy at Day 42 Using ITT and mITT Analysis Populations 91
Table 37: Analysis of Clinical Efficacy at Day 42 and Day 70 Using CE Analysis Population 92
Table 38: Subgroup Analysis of Clinical Efficacy by Timepoint – ITT Analysis Population 93
Table 39: Subgroup Analysis of Clinical Efficacy by Timepoint – mITT Analysis Population 94
Table 40: Subgroup Analysis of Clinical Efficacy by Timepoint – CE Analysis Population 95
Table 41: Summary of Clinical DOOR Components at Day 70 – ITT Analysis Population 96
Table 42: Summary of Clinical DOOR Components at Day 70 – mITT Analysis Population 96

Table 43: Summary of Clinical DOOR Components at Day 70 – CE Analysis Population 96

Table 44: Summary of Clinical DOOR Components at Day 42 – ITT Analysis Population 96

Table 45: Summary of Clinical DOOR Components at Day 42 – mITT Analysis Population 96

Table 46: Summary of Clinical DOOR Components at Day 42 – CE Analysis Population 96

Table 47: Summary of DOOR by Component at Day 70 – ITT Analysis Population 97

Table 48: Summary of DOOR by Component at Day 42 – ITT Analysis Population 97

Table 49: Analysis of Clinical Failure at Day 70 Using Weighted GEE and Weighted GLMM Models- ITT Analysis Population 98

Table 50: Analysis of Microbiologic Success Using ITT or mITT Analysis Populations 99

Table 51: Analysis of Microbiologic Success at Day 42 and Day 70 Using CE Analysis Population 101

Table 52: Subgroup Analysis of Microbiologic Success by Timepoint – ITT Analysis Population 102

Table 53: Subgroup Analysis of Microbiologic Success by Timepoint – mITT Analysis Population 102

Table 54: Subgroup Analysis of Microbiologic Success by Timepoint – CE Analysis Population 103

Table 55: Distribution of DOOR at Day 42 and Day 70 Using ITT, mITT, and CE Analysis Populations 104

Table 56: Distribution of DOOR at Day 42 by Subgroup Categories – ITT Analysis Population 105

Table 57: Distribution of DOOR at Day 70 by Subgroup Categories – ITT Analysis Population 105

Table 58: Summary Statistics of QoL Scores from the ARLG Bloodstream Infection QoL Measure at Day 42 and Day 70 Using ITT, mITT, and CE Analysis Populations 106

Table 59: Summary Statistics of QoL Scores Using the EQ-5D-5L Instrument at Day 42 and Day 70 Using ITT, mITT, and CE Analysis Populations 107

Table 60: Summary Statistics of QoL Scores Using the PROMIS Global Health Short Form at Day 42 and Day 70 Using ITT, mITT, and CE Analysis Populations 107

Table 61: Predictive Interval Plots (PIPS) Statistics for the Probability of Higher DOOR in the Dalbavancin Group at Day 70 – ITT Analysis Population 108

Table 62: Predictive Interval Plots (PIPS) Statistics for the Rates of Clinical Efficacy at Day 70 – ITT Analysis Population 108

Table 63: Number and Percentage of Subjects with Late Recurrence - Osteomyelitis Population 109

Table 64: Number and Percentage of Subjects reporting SAEs, AEs Leading Discontinuation, Grade 3 or Higher AEs, AESIs, and ALT/AST Elevations by Treatment Group - Safety Population 110

Table 65: Number and Percentage of subjects reporting SAEs, AEs Leading Discontinuation, Grade 3 or Higher AEs, AESIs, and ALT/AST Elevations by Standard of Care Antibiotics – Standard of Care Arm, Safety Population 111

Table 66: Summary of Treatment-Emergent Adverse Events by MedDRA System Organ Class and Preferred Term, Relationship, and Treatment Group 112

Table 67: Summary of Treatment-Emergent Adverse Events by MedDRA System Organ Class and Preferred Term, Maximum Severity, Relationship, and Treatment Group 113

Table 68: Summary of Treatment-Emergent Adverse Events by MedDRA System Organ Class and High Level Group Term, Relationship, and Treatment Group 114

Table 69: Summary of Treatment-Emergent Adverse Events by MedDRA System Organ Class and High Level Group Term, Maximum Severity, Relationship, and Treatment Group 115

Table 70: Summary of Treatment-Emergent Adverse Events by MedDRA System Organ Class, Maximum Severity, Relationship, and Treatment Group 116

Table 71: Summary of AESIs by MedDRA System Organ Class, Maximum Severity, Relationship, and Treatment Group 117

Table 72: Summary of Treatment-Emergent AEs, On-therapy SAEs, AESIs, and AEs Leading Discontinuation Occurring in 2% of Subjects in Any Treatment Group by MedDRA Preferred Term, and Treatment Group – Safety Population 118

Table 73: Summary of Fatal on-Therapy SAEs by MedDRA Preferred Term, and Treatment Group – Safety Population 119

Table 74: Summary of Fatal on-Therapy SAEs by MedDRA High Level Group Term, and Treatment Group – Safety Population 120

Table 75: Listing of Serious Adverse Events 121

Table 76: Listing of Adverse Events of Special Interest 121

Table 77: Listing of Adverse Events Leading to Discontinuation 121

Table 78: Listing of Subjects whose Outcome was Fatal During the Study 121

Table 79: Listing of Treatment-Emergent Adverse Events 122

Table 80: Listing of Potentially Clinically Significant Laboratory Results – Chemistry 124

Table 81: Listing of Potentially Clinically Significant Laboratory Results – Hematology....125

Table 82: Number and Percentage of Subjects with Potentially Clinically Significant Post-Baseline Laboratory Values by Parameter and Treatment Group – Chemistry Parameters....126

Table 83: Laboratory Summary Statistics by Parameter, Time Point, and Treatment Group – Serum Chemistry, Sodium....127

Table 84: Laboratory Summary Statistics by Parameter, Time Point, and Treatment Group – Serum Chemistry, Potassium....127

Table 85: Laboratory Summary Statistics by Parameter, Time Point, and Treatment Group – Serum Chemistry, Calcium....127

Table 86: Laboratory Summary Statistics by Parameter, Time Point, and Treatment Group – Serum Chemistry, Chloride....127

Table 87: Laboratory Summary Statistics by Parameter, Time Point, and Treatment Group – Serum Chemistry, Bicarbonate....127

Table 88: Laboratory Summary Statistics by Parameter, Time Point, and Treatment Group – Serum Chemistry, Glucose....127

Table 89: Laboratory Summary Statistics by Parameter, Time Point, and Treatment Group – Serum Chemistry, Blood Urea Nitrogen....127

Table 90: Laboratory Summary Statistics by Parameter, Time Point, and Treatment Group – Serum Chemistry, Creatinine....128

Table 91: Laboratory Summary Statistics by Parameter, Time Point, and Treatment Group – Serum Chemistry, Total Protein....128

Table 92: Laboratory Summary Statistics by Parameter, Time Point, and Treatment Group – Serum Chemistry, Alkaline Phosphatase....128

Table 93: Laboratory Summary Statistics by Parameter, Time Point, and Treatment Group – Serum Chemistry, Albumin....128

Table 94: Laboratory Summary Statistics by Parameter, Time Point, and Treatment Group – Serum Chemistry, Total Bilirubin....128

Table 95: Laboratory Summary Statistics by Parameter, Time Point, and Treatment Group – Serum Chemistry, AST....128

Table 96: Laboratory Summary Statistics by Parameter, Time Point, and Treatment Group – Serum Chemistry, ALT....128

Table 97: Number and Percentage of Subjects with Potentially Clinically Significant Post-Baseline Laboratory Values by Parameter and Treatment Group – Hematology Parameters....129

Table 98: Laboratory Summary Statistics by Parameter, Time Point, and Treatment Group – Hematology, Absolute WBC....130

PRIVILEGED AND CONFIDENTIAL COMMUNICATION

Table 99: Laboratory Summary Statistics by Parameter, Time Point, and Treatment Group – Hematology, Erythrocyte 130
Table 100: Laboratory Summary Statistics by Parameter, Time Point, and Treatment Group – Hematology, Hemoglobin 130
Table 101: Laboratory Summary Statistics by Parameter, Time Point, and Treatment Group – Hematology, Hematocrit 130
Table 102: Laboratory Summary Statistics by Parameter, Time Point, and Treatment Group – Hematology, Platelet Count........130
Table 103: Laboratory Summary Statistics by Parameter, Time Point, and Treatment Group – Hematology, Mean Corpuscular Volume........130
Table 104: Laboratory Summary Statistics by Parameter, Time Point, and Treatment Group – Hematology, Mean Corpuscular Hemoglobin........130
Table 105: Laboratory Summary Statistics by Parameter, Time Point, and Treatment Group – Hematology, Mean Corpuscular Hemoglobin Concentration........130
Table 106: Summary of Vital Signs Values by Vital Sign Parameter, Visit, and Treatment Group........131
Table 107: Summary of Change from Baseline Vital Signs Values by Vital Sign Parameter, Visit, and Treatment Group........133
Table 108: Number and Percentage of Subjects with Prior and Concurrent Medications by WHO Drug Classification and Treatment Group 135
Table 109: Number and Percentage of Subjects with Nondrug Interventions by MedDRA System Organ Class and Treatment Group – ITT Population 136

9.5.1 Efficacy and Safety Measurements Assessed and Flow Chart

Table 1: Schedule of Study Procedures

	Induction Period	Screening/ Enrollment	Open Label Treatment Period				Post-treatment Follow-up Period		
	Visit 0 (Pre-Screening, Day -10 to Day 1)	Visit 1 (Day –1 to Day 1)	Visit 2 (Baseline, Day 1)	Visit 3 (Day 8 ± 1 day)	Visit 4 (Day 22 ± 2 days)	Visit 5 (Day 42 ± 3 days)	Visit 6 (TOC, Day 70 ± 7 days)[a]	ET[b]	Visit 7 (Day 180 ± 14 days, Osteomyelitis group)[a]
Informed Consent		X							
Dalbavancin[c]			X	X					
Standard of care antibiotic therapy[c]	X	X	X (Duration 28-56 days)						
Medical history[d]		X	X			X	X	X	
Medication history[e]		X							
Randomization			X						
AEs/AESIs/SAEs			X	X	X	X	X	X	
Hematology and serum chemistry blood sampling[f]		X		X[g]	X	X			
Coagulation lab tests[f]		X							
Pregnancy test[h]		X							
PK sampling[i]			X	X	X	X	X	X	
Vital signs[j]		X	X	X[k]	X	X	X	X	X
Physical examination[l]		X	X	X	X	X	X	X	X
Echocardiogram[m]		X							
Investigator assessment of efficacy						X	X	X	X
Concomitant medications[n]		X	X	X	X	X	X	X	X
Concomitant nondrug interventions		X	X	X	X	X	X	X	X
QoL assessment[o]			X	X	X	X	X	X	X

PRIVILEGED AND CONFIDENTIAL COMMUNICATION

Table 1: Schedule of Study Procedures (continued)

	**Induction Period**	**Screening/ Enrollment**	**Open Label Treatment Period**				**Post-treatment Follow-up Period**		
	Visit 0 (Pre-Screening, Day -10 to Day 1)	Visit 1 (Day –1 to Day 1)	Visit 2 (Baseline, Day 1)	Visit 3 (Day 8 ± 1 day)	Visit 4 (Day 22 ± 2 days)	Visit 5 (Day 42 ± 3 days)	Visit 6 (TOC, Day 70 ± 7 days)[a]	ET[b]	Visit 7 (Day 180 ± 14 days, Osteomyelitis group)[a]

AEs = adverse events; AESIs = adverse events of special interest; eCRF = electronic case report form; ET = Early Termination; PK = pharmacokinetic; SAE = serious adverse event

[a]Telephone visit permissible if in-person visit is not possible; in person visit still preferred.

[b]Patients who prematurely discontinue therapy should have an ET Visit within 72 hours.

[c]All subjects will be receiving standard of care prior to randomization; after randomization, subjects will receive either dalbavancin or standard of care based on their assigned treatment group.

[d]Includes targeted/pertinent medical and surgical history only

[e]A complete medication history will be completed through 30 days prior to ICF signing; an extended 60 day review will be conducted for dalbavancin and oritavancin given the long half-lives of both drugs.

[f]Visit 1 hematology, coagulation lab tests (PT, PTT, and/or INR) and serum chemistry will be done in order to qualify the patient for the study, if not already collected per standard of care within 48 hours prior to randomization.

[g]A serum creatinine assessment will be required within the 72 hours prior to the 2nd (Day 8) dalbavancin dose. Whether a serum creatinine must be repeated on Day 8 will be at the discretion of the site investigator based upon stability of the serum creatinine in the preceding 72 hours and whether the serum creatinine is near the threshold where dose adjustment would be necessary (e.g., near 30 mL/min).

[h]Women of childbearing potential only, if not already performed (see Appendix B Definitions in the protocol); ensure test is negative within 48 hours before randomization. If the serum test results cannot be obtained before randomization, a urine pregnancy test may be used for enrollment.

[i]Dalbavancin PK samples will be drawn only for subjects receiving dalbvacancin. PK samples will be drawn at Day 1 prior to dose, at end of infusion ± 10 minutes, 6 ± 2 hours post end of dose, 12 ± 4 hours post end of dose, 24 ± 6 hours post end of dose), Day 8 (prior to 2nd dose), Day 22 ± 2 days (at time of clinic visit), day 42 ± 3 days, day 70 ± 7 days, and with any ET visit. Each sample must be accompanied by draw time and date.

[j]Vital signs include blood pressure, respiration rate, pulse rate, and temperature.

[k]Day 8 vital signs not required for subjects receiving SOC antibiotics if discharge occurs prior to day 8.

[l]A physical examination (including general appearance, examination of head, eyes, ears, nose, throat, neck, skin, heart, lungs, abdomen, neurologic system, musculoskeletal system, extremities, height, and body weight) will be done at Screening (Visit 1). If height or weight is not obtainable (eg, patient is immobilized), use the last known or stated height and weight. At subsequent visits, targeted physical exams will focus on changes from prior exams and on the evaluation of newly reported symptoms.

[m]Transthoracic echocardiogram or, if clinically indicated, transesophageal echocardiogram to be performed (local laboratory), unless one has been performed as standard of care for this episode of bacteremia/endocarditis

[n]All concomitant medications from Screening (Visit 1) through Day 42 (± 3 days) (Visit 5) must be recorded in the patient's medical record and on the eCRFs. Between the Day 42 Visit and Day 70 Visit, all concomitant medications for an AE or any antibacterial therapy should be recorded in the patient's medical record and on the eCRF.

[o]QoL assessments include the ARLG Bloodstream Infection QoL Measure (Appendix C in the protocol), the EQ-5D-5L (https://euroqol.org/eq-5d-instruments/sample-demo/), and the PROMIS Global Health Short Form (http://www.healthmeasures.net/administrator/components/com_instruments/uploads/Global%20Health%20Scale%20v1.2%2008.22.2016.pdf).

10.2 Protocol Deviations

Table 2: Distribution of Protocol Deviations by Category, Type, and Treatment Group – ITT Population

Category	Deviation Type	Dalbavancin (N=X)		Standard of Care (N=X)		All Subjects (N=X)	
		No. of Subj.	No. of Dev.	No. of Subj.	No. of Dev.	No. of Subj.	No. of Dev.
Eligibility/enrollment	Any type	x	x	x	x	x	x
	Did not meet inclusion criterion	x	x	x	x	x	x
	Met exclusion criterion	x	x	x	x	x	x
	ICF not signed prior to study procedures	x	x	x	x	x	x
	Other	x	x	x	x	x	x
Treatment administration schedule	Any type	x	x	x	x	x	x
	Out of window visit	x	x	x	x	x	x
	Missed visit/visit not conducted	x	x	x	x	x	x
	Missed treatment administration	x	x	x	x	x	x
	Delayed treatment administration	x	x	x	x	x	x
	Other	x	x	x	x	x	x
Follow-up visit schedule	Any type	x	x	x	x	x	x
	Out of window visit	x	x	x	x	x	x
	Missed visit/visit not conducted	x	x	x	x	x	x
	Other	x	x	x	x	x	x
Protocol procedure/assessment	Any type	x	x	x	x	x	x
	Incorrect version of ICF signed	x	x	x	x	x	x
	Blood not collected	x	x	x	x	x	x
	Other specimen not collected	x	x	x	x	x	x
	Too few aliquots obtained	x	x	x	x	x	x
	Specimen result not obtained	x	x	x	x	x	x
	Required procedure not conducted	x	x	x	x	x	x
	Required procedure done incorrectly	x	x	x	x	x	x
	Study product temperature excursion	x	x	x	x	x	x
	Specimen temperature excursion	x	x	x	x	x	x
	Other	x	x	x	x	x	x
Treatment administration	Any type	x	x	x	x	x	x
	Required procedure done incorrectly	x	x	x	x	x	x
	Study product temperature excursion	x	x	x	x	x	x
	Other	x	x	x	x	x	x
N= Number of subjects in the ITT Population.							

14.1 Description of Study Subjects

14.1.1 Disposition of Subjects

Table 3: Subject Disposition by Treatment Group

Subject Disposition	Dalbavancin (N=X)		Standard of Care (N=X)		All Subjects (N=X)	
	n	%	n	%	n	%
Screened	--	--	--	--	x	--
Enrolled	x	100	x	100	x	100
Randomized	x	xx	x	xx	x	xx
Received Treatment	x	xx	x	xx	x	xx
Completed the Treatment Period[a]	x	xx	x	xx	x	xx
Completed Treatment[b]	x	xx	x	xx	x	xx
Discontinued During the Treatment Period[c]	x	xx	x	xx	x	xx
Discontinued Treatment[b]	x	xx	x	xx	x	xx
Completed Day 22 Visit	x	xx	x	xx	x	xx
Completed Day 42 Visit	x	xx	x	xx	x	xx
Completed Day 70 Visit[c]	x	xx	x	xx	x	xx
Completed Day 180 Visit[d]	x	xx	x	xx	x	xx

N= Number of subjects in the ITT Population.
[a] The treatment period is defined as the period between Day 1 and Day 42 Visit.
[b] The treatment period for this row is Day 1 until 8 weeks to include standard of care subjects who took antibiotics for 8 weeks.
[c] Refer to Listing 16.2.1 for reasons subjects discontinued or terminated early.
[d] Day 180 Visit will only be completed by subjects with osteomyelitis.

Table 4: Analysis Populations by Treatment Group – ITT Population

Analysis Populations	Reason Subjects Excluded	Dalbavancin (N=X)		Standard of Care (N=X)		All Subjects (N=X)	
		n	%	n	%	%	n
ITT	Any Reason						
	Subject not randomized						
Safety	Any Reason						
	Subject not treated with at least one dose of study product						
Modified ITT	Any Reason						
	Subject was excluded from ITT population						
	Subject not treated with at least one dose of study product						
Clinically Evaluable at Day 42	Any Reason						
	Subject was excluded from mITT population						
	Subject had missing data or major protocol deviation at Day 42[a]						
Clinically Evaluable at Day 70	Any Reason						
	Subject was excluded from mITT population						
	Subject had missing data or major protocol deviation at Day 70[a]						

N= Number of subjects in the ITT Population.
[a] Major protocol deviation for analysis population purposes will be defined as those that prevent the adjudication committee from evaluating the outcomes at the given day.

Table 5: Dates of First Treatment by Site and Treatment Group

[Implementation note: Replace site numbers by site names and sort the site list alphabetically.]

Site	Treatment Group	February 2021- December 2021	January 2022-December 2022
Any Site	Any Treatment	x	x
Any Site	Dalbavancin	x	x
	Standard of Care	x	x
Site 1	Dalbavancin	x	x
	Standard of Care	x	x
Site 2	Dalbavancin	x	x
	Standard of Care	x	x
Site 3	Dalbavancin	x	x
	Standard of Care	x	x
Site 4	Dalbavancin	x	x
	Standard of Care	x	x
[Repeat for all sites that enrolled at least one subject.]			

Table 6: Treatment Compliance for the Dalbavancin Treatment Group – Safety Population

	Dalbavancin (N=X)	
	**n**	**%**
Received 1 Dose of Dalbavancin	x	x
Received 2 Doses of Dalbavancin	x	x
Lowered dose of Dalbavancin received[a]	x	x
Interrupted/Incomplete dose of Dalbavancin received	x	x
Interrupted/Incomplete dose of Dalbavancin received	x	x

N = Number of subjects in the Safety Population who received at least one dose of Dalbavancin.
[a] Summarizes the number of subjects that received a lower dose of dalbavancin due to an absence of regular hemodialysis or peritoneal dialysis and CrCl < 30 mL/min.

Table 7: Treatment Compliance in the Standard of Care Treatment Group Measured by Number of Days – Safety Population

Standard of Care Antibiotics	Statistic	Standard of Care (N=X)
Any Standard of Care Antibiotic	n	x
	Mean	x.x
	Standard Deviation	x.x
	Median	x
	Minimum	x
	Maximum	x
Cefazolin	n	n
	Mean	x.x
	Standard Deviation	x.x
	Median	x
	Minimum	x
	Maximum	x
Nafcillin	n	x
	Mean	x.x
	Standard Deviation	x.x
	Median	x
	Minimum	x
	Maximum	x
Oxacillin	n	x
	Mean	x.x
	Standard Deviation	x.x
	Median	x
	Minimum	x
	Maximum	x
Vancomycin	n	x
	Mean	x.x
	Standard Deviation	x.x
	Median	x
	Minimum	x
	Maximum	x
Daptomycin	n	x
	Mean	x.x
	Standard Deviation	x.x
	Median	x
	Minimum	x
	Maximum	x
[Create additional rows to add any other Standard of Care antibiotics administered in this study.]		
N = Number of subjects in the Safety Population in the standard of care arm.<br>n = Number of subjects in the Safety Population who received the corresponding standard of care antibiotic.		

Table 8: Ineligibility Summary of Screen Failures

Inclusion/ Exclusion Category	Inclusion/ Exclusion Criterion	n[a]	%[b]
All Subjects	Total number of subjects failing any eligibility criterion or who were eligible but not randomized.	x	100
Inclusion and Exclusion	Number of subjects failing any eligibility criterion		
Inclusion	Any inclusion criterion	x	xx
	[inclusion criterion 1]	x	xx
	[inclusion criterion 2]	x	xx
	[inclusion criterion 3]	x	xx
	…		
Exclusion	Any exclusion criterion	x	xx
	[exclusion criterion 1]	x	xx
	[exclusion criterion 2]	x	xx
	[exclusion criterion 3]	x	xx
	…		
Eligible but Not Enrolled	Any Reason	x	xx
	[Reason 1]	x	xx
	[Reason 2]	x	xx
	…		

[a] More than one criterion may be marked per subject.
[b] Denominator for percentages is the total number of subjects not enrolled in this study which include screen failures and subjects eligible but not enrolled.

14.1.2 Demographic Data by Study Group

Table 9: Summary of Categorical Demographic and Baseline Characteristics by Site – ITT Population

Demographic Category		Sex		Ethnicity				Race							Baseline Pathogen	
Characteristic		Male	Female	Not Hispanic or Latino	Hispanic or Latino	Not Reported	Unknown	American Indian or Alaska Native	Asian	Native Hawaiian or Other Pacific Islander	Black or African American	White	Multi-Racial	Unknown	MRSA	MSSA
[Site 1] (N=X)	n	x	x	x	x	x	x	x	x	x	x	x	x	x		
	%	x	x	x	x	x	x	x	x	x	x	x	x	x		
[Site 2] (N=X)	n	x	x	x	x	x	x	x	x	x	x	x	x	x		
	%	x	x	x	x	x	x	x	x	x	x	x	x	x		
All Subjects (N=X)	n	x	x	x	x	x	x	x	x	x	x	x	x	x		
	%	x	x	x	x	x	x	x	x	x	x	x	x	x		

N = Number of subjects in the ITT Population.

Note: Will repeat for all sites.

Table 10: Summary of Categorical Demographic and Baseline Characteristics by Site – Safety Population

The table will repeat Table 9 limited to the safety population

Table 11: Summary of Categorical Demographic and Baseline Characteristics by Treatment Group – ITT Population

Variable	Characteristic	Dalbavancin (N=X)		Standard of Care (N=X)		All Subjects (N=X)	
		n	%	n	%	n	%
Sex	Male	x	xx	x	xx	x	xx
	Female						
Ethnicity	Not Hispanic or Latino	x	xx	x	xx	x	xx
	Hispanic or Latino						
	Not Reported						
	Unknown						
Race	American Indian or Alaska Native	x	xx	x	xx	x	xx
	Asian						
	Native Hawaiian or Other Pacific Islander						
	Black or African American						
	White						
	Multi-Racial						
	Unknown						
Baseline Pathogen	MRSA						
	MSSA						
PWID Status	PWID						
	Non-PWID						
Infectious Disease Consultation	Yes						
	No						
TEE Performed	Yes						
	No						
Underlying Site of Infection	Endovascular						
	Bone and Joint						
	Skin						
	Pulmonary						
	Other/Unknown						
Immunosuppression	Yes						
	No						
Duration of Initial Bacteremia (Days)	< 2						
	2-4						
	> 4						
	Other/Unknown						
Pre-randomization Antibiotics	[Antibiotic 1]						
	[Antibiotic 2]						
	[Antibiotic 3]						
	…						

Table 11: Summary of Categorical Demographic and Baseline Characteristics by Treatment Group – ITT Population (continued)

Variable	Characteristic	Dalbavancin (N=X)		Standard of Care (N=X)		All Subjects (N=X)	
		n	%	n	%	n	%
Comorbid Conditions	Myocardial infarction						
	Congestive heart failure						
	Peripheral vascular disease						
	Cerebrovascular disease						
	Dementia						
	Chronic pulmonary disease						
	Rheumatic disease						
	Peptic ulcer disease						
	Mild liver disease						
	Diabetes without chronic complications						
	Diabetes with chronic complications						
	Hemiplegia/paraplegia						
	Renal disease						
	Hemodialysis						
	Malignancy						
	Moderate to severe liver disease						
	Metastatic cancer						
	HIV/AIDS						
Baseline Infection Characteristics	Right-sided endocarditis						
	ABSSSI						
	Septic pulmonary emboli						
	Osteomyelitis, non-vertebral						
	Vertebral osteomyelitis						
	Septic arthritis						
	Catheter-associated bloodstream infection						
	Cardiac device infection						
	Intravascular graft infection						
	Prosthetic valve infection						
	Prosthetic joint infection						
	Pneumonia						

N = Number of subjects in the ITT Population.
PWID = Persons Who Inject Drugs.
TEE = Transesophageal Echocardiography.
ABSSSI = Acute Bacterial Skin and Skin Structure Infection.

Table 12: Summary of Categorical Demographic and Baseline Characteristics by Treatment Group – Safety Population

This table will repeat Table 11 limited to the safety population.

Table 13: Summary of Continuous Demographic and Baseline Characteristics by Site – ITT Population

Variable	n	Mean	Standard Deviation	Median	Minimum	Maximum
**[Site 1 (N=X)]**						
Age (years)	x	x.x	x.x	x.x	x	x
BMI (kg/m^2)	x	x.xx	x.xx	x.xx	x.x	x.x
Height (cm)	x	x.xx	x.xx	x.xx	x.x	x.x
Weight (kg)	x	x.xx	x.xx	x.xx	x.x	x.x
Baseline QoL[a]	x	x.xx	x.xx	x.xx	x.x	x.x
Creatinine Clearance (mL/min)	x	x.xx	x.xx	x.xx	x.x	x.x
Duration of Bacteremia (days)	x	x.x	x.x	x.x	x	x
**[Site 2 (N=X)]**						
Age (years)	x	x.x	x.x	x.x	x	X
BMI (kg/m^2)	x	x.x	x.x	x.x	x	X
Height (cm)	x	x.x	x.x	x.x	x	X
Weight (kg)	x	x.x	x.x	x.x	x	X
Baseline QoL[a]	x	x.xx	x.xx	x.xx	x.x	x.x
Creatinine Clearance (mL/min)	x	x.xx	x.xx	x.xx	x.x	x.x
Duration of Bacteremia (days)	x	x.x	x.x	x.x	x	x
**[Repeat for all sites and all subjects]**						

N = Number of subjects in the ITT Population.
n = Number of subjects in the ITT Population with non-missing values for the corresponding baseline characteristic.
[a] Baseline QoL standardized score is obtained from the questions arising from the PROMIS physical function item bank (PROMIS Item Bank v2.0, short form 6b) item bank on the ARLG Bloodstream Infection QoL.

Table 14: Summary of Continuous Demographic and Baseline Characteristics by Site – Safety Population

This table will repeat Table 13 limited to the safety population.

Table 15: Summary of Continuous Demographic and Baseline Characteristics by Treatment Group – ITT Population

Variable	Statistic	Dalbavancin (N=X)	Standard of Care (N=X)	All Subjects (N=X)
Age (years)	n	x	x	x
	Mean	x.x	x.x	x.x
	Standard Deviation	x.x	x.x	x.x
	Median	x.x	x.x	x.x
	Minimum	x	x	x
	Maximum	x	x	x
BMI (kg/m^2)	n	x.xx	x.xx	x.xx
	Mean	x	x	x
	Standard Deviation	x.xx	x.xx	x.xx
	Median	x.xx	x.xx	x.xx
	Minimum	x.x	x.x	x.x
	Maximum	x.x	x.x	x.x
Height (cm)	n	x	x	x
	Mean	x.xx	x.xx	x.xx
	Standard Deviation	x.xx	x.xx	x.xx
	Median	x.xx	x.xx	x.xx
	Minimum	x	x	x
	Maximum	x	x	x
Weight (kg)	n	x	x	x
	Mean	x.xx	x.xx	x.xx
	Standard Deviation	x.xx	x.xx	x.xx
	Median	x.xx	x.xx	x.xx
	Minimum	x.x	x.x	x.x
	Maximum	x.x	x.x	x.x
Baseline QoL[a]	n	x	x	x
	Mean	x.xx	x.xx	x.xx
	Standard Deviation	x.xx	x.xx	x.xx
	Median	x.xx	x.xx	x.xx
	Minimum	x.x	x.x	x.x
	Maximum	x.x	x.x	x.x

Table 15: Summary of Continuous Demographic and Baseline Characteristics by Treatment Group – ITT Population (continued)

Variable	Statistic	Dalbavancin (N=X)	Standard of Care (N=X)	All Subjects (N=X)
Creatinine Clearance (mL/min)	n	x	x	x
	Mean	x.xx	x.xx	x.xx
	Standard Deviation	x.xx	x.xx	x.xx
	Median	x.xx	x.xx	x.xx
	Minimum	x.x	x.x	x.x
	Maximum	x.x	x.x	x.x
Duration of Bacteremia (days)	n	x	x	x
	Mean	x.xx	x.xx	x.xx
	Standard Deviation	x.xx	x.xx	x.xx
	Median	x.xx	x.xx	x.xx
	Minimum	x.x	x.x	x.x
	Maximum	x.x	x.x	x.x

N = Number of subjects in the ITT Population.
n = Number of subjects in the ITT Population with non-missing values for the corresponding baseline characteristic.
[a] Baseline QoL standardized score is obtained from the questions arising from the PROMIS physical function item bank (PROMIS Item Bank v2.0, short form 6b) item bank on the ARLG Bloodstream Infection QoL.

Table 16: Summary of Continuous Demographic and Baseline Characteristics by Treatment Group – Safety Population

This table will repeat Table 15 limited to the safety population.

14.1.3 Prior and Concurrent Medical Conditions

Table 17: Summary of Subjects with Pre-Existing Medical Conditions by MedDRA System Organ Class and Treatment Group – ITT Population

	Dalbavancin (N=X)		Standard of Care (N=X)		All Subjects (N=X)	
**MedDRA System Organ Class**	**n**	**%**	**n**	**%**	**n**	**%**
Any SOC	x	xx	x	xx	x	xx
[SOC 1]	x	xx	x	xx	x	xx
[SOC 2]	x	xx	x	xx	x	xx
[repeat for all SOC]						

N = Number of subjects in the ITT population.
n = Number of subjects reporting medical history within the specified SOC. A subject is only counted once per SOC.

14.2 Efficacy Data

Table 18: Percentage of Subjects with Missing Data by Study Endpoint, Timepoint, and Treatment Group – ITT Analysis Population

Endpoint	Dalbavancin (N=X)		Standard of Care (N=X)		All Subjects (N=X)	
	n	%	n	%	n	%
**Day 42**						
DOOR						
Clinical efficacy						
Microbiologic Success						
Clinical Failure						
**Day 70**						
DOOR						
Clinical efficacy						
Microbiologic Success						
Clinical Failure						

N = Number of subjects in the ITT population in the respective treatment group.
n = Number of subjects with missing data.

Table 19: Percentage of Subjects with Missing Data by Study Endpoint, Timepoint, and Treatment Group – mITT Analysis Population

This table will repeat Table 18 limited to the mITT population.

Table 20: Analysis of DOOR at Day 70 – ITT Analysis Population

[Implementation Note: This table will be included in the interim analysis.]

Model	Tie-Breaking[a]	Subjects with non-missing DOOR n (%)	Subjects with missing DOOR n (%)	Pr(Higher DOOR in Dalbavancin Arm)[b] (95% CI)[c]
IPW	Tie-Breaking	x (x)	x (x)	x.xx (x.xx, x.xx)
	No Tie-Breaking	x (x)	x (x)	x.xx (x.xx, x.xx)
Multiple Imputation	Tie-Breaking	x (x)	x (x)	x.xx (x.xx, x.xx)
	No Tie-Breaking	x (x)	x (x)	x.xx (x.xx, x.xx)

[a]DOOR analysis with tie-breaking is the primary analysis.
[b]Probability of Higher DOOR in Dalbavancin arm at Day 70 + 0.5 Probability of Equal DOOR.
[c]95% CI obtained through inversion of the F-test for MI or through bootstrapping for IPW. Superiority of Dalbavancin is concluded if the lower bound of the 95% CI for the DOOR probability is above 0.5.

Table 21: Analysis of DOOR at Day 70 – mITT Analysis Population

This table will repeat Table 20 limited to the mITT population.

Table 22: Analysis of DOOR at Day 42 – ITT Analysis Population

Model	Tie-Breaking[a]	Subjects with non-missing DOOR n (%)	Subjects with missing DOOR n (%)	Pr(Higher DOOR in Dalbavancin Arm)[b] (95% CI)[c]
IPW	Tie-Breaking	x (x)	x (x)	x.xx (x.xx, x.xx)
	No Tie-Breaking	x (x)	x (x)	x.xx (x.xx, x.xx)
Multiple Imputation	Tie-Breaking	x (x)	x (x)	x.xx (x.xx, x.xx)
	No Tie-Breaking	x (x)	x (x)	x.xx (x.xx, x.xx)

[a] DOOR analysis with tie-breaking is the primary analysis.
[b] Probability of Higher DOOR in Dalbavancin arm at Day 42 + 0.5 Probability of Equal DOOR.
[c] 95% CI obtained through inversion of the F-test for MI or through bootstrapping for IPW. Superiority of Dalbavancin is concluded if the lower bound of the 95% CI for the DOOR probability is above 0.5.

Table 23: Analysis of DOOR at Day 42 – mITT Analysis Population

This table will repeat Table 22 limited to the mITT population.

Table 24: Analysis of DOOR at Day 42 and Day 70 – CE Analysis Population

Timepoint	Tie-Breaking	N	Pr(Higher DOOR)[a]	Halperin 95% CI[b]	Bootstrap 95% CI[c]
Day 42	Tie-Breaking	x	x.xx	x.xx, x.xx	x.xx, x.xx
	No Tie-Breaking	x	x.xx	x.xx, x.xx	x.xx, x.xx
Day 70	Tie-Breaking	x	x.xx	x.xx, x.xx	x.xx, x.xx
	No Tie-Breaking	x	x.xx	x.xx, x.xx	x.xx, x.xx

N = Number of subjects with complete data in the given analysis population.
[a] Probability of Higher DOOR in Dalbavancin Arm at Day 42 (or Day 70) + 0.5 Probability of Equal DOOR.
[b] 95% CI obtained using the method described in Halperin et. al [2].
[c] 95% CI obtained using bootstrap.

Table 25: Subgroup Analysis of DOOR with Tie-Breaking by Timepoint – ITT Analysis Population

Timepoint	Variable	Level	N_d	N_s	DOOR Probability[a]	95% CI[b]
Day 42	Baseline Pathogen	MRSA	xx	xx	x.xx	x.xx, x.xx
		MSSA	xx	xx	x.xx	x.xx, x.xx
	PWID Status	PWID	xx	xx	x.xx	x.xx, x.xx
		Non-PWID	xx	xx	x.xx	x.xx, x.xx
	Infectious Disease Consultation	Yes	xx	xx	x.xx	x.xx, x.xx
		No	xx	xx	x.xx	x.xx, x.xx
	Underlying Site of Infection	Endovascular	xx	xx	x.xx	x.xx, x.xx
		Bone and Joint	xx	xx	x.xx	x.xx, x.xx
		Skin	xx	xx	x.xx	x.xx, x.xx
		Pulmonary	xx	xx	x.xx	x.xx, x.xx
	Immunosuppression	Yes	xx	xx	x.xx	x.xx, x.xx
		No	xx	xx	x.xx	x.xx, x.xx
	Duration of Initial Bacteremia (Days)	< 2	xx	xx	x.xx	x.xx, x.xx
		2-4	xx	xx	x.xx	x.xx, x.xx
		> 4	xx	xx	x.xx	x.xx, x.xx
Day 70	Baseline Pathogen	MRSA	xx	xx	x.xx	x.xx, x.xx
		MSSA	xx	xx	x.xx	x.xx, x.xx
	PWID Status	PWID	xx	xx	x.xx	x.xx, x.xx
		Non-PWID	xx	xx	x.xx	x.xx, x.xx
	Infectious Disease Consultation	Yes	xx	xx	x.xx	x.xx, x.xx
		No	xx	xx	x.xx	x.xx, x.xx
	Underlying Site of Infection	Endovascular	xx	xx	x.xx	x.xx, x.xx
		Bone and Joint	xx	xx	x.xx	x.xx, x.xx
		Skin	xx	xx	x.xx	x.xx, x.xx
		Pulmonary	xx	xx	x.xx	x.xx, x.xx
	Immunosuppression	Yes	xx	xx	x.xx	x.xx, x.xx
		No	xx	xx	x.xx	x.xx, x.xx
	Duration of Initial Bacteremia (Days)	< 2	xx	xx	x.xx	x.xx, x.xx
		2-4	xx	xx	x.xx	x.xx, x.xx
		> 4	xx	xx	x.xx	x.xx, x.xx

N_d = number of subjects in the ITT population who received dalbavancin within the subgroup category.
N_s = number of subjects in the ITT population who received standard of care within the subgroup category.
PWID = Persons Who Inject Drugs.
[a] Probability of Higher DOOR in Dalbavancin arm compared to standard of care + 0.5 Probability of Equal DOOR. This analysis uses IPW to handle missing values of DOOR and change in QoL as a tie breaker.
[b] Probability of Higher DOOR in Dalbavancin arm + 0.5 Probability of Equal DOOR. 95% CI obtained using bootstrap for IPW.

Table 26: Subgroup Analysis of DOOR with Tie-Breaking by Timepoint – mITT Analysis Population

This table will repeat Table 25 limited to the mITT population.

PRIVILEGED AND CONFIDENTIAL COMMUNICATION

Table 27: Subgroup Analysis of DOOR with Tie-Breaking by Timepoint – CE Analysis Population

Timepoint	Variable	Level	N_d	N_s	DOOR Probability[a]	Halperin 95% CI[b]
Day 42	Baseline Pathogen	MRSA	xx	xx	x.xx	x.xx, x.xx
		MSSA	xx	xx	x.xx	x.xx, x.xx
	PWID Status	PWID	xx	xx	x.xx	x.xx, x.xx
		Non-PWID	xx	xx	x.xx	x.xx, x.xx
	Infectious Disease Consultation	Yes	xx	xx	x.xx	x.xx, x.xx
		No	xx	xx	x.xx	x.xx, x.xx
	Underlying Site of Infection	Endovascular	xx	xx	x.xx	x.xx, x.xx
		Bone and Joint	xx	xx	x.xx	x.xx, x.xx
		Skin	xx	xx	x.xx	x.xx, x.xx
		Pulmonary	xx	xx	x.xx	x.xx, x.xx
	Immunosuppression	Yes	xx	xx	x.xx	x.xx, x.xx
		No	xx	xx	x.xx	x.xx, x.xx
	Duration of Initial Bacteremia (Days)	< 2	xx	xx	x.xx	x.xx, x.xx
		2-4	xx	xx	x.xx	x.xx, x.xx
		> 4	xx	xx	x.xx	x.xx, x.xx
Day 70	Baseline Pathogen	MRSA	xx	xx	x.xx	x.xx, x.xx
		MSSA	xx	xx	x.xx	x.xx, x.xx
	PWID Status	PWID	xx	xx	x.xx	x.xx, x.xx
		Non-PWID	xx	xx	x.xx	x.xx, x.xx
	Infectious Disease Consultation	Yes	xx	xx	x.xx	x.xx, x.xx
		No	xx	xx	x.xx	x.xx, x.xx
	Underlying Site of Infection	Endovascular	xx	xx	x.xx	x.xx, x.xx
		Bone and Joint	xx	xx	x.xx	x.xx, x.xx
		Skin	xx	xx	x.xx	x.xx, x.xx
		Pulmonary	xx	xx	x.xx	x.xx, x.xx
	Immunosuppression	Yes	xx	xx	x.xx	x.xx, x.xx
		No	xx	xx	x.xx	x.xx, x.xx
	Duration of Initial Bacteremia (Days)	< 2	xx	xx	x.xx	x.xx, x.xx
		2-4	xx	xx	x.xx	x.xx, x.xx
		> 4	xx	xx	x.xx	x.xx, x.xx

N_d = number of subjects in the CE population who received dalbavancin within the subgroup category.
N_s = number of subjects in the CE population who received standard of care within the subgroup category.
PWID = Persons Who Inject Drugs.
[a] Probability of Higher DOOR in Dalbavancin Arm + 0.5 Probability of Equal DOOR.
[b] 95% CI obtained using the method described in Halperin et. al [2].

Table 28: Analysis of DOOR Categories – ITT Analysis Population

[Implementation Note: This table will be included in the interim analysis.]

Timepoint	DOOR Category	Dalbavancin (N=X)		Standard of Care (N=X)	
		n	% (95% CI)[a]	n	% (95% CI)[a]
Day 42	Alive with no events	xx	xx (xx, xx)	xx	xx (xx, xx)
	Alive with 1 event	xx	xx (xx, xx)	xx	xx (xx, xx)
	Alive with 2 events	xx	xx (xx, xx)	xx	xx (xx, xx)
	Alive with 3 events	xx	xx (xx, xx)	xx	xx (xx, xx)
	Death	xx	xx (xx, xx)	xx	xx (xx, xx)
	Missing	xx	xx (xx, xx)	xx	xx (xx, xx)
Day 70	Alive with no events	xx	xx (xx, xx)	xx	xx (xx, xx)
	Alive with 1 event	xx	xx (xx, xx)	xx	xx (xx, xx)
	Alive with 2 events	xx	xx (xx, xx)	xx	xx (xx, xx)
	Alive with 3 events	xx	xx (xx, xx)	xx	xx (xx, xx)
	Death	xx	xx (xx, xx)	xx	xx (xx, xx)
	Missing	xx	xx (xx, xx)	xx	xx (xx, xx)

N = Number of subjects in the ITT Population in the given treatment group.
n = Number of subjects in the corresponding analysis population, treatment group, and DOOR category
[a] 95% CI estimated using the Wilson Method.
Note: Events that are both infectious complications and SAEs or AEs leading to study drug discontinuation count twice towards the DOOR.

Table 29: Analysis of DOOR Categories – mITT Analysis Population

This table will repeat Table 28 limited to the mITT population.

Table 30: Analysis of DOOR Categories – CE Analysis Population

This table will repeat Table 28 limited to the CE population.

[Implementation note: The missing category is removed from this analysis since the CE population has complete data.]

Table 31: Cumulative Proportions of DOOR - ITT Analysis Population

[Implementation Note: This table will be included in the interim analysis.]

Timepoint	DOOR Category	Dalbavancin				Standard of Care				Cumulative DOOR Probability
		N	n[a]	%[a]	95% CI	N	n[a]	%[a]	95% CI	% (95% CI)[b]
Day 42	Alive with no events	xx	xx	xx		xx	xx	xx		xx (x.x, x.x)
	Alive with less than 2 events	xx	xx	xx		xx	xx	xx		xx (x.x, x.x)
	Alive with less than 3 events	xx	xx	xx		xx	xx	xx		xx (x.x, x.x)
	Alive or Dead	xx	xx	xx		xx	xx	xx		xx (x.x, x.x)
Day 70	Alive with no events	xx	xx	xx		xx	xx	xx		xx (x.x, x.x)
	Alive with less than 2 events	xx	xx	xx		xx	xx	xx		xx (x.x, x.x)
	Alive with less than 3 events	xx	xx	xx		xx	xx	xx		xx (x.x, x.x)
	Alive or Dead	xx	xx	xx		xx	xx	xx		xx (x.x, x.x)

N = Number of subjects in the ITT Population in the given treatment group with non-missing cumulative DOOR category at the corresponding timepoint.
n = Number of subjects in the given cumulative DOOR category.
[a] n and % represent cumulative numbers.
[b] 95% CI for cumulative DOOR probability using the Wilcoxon-Mann-Whitney statistic corrected for ties as an estimate for the DOOR probability.
Note: Events that are both infectious complications and SAEs or AEs leading to study drug discontinuation count twice towards the DOOR.

Table 32: Cumulative Proportions of DOOR – mITT Analysis Population

This table will repeat Table 31 limited to the mITT population.

Table 33: Cumulative Proportions of DOOR – CE Analysis Population

This table will repeat Table 31 limited to the CE population.

Table 34: Summary of Expected Numbers Gained Loss at Day 42 and Day 70 by Analysis Population

Analysis Population	Timepoint	DOOR Category	Dalbavancin				Standard of Care				Gained Loss[a] (95% CI)
			N	n	%	Expected Number	N	n	%	Expected Number	
ITT	Day 42	Alive with no events	xx	xx	xx	xxx	xx	xx	xx	xxx	xx(xx, xx)
		Alive with 1 event	xx	xx	xx	xxx	xx	xx	xx	xxx	xx
		Alive with 2 events	xx	xx	xx	xxx	xx	xx	xx	xxx	xx
		Alive with 3 events	xx	xx	xx	xxx	xx	xx	xx	xxx	xx
		Death	xx	xx	xx	xxx	xx	xx	xx	xxx	xx
	Day 70	Alive with no events	xx	xx	xx	xxx	xx	xx	xx	xxx	xx
		Alive with 1 event	xx	xx	xx	xxx	xx	xx	xx	xxx	xx
		Alive with 2 events	xx	xx	xx	xxx	xx	xx	xx	xxx	xx
		Alive with 3 events	xx	xx	xx	xxx	xx	xx	xx	xxx	xx
		Death	xx	xx	xx	xxx	xx	xx	xx	xxx	xx
[Repeat for mITT and CE analysis populations]											

N = Number of subjects in the corresponding analysis population and treatment group with non-missing DOOR category at the corresponding timepoint.
n = Number of subjects in the corresponding analysis population, treatment group, and DOOR category.
[a] The gained loss is calculated as the difference in expected numbers, dalbavancin relative to standard of care.
Note: Events that are both infectious complications and SAEs or AEs leading to study drug discontinuation count twice towards the DOOR.

Table 35: Analysis of Clinical Efficacy at Day 70 Using ITT and mITT Analysis Populations

Analysis Population	Model	Statistic	Dalbavancin (N=X)	Standard of Care (N=X)
ITT	IPW	Subjects with non-missing clinical efficacy on Day 70 – n (%)	x (x)	x (x)
		Subjects with missing clinical efficacy on Day 70– n (%)	x (x)	x (x)
		Percent rate of clinical efficacy at Day 70 (95% CI)[a]	xx (xx, xx)	xx (xx, xx)
		Difference in rates (percent) of clinical efficacy, dalbavancin relative to standard of care (95% CI)[a]	xx (xx, xx)	-
		Conclusion of non-inferiority of dalbavancin to standard of care[b]	Yes/No	-
		Conclusion of superiority of dalbavancin to standard of care[c]	Yes/No	-
	Multiple Imputation	Subjects with non-missing clinical efficacy on Day 70 – n (%)	x (x)	x (x)
		Subjects with missing clinical efficacy on Day 70– n (%)	x (x)	x (x)
		Percent rate of clinical efficacy at Day 70 (95% CI)[a]	xx (xx, xx)	xx (xx, xx)
		Difference in rates (percent) of clinical efficacy, dalbavancin relative to standard of care (95% CI)[a]	xx (xx, xx)	-
		Conclusion of non-inferiority of dalbavancin to standard of care[b]	Yes/No	-
		Conclusion of superiority of dalbavancin to standard of care[c]	Yes/No	-
mITT	IPW	Subjects with non-missing clinical efficacy on Day 70 – n (%)	x (x)	x (x)
		Subjects with missing clinical efficacy on Day 70– n (%)	x (x)	x (x)
		Percent rate of clinical efficacy at Day 70 (95% CI)[a]	xx (xx, xx)	xx (xx, xx)
		Difference in rates (percent) of clinical efficacy, dalbavancin relative to standard of care (95% CI)[a]	xx (xx, xx)	-
		Conclusion of non-inferiority of dalbavancin to standard of care[b]	Yes/No	-
		Conclusion of superiority of dalbavancin to standard of care[c]	Yes/No	-
	Multiple Imputation	Subjects with non-missing clinical efficacy on Day 70 – n (%)	x (x)	x (x)
		Subjects with missing clinical efficacy on Day 70– n (%)	x (x)	x (x)
		Percent rate of clinical efficacy at Day 70 (95% CI)[a]	xx (xx, xx)	xx (xx, xx)
		Difference in rates (percent) of clinical efficacy, dalbavancin relative to standard of care (95% CI)[a]	xx (xx, xx)	-
		Conclusion of non-inferiority of dalbavancin to standard of care[b]	Yes/No	-
		Conclusion of superiority of dalbavancin to standard of care[c]	Yes/No	-

N = Number of subjects in the corresponding analysis Population. Multiple imputation was used to impute missing values.
[a] 95% CIs were obtained from linear regression model following multiple imputation adjusting for study day of Day 70.
[b] Non-inferiority of dalbavancin was concluded if the lower bound of the 95% CI for the difference in proportions is greater than -20%.
[c] Superiority of dalbavancin was concluded if the 95% CI for the difference in proportions does not contain 0.

Table 36: Analysis of Clinical Efficacy at Day 42 Using ITT and mITT Analysis Populations

This table will repeat Table 35 using Day 42.

Table 37: Analysis of Clinical Efficacy at Day 42 and Day 70 Using CE Analysis Population

[Implementation note: If the lower bound of 95% CI for the difference in rates of clinical efficacy is greater than -20%, annotate the interval with footnote b that states ‘Lower bound of confidence interval greater than -20% (non-inferiority margin).’]

		Rate of Clinical Efficacy			Difference in Rates		
**Timepoint**	**Treatment Group**	**n**	**%**	**95% CI[a]**	**%**	**95% CI[a]**	**Miettinen–Nurminen 95% CI**
Day 42	Dalbavancin (N=X)	x	xx	xx, xx	xx	xx, xx	xx, xx
	Standard of Care (N=X)	x	xx	xx, xx	Reference	-	-
Day 70	Dalbavancin (N=X)	x	xx	xx, xx	xx	xx, xx	xx, xx
	Standard of Care (N=X)	x	xx	xx, xx	Reference	-	-

N = Number of subjects in the CE analysis population at the corresponding timepoint.
n = Number of subjects in CE population with clinical efficacy at the corresponding timepoint.
[a] 95% CI obtained from linear regression.

Table 38: Subgroup Analysis of Clinical Efficacy by Timepoint – ITT Analysis Population

			Dalbavancin			Standard of Care			
**Timepoint**	**Variable**	**Level**	**N**	**n**	**Proportion % (95% CI)[a]**	**N**	**n**	**Proportion % (95% CI)[a]**	**Difference in Proportion % (95% CI)[a]**
Day 42	Baseline Pathogen	MRSA	xx	xx	xx (xx, xx)	xx	xx	xx (xx, xx)	xx (xx, xx)
		MSSA	xx	xx	xx (xx, xx)	xx	xx	xx (xx, xx)	xx (xx, xx)
	PWID Status	PWID	xx	xx	xx (xx, xx)	xx	xx	xx (xx, xx)	xx (xx, xx)
		Non-PWID	xx	xx	xx (xx, xx)	xx	xx	xx (xx, xx)	xx (xx, xx)
	Infectious Disease Consultation	Yes	xx	xx	xx (xx, xx)	xx	xx	xx (xx, xx)	xx (xx, xx)
		No	xx	xx	xx (xx, xx)	xx	xx	xx (xx, xx)	xx (xx, xx)
	Underlying Site of Infection	Endovascular	xx	xx	xx (xx, xx)	xx	xx	xx (xx, xx)	xx (xx, xx)
		Bone and Joint	xx	xx	xx (xx, xx)	xx	xx	xx (xx, xx)	xx (xx, xx)
		Skin	xx	xx	xx (xx, xx)	xx	xx	xx (xx, xx)	xx (xx, xx)
		Pulmonary	xx	xx	xx (xx, xx)	xx	xx	xx (xx, xx)	xx (xx, xx)
	Immunosuppression	Yes	xx	xx	xx (xx, xx)	xx	xx	xx (xx, xx)	xx (xx, xx)
		No	xx	xx	xx (xx, xx)	xx	xx	xx (xx, xx)	xx (xx, xx)
	Duration of Initial Bacteremia (Days)	< 2	xx	xx	xx (xx, xx)	xx	xx	xx (xx, xx)	xx (xx, xx)
		2-4	xx	xx	xx (xx, xx)	xx	xx	xx (xx, xx)	xx (xx, xx)
		>4	xx	xx	xx (xx, xx)	xx	xx	xx (xx, xx)	xx (xx, xx)
Day 70	Baseline Pathogen	MRSA	xx	xx	xx (xx, xx)	xx	xx	xx (xx, xx)	xx (xx, xx)
		MSSA	xx	xx	xx (xx, xx)	xx	xx	xx (xx, xx)	xx (xx, xx)
	PWID Status	PWID	xx	xx	xx (xx, xx)	xx	xx	xx (xx, xx)	xx (xx, xx)
		Non-PWID	xx	xx	xx (xx, xx)	xx	xx	xx (xx, xx)	xx (xx, xx)
	Infectious Disease Consultation	Yes	xx	xx	xx (xx, xx)	xx	xx	xx (xx, xx)	xx (xx, xx)
		No	xx	xx	xx (xx, xx)	xx	xx	xx (xx, xx)	xx (xx, xx)
	Underlying Site of Infection	Endovascular	xx	xx	xx (xx, xx)	xx	xx	xx (xx, xx)	xx (xx, xx)
		Bone and Joint	xx	xx	xx (xx, xx)	xx	xx	xx (xx, xx)	xx (xx, xx)
		Skin	xx	xx	xx (xx, xx)	xx	xx	xx (xx, xx)	xx (xx, xx)
		Pulmonary	xx	xx	xx (xx, xx)	xx	xx	xx (xx, xx)	xx (xx, xx)
	Immunosuppression	Yes	xx	xx	xx (xx, xx)	xx	xx	xx (xx, xx)	xx (xx, xx)
		No	xx	xx	xx (xx, xx)	xx	xx	xx (xx, xx)	xx (xx, xx)
	Duration of Initial Bacteremia (Days)	< 2	xx	xx	xx (xx, xx)	xx	xx	xx (xx, xx)	xx (xx, xx)
		2-4	xx	xx	xx (xx, xx)	xx	xx	xx (xx, xx)	xx (xx, xx)
		>4	xx	xx	xx (xx, xx)	xx	xx	xx (xx, xx)	xx (xx, xx)

N = Number of subjects in the ITT analysis population, treatment group and subgroup category
n = Number of subjects in the corresponding analysis population and treatment group who achieved clinical efficacy.
PWID = Persons Who Inject Drugs.
[a]95% CIs were obtained from linear regression model with IPW.
Note: IPW was used to handle missing values of clinical efficacy.

Table 39: Subgroup Analysis of Clinical Efficacy by Timepoint – mITT Analysis Population

This table will repeat Table 38 limited to the mITT population.

Table 40: Subgroup Analysis of Clinical Efficacy by Timepoint – CE Analysis Population

Timepoint	Variable	Level	Dalbavancin N	Dalbavancin n	Dalbavancin Proportion % (95% CI)[a]	Standard of Care N	Standard of Care n	Standard of Care Proportion % (95% CI)[a]	Difference in Proportion % (95% CI)[a]
Day 42	Baseline Pathogen	MRSA	xx	xx	xx (xx, xx)	xx	xx	xx (xx, xx)	xx (xx, xx)
		MSSA	xx	xx	xx (xx, xx)	xx	xx	xx (xx, xx)	xx (xx, xx)
	PWID Status	PWID	xx	xx	xx (xx, xx)	xx	xx	xx (xx, xx)	xx (xx, xx)
		Non-PWID	xx	xx	xx (xx, xx)	xx	xx	xx (xx, xx)	xx (xx, xx)
	Infectious Disease Consultation	Yes	xx	xx	xx (xx, xx)	xx	xx	xx (xx, xx)	xx (xx, xx)
		No	xx	xx	xx (xx, xx)	xx	xx	xx (xx, xx)	xx (xx, xx)
	Underlying Site of Infection	Endovascular	xx	xx	xx (xx, xx)	xx	xx	xx (xx, xx)	xx (xx, xx)
		Bone and Joint	xx	xx	xx (xx, xx)	xx	xx	xx (xx, xx)	xx (xx, xx)
		Skin	xx	xx	xx (xx, xx)	xx	xx	xx (xx, xx)	xx (xx, xx)
		Pulmonary	xx	xx	xx (xx, xx)	xx	xx	xx (xx, xx)	xx (xx, xx)
	Immunosuppression	Yes	xx	xx	xx (xx, xx)	xx	xx	xx (xx, xx)	xx (xx, xx)
		No	xx	xx	xx (xx, xx)	xx	xx	xx (xx, xx)	xx (xx, xx)
	Duration of Initial Bacteremia (Days)	< 2	xx	xx	xx (xx, xx)	xx	xx	xx (xx, xx)	xx (xx, xx)
		2-4	xx	xx	xx (xx, xx)	xx	xx	xx (xx, xx)	xx (xx, xx)
		>4	xx	xx	xx (xx, xx)	xx	xx	xx (xx, xx)	xx (xx, xx)
Day 70	Baseline Pathogen	MRSA	xx	xx	xx (xx, xx)	xx	xx	xx (xx, xx)	xx (xx, xx)
		MSSA	xx	xx	xx (xx, xx)	xx	xx	xx (xx, xx)	xx (xx, xx)
	PWID Status	PWID	xx	xx	xx (xx, xx)	xx	xx	xx (xx, xx)	xx (xx, xx)
		Non-PWID	xx	xx	xx (xx, xx)	xx	xx	xx (xx, xx)	xx (xx, xx)
	Infectious Disease Consultation	Yes	xx	xx	xx (xx, xx)	xx	xx	xx (xx, xx)	xx (xx, xx)
		No	xx	xx	xx (xx, xx)	xx	xx	xx (xx, xx)	xx (xx, xx)
	Underlying Site of Infection	Endovascular	xx	xx	xx (xx, xx)	xx	xx	xx (xx, xx)	xx (xx, xx)
		Bone and Joint	xx	xx	xx (xx, xx)	xx	xx	xx (xx, xx)	xx (xx, xx)
		Skin	xx	xx	xx (xx, xx)	xx	xx	xx (xx, xx)	xx (xx, xx)
		Pulmonary	xx	xx	xx (xx, xx)	xx	xx	xx (xx, xx)	xx (xx, xx)
	Immunosuppression	Yes	xx	xx	xx (xx, xx)	xx	xx	xx (xx, xx)	xx (xx, xx)
		No	xx	xx	xx (xx, xx)	xx	xx	xx (xx, xx)	xx (xx, xx)
	Duration of Initial Bacteremia (Days)	< 2	xx	xx	xx (xx, xx)	xx	xx	xx (xx, xx)	xx (xx, xx)
		2-4	xx	xx	xx (xx, xx)	xx	xx	xx (xx, xx)	xx (xx, xx)
		>4	xx	xx	xx (xx, xx)	xx	xx	xx (xx, xx)	xx (xx, xx)

N = Number of subjects in the CE analysis population, treatment group and subgroup category.
PWID = Persons Who Inject Drugs.
[a] 95% CIs were obtained from linear regression model.

Table 41: Summary of Clinical DOOR Components at Day 70 – ITT Analysis Population

DOOR Component	Dalbavancin				Standard of Care			
	N	n	%	95% CI[a]	N	n	%	95% CI[a]
Clinical Failure	x	x	x	(x.x, x.x)	x	x	x	(x.x, x.x)
Infectious Complication	x	x	x	(x.x, x.x)	x	x	x	(x.x, x.x)
SAEs	x	x	x	(x.x, x.x)	x	x	x	(x.x, x.x)
AEs Leading to Study Drug Discontinuation	x	x	x	(x.x, x.x)	x	x	x	(x.x, x.x)
All-cause Mortality	x	x	x	(x.x, x.x)	x	x	x	(x.x, x.x)

N = Number of subjects in the ITT population with non-missing values for the corresponding DOOR component.
n = Number of subjects with who experienced the corresponding DOOR component.
[a] 95% CI for DOOR Probability.

Table 42: Summary of Clinical DOOR Components at Day 70 – mITT Analysis Population

This table will repeat Table 43 limited to the mITT population.

Table 43: Summary of Clinical DOOR Components at Day 70 – CE Analysis Population

This table will repeat Table 43 limited to the CE population.

Table 44: Summary of Clinical DOOR Components at Day 42 – ITT Analysis Population

This table will repeat Table 43 limited to the ITT population at Day 42.

Table 45: Summary of Clinical DOOR Components at Day 42 – mITT Analysis Population

This table will repeat Table 43 limited to the mITT population at Day 42.

Table 46: Summary of Clinical DOOR Components at Day 42 – CE Analysis Population

This table will repeat Table 43 limited to the CE population at Day 42.

Table 47: Summary of DOOR by Component at Day 70 – ITT Analysis Population

DOOR	Clinical Failure	Infectious Complication	SAEs or AEs Leading to Study Drug Discontinuation	Dalbavancin (N=X)		Standard of Care (N=X)	
				n	%	N	%
1 - Alive with No Events	-	-	-				
2 - Alive with One Event	Yes	-	-				
	-	Yes	-				
	-	-	Yes				
3 - Alive with Two Events	Yes	Yes	-				
	Yes	-	Yes				
	-	Yes	Yes				
4 - Alive with Three Events	Yes	Yes	Yes				
5 - Death	-	-	-				
	Yes	-	-				
	-	Yes					
	-	-	Yes				
	Yes	Yes	-				
	Yes	-	Yes				
	-	Yes	Yes				
	Yes	Yes	Yes				
Note: Events that are both infectious complications and SAEs or AEs leading to study drug discontinuation count twice towards the DOOR.							

Table 48: Summary of DOOR by Component at Day 42 – ITT Analysis Population

This table will repeat Table 47 at Day 42.

Table 49: Analysis of Clinical Failure at Day 70 Using Weighted GEE and Weighted GLMM Models- ITT Analysis Population

	Dalbavancin		Standard of Care			
**Model**	**%**	**95% CI**	**%**	**95% CI**	**Difference (95% CI)**	**P-Value**
Weighted GEE	x.xxx	(x.xxx, x.xxx)	x.xx	(x.xx, x.xx)	(x.xxx, x.xxx)	x.xxx
Weighted GLMM	x.xxx	(x.xxx, x.xxx)	x.xx	(x.xx, x.xx)	(x.xxx, x.xxx)	x.xxx

Table 50: Analysis of Microbiologic Success Using ITT or mITT Analysis Populations

Timepoint	Analysis Population	Model	Statistic	Dalbavancin (N=X)	Standard of Care (N=X)
Day 42	ITT	IPW	Subjects with non-missing microbiologic success on Day 42– n (%)	x (x)	x (x)
			Subjects with missing microbiologic success on Day 42 – n (%)	x (x)	x (x)
			Percent rate of microbiologic success at 42 (95% CI)[a]	xx (xx, xx)	xx (xx, xx)
			Difference in rates (percent) of microbiologic success, dalbavancin relative to standard of care (95% CI)[a]	xx (xx, xx)	-
		Multiple Imputation	Subjects with non-missing microbiologic success on Day 42– n (%)	x (x)	x (x)
			Subjects with missing microbiologic success on Day 42 – n (%)	x (x)	x (x)
			Percent rate of microbiologic success at 42 (95% CI)[a]	xx (xx, xx)	xx (xx, xx)
			Difference in rates (percent) of microbiologic success, dalbavancin relative to standard of care (95% CI)[a]	xx (xx, xx)	-
	mITT	IPW	Subjects with non-missing microbiologic success on Day 42– n (%)	x (x)	x (x)
			Subjects with missing microbiologic success on Day 42 – n (%)	x (x)	x (x)
			Percent rate of microbiologic success at 42 (95% CI)[a]	xx (xx, xx)	xx (xx, xx)
			Difference in rates (percent) of microbiologic success, dalbavancin relative to standard of care (95% CI)[a]	xx (xx, xx)	-
		Multiple Imputation	Subjects with non-missing microbiologic success on Day 42– n (%)	x (x)	x (x)
			Subjects with missing microbiologic success on Day 42 – n (%)	x (x)	x (x)
			Percent rate of microbiologic success at 42 (95% CI)[a]	xx (xx, xx)	xx (xx, xx)
			Difference in rates (percent) of microbiologic success, dalbavancin relative to standard of care (95% CI)[a]	xx (xx, xx)	-
Day 70	ITT	IPW	Subjects with non-missing microbiologic success on Day 70– n (%)	x (x)	x (x)
			Subjects with missing microbiologic success on Day 70 – n (%)	x (x)	x (x)
			Percent rate of microbiologic success at Day 70 (95% CI)[a]	xx (xx, xx)	xx (xx, xx)
			Difference in rates (percent) of microbiologic success, dalbavancin relative to standard of care (95% CI)[a]	xx (xx, xx)	-
		Multiple Imputation	Subjects with non-missing microbiologic success on Day 70– n (%)	x (x)	x (x)
			Subjects with missing microbiologic success on Day 70 – n (%)	x (x)	x (x)
			Percent rate of microbiologic success at Day 70 (95% CI)[a]	xx (xx, xx)	xx (xx, xx)
			Difference in rates (percent) of microbiologic success, dalbavancin relative to standard of care (95% CI)[a]	xx (xx, xx)	-

Table 50: Analysis of Microbiologic Success Using ITT or mITT Analysis Populations (continued)

	mITT	IPW	Subjects with non-missing microbiologic success on Day 70– n (%)	x (x)	x (x)
			Subjects with missing microbiologic success on Day 70 – n (%)	x (x)	x (x)
			Percent rate of microbiologic success at 70 (95% CI)[a]	xx (xx, xx)	xx (xx, xx)
			Difference in rates (percent) of microbiologic success, dalbavancin relative to standard of care (95% CI)[a]	xx (xx, xx)	-
		Multiple Imputation	Subjects with non-missing microbiologic success on Day 70– n (%)	x (x)	x (x)
			Subjects with missing microbiologic success on Day 70 – n (%)	x (x)	x (x)
			Percent rate of microbiologic success at Day 70 (95% CI)[a]	xx (xx, xx)	xx (xx, xx)
			Difference in rates (percent) of microbiologic success, dalbavancin relative to standard of care (95% CI)[a]	xx (xx, xx)	-

N = Number of subjects in the corresponding analysis Population. Multiple imputation and IPW were used to handle missing values of microbiological success.
[a] 95% CIs were obtained from linear regression model following multiple imputation or IPW.

Table 51: Analysis of Microbiologic Success at Day 42 and Day 70 Using CE Analysis Population

Timepoint	Treatment Group	Proportion of Microbiologic Success			Difference in Proportion		
		n	%	95% CI[a]	%	95% CI[a]	Miettinen–Nurminen 95% CI
Day 42	Dalbavancin (N=X)	x	xx	xx, xx	xx	xx, xx	xx, xx
	Standard of Care (N=X)	x	xx	xx, xx	Reference	-	-
Day 70	Dalbavancin (N=X)	x	xx	xx, xx	xx	xx, xx	xx, xx
	Standard of Care (N=X)	x	xx	xx, xx	Reference	-	-

N = Number of subjects in the CE analysis population at the corresponding timepoint.
n = Number of subjects in CE population with microbiologic success at the corresponding timepoint.
[a] 95% CI obtained from linear regression.

Table 52: Subgroup Analysis of Microbiologic Success by Timepoint – ITT Analysis Population

Timepoint	Variable	Level	Dalbavancin N	Dalbavancin n	Dalbavancin Proportion % (95% CI)[a]	Standard of Care N	Standard of Care n	Standard of Care Proportion % (95% CI)[a]	Difference in Proportion % (95% CI)[a]
Day 42	Baseline Pathogen	MRSA	xx	xx	xx (xx, xx)	xx	xx	xx (xx, xx)	xx (xx, xx)
		MSSA	xx	xx	xx (xx, xx)	xx	xx	xx (xx, xx)	xx (xx, xx)
	PWID Status	PWID	xx	xx	xx (xx, xx)	xx	xx	xx (xx, xx)	xx (xx, xx)
		Non-PWID	xx	xx	xx (xx, xx)	xx	xx	xx (xx, xx)	xx (xx, xx)
	Infectious Disease Consultation	Yes	xx	xx	xx (xx, xx)	xx	xx	xx (xx, xx)	xx (xx, xx)
		No	xx	xx	xx (xx, xx)	xx	xx	xx (xx, xx)	xx (xx, xx)
	Underlying Site of Infection	Endovascular	xx	xx	xx (xx, xx)	xx	xx	xx (xx, xx)	xx (xx, xx)
		Bone and Joint	xx	xx	xx (xx, xx)	xx	xx	xx (xx, xx)	xx (xx, xx)
		Skin	xx	xx	xx (xx, xx)	xx	xx	xx (xx, xx)	xx (xx, xx)
		Pulmonary	xx	xx	xx (xx, xx)	xx	xx	xx (xx, xx)	xx (xx, xx)
	Immunosuppression	Yes	xx	xx	xx (xx, xx)	xx	xx	xx (xx, xx)	xx (xx, xx)
		No	xx	xx	xx (xx, xx)	xx	xx	xx (xx, xx)	xx (xx, xx)
	Duration of Initial Bacteremia (Days)	< 2	xx	xx	xx (xx, xx)	xx	xx	xx (xx, xx)	xx (xx, xx)
		2-4	xx	xx	xx (xx, xx)	xx	xx	xx (xx, xx)	xx (xx, xx)
		>4	xx	xx	xx (xx, xx)	xx	xx	xx (xx, xx)	xx (xx, xx)
Day 70	Baseline Pathogen	MRSA	xx	xx	xx (xx, xx)	xx	xx	xx (xx, xx)	xx (xx, xx)
		MSSA	xx	xx	xx (xx, xx)	xx	xx	xx (xx, xx)	xx (xx, xx)
	PWID Status	PWID	xx	xx	xx (xx, xx)	xx	xx	xx (xx, xx)	xx (xx, xx)
		Non-PWID	xx	xx	xx (xx, xx)	xx	xx	xx (xx, xx)	xx (xx, xx)
	Infectious Disease Consultation	Yes	xx	xx	xx (xx, xx)	xx	xx	xx (xx, xx)	xx (xx, xx)
		No	xx	xx	xx (xx, xx)	xx	xx	xx (xx, xx)	xx (xx, xx)
	Underlying Site of Infection	Endovascular	xx	xx	xx (xx, xx)	xx	xx	xx (xx, xx)	xx (xx, xx)
		Bone and Joint	xx	xx	xx (xx, xx)	xx	xx	xx (xx, xx)	xx (xx, xx)
		Skin	xx	xx	xx (xx, xx)	xx	xx	xx (xx, xx)	xx (xx, xx)
		Pulmonary	xx	xx	xx (xx, xx)	xx	xx	xx (xx, xx)	xx (xx, xx)
	Immunosuppression	Yes	xx	xx	xx (xx, xx)	xx	xx	xx (xx, xx)	xx (xx, xx)
		No	xx	xx	xx (xx, xx)	xx	xx	xx (xx, xx)	xx (xx, xx)
	Duration of Initial Bacteremia (Days)	< 2	xx	xx	xx (xx, xx)	xx	xx	xx (xx, xx)	xx (xx, xx)
		2-4	xx	xx	xx (xx, xx)	xx	xx	xx (xx, xx)	xx (xx, xx)
		>4	xx	xx	xx (xx, xx)	xx	xx	xx (xx, xx)	xx (xx, xx)

N = Number of subjects in the ITT analysis population, treatment group and subgroup category. IPW was used to handle missing values of microbiological success.
PWID = Persons Who Inject Drugs.
[a] 95% CIs were obtained from linear regression model with IPW.

Table 53: Subgroup Analysis of Microbiologic Success by Timepoint – mITT Analysis Population

This table will repeat Table 52 limited to the mITT population.

Table 54: Subgroup Analysis of Microbiologic Success by Timepoint – CE Analysis Population

Timepoint	Variable	Level	Dalbavancin			Standard of Care			Difference in Proportions % (95% CI)[a]
			N	n	Proportion % (95% CI)[a]	N	n	Proportion % (95% CI)[a]	
Day 42	Baseline Pathogen	MRSA	xx	xx	xx (xx, xx)	xx	xx	xx (xx, xx)	xx (xx, xx)
		MSSA	xx	xx	xx (xx, xx)	xx	xx	xx (xx, xx)	xx (xx, xx)
	PWID Status	PWID	xx	xx	xx (xx, xx)	xx	xx	xx (xx, xx)	xx (xx, xx)
		Non-PWID	xx	xx	xx (xx, xx)	xx	xx	xx (xx, xx)	xx (xx, xx)
	Infectious Disease Consultation	Yes	xx	xx	xx (xx, xx)	xx	xx	xx (xx, xx)	xx (xx, xx)
		No	xx	xx	xx (xx, xx)	xx	xx	xx (xx, xx)	xx (xx, xx)
	Underlying Site of Infection	Endovascular	xx	xx	xx (xx, xx)	xx	xx	xx (xx, xx)	xx (xx, xx)
		Bone and Joint	xx	xx	xx (xx, xx)	xx	xx	xx (xx, xx)	xx (xx, xx)
		Skin	xx	xx	xx (xx, xx)	xx	xx	xx (xx, xx)	xx (xx, xx)
		Pulmonary	xx	xx	xx (xx, xx)	xx	xx	xx (xx, xx)	xx (xx, xx)
	Immunosuppression	Yes	xx	xx	xx (xx, xx)	xx	xx	xx (xx, xx)	xx (xx, xx)
		No	xx	xx	xx (xx, xx)	xx	xx	xx (xx, xx)	xx (xx, xx)
	Duration of Initial Bacteremia (Days)	< 2	xx	xx	xx (xx, xx)	xx	xx	xx (xx, xx)	xx (xx, xx)
		2-4	xx	xx	xx (xx, xx)	xx	xx	xx (xx, xx)	xx (xx, xx)
		>4	xx	xx	xx (xx, xx)	xx	xx	xx (xx, xx)	xx (xx, xx)
Day 70	Baseline Pathogen	MRSA	xx	xx	xx (xx, xx)	xx	xx	xx (xx, xx)	xx (xx, xx)
		MSSA	xx	xx	xx (xx, xx)	xx	xx	xx (xx, xx)	xx (xx, xx)
	PWID Status	PWID	xx	xx	xx (xx, xx)	xx	xx	xx (xx, xx)	xx (xx, xx)
		Non-PWID	xx	xx	xx (xx, xx)	xx	xx	xx (xx, xx)	xx (xx, xx)
	Infectious Disease Consultation	Yes	xx	xx	xx (xx, xx)	xx	xx	xx (xx, xx)	xx (xx, xx)
		No	xx	xx	xx (xx, xx)	xx	xx	xx (xx, xx)	xx (xx, xx)
	Underlying Site of Infection	Endovascular	xx	xx	xx (xx, xx)	xx	xx	xx (xx, xx)	xx (xx, xx)
		Bone and Joint	xx	xx	xx (xx, xx)	xx	xx	xx (xx, xx)	xx (xx, xx)
		Skin	xx	xx	xx (xx, xx)	xx	xx	xx (xx, xx)	xx (xx, xx)
		Pulmonary	xx	xx	xx (xx, xx)	xx	xx	xx (xx, xx)	xx (xx, xx)
	Immunosuppression	Yes	xx	xx	xx (xx, xx)	xx	xx	xx (xx, xx)	xx (xx, xx)
		No	xx	xx	xx (xx, xx)	xx	xx	xx (xx, xx)	xx (xx, xx)
	Duration of Initial Bacteremia (Days)	< 2	xx	xx	xx (xx, xx)	xx	xx	xx (xx, xx)	xx (xx, xx)
		2-4	xx	xx	xx (xx, xx)	xx	xx	xx (xx, xx)	xx (xx, xx)
		>4	xx	xx	xx (xx, xx)	xx	xx	xx (xx, xx)	xx (xx, xx)

N = Number of subjects in the CE analysis population, treatment group and subgroup category.
PWID = Persons Who Inject Drugs.
[a] 95% CIs were obtained from linear regression model without multiple imputation.

Table 55: Distribution of DOOR at Day 42 and Day 70 Using ITT, mITT, and CE Analysis Populations

Analysis Population	Timepoint	DOOR Category	Dalbavancin (N=X)		Standard of Care (N=X)		All Subjects (N=X)		P-value[a]
			n	%	n	%	n	%	
ITT	Day 42	Any DOOR Category	x	xx	x	xx	**x**	**100**	**--**
		1 - Alive with no events	x	xx	x	xx	x	xx	x.xxx
		2 - Alive with 1 event							
		3 - Alive with 2 events							
		4 - Alive with 3 events							
		5 – Death							
	Day 70	Any DOOR Category							
		1 - Alive with no events							
		2 - Alive with 1 event							
		3 - Alive with 2 events							
		4 - Alive with 3 events							
		5 – Death							
Repeat for mITT and CE populations									

N = Number of subjects in the ITT analysis population.
n = Number of subjects in the given ITT population with non-missing values of DOOR.
[a] P-value calculated using Wilcoxon rank-sum Test.
Note: Events that are both infectious complications and SAEs or AEs leading to study drug discontinuation count twice towards the DOOR.

Table 56: Distribution of DOOR at Day 42 by Subgroup Categories – ITT Analysis Population

Subgroup	Subgroup Category	DOOR Category	Dalbavancin (N=X)		Standard of Care (N=X)		All Subjects (N=X)		P-value[a]
			n	%	n	%	n	%	
Baseline Pathogen	MRSA	Any DOOR Category	x	xx	x	xx	**x**	**100**	**--**
		1 - Alive with no events	x	xx	x	xx	x	xx	x.xxx
		2 - Alive with 1 event							
		3 - Alive with 2 events							
		4 - Alive with 3 events							
		5 – Death							
	MSSA	Any DOOR Category							
		1 - Alive with no events							
		2 - Alive with 1 event							
		3 - Alive with 2 events							
		4 - Alive with 3 events							
		5 – Death							
PWID Status	PWID	Repeat for all DOOR as above							
	Non-PWID								
Infectious Disease Consultation	Yes								
	No								
Underlying Site of Infection	Endovascular								
	Bone and Joint								
	Skin								
	Pulmonary								
Immunosuppressio n	Yes								
	No								
Duration of Initial Bacteremia (Days)	< 2								
	2-4								
	>4								

N = Number of subjects in the ITT analysis population.
n = Number of subjects in the given ITT population with non-missing values of DOOR.
[a] P-value calculated using Wilcoxon rank-sum Test.
Note: Events that are both infectious complications and SAEs or AEs leading to study drug discontinuation count twice towards the DOOR.

Table with similar format:

Table 57: Distribution of DOOR at Day 70 by Subgroup Categories – ITT Analysis Population

Table 58: Summary Statistics of QoL Scores from the ARLG Bloodstream Infection QoL Measure at Day 42 and Day 70 Using ITT, mITT, and CE Analysis Populations

			QoL Score					Change from Baseline				
**Analysis Population**	**Time Point**	**Treatment Group**	**n**	**Mean**	**Standard Deviation**	**Median**	**Min, Max**	**n**	**Mean**	**Standard Deviation**	**Median**	**Min, Max**
ITT	Baseline	Dalbavancin (N=X)	x	xx.x	xx.x	xx.x	xx.x, xx.x	NA	NA	NA	NA	NA
		Standard of Care (N=X)	x	xx.x	xx.x	xx.x	xx.x, xx.x	NA	NA	NA	NA	NA
	Day 42	Dalbavancin (N=X)	x	xx.x	xx.x	xx.x	xx.x, xx.x	x	xx.x	xx.x	xx.x	xx.x, xx.x
		Standard of Care (N=X)	x	xx.x	xx.x	xx.x	xx.x, xx.x	x	xx.x	xx.x	xx.x	xx.x, xx.x
	Day 70	Dalbavancin (N=X)	x	xx.x	xx.x	xx.x	xx.x, xx.x	x	xx.x	xx.x	xx.x	xx.x, xx.x
		Standard of Care (N=X)	x	xx.x	xx.x	xx.x	xx.x, xx.x	x	xx.x	xx.x	xx.x	xx.x, xx.x
mITT	Baseline	Dalbavancin (N=X)	x	xx.x	xx.x	xx.x	xx.x, xx.x	NA	NA	NA	NA	NA
		Standard of Care (N=X)	x	xx.x	xx.x	xx.x	xx.x, xx.x	NA	NA	NA	NA	NA
	Day 42	Dalbavancin (N=X)	x	xx.x	xx.x	xx.x	xx.x, xx.x	x	xx.x	xx.x	xx.x	xx.x, xx.x
		Standard of Care (N=X)	x	xx.x	xx.x	xx.x	xx.x, xx.x	x	xx.x	xx.x	xx.x	xx.x, xx.x
	Day 70	Dalbavancin (N=X)	x	xx.x	xx.x	xx.x	xx.x, xx.x	x	xx.x	xx.x	xx.x	xx.x, xx.x
		Standard of Care (N=X)	x	xx.x	xx.x	xx.x	xx.x, xx.x	x	xx.x	xx.x	xx.x	xx.x, xx.x
CE	Baseline	Dalbavancin (N=X)	x	xx.x	xx.x	xx.x	xx.x, xx.x	NA	NA	NA	NA	NA
		Standard of Care (N=X)	x	xx.x	xx.x	xx.x	xx.x, xx.x	NA	NA	NA	NA	NA
	Day 42	Dalbavancin (N=X)	x	xx.x	xx.x	xx.x	xx.x, xx.x	x	xx.x	xx.x	xx.x	xx.x, xx.x
		Standard of Care (N=X)	x	xx.x	xx.x	xx.x	xx.x, xx.x	x	xx.x	xx.x	xx.x	xx.x, xx.x
	Day 70	Dalbavancin (N=X)	x	xx.x	xx.x	xx.x	xx.x, xx.x	x	xx.x	xx.x	xx.x	xx.x, xx.x
		Standard of Care (N=X)	x	xx.x	xx.x	xx.x	xx.x, xx.x	x	xx.x	xx.x	xx.x	xx.x, xx.x

N = Number of subjects in the given analysis population.
n = Number of subjects in the given analysis population with non-missing QoL score values at the timepoint of interest. For the change from baseline, n represents the number of subjects in the given analysis population with non-missing values at baseline and at the timepoint being assessed.
Note: Baseline QoL standardized score is obtained from the questions arising from the PROMIS physical function item bank (PROMIS Item Bank v2.0, short form 6b) item bank on the ARLG Bloodstream Infection QoL.

Table 59: Summary Statistics of QoL Scores Using the EQ-5D-5L Instrument at Day 42 and Day 70 Using ITT, mITT, and CE Analysis Populations

This table will be similar to Table 58.

Table 60: Summary Statistics of QoL Scores Using the PROMIS Global Health Short Form at Day 42 and Day 70 Using ITT, mITT, and CE Analysis Populations

This table will be similar to Table 58.

Table 61: Predictive Interval Plots (PIPS) Statistics for the Probability of Higher DOOR in the Dalbavancin Group at Day 70 – ITT Analysis Population

[Implementation Note: this table will only be included in the interim analysis.]

Assumption	Current 95% CI	Width of Current 95% CI	Width of Predicted 95% CI Median [Q1, Q3]	Probability to Reject Null Hypothesis[a]
Observed Trend	(x.xx – x.xx)	x.xx	x.xx (x.xx – x.xx)	x.xx
Null Hypothesis	(x.xx – x.xx)	x.xx	x.xx (x.xx – x.xx)	x.xx
Alternative Hypothesis	(x.xx – x.xx)	x.xx	x.xx (x.xx – x.xx)	x.xx
Best case scenario for remaining outcome	(x.xx – x.xx)	x.xx	x.xx (x.xx – x.xx)	x.xx
Best case scenario for remaining outcome	(x.xx – x.xx)	x.xx	x.xx (x.xx – x.xx)	x.xx

Q1 = 25th percentile. Q3 = 75th percentile.
[a] Probability to Reject Null Hypothesis = proportion of PIs simulated that have a lower bound greater than 0.50.
Note: Statistics related to the PIPS are also presented in Figure 26.

Table 62: Predictive Interval Plots (PIPS) Statistics for the Rates of Clinical Efficacy at Day 70 – ITT Analysis Population

This table will be similar to Table 61.

Implementation Note: this table will only be included in the interim analysis.

Table 63: Number and Percentage of Subjects with Late Recurrence - Osteomyelitis Population

Analysis Group	Statistic	Dalbavancin (N=X)	Standard of Care (N=X)
Osteomyelitis Population	Number of subjects with late recurrence	x	x
	Percent rate of late recurrence (95% CI)[a]	xx (xx, xx)	xx (xx, xx)
	Difference in rates (percent) late recurrence, dalbavancin relative to standard of care (95% CI)[b]	xx (xx, xx)	-
Sensitivity Osteomyelitis Population	Number of subjects with late recurrence	x	x
	Percent rate of late recurrence (95% CI)[a]	xx (xx, xx)	xx (xx, xx)
	Difference in rates (percent) late recurrence, dalbavancin relative to standard of care (95% CI)[b]	xx (xx, xx)	-

N = Number of subjects in the diagnosed with osteomyelitis.
[a] 95% CI for proportions calculated using the Wilson method.
[b] 95% CI for the difference in proportions, dalbavancin relative to standard of care, calculated using the Miettinen–Nurminen method.
Note: The osteomyelitis population includes subjects in the ITT population diagnosed with osteomyelitis for whom consent and data were obtained for Visit 7. The sensitivity osteomyelitis population excludes subjects from the Osteomyelitis Population those subjects for whom consent was obtained retroactively. XX (of NN) and YY (of MM) subjects with osteomyelitis in the Dalbavancin and Standard of Care Arms, respectively, became eligible for Visit 7 data collection under protocol version 3.0 and re-consented to Visit 7 data collection.

Programming Note: In the Note above:

XX = subjects treated with Dalbavancin who became eligible for Visit 7 data collection under protocol version 3.0 **and** re-consented Visit 7 data collection.

NN = subjects treated with Dalbavancin who became eligible for Visit 7 data collection under protocol version 3.0.

YY = subjects treated with Standard of Care who became eligible for Visit 7 data collection under protocol version 3.0 **and** re-consented Visit 7 data collection.

MM = subjects treated with Standard of Care who became eligible for Visit 7 data collection under protocol version 3.0.

14.3 Safety Data

14.3.1 Displays of Adverse Events

Table 64: Number and Percentage of Subjects reporting SAEs, AEs Leading Discontinuation, Grade 3 or Higher AEs, AESIs, and ALT/AST Elevations by Treatment Group - Safety Population

[Implementation note: Sort this table by decreasing frequency for the treatment group.]

Adverse Event Type	Dalbavancin (N=X)			Standard of Care (N=X)			All Subjects (N=X)		
	n	%	Events	n	%	Events	n	%	Events
SAEs	x	x	x	x	x	x	x	x	x
AE Leading to Study Drug Discontinuation	x	x	x	x	x	x	x	x	x
Grade 3 or Higher AEs	x	x	x	x	x	x	x	x	x
AESIs	x	x	x	x	x	x	x	x	x
AST/ALT Elevation[a]	x	x	x	x	x	x	x	x	x

N = Number of subjects in the Safety Population.
[a] Occurrence of AST/ALT elevations >3x upper limit of normal (ULN) from first dose of study product through follow-up period.

Table 65: Number and Percentage of subjects reporting SAEs, AEs Leading Discontinuation, Grade 3 or Higher AEs, AESIs, and ALT/AST Elevations by Standard of Care Antibiotics – Standard of Care Arm, Safety Population

[Implementation note: If any other standard of care antibiotics not listed here are administered, extend the table to add more columns for new SOC antibiotics.]

	Cefazolin (N=X)			Nafcillin (N=X)			Oxacillin (N=X)			Vancomycin (N=X)			Daptomycin (N=X)		
**Adverse Event Type**	**n**	**%**	**Events**	**n**	**%**	**Events**	**n**	**%**	**Events**	**n**	**%**	**Events**	**n**	**%**	**Events**
SAEs	x	x	x	x	x	x	x	x	x	x	x	x	x	x	x
AE Leading to Study Drug Discontinuation	x	x	x	x	x	x	x	x	x	x	x	x	x	x	x
Grade 3 or Higher AEs	x	x	x	x	x	x	x	x	x	x	x	x	x	x	x
AESIs	x	x	x	x	x	x	x	x	x	x	x	x	x	x	x
AST/ALT Elevation[a]	x	x	x	x	x	x	x	x	x	x	x	x	x	x	x

N = Number of subjects in the Safety Population who received standard of care antibiotics.
[a] Occurrence of AST/ALT elevations >3x upper limit of normal (ULN) from first dose of study product through follow-up period.

14.3.1.2 Unsolicited Adverse Events

Table 66: Summary of Treatment-Emergent Adverse Events by MedDRA System Organ Class and Preferred Term, Relationship, and Treatment Group

MedDRA System Organ Class	Preferred Term	Dalbavancin (N=X)						Standard of Care (N=X)						All Subjects (N=X)					
		Related		Not Related		Total		Related		Not Related		Total		Related		Not Related		Total	
		n	%	n	%	n	%	n	%	n	%	n	%	n	%	n	%	n	%
Any SOC	Any PT	x	xx	x	xx	x	xx	x	xx	x	xx	x	xx	x	xx	x	xx	x	xx
		x	xx	x	xx	x	xx	x	xx	x	xx	x	xx	x	xx	x	xx	x	xx
		x	xx	x	xx	x	xx	x	xx	x	xx	x	xx	x	xx	x	xx	x	xx
		x	xx	x	xx	x	xx	x	xx	x	xx	x	xx	x	xx	x	xx	x	xx
		x	xx	x	xx	x	xx	x	xx	x	xx	x	xx	x	xx	x	xx	x	xx
		x	xx	x	xx	x	xx	x	xx	x	xx	x	xx	x	xx	x	xx	x	xx
		x	xx	x	xx	x	xx	x	xx	x	xx	x	xx	x	xx	x	xx	x	xx
SOC 1	PT 1	x	xx	x	xx	x	xx	x	xx	x	xx	x	xx	x	xx	x	xx	x	xx
		x	xx	x	xx	x	xx	x	xx	x	xx	x	xx	x	xx	x	xx	x	xx
		x	xx	x	xx	x	xx	x	xx	x	xx	x	xx	x	xx	x	xx	x	xx
		x	xx	x	xx	x	xx	x	xx	x	xx	x	xx	x	xx	x	xx	x	xx
		x	xx	x	xx	x	xx	x	xx	x	xx	x	xx	x	xx	x	xx	x	xx
		x	xx	x	xx	x	xx	x	xx	x	xx	x	xx	x	xx	x	xx	x	xx
		x	xx	x	xx	x	xx	x	xx	x	xx	x	xx	x	xx	x	xx	x	xx
	PT 2	x	xx	x	xx	x	xx	x	xx	x	xx	x	xx	x	xx	x	xx	x	xx
		x	xx	x	xx	x	xx	x	xx	x	xx	x	xx	x	xx	x	xx	x	xx
		x	xx	x	xx	x	xx	x	xx	x	xx	x	xx	x	xx	x	xx	x	xx
		x	xx	x	xx	x	xx	x	xx	x	xx	x	xx	x	xx	x	xx	x	xx
		x	xx	x	xx	x	xx	x	xx	x	xx	x	xx	x	xx	x	xx	x	xx
		x	xx	x	xx	x	xx	x	xx	x	xx	x	xx	x	xx	x	xx	x	xx
		x	xx	x	xx	x	xx	x	xx	x	xx	x	xx	x	xx	x	xx	x	xx
…	…																		

N = Number of subjects in the Safety Population.

Table 67: Summary of Treatment-Emergent Adverse Events by MedDRA System Organ Class and Preferred Term, Maximum Severity, Relationship, and Treatment Group

MedDRA System Organ Class	Preferred Term	Severity	Dalbavancin (N=X)						Standard of Care (N=X)						All Subjects (N=X)					
			Related		Not Related		Total		Related		Not Related		Total		Related		Not Related		Total	
			n	%	n	%	n	%	n	%	n	%	n	%	n	%	n	%	n	%
Any SOC	Any PT	Any Severity	x	xx	x	xx	x	xx	x	xx	x	xx	x	xx	x	xx	x	xx	x	xx
		Not Reported	x	xx	x	xx	x	xx	x	xx	x	xx	x	xx	x	xx	x	xx	x	xx
		Grade 1	x	xx	x	xx	x	xx	x	xx	x	xx	x	xx	x	xx	x	xx	x	xx
		Grade 2	x	xx	x	xx	x	xx	x	xx	x	xx	x	xx	x	xx	x	xx	x	xx
		Grade 3	x	xx	x	xx	x	xx	x	xx	x	xx	x	xx	x	xx	x	xx	x	xx
		Grade 4	x	xx	x	xx	x	xx	x	xx	x	xx	x	xx	x	xx	x	xx	x	xx
		Grade 5	x	xx	x	xx	x	xx	x	xx	x	xx	x	xx	x	xx	x	xx	x	xx
SOC 1	PT 1	Any Severity	x	xx	x	xx	x	xx	x	xx	x	xx	x	xx	x	xx	x	xx	x	xx
		Not Reported	x	xx	x	xx	x	xx	x	xx	x	xx	x	xx	x	xx	x	xx	x	xx
		Grade 1	x	xx	x	xx	x	xx	x	xx	x	xx	x	xx	x	xx	x	xx	x	xx
		Grade 2	x	xx	x	xx	x	xx	x	xx	x	xx	x	xx	x	xx	x	xx	x	xx
		Grade 3	x	xx	x	xx	x	xx	x	xx	x	xx	x	xx	x	xx	x	xx	x	xx
		Grade 4	x	xx	x	xx	x	xx	x	xx	x	xx	x	xx	x	xx	x	xx	x	xx
		Grade 5	x	xx	x	xx	x	xx	x	xx	x	xx	x	xx	x	xx	x	xx	x	xx
	PT 2	Any Severity	x	xx	x	xx	x	xx	x	xx	x	xx	x	xx	x	xx	x	xx	x	xx
		Not Reported	x	xx	x	xx	x	xx	x	xx	x	xx	x	xx	x	xx	x	xx	x	xx
		Grade 1	x	xx	x	xx	x	xx	x	xx	x	xx	x	xx	x	xx	x	xx	x	xx
		Grade 2	x	xx	x	xx	x	xx	x	xx	x	xx	x	xx	x	xx	x	xx	x	xx
		Grade 3	x	xx	x	xx	x	xx	x	xx	x	xx	x	xx	x	xx	x	xx	x	xx
		Grade 4	x	xx	x	xx	x	xx	x	xx	x	xx	x	xx	x	xx	x	xx	x	xx
		Grade 5	x	xx	x	xx	x	xx	x	xx	x	xx	x	xx	x	xx	x	xx	x	xx
…	…																			

N = Number of subjects in the Safety Population.

Table 68: Summary of Treatment-Emergent Adverse Events by MedDRA System Organ Class and High Level Group Term, Relationship, and Treatment Group

MedDRA System Organ Class	High Level Group Term	Dalbavancin (N=X)						Standard of Care (N=X)						All Subjects (N=X)					
		Related		Not Related		Total		Related		Not Related		Total		Related		Not Related		Total	
		n	%	n	%	n	%	n	%	n	%	n	%	n	%	n	%	n	%
Any SOC	Any HLGT	x	xx	x	xx	x	xx	x	xx	x	xx	x	xx	x	xx	x	xx	x	xx
	HLGT 1	x	xx	x	xx	x	xx	x	xx	x	xx	x	xx	x	xx	x	xx	x	xx
	HLGT 2	x	xx	x	xx	x	xx	x	xx	x	xx	x	xx	x	xx	x	xx	x	xx
	…																		
	Repeat for appliable HLGT																		
SOC 1	Any HLGT	x	xx	x	xx	x	xx	x	xx	x	xx	x	xx	x	xx	x	xx	x	xx
	HLGT 1	x	xx	x	xx	x	xx	x	xx	x	xx	x	xx	x	xx	x	xx	x	xx
	HLGT 2	x	xx	x	xx	x	xx	x	xx	x	xx	x	xx	x	xx	x	xx	x	xx
	…																		
	Repeat for appliable HLGT																		
SOC2	Any HLGT	x	xx	x	xx	x	xx	x	xx	x	xx	x	xx	x	xx	x	xx	x	xx
	HLGT 1	x	xx	x	xx	x	xx	x	xx	x	xx	x	xx	x	xx	x	xx	x	xx
	HLGT 2	x	xx	x	xx	x	xx	x	xx	x	xx	x	xx	x	xx	x	xx	x	xx
	…																		
	Repeat for appliable HLGT																		
….	….																		

N = Number of subjects in the Safety Population.

Table 69: Summary of Treatment-Emergent Adverse Events by MedDRA System Organ Class and High Level Group Term, Maximum Severity, Relationship, and Treatment Group

MedDRA System Organ Class	High Level Group Term	Severity	Dalbavancin (N=X)						Standard of Care (N=X)						All Subjects (N=X)					
			Related		Not Related		Total		Related		Not Related		Total		Related		Not Related		Total	
			n	%	n	%	n	%	n	%	n	%	n	%	n	%	n	%	n	%
Any SOC	Any HLGT	Any Severity	x	xx	x	xx	x	xx	x	xx	x	xx	x	xx	x	xx	x	xx	x	xx
		Not Reported	x	xx	x	xx	x	xx	x	xx	x	xx	x	xx	x	xx	x	xx	x	xx
		Grade 1	x	xx	x	xx	x	xx	x	xx	x	xx	x	xx	x	xx	x	xx	x	xx
		Grade 2	x	xx	x	xx	x	xx	x	xx	x	xx	x	xx	x	xx	x	xx	x	xx
		Grade 3	x	xx	x	xx	x	xx	x	xx	x	xx	x	xx	x	xx	x	xx	x	xx
		Grade 4	x	xx	x	xx	x	xx	x	xx	x	xx	x	xx	x	xx	x	xx	x	xx
		Grade 5	x	xx	x	xx	x	xx	x	xx	x	xx	x	xx	x	xx	x	xx	x	xx
SOC 1	HLGT 1	Any Severity	x	xx	x	xx	x	xx	x	xx	x	xx	x	xx	x	xx	x	xx	x	xx
		Not Reported	x	xx	x	xx	x	xx	x	xx	x	xx	x	xx	x	xx	x	xx	x	xx
		Grade 1	x	xx	x	xx	x	xx	x	xx	x	xx	x	xx	x	xx	x	xx	x	xx
		Grade 2	x	xx	x	xx	x	xx	x	xx	x	xx	x	xx	x	xx	x	xx	x	xx
		Grade 3	x	xx	x	xx	x	xx	x	xx	x	xx	x	xx	x	xx	x	xx	x	xx
		Grade 4	x	xx	x	xx	x	xx	x	xx	x	xx	x	xx	x	xx	x	xx	x	xx
		Grade 5	x	xx	x	xx	x	xx	x	xx	x	xx	x	xx	x	xx	x	xx	x	xx
	HLGT 2	Any Severity	x	xx	x	xx	x	xx	x	xx	x	xx	x	xx	x	xx	x	xx	x	xx
		Not Reported	x	xx	x	xx	x	xx	x	xx	x	xx	x	xx	x	xx	x	xx	x	xx
		Grade 1	x	xx	x	xx	x	xx	x	xx	x	xx	x	xx	x	xx	x	xx	x	xx
		Grade 2	x	xx	x	xx	x	xx	x	xx	x	xx	x	xx	x	xx	x	xx	x	xx
		Grade 3	x	xx	x	xx	x	xx	x	xx	x	xx	x	xx	x	xx	x	xx	x	xx
		Grade 4	x	xx	x	xx	x	xx	x	xx	x	xx	x	xx	x	xx	x	xx	x	xx
		Grade 5	x	xx	x	xx	x	xx	x	xx	x	xx	x	xx	x	xx	x	xx	x	xx
….	….																			

N = Number of subjects in the Safety Population.

PRIVILEGED AND CONFIDENTIAL COMMUNICATION

Table 70: Summary of Treatment-Emergent Adverse Events by MedDRA System Organ Class, Maximum Severity, Relationship, and Treatment Group

MedDRA System Organ Class	Severity	Dalbavancin (N=X)						Standard of Care (N=X)						All Subjects (N=X)					
		Related		Not Related		Total		Related		Not Related		Total		Related		Not Related		Total	
		n	%	n	%	n	%	n	%	n	%	n	%	n	%	n	%	n	%
Any SOC	Any Severity	x	xx	x	xx	x	xx	x	xx	x	xx	x	xx	x	xx	x	xx	x	xx
	Not Reported	x	xx	x	xx	x	xx	x	xx	x	xx	x	xx	x	xx	x	xx	x	xx
	Grade 1	x	xx	x	xx	x	xx	x	xx	x	xx	x	xx	x	xx	x	xx	x	xx
	Grade 2	x	xx	x	xx	x	xx	x	xx	x	xx	x	xx	x	xx	x	xx	x	xx
	Grade 3	x	xx	x	xx	x	xx	x	xx	x	xx	x	xx	x	xx	x	xx	x	xx
	Grade 4	x	xx	x	xx	x	xx	x	xx	x	xx	x	xx	x	xx	x	xx	x	xx
	Grade 5	x	xx	x	xx	x	xx	x	xx	x	xx	x	xx	x	xx	x	xx	x	xx
SOC 1	Any Severity	x	xx	x	xx	x	xx	x	xx	x	xx	x	xx	x	xx	x	xx	x	xx
	Not Reported	x	xx	x	xx	x	xx	x	xx	x	xx	x	xx	x	xx	x	xx	x	xx
	Grade 1	x	xx	x	xx	x	xx	x	xx	x	xx	x	xx	x	xx	x	xx	x	xx
	Grade 2	x	xx	x	xx	x	xx	x	xx	x	xx	x	xx	x	xx	x	xx	x	xx
	Grade 3	x	xx	x	xx	x	xx	x	xx	x	xx	x	xx	x	xx	x	xx	x	xx
	Grade 4	x	xx	x	xx	x	xx	x	xx	x	xx	x	xx	x	xx	x	xx	x	xx
	Grade 5	x	xx	x	xx	x	xx	x	xx	x	xx	x	xx	x	xx	x	xx	x	xx
SOC 2	Any Severity	x	xx	x	xx	x	xx	x	xx	x	xx	x	xx	x	xx	x	xx	x	xx
	Not Reported	x	xx	x	xx	x	xx	x	xx	x	xx	x	xx	x	xx	x	xx	x	xx
	Grade 1	x	xx	x	xx	x	xx	x	xx	x	xx	x	xx	x	xx	x	xx	x	xx
	Grade 2	x	xx	x	xx	x	xx	x	xx	x	xx	x	xx	x	xx	x	xx	x	xx
	Grade 3	x	xx	x	xx	x	xx	x	xx	x	xx	x	xx	x	xx	x	xx	x	xx
	Grade 4	x	xx	x	xx	x	xx	x	xx	x	xx	x	xx	x	xx	x	xx	x	xx
	Grade 5	x	xx	x	xx	x	xx	x	xx	x	xx	x	xx	x	xx	x	xx	x	xx
….																			

N = Number of subjects in the Safety Population.

Table 71: Summary of AESIs by MedDRA System Organ Class, Maximum Severity, Relationship, and Treatment Group

This table will be similar to Table 70.

PRIVILEGED AND CONFIDENTIAL COMMUNICATION

Table 72: Summary of Treatment-Emergent AEs, On-therapy SAEs, AESIs, and AEs Leading Discontinuation Occurring in 2% of Subjects in Any Treatment Group by MedDRA Preferred Term, and Treatment Group – Safety Population

[Implementation note: Sort this table by decreasing frequency for the treatment group.]

Preferred Term	Dalbavancin (N=X)			Standard of Care (N=X)			All Subjects (N=X)		
	n	%	Events	n	%	Events	n	%	Events
Any PT	x	x	x	x	x	x	x	x	x
PT1	x	x	x	x	x	x	x	x	x
PT2	x	x	x	x	x	x	x	x	x
Etc									

N = Number of subjects in the Safety Population.

Table 73: Summary of Fatal on-Therapy SAEs by MedDRA Preferred Term, and Treatment Group – Safety Population

Preferred Term	Dalbavancin (N=X)			Standard of Care (N=X)			All Subjects (N=X)		
	n	%	Events	n	%	Events	n	%	Events
Any PT	x	x	x	x	x	x	x	x	x
PT1	x	x	x	x	x	x	x	x	x
PT2	x	x	x	x	x	x	x	x	x
…									

N = Number of subjects in the Safety Population.

Table 74: Summary of Fatal on-Therapy SAEs by MedDRA High Level Group Term, and Treatment Group – Safety Population

	Dalbavancin (N=X)			Standard of Care (N=X)			All Subjects (N=X)		
**High Level Group Term**	**n**	**%**	**Events**	**n**	**%**	**Events**	**n**	**%**	**Events**
Any HLGT	x	x	x	x	x	x	x	x	x
HLGT1	x	x	x	x	x	x	x	x	x
HLTG2	x	x	x	x	x	x	x	x	x
…									
N = Number of subjects in the Safety Population.									

14.3.2 Listing of Deaths, Other Serious and Significant Adverse Events

Table 75: Listing of Serious Adverse Events

Adverse Event	Study Day of AE Onset	Duration (Days)	Severity	Relationship to Study Treatment	If Not Related, Alternative Etiology	Action Taken with Study Treatment	Subject Discontinued Due to AE	Outcome	MedDRA System Organ Class	MedDRA High Level Group Term	MedDRA Preferred Term
**Treatment Group: , Subject ID: , AE Number:**											
Comments:											
**Treatment Group: , Subject ID: , AE Number:**											
Comments:											
Note: For additional details about SAEs, see Table: xx.											

Table 76: Listing of Adverse Events of Special Interest

This table will be similar to Table 75.

Table 77: Listing of Adverse Events Leading to Discontinuation

This table will be similar to Table 75.

Table 78: Listing of Subjects whose Outcome was Fatal During the Study

This table will be similar to Table 75.

Table 79: Listing of Treatment-Emergent Adverse Events

Adverse Event	Associated with Dose No.	No. of Days Post Associated Dose (Duration)	Severity	Relationship to Study Treatment	If Not Related, Alternative Etiology	Action Taken with Study Treatment	Subject Discontinued Due to AE	Outcome	MedDRA System Organ Class	MedDRA Preferred Term	MedDRA High Level Group Term
**Subject ID: , Treatment Group: , AE Number:**											
Comments:											
**Subject ID: , Treatment Group: , AE Number:**											
Comments:											

14.3.3 Narratives of Deaths, Other Serious and Significant Adverse Events

(Not included in SAP, but this is a placeholder for the CSR.)

PRIVILEGED AND CONFIDENTIAL COMMUNICATION

14.3.4 Abnormal Laboratory Value Listings (by Subject)

Table 80: Listing of Potentially Clinically Significant Laboratory Results – Chemistry

Treatment Group	Subject ID	Sex	Age (years)	Planned Time Point	Actual Study Day	Laboratory Parameter (Units)	Result (Severity)

Notes: All laboratory results for a parameter are displayed for subjects with at least one PSC result for that parameter.
PCS Criteria is defined as follows for each parameter: Sodium (<130 mmol/L OR >150 mmol/L), Potassium (<3.0 mmol/L OR >5.5 mmol/L), Glucose (<55 mg/dL), Creatinine (Serum creatinine >1.5x baseline), Albumin (<3 g/dL), Alkaline phosphatase (>ULN - 2.5 x ULN if baseline was normal; 2.0 - 2.5 x baseline if baseline was abnormal), Total bilirubin (>ULN – 1.5 x ULN if baseline was normal; > 1.0 – 1.5 x baseline if baseline was abnormal), AST (>ULN - 3.0 x ULN if baseline was normal; 1.5 - 3.0 x baseline if baseline was abnormal).

Programming Notes: sort by treatment group (dalbavancin first), then subject ID.

[Implementation Note: **If a subject has at least one PCS, list all their laboratory results for that laboratory parameter.** The criteria for PCS will be defined below. Laboratory parameters not included in this table will be ignored.

- Laboratory parameter (Potentially Clinically Significant Result Criteria)
- Sodium (<130 mmol/L OR >150 mmol/L)
- Potassium (<3.0 mmol/L OR >5.5 mmol/L)
- Glucose (<55 mg/dL)
- Creatinine (Serum creatinine >1.5x baseline)
- Albumin (<3 g/dL)
- Alkaline phosphatase (>ULN - 2.5 x ULN if baseline was normal; 2.0 - 2.5 x baseline if baseline was abnormal)
- Total bilirubin (>ULN – 1.5 x ULN if baseline was normal; > 1.0 – 1.5 x baseline if baseline was abnormal)
- AST (>ULN - 3.0 x ULN if baseline was normal; 1.5 - 3.0 x baseline if baseline was abnormal)
- ALT (>ULN – 3.0 x ULN if baseline was normal; 1.5 – 3.0 x baseline if baseline was abnormal)]

Table 81: Listing of Potentially Clinically Significant Laboratory Results – Hematology

If a subject has at least one PCS, list all their laboratory results for that laboratory parameter.]

Treatment Group	Subject ID	Sex	Age (years)	Planned Time Point	Actual Study Day	Laboratory Parameter (Units)	Result (Severity)

Notes: All laboratory results for a parameter are displayed for subjects with at least one PSC result for that parameter.
PCS Criteria is defined as follows for each parameter: Hemoglobin (<10 g/dL), Platelets (<75 /µL), White blood cell count (<3.0 x 10e9), Absolute neutrophil count (ANC) (<1500 x 10e9), Absolute lymphocyte count (ALC) (<800 x 10e9).

Programming Note: sort by treatment group (dalbavancin first), then subject ID.

[Implementation Note: **If a subject has at least one PCS, list all their laboratory results for that laboratory parameter.** The criteria for PCS will be defined as follows:

- Laboratory parameter (Potentially Clinically Significant Result Criteria)
- Hemoglobin (<10 g/dL)
- Platelets (<75 /µL)
- White blood cell count (<3.0 x 10e9)
- Absolute neutrophil count (ANC) (<1500 x 10e9)
- Absolute lymphocyte count (ALC) (<800 x 10e9)

14.3.5 Displays of Laboratory Results

14.3.5.1 Chemistry Results

Table 82: Number and Percentage of Subjects with Potentially Clinically Significant Post-Baseline Laboratory Values by Parameter and Treatment Group – Chemistry Parameters

Serum Chemistry Parameter	Dalbavancin			Standard of Care		
	N	n	%	N	n	%
Any Serum Chemistry Parameter	x	x	xx	x	x	xx
Sodium	x	x	xx	x	x	xx
Potassium	x	x	xx	x	x	xx
Calcium	x	x	xx	x	x	xx
Chloride	x	x	xx	x	x	xx
Bicarbonate	x	x	xx	x	x	xx
Glucose	x	x	xx	x	x	xx
Blood Urea Nitrogen	x	x	xx	x	x	xx
Creatinine	x	x	xx	x	x	xx
Total Protein	x	x	xx	x	x	xx
Alkaline Phosphatase	x	x	xx	x	x	xx
Albumin	x	x	xx	x	x	xx
Total bilirubin	x	x	xx	x	x	xx
AST	x	x	xx	x	x	xx
ALT	x	x	xx	x	x	xx

PCS = Potentially Clinically Significant.
N = Number of subjects in the Safety Population with available non-PCS baseline values and at least 1 post-baseline assessment.
n = Number of subjects with at least one PCS post-baseline laboratory value.

Table 83: Laboratory Summary Statistics by Parameter, Time Point, and Treatment Group – Serum Chemistry, Sodium

[Implementation Note: The number of decimals for the minimum and maximum will be the same as the original values, while the number of decimals for the mean, standard deviation, and median will add an extra decimal point to that of the original values.]

		Laboratory Value					Change from Baseline				
Time Point	Treatment Group	n	Mean	Standard Deviation	Median	Min, Max	n	Mean	Standard Deviation	Median	Min, Max
Baseline	Dalbavancin (N=X)	x	xx.x	xx.x	xx.x	xx.x, xx.x	NA	NA	NA	NA	NA
	Standard of Care (N=X)	x	xx.x	xx.x	xx.x	xx.x, xx.x	NA	NA	NA	NA	NA
Day 8	Dalbavancin (N=X)	x	xx.x	xx.x	xx.x	xx.x, xx.x	x	xx.x	xx.x	xx.x	xx.x, xx.x
	Standard of Care (N=X)	x	xx.x	xx.x	xx.x	xx.x, xx.x	x	xx.x	xx.x	xx.x	xx.x, xx.x
Day 22	Dalbavancin (N=X)	x	xx.x	xx.x	xx.x	xx.x, xx.x	x	xx.x	xx.x	xx.x	xx.x, xx.x
	Standard of Care (N=X)	x	xx.x	xx.x	xx.x	xx.x, xx.x	x	xx.x	xx.x	xx.x	xx.x, xx.x
Day 42	Dalbavancin (N=X)	x	xx.x	xx.x	xx.x	xx.x, xx.x	x	xx.x	xx.x	xx.x	xx.x, xx.x
	Standard of Care (N=X)	x	xx.x	xx.x	xx.x	xx.x, xx.x	x	xx.x	xx.x	xx.x	xx.x, xx.x

N = Number of subjects in the Safety Population.
n = Number of subjects in the Safety Population with non-missing laboratory values at the timepoint of interest. For the change from baseline, n represents the number of subjects in the Safety population with non-missing values at baseline and at the timepoint being assessed.

Tables with similar format to Table 83:

Table 84: Laboratory Summary Statistics by Parameter, Time Point, and Treatment Group – Serum Chemistry, Potassium

Table 85: Laboratory Summary Statistics by Parameter, Time Point, and Treatment Group – Serum Chemistry, Calcium

Table 86: Laboratory Summary Statistics by Parameter, Time Point, and Treatment Group – Serum Chemistry, Chloride

Table 87: Laboratory Summary Statistics by Parameter, Time Point, and Treatment Group – Serum Chemistry, Bicarbonate

Table 88: Laboratory Summary Statistics by Parameter, Time Point, and Treatment Group – Serum Chemistry, Glucose

Table 89: Laboratory Summary Statistics by Parameter, Time Point, and Treatment Group – Serum Chemistry, Blood Urea Nitrogen

Tables with similar format to Table 83 *(continued)*

Table 90: Laboratory Summary Statistics by Parameter, Time Point, and Treatment Group – Serum Chemistry, Creatinine

Table 91: Laboratory Summary Statistics by Parameter, Time Point, and Treatment Group – Serum Chemistry, Total Protein

Table 92: Laboratory Summary Statistics by Parameter, Time Point, and Treatment Group – Serum Chemistry, Alkaline Phosphatase

Table 93: Laboratory Summary Statistics by Parameter, Time Point, and Treatment Group – Serum Chemistry, Albumin

Table 94: Laboratory Summary Statistics by Parameter, Time Point, and Treatment Group – Serum Chemistry, Total Bilirubin

Table 95: Laboratory Summary Statistics by Parameter, Time Point, and Treatment Group – Serum Chemistry, AST

Table 96: Laboratory Summary Statistics by Parameter, Time Point, and Treatment Group – Serum Chemistry, ALT

14.3.5.2 Hematology Results

Table 97: Number and Percentage of Subjects with Potentially Clinically Significant Post-Baseline Laboratory Values by Parameter and Treatment Group – Hematology Parameters

Serum Chemistry Parameter	Dalbavancin			Standard of Care		
	N	n	%	N	n	%
Any Hematology Parameter	x	x	xx	x	x	xx
Absolute WBC	x	x	xx	x	x	xx
Erythrocyte	x	x	xx	x	x	xx
Hemoglobin	x	x	xx	x	x	xx
Hematocrit	x	x	xx	x	x	xx
Platelet Count	x	x	xx	x	x	xx
Mean Corpuscular Volume	x	x	xx	x	x	xx
Mean Corpuscular Hemoglobin	x	x	xx	x	x	xx
Mean Corpuscular Hemoglobin Concentration	x	x	xx	x	x	xx

PCS = Potentially Clinically Significant.
N = Number of subjects in the Safety Population with available non-PCS baseline values and at least 1 post-baseline assessment.
n = Number of subjects with at least one PCS post-baseline laboratory value.

Table 98: Laboratory Summary Statistics by Parameter, Time Point, and Treatment Group – Hematology, Absolute WBC

[Implementation Note: The number of decimals for the minimum and maximum will be the same as the original values, while the number of decimals for the mean, standard deviation, and median will add an extra decimal point to that of the original values.]

		Laboratory Value					Change from Baseline				
**Time Point**	**Treatment Group**	**n**	**Mean**	**Standard Deviation**	**Median**	**Min, Max**	**n**	**Mean**	**Standard Deviation**	**Median**	**Min, Max**
Baseline	Dalbavancin (N=X)	x	xx.x	xx.x	xx.x	xx.x, xx.x	NA	NA	NA	NA	NA
	Standard of Care (N=X)	x	xx.x	xx.x	xx.x	xx.x, xx.x	NA	NA	NA	NA	NA
Day 8	Dalbavancin (N=X)	x	xx.x	xx.x	xx.x	xx.x, xx.x	x	xx.x	xx.x	xx.x	xx.x, xx.x
	Standard of Care (N=X)	x	xx.x	xx.x	xx.x	xx.x, xx.x	x	xx.x	xx.x	xx.x	xx.x, xx.x
Day 22	Dalbavancin (N=X)	x	xx.x	xx.x	xx.x	xx.x, xx.x	x	xx.x	xx.x	xx.x	xx.x, xx.x
	Standard of Care (N=X)	x	xx.x	xx.x	xx.x	xx.x, xx.x	x	xx.x	xx.x	xx.x	xx.x, xx.x
Day 42	Dalbavancin (N=X)	x	xx.x	xx.x	xx.x	xx.x, xx.x	x	xx.x	xx.x	xx.x	xx.x, xx.x
	Standard of Care (N=X)	x	xx.x	xx.x	xx.x	xx.x, xx.x	x	xx.x	xx.x	xx.x	xx.x, xx.x

N = Number of subjects in the Safety Population.
n = Number of subjects in the Safety Population with non-missing laboratory values at the timepoint of interest. For the change from baseline, n represents the number of subjects in the Safety population with non-missing values at baseline and at the timepoint being assessed.

Tables with similar format to Table 98:

Table 99: Laboratory Summary Statistics by Parameter, Time Point, and Treatment Group – Hematology, Erythrocyte

Table 100: Laboratory Summary Statistics by Parameter, Time Point, and Treatment Group – Hematology, Hemoglobin

Table 101: Laboratory Summary Statistics by Parameter, Time Point, and Treatment Group – Hematology, Hematocrit

Table 102: Laboratory Summary Statistics by Parameter, Time Point, and Treatment Group – Hematology, Platelet Count

Table 103: Laboratory Summary Statistics by Parameter, Time Point, and Treatment Group – Hematology, Mean Corpuscular Volume

Table 104: Laboratory Summary Statistics by Parameter, Time Point, and Treatment Group – Hematology, Mean Corpuscular Hemoglobin

Table 105: Laboratory Summary Statistics by Parameter, Time Point, and Treatment Group – Hematology, Mean Corpuscular Hemoglobin Concentration

14.3.6 Displays of Vital Signs

Table 106: Summary of Vital Signs Values by Vital Sign Parameter, Visit, and Treatment Group

[Implementation Note: The number of decimals for the minimum and maximum will be the same as the original values, while the number of decimals for the mean, standard deviation, and median will add an extra decimal point to that of the original values.]

Vital Sign	Time Point	Dalbavancin (N=X)					Standard of Care (N=X)				
		n	Mean	Standard Deviation	Median	Min, Max	n	Mean	Standard Deviation	Median	Min, Max
Temperature (°F)	Visit 1 (Screening)	x	xx.xx	xx.xx	xx.xx	xx.x, xx.x	x	xx.xx	xx.xx	xx.xx	xx.x, xx.x
	Visit 2 (Baseline)										
	Visit 3 (Day 8)										
	Visit 4 (Day 22)										
	Visit 5 (Day 42)										
	Visit 6 (Day 70)										
	Visit 7 (Day 180)										
	Early Termination Visit										
Systolic Blood Pressure (mmHg)	Visit 1 (Screening)	x	xx.x	xx.x	xx.	xx, xx	x	xx.x	xx.x	xx.x	xx, xx
	Visit 2 (Baseline)										
	Visit 3 (Day 8)										
	Visit 4 (Day 22)										
	Visit 5 (Day 42)										
	Visit 6 (Day 70)										
	Visit 7 (Day 180)										
	Early Termination Visit										

Table 106: Summary of Vital Signs Values by Vital Sign Parameter, Visit, and Treatment Group (continued)

Vital Sign	Time Point	Dalbavancin (N=X)					Standard of Care (N=X)				
		n	Mean	Standard Deviation	Median	Min, Max	n	Mean	Standard Deviation	Median	Min, Max
Diastolic Blood Pressure (mmHg)	Visit 1 (Screening)	x	xx.x	xx.x	xx.x	xx, xx	x	xx.x	xx.x	xx.x	xx, xx
	Visit 2 (Baseline)										
	Visit 3 (Day 8)										
	Visit 4 (Day 22)										
	Visit 5 (Day 42)										
	Visit 6 (Day 70)										
	Visit 7 (Day 180)										
	Early Termination Visit										
Respiratory Rate (breaths/min)	Visit 1 (Screening)	x	xx.x	xx.x	xx.x	xx, xx	x	xx.x	xx.x	xx.x	xx, xx
	Visit 2 (Baseline)										
	Visit 3 (Day 8)										
	Visit 4 (Day 22)										
	Visit 5 (Day 42)										
	Visit 6 (Day 70)										
	Visit 7 (Day 180)										
	Early Termination Visit										
Pulse (beats/min)	Visit 1 (Screening)	x	xx.x	xx.x	xx.x	xx, xx	x	xx.x	xx.x	xx.x	xx, xx
	Visit 2 (Baseline)										
	Visit 3 (Day 8)										
	Visit 4 (Day 22)										
	Visit 5 (Day 42)										
	Visit 6 (Day 70)										
	Visit 7 (Day 180)										
	Early Termination Visit										

N = Number of subjects in the Safety Population.
n = Number of subjects in the Safety Population with non-missing values for the corresponding vital sign at the given timepoint.

Table 107: Summary of Change from Baseline Vital Signs Values by Vital Sign Parameter, Visit, and Treatment Group

[Implementation Note: The number of decimals for the minimum and maximum will be the same as the original values, while the number of decimals for the mean, standard deviation, and median will add an extra decimal point to that of the original values.]

		Dalbavancin (N=X)					Standard of Care (N=X)				
**Vital Sign**	**Time Point**	**n**	**Mean**	**Standard Deviation**	**Median**	**Min, Max**	**n**	**Mean**	**Standard Deviation**	**Median**	**Min, Max**
Temperature (°F)	Visit 3 (Day 8)	x	xx.xx	xx.xx	xx.xx	xx.x, xx.x	x	xx.xx	xx.xx	xx.xx	xx.x, xx.x
	Visit 4 (Day 22)										
	Visit 5 (Day 42)										
	Visit 6 (Day 70)										
	Visit 7 (Day 180)										
	Early Termination Visit										
Systolic Blood Pressure (mmHg)	Visit 3 (Day 8)	x	xx.x	xx.x	xx.	xx, xx	x	xx.x	xx.x	xx.x	xx, xx
	Visit 4 (Day 22)										
	Visit 5 (Day 42)										
	Visit 6 (Day 70)										
	Visit 7 (Day 180)										
	Early Termination Visit										
Diastolic Blood Pressure (mmHg)	Visit 3 (Day 8)	x	xx.x	xx.x	xx.x	xx, xx	x	xx.x	xx.x	xx.x	xx, xx
	Visit 4 (Day 22)										
	Visit 5 (Day 42)										
	Visit 6 (Day 70)										
	Visit 7 (Day 180)										
	Early Termination Visit										

Table 107: Summary of Change from Baseline Vital Signs Values by Vital Sign Parameter, Visit, and Treatment Group (continued)

		Dalbavancin (N=X)					Standard of Care (N=X)				
**Vital Sign**	**Time Point**	**n**	**Mean**	**Standard Deviation**	**Median**	**Min, Max**	**n**	**Mean**	**Standard Deviation**	**Median**	**Min, Max**
Respiratory Rate (breaths/min)	Visit 3 (Day 8)										
	Visit 4 (Day 22)										
	Visit 5 (Day 42)										
	Visit 6 (Day 70)										
	Visit 7 (Day 180)										
	Early Termination Visit										
Pulse (beats/min)	Visit 3 (Day 8)										
	Visit 4 (Day 22)										
	Visit 5 (Day 42)										
	Visit 6 (Day 70)										
	Visit 7 (Day 180)										
	Early Termination Visit										

N = Number of subjects in the Safety Population.
n = Number of subjects in the Safety Population with non-missing values for the corresponding vital sign at both baseline and given timepoint.

14.4 Summary of Concomitant Medications and Nondrug Interventions

Table 108: Number and Percentage of Subjects with Prior and Concurrent Medications by WHO Drug Classification and Treatment Group

WHO Drug Code Level 1, Anatomic Group	WHO Drug Code Level 2, Therapeutic Subgroup	Dalbavancin (N=X)		Standard of Care (N=X)		All Subjects (N=X)	
		n	%	n	%	n	%
Any Level 1 Codes	Any Level 2 Codes	x	xx	x	xx	x	xx
[ATC Level 1 - 1]	Any [ATC 1 – 1]						
	[ATC 2 - 1]						
	[ATC 2 - 2]						
	[ATC 2 - 3]						
[ATC Level 1 – 2]	[ATC 2 - 1]						
	[ATC 2 - 2]						
	[ATC 2 - 3]						

N = Number of subjects in the Safety Population.
n = Number of subjects reporting taking at least one medication in the specific WHO Drug Class.

Table 109: Number and Percentage of Subjects with Nondrug Interventions by MedDRA System Organ Class and Treatment Group – ITT Population

MedDRA System Organ Class	Dalbavancin (N=X)		Standard of Care (N=X)		All Subjects (N=X)	
	n	%	n	%	n	%
Any SOC	x	xx	x	xx	x	xx
[SOC 1]						
[SOC 2]						

N = Number of subjects in the ITT population.
n = Number of subjects reporting a nondrug intervention within the specified SOC. A subject is only counted once per SOC.

APPENDIX 2. FIGURE MOCK-UPS

LIST OF FIGURES

Figure 1: CONSORT Flow Diagram 139
Figure 2: Forest Plot of DOOR Probability, Dalbavancin Relative to Standard of Care, of Clinical DOOR Components at Day 70 – ITT Population....140
Figure 3: Forest Plot of DOOR Probability, Dalbavancin Relative to Standard of Care, of Clinical DOOR Components at Day 70 – mITT Population....140
Figure 4: Forest Plot of DOOR Probability, Dalbavancin Relative to Standard of Care, of Clinical DOOR Components at Day 70 – CE Population....140
Figure 5: Forest Plot of DOOR Probability, Dalbavancin Relative to Standard of Care, of Clinical DOOR Components at Day 42 – ITT Population....140
Figure 6: Forest Plot of DOOR Probability, Dalbavancin Relative to Standard of Care, of Clinical DOOR Components at Day 42 – mITT Population....140
Figure 7: Forest Plot of DOOR Probability, Dalbavancin Relative to Standard of Care, of Clinical DOOR Components at Day 42 – CE Population....140
Figure 8: Distribution of DOOR at Day 70 – ITT Population 141
Figure 9: Distribution of DOOR at Day 70 – mITT Population 141
Figure 10: Distribution of DOOR at Day 70 – CE Population 141
Figure 11: Distribution of DOOR at Day 42 – ITT Population 141
Figure 12: Distribution of DOOR at Day 42 – mITT Population 141
Figure 13: Distribution of DOOR at Day 42 – CE Population 141
Figure 14: Distribution of DOOR at Day 70 by Baseline Pathogen – ITT Population....141
Figure 15: Distribution of DOOR at Day 70 by PWID Status – ITT Population 142
Figure 16: Distribution of DOOR at Day 70 by Infectious Disease Consultation – ITT Population....142
Figure 17: Distribution of DOOR at Day 70 by Underlying Site of Infection – ITT Population....142
Figure 18: Distribution of DOOR at Day 70 by Immunosuppression – ITT Population....142
Figure 19: Distribution of DOOR at Day 70 by Duration of Initial Bacteremia – ITT Population....142
Figure 20: Forest Plot of DOOR Probabilities at Day 70 – ITT Population....143
Figure 21: Forest Plot of DOOR Probabilities at Day 42 – ITT Population....143
Figure 22: Difference in Cumulative Proportions of DOOR Along with 95% CI....144

PRIVILEGED AND CONFIDENTIAL COMMUNICATION

Figure 23: Difference in Means of Partial Credit Score by Timepoint and Analysis Population 145

Figure 24: Bivariate Analysis of DOOR Probability vs Difference in Mean of Change in QoL Score from Baseline at Day 70 – ITT Analysis Population 146

Figure 25: Bivariate Analysis of DOOR Probability vs Difference in Mean of Change in QoL Score from Baseline at Day 42 – ITT Analysis Population 146

Figure 26: Predictive Interval Plots for the Probability of Higher DOOR in the Dalbavancin Group at Day 70 – ITT Analysis Population....147

Figure 27: Predictive Interval Plots Statistics for the Rates of Clinical Efficacy at Day 70 – ITT Analysis Population* 147

Figure 28: Frequency of Related Adverse Events by MedDRA System Organ Class, Severity, and Treatment Group....148

Figure 29: Frequency of Related Adverse Events by MedDRA High Level Group Term, Severity, and Treatment Group....148

Figure 30: Forest Plot of DOOR Probability of All Adverse Events by MedDRA System Organ Class....149

Figure 31: Forest Plot of DOOR Probability of All Adverse Events by MedDRA High Level Group Term 149

Figure 32: Forest Plot of DOOR Probability of Experiencing a Clinical Laboratory Abnormality by Laboratory Parameter – Hematology Parameters 150

Figure 33: Forest Plot of DOOR Probability of Experiencing a Clinical Laboratory Abnormality by Laboratory Parameter – Serum Chemistry Parameters....151

10.1 Disposition of Subjects

Figure 1: CONSORT Flow Diagram

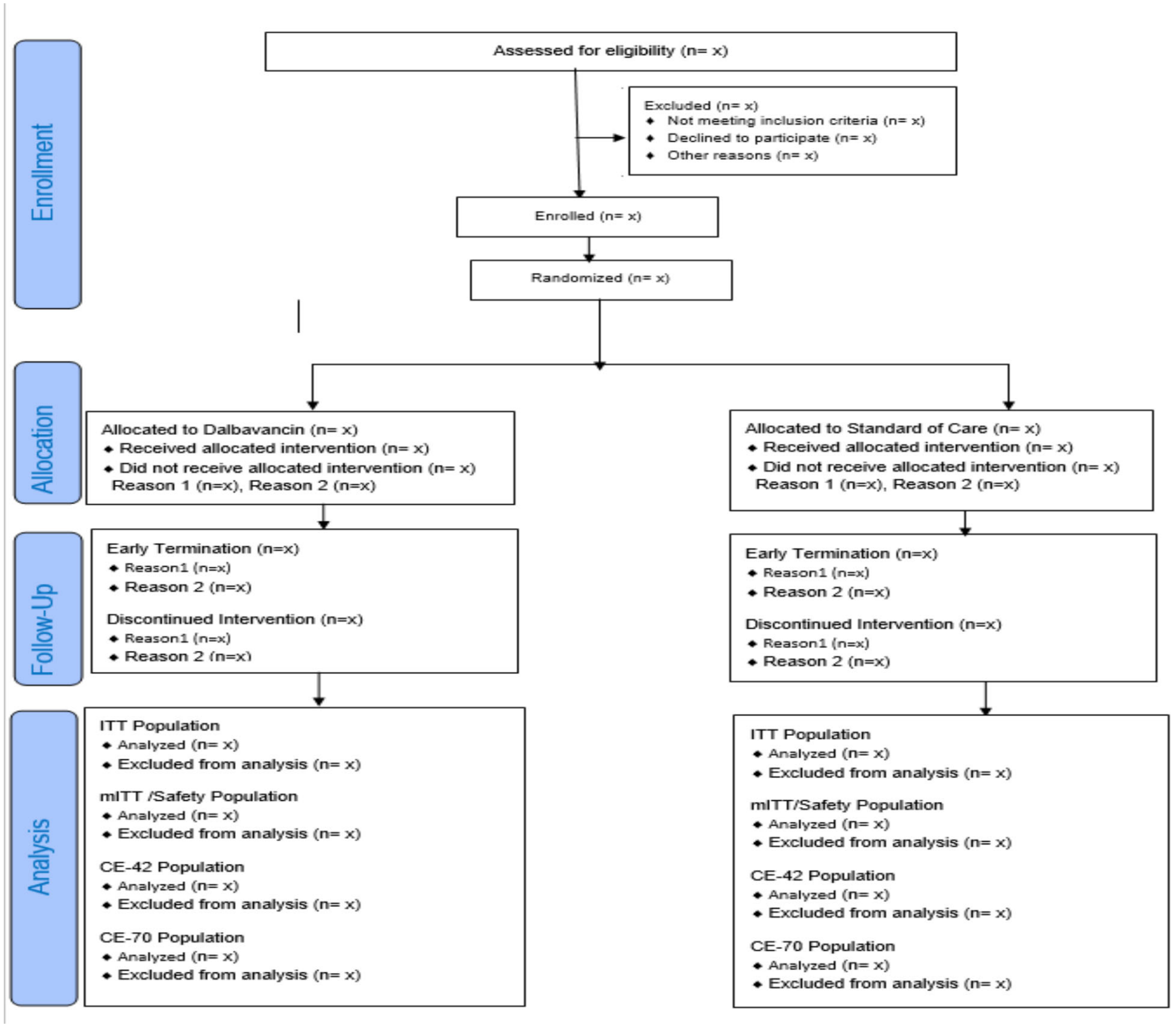

14.2.2 Efficacy Response Figures by Measure, Treatment, and Time Point

Figure 2: Forest Plot of DOOR Probability, Dalbavancin Relative to Standard of Care, of Clinical DOOR Components at Day 70 – ITT Population

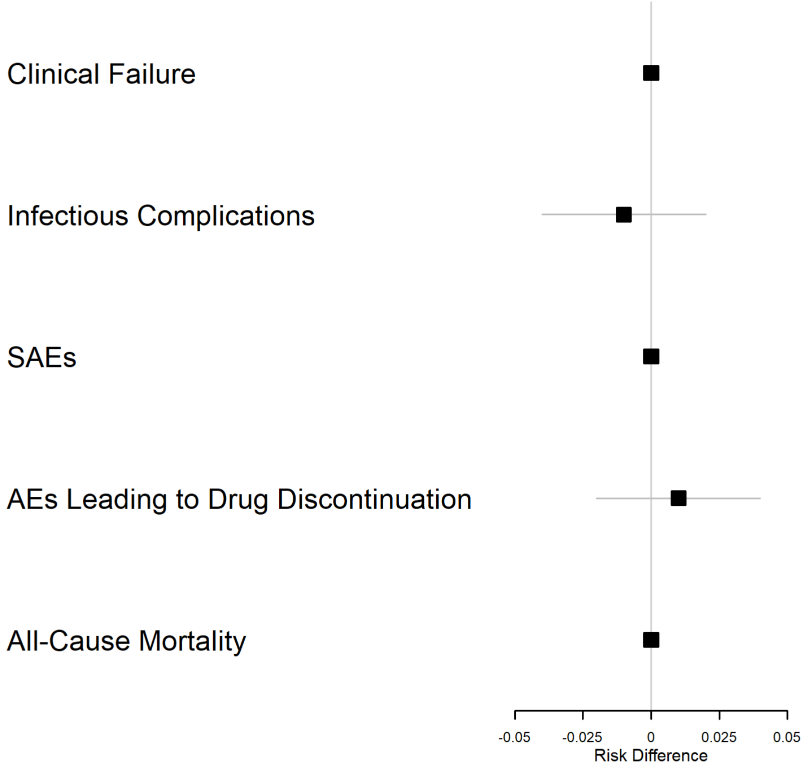

Programming Note: Include n (%) of subjects by arm as additional columns – next to description of each DOOR component. Additionally, change "Risk Difference" to DOOR Probability.

Figures similar to Figure 2:

Figure 3: Forest Plot of DOOR Probability, Dalbavancin Relative to Standard of Care, of Clinical DOOR Components at Day 70 – mITT Population

Figure 4: Forest Plot of DOOR Probability, Dalbavancin Relative to Standard of Care, of Clinical DOOR Components at Day 70 – CE Population

Figure 5: Forest Plot of DOOR Probability, Dalbavancin Relative to Standard of Care, of Clinical DOOR Components at Day 42 – ITT Population

Figure 6: Forest Plot of DOOR Probability, Dalbavancin Relative to Standard of Care, of Clinical DOOR Components at Day 42 – mITT Population

Figure 7: Forest Plot of DOOR Probability, Dalbavancin Relative to Standard of Care, of Clinical DOOR Components at Day 42 – CE Population

Figure 8: Distribution of DOOR at Day 70 – ITT Population

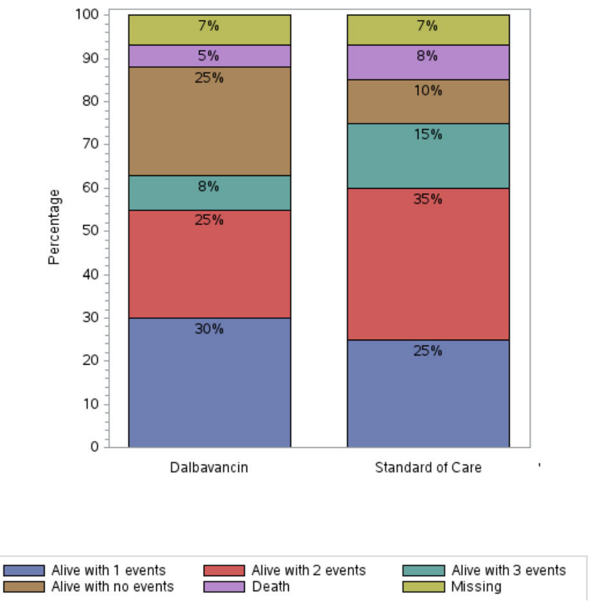

Figure 9: Distribution of DOOR at Day 70 – mITT Population

Figure will be similar to Figure 8.

Figure 10: Distribution of DOOR at Day 70 – CE Population

Figure will be similar to Figure 8.

[Implementation note: The missing category is removed from this analysis since the CE population has complete data.]

Figure 11: Distribution of DOOR at Day 42 – ITT Population

Figure will be similar to Figure 8.

Figure 12: Distribution of DOOR at Day 42 – mITT Population

Figure will be similar to Figure 8.

Figure 13: Distribution of DOOR at Day 42 – CE Population

Figure will be similar to Figure 8.

[Implementation note: The missing category is removed from this analysis since the CE population has complete data.]

Figure 14: Distribution of DOOR at Day 70 by Baseline Pathogen – ITT Population

Figure similar to Figure 8.

[Implementation note: Create two separate panels for MRSA and MSSA]

Figure 15: Distribution of DOOR at Day 70 by PWID Status – ITT Population

Figure similar to Figure 8.

[Implementation note: Create two separate panels for PWID and non-PWID]

Figure 16: Distribution of DOOR at Day 70 by Infectious Disease Consultation – ITT Population

Figure similar to Figure 8.

[Implementation note: Create two separate panels for Yes and No]

Figure 17: Distribution of DOOR at Day 70 by Underlying Site of Infection – ITT Population

Figure similar to Figure 8.

[Implementation note: Create four separate panels for the 4 different sites.]

Figure 18: Distribution of DOOR at Day 70 by Immunosuppression – ITT Population

[Implementation note: Create two separate panels for Yes and No]

Figure 19: Distribution of DOOR at Day 70 by Duration of Initial Bacteremia – ITT Population

Figure similar to Figure 8.

[Implementation note: Create three separate panels for <2, 2-4, >4 days]

Figure 20: Forest Plot of DOOR Probabilities at Day 70 – ITT Population

[Implementation note: This figure will be updated using SAS to add the rest of the subgroups and reformat the Y axis values as: Baseline Pathogen as title with indented categories MRSA, MSSA. Similar format update will be done for the rest of the subgroup categories. Caps will also be added to the error bars.]

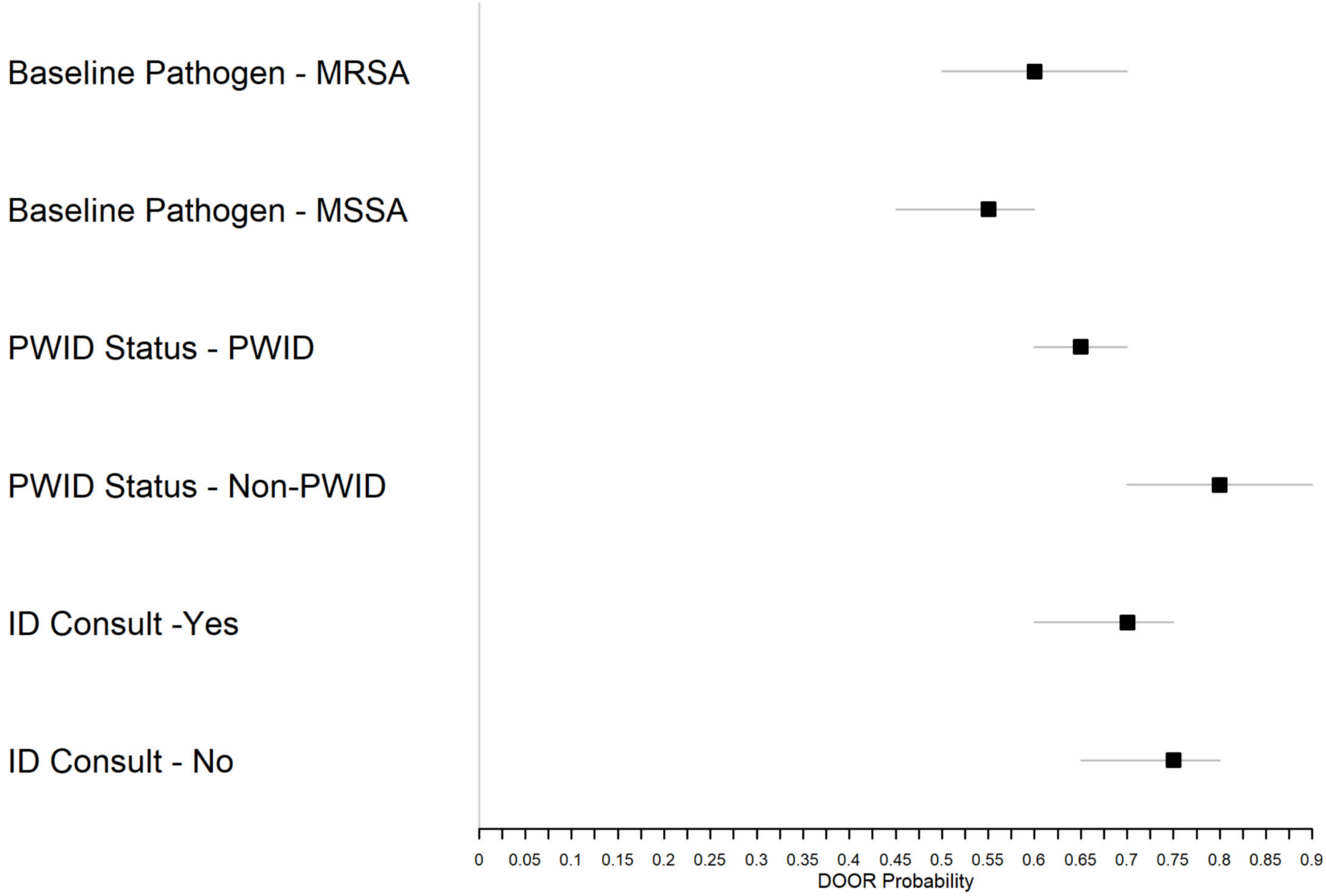

Similar to Figure 2.

Programming note: Include n (%) as columns.

Figure 21: Forest Plot of DOOR Probabilities at Day 42 – ITT Population

Figure similar to Figure 20.

Figure 22: Difference in Cumulative Proportions of DOOR Along with 95% CI

[Implementation note: Make a figure with 6 panels (2 rows representing Day 42 and Day 70; 3 columns representing the 3 analysis populations.]

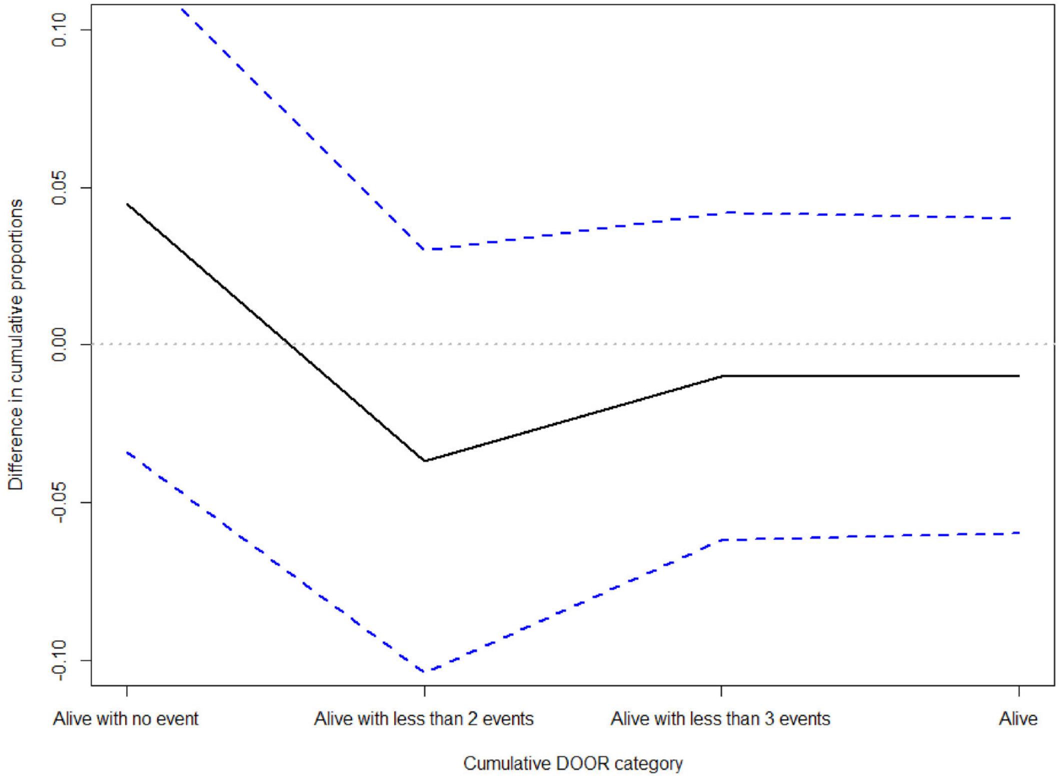

Figure 23: Difference in Means of Partial Credit Score by Timepoint and Analysis Population

[Implementation note: The figure will have 6 panels with two rows for Day 42 and Day 70 and two columns representing ITT, mITT, and CE analysis populations.]

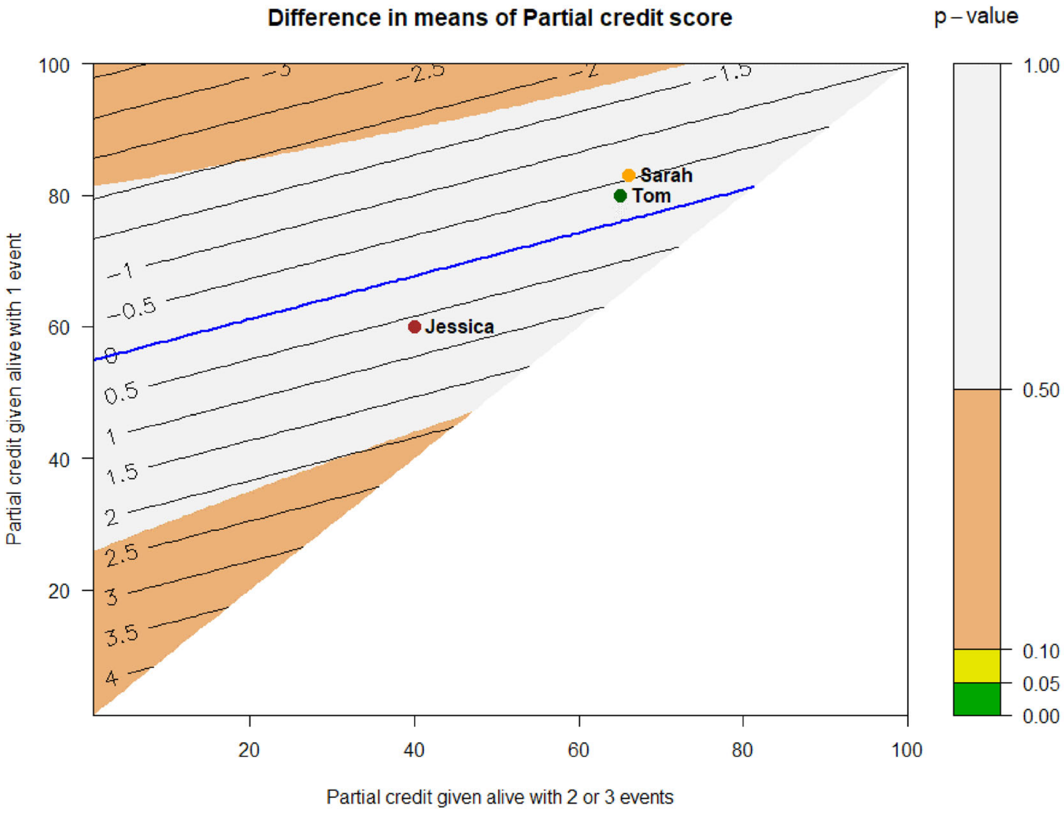

Figure 24: Bivariate Analysis of DOOR Probability vs Difference in Mean of Change in QoL Score from Baseline at Day 70 – ITT Analysis Population

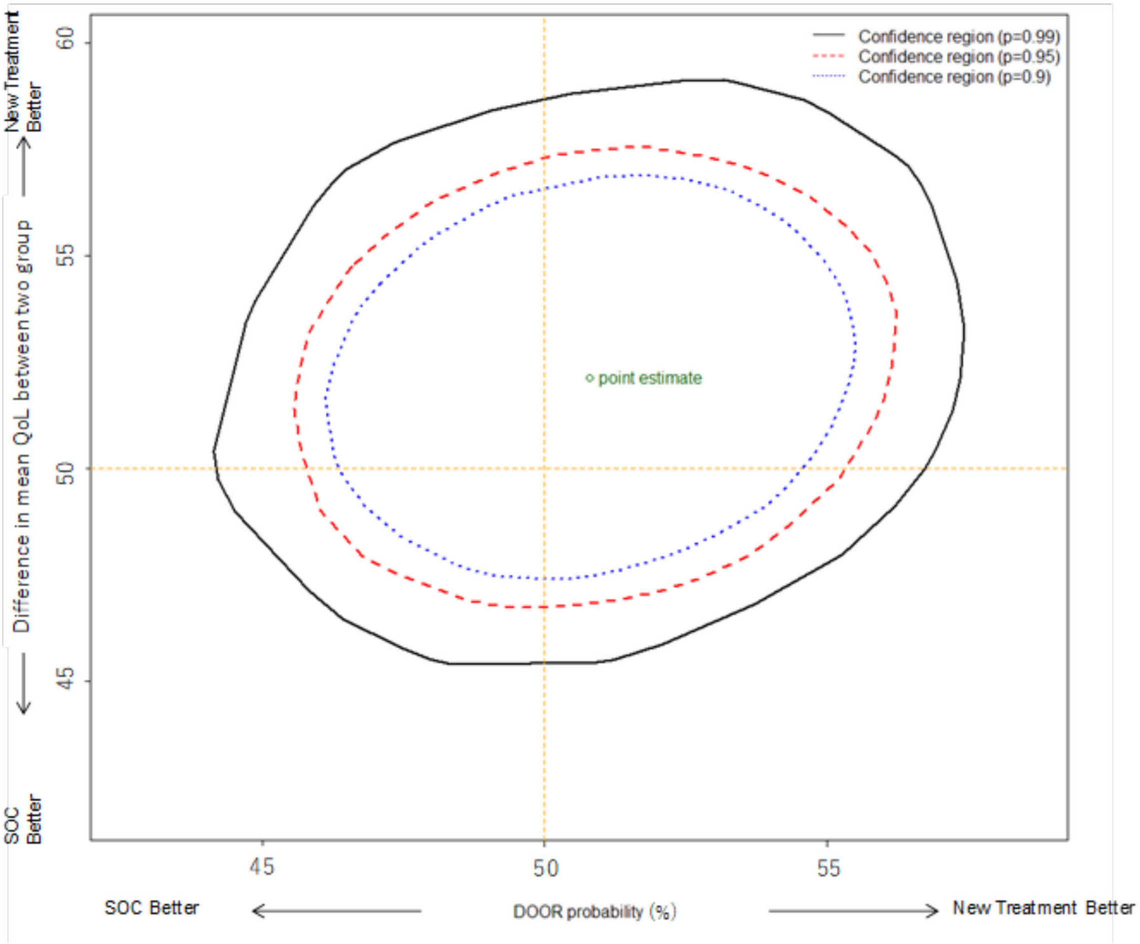

Figure 25: Bivariate Analysis of DOOR Probability vs Difference in Mean of Change in QoL Score from Baseline at Day 42 – ITT Analysis Population

Figure will be similar to Figure 24.

Figure 26: Predictive Interval Plots for the Probability of Higher DOOR in the Dalbavancin Group at Day 70 – ITT Analysis Population

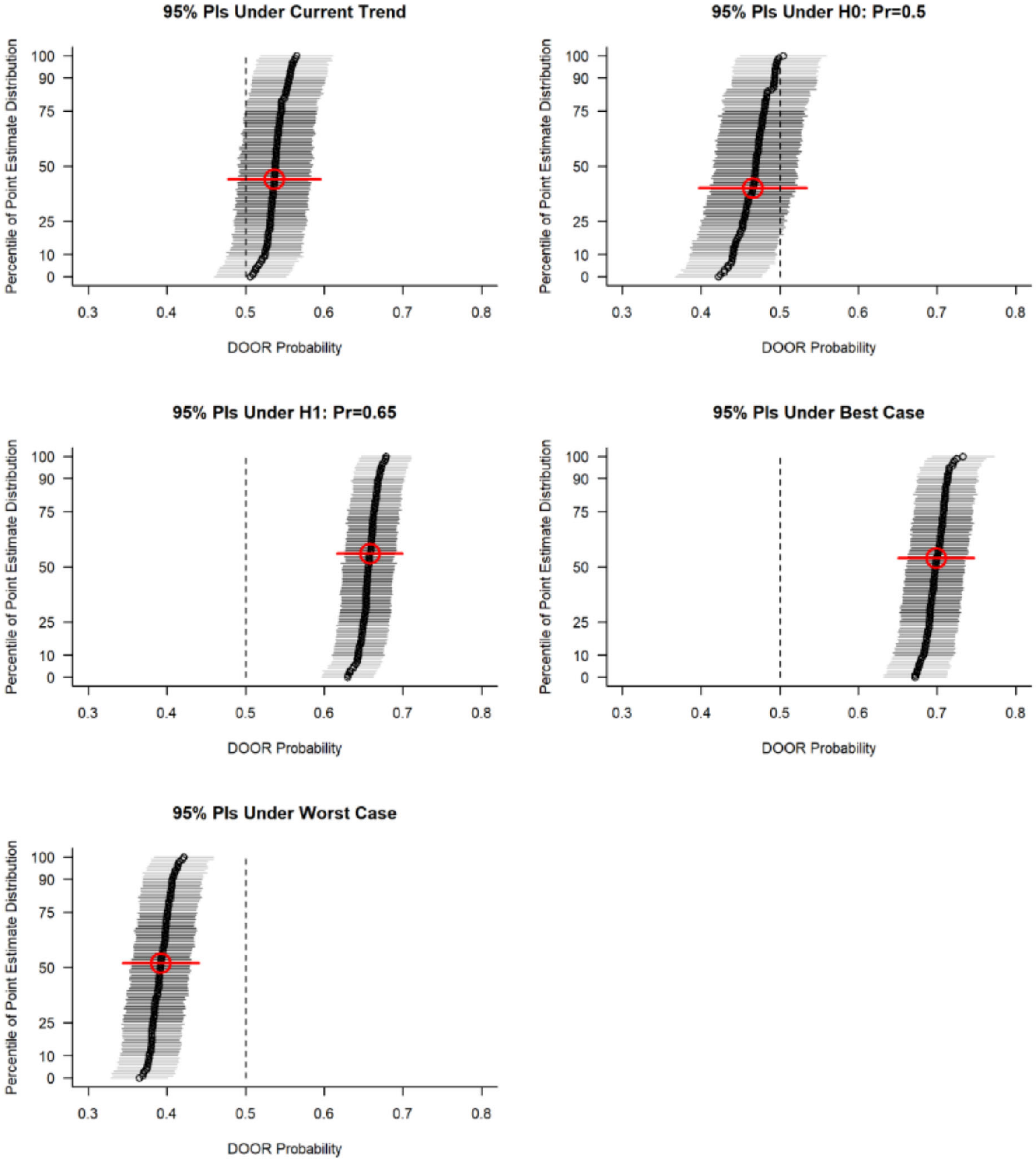

Figure with similar format:

Figure 27: Predictive Interval Plots Statistics for the Rates of Clinical Efficacy at Day 70 – ITT Analysis Population*

[Implementation note: Regenerate the figure for rates of clinical efficacy. Provide results for current trend, under null H0 with rate =0.5, under alternative H0 with rate=0.4, under best case and under worst case scenario.]

14.3.1.2 Unsolicited Adverse Events

Figure 28: Frequency of Related Adverse Events by MedDRA System Organ Class, Severity, and Treatment Group

[Implementation note: Panels for Dalbavancin and Standard of Care subjects will be presented. Grade 3 or higher will be reported for all AEs except AESIs which will include lower severities 1 and Grade 2.]

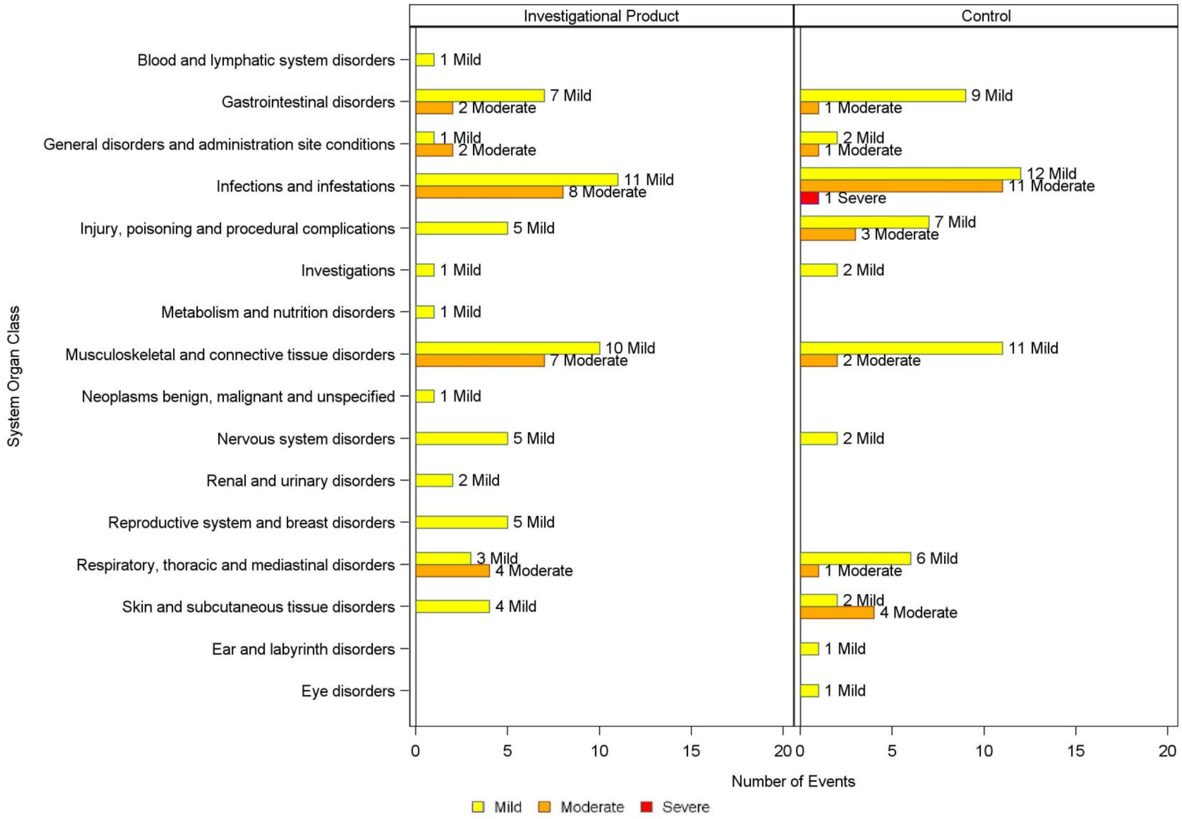

Figure 29: Frequency of Related Adverse Events by MedDRA High Level Group Term, Severity, and Treatment Group

Figure will be similar to Figure 28.

Figure 30: Forest Plot of DOOR Probability of All Adverse Events by MedDRA System Organ Class

[Implementation note: The 95% CI for the risk difference will be computed using the Miettinen-Nurminen method.]

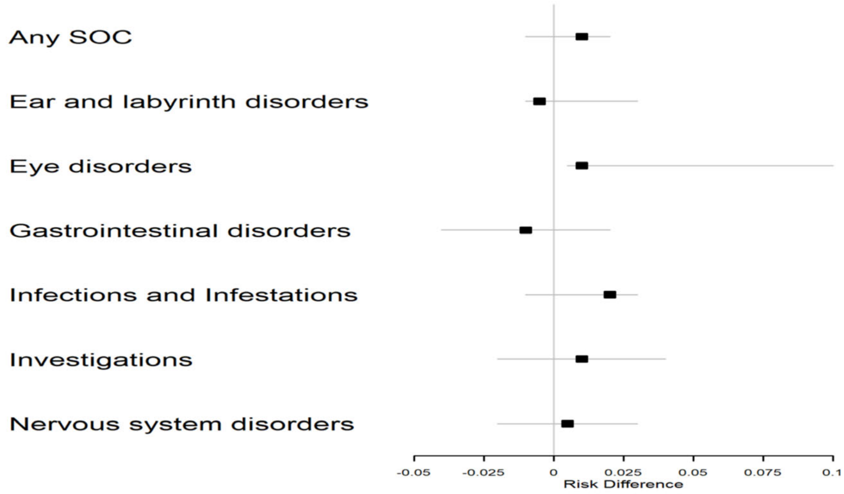

Programming Note: Change "Risk Difference" to DOOR Probability.

Implementation Note: Include n (%) as columns. See Figure 2 for example.

Figure 31: Forest Plot of DOOR Probability of All Adverse Events by MedDRA High Level Group Term

Figure will be similar to Figure 30.

Programming Note: Change "Risk Difference" to DOOR Probability.

Implementation Note: Include n (%) as columns. See Figure 2 for example.

14.3.5 Displays of Laboratory Results

Figure 32: Forest Plot of DOOR Probability of Experiencing a Clinical Laboratory Abnormality by Laboratory Parameter – Hematology Parameters

[Implementation note: Create separate panels for each visit. 95% confidence intervals for DOOR Probability will be computed using the Miettinen-Nurminen method.]

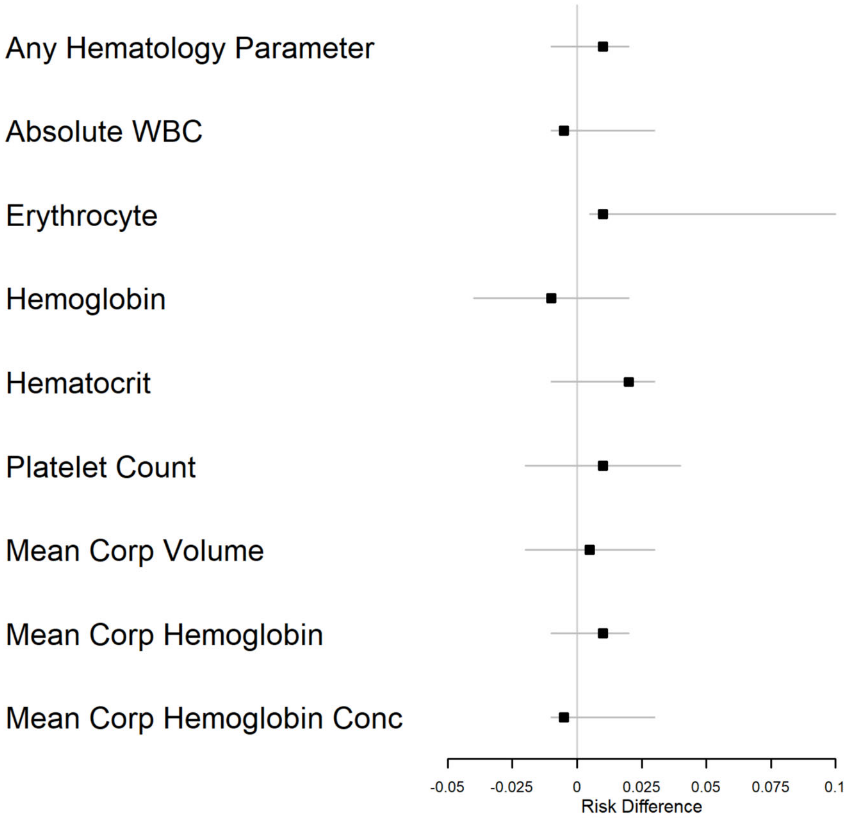

Programming Note: Change “Risk Difference” to DOOR Probability.

Figure 33: Forest Plot of DOOR Probability of Experiencing a Clinical Laboratory Abnormality by Laboratory Parameter – Serum Chemistry Parameters

[Implementation note: Create separate panels for each visit. 95% confidence intervals for DOOR Probability will be computed using the Miettinen-Nurminen method.]

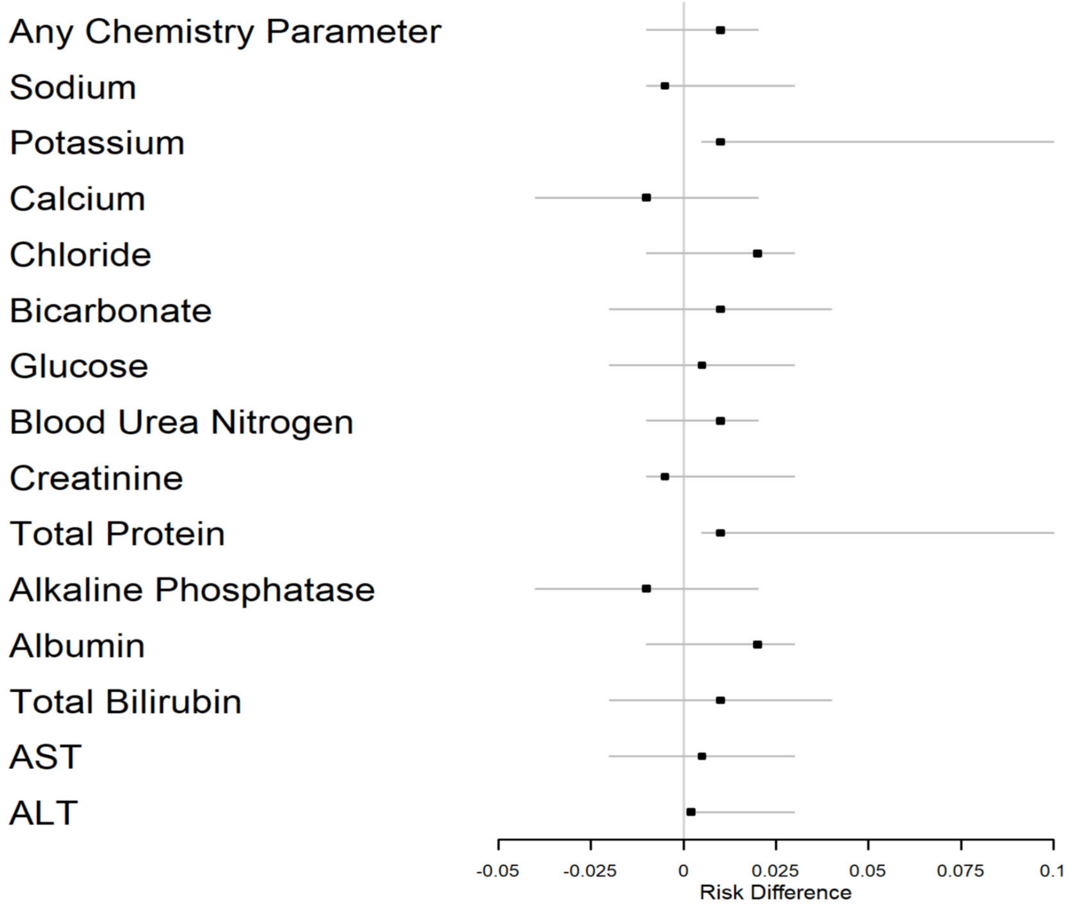

Programming Note: Change "Risk Difference" to DOOR Probability.

APPENDIX 3. LISTINGS MOCK-UPS

LISTINGS

Listing 1: 16.1.6: Listing of Subjects Receiving Investigational Product................................153
Listing 2: 16.2.1: Early Terminations or Discontinued Subjects 154
Listing 3: 16.2.2.1: Subject-Specific Protocol Deviations 155
Listing 4: 16.2.2.2: Non-Subject-Specific Protocol Deviations...156
Listing 5: 16.2.3: Subjects Excluded from Analysis Populations 157
Listing 6: 16.2.4.1: Demographic Data 158
Listing 7: 16.2.4.2: Pre-Existing and Concurrent Medical Conditions....................................159
Listing 8: 16.2.5: Compliance and/or Drug Concentration Data 160
Listing 9: 16.2.6.1: Individual DOOR Response Data...161
Listing 10: 16.2.6.2: Listing of Culture Results 162
Listing 11: 16.2.6.2: Individual QoL Data 163
Listing 12: 16.2.7.3: Listing of Treatment-Emergent Adverse Events 164
Listing 13: 16.2.7.3: Listing of All Adverse Events for Subjects with Potentially Clinically Significant Post-Baseline Clinical Laboratory or Vital Sign Values.......165
Listing 14: 16.2.8.1: Clinical Laboratory Results – Chemistry 166
Listing 15: 16.2.8.2: Clinical Laboratory Results – Hematology 167
Listing 16: 16.2.9.1: Vital Signs 168
Listing 17: 16.2.9.2: Physical Exam Findings 169
Listing 18: 16.2.10.1: Concomitant Medications...170
Listing 19: 16.2.10.1: Nondrug Interventions..170
Listing 20: 16.2.10.1: Echocardiogram Results...171
Listing 21: 16.2.11.1: Pregnancy Reports – Maternal Information 172
Listing 22: 16.2.11.2: Pregnancy Reports – Gravida and Para 172
Listing 23: 16.2.11.3: Pregnancy Reports – Live Birth Outcomes 173
Listing 24: 16.2.11.4: Pregnancy Reports – Still Birth Outcomes..173
Listing 25: 16.2.11.5: Pregnancy Reports – Spontaneous, Elective, or Therapeutic Abortion Outcomes...173

Listing 1: **16.1.6: Listing of Subjects Receiving Investigational Product**

(Not included in SAP, but this is a placeholder for the CSR)

16.2 Database Listings by Subject

16.2.1 Discontinued Subjects

Listing 2: 16.2.1: Early Terminations or Discontinued Subjects

Treatment Group	Subject ID	Category	Reason for Early Termination or Treatment Discontinuation	Study Day

16.2.2 Protocol Deviations

Listing 3: 16.2.2.1: Subject-Specific Protocol Deviations

Treatment Group	Subject ID	DV Number	Deviation	Deviation Category	Study Day	Reason for Deviation	Deviation Resulted in AE?	Deviation Resulted in Subject Termination?	Deviation Affected Product Stability?	Deviation Resolution	Comments

Listing 4: 16.2.2.2: Non-Subject-Specific Protocol Deviations

Site	Start Date	Deviation	End Date	Reason for Deviation	Deviation Resulted in Subject Termination?	Deviation Affected Product Stability?	Deviation Category	Deviation Resolution	Comments

16.2.3 Subjects Excluded from the Efficacy Analysis

Listing 5: 16.2.3: Subjects Excluded from Analysis Populations

Treatment Group	Subject ID	Analyses in which Subject is Included	Analyses from which Subject is Excluded	Reason Subject Excluded
Dalbavancin	SST.XXXX	[e.g., Safety, ITT, mITT]	CE42	
Dalbavancin	SST.XXXX		CE70	

PRIVILEGED AND CONFIDENTIAL COMMUNICATION

16.2.4 Demographic Data

Listing 6: 16.2.4.1: Demographic Data

Treatment Group	Subject ID	Sex	Age (years)	Ethnicity	Race	Height (cm)	Weight (kg)	BMI (kg/m^2)	Baseline Pathogen	Baseline QoL Score

Listing 7: 16.2.4.2: Pre-Existing and Concurrent Medical Conditions

Treatment Group	Subject ID	MH Number	Medical History Term	Condition Start Day	Condition End Day	MedDRA System Organ Class	MedDRA Preferred Term	MedDRA High Level Group Term

16.2.5 Compliance and/or Drug Concentration Data (if available)

Listing 8: 16.2.5: Compliance and/or Drug Concentration Data

[Implementation note: Sort by Treatment group and subject ID.]

Treatment Group	Subject ID	Dose Amount	Treatment Duration[a]	Creatinine Clearance[b]

[a] Treatment duration is defined as the number of doses for dalbavancin while it is defined as the duration between start and end dates for standard of care.
[b] Only included for subjects treated with Dalbavancin.

16.2.6 Individual Efficacy Response Data

Listing 9: 16.2.6.1: Individual DOOR Response Data

[Implementation note: Sort by treatment group, subject ID, and Planned Time Point.]

Treatment Group	Subject ID	Planned Time Point	Clinical Failure	Infectious Complications	SAEs	AEs Leading to Study Drug Discontinuation	All-cause Mortality	DOOR Category	Change in QoL from Baseline	DOOR	Clinical Efficacy	Microbiological Success
Dalbavancin	SST.XXXX	Day 42	Yes	No	No	No	No	2	40	120	No	Yes
Dalbavancin	SST.XXXX	Day 70	No	No	No	No	No	1	60	20	Yes	Yes

Listing 10: 16.2.6.2: Listing of Culture Results

Treatment Group	Subject ID	Culture Site	Collection Date	Collection Time	Culture Result	Pathogen Detected
Dalbavancin	SST.XXXX	Blood or Urine or Sputum, …	XXMMMYYYY	HH:MM	Positive or Negative	

Listing 11: 16.2.6.2: Individual QoL Data

Treatment Group	Subject ID	Planned Time Point	QoL Score Using ARLG Bloodstream Infection[a]	QoL Score Using PROMIS Global Health Short Form	QoL Score Using EQ-5D-5L instrument
Dalbavancin	SST.XXXX	Baseline			
Dalbavancin	SST.XXXX	Day 42			
Dalbavancin	SST.XXXX	Day 70			

[a] Standardized score is obtained from the items selected from the PROMIS physical function item bank (PROMIS Item Bank v2.0, short form 6b) item bank on the ARLG Bloodstream Infection QoL

16.2.7 Adverse Events

Listing 12: 16.2.7.3: Listing of Treatment-Emergent Adverse Events

Adverse Event	Study Day of AE Onset	Duration (Days)	Severity	Relationship to Study Treatment	If Not Related, Alternative Etiology	Action Taken with Study Treatment	Subject Discontinued Due to AE	Outcome	MedDRA System Organ Class	MedDRA Preferred Term	MedDRA High Level Group Term
**Treatment Group: , Subject ID: , AE Number:**											
Comments:											
**Treatment Group: , Subject ID: , AE Number:**											
Comments:											

PRIVILEGED AND CONFIDENTIAL COMMUNICATION

Listing 13: 16.2.7.3: Listing of All Adverse Events for Subjects with Potentially Clinically Significant Post-Baseline Clinical Laboratory or Vital Sign Values

Adverse Event	Study Day of AE Onset	Duration (Days)	Severity	Relationship to Study Treatment	If Not Related, Alternative Etiology	Action Taken with Study Treatment	Subject Discontinued Due to AE	Outcome	MedDRA System Organ Class	MedDRA Preferred Term	MedDRA High Level Group Term
**Treatment Group: , Subject ID: , AE Number:**											
Comments:											
**Treatment Group: , Subject ID: , AE Number:**											
Comments:											

16.2.8 Individual Laboratory Measurements

Listing 14: 16.2.8.1: Clinical Laboratory Results – Chemistry

Treatment Group	Subject ID	Planned Time Point	Actual Study Day	Sex	Age (years)	Laboratory Parameter (Units)	Result (Severity Grade)	Reference Range Low	Reference Range High

Listing 15: 16.2.8.2: Clinical Laboratory Results – Hematology

Treatment Group	Subject ID	Planned Time Point	Actual Study Day	Sex	Age (years)	Laboratory Parameter (Units)	Result (Severity Grade)	Reference Range Low	Reference Range High

16.2.9 Vital Signs and Physical Exam Findings

Listing 16: 16.2.9.1: Vital Signs

Treatment Group	Subject ID	Planned Time Point	Actual Study Day	Temperature (°F)	Systolic Blood Pressure (mmHg)	Diastolic Blood Pressure (mmHg)	Pulse (beats/min)	Respiratory Rate (breaths/min)

Listing 17: 16.2.9.2: Physical Exam Findings

Treatment Group	Subject ID	Planned Time Point	Actual Study Day	Body System	Abnormal Finding	Reported as an AE? (AE Description; Number)

16.2.10 Concomitant Medications

Listing 18: 16.2.10.1: Concomitant Medications

Treatment Group	Subject ID	CM Number	Medication	Medication Start Day	Medication End Day	Indication	Taken for an AE? (AE Description; Number)	Taken for a condition on Medical History? (MH Description; Number)	ATC Level 1 (ATC Level 2)

Listing 19: 16.2.10.1: Nondrug Interventions

Treatment Group	Subject ID	ND Number	Surgery/Procedure	Date of Procedure	Indication

Listing 20: 16.2.10.1: Echocardiogram Results

Treatment Group	Subject ID	Date of Procedure	Procedure Type	Result
			Transthoracic	Normal
			Transesophageal	Abnormal, not clinically significant
			Transthoracic	Abnormal, with evidence of left-sided endocarditis
			Transthoracic	Abnormal, with other clinically significant findings: specify

PRIVILEGED AND CONFIDENTIAL COMMUNICATION

16.2.11 Pregnancy Reports

Listing 21: 16.2.11.1: Pregnancy Reports – Maternal Information

Treatment Group	Subject ID	Pregnancy Number	Study Day Corresponding to Estimated Date of Conception	Source of Maternal Information	Pregnancy Status	Mother's Pre-Pregnancy BMI	Mother's Weight Gain During Pregnancy	Tobacco, Alcohol, or Drug Use During Pregnancy?	Medications During Pregnancy?	Maternal Complications During Pregnancy?	Maternal Complications During Labor, Delivery, or Post-Partum?
Note: Maternal Complications are included in the Adverse Event listing. Medications taken during pregnancy are included in the Concomitant Medications Listing.											

Listing 22: 16.2.11.2: Pregnancy Reports – Gravida and Para

			Live Births												
Subject ID	Pregnancy Number	Gravida	Extremely PB[a]	Very Early PB[a]	Early PB[a]	Late PB[a]	Early TB[b]	Full TB[a]	Late TB[a]	Post TB[a]	Still Births	Spontaneous Abortion/ Miscarriage	Elective Abortions	Therapeutic Abortions	Major Congenital Anomaly with Previous Pregnancy?
Note: Gravida includes the current pregnancy, para events do not.															

[a] Preterm Birth
[a] Term Birth

Listing 23: 16.2.11.3: Pregnancy Reports – Live Birth Outcomes

Subject ID	Pregnancy Number	Fetus Number	Pregnancy Outcome (for this Fetus)	Fetal Distress During Labor and Delivery?	Delivery Method	Gestational Age at Live Birth	Size for Gestational Age	Apgar Score, 1 minute	Apgar Score, 5 minutes	Cord pH	Congenital Anomalies?	Illnesses/ Hospitalizations within 1 Month of Birth?
Note: Congenital Anomalies are included in the Adverse Event listing.												

Listing 24: 16.2.11.4: Pregnancy Reports – Still Birth Outcomes

Subject ID	Date of Initial Report	Fetus Number	Pregnancy Outcome (for this Fetus)	Fetal Distress During Labor and Delivery?	Delivery Method	Gestational Age at Still Birth	Size for Gestational Age	Cord pH	Congenital Anomalies?	Autopsy Performed?	If Autopsy, Etiology for Still Birth Identified?

Listing 25: 16.2.11.5: Pregnancy Reports – Spontaneous, Elective, or Therapeutic Abortion Outcomes

Subject ID	Date of Initial Report	Fetus Number	Pregnancy Outcome (for this Fetus)	Gestational Age at Termination	Abnormality in Product of Conception?	Reason for Therapeutic Abortion

APPENDIX 4. NCA TEMPLATE

See separate document, if applicable.

PRIVILEGED AND CONFIDENTIAL COMMUNICATION

CLINICAL RESEARCH IN INFECTIOUS DISEASES

STATISTICAL ANALYSIS PLAN
for
DMID Protocol: 20-0002

Study Title:

Dalbavancin as an Option for Treatment of *S. aureus* Bacteremia (DOTS): A Phase 2b, Multicenter, Randomized, Open-Label, Assessor-Blinded Superiority Study to Compare the Efficacy and Safety of Dalbavancin to Standard of Care Antibiotic Therapy for the Completion of Treatment of Patients with Complicated *S. aureus* Bacteremia

NCT04775953

Version 2.0

DATE: 03JAN2024

Prepared and Distributed by:
Emmes
Rockville, Maryland USA

RESTRICTED

STUDY TITLE

**Protocol Number Code:**	**DMID Protocol: 20-0002**
**Development Phase:**	Phase 2b
**Products:**	Dalbavancin<br>Standard of Care Antibiotics: Cefazolin, nafcillin, oxacillin, vancomycin, and daptomycin
**Form/Route:**	IV
**Indication Studied:**	Complicated *S. aureus* Bacteremia
**Sponsor:**	Division of Microbiology and Infectious Diseases<br>National Institute of Allergy and Infectious Diseases<br>National Institutes of Health
**Clinical Trial Initiation Date:**	15MAR2021
**Clinical Trial Completion Date:**	TBD
**Date of the Analysis Plan:**	03JAN2024
**Version Number:**	Version 2.0

This study was performed in compliance with Good Clinical Practice.

Information contained in this publication is the property of Division of Microbiology and Infectious Diseases and is confidential. This information may not be disclosed to third parties without written authorization from Division of Microbiology and Infectious Diseases. This report may not be reproduced, stored in a retrieval system or transmitted in any form or by any means - electronic, mechanical, recording or otherwise - without the prior authorization from Division of Microbiology and Infectious Diseases. This document must be returned to Division of Microbiology and Infectious Diseases upon request.

TABLE OF CONTENTS

STUDY TITLE 2
TABLE OF CONTENTS..3
LIST OF ABBREVIATIONS..7
1. PREFACE..9
2. INTRODUCTION 10
2.1. Purpose of the Analyses..10
3. STUDY OBJECTIVES AND ENDPOINTS..11
3.1. Study Objectives 11
3.1.1. Primary 11
3.1.2. Secondary 11
3.1.3. Exploratory 11
3.2. Endpoints 12
3.2.1. Primary 12
3.2.2. Secondary 12
3.2.3. Exploratory 13
3.3. Study Definitions and Derived Variables 13
4. INVESTIGATIONAL PLAN..15
4.1. Overall Study Design and Plan..15
4.2. Discussion of Study Design, Including the Choice of Control Groups..15
4.3. Selection of Study Population 16
4.3.1. Inclusion Criteria 16
4.3.2. Exclusion Criteria 16
4.3.3. Reasons for Withdrawal 16
4.4. Treatments 18
4.4.1. Treatments Administered..18
4.4.2. Identity of Investigational Product(s) 18
4.4.3. Method of Assigning Subjects to Treatment Groups (Randomization) 18
4.4.4. Selection of Doses in the Study 18
4.4.5. Blinding 19
4.4.6. Prior and Concomitant Therapy..19

RESTRICTED

Table of Contents (continued)

4.4.7. Treatment Compliance 19
4.5. Efficacy and Safety Variables 20
5. SAMPLE SIZE CONSIDERATIONS 21
6. GENERAL STATISTICAL CONSIDERATIONS 22
6.1. General Principles 22
6.2. Timing of Analyses 22
6.3. Analysis Populations 22
6.3.1. Screened Analysis Population 23
6.3.2. Intent-to-Treat Analysis Population 23
6.3.3. Safety Population 23
6.3.4. Modified Intent-to-Treat Population 23
6.3.5. Clinically Evaluable Analysis Population 23
6.4. Covariates and Subgroups 24
6.5. Missing Data 24
6.5.1. DOOR Categories 24
6.5.2. Quality of Life Score (QoL) 25
6.5.3. Desirability of Outcome Ranking at Day 70 or Day 42 26
6.6. Interim Analyses and Data Monitoring 27
6.7. Multicenter Studies 27
6.8. Multiple Comparisons/Multiplicity 27
7. STUDY SUBJECTS 28
7.1. Disposition of Subjects 28
7.2. Protocol Deviations 28
8. EFFICACY EVALUATION 29
8.1. Primary Efficacy Analysis 29
8.1.1. Analysis of DOOR at Day 70 Using ITT Analysis Population 29
8.1.1.1. ITT Analysis of DOOR using IPW 29
8.1.1.2. ITT Analysis of DOOR using Multiple Imputation 31
8.2. Secondary Efficacy Analyses 33
8.2.1. Analysis of Clinical Efficacy at Day 70 using IPW for the ITT and mITT Analysis Populations 33

RESTRICTED

Table of Contents (continued)

8.2.2. Sensitivity Analysis of Clinical Efficacy at Day 70 using Multiple Imputation for the ITT and mITT Analysis Populations 34
8.2.3. Analysis of DOOR Components Using ITT Analysis Population 36
8.2.4. Analysis of Clinical Failure at Day 70 Using ITT Population 36
8.3. Exploratory Efficacy Analyses 37
8.3.1. Analysis of Clinical Efficacy at Day 42 37
8.3.2. Additional Analyses of DOOR 37
8.3.2.1. Analysis of DOOR at Day 42 Using ITT and mITT Analysis Populations 37
8.3.2.2. Subgroup Analyses of DOOR 37
8.3.3. Analysis of DOOR Components at Day 42 and Day 70 Using mITT Analysis Populations 38
8.3.3.1. Analysis of DOOR Categories at Day 42 and Day 70 Using ITT and mITT Analysis Populations 38
8.3.3.2. Distribution of DOOR by Treatment Group Using ITT and mITT Analysis Populations 38
8.3.3.3. Cumulative Difference in DOOR Categories Using ITT and mITT Analysis Populations 38
8.3.3.4. Analysis of Difference in Mean Partial Credit Using ITT and mITT Analysis Populations 38
8.3.3.5. Analysis of Expected DOOR Category Distribution and Expected Numbers Gained Loss Using ITT and mITT Analysis Populations 38
8.3.4. Analysis of Microbiological Success at Day 42 and Day 70 using the ITT and mITT Analysis Populations 39
8.3.5. Analysis of QoL Score using ITT and mITT Analysis Populations 39
8.3.6. Bivariate Analysis of DOOR Probability vs Difference in Mean of Change in QoL Score from Baseline 39
8.3.7. Analysis of Late Recurrence Within ITT Population with Osteomyelitis 40
8.4. Supplemental Efficacy Analyses 40
8.4.1. Analysis of DOOR at Day 42 and 70 Using CE Analysis Populations 40
8.4.2. Analysis of Clinical Efficacy at Day 70 Using CE Analysis Population 41
8.4.3. Analysis of DOOR at Day 42 and Day 70 Using CE Analysis Population 41
8.4.3.1. Cumulative Difference in DOOR Using CE Analysis Population 41
8.4.3.2. Analysis of Difference in Mean Partial Credit Using CE Analysis Population 41
8.4.3.3. Analysis of Expected DOOR Distribution and Expected Numbers Gained Loss 42

Table of Contents (continued)

8.4.4. Analysis of Microbiological Success at Day 42 and Day 70 using the CE Analysis Populations 42
8.5. Interim Analyses 42
8.6. Imputation of Missing Data 43
8.6.1. Multiple Imputation of Missing DOOR Day 42 and Day 70 43
8.6.2. Multiple Imputation (MI) of Missing Values of Clinical Efficacy and Microbiologic Success on Day 42 and Day 70....44
9. SAFETY EVALUATION 46
9.1. Demographic and Other Baseline Characteristics 46
9.1.1. Prior and Concurrent Medical Conditions....46
9.1.2. Prior or Concomitant Medications and Nondrug Interventions 46
9.2. Measurements of Treatment Compliance....47
9.3. Adverse Events 47
9.3.1. Treatment-Emergent Adverse Events....47
9.4. Deaths, Serious Adverse Events, and Other Significant Adverse Events 48
9.5. Pregnancies....48
9.6. Clinical Laboratory Evaluations....48
9.7. Vital Signs and Physical Evaluations 49
9.8. Concomitant Medications and Nondrug Interventions....49
10. PHARMACOKINETICS 50
11. OTHER ANALYSES 51
12. REPORTING CONVENTIONS 52
13. TECHNICAL DETAILS....53
14. SUMMARY OF CHANGES IN THE CONDUCT OF THE STUDY OR PLANNED ANALYSES....54
15. REFERENCES 55
16. LISTING OF TABLES, FIGURES, AND LISTINGS 56
APPENDICES 57
APPENDIX 1. TABLE MOCK-UPS....58
APPENDIX 2. FIGURE MOCK-UPS....135
APPENDIX 3. LISTINGS MOCK-UPS....150

LIST OF ABBREVIATIONS

ABSSSI	Acute Bacterial Skin and Skin Structure Infections
AE	Adverse Event
AESI	Adverse Event of Special Interest
ALT	Alanine Aminotransferase
ARLG	Antibacterial Resistance Leadership Group
AST	Aspartate Aminotransferase
BP	Blood Pressure
BUN	Blood Urea Nitrogen
CE	Clinically Evaluable
CI	Confidence Interval
CRF	Case Report Form
CrCl	Creatinine Clearance
DCC	Data Coordinating Center
DMID	Division of Microbiology and Infectious Diseases
DSMB	Data and Safety Monitoring Board
DOOR	Desirability of Outcome Ranking
DOTS	Dalbavancin as an Option for Treatment of *S. aureus* Bacteremia
eCRF	Electronic Clinical Report Form
EDC	Electronic Data Capture
ET	Early Termination
F	Fahrenheit
FDA	Food and Drug Administration
GEE	Generalized Estimating Equations
GLMM	Generalized Linear Mixed Model
HLGT	High Level Group Term
ICH	International Council for Harmonisation
IE	Infective Endocarditis
IPW	Inverse Probability Weighting
IRB	Institutional Review Board
ITT	Intent-to-Treat
L	Liter
MAR	Missing at Random
MCAR	Missing Completely at Random

List of Abbreviations (continued)

MedDRA	Medical Dictionary for Regulatory Activities
mEq	Milliequivalent
mg	Milligram
mITT	Modified Intent-to-Treat
mL	Milliliter
MNAR	Missing Not at Random
MRSA	Methicillin-resistant *Staphylococcus aureus*
MSSA	Methicillin-sensitive *Staphylococcus aureus*
MVUE	Minimum Variance Unbiased Estimator
NIAID	National Institute of Allergy and Infectious Diseases
NIH	National Institutes of Health
PCS	Potentially Clinically Significant
PD	Pharmacodynamics
PI	Principal Investigator
PID	Patient Identification
PIPS	Predicted Interval Plots
PK	Pharmacokinetics
PT	Preferred Term
PTime	Prothrombin Time
PTT	Partial Thromboplastin Time
PWID	Person Who Injects Drugs
SAE	Serious Adverse Event
SD	Standard Deviation
SDCC	Statistical and Data Coordinating Center
SMC	Safety Monitoring Committee
SOC	System Organ Class
SOP	Standard Operating Procedures
TEE	Transesophageal Echocardiography
TOC	Test of Cure
ULN	Upper Limit of Normal
WBC	White Blood Cell
WHO	World Health Organization

1. PREFACE

The Statistical Analysis Plan (SAP) for "Dalbavancin as an Option for Treatment of *Staphylococcus aureus* (*S. aureus)* Bacteremia (DOTS): A Phase 2b, Multicenter, Randomized, Open-Label, Assessor-Blinded Superiority Study to Compare the Efficacy and Safety of Dalbavancin to Standard of Care Antibiotic Therapy for the Completion of Treatment of Patients with Complicated *S. aureus* Bacteremia" (DMID Protocol 20-0002) describes and expands upon the statistical information presented in the protocol.

This document describes all planned analyses and provides reasons and justifications for these analyses. It also includes sample tables, listings, and figures planned for the final analyses. Regarding the final analyses and Clinical Study Report (CSR), this SAP follows the International Council for Harmonisation of Technical Requirements for Registration of Pharmaceuticals for Human Use (ICH) Guidelines, as indicated in Topic E3 (Structure and Content of Clinical Study Reports), and more generally is consistent with Topic E6 (Good Clinical Practice: Integrated Addendum to ICH E6(R2)), Topic E8 (General Considerations for Clinical Trials), Topic E9 (Statistical Principles for Clinical Trials), Topic E9 (R1: Addendum on Estimands and Sensitivity Analysis in Clinical Trials to the Guidelines on Statistical Principles for Clinical Trials) and Topic E10 (Choice of Control Group and Related Issues in Clinical Trials). The structure and content of the SAP provides sufficient detail to meet the requirements identified by the Food and Drug Administration (FDA) and ICH, while all work planned and reported for this SAP will follow internationally accepted guidelines published by the American Statistical Association and the Royal Statistical Society for statistical practice.

This document contains four sections: (1) a review of the study design, (2) general statistical considerations, (3) comprehensive statistical analysis methods for efficacy and safety outcomes, and (4) a list of proposed tables and figures. Within the table, figure, and listing mock-ups (Appendices 1, 2, and 3), references to CSR sections are included. Any deviation from this SAP will be described and justified in protocol amendments and/or in the CSR, as appropriate. The reader of this SAP is encouraged to also review the study protocol for details on conduct of the study and the operational aspects of clinical assessments.

2. INTRODUCTION

This is a Phase 2b, multicenter, randomized, open-label, assessor-blinded superiority clinical trial of dalbavancin vs. standard of care antibiotic therapy for treating complicated *S. aureus* bacteremia. The study is designed and powered for the primary analysis of a comparison of the efficacy of dalbavancin to standard of care therapy for the completion of therapy in patients with complicated bacteremia or right-sided native valve Infective Endocarditis (IE) caused by *S. aureus* who have cleared their baseline bacteremia using a superiority approach.

Approximately 200 subjects will be randomized 1:1 to receive either dalbavancin or a standard of care antibiotic regimen that is based upon the identification and antibiotic susceptibility pattern of the baseline organism. Those randomized to the dalbavancin treatment group will receive 2 doses of dalbavancin intravenous (IV) 1 week apart (1500 mg on Day 1 and Day 8 after randomization, with renal dose adjustment if appropriate). Those subjects randomized to the standard of care antibiotic therapy treatment group will receive an antibiotic regimen considered to be standard of care based on the methicillin susceptibility pattern of the pathogen isolated at Baseline for a duration of 4 to 6 weeks. The duration of standard of care antibiotics may be extended to a maximum of 8 weeks at the discretion of the treating clinician.

2.1. Purpose of the Analyses

Analysis of Desirability of Outcome Ranking (DOOR) at Day 70 will be used to assess the efficacy of dalbavancin versus standard of care therapy. Superiority of dalbavancin versus standard of care on Day 70 based on DOOR will be the primary analysis. With DOOR being a composite endpoint, the advantage of dalbavancin on the DOOR analysis does not necessarily imply an advantage on all DOOR components. Thus, examination of the effects on the overall clinical outcome and each component of DOOR is standardly conducted via a secondary or sensitivity analysis. Hence, the secondary outcome measures which include a comparison of clinical outcomes, safety outcomes, and comparison of each individual DOOR component will be conducted. Note that DOOR categories will be calculated using cumulative data from Day 1 through Day 70, except that clinical success or failure is specific to the date of assessment. For example, suppose a subject had experienced an SAE and clinical failure at Day 42 but at Day 70 they were considered to be cured. In this case, the SAE would still contribute to the DOOR at Day 70; however, the change from clinical failure at Day 42 to clinical success at Day 70 would result in a lower (better) DOOR at Day 70, reflective of the improvement in clinical outcome.

3. STUDY OBJECTIVES AND ENDPOINTS

3.1. Study Objectives

3.1.1. Primary

To compare DOOR at Day 70 of dalbavancin to that of standard of care antibiotic therapy used to consolidate therapy for the treatment of subjects with complicated *S. aureus* bacteremia in the intent-to-treat (ITT) population.

3.1.2. Secondary

1. To compare the clinical outcomes of dalbavancin with the standard of care antibiotic therapy at Day 70 in the modified intent-to-treat (mITT) population (see Section 6.3 for definitions of study populations).
2. To compare the safety of dalbavancin with that of the standard of care treatment in the mITT population.
3. To compare each individual component of DOOR outcome by treatment arm, in the ITT population.

3.1.3. Exploratory

1. To compare the clinical outcomes of dalbavancin with the standard of care antibiotic therapy at Day 70 in the Clinically Evaluable (CE) population (see Section 6.3 for definition).
2. To compare the DOOR endpoint of subjects on dalbavancin with that of subjects receiving standard of care antimicrobial therapy at Day 42, in the ITT, mITT and CE populations.
3. To compare the clinical and microbiologic outcomes of dalbavancin with the standard of care antibiotic therapy at Day 42 in ITT, mITT and CE populations.
4. To compare clinical and microbiologic outcomes of dalbavancin with standard of care antibiotic therapy between clinically important subgroups, including a) those with MSSA versus MRSA; b) persons who inject drugs (PWID) vs non-PWID; c) those who received infectious disease consultation vs those who did not; d) underlying site of infection (endovascular, bone and joint, skin/skin structure pulmonary); e) subjects with immune-suppression (not severe enough to trigger exclusion)[1]; f) divided by duration of initial bacteremia, in the ITT, mITT, and CE populations.
5. To compare Quality of Life (QoL) of subjects on dalbavancin with that of subjects receiving standard of care antibiotic therapy at Baseline, Day 42, and Day 70, in the ITT, mITT, and CE populations.

---

1 Defined as: On chemotherapy or immunotherapy for active hematologic malignancy expected to cause ANC < 500 cells/mm3 lasting > 7 days during the study period, chronic high dose oral steroids (equivalent of ≥ 20 mg prednisolone per day for or equivalent, for >2 weeks within the last month), HIV infection with a CD4 cell count < 100 cells/mm3 based on last known measurement or patient-reported value

6. To characterize the population pharmacokinetic (PK) profile for dalbavancin administered via a 2-dose regimen (1500 mg on day 1 and 1500 mg on day 8; renally adjusted when appropriate) in patients with *S. aureus* bacteremia.
7. To assess patient-level and clinical covariates associated with dalbavancin pharmacokinetics in patients with *S. aureus* bacteremia.
8. Examine the association between individualized plasma concentration profiles and clinical and microbiologic outcomes at Day 42 and Test of Cure (TOC).
9. Examine the association between individualized plasma concentration profiles and occurrence of adverse drug events, including AST/ALT elevations >3X upper limit of normal.
10. Examine the association between individualized plasma concentration profiles and late recurrence risk among the subset of patients with osteomyelitis and a 6-month follow-up visit.

3.2. Endpoints

3.2.1. Primary

The primary outcome measure is the DOOR endpoint at Day 70. The clinical components of the DOOR endpoint (success/failure and infectious complications) will be completed by an independent adjudication committee, blinded to treatment assignment. Day 70 was selected in this study as it occurs at least 4 weeks after treatment completion for most patients, allowing time for detection of relapse.

Clinical failure, infectious complications, and adverse events are each binary (yes/no) components of DOOR.

For the primary analysis of DOOR, QoL will be used as a tie-breaker and will be calculated as change from baseline QoL to Day 70 QoL score, as assessed by questions from the PROMIS physical function item bank (PROMIS Item Bank v2.0, short form 6b) on the Antibacterial Resistance Leadership Group (ARLG) Bloodstream Infection QoL Measure (Appendix C of the protocol).

3.2.2. Secondary

The **secondary efficacy outcome** is as follows.

Clinical efficacy, defined as none of 1) Clinical failure; 2) Infectious complications; 3) All-cause mortality

The **secondary safety outcome** is as follows.

Safety, defined as proportion of patients who have either 1) an SAE; or 2) an AE leading to study drug discontinuation

Each component of DOOR will also be examined separately:

- clinical failure
- infectious complications
- SAEs
- AEs leading to study drug discontinuation
- all-cause mortality

3.2.3. Exploratory

1. Clinical efficacy by treatment group in the CE population at Day 70.
2. DOOR endpoint by treatment group at Day 42 in the ITT, mITT, and CE populations.
3. Clinical and microbiologic outcomes by treatment group at Day 42 in the ITT, mITT, and CE populations. A microbiologic success will be defined as no post-randomization growth (e.g., no positive cultures) of the baseline pathogen from blood cultures or another sterile body site.
4. Clinical and microbiologic outcomes by treatment group, within each subgroup of clinical interest, at Day 42 and Day 70 in the ITT, mITT, and CE populations.
5. QoL at Baseline, Day 42, and Day 70, which will be assessed by the score obtained from questions from the PROMIS physical function item bank (PROMIS Item Bank v2.0, short form 6b), included in the ARLG Bloodstream Infection QoL Measure (Appendix C of the protocol), as well as two additional comparator measures (EQ-5D-5L - https://euroqol.org/eq-5d-instruments/sample-demo/ and PROMIS Global Health short form - http://www.healthmeasures.net/administrator/components/com_instruments/uploads/Global%20Health%20Scale%20v1.2%2008.22.2016.pdf) in the ITT, mITT, and CE populations.
6. Population mean PK parameter estimates and the magnitude of the associated inter individual variability for the 2-dose dalbavancin regimen in patients with *S. aureus* bacteremia.
7. Individual post hoc PK parameter estimates and calculated exposure measures for the 2-dose dalbavancin regimen in patients with *S. aureus* bacteremia.
8. Clinical and microbiologic response at Day 42 and Day 70 according to individual plasma dalbavancin concentration curves.
9. Occurrence of grade 3 or higher adverse drug events, adverse events of special interest (AESIs), and occurrence of AST/ALT elevations >3x upper limit of normal (ULN) from first dose of dalbavancin through follow-up period.
10. Late recurrence within the osteomyelitis population will be defined by the presence of the following up to 6 months after randomization: progressive imaging changes along with isolation of *S. aureus* from blood, bone biopsy, associated fluid aspiration, or operative tissue.

3.3. Study Definitions and Derived Variables

DOOR:

There are 5 possibilities for the DOOR:

Rank 1: Alive without any of the following: (1) evidence of clinical failure; (2) an infectious complication; or (3) any SAE, or an AE leading to study drug discontinuation

Rank 2: Alive but with one of the following: (1) evidence of clinical failure; (2) an infectious complication; or (3) any SAE, or an AE leading to study drug discontinuation

Rank 3: Alive but with two of the following: (1) evidence of clinical failure; (2) an infectious complication; or (3) any SAE, or an AE leading to study drug discontinuation

Rank 4: Alive but with all of the following: (1) evidence of clinical failure; (2) an infectious complication; or (3) any SAE, or an AE leading to study drug discontinuation

Rank 5: Death

Note that if an infectious complication is also an SAE, or an AE leading to study drug discontinuation the event will count twice towards the DOOR. For example, if a subject is alive at Day 70 point and the only observed event between Day 1 and Day 70 is a serious infectious complication, the subject will have Rank 3 at Day 70. Additionally, DOOR is calculated using cumulative data from Day 1 through Day 70, except that clinical success or failure is specific to the date of assessment.

Additional details for deriving DOOR, including handling of missing data, are provided in Section 6.5.

Clinical Success: Resolution of clinical signs and symptoms of *S. aureus* bacteremia such that no additional antibiotic therapy is required or anticipated for its treatment.

Note that it is possible to achieve this overall Clinical Success status at Day 70 even if infectious complications have occurred prior to that time. For example, a patient who has a new metastatic focus of infection diagnosed after randomization, but who subsequently completes treatment and is felt to be cured at Day 70 would be considered a Clinical Success (and the Infectious Complication would result in a lower DOOR). That is, Clinical Success reflects the patient's overall status at the time of that assessment.

Determination of clinical success/failure and infectious complications at Day 42 and Day 70 will be determined by the adjudication committee after review of all relevant data up to Day 42 and Day 70, respectively.

Clinical Failure: Absence of clinical success

Infectious Complications: Occurrence of any of the following, between randomization and Day 70:

- Endocarditis
- New evidence of metastatic foci of infection – e.g., osteomyelitis, visceral abscess, septic joint
- Relapse – isolation of baseline *S. aureus* pathogen from a blood culture drawn after randomization
- Readmission for subsequent care of indication under study
- Need for additional unplanned source control procedures – e.g., abscess debridement or drainage, cardiac valve replacement
- Change in antibiotic therapy due to inadequate clinical response. For any changes to study drug in the standard of care group, or when new antibiotics are started in either treatment group, the site PI will record the reason for the antibiotic change.

Microbiologic Success: No post-randomization growth (e.g., no positive cultures) of the baseline pathogen from blood cultures or another sterile body site. Subjects who discontinue from the study before Day 42 and have no post-randomization growth will have missing microbiologic success.

4. INVESTIGATIONAL PLAN

4.1. Overall Study Design and Plan

This is a Phase 2b, multicenter, randomized, open-label, assessor-blinded, superiority study to compare dalbavancin to standard of care antibiotic therapy for the completion of therapy in patients with complicated bacteremia or right-sided native valve IE caused by *S. aureus* who have cleared their baseline bacteremia.

Approximately 200 subjects will be randomized 1:1 to receive either dalbavancin or a standard of care antibiotic regimen that is based upon the identification and antibiotic susceptibility pattern of the baseline organism. Those randomized to the dalbavancin treatment group will receive 2 doses of dalbavancin IV 1 week apart (1500 mg on Day 1 and Day 8 after randomization, with renal dose adjustment if appropriate). Those subjects randomized to the standard of care antibiotic therapy treatment group will receive an antibiotic regimen considered to be standard of care based on the methicillin susceptibility pattern of the pathogen isolated at Baseline for a duration of 4 to 6 weeks. The duration of standard of care antibiotics may be extended to a maximum of 8 weeks at the discretion of the treating clinician.

Figure 1 of the protocol provides a schematic of the study design. Study procedures are presented in Section 6 of the study protocol. Detailed descriptions of each study visit can be found in Section 6.3 of the protocol.

The primary outcome measure is the superiority of dalbavancin versus standard of care therapy on Day 70 based on DOOR using the ITT population. Secondary endpoints include comparison of clinical efficacy and clinical safety with clinical efficacy measured by clinical failure, infectious complications, and all-cause mortality while clinical safety is measured by the proportion of SAEs and AEs leading to study drug discontinuation.

4.2. Discussion of Study Design, Including the Choice of Control Groups

The current standard of care for the antibiotic treatment of complicated bacteremia or IE uses a stepwise approach. The initial phase of treatment involves the initiation of empirical antibiotic therapy, definitive diagnosis (as per the modified Duke criteria), and the assessment of the need for early valve replacement, if applicable. Subsequent identification of the causative pathogen, including antibiotic susceptibility and MICs supports the choice of definitive antibiotic therapy and determination of the required duration of antibiotic treatment. Guidelines for the use of outpatient parenteral antibiotic therapy in the treatment of complicated bacteremia or IE similarly advocate that antibiotic therapy can be divided into an initial phase during which life-threatening complications of complicated bacteremia or IE are likely to occur (approximately 14 days) and a completion phase of therapy (2 weeks to 6 weeks) [9].

The proposed clinical study design of dalbavancin in the treatment of complicated bacteremia or IE is consistent with this standard of care. Specifically, prior to study eligibility, patients will receive pre-randomization antibiotic therapy pending a definitive diagnosis of complicated *S. aureus* bacteremia or IE, as well as the resolution of bacteremia. Eligible patients will then be randomized into the study to complete their antibiotic therapy with either a 2-dose regimen of dalbavancin or the current standard of care with daily IV administration of antibiotic therapy for a total duration of 4 to 6 weeks [10] for most patients, and up to 8 weeks for patients with osteomyelitis/discitis.

The proposed clinical study design offers several advantages. First, it will support enrollment of patients with a confirmed diagnosis of complicated bacteremia or IE. Prior studies have been limited by the inability to confidently determine complicated vs uncomplicated status prior to randomization [13]. Second, the proposed study design addresses a true area of need in *S. aureus* bacteremia management and reflects the likely pattern of "real world" dalbavancin use by clinicians for the completion of systemic antibiotic therapy for complicated bacteremia or IE without the need for indwelling IV access to support daily therapy. This takes full advantage of the unusual PK profile of dalbavancin and the introduction of this therapy into clinical practice would potentially have a major impact on patient well-being and QoL. In this study, presence or absence of these potential QoL effects will be assessed using an ARLG Bloodstream Infection QoL Measure developed specifically for this purpose [14 and 15]. Additionally, two previously validated measures (EQ-5D-5L, https://euroqol.org/eq-5d-instruments/sample-demo/ and the PROMIS Global Health short form, http://www.healthmeasures.net/administrator/components/com_instruments/uploads/Global%20Health%20Scale%20v1.2%2008.22.2016.pdf) will also be collected. Third, adequate treatment of complicated bacteremia or IE requires prolonged systemic antibiotic therapy to prevent relapse. Introduction of the 2-dose dalbavancin regimen may decrease the risk of relapse. Finally, the proposed design of this clinical study is consistent with antibiotic stewardship principles, reserving dalbavancin therapy for patients with fully characterized infections and pathogens.

4.3. Selection of Study Population

The study will enroll approximately 200 eligible subjects to the study in the United States and Canada. Eligible subjects are adults 18 years of age or older who have been diagnosed with complicated bacteremia or right-sided IE due to *S. aureus*, have been treated with appropriate empiric/targeted antibiotic therapy, and in whom the blood cultures have tested negative after at least 72 hours of initial antibiotic therapy (maximum 10 days). Subjects with uncomplicated bacteremia due to *S. aureus* will be excluded.

Subject Inclusion and Exclusion Criteria must be confirmed by a study investigator listed on the Form FDA 1572. No exemptions are granted on Subject Inclusion/Exclusion Criteria in DMID-sponsored studies. Clarifications regarding applicability of specific inclusion and exclusion criteria may be discussed with a protocol clinician. Questions about eligibility will also be directed toward the DMID Medical Officer.

4.3.1. Inclusion Criteria

For a list of inclusion criteria, see the most recent version of the Protocol.

4.3.2. Exclusion Criteria

For a list of exclusion criteria, see the most recent version of the Protocol.

4.3.3. Reasons for Withdrawal

Subject Withdrawal:

Subjects may voluntarily withdraw their consent for study participation at any time without penalty or loss of benefits to which they are otherwise entitled.

If a subject withdraws or is withdrawn prior to completion of the study, the reason for this decision must be recorded in the case report forms (CRFs).

The reasons to withdraw from the study might include, but are not limited to, the following:

- Subject no longer meets eligibility criteria
- Subject withdraws consent
- Subject lost to follow-up
- Subject becomes non-compliant
- Medical disease or condition, or new clinical finding(s) for which continued participation, in the opinion of the investigator might compromise the safety of the subject, interfere with the subject's successful completion of this study, or interfere with the evaluation of responses
- Study or site prematurely terminated by the sponsor for any reason

Discontinuation of Treatment:

An investigator may also discontinue a subject from receiving the study product for any reason. Follow-up safety evaluations for discontinued subjects will be conducted if the subject agrees.

The reasons to discontinue study product might include, but are not limited to, the following:

- Subject meets individual halting criteria (see Protocol)
- Subject becomes pregnant, if applicable
- Occurrence of an AE that, in the opinion of the investigator, warrants the subject's permanent discontinuation from IV study drug
- Subject has an insufficient therapeutic response to study drug (i.e., lack of efficacy for SAB). A patient who does not show signs of improvement despite treatment with study drug for an appropriate length of time or a patient who shows signs of clinical worsening at any time may be prematurely discontinued from study drug therapy and treated with salvage therapy as directed by their treating clinician. These patients would continue to be followed in the study unless withdrawn for another reason.

A subject who is prematurely discontinued from study drug or withdrawn from the study should have the assessments for Early Termination (ET) as detailed in the Schedule of Events (Table 1). A clear description of reason for early withdrawal or discontinuation from investigational product must be documented. The reasons for early withdrawal or premature discontinuation from study drug will be reflected on the relevant disposition page of the electronic case report form (eCRF).

The investigator should be explicit regarding study follow-up (e.g., safety and efficacy follow-up) that might be carried out despite the fact the subject will not receive further study product. If the subject consents, every attempt will be made to follow all AEs through resolution. The procedures that collect safety data for the purposes of research must be inclusive in the original informed consent.

The investigator will inform the subject that already collected data will be retained and analyzed even if the subject withdraws from this study.

Subjects who withdraw, or are withdrawn from this study, or are lost to follow-up after signing the informed consent form (ICF) and administration of the study product will not be replaced. Subjects who withdraw, or are withdrawn from this study, or are lost to follow-up after randomization but before administration of the study product will not be replaced.

4.4. Treatments

4.4.1. Treatments Administered

Subjects will be randomized to either receive dalbavancin or standard of care antibiotics. Dalbavancin 1500 mg will be administered IV over 30 (± 10) minutes on Day 1 and Day 8, renally dose-adjusted to 1125 mg for subjects with Creatinine Clearance (CrCl) <30 or not on dialysis.

Standard of care antibiotics will be administered based on the methicillin susceptibility pattern of the pathogen isolated at baseline. Subjects with methicillin-sensitive S. aureus (MSSA) will receive nafcillin (2 g IV q4h × 4-6 weeks) OR oxacillin (2 g IV Q4h x 4-6 weeks) OR cefazolin (2 g IV q8h × 4-6 weeks) while subjects with methicillin-resistant S. aureus (MRSA) will receive vancomycin (dose per local standard of care × 4-6 weeks) OR daptomycin (6-10 mg/kg IV daily × 4-6 weeks).

4.4.2. Identity of Investigational Product(s)

Dalbavancin is a lyophilized, white to off-white to pale yellow solid. It is a lipoglycopeptide synthesized from a fermentation product of *Nonomuraea* species. Dalbavancin is supplied in clear glass vials as a sterile, lyophilized, preservative-free, white to off-white to pale yellow solid. Each vial contains dalbavancin HCl equivalent to 500 mg of dalbavancin.

Investigational dalbavancin vials will be labeled according to manufacturer or regulatory specifications and include the statement "Caution: New Drug – Limited by Federal Law to Investigational Use." The dispensed study product (IV bags) will be labeled with the cautionary statement "For Investigational Use Only."

The standard of care antibiotics (Cefazolin, nafcillin, oxacillin, vancomycin, and daptomycin) will be prepared and labeled in accordance with the clinical site pharmacy's standard operating procedures (SOPs).

4.4.3. Method of Assigning Subjects to Treatment Groups (Randomization)

Once consented and upon entry of demographic data and confirmation of eligibility for the trial, the subject will be enrolled. Enrollment of subjects will be done online using the enrollment module of Advantage eClinical. Subjects will be randomized 1:1 to dalbavancin or standard of care. Randomization will be stratified based on screening pathogen, MSSA vs. MRSA.

The list of randomized treatment assignments will be prepared by statisticians at the Statistical and Data Coordinating Center (SDCC) (The Emmes Company). Emmes will assign each subject a treatment code and treatment assignment from the list after demographic and eligibility data have been entered.

4.4.4. Selection of Doses in the Study

The dalbavancin dosing regimen consists of 1500 mg on Day 1 and 1500 mg on Day 8 for subjects with normal renal function (i.e., CrCl >= 30 mL/min) or who are receiving regular hemodialysis or peritoneal dialysis, administered over 30 minutes by IV infusion. Patients with CrCl <30 mL/min who are not receiving regular hemodialysis or peritoneal dialysis will receive a reduced dose (1125mg on Day 1 and 1125 mg on Day 8). Based on a comparison to the updated nonclinical pharmacokinetic /pharmacodynamics (PD) target of the area under the unbound drug concentration-time curve [fAUC]/MIC ([11]), this regimen is expected to provide sufficient therapeutic concentrations of free drug against *S. aureus* through Day 42.

4.4.5. Blinding

Study subjects and treating physicians will not be masked to treatment, as this is an open label study. Treatment group will be masked for study adjudicators.

4.4.6. Prior and Concomitant Therapy

Medication history during the 30 days prior to ICF signing will be recorded at Screening (Visit 1) in the eCRF. Thereafter, any changes in concomitant medications or new medications added will be recorded in the eCRF.

Any systemic medication taken by the subject, other than study drugs, is considered a concomitant medication. Topical medications including eye drops, ear drops, or dermatologic treatments do not need to be recorded in the eCRFs. All concomitant systemic medications from Screening (Visit 1) through Day 70 ± 7 days (Visit 6) must be recorded in the subject's medical record and on the eCRFs. After Visit 6, for subjects with osteomyelitis, who are followed until Day 180 ± 14 days (Visit 7), the only concomitant medications that must be recorded are new antibiotics that are prescribed for the treatment of osteomyelitis.

At each visit the investigator will obtain information on any therapeutic interventions (e.g., drug and nondrug therapy or surgery) provided. Subjects may not participate in any other antibiotic treatment trials or interventional studies involving non-FDA approved investigational products concomitantly while in this study.

Concomitant systemic antibacterials (other than dalbavancin or comparator study drug) for adjunctive therapy of the subject's *S. aureus* bacteremia are prohibited during the study, up to Day 70. This includes concomitant treatment with an aminoglycoside.

Patients who require additional therapy due to inadequate clinical response will be assessed as having lack of efficacy of study drug.

- Where possible, antibiotic treatment of intercurrent infections should be done with antibiotics that are not active against the patient's *S. aureus* isolate. Discussion with the DMID Medical Officer is encouraged before or within 24 hours of initiation of concomitant antibiotics for another indication. Exceptions include Vancomycin oral 125 mg up to 500mg every 6 hours may be used in both treatment groups for the treatment of *Clostridioides difficile (C. difficile)* infections and may be continued as required throughout the duration of the study. The sponsor will not provide oral vancomycin.
- Metronidazole IV or oral 500 mg every 8 hours may be used in both treatment groups for the treatment of *C. difficile* infections and may be continued as required throughout the duration of the study. The sponsor will not provide metronidazole.
- Other antibacterials that do not achieve therapeutic levels in the serum (e.g., nitrofurantoin) may be considered. Consultation with the DMID Medical Officer is advised before use of these antibiotics.

4.4.7. Treatment Compliance

Dalbavancin will be administered under the supervision of investigative site personnel, and infusion date, start, and stop time will be documented in the eCRF, as well as any infusion interruptions.

4.5. Efficacy and Safety Variables

The primary efficacy variable for this study is DOOR at Day 70. Secondary analyses will analyze DOOR components measured by clinical failure, infectious complications, and AE leading to study drug discontinuation. Quality of life score will be used as a tie-breaker for DOOR analysis.

Safety will be assessed by the frequency of SAEs, AESIs, and the frequency of AEs leading to study drug discontinuation in each treatment group.

5. SAMPLE SIZE CONSIDERATIONS

The study is powered for a superiority comparison based on the primary objective, a comparison of DOOR. The probability of a subject from the dalbavancin arm having a superior DOOR relative to a subject from the standard of care arm will be calculated along with a 95% confidence interval. Superiority will be considered to have been achieved if the 95% confidence interval for probability of having a superior DOOR with dalbavancin does not cross 50%. If the confidence interval crosses 50% however, the null hypothesis cannot be rejected.

Sample size was calculated based on the primary hypothesis. Assuming a 65% probability of a better DOOR in the dalbavancin treatment group versus the standard of care treatment group, with a 90% power and alpha=0.025 (by one-sided Wilcoxon rank sum test), 78 subjects would be required in each treatment group. To allow for some inflation assuming around 12% of missing data or other study imperfections, using the method described in Lachin, et al, [12] we plan to recruit 100 per arm (200 subjects in total). Sample size was calculated using nQuery (MTT1-1 Module) (Version 8, Statistical Solution Ltd).

6. GENERAL STATISTICAL CONSIDERATIONS

6.1. General Principles

All continuous variables will be summarized using the following descriptive statistics: n (non-missing sample size), mean, standard deviation, median, maximum, and minimum. The frequency and percentages (based on the non-missing sample size) of observed levels will be reported for all categorical measures. In general, all data will be listed, sorted by treatment and subject, and when appropriate by visit number within subject. All summary tables will be structured with a column for each treatment group in the following order:

- Dalbavancin
- Standard of Care

All summary tables will be annotated with the total population size relevant to that table/treatment, including any missing observations.

6.2. Timing of Analyses

There will be one planned interim analysis for futility after approximately 50% of subjects have completed the trial. The interim analysis for futility will be performed by the SDCC and will consist of a quantitative evaluation of potential effect sizes and associated precision using a predicted intervals and predicted interval plots (PIPS) approach [7 and 8]. The results of the interim analysis will be presented in the closed session of the Data and Safety Monitoring Board (DSMB).

The DSMB will evaluate safety annually; however, ongoing review and summary of subject safety will occur to allow for early detection of a safety signal that may result from an AE or lack of efficacy of study drug. The DSMB will advise DMID on whether to continue, modify, or terminate the trial based on a risk-benefit assessment.

The final analysis will be performed after database lock.

6.3. Analysis Populations

The primary analysis will be done using the ITT analysis population. Other analyses might use mITT and/or CE analysis populations. Analyses using ITT and mITT will include imputations for missing values using multiple imputation or adjusting for missing data using IPW for DOOR, clinical efficacy, and microbiologic success.

Reasons for exclusion from the screened analysis population are summarized in Table 8 while reasons for exclusions from the rest of the analysis populations (ITT, Safety, mITT, CE) are summarized in Table 4 by treatment group. Individual subject listing of exclusion reasons is also provided in Listing 5. Excluded subjects might satisfy multiple criteria justifying their exclusion but will have only one reason indicated in Table 4 and Listing 5. The exclusion reason indicated will be determined by first exclusion reason met based on the following rules in the order they are listed for each analysis population.

Screened Population Exclusions:

- Subject did not complete the Screening Visit (Visit 1)

ITT Population Exclusions:

- Subject not randomized to receive study product

Safety Population Exclusions:

- Subject not treated with at least one dose of study product

Modified ITT Population Exclusions:

- Subject was excluded from ITT population
- Subject not treated with at least one dose of study product

Clinically Evaluable at Day 42 Population Exclusions:

- Subject was excluded from mITT population
- Subject has missing data or major protocol violation preventing the adjudication committee from evaluating their outcomes at Day 42.

Clinically Evaluable at Day 70 Population Exclusions:

- Subject was excluded from mITT population
- Subject has missing data or major protocol violation preventing the adjudication committee from evaluating their outcomes at Day 70.

6.3.1. Screened Analysis Population

The screened population will consist of all patients who undergo the Screening Visit (Visit 1), signed informed consent, and receive a Patient Identification (PID) number. This population will include both enrolled and not enrolled subjects.

6.3.2. Intent-to-Treat Analysis Population

The intent-to-treat (ITT) population will consist of all randomized patients regardless of whether or not they received study treatment. This analysis population will be used for primary, secondary, and some exploratory analyses. Patients will be analyzed based on the treatment they were randomized to.

6.3.3. Safety Population

The safety population will consist of all randomized patients who received at least 1 dose of study drug. This population will be used for all safety analyses, and patients will be analyzed based on the treatment received.

6.3.4. Modified Intent-to-Treat Population

The modified intent-to-treat (mITT) population will consist of all patients in the ITT population who received at least one dose of study drug. This analysis population will be used for secondary and some exploratory analyses. Patients will be analyzed based on the treatment received.

6.3.5. Clinically Evaluable Analysis Population

The CE populations will consist of all patients in the mITT population who met criteria for clinical evaluability. Patients will be considered clinically evaluable at Day 70 if they have a primary outcome assessment of DOOR at Day 70 and do not have missing data or major protocol violations that prevent the

adjudication committee from evaluating their outcomes at Day 70. Similarly, clinically evaluable at Day 42 if they have a primary outcome assessment of DOOR at Day 42 and do not have missing data or major protocol violations that prevent the adjudication committee from evaluating their outcomes at Day 42. These analysis populations will be used for exploratory analyses and patients will be analyzed based on the treatment received.

6.4. Covariates and Subgroups

Subgroup analyses comparing clinical and microbiologic outcomes will be conducted and will include a) those with MSSA versus MRSA; b) persons who inject drugs (PWID) vs non-PWID; c) those who received infectious disease consultation vs those who did not; d) underlying site of infection (endovascular, bone and joint, skin/skin structure pulmonary); e) subjects with immune-suppression[2] (Yes vs. No); f) duration of initial bacteremia (<2, 2-4, >4 days). Note that, if the number of subjects in a subgroup category is less than 5 for at least one of the treatment groups, that subgroup category will be combined with the next subgroup category with the least number of subjects to form a combined subgroup category with that has at least 5 subjects for at least one of the study groups. However, if a subgroup only has two categories (Yes and No for example), the subgroup category with less than 10 subjects will be excluded from the analyses.

6.5. Missing Data

While all efforts will be made to minimize missing data, some missing data are expected. Whenever possible, subjects terminating from the study early will be given an early termination visit during which the available components of DOOR and related measures can be recorded. The analyses of DOOR, clinical efficacy, and microbiological success for ITT and mITT analyses populations will use Inverse Probability Weighing (IPW) and multiple imputation with linear models to impute values using available information (treatment, randomization strata variables, and available visit information), assuming a missing at random (MAR) model.

The effect that any missing data might have on results will be assessed via sensitivity analysis. If the pattern of missing data are different to that envisaged at the design stage, further sensitivity analyses will be provided that are tailored to the missing data pattern observed.

6.5.1. DOOR Categories

Subjects will be grouped into the five categories based their clinical outcomes. The clinical components of DOOR which include clinical failure, infectious complication, SAE or AE leading to study drug discontinuation will be determined by an independent adjudication committee. DOOR at Day 70 will be defined as follows:

1. If a subject died at any point prior or on to Day 70, then the DOOR at Day 70 will be **Rank 5**.
2. If a subject is alive but has at least one of clinical failure at Day 70, infectious complication, any SAE (except for death), or an AE leading to study drug discontinuation prior or on Day 70, then the DOOR at Day 70 will be **2, 3 or 4**, depending on how many events the subject experienced.

---

2 Defined as: On chemotherapy or immunotherapy for active hematologic malignancy expected to cause ANC < 500 cells/mm3 lasting > 7 days during the study period, chronic high dose oral steroids (equivalent of ≥ 20 mg prednisolone per day for or equivalent, for >2 weeks within the last month), HIV infection with a CD4 cell count < 100 cells/mm3 based on last known measurement or patient-reported value

- Rank 2: Alive but with one of the following: (1) evidence of clinical failure at the specified time point; (2) an infectious complication by the specified time-point; or (3) any SAE, or an AE leading to study drug discontinuation by the specified time-point
- Rank 3: Alive but with two of the following: (1) evidence of clinical failure at the specified time point; (2) an infectious complication by the specified time-point; or (3) any SAE, or an AE leading to study drug discontinuation by the specified time-point
- Rank 4: Alive but with all of the following: (1) evidence of clinical failure at the specified time point; (2) an infectious complication by the specified time-point; or (3) any SAE, or an AE leading to study drug discontinuation by the specified time-point

3. Even if the adjudication committee does not have sufficient evidence to determine clinical failure, if a subject is alive but with any event of infectious complication, any SAE (except for death), or an AE leading to study drug discontinuation by Day 70, then DOOR at Day 70 will be **Rank 3 or 4**, depending on how many events the subject experienced by Day 70.
   - Rank 3: Alive but with one of the following: (2) an infectious complication; or (3) any SAE, or an AE leading to study drug discontinuation
   - Rank 4: Alive but with both of the following: (2) an infectious complication; or (3) any SAE, or an AE leading to study drug discontinuation
4. If a subject is alive (a) with no event of infectious complication, any SAE (except for death), or an AE leading to study drug discontinuation, (b) but the adjudication committee does not have sufficient evidence to determine clinical failure, then the DOOR will be **Rank 2**.
5. If a subject is withdrawn from the study before any assessment (no assessments on any components of DOOR after randomization), then the DOOR will be **missing**.

A similar algorithm will be used to determine DOOR at Day 42.

6.5.2. Quality of Life Score (QoL)

Three types of instruments will be used to assess qualify of life: questions from the PROMIS physical function item bank (PROMIS Item Bank v2.0, short form 6b) on the ARLG Bloodstream Infection QoL Measure, the full PROMIS Global Health short form, and the EQ-5D-5L. For the QoL assessments that utilize PROMIS questions the HealthMeasures Scoring Service (https://assessmentcenter.net/ac_scoringservice) will be used to obtain the final QoL score for each subject for a given item of the quality-of-life instrument and for each assessed timepoint. HeathMeasures is a free publicly available software which requires the user to submit subject responses. No data are stored or saved by this service, and no identifying information is submitted. Even so, as an extra precaution each subject will be assigned a dummy identifier with their corresponding responses. The patient ID assigned by eClinical will not be shared.

A change in QoL from baseline at Day 70 (or Day 42) is calculated by taking the QoL score at Day 70 (or Day 42) minus QoL score at baseline.

- **PROMIS Global Health short form**
  - A value of 5 represents an excellent QoL outcome while a value of 1 represents a poor QoL outcome using the PROMIS Global Health short form.
  - Higher QoL scores represent a better QoL outcome.
  - Higher change in QoL scores from baseline represents a better QoL outcome.

- **ARLG Bloodstream Infection QoL Measure**
  - A value of 5 represents an excellent QoL outcome while a value of 1 represents a poor QoL outcome using the ARLG Bloodstream Infection QoL Measure.
  - Higher QoL scores represent a better QoL outcome.
  - Higher change in QoL scores from baseline represents a better QoL outcome.
- **EQ-5D-5L**
  - A value of 1 represents an excellent QoL outcome while a value of 5 represents a poor QoL outcome using the EQ-5D-5L instrument.
  - Lower QoL scores represent a better QoL outcome
  - Lower change in QoL scores from baseline represents better outcomes.

A subject could have missing data for QoL if a subject missed a visit and/or responses to all the items of the QoL form are missing.

The change in the ARLG Bloodstream Infection QoL from baseline to Day 70 (or Day 42) will be utilized as a tie breaker for DOOR calculations as described in Section 6.5.3 below. Please note that for tie breaking, the standardized score is obtained from the questions arising from the PROMIS physical function item bank (PROMIS Item Bank v2.0, short form 6b) item bank on the ARLG Bloodstream Infection QoL.

6.5.3. Desirability of Outcome Ranking at Day 70 or Day 42

The QoL-adjusted DOOR considers QoL as a tie breaker when calculating the DOOR probability. This is accomplished by using the standardized score from the questions from the PROMIS physical function item bank (PROMIS Item Bank v2.0, short form 6b) item bank on the ARLG Bloodstream Infection QoL (higher QoL is better) as a tie-breaker for subjects with the same DOOR. However, if both subjects have a DOOR of 5, the change in QoL will not be used as a tie-breaker. If change in QoL cannot be calculated due to missing data, we proceed as follows:

- If two subjects have the same DOOR at Day 70 but the QoL is missing for one of the subjects at that timepoint then the subject with missing QoL will be ranked below the subject with non-missing QoL.
- If two subjects have the same DOOR at Day 70 but the QoL is missing for both of the subjects at that timepoint then they will have the same rank and will be indistinguishable for the primary endpoint analysis.

The tie-breaking algorithm to obtain the QoL-Adjusted DOOR from DOOR and change in QoL is implemented as follows. First, subjects are ranked based on their DOOR and then by their change in QoL. Next, the QoL-Adjusted DOOR is calculated from the rank based on the subjects DOOR and the change in QoL. If no two subjects have the same DOOR and change in QoL, then the QoL-Adjusted DOOR is the same as the rank. For subjects with tied DOOR and tied change in QoL the QoL-Adjusted DOOR is calculated as the mean of their ranks. The QoL-Adjusted DOOR, obtained after the tie-breaking algorithm, will be denoted as R_i. This algorithm is exemplified below.

Subject	DOOR	Change in QOL
A	1	10
B	1	-10
C	2	5
D	1	-5
E	2	-5
F	2	5
G	5	NA
H	5	NA

→

DOOR	Subject	Change in QOL	QoL-Adjusted DOOR
1	A	10	1
1	D	-5	2
1	B	-10	3
2	C	5	4.5
2	F	5	4.5
2	E	-5	6
5	G	NA	7.5
5	H	NA	7.5

Since subjects A, D, and B have a DOOR of 1, the change in QOL is used as a tie-breaker among these subjects and the QoL-Adjusted DOOR is 1, 2, and 3 for subjects A, D, and B, respectively. Similarly, since subjects C, F, and E all have a DOOR of 2, the change in QOL is used as a tie-breaker among these 3 subjects. Notice that since subjects C and F have the same change in QOL their QoL-Adjusted DOOR is equal to the mean of their rank.

DOOR at Day 70 without tie-breaking is defined by ranking all subjects (pooling together both treatment group) according to their DOOR at Day 70 (lower is more desirable) ignoring their change in QoL score.

The QoL-Adjusted DOOR at Day 42 is calculated similarly using the change in QoL at Day 42.

6.6. Interim Analyses and Data Monitoring

A single interim analysis will be performed after approximately 50% of subjects have completed the trial. The interim analysis for futility will consist of a quantitative evaluation of potential effect sizes and associated precision using a predicted intervals and PIPS approach. Briefly, predicted intervals for both primary DOOR and secondary clinical failure outcomes will be modeled under a range of assumptions including: 1) the trends observed at interim analysis continue to end of study, 2) the null hypothesis is true (i.e., the DOOR distributions are identical between treatment groups), and 3) the worst-case scenario as an alternative outcome. By relying on prediction intervals, no statistical hypothesis testing is required, and no power is lost at interim analysis. Details of interim analysis, including best and worst case scenarios for alternative outcomes are provided in Section 8.5.

6.7. Multicenter Studies

This is a multicenter study, but randomization is not stratified by site. Data will be pooled across all clinical sites and analyses will not adjust for potential site effects.

6.8. Multiple Comparisons/Multiplicity

Only one hypothesis test will be performed for the primary analysis. Secondary and exploratory analyses will not be corrected for multiplicity.

7. STUDY SUBJECTS

7.1. Disposition of Subjects

Reasons for screening failures will be summarized in Table 8. The completion status and reasons for early termination or treatment discontinuation by Day 42 will be summarized (Table 3 and Listing 2) for each treatment group and pooled across treatment groups for the ITT population. A subject could be discontinued early due to an adverse event (AE) (serious or non-serious), loss to follow-up, non-compliance with study, voluntary withdrawal, withdrawal at the investigator request, termination of the site by the sponsor, termination of the study by the sponsor, death, lack of eligibility at enrollment, inadequate clinical response, or becoming ineligible after enrollment. Number and percentage of subjects completing each visit will be presented in Table 3 by treatment group for all randomized subjects.

Subject disposition and eligibility for analysis will be summarized in a CONSORT flow diagram (Figure 1).

7.2. Protocol Deviations

A summary of subject-specific protocol deviations will be presented by the reason for the deviation, the deviation category, and treatment group for all subjects (Table 2 and Listing 3). Non-subject specific protocol deviations will be in Listing 4. All subject-specific protocol deviations and non-subject specific protocol deviations will be presented. Major protocol deviations preventing the adjudication committee from evaluating the outcome will be determined by the adjudication committee.

RESTRICTED

8. EFFICACY EVALUATION

All efficacy variables will be listed by subject. Data will be summarized by treatment group. Continuous efficacy variables will be summarized with the number of observations, mean, median, standard deviation, minimum, and maximum. Categorical efficacy variables will be summarized by number and percent in each category.

All statistical tests are two-sided and performed at the α=0.05 significance level; all confidence intervals are two-sided with 95% confidence level.

8.1. Primary Efficacy Analysis

The primary efficacy endpoint is DOOR assessed at Day 70 post study entry (TOC) performed on the ITT analysis population.

8.1.1. Analysis of DOOR at Day 70 Using ITT Analysis Population

DOOR at Day 70 is defined in Section 6.5.3 with and without tie-breaking. The primary analysis in this section is based on the QoL-Adjusted DOOR, which is calculated after using QoL as a tie-breaker.

The null and alternative hypotheses corresponding to the primary analysis of this study are:

H_0: $Pr[DOOR_D > DOOR_C] + ½ Pr[DOOR_D = DOOR_C] \leq 50\%$ (i.e., no difference in DOOR at Day 70).

H_1: $Pr[DOOR_D > DOOR_C] + ½ Pr[DOOR_D = DOOR_C] > 50\%$ (i.e., difference in DOOR at Day 70).

where $DOOR_D$ and $DOOR_C$ are the QoL-Adjusted DOOR for dalbavancin and control, or standard of care, groups, respectively, and $Pr[DOOR_D > DOOR_C]$ is the probability of a DOOR from dalbavancin being more desirable than a DOOR from standard care and $Pr[DOOR_D = DOOR_C]$ is the probability of two DOOR being the same.

$Pr[DOOR_D > DOOR_C] + ½ Pr[DOOR_D = DOOR_C]$ will be referred to as the DOOR probability throughout this SAP.

The superiority of Dalbavancin vs Standard of Care is concluded if the lower bound of the 95% CI for the DOOR probability is larger than 50%.

Due to the presence of missing data, IPW and multiple imputation (MI) will be used to handle missing data. For the primary analysis of DOOR, IPW method will be used. MI described in Section 8.1.1.2 will be used for sensitivity analysis.

8.1.1.1. ITT Analysis of DOOR using IPW

ITT analysis requires that all randomized subjects be included in the analysis. However, missing data are prone to happen in clinical trials due to missing scheduled visits or loss to follow up for example. In this case, analysis that is only based on complete data may be biased if the excluded subjects are systematically different from those included. IPW is one approach commonly used to reduce this bias under a missing at random (MAR) assumption. This is achieved by weighting complete cases with the inverse of their probability of being a complete case. While subjects missing the DOOR are excluded from the analysis per se, they may still inform the fitting of the logistic regression model used to provide predictions of the probability of completeness which are used to calculate the weights.

Specifically, let Y_i represent the outcome of interest (QoL-Adjusted DOOR; see Section 6.5.3), X_i represent the covariates of interest, Z_i represent any other variables measured in the data but not used in the analysis model. For the IPW approach, we first define the missingness model to estimate the weights (w_i) using a logistic regression model with outcome L and covariates taken from set (X, Z) where L_i is defined as 1 if DOOR data are complete (not missing) and 0 otherwise. Through this model, we obtain the fitted probabilities of each subject being complete, denoted as π_i. Note that as described in Section 6.5.3, the QoL-Adjusted DOOR is calculated by accounting for tied DOORs using QoL as a tie-breaker for primary analysis.

The DOOR probability can then be calculated using the QoL-Adjusted DOOR at Day 70 as the outcome following the algorithm below:

1. Name QoL-Adjusted DOOR from the group that received standard of care antibiotics "sample 1" and the QoL-Adjusted DOOR from the group that received dalbavancin "sample 2". Rename weights from sample 1 as w_{1i} and weights from sample 2 as w_{2i}.
2. For each observation in sample 2. If an observation in sample 2 has a smaller QoL-Adjusted DOOR than an observation in sample 1, then that observation in sample 2 gets an indicator of value of 1. Else if the observation in sample 2 is equal to the observation in sample 1, the observation in sample 2 gets an indicator value of ½.
3. Let π_{1i} be the fitted probability of being complete for each observation in sample 1 and π_{2i} be the fitted probability for being complete for each observation in sample 2 obtained from the logistic regression model with an indicator for having non-missing QoL-Adjusted DOOR as the outcome.
4. For each pair in step 2, create the weight w_j as the inverse of the probability of both values in the pair being non-missing, i.e., $w_j = 1/ (\pi_{1i} \text{ x } \pi_{2i})$ with j being the index for observations in sample 2
5. For each pair in step 2, create the weighted indicator value as $w\_ind_j = w_j \text{ x } indicator_j$
6. Repeat step 2 through 5 for all observations in sample 2.
7. The DOOR probability can then be obtained by $DOOR\_prob\_IPW$ = weighted average of all the indicator values in step 5, i.e., $DOOR\_prob\_IPW = \frac{\sum_j w\_ind_j}{\sum w_j}$,

The DOOR probability (i.e. Pr(Desirable DOOR in dalbavancin) + 0.5 Pr(Equal DOOR)) using IPW is given by the value in *DOOR_prob_IPW*.

To estimate the 95% confidence intervals for the DOOR probability, the approach discussed in Halperin et al. after incorporating IPW weights [2]. The superiority of Dalbavancin vs Standard of Care is concluded if the lower bound of the 95% CI for the DOOR probability is larger than 50%.

Pseudocode for Missingness model to estimate fitted probabilities and IPW weights:

Define complete as 1 for complete and 0 for missing and trt=1 for Standard of care and 2 for Dalbavancin.

```
proc logistic data = dat;
  model complete (event='1') = [trt baseline_pathogen age infection_site
bacteremia_duration ];
  output out = out1 p = probs xbeta = logit;
run;
proc transpose data=out1 out=out2;by patid;id trt; var probs;run;
data out2;
      set out2;
      wts=1/(probs_dalba * probs_soc);
run;
```

8.1.1.2. ITT Analysis of DOOR using Multiple Imputation

As a sensitivity analysis, multiple imputation with a linear model to impute missing DOOR at Day 70 will be used. Details of multiple imputation methods are described in Section 8.6.1.

For each of the 20 complete multiple imputation datasets, a DOOR probability, estimated by the Wilcoxon Mann-Whitney U Statistic corrected for ties, will be computed using randomization to dalbavancin versus randomization to standard of care therapy to define the binary grouping and DOOR at Day 70 as the outcome. The U statistics are asymptotically normally distributed, and so they can be combined into a single test statistic using Rubin's Rules [1].

Defining the following:

n_1: number of subjects in ITT population randomized to standard of care

n_2: number of subjects in ITT population randomized to dalbavancin

m: number of imputed datasets ($m = 20$)

Q_i: U statistic computed from the ith multiply imputed dataset

$\bar{Q} = \frac{1}{m}\sum_{i=1}^{m} Q_i$

Q_0: the expected value of a U statistic under the null hypothesis ($Q_0 = \frac{n_1 n_2}{2}$)

U_i: The variance from the ith multiply imputed dataset (this is not the U statistic). Correcting for ties, the formula for the variance of the Mann-Whitney U statistic, as described in Halperin et al. [2], is:

$$U_i = Var(Q_i) = \frac{1}{n_1 n_2}[n_1 + n_2 + 1 - (n_1 + n_2 - 2)\theta]\zeta(1-\zeta)$$

Where

$$\zeta = \frac{1}{n_1 n_2} Q_i$$

$$\theta = \frac{[(n_1 + n_2 - 2)\zeta - (n_2 - 1)A - (n_1 - 1)B]}{(m + n - 2)\zeta(1-\zeta)}$$

To obtain an estimator $\hat{\theta}$ of θ, use the following formulas for A and B, respectively:

$$A = A_1 - \frac{1}{n_2 - 1}\sum_{i=1}^{D-1} p_{1i}\left[q_{2i}\sum_{j=i+1}^{D} p_{2j} - \left(\sum_{j=i+1}^{D} p_{2j}\right)^2\right] - \frac{1}{4(n_2 - 1)}\sum_{i=1}^{D} p_{1i}p_{2i}q_{2i}$$

Where

$$A_1 = \sum_{i=1}^{D-1} p_{1i}\left[\sum_{j=i+1}^{D} p_{2j} + \frac{p_{2i}}{2}\right]^2 + \frac{p_{1D}p_{2D}^2}{4}$$

And

$$B = B_1 - \frac{1}{n_1 - 1}\sum_{j=2}^{D} p_{2j}\left[q_{1j}\sum_{i=1}^{j-1} p_{1i} - \left(\sum_{i=1}^{j-1} p_{1i}\right)^2\right] - \frac{1}{4(n_1 - 1)}\sum_{j=1}^{D} p_{2i}p_{1i}q_{1i}$$

Where

$$B_1 = \sum_{j=2}^{D} p_{2j}\left[\sum_{i=1}^{j-1} p_{1i} + \frac{p_{1j}}{2}\right]^2 + \frac{p_{11}^2 p_{21}}{4}$$

In the equations for A and B above, D is the number of distinct values of DOOR in the dataset; p_{1i}, for i = 1, 2, …, D, represents the proportion of subjects randomized to standard of care with the ith value of DOOR; p_{2j}, for j = 1, 2, … D, represents the proportion of subjects randomized to dalbavancin with the jth value of DOOR; and q = 1 – p in general.

Additionally, the equation for an unbiased estimate of $\zeta(1-\zeta)$ is given by:

$$\frac{(n_1 n_2 - n_1 - n_2 + 2)\zeta - n_1 n_2 \zeta^2}{(n_1 - 1)(n_2 - 1)} + \frac{A}{n_1 - 1} + \frac{B}{n_2 - 1}$$

After substituting the values of A, B and $\zeta(1-\zeta)$ in the equation θ to obtain an estimate $\hat{\theta}$ of θ, define θ as follows: If $\hat{\theta} < 0$ then $\theta = 0$, if $\hat{\theta} > 1$ then $\theta = 1$, otherwise $\hat{\theta} = \theta$.

$\bar{U} = \frac{1}{m}\sum_{i=1}^{m} U_i$ (The within imputation variance. This is not the mean of the U statistics.)

$B = \frac{1}{m-1}\sum_{i=1}^{m}(Q_i - \bar{Q})^2$

$T = \bar{U} + \frac{m+1}{m}B$

$W = \frac{(\bar{Q}-Q_0)^2}{T}$

$r = \frac{m+1}{m}\frac{B}{\bar{U}}$

$$\nu = (m-1)\left(1 + \frac{1}{r}\right)^2$$

As a sensitivity analysis, a 95% CI for U will be computed using the overall test statistic W through the inversion of the F-test. Dividing the bounds of this CI by $n_1 n_2$ will yield the bounds for the 95% CI of the DOOR probability. Thus, the CI for DOOR probability is given by:

$$95\%\text{ CI: } \left(\frac{\bar{Q} - \sqrt{T \times F_{0.95,1,\nu}}}{n_1 n_2}, \frac{\bar{Q} + \sqrt{T \times F_{0.95,1,\nu}}}{n_1 n_2}\right)$$

A point estimate of the DOOR probability will be obtained by dividing $\bar{Q}$ by $n_1 n_2$. Results will be shown in Table 20 for ITT population with and without QoL as a tie-breaker. This analysis will be repeated in the mITT population and results will be reported in Table 21 with and without QoL as a tie-breaker. A listing of DOOR and its components is provided in Listing 10. A listing of investigator assessments of clinical success and infectious complications are provided in Listing 11 and Listing 12, respectively.

8.2. Secondary Efficacy Analyses

8.2.1. Analysis of Clinical Efficacy at Day 70 using IPW for the ITT and mITT Analysis Populations

Let Y_i represent the outcome of interest (clinical efficacy), *X* represent the covariates of interest, *Z* represent any other variables measured in the data but not used in the analysis model. For the IPW approach, we first define the missingness model to estimate the weights (w_i) using a logistic regression model with outcome *R* and covariates taken from set (X, Z) where R_i is defined as 1 if is complete (not missing clinical success) and 0 otherwise. Through this model, we obtain the fitted probabilities of being complete, denoted as π_i. Weights w_i are then obtained by the inverse of the fitted probabilities of being complete ($w_i = 1/\pi_i$).

To estimate the proportions of clinical efficacy, we will fit a linear regression model with treatment as a covariate adjusting for IPW weights ($Y_i = \beta_0 + \beta_1 trt + \epsilon_i$, where $\epsilon_i \sim N(0, w_i\sigma^2)$

The difference in proportions of clinical efficacy for dalbavancin compared to standard of care ($p_d - p_s$) will then be obtained by β_1. The rate of clinical efficacy for dalbavancin will be estimated as $p_d = \beta_0 + \beta_1$ and the rate of clinical efficacy for standard of care will be obtained by $p_s = \beta_0$. The two-sided 95% CI for the proportions of clinical efficacy and difference in proportions in clinical efficacy will use estimates from the linear regression with IPW.

Proportions of clinical efficacy for each treatment group along with their 95% CI, a point estimate of the difference in proportions of clinical efficacy at Day 70 along with 95% CI obtained from linear regression with multiple imputation model as described above will be provided in Table 35 for Day 70 using the ITT analysis population. These analyses will be repeated for the mITT analysis population and results will also be provided in Table 36.

The null hypothesis will be rejected and non-inferiority of dalbavancin versus standard of care with respect to clinical efficacy at Day 70 using a non-inferiority margin of 20% will be concluded if the lower bound of the 95% CI for the difference in proportions of clinical efficacy for dalbavancin relative to standard of care is greater than -20%.

Subgroup analyses of clinical efficacy at Day 70 will be performed for the ITT and mITT populations using IPW for different clinically important subgroups including a) those with MSSA versus MRSA; b) persons who inject drugs (PWID) vs non-PWID; c) those who received infectious disease consultation vs those who did not; d) underlying site of infection (endovascular, bone and joint, skin, pulmonary); e) subjects with immune-suppression; f) divided by duration of initial bacteremia, in the ITT, mITT, and CE populations and results will be reported in Table 38 for ITT and Table 39 for mITT analysis population. Proportions and difference in proportions of clinical efficacy along with their 95% CI estimated from linear regression model following multiple imputation will be reported.

Pseudocode:

Missingness model to estimate fitted probabilities and IPW weights:

Define complete as 1 for complete and 0 for missing and trt=1 for standard of care and 2 for Dalbavancin.

```
proc logistic data = dat;
  model complete (event='1') = [age sex trt and other covariates that affect
completeness of the data];
  output out = out1 p = probs xbeta = logit;
run;
data out1;
      set out1;
      wts=1/probs;
run;
```

Final model:

```
proc glm data=out1;
      class trt(ref='1');
      model clinsuccess= trt solution;
      weight wts;
      lsmeans TRTPN/ pdiff=all tdiff cl stderr;
run;
```

8.2.2. Sensitivity Analysis of Clinical Efficacy at Day 70 using Multiple Imputation for the ITT and mITT Analysis Populations

Clinical efficacy at Day 70 is defined as absence of clinical failure, infectious complications, and all-cause mortality. A subject will be defined as not having clinical efficacy of at least one of these three components occurred. This endpoint will be analyzed using a non-inferiority approach. The corresponding hypotheses are:

Null hypothesis: $\pi_{\text{dalbavancin}} - \pi_{\text{standard of care}} \leq$ -20%,

Alternative hypothesis(non-inferiority): $\pi_{\text{dalbavancin}} - \pi_{\text{standard of care}} >$ -20%,

where π represents the probability of clinical efficacy at study Day 70. 20% is the non-inferiority margin used for this study and is also used for all secondary non-inferiority analyses of clinical efficacy.

The non-inferiority of dalbavancin versus standard of care with respect to clinical efficacy using a non-inferiority margin of 20%, will be determined for the ITT analysis population using a two-sided 95% CI of the difference in proportions of clinical efficacy as constructed using multiple imputation of clinical efficacy with linear regression. A lower bound of the CI greater than -20% will result in the conclusion of non-inferiority of dalbavancin. The imputation model will utilize available information collected at baseline and any completed study visits.

The secondary analysis will use multiple imputation with a linear regression model without rounding to impute missing values of clinical efficacy at Day 70 using the ITT population [3 and 4].

Although the linear regression without rounding can sometimes yield implausible imputed values of treatment success, Horton et.al [4] showed that this method yields an unbiased estimate of the binomial proportion.

- Let $Y_1, Y_2, \ldots, Y_N$ be independent and identically distributed (iid) Bernouilli random variables
- Let $p = E(Y_i)$ be the probability of success

- Assume that only n out of the N Bernoulli data points are observed; the rest are missing. For simplicity, assume Let $Y_1, Y_2, \dots, Y_n$ are observed and Let $Y_{n+1}, \dots, Y_N$ are missing. Further assume that data are Missing Completely at Random (MCAR).

For estimating p, the minimum variance unbiased estimate (MVUE) of p denoted by $\hat{p}$ which is simply the mean of observed data, i.e.,

$$\hat{p} = \frac{1}{n}\sum_{i=1}^{n} Y_i$$

Rubin and Schenker [5] proposed using a full normal imputation method to impute missing values $Y_{n+1}, \dots, Y_N$ which assumes that the Y_i are iid from normal distribution with mean p and variance σ^2. This method follows the following algorithm to generate the missing values.

This full normal imputation method without rounding incorrectly assumes a normal distribution and can sometimes yield implausible imputed values (above 1 or below 0). However, it produces an unbiased estimate of the probability of success p.

Allison [3] showed that this approach can be extended to allow covariates in the model. Hence, using simulation studies, Allison showed that multiple imputation using linear regression performed well in estimating regression coefficients in different missing data scenarios (MCAR, MAR) even when compared to logistic regression. The added benefit of using the linear regression model is that it directly provides proportion differences along with their 95% CI after applying PROC MIANALYZE to the model fits from the M multiply imputed datasets. This approach will follow the three steps described below:

Step 1: A multiple regression model: $Y_i = \beta_0 + \beta_1 X_1 + \beta_2 X_2 + \beta_3 X_3 + \cdots + \beta_c X_c + \epsilon_i \quad, \epsilon_i \sim N(0, \sigma^2)$ where Y_i represents the indicator for clinical efficacy and X_c are the covariates described in Section 8.6.2 to be used in the multiple imputation model to generate *M* multiply imputed datasets.

Step 2: A linear regression model $Y_i = \beta_0 + \beta_1 trt + \epsilon_i$ will be fit on each of the *M* multiply imputed datasets with *trt* defined as 0 for standard of care and 1 for dalbavancin.

Step 3: The final clinical efficacy estimates will be obtained by combining *M* estimates of clinical efficacy estimates using PROC MIANALYZE as described in in Section 8.6.2. The rate of clinical efficacy for dalbavancin will be estimated as $p_d = \beta_0 + \beta_1$ and the rate of clinical efficacy for standard of care will be obtained by $p_s = \beta_0$. The difference in proportions of clinical efficacy for dalbavancin compared to standard of care $(p_d - p_s)$ will then be obtained by β_1. The two-sided 95% CI for the proportions of clinical efficacy and difference in proportions in clinical efficacy will use estimates from the linear regression with multiple imputation.

Proportions of clinical efficacy for each treatment group along with their 95% CI, a point estimate of the difference in proportions of clinical efficacy at Day 70 along with 95% CI obtained from linear regression with multiple imputation model as described above will be provided in Table 35 for Day 70 using the ITT analysis population. These analyses will be repeated for the mITT analysis population and results will also be provided in Table 35.

The null hypothesis will be rejected and non-inferiority of dalbavancin versus standard of care with respect to clinical efficacy at Day 70 using a non-inferiority margin of 20% will be concluded if the lower bound of the 95% CI for the difference in proportions of clinical efficacy for dalbavancin relative to standard of care is greater than -20%.

An individual listing of observed values of clinical efficacy components is provided in Listing 10.

8.2.3. Analysis of DOOR Components Using ITT Analysis Population

Results for the analysis of DOOR components at Day 70 will be presented. Proportions of subjects with clinical failure, infectious complications, SAEs & AEs leading to study drug discontinuation, and all-cause mortality at Day 70 will be reported in Table 42 by treatment group. Note that this analysis will consider subjects in the ITT population who have non-missing values for the corresponding DOOR component. The DOOR component will be summarized by the DOOR probability and 95% confidence interval (computed as the probability that a randomly selected patient will have a better DOOR if assigned to the intervention arm using the Wilcoxon-Mann-Whitney statistic corrected for ties). A summary of DOOR by component will be presented at Day 70 for the ITT population in Table 47. The DOOR probability will also be presented graphically using forest plots in Figure 2. These analyses will be repeated for Day 42, and analogous results will be reported in Table 45, Table 48, and Figure 5, respectively.

8.2.4. Analysis of Clinical Failure at Day 70 Using ITT Population

Clinical failure at Day 70 will be analyzed using weighted generalized estimating equations (GEE) assuming an unstructured correlation structure, including clinical failure at Day 42. The difference in proportions of clinical failure between the two groups at Day 70 will be calculated with the corresponding 95% confidence interval (Table 49). The GEE model will use subject random effects with an unstructured correlation structure to generate the average difference in proportions of clinical failure for dalbavancin compared to standard of care at Day 70 and IPW weights will be obtained using a similar approach as that described in Section 8.2.1 using the following pseudocode:

```
Weighted GEE:
proc gee data=temp descending;
class id trt clinfailure time/param=ref
missmodel trt time trt*time / type=obslevel; /* missingness model */
model clinfailure= trt time trt*time; /* marginal model */
repeated subject=id/ corr=un;
    estimate 'trt 1 at time=2' intercept 1 trt 1 time 2 trt*time 2;
    estimate 'trt 0 at time=2' intercept 1 time 2;
    estimate 'trt 1 vs 0 at time=2' trt 1 trt*time 2;
run;
```

where temp is a dataset with one row per timepoint per subject (one row for time=1 and another row for time=2 per subject). Clinfailure is a binary variable with a value of 1 indicating clinical failure and a value of 0 indicating clinical success, trt is a binary variable with a value of 1 indicating dalbavancin and a value of 0 indicating standard of care, id indicates each subject identifier, and time is binary variable with a value of 1 indicating Day 42 and a value of 2 indicating a Day 70 timepoint.

A linear model was used in this scenario since the outcome of interest is difference in proportions instead of odds ratios. The difference in proportions will be given by the sum of coefficient for the treatment and the coefficient for treatment*time interaction and will be interpreted as ‘on average, the proportion clinical failure at Day 70 is increased (or decreased if the sign if negative) by xx amount when taking dalbavancin compared to standard of care’.

As a sensitivity analysis, generalized linear mixed model (GLMM) approach will be used to analyze clinical failure (Table 49) to model individual treatment differences for dalbavancin compared to standard of care using the following pseudocode:

```
proc mixed data=temp;
     class id;
     weight wts;
     model clinfailure=trt time trt*time;
     repeated/ subject=id type=un;
    estimate 'trt 1 at time=2' intercept 1 trt 1 time 1 trt*time 1;
    estimate 'trt 0 at time=2' intercept 1 time 1;
    estimate 'trt 1 vs 0 at time=2' trt 1 trt*time 1;

run;
```

Note that wts will be calculated from a similar missingness logistic regression model as that used for the weighted GEE model above.

Similarly, to GEE, the difference in proportions using GLMM will be given by the sum of coefficient for the treatment and the coefficient for treatment*time interaction. This treatment difference will be interpreted as 'for a specific individual, the proportion clinical failure at Day 70 is increased (or decreased if the sign if negative) by xx amount when taking dalbavancin compared to standard of care'.

8.3. Exploratory Efficacy Analyses

8.3.1. Analysis of Clinical Efficacy at Day 42

Clinical efficacy at Day 42 will be analyzed using a similar approach as that described in Section 8.2.1 using IPW and as described in Section 8.2.2 using multiple imputation for the ITT and mITT analysis populations, and results will be reported in Table 36.

Subgroup analyses of clinical efficacy at Day 42 will be performed for the ITT and mITT populations using IPW for different clinically important subgroups including a) those with MSSA versus MRSA; b) persons who inject drugs (PWID) vs non-PWID; c) those who received infectious disease consultation vs those who did not; d) underlying site of infection (endovascular, bone and joint, skin, pulmonary); e) divided by duration of initial bacteremia and results will be reported in Table 38 for ITT and Table 39 for mITT analysis population. Proportions and difference in proportions of clinical efficacy along with their 95% CI estimated from linear regression model following multiple imputation will be reported.

8.3.2. Additional Analyses of DOOR

8.3.2.1. Analysis of DOOR at Day 42 Using ITT and mITT Analysis Populations

The analysis of DOOR at Day 42 for the ITT and mITT analysis populations will be performed in an analogous manner as those described in Section 8.1.1.1 for the primary analysis using IPW and as described in Section 8.1.1.2 using multiple imputation and results will be reported in Table 22 for the ITT population and Table 23 for the mITT population.

8.3.2.2. Subgroup Analyses of DOOR

Subgroup analysis of DOOR will be performed using subjects in each of the following subgroups a) those with MSSA versus MRSA; b) persons who inject drugs (PWID) vs non-PWID; c) those who received infectious disease consultation vs those who did not; d) underlying site of infection (endovascular, bone and joint, skin, pulmonary); e) subjects with immune-suppression; f) divided by duration of initial bacteremia, in the ITT, mITT, and CE populations for Day 42 and Day 70. Analyses for the ITT and mITT will use a similar approach as that described in Section 8.1.1.1 using IPW and results will be reported in Table 25 for ITT

population and Table 26 for mITT population. Subgroup analysis of DOOR for the CE population will use the approach described in Section 8.3.3.2 for complete data and results will be reported in Table 27. Forest plots for DOOR probabilities are also presented for the ITT analysis population in Figure 20 for Day 70 and Figure 21 for Day 42.

8.3.3. Analysis of DOOR Components at Day 42 and Day 70 Using mITT Analysis Populations

Results for the analysis of DOOR components at Day 70 will be presented. Proportions of subjects with clinical failure, infectious complications, SAEs, AEs leading to study drug discontinuation, and all-cause mortality at Day 70 will be reported in Table 42 for each treatment group using mITT population. The DOOR component will be summarized by the DOOR probability and 95% confidence interval. The DOOR probability will also be presented graphically using forest plots in Figure 3 for the mITT population. This analysis will be repeated for Day 42, and results with be reported in Table 45 and Figure 6.

8.3.3.1. Analysis of DOOR Categories at Day 42 and Day 70 Using ITT and mITT Analysis Populations

The number and percent of subjects along with 95% CI for the percentages computed using the Wilson method in each DOOR category will be presented in Table 28, Table 29, and Table 30 for the ITT, mITT, and CE analysis populations, respectively. The percentage of subjects in each DOOR category will also be presented graphically in Figure 8, Figure 9, Figure 10 for Day 70 and Figure 11, Figure 12, Figure 13 for Day 42. The percentage of subjects in each DOOR category is also presented per subgroup for Day 70 using the ITT analysis populations starting with Figure 14 through Figure 19.

8.3.3.2. Distribution of DOOR by Treatment Group Using ITT and mITT Analysis Populations

The distribution of DOOR by treatment group will be presented by number and percentage of by Subgroup Categories for the ITT analysis population in Table 55 for Day 42 and Table 56 for Day 70.

8.3.3.3. Cumulative Difference in DOOR Categories Using ITT and mITT Analysis Populations

Cumulative DOOR probability as well as 95% CI will be provided in Table 31 for Day 42 and Day 70 Using the ITT analysis population. The Wilcoxon-Mann-Whitney statistic corrected for ties will be used as an estimate of the cumulative DOOR probability. Analogous results will be presented for the mITT population in Table 32. These results will also be presented graphically in Figure 22.

8.3.3.4. Analysis of Difference in Mean Partial Credit Using ITT and mITT Analysis Populations

The partial credit will be calculated assigning a partial credit score to each of the DOOR categories. For this analysis, DOOR categories 3 (alive with two events) and 4 (alive with three events) will be combined such that DOOR will be analyzed using a 4-category ranking. QoL is not considered for analysis of partial credit score. The difference in mean partial credit score will be summarized in Figure 23.

8.3.3.5. Analysis of Expected DOOR Category Distribution and Expected Numbers Gained Loss Using ITT and mITT Analysis Populations

The expected number for the DOOR category will be calculated by multiplying the proportion of subjects in that DOOR category by 1000 separately for each treatment group. The gained loss for each DOOR category will be calculated by taking the difference in expected number for Dalbavancin minus the expected number for standard of care. The total gained loss will be obtained by summing up all the gained losses for all the

DOOR categories (i.e., add up all the differences in expected numbers for dalbavancin minus standard of care) and results will be reported in Table 34 for Day 42 and 70 for ITT and mITT analysis populations.

8.3.4. Analysis of Microbiological Success at Day 42 and Day 70 using the ITT and mITT Analysis Populations

Microbiological success at Day 42 is defined as the absence of a post-randomization growth (i.e., no positive cultures) of the baseline pathogen from blood cultures drawn post randomization or from another sterile body site until Day 42. Subjects who will still be in the study at Day 42 and have no post randomization cultures will be considered as having microbiological success. Subjects who are lost to follow up by Day 42 and have no post-randomization blood cultures will have missing microbiologic success. Due to the potential of having missing values of microbiologic success in the ITT and mITT, microbiologic success at Day 42 will be analyzed using a similar approach as that described in Section 8.2.1 using IPW and in Section 8.2.2 using multiple imputation for the ITT and mITT analysis populations and results will be reported in Table 50. These analyses will also be repeated for Day 70 and results will be reported in Table 50 for ITT and mITT analysis populations. Subgroup analyses of microbiologic success will be performed for the ITT and mITT populations using IPW for different clinically important subgroups including a) those with MSSA versus MRSA; b) persons who inject drugs (PWID) vs non-PWID; c) those who received infectious disease consultation vs those who did not; d) underlying site of infection (endovascular, bone and joint, skin, pulmonary); e) subjects with immune-suppression; f) divided by duration of initial bacteremia and results will be reported in Table 52 for ITT and Table 53 for mITT analysis population. Proportion and difference in proportions of microbiologic success along with their 95% CI estimated from linear regression model with IPW will be reported.

An individual listing of observed values of microbiologic success is provided in Listing 10 and a listing of culture results used to define microbiologic success is provided in Listing 13.

8.3.5. Analysis of QoL Score using ITT and mITT Analysis Populations

Summary statistics (number of subjects, mean, standard deviation, minimum, maximum) for QoL scores and change from baseline of QoL scores obtained from the ARLG Bloodstream Infection QoL Measure at Day 42 and Day 70 will be provided for each analysis population in Table 57. Similarly, summary statistics for QoL scores from the EQ-5D-5L instrument and PROMIS Global Health Short Form will be provided in Table 58 and Table 59, respectively. A listing of QoL data from the three instruments is provided in Listing 14.

Additionally, descriptive statistics of QoL measures will also be presented by item.

8.3.6. Bivariate Analysis of DOOR Probability vs Difference in Mean of Change in QoL Score from Baseline

In addition to the DOOR analyses described in Section 8.1.1 and Section 8.4.1, a two-dimensional analysis of the DOOR probability and difference in the mean change in QoL from baseline in the two treatment groups using the ARLG Bloodstream Infection QoL Measure will also be performed at Day 70. Only subjects with non-missing DOOR and change in QoL from baseline will be included in these analyses. The horizontal axis is the DOOR probability (probability of a more desirable DOOR category when assigned to Dalbavancin vs. Standard of care) based on the DOOR categories without using mean change in QoL from baseline as a tie-breaker. The vertical axis is the difference in the means of the observed difference in mean change in QoL from baseline (dalbavancin minus standard of care). A result in the upper right quadrant represents more desirable results for dalbavancin group, while a result in the lower left represents more desirable standard of care. The other two quadrants represent tradeoffs for clinical outcomes and QoL score.

1000 bootstrap samples will be generated by resampling with replacement from the empirical distribution of DOOR and change in QoL from baseline at Day 70. The DOOR probability and mean difference in the mean change in QoL from baseline will be estimated using the bootstrap samples and plotted as a scatter plot. A 95% joint region of DOOR probability and difference in mean change in QoL from baseline will be constructed first using a parametric method. The parametric method will estimate the confidence region using the ellipse method from the CAR R package which uses a bivariate normal distribution. The 90%, 95%, and 99% confidence regions will be reported. These results will be reported in Figure 24 for Day 70 and Figure 25 for Day 42 using the ITT analysis population. Subjects in the ITT analysis population with missing values of DOOR categories and change in QoL from baseline will be excluded from this analysis.

8.3.7. Analysis of Late Recurrence Within ITT Population with Osteomyelitis

The osteomyelitis population includes all randomized subjects diagnosed with osteomyelitis. Subjects in this population will have an extra follow-up visit approximately 6 months after randomization to evaluate long term recurrence risk. Late recurrence within the osteomyelitis population will be defined by the presence of the following up to 6 months after randomization: progressive imaging changes along with isolation of *S. aureus* from blood, bone biopsy, associated fluid aspiration, or operative tissue.

Protocol amendment version 3.0 expanded Visit 7 (Day 180) to include all subjects with osteomyelitis, instead of only those with vertebral osteomyelitis at baseline (version 2.0). When possible, any subjects who were not originally eligible for Visit 7 under protocol version 2.0, but who were eligible under protocol version 3.0 were re-consented and data was collected. Thus, in addition to the planned analysis, which includes all subjects for whom Visit 7 consent and data were obtained, an additional sensitivity analysis will be conducted that excludes subjects enrolled under protocol version 2.0 who were not eligible prior to protocol version 3.0. That is, the planned analyses will be performed in the following groups:

1. All subjects for whom Visit 7 consent and data was obtained (either at the time of enrollment or retroactively following protocol version 3.0), and
2. Subjects who enrolled after protocol version 3.0 or later (i.e., excluding subjects those enrolled initially under protocol version 2.0 that re-consented under protocol version 3.0).

Note that no eligible subjects were enrolled under protocol version 1.0. If no data are available for subjects who were re-consented the sensitivity analysis will be excluded.

Number and percentage of subjects in each of the osteomyelitis populations above will be presented in Table 62 by treatment group. Difference in proportions along with their 95% CI will also be reported.

8.4. Supplemental Efficacy Analyses

All efficacy analyses performed using the CE analysis population will be considered supplemental.

8.4.1. Analysis of DOOR at Day 42 and 70 Using CE Analysis Populations

Additional analyses of DOOR at Day 42 and Day 70 will be performed using only subjects in the CE population with complete data. These analyses will evaluate the null hypotheses described above using the DOOR probability Pr(Desirable DOOR in dalbavancin) + 0.5 Pr(Equal DOOR), estimated by Wilcoxon-Mann-Whitney Statistic corrected for ties, divided by the product of the two group sample sizes and corresponding CIs calculated by the method described in Halperin et al. [2]. The methods described in Section 8.1.1.2 will be implemented for this analysis.

Results from the analysis using CE analysis population with complete data will be reported in Table 24 for both Day 42 and Day 70.

8.4.2. Analysis of Clinical Efficacy at Day 70 Using CE Analysis Population

The CE analysis populations at Day 70 will have no missing values of clinical efficacy, therefore the analysis of clinical efficacy at Day 70 will be performed using a linear regression without multiple imputation with treatment group as a covariate ($Y_i = \beta_0 + \beta_1 trt_i + \epsilon_i$) for subjects in the CE analysis population at Day 70. The rate of clinical efficacy in the dalbavancin arm will then be estimated by $p_d = \beta_0 + \beta_1$ and the rate of clinical efficacy in the standard of care arm will be provided by $p_s = \beta_0$.

The estimate for the difference in proportions of clinical efficacy will be provided by β_1 and its 95% CI will be calculated first by using the 95% CI for β_1 from the linear regression model above. As a sensitivity analysis, the 95% CI for the difference in proportions of clinical efficacy will be recalculated using the Miettinen–Nurminen method from PROC FREQ with RISKDIFF (CL= MN) in SAS.

Proportions of clinical efficacy for each treatment group, a point estimate of the difference in proportions of clinical improvement at Day 70 along with their 95% CIs obtained methods described above will be provided in Table 37 using the CE analysis population at Day 70. If the lower bound of the 95% CI for the difference in proportions of clinical efficacy is greater than -20%, it will be annotated by a footnote b.

Results from the subgroup analysis of clinical efficacy at Day 70 using CE population will be reported in Table 40.

Proportions and difference in proportions of clinical efficacy along with their 95% CI estimated from linear regression model without imputation will be reported for the CE analysis population.

Clinical efficacy will also be analyzed for the CE analysis population at Day 42 using a similar approach as that used for Day 70, and results will be reported in Table 37. Results from the subgroup analysis of clinical efficacy at Day 42 using CE population will also be reported in Table 40.

8.4.3. Analysis of DOOR at Day 42 and Day 70 Using CE Analysis Population

Analyses described in Section 8.3.3 will be performed using the CE analysis population. Proportions of subjects with clinical failure, infectious complications, SAEs, AEs leading to study drug discontinuation, and all-cause mortality at Day 70 will be reported in Table 43 using the CE population. The DOOR component will be summarized by the DOOR probability and 95% confidence interval. The DOOR probability will also be presented graphically using forest plots in Figure 4 for the CE population. This analysis will be repeated for Day 42 and results with be reported in Table 46 and Figure 7.

8.4.3.1. Cumulative Difference in DOOR Using CE Analysis Population

Cumulative DOOR probability as well as 95% CI will be provided in Table 33 for Day 42 and Day 70 Using the CE analysis population. The Wilcoxon-Mann-Whitney statistic corrected for ties will be used as an estimate of the cumulative DOOR probability. These results will also be presented graphically in Figure 22.

8.4.3.2. Analysis of Difference in Mean Partial Credit Using CE Analysis Population

Analysis of mean partial credit scores described in Section 8.3.3.4 will be repeated using the CE analysis population and results will provided in Figure 22.

8.4.3.3. Analysis of Expected DOOR Distribution and Expected Numbers Gained Loss

Analysis of expected DOOR distribution and expected gained loss described in Section 8.3.3.4 will be repeated for the CE analysis population and results will be reported in Table 34.

8.4.4. Analysis of Microbiological Success at Day 42 and Day 70 using the CE Analysis Populations

Analysis of microbiological success described in Section 8.3.4 will be repeated using the CE analysis population at Day 42 and Day 70. Results will be presented in Table 51. Subgroup analysis of microbiological success at Day 42 and Day 70 will also be performed using the CE population and results will be presented in Table 54.

8.5. Interim Analyses

Predictive intervals and predictive interval plots will be generated for DOOR and clinical efficacy under a range of assumptions including: 1) the trends observed at interim analysis continue to end of study, 2) the null hypothesis is true (i.e., the DOOR distributions are identical between treatment groups), and 3) and the worst-case scenario as an alternative outcome. These predicted interval plots provide a prediction of the trial results were the trial to continue as planned under varying assumptions regarding future data (e.g., current trend continues, null hypothesis is true, and worst-case scenario). For example, using then current trend, the remaining subjects will be simulated assuming outcomes continue to occur at the rates observed at the time of interim analysis. For each assumption, 10,000 complete datasets (N=200 subjects) will be simulated and used to calculate the probability of rejecting the null hypothesis for each scenario. By relying on prediction intervals, no statistical hypothesis testing is required, and no power is lost at interim analysis [7 and 8]. Note interim results will only be presented in the DSMB closed session.

Suppose the following distribution of DOOR is observed at the interim analysis, i.e. $\hat{p}_{D1}$ is the proportion of subjects in the Dalbavancin group observed to have a DOOR of 1 (alive with no events).

DOOR	Dalbavancin	Standard of Care
1 – Alive with no events	$\hat{p}_{D1}$	$\hat{p}_{C1}$
2 – Alive with 1 event	$\hat{p}_{D2}$	$\hat{p}_{C2}$
3 – Alive with 2 events	$\hat{p}_{D3}$	$\hat{p}_{C3}$
4 – Alive with 3 events	$\hat{p}_{D4}$	$\hat{p}_{C4}$
5 – Death	$\hat{p}_{D5}$	$\hat{p}_{C5}$

Let p_{Dk} and p_{Ck} denote the proportions of subjects in the Dalbavancin and Standard of Care groups with DOOR = *k,* for *k* = 1, 2, …, 5, used to simulate the predicted data.

- Under scenario 1, the trend observed at the interim continues, we assume that $p_{Dk} = \hat{p}_{Dk}$ and $p_{Ck} = \hat{p}_{Ck}$ for *k* = 1, 2, …, 5.
- Under scenario 2, the null hypothesis is true, we assume $p_{Dk} = p_{Ck} = \bar{p}_k = \frac{n_d}{n_d + n_c}\hat{p}_{Dk} + \frac{n_c}{n_d + n_c}\hat{p}_{Ck}$; where n_d is the number of subjects in the Dalbavancin group and n_c is the number of subjects in the standard of care arm.
- Under scenario 3, the worst-case scenario as an alternative outcome, we assume that $p_{Dk} = \hat{p}_{Ck}$ and $p_{Ck} = \hat{p}_{Dk}$ if the Standard of Care group is better at the interim analysis (as determined by DOOR probability).

Summary of PIPs statistics are presented in Table 60 and Figure 26 for DOOR at Day 70 and in Table 61 and Figure 27 for clinical efficacy at Day 70 using the ITT analysis population. Other tables and figures from the main analyses will also be presented in the interim report and are indicated via implementation notes in this SAP.

8.6. Imputation of Missing Data

8.6.1. Multiple Imputation of Missing DOOR Day 42 and Day 70

Several analyses depend on multiple imputation of DOOR at Day 70 and Day 42 for ITT and mITT analysis populations. First, a table showing the number and percentage of missing data for DOOR on Day 42 and Day 70 will be presented in Table 18 for ITT and Table 19 for mITT analysis population. In order to use the multiple imputation model to adjust for bias caused by missing data, we assume that data are missing at random (MAR).

Multiple imputations of each of these missing endpoints will be performed independently, and each subject will have their missing endpoints imputed independently of other subject's imputations using a subject-specific imputation model.

Before performing multiple imputation, an ordered list of variables to include in the subject-specific imputation model is constructed. Ordering is specified so that exact imputation results from final data are prespecified may be replicated in SAS (using seeds described below). The complete ordered list of variables for the imputation models for DOOR at Day 70 is below.

- Indicator of dalbavancin as study treatment (binary indicator, standard of care is the reference group)
- Baseline pathogen (binary indicator, MRSA is the reference group)
- Age at enrollment
- Site of infection
- Duration of bacteremia
- Observed DOOR category at Day 70

For DOOR at Day 42, the complete list of model variables is identical to the above with Day 70 being replaced by Day 42.

The actual list of model variables for each subject-specific imputation model will follow the ordering above but omit variables with missing values. The below pseudo-code / SAS code outlines the creation of 20 multiple imputation datasets. Note that the seeds used in the actual analysis must follow the specification given in the pseudo-code and subjects must be processed in the order described in the pseudo-code. The pseudo-code is in terms of Day 70 endpoints, but the general logic is also applicable to the Day 42 endpoints (with references to "D70" replaced with references to "D42").

```
DEFINE i=index variable for subjects having DOOR imputed.
      Subjects requiring imputation are sorted in ascending order
      by PATID.
DEFINE N=number of subjects requiring imputation
DEFINE g&i=analysis dataset containing predictors and DOOR for Subjects with complete
       DOOR at D70 as well as subject i (only one subject with missing data are
       included).  Note that subjects with complete DOOR that are missing a value for
       one or more variables in the subject-specific imputation model are excluded.
```

```
DEFINE imp_g&i = g&i, with 20 imputed values for the missing DOOR
      added by PROC MI
DEFINE &&modelVars_&i  = list of observed variables in subject i, to
      be used for imputation of DOOR.
%do i=1 %to &N;
PROC MI data=g&i out=imp_g&i seed=500&i NIMPUTE=20 noprint;
      var &&modelVars_&i DOOR;
      monotone reg(DOOR_D70 = &&modelVars_&i);
run;
%end;
imp_g&i will be subset to contain only rows for the subjects with imputed DOOR and
merged together and with subjects with complete data to create the twenty complete
multiply imputed datasets
```

8.6.2. Multiple Imputation (MI) of Missing Values of Clinical Efficacy and Microbiologic Success on Day 42 and Day 70

Secondary and exploratory analyses of clinical efficacy and microbiologic success for ITT and mITT populations depend on multiple imputation. First, a table showing the number and percentage of missing data for clinical efficacy and microbiologic success on Day 42 and Day 70 will be presented in Table 18 for ITT and Table 19 for mITT analysis population. In order to use the MI model to adjust for bias caused by missing data, we assume that data are missing at random (MAR).

For missing clinical efficacy on Day 42 and Day 70, multiple imputations of missing clinical efficacy on Day 42 and Day 70 will be performed independently, and each subject will have their missing clinical efficacy imputed independently of other subjects' imputations using a subject-specific imputation model. The pseudocode shown below details how missing data for clinical efficacy for Day 70 (Day 42) will be imputed using *m* multiply imputed datasets from linear models. The following covariates will be used non-missing for the MI model: treatment group, baseline pathogen, age, gender. The number of imputed, *m*, datasets will be chosen based on the average percent of missing data. Default value will be *m*=20 since sample size calculation assumed close to 20% drop-out rate.

As a first step to multiple imputation, an ordered list of variables to include in the subject-specific imputation model is constructed. Ordering is specified so that exact imputation results from final data are prespecified may be replicated in SAS (using seeds described below). The complete ordered list of variables for the imputation models for clinical improvement is below:

- Indicator of dalbavancin as study treatment (binary indicator, standard of care is the reference group
- Baseline pathogen (binary indicator, MRSA is the reference group)
- Age at enrollment
- Site of infection
- Duration of bacteremia
- Observed DOOR

The actual list of MI model variables for each subject-specific imputation model will follow the ordering above but omit variables with missing values. The below pseudo-code / SAS code outlines the creation of 20 multiple imputation datasets. Note that the seeds used in the actual analysis must follow the specification

given in the pseudo-code, and subjects must be processed in the order described in the pseudo-code. The pseudo-code is for Day 70, but the general logic is also applicable to Day 42.

```
*************************************************************
```

*Outcome variables: clinefficacy_D70

DEFINE i=index variable for subjects having clinical efficacy imputed.

Subjects requiring imputation are sorted in ascending order by PATID.

DEFINE N=number of subjects requiring imputation

DEFINE g&i=analysis dataset containing predictors and clinical efficacy for subjects with non-missing efficacy at Day 70 as well as subject i (only one subject with missing clinical efficacy is included). Note that subjects with complete clinical efficacy at Day 70 that are missing a value for one or more variables in the subject-specific imputation model are excluded.

DEFINE imp_g&i = g&i, with 20 imputed values for the missing clinical efficacy

added by PROC MI

DEFINE &&modelVars_&i = list of observed variables in subject i to be used for imputation of clinical efficacy

Step 1: Imputation model: This model will generate 20 datasets with each dataset containing original complete data along with imputed values for subjects with missing endpoint.

```
%do i=1 %to &N;
PROC MI data= g&i out= imp_g&i seed= 22131&i NIMPUTE=20 noprint;
     Var &&modelVars_&i clinefficacy_D70;
     monotone reg(clinefficacy_D70 = &&modelVars_&i;
run;
%end;
```

imp_g&i will be subset to contain only rows for the subjects with imputed clinical improvement and merged together and with ATP-5 data to create the twenty complete multiply imputed datasets

Step 2: Analysis model: This model will fit regression models to the 20 complete datasets to obtain parameter estimates for treatment success, clinical cure, microbiological success.

```
proc reg data= imp_g outest= out_clineff_D70 covout noprint;
model clinefficacy_D70= trt /clb alpha=0.05;
by _imputation_;
run;
```

Step 3: Combine estimates from models in step 2 to obtain overall estimates summarized over 20 imputed datasets;

```
proc mianalyze data= out_clineff_D70 alpha = 0.05;
modeleffects intercept trt;
ods output ParameterEstimates=parms_trts;
run;
*************************************************************
```

The multiple imputation model for microbiologic success will use a similar algorithm and imputation variables as the clinical efficacy model described above.

9. SAFETY EVALUATION

9.1. Demographic and Other Baseline Characteristics

Summaries of race, ethnicity, sex, age, weight, height, body mass index (BMI), baseline pathogen, baseline QoL, will be presented for the ITT population by site (Table 9 and Table 13) and by treatment group (Table 11 and Table 15). Similar tables will be presented for the Safety population by site in Table 10 and Table 14 and by treatment group in Table 12 and Table 16. Age, weight, height, baseline QoL, and BMI will be summarized as continuous variables. The baseline QoL standardized score is obtained from the questions arising from the PROMIS physical function item bank (PROMIS Item Bank v2.0, short form 6b) item bank on the ARLG Bloodstream Infection QoL. Ethnicity will be categorized as Hispanic or Latino, or not Hispanic and not Latino. In accordance with NIH reporting policy, subjects may self-designate as belonging to more than one race or may refuse to identify a race, the latter reflected in the case report form (CRF) as "No" to each racial option. For subjects that were previously enrolled in this study, only information associated with their second enrollment will be reported and used for analysis.

Summaries of subject's medical history will be presented by MedDRA® V24.1 or higher system organ class (SOC) and treatment group (Table 17).

Individual subject listings will be presented for all demographics and baseline characteristics (Listing 6),pre-existing medical conditions (Listing 7), and investigator assessment of baseline *S. aureus* bacteremia diagnoses (Listing 8).

9.1.1. Prior and Concurrent Medical Conditions

Number and percentage of subjects' pre-existing and concurrent medical conditions will be presented by MedDRA system organ class and treatment group for the ITT population (Table 17).

Individual subject listings will be presented for all pre-existing medical conditions (Listing 7).

9.1.2. Prior or Concomitant Medications and Nondrug Interventions

Prior medication is defined as any medication taken before the date of the first dose of investigational product. Concomitant medication is defined as any medication started on or after the date of the first dose of investigational product. Concomitant medications will be coded to the Anatomical Therapeutic Classification using the WHO Drug Dictionary.

Both prior and concomitant medication use will be summarized by the number and proportion of subjects in each treatment group receiving each medication within each therapeutic class (ATC1 and ATC2) for the safety population in Table 107. If a subject took a specific medication multiple times or took multiple medications within a specific therapeutic class, that subject would be counted only once for the coded drug name or therapeutic class. A summary of subjects taking nondrug interventions by SOC is provided in Table 108.

Individual subject listings will be presented for all concomitant medications (Listing 21). A listing of nondrug interventions is provided in Listing 22.

9.2. Measurements of Treatment Compliance

Dates of first treatment will be summarized by site and treatment group in Table 5. Exposure to investigational product for the safety population will be summarized for treatment duration calculated as the number of doses of dalbavancin received for patients in the dalbavancin group. Number and percentage of subjects in the dalbavancin group receiving 1 or 2 doses of dalbavancin will be reported in Table 6. For subjects in the standard of care group, the duration of antibiotics will be calculated using the start and stop dates for standard of care antibiotics. Duration of antibiotics will be calculated using three different start dates: (1) date of first positive blood culture, (2) date of first negative blood culture, and (3) date of randomization. The stop date will be the latest end date recorded for each antibiotic. Descriptive statistics for each duration of standard of care antibiotics (n, mean, standard deviation, minimum, median, and maximum) will be presented in Table 7. A listing of individual subjects' treatment duration is provided in Listing 9. A listing of all subjects who took at least one dose of study product is provided in Listing 1.

9.3. Adverse Events

Safety analyses will be based on the safety population. Safety will be assessed using descriptive statistics of AEs, SAEs, AESIs, AEs leading to study discontinuation, vital signs, and laboratory tests by treatment group. For each safety parameter, the last assessment made before the first dose of investigational product will be used as the baseline for all analyses of that safety parameter.

When calculating the incidence of adverse events (i.e., on a per subject basis), each subject will only be counted once and any repetitions of adverse events within a subject will be ignored; the denominator will be the number of subjects in the safety population. All adverse events reported will be included in the summaries and analyses. Safety analyses will be based on the safety population. Safety analyses encompass the component events to the DOOR calculation.

9.3.1. Treatment-Emergent Adverse Events

An AE (classified by preferred term) that occurs during the treatment period will be considered a treatment-emergent AE if it was not present before the first dose of investigational product or was present before the first dose of investigational product and increased in severity during the treatment period.

A summary of all treatment-emergent AEs in each treatment group will be tabulated by MedDRA SOC, preferred term (PT), and relationship to the investigational product in Table 65 and by MedDRA system organ class, high level group term (HLGT), and relationship to the investigational product in Table 67. A listing of all treatment-emergent AEs will be presented in Listing 15.

A summary of all treatment-emergent AEs will be tabulated by MedDRA SOC, PT, maximum severity, and causal relationship to the investigational product in Table 66. If more than one AE is coded to the same PT for the same subject, the subject will be counted only once for that PT using the most severe and most related occurrence for the summarization by severity and by causal relationship to the investigational product. Similarly, a summary of all treatment-emergent AEs will be tabulated by MedDRA, SOC, HLGT, maximum severity, and causal relationship to the investigational product in Table 68. If more than one AE is coded to the same HLGT for the same subject, the subject will be counted only once for that HLGT using the most severe and most related occurrence for the summarization by severity and by causal relationship to the investigational product.

The distribution of treatment-emergent AEs by severity and causal relationship to the investigational product will be summarized by treatment group in Table 69.

The distribution of AESIs by severity and causal relationship to the investigational product will be summarized by treatment group in Table 70.

The incidence of common (≥ 2% of patients in any treatment group) treatment-emergent AEs, on-therapy SAEs, AESIs, and AEs leading to premature discontinuation of the investigational product will be summarized by PT and treatment group and will be sorted by decreasing frequency for the investigational product Table 71. In addition, the incidence of fatal on-therapy SAEs (i.e., events that caused death) will be summarized separately by treatment group and PT in Table 72 and by treatment group and HLGT in Table 73. An SAE will be defined as an on-therapy SAE if it occurred during or after the first infusion of investigational product.

Bar charts of all adverse events will be presented by MedDRA SOC, severity and treatment group in Figure 28 and by MedDRA HLGT, severity, and treatment group in Figure 29. Forest plots of differences in risks of experiencing adverse events will be presented by SOC in Figure 30 and by HLGT in Figure 31. The 95% confidence intervals for difference in proportions will be computed using the Miettinen-Nurminen method.

9.4. Deaths, Serious Adverse Events, and Other Significant Adverse Events

Detailed narratives will be given for any deaths, SAEs, AESIs, and AEs leading to study product discontinuation that occurred during the study. Listings will include Subject IDs, AE description, AE onset date/end date, relationship to treatment, alternate etiology if not related, outcome, and duration of event (days).

Listings for SAEs, AESIs, subjects with AEs leading to discontinuation, and subjects who die (if any) will be presented in Table 74, Table 75, Table 76, and Table 77, respectively. A listing of all treatment-emergent AEs is presented in Table 78.

The number and percentage of subjects reporting SAEs, AEs Leading Discontinuation, Grade 3 or Higher AEs, AESIs, and ALT/AST Elevations will be reported by treatment group in Table 63 for the safety population by study arm and in Table 64 for standard of care antibiotics.

9.5. Pregnancies

For any subject in the Safety population who became pregnant during the study, every attempt will be made to follow these subjects to completion of pregnancy to document the outcome, including information regarding any complications with pregnancy and/or delivery. Listing 25, Listing 26, Listing 27, Listing 28, and Listing 29 will present any study pregnancies and their outcomes.

9.6. Clinical Laboratory Evaluations

Descriptive statistics for clinical laboratory values and changes from the baseline values at each assessment time point will be presented by treatment group for each clinical laboratory parameter starting with Table 82 and ending with Table 95 for serum chemistry parameters and starting with Table 97 and ending with Table 104 for hematology parameters. Forest plots of difference in risks of experiencing abnormal clinical laboratory events by clinical laboratory parameters will be presented in Figure 32 for hematology parameters and in Figure 33 for serum chemistry parameters. The 95% confidence intervals for difference in proportions will be computed using the Miettinen-Nurminen method.

The number and percentage of subjects with potentially clinically significant (PCS) post-baseline clinical laboratory values will be tabulated by treatment group in Table 81 for serum chemistry parameters and

Table 96 for hematology parameters. The criteria for PCS laboratory values will be detailed in the table footnotes. The percentages will be calculated relative to the number of subjects with available non-PCS baseline values and at least 1 post-baseline assessment. The numerator will be the total number of subjects with available non-PCS baseline values and at least 1 PCS post-baseline value. A supportive listing of subjects with PCS post-baseline values will be provided, including the PID number, study center number, and baseline and post-baseline values. A listing of PCS chemistry and hematology laboratory results is provided in Table 79 and Table 80, respectively. A listing of all AEs that occur in subjects who have PCS laboratory values or vital signs will also be provided in Listing 16. Individual laboratory results are provided in Listing 17 for chemistry laboratory parameters and in Listing 18 for hematology laboratory parameters.

9.7. Vital Signs and Physical Evaluations

Vital signs including pulse rate, systolic blood pressure (BP), diastolic BP, respiratory rate, and temperature will be collected at each visit from Visit 1 through Visit 7 and at the early termination visit. Descriptive statistics for vital sign values and changes from baseline will be presented at each timepoint by treatment group in Table 105 for vital signs values and Table 106 for changes from baseline. Individual vital signs measurements will be provided in Listing 19.

Targeted physical examinations will be performed, if indicated, based on a subject's medical history. A listing of physical exam findings will be presented in Listing 20. Echocardiogram will be performed if it has not already been done as part of standard of care for this episode of bacteremia/endocarditis. Either a transthoracic or transesophageal echocardiogram is acceptable, and results of this echocardiogram will be provided in Listing 23. A listing of all subject hospitalizations will be presented in Listing 24.

9.8. Concomitant Medications and Nondrug Interventions

Concomitant medications will be collected for the 30 days prior to ICF signing through Day 70 Visit. Concomitant medications will be coded to the Anatomical Therapeutic Classification using the WHO Drug Dictionary. The use of prior and concomitant medications taken during the study will be recorded on the CRFs. A by-subject listing of concomitant medication use will be presented (Listing 21). A listing of non-drug interventions will be presented in Listing 22. The use of concomitant medications during the study will be summarized by ATC1, ATC2 code, and study treatment for the Safety population (Table 107). A summary of subjects taking nondrug interventions by SOC is provided in Table 108.

10. PHARMACOKINETICS

Analysis of PK endpoints will be provided in separate analysis plan document.

11. OTHER ANALYSES

No other analyses are planned.

12. REPORTING CONVENTIONS

For the primary and secondary analyses percentages and probabilities will be reported to one decimal place. P-values ≥0.001 and ≤0.999 will be reported to 3 decimal places; p-values less than 0.001 will be reported as "<0.001"; p-values greater than 0.999 will be reported as "> 0.999". The mean, standard deviation, and any other statistics other than quantiles, will be reported to one decimal place greater than the original data. Quantiles, such as median, or minimum and maximum will use the same number of decimal places as the original data. Proportions will be presented as two decimal places; values <0.01 will be presented as "<0.01". Percentages will be reported to the nearest whole number; values < 1% will be presented as "<1" and values > 99% but below 100% will be presented as ">99". Estimated parameters, not on the same scale as raw observations (e.g., regression coefficients) will be reported to 3 significant figures.

13. TECHNICAL DETAILS

SAS version 9.4 or above or R version 3.2 or above will be used to perform analyses and to generate all tables, figures and listings.

14. SUMMARY OF CHANGES IN THE CONDUCT OF THE STUDY OR PLANNED ANALYSES

No changes in the conduct of the study or planned analysis.

15. REFERENCES

1. Marshall, A., Altman, D.G., Holder, R.L. and Royston, P., 2009. Combining estimates of interest in prognostic modelling studies after multiple imputation: current practice and guidelines. BMC medical research methodology, 9(1), p.1.
2. Halperin, M., Hamdy, M.I., and Thall, P.F. Distribution-free confidence intervals for a parameter of Wilcoxon-Mann-Whitney type for ordered categories and progressive censoring. Biometrics. 1989 Jun;45(2):509-21. PMID: 2765635.
3. Allison, P. (2005). Imputation of categorical variables with PROC MI.
4. Horton, N.J., Lipsitz, S.R., and Parzen, M. (2003) A potential for bias when rounding in multiple imputation. American Statistician 57: 229-232.
5. Rubin, D. B., and Schenker, N. (1986). Multiple Imputation for Interval Estimation from Simple Random Samples with Ignorable Nonresponse. Journal of the American Statistical Association, 81, 366–374.
6. Miettinen, O., and Nurminen, M.M. Comparative analysis of two rates. Statistics in Medicine 4 2 (1985): 213-26.
7. Evans, S., Li, L. and Wei, L. Data monitoring in clinical trials using prediction. Drug Inform Journal 41, 73-742 (2007).
8. Li, L., Evans, S., Uno, H., and Wei, L. A graphical tool for data monitoring in clinical trials. Statistical Biopharmaceutical Research. 2009; 1:348-355.
9. Holland TL, Raad I, Boucher HW, et al. Effect of Algorithm-Based Therapy vs Usual Care on Clinical Success and Serious Adverse Events in Patients with Staphylococcal Bacteremia: A Randomized Clinical Trial. JAMA. 2018;320(12):1249-1258.
10. Baddour LM, Wilson WR, Bayer AS, et al. Infective Endocarditis in Adults: Diagnosis, Antimicrobial Therapy, and Management of Complications: A Scientific Statement for Healthcare Professionals from the American Heart Association. Circulation.2015;132(15):1435-1486
11. Lepak A, Marchillo K, VanHecker J, and Andes D. Impact of Glycopeptide Resistance in *Staphylococcus aureus* on the Dalbavancin In Vivo Pharmacodynamic Target. Antimicrob Agents Chemother. 2015;59(12):7833-7836.
12. Lachin JL. Introduction to Sample Size Determination and Power Analysis for Clinical Trials. Controlled Clinical Trials 198; 2:93-113
13. Corey GR. Staphylococcus aureus bloodstream infections: definitions and treatment. Clin Infect Dis. 2009;48 Suppl 4: S254-259.
14. King HA, Doernberg SB, Miller J, et al. Patients' experiences with Staphylococcus aureus and Gram-negative bacterial bloodstream infections: A qualitative descriptive study and concept elicitation phase to inform measurement of patient-reported quality of life. Clin infect Dis 2021;73(2):237-247.
15. King HA, Doernberg SB, Grover K, et al. Patients experiences with Staphylococcus aureus and Gram-negative bacterial bloodstream infections: results from cognitive interviews to inform assessment of health-related quality of life. Open Forum Infect Dis 2021;9(2): ofab622

16. LISTING OF TABLES, FIGURES, AND LISTINGS

Table, figure, and listing shells are presented in Appendices 1, 2, and 3.

APPENDICES

APPENDIX 1. TABLE MOCK-UPS

LIST OF TABLES

Table 1: Schedule of Study Procedures 64
Table 2: Distribution of Protocol Deviations by Category, Type, and Treatment Group – ITT Population....66
Table 3: Subject Disposition by Treatment Group 67
Table 4: Analysis Populations by Treatment Group – ITT Population 68
Table 5: Dates of First Treatment by Site and Treatment Group 69
Table 6: Treatment Compliance for the Dalbavancin Treatment Group – Safety Population....70
Table 7: Treatment Compliance in the Standard of Care Treatment Group Measured by Number of Days – Safety Population 71
Table 8: Ineligibility Summary of Screen Failures....72
Table 9: Summary of Categorical Demographic and Baseline Characteristics by Site – ITT Population....73
Table 10: Summary of Categorical Demographic and Baseline Characteristics by Site – Safety Population....73
Table 11: Summary of Categorical Demographic and Baseline Characteristics by Treatment Group – ITT Population 74
Table 12: Summary of Categorical Demographic and Baseline Characteristics by Treatment Group – Safety Population 75
Table 13: Summary of Continuous Demographic and Baseline Characteristics by Site – ITT Population....76
Table 14: Summary of Continuous Demographic and Baseline Characteristics by Site – Safety Population....76
Table 15: Summary of Continuous Demographic and Baseline Characteristics by Treatment Group – ITT Population 77
Table 16: Summary of Continuous Demographic and Baseline Characteristics by Treatment Group – Safety Population 78
Table 17: Summary of Subjects with Pre-Existing Medical Conditions by MedDRA System Organ Class and Treatment Group – ITT Population....79
Table 18: Percentage of Subjects with Missing Data by Study Endpoint, Timepoint, and Treatment Group – ITT Analysis Population 80
Table 19: Percentage of Subjects with Missing Data by Study Endpoint, Timepoint, and Treatment Group – mITT Analysis Population 80

Table 20: Analysis of DOOR at Day 70 – ITT Analysis Population..81
Table 21: Analysis of DOOR at Day 70 – mITT Analysis Population 81
Table 22: Analysis of DOOR at Day 42 – ITT Analysis Population..82
Table 23: Analysis of DOOR at Day 42 – mITT Analysis Population 82
Table 24: Analysis of DOOR at Day 42 and Day 70 – CE Analysis Population 83
Table 25: Subgroup Analysis of DOOR with Tie-Breaking by Timepoint – ITT Analysis Population 84
Table 26: Subgroup Analysis of DOOR with Tie-Breaking by Timepoint – mITT Analysis Population 85
Table 27: Subgroup Analysis of DOOR with Tie-Breaking by Timepoint – CE Analysis Population 86
Table 28: Analysis of DOOR Categories – ITT Analysis Population 87
Table 29: Analysis of DOOR Categories – mITT Analysis Population....................................87
Table 30: Analysis of DOOR Categories – CE Analysis Population 87
Table 31: Cumulative Proportions of DOOR - ITT Analysis Population 88
Table 32: Cumulative Proportions of DOOR – mITT Analysis Population..............................88
Table 33: Cumulative Proportions of DOOR – CE Analysis Population.................................88
Table 34: Summary of Expected Numbers Gained Loss at Day 42 and Day 70 by Analysis Population 89
Table 35: Analysis of Clinical Efficacy at Day 70 Using ITT and mITT Analysis Populations 90
Table 36: Analysis of Clinical Efficacy at Day 42 Using ITT and mITT Analysis Populations 90
Table 37: Analysis of Clinical Efficacy at Day 42 and Day 70 Using CE Analysis Population 91
Table 38: Subgroup Analysis of Clinical Efficacy by Timepoint – ITT Analysis Population 92
Table 39: Subgroup Analysis of Clinical Efficacy by Timepoint – mITT Analysis Population 93
Table 40: Subgroup Analysis of Clinical Efficacy by Timepoint – CE Analysis Population 94
Table 41: Summary of Clinical DOOR Components at Day 70 – ITT Analysis Population 95
Table 42: Summary of Clinical DOOR Components at Day 70 – mITT Analysis Population 95

Table 43: Summary of Clinical DOOR Components at Day 70 – CE Analysis Population 95
Table 44: Summary of Clinical DOOR Components at Day 42 – ITT Analysis Population 95
Table 45: Summary of Clinical DOOR Components at Day 42 – mITT Analysis Population 95
Table 46: Summary of Clinical DOOR Components at Day 42 – CE Analysis Population 95
Table 47: Summary of DOOR by Component at Day 70 – ITT Analysis Population 96
Table 48: Summary of DOOR by Component at Day 42 – ITT Analysis Population 96
Table 49: Analysis of Clinical Failure at Day 70 Using Weighted GEE and Weighted GLMM Models- ITT Analysis Population 97
Table 50: Analysis of Microbiologic Success Using ITT or mITT Analysis Populations 98
Table 51: Analysis of Microbiologic Success at Day 42 and Day 70 Using CE Analysis Population 100
Table 52: Subgroup Analysis of Microbiologic Success by Timepoint – ITT Analysis Population 101
Table 53: Subgroup Analysis of Microbiologic Success by Timepoint – mITT Analysis Population 101
Table 54: Subgroup Analysis of Microbiologic Success by Timepoint – CE Analysis Population 102
Table 55: Distribution of DOOR at Day 42 by Subgroup Categories – ITT Analysis Population 103
Table 56: Distribution of DOOR at Day 70 by Subgroup Categories – ITT Analysis Population 103
Table 57: Summary Statistics of QoL Scores from the ARLG Bloodstream Infection QoL Measure at Day 42 and Day 70 Using ITT, mITT, and CE Analysis Populations 104
Table 58: Summary Statistics of QoL Scores Using the EQ-5D-5L Instrument at Day 42 and Day 70 Using ITT, mITT, and CE Analysis Populations 105
Table 59: Summary Statistics of QoL Scores Using the PROMIS Global Health Short Form at Day 42 and Day 70 Using ITT, mITT, and CE Analysis Populations 105
Table 60: Predictive Interval Plots (PIPS) Statistics for the Probability of Higher DOOR in the Dalbavancin Group at Day 70 – ITT Analysis Population 106
Table 61: Predictive Interval Plots (PIPS) Statistics for the Rates of Clinical Efficacy at Day 70 – ITT Analysis Population 106
Table 62: Number and Percentage of Subjects with Late Recurrence - Osteomyelitis Population 107

RESTRICTED

Table 63: Number and Percentage of Subjects reporting SAEs, AEs Leading Discontinuation, Grade 3 or Higher AEs, AESIs, and ALT/AST Elevations by Treatment Group - Safety Population 108

Table 64: Number and Percentage of subjects reporting SAEs, AEs Leading Discontinuation, Grade 3 or Higher AEs, AESIs, and ALT/AST Elevations by Standard of Care Antibiotics – Standard of Care Arm, Safety Population 109

Table 65: Summary of Treatment-Emergent Adverse Events by MedDRA System Organ Class and Preferred Term, Relationship, and Treatment Group....110

Table 66: Summary of Treatment-Emergent Adverse Events by MedDRA System Organ Class and Preferred Term, Maximum Severity, Relationship, and Treatment Group....111

Table 67: Summary of Treatment-Emergent Adverse Events by MedDRA System Organ Class and High Level Group Term, Relationship, and Treatment Group 112

Table 68: Summary of Treatment-Emergent Adverse Events by MedDRA System Organ Class and High Level Group Term, Maximum Severity, Relationship, and Treatment Group....113

Table 69: Summary of Treatment-Emergent Adverse Events by MedDRA System Organ Class, Maximum Severity, Relationship, and Treatment Group 114

Table 70: Summary of AESIs by MedDRA System Organ Class, Maximum Severity, Relationship, and Treatment Group....115

Table 71: Summary of Treatment-Emergent AEs, On-therapy SAEs, AESIs, and AEs Leading Discontinuation Occurring in 2% of Subjects in Any Treatment Group by MedDRA Preferred Term, and Treatment Group – Safety Population....116

Table 72: Summary of Fatal on-Therapy SAEs by MedDRA Preferred Term, and Treatment Group – Safety Population 117

Table 73: Summary of Fatal on-Therapy SAEs by MedDRA High Level Group Term, and Treatment Group – Safety Population 118

Table 74: Listing of Serious Adverse Events 119

Table 75: Listing of Adverse Events of Special Interest 119

Table 76: Listing of Adverse Events Leading to Discontinuation....119

Table 77: Listing of Subjects whose Outcome was Fatal During the Study 119

Table 78: Listing of Treatment-Emergent Adverse Events 120

Table 79: Listing of Potentially Clinically Significant Laboratory Results – Chemistry....122

Table 80: Listing of Potentially Clinically Significant Laboratory Results – Hematology....123

Table 81: Number and Percentage of Subjects with Potentially Clinically Significant Post-Baseline Laboratory Values by Parameter and Treatment Group – Chemistry Parameters 124

Table 82: Laboratory Summary Statistics by Parameter, Time Point, and Treatment Group – Serum Chemistry, Sodium....125

Table 83: Laboratory Summary Statistics by Parameter, Time Point, and Treatment Group – Serum Chemistry, Potassium....125

Table 84: Laboratory Summary Statistics by Parameter, Time Point, and Treatment Group – Serum Chemistry, Calcium 125

Table 85: Laboratory Summary Statistics by Parameter, Time Point, and Treatment Group – Serum Chemistry, Chloride 125

Table 86: Laboratory Summary Statistics by Parameter, Time Point, and Treatment Group – Serum Chemistry, Bicarbonate....125

Table 87: Laboratory Summary Statistics by Parameter, Time Point, and Treatment Group – Serum Chemistry, Glucose 125

Table 88: Laboratory Summary Statistics by Parameter, Time Point, and Treatment Group – Serum Chemistry, Blood Urea Nitrogen 125

Table 89: Laboratory Summary Statistics by Parameter, Time Point, and Treatment Group – Serum Chemistry, Creatinine 125

Table 90: Laboratory Summary Statistics by Parameter, Time Point, and Treatment Group – Serum Chemistry, Total Protein 126

Table 91: Laboratory Summary Statistics by Parameter, Time Point, and Treatment Group – Serum Chemistry, Alkaline Phosphatase 126

Table 92: Laboratory Summary Statistics by Parameter, Time Point, and Treatment Group – Serum Chemistry, Albumin....126

Table 93: Laboratory Summary Statistics by Parameter, Time Point, and Treatment Group – Serum Chemistry, Total Bilirubin 126

Table 94: Laboratory Summary Statistics by Parameter, Time Point, and Treatment Group – Serum Chemistry, AST 126

Table 95: Laboratory Summary Statistics by Parameter, Time Point, and Treatment Group – Serum Chemistry, ALT 126

Table 96: Number and Percentage of Subjects with Potentially Clinically Significant Post-Baseline Laboratory Values by Parameter and Treatment Group – Hematology Parameters....127

Table 97: Laboratory Summary Statistics by Parameter, Time Point, and Treatment Group – Hematology, Absolute WBC....128

Table 98: Laboratory Summary Statistics by Parameter, Time Point, and Treatment Group – Hematology, Erythrocyte 128

Table 99: Laboratory Summary Statistics by Parameter, Time Point, and Treatment Group – Hematology, Hemoglobin 128

Table 100: Laboratory Summary Statistics by Parameter, Time Point, and Treatment Group – Hematology, Hematocrit 128

Table 101: Laboratory Summary Statistics by Parameter, Time Point, and Treatment Group – Hematology, Platelet Count....128

Table 102: Laboratory Summary Statistics by Parameter, Time Point, and Treatment Group – Hematology, Neutrophils 128

Table 103: Laboratory Summary Statistics by Parameter, Time Point, and Treatment Group – Hematology, Lymphocytes....128

Table 104: Laboratory Summary Statistics by Parameter, Time Point, and Treatment Group – Hematology, Monocytes....128

Table 105: Summary of Vital Signs Values by Vital Sign Parameter, Visit, and Treatment Group....129

Table 106: Summary of Change from Baseline Vital Signs Values by Vital Sign Parameter, Visit, and Treatment Group....131

Table 107: Number and Percentage of Subjects with Prior and Concurrent Medications by WHO Drug Classification and Treatment Group....133

Table 108: Number and Percentage of Subjects with Nondrug Interventions by MedDRA System Organ Class and Treatment Group – ITT Population....134

9.5.1 Efficacy and Safety Measurements Assessed and Flow Chart

Table 1: Schedule of Study Procedures

	Induction Period	Screening/ Enrollment	Open Label Treatment Period				Post-treatment Follow-up Period		
	Visit 0 (Pre-Screening, Day -10 to Day 1)	Visit 1 (Day –1 to Day 1)	Visit 2 (Baseline, Day 1)	Visit 3 (Day 8 ± 1 day)	Visit 4 (Day 22 ± 2 days)	Visit 5 (Day 42 ± 3 days)	Visit 6 (TOC, Day 70 ± 7 days)[a]	ET[b]	Visit 7 (Day 180 ± 14 days, Osteomyelitis group)[a]
Informed Consent		X							
Dalbavancin[c]			X	X					
Standard of care antibiotic therapy[c]	X	X	X (Duration 28-56 days)						
Medical history[d]		X	X			X	X	X	
Medication history[e]		X							
Randomization			X						
AEs/AESIs/SAEs			X	X	X	X	X	X	
Hematology and serum chemistry blood sampling[f]		X		X[g]	X	X			
Coagulation lab tests[f]		X							
Pregnancy test[h]		X							
PK sampling[i]			X	X	X	X	X	X	
Vital signs[j]		X	X	X[k]	X	X	X	X	X
Physical examination[l]		X	X	X	X	X	X	X	X
Echocardiogram[m]		X							
Investigator assessment of efficacy						X	X	X	X
Concomitant medications[n]		X	X	X	X	X	X	X	X
Concomitant nondrug interventions		X	X	X	X	X	X	X	X
QoL assessment[o]			X	X	X	X	X	X	X

Table 1: Schedule of Study Procedures (continued)

	Induction Period	Screening/ Enrollment	Open Label Treatment Period				Post-treatment Follow-up Period		
	Visit 0 (Pre-Screening, Day -10 to Day 1)	Visit 1 (Day –1 to Day 1)	Visit 2 (Baseline, Day 1)	Visit 3 (Day 8 ± 1 day)	Visit 4 (Day 22 ± 2 days)	Visit 5 (Day 42 ± 3 days)	Visit 6 (TOC, Day 70 ± 7 days)[a]	ET[b]	Visit 7 (Day 180 ± 14 days, Osteomyelitis group)[a]

AEs = adverse events; AESIs = adverse events of special interest; eCRF = electronic case report form; ET = Early Termination; PK = pharmacokinetic; SAE = serious adverse event

[a]Telephone visit permissible if in-person visit is not possible; in person visit still preferred.

[b]Patients who prematurely discontinue therapy should have an ET Visit within 72 hours.

[c]All subjects will be receiving standard of care prior to randomization; after randomization, subjects will receive either dalbavancin or standard of care based on their assigned treatment group.

[d]Includes targeted/pertinent medical and surgical history only

[e]A complete medication history will be completed through 30 days prior to ICF signing; an extended 60 day review will be conducted for dalbavancin and oritavancin given the long half-lives of both drugs.

[f]Visit 1 hematology, coagulation lab tests (PT, PTT, and/or INR) and serum chemistry will be done in order to qualify the patient for the study, if not already collected per standard of care within 48 hours prior to randomization.

[g]A serum creatinine assessment will be required within the 72 hours prior to the 2nd (Day 8) dalbavancin dose. Whether a serum creatinine must be repeated on Day 8 will be at the discretion of the site investigator based upon stability of the serum creatinine in the preceding 72 hours and whether the serum creatinine is near the threshold where dose adjustment would be necessary (e.g., near 30 mL/min).

[h]Women of childbearing potential only, if not already performed (see Appendix B Definitions in the protocol); ensure test is negative within 48 hours before randomization. If the serum test results cannot be obtained before randomization, a urine pregnancy test may be used for enrollment.

[i]Dalbavancin PK samples will be drawn only for subjects receiving dalbvacancin. PK samples will be drawn at Day 1 prior to dose, at end of infusion ± 10 minutes, 6 ± 2 hours post end of dose, 12 ± 4 hours post end of dose, 24 ± 6 hours post end of dose), Day 8 (prior to 2nd dose), Day 22 ± 2 days (at time of clinic visit), day 42 ± 3 days, day 70 ± 7 days, and with any ET visit. Each sample must be accompanied by draw time and date.

[j]Vital signs include blood pressure, respiration rate, pulse rate, and temperature.

[k]Day 8 vital signs not required for subjects receiving SOC antibiotics if discharge occurs prior to day 8.

[l]A physical examination (including general appearance, examination of head, eyes, ears, nose, throat, neck, skin, heart, lungs, abdomen, neurologic system, musculoskeletal system, extremities, height, and body weight) will be done at Screening (Visit 1). If height or weight is not obtainable (eg, patient is immobilized), use the last known or stated height and weight. At subsequent visits, targeted physical exams will focus on changes from prior exams and on the evaluation of newly reported symptoms.

[m]Transthoracic echocardiogram or, if clinically indicated, transesophageal echocardiogram to be performed (local laboratory), unless one has been performed as standard of care for this episode of bacteremia/endocarditis

[n]All concomitant medications from Screening (Visit 1) through Day 42 (± 3 days) (Visit 5) must be recorded in the patient's medical record and on the eCRFs. Between the Day 42 Visit and Day 70 Visit, all concomitant medications for an AE or any antibacterial therapy should be recorded in the patient's medical record and on the eCRF.

[o]QoL assessments include the ARLG Bloodstream Infection QoL Measure (Appendix C in the protocol), the EQ-5D-5L (https://euroqol.org/eq-5d-instruments/sample-demo/), and the PROMIS Global Health Short Form (http://www.healthmeasures.net/administrator/components/com_instruments/uploads/Global%20Health%20Scale%20v1.2%2008.22.2016.pdf).

10.2 Protocol Deviations

Table 2: Distribution of Protocol Deviations by Category, Type, and Treatment Group – ITT Population

Category	Deviation Type	Dalbavancin (N=X)		Standard of Care (N=X)		All Subjects (N=X)	
		No. of Subj.	No. of Dev.	No. of Subj.	No. of Dev.	No. of Subj.	No. of Dev.
Eligibility/enrollment	Any type	X	x	x	x	x	x
	Did not meet inclusion criterion	X	x	x	x	x	x
	Met exclusion criterion	X	x	x	x	x	x
	ICF not signed prior to study procedures	X	x	x	x	x	x
	Other	X	x	x	x	x	x
Treatment administration schedule	Any type	X	x	x	x	x	x
	Out of window visit	X	x	x	x	x	x
	Missed visit/visit not conducted	X	x	x	x	x	x
	Missed treatment administration	X	x	x	x	x	x
	Delayed treatment administration	X	x	x	x	x	x
	Other	X	x	x	x	x	x
Follow-up visit schedule	Any type	X	x	x	x	x	x
	Out of window visit	X	x	x	x	x	x
	Missed visit/visit not conducted	X	x	x	x	x	x
	Other	X	x	x	x	x	x
Protocol procedure/assessment	Any type	X	x	x	x	x	x
	Incorrect version of ICF signed	X	x	x	x	x	x
	Blood not collected	X	x	x	x	x	x
	Other specimen not collected	X	x	x	x	x	x
	Too few aliquots obtained	X	x	x	x	x	x
	Specimen result not obtained	X	x	x	x	x	x
	Required procedure not conducted	X	x	x	x	x	x
	Required procedure done incorrectly	X	x	x	x	x	x
	Study product temperature excursion	X	x	x	x	x	x
	Specimen temperature excursion	X	x	x	x	x	x
	Other	X	x	x	x	x	x
Treatment administration	Any type	X	x	x	x	x	x
	Required procedure done incorrectly	X	x	x	x	x	x
	Study product temperature excursion	X	x	x	x	x	x
	Other	X	x	x	x	x	x

N= Number of subjects in the ITT Population.

14.1 Description of Study Subjects

14.1.1 Disposition of Subjects

Table 3: Subject Disposition by Treatment Group

Subject Disposition	Dalbavancin (N=X)		Standard of Care (N=X)		All Subjects (N=X)	
	n	%	n	%	n	%
Screened	--	--	--	--	x	--
Enrolled	x	100	x	100	x	100
Randomized	x	xx	x	xx	x	xx
Received Treatment	x	xx	x	xx	x	xx
Completed All Scheduled Dalbavancin Treatments	x	xx	N/A	N/A	x	xx
Completed <4 Weeks of Standard of Care Treatment[a]	N/A	N/A	x	xx	x	xx
Completed 4 - <6 Weeks of Standard of Care Treatment[a]	N/A	N/A	x	xx	x	xx
Completed 6 - <8 Weeks of Standard of Care Treatment[a]	N/A	N/A	x	xx	x	xx
Completed ≥8 Weeks of Standard of Care Treatment[a]	N/A	N/A	x	xx	x	xx
Discontinued Treatment[b]	x	xx	x	xx	x	xx
Completed Day 8 Visit[b]	x	xx	x	xx	x	xx
Completed Day 22 Visit[b]	x	xx	x	xx	x	xx
Completed Day 42 Visit[b]	x	xx	x	xx	x	xx
Completed Day 70 Visit[b]	x	xx	x	xx	x	xx
Completed Day 180 Visit[c]	x	xx	x	xx	x	xx

N= Number of subjects in the ITT Population.
[a] Duration of standard of care treatment calculated from date of first negative blood culture to end date of last antibiotic received.
[b] Refer to Listing 16.2.1 for reasons subjects discontinued or terminated early.
[c] Day 180 Visit will only be completed by subjects with osteomyelitis.

Table 4: Analysis Populations by Treatment Group – ITT Population

Analysis Populations	Reason Subjects Excluded	Dalbavancin (N=X)		Standard of Care (N=X)		All Subjects (N=X)	
		n	%	n	%	%	n
ITT	Any Reason						
	Subject not randomized						
Safety	Any Reason						
	Subject not treated with at least one dose of study product						
Modified ITT	Any Reason						
	Subject was excluded from ITT population						
	Subject not treated with at least one dose of study product						
Clinically Evaluable at Day 42	Any Reason						
	Subject was excluded from mITT population						
	Subject had missing data or major protocol deviation at Day 42[a]						
Clinically Evaluable at Day 70	Any Reason						
	Subject was excluded from mITT population						
	Subject had missing data or major protocol deviation at Day 70[a]						

N= Number of subjects in the ITT Population.
[a] Major protocol deviation for analysis population purposes will be defined as those that prevent the adjudication committee from evaluating the outcomes at the given day.

Table 5: Dates of First Treatment by Site and Treatment Group

[Implementation note: Replace site numbers by site names and sort the site list alphabetically.]

Site	Treatment Group	February 2021- December 2021	January 2022-December 2022
Any Site	Any Treatment	x	x
Any Site	Dalbavancin	x	x
	Standard of Care	x	x
Site 1	Dalbavancin	x	x
	Standard of Care	x	x
Site 2	Dalbavancin	x	x
	Standard of Care	x	x
Site 3	Dalbavancin	x	x
	Standard of Care	x	x
Site 4	Dalbavancin	x	x
	Standard of Care	x	x
[Repeat for all sites that enrolled at least one subject.]			

Table 6: Treatment Compliance for the Dalbavancin Treatment Group – Safety Population

	Dalbavancin (N=X)	
	**n**	**%**
Received 1 Dose of Dalbavancin	x	x
Received 2 Doses of Dalbavancin	x	x
Lowered dose of Dalbavancin received[a]	x	x
Interrupted/Incomplete dose of Dalbavancin received	x	x

N = Number of subjects in the Safety Population who received at least one dose of Dalbavancin.
[a] Summarizes the number of subjects that received a lower dose of dalbavancin due to an absence of regular hemodialysis or peritoneal dialysis and CrCl < 30 mL/min.

Table 7: Treatment Compliance in the Standard of Care Treatment Group Measured by Number of Days – Safety Population

Standard of Care Antibiotics	Statistic	Standard of Care (N=X)		
		Duration from Date of First Positive Blood Culture (Days)	Duration from Date of First Negative Blood Culture (Days)	Duration from Date of Randomization (Days)
Any Standard of Care Antibiotic	N	x	x	x
	Mean	x.x	x.x	x.x
	Standard Deviation	x.x	x.x	x.x
	Median	x	x	x
	Minimum	x	x	x
	Maximum	x	x	x
Cefazolin	N	n	n	n
	Mean	x.x	x.x	x.x
	Standard Deviation	x.x	x.x	x.x
	Median	x	x	x
	Minimum	x	x	x
	Maximum	x	x	x
Nafcillin	N	x	x	x
	Mean	x.x	x.x	x.x
	Standard Deviation	x.x	x.x	x.x
	Median	x	x	x
	Minimum	x	x	x
	Maximum	x	x	x
Oxacillin	N	x	x	x
	Mean	x.x	x.x	x.x
	Standard Deviation	x.x	x.x	x.x
	Median	x	x	x
	Minimum	x	x	x
	Maximum	x	x	x
Vancomycin	N	x	x	x
	Mean	x.x	x.x	x.x
	Standard Deviation	x.x	x.x	x.x
	Median	x	x	x
	Minimum	x	x	x
	Maximum	x	x	x
Daptomycin	N	x	x	x
	Mean	x.x	x.x	x.x
	Standard Deviation	x.x	x.x	x.x
	Median	x	x	x
	Minimum	x	x	x
	Maximum	x	x	x
[Create additional rows to add any other Standard of Care antibiotics administered in this study.]				
N = Number of subjects in the Safety Population in the standard of care arm.<br>n = Number of subjects in the Safety Population who received the corresponding standard of care antibiotic.				

Table 8: Ineligibility Summary of Screen Failures

Inclusion/ Exclusion Category	Inclusion/ Exclusion Criterion	n[a]	%[b]
All Subjects	Total number of subjects failing any eligibility criterion or who were eligible but not randomized.	x	100
Inclusion and Exclusion	Number of subjects failing any eligibility criterion		
Inclusion	Any inclusion criterion	x	xx
	[inclusion criterion 1]	x	xx
	[inclusion criterion 2]	x	xx
	[inclusion criterion 3]	x	xx
	…		
Exclusion	Any exclusion criterion	x	xx
	[exclusion criterion 1]	x	xx
	[exclusion criterion 2]	x	xx
	[exclusion criterion 3]	x	xx
	…		
Eligible but Not Enrolled	Any Reason	x	xx
	[Reason 1]	x	xx
	[Reason 2]	x	xx
	…		

[a] More than one criterion may be marked per subject.
[b] Denominator for percentages is the total number of subjects not enrolled in this study which include screen failures and subjects eligible but not enrolled.

14.1.2 Demographic Data by Study Group

Table 9: Summary of Categorical Demographic and Baseline Characteristics by Site – ITT Population

Demographic Category		Sex		Ethnicity				Race							Baseline Pathogen	
Characteristic		Male	Female	Not Hispanic or Latino	Hispanic or Latino	Not Reported	Unknown	American Indian or Alaska Native	Asian	Native Hawaiian or Other Pacific Islander	Black or African American	White	Multi-Racial	Unknown	MRSA	MSSA
[Site 1] (N=X)	n	x	x	x	x	x	X	x	x	x	x	x	x	x		
	%	x	x	x	x	x	X	x	x	x	x	x	x	x		
[Site 2] (N=X)	n	x	x	x	x	x	X	x	x	x	x	x	x	x		
	%	x	x	x	x	x	X	x	x	x	x	x	x	x		
All Subjects (N=X)	n	x	x	x	x	x	X	x	x	x	x	x	x	x		
	%	x	x	x	x	x	X	x	x	x	x	x	x	x		

N = Number of subjects in the ITT Population.

Note: Will repeat for all sites.

Table 10: Summary of Categorical Demographic and Baseline Characteristics by Site – Safety Population

The table will repeat Table 9 limited to the safety population

Table 11: Summary of Categorical Demographic and Baseline Characteristics by Treatment Group – ITT Population

Variable	Characteristic	Dalbavancin (N=X)		Standard of Care (N=X)		All Subjects (N=X)	
		n	%	n	%	n	%
Sex	Male	x	xx	x	xx	x	xx
	Female						
Ethnicity	Not Hispanic or Latino	x	xx	x	xx	x	xx
	Hispanic or Latino						
	Not Reported						
	Unknown						
Race	American Indian or Alaska Native	x	xx	x	xx	x	xx
	Asian						
	Native Hawaiian or Other Pacific Islander						
	Black or African American						
	White						
	Multi-Racial						
	Unknown						
Baseline Pathogen	MRSA						
	MSSA						
PWID Status	PWID						
	Non-PWID						
Infectious Disease Consultation	Yes						
	No						
TEE Performed	Yes						
	No						
Underlying Site of Infection	Endovascular						
	Bone and Joint						
	Skin						
	Pulmonary						
	Other/Unknown						
Immunosuppression	Yes						
	No						
Duration of Initial Bacteremia (Days)	< 2						
	2-4						
	> 4						
	Other/Unknown						

Table 11: Summary of Categorical Demographic and Baseline Characteristics by Treatment Group – ITT Population (continued)

Variable	Characteristic	Dalbavancin (N=X)		Standard of Care (N=X)		All Subjects (N=X)	
		n	%	n	%	n	%
Pre-randomization Antibiotics	[Antibiotic 1]						
	[Antibiotic 2]						
	[Antibiotic 3]						
	…						
Comorbid Conditions	Heart failure						
	Chronic kidney disease						
	Diabetes						
	Liver disease						
	Cancer						
Baseline Infection Characteristics	Right-sided endocarditis						
	ABSSSI						
	Septic pulmonary emboli						
	Osteomyelitis, non-vertebral						
	Vertebral osteomyelitis						
	Septic arthritis						
	Catheter-associated bloodstream infection						
	Cardiac device infection						
	Intravascular graft infection						
	Prosthetic valve infection						
	Prosthetic joint infection						
	Pneumonia						

N = Number of subjects in the ITT Population.
PWID = Persons Who Inject Drugs.
TEE = Transesophageal Echocardiography.
ABSSSI = Acute Bacterial Skin and Skin Structure Infection.

Table 12: Summary of Categorical Demographic and Baseline Characteristics by Treatment Group – Safety Population

This table will repeat Table 11 limited to the safety population.

Table 13: Summary of Continuous Demographic and Baseline Characteristics by Site – ITT Population

Variable	n	Mean	Standard Deviation	Median	Minimum	Maximum
**[Site 1 (N=X)]**						
Age (years)	x	x.x	x.x	x.x	x	x
BMI (kg/m^2)	x	x.xx	x.xx	x.xx	x.x	x.x
Height (cm)	x	x.xx	x.xx	x.xx	x.x	x.x
Weight (kg)	x	x.xx	x.xx	x.xx	x.x	x.x
Baseline QoL[a]	x	x.xx	x.xx	x.xx	x.x	x.x
Creatinine Clearance (mL/min)	x	x.xx	x.xx	x.xx	x.x	x.x
Duration of Bacteremia (days)	x	x.x	x.x	x.x	x	x
**[Site 2 (N=X)]**						
Age (years)	x	x.x	x.x	x.x	x	X
BMI (kg/m^2)	x	x.x	x.x	x.x	x	X
Height (cm)	x	x.x	x.x	x.x	x	X
Weight (kg)	x	x.x	x.x	x.x	x	X
Baseline QoL[a]	x	x.xx	x.xx	x.xx	x.x	x.x
Creatinine Clearance (mL/min)	x	x.xx	x.xx	x.xx	x.x	x.x
Duration of Bacteremia (days)	x	x.x	x.x	x.x	x	x
**[Repeat for all sites and all subjects]**						

N = Number of subjects in the ITT Population.
n = Number of subjects in the ITT Population with non-missing values for the corresponding baseline characteristic.
[a] Baseline QoL standardized score is obtained from the questions arising from the PROMIS physical function item bank (PROMIS Item Bank v2.0, short form 6b) item bank on the ARLG Bloodstream Infection QoL.

Table 14: Summary of Continuous Demographic and Baseline Characteristics by Site – Safety Population

This table will repeat Table 13 limited to the safety population.

Table 15: Summary of Continuous Demographic and Baseline Characteristics by Treatment Group – ITT Population

Variable	Statistic	Dalbavancin (N=X)	Standard of Care (N=X)	All Subjects (N=X)
Age (years)	n	X	x	x
	Mean	x.x	x.x	x.x
	Standard Deviation	x.x	x.x	x.x
	Median	x.x	x.x	x.x
	Minimum	X	x	x
	Maximum	X	x	x
BMI (kg/m^2)	n	x.xx	x.xx	x.xx
	Mean	X	x	x
	Standard Deviation	x.xx	x.xx	x.xx
	Median	x.xx	x.xx	x.xx
	Minimum	x.x	x.x	x.x
	Maximum	x.x	x.x	x.x
Height (cm)	n	X	x	x
	Mean	x.xx	x.xx	x.xx
	Standard Deviation	x.xx	x.xx	x.xx
	Median	x.xx	x.xx	x.xx
	Minimum	X	x	x
	Maximum	X	x	x
Weight (kg)	n	X	x	x
	Mean	x.xx	x.xx	x.xx
	Standard Deviation	x.xx	x.xx	x.xx
	Median	x.xx	x.xx	x.xx
	Minimum	x.x	x.x	x.x
	Maximum	x.x	x.x	x.x
Baseline QoL[a]	n	X	x	x
	Mean	x.xx	x.xx	x.xx
	Standard Deviation	x.xx	x.xx	x.xx
	Median	x.xx	x.xx	x.xx
	Minimum	x.x	x.x	x.x
	Maximum	x.x	x.x	x.x

Table 15: Summary of Continuous Demographic and Baseline Characteristics by Treatment Group – ITT Population (continued)

Variable	Statistic	Dalbavancin (N=X)	Standard of Care (N=X)	All Subjects (N=X)
Creatinine Clearance (mL/min)	n	X	x	x
	Mean	x.xx	x.xx	x.xx
	Standard Deviation	x.xx	x.xx	x.xx
	Median	x.xx	x.xx	x.xx
	Minimum	x.x	x.x	x.x
	Maximum	x.x	x.x	x.x
Duration of Bacteremia (days)	n	X	x	x
	Mean	x.xx	x.xx	x.xx
	Standard Deviation	x.xx	x.xx	x.xx
	Median	x.xx	x.xx	x.xx
	Minimum	x.x	x.x	x.x
	Maximum	x.x	x.x	x.x

N = Number of subjects in the ITT Population.
n = Number of subjects in the ITT Population with non-missing values for the corresponding baseline characteristic.
[a] Baseline QoL standardized score is obtained from the questions arising from the PROMIS physical function item bank (PROMIS Item Bank v2.0, short form 6b) item bank on the ARLG Bloodstream Infection QoL.

Table 16: Summary of Continuous Demographic and Baseline Characteristics by Treatment Group – Safety Population

This table will repeat Table 15 limited to the safety population.

14.1.3 Prior and Concurrent Medical Conditions

Table 17: Summary of Subjects with Pre-Existing Medical Conditions by MedDRA System Organ Class and Treatment Group – ITT Population

MedDRA System Organ Class	Dalbavancin (N=X)		Standard of Care (N=X)		All Subjects (N=X)	
	n	%	n	%	n	%
Any SOC	x	xx	x	xx	x	xx
[SOC 1]	x	xx	x	xx	x	xx
[SOC 2]	x	xx	x	xx	x	xx
[repeat for all SOC]						

N = Number of subjects in the ITT population.
n = Number of subjects reporting medical history within the specified SOC. A subject is only counted once per SOC.

14.2 Efficacy Data

Table 18: Percentage of Subjects with Missing Data by Study Endpoint, Timepoint, and Treatment Group – ITT Analysis Population

Endpoint	Dalbavancin (N=X)		Standard of Care (N=X)		All Subjects (N=X)	
	n	%	N	%	n	%
**Day 42**						
DOOR						
Clinical efficacy						
Microbiologic Success						
Clinical Failure						
**Day 70**						
DOOR						
Clinical efficacy						
Microbiologic Success						
Clinical Failure						

N = Number of subjects in the ITT population in the respective treatment group.
n = Number of subjects with missing data.

Table 19: Percentage of Subjects with Missing Data by Study Endpoint, Timepoint, and Treatment Group – mITT Analysis Population

This table will repeat Table 18 limited to the mITT population.

Table 20: Analysis of DOOR at Day 70 – ITT Analysis Population

[Implementation Note: This table will be included in the interim analysis.]

Model	Tie-Breaking[a]	Subjects with non-missing DOOR n (%)	Subjects with missing DOOR n (%)	Pr(Better DOOR in Dalbavancin Arm)[b] (95% CI)[c]
IPW	Tie-Breaking	x (x)	x (x)	x.xx (x.xx, x.xx)
	No Tie-Breaking	x (x)	x (x)	x.xx (x.xx, x.xx)
Multiple Imputation	Tie-Breaking	x (x)	x (x)	x.xx (x.xx, x.xx)
	No Tie-Breaking	x (x)	x (x)	x.xx (x.xx, x.xx)

[a]DOOR analysis with tie-breaking is the primary analysis.
[b]Probability of Better DOOR in Dalbavancin arm at Day 70 + 0.5 Probability of Equal DOOR.
[c]95% CI obtained using the method described in Halperin et. al. Superiority of Dalbavancin is concluded if the lower bound of the 95% CI for the DOOR probability is above 0.5.

Table 21: Analysis of DOOR at Day 70 – mITT Analysis Population

This table will repeat Table 20 limited to the mITT population.

Table 22: Analysis of DOOR at Day 42 – ITT Analysis Population

Model	Tie-Breaking[a]	Subjects with non-missing DOOR n (%)	Subjects with missing DOOR n (%)	Pr(Better DOOR in Dalbavancin Arm)[b] (95% CI)[c]
IPW	Tie-Breaking	x (x)	x (x)	x.xx (x.xx, x.xx)
	No Tie-Breaking	x (x)	x (x)	x.xx (x.xx, x.xx)
Multiple Imputation	Tie-Breaking	x (x)	x (x)	x.xx (x.xx, x.xx)
	No Tie-Breaking	x (x)	x (x)	x.xx (x.xx, x.xx)

[a] DOOR analysis with tie-breaking is the primary analysis.
[b] Probability of Better DOOR in Dalbavancin arm at Day 42 + 0.5 Probability of Equal DOOR.
[c] 95% CI obtained using the method described in Halperin et. al. Superiority of Dalbavancin is concluded if the lower bound of the 95% CI for the DOOR probability is above 0.5.

Table 23: Analysis of DOOR at Day 42 – mITT Analysis Population

This table will repeat Table 22 limited to the mITT population.

Table 24: Analysis of DOOR at Day 42 and Day 70 – CE Analysis Population

Timepoint	Tie-Breaking	N	Pr(Better DOOR)[a]	Halperin 95% CI[b]
Day 42	Tie-Breaking	x	x.xx	x.xx, x.xx
	No Tie-Breaking	x	x.xx	x.xx, x.xx
Day 70	Tie-Breaking	x	x.xx	x.xx, x.xx
	No Tie-Breaking	x	x.xx	x.xx, x.xx

N = Number of subjects with complete data in the given analysis population.
[a] Probability of Better DOOR in Dalbavancin Arm at Day 42 (or Day 70) + 0.5 Probability of Equal DOOR.
[b] 95% CI obtained using the method described in Halperin et. al.

Table 25: Subgroup Analysis of DOOR with Tie-Breaking by Timepoint – ITT Analysis Population

Timepoint	Variable	Level	N_d	N_s	DOOR Probability[a]	95% CI[b]
Day 42	Baseline Pathogen	MRSA	xx	xx	x.xx	x.xx, x.xx
		MSSA	xx	xx	x.xx	x.xx, x.xx
	PWID Status	PWID	xx	xx	x.xx	x.xx, x.xx
		Non-PWID	xx	xx	x.xx	x.xx, x.xx
	Infectious Disease Consultation	Yes	xx	xx	x.xx	x.xx, x.xx
		No	xx	xx	x.xx	x.xx, x.xx
	Underlying Site of Infection	Endovascular	xx	xx	x.xx	x.xx, x.xx
		Bone and Joint	xx	xx	x.xx	x.xx, x.xx
		Skin	xx	xx	x.xx	x.xx, x.xx
		Pulmonary	xx	xx	x.xx	x.xx, x.xx
	Immunosuppression	Yes	xx	xx	x.xx	x.xx, x.xx
		No	xx	xx	x.xx	x.xx, x.xx
	Duration of Initial Bacteremia (Days)	< 2	xx	xx	x.xx	x.xx, x.xx
		2-4	xx	xx	x.xx	x.xx, x.xx
		> 4	xx	xx	x.xx	x.xx, x.xx
Day 70	Baseline Pathogen	MRSA	xx	xx	x.xx	x.xx, x.xx
		MSSA	xx	xx	x.xx	x.xx, x.xx
	PWID Status	PWID	xx	xx	x.xx	x.xx, x.xx
		Non-PWID	xx	xx	x.xx	x.xx, x.xx
	Infectious Disease Consultation	Yes	xx	xx	x.xx	x.xx, x.xx
		No	xx	xx	x.xx	x.xx, x.xx
	Underlying Site of Infection	Endovascular	xx	xx	x.xx	x.xx, x.xx
		Bone and Joint	xx	xx	x.xx	x.xx, x.xx
		Skin	xx	xx	x.xx	x.xx, x.xx
		Pulmonary	xx	xx	x.xx	x.xx, x.xx
	Immunosuppression	Yes	xx	xx	x.xx	x.xx, x.xx
		No	xx	xx	x.xx	x.xx, x.xx
	Duration of Initial Bacteremia (Days)	< 2	xx	xx	x.xx	x.xx, x.xx
		2-4	xx	xx	x.xx	x.xx, x.xx
		> 4	xx	xx	x.xx	x.xx, x.xx

N_d = number of subjects in the ITT population who received dalbavancin within the subgroup category.
N_s = number of subjects in the ITT population who received standard of care within the subgroup category.
PWID = Persons Who Inject Drugs.
[a] Probability of Better DOOR in Dalbavancin arm compared to standard of care + 0.5 Probability of Equal DOOR. This analysis uses IPW to handle missing values of DOOR and change in QoL as a tie breaker.
[b] 95% CI obtained using the method described in Halperin et. al.

Table 26: Subgroup Analysis of DOOR with Tie-Breaking by Timepoint – mITT Analysis Population

This table will repeat Table 25 limited to the mITT population.

Table 27: Subgroup Analysis of DOOR with Tie-Breaking by Timepoint – CE Analysis Population

Timepoint	Variable	Level	N_d	N_s	DOOR Probability[a]	Halperin 95% CI[b]
Day 42	Baseline Pathogen	MRSA	xx	xx	x.xx	x.xx, x.xx
		MSSA	xx	xx	x.xx	x.xx, x.xx
	PWID Status	PWID	xx	xx	x.xx	x.xx, x.xx
		Non-PWID	xx	xx	x.xx	x.xx, x.xx
	Infectious Disease Consultation	Yes	xx	xx	x.xx	x.xx, x.xx
		No	xx	xx	x.xx	x.xx, x.xx
	Underlying Site of Infection	Endovascular	xx	xx	x.xx	x.xx, x.xx
		Bone and Joint	xx	xx	x.xx	x.xx, x.xx
		Skin	xx	xx	x.xx	x.xx, x.xx
		Pulmonary	xx	xx	x.xx	x.xx, x.xx
	Immunosuppression	Yes	xx	xx	x.xx	x.xx, x.xx
		No	xx	xx	x.xx	x.xx, x.xx
	Duration of Initial Bacteremia (Days)	< 2	xx	xx	x.xx	x.xx, x.xx
		2-4	xx	xx	x.xx	x.xx, x.xx
		> 4	xx	xx	x.xx	x.xx, x.xx
Day 70	Baseline Pathogen	MRSA	xx	xx	x.xx	x.xx, x.xx
		MSSA	xx	xx	x.xx	x.xx, x.xx
	PWID Status	PWID	xx	xx	x.xx	x.xx, x.xx
		Non-PWID	xx	xx	x.xx	x.xx, x.xx
	Infectious Disease Consultation	Yes	xx	xx	x.xx	x.xx, x.xx
		No	xx	xx	x.xx	x.xx, x.xx
	Underlying Site of Infection	Endovascular	xx	xx	x.xx	x.xx, x.xx
		Bone and Joint	xx	xx	x.xx	x.xx, x.xx
		Skin	xx	xx	x.xx	x.xx, x.xx
		Pulmonary	xx	xx	x.xx	x.xx, x.xx
	Immunosuppression	Yes	xx	xx	x.xx	x.xx, x.xx
		No	xx	xx	x.xx	x.xx, x.xx
	Duration of Initial Bacteremia (Days)	< 2	xx	xx	x.xx	x.xx, x.xx
		2-4	xx	xx	x.xx	x.xx, x.xx
		> 4	xx	xx	x.xx	x.xx, x.xx

N_d = number of subjects in the CE population who received dalbavancin within the subgroup category.
N_s = number of subjects in the CE population who received standard of care within the subgroup category.
PWID = Persons Who Inject Drugs.
[a] Probability of Better DOOR in Dalbavancin Arm + 0.5 Probability of Equal DOOR.
[b] 95% CI obtained using the method described in Halperin et. al.

Table 28: Analysis of DOOR Categories – ITT Analysis Population

[Implementation Note: This table will be included in the interim analysis.]

Timepoint	DOOR Category	Dalbavancin (N=X)		Standard of Care (N=X)	
		n	% (95% CI)[a]	n	% (95% CI)[a]
Day 42	Alive with no events	xx	xx (xx, xx)	xx	xx (xx, xx)
	Alive with 1 event	xx	xx (xx, xx)	xx	xx (xx, xx)
	Alive with 2 events	xx	xx (xx, xx)	xx	xx (xx, xx)
	Alive with 3 events	xx	xx (xx, xx)	xx	xx (xx, xx)
	Death	xx	xx (xx, xx)	xx	xx (xx, xx)
	Missing	xx	xx (xx, xx)	xx	xx (xx, xx)
Day 70	Alive with no events	xx	xx (xx, xx)	xx	xx (xx, xx)
	Alive with 1 event	xx	xx (xx, xx)	xx	xx (xx, xx)
	Alive with 2 events	xx	xx (xx, xx)	xx	xx (xx, xx)
	Alive with 3 events	xx	xx (xx, xx)	xx	xx (xx, xx)
	Death	xx	xx (xx, xx)	xx	xx (xx, xx)
	Missing	xx	xx (xx, xx)	xx	xx (xx, xx)

N = Number of subjects in the ITT Population in the given treatment group.
n = Number of subjects in the corresponding analysis population, treatment group, and DOOR category
[a] 95% CI estimated using the Wilson Method.
Note: Events that are both infectious complications and SAEs or AEs leading to study drug discontinuation count twice towards the DOOR.

Table 29: Analysis of DOOR Categories – mITT Analysis Population

This table will repeat Table 28 limited to the mITT population.

Table 30: Analysis of DOOR Categories – CE Analysis Population

This table will repeat Table 28 limited to the CE population.

[Implementation note: The missing category is removed from this analysis since the CE population has complete data.]

Table 31: Cumulative Proportions of DOOR - ITT Analysis Population

[Implementation Note: This table will be included in the interim analysis.]

Timepoint	DOOR Category	Dalbavancin			Standard of Care			Cumulative DOOR Probability
		N	n[a]	%[a]	N	n[a]	%[a]	% (95% CI)[b]
Day 42	Alive with no events	xx	xx	xx	xx	xx	xx	xx (x.x, x.x)
	Alive with less than 2 events	xx	xx	xx	xx	xx	xx	xx (x.x, x.x)
	Alive with less than 3 events	xx	xx	xx	xx	xx	xx	xx (x.x, x.x)
	Alive or Dead	xx	xx	xx	xx	xx	xx	xx (x.x, x.x)
Day 70	Alive with no events	xx	xx	xx	xx	xx	xx	xx (x.x, x.x)
	Alive with less than 2 events	xx	xx	xx	xx	xx	xx	xx (x.x, x.x)
	Alive with less than 3 events	xx	xx	xx	xx	xx	xx	xx (x.x, x.x)
	Alive or Dead	xx	xx	xx	xx	xx	xx	xx (x.x, x.x)

N = Number of subjects in the ITT Population in the given treatment group with non-missing cumulative DOOR category at the corresponding timepoint.
n = Number of subjects in the given cumulative DOOR category.
[a] n and % represent cumulative numbers.
[b] 95% CI for cumulative DOOR probability obtained using the method described in Halperin et. al.
Note: Events that are both infectious complications and SAEs or AEs leading to study drug discontinuation count twice towards the DOOR.

Table 32: Cumulative Proportions of DOOR – mITT Analysis Population

This table will repeat Table 31 limited to the mITT population.

Table 33: Cumulative Proportions of DOOR – CE Analysis Population

This table will repeat Table 31 limited to the CE population.

Table 34: Summary of Expected Numbers Gained Loss at Day 42 and Day 70 by Analysis Population

Analysis Population	Timepoint	DOOR Category	Dalbavancin				Standard of Care				Gained Loss[a] (95% CI)
			N	n	%	Expected Number	N	n	%	Expected Number	
ITT	Day 42	Alive with no events	xx	xx	xx	xxx	xx	xx	xx	xxx	xx(xx, xx)
		Alive with 1 event	xx	xx	xx	xxx	xx	xx	xx	xxx	xx
		Alive with 2 events	xx	xx	xx	xxx	xx	xx	xx	xxx	xx
		Alive with 3 events	xx	xx	xx	xxx	xx	xx	xx	xxx	xx
		Death	xx	xx	xx	xxx	xx	xx	xx	xxx	xx
	Day 70	Alive with no events	xx	xx	xx	xxx	xx	xx	xx	xxx	xx
		Alive with 1 event	xx	xx	xx	xxx	xx	xx	xx	xxx	xx
		Alive with 2 events	xx	xx	xx	xxx	xx	xx	xx	xxx	xx
		Alive with 3 events	xx	xx	xx	xxx	xx	xx	xx	xxx	xx
		Death	xx	xx	xx	xxx	xx	xx	xx	xxx	xx
[Repeat for mITT and CE analysis populations]											

N = Number of subjects in the corresponding analysis population and treatment group with non-missing DOOR category at the corresponding timepoint.
n = Number of subjects in the corresponding analysis population, treatment group, and DOOR category.
[a] The gained loss is calculated as the difference in expected numbers, dalbavancin relative to standard of care.
Note: Events that are both infectious complications and SAEs or AEs leading to study drug discontinuation count twice towards the DOOR.

Table 35: Analysis of Clinical Efficacy at Day 70 Using ITT and mITT Analysis Populations

Analysis Population	Model	Statistic	Dalbavancin (N=X)	Standard of Care (N=X)
ITT	IPW	Subjects with non-missing clinical efficacy on Day 70 – n (%)	x (x)	x (x)
		Subjects with missing clinical efficacy on Day 70– n (%)	x (x)	x (x)
		Percent rate of clinical efficacy at Day 70 (95% CI)[a]	xx (xx, xx)	xx (xx, xx)
		Difference in rates (percent) of clinical efficacy, dalbavancin relative to standard of care (95% CI)[a]	xx (xx, xx)	-
		Conclusion of non-inferiority of dalbavancin to standard of care[b]	Yes/No	-
		Conclusion of superiority of dalbavancin to standard of care[c]	Yes/No	-
	Multiple Imputation	Subjects with non-missing clinical efficacy on Day 70 – n (%)	x (x)	x (x)
		Subjects with missing clinical efficacy on Day 70– n (%)	x (x)	x (x)
		Percent rate of clinical efficacy at Day 70 (95% CI)[a]	xx (xx, xx)	xx (xx, xx)
		Difference in rates (percent) of clinical efficacy, dalbavancin relative to standard of care (95% CI)[a]	xx (xx, xx)	-
		Conclusion of non-inferiority of dalbavancin to standard of care[b]	Yes/No	-
		Conclusion of superiority of dalbavancin to standard of care[c]	Yes/No	-
mITT	IPW	Subjects with non-missing clinical efficacy on Day 70 – n (%)	x (x)	x (x)
		Subjects with missing clinical efficacy on Day 70– n (%)	x (x)	x (x)
		Percent rate of clinical efficacy at Day 70 (95% CI)[a]	xx (xx, xx)	xx (xx, xx)
		Difference in rates (percent) of clinical efficacy, dalbavancin relative to standard of care (95% CI)[a]	xx (xx, xx)	-
		Conclusion of non-inferiority of dalbavancin to standard of care[b]	Yes/No	-
		Conclusion of superiority of dalbavancin to standard of care[c]	Yes/No	-
	Multiple Imputation	Subjects with non-missing clinical efficacy on Day 70 – n (%)	x (x)	x (x)
		Subjects with missing clinical efficacy on Day 70– n (%)	x (x)	x (x)
		Percent rate of clinical efficacy at Day 70 (95% CI)[a]	xx (xx, xx)	xx (xx, xx)
		Difference in rates (percent) of clinical efficacy, dalbavancin relative to standard of care (95% CI)[a]	xx (xx, xx)	-
		Conclusion of non-inferiority of dalbavancin to standard of care[b]	Yes/No	-
		Conclusion of superiority of dalbavancin to standard of care[c]	Yes/No	-

N = Number of subjects in the corresponding analysis Population. Multiple imputation was used to impute missing values.
[a] 95% CIs were obtained from linear regression model following multiple imputation adjusting for study day of Day 70.
[b] Non-inferiority of dalbavancin was concluded if the lower bound of the 95% CI for the difference in proportions is greater than -20%.
[c] Superiority of dalbavancin was concluded if the 95% CI for the difference in proportions does not contain 0.

Table 36: Analysis of Clinical Efficacy at Day 42 Using ITT and mITT Analysis Populations

This table will repeat Table 35 using Day 42.

Table 37: Analysis of Clinical Efficacy at Day 42 and Day 70 Using CE Analysis Population

[Implementation note: If the lower bound of 95% CI for the difference in rates of clinical efficacy is greater than -20%, annotate the interval with footnote b that states 'Lower bound of confidence interval greater than -20% (non-inferiority margin).']

Timepoint	Treatment Group	Rate of Clinical Efficacy			Difference in Rates		
		n	%	95% CI[a]	%	95% CI[a]	Miettinen–Nurminen 95% CI
Day 42	Dalbavancin (N=X)	x	xx	xx, xx	xx	xx, xx	xx, xx
	Standard of Care (N=X)	x	xx	xx, xx	Reference	-	-
Day 70	Dalbavancin (N=X)	x	xx	xx, xx	xx	xx, xx	xx, xx
	Standard of Care (N=X)	x	xx	xx, xx	Reference	-	-

N = Number of subjects in the CE analysis population at the corresponding timepoint.
n = Number of subjects in CE population with clinical efficacy at the corresponding timepoint.
[a] 95% CI obtained from linear regression.

Table 38: Subgroup Analysis of Clinical Efficacy by Timepoint – ITT Analysis Population

			Dalbavancin			Standard of Care			
**Timepoint**	**Variable**	**Level**	**N**	**n**	**Proportion % (95% CI)[a]**	**N**	**n**	**Proportion % (95% CI)[a]**	**Difference in Proportion % (95% CI)[a]**
Day 42	Baseline Pathogen	MRSA	xx	xx	xx (xx, xx)	xx	xx	xx (xx, xx)	xx (xx, xx)
		MSSA	xx	xx	xx (xx, xx)	xx	xx	xx (xx, xx)	xx (xx, xx)
	PWID Status	PWID	xx	xx	xx (xx, xx)	xx	xx	xx (xx, xx)	xx (xx, xx)
		Non-PWID	xx	xx	xx (xx, xx)	xx	xx	xx (xx, xx)	xx (xx, xx)
	Infectious Disease Consultation	Yes	xx	xx	xx (xx, xx)	xx	xx	xx (xx, xx)	xx (xx, xx)
		No	xx	xx	xx (xx, xx)	xx	xx	xx (xx, xx)	xx (xx, xx)
	Underlying Site of Infection	Endovascular	xx	xx	xx (xx, xx)	xx	xx	xx (xx, xx)	xx (xx, xx)
		Bone and Joint	xx	xx	xx (xx, xx)	xx	xx	xx (xx, xx)	xx (xx, xx)
		Skin	xx	xx	xx (xx, xx)	xx	xx	xx (xx, xx)	xx (xx, xx)
		Pulmonary	xx	xx	xx (xx, xx)	xx	xx	xx (xx, xx)	xx (xx, xx)
	Immunosuppression	Yes	xx	xx	xx (xx, xx)	xx	xx	xx (xx, xx)	xx (xx, xx)
		No	xx	xx	xx (xx, xx)	xx	xx	xx (xx, xx)	xx (xx, xx)
	Duration of Initial Bacteremia (Days)	< 2	xx	xx	xx (xx, xx)	xx	xx	xx (xx, xx)	xx (xx, xx)
		2-4	xx	xx	xx (xx, xx)	xx	xx	xx (xx, xx)	xx (xx, xx)
		>4	xx	xx	xx (xx, xx)	xx	xx	xx (xx, xx)	xx (xx, xx)
Day 70	Baseline Pathogen	MRSA	xx	xx	xx (xx, xx)	xx	xx	xx (xx, xx)	xx (xx, xx)
		MSSA	xx	xx	xx (xx, xx)	xx	xx	xx (xx, xx)	xx (xx, xx)
	PWID Status	PWID	xx	xx	xx (xx, xx)	xx	xx	xx (xx, xx)	xx (xx, xx)
		Non-PWID	xx	xx	xx (xx, xx)	xx	xx	xx (xx, xx)	xx (xx, xx)
	Infectious Disease Consultation	Yes	xx	xx	xx (xx, xx)	xx	xx	xx (xx, xx)	xx (xx, xx)
		No	xx	xx	xx (xx, xx)	xx	xx	xx (xx, xx)	xx (xx, xx)
	Underlying Site of Infection	Endovascular	xx	xx	xx (xx, xx)	xx	xx	xx (xx, xx)	xx (xx, xx)
		Bone and Joint	xx	xx	xx (xx, xx)	xx	xx	xx (xx, xx)	xx (xx, xx)
		Skin	xx	xx	xx (xx, xx)	xx	xx	xx (xx, xx)	xx (xx, xx)
		Pulmonary	xx	xx	xx (xx, xx)	xx	xx	xx (xx, xx)	xx (xx, xx)
	Immunosuppression	Yes	xx	xx	xx (xx, xx)	xx	xx	xx (xx, xx)	xx (xx, xx)
		No	xx	xx	xx (xx, xx)	xx	xx	xx (xx, xx)	xx (xx, xx)
	Duration of Initial Bacteremia (Days)	< 2	xx	xx	xx (xx, xx)	xx	xx	xx (xx, xx)	xx (xx, xx)
		2-4	xx	xx	xx (xx, xx)	xx	xx	xx (xx, xx)	xx (xx, xx)
		>4	xx	xx	xx (xx, xx)	xx	xx	xx (xx, xx)	xx (xx, xx)

N = Number of subjects in the ITT analysis population, treatment group and subgroup category
n = Number of subjects in the corresponding analysis population and treatment group who achieved clinical efficacy.
PWID = Persons Who Inject Drugs.
[a]95% CIs were obtained from linear regression model with IPW.
Note: IPW was used to handle missing values of clinical efficacy.

Table 39: Subgroup Analysis of Clinical Efficacy by Timepoint – mITT Analysis Population

This table will repeat Table 38 limited to the mITT population.

Table 40: Subgroup Analysis of Clinical Efficacy by Timepoint – CE Analysis Population

Timepoint	Variable	Level	Dalbavancin			Standard of Care			
			N	n	Proportion % (95% CI)[a]	N	n	Proportion % (95% CI)[a]	Difference in Proportion % (95% CI)[a]
Day 42	Baseline Pathogen	MRSA	xx	xx	xx (xx, xx)	xx	xx	xx (xx, xx)	xx (xx, xx)
		MSSA	xx	xx	xx (xx, xx)	xx	xx	xx (xx, xx)	xx (xx, xx)
	PWID Status	PWID	xx	xx	xx (xx, xx)	xx	xx	xx (xx, xx)	xx (xx, xx)
		Non-PWID	xx	xx	xx (xx, xx)	xx	xx	xx (xx, xx)	xx (xx, xx)
	Infectious Disease Consultation	Yes	xx	xx	xx (xx, xx)	xx	xx	xx (xx, xx)	xx (xx, xx)
		No	xx	xx	xx (xx, xx)	xx	xx	xx (xx, xx)	xx (xx, xx)
	Underlying Site of Infection	Endovascular	xx	xx	xx (xx, xx)	xx	xx	xx (xx, xx)	xx (xx, xx)
		Bone and Joint	xx	xx	xx (xx, xx)	xx	xx	xx (xx, xx)	xx (xx, xx)
		Skin	xx	xx	xx (xx, xx)	xx	xx	xx (xx, xx)	xx (xx, xx)
		Pulmonary	xx	xx	xx (xx, xx)	xx	xx	xx (xx, xx)	xx (xx, xx)
	Immunosuppression	Yes	xx	xx	xx (xx, xx)	xx	xx	xx (xx, xx)	xx (xx, xx)
		No	xx	xx	xx (xx, xx)	xx	xx	xx (xx, xx)	xx (xx, xx)
	Duration of Initial Bacteremia (Days)	< 2	xx	xx	xx (xx, xx)	xx	xx	xx (xx, xx)	xx (xx, xx)
		2-4	xx	xx	xx (xx, xx)	xx	xx	xx (xx, xx)	xx (xx, xx)
		>4	xx	xx	xx (xx, xx)	xx	xx	xx (xx, xx)	xx (xx, xx)
Day 70	Baseline Pathogen	MRSA	xx	xx	xx (xx, xx)	xx	xx	xx (xx, xx)	xx (xx, xx)
		MSSA	xx	xx	xx (xx, xx)	xx	xx	xx (xx, xx)	xx (xx, xx)
	PWID Status	PWID	xx	xx	xx (xx, xx)	xx	xx	xx (xx, xx)	xx (xx, xx)
		Non-PWID	xx	xx	xx (xx, xx)	xx	xx	xx (xx, xx)	xx (xx, xx)
	Infectious Disease Consultation	Yes	xx	xx	xx (xx, xx)	xx	xx	xx (xx, xx)	xx (xx, xx)
		No	xx	xx	xx (xx, xx)	xx	xx	xx (xx, xx)	xx (xx, xx)
	Underlying Site of Infection	Endovascular	xx	xx	xx (xx, xx)	xx	xx	xx (xx, xx)	xx (xx, xx)
		Bone and Joint	xx	xx	xx (xx, xx)	xx	xx	xx (xx, xx)	xx (xx, xx)
		Skin	xx	xx	xx (xx, xx)	xx	xx	xx (xx, xx)	xx (xx, xx)
		Pulmonary	xx	xx	xx (xx, xx)	xx	xx	xx (xx, xx)	xx (xx, xx)
	Immunosuppression	Yes	xx	xx	xx (xx, xx)	xx	xx	xx (xx, xx)	xx (xx, xx)
		No	xx	xx	xx (xx, xx)	xx	xx	xx (xx, xx)	xx (xx, xx)
	Duration of Initial Bacteremia (Days)	< 2	xx	xx	xx (xx, xx)	xx	xx	xx (xx, xx)	xx (xx, xx)
		2-4	xx	xx	xx (xx, xx)	xx	xx	xx (xx, xx)	xx (xx, xx)
		>4	xx	xx	xx (xx, xx)	xx	xx	xx (xx, xx)	xx (xx, xx)

N = Number of subjects in the CE analysis population, treatment group and subgroup category.
PWID = Persons Who Inject Drugs.
[a] 95% CIs were obtained from linear regression model.

Table 41: Summary of Clinical DOOR Components at Day 70 – ITT Analysis Population

DOOR Component	Dalbavancin			Standard of Care			DOOR Probability
	N	n	%	N	n	%	% (95% CI)[a]
Clinical Failure	x	x	x	x	x	x	xx (x.x, x.x)
Infectious Complication	x	x	x	x	x	x	xx (x.x, x.x)
SAEs	x	x	x	x	x	x	xx (x.x, x.x)
AEs Leading to Study Drug Discontinuation	x	x	x	x	x	x	xx (x.x, x.x)
All-cause Mortality	x	x	x	x	x	x	xx (x.x, x.x)

N = Number of subjects in the ITT population with non-missing values for the corresponding DOOR component.
n = Number of subjects with who experienced the corresponding DOOR component.
[a] 95% CI for DOOR Probability obtained through method described in Halperin et. al.

Table 42: Summary of Clinical DOOR Components at Day 70 – mITT Analysis Population

This table will repeat Table 41 limited to the mITT population.

Table 43: Summary of Clinical DOOR Components at Day 70 – CE Analysis Population

This table will repeat Table 41 limited to the CE population.

Table 44: Summary of Clinical DOOR Components at Day 42 – ITT Analysis Population

This table will repeat Table 41 limited to the ITT population at Day 42.

Table 45: Summary of Clinical DOOR Components at Day 42 – mITT Analysis Population

This table will repeat Table 41 limited to the mITT population at Day 42.

Table 46: Summary of Clinical DOOR Components at Day 42 – CE Analysis Population

This table will repeat Table 41 limited to the CE population at Day 42.

RESTRICTED

Table 47: Summary of DOOR by Component at Day 70 – ITT Analysis Population

DOOR	Clinical Failure	Infectious Complication	SAEs or AEs Leading to Study Drug Discontinuation	Dalbavancin (N=X)		Standard of Care (N=X)	
				n	%	N	%
1 - Alive with No Events	-	-	-				
2 - Alive with One Event	Yes	-	-				
	-	Yes	-				
	-	-	Yes				
3 - Alive with Two Events	Yes	Yes	-				
	Yes	-	Yes				
	-	Yes	Yes				
4 - Alive with Three Events	Yes	Yes	Yes				
5 - Death	-	-	-				
	Yes	-	-				
	-	Yes					
	-	-	Yes				
	Yes	Yes	-				
	Yes	-	Yes				
	-	Yes	Yes				
	Yes	Yes	Yes				
Note: Events that are both infectious complications and SAEs or AEs leading to study drug discontinuation count twice towards the DOOR.							

Table 48: Summary of DOOR by Component at Day 42 – ITT Analysis Population

This table will repeat Table 47 at Day 42.

Table 49: Analysis of Clinical Failure at Day 70 Using Weighted GEE and Weighted GLMM Models- ITT Analysis Population

	Dalbavancin		Standard of Care			
**Model**	**%**	**95% CI**	**%**	**95% CI**	**Difference (95% CI)**	**P-Value**
Weighted GEE	x.xxx	(x.xxx, x.xxx)	x.xx	(x.xx, x.xx)	(x.xxx, x.xxx)	x.xxx
Weighted GLMM	x.xxx	(x.xxx, x.xxx)	x.xx	(x.xx, x.xx)	(x.xxx, x.xxx)	x.xxx

Table 50: Analysis of Microbiologic Success Using ITT or mITT Analysis Populations

Timepoint	Analysis Population	Model	Statistic	Dalbavancin (N=X)	Standard of Care (N=X)
Day 42	ITT	IPW	Subjects with non-missing microbiologic success on Day 42– n (%)	x (x)	x (x)
			Subjects with missing microbiologic success on Day 42 – n (%)	x (x)	x (x)
			Percent rate of microbiologic success at 42 (95% CI)[a]	xx (xx, xx)	xx (xx, xx)
			Difference in rates (percent) of microbiologic success, dalbavancin relative to standard of care (95% CI)[a]	xx (xx, xx)	-
		Multiple Imputation	Subjects with non-missing microbiologic success on Day 42– n (%)	x (x)	x (x)
			Subjects with missing microbiologic success on Day 42 – n (%)	x (x)	x (x)
			Percent rate of microbiologic success at 42 (95% CI)[a]	xx (xx, xx)	xx (xx, xx)
			Difference in rates (percent) of microbiologic success, dalbavancin relative to standard of care (95% CI)[a]	xx (xx, xx)	-
	mITT	IPW	Subjects with non-missing microbiologic success on Day 42– n (%)	x (x)	x (x)
			Subjects with missing microbiologic success on Day 42 – n (%)	x (x)	x (x)
			Percent rate of microbiologic success at 42 (95% CI)[a]	xx (xx, xx)	xx (xx, xx)
			Difference in rates (percent) of microbiologic success, dalbavancin relative to standard of care (95% CI)[a]	xx (xx, xx)	-
		Multiple Imputation	Subjects with non-missing microbiologic success on Day 42– n (%)	x (x)	x (x)
			Subjects with missing microbiologic success on Day 42 – n (%)	x (x)	x (x)
			Percent rate of microbiologic success at 42 (95% CI)[a]	xx (xx, xx)	xx (xx, xx)
			Difference in rates (percent) of microbiologic success, dalbavancin relative to standard of care (95% CI)[a]	xx (xx, xx)	-
Day 70	ITT	IPW	Subjects with non-missing microbiologic success on Day 70– n (%)	x (x)	x (x)
			Subjects with missing microbiologic success on Day 70 – n (%)	x (x)	x (x)
			Percent rate of microbiologic success at Day 70 (95% CI)[a]	xx (xx, xx)	xx (xx, xx)
			Difference in rates (percent) of microbiologic success, dalbavancin relative to standard of care (95% CI)[a]	xx (xx, xx)	-
		Multiple Imputation	Subjects with non-missing microbiologic success on Day 70– n (%)	x (x)	x (x)
			Subjects with missing microbiologic success on Day 70 – n (%)	x (x)	x (x)
			Percent rate of microbiologic success at Day 70 (95% CI)[a]	xx (xx, xx)	xx (xx, xx)
			Difference in rates (percent) of microbiologic success, dalbavancin relative to standard of care (95% CI)[a]	xx (xx, xx)	-

Table 50: Analysis of Microbiologic Success Using ITT or mITT Analysis Populations (continued)

	mITT	IPW	Subjects with non-missing microbiologic success on Day 70– n (%)	x (x)	x (x)
			Subjects with missing microbiologic success on Day 70 – n (%)	x (x)	x (x)
			Percent rate of microbiologic success at 70 (95% CI)[a]	xx (xx, xx)	xx (xx, xx)
			Difference in rates (percent) of microbiologic success, dalbavancin relative to standard of care (95% CI)[a]	xx (xx, xx)	-
		Multiple Imputation	Subjects with non-missing microbiologic success on Day 70– n (%)	x (x)	x (x)
			Subjects with missing microbiologic success on Day 70 – n (%)	x (x)	x (x)
			Percent rate of microbiologic success at Day 70 (95% CI)[a]	xx (xx, xx)	xx (xx, xx)
			Difference in rates (percent) of microbiologic success, dalbavancin relative to standard of care (95% CI)[a]	xx (xx, xx)	-

N = Number of subjects in the corresponding analysis Population. Multiple imputation and IPW were used to handle missing values of microbiological success.
[a] 95% CIs were obtained from linear regression model following multiple imputation or IPW.

Table 51: Analysis of Microbiologic Success at Day 42 and Day 70 Using CE Analysis Population

Timepoint	Treatment Group	Proportion of Microbiologic Success			Difference in Proportion		
		n	%	95% CI[a]	%	95% CI[a]	Miettinen–Nurminen 95% CI
Day 42	Dalbavancin (N=X)	x	xx	xx, xx	xx	xx, xx	xx, xx
	Standard of Care (N=X)	x	xx	xx, xx	Reference	-	-
Day 70	Dalbavancin (N=X)	x	xx	xx, xx	xx	xx, xx	xx, xx
	Standard of Care (N=X)	x	xx	xx, xx	Reference	-	-

N = Number of subjects in the CE analysis population at the corresponding timepoint.
n = Number of subjects in CE population with microbiologic success at the corresponding timepoint.
[a] 95% CI obtained from linear regression.

Table 52: Subgroup Analysis of Microbiologic Success by Timepoint – ITT Analysis Population

Timepoint	Variable	Level	Dalbavancin			Standard of Care			Difference in Proportion % (95% CI)[a]
			N	n	Proportion % (95% CI)[a]	N	n	Proportion % (95% CI)[a]	
Day 42	Baseline Pathogen	MRSA	xx	xx	xx (xx, xx)	xx	xx	xx (xx, xx)	xx (xx, xx)
		MSSA	xx	xx	xx (xx, xx)	xx	xx	xx (xx, xx)	xx (xx, xx)
	PWID Status	PWID	xx	xx	xx (xx, xx)	xx	xx	xx (xx, xx)	xx (xx, xx)
		Non-PWID	xx	xx	xx (xx, xx)	xx	xx	xx (xx, xx)	xx (xx, xx)
	Infectious Disease Consultation	Yes	xx	xx	xx (xx, xx)	xx	xx	xx (xx, xx)	xx (xx, xx)
		No	xx	xx	xx (xx, xx)	xx	xx	xx (xx, xx)	xx (xx, xx)
	Underlying Site of Infection	Endovascular	xx	xx	xx (xx, xx)	xx	xx	xx (xx, xx)	xx (xx, xx)
		Bone and Joint	xx	xx	xx (xx, xx)	xx	xx	xx (xx, xx)	xx (xx, xx)
		Skin	xx	xx	xx (xx, xx)	xx	xx	xx (xx, xx)	xx (xx, xx)
		Pulmonary	xx	xx	xx (xx, xx)	xx	xx	xx (xx, xx)	xx (xx, xx)
	Immunosuppression	Yes	xx	xx	xx (xx, xx)	xx	xx	xx (xx, xx)	xx (xx, xx)
		No	xx	xx	xx (xx, xx)	xx	xx	xx (xx, xx)	xx (xx, xx)
	Duration of Initial Bacteremia (Days)	< 2	xx	xx	xx (xx, xx)	xx	xx	xx (xx, xx)	xx (xx, xx)
		2-4	xx	xx	xx (xx, xx)	xx	xx	xx (xx, xx)	xx (xx, xx)
		>4	xx	xx	xx (xx, xx)	xx	xx	xx (xx, xx)	xx (xx, xx)
Day 70	Baseline Pathogen	MRSA	xx	xx	xx (xx, xx)	xx	xx	xx (xx, xx)	xx (xx, xx)
		MSSA	xx	xx	xx (xx, xx)	xx	xx	xx (xx, xx)	xx (xx, xx)
	PWID Status	PWID	xx	xx	xx (xx, xx)	xx	xx	xx (xx, xx)	xx (xx, xx)
		Non-PWID	xx	xx	xx (xx, xx)	xx	xx	xx (xx, xx)	xx (xx, xx)
	Infectious Disease Consultation	Yes	xx	xx	xx (xx, xx)	xx	xx	xx (xx, xx)	xx (xx, xx)
		No	xx	xx	xx (xx, xx)	xx	xx	xx (xx, xx)	xx (xx, xx)
	Underlying Site of Infection	Endovascular	xx	xx	xx (xx, xx)	xx	xx	xx (xx, xx)	xx (xx, xx)
		Bone and Joint	xx	xx	xx (xx, xx)	xx	xx	xx (xx, xx)	xx (xx, xx)
		Skin	xx	xx	xx (xx, xx)	xx	xx	xx (xx, xx)	xx (xx, xx)
		Pulmonary	xx	xx	xx (xx, xx)	xx	xx	xx (xx, xx)	xx (xx, xx)
	Immunosuppression	Yes	xx	xx	xx (xx, xx)	xx	xx	xx (xx, xx)	xx (xx, xx)
		No	xx	xx	xx (xx, xx)	xx	xx	xx (xx, xx)	xx (xx, xx)
	Duration of Initial Bacteremia (Days)	< 2	xx	xx	xx (xx, xx)	xx	xx	xx (xx, xx)	xx (xx, xx)
		2-4	xx	xx	xx (xx, xx)	xx	xx	xx (xx, xx)	xx (xx, xx)
		>4	xx	xx	xx (xx, xx)	xx	xx	xx (xx, xx)	xx (xx, xx)

N = Number of subjects in the ITT analysis population, treatment group and subgroup category. IPW was used to handle missing values of microbiological success.
PWID = Persons Who Inject Drugs.
[a] 95% CIs were obtained from linear regression model with IPW.

Table 53: Subgroup Analysis of Microbiologic Success by Timepoint – mITT Analysis Population

This table will repeat Table 52 limited to the mITT population.

Table 54: Subgroup Analysis of Microbiologic Success by Timepoint – CE Analysis Population

Timepoint	Variable	Level	Dalbavancin N	Dalbavancin n	Dalbavancin Proportion % (95% CI)[a]	Standard of Care N	Standard of Care n	Standard of Care Proportion % (95% CI)[a]	Difference in Proportions % (95% CI)[a]
Day 42	Baseline Pathogen	MRSA	xx	xx	xx (xx, xx)	xx	xx	xx (xx, xx)	xx (xx, xx)
		MSSA	xx	xx	xx (xx, xx)	xx	xx	xx (xx, xx)	xx (xx, xx)
	PWID Status	PWID	xx	xx	xx (xx, xx)	xx	xx	xx (xx, xx)	xx (xx, xx)
		Non-PWID	xx	xx	xx (xx, xx)	xx	xx	xx (xx, xx)	xx (xx, xx)
	Infectious Disease Consultation	Yes	xx	xx	xx (xx, xx)	xx	xx	xx (xx, xx)	xx (xx, xx)
		No	xx	xx	xx (xx, xx)	xx	xx	xx (xx, xx)	xx (xx, xx)
	Underlying Site of Infection	Endovascular	xx	xx	xx (xx, xx)	xx	xx	xx (xx, xx)	xx (xx, xx)
		Bone and Joint	xx	xx	xx (xx, xx)	xx	xx	xx (xx, xx)	xx (xx, xx)
		Skin	xx	xx	xx (xx, xx)	xx	xx	xx (xx, xx)	xx (xx, xx)
		Pulmonary	xx	xx	xx (xx, xx)	xx	xx	xx (xx, xx)	xx (xx, xx)
	Immunosuppression	Yes	xx	xx	xx (xx, xx)	xx	xx	xx (xx, xx)	xx (xx, xx)
		No	xx	xx	xx (xx, xx)	xx	xx	xx (xx, xx)	xx (xx, xx)
	Duration of Initial Bacteremia (Days)	< 2	xx	xx	xx (xx, xx)	xx	xx	xx (xx, xx)	xx (xx, xx)
		2-4	xx	xx	xx (xx, xx)	xx	xx	xx (xx, xx)	xx (xx, xx)
		>4	xx	xx	xx (xx, xx)	xx	xx	xx (xx, xx)	xx (xx, xx)
Day 70	Baseline Pathogen	MRSA	xx	xx	xx (xx, xx)	xx	xx	xx (xx, xx)	xx (xx, xx)
		MSSA	xx	xx	xx (xx, xx)	xx	xx	xx (xx, xx)	xx (xx, xx)
	PWID Status	PWID	xx	xx	xx (xx, xx)	xx	xx	xx (xx, xx)	xx (xx, xx)
		Non-PWID	xx	xx	xx (xx, xx)	xx	xx	xx (xx, xx)	xx (xx, xx)
	Infectious Disease Consultation	Yes	xx	xx	xx (xx, xx)	xx	xx	xx (xx, xx)	xx (xx, xx)
		No	xx	xx	xx (xx, xx)	xx	xx	xx (xx, xx)	xx (xx, xx)
	Underlying Site of Infection	Endovascular	xx	xx	xx (xx, xx)	xx	xx	xx (xx, xx)	xx (xx, xx)
		Bone and Joint	xx	xx	xx (xx, xx)	xx	xx	xx (xx, xx)	xx (xx, xx)
		Skin	xx	xx	xx (xx, xx)	xx	xx	xx (xx, xx)	xx (xx, xx)
		Pulmonary	xx	xx	xx (xx, xx)	xx	xx	xx (xx, xx)	xx (xx, xx)
	Immunosuppression	Yes	xx	xx	xx (xx, xx)	xx	xx	xx (xx, xx)	xx (xx, xx)
		No	xx	xx	xx (xx, xx)	xx	xx	xx (xx, xx)	xx (xx, xx)
	Duration of Initial Bacteremia (Days)	< 2	xx	xx	xx (xx, xx)	xx	xx	xx (xx, xx)	xx (xx, xx)
		2-4	xx	xx	xx (xx, xx)	xx	xx	xx (xx, xx)	xx (xx, xx)
		>4	xx	xx	xx (xx, xx)	xx	xx	xx (xx, xx)	xx (xx, xx)

N = Number of subjects in the CE analysis population, treatment group and subgroup category.
PWID = Persons Who Inject Drugs.
[a] 95% CIs were obtained from linear regression model without multiple imputation.

Table 55: Distribution of DOOR at Day 42 by Subgroup Categories – ITT Analysis Population

Subgroup	Subgroup Category	DOOR Category	Dalbavancin (N=X)		Standard of Care (N=X)		All Subjects (N=X)	
			n	%	n	%	n	%
Baseline Pathogen	MRSA	Any DOOR Category	x	xx	x	xx	**x**	**100**
		Alive with no events	x	xx	x	xx	x	xx
		Alive with 1 event						
		Alive with 2 events						
		Alive with 3 events						
		Death						
	MSSA	Any DOOR Category						
		Alive with no events						
		Alive with 1 event						
		Alive with 2 events						
		Alive with 3 events						
		Death						
PWID Status	PWID	Repeat for all DOOR as above						
	Non-PWID							
Infectious Disease Consultation	Yes							
	No							
Underlying Site of Infection	Endovascular							
	Bone and Joint							
	Skin							
	Pulmonary							
Immunosuppression	Yes							
	No							
Duration of Initial Bacteremia (Days)	< 2							
	2-4							
	>4							

N = Number of subjects in the ITT analysis population.
n = Number of subjects in the given ITT population with non-missing values of DOOR.
Note: Events that are both infectious complications and SAEs or AEs leading to study drug discontinuation count twice towards the DOOR.

Table with similar format:

Table 56: Distribution of DOOR at Day 70 by Subgroup Categories – ITT Analysis Population

Table 57: Summary Statistics of QoL Scores from the ARLG Bloodstream Infection QoL Measure at Day 42 and Day 70 Using ITT, mITT, and CE Analysis Populations

			QoL Score					Change from Baseline				
Analysis Population	Time Point	Treatment Group	n	Mean	Standard Deviation	Median	Min, Max	n	Mean	Standard Deviation	Median	Min, Max
ITT	Baseline	Dalbavancin (N=X)	x	xx.x	xx.x	xx.x	xx.x, xx.x	NA	NA	NA	NA	NA
		Standard of Care (N=X)	x	xx.x	xx.x	xx.x	xx.x, xx.x	NA	NA	NA	NA	NA
	Day 42	Dalbavancin (N=X)	x	xx.x	xx.x	xx.x	xx.x, xx.x	x	xx.x	xx.x	xx.x	xx.x, xx.x
		Standard of Care (N=X)	x	xx.x	xx.x	xx.x	xx.x, xx.x	x	xx.x	xx.x	xx.x	xx.x, xx.x
	Day 70	Dalbavancin (N=X)	x	xx.x	xx.x	xx.x	xx.x, xx.x	x	xx.x	xx.x	xx.x	xx.x, xx.x
		Standard of Care (N=X)	x	xx.x	xx.x	xx.x	xx.x, xx.x	x	xx.x	xx.x	xx.x	xx.x, xx.x
mITT	Baseline	Dalbavancin (N=X)	x	xx.x	xx.x	xx.x	xx.x, xx.x	NA	NA	NA	NA	NA
		Standard of Care (N=X)	x	xx.x	xx.x	xx.x	xx.x, xx.x	NA	NA	NA	NA	NA
	Day 42	Dalbavancin (N=X)	x	xx.x	xx.x	xx.x	xx.x, xx.x	x	xx.x	xx.x	xx.x	xx.x, xx.x
		Standard of Care (N=X)	x	xx.x	xx.x	xx.x	xx.x, xx.x	x	xx.x	xx.x	xx.x	xx.x, xx.x
	Day 70	Dalbavancin (N=X)	x	xx.x	xx.x	xx.x	xx.x, xx.x	x	xx.x	xx.x	xx.x	xx.x, xx.x
		Standard of Care (N=X)	x	xx.x	xx.x	xx.x	xx.x, xx.x	x	xx.x	xx.x	xx.x	xx.x, xx.x
CE	Baseline	Dalbavancin (N=X)	x	xx.x	xx.x	xx.x	xx.x, xx.x	NA	NA	NA	NA	NA
		Standard of Care (N=X)	x	xx.x	xx.x	xx.x	xx.x, xx.x	NA	NA	NA	NA	NA
	Day 42	Dalbavancin (N=X)	x	xx.x	xx.x	xx.x	xx.x, xx.x	x	xx.x	xx.x	xx.x	xx.x, xx.x
		Standard of Care (N=X)	x	xx.x	xx.x	xx.x	xx.x, xx.x	x	xx.x	xx.x	xx.x	xx.x, xx.x
	Day 70	Dalbavancin (N=X)	x	xx.x	xx.x	xx.x	xx.x, xx.x	x	xx.x	xx.x	xx.x	xx.x, xx.x
		Standard of Care (N=X)	x	xx.x	xx.x	xx.x	xx.x, xx.x	x	xx.x	xx.x	xx.x	xx.x, xx.x

N = Number of subjects in the given analysis population.
n = Number of subjects in the given analysis population with non-missing QoL score values at the timepoint of interest. For the change from baseline, n represents the number of subjects in the given analysis population with non-missing values at baseline and at the timepoint being assessed.
Note: Baseline QoL standardized score is obtained from the questions arising from the PROMIS physical function item bank (PROMIS Item Bank v2.0, short form 6b) item bank on the ARLG Bloodstream Infection QoL.

Table 58: Summary Statistics of QoL Scores Using the EQ-5D-5L Instrument at Day 42 and Day 70 Using ITT, mITT, and CE Analysis Populations

This table will be similar to Table 57.

Table 59: Summary Statistics of QoL Scores Using the PROMIS Global Health Short Form at Day 42 and Day 70 Using ITT, mITT, and CE Analysis Populations

This table will be similar to Table 57.

Table 60: Predictive Interval Plots (PIPS) Statistics for the Probability of Higher DOOR in the Dalbavancin Group at Day 70 – ITT Analysis Population

[Implementation Note: this table will only be included in the interim analysis.]

Assumption	Current 95% CI	Width of Current 95% CI	Width of Predicted 95% CI Median [Q1, Q3]	Probability to Reject Null Hypothesis[a]
Observed Trend	(x.xx – x.xx)	x.xx	x.xx (x.xx – x.xx)	x.xx
Null Hypothesis	(x.xx – x.xx)	x.xx	x.xx (x.xx – x.xx)	x.xx
Alternative Hypothesis	(x.xx – x.xx)	x.xx	x.xx (x.xx – x.xx)	x.xx
Best case scenario for remaining outcome	(x.xx – x.xx)	x.xx	x.xx (x.xx – x.xx)	x.xx
Best case scenario for remaining outcome	(x.xx – x.xx)	x.xx	x.xx (x.xx – x.xx)	x.xx

Q1 = 25th percentile. Q3 = 75th percentile.
[a] Probability to Reject Null Hypothesis = proportion of PIs simulated that have a lower bound greater than 0.50.
Note: Statistics related to the PIPS are also presented in Figure 26.

Table 61: Predictive Interval Plots (PIPS) Statistics for the Rates of Clinical Efficacy at Day 70 – ITT Analysis Population

This table will be similar to Table 60.

Implementation Note: this table will only be included in the interim analysis.

Table 62: Number and Percentage of Subjects with Late Recurrence - Osteomyelitis Population

Analysis Group	Statistic	Dalbavancin (N=X)	Standard of Care (N=X)
Osteomyelitis Population	Number of subjects with late recurrence	x	x
	Percent rate of late recurrence (95% CI)[a]	xx (xx, xx)	xx (xx, xx)
	Difference in rates (percent) late recurrence, dalbavancin relative to standard of care (95% CI)[b]	xx (xx, xx)	-
Sensitivity Osteomyelitis Population	Number of subjects with late recurrence	x	x
	Percent rate of late recurrence (95% CI)[a]	xx (xx, xx)	xx (xx, xx)
	Difference in rates (percent) late recurrence, dalbavancin relative to standard of care (95% CI)[b]	xx (xx, xx)	-

N = Number of subjects in the diagnosed with osteomyelitis.
[a] 95% CI for proportions calculated using the Wilson method.
[b] 95% CI for the difference in proportions, dalbavancin relative to standard of care, calculated using the Miettinen–Nurminen method.
Note: The osteomyelitis population includes subjects in the ITT population diagnosed with osteomyelitis for whom consent and data were obtained for Visit 7. The sensitivity osteomyelitis population excludes subjects from the Osteomyelitis Population those subjects for whom consent was obtained retroactively. XX (of NN) and YY (of MM) subjects with osteomyelitis in the Dalbavancin and Standard of Care Arms, respectively, became eligible for Visit 7 data collection under protocol version 3.0 and re-consented to Visit 7 data collection.

Programming Note: In the Note above:

XX = subjects treated with Dalbavancin who became eligible for Visit 7 data collection under protocol version 3.0 **and** re-consented Visit 7 data collection.

NN = subjects treated with Dalbavancin who became eligible for Visit 7 data collection under protocol version 3.0.

YY = subjects treated with Standard of Care who became eligible for Visit 7 data collection under protocol version 3.0 **and** re-consented Visit 7 data collection.

MM = subjects treated with Standard of Care who became eligible for Visit 7 data collection under protocol version 3.0.

14.3 Safety Data

14.3.1 Displays of Adverse Events

Table 63: Number and Percentage of Subjects reporting SAEs, AEs Leading Discontinuation, Grade 3 or Higher AEs, AESIs, and ALT/AST Elevations by Treatment Group - Safety Population

[Implementation note: Sort this table by decreasing frequency for the treatment group.]

	Dalbavancin (N=X)			Standard of Care (N=X)			All Subjects (N=X)		
**Adverse Event Type**	**n**	**%**	**Events**	**n**	**%**	**Events**	**n**	**%**	**Events**
SAEs	x	x	x	x	x	x	x	x	x
AE Leading to Study Drug Discontinuation	x	x	x	x	x	x	x	x	x
Grade 3 or Higher AEs	x	x	x	x	x	x	x	x	x
AESIs	x	x	x	x	x	x	x	x	x
AST/ALT Elevation[a]	x	x	x	x	x	x	x	x	x

N = Number of subjects in the Safety Population.
[a] Occurrence of AST/ALT elevations >3x upper limit of normal (ULN) from first dose of study product through follow-up period.

Table 64: Number and Percentage of subjects reporting SAEs, AEs Leading Discontinuation, Grade 3 or Higher AEs, AESIs, and ALT/AST Elevations by Standard of Care Antibiotics – Standard of Care Arm, Safety Population

[Implementation note: If any other standard of care antibiotics not listed here are administered, extend the table to add more columns for new SOC antibiotics.]

	Cefazolin (N=X)			Nafcillin (N=X)			Oxacillin (N=X)			Vancomycin (N=X)			Daptomycin (N=X)		
**Adverse Event Type**	**n**	**%**	**Events**	**n**	**%**	**Events**	**n**	**%**	**Events**	**n**	**%**	**Events**	**n**	**%**	**Events**
SAEs	x	x	x	x	x	x	x	x	x	x	x	x	x	x	x
AE Leading to Study Drug Discontinuation	x	x	x	x	x	x	x	x	x	x	x	x	x	x	x
Grade 3 or Higher AEs	x	x	x	x	x	x	x	x	x	x	x	x	x	x	x
AESIs	x	x	x	x	x	x	x	x	x	x	x	x	x	x	x
AST/ALT Elevation[a]	x	x	x	x	x	x	x	x	x	x	x	x	x	x	x

N = Number of subjects in the Safety Population who received standard of care antibiotics.
[a] Occurrence of AST/ALT elevations >3x upper limit of normal (ULN) from first dose of study product through follow-up period.

14.3.1.2 Unsolicited Adverse Events

Table 65: Summary of Treatment-Emergent Adverse Events by MedDRA System Organ Class and Preferred Term, Relationship, and Treatment Group

MedDRA System Organ Class	Preferred Term	Dalbavancin (N=X)						Standard of Care (N=X)						All Subjects (N=X)					
		Related		Not Related		Total		Related		Not Related		Total		Related		Not Related		Total	
		n	%	n	%	n	%	n	%	n	%	n	%	n	%	n	%	n	%
Any SOC	Any PT	x	xx	x	xx	x	xx	x	xx	x	xx	x	xx	x	xx	x	xx	x	xx
SOC 1	PT 1	x	xx	x	xx	x	xx	x	xx	x	xx	x	xx	x	xx	x	xx	x	xx
	PT 2	x	xx	x	xx	x	xx	x	xx	x	xx	x	xx	x	xx	x	xx	x	xx
…	…																		

N = Number of subjects in the Safety Population.

Table 66: Summary of Treatment-Emergent Adverse Events by MedDRA System Organ Class and Preferred Term, Maximum Severity, Relationship, and Treatment Group

MedDRA System Organ Class	Preferred Term	Severity	Dalbavancin (N=X)						Standard of Care (N=X)						All Subjects (N=X)					
			Related		Not Related		Total		Related		Not Related		Total		Related		Not Related		Total	
			n	%	n	%	n	%	n	%	n	%	n	%	n	%	n	%	n	%
Any SOC	Any PT	Any Severity	x	xx	x	xx	x	xx	x	xx	x	xx	x	xx	x	xx	x	xx	x	xx
		Not Reported	x	xx	x	xx	x	xx	x	xx	x	xx	x	xx	x	xx	x	xx	x	xx
		Grade 1	x	xx	x	xx	x	xx	x	xx	x	xx	x	xx	x	xx	x	xx	x	xx
		Grade 2	x	xx	x	xx	x	xx	x	xx	x	xx	x	xx	x	xx	x	xx	x	xx
		Grade 3	x	xx	x	xx	x	xx	x	xx	x	xx	x	xx	x	xx	x	xx	x	xx
		Grade 4	x	xx	x	xx	x	xx	x	xx	x	xx	x	xx	x	xx	x	xx	x	xx
		Grade 5	x	xx	x	xx	x	xx	x	xx	x	xx	x	xx	x	xx	x	xx	x	xx
SOC 1	PT 1	Any Severity	x	xx	x	xx	x	xx	x	xx	x	xx	x	xx	x	xx	x	xx	x	xx
		Not Reported	x	xx	x	xx	x	xx	x	xx	x	xx	x	xx	x	xx	x	xx	x	xx
		Grade 1	x	xx	x	xx	x	xx	x	xx	x	xx	x	xx	x	xx	x	xx	x	xx
		Grade 2	x	xx	x	xx	x	xx	x	xx	x	xx	x	xx	x	xx	x	xx	x	xx
		Grade 3	x	xx	x	xx	x	xx	x	xx	x	xx	x	xx	x	xx	x	xx	x	xx
		Grade 4	x	xx	x	xx	x	xx	x	xx	x	xx	x	xx	x	xx	x	xx	x	xx
		Grade 5	x	xx	x	xx	x	xx	x	xx	x	xx	x	xx	x	xx	x	xx	x	xx
	PT 2	Any Severity	x	xx	x	xx	x	xx	x	xx	x	xx	x	xx	x	xx	x	xx	x	xx
		Not Reported	x	xx	x	xx	x	xx	x	xx	x	xx	x	xx	x	xx	x	xx	x	xx
		Grade 1	x	xx	x	xx	x	xx	x	xx	x	xx	x	xx	x	xx	x	xx	x	xx
		Grade 2	x	xx	x	xx	x	xx	x	xx	x	xx	x	xx	x	xx	x	xx	x	xx
		Grade 3	x	xx	x	xx	x	xx	x	xx	x	xx	x	xx	x	xx	x	xx	x	xx
		Grade 4	x	xx	x	xx	x	xx	x	xx	x	xx	x	xx	x	xx	x	xx	x	xx
		Grade 5	x	xx	x	xx	x	xx	x	xx	x	xx	x	xx	x	xx	x	xx	x	xx
…	…																			

N = Number of subjects in the Safety Population.

Table 67: Summary of Treatment-Emergent Adverse Events by MedDRA System Organ Class and High Level Group Term, Relationship, and Treatment Group

MedDRA System Organ Class	High Level Group Term	Dalbavancin (N=X)						Standard of Care (N=X)						All Subjects (N=X)					
		Related		Not Related		Total		Related		Not Related		Total		Related		Not Related		Total	
		n	%	n	%	n	%	n	%	n	%	n	%	n	%	n	%	n	%
Any SOC	Any HLGT	x	xx	x	xx	x	xx	x	xx	x	xx	x	xx	x	xx	x	xx	x	xx
	HLGT 1	x	xx	x	xx	x	xx	x	xx	x	xx	x	xx	x	xx	x	xx	x	xx
	HLGT 2	x	xx	x	xx	x	xx	x	xx	x	xx	x	xx	x	xx	x	xx	x	xx
	…																		
	Repeat for applicable HLGT																		
SOC 1	Any HLGT	x	xx	x	xx	x	xx	x	xx	x	xx	x	xx	x	xx	x	xx	x	xx
	HLGT 1	x	xx	x	xx	x	xx	x	xx	x	xx	x	xx	x	xx	x	xx	x	xx
	HLGT 2	x	xx	x	xx	x	xx	x	xx	x	xx	x	xx	x	xx	x	xx	x	xx
	…																		
	Repeat for applicable HLGT																		
SOC2	Any HLGT	x	xx	x	xx	x	xx	x	xx	x	xx	x	xx	x	xx	x	xx	x	xx
	HLGT 1	x	xx	x	xx	x	xx	x	xx	x	xx	x	xx	x	xx	x	xx	x	xx
	HLGT 2	x	xx	x	xx	x	xx	x	xx	x	xx	x	xx	x	xx	x	xx	x	xx
	…																		
	Repeat for applicable HLGT																		
….	….																		

N = Number of subjects in the Safety Population.

Table 68: Summary of Treatment-Emergent Adverse Events by MedDRA System Organ Class and High Level Group Term, Maximum Severity, Relationship, and Treatment Group

MedDRA System Organ Class	High Level Group Term	Severity	Dalbavancin (N=X)						Standard of Care (N=X)						All Subjects (N=X)					
			Related		Not Related		Total		Related		Not Related		Total		Related		Not Related		Total	
			n	%	n	%	n	%	n	%	n	%	n	%	n	%	n	%	n	%
Any SOC	Any HLGT	Any Severity	x	xx	x	xx	x	xx	x	xx	x	xx	x	xx	x	xx	x	xx	x	xx
		Not Reported	x	xx	x	xx	x	xx	x	xx	x	xx	x	xx	x	xx	x	xx	x	xx
		Grade 1	x	xx	x	xx	x	xx	x	xx	x	xx	x	xx	x	xx	x	xx	x	xx
		Grade 2	x	xx	x	xx	x	xx	x	xx	x	xx	x	xx	x	xx	x	xx	x	xx
		Grade 3	x	xx	x	xx	x	xx	x	xx	x	xx	x	xx	x	xx	x	xx	x	xx
		Grade 4	x	xx	x	xx	x	xx	x	xx	x	xx	x	xx	x	xx	x	xx	x	xx
		Grade 5	x	xx	x	xx	x	xx	x	xx	x	xx	x	xx	x	xx	x	xx	x	xx
SOC 1	HLGT 1	Any Severity	x	xx	x	xx	x	xx	x	xx	x	xx	x	xx	x	xx	x	xx	x	xx
		Not Reported	x	xx	x	xx	x	xx	x	xx	x	xx	x	xx	x	xx	x	xx	x	xx
		Grade 1	x	xx	x	xx	x	xx	x	xx	x	xx	x	xx	x	xx	x	xx	x	xx
		Grade 2	x	xx	x	xx	x	xx	x	xx	x	xx	x	xx	x	xx	x	xx	x	xx
		Grade 3	x	xx	x	xx	x	xx	x	xx	x	xx	x	xx	x	xx	x	xx	x	xx
		Grade 4	x	xx	x	xx	x	xx	x	xx	x	xx	x	xx	x	xx	x	xx	x	xx
		Grade 5	x	xx	x	xx	x	xx	x	xx	x	xx	x	xx	x	xx	x	xx	x	xx
	HLGT 2	Any Severity	x	xx	x	xx	x	xx	x	xx	x	xx	x	xx	x	xx	x	xx	x	xx
		Not Reported	x	xx	x	xx	x	xx	x	xx	x	xx	x	xx	x	xx	x	xx	x	xx
		Grade 1	x	xx	x	xx	x	xx	x	xx	x	xx	x	xx	x	xx	x	xx	x	xx
		Grade 2	x	xx	x	xx	x	xx	x	xx	x	xx	x	xx	x	xx	x	xx	x	xx
		Grade 3	x	xx	x	xx	x	xx	x	xx	x	xx	x	xx	x	xx	x	xx	x	xx
		Grade 4	x	xx	x	xx	x	xx	x	xx	x	xx	x	xx	x	xx	x	xx	x	xx
		Grade 5	x	xx	x	xx	x	xx	x	xx	x	xx	x	xx	x	xx	x	xx	x	xx
….	….																			

N = Number of subjects in the Safety Population.

Table 69: Summary of Treatment-Emergent Adverse Events by MedDRA System Organ Class, Maximum Severity, Relationship, and Treatment Group

MedDRA System Organ Class	Severity	Dalbavancin (N=X)						Standard of Care (N=X)						All Subjects (N=X)					
		Related		Not Related		Total		Related		Not Related		Total		Related		Not Related		Total	
		n	%	n	%	n	%	n	%	n	%	n	%	n	%	n	%	n	%
Any SOC	Any Severity	x	xx	x	xx	x	xx	x	xx	x	xx	x	xx	x	xx	x	xx	x	xx
	Not Reported	x	xx	x	xx	x	xx	x	xx	x	xx	x	xx	x	xx	x	xx	x	xx
	Grade 1	x	xx	x	xx	x	xx	x	xx	x	xx	x	xx	x	xx	x	xx	x	xx
	Grade 2	x	xx	x	xx	x	xx	x	xx	x	xx	x	xx	x	xx	x	xx	x	xx
	Grade 3	x	xx	x	xx	x	xx	x	xx	x	xx	x	xx	x	xx	x	xx	x	xx
	Grade 4	x	xx	x	xx	x	xx	x	xx	x	xx	x	xx	x	xx	x	xx	x	xx
	Grade 5	x	xx	x	xx	x	xx	x	xx	x	xx	x	xx	x	xx	x	xx	x	xx
SOC 1	Any Severity	x	xx	x	xx	x	xx	x	xx	x	xx	x	xx	x	xx	x	xx	x	xx
	Not Reported	x	xx	x	xx	x	xx	x	xx	x	xx	x	xx	x	xx	x	xx	x	xx
	Grade 1	x	xx	x	xx	x	xx	x	xx	x	xx	x	xx	x	xx	x	xx	x	xx
	Grade 2	x	xx	x	xx	x	xx	x	xx	x	xx	x	xx	x	xx	x	xx	x	xx
	Grade 3	x	xx	x	xx	x	xx	x	xx	x	xx	x	xx	x	xx	x	xx	x	xx
	Grade 4	x	xx	x	xx	x	xx	x	xx	x	xx	x	xx	x	xx	x	xx	x	xx
	Grade 5	x	xx	x	xx	x	xx	x	xx	x	xx	x	xx	x	xx	x	xx	x	xx
SOC 2	Any Severity	x	xx	x	xx	x	xx	x	xx	x	xx	x	xx	x	xx	x	xx	x	xx
	Not Reported	x	xx	x	xx	x	xx	x	xx	x	xx	x	xx	x	xx	x	xx	x	xx
	Grade 1	x	xx	x	xx	x	xx	x	xx	x	xx	x	xx	x	xx	x	xx	x	xx
	Grade 2	x	xx	x	xx	x	xx	x	xx	x	xx	x	xx	x	xx	x	xx	x	xx
	Grade 3	x	xx	x	xx	x	xx	x	xx	x	xx	x	xx	x	xx	x	xx	x	xx
	Grade 4	x	xx	x	xx	x	xx	x	xx	x	xx	x	xx	x	xx	x	xx	x	xx
	Grade 5	x	xx	x	xx	x	xx	x	xx	x	xx	x	xx	x	xx	x	xx	x	xx
….																			

N = Number of subjects in the Safety Population.

Table 70: Summary of AESIs by MedDRA System Organ Class, Maximum Severity, Relationship, and Treatment Group

This table will be similar to Table 69.

Table 71: Summary of Treatment-Emergent AEs, On-therapy SAEs, AESIs, and AEs Leading Discontinuation Occurring in 2% of Subjects in Any Treatment Group by MedDRA Preferred Term, and Treatment Group – Safety Population

[Implementation note: Sort this table by decreasing frequency for the treatment group.]

Preferred Term	Dalbavancin (N=X)			Standard of Care (N=X)			All Subjects (N=X)		
	n	%	Events	n	%	Events	n	%	Events
Any PT	x	x	x	x	x	x	x	x	x
PT1	x	x	x	x	x	x	x	x	x
PT2	x	x	x	x	x	x	x	x	x
Etc									
N = Number of subjects in the Safety Population.									

Table 72: Summary of Fatal on-Therapy SAEs by MedDRA Preferred Term, and Treatment Group – Safety Population

Preferred Term	Dalbavancin (N=X)			Standard of Care (N=X)			All Subjects (N=X)		
	n	%	Events	n	%	Events	n	%	Events
Any PT	x	x	x	x	x	x	x	x	x
PT1	x	x	x	x	x	x	x	x	x
PT2	x	x	x	x	x	x	x	x	x
…									

N = Number of subjects in the Safety Population.

Table 73: Summary of Fatal on-Therapy SAEs by MedDRA High Level Group Term, and Treatment Group – Safety Population

High Level Group Term	Dalbavancin (N=X)			Standard of Care (N=X)			All Subjects (N=X)		
	n	%	Events	n	%	Events	n	%	Events
Any HLGT	x	x	x	x	x	x	x	x	x
HLGT1	x	x	x	x	x	x	x	x	x
HLTG2	x	x	x	x	x	x	x	x	x
…									
N = Number of subjects in the Safety Population.									

14.3.2 Listing of Deaths, Other Serious and Significant Adverse Events

Table 74: Listing of Serious Adverse Events

Adverse Event	Study Day of AE Onset	Duration (Days)	Severity	Relationship to Study Treatment	If Not Related, Alternative Etiology	Action Taken with Study Treatment	Subject Discontinued Due to AE	Outcome	MedDRA System Organ Class	MedDRA High Level Group Term	MedDRA Preferred Term
**Treatment Group: , Subject ID: , AE Number:**											
Comments:											
**Treatment Group: , Subject ID: , AE Number:**											
Comments:											
Note: For additional details about SAEs, see Table: xx.											

Table 75: Listing of Adverse Events of Special Interest

This table will be similar to Table 74.

Table 76: Listing of Adverse Events Leading to Discontinuation

This table will be similar to Table 74.

Table 77: Listing of Subjects whose Outcome was Fatal During the Study

This table will be similar to Table 74.

Table 78: Listing of Treatment-Emergent Adverse Events

Adverse Event	Associated with Dose No.	No. of Days Post Associated Dose (Duration)	Severity	Relationship to Study Treatment	If Not Related, Alternative Etiology	Action Taken with Study Treatment	Subject Discontinued Due to AE	Outcome	MedDRA System Organ Class	MedDRA Preferred Term	MedDRA High Level Group Term
**Subject ID: , Treatment Group: , AE Number:**											
Comments:											
**Subject ID: , Treatment Group: , AE Number:**											
Comments:											

14.3.3 Narratives of Deaths, Other Serious and Significant Adverse Events

(Not included in SAP, but this is a placeholder for the CSR.)

14.3.4 Abnormal Laboratory Value Listings (by Subject)

Table 79: Listing of Potentially Clinically Significant Laboratory Results – Chemistry

Treatment Group	Subject ID	Sex	Age (years)	Planned Time Point	Actual Study Day	Laboratory Parameter (Units)	Result (Severity)

Notes: All laboratory results for a parameter are displayed for subjects with at least one PCS result for that parameter.
PCS Criteria is defined as follows for each parameter: Sodium (<130 mmol/L OR >150 mmol/L), Potassium (<3.0 mmol/L OR >5.5 mmol/L), Glucose (<55 mg/dL), Creatinine (Serum creatinine >1.5x baseline), Albumin (<3 g/dL), Alkaline phosphatase (>ULN if baseline was normal; >2.0 x baseline if baseline was abnormal), Total bilirubin (>ULN if baseline was normal; > 1.0 x baseline if baseline was abnormal), AST (>ULN if baseline was normal; >1.5 x baseline if baseline was abnormal), ALT (>ULN if baseline was normal; >1.5 x baseline if baseline was abnormal).

Programming Notes: sort by treatment group (dalbavancin first), then subject ID.

[Implementation Note: **If a subject has at least one PCS, list all their laboratory results for that laboratory parameter.** The criteria for PCS will be defined below. Laboratory parameters not included in this table will be ignored.

- Laboratory parameter (Potentially Clinically Significant Result Criteria)
- Sodium (<130 mmol/L OR >150 mmol/L)
- Potassium (<3.0 mmol/L OR >5.5 mmol/L)
- Glucose (<55 mg/dL)
- Creatinine (Serum creatinine >1.5x baseline)
- Albumin (<3 g/dL)
- Alkaline phosphatase (>ULN if baseline was normal; >2.0 x baseline if baseline was abnormal)
- Total bilirubin (>ULN if baseline was normal; > 1.0 x baseline if baseline was abnormal)
- AST (>ULN if baseline was normal; >1.5 x baseline if baseline was abnormal)
- ALT (>ULN if baseline was normal; >1.5 x baseline if baseline was abnormal)]

Table 80: Listing of Potentially Clinically Significant Laboratory Results – Hematology

If a subject has at least one PCS, list all their laboratory results for that laboratory parameter.]

Treatment Group	Subject ID	Sex	Age (years)	Planned Time Point	Actual Study Day	Laboratory Parameter (Units)	Result (Severity)

Notes: All laboratory results for a parameter are displayed for subjects with at least one PCS result for that parameter.
PCS Criteria is defined as follows for each parameter: Hemoglobin (<10 g/dL), Platelets (<75 /µL), White blood cell count (<3.0 x 10e9), Absolute neutrophil count (ANC) (<1500 x 10e9), Absolute lymphocyte count (ALC) (<800 x 10e9).

Programming Note: sort by treatment group (dalbavancin first), then subject ID.

[Implementation Note: **If a subject has at least one PCS, list all their laboratory results for that laboratory parameter.** The criteria for PCS will be defined as follows:

- Laboratory parameter (Potentially Clinically Significant Result Criteria)
- Hemoglobin (<10 g/dL)
- Platelets (<75 /µL)
- White blood cell count (<3.0 x 10e9)
- Absolute neutrophil count (ANC) (<1500 x 10e9)
- Absolute lymphocyte count (ALC) (<800 x 10e9)

14.3.5 Displays of Laboratory Results

14.3.5.1 Chemistry Results

Table 81: Number and Percentage of Subjects with Potentially Clinically Significant Post-Baseline Laboratory Values by Parameter and Treatment Group – Chemistry Parameters

Serum Chemistry Parameter	Dalbavancin			Standard of Care		
	N	n	%	N	n	%
Any Serum Chemistry Parameter	x	x	xx	x	x	xx
Sodium	x	x	xx	x	x	xx
Potassium	x	x	xx	x	x	xx
Glucose	x	x	xx	x	x	xx
Creatinine	x	x	xx	x	x	xx
Alkaline Phosphatase	x	x	xx	x	x	xx
Albumin	x	x	xx	x	x	xx
Total bilirubin	x	x	xx	x	x	xx
AST	x	x	xx	x	x	xx
ALT	x	x	xx	x	x	xx

PCS = Potentially Clinically Significant. PCS Criteria is defined as follows for each parameter: Sodium (<130 mmol/L OR >150 mmol/L), Potassium (<3.0 mmol/L OR >5.5 mmol/L), Glucose (<55 mg/dL), Creatinine (Serum creatinine >1.5x baseline), Albumin (<3 g/dL), Alkaline phosphatase (>ULN - 2.5 x ULN if baseline was normal; >2.0 - 2.5 x baseline if baseline was abnormal), Total bilirubin (>ULN – 1.5 x ULN if baseline was normal; > 1.0 – 1.5 x baseline if baseline was abnormal), AST (>ULN - 3.0 x ULN if baseline was normal; >1.5 - 3.0 x baseline if baseline was abnormal), ALT (>ULN if baseline was normal; >1.5 x baseline if baseline was abnormal).
N = Number of subjects in the Safety Population with available non-PCS baseline values and at least 1 post-baseline assessment.
n = Number of subjects with at least one PCS post-baseline laboratory value.

Table 82: Laboratory Summary Statistics by Parameter, Time Point, and Treatment Group – Serum Chemistry, Sodium

[Implementation Note: The number of decimals for the minimum and maximum will be the same as the original values, while the number of decimals for the mean, standard deviation, and median will add an extra decimal point to that of the original values.]

		Laboratory Value					Change from Baseline				
**Time Point**	**Treatment Group**	**n**	**Mean**	**Standard Deviation**	**Median**	**Min, Max**	**n**	**Mean**	**Standard Deviation**	**Median**	**Min, Max**
Baseline	Dalbavancin (N=X)	x	xx.x	xx.x	xx.x	xx.x, xx.x	NA	NA	NA	NA	NA
	Standard of Care (N=X)	x	xx.x	xx.x	xx.x	xx.x, xx.x	NA	NA	NA	NA	NA
Day 8	Dalbavancin (N=X)	x	xx.x	xx.x	xx.x	xx.x, xx.x	x	xx.x	xx.x	xx.x	xx.x, xx.x
	Standard of Care (N=X)	x	xx.x	xx.x	xx.x	xx.x, xx.x	x	xx.x	xx.x	xx.x	xx.x, xx.x
Day 22	Dalbavancin (N=X)	x	xx.x	xx.x	xx.x	xx.x, xx.x	x	xx.x	xx.x	xx.x	xx.x, xx.x
	Standard of Care (N=X)	x	xx.x	xx.x	xx.x	xx.x, xx.x	x	xx.x	xx.x	xx.x	xx.x, xx.x
Day 42	Dalbavancin (N=X)	x	xx.x	xx.x	xx.x	xx.x, xx.x	x	xx.x	xx.x	xx.x	xx.x, xx.x
	Standard of Care (N=X)	x	xx.x	xx.x	xx.x	xx.x, xx.x	x	xx.x	xx.x	xx.x	xx.x, xx.x

N = Number of subjects in the Safety Population.
n = Number of subjects in the Safety Population with non-missing laboratory values at the timepoint of interest. For the change from baseline, n represents the number of subjects in the Safety population with non-missing values at baseline and at the timepoint being assessed.

Tables with similar format to Table 82:

Table 83: Laboratory Summary Statistics by Parameter, Time Point, and Treatment Group – Serum Chemistry, Potassium

Table 84: Laboratory Summary Statistics by Parameter, Time Point, and Treatment Group – Serum Chemistry, Calcium

Table 85: Laboratory Summary Statistics by Parameter, Time Point, and Treatment Group – Serum Chemistry, Chloride

Table 86: Laboratory Summary Statistics by Parameter, Time Point, and Treatment Group – Serum Chemistry, Bicarbonate

Table 87: Laboratory Summary Statistics by Parameter, Time Point, and Treatment Group – Serum Chemistry, Glucose

Table 88: Laboratory Summary Statistics by Parameter, Time Point, and Treatment Group – Serum Chemistry, Blood Urea Nitrogen

Table 89: Laboratory Summary Statistics by Parameter, Time Point, and Treatment Group – Serum Chemistry, Creatinine

Tables with similar format to Table 82 *(continued)*

Table 90: Laboratory Summary Statistics by Parameter, Time Point, and Treatment Group – Serum Chemistry, Total Protein

Table 91: Laboratory Summary Statistics by Parameter, Time Point, and Treatment Group – Serum Chemistry, Alkaline Phosphatase

Table 92: Laboratory Summary Statistics by Parameter, Time Point, and Treatment Group – Serum Chemistry, Albumin

Table 93: Laboratory Summary Statistics by Parameter, Time Point, and Treatment Group – Serum Chemistry, Total Bilirubin

Table 94: Laboratory Summary Statistics by Parameter, Time Point, and Treatment Group – Serum Chemistry, AST

Table 95: Laboratory Summary Statistics by Parameter, Time Point, and Treatment Group – Serum Chemistry, ALT

RESTRICTED

14.3.5.2 Hematology Results

Table 96: Number and Percentage of Subjects with Potentially Clinically Significant Post-Baseline Laboratory Values by Parameter and Treatment Group – Hematology Parameters

	Dalbavancin			Standard of Care		
**Serum Chemistry Parameter**	**N**	**n**	**%**	**N**	**n**	**%**
Any Hematology Parameter	x	x	xx	x	x	xx
Absolute WBC	x	x	xx	x	x	xx
Hemoglobin	x	x	xx	x	x	xx
Platelet Count	x	x	xx	x	x	xx
Absolute Neutrophil Count	x	x	xx	x	x	xx
Absolute Lymphocyte Count	x	x	xx	x	x	xx

PCS = Potentially Clinically Significant. PCS Criteria is defined as follows for each parameter: Hemoglobin (<10 g/dL), Platelets (<75 /µL), White blood cell count (<3.0 x 10e9), Absolute neutrophil count (ANC) (<1500 x 10e9), Absolute lymphocyte count (ALC) (<800 x 10e9).
N = Number of subjects in the Safety Population with available non-PCS baseline values and at least 1 post-baseline assessment.
n = Number of subjects with at least one PCS post-baseline laboratory value.

Table 97: Laboratory Summary Statistics by Parameter, Time Point, and Treatment Group – Hematology, Absolute WBC

[Implementation Note: The number of decimals for the minimum and maximum will be the same as the original values, while the number of decimals for the mean, standard deviation, and median will add an extra decimal point to that of the original values.]

		Laboratory Value					Change from Baseline				
**Time Point**	**Treatment Group**	**n**	**Mean**	**Standard Deviation**	**Median**	**Min, Max**	**n**	**Mean**	**Standard Deviation**	**Median**	**Min, Max**
Baseline	Dalbavancin (N=X)	x	xx.x	xx.x	xx.x	xx.x, xx.x	NA	NA	NA	NA	NA
	Standard of Care (N=X)	x	xx.x	xx.x	xx.x	xx.x, xx.x	NA	NA	NA	NA	NA
Day 8	Dalbavancin (N=X)	x	xx.x	xx.x	xx.x	xx.x, xx.x	x	xx.x	xx.x	xx.x	xx.x, xx.x
	Standard of Care (N=X)	x	xx.x	xx.x	xx.x	xx.x, xx.x	x	xx.x	xx.x	xx.x	xx.x, xx.x
Day 22	Dalbavancin (N=X)	x	xx.x	xx.x	xx.x	xx.x, xx.x	x	xx.x	xx.x	xx.x	xx.x, xx.x
	Standard of Care (N=X)	x	xx.x	xx.x	xx.x	xx.x, xx.x	x	xx.x	xx.x	xx.x	xx.x, xx.x
Day 42	Dalbavancin (N=X)	x	xx.x	xx.x	xx.x	xx.x, xx.x	x	xx.x	xx.x	xx.x	xx.x, xx.x
	Standard of Care (N=X)	x	xx.x	xx.x	xx.x	xx.x, xx.x	x	xx.x	xx.x	xx.x	xx.x, xx.x

N = Number of subjects in the Safety Population.
n = Number of subjects in the Safety Population with non-missing laboratory values at the timepoint of interest. For the change from baseline, n represents the number of subjects in the Safety population with non-missing values at baseline and at the timepoint being assessed.

Tables with similar format to Table 97:

Table 98: Laboratory Summary Statistics by Parameter, Time Point, and Treatment Group – Hematology, Erythrocyte

Table 99: Laboratory Summary Statistics by Parameter, Time Point, and Treatment Group – Hematology, Hemoglobin

Table 100: Laboratory Summary Statistics by Parameter, Time Point, and Treatment Group – Hematology, Hematocrit

Table 101: Laboratory Summary Statistics by Parameter, Time Point, and Treatment Group – Hematology, Platelet Count

Table 102: Laboratory Summary Statistics by Parameter, Time Point, and Treatment Group – Hematology, Neutrophils

Table 103: Laboratory Summary Statistics by Parameter, Time Point, and Treatment Group – Hematology, Lymphocytes

Table 104: Laboratory Summary Statistics by Parameter, Time Point, and Treatment Group – Hematology, Monocytes

14.3.6 Displays of Vital Signs

Table 105: Summary of Vital Signs Values by Vital Sign Parameter, Visit, and Treatment Group

[Implementation Note: The number of decimals for the minimum and maximum will be the same as the original values, while the number of decimals for the mean, standard deviation, and median will add an extra decimal point to that of the original values.]

Vital Sign	Time Point	Dalbavancin (N=X)					Standard of Care (N=X)				
		n	Mean	Standard Deviation	Median	Min, Max	n	Mean	Standard Deviation	Median	Min, Max
Temperature (°F)	Visit 1 (Screening)	x	xx.xx	xx.xx	xx.xx	xx.x, xx.x	x	xx.xx	xx.xx	xx.xx	xx.x, xx.x
	Visit 2 (Baseline)										
	Visit 3 (Day 8)										
	Visit 4 (Day 22)										
	Visit 5 (Day 42)										
	Visit 6 (Day 70)										
	Visit 7 (Day 180)										
	Early Termination Visit										
Systolic Blood Pressure (mmHg)	Visit 1 (Screening)	x	xx.x	xx.x	xx.	xx, xx	x	xx.x	xx.x	xx.x	xx, xx
	Visit 2 (Baseline)										
	Visit 3 (Day 8)										
	Visit 4 (Day 22)										
	Visit 5 (Day 42)										
	Visit 6 (Day 70)										
	Visit 7 (Day 180)										
	Early Termination Visit										
Diastolic Blood Pressure (mmHg)	Visit 1 (Screening)	x	xx.x	xx.x	xx.x	xx, xx	x	xx.x	xx.x	xx.x	xx, xx
	Visit 2 (Baseline)										
	Visit 3 (Day 8)										
	Visit 4 (Day 22)										

Table 105: Summary of Vital Signs Values by Vital Sign Parameter, Visit, and Treatment Group (continued)

Vital Sign	Time Point	Dalbavancin (N=X)					Standard of Care (N=X)				
		n	Mean	Standard Deviation	Median	Min, Max	n	Mean	Standard Deviation	Median	Min, Max
	Visit 5 (Day 42)										
	Visit 6 (Day 70)										
	Visit 7 (Day 180)										
	Early Termination Visit										
Respiratory Rate (breaths/min)	Visit 1 (Screening)	x	xx.x	xx.x	xx.x	xx, xx	x	xx.x	xx.x	xx.x	xx, xx
	Visit 2 (Baseline)										
	Visit 3 (Day 8)										
	Visit 4 (Day 22)										
	Visit 5 (Day 42)										
	Visit 6 (Day 70)										
	Visit 7 (Day 180)										
	Early Termination Visit										
Pulse (beats/min)	Visit 1 (Screening)	x	xx.x	xx.x	xx.x	xx, xx	x	xx.x	xx.x	xx.x	xx, xx
	Visit 2 (Baseline)										
	Visit 3 (Day 8)										
	Visit 4 (Day 22)										
	Visit 5 (Day 42)										
	Visit 6 (Day 70)										
	Visit 7 (Day 180)										
	Early Termination Visit										

N = Number of subjects in the Safety Population.
n = Number of subjects in the Safety Population with non-missing values for the corresponding vital sign at the given timepoint.

Table 106: Summary of Change from Baseline Vital Signs Values by Vital Sign Parameter, Visit, and Treatment Group

[Implementation Note: The number of decimals for the minimum and maximum will be the same as the original values, while the number of decimals for the mean, standard deviation, and median will add an extra decimal point to that of the original values.]

		Dalbavancin (N=X)					Standard of Care (N=X)				
**Vital Sign**	**Time Point**	**n**	**Mean**	**Standard Deviation**	**Median**	**Min, Max**	**n**	**Mean**	**Standard Deviation**	**Median**	**Min, Max**
Temperature (°F)	Visit 3 (Day 8)	x	xx.xx	xx.xx	xx.xx	xx.x, xx.x	x	xx.xx	xx.xx	xx.xx	xx.x, xx.x
	Visit 4 (Day 22)										
	Visit 5 (Day 42)										
	Visit 6 (Day 70)										
	Visit 7 (Day 180)										
	Early Termination Visit										
Systolic Blood Pressure (mmHg)	Visit 3 (Day 8)	x	xx.x	xx.x	xx.	xx, xx	x	xx.x	xx.x	xx.x	xx, xx
	Visit 4 (Day 22)										
	Visit 5 (Day 42)										
	Visit 6 (Day 70)										
	Visit 7 (Day 180)										
	Early Termination Visit										
Diastolic Blood Pressure (mmHg)	Visit 3 (Day 8)	x	xx.x	xx.x	xx.x	xx, xx	x	xx.x	xx.x	xx.x	xx, xx
	Visit 4 (Day 22)										
	Visit 5 (Day 42)										
	Visit 6 (Day 70)										
	Visit 7 (Day 180)										
	Early Termination Visit										

Table 106: Summary of Change from Baseline Vital Signs Values by Vital Sign Parameter, Visit, and Treatment Group (continued)

		Dalbavancin (N=X)					Standard of Care (N=X)				
**Vital Sign**	**Time Point**	**n**	**Mean**	**Standard Deviation**	**Median**	**Min, Max**	**n**	**Mean**	**Standard Deviation**	**Median**	**Min, Max**
Respiratory Rate (breaths/min)	Visit 3 (Day 8)										
	Visit 4 (Day 22)										
	Visit 5 (Day 42)										
	Visit 6 (Day 70)										
	Visit 7 (Day 180)										
	Early Termination Visit										
Pulse (beats/min)	Visit 3 (Day 8)										
	Visit 4 (Day 22)										
	Visit 5 (Day 42)										
	Visit 6 (Day 70)										
	Visit 7 (Day 180)										
	Early Termination Visit										

N = Number of subjects in the Safety Population.
n = Number of subjects in the Safety Population with non-missing values for the corresponding vital sign at both baseline and given timepoint.

14.4 Summary of Concomitant Medications and Nondrug Interventions

Table 107: Number and Percentage of Subjects with Prior and Concurrent Medications by WHO Drug Classification and Treatment Group

WHO Drug Code Level 1, Anatomic Group	WHO Drug Code Level 2, Therapeutic Subgroup	Dalbavancin (N=X)		Standard of Care (N=X)		All Subjects (N=X)	
		n	%	n	%	n	%
Any Level 1 Codes	Any Level 2 Codes	x	xx	x	xx	x	xx
[ATC Level 1 - 1]	Any [ATC 1 – 1]						
	[ATC 2 - 1]						
	[ATC 2 - 2]						
	[ATC 2 - 3]						
[ATC Level 1 – 2]	[ATC 2 - 1]						
	[ATC 2 - 2]						
	[ATC 2 - 3]						

N = Number of subjects in the Safety Population.
n = Number of subjects reporting taking at least one medication in the specific WHO Drug Class.

Table 108: Number and Percentage of Subjects with Nondrug Interventions by MedDRA System Organ Class and Treatment Group – ITT Population

MedDRA System Organ Class	Dalbavancin (N=X)		Standard of Care (N=X)		All Subjects (N=X)	
	n	%	n	%	n	%
Any SOC	x	xx	x	xx	x	xx
[SOC 1]						
[SOC 2]						

N = Number of subjects in the ITT population.
n = Number of subjects reporting a nondrug intervention within the specified SOC. A subject is only counted once per SOC.

APPENDIX 2. FIGURE MOCK-UPS

LIST OF FIGURES

Figure 1: CONSORT Flow Diagram 137
Figure 2: Forest Plot of DOOR Probability, Dalbavancin Relative to Standard of Care, of Clinical DOOR Components at Day 70 – ITT Population....138
Figure 3: Forest Plot of DOOR Probability, Dalbavancin Relative to Standard of Care, of Clinical DOOR Components at Day 70 – mITT Population....138
Figure 4: Forest Plot of DOOR Probability, Dalbavancin Relative to Standard of Care, of Clinical DOOR Components at Day 70 – CE Population....138
Figure 5: Forest Plot of DOOR Probability, Dalbavancin Relative to Standard of Care, of Clinical DOOR Components at Day 42 – ITT Population....138
Figure 6: Forest Plot of DOOR Probability, Dalbavancin Relative to Standard of Care, of Clinical DOOR Components at Day 42 – mITT Population....138
Figure 7: Forest Plot of DOOR Probability, Dalbavancin Relative to Standard of Care, of Clinical DOOR Components at Day 42 – CE Population....138
Figure 8: Distribution of DOOR at Day 70 – ITT Population 139
Figure 9: Distribution of DOOR at Day 70 – mITT Population 139
Figure 10: Distribution of DOOR at Day 70 – CE Population 139
Figure 11: Distribution of DOOR at Day 42 – ITT Population 139
Figure 12: Distribution of DOOR at Day 42 – mITT Population 139
Figure 13: Distribution of DOOR at Day 42 – CE Population 139
Figure 14: Distribution of DOOR at Day 70 by Baseline Pathogen – ITT Population....139
Figure 15: Distribution of DOOR at Day 70 by PWID Status – ITT Population 140
Figure 16: Distribution of DOOR at Day 70 by Infectious Disease Consultation – ITT Population....140
Figure 17: Distribution of DOOR at Day 70 by Underlying Site of Infection – ITT Population....140
Figure 18: Distribution of DOOR at Day 70 by Immunosuppression – ITT Population....140
Figure 19: Distribution of DOOR at Day 70 by Duration of Initial Bacteremia – ITT Population....140
Figure 20: Forest Plot of DOOR Probabilities at Day 70 – ITT Population....141
Figure 21: Forest Plot of DOOR Probabilities at Day 42 – ITT Population....141
Figure 22: Difference in Cumulative Proportions of DOOR Along with 95% CI....142

Figure 23: Difference in Means of Partial Credit Score by Timepoint and Analysis Population 143
Figure 24: Bivariate Analysis of DOOR Probability vs Difference in Mean of Change in QoL Score from Baseline at Day 70 – ITT Analysis Population 144
Figure 25: Bivariate Analysis of DOOR Probability vs Difference in Mean of Change in QoL Score from Baseline at Day 42 – ITT Analysis Population 144
Figure 26: Predictive Interval Plots for the Probability of Higher DOOR in the Dalbavancin Group at Day 70 – ITT Analysis Population....145
Figure 27: Predictive Interval Plots Statistics for the Rates of Clinical Efficacy at Day 70 – ITT Analysis Population* 145
Figure 28: Frequency of Related Adverse Events by MedDRA System Organ Class, Severity, and Treatment Group....146
Figure 29: Frequency of Related Adverse Events by MedDRA High Level Group Term, Severity, and Treatment Group....146
Figure 30: Forest Plot of Risk Differences of All Adverse Events by MedDRA System Organ Class....147
Figure 31: Forest Plot of Risk Differences of All Adverse Events by MedDRA High Level Group Term 147
Figure 32: Forest Plot of Risk Differences of Experiencing a Clinical Laboratory Abnormality by Laboratory Parameter – Hematology Parameters 148
Figure 33: Forest Plot of Risk Differences of Experiencing a Clinical Laboratory Abnormality by Laboratory Parameter – Serum Chemistry Parameters 149

10.1 Disposition of Subjects

Figure 1: CONSORT Flow Diagram

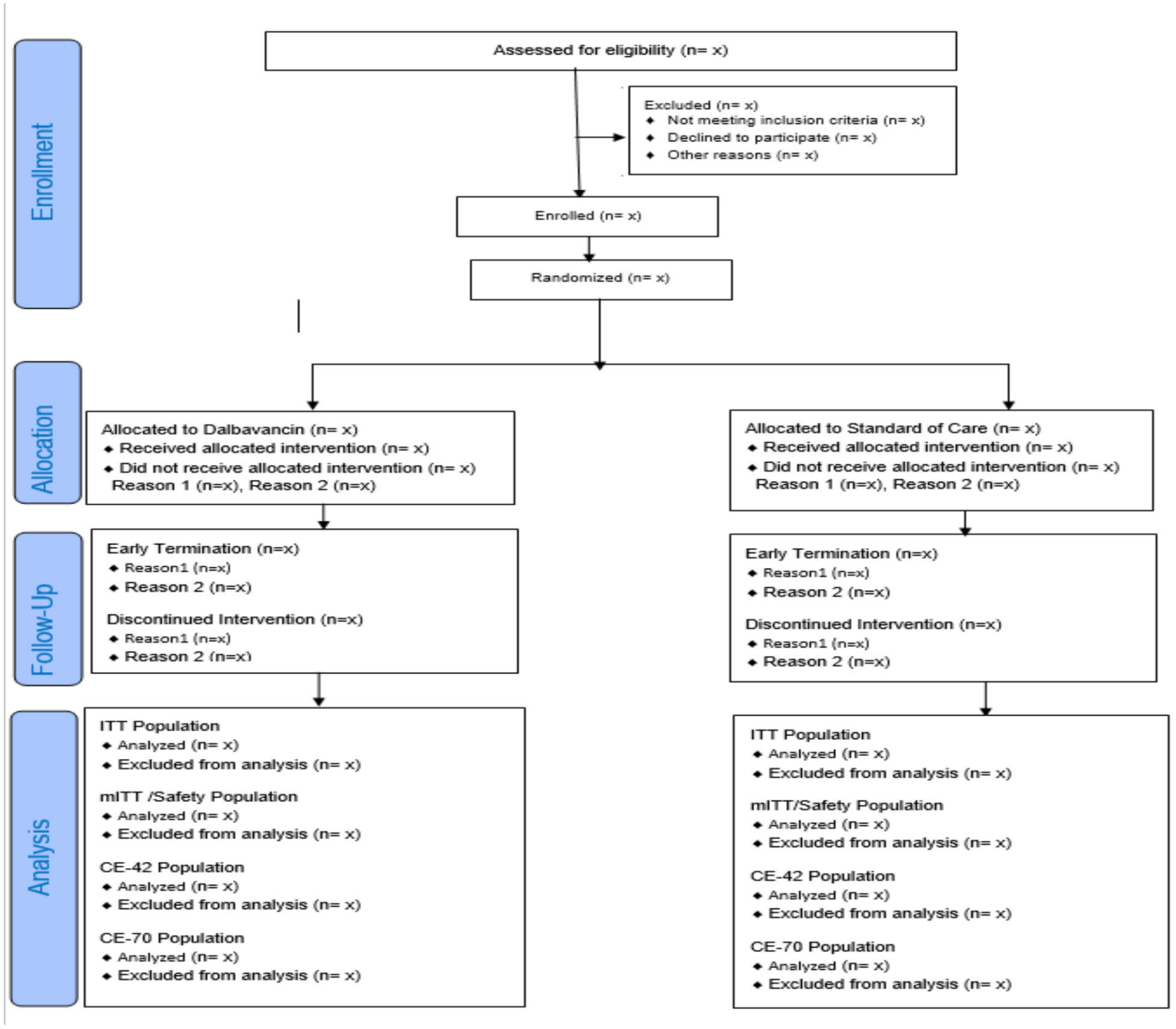

14.2.2 Efficacy Response Figures by Measure, Treatment, and Time Point

Figure 2: Forest Plot of DOOR Probability, Dalbavancin Relative to Standard of Care, of Clinical DOOR Components at Day 70 – ITT Population

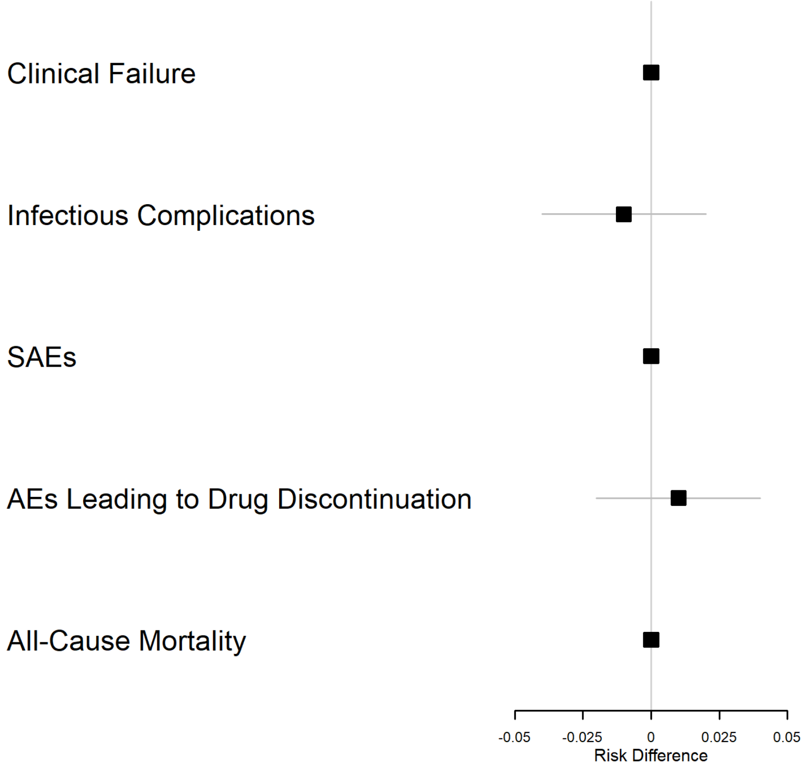

Programming Note: Include n (%) of subjects by arm as additional columns – next to description of each DOOR component. Additionally, change "Risk Difference" to DOOR Probability.

Figures similar to Figure 2:

Figure 3: Forest Plot of DOOR Probability, Dalbavancin Relative to Standard of Care, of Clinical DOOR Components at Day 70 – mITT Population

Figure 4: Forest Plot of DOOR Probability, Dalbavancin Relative to Standard of Care, of Clinical DOOR Components at Day 70 – CE Population

Figure 5: Forest Plot of DOOR Probability, Dalbavancin Relative to Standard of Care, of Clinical DOOR Components at Day 42 – ITT Population

Figure 6: Forest Plot of DOOR Probability, Dalbavancin Relative to Standard of Care, of Clinical DOOR Components at Day 42 – mITT Population

Figure 7: Forest Plot of DOOR Probability, Dalbavancin Relative to Standard of Care, of Clinical DOOR Components at Day 42 – CE Population

Figure 8: Distribution of DOOR at Day 70 – ITT Population

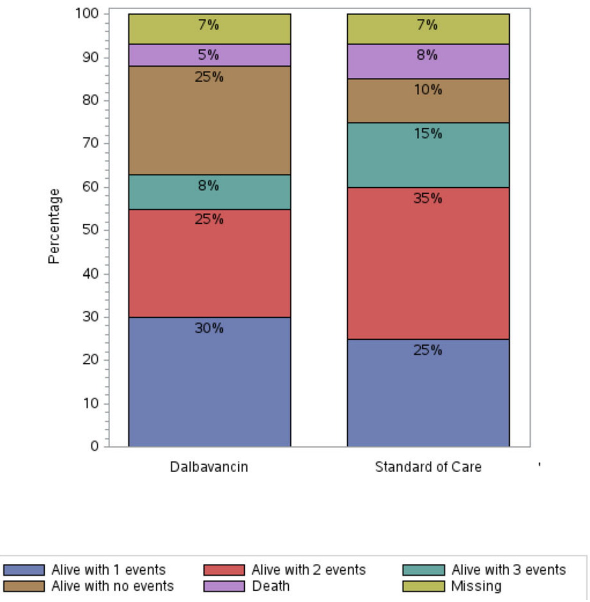

Figure 9: Distribution of DOOR at Day 70 – mITT Population

Figure will be similar to Figure 8.

Figure 10: Distribution of DOOR at Day 70 – CE Population

Figure will be similar to Figure 8.

[Implementation note: The missing category is removed from this analysis since the CE population has complete data.]

Figure 11: Distribution of DOOR at Day 42 – ITT Population

Figure will be similar to Figure 8.

Figure 12: Distribution of DOOR at Day 42 – mITT Population

Figure will be similar to Figure 8.

Figure 13: Distribution of DOOR at Day 42 – CE Population

Figure will be similar to Figure 8.

[Implementation note: The missing category is removed from this analysis since the CE population has complete data.]

Figure 14: Distribution of DOOR at Day 70 by Baseline Pathogen – ITT Population

Figure similar to Figure 8.

[Implementation note: Create two separate panels for MRSA and MSSA]

Figure 15: Distribution of DOOR at Day 70 by PWID Status – ITT Population

Figure similar to Figure 8.

[Implementation note: Create two separate panels for PWID and non-PWID]

Figure 16: Distribution of DOOR at Day 70 by Infectious Disease Consultation – ITT Population

Figure similar to Figure 8.

[Implementation note: Create two separate panels for Yes and No]

Figure 17: Distribution of DOOR at Day 70 by Underlying Site of Infection – ITT Population

Figure similar to Figure 8.

[Implementation note: Create four separate panels for the 4 different sites.]

Figure 18: Distribution of DOOR at Day 70 by Immunosuppression – ITT Population

[Implementation note: Create two separate panels for Yes and No]

Figure 19: Distribution of DOOR at Day 70 by Duration of Initial Bacteremia – ITT Population

Figure similar to Figure 8.

[Implementation note: Create three separate panels for <2, 2-4, >4 days]

Figure 20: Forest Plot of DOOR Probabilities at Day 70 – ITT Population

[Implementation note: This figure will be updated using SAS to add the rest of the subgroups and reformat the Y axis values as: Baseline Pathogen as title with indented categories MRSA, MSSA. Similar format update will be done for the rest of the subgroup categories. Caps will also be added to the error bars.]

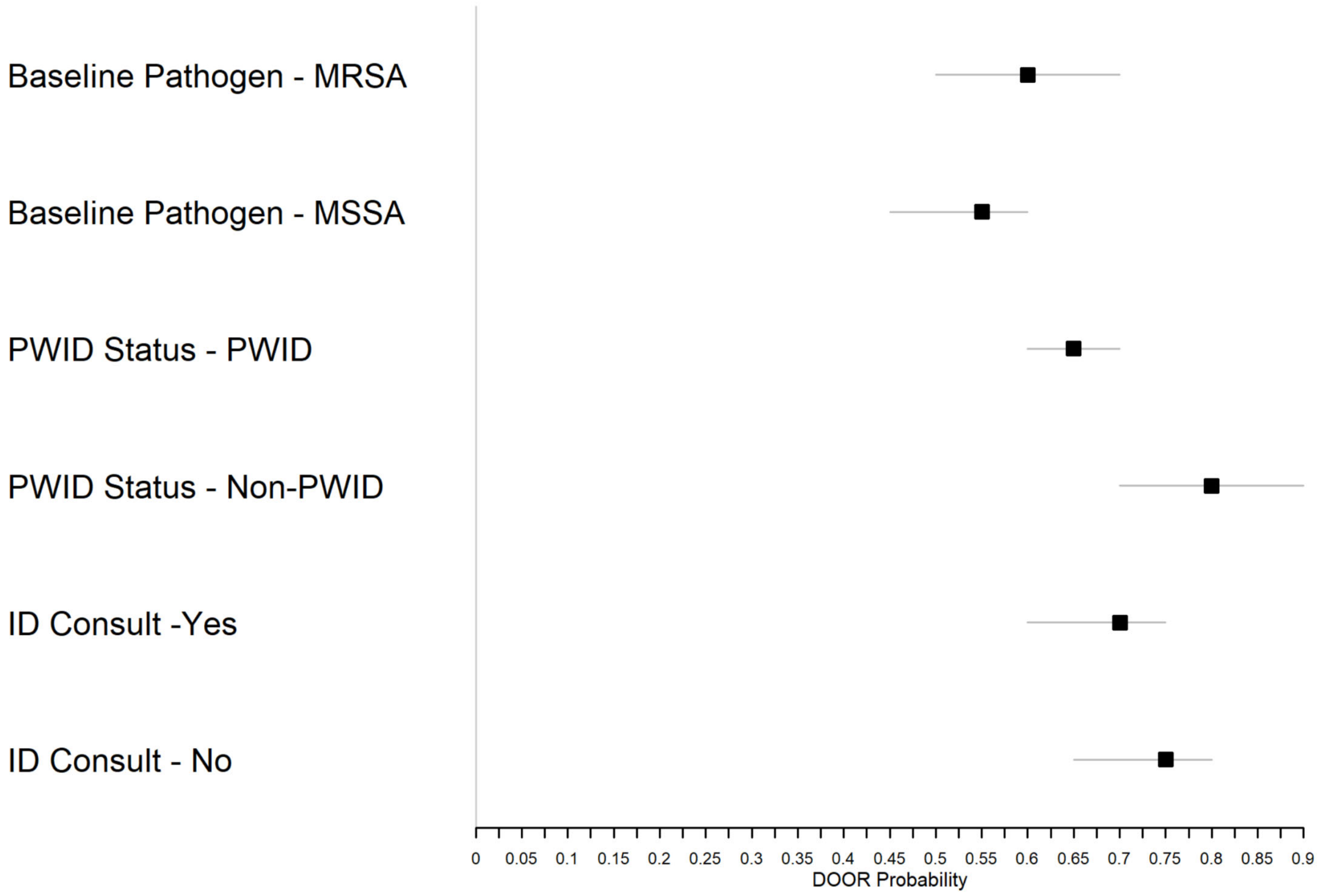

Similar to Figure 2.

Programming note: Include n (%) as columns.

Figure 21: Forest Plot of DOOR Probabilities at Day 42 – ITT Population

Figure similar to Figure 20.

Figure 22: Difference in Cumulative Proportions of DOOR Along with 95% CI

[Implementation note: Make a figure with 6 panels (2 rows representing Day 42 and Day 70; 3 columns representing the 3 analysis populations.]

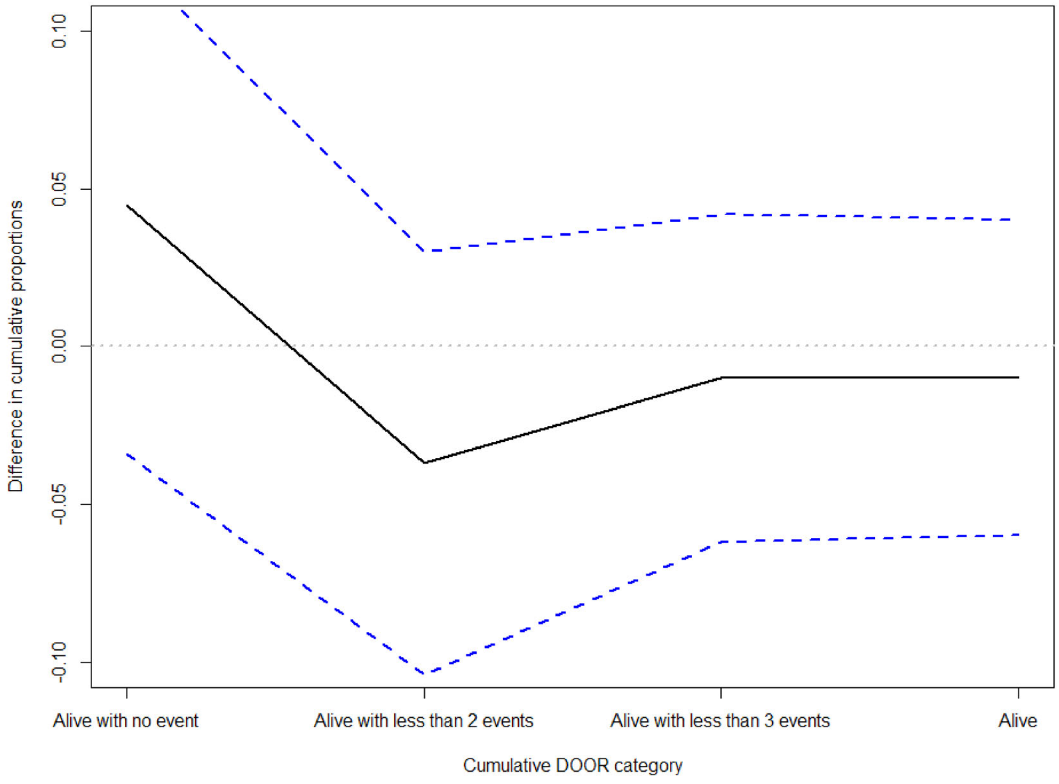

Figure 23: Difference in Means of Partial Credit Score by Timepoint and Analysis Population

[Implementation note: The figure will have 6 panels with two rows for Day 42 and Day 70 and two columns representing ITT, mITT, and CE analysis populations.]

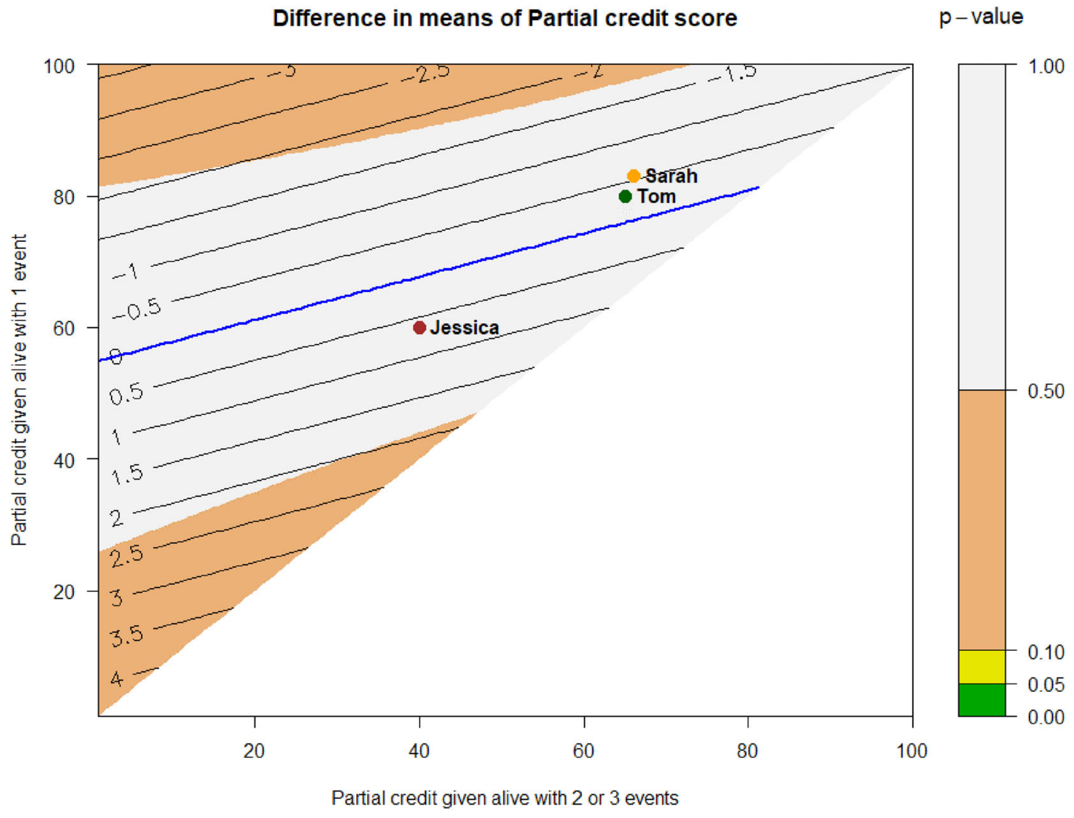

Figure 24: Bivariate Analysis of DOOR Probability vs Difference in Mean of Change in QoL Score from Baseline at Day 70 – ITT Analysis Population

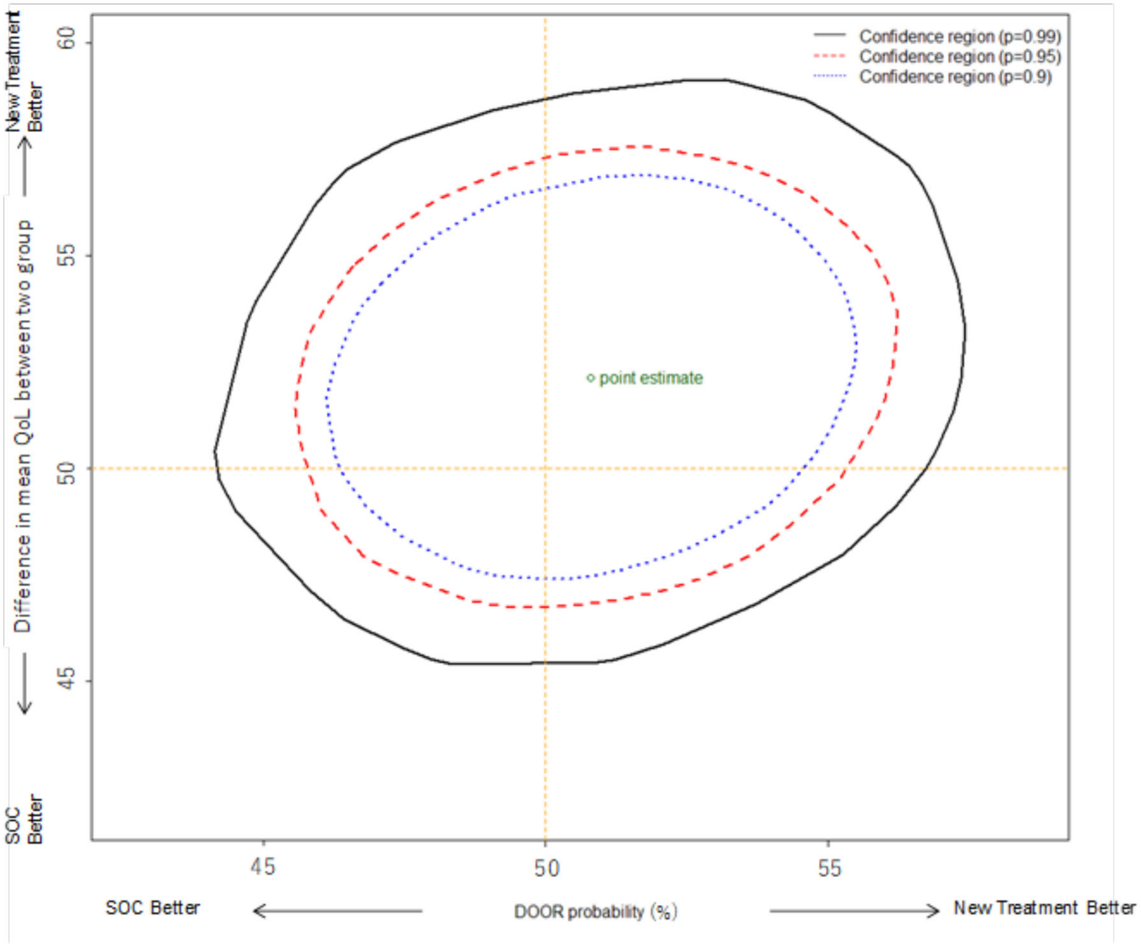

Figure 25: Bivariate Analysis of DOOR Probability vs Difference in Mean of Change in QoL Score from Baseline at Day 42 – ITT Analysis Population

Figure will be similar to Figure 24.

Figure 26: Predictive Interval Plots for the Probability of Higher DOOR in the Dalbavancin Group at Day 70 – ITT Analysis Population

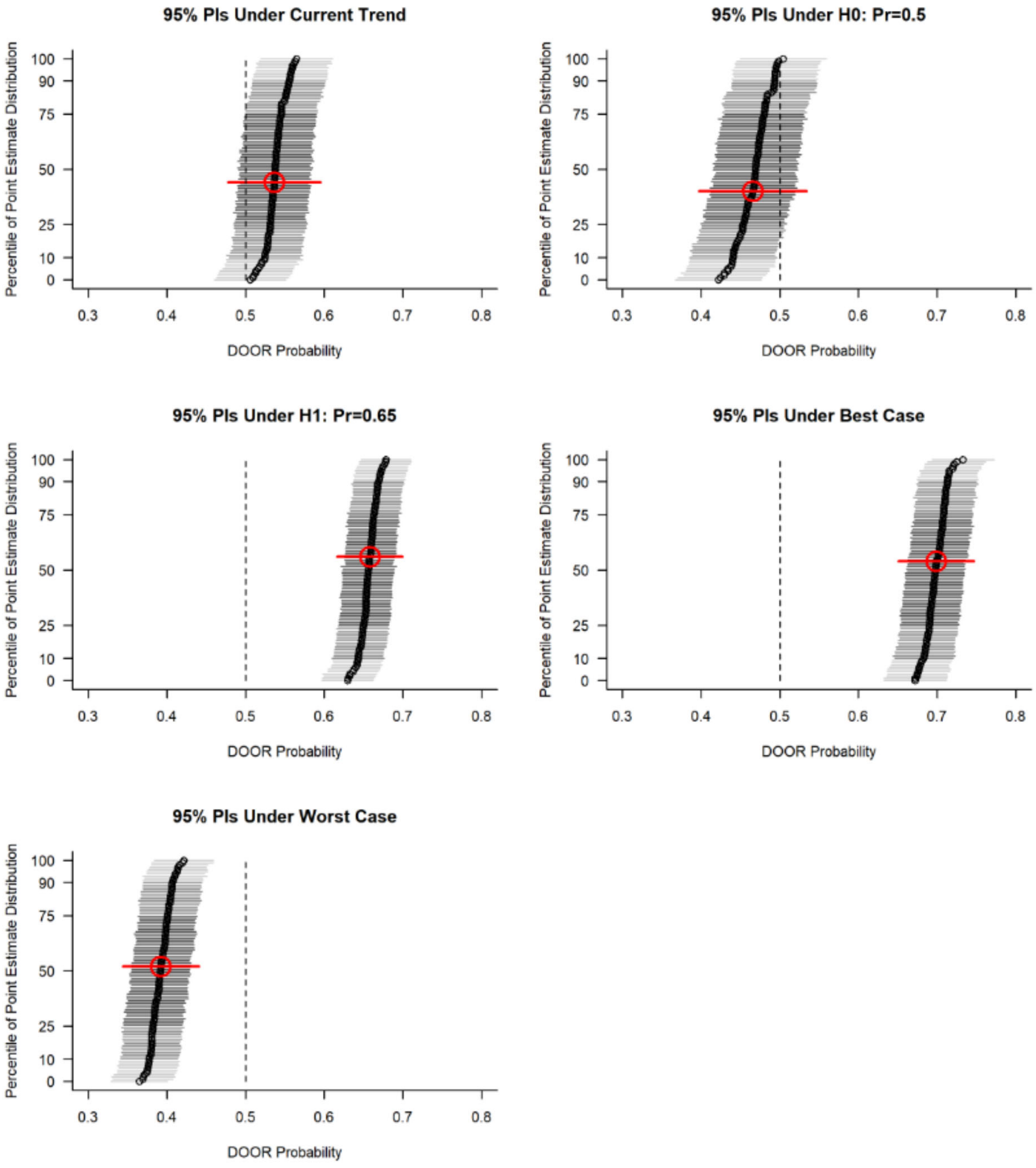

Figure with similar format:

Figure 27: Predictive Interval Plots Statistics for the Rates of Clinical Efficacy at Day 70 – ITT Analysis Population*

[Implementation note: Regenerate the figure for rates of clinical efficacy. Provide results for current trend, under null H0 with rate =0.5, under alternative H0 with rate=0.4, under best case and under worst case scenario.]

14.3.1.2 Unsolicited Adverse Events

Figure 28: Frequency of Related Adverse Events by MedDRA System Organ Class, Severity, and Treatment Group

[Implementation note: Panels for Dalbavancin and Standard of Care subjects will be presented. Grade 3 or higher will be reported for all AEs except AESIs which will include lower severities 1 and Grade 2.]

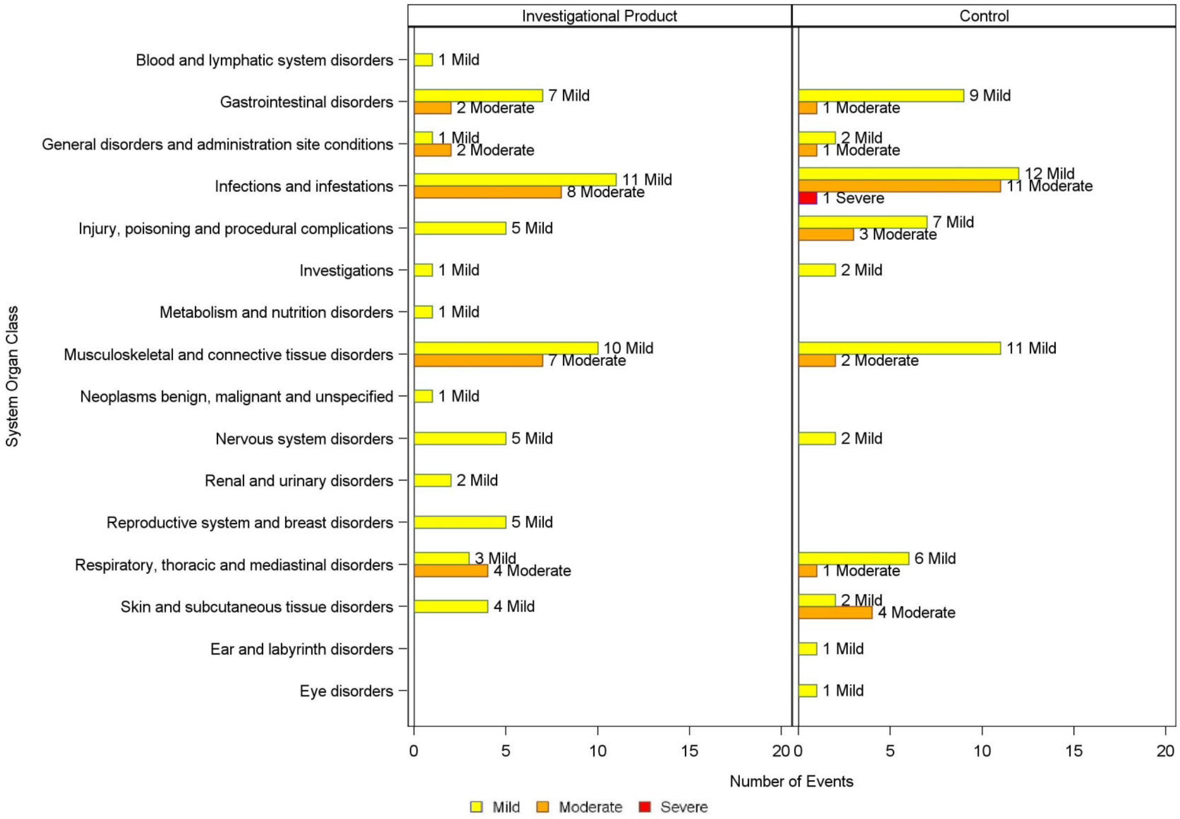

Figure 29: Frequency of Related Adverse Events by MedDRA High Level Group Term, Severity, and Treatment Group

Figure will be similar to Figure 28.

Figure 30: Forest Plot of Risk Differences of All Adverse Events by MedDRA System Organ Class

[Implementation note: The 95% CI for the risk difference will be computed using the Miettinen-Nurminen method.]

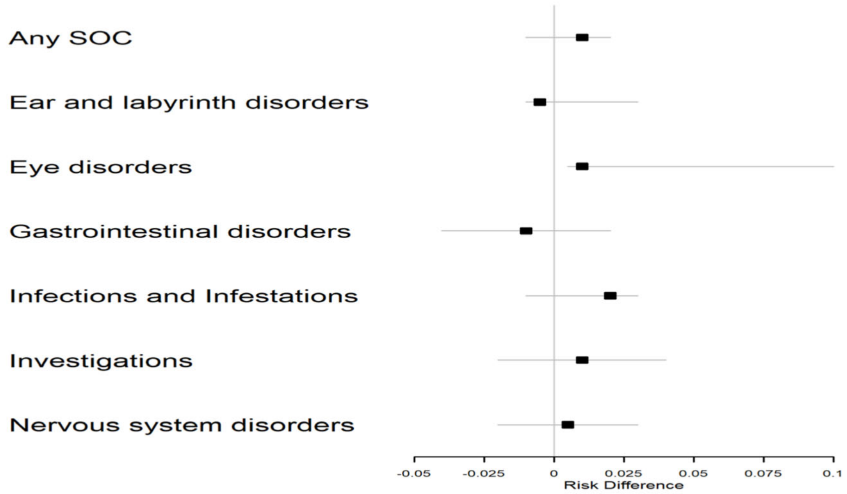

Implementation Note: Include n (%) as columns. See Figure 2 for example.

Figure 31: Forest Plot of Risk Differences of All Adverse Events by MedDRA High Level Group Term

Figure will be similar to Figure 30.

Implementation Note: Include n (%) as columns. See Figure 2 for example.

14.3.5 Displays of Laboratory Results

Figure 32: Forest Plot of Risk Differences of Experiencing a Clinical Laboratory Abnormality by Laboratory Parameter – Hematology Parameters

[Implementation note: Create separate panels for each visit. 95% confidence intervals for the risk differences will be computed using the Miettinen-Nurminen method.]

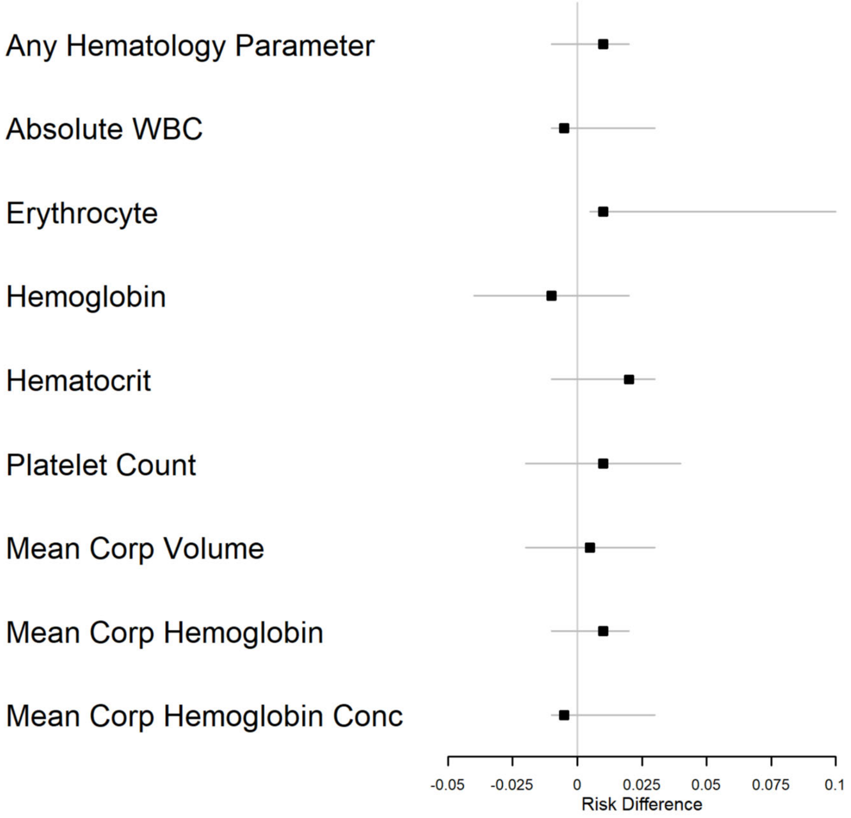

Figure 33: Forest Plot of Risk Differences of Experiencing a Clinical Laboratory Abnormality by Laboratory Parameter – Serum Chemistry Parameters

[Implementation note: Create separate panels for each visit. 95% confidence intervals for the risk differences will be computed using the Miettinen-Nurminen method.]

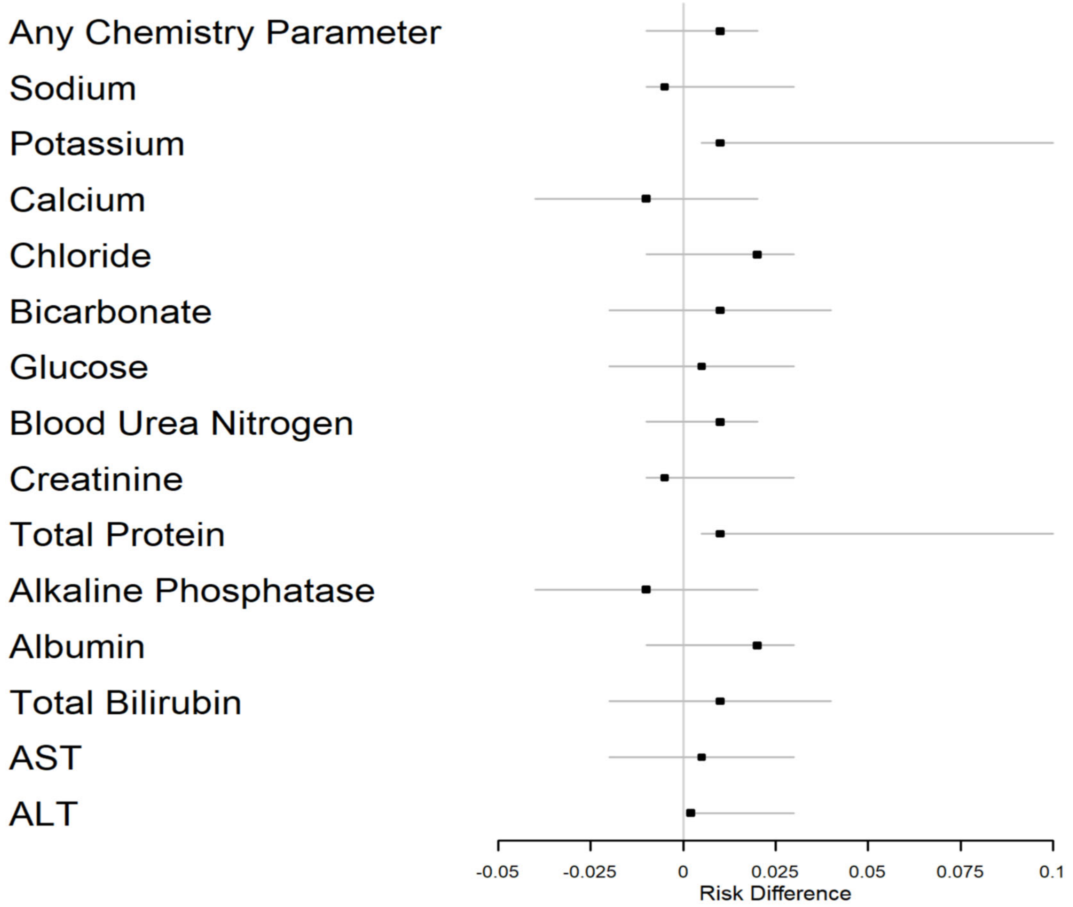

APPENDIX 3. LISTINGS MOCK-UPS

LISTINGS

Listing 1: 16.1.6: Listing of Subjects Receiving Investigational Product....152
Listing 2: 16.2.1: Early Terminations or Discontinued Subjects....153
Listing 3: 16.2.2.1: Subject-Specific Protocol Deviations....154
Listing 4: 16.2.2.2: Non-Subject-Specific Protocol Deviations....155
Listing 5: 16.2.3: Subjects Excluded from Analysis Populations....156
Listing 6: 16.2.4.1: Demographic Data....157
Listing 7: 16.2.4.2: Pre-Existing and Concurrent Medical Conditions....158
Listing 8: 16.2.4.3: Investigator Assessment of Baseline *S. aureus* Bacteremia Diagnoses....159
Listing 9: 16.2.5: Compliance and/or Drug Concentration Data....160
Listing 10: 16.2.6.1: Individual DOOR Response Data....161
Listing 11: 16.2.6.1: Investigator Assessment of Efficacy – Clinical Success....162
Listing 12: 16.2.6.1: Investigator Assessment of Efficacy – Infectious Complications....163
Listing 13: 16.2.6.2: Listing of Culture Results....164
Listing 14: 16.2.6.2: Individual QoL Data....165
Listing 15: 16.2.7.3: Listing of Treatment-Emergent Adverse Events....166
Listing 16: 16.2.7.3: Listing of All Adverse Events for Subjects with Potentially Clinically Significant Post-Baseline Clinical Laboratory or Vital Sign Values....167
Listing 17: 16.2.8.1: Clinical Laboratory Results – Chemistry....168
Listing 18: 16.2.8.2: Clinical Laboratory Results – Hematology....169
Listing 19: 16.2.9.1: Vital Signs....170
Listing 20: 16.2.9.2: Physical Exam Findings....171
Listing 21: 16.2.10.1: Concomitant Medications....172
Listing 22: 16.2.10.1: Nondrug Interventions....172
Listing 23: 16.2.10.1: Echocardiogram Results....173
Listing 24: 16.2.10.2: Hospitalization Events....174
Listing 25: 16.2.11.1: Pregnancy Reports – Maternal Information....175
Listing 26: 16.2.11.2: Pregnancy Reports – Gravida and Para....175
Listing 27: 16.2.11.3: Pregnancy Reports – Live Birth Outcomes....176

Listing 28: 16.2.11.4: Pregnancy Reports – Still Birth Outcomes 176
Listing 29: 16.2.11.5: Pregnancy Reports – Spontaneous, Elective, or Therapeutic Abortion Outcomes 176

Listing 1: **16.1.6: Listing of Subjects Receiving Investigational Product**

(Not included in SAP, but this is a placeholder for the CSR)

16.2 Database Listings by Subject

16.2.1 Discontinued Subjects

Listing 2: 16.2.1: Early Terminations or Discontinued Subjects

Treatment Group	Subject ID	Category	Reason for Early Termination or Treatment Discontinuation	Study Day

16.2.2 Protocol Deviations

Listing 3: 16.2.2.1: Subject-Specific Protocol Deviations

Treatment Group	Subject ID	DV Number	Deviation	Deviation Category	Study Day	Reason for Deviation	Deviation Resulted in AE?	Deviation Resulted in Subject Termination?	Deviation Affected Product Stability?	Deviation Resolution	Comments

Listing 4: 16.2.2.2: Non-Subject-Specific Protocol Deviations

Site	Start Date	Deviation	End Date	Reason for Deviation	Deviation Resulted in Subject Termination?	Deviation Affected Product Stability?	Deviation Category	Deviation Resolution	Comments

16.2.3 Subjects Excluded from the Efficacy Analysis

Listing 5: 16.2.3: Subjects Excluded from Analysis Populations

Treatment Group	Subject ID	Analyses in which Subject is Included	Analyses from which Subject is Excluded	Reason Subject Excluded
Dalbavancin	SST.XXXX	[e.g., Safety, ITT, mITT]	CE42	
Dalbavancin	SST.XXXX		CE70	

16.2.4 Demographic Data

Listing 6: 16.2.4.1: Demographic Data

Treatment Group	Subject ID	Sex	Age (years)	Ethnicity	Race	Height (cm)	Weight (kg)	BMI (kg/m^2)	Baseline Pathogen	Baseline QoL Score

Listing 7: 16.2.4.2: Pre-Existing and Concurrent Medical Conditions

Treatment Group	Subject ID	MH Number	Medical History Term	Condition Start Day	Condition End Day	MedDRA System Organ Class	MedDRA Preferred Term	MedDRA High Level Group Term

Listing 8: 16.2.4.3: Investigator Assessment of Baseline *S. aureus* Bacteremia Diagnoses

Treatment Group	Subject ID	*S. aureus* Infectious Complication	Evidence of Infectious Complication?
Dalbavancin	SST.XXXX	Acute bacterial skin and skin structure infection (ABSSSI)	Yes/No
Dalbavancin	SST.XXXX	Other (Abcess)	Yes

RESTRICTED

16.2.5 Compliance and/or Drug Concentration Data (if available)

Listing 9: 16.2.5: Compliance and/or Drug Concentration Data

[Implementation note: Sort by Treatment group and subject ID.]

Treatment Group	Subject ID	Dose Amount	Treatment Duration[a]	Creatinine Clearance[b]

[a] Treatment duration is defined as the number of doses for dalbavancin while it is defined as the duration between start and end dates for standard of care.
[b] Only included for subjects treated with Dalbavancin.

16.2.6 Individual Efficacy Response Data

Listing 10: 16.2.6.1: Individual DOOR Response Data

[Implementation note: Sort by treatment group, subject ID, and Planned Time Point.]

Treatment Group	Subject ID	Planned Time Point	Clinical Failure	Infectious Complications	SAEs	AEs Leading to Study Drug Discontinuation	All-cause Mortality	DOOR Category	Change in QoL from Baseline	DOOR	Clinical Efficacy	Microbiological Success
Dalbavancin	SST.XXXX	Day 42	Yes	No	No	No	No	2	40	120	No	Yes
Dalbavancin	SST.XXXX	Day 70	No	No	No	No	No	1	60	20	Yes	Yes

Listing 11: 16.2.6.1: Investigator Assessment of Efficacy – Clinical Success

[Implementation note: Sort by treatment group, subject ID, and Planned Time Point.]

Treatment Group	Subject ID	Planned Time Point	Has the Subject Died?	Have additional antibiotics not specified by the protocol been required for the treatment of *S. aureus* bacteremia?	How many antibiotics have been used?	Is it anticipated that additional antibiotic therapy beyond that specified by the protocol will be required for the treatment of *S. aureus* bacteremia?
Dalbavancin	SST.XXXX	Day 42	Yes	No	N/A	No
Dalbavancin	SST.XXXX	Day 70	No	Yes	3	N/A

Listing 12: 16.2.6.1: Investigator Assessment of Efficacy – Infectious Complications

[Implementation note: Sort by treatment group, subject ID, and Planned Time Point.]

Treatment Group	Subject ID	Planned Time Point	Is the subject experiencing clinical failure?	Has the subject had endocarditis since randomization/ last assessment?	Has the subject had new evidence of metastatic foci of infection since randomization/ last assessment?	Has the subject relapsed since randomization/ last assessment?	Has the subject been re-hospitalized since randomization/last assessment for subsequent care of indication under study?	Has the subject had an additional unplanned source control procedure since randomization/last assessment?	Has the subject had a change in antibiotic therapy due to inadequate clinical response?	Has the subject achieved clinical success?
Dalbavancin	SST.XXXX	Day 42	Yes	No	No	No	No	No	Yes	No
Dalbavancin	SST.XXXX	Day 70	No	No	No	No	No	No	No	Yes

Listing 13: 16.2.6.2: Listing of Culture Results

Treatment Group	Subject ID	Culture Site	Collection Date	Actual Study Day	Collection Time	Culture Result	Pathogen Detected
Dalbavancin	SST.XXXX	Blood or Urine or Sputum, …	XXMMMYYYY		HH:MM	Positive or Negative	

Listing 14: 16.2.6.2: Individual QoL Data

Treatment Group	Subject ID	Planned Time Point	QoL Score Using ARLG Bloodstream Infection[a]	QoL Score Using PROMIS Global Health Short Form	QoL Score Using EQ-5D-5L instrument
Dalbavancin	SST.XXXX	Baseline			
Dalbavancin	SST.XXXX	Day 42			
Dalbavancin	SST.XXXX	Day 70			

[a] Standardized score is obtained from the items selected from the PROMIS physical function item bank (PROMIS Item Bank v2.0, short form 6b) item bank on the ARLG Bloodstream Infection QoL

16.2.7 Adverse Events

Listing 15: 16.2.7.3: Listing of Treatment-Emergent Adverse Events

Adverse Event	Study Day of AE Onset	Duration (Days)	Severity	Relationship to Study Treatment	If Not Related, Alternative Etiology	Action Taken with Study Treatment	Subject Discontinued Due to AE	Outcome	MedDRA System Organ Class	MedDRA Preferred Term	MedDRA High Level Group Term
**Treatment Group: , Subject ID: , AE Number:**											
Comments:											
**Treatment Group: , Subject ID: , AE Number:**											
Comments:											

Listing 16: 16.2.7.3: Listing of All Adverse Events for Subjects with Potentially Clinically Significant Post-Baseline Clinical Laboratory or Vital Sign Values

Adverse Event	Study Day of AE Onset	Duration (Days)	Severity	Relationship to Study Treatment	If Not Related, Alternative Etiology	Action Taken with Study Treatment	Subject Discontinued Due to AE	Outcome	MedDRA System Organ Class	MedDRA Preferred Term	MedDRA High Level Group Term
**Treatment Group: , Subject ID: , AE Number:**											
Comments:											
**Treatment Group: , Subject ID: , AE Number:**											
Comments:											

16.2.8 Individual Laboratory Measurements

Listing 17: 16.2.8.1: Clinical Laboratory Results – Chemistry

Treatment Group	Subject ID	Planned Time Point	Actual Study Day	Sex	Age (years)	Laboratory Parameter (Units)	Result (Severity Grade)	Reference Range Low	Reference Range High

Listing 18: 16.2.8.2: Clinical Laboratory Results – Hematology

Treatment Group	Subject ID	Planned Time Point	Actual Study Day	Sex	Age (years)	Laboratory Parameter (Units)	Result (Severity Grade)	Reference Range Low	Reference Range High

16.2.9 Vital Signs and Physical Exam Findings

Listing 19: 16.2.9.1: Vital Signs

Treatment Group	Subject ID	Planned Time Point	Actual Study Day	Temperature (°F)	Systolic Blood Pressure (mmHg)	Diastolic Blood Pressure (mmHg)	Pulse (beats/min)	Respiratory Rate (breaths/min)

Listing 20: 16.2.9.2: Physical Exam Findings

Treatment Group	Subject ID	Planned Time Point	Actual Study Day	Body System	Abnormal Finding	Reported as an AE? (AE Description; Number)

16.2.10 Concomitant Medications

Listing 21: 16.2.10.1: Concomitant Medications

Treatment Group	Subject ID	CM Number	Medication	Medication Start Day	Medication End Day	Indication	Taken for an AE? (AE Description; Number)	Taken for a condition on Medical History? (MH Description; Number)	ATC Level 1 (ATC Level 2)

Listing 22: 16.2.10.1: Nondrug Interventions

Treatment Group	Subject ID	ND Number	Surgery/Procedure	Date of Procedure	Indication

RESTRICTED

Listing 23: 16.2.10.1: Echocardiogram Results

Treatment Group	Subject ID	Date of Procedure	Procedure Type	Result
			Transthoracic	Normal
			Transesophageal	Abnormal, not clinically significant
			Transthoracic	Abnormal, with evidence of left-sided endocarditis
			Transthoracic	Abnormal, with other clinically significant findings: specify

Listing 24: 16.2.10.2: Hospitalization Events

Treatment Group	Subject ID	Date of Admission	Reason for Admission	Date of Discharge	Discharge Status	Discharge Diagnosis

16.2.11 Pregnancy Reports

Listing 25: 16.2.11.1: Pregnancy Reports – Maternal Information

Treatment Group	Subject ID	Pregnancy Number	Study Day Corresponding to Estimated Date of Conception	Source of Maternal Information	Pregnancy Status	Mother's Pre-Pregnancy BMI	Mother's Weight Gain During Pregnancy	Tobacco, Alcohol, or Drug Use During Pregnancy?	Medications During Pregnancy?	Maternal Complications During Pregnancy?	Maternal Complications During Labor, Delivery, or Post-Partum?
Note: Maternal Complications are included in the Adverse Event listing. Medications taken during pregnancy are included in the Concomitant Medications Listing.											

Listing 26: 16.2.11.2: Pregnancy Reports – Gravida and Para

			Live Births												
Subject ID	Pregnancy Number	Gravida	Extremely PB[a]	Very Early PB[a]	Early PB[a]	Late PB[a]	Early TB[b]	Full TB[b]	Late TB[b]	Post TB[b]	Still Births	Spontaneous Abortion/ Miscarriage	Elective Abortions	Therapeutic Abortions	Major Congenital Anomaly with Previous Pregnancy?
Note: Gravida includes the current pregnancy, para events do not.															

[a] Preterm Birth
[b] Term Birth

Listing 27: 16.2.11.3: Pregnancy Reports – Live Birth Outcomes

Subject ID	Pregnancy Number	Fetus Number	Pregnancy Outcome (for this Fetus)	Fetal Distress During Labor and Delivery?	Delivery Method	Gestational Age at Live Birth	Size for Gestational Age	Apgar Score, 1 minute	Apgar Score, 5 minutes	Cord pH	Congenital Anomalies?	Illnesses/ Hospitalizations within 1 Month of Birth?

Note: Congenital Anomalies are included in the Adverse Event listing.

Listing 28: 16.2.11.4: Pregnancy Reports – Still Birth Outcomes

Subject ID	Date of Initial Report	Fetus Number	Pregnancy Outcome (for this Fetus)	Fetal Distress During Labor and Delivery?	Delivery Method	Gestational Age at Still Birth	Size for Gestational Age	Cord pH	Congenital Anomalies?	Autopsy Performed?	If Autopsy, Etiology for Still Birth Identified?

Listing 29: 16.2.11.5: Pregnancy Reports – Spontaneous, Elective, or Therapeutic Abortion Outcomes

Subject ID	Date of Initial Report	Fetus Number	Pregnancy Outcome (for this Fetus)	Gestational Age at Termination	Abnormality in Product of Conception?	Reason for Therapeutic Abortion

DMID 20-0002 SAP Version 1.0, 03OCT2023
Updated in
DMID 20-0002 SAP Version 2.0, 03JAN2024

#	Section # and Name	Description of Change	Rationale for Change
1	Various	Updated all uses of "participant" to "subject"	Consistency with protocol and throughout SAP
2	3.1.2, Secondary	Updated language for secondary objective measure #3: To compare each individual component of DOOR outcome by treatment arm, in the ITT population.	Consistency with language in protocol
3	3.2.3, Exploratory	Updated language for exploratory endpoint #2: DOOR endpoint by treatment group at Day 42 in the ITT, mITT, and CE populations.	Consistency with language in protocol
4	8.1.1, Analysis of DOOR at Day 70 Using ITT Analysis Population	Modified text: … where $DOOR_D$ and $DOOR_C$ are the QoL-Adjusted DOOR for dalbavancin and control, or standard of care, groups, respectively, and $\Pr[DOOR_D > DOOR_C]$ is the probability of a DOOR from dalbavancin being more desirable than a DOOR from standard care and $\Pr[DOOR_D = DOOR_C]$ is the probability of two DOOR being the same.	Clarification
5	8.1.1.2, ITT Analysis of DOOR using Multiple Imputation	Modified text: For each of the 20 complete multiple imputation datasets, a DOOR probability, estimated by the Wilcoxon Mann-Whitney U Statistic corrected for ties, will be computed using randomization to dalbavancin versus randomization to standard of care therapy to define the binary grouping and DOOR at Day 70 as the outcome.	Clarification
6	8.1.1.2, ITT Analysis of DOOR using Multiple Imputation<br>Appendix C, Listing 11, Listing 12	Added listings of investigator assessments of clinical success and infectious complications.	New listings added
7	Section 8.3.3.2, Distribution of DOOR by Treatment Group Using ITT and mITT Analysis Populations<br>Appendix A, Table 55	Removed Wilcoxon Rank-Sum test analysis	Removed Wilcoxon Rank-Sum test as this analysis will not aid in interpretation of overall study results, which are primarily based on 95% CIs.

#	Section # and Name	Description of Change	Rationale for Change
8	8.3.3.4, Analysis of Difference in Mean Partial Credit Using ITT and mITT Analysis Populations	Clarified partial credit analysis: The partial credit will be calculated assigning a partial credit score to each of the DOOR categories. For this analysis, DOOR categories 3 (alive with two events) and 4 (alive with three events) will be combined such that DOOR will be analyzed using a 4-category ranking. QoL is not considered for analysis of partial credit score. The difference in mean partial credit score will be summarized in Figure 23.	Removed unnecessary text and clarified DOOR categories will be combined
9	8.4.1, Analysis of DOOR at Day 42 and Day 70 Using CE Analysis Population	Updated text: These analyses will evaluate the null hypotheses described above using the DOOR probability Pr(Desirable DOOR in dalbavancin) + 0.5 Pr(Equal DOOR), estimated by Wilcoxon-Mann-Whitney Statistic corrected for ties, divided by the product of the two group sample sizes and corresponding CIs calculated by the method described in Halperin et al. [**Error! Reference source not found.**].	Clarified that DOOR probability is being used
10	N/A	Removed Section 8.4.3.1 from SAP v1.0	Removed section as analyses using the Wilcoxon rank-sum test will not be performed
11	9.1, Demographic and Other Baseline Characteristics<br>Appendix C	Added additional listing for baseline *S. aureus* bacteremia diagnoses	New listing added
12	9.2, Measurements of Treatment Compliance	Added clarification text for how duration of antibiotics will be calculated: Duration of antibiotics will be calculated using three different start dates: (1) date of first positive blood culture, (2) date of first negative blood culture, and (3) date of randomization. The stop date will be the latest end date recorded for each antibiotic.	Clarification and additional information
13	9.6, Clinical Laboratory Evaluations	Updated text noting where PCS criteria are listed: The criteria for PCS laboratory values will be detailed in the table footnotes.	Clarification
14	9.7, Vital Signs and Physical Evaluations<br>Appendix C, Listing 24	Added listing of all subject hospitalizations.	New listing added

DMID 20-0002 SAP Version 1.0, 03OCT2023
Updated in
DMID 20-0002 SAP Version 2.0, 03JAN2024

#	Section # and Name	Description of Change	Rationale for Change
15	Appendix A, Table 3	Updated disposition categories for standard of care treatment. Clarified in table footnote how duration of standard of care treatment is calculated.	Updated to better align with how standard of care antibiotics were collected.
16	Appendix A, Table 6	Removed duplicate row	Correction
17	Appendix A, Table 7	Added three duration columns: Duration from Date of First Positive Blood Culture; Duration from Date of First Negative Blood Culture; Duration from Date of Randomization	Clarification. Updated based on discussion with PIs
18	Appendix A, Table 11	Modified list of comorbid conditions in Table	Clarification. Updated based on discussion with PIs
19	Appendix A, Table 20, Table 22, Table 25, Table 31	Updated column header to Pr(Better DOOR in Dalbavancin Arm)<br>Updated language in footnote to clarify that 95% CI will be calculated using method from Halperin et. al.	Correction
20	Appendix A, Table 24	Removed Bootstrap 95% CI	Correction
21	Appendix A, Table 41	Updated shell to remove individual 95% CIs for each treatment group and add a column for overall DOOR probability and 95% CI	Correction
22	N/A	Removed Appendix A, Table 55 from SAP v1.0	Removed table as analyses using the Wilcoxon rank-sum test will not be performed and remaining data are reported in other tables.
23	Appendix A, Table 79, Table 81	Updated implementation note and corresponding footnotes for PCS criteria and criteria for ALP, Total Bilirubin, AST, and ALT	Correction
24	Appendix A, Table 96	Updated to only include serum chemistry parameters with PCS criteria	Correction
25	Appendix A, Table 102, Table 103, Table 104	Removed tables of laboratory summary statistics for Mean Corpuscular Volume, Mean Corpuscular Hemoglobin, and Mean Corpuscular Hemoglobin Concentration and replaced with Neutrophils, Lymphocytes, and Monocytes	Updated due to protocol amendment which removed MCH, MCV, MCHC and correction to add missing tests
26	Appendix B, Figure 30, Figure 31, Figure 32, Figure 33	Updated title to specify Risk Difference instead of DOOR Probability	Correction
27	Appendix C, Listing C	Added column for Actual Study Day	Clarification and consistency across listings
28	N/A	Removed Appendix 4, NCA Template	Correction

CLINICAL RESEARCH IN INFECTIOUS DISEASES

Pharmacokinetic
Data Analysis Plan

For DMID Protocol: 20-0002

Study Title: Dalbavancin as an Option for Treatment of *S. aureus* Bacteremia (DOTS): A Phase 2b, Multicenter, Randomized, Open-Label, Assessor Blinded Superiority Study to Compare the Efficacy and Safety of Dalbavancin to Standard of Care Antibiotic Therapy for the Completion of Treatment of Patients with Complicated *S. aureus* Bacteremia

Version 2.0

19JAN2024

Prepared and Distributed by:
Emmes
Rockville, Maryland USA

RESTRICTED

TABLE OF CONTENTS

ACRONYMS AND ABBREVIATIONS 3
1. PREFACE 6
2. CLINICAL STUDY METHODS 7
2.1. Analysis Groups 7
2.2. Dose Administration 7
2.3. PK Sampling Schedule 8
2.4. Analytical Methods 8
2.5. Collection of Pharmacodynamic Endpoints 8
3. ANALYSIS DATASET METHODS 10
3.1. Analysis Population 10
3.2. Covariates 10
3.3. Handling of Missing Data 11
4. PHARMACOKINETIC DATA ANALYSIS METHODS 13
4.1. Analysis Overview and General Methods 13
4.2. Exploratory Data Analysis 14
4.3. Estimation of Individual Noncompartmental (NCA) PK Parameters 14
4.4. Population PK Analysis 16
4.5. Exposure-Response Relationship 21
4.6. Reporting Conventions 25
4.7. Additional Analysis 26
5. REFERENCES 27
APPENDICES 28
APPENDIX 1. TABLES MOCKUPS 29
APPENDIX 2. FIGURES MOCKUPS 48
APPENDIX 3. LISTINGS MOCKUPS 67

ACRONYMS AND ABBREVIATIONS

Standard acronyms and abbreviations are listed below.

Abbreviation	Definition
AIC	Akaike Information Criterion
ALT	Alanine Aminotransferase
AST	Aspartate Aminotransferase
AUC	Area Under the Concentration-Time Curve
$AUC_{0\text{-}1day}$	AUC from the first 24 hours
$AUC_{0\text{-}8day}$	AUC from Days 0-8
$AUC_{0\text{-}22day}$	AUC from Days 0-22
$AUC_{0\text{-}42day}$	AUC from Days 0-42
$AUC_{0\text{-}70day}$	AUC from Days 0-70
$AUC_{8\text{-}22day}$	AUC from Days 8-22
$AUC_{8\text{-}15day}$	AUC from Days 15-22
$AUC_{22\text{-}42day}$	AUC from Days 22-42
$AUC_{42\text{-}70day}$	AUC from Days 42-70
$AUC_{0\text{-}last}$	AUC to the Last Measurable Concentration
$AUC_{0\text{-}\infty}$	AUC Extrapolated to Infinity
$\%AUC_{ex}$	Percentage of $AUC_{0\text{-}\infty}$ obtained by extrapolation from the time of the last measured concentration to infinity
BMI	Body Mass Index
BQL	Below the Quantification Limit
BSA	Body Surface Area
C_{max}	Maximum Plasma Concentration after the First Dose on Day 1
C_{8day}	Plasma Concentration on Day 8
C_{22day}	Plasma Concentration on Day 22
C_{42day}	Plasma Concentration on Day 42
C_{70day}	Plasma Concentration on Day 70
C_{last}	Last Measurable Concentration (above the quantification limit)
CCV	Constant Coefficient of Variation
CI	Confidence Interval
CL	Clearance
C_{max}	Maximum Concentration
CrCl	Serum Creatinine Clearance
CV	Coefficient of Variation
CWRES	Conditional Weighted Residuals

ACRONYMS AND ABBREVIATIONS (*continued*)

Abbreviation	Definition
df	Degrees of Freedom
DOOR	Desirability of Outcome Ranking
EBE	Empirical Bayes Estimate
FDA	Food and Drug Administration
FOCE	First-Order Conditional Estimation method
FOCEI	FOCE with Interaction
f_u	Fraction of Unbound Plasma Concentration
GM	Geometric Mean
GSD	Geometric Standard Deviation
IBW	Ideal Body Weight
IIV	Inter-Individual Variability
IPRED	Individual Predicted Concentrations
IWRES	Individual Weighted Residuals
LL	Log-Likelihood
LLOQ	Lower Limit of Quantification
LRT	Likelihood Ratio Test
mL	Milliliter
Max	Maximum
Min	Minimum
MRSA	Methicillin-Resistant *Staphylococcus aureus*
MSSA	Methicillin-Susceptible *Staphylococcus aureus*
MVOF	Minimum Value of the Objective Function
NCA	Noncompartmental Analysis
NONMEM	Nonlinear Mixed Effects Model
NPDE	Normalized Prediction Distribution Error
pcVPC	Prediction-Corrected Visual Predictive Check
PK	Pharmacokinetic
PopPK	Population Pharmacokinetics
CPRED	Predicted Concentrations Using Final Pop-PK Model
QoL	Quality of Life
$\|r\|$	Absolute Value of Pearson Correlation
RRT	Renal Replacement Therapy
RSE	Relative Standard Error
SCr	Serum Creatinine
SD	Standard Deviation

ACRONYMS AND ABBREVIATIONS (*continued*)

Abbreviation	Definition
τ	Length of the Dosing Interval
$t_{1/2}$	Apparent Terminal Elimination Half-Life
T_{last}	Time of Last Measurable Concentration
T_{max}	Time to Obtain Maximum Concentration corresponding to C_{max}
TOC	Test of Cure
TVP	Typical Value of Population Parameter
ULOQ	Upper Limit of Quantification
V_c	Volume of Distribution in Central Compartment
V_d	Volume of Distribution During Terminal Phase
V_p	Volume of Distribution in Peripheral Compartment
VPC	Visual Prediction Check
V_{ss}	Steady-state Volume of Distribution
λ_z	Terminal Phase Elimination Rate Constant

1. PREFACE

DMID Protocol 20-0002 is a phase 2b, multicenter, randomized, open-label, assessor-blinded superiority study to compare the efficacy and safety of dalbavancin to standard of care (SOC) antibiotic therapy for the completion of Treatment of Patients with Complicated *Staphylococcus aureus* Bacteremia. Statistical analysis of safety and efficacy will be described in the main statistical analysis plan (SAP). This SAP is limited to analysis of pharmacokinetics (PK) of plasma concentrations of dalbavancin. Blood samples are collected from subjects randomized to dalbavancin only. Approximately 200 subjects will be randomized to dalbavancin or SOC treatment groups in a ratio of 1:1. Statistical methods and mockups for tables, figures, and listings are provided for summarizing results of analysis of dalbavancin plasma-concentration and PK parameters, and their association with clinical outcomes. Pop-PK analysis will be performed to study association with relevant covariates. PK analysis in this study is regarded as exploratory with the following objectives.

- To characterize the population pharmacokinetic profile for dalbavancin administered via a 2-dose regimen (1500 mg on day 1 and day 8, renally adjusted when appropriate) in patients with *Staphylococcus aureus* bacteremia
- To assess patient-level and clinical covariates associated with dalbavancin pharmacokinetics in patients with *Staphylococcus aureus* bacteremia
- Examine the association between individualized plasma concentration profiles and clinical and microbiologic outcomes at Day 42 and Test of Cure (TOC) at Day 70
- Examine the association between individualized plasma concentration profiles and occurrence of adverse drug events, including occurrence of AST/ALT elevation >3x upper limit of normal
- Examine the association between individualized plasma concentration profiles and late recurrence risk among the subset of patients with osteomyelitis and a 6-month follow-up visit

2. CLINICAL STUDY METHODS

2.1. Analysis Groups

Approximately 200 subjects are being randomized 1:1 to receive either dalbavancin or standard of care antibiotics for completion of treatment of their complicated *S. aureus* bacteremia. Subjects are stratified by presence of Methicillin-Resistant *S. aureus* (MRSA) vs Methicillin-Susceptible *S. aureus* (MSSA). Blood samples for PK are only being collected from subjects who receive dalbavancin, and PK analyses will be limited to those. Subjects will be divided into 6 groups according to the dose(s) of dalbavancin administered, depending on the level of serum creatinine clearance (CrCl), described in Section 2.2.

i) Subjects receiving a first dose of 1500 mg and a second dose of 1500 mg IV dalbavancin

ii) Subjects receiving a first dose of 1500 mg and a second dose of 1125 mg IV dalbavancin

iii) Subjects receiving a first dose of 1125 mg and a second dose of 1500 mg IV dalbavancin

iv) Subjects receiving a first dose of 1125 mg and a second dose of 1125 mg IV dalbavancin

v) Subjects receiving a single dose 1500 mg IV dalbavancin

vi) Subjects receiving a single dose 1125 mg IV dalbavancin

vii) All subjects who received dalbavancin

2.2. Dose Administration

The first dose of dalbavancin is administered on Day 1, and the second dose is scheduled for Day 8±1 but second doses administered out-of-window are included in the analysis and formulation of analysis groups. At either dosing visit, the dosage of dalbavancin administered will be determined based on individual estimated serum creatinine clearance (CrCl) levels as follows:

Subjects with CrCl ≥ 30 mL/min and subjects receiving regular hemodialysis or peritoneal dialysis will receive 1500 mg IV dalbavancin over 30 (± 10) minutes. Subjects with CrCl < 30 mL/min who are not receiving regular hemodialysis or peritoneal dialysis will receive 1125 mg IV dalbavancin over 30 (± 10) minutes.

A repeat serum creatinine will be required within the 72 hours prior to the second dalbavancin dose. The need for additional serum creatinine measurements at the second dosing visit is at the clinical discretion of the study investigator. If CrCl transverses the 30 mL/min threshold in either direction after Day 1 but before the second dose, then the dose should be adjusted accordingly. For example, if a subject has a CrCl ≥ 30 mL/min on Day 1 and received 1500 mg IV dalbavancin, but the CrCl is < 30 mL/min on day 8, then that subject will receive 1125 mg IV for the second dose. If CrCl is <30 mL/min on Day 1 but increases to ≥ 30 mL/min between Day 1 and Day 8, then the subject will receive 1500 mg IV dalbavancin for the second dose.

Data will be summarized by dalbavancin dose groups as specified in Section 2.1. Subject actual dose administration times will be presented (Listing 1).

2.3. PK Sampling Schedule

Dalbavancin PK samples will be drawn only for subjects receiving dalbavancin. PK samples are scheduled to be drawn on Day 1 prior to dose, at end of infusion +10 minutes, 6 ± 2 hours post end of dose, 12 ± 4 hours post end of dose, 24 ± 6 hours post end of dose), Day 8 ± 1 day (prior to 2nd dose), Day 22 ± 2 days (at time of clinic visit), Day 42 ± 3 days, Day 70 ± 7 days, and with any Early Termination (ET) visit. The actual date and time of each blood sample collection will be recorded in the subjects' source document and the eCRF.

Blood collected will be processed for plasma separation at the study site's laboratory; subsequent PK analyses will be performed on the plasma samples at a central laboratory. Plasma PK specimens will be processed and stored in a -70ºC freezer until time of shipment to the central PK laboratory for analysis. Detailed instructions for the preparation, handling, and storage of plasma PK specimens are detailed in the study MOP including aliquots of specimens, temperature requirements, where they will be stored, and how they will be labeled.

Dalbavancin plasma concentrations will be listed by dosage group, subject, nominal timepoint, and actual time of sample collection (Listing 2).

2.4. Analytical Methods

Plasma samples for PK analysis will be analyzed at a central laboratory, Keystone Bioanalytical, Inc (Keystone) for dalbavancin concentration. The central laboratory will complete bioanalysis to determine bound and unbound dalbavancin concentration in plasma. The calibration curve for this assay is 0.05 µg/mL to 50 µg/mL: the lower limit of quantification (LLOQ) is 0.05 µg/mL, and the upper limit of quantification (ULOQ) is 50 µg/mL. Key coefficients of variation for this assay are as follows:

- Inter-Assay Precision for Standards: %CV: 0.20% to 4.74%
- Intra-Assay Precision for QCs: %CV: 0.46% to 19.09%
- Inter-Assay Precision for QCs: %CV: 0.59% to 15.33%

2.5. Collection of Pharmacodynamic Endpoints

The clinical and microbiological endpoints to be used in pharmacodynamics analysis are the following:

- Desirability of Outcome Ranking (DOOR) at Day 42 and Day 70
- Clinical efficacy at Day 42 and Day 70
- Occurrence of AST/ALT elevations >3x ULN from first dose through Day 42
- Late recurrence risk among patients with osteomyelitis

The clinical components of the DOOR endpoint will be completed by an independent adjudication committee, blinded to treatment assignment. The DOOR endpoint will be based on a combination of clinical failure, infectious complications, SAEs or AEs leading to study drug discontinuation, and mortality.

Clinical success is defined as the resolution of clinical signs and symptoms of *S. aureus* bacteremia such that no additional antibiotic therapy is required or anticipated for its treatment at the specified timepoint. It is possible to achieve overall clinical success status even if infectious complications have occurred prior to the time at which it is assessed. Clinical success reflects the patient's overall status at the time of that assessment. Clinical failure is defined as the absence of clinical success.

Infectious complications are defined as occurrence of any of the following, between randomization and the specified timepoint:

- Endocarditis
- New evidence of metastatic foci of infection – e.g., osteomyelitis, visceral abscess, septic joint
- Relapse – isolation of baseline *S. aureus* pathogen from a blood culture drawn after randomization
- Readmission for subsequent care of indication under study
- Need for additional unplanned source control procedures – e.g., abscess debridement or drainage, cardiac valve replacement
- Change in antibiotic therapy due to inadequate clinical response. For any changes to study drug in the standard of care group, or when new antibiotics are started in either treatment group, the site PI will record the reason for the antibiotic change.

Clinical efficacy is defined as the absence of clinical failure, infectious complications, and all-cause mortality.

Late recurrence risk is defined by the presence of the following up to 6 months after randomization: progressive image changes along with isolation of *S. aureus* from blood, bone biopsy, associated fluid aspiration, or operative tissue culture.

These endpoints are described in more detail in the protocol.

3. ANALYSIS DATASET METHODS

This section describes the definitions and processes to be used in the creation of analysis-ready datasets.

3.1. Analysis Population

The analysis dataset for use in all PK analyses will consist of the PK evaluable population, defined as all subjects who receive at least 1 dose and have at least 1 measurable plasma concentration value.

3.2. Covariates

Patient demographic and clinical descriptors that will be evaluated for their potential to explain a portion of the inter-individual variability (IIV) in selected unbound and total plasma dalbavancin PK parameters include sex, age in years, actual weight in kg, lean body weight, body surface area (BSA) in m^2, body mass index (BMI) in kg/m2, and ideal body weight (IBW) in kg. BSA will be calculated using the method of Gehan and George [1] as shown below in Equation 1.

(5) $BSA = 0.0235 \times \text{height (cm)}^{0.42246} \times \text{weight (kg)}^{0.51456}$

IBW will be calculated separately for males and females using reference equations [2] as shown in Equations 2 and 3:

(2) For males: IBW = 50 + 0.91× (height(cm) – 152.4)

(3) For females: IBW = 45.5 + 0.91× (height(cm) – 152.4)

Baseline clinical laboratory data utilized as covariates will include serum creatinine (SCr) in mg/dL and albumin in g/dL. CrCl in mL/min/1.73 m^2 will be calculated using the Cockcroft and Gault method [3] and normalized to a BSA of 1.73 m^2. Depending on the magnitude of variation over time of these variables, these may be included as time-varying covariates.

Renal replacement therapy (RRT) will also be considered as a covariate. This will be implemented dependent on the type, timing, and duration of RRT with respect to the course of PK sample collection and to dalbavancin dosing.

Summaries of categorical demographic and clinical covariates considered for modeling will be presented for the PK population by dalbavancin dosage group and overall (Table 2). Continuous covariates will be similarly summarized in Table 3. Covariates to be evaluated are presented in Table 1 along with which PK parameters (clearance or volume of distribution) they are to be evaluated on.

Table 1: Covariates Evaluated in PopPK Modeling

Covariate	Time Varying	Pharmacokinetic Parameter		
		Clearance	Volume of Distribution (Central or Peripheral Volume)	Fraction Unbound
Age	No	X	X	X
Sex	No	X	X	X
Actual Body Weight	No	X	X	
Lean Body Weight	No	X	X	
BMI	No	X	X	
BSA	No	X	X	
IBW	No	X	X	
CrCl	Yes	X		X
SCr	Yes	X		X
Albumin	Yes	X	X	X
RRT	Yes	X	X	

3.3. Handling of Missing Data

3.3.1. Handling of Missing Concentrations

To handle missing concentrations and those below the lower limit of quantification (BQL), the Method 4 described by Beal [5] will be used. In the case where >10% of concentrations are BQL, other methods will be considered. This method allows BQL to be retained but handle them as censored. It also assumes that the measured concentrations cannot be negative.

3.3.2. Identification and Handling of Outliers

An outlier is defined as an aberrant observation that substantially deviated from the rest of the observations within an individual. PK outlier concentrations identified as outlying data points are to be excluded from this analysis given the potential for these observations to negatively impact model convergence and/or the final parameter estimates. Observations of concentrations or subjects that may be outliers will be examined with the study team to determine potential reasons for outlying values.

Outlier detection in the analysis dataset is based primarily upon visual inspection of individual and pooled unbound and total plasma dalbavancin concentration-time data. As suggested by the FDA guidance, searching for additional outliers during the analysis will be based upon graphical exploration of individual and population conditional weighted residuals during structural PK model development; observations with normalized weighted residuals greater than 5 were identified as outliers. All concentrations from a single patient could be excluded cautiously only if the entire intensively sampled PK profile failed to follow a reasonable pattern relative to the dosing [4]. If most of the suspected outlier concentrations appear to occur at roughly the same time since last dose, additional attempts will be made to update the structural model to try to capture these observations. Outliers will be excluded from the analysis data, but sensitivity analyses will be performed comparing the inclusion of outliers on the final model.

3.3.3. Handling of Missing Covariate Data

If more than 20% of subjects have missing data for a given covariate, then that covariate will be excluded from the analysis. For covariates with <20% missingness in the analysis dataset, efforts will be made to identify any patterns to the missingness with respect to dosage and other covariates of interest. If a covariate is determined to reasonably be missing completely at random, then missing values will be imputed using simplistic methods: stationary categorical variables will be imputed using the most common value in the dataset; stationary continuous variables will be imputed using the median data value; time-varying covariates will be imputed using last-observation-carried-forward methods for non-baseline values, and if a baseline value is missing, it will be imputed using the next available value; if all time-varying values for a subject are missing, the group-specific median will be used to impute the baseline value, where group is determined appropriately for the type of data point.

4. PHARMACOKINETIC DATA ANALYSIS METHODS

4.1. Analysis Overview and General Methods

Initial steps of the modeling will be applied to total and unbound plasma concentration-time profiles separately, followed by simultaneous modeling of total and unbound plasma to establish a population PK model that simultaneously fits the total and unbound dalbavancin plasma concentration-time data:

1. Exploratory data analysis (EDA) – total and unbound profiles separately
2. Noncompartmental Pharmacokinetic Analysis (NCA) – total and unbound profiles separately
3. Population Pharmacokinetic Analysis (PopPK) – total and unbound simultaneously
    a. Base model development
    b. Covariate analysis
    c. Model refinement
    d. Model evaluation

EDA will be performed using R version 4.0 or later and/or SAS software version 9.4 (SAS Institute Inc., Cary, NC). NCA will be performed using version 8.2 or later of Phoenix® WinNonlin® software (Certara USA, Inc. Princeton, NJ). PopPK model development will utilize the non-linear mixed effects modeling software NONMEM Version 7.3 or higher (ICON Development Solutions, Ellicott City, MD) or Pumas software.

Throughout the PopPK analysis, model development will be guided using graphical and numerical methods of model diagnostics. These methods include, but are not restricted to:

- Convergence of NONMEM estimation and gradients of parameters in final steps of iteration.
- Reasonable parameter estimates and precision (% relative standard error).
- Comparisons of IIV and RV estimates and precision.
- Scatterplots of individual and population-predicted total and unbound concentrations vs. respective observed concentrations.
- Scatterplots of population-predicted total and unbound concentrations vs. respective residuals and weighted residuals
- Scatterplots of weighted residuals (population, and individual) over time since first or last dose
- Scatterplots of individual predicted total and unbound concentrations vs. respective individual weighted residuals and their absolute values.
- Histograms of individual eta-values.
- Scatterplots of individual eta-values of parameter pairs (eta biplots)

Many of these diagnostic plots will be stratified according to dosing group as described in Section 2.1.

4.2. Exploratory Data Analysis

Exploratory analyses of unbound and total dalbavancin plasma concentration-time data are presented on both a linear and semi-log scale to visually ascertain the general model structure that may best describe the data. These will include, but may not be limited to:

- Descriptive summary statistics including mean, standard deviation (SD), median, minimum, maximum, coefficient of variation as a percent (CV%), geometric mean (GM) and geometric mean standard deviation (GSD) for total and free plasma concentrations at each sampling time by dalbavancin dosage group and overall (Table 4).
- Scatterplots of individual subject total and free plasma PK profiles at all post-dose time points by dalbavancin dosage group and all dalbavancin subjects (Figure 1).
- Linear and semi-log plots of total and free plasma linear/geometric mean concentration ± 1 SD/GSD by dalbavancin dosage group (Figure 2, Figure 3, Figure 4, and Figure 5).
- Linear and semi-log scatterplots of fraction unbound concentration over time since last dose (Figure 6).
- Scatterplots of fraction unbound concentration vs. total concentration (Figure 7).
- Scatterplots/Histograms/Frequency tables describing relationship between covariates (continuous/categorical)

These figures will be used to investigate outlier concentration-time data for erroneous time or concentration data point entries.

4.3. Estimation of Individual Noncompartmental (NCA) PK Parameters

PK parameters for total and free dalbavancin observed concentration-time data will be estimated through a NCA using version 8.2 or later of Phoenix WinNonlin software (Table 5). Actual post-dose time will be used for the estimation of PK parameters instead of nominal times, but nominal times are used for labels (e.g., “Day 8” is used for Visit 2 where draws or doses may be out-of-window). The PK parameters will include maximum total and free plasma concentration (C_{max}) after the first dose on Day 1, time to C_{max} (T_{max}), plasma concentration on Day 8 (C_{8day}), plasma concentration on day 22 (C_{22day}), concentration on day 42 (C_{42day}), concentration on day 70 (C_{70day}), area under the plasma concentration-time curve (AUC) in the first 24 hours ($AUC_{0\text{-}1day}$), AUC from days 0-8 ($AUC_{0\text{-}8day}$), AUC from days 0-22 ($AUC_{0\text{-}22day}$), AUC from days 0-42 ($AUC_{0\text{-}42day}$), days 0-70 ($AUC_{0\text{-}70day}$), AUC from days 8-22 ($AUC_{8\text{-}22day}$), AUC from days 22-42 ($AUC_{22\text{-}42day}$), AUC from days 42-70 ($AUC_{42\text{-}70day}$), AUC to the last quantifiable sample ($AUC_{0\text{–}last}$), AUC to infinity ($AUC_{0\text{–}\infty}$), $T_{1/2}$, CL, V_d, f_u, and λ_z. PK parameters estimated from the NCA will be summarized by dosage group in Table 5 and a listing of parameters provided (Listing 3).

Following settings of Phoenix and WinNonlin will be used to compute parameters.

- Linear Up Log Down calculation method
- Uniform weighting
- IV dosing
- Lambda Z Acceptance Criteria
  - Rsq_adjusted $\geq$ 0.90
  - Span $\geq$ 3 half-lives
  - Includes at least 3 timepoints after T_{max}

Actual infusion and sampling times will be used in the NCA. Only the PK parameters that meet the predefined criteria listed above will be included in summary tables and figures and statistical analyses. In the case that the Rsq_adjusted Lambda Z acceptance criteria is not met, the terminal phase PK parameters (λ_z, $t_{1/2}$, $AUC_{0-\infty}$,) will be excluded from summary presentations and statistical analyses. Regardless of whether criteria are met, all PK parameters will be reported in listings.

C_{max}

C_{max}, maximum plasma concentration after the first dose on Day 1, is defined as the maximum observed drug concentration observed in plasma over all PK sample concentrations post-dose on Day 1. C_{max} will be obtained from the **C**$_{max}$ parameter calculated using WinNonlin. If there is no measurable concentration in the subject's PK profile, then C_{max} will be missing for that subject. Dalbavancin plasma concentration on Day 8, Day 22, Day 42, Day 70, and last measured plasma concentration will be described using notations C_{8day}, C_{22day}, C_{42day}, C_{70day}, and C_{last}, respectively. All concentration parameters will be estimated on observed concentration-time data as well as simulated concentration-time data produced from the final pop PK model. Concentration parameters will be reported in units of µg/mL.

T_{max}

Time of maximum concentration (T_{max}) is defined as the time at which the maximum concentration on Day 1 that (C_{max}) occurs. T_{max} will be obtained from the T_{max} parameter calculated by WinNonlin. If there is no measurable C_{max} in the subject's PK profile, then T_{max} will be missing for that subject. T_{max} will be reported in units of hours.

Λ_z

The terminal phase elimination rate constant (λ_z) is defined as the first-order rate constant describing the rate of decrease of drug concentration in the terminal phase (defined as the terminal region of the PK curve where drug concentration follows first-order elimination kinetics). Λ_z will be computed as the slope of a terminal region consisting of $\geq$ 3 successive points in the plot of log-transformed concentration data versus time. Λ_z will be estimated using uniform weighting. Timepoints used in the estimation of λ_z will only be selected using the WinNonlin automatic algorithm. The λ_z parameter will be obtained from the Lambda_z parameter calculated by WinNonlin and will be reported in units of 1/h.

$T_{1/2}$

The terminal phase half-life ($T_{1/2}$) is defined as the time required for the drug to decrease by a factor of one-half in the terminal phase. The $T_{1/2}$ parameter can be estimated as ln(2)/ λ_z and will be obtained from the HL_Lambda_z parameter calculated by WinNonlin. Half-life will be reported in units of hours.

AUC

Area under the curve (AUC) will be estimated by the WinNonlin software using the Linear Up Log Down calculation method. The AUC from time of the first dose (time 0) to Day 1 (24 hours), Day 8, Day 22, Day 42, Day 70, last observed concentration, and extrapolated to infinity will be described using notations AUC_{0-1day}, AUC_{0-8day}, $AUC_{0-22day}$, $AUC_{0-42day}$, $AUC_{0-70day}$, AUC_{0-last}, and $AUC_{0-\infty}$, respectively. The AUC for the time-intervals Day 8 to Day 22, Day 15 to Day 22, Day 22 to Day 42, and Day 42 to Day 70 will be described using notations $AUC_{8-22day}$, $AUC_{15-22day}$, $AUC_{22-42day}$, $AUC_{42-70day}$, respectively. All AUCs will be reported in units of µg*h/mL.

$AUC_{0-\infty}$ is defined as the total area under the concentration-time curve from the time of the first dose on Day 1 (time 0) taken to the limit as the end time becomes arbitrarily large, based on the last observed concentration, as seen in Equation 4:

(4) $AUC_{(0-\infty)} = AUC_{(0-\text{last})} + \frac{C_{\text{last}}}{\lambda_z}$,

where C_{last} is the last measured concentration > LLOQ. $AUC_{0-\infty}$ will be obtained from the AUCINF parameter from WinNonlin. If the amount extrapolated portion of $AUC_{0-\infty}$ is >25%, the estimated $AUC_{(0-\infty)}$ value will be flagged when listed in the report and will be excluded from statistical summaries of parameter estimates and downstream calculations.

$\%AUC_{ex}$ is defined as the percentage of $AUC_{0-\infty}$ obtained by extrapolation from the time of the last measured concentration to infinity. $\%AUC_{ex}$ can be calculated by dividing the difference between $AUC_{0-\infty}$ and AUC_{0-last} by $AUC_{0-\infty}$.

If $\%AUC_{ex}$ is >20%, the estimated $AUC_{(0-\infty)}$ will be excluded from statistical summaries of PK parameter estimates and downstream calculations. $\%AUC_{ex}$ will be obtained from the AUC_%Extrap_obs parameter calculated by WinNonlin

AUC parameters will be estimated from observed concentration-time data as well as simulated concentration-time data produced from the final pop-PK model.

CL

Clearance (CL) is defined as the volume of plasma completely cleared of drug per unit time and is estimated as the dose divided by $AUC_{0-\infty}$. This parameter is not reported if the extrapolated portion of $AUC_{0-\infty}$ exceeds 25%.

V_d

Volume of distribution (V_d) is estimated as CL/ λ_z. This parameter is not reported if the extrapolated portion of $AUC_{0-\infty}$ exceeds 25%.

F_u

Unbound fraction (f_u) is estimated as the ratio of unbound to total dalbavancin concentration in plasma. This will be measured at each nominal post-dose timepoint where both unbound and total concentrations are available. Timepoints to be reported will be at 24h, Day 8, Day 22, Day 46, and Day 70; respectively denoted as f_{u1day}, f_{u8day}, f_{u22day}, f_{u42day}, and f_{u70day}. These estimates may not be estimable using aforementioned NCA procedures, since NCA is performed in unbound and total concentrations separately but will nonetheless be reported alongside other NCA parameters in tables and listings.

4.4. Population PK Analysis

4.4.1. Base Structural Model

Base model development will consist of the determination of structural, interindividual variability (IIV), and residual unexplained variability (RUV) models. The overall goal of base model development will be to determine stable and parsimonious model that provide an adequate description of the data. The adequacy of the model to describe the available data will be assessed using standard goodness-of-fit diagnostic plots, and the stability and parsimoniousness of the model will be assessed based on Akaike Information Criteria (AIC), as well as condition number and precision of parameter estimates. It is important to obtain a stable parsimonious base model to facilitate the assessment of covariate effects on structural model parameters in the

subsequent development of the population model. AIC is selected as the criteria to assess the parsimoniousness of the model as it is applicable to both nested and non-nested models. AIC is defined as $2p \quad -2LL$, where p is the number of parameters estimated in the model, and LL is the log-likelihood. A summary of the model selection process is given in Table 6.

Based on prior dalbavancin PK modeling studies [6], a three-compartment model with zero-order input and first-order elimination to simultaneously fit the total and unbound plasma dalbavancin concentration-time data will be used as the initial structural PK model. Other two- and three-compartment models will be considered as necessary. PopPK models will be described by estimation of the typical values of structural model parameters of total plasma such as intercompartmental rate constants (k_{ij}), volumes of distribution in central and peripheral compartment(s) (Vc, Vp or Vp_i), central clearance (CL), and/or intercompartmental clearance(s) (Q or Q_i), as well as a function of fraction unbound (f_u) in the context of simultaneous unbound-total plasma modeling.

In the initial base model, the fraction of dalbavancin unbound (f_u) will be assumed to be a constant function of total dalbavancin concentration, and the concentration dependence of f_u will be considered as necessary. Specifically, non-linear plasma protein binding as a function of total dalbavancin concentration will also be considered to identify the most parsimonious model which provides the most plausible fit to the data. If necessary, saturable protein binding will be implemented using a quasi-equilibrium binding model, similar in nature to a target-mediated drug disposition model [12, 13], in which the maximal binding capacity (Bmax, in mg/L) and binding dissociation constant (KD, in mg/L) are estimated. Using this approach, f_u would not be constant and free dalbavancin concentrations would instead be a non-linear function of the total dalbavancin concentrations.

First-order conditional estimation method with interaction (FOCEI) will be utilized to fit the models as the RUV model is expected to be heteroscedastic.

4.4.1.1. Modeling Interindividual Variability

Initially, interindividual variability (IIV) will be estimated for all parameters using the exponential model as described in Equation 5, assuming a log-normal distribution of individual-specific parameter estimates

(5) $X_j = \tilde{X}_j \times \exp{(\eta_i^x)}$

where X_j is the individual-specific estimate of the X parameter in subject j, $\tilde{X}_j$ is the typical value of the parameter X in subject j and η_j^x is the persistent different between the individual-specific and typical values of the parameter in subject j, a random variable distributed $iid \sim N(0, {\omega_x}^2)$. With this variability model, estimates will be presented as coefficients of variation, estimated as $\%CV_x = 100 \times \sqrt{{\omega_x}^2}$.

4.4.1.2. Modeling Residual Variability

Residual variability (RV) represents a composite of assay variability, intra-subject variability, model misspecification, errors in timing of dose and sample information, subject non-compliance, and other unexplained errors. Goodness-of-fit plots will inform the choice of RV models, evaluated using one of the forms below:

- Constant Coefficient of Variation (CCV) Error Model: $C_{ijk} = \hat{C}_{ijk} \times (1 + \varepsilon_{ijk})$
- Additive Plus CCV Error Model: $C_{ijk} = \hat{C}_{ijk} + \varepsilon_{1ijk} + \hat{C}_{ijk} \times (1 + \varepsilon_{2ijk})$
- Log Error Model: $\log{(C_{ijk})} = \log{(\hat{C}_{ijk})} + \varepsilon_{ijk}$

Where C_{ijk} is the jth measured value in the ith subject, where k=1,2 for total or unbound concentration, $\hat{C}_{ijk}$ is the jth predicted value in the ith subject using the specified model, and $\varepsilon_{ijk}/\varepsilon_{1ijk}/\varepsilon_{2ijk}$ are random variables describing random errors, distributed $iid \sim N(0, {\sigma_k}^2)/N(0, {\sigma_{1k}}^2)/\ N(0, {\sigma_{2k}}^2)$.

4.4.2. Covariate Model Development

Following the development of an appropriate base structural model, a covariate model will be developed describing the influence of covariates on selected parameters, as described in Table 1. The purpose of covariate model development is to determine robust and generalizable parameter-covariate relationships.

Covariate model development will consist of exploratory analysis to select parameter-covariate relationships to be considered for inclusion in the full model, followed by development of the full model using forward selection with pre-determined ranks of correlated covariates and functional forms, then backward elimination. This approach is expected to mitigate selection of spurious covariate-parameter relationships by over-fitting of the available data [7].

4.4.2.1. Exploratory Analysis of Parameter-Covariate Relationships

The potential effects of clinical covariates on PK parameters will be explored by visual inspection of scatter and box plots (continuous and categorical variables, respectively) of the individual deviations from the population-typical value PK parameters (ETAs) against candidate covariates. To mitigate the risk of confounding due to collinearity between covariates, the correlation of continuous covariates will be determined, and for correlated covariates ($|r| > 0.6$) only a single covariate will be considered for inclusion in the full covariate model. For categorical covariates, if one subgroup represents less than 10% of the overall population, the categories may be re-grouped to avoid poorly estimated parameters due to small sample sizes.

Using the base structural PK model, individual post-hoc PK parameters will be first obtained for each subject. Each covariate will be plotted against deviations of these individual post-hoc parameter estimates from the population typical values (delta plots). These will be used as a predictive check. Functional forms of covariates will be initially pre-specified for inclusion in the model per Section 4.4.2.2.

4.4.2.2. Functional Forms of Covariate Models

Continuous covariates will be initially evaluated in the model using a power model, given by:

$$\tilde{X}_i = \theta_X^{int} \times (cov_i/\overline{cov})^{\theta_X^{cov}}$$

where $\tilde{X}_i$ is the estimated typical parameter value for the ith subject, θ_X^{int} the typical parameter value for individuals at the mean/median value of a particular covariate in the population, θ_X^{cov} the population typical value estimate of exponential effect of change in covariate value on parameter value, cov_i the covariate value in the ith subject, and $\overline{cov}$ the estimated mean or median value of the covariate in the population.

Other functional forms will be considered as necessary, e.g., the linear model:

$$\tilde{X}_i = \theta_X^{int} + \theta_X^{cov} \times (cov_i - \overline{cov})$$

Categorical covariates with N levels will be evaluated using N-1 dichotomous indicator variables, such that each of the N-1 groups is compared to one reference group. Each indicator is added to the model using the exponential shift model, given by:

$$\tilde{X}_i = \theta_X^{int} \times \exp\,(\theta_X^{cov} \times cov_i)$$

where $\tilde{X}_i$ is the estimated typical parameter value for the ith subject, θ_X^{int} the typical parameter value for individuals with $cov_i = 0$, θ_X^{cov} the population typical value estimate of function-dependent effect on θ_X^{cov} for individuals with $cov_i = 1$, and cov_i the dichotomous covariate value in the ith subject.

4.4.2.3. Forward Inclusion of Covariate-Parameter Relationships

The full covariate model will be developed by forward inclusion of the covariate-parameter relationships selected for further consideration by the exploratory analysis. The model-based evaluation of covariate effects will start with a univariate analysis of each parameter-covariate relationship, followed by a stepwise forward selection guided by pre-specified prioritizations of correlated covariates and functional forms. Terms are selected if they result in p-value<0.01 from a likelihood ratio test (LRT) of the nested models. In the case of correlated covariates, de-prioritized covariates would only be selected if they provide a significantly better model fit compared to the prioritized covariate, evaluated at the discretion of the modeler (e.g., AIC<2). A similar approach is taken with respect to functional forms. Additional criteria for inclusion in the stepwise process are: (1) there is a decrease in ω^2 for the parameter on which the covariate is added, and (2) there is not a substantial compensatory increase in σ^2 or ω^2 for the other PK parameters. Results from forward selection will be summarized in Table 8.

4.4.2.4. Full Multivariate Model Evaluation

After completion of forward selection, the IIV models and covariate functional forms will be re-evaluated. Pair-wise comparisons of the IIV terms for each parameter (ETA or η) will be graphically examined. If at least moderate correlations are observed between the η for any pairs of PK parameters (e.g., Pearson's correlation $r^2 > 0.25$), an attempt will be made to estimate the corresponding covariance (e.g., off diagonal elements of the variance-covariance matrix) between those parameters in the population PK model. In addition, the distribution of the η for each parameter will be examined for irregularities (e.g., skewness, bi-modalities, etc.) and alternative IIV models or transformations (e.g., Box-Cox) will be considered if necessary. In addition, diagnostic plots of full-model η vs. covariates will be examined to confirm that there are no evident trends in the unexplained variability of PK parameters. Other functional forms of covariate-parameter relationships will be considered to resolve any trends that are evident.

After making any potential adjustments to the IIV models or the variance-covariance matrix structure, attempts will be made toward correcting any potential biases or looking for ways to simplify the residual variability model, e.g., simplifying an additive plus CCV residual error model to a CCV error model if the data does not continue to support the estimation of the more complex model.

4.4.2.5. Backward Elimination of Covariate-Parameter Relationships

Univariate stepwise backward elimination will be applied after all adjustments are made to the IIV and residual variability models. A covariate is considered statistically significant if an LRT p-value<0.001 results when it is removed from the model. During each step of backward elimination, the most non-significant covariate (the highest p-value≥0.001) is removed from the model until all remaining covariates in the model were statistically significant (p-value<0.001). These results will be presented in Table 9.

4.4.3. Final Population Pharmacokinetic Model

The final PopPK model will be assessed using the same model diagnostic and evaluation criteria as described previously. IIV models will again be evaluated similarly as described in Section 4.4.2.4. In addition, the overall distribution of the normalized prediction distribution errors (NPDE) for the unbound and total plasma dalbavancin concentrations will be evaluated and compared to a normal distribution to determine if the fixed or random effects models are biased.

4.4.4. Final Model Evaluation

The final population PK model will be further evaluated and qualified by performing all diagnostics mentioned above. Focus will be on the visual predictive check (VPC), which graphically examines the agreement between the 5th, 50th, and 95th percentiles of the observed and the individual simulated unbound and total plasma dalbavancin concentrations across time intervals. The original analysis dataset will be used as a template to simulate unbound and total dalbavancin PK data for the same number of subjects in 1,000 new datasets with each dataset featuring the same study design and data collection scheme. Based upon graphical review of these plots, the population PK model will be refined as appropriate to try to correct any substantial issues with respect to the fixed or random effects parameters in the model if there is discordance between the 5th, 50th, and 95th percentiles of the observed and the individual simulated unbound and total plasma dalbavancin concentrations over time. Due to difference in baseline covariates and dosage schemes, the prediction-corrected visual predictive check (pcVPC) method [8] will be applied, wherein the predicted and observed concentrations are normalized to the median independent variables within the simulation bin.

Further qualification of the final model will be performed by bootstrapping the original dataset (1000 replicates) and estimating the model on each replicate to generate confidence intervals on population-level model parameters and all other measures of exposure.

The following will be provided as results from the final model. Note that in the case where the fraction unbound in the final structural model is concentration-dependent, the following figures will be duplicated as needed for both total and unbound concentrations.

- Observed concentrations vs. population predicted concentrations (CPRED) (Figure 8)
- Observed concentrations vs. individual predicted concentrations (IPRED) (Figure 9)
- Quantile-quantile plot of individual weighted residuals (IWRES) and conditional weighted residuals (CWRES) (Figure 10)
- CWRES vs. Time Since Last Dose (Figure 11) and Time Since First Dose (Figure 12)
- CWRES vs. PRED (Figure 13)
- IWRES vs. IPRED (Figure 14)
- |IWRES| vs. IPRED (Figure 15)
- Plots of observed, individual predicted (IPRED), and pop-PK predicted (CPRED) by subject (Figure 16)
- Confidence Interval pcVPC plots for total (Figure 17) and unbound (Figure 18) concentration.
- Model-predicted covariates vs. typical value parameters, overlaid upon individual post-hoc parameter values (Figure 19).

4.4.5. Empirical Bayes Estimation of parameters and exposures

Individual empirical Bayes estimates (EBEs) of PK parameters derived from the final population PK model fit for each patient's individual dosing and covariates will be generated.

Where appropriate, the steady-state total plasma volume of distribution (V_{ss}) will be calculated as the sum of the central (V_c) and peripheral volume terms (V_{p1} and V_{p2}). The alpha-phase half-life ($T_{1/2,\alpha}$) beta-phase half-life ($T_{1/2,\beta}$) and gamma-phase half-life ($T_{1/2,\gamma}$) will be calculated for each patient using the individual PK parameters. Summary statistics (mean, standard deviation, median, minimum, and maximum) will be calculated for CL, V_c, V_{p1}, V_{p2}, V_{ss}, $T_{1/2,\alpha}$, $T_{1/2,\beta}$, $T_{1/2,\gamma}$ values, f_u, and/or additional parameters of the functional

form of f_u. Summary statistics of PopPK model-fit parameters and bootstrapping analysis and estimates of PK parameters from the base structural model will be presented in Table 7.

The following exposure variable EBEs will be estimated for each patient from simulated data for free and total plasma concentrations: C_{max}, C_{8day}, C_{22day}, C_{42day}, C_{70day}, $AUC_{0\text{-}1day}$, $AUC_{0\text{-}8day}$, $AUC_{0\text{-}22day}$, $AUC_{0\text{-}42day}$, $AUC_{0\text{-}70day}$, $AUC_{8\text{-}22day}$, $AUC_{15\text{-}22day}$, $AUC_{22\text{-}42day}$, $AUC_{42\text{-}70day}$, and $AUC_{0\text{-}\infty}$. Summary statistics for these estimates will be presented in Table 10 by dosage group. A listing of PK parameters and exposure variables estimated from pop PK model will also be provided (Listing 4). Exploratory data analysis of these estimates will be further performed for these exposures with respect to dosage group and covariates, as well as any other clinical or demographic characteristic of interest. For instance, the relationship of injection drug use history and dalbavancin exposure is of particular interest.

4.5. Exposure-Response Relationship

The associations between simulated total and unbound dalbavancin exposures and each outcome of interest will be explored using standard exposure-response methodologies. For the efficacy exposure-response analyses, the relationship between exposure and various clinical and microbiological outcomes, the exposures of interest will be limited to the following total and free dalbavancin exposure variables: C_{22day}, $AUC_{0\text{-}22day}$, $AUC_{8\text{-}22day}$ and $AUC_{15\text{-}22day}$. Later exposure variables will not be examined as dalbavancin exhibits linear PK and exposures will be proportional over time [6], limiting the need to examine exposure more distal from dosing.

The following efficacy outcomes will be evaluated in the efficacy exposure-response analyses: (1) DOOR outcome (coded 1 to 5) at Day 42, (2) DOOR outcome (coded 1 to 5) at Day 70, (3) clinical efficacy (coded as 1 or 0), defined as absence of clinical failure, infectious complications, and infection-related mortality, at Day 42, (4) clinical efficacy at Day 70 (coded as 1 or 0), and (7) late recurrence at 6 months (within osteomyelitis subset) (coded as 0 or 1). Patients who were discontinued or died prior to Day 22 will be censored from the efficacy exposure-response analyses due to 22-day exposures having not been measured. If >10% of subjects who received dalbavancin fall into this category, sensitivity analyses using shorter-term exposures will be considered to assess the level of bias from excluding subjects with negative clinical outcomes.

For the safety exposure-response analyses, the associations between Day 1 total and unbound exposures (i.e., C_{max}, $AUC_{0\text{-}1day}$) and occurrence of AST/ALT elevation >3x upper limit of normal during treatment up to Day 42 (coded as 0 or 1) will be assessed. Day 1 exposures will be examined in the safety exposure-response analyses to ensure the exposure variable preceded the occurrence of AST/ALT elevations >3x upper limit of normal during treatment up to Day 42.

All models described will include *S. aureus* strain (MSSA vs. MRSA) initially detected as a covariate adjusting for exposure and subsequently as a stratification variable for subgroup analysis. Should these models display a clinically significant modulation from strain of exposure effect on outcome, further modeling will be undertaken for the interaction between exposure and strain.

Descriptive statistics of each exposure of interest with the above efficacy and safety outcomes will be provided (Table 11).

4.5.1. Categorization of Exposures

Dalbavancin exposure variables will be modeled as both continuous and categorical variables. For each dalbavancin exposure-outcome analysis, breakpoints in the distribution of each continuous dalbavancin exposure variables will be sought by Classification and Regression Tree (CART) and by exploratory threshold analyses.

RESTRICTED

CART is a recursive partitioning algorithm employed to identify a potential categorical classification of each dalbavancin exposure variable for the prediction of an outcome. For the CART analysis, node splitting will be based on the goodness of split statistic and optimal tree selection will be performed based on pruning and 10-fold cross-validation. In the CART models, node sizes will be set as follows: parent and terminal node sizes set to 50, depth set to 3, and choice of the maximum number of nodes will be automated. For interpretability of final modeling results, categorization is only considered for CART models with at most 4 terminal nodes. Exposure-outcome pairs where the exposure variable is split into 5 or more bins will not have this type of categorization considered. Subsequent exposure-outcome analysis will be presented as contrasts between exposure group pairs.

Additionally, exploratory threshold analyses will be performed to identify dalbavancin exposure threshold values associated with an increased risk of each outcome. For the exploratory threshold analyses, a series of equally spaced threshold values for each dalbavancin exposure will be divided patients into 2 groups. For each threshold (x-axis), the following are plotted: relative risk (RR) of outcome (high compared to low group), endpoints of the corresponding 95% confidence interval, and the Akaike Information Criterion (AIC; measure of the fit of a statistical model; smaller value indicates better fit). The optimal threshold is associated with the narrowest confidence interval (most precise estimate), the highest estimated relative risk, and the lowest AIC value. Subsequent exposure-outcome analysis will utilize this threshold as a dichotomization of the exposure variable.

4.5.2. Covariate Selection

Bivariate associations between baseline covariates and each outcome will also be performed to identify potential confounding variables for the multivariate analyses. For continuous covariates, a Kruskal-Wallis or Mann-Whitney test will provide a measure of association with the categorical covariates, while for categorical covariates, a Fisher's Exact test will be used. Baseline covariates associated with outcomes at a P-value < 0.1 will be considered as potential confounders in the multivariate analyses. Collinear covariates will be removed as necessary, while retaining strain. These bivariate associations are described in Table 13.

4.5.3. Description of Models

The relationship between each dalbavancin exposure variable of interest and each outcome will be quantified using multivariate regression analyses, adjusting for a subgrouping by *S. aureus* strain (MRSA vs. MSSA). For each exposure-outcome analysis, the outcome will be regressed upon using the continuous exposure variable, the CART-categorized exposure, and categorical exposure variable derived from threshold analysis. Covariates will be selected based on the previously described bivariate analyses.

4.5.3.1. DOOR

The associations between exposures and DOOR will be quantified using ordinal logistic regression. In contrast to the primary analysis, Quality of Life (QoL) will not be used as a tiebreaker in the exposure-response analysis, instead using only the 5-point DOOR as the only outcome. Initially, a proportional odds model will be applied, but in the case where the proportional odds assumption is severely violated (Score test $p<0.01$), a partial proportional odds model will be considered, where the proportional odds assumption is relaxed for the intercept and all covariates. Odds ratios (OR) and confidence intervals for the exposure variable will be presented from the regression models.

In addition, for categorical exposures only, predicted DOOR distributions for each exposure level will be generated for mean values of covariates (for categorical covariates including *S. aureus* strain, use proportion from corresponding columns of the design matrix), which in turn will be used to present DOOR probabilities

and their confidence intervals in comparing two adjacent exposure groups, using the methods described in Halperin et al 1989 [14]. DOOR probability between groups 1 and 2, denoted by π_{12}, the probability that a randomly selected subject from group 1 has a better DOOR than a randomly selected subject from group 2, can be estimated using Equation 6, where p_{ik} is the estimated proportion in group i=1 or 2 of DOOR=k (k=1-5) and $q_{ik} = 1 - p_{ik}$.

(6) $\hat{\pi}_{12} = \sum_{k=1}^{4} p_{1k}\left(\sum_{j=k+1}^{5} p_{2j}\right) + \frac{1}{2}\sum_{k=1}^{5} p_{1k}p_{2k}$

The 95% confidence interval for each DOOR probability is denoted by the limits presented in Equation 7, where n_1 and n_2 are the number of subjects included in the model from groups 1 and 2, respectively.

(7) $\frac{1}{2(C+1)}\left(C + 2\widehat{\pi_{12}} \pm \sqrt{C^2 + 4C\widehat{\pi_{12}}(1-\widehat{\pi_{12}})}\right)$ where

(8) $C = \frac{1}{n_1 n_2}{\chi^2}_{(1),0.95}[(n_1 + n_2 - 1) - (n_1 + n_2 - 2)\hat{\theta}]$, and

(9) $\hat{\theta} = \begin{cases} 0, & \theta_0 < 0 \\ \theta_0, & 0 \le \theta_0 \le 1 \\ 1, & \theta_0 > 1 \end{cases}$ where $\theta_0 = \frac{(n_1+n_2-2)\hat{\pi}_{12} - (n_2-1)A - (n_1-1)B}{(n_1+n_2-2)D}$

A, B, and D are derived in equations (10), (11), and (12), respectively.

(10) $A = \sum_{k=1}^{4} p_{1k}\left(\frac{p_{2k}}{2} + \sum_{j=k+1}^{5} p_{2j}\right)^2 + \frac{p_{15}{p_{25}}^2}{4} - \frac{1}{n_2-1}\sum_{k=1}^{4} p_{1k}\left[q_{2k}\sum_{j=k+1}^{5} p_{2j} - \left(\sum_{j=k+1}^{5} p_{2j}\right)^2\right] - \frac{1}{4(n_2-1)}\sum_{k=1}^{5} p_{1k}p_{2k}q_{2k}$

(11) $B = \sum_{k=2}^{5} p_{2k}\left(\frac{p_{1k}}{2} + \sum_{j=1}^{k-1} p_{1j}\right)^2 + \frac{p_{21}{p_{11}}^2}{4} - \frac{1}{n_1-1}\sum_{k=2}^{5} p_{2k}\left[q_{1k}\sum_{j=1}^{k-1} p_{1j} - \left(\sum_{j=1}^{k-1} p_{1j}\right)^2\right] - \frac{1}{4(n_1-1)}\sum_{k=1}^{5} p_{2k}p_{1k}q_{1k}$

(12) $D = \frac{(n_1n_2 - n_1 - n_2)\widehat{\pi_{12}} - n_1n_2{\widehat{\pi_{12}}}^2}{(n_1-1)(n_2-1)} + \frac{A}{n_1-1} + \frac{B}{n_2-1}$

Results from these associations adjusting for strain are presented in Table 14, and from subgroup analyses of MSSA and MRSA in Table 15 and Table 16, respectively.

Pseudocode:

Proportional Odds Model (for multi-category exposure in strain-adjusted model)

```
proc logistic data=door order=internal outmodel=pout;
     class expos2(param=ref ref='Low');
     model door(descending) = expos2 strainMRSA sexM weight;
     contrast 'exposure Low v Mid' expos2   1   /e estimate=parm;
     contrast 'exposure Mid v High' expos2 -1 1  /e estimate=parm;
run;
```

Partial Proportional Odds Model (for binary exposure in strain-adjusted model)

```
proc logistic data=door order=internal outmodel=pout;
     class expos(param=ref ref='Low');
     model door(descending) = expos strainMRSA sexM weight / unequalslopes= (sexM 
weight);
run;
```

Obtaining predicted DOOR distributions by exposure class

```
proc sql;
     create table preddat as
     select expos, mean(strainMRSA) as strainMRSA, mean(sexM) as sexM,
          mean(weight) as weight
     from door
     group by expos;
quit;

proc logistic inmodel=pout;
     score clm data=preddat out=predprob;
run;
```

4.5.3.2. Clinical Efficacy and Late Recurrence in Osteomyelitis Subset

For all binary efficacy outcomes, log-binomial regression will be used to quantify the association with each dalbavancin exposure (as both continuous and categorical) after adjusting for covariates. Associations between each exposure variable and each outcome will be assessed on both the relative (i.e., relative risk) and absolute scale (i.e., risk differences). Note that log-binomial is preferred over logistic regression because the log-binomial model estimates RR while logistic model estimates ORs (not a good approximation to RR when event rates are not rare). Results from the strain-adjusted model will be presented in Table 17, and from subgroup analyses of MSSA and MRSA in Table 18 and Table 19, respectively.

Pseudocode:

```
proc genmod data=indat;
     model BinaryOutcome = expos <covariates> / dist=binomial link=log;
     estimate ‘beta’ expos 1/exp;
     ods output estimates=RR;
run;
```

4.5.3.3. Elevated AST/ALT

The lone safety endpoint in the exposure-outcome analysis, AST/ALT levels over three times the upper limit of normal will be assessed through Day 42. With early exposures (C_{max}, $AUC_{0\text{-}1day}$) as predictors, this outcome will be modeled using a time-to-event approach. A Cox proportional hazards model will be used to estimate an exposure hazard ratio adjusted for covariates, along with Wald confidence intervals and p-values from the score test. The proportional hazards assumption will be evaluated using Kaplan-Meier plots. Results will be presented from the strain-adjusted model in Table 20 and from subgroup analyses of MSSA and MRSA in Table 21 and Table 22, respectively.

Pseudocode:

Cox Proportional Hazards Model (for 3-level categorical exposure)

```
proc phreg data=indat;
     class expos(ref='Low');
     model timevariable * censorvariable(1) = expos <covariates> ties=efron;
     hazardratio expos / diff = ref cl=wald;
     contrast 'exposure Low v Mid' expos   1    /e estimate=exp;
     contrast 'exposure Mid v High' expos -1 1  /e estimate=exp;
run;
```

4.6. Reporting Conventions

P-values ≥0.001 and ≤0.999 will be reported to 3 decimal places; p-values less than 0.001 will be reported as “<0.001” The mean, standard deviation, and other statistics will be reported to 1 decimal place greater than the original data. The minimum and maximum will use the same number of decimal places as the original data. Proportions will be presented as 2 decimal places; values greater than zero but <0.01 will be presented as “<0.01”. Percentages will be reported to the nearest whole number; values greater than zero but < 1% will be presented as “<1”; values greater than 99% but less than 100% will be reported as >99%. Estimated parameters, not on the same scale as raw observations (e.g., regression coefficients) will be reported to 3 significant figures.

For PK, AUCs will be reported as whole numbers (or using 3 significant digits if less than 100). $T_{1/2}$, T_{max}, CL, and V_d (V_{ss}) values will be reported to one decimal place (or to 2 significant digits if less than 1). Λ_z values will be reported to 3 significant digits. C_{max} will be reported with the same number of significant digits as the measurement.

Listings of individual subject data include a Subject ID column. The subject identifiers assigned by site staff are replaced throughout this report with the SDTM variable USUBJID to protect the confidentiality of those who volunteered to participate in this protocol. USUBJID has been created as a composite of the 3-letter EDC platform code followed by a numeric identifier assigned chronologically to enrolled subjects as well as screening failures across all sites and protocols in the EDC platform. Any data sharing activities will include the USUBJID and not the subject identifiers assigned at the site.

4.7. Additional Analysis

Additional analysis will be performed as needed. If exposures associated with outcomes are identified in the exposure-response analyses, 5,000 subject Monte Carlo simulations will be performed to determine the ability of the studied dalbavancin regimens to achieve the identified critical exposures.

5. REFERENCES

1. Gehan EA, George SL. Estimation of human body surface area from height and weight. Cancer Chemotherapy Reports. August 1970; 54(4):225-35. PMID: 5527019.
2. Acute Respiratory Distress Syndrome Network: Ventilation with lower tidal volumes as compared with traditional tidal volumes for acute lung injury and the respiratory distress syndrome. N Engl J Med 2000; 342:1301-8. PMID: 10793162.
3. Cockcroft DW, Gault MH. Prediction of creatinine clearance from serum creatinine, Nephron. 1976. 16(1): 31-41.
4. Food and Drug Administration. Guidance for Industry: Population Pharmacokinetics, February 2022.
5. Beal, S.L. Ways to Fit a PK Model with Some Data Below the Quantification Limit Journal of Pharmacokinetics and Pharmacodynamics, Vol. 28, No. 5, October 2001.
6. Carrothers TJ, Chittenden JT, Critchley I. Dalbavancin Population Pharmacokinetic Modeling and Target Attainment Analysis. Clin Pharmacol Drug Dev. Jan 2020; 9(1):21-31. PMID: 31087630.
7. Ribbing, J., Niclas Jonsson, E. Power, Selection Bias and Predictive Performance of the Population Pharmacokinetic Covariate Model. J Pharmacokinet Pharmacodyn 31, 109–134 (2004). https://doi.org/10.1023/B:JOPA.0000034404.86036.72. PMID: 15379381.
8. Bergstrand M, Hooker AC, Wallin JE, Karlsson MO. Prediction-corrected visual predictive checks for diagnosing nonlinear mixed-effects models. AAPS J. 2011;13(2):143-151. PMID: 21302010.
9. Food and Drug Administration. Guidance for Industry: Exposure-Response Relationships – Study Design, Data Analysis, and Regulatory Applications, April 2003.
10. Johansson, A.M. and Karlsson, M.O. Comparison of Methods for Handling Missing Covariate Data. The AAPS Journal, Vol. 15, No. 4, October 2013.
11. Smith, Brian P., et al. Confidence interval criteria for assessment of dose proportionality. Pharmaceutical Research 17.10 (2000): 1278-1283.
12. Mager DE, Krzyzanski W. Quasi-equilibrium pharmacokinetic model for drugs exhibiting target-mediated drug disposition. Pharm Res. 2005; 22(10):1589-1596.
13. Chiou WL. The phenomenon and rationale of marked dependence of drug concentration on blood sampling site: implications in pharmacokinetics, pharmacodynamics, toxicology and therapeutics (Part I). Clin Pharmacokinet 1983. 17(3): 175-199.
14. Halperin M, Hamdy MI, Thall PF. Distribution-Free Confidence Intervals for a Parameter of Wilcoxon-Mann-Whitney Type for Ordered Categories and Progressive Censoring. Biometrics 1989. 45(2): 509-521. PMID: 2765635.

APPENDICES

APPENDIX 1. TABLES MOCKUPS

LIST OF TABLES

Table 1: Covariates Evaluated in PopPK Modeling 11

Table 2: Summary of Categorical Demographic and Baseline Characteristics by Dalbavancin Dosage Group 31

Table 3: Summary of Continuous Demographic and Baseline Characteristics by Dalbavancin Dosage Group 32

Table 4: Summary Statistics of Total and Unbound Plasma Concentration (µg/mL) by Dalbavancin Dosage Group and Nominal Time 33

Table 5: Descriptive Statistics of PK Parameters from Observed Total and Unbound Plasma Concentration – Time Data by Dalbavancin Dosage Group 34

Table 6: Summary of Pop-PK Analysis Models Considered – PK Population 36

Table 7: Estimation Summary of Plasma Pop-PK Parameters and Exposures Estimated Using the Final Model – PK Population 37

Table 8: Summary of the Forward Selection Process of Covariates for the Pop-PK Model 38

Table 9: Summary of the Backward Elimination Process for Covariates from the Pop-PK Model 39

Table 10: Summary Statistics of PK Parameters from Pop-PK Model by Dalbavancin Dosage Group – PK Population 40

Table 11: Summary of Exposure Endpoints by Categories of Efficacy Outcome 41

Table 12: Summary of Exposure Endpoints by Categories of Safety Outcome 42

Table 13: Bivariate Associations of Outcomes by Covariates of Interest 43

Table 14: Adjusted Odds Ratios and DOOR Probabilities for Various Levels of PopPK-Model-Estimated Exposure using Proportional or Partial Proportional Odds Model in Ordinal Regression on DOOR – *S. Aureus* Strain-Adjusted Model 44

Table 15: Adjusted Odds Ratios and DOOR Probabilities for Various Levels of PopPK-Model-Estimated Exposure using Proportional or Partial Proportional Odds Model in Ordinal Regression on DOOR – MSSA Subgroup Analysis 45

Table 16: Adjusted Odds Ratios and DOOR Probabilities for Various Levels of PopPK-Model-Estimated Exposure using Proportional or Partial Proportional Odds Model in Ordinal Regression on DOOR – MRSA Subgroup Analysis 45

Table 17: Adjusted Relative Risks of Outcome Improvement for Various Levels of PopPK-Model-Estimated Exposure using Generalized Linear Models for Binary Outcomes – *S. aureus* Strain-Adjusted Model 46

Table 18: Adjusted Relative Risks of Outcome Improvement for Various Levels of PopPK-Model-Estimated Exposure using Generalized Linear Models for Binary Outcomes – MSSA Subgroup Analysis 46

Table 19: Adjusted Relative Risks of Outcome Improvement for Various Levels of PopPK-Model-Estimated Exposure using Generalized Linear Models for Binary Outcomes – MSSA Subgroup Analysis 46

Table 20: Adjusted Hazard Ratios of AST/ALT > 3x ULN for Various Levels of PopPK-Model-Estimated Early Exposure using a Cox Proportional Hazards Model – *S. aureus* Strain-Adjusted Model 47

Table 21: Adjusted Hazard Ratios of AST/ALT > 3x ULN for Various Levels of PopPK-Model-Estimated Early Exposure using a Cox Proportional Hazards Model – MSSA Subgroup Analysis 47

Table 22: Adjusted Hazard Ratios of AST/ALT > 3x ULN for Various Levels of PopPK-Model-Estimated Early Exposure using a Cox Proportional Hazards Model – MRSA Subgroup Analysis 47

Table 2: Summary of Categorical Demographic and Baseline Characteristics by Dalbavancin Dosage Group

Variable	Characteristic	Dalbavancin 1500 mg + 1500 mg	Dalbavancin 1500 mg + 1125 mg	Dalbavancin 1125 mg + 1500 mg	Dalbavancin 1125 mg + 1125 mg	Dalbavancin 1500 mg only	Dalbavancin 1125 mg only	All Dalbavancin Subjects
		(N=X)	(N=X)	(N=X)	(N=X)	(N=X)	(N=X)	(N=X)
		n (%)	n (%)	n (%)	n (%)	n (%)	n (%)	n (%)
Sex	Male	x (xx.x)	x (xx.x)	x (xx.x)	x (xx.x)	x (xx.x)	x (xx.x)	x (xx.x)
	Female	x (xx.x)	x (xx.x)	x (xx.x)	x (xx.x)	x (xx.x)	x (xx.x)	x (xx.x)
Ethnicity	Not Hispanic or Latino	x (xx.x)	x (xx.x)	x (xx.x)	x (xx.x)	x (xx.x)	x (xx.x)	x (xx.x)
	Hispanic or Latino	x (xx.x)	x (xx.x)	x (xx.x)	x (xx.x)	x (xx.x)	x (xx.x)	x (xx.x)
	Not Reported	x (xx.x)	x (xx.x)	x (xx.x)	x (xx.x)	x (xx.x)	x (xx.x)	x (xx.x)
	Unknown	x (xx.x)	x (xx.x)	x (xx.x)	x (xx.x)	x (xx.x)	x (xx.x)	x (xx.x)
Race	American Indian or Alaska Native	x (xx.x)	x (xx.x)	x (xx.x)	x (xx.x)	x (xx.x)	x (xx.x)	x (xx.x)
	Asian	x (xx.x)	x (xx.x)	x (xx.x)	x (xx.x)	x (xx.x)	x (xx.x)	x (xx.x)
	Native Hawaiian or Other Pacific Islander	x (xx.x)	x (xx.x)	x (xx.x)	x (xx.x)	x (xx.x)	x (xx.x)	x (xx.x)
	Black or African American	x (xx.x)	x (xx.x)	x (xx.x)	x (xx.x)	x (xx.x)	x (xx.x)	x (xx.x)
	White	x (xx.x)	x (xx.x)	x (xx.x)	x (xx.x)	x (xx.x)	x (xx.x)	x (xx.x)
	Multi-Racial	x (xx.x)	x (xx.x)	x (xx.x)	x (xx.x)	x (xx.x)	x (xx.x)	x (xx.x)
	Unknown	x (xx.x)	x (xx.x)	x (xx.x)	x (xx.x)	x (xx.x)	x (xx.x)	x (xx.x)

N = Number of subjects in dalbavancin dose groups.
N = Number of subjects with non-missing values for the corresponding baseline characteristics.

RESTRICTED

Table 3: Summary of Continuous Demographic and Baseline Characteristics by Dalbavancin Dosage Group

Variable	Statistic	Dalbavancin 1500 mg + 1500 mg (N=X)	Dalbavancin 1500 mg + 1125 mg (N=X)	Dalbavancin 1125 mg + 1500 mg (N=X)	Dalbavancin 1125 mg + 1125 mg (N=X)	Dalbavancin 1500 mg only (N=X)	Dalbavancin 1125 mg only (N=X)	All Dalbavancin Subjects (N=X)
Age (years)	n	xx	xx	xx	xx	xx	xx	xx
	Mean	xx	xx	xx	xx	xx	xx	xx
	Standard Deviation	xx	xx	xx	xx	xx	xx	xx
	Median	xx	xx	xx	xx	xx	xx	xx
	Minimum	x	x	x	x	x	x	x
	Maximum	x	x	x	x	x	x	x
BMI (kg/m^2)	n	xx.xxx	xx.xxx	xx.xxx	xx.xxx	xx.xxx	xx.xxx	xx.xxx
	Mean	xx.xxx	xx.xxx	xx.xxx	xx.xxx	xx.xxx	xx.xxx	xx.xxx
	Standard Deviation	xx.xxx	xx.xxx	xx.xxx	xx.xxx	xx.xxx	xx.xxx	xx.xxx
	Median	xx.xxx	xx.xxx	xx.xxx	xx.xxx	xx.xxx	xx.xxx	xx.xxx
	Minimum	x.xx	x.xx	x.xx	x.xx	x.xx	x.xx	x.xx
	Maximum	x.xx	x.xx	x.xx	x.xx	x.xx	x.xx	x.xx
Height (cm)	n	x.xx	x.xx	x.xx	x.xx	x.xx	x.xx	x.xx
	Mean	x.xx	x.xx	x.xx	x.xx	x.xx	x.xx	x.xx
	Standard Deviation	x.xx	x.xx	x.xx	x.xx	x.xx	x.xx	x.xx
	Median	x.xx	x.xx	x.xx	x.xx	x.xx	x.xx	x.xx
	Minimum	x.x	x.x	x.x	x.x	x.x	x.x	x.x
	Maximum	x.x	x.x	x.x	x.x	x.x	x.x	x.x
Weight (kg)	n	x.xx	x.xx	x.xx	x.xx	x.xx	x.xx	x.xx
	Mean	x.xx	x.xx	x.xx	x.xx	x.xx	x.xx	x.xx
	Standard Deviation	x.xx	x.xx	x.xx	x.xx	x.xx	x.xx	x.xx
	Median	x.xx	x.xx	x.xx	x.xx	x.xx	x.xx	x.xx
	Minimum	x.x	x.x	x.x	x.x	x.x	x.x	x.x
	Maximum	x.x	x.x	x.x	x.x	x.x	x.x	x.x

N = Number of subjects in dalbavancin dose groups.
n = Number of subjects with non-missing values for the corresponding baseline characteristics.

Table 4: Summary Statistics of Total and Unbound Plasma Concentration (µg/mL) by Dalbavancin Dosage Group and Nominal Time

	Dalbavancin Plasma Concentration (µg/mL) Mean (SD) [n]						
	Dalbavancin 1500 mg + 1500 mg	Dalbavancin 1500 mg + 1125 mg	Dalbavancin 1125 mg + 1500 mg	Dalbavancin 1125 mg + 1125 mg	Dalbavancin 1500 mg only	Dalbavancin 1125 mg only	All Dalbavancin Subjects
Nominal Time[1]	(N=X)	(N=X)	(N=X)	(N=X)	(N=X)	(N=X)	(N=X)
**Total Plasma Concentrations**							
Day 1 – Pre 1st IV Admin							
n	xx	xx	xx	xx	xx	xx	xx
Mean	xx.xx	xx.xx	xx.xx	xx.xx	xx.xx	xx.xx	xx.xx
SD	xx.xx	xx.xx	xx.xx	xx.xx	xx.xx	xx.xx	xx.xx
Min	xx.x	xx.x	xx.x	xx.x	xx.x	xx.x	xx.x
Median	xx.x	xx.x	xx.x	xx.x	xx.x	xx.x	xx.x
Max	xx.x	xx.x	xx.x	xx.x	xx.x	xx.x	xx.x
Day 1 – End of 1st Infusion							
Day 1 – 6 h							
Day 1 – 12 h							
Day 2 – 24 h							
Day 8 – Pre 2nd IV Admin							
Day 22							
Day 42							
Day 70							
**Unbound Plasma Concentrations**							
Day 1 – Pre 1st IV Admin							
…							

Notes: N=Number of subjects in dalbavancin dose groups.
n= Number of subjects with non-missing results at each timepoint.
[1]Times are relative to the end of the first infusion.

Table 5: Descriptive Statistics of PK Parameters from Observed Total and Unbound Plasma Concentration – Time Data by Dalbavancin Dosage Group

	PK Parameters						
	**Dalbavancin 500 mg + 1500 mg**	**Dalbavancin 1500 mg + 1125 mg**	**Dalbavancin 1125 mg + 1500 mg**	**Dalbavancin 1125 mg + 1125 mg**	**Dalbavancin 1500 mg only**	**Dalbavancin 1125 mg only**	**All Dalbavancin Subjects**
**PK Parameter (units)**	**(N=X)**	**(N=X)**	**(N=X)**	**(N=X)**	**(N=X)**	**(N=X)**	**(N=X)**
**C_{max}, total (µg/mL)**							
n	xx	xx	xx	xx	xx	xx	xx
Mean	xx.xx	xx.xx	xx.xx	xx.xx	xx.xx	xx.xx	xx.xx
SD	xx.xx	xx.xx	xx.xx	xx.xx	xx.xx	xx.xx	xx.xx
Min	xx.x	xx.x	xx.x	xx.x	xx.x	xx.x	xx.x
Max	xx.x	xx.x	xx.x	xx.x	xx.x	xx.x	xx.x
GM	xx.x	xx.x	xx.x	xx.x	xx.x	xx.x	xx.x
GSD	xx.x	xx.x	xx.x	xx.x	xx.x	xx.x	xx.x
CV%	xx.x	xx.x	xx.x	xx.x	xx.x	xx.x	xx.x
**C_{max}, unbound(µg/mL)**							
**C_{8day}, total(µg/mL)**							
**C_{8day}, unbound(µg/mL)**							
**C_{22day}, total(µg/mL)**							
**C_{22day}, unbound (µg/mL)**							
**C_{42day}, total (µg/mL)**							
**C_{42day}, unbound (µg/mL)**							
**C_{70day}, total (µg/mL)**							
**C_{70day}, unbound (µg/mL)**							
**T_{max}, total (h)**							
**T_{max}, unbound (h)**							
**λ_z, total (1/h)**							
**λ_z, unbound (1/h)**							
**$T_{1/2}$, total (h)**							
**$T_{1/2}$, unbound (h)**							
**AUC_{0-1day}, total (µg*h/mL)**							
**AUC_{0-1day}, unbound (µg*h/mL)**							

TABLE 5: Descriptive Statistics of PK Parameters from Observed Total and Unbound Plasma Concentration - Time Data by Dalbavancin Dosage Group *(continued)*

	PK Parameters						
	**Dalbavancin 500 mg + 1500 mg**	**Dalbavancin 1500 mg + 1125 mg**	**Dalbavancin 1125 mg + 1500 mg**	**Dalbavancin 1125 mg + 1125 mg**	**Dalbavancin 1500 mg only**	**Dalbavancin 1125 mg only**	**All Dalbavancin Subjects**
**PK Parameter (units)**	**(N=X)**	**(N=X)**	**(N=X)**	**(N=X)**	**(N=X)**	**(N=X)**	**(N=X)**
**$AUC_{0\text{-}8days}$, total(µg*h/mL)**							
**$AUC_{0\text{-}8days}$, unbound (µg*h/mL)**							
**$AUC_{0\text{-}22days}$, total (µg*h/mL)**							
**$AUC_{0\text{-}22days}$, unbound (µg*h/mL)**							
**$AUC_{0\text{-}42days}$, total (µg*h/mL)**							
**$AUC_{0\text{-}42days}$, unbound (µg*h/mL)**							
**$AUC_{0\text{-}70days}$, total (µg*h/mL)**							
**$AUC_{0\text{-}70days}$, unbound (µg*h/mL)**							
**$AUC_{8\text{-}22days}$, total (µg*h/mL)**							
**$AUC_{8\text{-}22days}$, unbound (µg*h/mL)**							
**$AUC_{15\text{-}22days}$, total (µg*h/mL)**							
**$AUC_{15\text{-}22days}$, unbound (µg*h/mL)**							
**$AUC_{22\text{-}42days}$, total (µg*h/mL)**							
**$AUC_{22\text{-}42days}$, unbound (µg*h/mL)**							
**$AUC_{42\text{-}70days}$, total (µg*h/mL)**							
**$AUC_{42\text{-}70days}$, unbound (µg*h/mL)**							
**$AUC_{0\text{-}last}$, total (µg*h/mL)**							
**$AUC_{0\text{-}last}$, unbound (µg*h/mL)**							
**$AUC_{0\text{-}\infty}$, total (µg*h/mL)**							
**$AUC_{0\text{-}\infty}$, unbound (µg*h/mL)**							
**f_{u1day}**							
**f_{u8day}**							
**f_{u22day}**							
**f_{u42day}**							
**f_{u70day}**							

Notes: N=Number of subjects in dalbavancin dose groups.
n=Number of subjects with results meeting analysis criteria contributing to summary statistics.
Values of GM (CV%) [n] are shown for all parameters except for T_{max} for which values of median (min – max) are shown.

Table 6: Summary of Pop-PK Analysis Models Considered – PK Population

[Implementation Note: Bold the text in the row describing the final model.]

Model	Lineage	Description	Objective Function (-2LL)	# Parameters	p-value
xxx	xxx	One-compartment linear model. Parameters CL, V	xxxx.xx	xx	x.xxx
		Two-compartment linear mammillary model. Parameters: CL, V1, Q, V2			
xxx	xxx	Two-compartment linear mammillary model with first-order absorption. Parameters: CL, V2, Q, V3, KA	xxxx.xx	xx	x.xxx
xxx	xxx	Three-compartment linear mammillary model with first-order absorption. Parameters: CL, V2, Q3, V3, Q4, V4, KA	xxxx.xx	xx	x.xxx
xxx	xxx	Xxxx xxxxx xxxx	xxxx.xx	xx	x.xxx
xxx	xxx	Xxxx xxxxx xxxx	xxxx.xx	xx	x.xxx

Table 7: Estimation Summary of Plasma Pop-PK Parameters and Exposures Estimated Using the Final Model – PK Population

[Implementation note: BSV and shrinkage only provided for parameters with IIV specified in the model. Also, make sure to include all total and unbound exposures.]

Parameter[1]	Units	Estimate	Bootstrap Median	Bootstrap Relative Standard Error (%)	Bootstrap 95% CI	BSV (CV%)	Shrinkage
CL	xx	xxx.xx	xxx.xx	xxx.xx	(xx.xx, xx.xx)	xx.xx	xx.xx
V_C	xx	xxx.xx	xxx.xx	xxx.xx	(xx.xx, xx.xx)	xx.xx	xx.xx
Q1	xx	xxx.xx	xxx.xx	xxx.xx	(xx.xx, xx.xx)	xx.xx	xx.xx
Vp_1	xx	xxx.xx	xxx.xx	xxx.xx	(xx.xx, xx.xx)	xx.xx	xx.xx
Q2	xx	xxx.xx	xxx.xx	xxx.xx	(xx.xx, xx.xx)	xx.xx	xx.xx
Vp_2	xx	xxx.xx	xxx.xx	xxx.xx	(xx.xx, xx.xx)	xx.xx	xx.xx
KA	xx	xxx.xx	xxx.xx	xxx.xx	(xx.xx, xx.xx)	xx.xx	xx.xx
f_u							
σ^2_1	xx	xxx.xx	xxx.xx	xxx.xx	(xx.xx, xx.xx)	xx.xx	xx.xx
σ^2_2	xx	xxx.xx	xxx.xx	xxx.xx	(xx.xx, xx.xx)	xx.xx	xx.xx
C_{max} (total)							
C_{max} (unbound)							
…							
…							

[1]Parameters will be specific to the final model.
BSV = Between Subjects Variability.

RESTRICTED

Table 8: Summary of the Forward Selection Process of Covariates for the Pop-PK Model

Step	Parameter	Covariate Added	Functional Form	Objective Function (-2LL)	Change in Objective Function	df	P-value
0	Base structural model			xxxxx.xx	-		
1	Central Volume	Age	Power	xxxxx.xx	xxxxx.xx	xx	x.xxx
2	Central Volume	Sex	Proportional	xxxxx.xx	xxxxx.xx	xx	x.xxx
3	Clearance	Sex	Proportional	xxxxx.xx	xxxxx.xx	xx	x.xxx
4	Clearance	BMI	Power	xxxxx.xx	xxxxx.xx	xx	x.xxx
5	Clearance	CrCl	Power	xxxxx.xx	xxxxx.xx	xx	x.xxx
6	Clearance	Albumin	Power	xxxxx.xx	xxxxx.xx	xx	x.xxx
7	Peripheral Volume	Sex	Proportional	xxxxx.xx	xxxxx.xx	xx	x.xxx
x							
x							

Table 9: Summary of the Backward Elimination Process for Covariates from the Pop-PK Model

Step	Parameter	Covariate Dropped	Functional Form	Objective Function (-2LL)	Change in Objective Function	df	P-value
0	Base structural model			xxxxx.xx	-		
1	Central Volume	Age	Power	xxxxx.xx	xxxxx.xx	xx	x.xxx
2	Clearance	BMI	Power	xxxxx.xx	xxxxx.xx	xx	x.xxx
3	Clearance	CrCl	Power	xxxxx.xx	xxxxx.xx	xx	x.xxx
x							
x							

Table 10: Summary Statistics of PK Parameters from Pop-PK Model by Dalbavancin Dosage Group – PK Population

Programming Note: This table will repeat Table 4 to display summaries of PK parameters derived from Pop-PK model.

Table 11: Summary of Exposure Endpoints by Categories of Efficacy Outcome

Programming Note: For efficacy endpoints, exclude subjects being excluded from exposure-outcome analysis, i.e., censored before Day 22.

			Total Plasma Concentration								Unbound Plasma Concentration							
			C_{22day}		$AUC_{0-22day}$		$AUC_{8-22day}$		$AUC_{15-22day}$		C_{22day}		$AUC_{0-22day}$		$AUC_{8-22day}$		$AUC_{15-22day}$	
**Efficacy Endpoint**	**Category**	**n**	**Mean**	**SD**	**Mean**	**SD**	**Mean**	**SD**	**Mean**	**SD**	**Mean**	**SD**	**Mean**	**SD**	**Mean**	**SD**	**Mean**	**SD**
DOOR at Day 42	Alive with no events	x	x.xx	x.xx	x.xx	x.xx	x.xx	x.xx	x.xx	x.xx	x.xx	x.xx	x.xx	x.xx	x.xx	x.xx	x.xx	x.xx
	Alive with 1 event																	
	Alive with 2 events																	
	Alive with 3 events																	
	Death																	
	Missing																	
DOOR at Day 70	Alive with no events																	
	Alive with 1 event																	
	Alive with 2 events																	
	Alive with 3 events																	
	Death																	
	Missing																	
Clinical Efficacy at Day 42	Yes																	
	No																	
Clinical Efficacy at Day 70	Yes																	
	No																	
Late recurrence at 6 months (within osteomyelitis subset)	Yes																	
	No																	

RESTRICTED

Table 12: Summary of Exposure Endpoints by Categories of Safety Outcome

Safety Endpoint	Category	n	Total Plasma Concentration				Unbound Plasma Concentration			
			C_{max}		AUC_{0-1day}		C_{max}		AUC_{0-1day}	
			Mean	SD	Mean	SD	Mean	SD	Mean	SD
AST/ALT elevation >3x upper limit of normal	Yes	x	x.xx	x.xx	x.xx	x.xx	x.xx	x.xx	x.xx	x.xx
	No									

Table 13: Bivariate Associations of Outcomes by Covariates of Interest

[Implementation note: Use covariates from the final PopPK model and other covariates of interest.]

Outcome variable	Covariate	P[a]
DOOR	BMI (kg/m^2)	x.xx
	Sex	

[a] P: p-value of bivariate association between covariate and exposure variable. For continuous covariates, p is from a test of a Mann-Whitney test for two-group outcomes or a Kruskal-Wallis test for multi-group outcomes. For categorical covariates, p is from Fisher's Exact Test.

Table 14: Adjusted Odds Ratios and DOOR Probabilities for Various Levels of PopPK-Model-Estimated Exposure using Proportional or Partial Proportional Odds Model in Ordinal Regression on DOOR – *S. Aureus* Strain-Adjusted Model

[Implementation note: provide actual breakpoints for exposure variable dichotomization in the “xxx” placeholders in the Exposure Variables names]

			**Odds Ratio***				**Estimated DOOR Proportions for Mean-Covariate Subject (%)**						**DOOR Probability**		
**Outcome Endpoint**	**Exposure Measure**	**Proportional Odds (Yes/No)**	**Exposure Contrast**	**Estimate**	**95% CI**	**P-value**	**Exposure Category**	**1**	**2**	**3**	**4**	**5**	**Exposure Contrast**	**Estimate (%)**	**95% CI**
**DOOR at Day 42**	Total C_{22day} (continuous)		Per µg/mL	x.xxx	x.xxx, x.xxx	x.xxx		--	--	--	--	--		--	--
	Total C_{22day} CART cutpoint: Low ≤ xxx<Medium≤ xxx<High		High vs. Low	x.xxx	x.xxx, x.xxx	x.xxx	Low	xx	xx	xx	xx	xx	High vs. Low	xx.x	xx.x, xx.x
			High vs. Medium				Medium	xx	xx	xx	xx	xx	High vs. Medium		
			Medium vs. Low				High	xx	xx	xx	xx	xx	Medium vs. Low		
	Total C_{22day} RR cutpoint: Low ≤xxx<High		High vs. Low				Low	xx	xx	xx	xx	xx	High vs. Low		
							High	xx	xx	xx	xx	xx			
	Total $AUC_{0\text{-}22day}$ (cont.)		Per µg*h/mL					--	--	--	--	--		--	--
	Total $AUC_{0\text{-}22day}$ CART cutpoint: Low ≤ xxx<Medium≤ xxx<High		High vs. Low												
			High vs. Medium												
			Medium vs. Low												
	Total $AUC_{0\text{-}22day}$ RR cutpoint: Low ≤xxx<High		High vs. Low												
	… [repeat for total $AUC_{8\text{-}22day}$ and $AUC_{15\text{-}22day}$ and all unbound exposure variables]														
**DOOR at Day 70**	[repeat analogous analyses from Day 42]	..	..	..	..	..		..	..	..	..	..		..	..

* For continuous exposure variables, Odds Ratio is quantified per unit increase of exposure. For dichotomized exposure variables, Odds Ratio is quantified for high values relative to low values of exposure measure in relation to breakpoint for improved outcome.

Tables with similar format:

Table 15: Adjusted Odds Ratios and DOOR Probabilities for Various Levels of PopPK-Model-Estimated Exposure using Proportional or Partial Proportional Odds Model in Ordinal Regression on DOOR – MSSA Subgroup Analysis

Table 16: Adjusted Odds Ratios and DOOR Probabilities for Various Levels of PopPK-Model-Estimated Exposure using Proportional or Partial Proportional Odds Model in Ordinal Regression on DOOR – MRSA Subgroup Analysis

RESTRICTED

Table 17: Adjusted Relative Risks of Outcome Improvement for Various Levels of PopPK-Model-Estimated Exposure using Generalized Linear Models for Binary Outcomes – *S. aureus* Strain-Adjusted Model

[Implementation note: provide actual breakpoints for exposure variable dichotomization in the "xxx" placeholders in the Exposure Variables names]

Outcome Endpoint	Exposure Measure	Exposure Contrast	Relative Risk*			Risk Difference*		
			Estimate	95% CI	P-value	Estimate	95% CI	P-value
Clinical Efficacy at Day 42	Total C_{22day} (continuous)	Per µg/mL	x.xxx	x.xxx, x.xxx	x.xxx	x.xxx	x.xxx, x.xxx	x.xxx
	Total C_{22day} CART cutpoint: Low ≤ xxx<Medium≤ xxx<High	High vs. Low						
		High vs. Medium						
		Medium vs. Low						
	Total C_{22day} RR cutpoint: Low ≤xxx<High	High vs. Low						
	Total $AUC_{0\text{-}22day}$ (cont.)	Per µg*h/mL						
	Total $AUC_{0\text{-}22day}$ CART cutpoint: Low ≤ xxx<Medium≤ xxx<High	High vs. Low						
		High vs. Medium						
		Medium vs. Low						
	Total $AUC_{0\text{-}22day}$ RR cutpoint: Low ≤xxx<High	High vs. Low						
	[repeat for total $AUC_{8\text{-}22day}$ and $AUC_{15\text{-}22day}$ and all unbound exposure variables] …							
[repeat for all efficacy endpoints]								

* For continuous exposure variables, Relative Risk and Risk Difference is quantified per unit increase of exposure. For dichotomized exposure variables, they are quantified for high values relative to low values of exposure measure in relation to breakpoint for improved outcome.

Tables with similar format:

Table 18: Adjusted Relative Risks of Outcome Improvement for Various Levels of PopPK-Model-Estimated Exposure using Generalized Linear Models for Binary Outcomes – MSSA Subgroup Analysis

Table 19: Adjusted Relative Risks of Outcome Improvement for Various Levels of PopPK-Model-Estimated Exposure using Generalized Linear Models for Binary Outcomes – MSSA Subgroup Analysis

Table 20: Adjusted Hazard Ratios of AST/ALT > 3x ULN for Various Levels of PopPK-Model-Estimated Early Exposure using a Cox Proportional Hazards Model – *S. aureus* Strain-Adjusted Model

[Implementation note: provide actual breakpoints for exposure variable dichotomization in the "xxx" placeholders in the Exposure Variables names]

			Hazard Ratio*		
**Outcome endpoint**	**Exposure Measure**	**Exposure category**	**Estimate**	**95% CI**	**P-value**
**AST/ALT elevation >3x upper limit of normal**	Total C_{max} (continuous)	Continuous (µg/mL)	x.xxx	x.xxx, x.xxx	x.xxx
	Total C_{max} CART cutpoint: Low ≤ xxx<Medium≤ xxx<High	High vs. Low			
		High vs. Medium			
		Medium vs. Low			
	Total C_{max} RR cutpoint: Low ≤xxx<High	High vs. Low			
	Total AUC_{0-1day} (cont.)	Continuous (µg*h/mL)			
	Total AUC_{0-1day} CART cutpoint: Low ≤ xxx<Medium≤ xxx<High	High vs. Low			
		High vs. Medium			
		Medium vs. Low			
	Total AUC_{0-1day} RR cutpoint: Low ≤xxx<High	High vs. Low			
	[repeat for all unbound exposure variables]	…			

Tables with similar format:

Table 21: Adjusted Hazard Ratios of AST/ALT > 3x ULN for Various Levels of PopPK-Model-Estimated Early Exposure using a Cox Proportional Hazards Model – MSSA Subgroup Analysis

Table 22: Adjusted Hazard Ratios of AST/ALT > 3x ULN for Various Levels of PopPK-Model-Estimated Early Exposure using a Cox Proportional Hazards Model – MRSA Subgroup Analysis

APPENDIX 2. FIGURES MOCKUPS

LIST OF FIGURES

Figure 1: Scatter Plot of Individual Subject Plasma Concentration by Dalbavancin Dosage Group and Time Point – PK Population 50

Figure 2: Semi-Log Plot of Plasma Linear Mean Concentration by Dalbavancin Dosage Group and Time Point – PK Population 51

Figure 3: Semi-Log Plots of Geometric Mean of Plasma Concentration by Dalbavancin Dosage Group and Time Point – PK Population....52

Figure 4: Semi-Log Plots of Geometric Mean of Plasma Concentration by Dalbavancin Dosage Group and Time Point – PK Population....53

Figure 5: Semi-Log Plots of Geometric Mean of Plasma Concentration by Dalbavancin Dosage Group and Time Point – PK Population....54

Figure 6: Fraction Unbound Concentration versus Time Since Last Dose in Linear (A) and Semi-Log (B) Scales 55

Figure 7: Fraction Unbound Concentration versus Total (A) and Unbound (B) Concentration....56

Figure 8: Final Model Diagnostics: Observed Concentrations versus Population Predicted Concentrations (ug/mL) of Total Dalbavancin....57

Figure 9: Final Model Diagnostics: Observed Concentrations versus Individual Predicted Concentrations (ug/mL) of Total Dalbavancin....58

Figure 10: Final Model Diagnostics: Quantile-Quantile Plot of Individual Weighted Residuals....59

Figure 11: Final Model Diagnostics: Weighted Residuals versus Time Since Last Dose (h)....60

Figure 12: Final Model Diagnostics: Weighted Residuals versus Time Since First Dose (h)....61

Figure 13: Final Model Diagnostics: Weighted Residuals versus Population Predicted Concentration (ug/mL) of Total Dalbavancin 62

Figure 14: Final Model Diagnostics: Individual Weighted Residuals versus Individual Predicted Concentration (ug/mL) of Total Dalbavancin 62

Figure 15: Final Model Diagnostics: Absolute Value of Individual Weighted Residuals versus Individual Predicted Concentration (ug/mL) of Total Dalbavancin....63

Figure 16: Final Model Diagnostics: Plots of Observed, Individual Predicted, and Population Predicted Concentrations (ug/mL) of Total Dalbavancin versus Time Since First Dose(h) – By Subject 64

Figure 17: Final Model Diagnostics: Prediction-Corrected Visual Predictive Check for Fit on Total Plasma Dalbavancin Concentration 65

Figure 18: Final Model Diagnostics: Prediction-Corrected Visual Predictive Check for Fit on Unbound Plasma Dalbavancin Concentration..65

Figure 19: Final PopPK Model-predicted Typical and Individual Empirical Bayes Estimates versus Covariates 66

Figure 1: Scatter Plot of Individual Subject Plasma Concentration by Dalbavancin Dosage Group and Time Point – PK Population

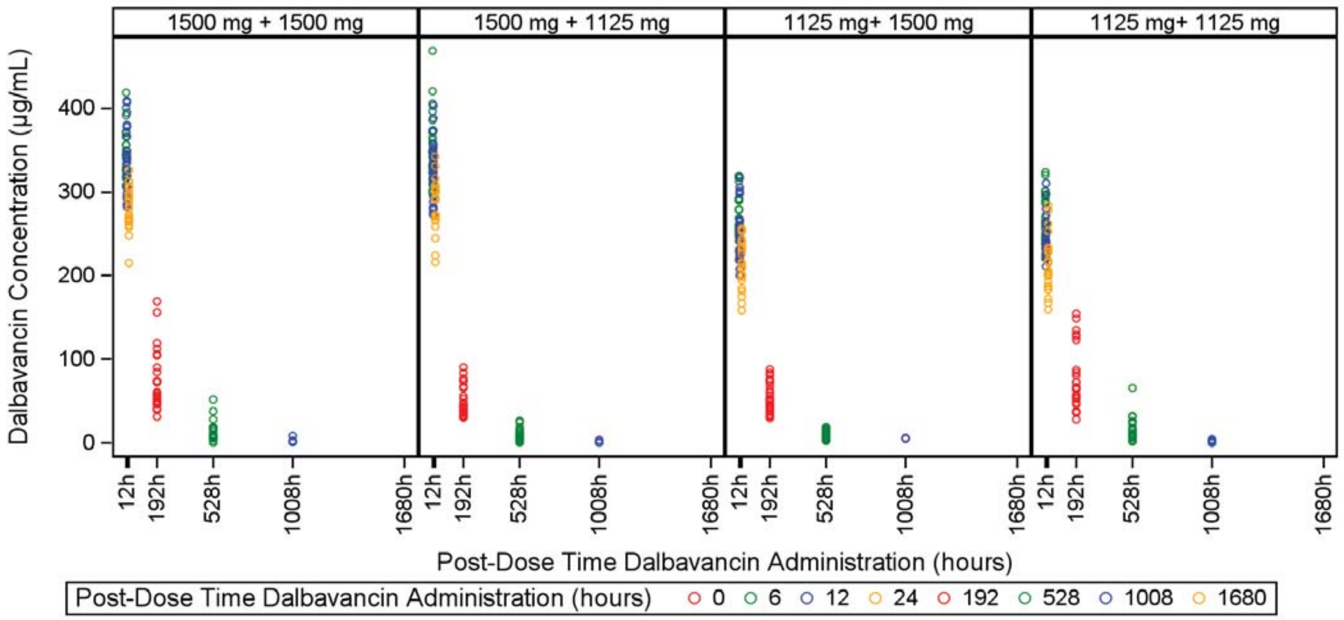

Programming Note: Add scatter plot for 1500 mg only and 1125 mg only.

Figure 2: Semi-Log Plot of Plasma Linear Mean Concentration by Dalbavancin Dosage Group and Time Point – PK Population

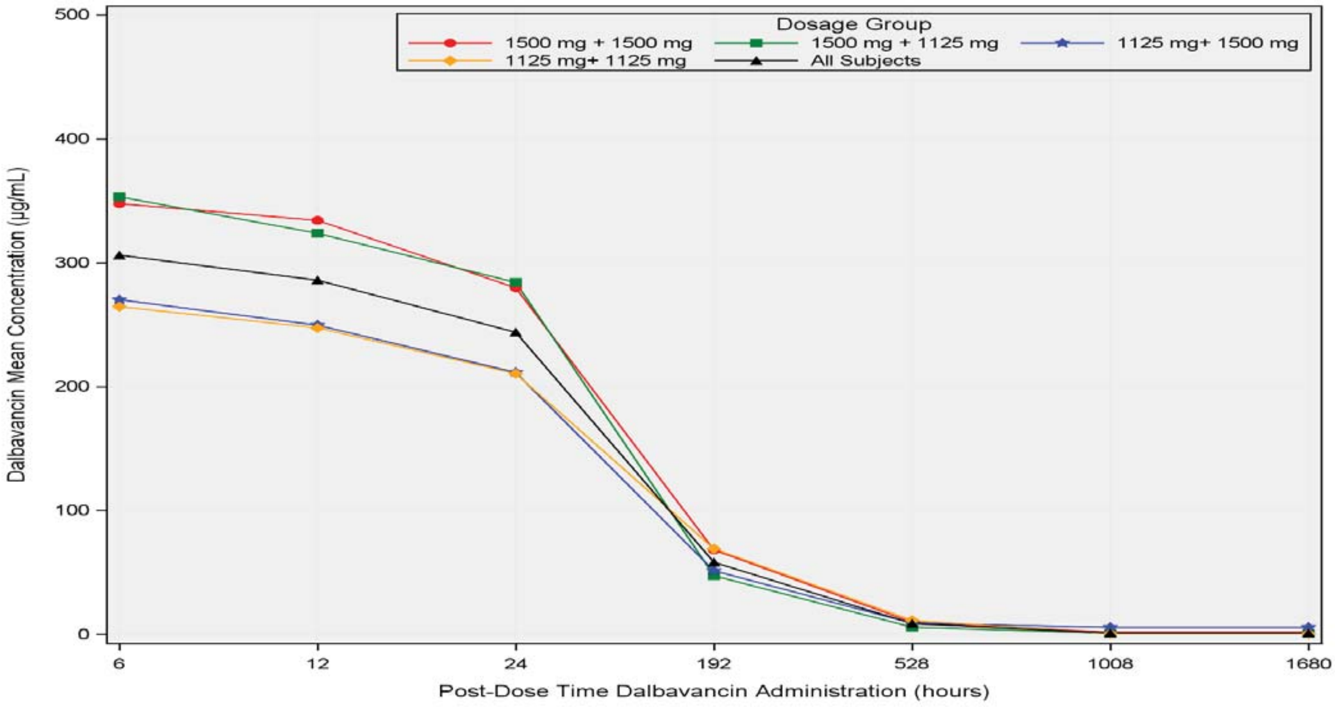

Programming Note: Add plot for 1500 mg only and 1125 mg only.

Figure 3: Semi-Log Plots of Geometric Mean of Plasma Concentration by Dalbavancin Dosage Group and Time Point – PK Population

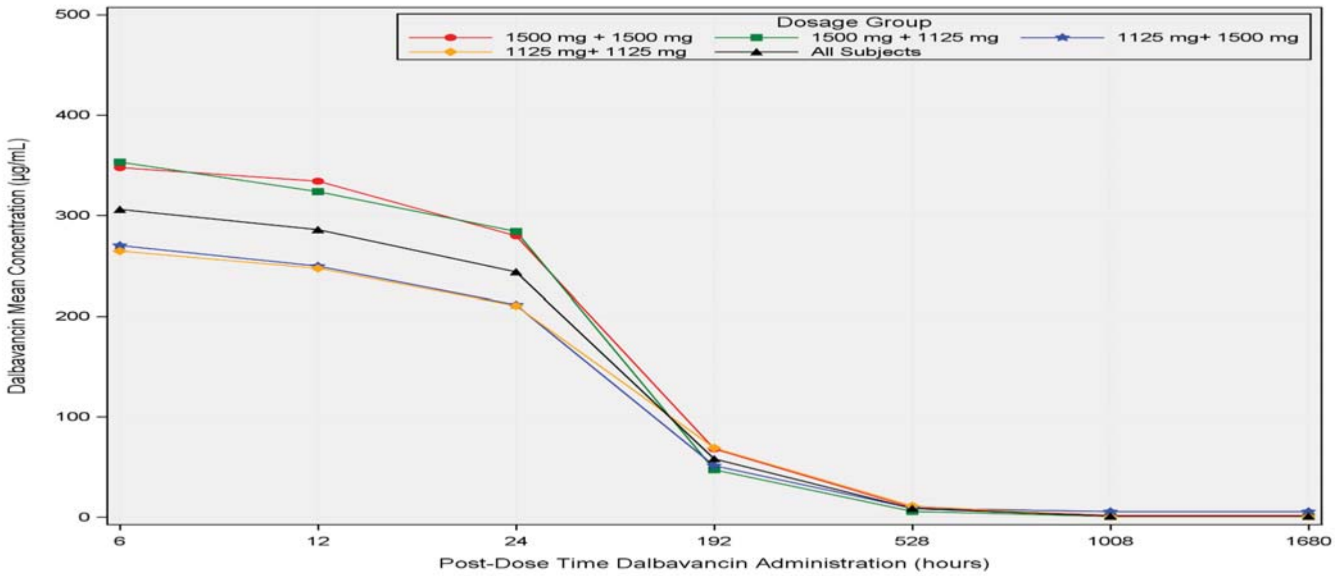

Programming Note: Add plot for 1500 mg only and 1125 mg only.

Figure 4: Semi-Log Plots of Geometric Mean of Plasma Concentration by Dalbavancin Dosage Group and Time Point – PK Population

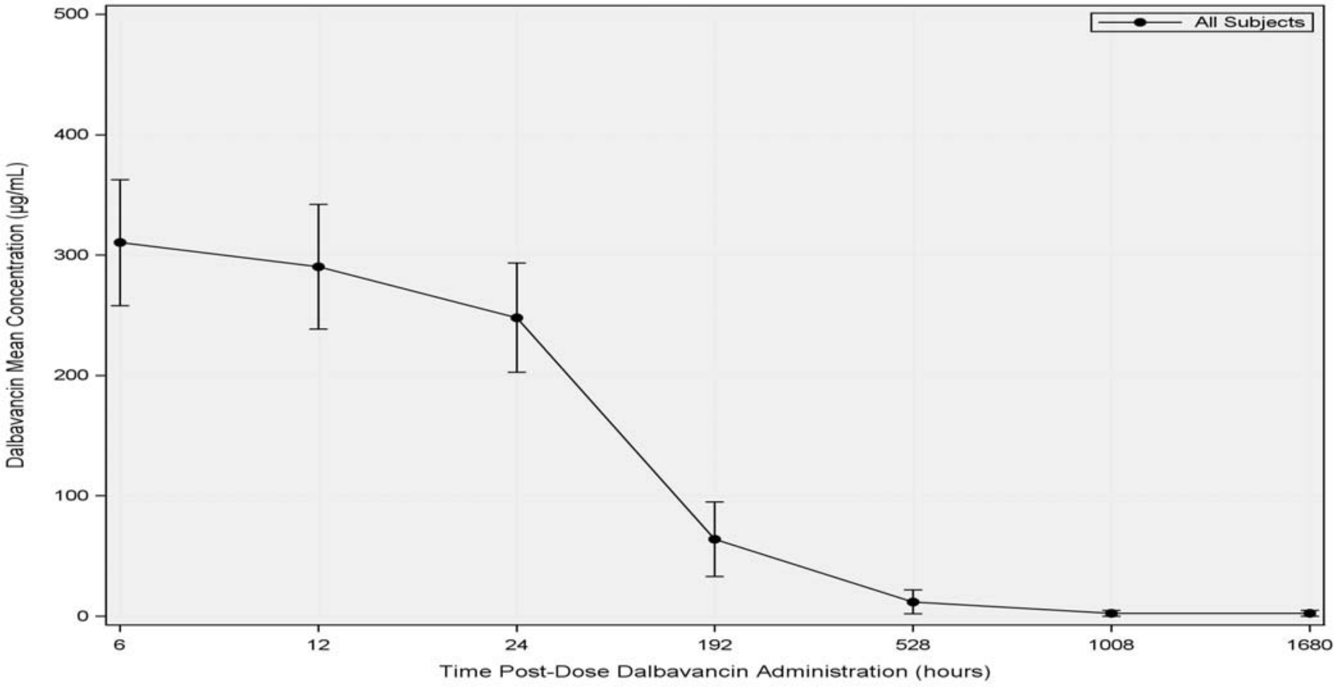

Programming Note: Add plot for 1500 mg only and 1125 mg only.

Figure 5: Semi-Log Plots of Geometric Mean of Plasma Concentration by Dalbavancin Dosage Group and Time Point – PK Population

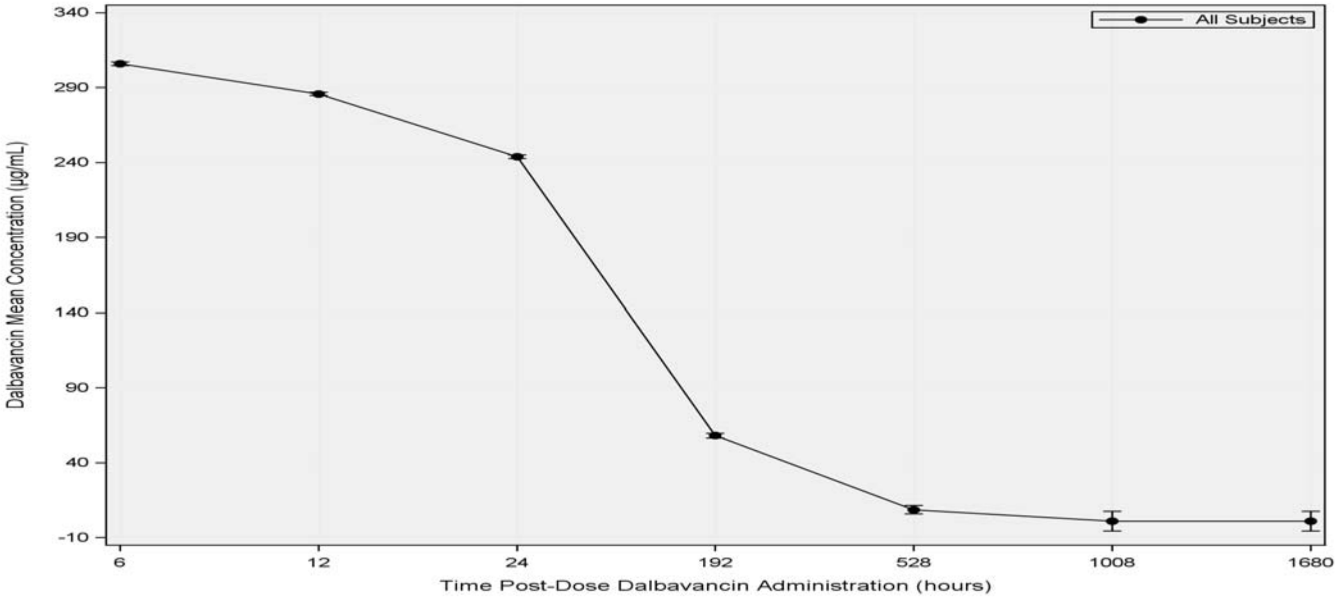

Programming Note: Add plot for 1500 mg only and 1125 mg only.

Figure 6: Fraction Unbound Concentration versus Time Since Last Dose in Linear (A) and Semi-Log (B) Scales

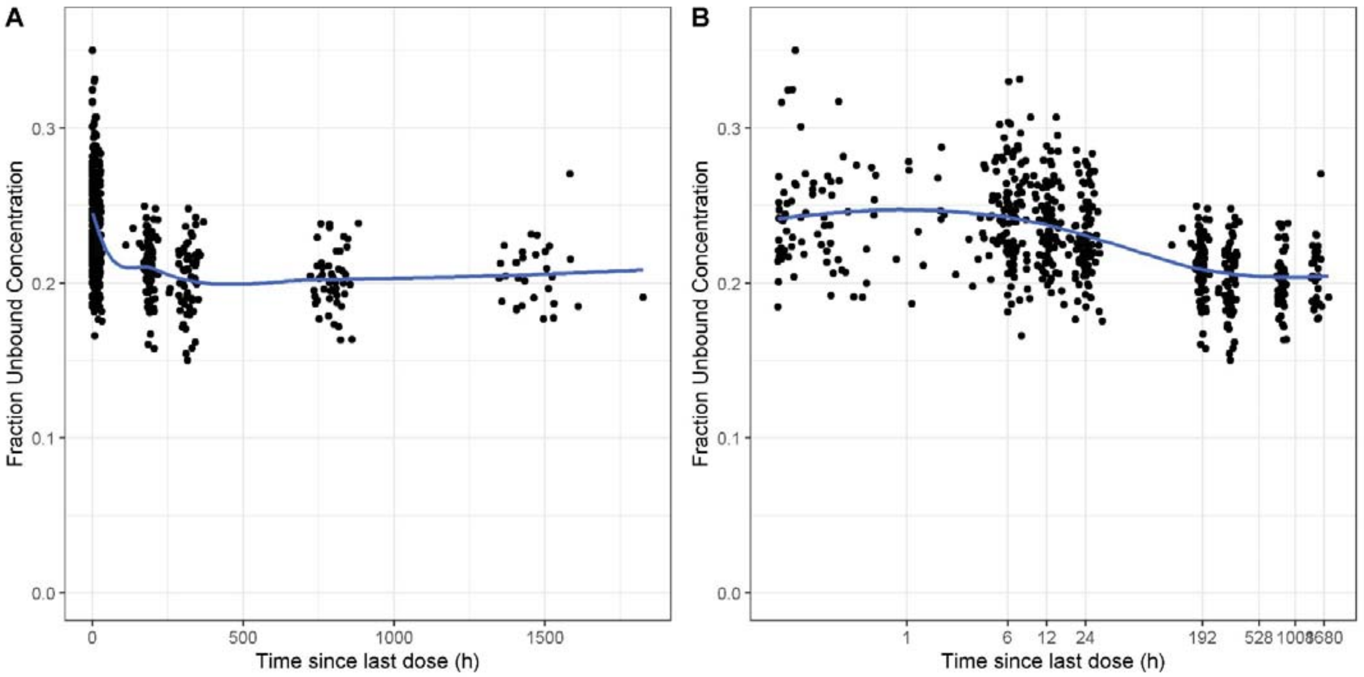

Figure 7: Fraction Unbound Concentration versus Total (A) and Unbound (B) Concentration

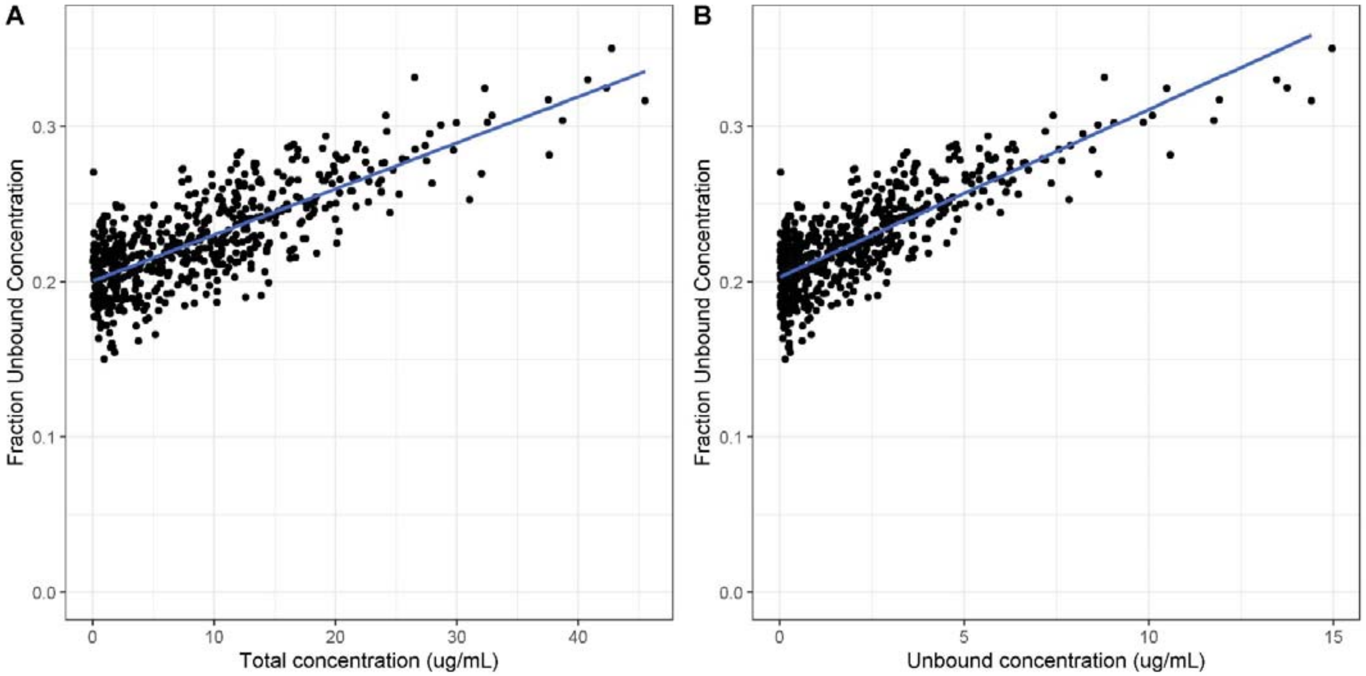

Figure 8: Final Model Diagnostics: Observed Concentrations versus Population Predicted Concentrations (ug/mL) of Total Dalbavancin

Similar format and implementation to following figure.

Figure 9: Final Model Diagnostics: Observed Concentrations versus Individual Predicted Concentrations (ug/mL) of Total Dalbavancin

[Implementation Note: If final PopPK model has variable fraction unbound, we need two figures (XXa and XXb) for both total and unbound dalbavancin concentrations]

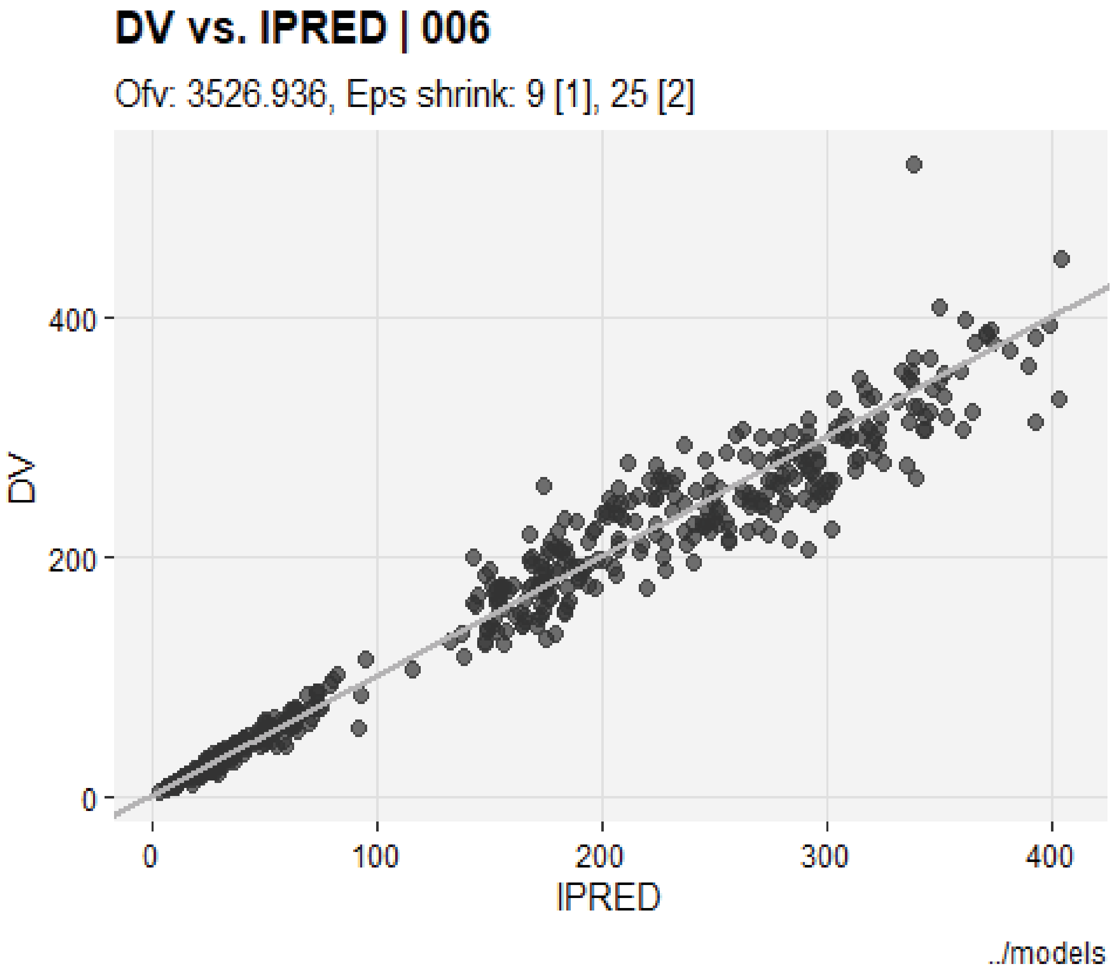

Figure 10: Final Model Diagnostics: Quantile-Quantile Plot of Individual Weighted Residuals

[Implementation Note: If final PopPK model has variable fraction unbound, we need two figures (XXa and XXb) for both total and unbound dalbavancin concentrations]

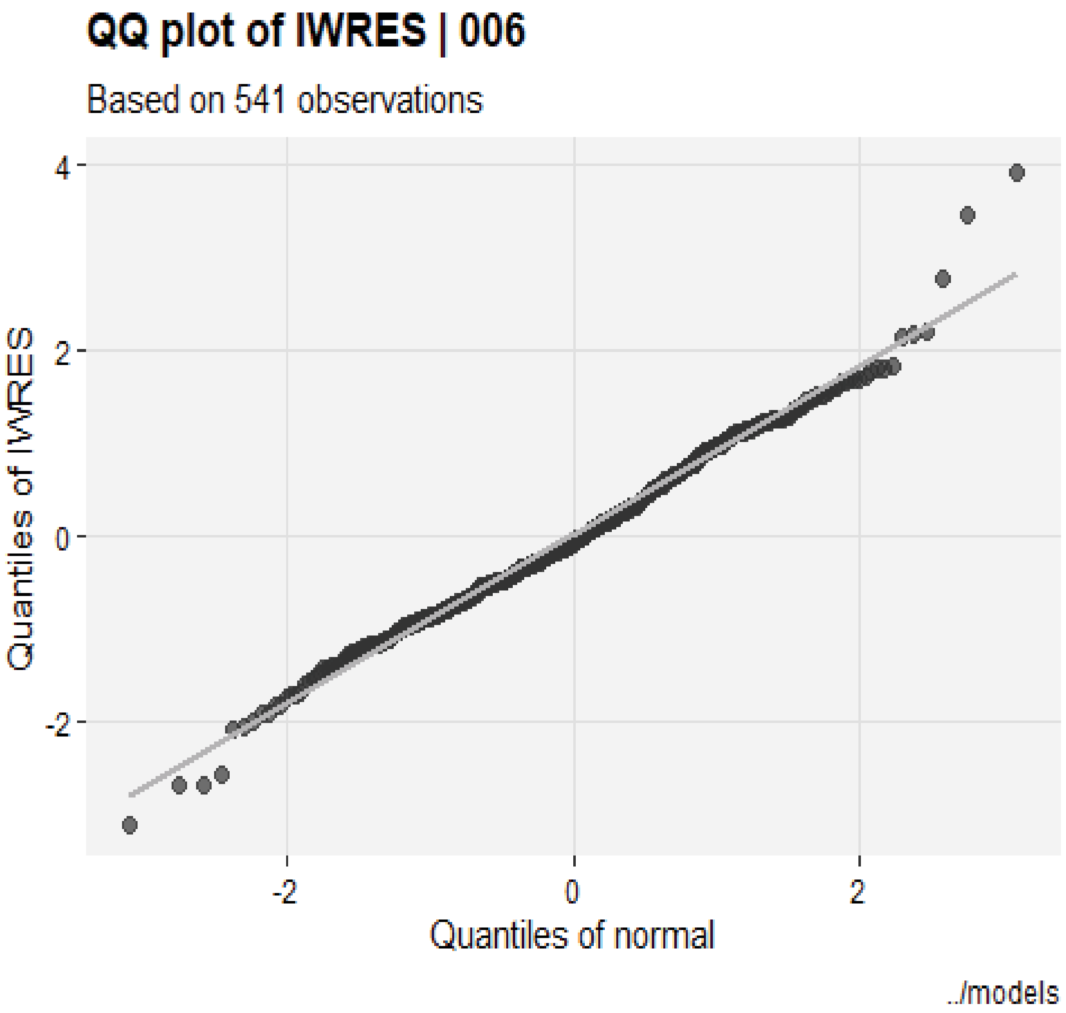

Figure 11: Final Model Diagnostics: Weighted Residuals versus Time Since Last Dose (h)

[Implementation Note: If final PopPK model has variable fraction unbound, we need two figures (XXa and XXb) for both total and unbound dalbavancin concentrations]

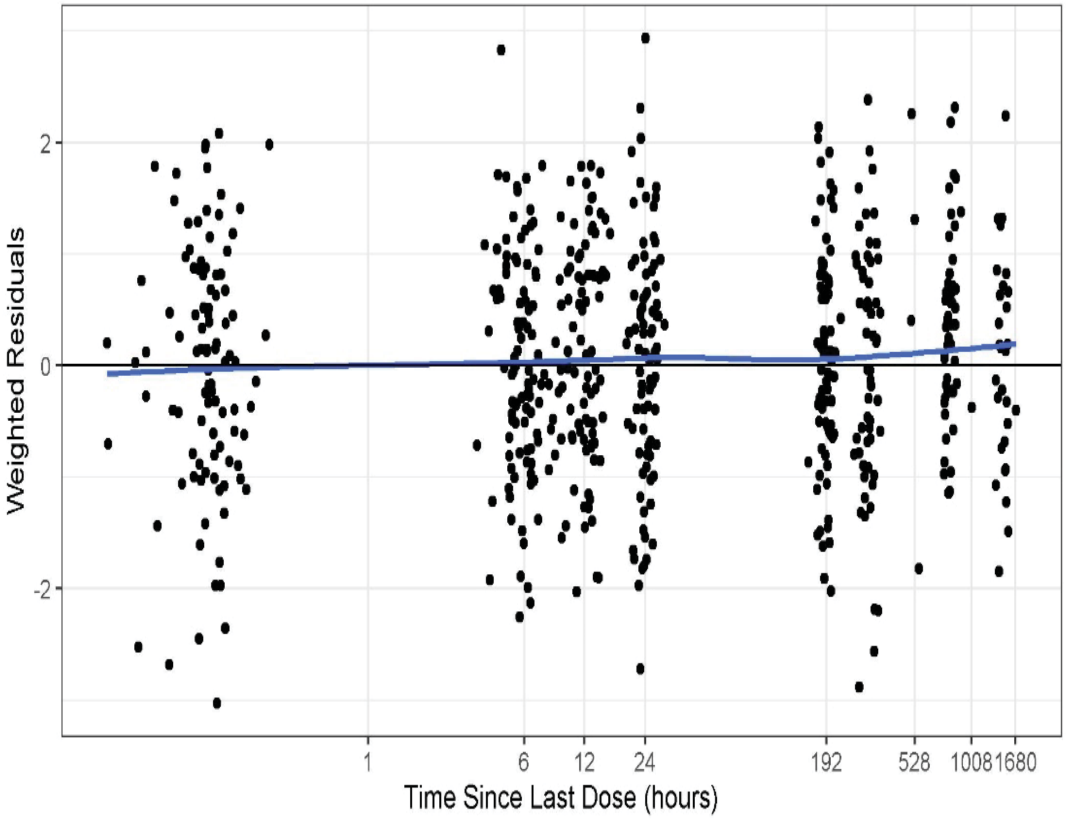

Figure 12: Final Model Diagnostics: Weighted Residuals versus Time Since First Dose (h)

[Implementation Note: If final PopPK model has variable fraction unbound, we need two figures (XXa and XXb) for both total and unbound dalbavancin concentrations]

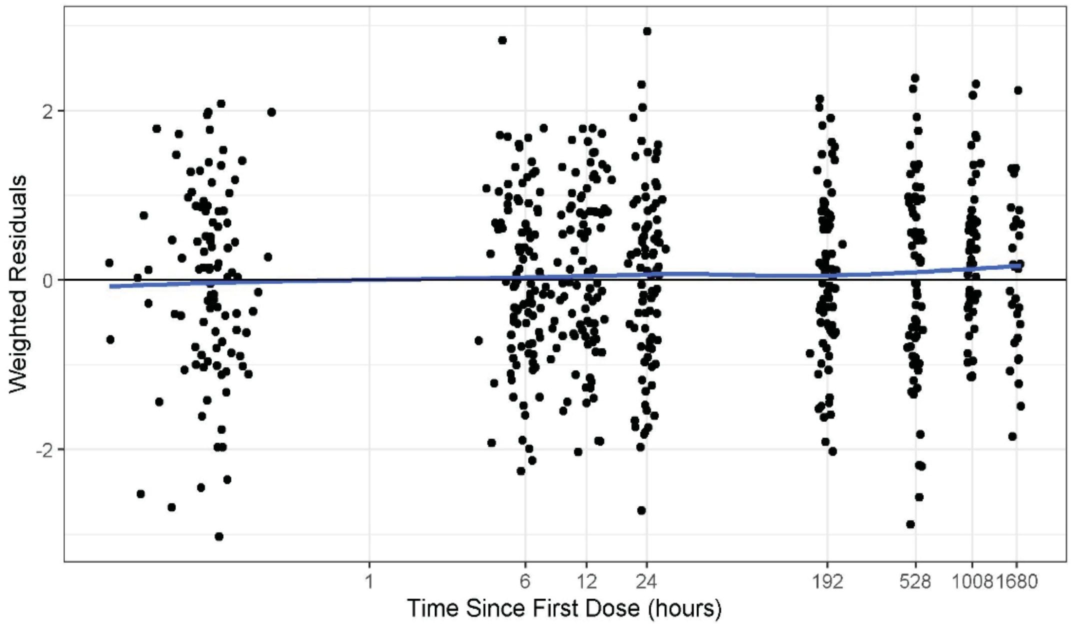

Figure 13: Final Model Diagnostics: Weighted Residuals versus Population Predicted Concentration (ug/mL) of Total Dalbavancin

Similar format to following figure.

Figure 14: Final Model Diagnostics: Individual Weighted Residuals versus Individual Predicted Concentration (ug/mL) of Total Dalbavancin

[Implementation Note: If final PopPK model has variable fraction unbound, we need two figures (XXa and XXb) for both total and unbound dalbavancin concentrations]

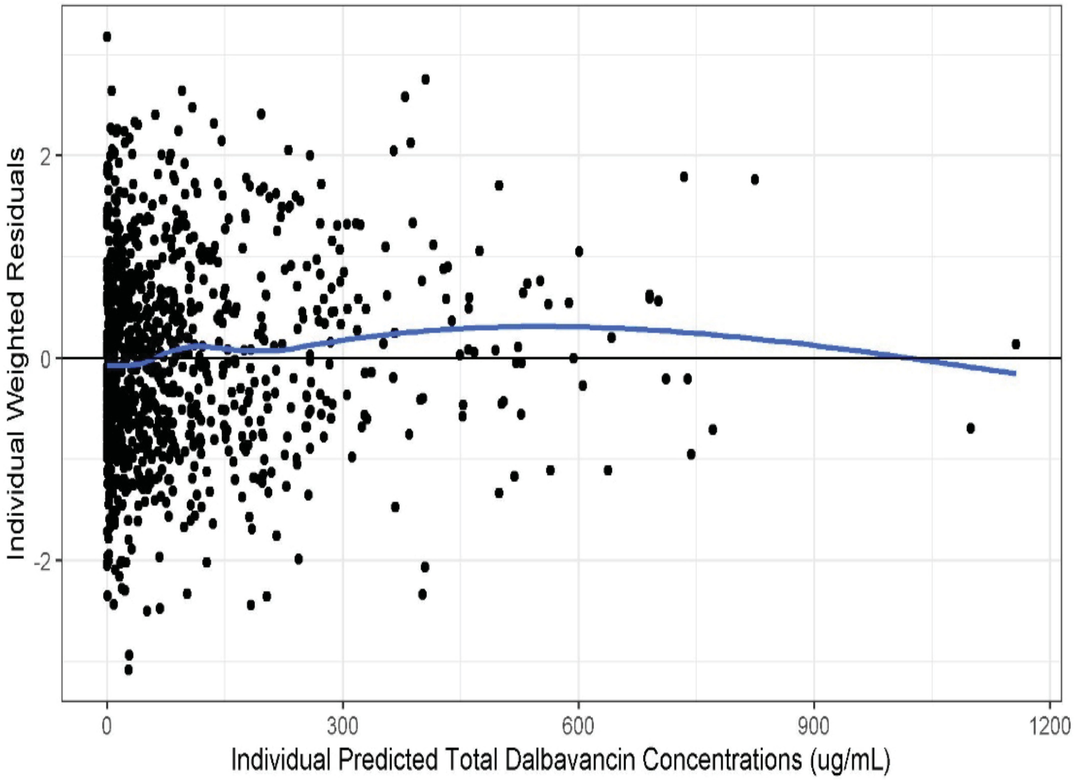

Figure 15: Final Model Diagnostics: Absolute Value of Individual Weighted Residuals versus Individual Predicted Concentration (ug/mL) of Total Dalbavancin

[Implementation Note: If final PopPK model has variable fraction unbound, we need two figures (XXa and XXb) for both total and unbound dalbavancin concentrations]

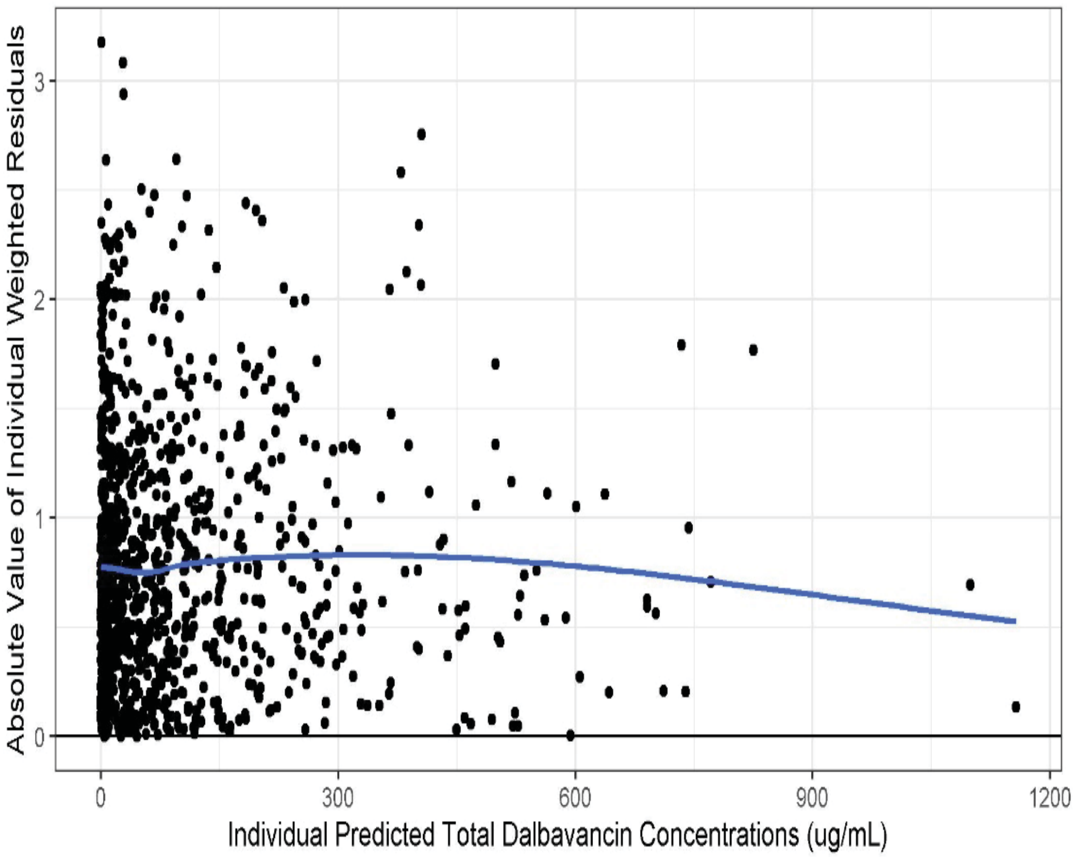

Figure 16: Final Model Diagnostics: Plots of Observed, Individual Predicted, and Population Predicted Concentrations (ug/mL) of Total Dalbavancin versus Time Since First Dose(h) – By Subject

[Implementation Note: If final PopPK model has variable fraction unbound, we need two figures (XXa and XXb) for both total and unbound dalbavancin concentrations]

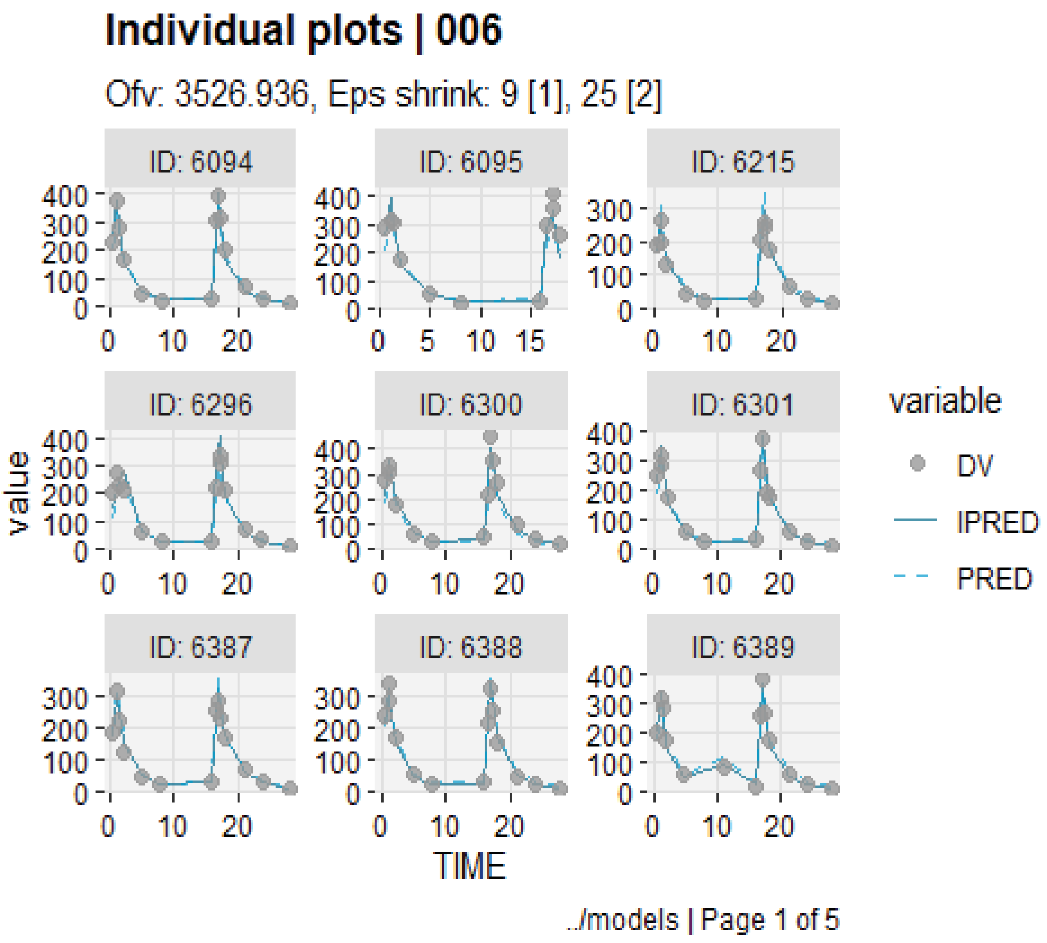

Figure 17: Final Model Diagnostics: Prediction-Corrected Visual Predictive Check for Fit on Total Plasma Dalbavancin Concentration

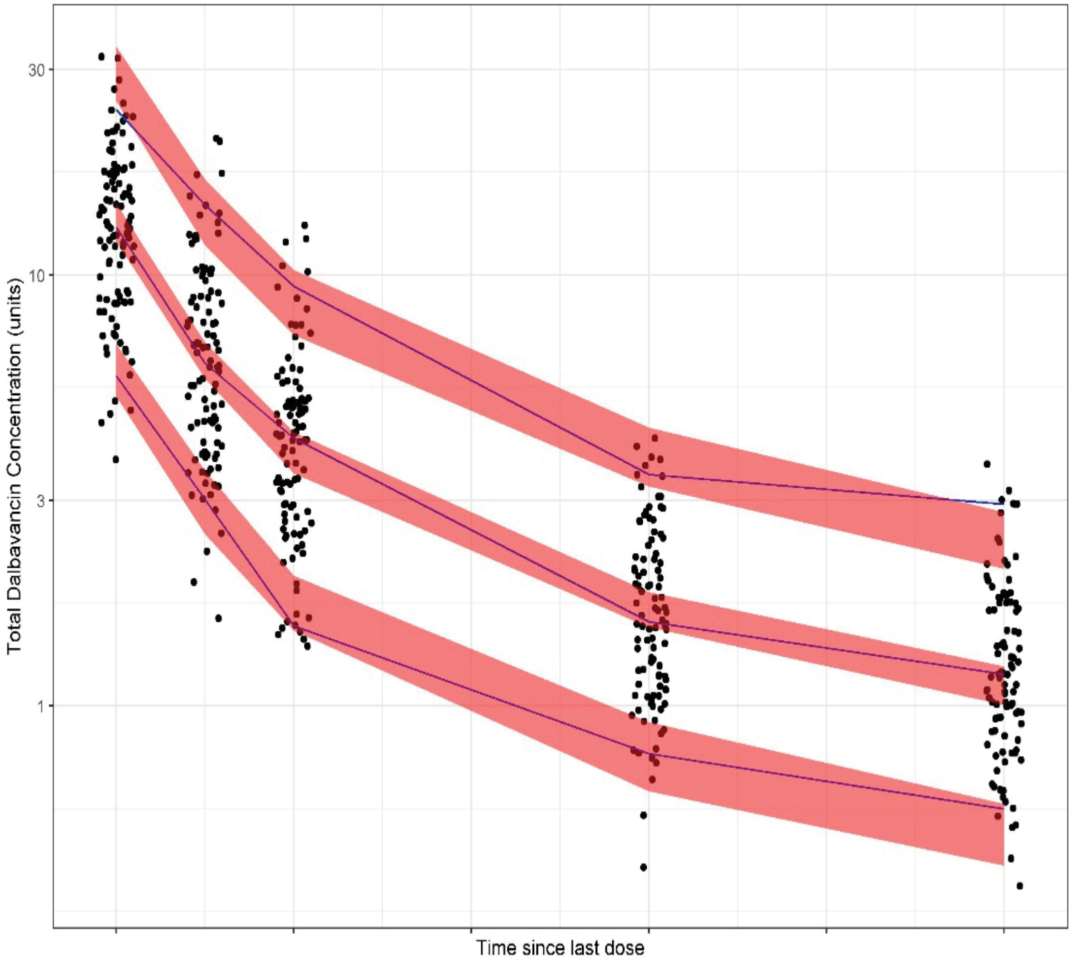

Figure with similar format:

Figure 18: Final Model Diagnostics: Prediction-Corrected Visual Predictive Check for Fit on Unbound Plasma Dalbavancin Concentration

Figure 19: Final PopPK Model-predicted Typical and Individual Empirical Bayes Estimates versus Covariates

[Implementation note: the PK parameters and covariates will be from the actual final PopPK model]

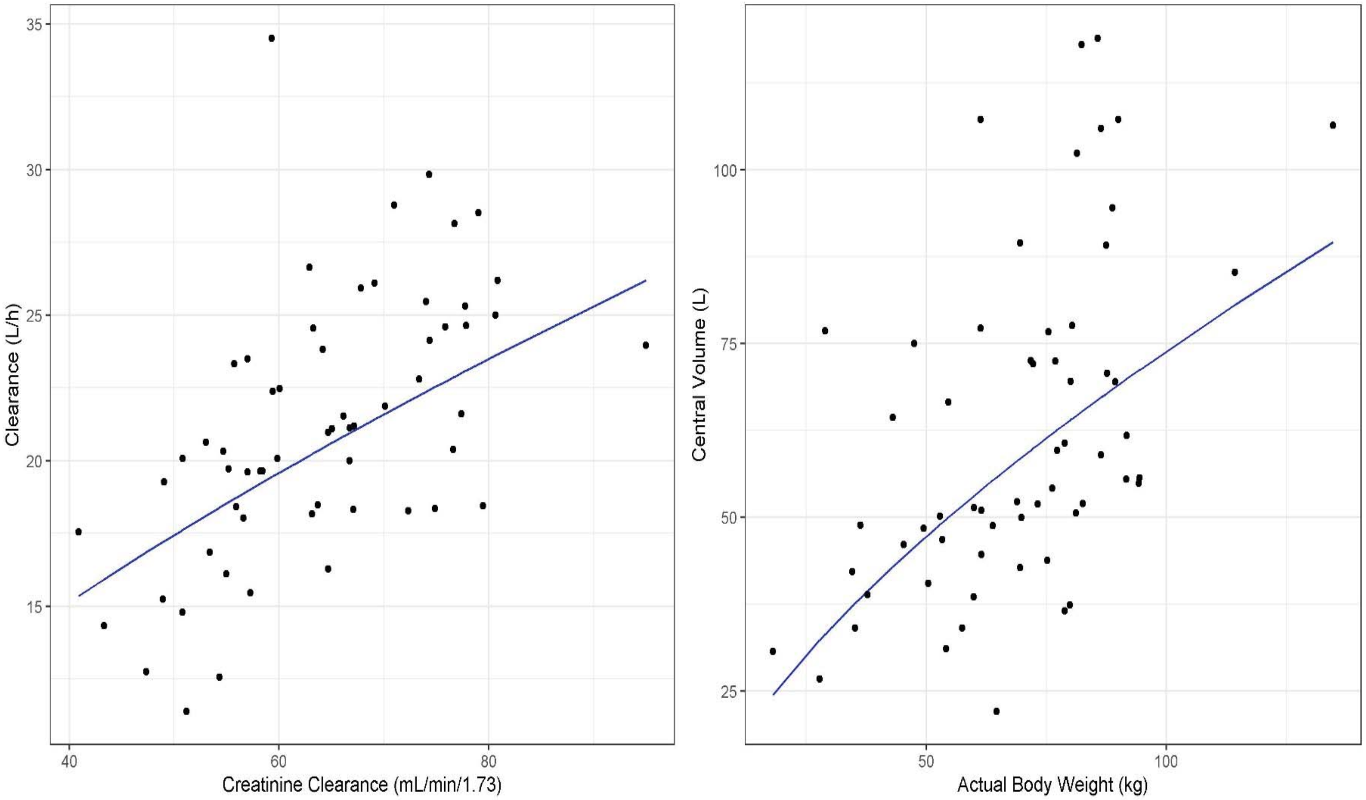

APPENDIX 3. LISTINGS MOCKUPS

TABLE OF LISTINGS

Listing 1: Subject Level Dalbavancin Doses – PK Population 68
Listing 2: Subject Level Dalbavancin Concentrations in Plasma – PK Population 69
Listing 3: Subject Specific NCA PK Parameters 70
Listing 4: Subject-Specific PK Parameters Estimated from PopPK Model 70

RESTRICTED

Listing 1: Subject Level Dalbavancin Doses – PK Population

[Implementation Note: Sort listing by PK Treatment Group (1500 mg + 1500 mg, 1500 mg + 1125 mg, 1125 mg + 1500 mg, 1125 mg + 1125 mg, and Standard of Care), Subject ID, Administration Date, and Infusion Start Time.]

		First IV Administration			Second IV Administration				
**Dalbavancin Dosage Group**	**Subject ID**	**Administration Date**	**Infusion Start Time**	**Infusion End Time**	**Administration Date**	**Actual Study Day[1]**	**Infusion Start Time**	**Infusion End Time**	**Both Administered Per Protocol? (Reason, if No)**
1500 mg + 1500 mg	**100001**								
1500 mg + 1125 mg	**100001**								
1125 mg + 1500 mg	**100001**								
1125 mg + 1125 mg	**100001**								
XXXXX + XXXXX	**100002**								

Note: [1] Actual study day of the second IV administration is relative to the date of the first IV administration (Day 1).

Listing 2: Subject Level Dalbavancin Concentrations in Plasma – PK Population

[Implementation Note: Sort listing by PK Treatment Group (1500 mg + 1500 mg, 1500 mg + 1125 mg, 1125 mg + 1500 mg, 1125 mg + 1125 mg, and Standard of Care), Subject ID, and Nominal Time.]

Dalbavancin Dosage Group	Subject ID	Nominal Time	Actual Time[1]	Sample Within Time Window (Y/N)	Laboratory Reported Concentration (ug/mL)	Analysis Concentration (ug/mL)	Used in NCA? (Reason, if No)	Used in λ_z Calculations? (Reason, if No)
1500 mg + 1500 mg	**100001**						BQL	
1500 mg + 1125 mg	**100001**							
1125 mg + 1500 mg	**100001**							
1125 mg + 1125 mg	**100001**							
XXXXX + XXXXX	**100002**							

Notes: [1]Times are relative to the end of the first infusion. For actual time, out of window times are indicated by an (*), substantially out of window times are indicated by two asterisks (**) and imputed times are indicated by three asterisks (***).
BQL = Below the Limit of Quantification.
λ_z = Terminal Phase Elimination Rate Constant

Listing 3: Subject Specific NCA PK Parameters

[Implementation Note: Sort listing by PK Treatment Group (1500 mg + 1500 mg, 1500 mg + 1125 mg, 1125 mg + 1500 mg, 1125 mg + 1125 mg, and Standard of Care) and Subject ID]

Dalbavancin Dosage Group	Subject ID	C_{max} (ng/mL)	T_{max} (hr)	T_{last} (hr)	$AUC_{0\text{-}last}$ (ng·hr/mL)	$AUC_{0\text{-}\infty}$ (ng·hr/mL)	λ_z (1/hr)	[Repeat for all parameters]

λ_z = Terminal Phase Elimination Rate Constant

Listing with similar format:

Listing 4: Subject-Specific PK Parameters Estimated from PopPK Model
