# Supplementary material for: Pharmacokinetics of Dalbavancin in Complicated Staphylococcus aureus Bacteremia: A Secondary Analysis of the DOTS Randomized Clinical Trial
Source: JAMA Netw Open. 2026 Apr 18;9(4):e2611652. doi: 10.1001/jamanetworkopen.2026.11652 (PMC13092111; doi:10.1001/jamanetworkopen.2026.11652)
Supplement: Supplement 2. — eAppendix. [file jamanetwopen-e2611652-s002.pdf]

Appendix A: Dalbavancin PK Analyses

Table of Contents

METHODS 4

Data 4

Study Design and Populations 4

Bioanalytical Methods 4

Covariates 4

Handling of BQL Data 5

Handling of Missing Data 5

Identification and Handling of Outliers 5

Base Structural Model Development 6

Covariate Model Development 6

Final Model Evaluation 7

Empirical Bayes Estimation of Parameters and Exposures 7

RESULTS 8

Data 8

Population PK Modeling 9

Base Structural Population PK Model 9

Covariate Model 10

Final Model Evaluation 11

Exposure Estimates 12

REFERENCES 13

LIST OF TABLES 14

Table 1: Subject and PK Sample Disposition for the Dalbavancin Treatment Group 14

Table 2: Summary of Categorical Demographic and Baseline Characteristics by Dalbavancin Dosage Group - PK Population 15

Table 3: Summary of Continuous Demographic and Baseline Characteristics by Dalbavancin Dosage Group - PK Population 16

Table 4: Summary Statistics of Total and Unbound Plasma Concentrations (µg/mL) by Dalbavancin Dosage Group and Nominal Time - PK Population 19

Table 5: Summary of Pop-PK Analysis Models Considered 24

Table 6: Estimation Summary of Plasma Pop-PK Parameters Estimated in the Base Structural Model and the Final Covariate Model 25

Table 7: Summary of the Forward Selection Process of Covariates for the Pop-PK Model.........28
Table 8: Summary of the Backward Elimination Process for Covariates from the Pop-PK Model30
Table 9: Summary Statistics of PK Parameters from Pop-PK Model by Dalbavancin Dosage Group - PK Population 33
LIST OF FIGURES 38
Figure 1: Distribution of Total and Unbound Dalbavancin Concentrations, by BQL Status 38
Figure 2: Observed Total Plasma Concentration Scatterplot vs. Time from First Dose by Dalbavancin Dosage Group, BQL Values Omitted 39
Figure 3: Observed Unbound Plasma Concentration Scatterplot vs. Time from First Dose by Dalbavancin Dosage Group, BQL Values Omitted...40
Figure 4: Observed Total Plasma Concentration Scatterplot vs. Time from First Dose by BQL and Analytical Exclusion Status 41
Figure 5: Observed Unbound Plasma Concentration Scatterplot vs. Time from First Dose by BQL and Analytical Exclusion Status 42
Figure 6: Summarized Total Plasma Concentrations by Nominal Time Since First Dose - Geometric Mean ±1 Geometric SD 43
Figure 7: Summarized Total Plasma Concentrations by Nominal Time Since First Dose and Dalbavancin Dosage Group - Geometric Mean..44
Figure 8: Summarized Unbound Plasma Concentrations by Nominal Time Since First Dose - Geometric Mean ±1 Geometric SD, BQL Values Excluded or Imputed 45
Figure 9: Summarized Unbound Plasma Concentrations by Nominal Time Since First Dose and Dalbavancin Dosage Group - Geometric Mean, BQL Values Excluded 46
Figure 10: Summarized Unbound Plasma Concentrations by Nominal Time Since First Dose and Dalbavancin Dosage Group - Geometric Mean, Imputed BQL Values Included 47
Figure 11: Observed Total vs. Unbound Concentrations, Including BQL Observations 48
Figure 12: Observed Fraction Unbound Concentration vs. Time Since First Dose, Imputed BQL Values Included (A) and Excluded (B) 49
Figure 13: Final Model Diagnostics: Observed Concentrations versus Population Predicted Concentrations (µg/mL) of Total and Unbound Dalbavancin..................................50
Figure 14: Final Model Diagnostics: Observed Concentrations versus Individual Predicted Concentrations (µg/mL) of Total and Unbound Dalbavancin..................................51
Figure 15: Final Model Diagnostics: Quantile-Quantile Plot of Conditional Weighted Residuals52
Figure 16: Final Model Diagnostics: Quantile-Quantile Plot of Individual Weighted Residuals .53
Figure 17: Final Model Diagnostics: Conditional Weighted Residuals versus Time Since First Dose 54
Figure 18: Final Model Diagnostics: Individual Weighted Residuals versus Time Since First Dose 55
Figure 19: Final Model Diagnostics: Conditional Weighted Residuals versus Population Predicted Concentrations (µg/mL) of Total and Unbound Dalbavancin..................................56
Figure 20: Final Model Diagnostics: Individual Weighted Residuals versus Individual Predicted Concentrations (µg/mL) of Total and Unbound Dalbavancin..................................57

Figure 21: Final Model Diagnostics: Absolute Value of Individual Weighted Residuals versus Individual Predicted Concentrations (µg/mL) of Total and Unbound Dalbavancin ..58

Figure 22: Final Model Diagnostics: Prediction-Corrected Visual Predictive Check for Fit on Total Plasma Dalbavancin Concentrations 59

Figure 23: Final Model Diagnostics: Prediction-Corrected Visual Predictive Check for Fit on Unbound Plasma Dalbavancin Concentrations 60

Figure 24: Final Pop-PK Model-predicted Typical and Individual Empirical Bayes Estimates versus Covariates 61

APPENDICES (SEPARATE DOCUMENTS)...62

Appendix B: Base PopPK Model - Results and Diagnostics plots (Pumas) 62

Appendix C: Full PopPK Model - Results and Diagnostics plots (Pumas).................................62

Appendix D: Final Model Diagnostics: Plots of Observed, Individual Predicted, and Population Predicted Concentrations (µg/mL) of Total and Unbound Dalbavancin versus Time Since First Dose by Subject...62

Appendix E: Final PopPK Model - Results and Diagnostics plots (Pumas) 62

METHODS

Data

Study Design and Populations

In the DOTS trial, 200 subjects were randomized 1:1 to receive either dalbavancin or standard of care antibiotics for completion of treatment of their complicated S. aureus bacteremia. Subjects were stratified by presence of methicillin resistant *S. aureus* (MRSA) versus methicillin-susceptible *S. aureus* (MSSA). Those randomized to the dalbavancin treatment group received two doses of dalbavancin IV one week apart on Day 1 and Day 8 after randomization, each dose being either 1125 mg or 1500 mg depending on measured CrCL before the respective dose. Subjects with CrCL ≥ 30 mL/min and subjects receiving regular hemodialysis or peritoneal dialysis would receive 1500 mg IV dalbavancin over 30 (± 10) minutes. Subjects with CrCL < 30 mL/min who were not receiving regular hemodialysis or peritoneal dialysis would receive 1125 mg IV dalbavancin over 30 (± 10) minutes. Subjects were planned to be divided into 6 groups according to the dose(s) of dalbavancin administered:

- 1500 mg dalbavancin for both doses
- 1500 mg dalbavancin for the first dose and 1125 mg for the second
- 1125 mg dalbavancin for the first dose and 1500 mg for the second
- 1125 mg dalbavancin for both doses
- 1500 mg dalbavancin for a single dose, no second dose received
- 1125 mg dalbavancin for a single dose, no second dose received (Note: No subjects received this dose schedule)

Bioanalytical Methods

Plasma samples for PK analysis were analyzed at a central laboratory to determine total (bound and unbound) and unbound dalbavancin concentrations (µg/mL). The lower limit of quantification (LLOQ) for total and unbound concentrations were respectively 0.5 and 0.05 µg/mL.[1]

Covariates

The following patient demographic and clinical descriptors were evaluated for their potential to explain a portion of the inter-individual variability (IIV) in selected unbound and total plasma dalbavancin PK parameters:

- Sex
- Age (years)
- Weight (kg)
- Lean body weight (LBW) (kg)
- Body surface area (BSA) (m^2)
- Body mass index (BMI) (kg/m^2)
- Ideal body weight (IBW) (kg)
- Serum creatinine (mg/dL) at baseline and time-varying
- Creatinine clearance (mL/min) at baseline and time-varying: calculated using the Cockcroft-Gault method[2]
- Serum albumin (g/dL) at baseline and time-varying

- Renal replacement therapy (RRT): a flag (0/1) from a clinical form collected on treatment visits (Days 1 and 8) for whether the subject was receiving dialysis.

Handling of BQL Data

In descriptive analyses, unbound and total concentrations that were below the lower limit of quantification (BQL) were either excluded or imputed with LLOQ/2 (or both for comparison), depending on the analytical context. A similar approach was taken for descriptions of fraction unbound, which would either be missing for either total or unbound component being BQL or calculated using the imputed concentration (LLOQ/2). For population PK analyses, BQL concentrations were modeled using either Method 3 (M3) or Method 4 (M4) described by Beal.[3] Both methods employ censored distributions that places all the observations below LLOQ in a point mass at the LLOQ. M4 additionally includes the assumption that the measured concentrations cannot be negative.

Handling of Missing Data

Missing concentrations were not imputed at any point. Any actual time measurement that was missing, e.g., PK sampling time or dosing time, was imputed using nominal timepoint as long as there was reasonable confidence it was done within the protocol-defined window and flagged for sensitivity analyses. Missing nominal time was not imputed. Missing dosing data was investigated and either imputed with protocol-defined targets or removed from analysis, depending on the pattern of missingness.

Covariates with more than 20% missingness were excluded from the analysis. For covariates with <20% missingness in the analysis dataset, efforts were made to identify patterns to the missingness with respect to dosage and other covariates of interest. If a covariate was determined reasonably to be missing completely at random, then missing values were imputed using simplistic methods: stationary categorical variables were imputed using the most common value in the dataset; stationary continuous variables were imputed using the median data value; time-varying covariates were imputed using last-observation-carried-forward (LOCF) if occurring after a non-missing measurement or the imputed baseline value, or first-observation-carried-backward (FOCB) for time points before the first non-missing observations; if all time-varying values for a subject were missing, the group-specific median was used to impute the baseline value, where group was determined appropriately for the variable.

Identification and Handling of Outliers

An outlier was defined as an aberrant observation that substantially deviated from the rest of the observations. PK outlier concentrations identified as outlying data points were excluded from the PK analyses given the potential for these observations to negatively impact model convergence and/or the final parameter estimates. Observations of outlying concentrations or potentially outlying subjects were examined with the study team to determine potential reasons for deviation.

Outlier detection in the analysis dataset was based primarily upon visual inspection of individual and pooled unbound and total plasma dalbavancin concentration-time data. As suggested by the FDA guidance, searching for additional outliers during the analysis were based upon graphical exploration of individual and population conditional weighted residuals (CWRES) during structural PK model development; observations with normalized weighted residuals greater than 5 were identified or confirmed as outliers. All concentrations from a single patient could be excluded cautiously only if the entire intensively sampled PK profile failed to follow a reasonable pattern relative to the dosing.[4] If most of the suspected outlier concentrations appeared to occur at roughly the same time since last dose, additional attempts were made to update the structural model to try

to capture these observations. Outliers were excluded from the analysis data, but sensitivity analyses were performed comparing the inclusion of outliers on the final model.

Base Structural Model Development

Base model development consisted of the determination of structural, between subject variability (BSV), and residual unexplained variability (RUV) models. The overall goal of base model development was to determine a stable and parsimonious model that provided an adequate description of the data. The adequacy of the model to describe the available data was assessed using standard goodness-of-fit diagnostic plots, and the stability and parsimoniousness of the model was assessed based on Akaike Information Criteria (AIC), as well as condition number and precision of parameter estimates. The condition number is the ratio between the largest and smallest eigenvalues of the correlation matrix and serves as a check to ensure the model is not over-parametrized if it is under 1000 or doesn't undergo a large increase with the addition of a parameter to the model.

Based on prior dalbavancin PK modeling studies, a three-compartment model with zero-order input and first-order elimination to simultaneously fit the total and unbound plasma dalbavancin concentration-time data was used as the initial structural PK model.[5] Other two- and three-compartment models were considered as necessary. PopPK models were described by estimation of the typical values of structural model parameters of total plasma such as volumes of distribution in central (V_1) and peripheral compartment(s) (V_2, V_3, etc.), central clearance (CL), and/or intercompartmental clearance(s) (Q_2, Q_3, etc.), as well as a model of unbound and total concentrations or fu. In the initial base model, the f_u of dalbavancin was assumed to be a constant function of total dalbavancin concentration, and the concentration dependence of fu was considered as necessary. Non-linear (non-saturable, saturable, and empirical) models of plasma protein binding as a function of total dalbavancin concentration were also considered to identify the most parsimonious model which provided the most plausible fit to the data. Using this approach, f_u would not be constant and free dalbavancin concentrations would instead be a non-linear function of the total dalbavancin concentrations.

All the models were estimated using the second order Laplace approximation with interaction method in Pumas. This estimation method was necessary for the implementation of M3 and M4 models of BQL concentrations which can't be achieved with first-order conditional estimation method with interaction (FOCEI). Initially, BSV was estimated for all parameters using the exponential model, assuming a log-normal distribution of individual-specific parameter estimates. With this variability model, estimates were easily presented as coefficients of variation. RUV was modeled with proportional (i.e., constant coefficient of variation (CCV)), additive, combined (additive plus proportional), and log error models. Goodness-of-fit plots and estimation performance informed the choice of RUV models.

Covariate Model Development

Following the development of an appropriate base structural model, a covariate model was developed describing the influence of covariates on selected parameters. The purpose of covariate model development was to determine robust and generalizable parameter-covariate relationships. Covariate model development consisted of exploratory analysis to select parameter-covariate relationships to be considered for inclusion in the full model, followed by development of the full model using forward selection with pre-determined ranks of correlated covariates and functional forms, then backward elimination. This approach was expected to mitigate selection of spurious covariate-parameter relationships by over-fitting of the available data.[6]

Parameter-covariate relationships were initially explored in eta-plots of individual subject parameter deviation vs. candidate covariates. Correlation of continuous covariates were explored for choice of candidate covariates among correlated groups. Continuous covariates were initially evaluated in the model as a power

model for all candidate parameters, using the PK population median as a reference value to represent the intercept parameter of the model. Linear functional forms may have been subsequently considered in the model building process. All candidate categorical covariates were dichotomous and modeled with an exponential shift from reference level.

The full popPK model was constructed through stepwise forward inclusion of covariate-parameter relationships that exhibited statistical significance in univariate analysis (Likelihood Ratio Test (LRT) $p<0.05$), starting with the most significant ones. Covariate-parameter relationships were retained in the full model if they resulted in a p-value < 0.01. Additional criteria for inclusion in the stepwise process were also considered, including model stability, a decrease in BSV for the parameter of interest and no compensatory increase in other BSV. After completion of forward selection, the BSV models and covariate functional forms were re-evaluated. Pair-wise comparisons of the η-values for each parameter were graphically examined to determine unmodeled covariance structure between those parameters in the popPK model. Distribution of η-values for each parameter were also graphically examined. In addition, diagnostic plots of full-model η vs. covariates were examined to confirm that there were no evident trends in the unexplained variability of PK parameters. Other functional forms of covariate-parameter relationships were considered to resolve any trends that were evident.

Univariate stepwise backward elimination was conducted on the full popPK model. A covariate-parameter relationship was considered statistically significant if its removal from the model resulted in an LRT p-value<0.001. In each step of backward elimination, the least significant covariate (with the highest p-value≥0.001) was systematically removed from the model until all remaining covariates in the model demonstrated statistical significance (p-value<0.001).

Final Model Evaluation

The final popPK model was assessed using the same model diagnostic and evaluation criteria as described previously. In addition, the final popPK model was assessed using the visual predictive check (VPC), which graphically examined the agreement between the 5th, 50th, and 95th percentiles of the observed and the individual simulated unbound and total plasma dalbavancin concentrations across time intervals using 1,000 dataset simulations. Due to differences in baseline covariates and dosage schemes, the prediction-corrected visual predictive check (pcVPC) method[7] was applied, wherein the predicted and observed concentrations were normalized to the median independent variables within the simulation bin.

Further qualification of the final model was performed by bootstrapping the original dataset (1000 replicates) and estimating the model on each replicate to generate confidence intervals on population-level model parameters and all other measures of exposure. Standard diagnostic plots were output for the final model and presented in this report, as well as exploratory plots of model-predicted covariates vs. typical value parameters, overlaid upon individual post-hoc parameter values.

Empirical Bayes Estimation of Parameters and Exposures

Individual empirical Bayes estimates (EBEs) of PK parameters and exposure measures derived from the final population PK model fit for each patient's individual dosing and covariates were generated. All PK parameters with BSV in the final model were estimated. In addition, the steady-state total plasma volume of distribution (V_{ss}) was estimated as the sum of the central (V_1) and peripheral volume terms (V_2, etc.). The following exposure variable EBEs were estimated for each patient from simulated data for free and total plasma concentrations: plasma concentration at day 22 (C_{22day}) and $AUC_{0\text{-}22day}$.

RESULTS

Data

Table 1 describes subject and PK sample disposition with respect to study design and PK objectives. One hundred (100) subjects were randomized to receive dalbavancin treatment as per the protocol. Ninety-eight (98) subjects contributed a total of 645 PK samples drawn and analyzed. The number of PK samples, measurable and BQL, by sampling time point is shown in **Figure 1** for all total and unbound dalbavancin concentrations received, before exclusion.

Of the 98 subjects with PK samples drawn, 97 subjects comprised the PK Population with at least one measurable dalbavancin concentration observed (the one remaining subject had one BQL pre-dose sample). Most subjects in the PK Population received two 1500 mg doses of IV dalbavancin (N=86). The remaining 11 subjects were in the following groups: 1500 mg + 1125 mg (N=2), 1125 mg + 1500 mg (N=3), 1125 mg + 1125 mg (N=3), and 1500 mg only (N = 3). No subjects received only a first dose of 1125 mg.

Of the 97 subjects in the PK Population, 71% were male. American Indian or Alaska Native was a race reported for 1% of subjects, 5% reported as Asian, 21% as Black or African American, and 69% as White. The median age was 56 years, with a range of 20 to 83 years. Median (range) weight was 83.8 (48.5-151.5) kg, and median (range) BMI was 28.02 (16.9-50.2) kg/m2. 12% of subjects were on RRT at baseline. Median (range) creatinine clearance at baseline overall was 101.263 (4.17-371.99) mL/min but varied by dosage groups.

Summary statistics of demographic and other baseline clinical characteristics of the subjects in the PK Population, stratified by dose groups, are presented in detail in **Table 2** and **Table 3** for categorical and continuous variables, respectively.

Two subjects likely had their sample draws from pre- and post-dose transposed in the laboratory before bioanalysis, resulting in both the total and unbound samples appearing to reach maximum concentration in the pre-dose sample and BQL in the first sample after the first dose. These 8 concentrations were flagged and removed from all exploratory and PK analyses. After this exclusion, exploratory analyses included 640 samples—557 of which were post-dose—from 97 subjects, and all pre-dose concentrations of total and unbound dalbavancin were BQL. Numerical summaries of concentrations are shown in **Table 4**. No BQL data were observed among post-dose total concentrations, while 20% of unbound concentrations were BQL, mostly at the later time points: 54 of 74 (73%) samples were BQL at Day 42 and 51 of 52 (98%) were BQL at Day 70.

Two PK samples each contributed outlying total and unbound dalbavancin concentrations which were identified in exploratory analysis and later confirmed in the popPK analysis using the criteria |CWRES|<5. These two samples (four concentrations), as well as all Day 1 pre-dose draws were excluded from popPK analyses (an additional 83 samples or 166 total/unbound concentrations), including two subjects whose nominal pre-dose samples were taken shortly after infusion.

Finally, one high-leverage concentration – the single unbound observation that was above LLOQ at Day 70 – was flagged and removed, leaving 555 total and 554 unbound concentrations for Population PK analysis. Overall, a median of 6 available PK samples were available per subject with a minimum of 1 sample and a maximum of 8 samples. See **Table 1** for more detail about sample availability and disposition. Concentrations over time since first dose are shown by dose group after applying exclusions in total (**Figure 2**) and unbound (**Figure 3**) dalbavancin. Excluded concentrations are shown among the analogous scatterplots in **Figure 4** and **Figure 5**.

Concentrations are summarized (geometric mean (GM) and geometric standard deviation (GSD)) by nominal time and presented for total dalbavancin in plasma in **Figure 6** and stratified by dose group in **Figure 7**. Due to the large number of unbound BQL in later time points, summaries of unbound dalbavancin were presented

both by removing BQLs and imputing BQL concentrations: overall (Figure 8) and stratified by dose group excluding BQLs (**Figure 9**) and imputing (**Figure 10**).

Unbound concentrations are plotted against total concentrations (**Figure 11**), showing a relatively linear relationship considering only the concentrations above BQL but not ruling out a non-linear relationship with or without BQL concentrations. Plots of fraction unbound over time (**Figure 12**) suggest a loose non-linear decrease of fraction unbound over time since first dose.

Population PK Modeling

Base Structural Population PK Model

Based on prior dalbavancin PK modeling studies, a three-compartment model with zero-order input and first-order elimination was used as the initial structural PK model to describe total plasma PK.[5] A two-compartment model was also evaluated but resulted in a higher AIC (4110 vs. 3876) and showed bias in diagnostic plots for both total and free concentrations.

An overview of the key model building steps is summarized in **Table 5**. The selection and adequacy of the model to describe the available data was assessed using standard diagnostic plots, AIC, as well as condition number and precision of parameter estimates. In the initial base model, the fraction of dalbavancin f_u was assumed to be a constant, i.e., $C_U(t) = C_T(t) * f_u$, where $C_U(t)$ and $C_T(t)$ are, respectively, unbound and total concentrations of dalbavancin and any given post-dose time, t. This model did not properly describe the unbound concentrations, with under-prediction of high values. Various empirical non-linear relationships (e.g., exponential, logarithm, power, sigmoidal relationships) between unbound and total concentrations were evaluated. The best model, that resulted in the lowest AIC and best diagnostic plots, was a power relationship defined as follows:

$$C_U(t) = A * C_T(t)^K$$

where A (ug/mL) is a scaling factor that determines the magnitude of the relationship between total concentration and unbound concentration (it represents the baseline level of unbound concentration when the total concentration is equal to 1), and K (unitless) is the exponent that describes the shape of the relationship between total and unbound concentration. The value of K influences how quickly unbound concentration changes in response to changes in total concentration.

A model with a saturable quasi-equilibrium protein binding function, in which the maximal binding capacity (B_{max}) and binding dissociation constant (KD) are estimated, was also evaluated, but the most parsimonious model with the lowest AIC was the power relationship (AIC 3544 vs 3653).

The residual error was first modeled using a combined error model, but the additive portion for total concentrations was poorly estimated with high uncertainty. Thus, a proportional RUV model was selected for total concentrations and a combined RUV model was used for unbound concentrations. BQL data from unbound concentrations were modeled using M3. M4 was also tested but resulted in models not converging, unsuccessful minimization procedures, and/or failed attempts to estimate standard errors which resulted in numerical anomalies in the gradient.

Adding BSV on Q_2, Q_3, and K parameters resulted in over-parametrization, including convergence and numerical instability. BSV from all the other PK parameters were estimated using the exponential model and assuming a log-normal distribution. Correlations between BSV terms from each parameter were evaluated based on initial graphical exploration and the best model included correlations between CL, V_1, V_3 and A. Correlations were incorporated into the base model to establish the most stable model possible before introducing covariates. Of note, they were then re-evaluated within the full model.

To summarize, a model with three-compartments, zero-order input and first-order elimination was selected as the base model to describe total plasma PK. Unbound concentrations were modeled as dependent on total concentrations using a power relationship. The popPK model was parametrized with CL, V_1, V_2, V_3, Q_2, Q_3, A, and K. The RUV was modeled using a proportional error model for total concentrations and a combined error model for free concentrations. The BSV included log-normally distributed random effects on CL, V_1, V_2, V_3 and A. Covariance structure was incorporated into the BSV on parameters CL, V_1, V_3 and A.

Parameter estimates from the base model are presented in **Table 6**. All parameters from the base model were well estimated, with acceptable relative standard error (RSE; maximum 39%). Population clearance and V_1 were estimated at 0.063 L/hr and 5.654 L, respectively, with moderate variability (CV% = 30.2% and 26.3%, respectively). Q_2 and Q_3 were estimated at 0.026 L/hr and 0.942 L/hr, respectively. V_2 and V_3 were estimated at 8.978 L and 10.661 L, respectively, with moderate variability (CV% = 33.5% and 38.9%, respectively). A was estimated at 0.00135 ug/mL with a moderate variability (CV% = 38.3%) and K at 1.324, showing that the unbound vs total concentrations relationship exhibits a curvature. Strongest correlations were estimated between V_1 and V_3 (73.3%) and between CL and V_1 (66.2%). All eta and epsilon shrinkage were acceptable (maximum 33.1% for BSV on V_2).

Diagnostic plots for the selected structural model suggest that it describes the total and unbound PK data adequately (**Appendix B**). The observed concentration vs. model prediction (population and individual) plots demonstrated a reasonable agreement between data and model predictions. The residual plots did not show any strong pattern with either time or concentration, and closely resembles a normal distribution. A trend can be seen in the residuals of unbound concentrations at later time points, but this concerned only few BQL observations.

Covariate Model

Each pre-specified covariate-parameter relationship was incorporated into the base model to conduct univariate analysis (one model per covariate-parameter relationship). Key results, including objective function value (OFV), p-values from LRT and BSV for each parameter, are presented in **Appendix C**. Numerous covariate-parameter relationships were found to be statistically significant (LRT $p<0.05$). The most pronounced effects (reflected by a decrease in OFV from the base model) were observed for: CrCL on CL, age on V_3, creatinine on CL and age on CL. The modeling process encountered convergence issues when incorporating time-varying albumin; therefore, baseline albumin (ALBBL) was examined, resulting in more stable models. All continuous covariates were initially assessed using a power model. Linear covariate-parameter relationships were also considered for relevant covariates but did not yield any improvement in model fit.

Due to the observed correlation between CrCL and creatinine, only CrCL-parameter relationships were evaluated for the full model (inclusion of baseline serum creatinine and body weight separately proved to be less explanatory in univariate relationships and with no significant improvement over CrCL alone in forward selection with less parsimony). Among all the body size covariates, exhibiting correlation, baseline body weight was exclusively evaluated, as it showed the strongest effect on V1 and the largest reduction in its BSV.

Selected models from each step, including OFV or AIC, p-values from LRT and BSV for each parameter, are presented in **Table 7**. In this initial forward inclusion process, 10 model parameters were added to the base model. Following completion of the forward selection, RUV, BSV and covariance structure were reassessed. No changes were needed in the RUV and BSV models. However, exclusion of A from the correlation matrix resulted in a more parsimonious model based on a reduction in AIC. Remaining trends were noted between weight and CL and A, as well as between baseline albumin and CL, V_1 and V_3. These effects were re-evaluated, contributing two additional parameters to the model. The following covariate-parameter relationships were included in the full model:

- Covariates on CL: CrCL, RRT and baseline body weight (WTBL)
- Covariates on V_1: WTBL
- Covariates on V_2: WTBL, ALBBL and RRT
- Covariates on V_3: age and WTBL
- Covariates on A: ALBBL, sex and WTBL

Selected functional forms of the above covariates were all power models for continuous variables (CrCL, WTBL, ALBBL, age) and exponential shift models for categorical variables (RRT, sex). All IIV models were exponential, but A necessitated logit transformation in order to maintain the constraint of the parameter between 0 and 1. Parameter estimates and diagnostic plots from the full model are presented in (**Appendix C**). All parameters from the base model were well estimated, with acceptable RSE and consistent with the base model. Only the sex effect on A showed a high RSE (64.5%). Compared to the base model, reductions were observed in all BSV (CV%):

- BSV on CL was reduced from 30.2% to 19.7%
- BSV on V1 was reduced from 26.3% to 19.7%
- BSV on V2 was reduced from 33.5% to 27.9%
- BSV on V3 was reduced from 38.9% to 29%
- BSV on A was reduced from 38.3% to 29.8%

The condition number was found to be 117, indicating that the full popPK model was stable (as the value is < 1000). Diagnostic plots did not reveal anything of actionable concern pertaining to the model structure or any yet untested apparent covariate trends.

The results of the backward elimination process, including sequential changes in OFV values from the full to the final models, are summarized in **Table 8**. The analysis resulted in the exclusion of the following covariates: renal replacement therapy effect on CL, V_1 and V_2; the effect of sex on A; and the effect of baseline body weight on CL and A.

Final Model Evaluation

The final popPK model retained the following covariate effects:

- CrCL on CL
- Baseline weight (WTBL) on V_1, V_2 and V*3*
- Baseline albumin (ALBBL) on V_2 and A
- Age on V_3

Parameter estimates from the final popPK model are presented in **Table 6**. The model is characterized by the following functional forms, which include numerical values of typical value parameters, covariate reference values, and covariate effect estimates, as well as symbolic representation of IIV:

$$CL = 0.0658 \cdot (CrCL/101.26)^{0.214} \cdot \exp\,(\eta_{CLV1V3[1]})$$

$$V1 = 5.67 \cdot (WTBL/83.8)^{0.57} \cdot \exp\,(\eta_{CLV1V\ \ [2]})$$

$$V2 = 8.91 \cdot (WTBL/83.8)^{0.82} \cdot (ALBBL/2.8)^{-0.806} \cdot \exp\,(\eta_{V2})$$

$$V3 = 11.1 \cdot (Age/56)^{0.628} \cdot (WTBL/83.8)^{0.559} \cdot \exp\,(\eta_{CLV1V3[3]})$$

$$A = logistic(logit(0.00136 \cdot (ALBBL/2.8)^{-0.782}) + \exp(\eta_A))$$

$$Q2 = 0.0259\ ; Q3 = 0.921\ ; K = 1.32$$

All parameters from the final popPK model were well estimated, with acceptable RSE. Population clearance and central volume of distribution were estimated at 0.0658 L/hr and 5.67 L, respectively, with moderate variability (CV% = 22.6% and 19.7%, respectively, reduced from 30.2% and 26.4% in the base model). Q_2 and Q_3 were estimated at 0.0259 L/hr and 0.921 L/hr, respectively. V_2 and V_3 were estimated at 8.91 L and 11.1 L, respectively, with moderate variability (CV% = 30% and 29.3%, respectively, reduced from 33.5% and 38.9% in the base model). A was estimated at 0.00136 ug/mL with a moderate variability (CV% = 32.6%, reduced from 38.3% in the base model) and k at 1.32, indicating a curvature in the relationship between unbound and total concentrations. All eta and epsilon shrinkage were acceptable. The condition number was found to be 60, indicating that the final popPK model was stable (as the value is < 1000).

Diagnostic plots confirmed that the final popPK model described the total and unbound PK data adequately. The plots of observed concentration vs. population (**Figure 13**) and individual (**Figure 14**) model predictions demonstrated a reasonable agreement between data and model. The distributions appeared to be reasonably normal for both conditional (**Figure 15**) and individual (**Figure 16**) weighted residuals of total and unbound dalbavancin concentrations. There is no strong temporal pattern show by plots against conditional (**Figure 17**) and individual (**Figure 18**) weighted residuals of total concentrations. Although a slight trend in the residuals of unbound concentrations at later time points was observed, it only pertained to a few points. Diagnostic plots also revealed no clear association of concentration with CWRES (**Figure 19**), individual weighted residuals (IWRES) (**Figure 20**), or |IWRES| (**Figure 21**). Population and individual predicted profiles of each subject with observed data are shown in **Appendix D** for total and unbound concentrations and reveal nothing extraordinary. The prediction-corrected Visual Predictive Checks (pcVPC) showed good agreement between observed and simulated total (**Figure 22**) and unbound (**Figure 23**) dalbavancin PK, as well as for the BQL data from unbound concentrations. NPDE plots show a reasonable alignment with a normal distribution (**Appendix E**). Additionally, individual model predicted popPK parameters were plotted against significant covariates from the final popPK model in **Figure 24**.

Exposure Estimates

Individual Empirical Bayes Estimates (EBEs) of PK parameters were obtained from the final popPK model for each patient within the analysis population. Individual PK parameters and exposures were summarized by dose group in **Table 9**.

REFERENCES

1. Turner NA, Xu A, Zaharoff S, Holland TL, Lodise TP. Determination of plasma protein binding of dalbavancin. *J Antimicrob Chemother.* 2022;77(7):1899-1902.
2. Cockcroft DW, Gault MH. Prediction of creatinine clearance from serum creatinine. *Nephron.* 1976;16(1):31-41.
3. Beal SL. Ways to fit a PK model with some data below the quantification limit. *J Pharmacokinet Pharmacodyn.* 2001;28(5):481-504.
4. Population Pharmacokinetics Guidance for Industry. U.S. Department of Health and Human Services Food and Drug Administration Center for Drug Evaluation and Research (CDER) Center for Biologics Evaluation and Research (CBER). February 2022. https://www.fda.gov/regulatory-information/search-fda-guidance-documents/population-pharmacokinetics
5. Carrothers TJ, Chittenden JT, Critchley I. Dalbavancin Population Pharmacokinetic Modeling and Target Attainment Analysis. *Clin Pharmacol Drug Dev.* 2020;9(1):21-31.
6. Ribbing J, Jonsson EN. Power, selection bias and predictive performance of the Population Pharmacokinetic Covariate Model. *J Pharmacokinet Pharmacodyn.* 2004;31(2):109-134.
7. Bergstrand M, Hooker AC, Wallin JE, Karlsson MO. Prediction-corrected visual predictive checks for diagnosing nonlinear mixed-effects models. *AAPS J.* 2011;13(2):143-151.

LIST OF TABLES

Table 1: Subject and PK Sample Disposition for the Dalbavancin Treatment Group

	All Timepoints			Post-Dose Timepoints			Post-Dose Concentrations				
	Subjects	PK Samples		Subjects	PK Samples		All	Total Dalbavancin		Unbound Dalbavancin	
Disposition	N	n	n per Subject	N	n	n per Subject	n	n	BQL	n	BQL
**Target design**	100	900	9.0 (9 - 9)	100	800	8.0 (8 - 8)	1600	800	--	800	--
**Randomized to dalbavancin**	100	--	--	--	--	--	--	--	--	--	--
**Missed PK visits**	20	40	2.0 (1 - 4)	20	40	2.0 (1 - 4)	--	--	--	--	--
Discontinued before visit	15	34	2.3 (1 - 4)	15	34	2.3 (1 - 4)	--	--	--	--	--
PK visit not completed for other reason while still enrolled	5	6	1.2 (1 - 2)	5	6	1.2 (1 - 2)	--	--	--	--	--
**Missed sample draw at PK visit**	73	215	2.9 (1 - 9)	73	201	2.8 (1 - 8)	--	--	--	--	--
Subject illness or injury[a]	2	2	1.0 (1 - 1)	2	2	1.0 (1 - 1)	--	--	--	--	--
Subject refusal[a]	2	2	1.0 (1 - 1)	2	2	1.0 (1 - 1)	--	--	--	--	--
Scheduling difficulties[a]	25	70	2.8 (1 - 5)	24	65	2.7 (1 - 4)	--	--	--	--	--
Site decision/error[a]	5	8	1.6 (1 - 3)	5	8	1.6 (1 - 3)	--	--	--	--	--
Other[a]	12	31	2.6 (1 - 4)	12	31	2.6 (1 - 4)	--	--	--	--	--
Unknown[b]	51	102	2.0 (1 - 7)	50	93	1.9 (1 - 6)	--	--	--	--	--
**Subjects with at least one PK draw**	98	645	6.6 (1 - 9)	97	559	5.8 (1 - 8)	1118	559	2	559	115
**Subjects with at least one measurable post-dose PK concentration[c]**	97	644	6.6 (1 - 9)	97	559	5.8 (1 - 8)	1118	559	2	559	115
**Excluding pairs of pre- and post- dose samples that were suspected to be swapped[d]**	97	640	6.6 (1 - 9)	97	557	5.7 (1 - 8)	1114	557	--	557	113
**Excluding samples with outlying post-dose concentrations (unbound and total) and all pre-dose samples[e]**	97	557	5.7 (1 - 8)	97	557	5.7 (1 - 8)	1114	557	--	557	113
**Excluding single non-BQL unbound concentration at Day 70[f]**	97	555	5.7 (1 - 8)	97	555	5.7 (1 - 8)	1109	555	--	554	112

Notes: N = Number of subjects satisfying the given criterion or with at least one PK sample satisfying the given criterion.
n = Number of PK samples satisfying the given criterion.
n per subject = Number of PK samples per subject within the given criterion, expressed as mean (range).
BQL = Number (percentage) of BQL samples among the number of samples within the given criterion.
[a] Reasons for PK sample not drawn only collected for Visits 02A-02E, i.e. pre-dose through 24h post-dose.
[b] For Visits 03-06 (Day 8, 22, 42, 70) reason for PK sample not drawn was not collected.
[c] Defines subjects in the PK Population. These are the basis of subject-level summary analyses.
[d] Samples used in exploratory analyses of PK concentrations.
[e] Samples used in NCA.
[f] Samples used in PopPK modeling.

Table 2: Summary of Categorical Demographic and Baseline Characteristics by Dalbavancin Dosage Group - PK Population

		Dalbavancin 1500 mg + 1500 mg (N=86)		Dalbavancin 1500 mg + 1125 mg (N=2)		Dalbavancin 1125 mg + 1500 mg (N=3)		Dalbavancin 1125 mg + 1125 mg (N=3)		Dalbavancin 1500 mg only (N=3)		All Dalbavancin Subjects (N=97)	
**Variable**	**Characteristic**	**n**	**%**	**n**	**%**	**n**	**%**	**n**	**%**	**n**	**%**	**n**	**%**
Sex	Male	63	73	1	50	2	67	-	-	3	100	69	71
	Female	23	27	1	50	1	33	3	100	-	-	28	29
Ethnicity	Not Hispanic or Latino	74	86	2	100	3	100	2	67	1	33	82	85
	Hispanic or Latino	9	10	-	-	-	-	1	33	1	33	11	11
	Not Reported	2	2	-	-	-	-	-	-	-	-	2	2
	Unknown	1	1	-	-	-	-	-	-	1	33	2	2
Race	American Indian or Alaska Native	1	1	-	-	-	-	-	-	-	-	1	1
	Asian	5	6	-	-	-	-	-	-	-	-	5	5
	Native Hawaiian or Other Pacific Islander	-	-	-	-	-	-	-	-	-	-	-	-
	Black or African American	17	20	1	50	1	33	1	33	-	-	20	21
	White	60	70	1	50	2	67	2	67	2	67	67	69
	Multi-Racial	-	-	-	-	-	-	-	-	-	-	-	-
	Unknown	3	3	-	-	-	-	-	-	1	33	4	4
Renal Replacement Therapy	Yes	9	10	-	-	-	-	2	67	1	33	12	12
	No	77	90	2	100	3	100	1	33	2	67	85	88

Notes: N = Number of subjects in the PK Population in the respective dalbavancin dose group.
n = Number of subjects with non-missing values for the corresponding baseline characteristic.

Table 3: Summary of Continuous Demographic and Baseline Characteristics by Dalbavancin Dosage Group - PK Population

Variable	Statistic	Dalbavancin 1500 mg + 1500 mg (N=86)	Dalbavancin 1500 mg + 1125 mg (N=2)	Dalbavancin 1125 mg + 1500 mg (N=3)	Dalbavancin 1125 mg + 1125 mg (N=3)	Dalbavancin 1500 mg only (N=3)	All Dalbavancin Subjects (N=97)
Age (years)	n	86	2	3	3	3	97
	Mean	53.8	55.5	70.3	53.3	59.7	54.5
	Standard Deviation	15.7	24.7	10.8	25.0	4.6	15.8
	Median	55.5	55.5	75.0	52.0	57.0	56.0
	Minimum	20	38	58	29	57	20
	Maximum	83	73	78	79	65	83
BMI (kg/m^2)	n	86	2	3	3	3	97
	Mean	29.60	20.70	28.92	21.08	26.24	29.03
	Standard Deviation	6.84	1.04	3.99	0.60	8.13	6.86
	Median	28.42	20.70	31.01	21.42	29.72	28.02
	Minimum	17.2	20.0	24.3	20.4	16.9	16.9
	Maximum	50.2	21.4	31.4	21.4	32.1	50.2
Height (cm)	n	86	2	3	3	3	97
	Mean	173.40	181.65	175.27	163.87	173.53	173.34
	Standard Deviation	9.41	1.91	10.15	16.50	7.74	9.58
	Median	172.70	181.65	175.30	155.00	175.20	172.70
	Minimum	152.4	180.3	165.1	153.7	165.1	152.4
	Maximum	193.0	183.0	185.4	182.9	180.3	193.0
Weight (kg)	n	86	2	3	3	3	97
	Mean	89.15	68.35	89.83	57.10	79.07	87.44
	Standard Deviation	21.94	4.88	20.98	12.67	26.15	22.24
	Median	85.35	68.35	96.60	50.60	81.00	83.80
	Minimum	48.5	64.9	66.3	49.0	52.0	48.5
	Maximum	151.5	71.8	106.6	71.7	104.2	151.5
LBW (kg)	n	86	2	3	3	3	97
	Mean	59.72	51.46	60.70	37.98	58.32	58.86
	Standard Deviation	11.75	10.45	16.81	8.19	12.22	12.22

Variable	Statistic	Dalbavancin 1500 mg + 1500 mg (N=86)	Dalbavancin 1500 mg + 1125 mg (N=2)	Dalbavancin 1125 mg + 1500 mg (N=3)	Dalbavancin 1125 mg + 1125 mg (N=3)	Dalbavancin 1500 mg only (N=3)	All Dalbavancin Subjects (N=97)
	Median	60.37	51.46	66.48	33.49	57.32	59.35
	Minimum	32.8	44.1	41.8	33.0	46.6	32.8
	Maximum	86.7	58.8	73.9	47.4	71.0	86.7
IBW (kg)	n	86	2	3	3	3	97
	Mean	67.91	74.37	69.31	55.93	69.23	67.76
	Standard Deviation	9.82	4.92	11.56	15.01	7.04	9.98
	Median	68.47	74.37	70.84	47.87	70.75	68.47
	Minimum	45.5	70.9	57.1	46.7	61.6	45.5
	Maximum	86.9	77.8	80.0	73.3	75.4	86.9
BSA (m^2)	n	86	2	3	3	3	97
	Mean	2.08	1.86	2.10	1.62	1.95	2.06
	Standard Deviation	0.28	0.08	0.31	0.25	0.36	0.29
	Median	2.06	1.86	2.19	1.49	1.95	2.03
	Minimum	1.5	1.8	1.8	1.5	1.6	1.5
	Maximum	2.8	1.9	2.4	1.9	2.3	2.8
Baseline Albumin (g/dL)[a]	n	86	2	3	3	2	96
	Mean	2.82	3.35	2.53	2.53	2.50	2.80
	Standard Deviation	0.61	0.49	0.71	0.74	0.28	0.61
	Median	2.80	3.35	2.40	2.80	2.50	2.80
	Minimum	1.2	3.0	1.9	1.7	2.3	1.2
	Maximum	4.1	3.7	3.3	3.1	2.7	4.1
Baseline Creatinine (mg/dL)[a]	n	86	2	3	3	3	97
	Mean	1.380	2.040	2.977	5.753	1.509	1.582
	Standard Deviation	1.900	0.085	1.442	3.427	0.264	2.033
	Median	0.810	2.040	3.300	6.900	1.610	0.850
	Minimum	0.39	1.98	1.40	1.90	1.21	0.39
	Maximum	11.51	2.10	4.23	8.46	1.71	11.51
	n	86	2	3	3	3	97

Variable	Statistic	Dalbavancin 1500 mg + 1500 mg (N=86)	Dalbavancin 1500 mg + 1125 mg (N=2)	Dalbavancin 1125 mg + 1500 mg (N=3)	Dalbavancin 1125 mg + 1125 mg (N=3)	Dalbavancin 1500 mg only (N=3)	All Dalbavancin Subjects (N=97)
Baseline Creatinine Clearance (mL/min)[a]	Mean	121.980	35.643	29.945	15.152	59.812	112.127
	Standard Deviation	69.059	5.412	4.380	11.823	21.543	70.960
	Median	118.941	35.643	29.162	13.617	69.731	101.263
	Minimum	7.62	31.82	26.01	4.17	35.10	4.17
	Maximum	371.99	39.47	34.66	27.67	74.61	371.99

Notes: N = Number of subjects in the PK Population in the respective dalbavancin dose group.
n = Number of subjects with non-missing values for the corresponding baseline characteristic.
[a]Values at baseline may be imputed using the last observation carried forward or first observation carried backward.

Table 4: Summary Statistics of Total and Unbound Plasma Concentrations (µg/mL) by Dalbavancin Dosage Group and Nominal Time - PK Population

	Dalbavancin Plasma Concentration (µg/mL)					
**Nominal Time[a]**	**Dalbavancin 1500 mg + 1500 mg (N=86)**	**Dalbavancin 1500 mg + 1125 mg (N=2)**	**Dalbavancin 1125 mg + 1500 mg (N=3)**	**Dalbavancin 1125 mg + 1125 mg (N=3)**	**Dalbavancin 1500 mg only (N=3)**	**All Dalbavancin Subjects (N=97)**
**Total Plasma Dalbavancin Concentrations (µg/mL)**						
**Day 1 – Pre 1st IV Admin**						
n	74	2	2	2	3	83
Mean (SD)	0.000 (0.000)	0.000 (0.000)	0.000 (0.000)	0.000 (0.000)	0.000 (0.000)	0.000 (0.000)
Geometric Mean (GSD)	N/A	N/A	N/A	N/A	N/A	N/A
Median	0.00	0.00	0.00	0.00	0.00	0.00
Min - Max	0.00 - 0.00	0.00 - 0.00	0.00 - 0.00	0.00 - 0.00	0.00 - 0.00	0.00 - 0.00
**Day 1 – End of 1st Infusion**						
n	63	2	1	1	3	70
Mean (SD)	256.449 (60.476)	285.186 (83.727)	175.415 (N/A)	295.787 (N/A)	241.795 (100.046)	256.047 (61.877)
Geometric Mean (GSD)	249.849 (1.256)	278.973 (1.347)	175.415 (N/A)	295.787 (N/A)	226.693 (1.574)	248.937 (1.269)
Median	247.84	285.19	175.42	295.79	248.48	248.16
Min - Max	165.78 - 404.96	225.98 - 344.39	175.42 - 175.42	295.79 - 295.79	138.57 - 338.33	138.57 - 404.96
**Day 1 - 6 h**						
n	60	2	2	3	2	69
Mean (SD)	128.125 (32.686)	120.030 (38.327)	91.241 (7.818)	131.279 (37.807)	83.202 (11.949)	125.656 (33.003)
Geometric Mean (GSD)	124.088 (1.293)	116.930 (1.384)	91.073 (1.090)	127.329 (1.364)	82.772 (1.155)	121.472 (1.301)
Median	122.07	120.03	91.24	138.93	83.20	120.01
Min - Max	57.57 - 207.06	92.93 - 147.13	85.71 - 96.77	90.23 - 164.68	74.75 - 91.65	57.57 - 207.06
**Day 1 - 12 h**						
n	51	2	3	2	2	60
Mean (SD)	95.281 (27.574)	103.148 (48.551)	62.228 (25.508)	102.062 (3.384)	58.163 (2.578)	92.880 (28.403)

	Dalbavancin Plasma Concentration (µg/mL)					
**Nominal Time[a]**	**Dalbavancin 1500 mg + 1500 mg (N=86)**	**Dalbavancin 1500 mg + 1125 mg (N=2)**	**Dalbavancin 1125 mg + 1500 mg (N=3)**	**Dalbavancin 1125 mg + 1125 mg (N=3)**	**Dalbavancin 1500 mg only (N=3)**	**All Dalbavancin Subjects (N=97)**
Geometric Mean (GSD)	90.430 (1.440)	97.267 (1.631)	57.920 (1.634)	102.033 (1.034)	58.134 (1.045)	87.709 (1.457)
Median	92.21	103.15	74.68	102.06	58.16	91.15
Min - Max	14.60 - 171.18	68.82 - 137.48	32.89 - 79.12	99.67 - 104.45	56.34 - 59.99	14.60 - 171.18
**Day 1 - 24 h**						
n	59	2	3	2	2	68
Mean (SD)	80.008 (21.296)	82.447 (38.211)	55.488 (19.911)	76.873 (6.407)	52.908 (1.664)	78.109 (21.738)
Geometric Mean (GSD)	77.323 (1.302)	77.894 (1.618)	52.665 (1.513)	76.739 (1.087)	52.894 (1.032)	75.179 (1.325)
Median	77.04	82.45	64.08	76.87	52.91	74.58
Min - Max	38.72 - 145.18	55.43 - 109.47	32.72 - 69.66	72.34 - 81.40	51.73 - 54.08	32.72 - 145.18
**Day 8 – Pre 2nd IV Admin**						
n	80	2	3	3	1	89
Mean (SD)	35.792 (11.001)	51.114 (15.436)	25.455 (15.966)	43.352 (5.834)	39.368 (N/A)	36.083 (11.353)
Geometric Mean (GSD)	34.167 (1.362)	49.935 (1.359)	22.475 (1.822)	43.076 (1.151)	39.368 (N/A)	34.297 (1.386)
Median	35.35	51.11	19.21	45.89	39.37	36.40
Min - Max	16.93 - 66.51	40.20 - 62.03	13.56 - 43.60	36.68 - 47.49	39.37 - 39.37	13.56 - 66.51
**Day 22**						
n	70	2	2	1	-	75
Mean (SD)	40.978 (44.145)	54.542 (3.647)	47.668 (16.422)	26.438 (N/A)	-	41.325 (42.777)
Geometric Mean (GSD)	34.365 (1.651)	54.481 (1.069)	46.232 (1.421)	26.438 (N/A)	-	34.944 (1.640)
Median	33.27	54.54	47.67	26.44	-	34.65
Min - Max	13.62 - 384.36	51.96 - 57.12	36.06 - 59.28	26.44 - 26.44	-	13.62 - 384.36
**Day 42**						
n	68	2	3	1	-	74
Mean (SD)	10.188 (5.176)	23.947 (2.442)	11.994 (6.434)	6.982 (N/A)	-	10.590 (5.577)

Nominal Time[a]	Dalbavancin Plasma Concentration (µg/mL)					
	Dalbavancin 1500 mg + 1500 mg (N=86)	Dalbavancin 1500 mg + 1125 mg (N=2)	Dalbavancin 1125 mg + 1500 mg (N=3)	Dalbavancin 1125 mg + 1125 mg (N=3)	Dalbavancin 1500 mg only (N=3)	All Dalbavancin Subjects (N=97)
Geometric Mean (GSD)	8.981 (1.675)	23.884 (1.108)	10.533 (1.955)	6.982 (N/A)	-	9.250 (1.704)
Median	9.43	23.95	13.54	6.98	-	9.54
Min - Max	2.80 - 25.89	22.22 - 25.67	4.93 - 17.51	6.98 - 6.98	-	2.80 - 25.89
**Day 70**						
n	49	1	2	-	-	52
Mean (SD)	4.351 (2.139)	9.596 (N/A)	5.951 (5.435)	-	-	4.514 (2.344)
Geometric Mean (GSD)	3.815 (1.721)	9.596 (N/A)	4.544 (2.963)	-	-	3.910 (1.757)
Median	3.73	9.60	5.95	-	-	3.86
Min - Max	0.98 - 9.78	9.60 - 9.60	2.11 - 9.79	-	-	0.98 - 9.79
**Unbound Plasma Dalbavancin Concentrations (µg/mL)**						
**Day 1 – Pre 1st IV Admin**						
n	74	2	2	2	3	83
Mean (SD)	0.000 (0.000)	0.000 (0.000)	0.000 (0.000)	0.000 (0.000)	0.000 (0.000)	0.000 (0.000)
Geometric Mean (GSD)	N/A	N/A	N/A	N/A	N/A	N/A
Median	0.00	0.00	0.00	0.00	0.00	0.00
Min - Max	0.00 - 0.00	0.00 - 0.00	0.00 - 0.00	0.00 - 0.00	0.00 - 0.00	0.00 - 0.00
**Day 1 – End of 1st Infusion**						
n	63	2	1	1	3	70
Mean (SD)	1.969 (0.772)	1.267 (0.177)	1.409 (N/A)	1.851 (N/A)	1.768 (1.738)	1.931 (0.802)
Geometric Mean (GSD)	1.823 (1.499)	1.260 (1.151)	1.409 (N/A)	1.851 (N/A)	1.302 (2.514)	1.772 (1.530)
Median	1.84	1.27	1.41	1.85	0.77	1.81
Min - Max	0.73 - 4.32	1.14 - 1.39	1.41 - 1.41	1.85 - 1.85	0.76 - 3.78	0.73 - 4.32
**Day 1 - 6 h**						
n	60	2	2	3	2	69

	Dalbavancin Plasma Concentration (µg/mL)					
**Nominal Time[a]**	**Dalbavancin 1500 mg + 1500 mg (N=86)**	**Dalbavancin 1500 mg + 1125 mg (N=2)**	**Dalbavancin 1125 mg + 1500 mg (N=3)**	**Dalbavancin 1125 mg + 1125 mg (N=3)**	**Dalbavancin 1500 mg only (N=3)**	**All Dalbavancin Subjects (N=97)**
Mean (SD)	0.896 (0.431)	0.385 (0.075)	0.638 (0.039)	0.913 (0.620)	0.306 (0.138)	0.858 (0.437)
Geometric Mean (GSD)	0.821 (1.507)	0.381 (1.216)	0.637 (1.063)	0.783 (1.971)	0.290 (1.596)	0.772 (1.583)
Median	0.84	0.39	0.64	0.71	0.31	0.77
Min - Max	0.33 - 3.08	0.33 - 0.44	0.61 - 0.67	0.42 - 1.61	0.21 - 0.40	0.21 - 3.08
**Day 1 - 12 h**						
n	50	2	3	2	2	59
Mean (SD)	0.651 (0.325)	0.309 (0.097)	0.482 (0.136)	0.504 (0.040)	0.336 (0.222)	0.615 (0.315)
Geometric Mean (GSD)	0.585 (1.603)	0.301 (1.376)	0.467 (1.366)	0.503 (1.082)	0.297 (2.047)	0.550 (1.620)
Median	0.57	0.31	0.54	0.50	0.34	0.55
Min - Max	0.18 - 2.02	0.24 - 0.38	0.33 - 0.58	0.48 - 0.53	0.18 - 0.49	0.18 - 2.02
**Day 1 - 24 h**						
n	59	2	3	2	2	68
Mean (SD)	0.468 (0.193)	0.248 (0.013)	0.442 (0.164)	0.381 (0.187)	0.233 (0.111)	0.451 (0.192)
Geometric Mean (GSD)	0.431 (1.510)	0.248 (1.053)	0.418 (1.520)	0.357 (1.667)	0.219 (1.644)	0.413 (1.532)
Median	0.45	0.25	0.48	0.38	0.23	0.41
Min - Max	0.16 - 1.12	0.24 - 0.26	0.26 - 0.58	0.25 - 0.51	0.15 - 0.31	0.15 - 1.12
**Day 8 – Pre 2nd IV Admin**						
n	78	2	3	3	1	87
Mean (SD)	0.155 (0.047)	0.120 (0.025)	0.164 (0.016)	0.208 (0.067)	0.123 (N/A)	0.156 (0.047)
Geometric Mean (GSD)	0.148 (1.377)	0.118 (1.232)	0.163 (1.102)	0.200 (1.387)	0.123 (N/A)	0.149 (1.371)
Median	0.16	0.12	0.17	0.20	0.12	0.15
Min - Max	0.06 - 0.28	0.10 - 0.14	0.15 - 0.18	0.14 - 0.28	0.12 - 0.12	0.06 - 0.28

	Dalbavancin Plasma Concentration (μg/mL)					
**Nominal Time[a]**	**Dalbavancin 1500 mg + 1500 mg (N=86)**	**Dalbavancin 1500 mg + 1125 mg (N=2)**	**Dalbavancin 1125 mg + 1500 mg (N=3)**	**Dalbavancin 1125 mg + 1125 mg (N=3)**	**Dalbavancin 1500 mg only (N=3)**	**All Dalbavancin Subjects (N=97)**
**Day 22**						
n	65	2	2	1	-	70
Mean (SD)	0.184 (0.267)	0.146 (0.001)	0.247 (0.094)	0.066 (N/A)	-	0.183 (0.258)
Geometric Mean (GSD)	0.144 (1.760)	0.145 (1.005)	0.237 (1.479)	0.066 (N/A)	-	0.145 (1.753)
Median	0.14	0.15	0.25	0.07	-	0.14
Min - Max	0.06 - 2.23	0.15 - 0.15	0.18 - 0.31	0.07 - 0.07	-	0.06 - 2.23
**Day 42**						
n	16	2	2	-	-	20
Mean (SD)	0.063 (0.010)	0.072 (0.003)	0.077 (0.030)	-	-	0.066 (0.012)
Geometric Mean (GSD)	0.063 (1.163)	0.072 (1.040)	0.074 (1.485)	-	-	0.065 (1.190)
Median	0.06	0.07	0.08	-	-	0.07
Min - Max	0.05 - 0.08	0.07 - 0.07	0.06 - 0.10	-	-	0.05 - 0.10
**Day 70**						
n	1	-	-	-	-	1
Mean (SD)	0.054 (N/A)	-	-	-	-	0.054 (N/A)
Geometric Mean (GSD)	0.054 (N/A)	-	-	-	-	0.054 (N/A)
Median	0.05	-	-	-	-	0.05
Min - Max	0.05 - 0.05	-	-	-	-	0.05 - 0.05

Notes: For calculation of summary statistics, BQL values were imputed as 0 if the sample was taken before the first measurable PK sample with a concentration above the LLOQ. BQL values were treated as missing otherwise.
N = Number of subjects in the PK Population in the respective dalbavancin dose group.
n = Number of data points used to compute the summary statistics.
[a]Times are relative to the end of the first infusion.

Table 5: Summary of Pop-PK Analysis Models Considered

Model	Description	Reference Model	# Parameters	AIC	AIC of Reference Model	Change in AIC	Comments
Base10	Initial base model	NA	16	3875.833	NA	NA	Under-predictions of high free concentrations
Base11	2 compartments	Base10	14	4110.121	3875.833	234.288	Total and free concentrations not properly described
Base12	Exponential relationship Free vs Total	Base10	17	3879.777	3875.833	3.944	Under-predictions of high free concentrations
**Base13**	**Power relationship Free vs Total**	**Base10**	**17**	**3544.523**	**3875.833**	**-331.310**	**Ok. High RSE additive RUV**
Base14	Logarithm relationship Free vs Total	Base10	16	5149.378	3875.833	1273.545	Under-predictions of high free concentrations
Base15	Sigmoidal relationship Free vs Total	Base10	19	3545.904	3875.833	-329.929	Overparameterized
Base17	Protein binding relationship Free vs Total	Base10	17	3652.869	3875.833	-222.964	Ok
Base100	BLOQ M4 method	Base13	17	3544.483	3544.523	-0.040	Numerical and convergence issues.
**Base101**	**Proportional RUV for Total**	**Base13**	**16**	**3543.608**	**3544.523**	**-0.915**	**Ok**
Base102	Proportional RUV for Free	Base101	15	3550.885	3543.608	7.277	High epsilon shrinkage
Base103	Add BSV on Q2	Base101	17	3533.397	3543.608	-10.211	Overparameterized
Base104	Add BSV on Q3	Base101	17	3705.424	3543.608	161.816	Overparameterized
Base105	Add BSV on K	Base101	17	3684.750	3543.608	141.142	Overparameterized
**Base106**	**Corr V1-V3**	**Base101**	**17**	**3520.932**	**3543.608**	**-22.676**	**Ok**
**Base107**	**Corr CL-V1-V3**	**Base106**	**19**	**3493.786**	**3520.932**	**-27.146**	**Ok**
Base108	Corr CL-V1-V2-V3	Base107	22	3517.278	3493.786	23.492	Overparameterized
Base109	Corr V1-V3 and CL-V2	Base106	18	3511.240	3520.932	-9.692	Ok
**Base110**	**Corr CL-V1-V3-A**	**Base107**	**22**	**3478.518**	**3493.786**	**-15.268**	**Best base model**
Base111	Corr CL-V1-V2-V3-A	Base110	26	3520.499	3478.518	41.981	Overparameterized

Note: Bolded rows represent either the final selected base model or models that were selected as the best intermediate structures which are used as reference models for subsequently tested models.

Table 6: Estimation Summary of Plasma Pop-PK Parameters Estimated in the Base Structural Model and the Final Covariate Model

Parameter	Description	Estimate	RSE (%)	95% CI	BSV (CV%)	Corr (%)	Shrinkage (%)
**Base Model**							
CL	Clearance (L/hr)	0.063	3.2	[0.0591; 0.067]	-	-	-
V1	Volume of distribution in the central compartment (L)	5.65	3.2	[5.3; 6.01]	-	-	-
Q2	Intercompartmental clearance from the 1st to the 2nd compartment (L/hr)	0.026	9	[0.0214; 0.0305]	-	-	-
V2	Volume of distribution of 2nd compartment (L)	8.98	6.5	[7.84; 10.1]	-	-	-
Q3	Intercompartmental clearance from the 1st to the 3rd compartment (L/hr)	0.942	6.1	[0.829; 1.05]	-	-	-
V3	Volume of distribution of 3rd compartment (L)	10.7	4.4	[9.73; 11.6]	-	-	-
A	Scaling factor (ug/mL)	0.00135	8.9	[0.00111; 0.00158]	-	-	-
k	Exponent of the power function	1.32	1.4	[1.29; 1.36]	-	-	-
$\Omega_{1,1}$	BSV V2	0.112	26.1	[0.0547; 0.17]	33.5	-	33.1
$\Omega\_CLV1V3A_{1,1}$	BSV CL	0.0913	15	[0.0644; 0.118]	30.2	-	3.5
$\Omega\_CLV1V3A_{2,1}$	Covariance CL-V1	0.0528	17.7	[0.0344; 0.0711]	-	66.2	-
$\Omega\_CLV1V3A_{3,1}$	Covariance CL-V3	0.0495	21.4	[0.0288; 0.0703]	-	42.3	-
$\Omega\_CLV1V3A_{4,1}$	Covariance CL-A	0.05	26.7	[0.0238; 0.0762]	-	43.3	-
$\Omega\_CLV1V3A_{2,2}$	BSV V1	0.0695	20.4	[0.0417; 0.0973]	26.3	-	10.4
$\Omega\_CLV1V3A_{3,2}$	Covariance V1-V3	0.0749	18.2	[0.0482; 0.102]	-	73.3	-
$\Omega\_CLV1V3A_{4,2}$	Covariance V1-A	0.042	27.5	[0.0194; 0.0646]	-	41.7	-
$\Omega\_CLV1V3A_{3,3}$	BSV V3	0.151	18.8	[0.0949; 0.206]	38.9	-	8.9
$\Omega\_CLV1V3A_{4,3}$	Covariance V3-A	0.0559	39	[0.0132; 0.0986]	-	37.6	-
$\Omega\_CLV1V3A_{4,4}$	BSV A	0.147	18	[0.0948; 0.198]	38.3	-	5.1
σ_proptot	Proportional Total RUV	0.13	6.7	[0.113; 0.147]	-	-	-
σ_propfree	Proportional Unbound RUV	0.19	6.6	[0.166; 0.215]	-	-	-
σ_addfree	Additive Unbound RUV	0.0126	16.7	[0.00844; 0.0167]	-	-	-
Epsilon shrinkage Total	-	-	-	-	-	-	19.7
Epsilon shrinkage Unbound	-	-	-	-	-	-	9.6
**Final Model**							

Parameter	Description	Estimate	RSE (%)	95% CI	BSV (CV%)	Corr (%)	Shrinkage (%)
CL	Clearance (L/hr)	0.0658	2.6	[0.0624; 0.0692]	-	-	-
V1	Volume of distribution in the central compartment (L)	5.67	2.7	[5.37; 5.99]	-	-	-
Q2	Intercompartmental clearance from the 1st to the 2nd compartment (L/hr)	0.0259	7.9	[0.0224; 0.0302]	-	-	-
V2	Volume of distribution of 2nd compartment (L)	8.91	6.2	[8.04; 10.2]	-	-	-
Q3	Intercompartmental clearance from the 1st to the 3rd compartment (L/hr)	0.921	5.3	[0.83; 1.01]	-	-	-
V3	Volume of distribution of 3rd compartment (L)	11.1	3.8	[10.3; 12]	-	-	-
A	Scaling factor (ug/mL)	0.00136	8.1	[0.00115; 0.00159]	-	-	-
k	Exponent of the power function	1.32	1.4	[1.28; 1.35]	-	-	-
CL~CRCL	CRCL effect on CL	0.214	15.8	[0.16; 0.295]	-	-	-
V1~WTBL	WTBL effect on V1	0.57	21.1	[0.37; 0.855]	-	-	-
V2~WTBL	WTBL effect on V2	0.82	32.2	[0.374; 1.46]	-	-	-
V2~ALBBL	ALBBL effect on V2	-0.806	47.2	[-1.79; -0.318]	-	-	-
V3~AGE	AGE effect on V3	0.628	16.1	[0.44; 0.832]	-	-	-
V3~WTBL	WTBL effect on V3	0.559	24.5	[0.295; 0.816]	-	-	-
A~ALBBL	ALBBL effect on A	-0.782	14.3	[-0.984; -0.539]	-	-	-
Ω_CLV1V3$_{1,1}$	BSV CL	0.0508	14.9	[0.0359; 0.0653]	22.6	-	4.2
Ω_CLV1V3$_{2,2}$	BSV V1	0.0386	29.5	[0.0191; 0.0623]	19.7	-	10.4
$\Omega_{1,1}$	BSV V2	0.0897	27.6	[0.0239; 0.123]	30	-	39.4
Ω_CLV1V3$_{3,3}$	BSV V3	0.0861	16.6	[0.0566; 0.113]	29.3	-	8.6
$\Omega_{2,2}$	BSV A	0.106	21.5	[0.0616; 0.153]	32.6	-	6.8
Ω_CLV1V3$_{2,1}$	Covariance CL-V1	0.0264	29.6	[0.01; 0.0409]	-	59.7	-
Ω_CLV1V3$_{3,1}$	Covariance CL-V3	0.0416	22	[0.0238; 0.0578]	-	63	-
Ω_CLV1V3$_{3,2}$	Covariance V1-V3	0.0478	18.2	[0.0307; 0.0643]	-	82.9	-
σ_proptot	Proportional RUV for Total	0.127	6.1	[0.112; 0.142]	-	-	-
σ_propfree	Proportional RUV for Unbound	0.198	6.2	[0.173; 0.219]	-	-	-
σ_addfree	Additive RUV for Unbound	0.0124	21.9	[0.00521; 0.016]	-	-	-
Epsilon shrinkage Total	-	-	-	-	-	-	18
Epsilon shrinkage Unbound	-	-	-	-	-	-	8.4

Parameter	Description	Estimate	RSE (%)	95% CI	BSV (CV%)	Corr (%)	Shrinkage (%)

Notes: (i) Estimates for BSV are the variances. (ii) Estimates for RUV are the standard deviation. (iii) The CV % was derived as: sqrt(var)*100. (iv) RSE % was derived as: se/estimate*100. (v) CI were generated by bootstrapping the original dataset (1000 replicates). (vi) A typical subject is a patient aged 56 years old, weighing 83.8 kg, with a baseline albumin value of 2.8 g/dL and a creatinine clearance value of 101.26 mL/min. These reference values represent the median values observed within the analysis population.
Abbreviations: ALBBL = baseline albumin; BSV = between-subject variability; CI = confidence interval; CRCL = creatinine clearance; CV = coefficient of variation; RSE = relative standard error; RUV = residual unexplained variability; WTBL = baseline weight.

Table 7: Summary of the Forward Selection Process of Covariates for the Pop-PK Model

Model	Reference Model	Covariate Added	Objective Function (-2LL)/AIC[a]	Change in Objective Function/AIC[a]	P-value	BSV CL	BSV V1	BSV V2	BSV V3	BSV A	Comments
**Initial Forward Selection**											
0	NA	Base	3434.518	NA	NA	0.0913	0.0695	0.1121	0.1506	0.1465	-
1	0	CRCL on CL	3385.755	-48.763	<0.001	0.0577	0.0711	0.1168	0.1489	0.1467	Covariate added
2	1	Age on V3	3345.722	-40.033	<0.001	0.0566	0.0745	0.1071	0.1304	0.1455	Covariate added
3	2	Age on CL	3343.808	-1.914	0.167	0.0550	0.0748	0.1073	0.1299	0.1451	Covariate rejected
4	2	ALB on V2	3327.698	-18.024	<0.001	0.0545	0.0741	0.1088	0.1259	0.1449	Covariate rejected. Unstable model.
5	2	WTBL on V1	3327.860	-17.862	<0.001	0.0548	0.0457	0.1200	0.1243	0.1438	Covariate added
6	5	WTBL on V2	3318.622	-9.238	0.002	0.0538	0.0454	0.1006	0.1218	0.1437	Covariate added
7	6	ALBBL on A	3306.986	-11.636	<0.001	0.0549	0.0470	0.1180	0.1213	0.1113	Covariate added
8	7	WTBL on CL	3306.275	-0.711	0.399	0.0530	0.0457	0.1008	0.1227	0.1129	Covariate rejected
9	7	ALBBL on V2	3293.285	-13.701	<0.001	0.0531	0.0451	0.0825	0.1221	0.1114	Covariate added
10	9	Sex on A	3289.147	-4.138	0.042	0.0538	0.0447	0.0801	0.1217	0.1140	Covariate added
11	10	RRTFL on V2	3283.304	-5.843	0.016	0.0543	0.0463	0.0760	0.1234	0.1122	Covariate added
12	11	WTBL on V3	3273.624	-9.680	0.002	0.0540	0.0429	0.1047	0.0910	0.1121	Covariate added
13	12	Sex on CL	3269.187	-4.437	0.035	0.0506	0.0417	0.0773	0.0906	0.1094	Covariate rejected
14	12	Sex on V3	3270.344	-3.280	0.070	0.0528	0.0418	0.0774	0.0893	0.1102	Covariate rejected
15	12	RRTFL on CL	3264.686	-8.938	0.003	0.0449	0.0420	0.0810	0.0946	0.1115	Covariate added
16	15	Age on V1	3263.336	-1.350	0.245	0.0457	0.0412	0.1102	0.0924	0.1128	Covariate rejected
**Assessment of RUV, BSV, and Correlations**											
Full01	NA	Full model	3328.686	NA	-	-	-	-	-	-	-
Full02	Full01	Combined error Total	3330.909	2.223	-	-	-	-	-	-	-
Full03	Full01	Proportional error Free	3333.175	4.489	-	-	-	-	-	-	-
Full04	Full01	Add BSV on K	3326.460	-2.226	-	-	-	-	-	-	-
Full05	Full01	Correlation CL-V1-V3	3332.338	3.652	-	-	-	-	-	-	-
Full06	Full05	Correlation V-V3	3366.137	33.799	-	-	-	-	-	-	-
Full07	Full05	Correlation CL-V1	3373.735	41.397	-	-	-	-	-	-	-
**Re-Assessment of Remaining Trend with Covariates**											
100	NA	Full05	3274.338	NA	NA	0.0432	0.0395	0.0815	0.0876	0.1029	-

Model	Reference Model	Covariate Added	Objective Function (-2LL)/AIC[a]	Change in Objective Function/AIC[a]	P-value	BSV CL	BSV V1	BSV V2	BSV V3	BSV A	Comments
101	100	WTBL on A	3265.125	-9.213	0.002	0.0428	0.0411	0.0841	0.0881	0.0880	-
102	101	WTBL on CL	3255.315	-9.810	0.002	0.0387	0.0386	0.0785	0.0835	0.0886	-
103	102	ALBBL on CL	3252.234	-3.081	0.079	0.0339	0.0384	0.0777	0.0830	0.0884	-
104	102	ALBBL on V1	3253.616	-1.699	0.192	0.0381	0.0348	0.0774	0.0815	0.0885	-
105	102	ALBBL on V3	3252.873	-2.442	0.118	0.0389	0.0380	0.0772	0.0749	0.0885	-

Abbreviations: ALB = albumin; ALBBL = baseline albumin; BSV = between-subject variability; CRCL = creatinine clearance; RRTFL = renal replacement therapy flag; RUV = residual unexplained variability; WTBL = baseline weight.

[a]For Initial Forward Selection and Re-Assessment of Remaining Trend with Covariates, objective function and change in objective function are presented. For Assessment of RUV, BSV, and Correlations, AIC and change in AIC are presented.

Table 8: Summary of the Backward Elimination Process for Covariates from the Pop-PK Model

Step #	Excluded Covariate-Parameter Relationship from Previous Step	Included Covariate-parameter Relationships for Step-selected Model	LRT p<0.001?	Change in Objective Function (Δ-2LL) from Previous Selected Model
**0 (Full Model)**	**NA**	**CL ~ CRCL, CL ~ RRTFL, CL ~ WTBL, V1 ~ WTBL, V2 ~ WTBL, V2 ~ ALBBL, V2 ~ RRTFL, V3 ~ AGE, V3 ~ WTBL, A ~ ALBBL, A ~ SEX, A ~ WTBL**	**NA**	**NA**
1	CL ~ CRCL	-	TRUE	47.05
1	V1 ~ WTBL	-	TRUE	44.35
1	V3 ~ AGE	-	TRUE	37.54
1	V3 ~ WTBL	-	TRUE	23.99
1	A ~ ALBBL	-	TRUE	23.1
1	V2 ~ ALBBL	-	TRUE	12.05
1	A ~ WTBL	-	FALSE	9.85
1	CL ~ WTBL	-	FALSE	9.81
1	V2 ~ WTBL	-	FALSE	6.12
1	CL ~ RRTFL	-	FALSE	5.67
1	V2 ~ RRTFL		FALSE	4.3
**1**	**A ~ SEX**	**CL ~ CRCL, CL ~ RRTFL, CL ~ WTBL, V1 ~ WTBL, V2 ~ WTBL, V2 ~ ALBBL, V2 ~ RRTFL, V3 ~ AGE, V3 ~ WTBL, A ~ ALBBL, A ~ WTBL**	**FALSE**	**3.72**
2	CL ~ CRCL	-	TRUE	46.98
2	V1 ~ WTBL	-	TRUE	42.53
2	V3 ~ AGE	-	TRUE	37.24
2	A ~ ALBBL	-	TRUE	25.36
2	V3 ~ WTBL	-	TRUE	23.35
2	V2 ~ ALBBL	-	TRUE	11.55
2	CL ~ WTBL	-	FALSE	9.19
2	A ~ WTBL	-	FALSE	7.94
2	V2 ~ WTBL	-	FALSE	5.88
2	CL ~ RRTFL	-	FALSE	5.04
**2**	**V2 ~ RRTFL**	**CL ~ CRCL, CL ~ RRTFL, CL ~ WTBL, V1 ~ WTBL, V2 ~ WTBL, V2 ~ ALBBL, V3 ~ AGE, V3 ~ WTBL, A ~ ALBBL, A ~ WTBL**	**FALSE**	**4.12**
3	CL ~ CRCL	-	TRUE	47.46
3	V1 ~ WTBL	-	TRUE	44.6

Step #	Excluded Covariate-Parameter Relationship from Previous Step	Included Covariate-parameter Relationships for Step-selected Model	LRT p<0.001?	Change in Objective Function (Δ-2LL) from Previous Selected Model
3	V3 ~ AGE	-	TRUE	37.54
3	V3 ~ WTBL	-	TRUE	25.89
3	A ~ ALBBL	-	TRUE	24.48
3	V2 ~ ALBBL	-	TRUE	11.76
3	V2 ~ WTBL	-	TRUE	11.74
3	CL ~ WTBL	-	FALSE	10.42
3	CL ~ RRTFL	-	FALSE	9.27
**3**	**A ~ WTBL**	**CL ~ CRCL, CL ~ RRTFL, CL ~ WTBL, V1 ~ WTBL, V2 ~ WTBL, V2 ~ ALBBL, V3 ~ AGE, V3 ~ WTBL, A ~ ALBBL**	**FALSE**	**8.85**
4	CL ~ CRCL	-	TRUE	45.89
4	V1 ~ WTBL	-	TRUE	41.97
4	V3 ~ AGE	-	TRUE	39.15
4	A ~ ALBBL	-	TRUE	25.2
4	V3 ~ WTBL	-	TRUE	22.83
4	V2 ~ ALBBL	-	TRUE	12.23
4	V2 ~ WTBL	-	FALSE	10.57
4	CL ~ RRTFL	-	FALSE	8.88
**4**	**CL ~ WTBL**	**CL ~ CRCL, CL ~ RRTFL, V1 ~ WTBL, V2 ~ WTBL, V2 ~ ALBBL, V3 ~ AGE, V3 ~ WTBL, A ~ ALBBL**	**FALSE**	**8.58**
5	CL ~ CRCL	-	TRUE	56.92
5	V3 ~ AGE	-	TRUE	36.5
5	V1 ~ WTBL	-	TRUE	31.43
5	A ~ ALBBL	-	TRUE	22.67
5	V3 ~ WTBL	-	TRUE	16.06
5	V2 ~ ALBBL	-	TRUE	13.37
5	V2 ~ WTBL	-	TRUE	12.47
**5 (Final Model)**	**CL ~ RRTFL**	**CL ~ CRCL, V1 ~ WTBL, V2 ~ WTBL, V2 ~ ALBBL, V3 ~ AGE, V3 ~ WTBL, A ~ ALBBL**	**FALSE**	**7.21**
6	CL ~ CRCL	-	TRUE	63.02
6	V3 ~ AGE	-	TRUE	42.37

Step #	Excluded Covariate-Parameter Relationship from Previous Step	Included Covariate-parameter Relationships for Step-selected Model	LRT p<0.001?	Change in Objective Function (Δ-2LL) from Previous Selected Model
6	V1 ~ WTBL	-	TRUE	31.48
6	A ~ ALBBL	-	TRUE	25.49
6	V3 ~ WTBL	-	TRUE	16.29
6	V2 ~ ALBBL	-	TRUE	13.72
6	V2 ~ WTBL	-	TRUE	12.21

Notes: Bolded rows indicate the covariate removed from each step and the covariates still included in the model.
Abbreviations: ALBBL = baseline albumin; CRCL = creatinine clearance; RRTFL = renal replacement therapy flag; WTBL = baseline weight.

Table 9: Summary Statistics of PK Parameters from Pop-PK Model by Dalbavancin Dosage Group - PK Population

	Dalbavancin Dosage Group					
PK Parameter (units)	Dalbavancin 1500 mg + 1500 mg (N=86)	Dalbavancin 1500 mg + 1125 mg (N=2)	Dalbavancin 1125 mg + 1500 mg (N=3)	Dalbavancin 1125 mg + 1125 mg (N=3)	Dalbavancin 1500 mg only (N=3)	All Dalbavancin Subjects (N=97)
**Pop-PK Total Plasma Concentration (µg/mL), Day 22**						
n	86	2	3	3	3	97
Mean	28.99	48.53	28.44	38.02	14.47	29.20
SD	10.56	6.869	15.00	22.48	3.172	11.48
Min	12.4	43.7	15.0	24.1	10.9	10.9
Max	61.5	53.4	44.6	64.0	17.0	64.0
GM	27.22	48.28	25.81	34.23	14.22	27.14
GSD	1.430	1.153	1.726	1.721	1.263	1.471
CV%	37	14	59	59	24	40
**Pop-PK Free Plasma Concentration (µg/mL), Day 22**						
n	86	2	3	3	3	97
Mean	0.1209	0.1316	0.1552	0.1636	0.04311	0.1211
SD	0.05969	0.009081	0.07760	0.1178	0.01539	0.06213
Min	0.0214	0.125	0.0662	0.0789	0.0315	0.0214
Max	0.301	0.138	0.209	0.298	0.0606	0.301
GM	0.1072	0.1315	0.1381	0.1389	0.04143	0.1062
GSD	1.661	1.071	1.894	1.987	1.404	1.700
CV%	54	7	71	78	35	57
**$AUC_{0\text{-}22\ days}$, total (µg*h/mL)**						
n	86	2	3	3	3	97
Mean	32593	39328	29287	35762	18338	32287
SD	7198	10192	11119	7874	5182	7697
Min	20663	32122	20440	29211	13708	13708
Max	51489	46535	41768	44497	23935	51489
GM	31840	38662	27978	35208	17863	31373

PK Parameter (units)	Dalbavancin Dosage Group					
	Dalbavancin 1500 mg + 1500 mg (N=86)	Dalbavancin 1500 mg + 1125 mg (N=2)	Dalbavancin 1125 mg + 1500 mg (N=3)	Dalbavancin 1125 mg + 1125 mg (N=3)	Dalbavancin 1500 mg only (N=3)	All Dalbavancin Subjects (N=97)
GSD	1.242	1.300	1.441	1.239	1.323	1.276
CV%	22	27	38	22	29	25
**$AUC_{0\text{-}22\ days}$, unbound (μg*h/mL)**						
n	86	2	3	3	3	97
Mean	178.6	122.9	199.3	191.9	83.71	175.6
SD	60.24	11.10	37.52	65.10	53.86	61.23
Min	62.9	115	176	117	41.5	41.5
Max	343	131	243	231	144	343
GM	168.9	122.7	197.1	183.2	73.12	164.7
GSD	1.406	1.095	1.197	1.477	1.880	1.452
CV%	35	9	18	41	70	39
GSD	1.422	1.040	1.255	1.567	1.787	1.466
CV%	36	4	23	47	63	40
**CL, total (L/h)**						
n	86	2	3	3	3	97
Mean	0.0691	0.0390	0.0543	0.0420	0.0623	0.0670
SD	0.0175	0.00566	0.0159	0.0132	0.0132	0.0182
Min	0.038	0.035	0.036	0.027	0.048	0.027
Max	0.11	0.043	0.065	0.052	0.074	0.11
GM	0.0669	0.0388	0.0525	0.0404	0.0613	0.0645
GSD	1.29	1.16	1.39	1.42	1.25	1.32
CV%	26	15	34	36	22	29
**Central Volume of Distribution (L)**						
n	86	2	3	3	3	97
Mean	5.96	5.34	5.89	4.38	6.57	5.92
SD	1.41	1.07	2.08	1.28	2.74	1.46
Min	3.6	4.6	4.1	3.6	3.9	3.6

PK Parameter (units)	Dalbavancin Dosage Group					
	Dalbavancin 1500 mg + 1500 mg (N=86)	Dalbavancin 1500 mg + 1125 mg (N=2)	Dalbavancin 1125 mg + 1500 mg (N=3)	Dalbavancin 1125 mg + 1125 mg (N=3)	Dalbavancin 1500 mg only (N=3)	All Dalbavancin Subjects (N=97)
Max	10.8	6.1	8.2	5.9	9.3	10.8
GM	5.81	5.28	5.66	4.26	6.16	5.75
GSD	1.26	1.22	1.41	1.32	1.56	1.28
CV%	24	20	36	28	47	25
**Peripheral Volume of Distribution 2 (L)**						
n	86	2	3	3	3	97
Mean	9.64	5.69	12.24	6.67	8.94	9.52
SD	3.43	0.290	3.42	2.10	3.33	3.43
Min	4.0	5.5	8.6	4.9	5.9	4.0
Max	22.1	5.9	15.4	9.0	12.5	22.1
GM	9.11	5.68	11.90	6.46	8.53	8.98
GSD	1.39	1.05	1.35	1.36	1.46	1.40
CV%	34	5	30	32	39	35
**Peripheral Volume of Distribution 3 (L)**						
n	86	2	3	3	3	97
Mean	11.45	11.82	13.74	7.55	13.76	11.48
SD	4.10	6.29	8.55	0.788	5.49	4.27
Min	4.0	7.4	8.6	6.7	7.5	4.0
Max	24.2	16.3	23.6	8.2	17.5	24.2
GM	10.78	10.95	12.24	7.52	12.87	10.77
GSD	1.42	1.75	1.77	1.11	1.61	1.43
CV%	36	61	62	11	50	37
**Pop-PK Scaling Factor (ug/mL)**						
n	86	2	3	3	3	97
Mean	0.00147	0.000850	0.00197	0.00133	0.00133	0.00146
SD	0.000568	0.000212	0.000603	0.000321	0.000462	0.000563
Min	0.00050	0.00070	0.0014	0.0011	0.00080	0.00050

	Dalbavancin Dosage Group					
PK Parameter (units)	Dalbavancin 1500 mg + 1500 mg (N=86)	Dalbavancin 1500 mg + 1125 mg (N=2)	Dalbavancin 1125 mg + 1500 mg (N=3)	Dalbavancin 1125 mg + 1125 mg (N=3)	Dalbavancin 1500 mg only (N=3)	All Dalbavancin Subjects (N=97)
Max	0.0035	0.0010	0.0026	0.0017	0.0016	0.0035
GM	0.00138	0.000837	0.00191	0.00131	0.00127	0.00137
GSD	1.42	1.29	1.36	1.26	1.49	1.43
CV%	36	26	32	23	42	37
**Steady-State Volume (L)**						
n	86	2	3	3	3	97
Mean	27.05	22.84	31.87	18.59	29.27	26.92
SD	7.72	7.08	13.55	1.61	11.22	7.95
Min	15.7	17.8	21.4	16.8	17.2	15.7
Max	54.4	27.8	47.2	19.8	39.3	54.4
GM	26.06	22.28	30.11	18.55	27.65	25.87
GSD	1.31	1.37	1.50	1.09	1.53	1.32
CV%	28	32	42	9	45	28
**$T_{1/2,\alpha}$ (h)**						
n	86	2	3	3	3	97
Mean	2.79	2.64	2.93	2.00	3.19	2.78
SD	0.712	0.806	1.23	0.387	1.27	0.741
Min	1.4	2.1	2.0	1.7	1.9	1.4
Max	5.1	3.2	4.3	2.4	4.4	5.1
GM	2.70	2.58	2.78	1.98	3.00	2.69
GSD	1.29	1.36	1.49	1.21	1.55	1.30
CV%	26	32	41	19	46	27
**$T_{1/2,\beta}$ (day)**						
n	86	2	3	3	3	97
Mean	4.50	4.20	5.79	3.61	5.18	4.53
SD	1.17	0.636	2.43	0.115	1.66	1.22
Min	2.7	3.8	4.3	3.5	3.4	2.7

	Dalbavancin Dosage Group					
**PK Parameter (units)**	**Dalbavancin 1500 mg + 1500 mg (N=86)**	**Dalbavancin 1500 mg + 1125 mg (N=2)**	**Dalbavancin 1125 mg + 1500 mg (N=3)**	**Dalbavancin 1125 mg + 1125 mg (N=3)**	**Dalbavancin 1500 mg only (N=3)**	**All Dalbavancin Subjects (N=97)**
Max	8.5	4.7	8.6	3.7	6.6	8.6
GM	4.36	4.18	5.49	3.61	4.99	4.38
GSD	1.28	1.16	1.48	1.03	1.42	1.28
CV%	25	15	41	3	36	25
**$T_{1/2,\gamma}$ (day)**						
n	86	2	3	3	3	97
Mean	18.13	19.15	25.24	17.43	18.23	18.35
SD	5.04	2.23	4.98	2.86	4.68	5.03
Min	10.5	17.6	22.2	14.2	13.6	10.5
Max	34.4	20.7	31.0	19.7	22.9	34.4
GM	17.52	19.08	24.94	17.26	17.82	17.74
GSD	1.29	1.12	1.21	1.19	1.30	1.29
CV%	26	12	19	17	27	26

Notes: N = Number of subjects in the PK Population in the respective dalbavancin dose group.
n = Number of subjects with results meeting analysis criteria contributing to summary statistics.

LIST OF FIGURES

Figure 1: Distribution of Total and Unbound Dalbavancin Concentrations, by BQL Status

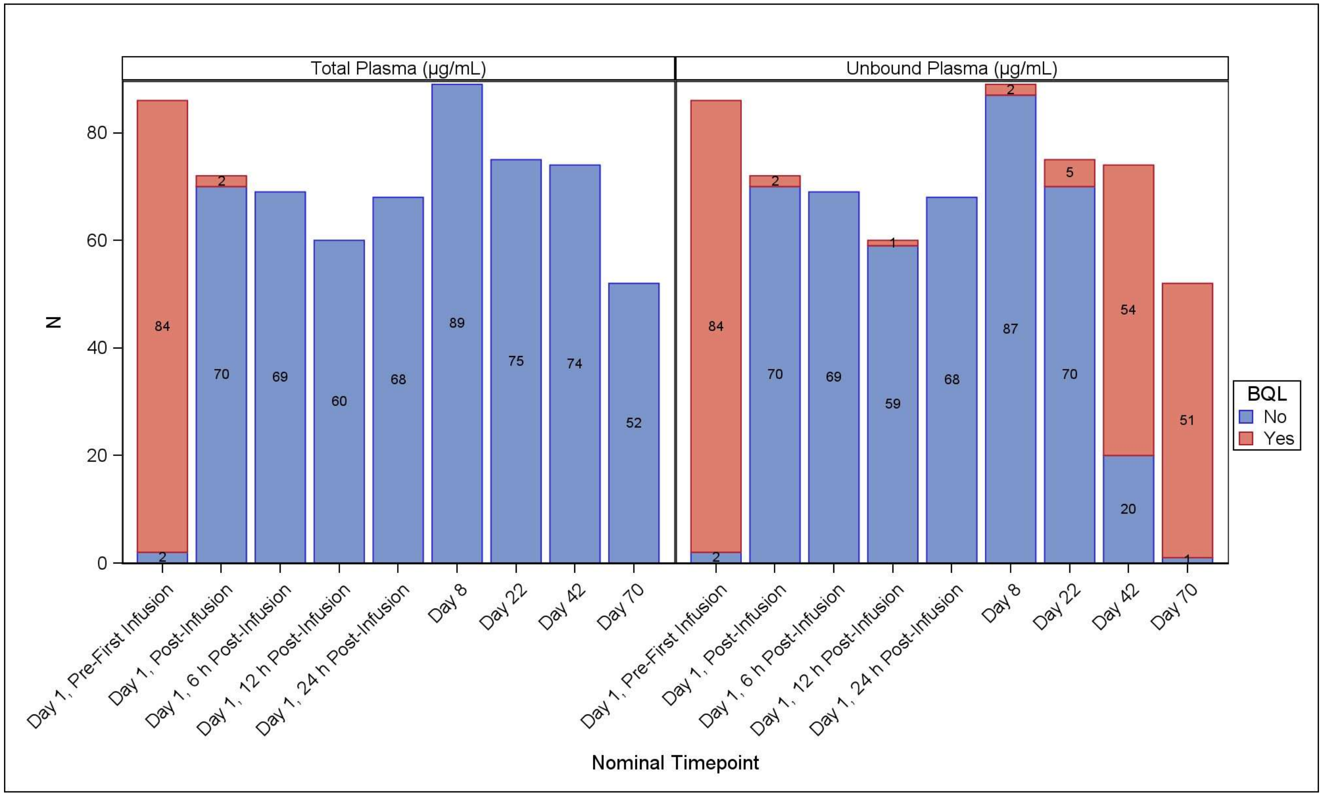

Figure 2: Observed Total Plasma Concentration Scatterplot vs. Time from First Dose by Dalbavancin Dosage Group, BQL Values Omitted

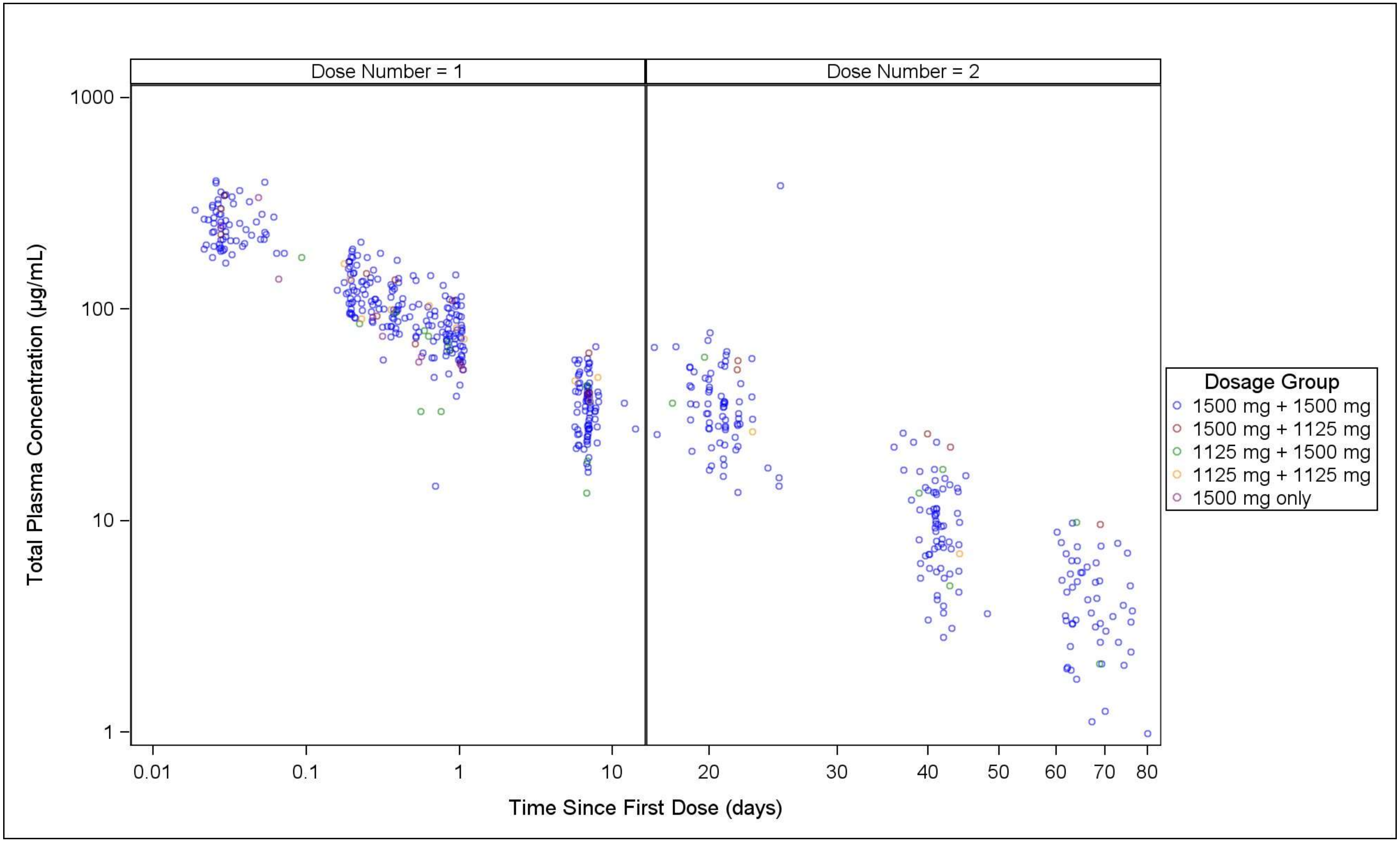

Figure 3: Observed Unbound Plasma Concentration Scatterplot vs. Time from First Dose by Dalbavancin Dosage Group, BQL Values Omitted

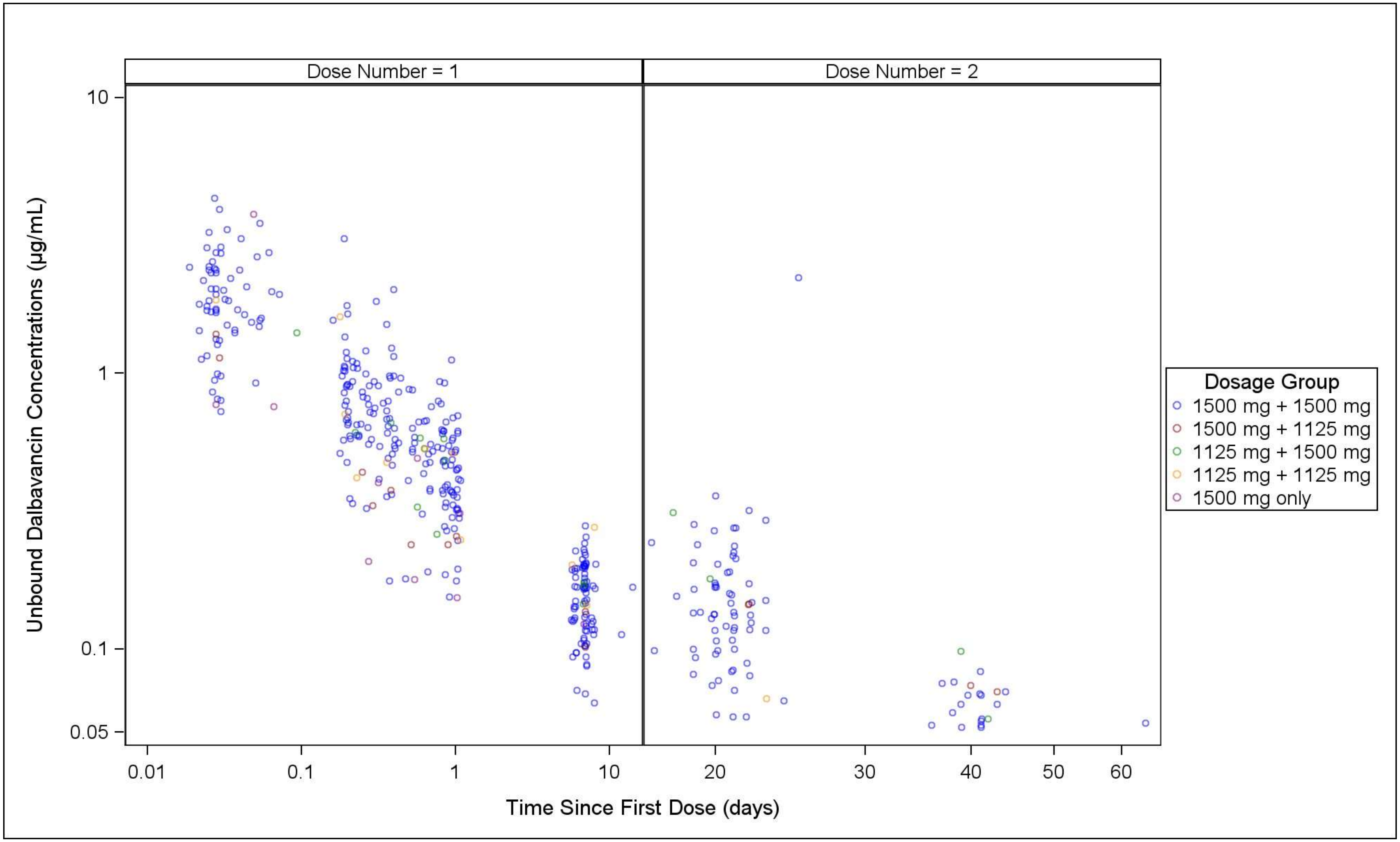

Figure 4: Observed Total Plasma Concentration Scatterplot vs. Time from First Dose by BQL and Analytical Exclusion Status

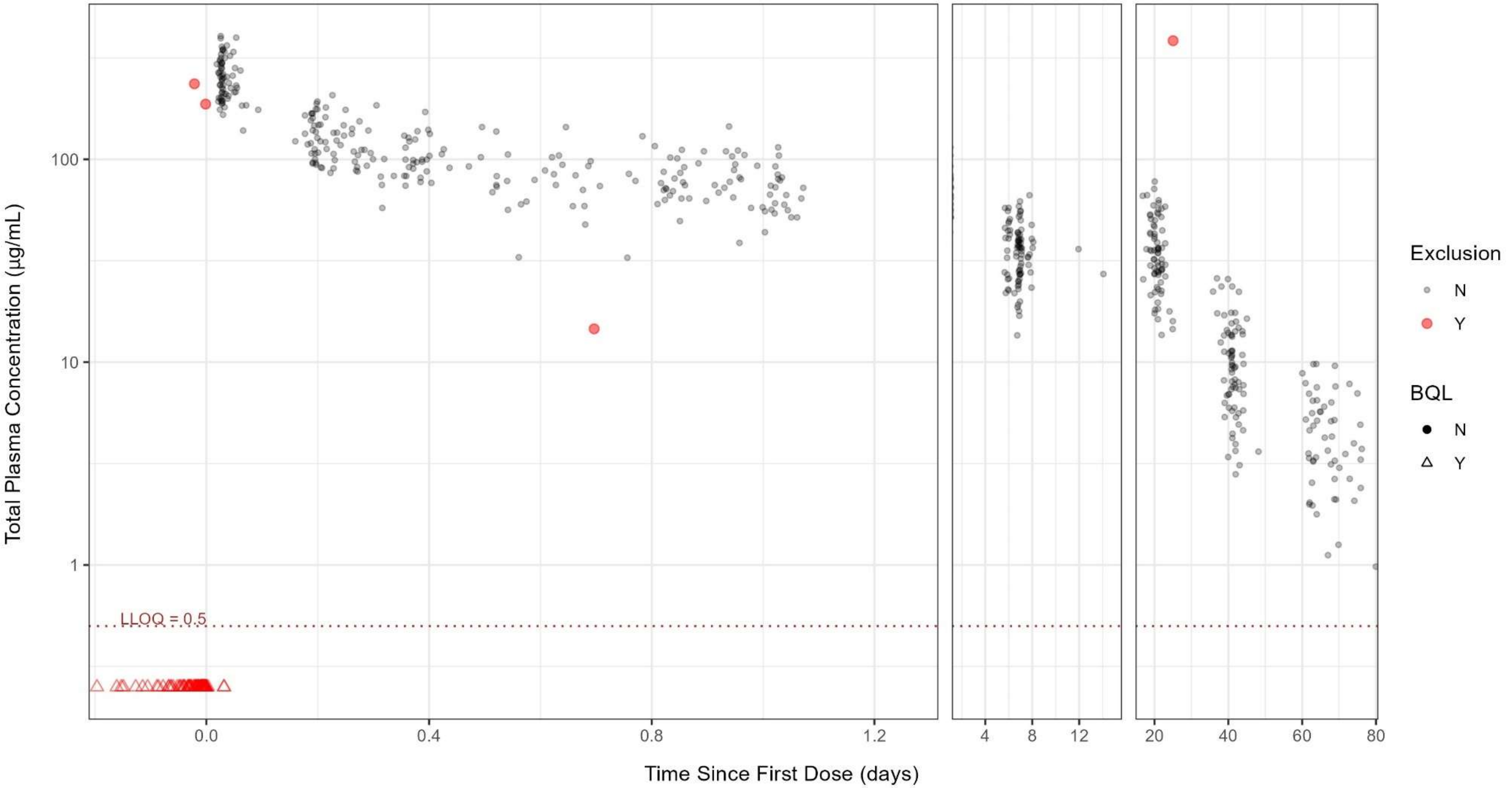

Plotted concentrations values for BQL observations are imputed as LLOQ/2

Figure 5: Observed Unbound Plasma Concentration Scatterplot vs. Time from First Dose by BQL and Analytical Exclusion Status

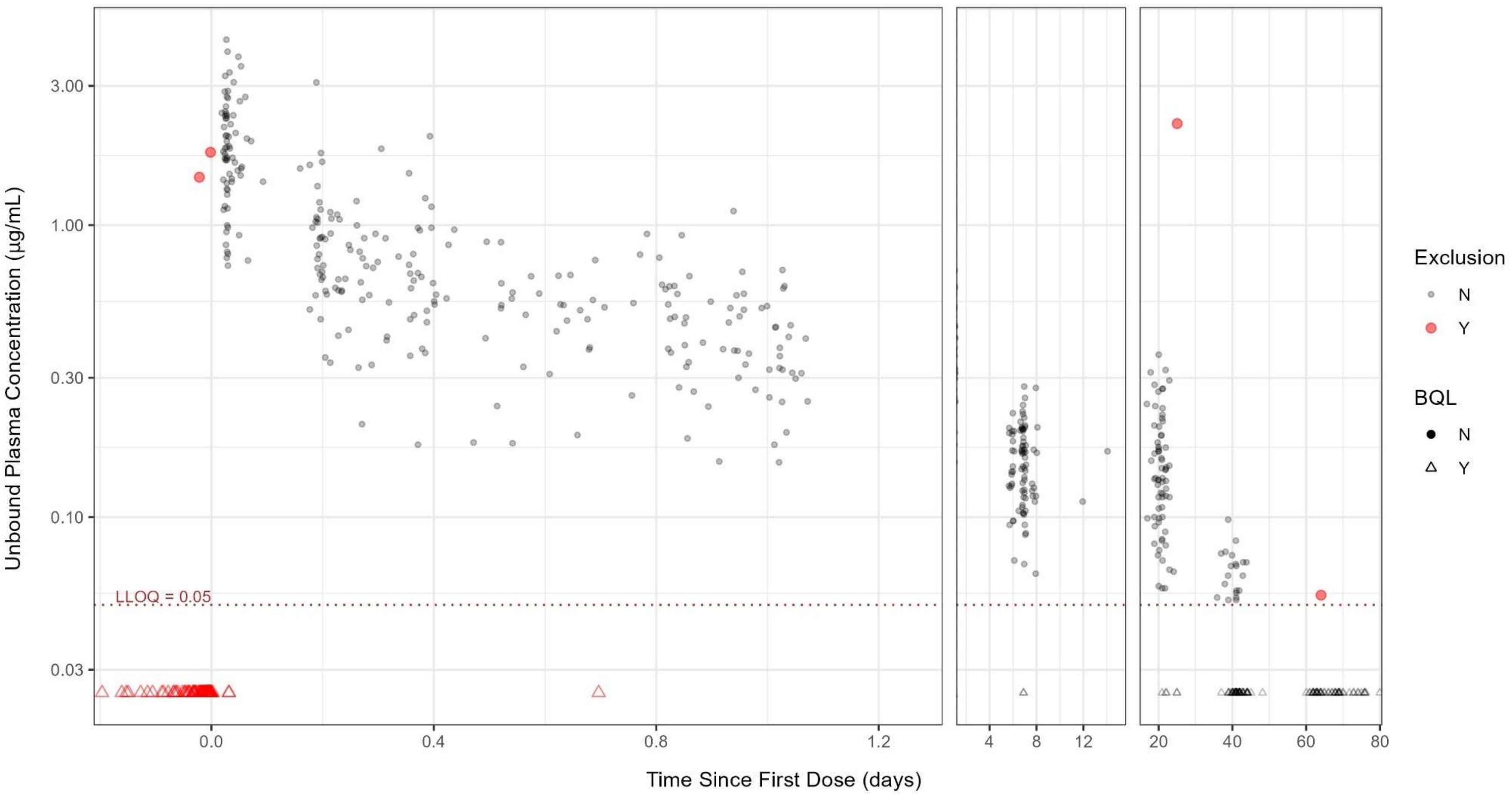

Plotted concentrations values for BQL observations are imputed as LLOQ/2

Figure 6: Summarized Total Plasma Concentrations by Nominal Time Since First Dose - Geometric Mean ±1 Geometric SD

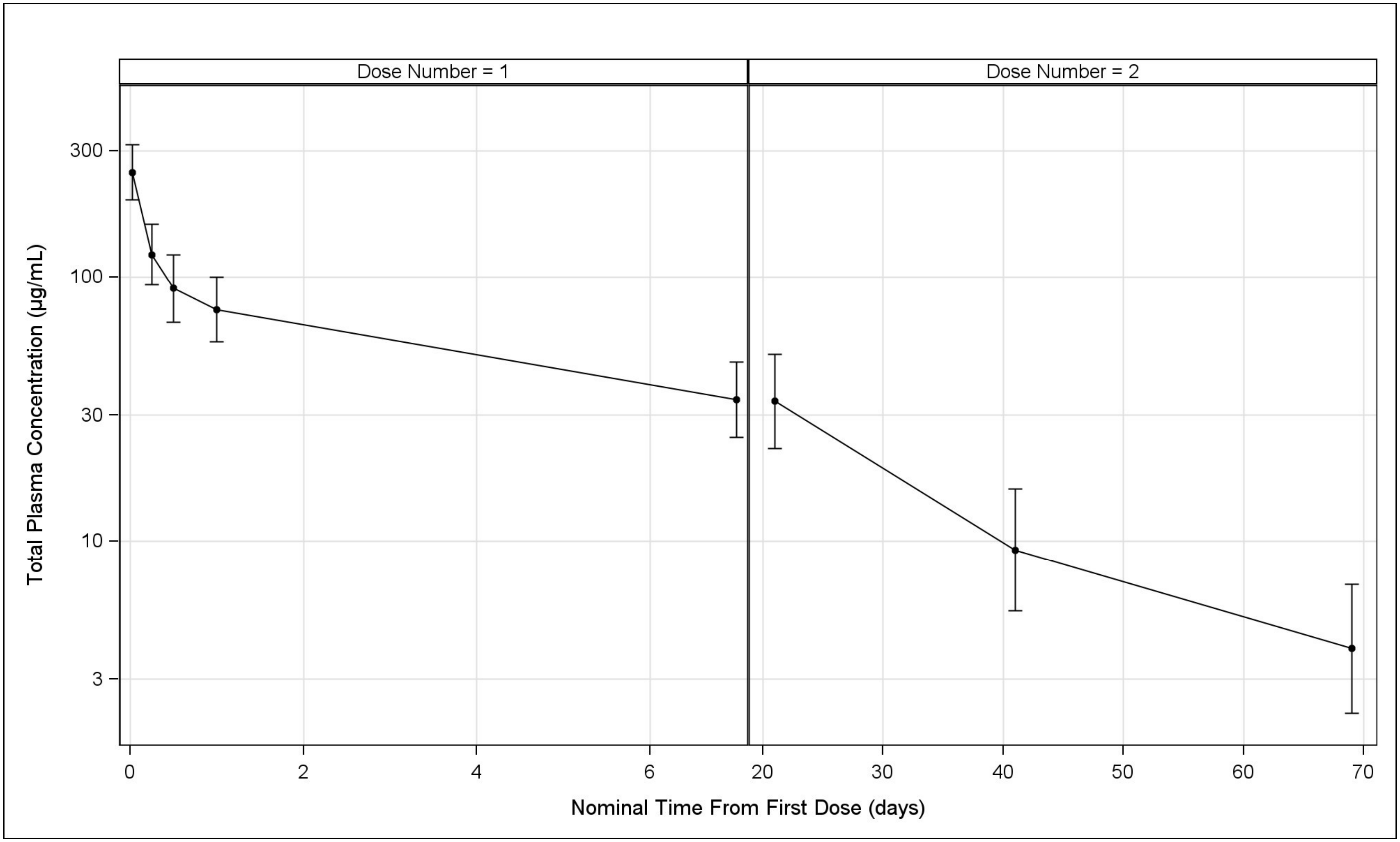

Figure 7: Summarized Total Plasma Concentrations by Nominal Time Since First Dose and Dalbavancin Dosage Group - Geometric Mean

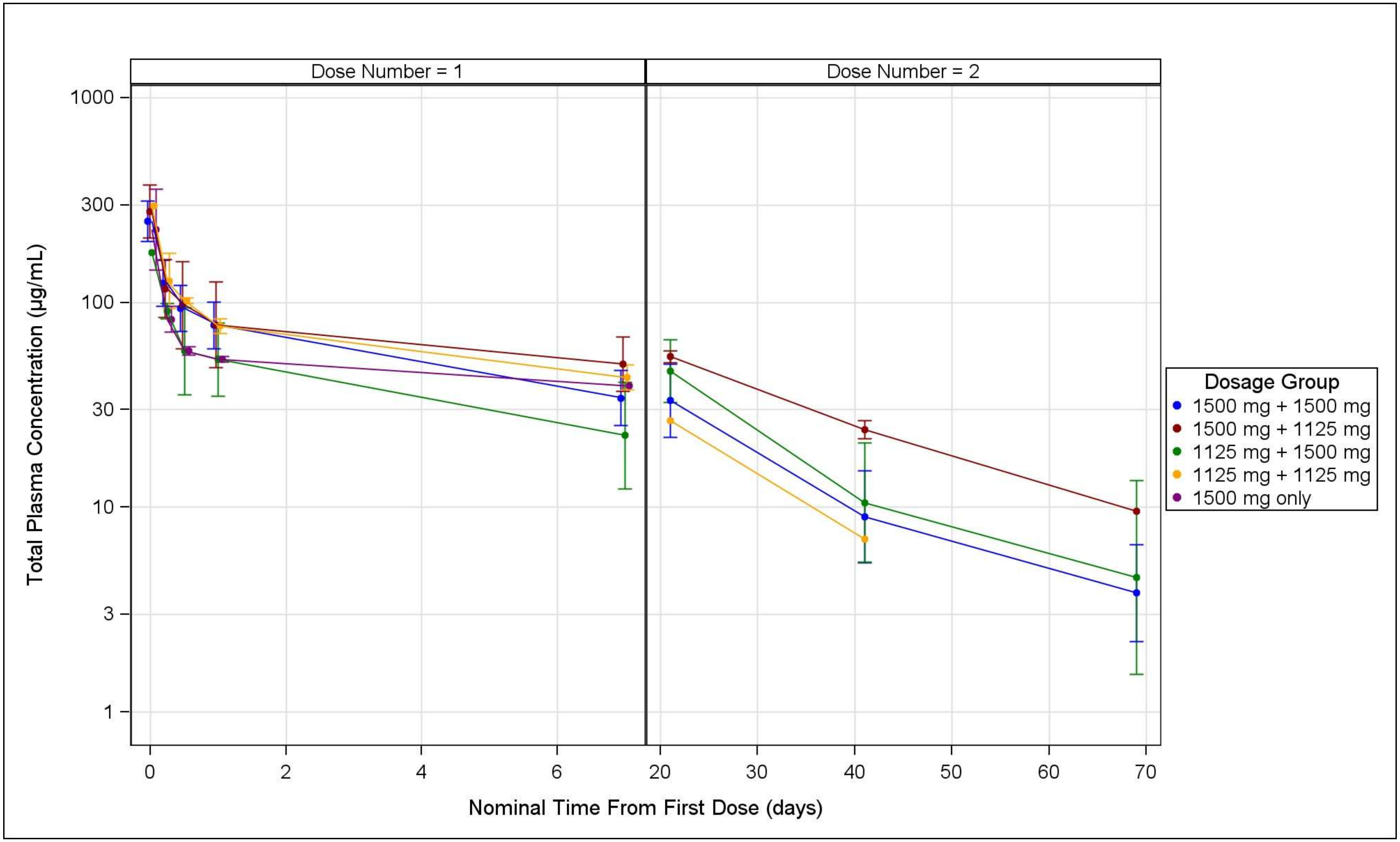

Figure 8: Summarized Unbound Plasma Concentrations by Nominal Time Since First Dose - Geometric Mean ±1 Geometric SD, BQL Values Excluded or Imputed

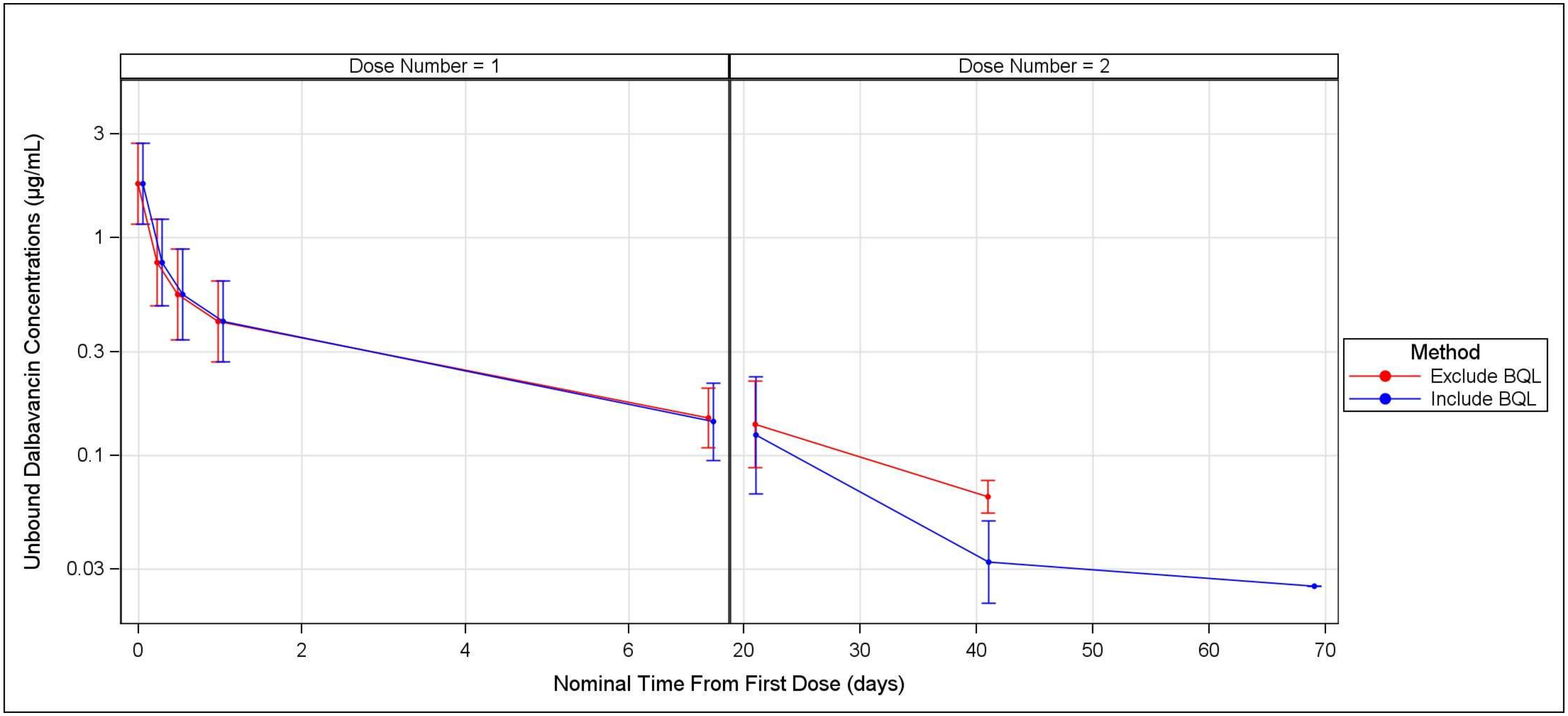

Figure 9: Summarized Unbound Plasma Concentrations by Nominal Time Since First Dose and Dalbavancin Dosage Group - Geometric Mean, BQL Values Excluded

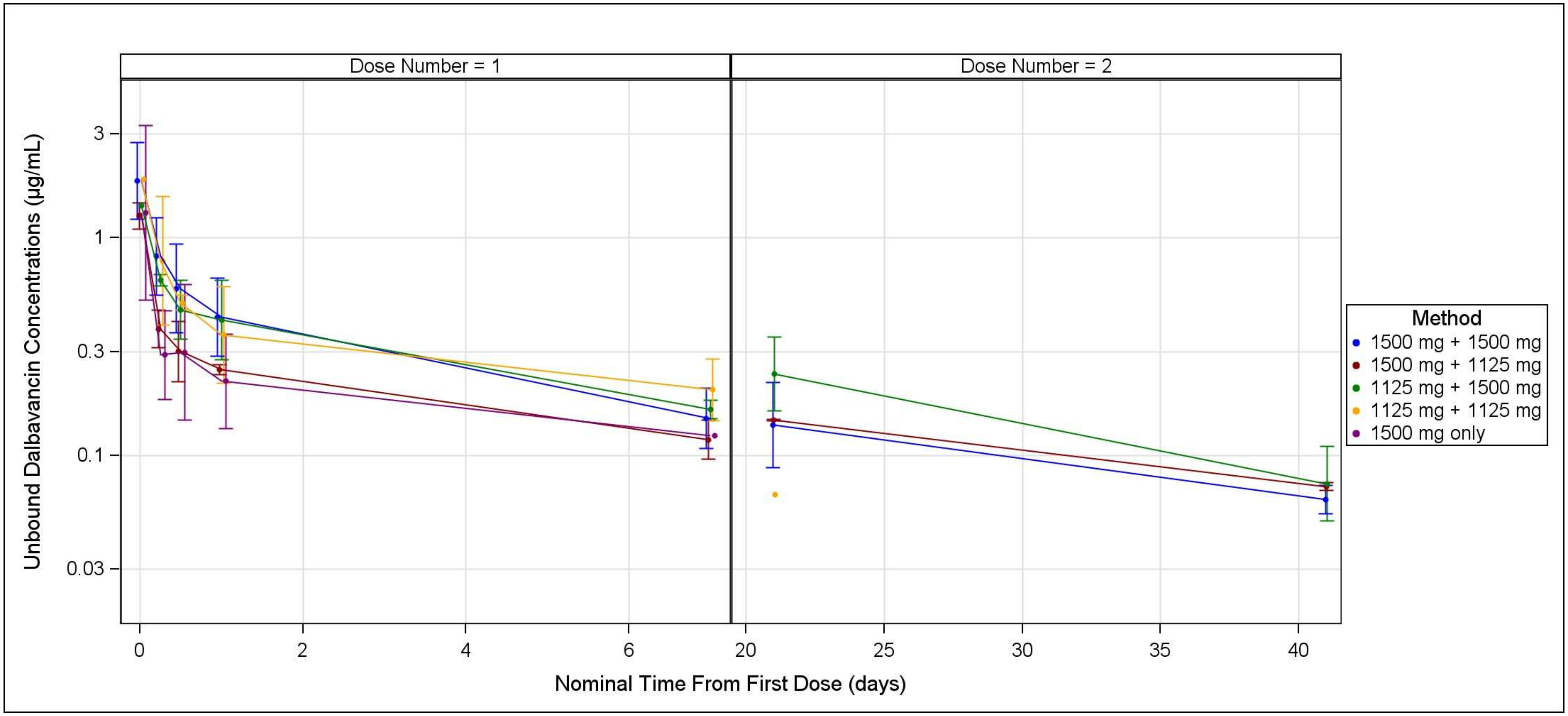

Figure 10: Summarized Unbound Plasma Concentrations by Nominal Time Since First Dose and Dalbavancin Dosage Group - Geometric Mean, Imputed BQL Values Included

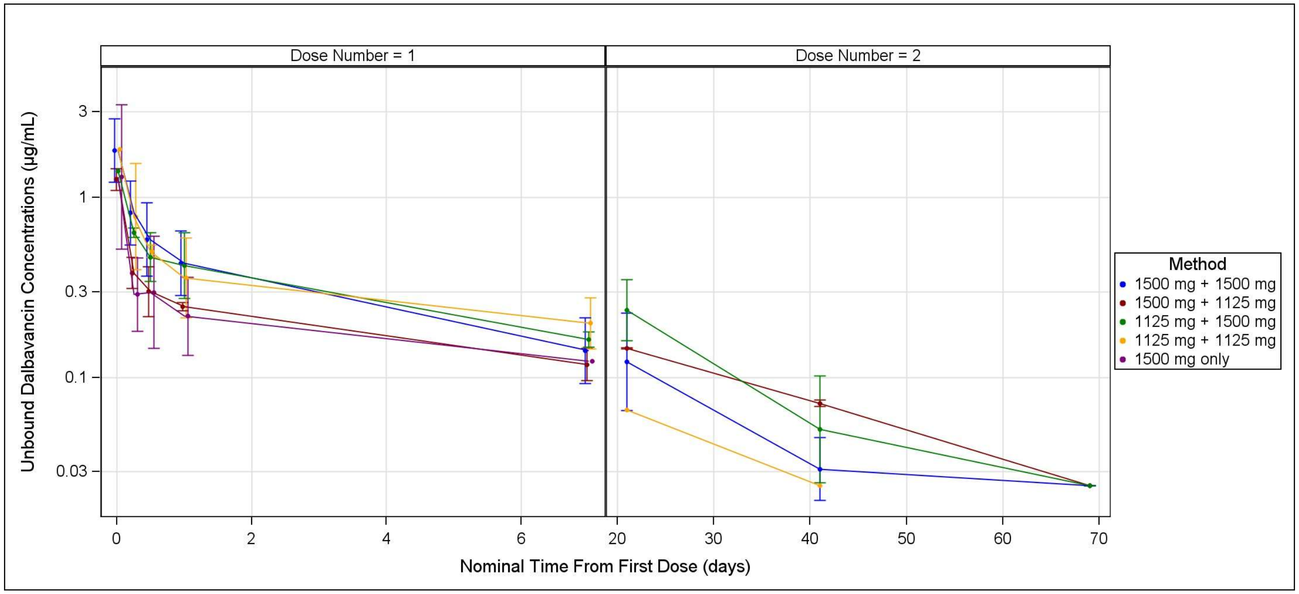

Figure 11: Observed Total vs. Unbound Concentrations, Including BQL Observations

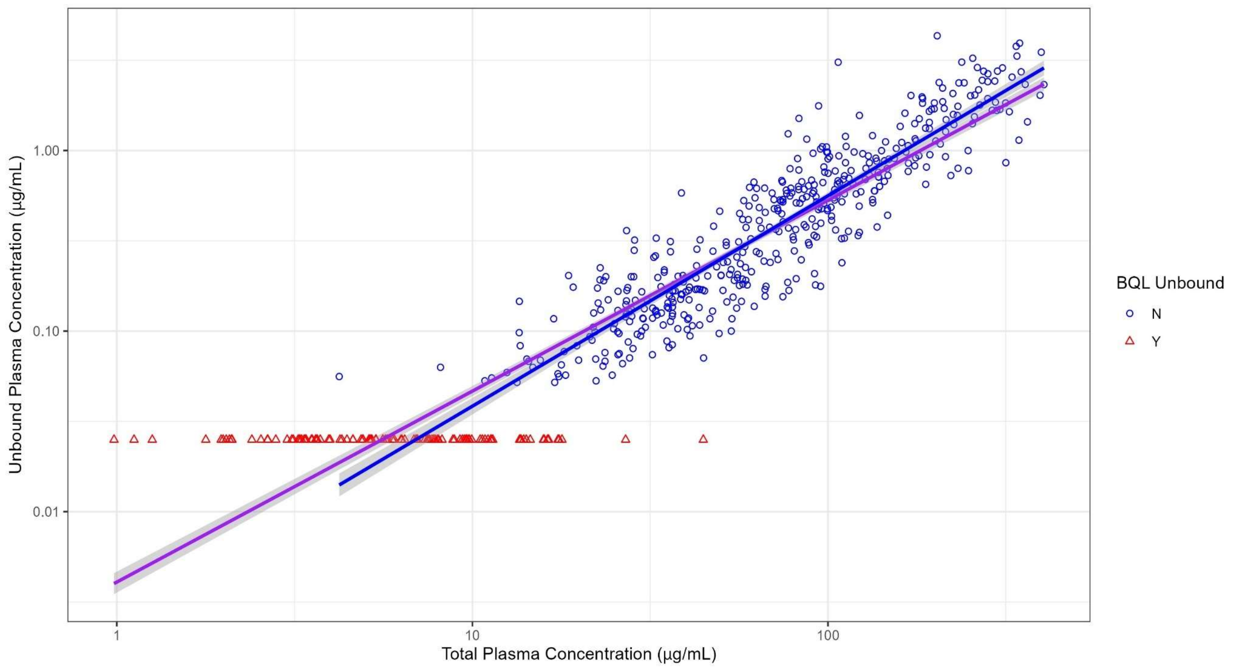

Plotted concentrations values for BQL observations are imputed as LLOQ/2

Figure 12: Observed Fraction Unbound Concentration vs. Time Since First Dose, Imputed BQL Values Included (A) and Excluded (B)

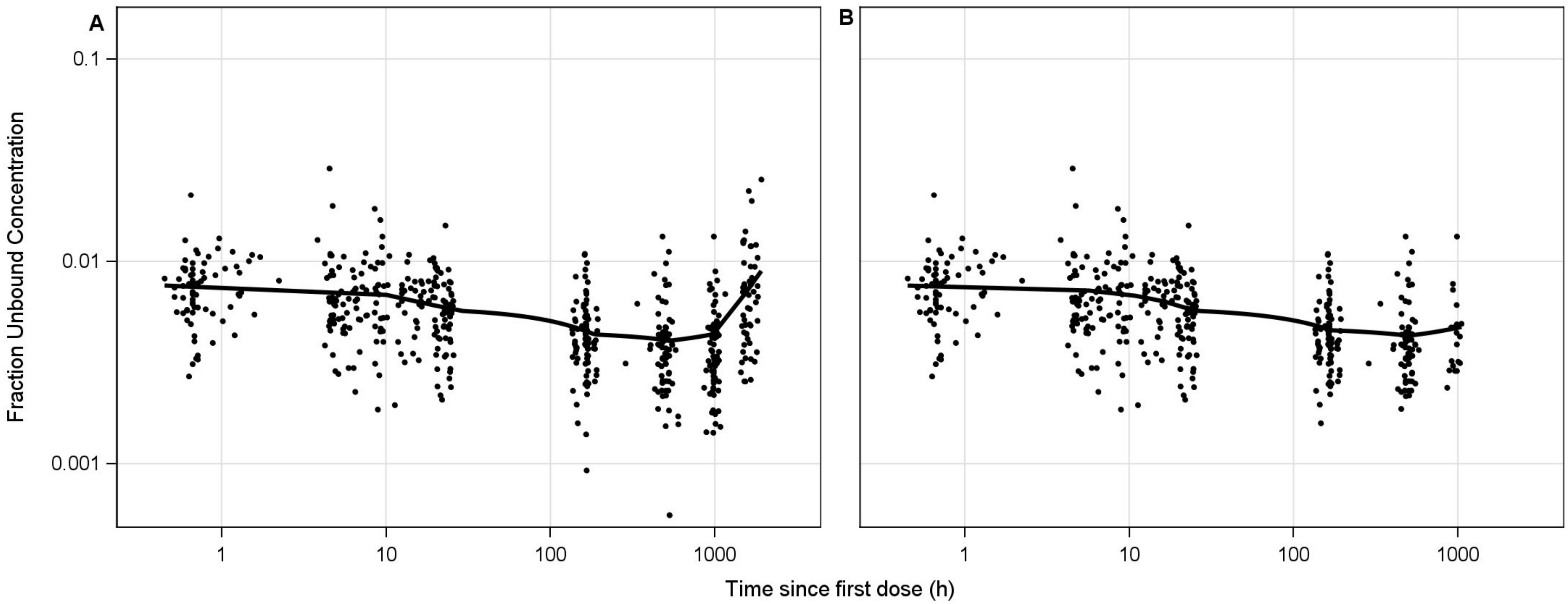

A: fraction unbound calculated with BQL concentrations imputed as LLOQ/2
B: fraction unbound plotted only for total and unbound concentrations above LLOQ.

Figure 13: Final Model Diagnostics: Observed Concentrations versus Population Predicted Concentrations (µg/mL) of Total and Unbound Dalbavancin

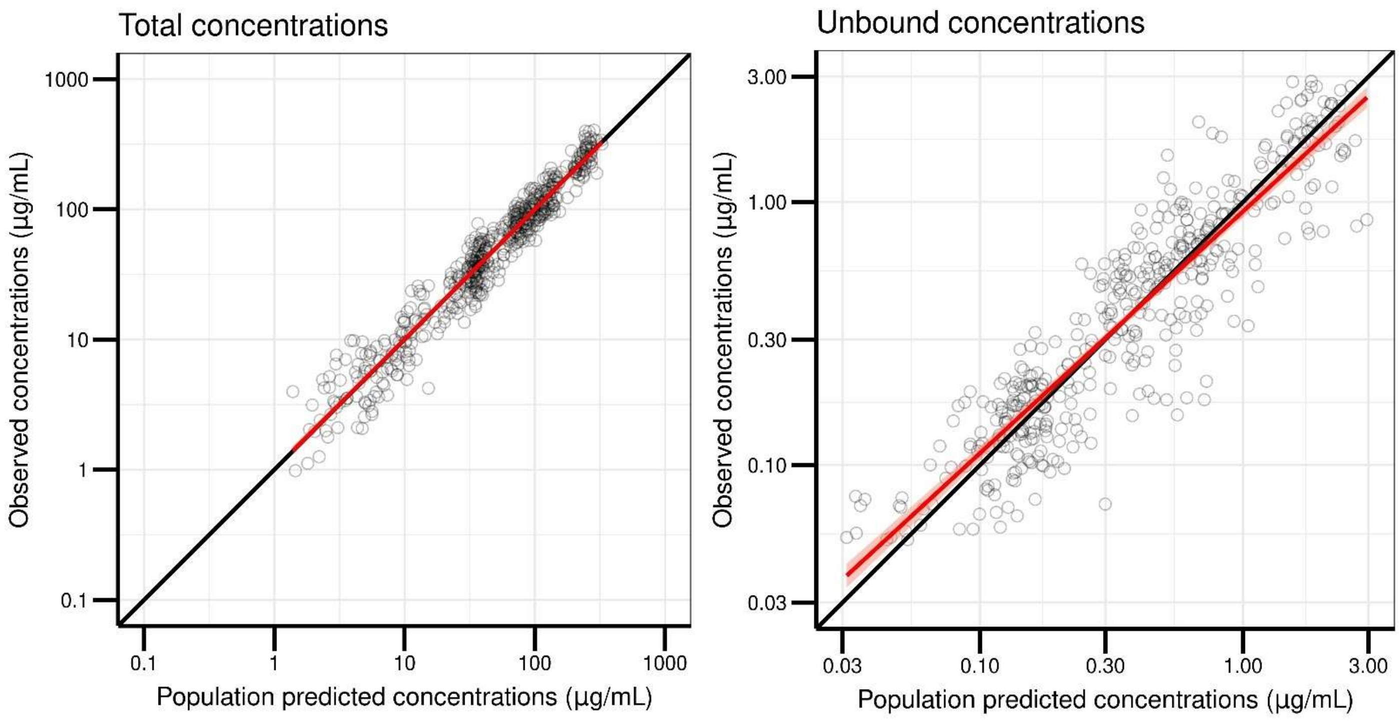

Figure 14: Final Model Diagnostics: Observed Concentrations versus Individual Predicted Concentrations (µg/mL) of Total and Unbound Dalbavancin

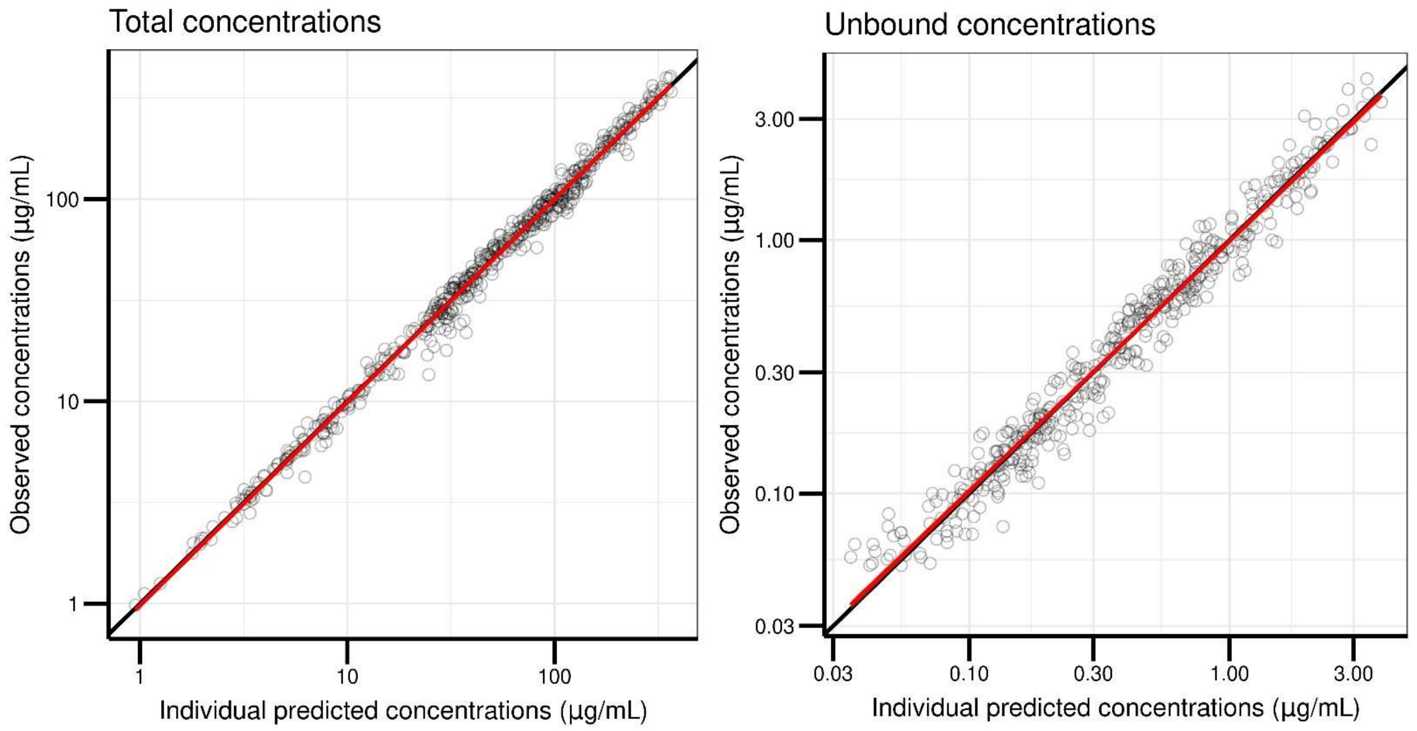

Figure 15: Final Model Diagnostics: Quantile-Quantile Plot of Conditional Weighted Residuals

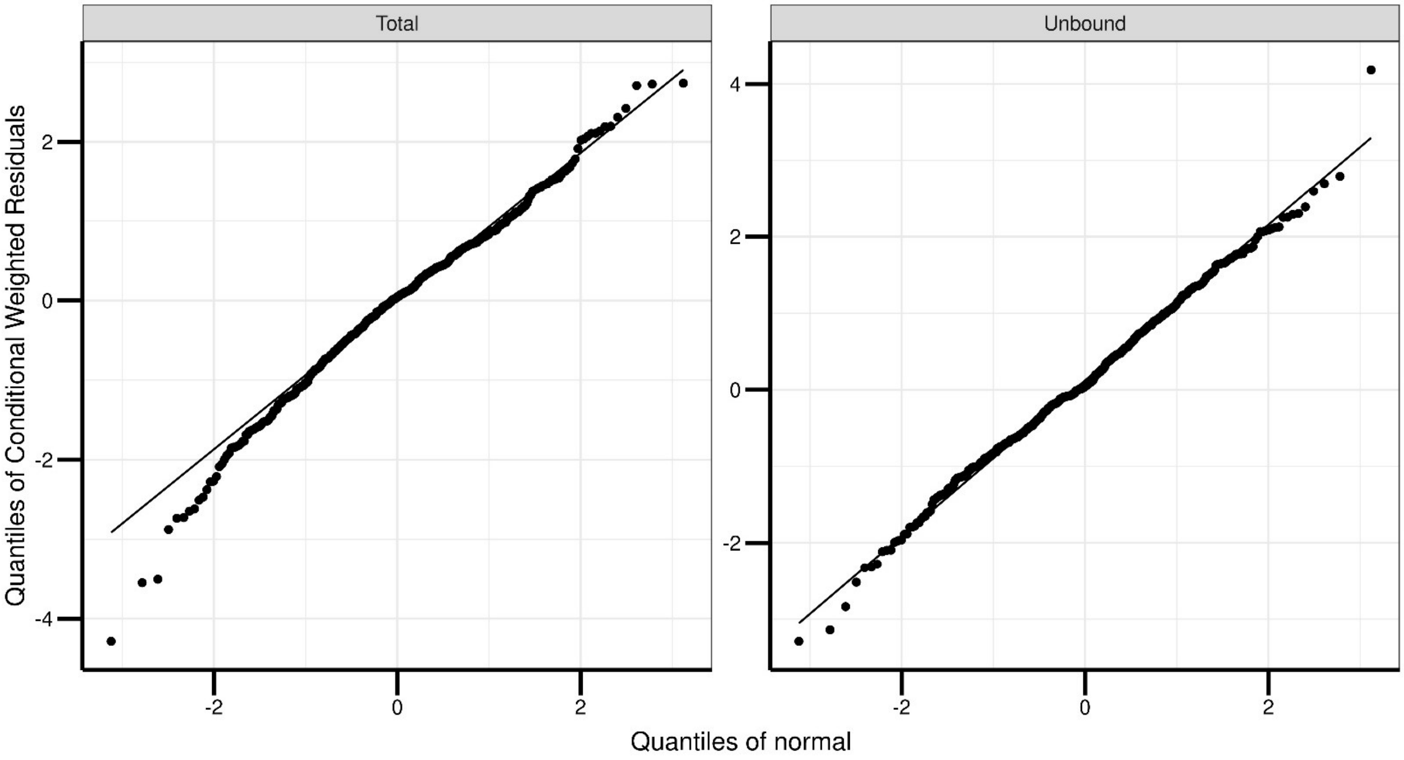

Figure 16: Final Model Diagnostics: Quantile-Quantile Plot of Individual Weighted Residuals

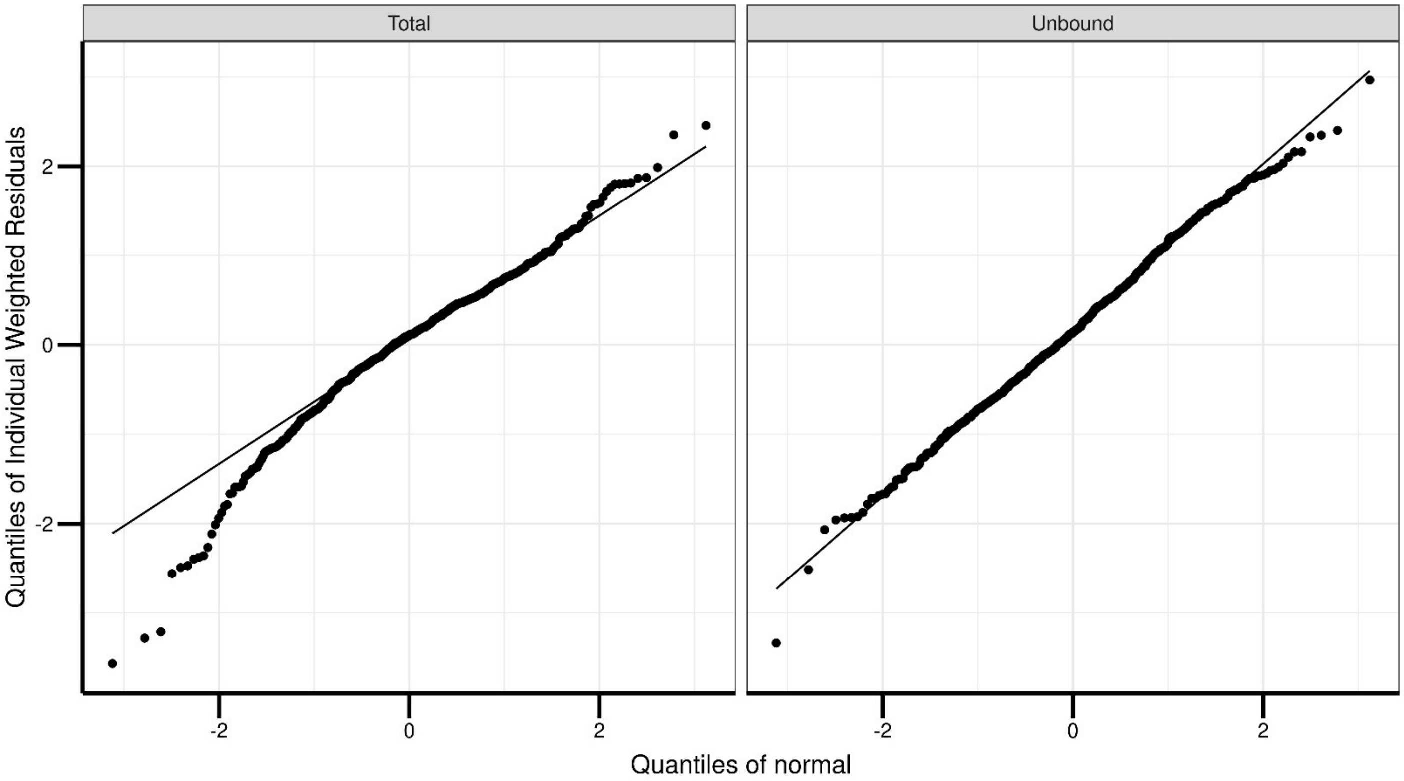

Figure 17: Final Model Diagnostics: Conditional Weighted Residuals versus Time Since First Dose

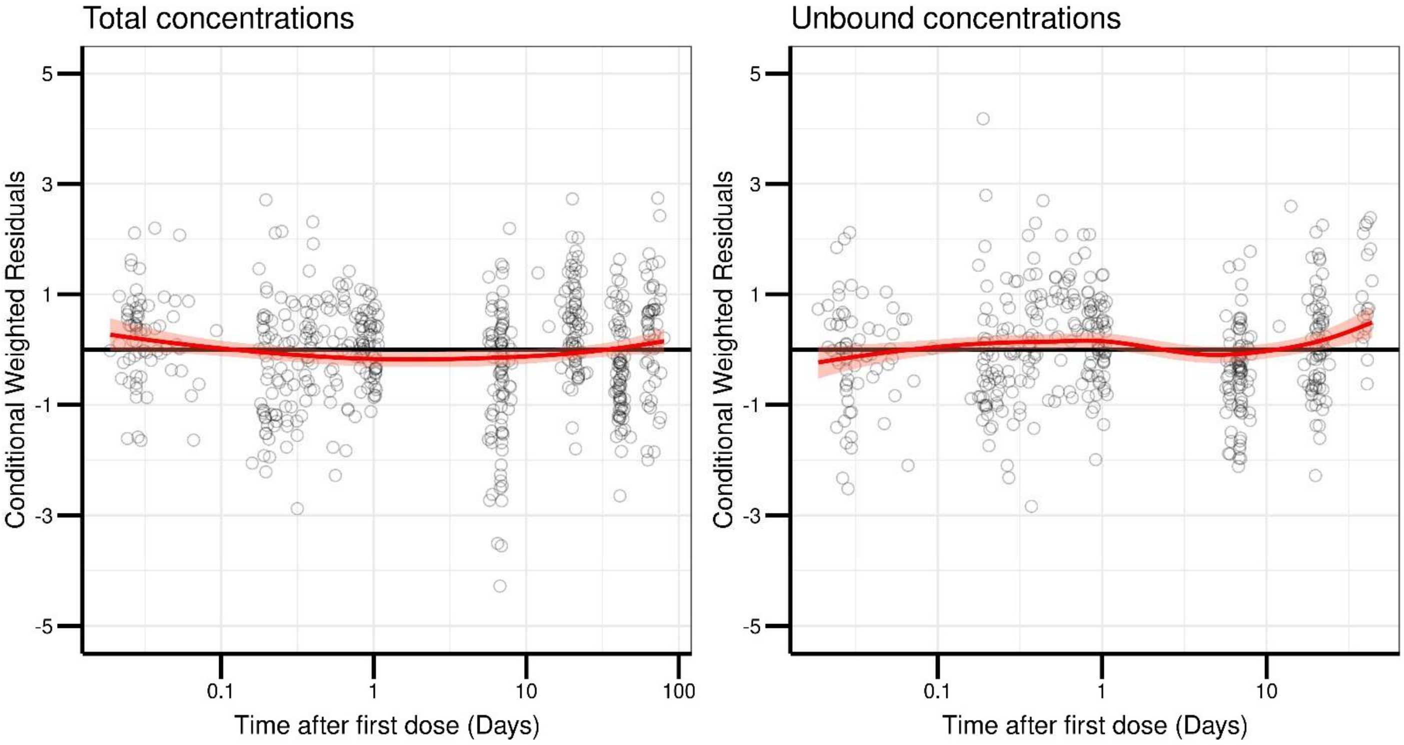

Figure 18: Final Model Diagnostics: Individual Weighted Residuals versus Time Since First Dose

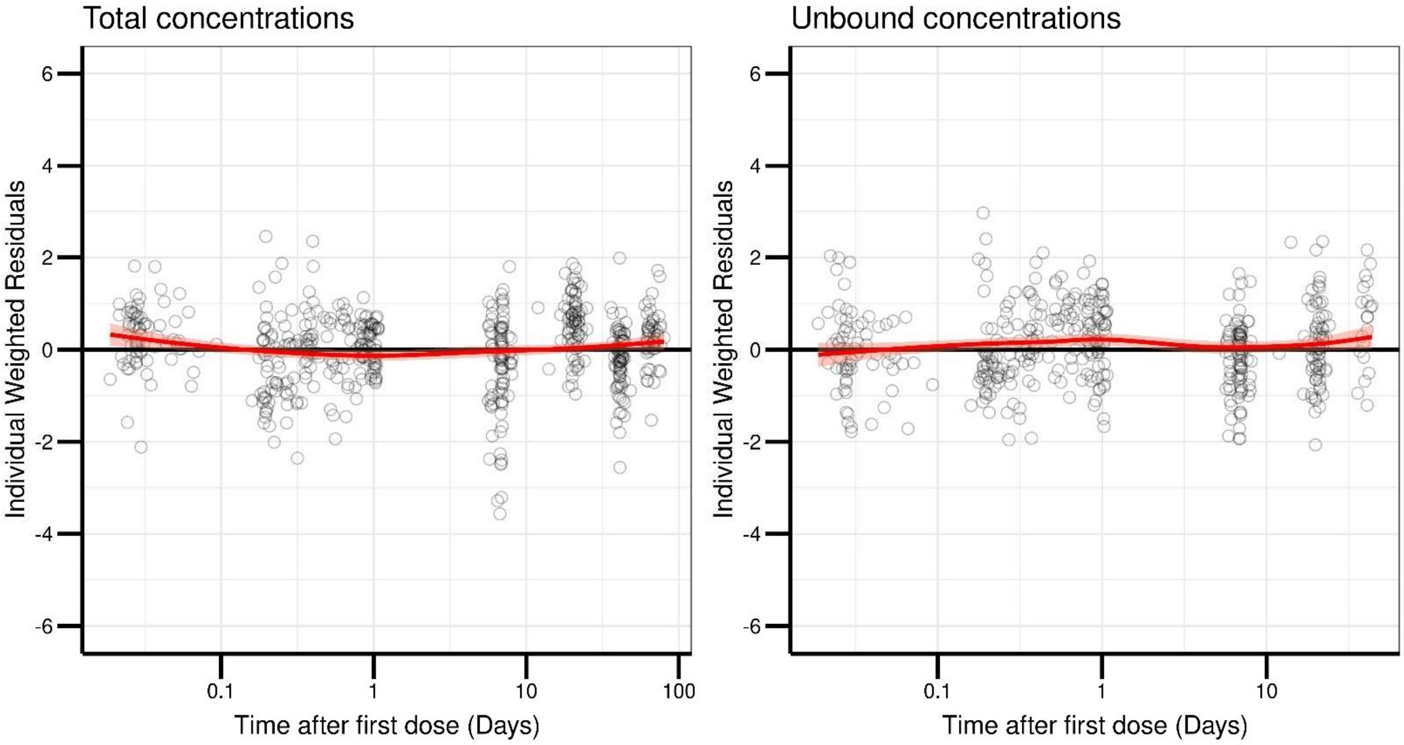

Figure 19: Final Model Diagnostics: Conditional Weighted Residuals versus Population Predicted Concentrations (µg/mL) of Total and Unbound Dalbavancin

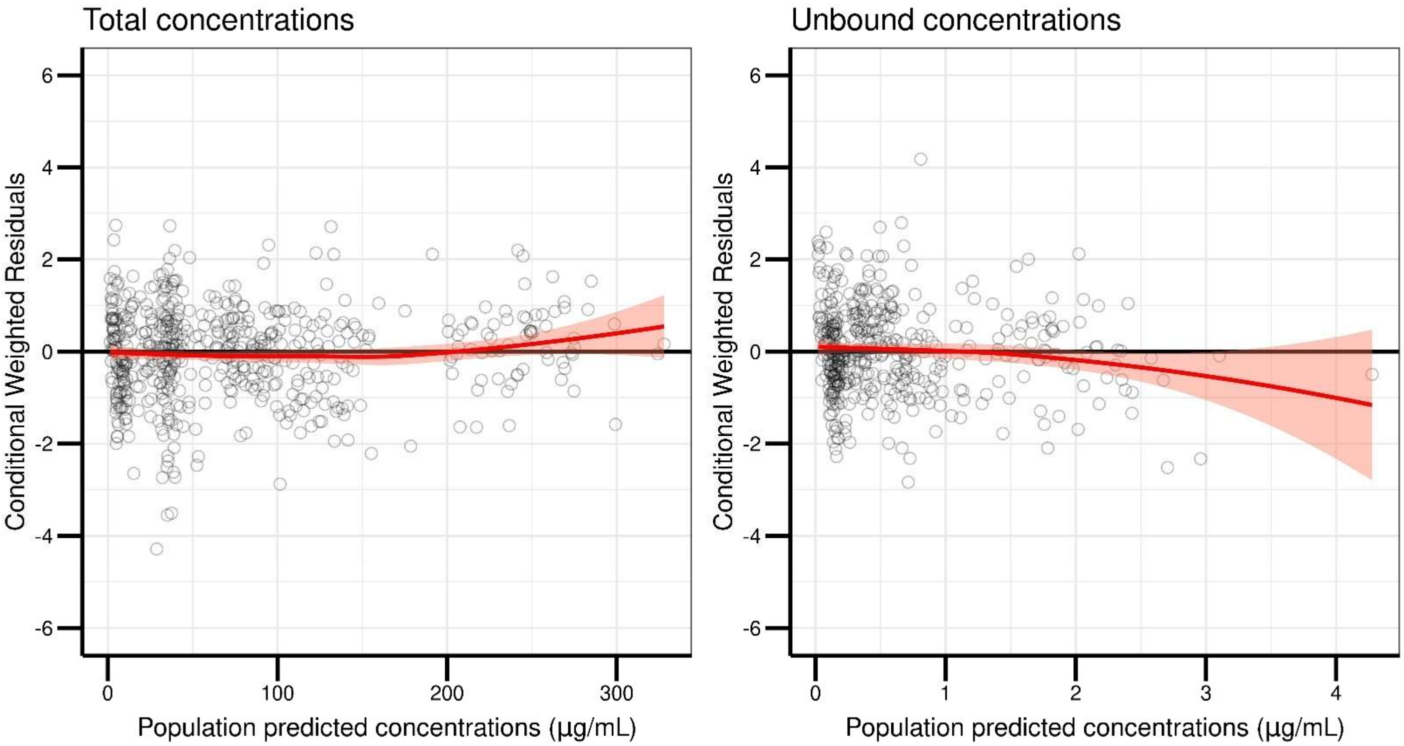

Figure 20: Final Model Diagnostics: Individual Weighted Residuals versus Individual Predicted Concentrations (µg/mL) of Total and Unbound Dalbavancin

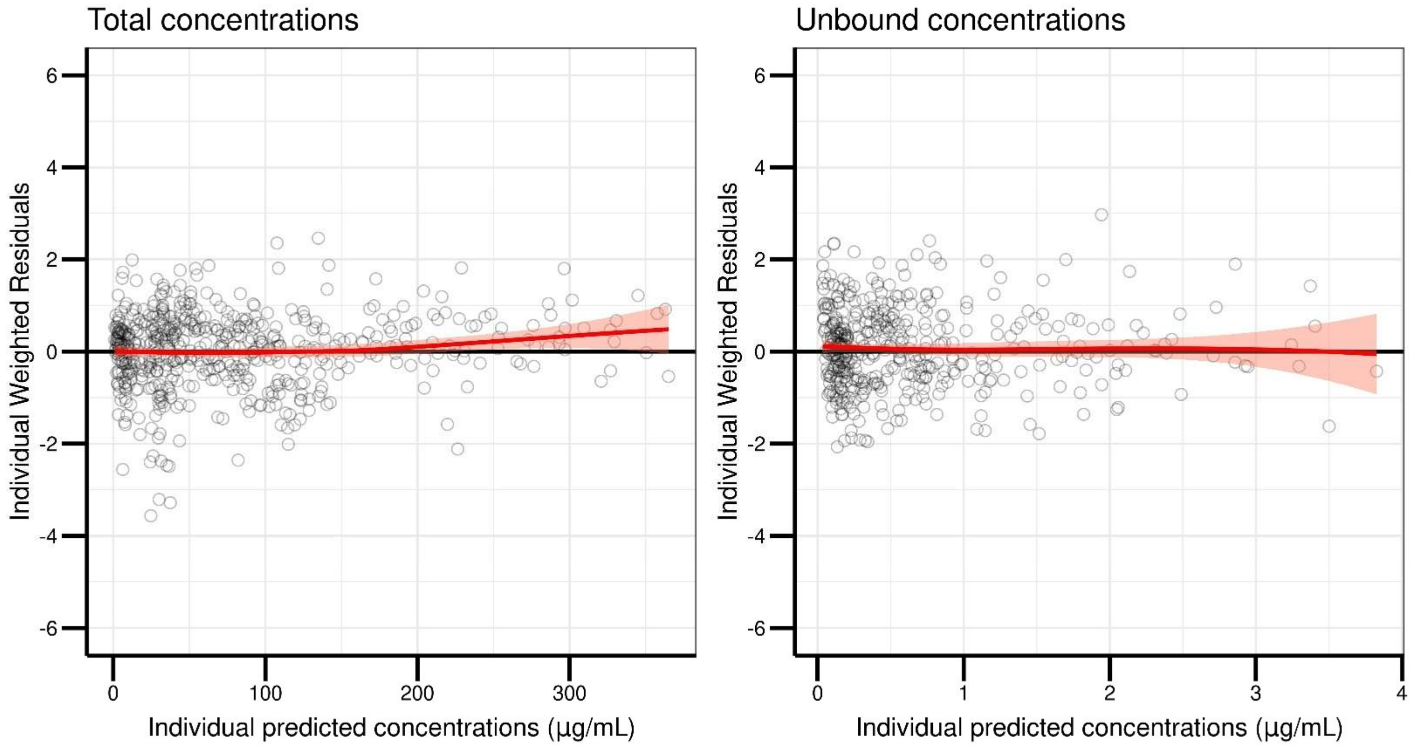

Figure 21: Final Model Diagnostics: Absolute Value of Individual Weighted Residuals versus Individual Predicted Concentrations (µg/mL) of Total and Unbound Dalbavancin

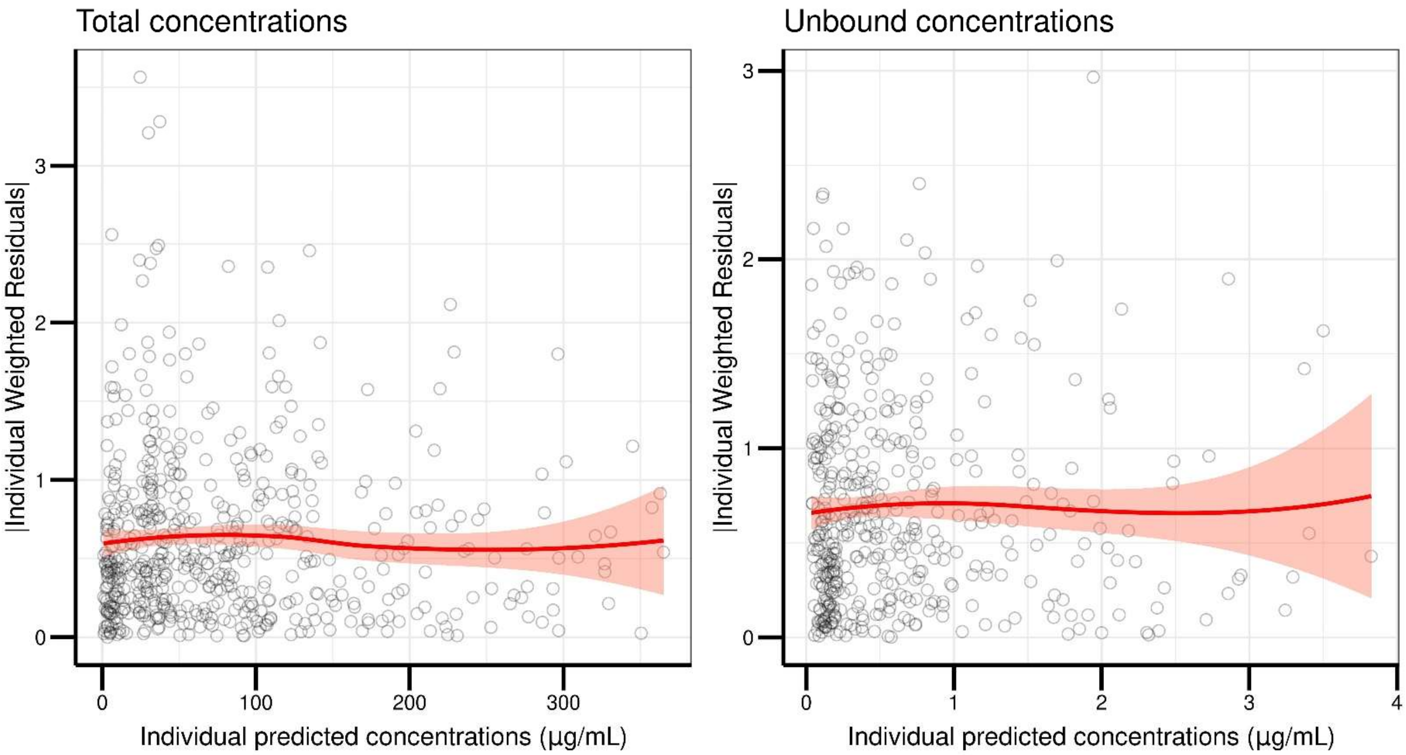

Figure 22: Final Model Diagnostics: Prediction-Corrected Visual Predictive Check for Fit on Total Plasma Dalbavancin Concentrations

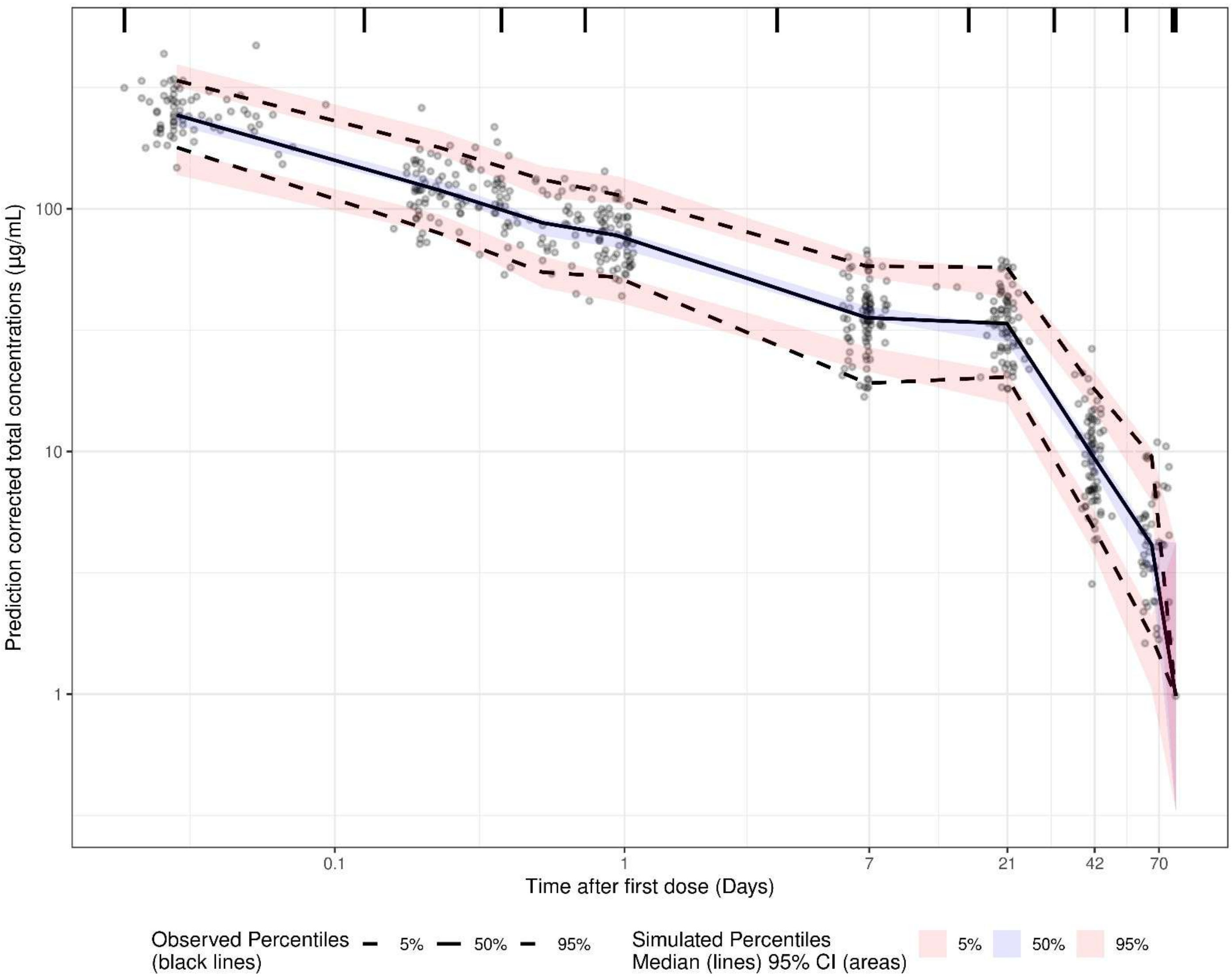

Figure 23: Final Model Diagnostics: Prediction-Corrected Visual Predictive Check for Fit on Unbound Plasma Dalbavancin Concentrations

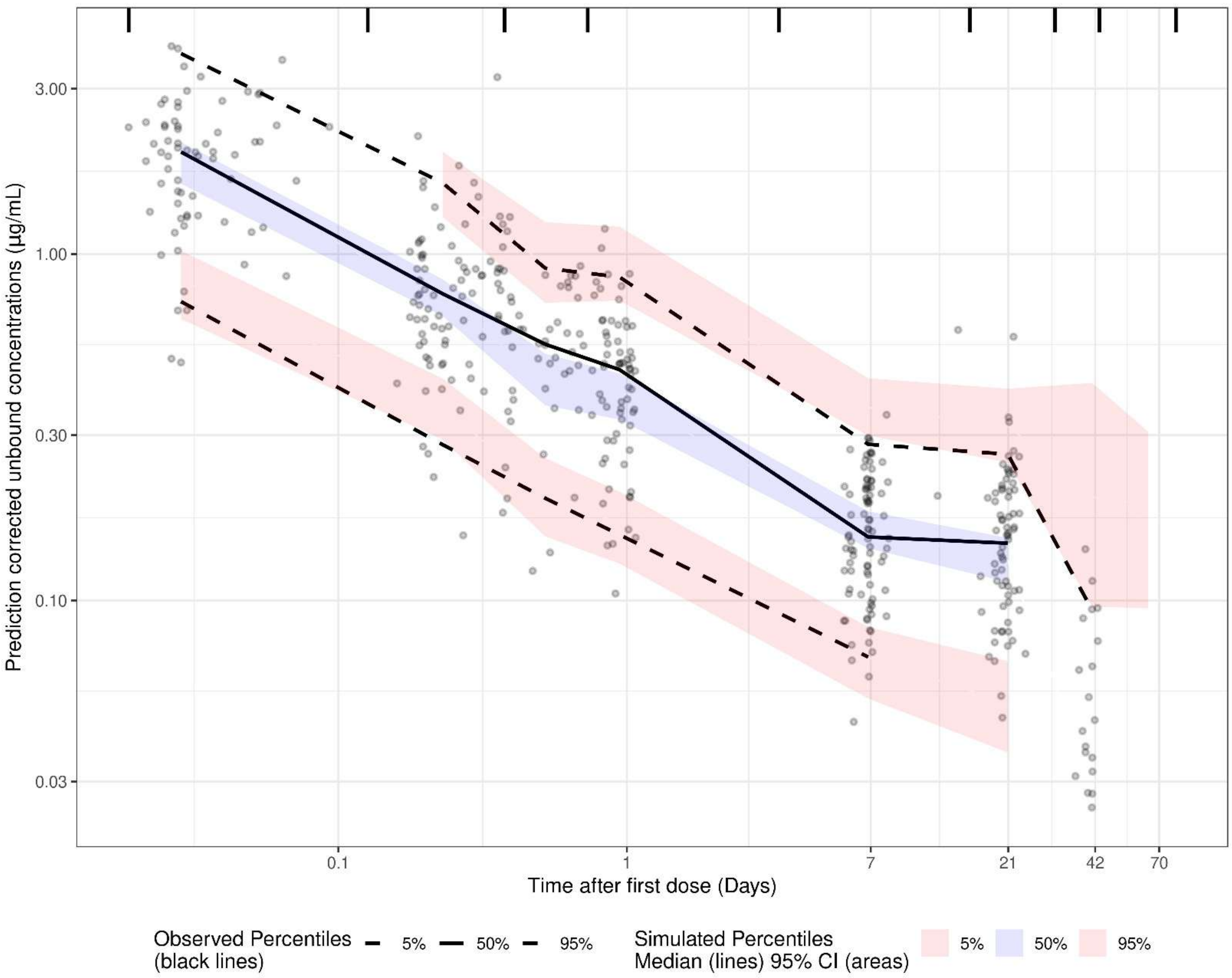

Figure 24: Final Pop-PK Model-predicted Typical and Individual Empirical Bayes Estimates versus Covariates

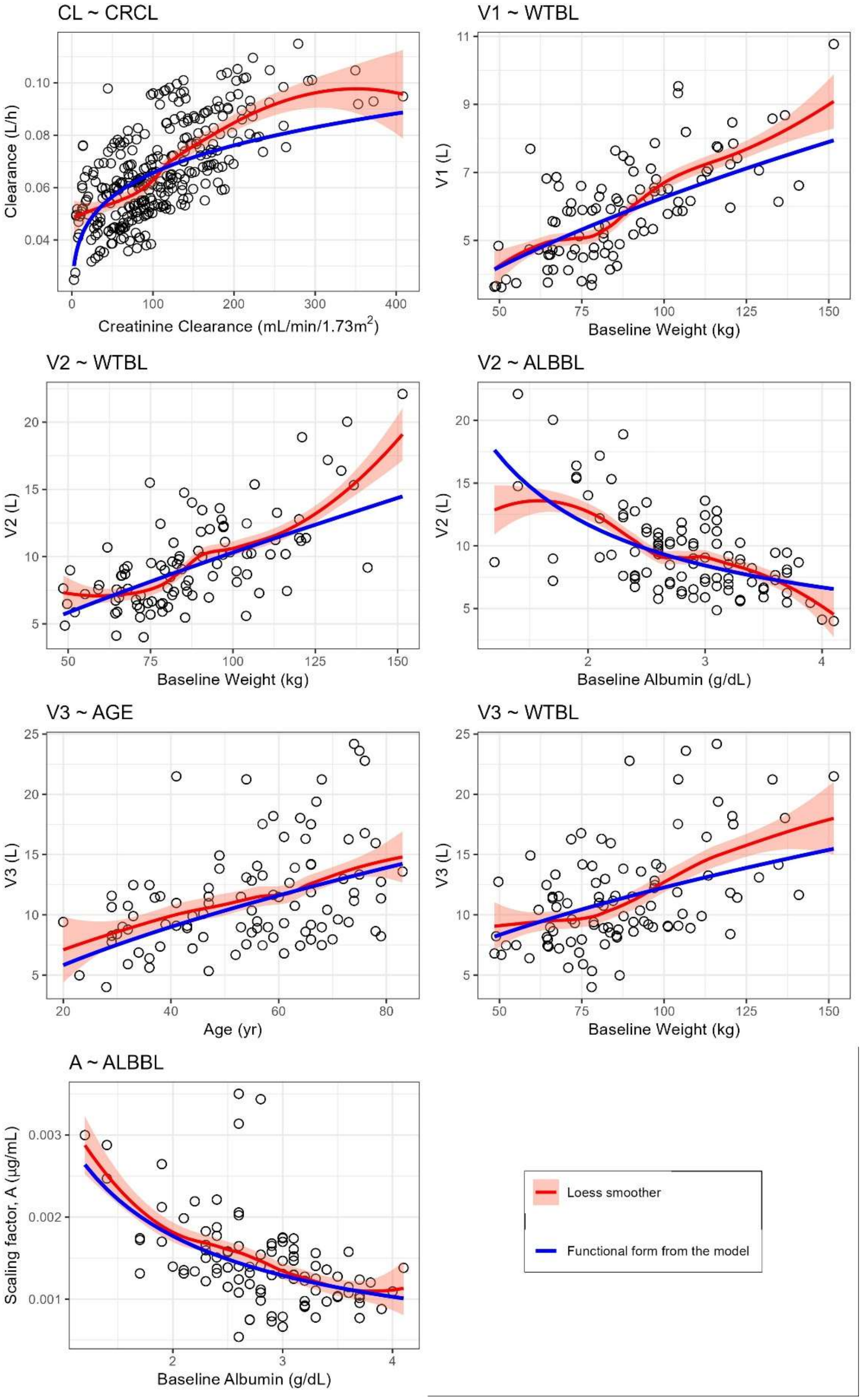

APPENDICES (SEPARATE DOCUMENTS)

Appendix B: Base PopPK Model - Results and Diagnostics plots (Pumas)

Appendix C: Full PopPK Model - Results and Diagnostics plots (Pumas)

Appendix D: Final Model Diagnostics: Plots of Observed, Individual Predicted, and Population Predicted Concentrations (µg/mL) of Total and Unbound Dalbavancin versus Time Since First Dose by Subject

Appendix E: Final PopPK Model - Results and Diagnostics plots (Pumas)

Task03: Dalbavancin Base PopPK Model - Results and Diagnostic plots

Final

Melanie Wilbaux Jessica Wojciechowski Daniel Selig

07-Jun-2024

Contents

1 BACKGROUND 2
1.1 Purpose 2
1.2 Executive Summary 2

2 INITIALIZE ANALYSIS 5
2.1 Initialize R Session 5
2.2 Load packages 5
2.3 Set plotting theme 6
2.4 Define functions 6

3 KEY MODEL BUILDING STEPS 6

4 IMPORT BASE MODEL RESULTS 7

5 PARAMETER ESTIMATES 7

6 DIAGNOSTIC PLOTS 9
6.1 DV vs. PRED 9
6.1.1 Linear scale 9
6.1.2 Log scale 10
6.2 DV vs. IPRED 11
6.2.1 Linear scale 11
6.2.2 Log scale 12
6.3 RES vs. Time after first dose 13
6.3.1 CWRES vs. Time 13
6.3.2 CWRES vs. Time (log scale) 14

6.3.3 IWRES vs. Time 15
6.3.4 IWRES vs. Time (log scale) 16
6.4 RES vs. Time from previous dose 17
6.4.1 CWRES vs. Time 17
6.4.2 CWRES vs. Time (log scale) 18
6.4.3 IWRES vs. Time 19
6.4.4 iWRES vs. Time (log scale) 20
6.5 RES vs. PRED 21
6.5.1 CWRES vs. PRED 21
6.5.2 IWRES vs. IPRED 22
6.5.3 |IWRES| vs IPRED 23
6.6 RES Distribution 24
6.6.1 CWRES hist 24
6.6.2 CWRES qqplot 25
6.6.3 IWRES hist 26
6.6.4 IWRES qqplot 27
6.7 BSV Distribution 28
6.7.1 Hist 28
6.7.2 QQplots 29
6.8 ETAs Correlations 30
6.9 BSV vs. Cov 31
6.9.1 Continuous covariates 32
6.9.2 Continuous covariates (Log scale) 32
6.9.3 Categorical covariates 34

7 INDIVIDUAL FITS **34**

8 REPRODUCIBILITY **35**

1 BACKGROUND

1.1 Purpose

The purpose of this script is to summarize the results from the dalbavancin base popPK model development and to generate diagnostic plots.

1.2 Executive Summary

Analysis Platform

All analyses were performed on the JuliaHub computing platform (version 6.3.0) hosted on the Amazon Web Services cloud computing platform. All modeling analyses were conducted using Pumas version 2.5.1 (PumasAI Inc, Dover, DE, USA). Analysis dataset preparation for Pumas, exploratory data analyses, and graphical and tabular summaries of modeling results were performed using R (version 4.2.3).

Base Model Development

The base popPK model development consisted of the determination of structural, between-subject variability (BSV), and residual unexplained variability (RUV) models to simultaneously fit the total and unbound plasma dalbavancin PK data.

An overview of the key model building steps was summarized in Section 3 (*Table 1 from ./results/03-base-model/*). The selection and adequacy of the model to describe the available data was assessed using standard diagnostic plots, Akaike Information Criteria (AIC), as well as condition number and precision of parameter estimates.

Based on prior dalbavancin PK modeling studies, a three-compartment model with zero-order input and first-order elimination was used as the initial structural PK model to describe total plasma PK [*ref Carrothers et al., 2020*]. A two-compartment model was also evaluated but resulted in a higher AIC (4110 vs. 3876) and showed bias in diagnostic plots for both total and unbound concentrations.

In the initial base model, the fraction of dalbavancin unbound (fu) was assumed to be a constant function of total dalbavancin concentration (Unbound = Total * Fu). This model did not properly describe the unbound concentrations, with under-prediction of high values. Various empirical non-linear relationships (e.g., exponential, logarithm, power, sigmoidal relationships) between unbound and total concentrations were evaluated. The best model, that resulted in the lowest AIC and best diagnostic plots, was a power relationship defined as follow:

Unbound = A * Total ^ K

With:

- Unbound [μg/mL]: unbound plasma concentrations;
- Total [μg/mL]: total plasma concentrations;
- A [μg/mL]: scaling factor that determines the magnitude of the relationship between total concentration and unbound concentration. It represents the baseline level of unbound concentration when the total concentration is equal to 1.
- B [unitless]: exponent that describes the shape of the relationship between total concentration and unbound concentration. The value of K influences how quickly unbound concentration changes in response to changes in total concentration.

A model with a saturable quasi-equilibirum protein binding function, in which the maximal binding capacity (Bmax) and binding dissociation constant (KD) are estimated, was also evaluated, but the most parsimonious model with the lowest AIC was the power relationship (AIC 3544 vs 3653).

The residual error was first modeled using a combined error model, but the additive part of the total concentrations was poorly estimated with high uncertainty. Thus, a proportional RUV model was selected for total concentrations and a combined RUV model was used for unbound concentrations. BLOQ data from unbound concentrations were modeled using a censored distribution, that places all the observations below LLOQ in a point mass at the LLOQ (M3 method). A model including the assumption that the measured concentrations cannot be negative was also evaluated (M4 method), but resulted in numerical and convergence issues.

Adding BSV on Q2, Q3 and K parameters resulted in over-parameterization, including convergence and numerical instability. BSV from all the other PK parameters were estimated using the exponential model and assuming a log-normal distribution. Correlations between BSV terms from each parameter were evaluated based on initial graphical exploration; and the best model included correlations between CL, V1, V3 and A. Correlations were incorporated into the base model to establish the most stable model possible before introducing covariates. Of note, they were then re-evaluated within the full model.

All the models were estimated using the second order Laplace approximation with interaction method in Pumas.

Base Model

To summarize, a model with three-compartments, zero-order input and first-order elimination was selected as the base model to describe total plasma PK. Unbound concentrations were assumed to be dependent on total concentrations with a power relationship. The popPK model was coded with parameters CL (clearance), V1 (volume of distribution in the central compartment), Q2 (intercompartmental clearance from compartment 1 to 2), V2 (volume of distribution in the second compartment), Q3 (intercompartmental clearance from compartment 1 to 3), V3 (volume of distribution in the third compartment), A (scaling factor) and K (exponent of the power). The RUV was modeled using a proportional error model for total concentrations and a combined error model for unbound concentrations. The BSV included log-normally distributed random effects on CL, V1, V2, V3 and A. The base popPK model did not include any covariate. The BSV on parameters, CL, V1, V3 and A was modeled using a four-dimensional matrix taking into account correlations among the parameters.

Parameter estimates from the base model were presented in Section 5 (*Table 2 from ./results/03-base-model/*). All parameters from the base model were well estimated, with acceptable RSE. Population clearance and central volume of distribution were estimated at 0.063 L/hr and 5.65 L, respectively, with moderate variability (CV% = 30.2% and 26.4%, respectively). Q2 and Q3 were estimated at 0.026 L/hr and 0.942 L/hr, respectively. V2 and V3 were estimated at 8.98 L and 10.7 L, respectively, with moderate variability (CV% = 33.5% and 38.9%, respectively). A was estimated at 0.00135 μg/mL with a moderate variability (CV% = 38.3%) and K at 1.32, showing that the unbound vs total concentrations relationship exhibits a curvature. Strongest correlations were estimated between V1 and V3 (73.3%) and between CL and V1 (66.2%). All eta and epsilon shrinkage were acceptable. The

condition number, calculated as the ratio between the largest and smallest eigenvalues of the correlation matrix, was found to be 84, indicating that the base popPK model was stable and not over-parameterized (as the value is < 1000).

All the diagnostic plots from Section 6 suggested that the base popPK model described the total and unbound PK data adequately. The observed concentration vs. model predictions (population and individual) plots demonstrated a reasonable agreement between data and model predictions. The residual plots do not show any strong pattern with either time or concentration, and closely resembles a normal distribution. A slight trend in the residuals of unbound concentrations at later time points was observed; but it did not raise concerns as it only pertained to a few points, and all other diagnostic plots were acceptable.

Exploratory Analysis of Parameter-Covariate Relationships

The random effects of PK parameters vs. covariates plot in Section 6.9 showed trend between:

- BSV on CL and age, body-size related covariates, albumin, CrCl and sex;
- BSV on V1 and body-size related covariates, albumin, and CrCl;
- BSV on V2 and albumin, and CrCl;
- BSV on V3 and body-size related covariates and albumin;
- BSV on A albumin.

2 INITIALIZE ANALYSIS

2.1 Initialize R Session

Initialize the global R environment and result directories.

```
[1] "/mnt/data/code/SSC/Emmes/Emmes-Dalbavancin-DOTS"
```

2.2 Load packages

```
[1] "/mnt/data/code/SSC/Emmes/Emmes-Dalbavancin-DOTS/renv/library/R-4.2/x86_64-pc-linux-gnu"
[2] "/mnt/data/.cache/R/renv/sandbox/R-4.2/x86_64-pc-linux-gnu/e11edd0e"
```

2.3 Set plotting theme

2.4 Define functions

3 KEY MODEL BUILDING STEPS

Model	Description	Ref	nparam	AIC	AIC_ref	deltaAIC_fromRef	Comments
Base10	Initial base model	/	16	3,875.8			Under-predictions of high unbound concentrations
Base11	2 compartments	Base10	14	4,110.1	3,875.8	234.3	Total and unbound concentrations not properly described
Base12	Exponential relationship Unbound vs Total	Base10	17	3,879.8	3,875.8	3.9	Under-predictions of high unbound concentrations
Base13	Power relationship Unbound vs Total	Base10	17	3,544.5	3,875.8	-331.3	Ok. High RSE additive RUV
Base14	Logarithm relationship Unbound vs Total	Base10	16	5,149.4	3,875.8	1,273.5	Under-predictions of high unbound concentrations
Base15	Sigmoidal relationship Unbound vs Total	Base10	19	3,545.9	3,875.8	-329.9	Overparameterized
Base17	Protein binding relationship Unbound vs Total	Base10	17	3,652.9	3,875.8	-223.0	Ok
Base100	BLOQ M4 method	Base13	17	3,544.5	3,544.5	-0.0	Numerical and convergence issues.
Base101	Proportional RUV for Total	Base13	16	3,543.6	3,544.5	-0.9	Ok
Base102	Proportional RUV for Unbound	Base101	15	3,550.9	3,543.6	7.3	High epsilon shrinkage
Base103	Add BSV on Q2	Base101	17	3,533.4	3,543.6	-10.2	Overparameterized
Base104	Add BSV on Q3	Base101	17	3,705.4	3,543.6	161.8	Overparameterized
Base105	Add BSV on k	Base101	17	3,684.8	3,543.6	141.1	Overparameterized
Base106	Corr V1-V3	Base101	17	3,520.9	3,543.6	-22.7	Ok
Base107	Corr CL-V1-V3	Base106	19	3,493.8	3,520.9	-27.1	Ok
Base108	Corr CL-V1-V2-V3	Base107	22	3,517.3	3,493.8	23.5	Overparameterized
Base109	Corr V1-V3 and CL-V2	Base106	18	3,511.2	3,520.9	-9.7	Ok
Base110	Corr CL-V1-V3-A	Base107	22	3,478.5	3,493.8	-15.3	Best base model
Base111	Corr CL-V1-V2-V3-A	Base110	26	3,520.5	3,478.5	42.0	Overparameterized

! Important

The best base popPK model is the Base110:

- 3-compartment model, zero-order input and first-order elimination;
- Unbound concentrations assumed to be dependent on total concentrations with a power relationship;
- RUV modeled using a proportional error model for total concentrations and a combined error model for unbound concentrations;
- BSV on CL, V1, V2, V3 and A;
- Correlations between CL, V1, V3 and A.

4 IMPORT BASE MODEL RESULTS

Note

The dalbavancin Base popPK model was executed in the Pumas script: ./programs/pumas/base/base110.jl, to generate the output csv files.

5 PARAMETER ESTIMATES

Table 2: Parameter estimates - Base Model

Parameter	Description	Estim	RSE (%)	95% CI	CV (%)	Corr (%)	Shrinkage (%)
tvcl	Clearance (L/hr)	0.063	3.2	[0.0591 ; 0.067]	-	-	-
tvv1	Volume of Distribution in the central cmt (L)	5.65	3.2	[5.3 ; 6.01]	-	-	-
tvq2	Intercompartmental Clearance from cmt 1 to 2 (L/hr)	0.026	9	[0.0214 ; 0.0305]	-	-	-
tvv2	Volume of Distribution of 2nd cmt (L)	8.98	6.5	[7.84 ; 10.1]	-	-	-
tvq3	Intercompartmental Clearance from cmt 1 to 3 (L/hr)	0.942	6.1	[0.829 ; 1.05]	-	-	-
tvv3	Volume of Distribution of 3rd cmt (L)	10.7	4.4	[9.73 ; 11.6]	-	-	-
tva	Intercept of power when ctot=1	0.00135	8.9	[0.00111 ; 0.00158]	-	-	-

Parameter	Description	Estim	RSE (%)	95% CI	CV (%)	Corr (%)	Shrinkage (%)
tvk	Exponent of the power function	1.32	1.4	[1.29 ; 1.36]	-	-	-
Ω ,	BSV V2	0.112	26.1	[0.0547 ; 0.17]	33.5	-	33.1
Ω_CLV1V3A ,	BSV CL	0.0913	15	[0.0644 ; 0.118]	30.2	-	3.5
Ω_CLV1V3A ,	Corr CL-V1	0.0528	17.7	[0.0344 ; 0.0711]	-	66.2	-
Ω_CLV1V3A ,	Corr CL-V3	0.0495	21.4	[0.0288 ; 0.0703]	-	42.3	-
Ω_CLV1V3A ,	Corr CL-A	0.05	26.7	[0.0238 ; 0.0762]	-	43.3	-
Ω_CLV1V3A ,	BSV V1	0.0695	20.4	[0.0417 ; 0.0973]	26.4	-	10.4
Ω_CLV1V3A ,	Corr V1-V3	0.0749	18.2	[0.0482 ; 0.102]	-	73.3	-
Ω_CLV1V3A ,	Corr V1-A	0.042	27.5	[0.0194 ; 0.0646]	-	41.7	-
Ω_CLV1V3A ,	BSV V3	0.151	18.8	[0.0949 ; 0.206]	38.9	-	8.9
Ω_CLV1V3A ,	Corr V3-A	0.0559	39	[0.0132 ; 0.0986]	-	37.6	-
Ω_CLV1V3A ,	BSV A	0.147	18	[0.0948 ; 0.198]	38.3	-	5.1
_proptot	Proportional Total RUV	0.13	6.7	[0.113 ; 0.147]	-	-	-
_propfree	Proportional Unbound RUV	0.19	6.6	[0.166 ; 0.215]	-	-	-
_addfree	Additive Unbound RUV	0.0126	16.7	[0.00844 ; 0.0167]	-	-	-
Epsilon shrinkage Total	-	-	-	-	-	-	19.7
Epsilon shrinkage Unbound	-	-	-	-	-	-	9.6

Abbreviations: BSV = between-subject variability; CI = confidence interval; CV = coefficient of variation; RSE = relative standard error; RUV = residual unexplained variability.

Notes:

- The condition number is equal to 84.
- RSE % was derived as: se/estimate*100.
- Estimates for BSV are the variances.
- The CV % was derived as: sqrt(var)*100.

6 DIAGNOSTIC PLOTS

Note

- The diagnostic plots are not stratified by dose group, as the number of patients are too limited in the groups different from Dalbavancin 1500 mg + 1500 mg.
- BLOQ values for unbound concentrations (<0.05 μg/mL) are removed for all the diagnostic plots.

6.1 DV vs. PRED

6.1.1 Linear scale

Observed vs. Population predicted dalbavancin concentrations - Base Model

Note: Solid black lines represent the lines of identity, and red lines the linear regression lines.

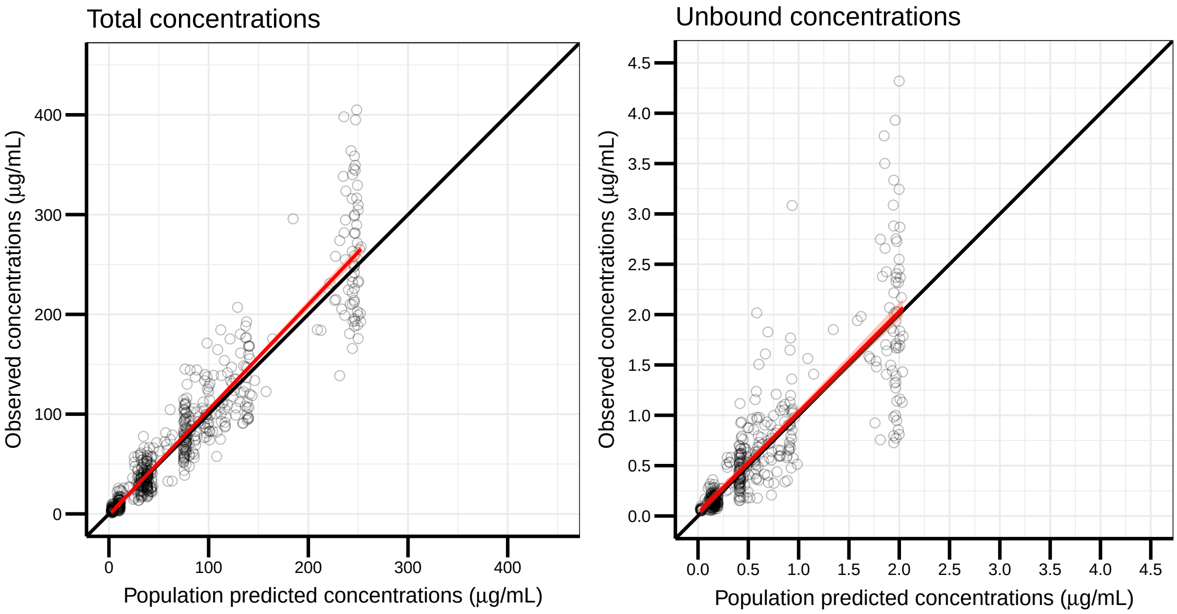

6.1.2 Log scale

Observed vs. Population predicted dalbavancin concentrations - Base Model

Note: Solid black lines represent the lines of identity, and red lines the linear regression lines.

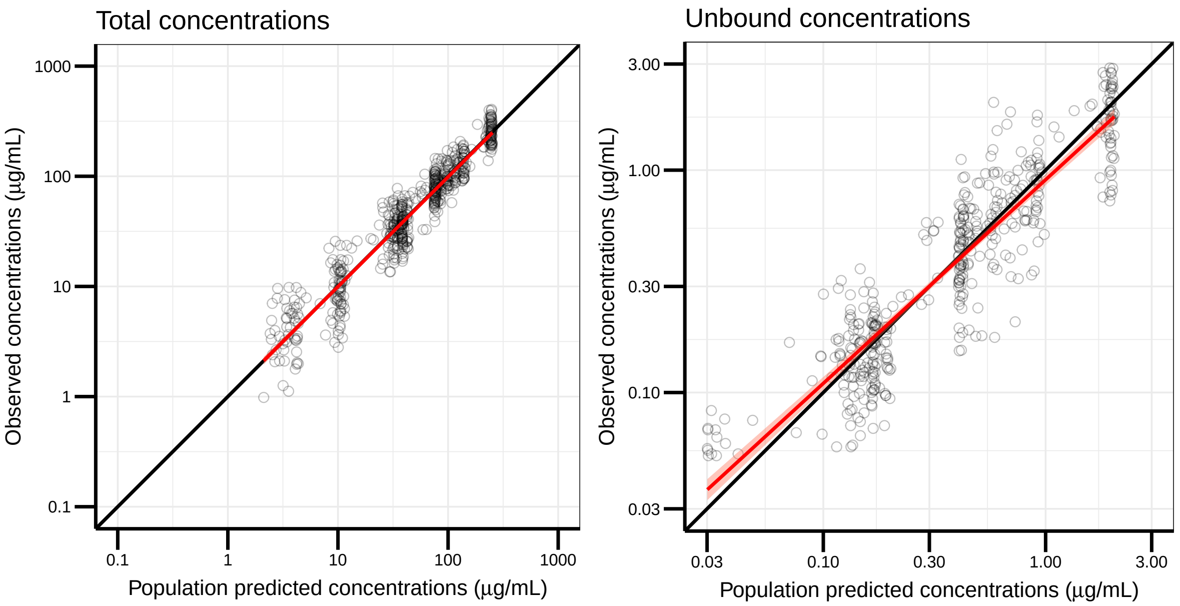

6.2 DV vs. IPRED

6.2.1 Linear scale

Observed vs. Individual predicted dalbavancin concentrations - Base Model

Note: Solid black lines represent the lines of identity, and red lines the linear regression lines (with 95% CI).

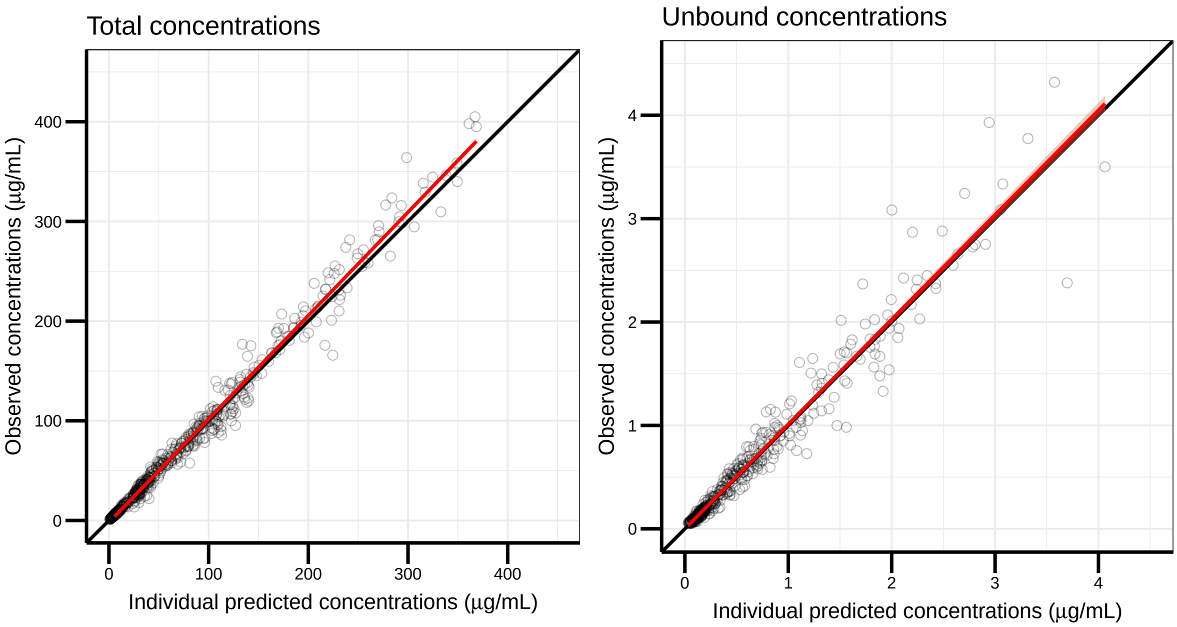

6.2.2 Log scale

Observed vs. Individual predicted dalbavancin concentrations - Base Model

Note: Solid black lines represent the lines of identity, and red lines the linear regression lines (with 95% CI).

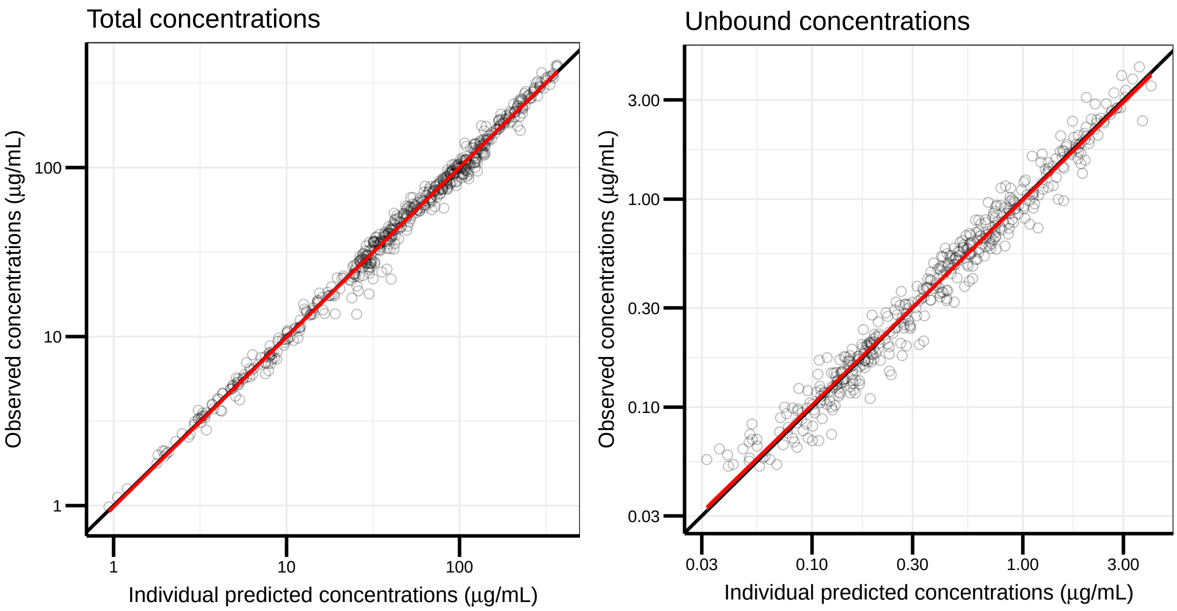

6.3 RES vs. Time after first dose

6.3.1 CWRES vs. Time

Conditional Weighted Residuals (CWRES) vs. Time after first dose - Base Model

Note: Solid black lines represent the lines of identity, and red curves the lowess smooth regression curves (with 95% CI).

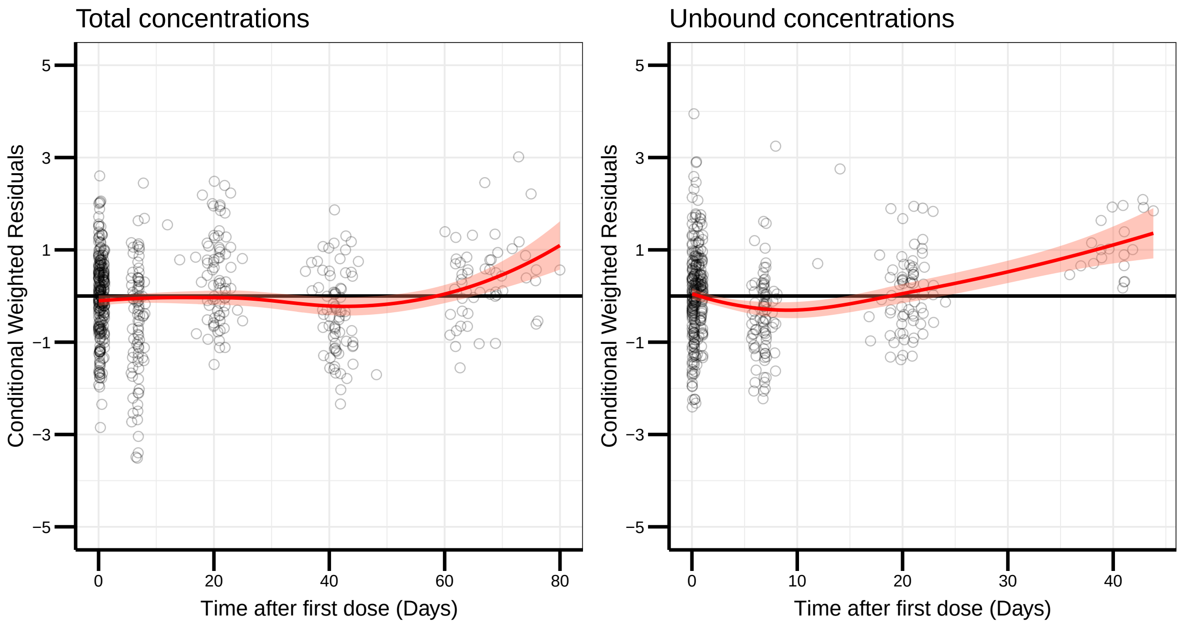

6.3.2 CWRES vs. Time (log scale)

Conditional Weighted Residuals (CWRES) vs. Time after first dose - Base Model

Note: Solid black lines represent the lines of identity, and red curves the lowess smooth regression curves (with 95% CI).

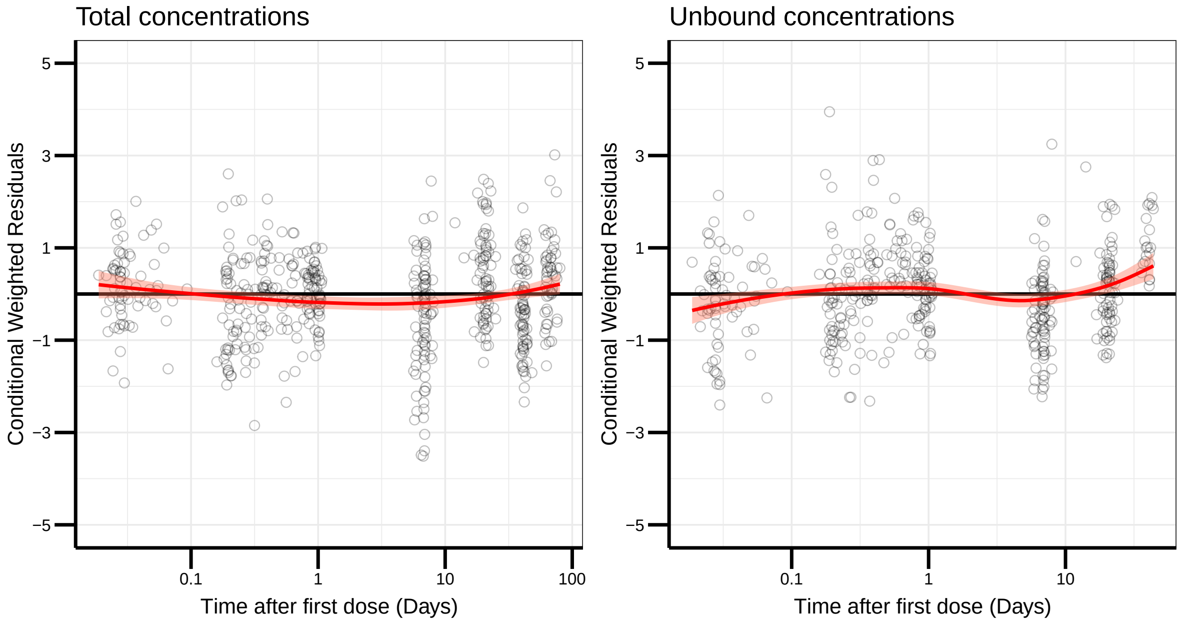

6.3.3 IWRES vs. Time

Individual Weighted Residuals (IWRES) vs. Time after first dose - Base Model

Note: Solid black lines represent the lines of identity, and red curves the lowess smooth regression curves (with 95% CI).

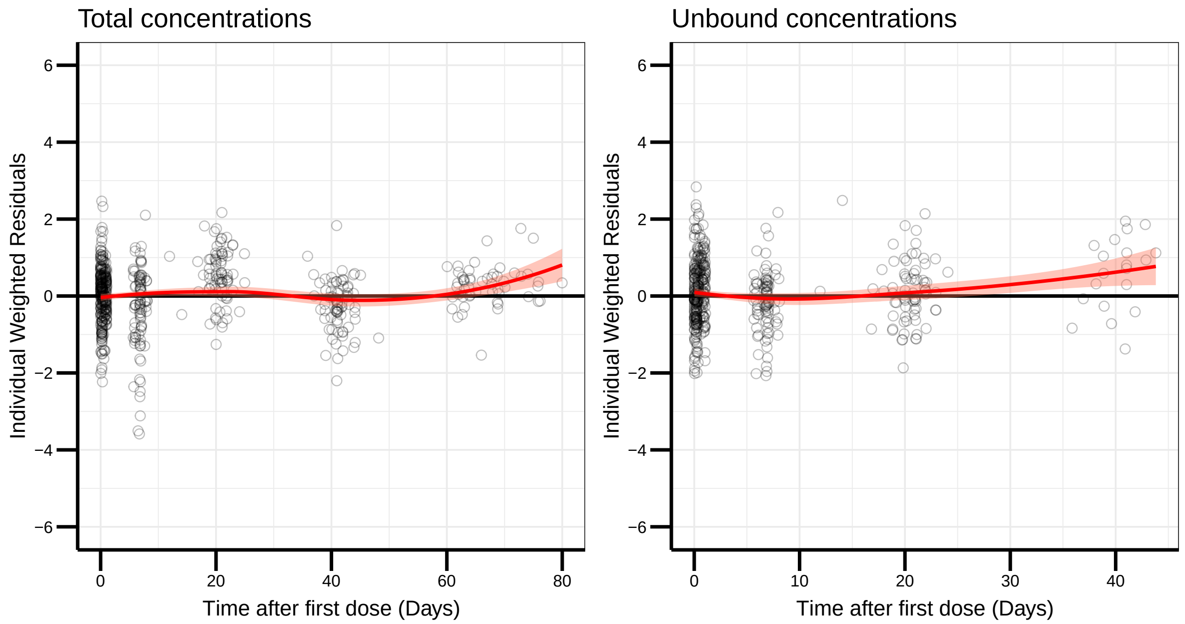

6.3.4 IWRES vs. Time (log scale)

Individual Weighted Residuals (IWRES) vs. Time after first dose - Base Model

Note: Solid black lines represent the lines of identity, and red curves the lowess smooth regression curves (with 95% CI).

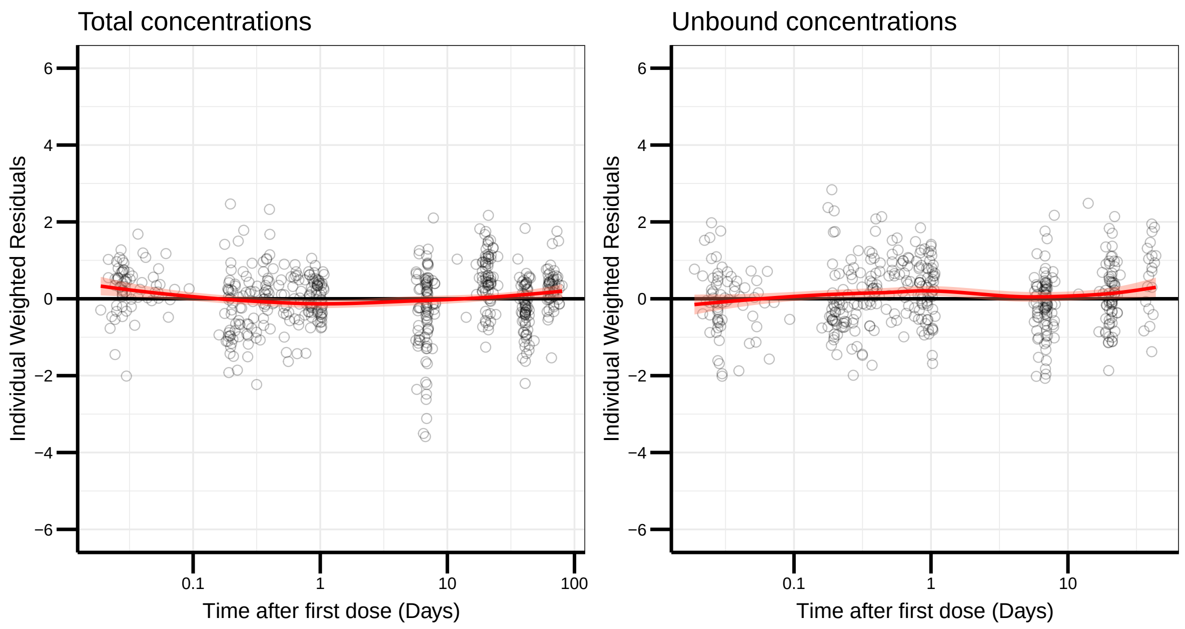

6.4 RES vs. Time from previous dose

6.4.1 CWRES vs. Time

Conditional Weighted Residuals (CWRES) vs. Time from previous dose - Base Model

Note: Solid black lines represent the lines of identity, and red curves the lowess smooth regression curves (with 95% CI).

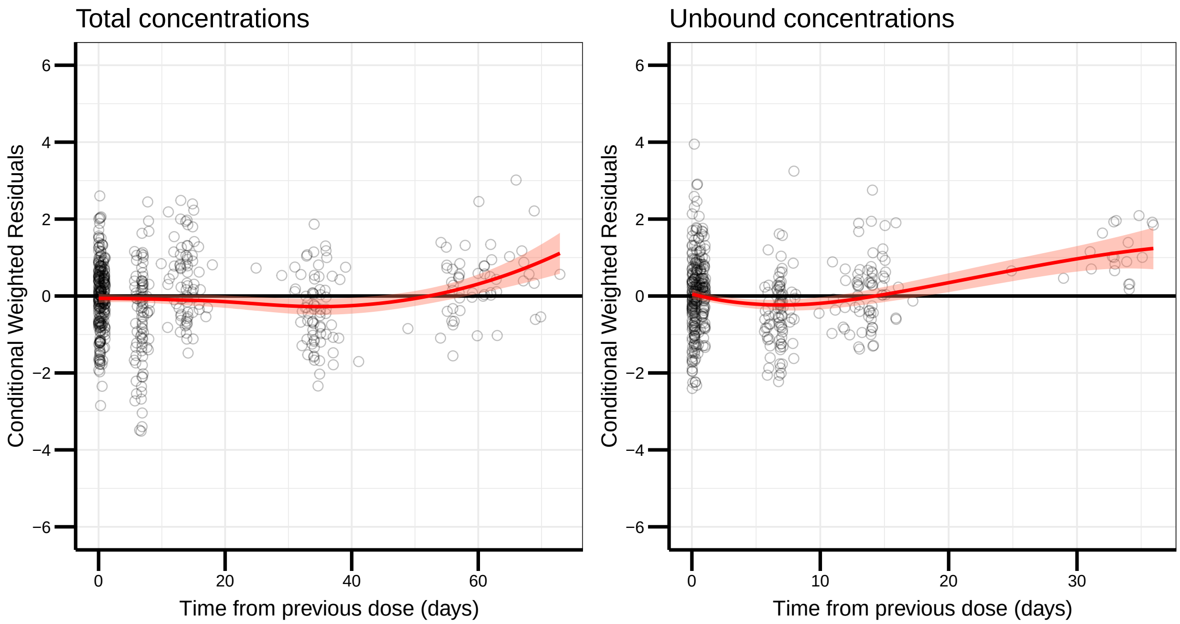

6.4.2 CWRES vs. Time (log scale)

Conditional Weighted Residuals (CWRES) vs. Time from previous dose - Base Model

Note: Solid black lines represent the lines of identity, and red curves the lowess smooth regression curves (with 95% CI).

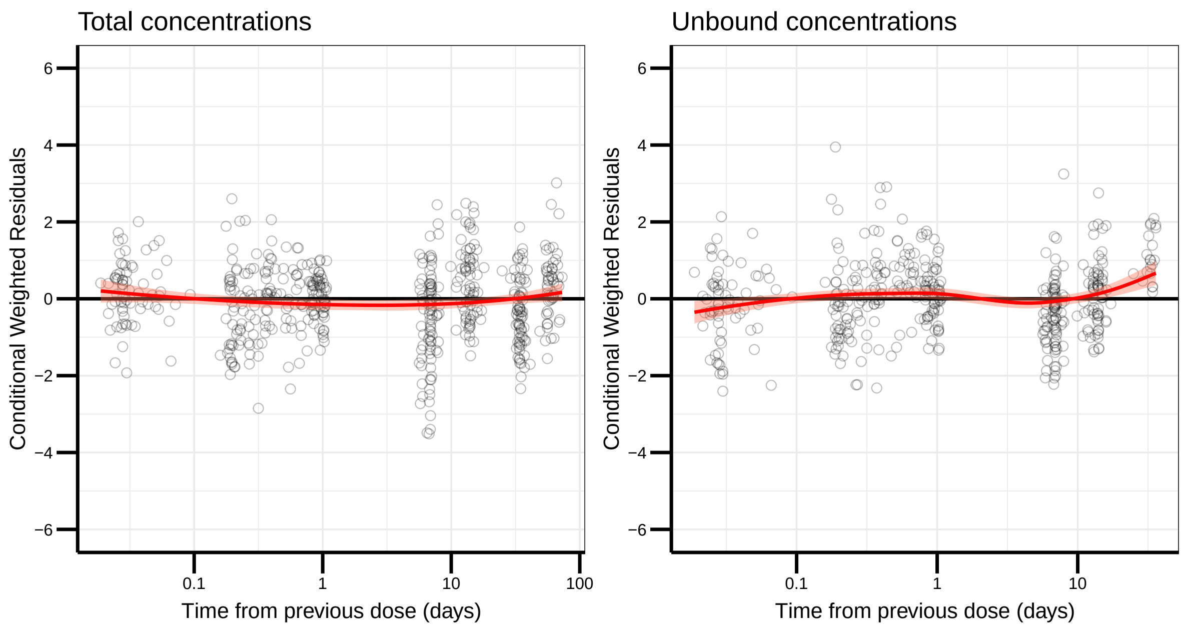

6.4.3 IWRES vs. Time

Individual Weighted Residuals (IWRES) vs. Time from previous dose - Base Model

Note: Solid black lines represent the lines of identity, and red curves the lowess smooth regression curves (with 95% CI).

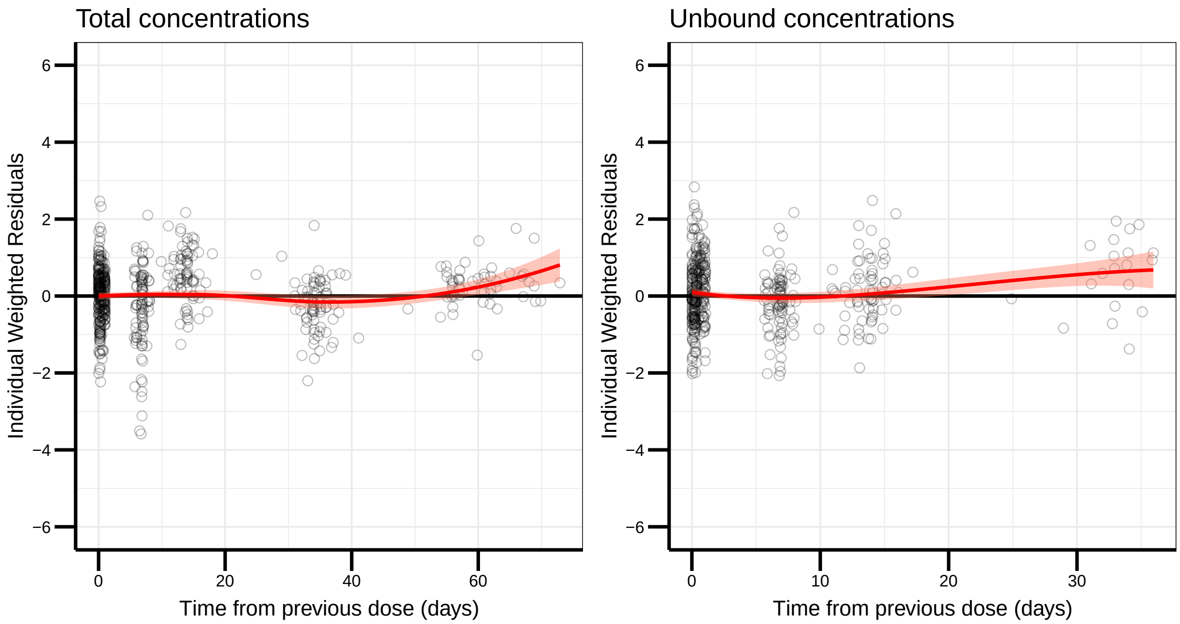

6.4.4 iWRES vs. Time (log scale)

Individual Weighted Residuals (IWRES) vs. Time from previous dose - Base Model

Note: Solid black lines represent the lines of identity, and red curves the lowess smooth regression curves (with 95% CI).

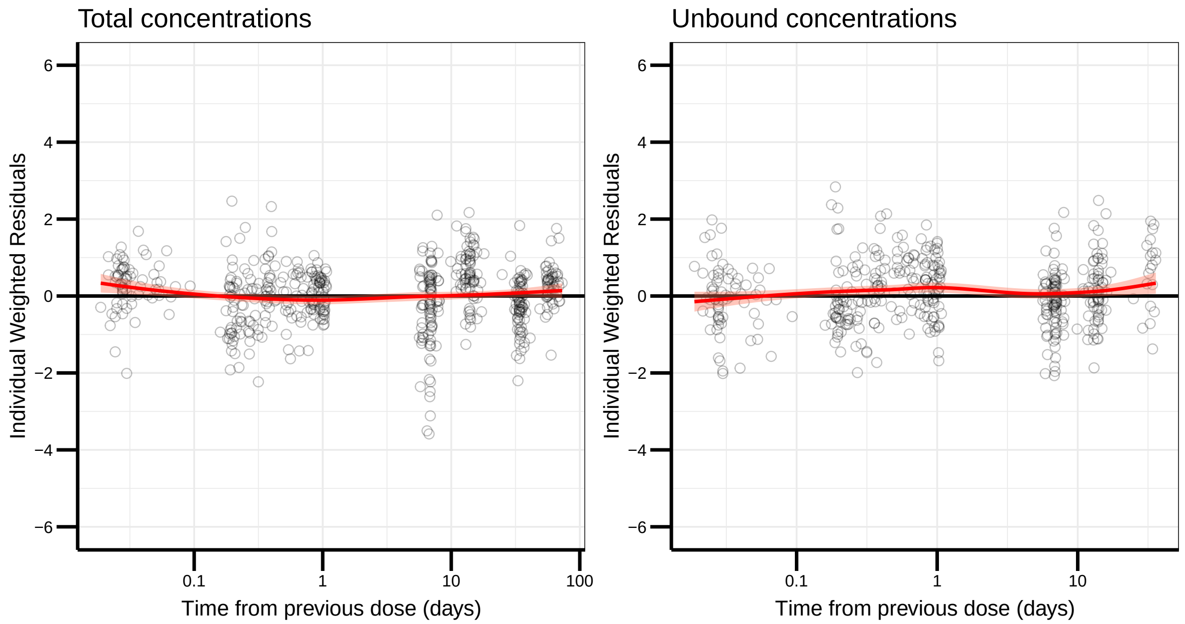

6.5 RES vs. PRED

6.5.1 CWRES vs. PRED

Conditional Weighted Residuals (CWRES) vs. Population predictions - Base Model

Note: Solid black lines represent the lines of identity, and red curves the lowess smooth regression curves (with 95% CI).

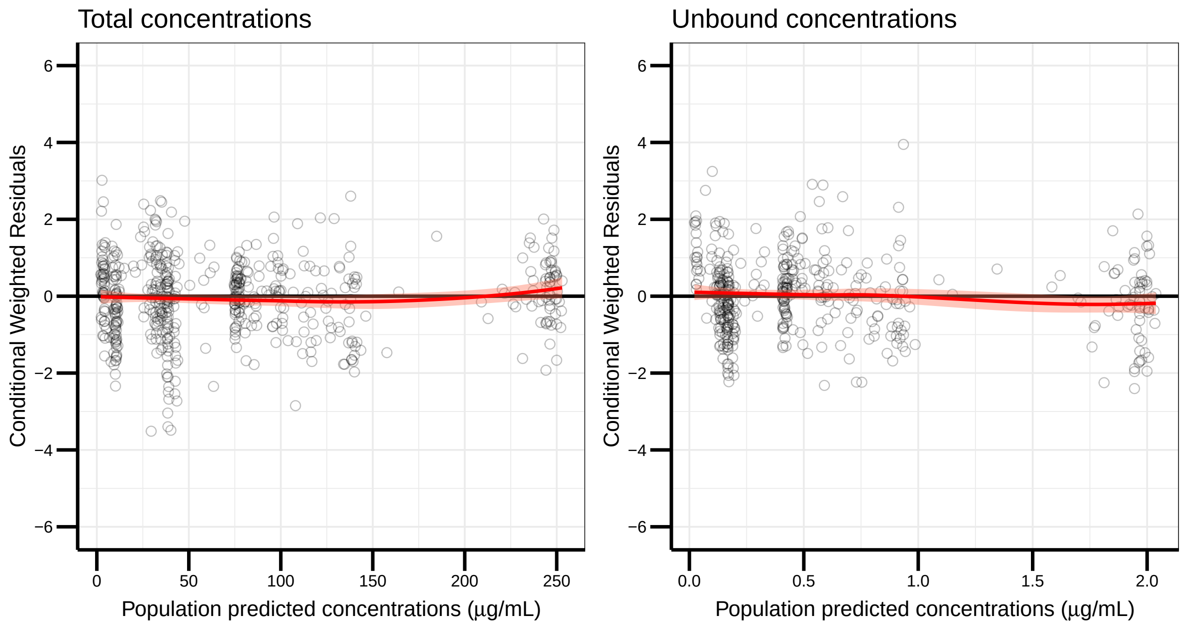

6.5.2 IWRES vs. IPRED

Individual Weighted Residuals (IWRES) vs. Individual predictions - Base Model

Note: Solid black lines represent the lines of identity, and red curves the lowess smooth regression curves (with 95% CI).

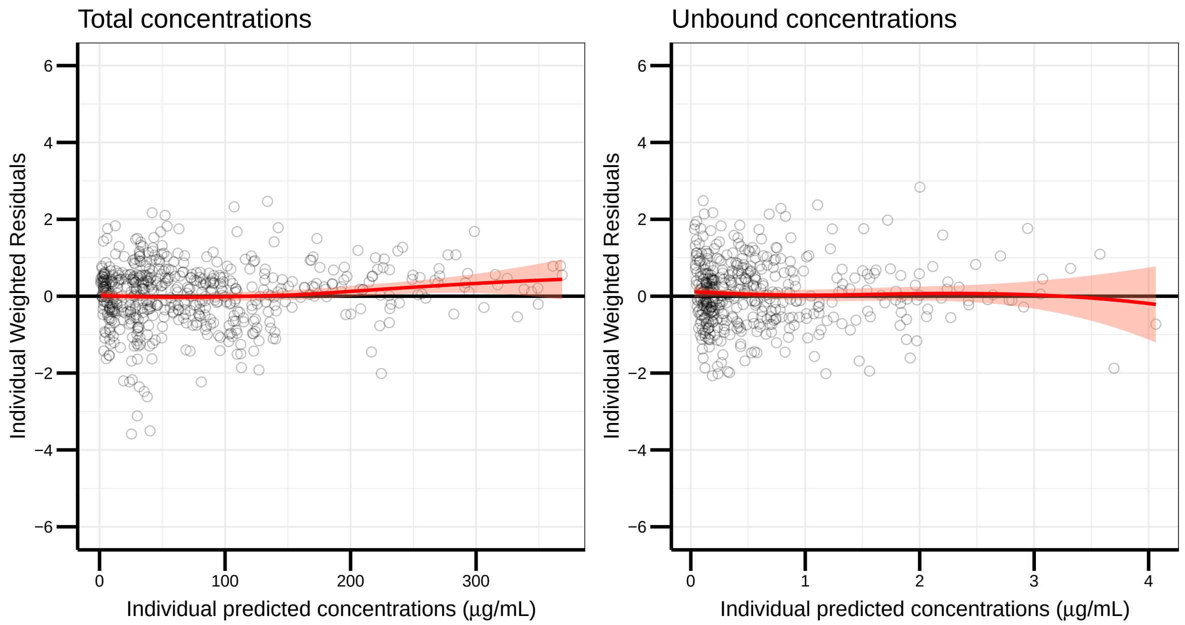

6.5.3 |IWRES| vs IPRED

|Individual Weighted Residuals| (|IWRES|) vs. Individual predictions - Base Model

Note: The red curves are the lowess smooth regression curves (with 95% CI).

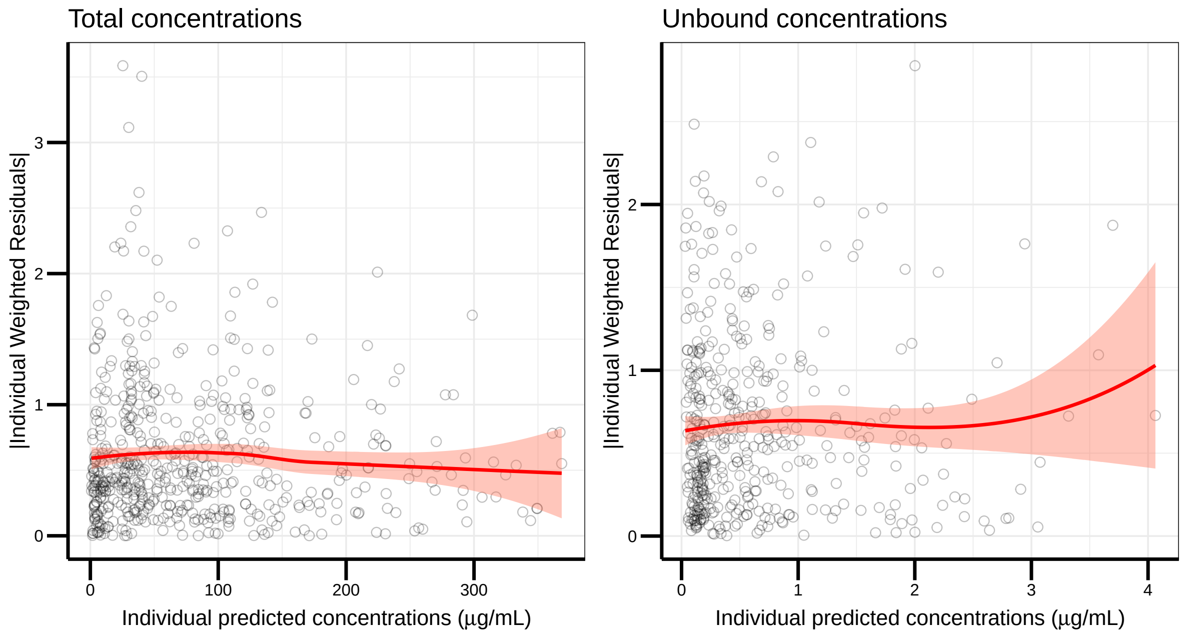

6.6 RES Distribution

6.6.1 CWRES hist

Distribution of Conditional Weighted Residuals (CWRES)

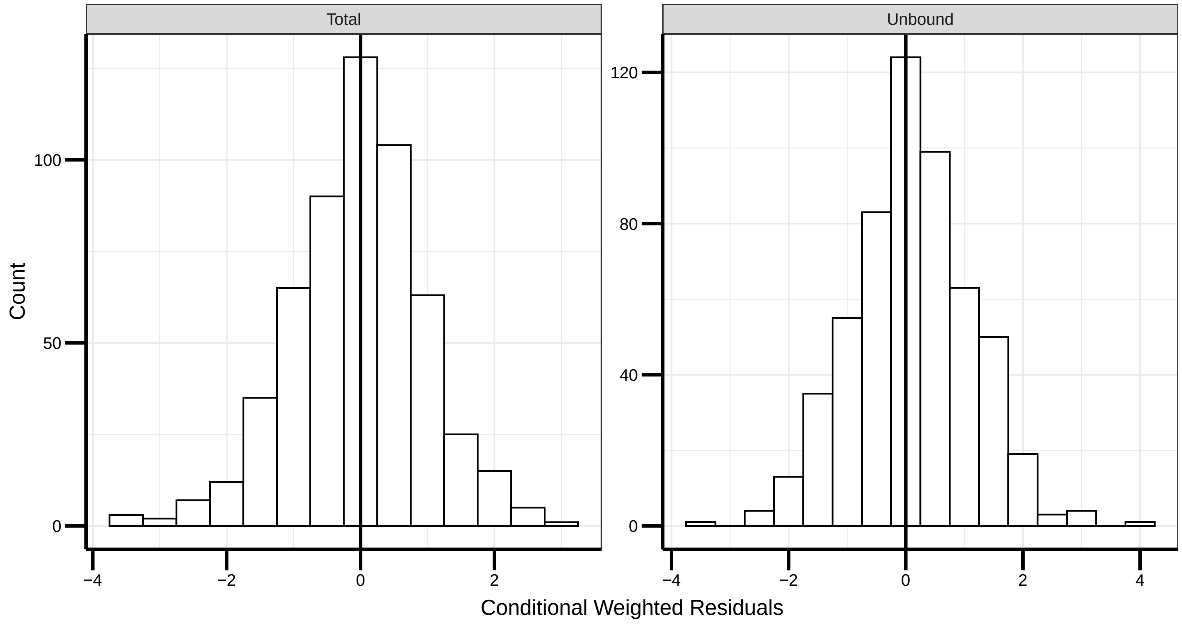

6.6.2 CWRES qqplot

QQplot of Conditional Weighted Residuals (CWRES)

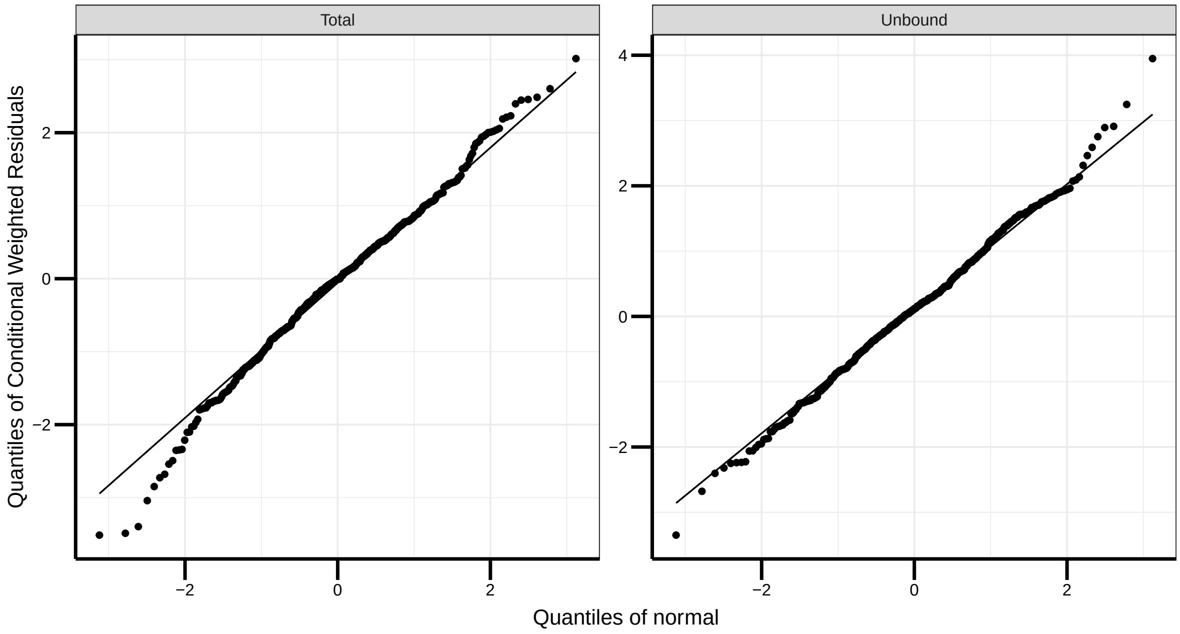

6.6.3 IWRES hist

Distribution of Individual Weighted Residuals (IWRES)

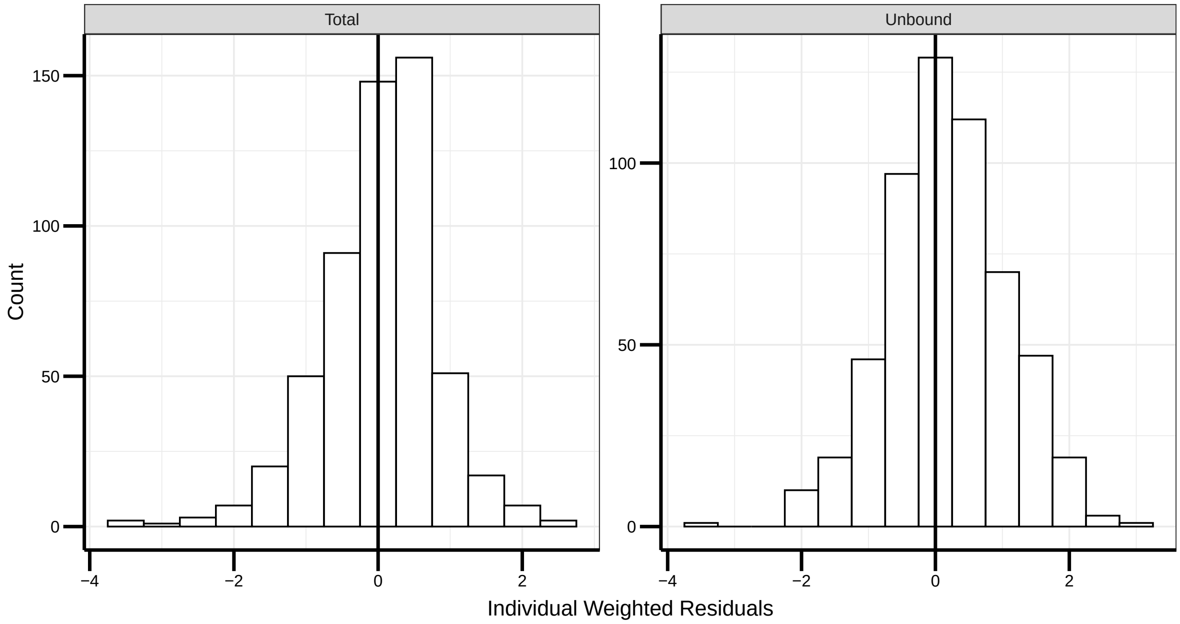

6.6.4 IWRES qqplot

QQplot of Individual Weighted Residuals (IWRES)

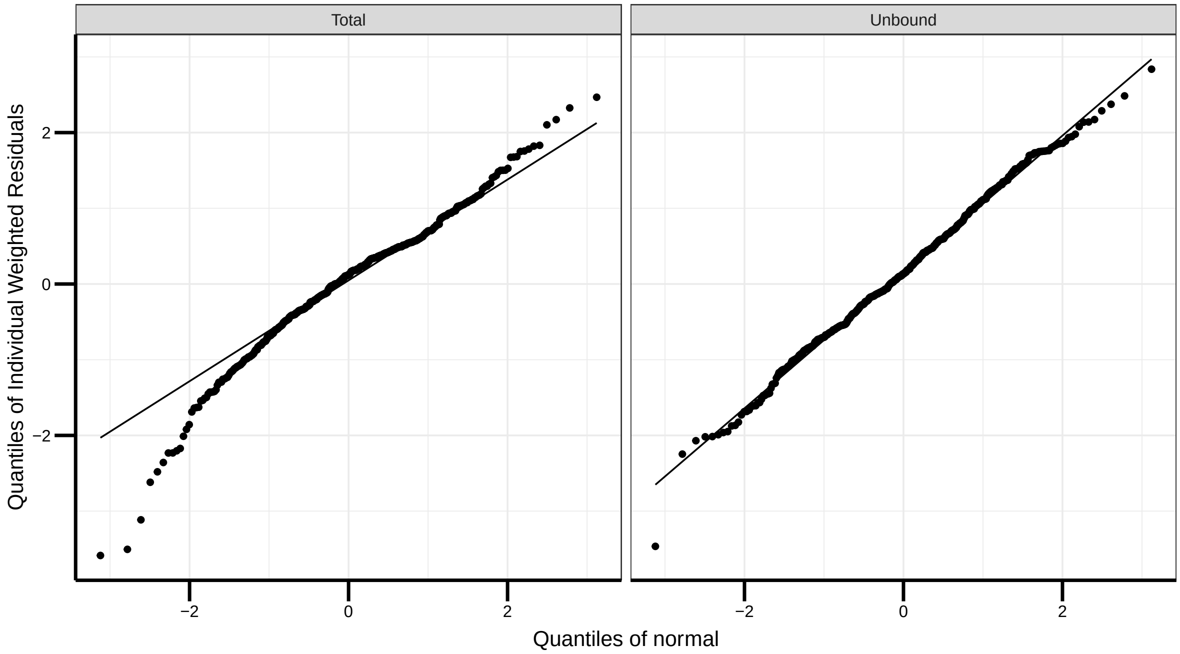

6.7 BSV Distribution

6.7.1 Hist

Distribution of between-subject variability (ETAs)

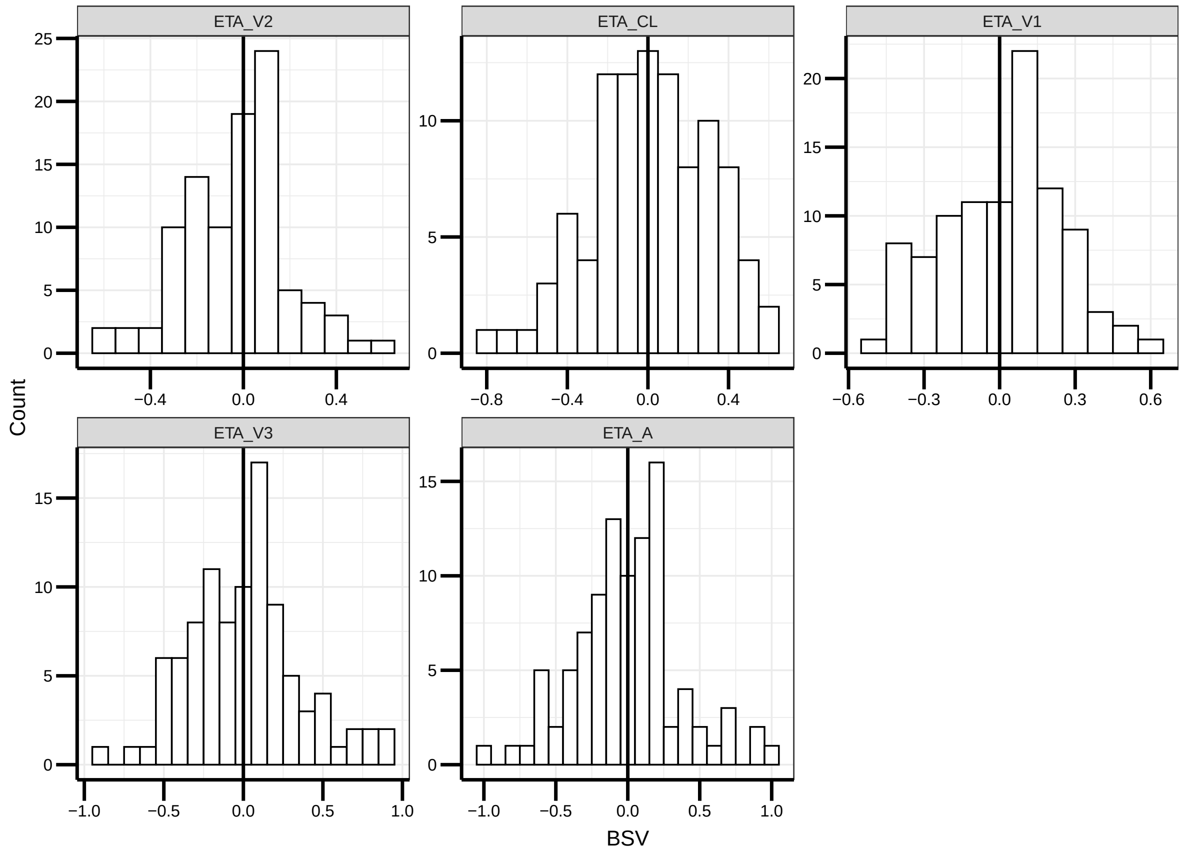

6.7.2 QQplots

QQplot of between-subject variability (ETAs)

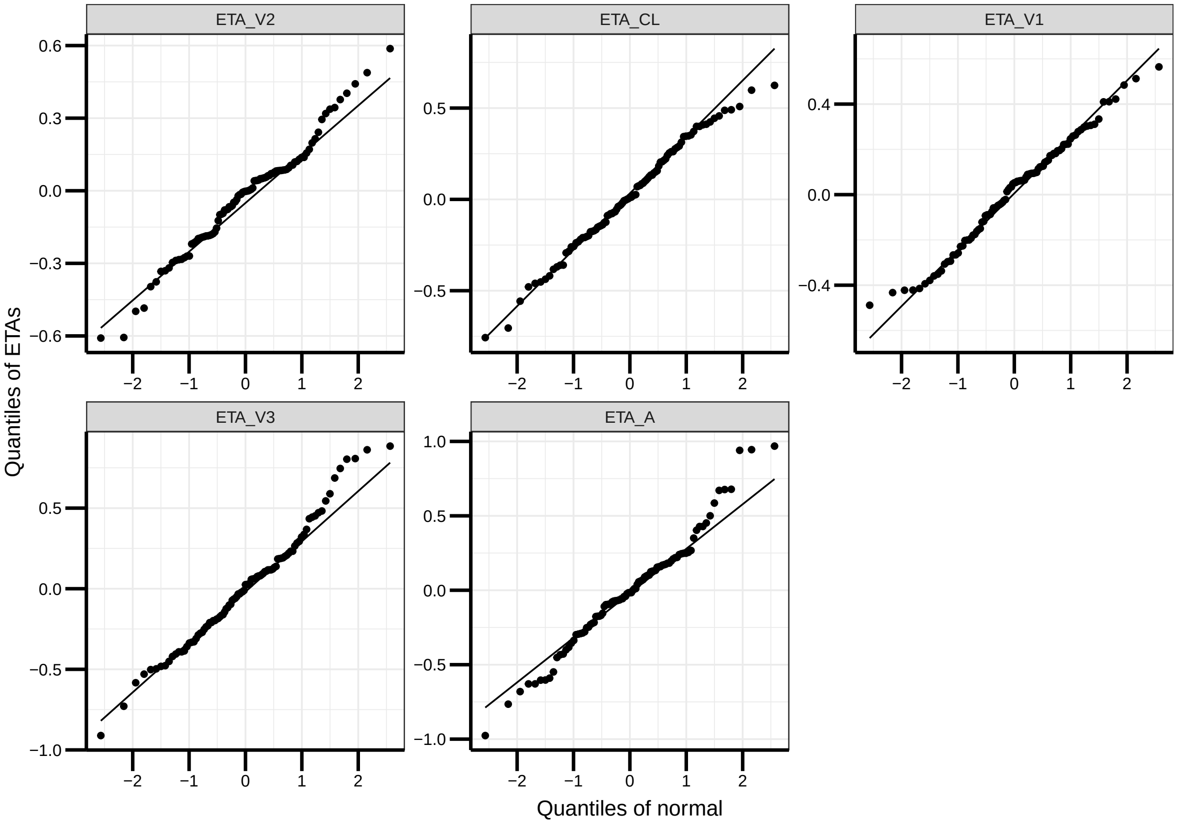

6.8 ETAs Correlations

Correlations of between-subject variability (ETAs)

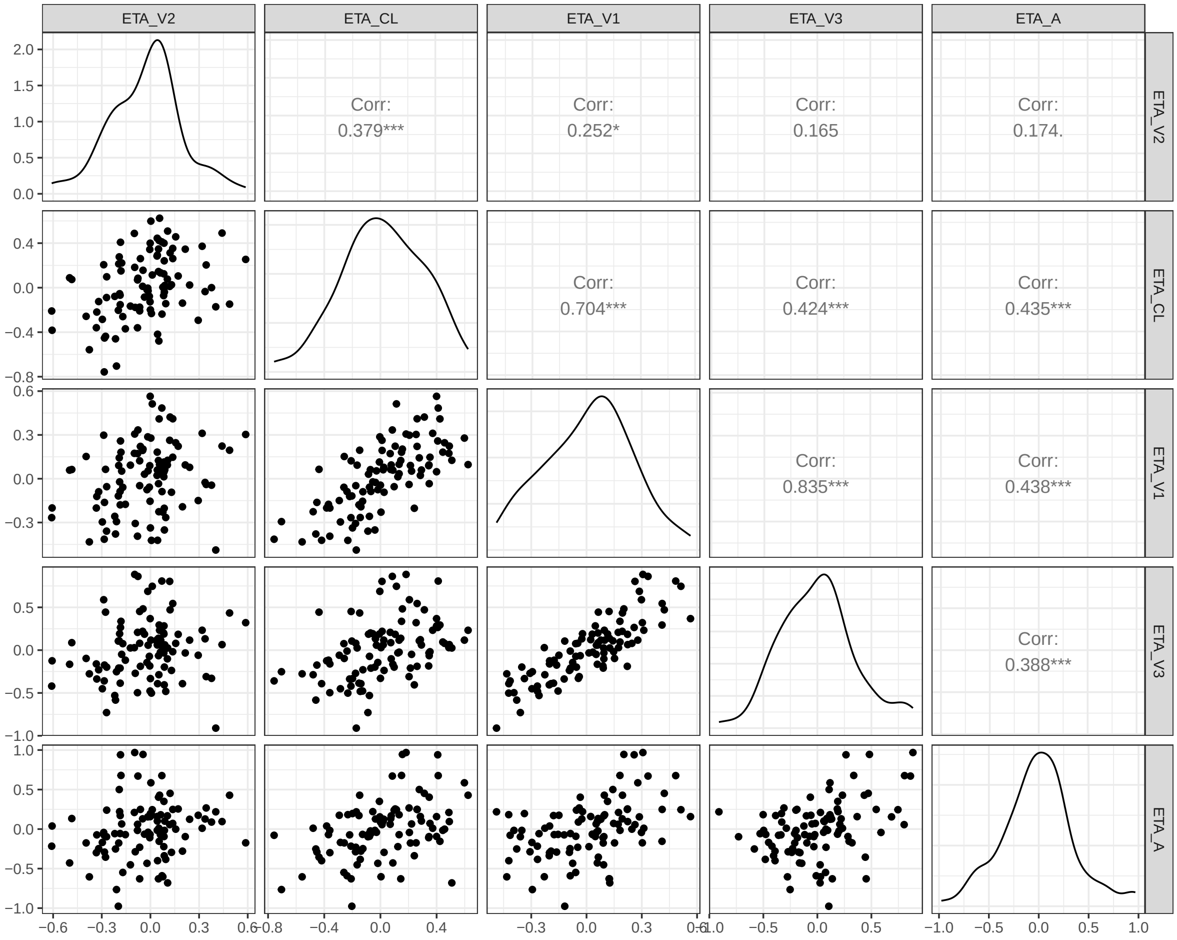

6.9 BSV vs. Cov

> **Note**
>
> - Lab values correspond to baseline values.

Between-subject variability (ETAs) vs. continuous and categorical covariates

Note: Red curves correspond to the lowess smooth regression curves (with 95% CI).

6.9.1 Continuous covariates

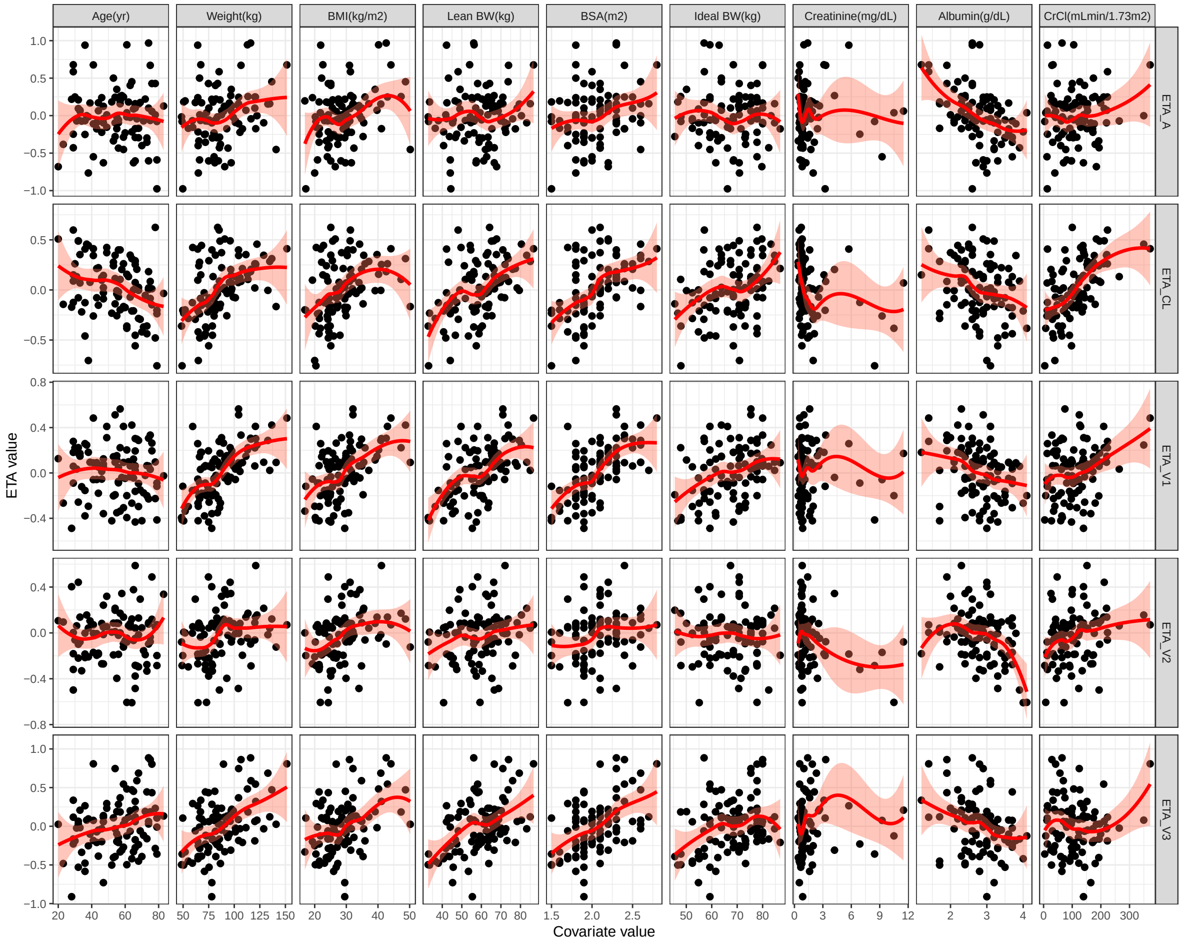

6.9.2 Continuous covariates (Log scale)

Covariate values in log scale

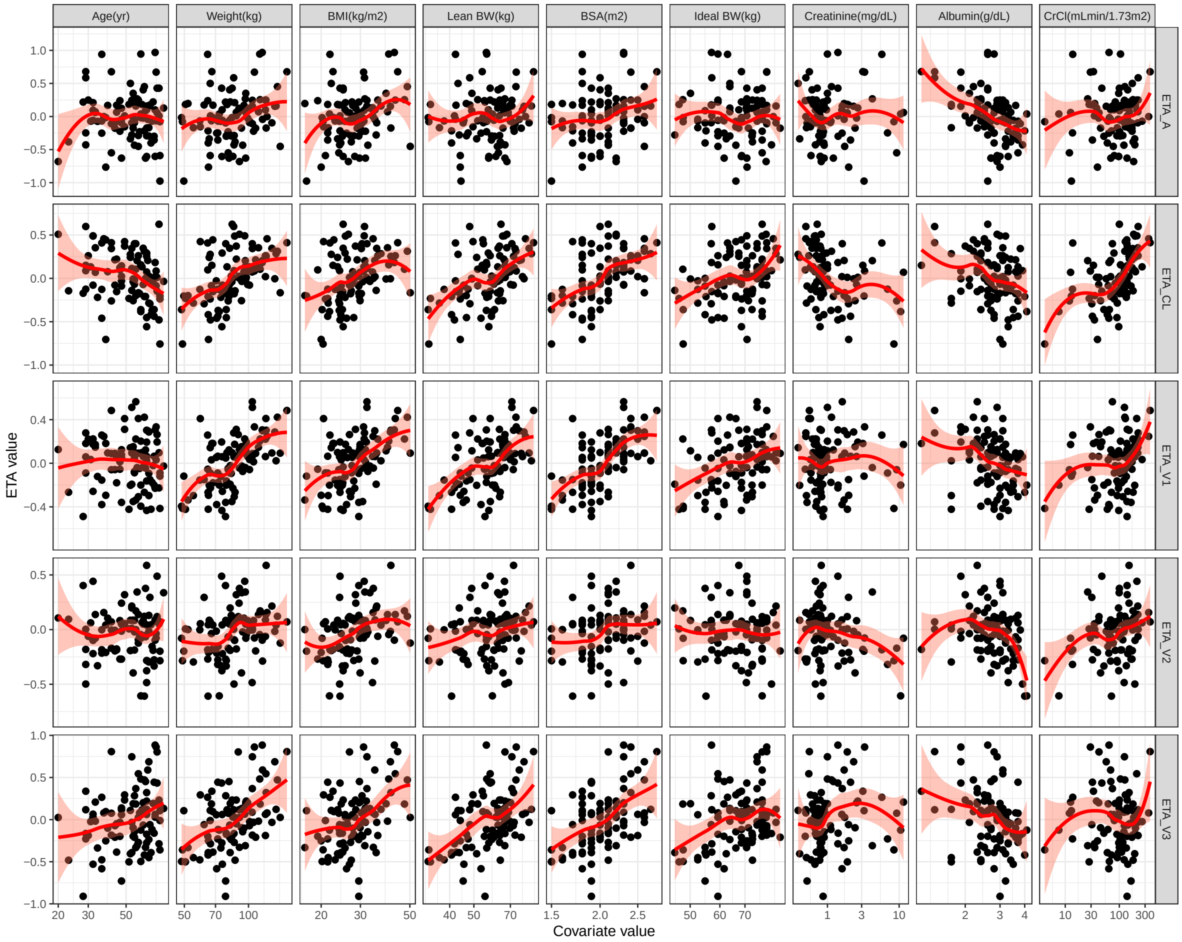
Age(yr)
Weight(kg)
BMI(kg/m2)
Lean BW(kg)
BSA(m2)
Ideal BW(kg)
Creatinine(mg/dL)
Albumin(g/dL)
CrCl(mLmin/1.73m2)
ETA_A
ETA_CL
ETA_V1
ETA_V2
ETA_V3
ETA value
Covariate value

6.9.3 Categorical covariates

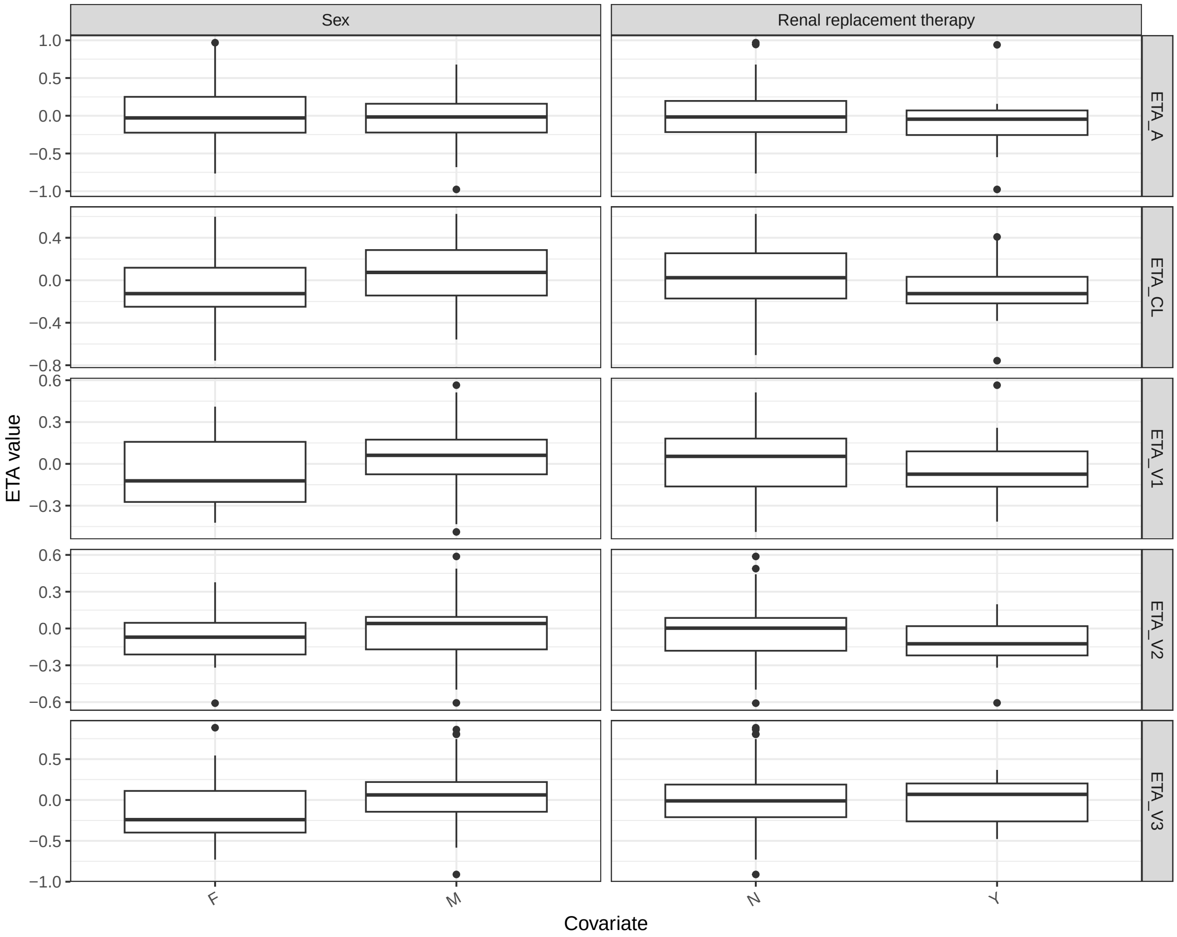

7 INDIVIDUAL FITS

Individual fits were generated in R and exported as a .pdf file in : ./results/03-base-model/indiv-fits-base.pdf

Individual fits with logged time axis in: ./results/03-base-model/indiv-fits-loggedtime-base.pdf

```
pdf
  2
```

```
pdf
  2
```

8 REPRODUCIBILITY

The program code for this analysis is in the following file:

/mnt/data/code/SSC/Emmes/Emmes-Dalbavancin-DOTS/03-base-model.qmd

The packages used in this analysis are listed below:

```
- Session info ---------------------------------------------------------------
 setting  value
 version  R version 4.2.3 (2023-03-15)
 os       Ubuntu 20.04.6 LTS
 system   x86_64, linux-gnu
 ui       X11
 language (EN)
 collate  en_US.UTF-8
 ctype    en_US.UTF-8
 tz       UTC
 date     2024-06-07
 pandoc   2.19.2 @ /usr/lib/rstudio-server/bin/quarto/bin/tools/ (via rmarkdown)

- Packages -------------------------------------------------------------------
 !  package           * version  date (UTC) lib source
 P  arsenal           * 3.6.3    2021-06-04 [?] CRAN (R 4.2.3)
 P  askpass             1.2.0    2023-09-03 [?] CRAN (R 4.2.3)
 P  backports           1.4.1    2021-12-13 [?] CRAN (R 4.2.3)
 P  base64enc           0.1-3    2015-07-28 [?] CRAN (R 4.2.3)
 P  broom.helpers       1.14.0   2023-08-07 [?] CRAN (R 4.2.3)
 P  checkmate           2.3.1    2023-12-04 [?] CRAN (R 4.2.3)
 P  class               7.3-21   2023-01-23 [?] CRAN (R 4.2.3)
 P  classInt            0.4-10   2023-09-05 [?] CRAN (R 4.2.3)
 P  cli                 3.6.1    2023-03-23 [?] CRAN (R 4.2.3)
 P  clisymbols          1.2.0    2017-05-21 [?] CRAN (R 4.2.3)
 P  codetools           0.2-19   2023-02-01 [?] CRAN (R 4.2.3)
 P  colorspace          2.1-0    2023-01-23 [?] CRAN (R 4.2.3)
 P  crayon              1.5.2    2022-09-29 [?] CRAN (R 4.2.3)
 P  crul                1.4.2    2024-04-09 [?] CRAN (R 4.2.3)
 P  curl                5.2.0    2023-12-08 [?] CRAN (R 4.2.3)
 P  data.table          1.14.8   2023-02-17 [?] CRAN (R 4.2.3)
```

```
P  digest              0.6.33   2023-07-07 [?] CRAN (R 4.2.3)
P  dplyr             * 1.1.4    2023-11-17 [?] CRAN (R 4.2.3)
VP e1071               1.7-13   2023-12-06 [?] CRAN (R 4.2.3) (on disk 1.7.14)
P  ellipsis            0.3.2    2021-04-29 [?] CRAN (R 4.2.3)
P  evaluate            0.23     2023-11-01 [?] CRAN (R 4.2.3)
P  fansi               1.0.6    2023-12-08 [?] CRAN (R 4.2.3)
P  farver              2.1.1    2022-07-06 [?] CRAN (R 4.2.3)
P  fastmap             1.1.1    2023-02-24 [?] CRAN (R 4.2.3)
P  flextable         * 0.9.6    2024-05-05 [?] CRAN (R 4.2.3)
P  fontBitstreamVera   0.1.1    2017-02-01 [?] CRAN (R 4.2.3)
P  fontLiberation      0.1.0    2016-10-15 [?] CRAN (R 4.2.3)
P  fontquiver          0.2.1    2017-02-01 [?] CRAN (R 4.2.3)
P  forcats           * 1.0.0    2023-01-29 [?] CRAN (R 4.2.3)
P  fs                  1.6.3    2023-07-20 [?] CRAN (R 4.2.3)
P  gdtools             0.3.7    2024-03-05 [?] CRAN (R 4.2.3)
P  generics            0.1.3    2022-07-05 [?] CRAN (R 4.2.3)
P  gfonts              0.2.0    2023-01-08 [?] CRAN (R 4.2.3)
P  GGally            * 2.2.0    2023-11-22 [?] CRAN (R 4.2.3)
P  ggforce           * 0.4.1    2022-10-04 [?] CRAN (R 4.2.3)
P  ggplot2           * 3.4.4    2023-10-12 [?] CRAN (R 4.2.3)
P  ggstats             0.5.1    2023-11-21 [?] CRAN (R 4.2.3)
P  glue                1.6.2    2022-02-24 [?] CRAN (R 4.2.3)
P  gridExtra         * 2.3      2017-09-09 [?] CRAN (R 4.2.3)
P  gt                * 0.10.1   2024-01-17 [?] CRAN (R 4.2.3)
P  gtable              0.3.4    2023-08-21 [?] CRAN (R 4.2.3)
P  gtsummary         * 1.7.2    2023-07-15 [?] CRAN (R 4.2.3)
P  haven             * 2.5.4    2023-11-30 [?] CRAN (R 4.2.3)
P  hms               * 1.1.3    2023-03-21 [?] CRAN (R 4.2.3)
P  htmltools           0.5.7    2023-11-03 [?] CRAN (R 4.2.3)
P  httpcode            0.3.0    2020-04-10 [?] CRAN (R 4.2.3)
P  httpuv              1.6.12   2023-10-23 [?] CRAN (R 4.2.3)
P  janitor           * 2.2.0    2023-02-02 [?] CRAN (R 4.2.3)
P  jsonlite            1.8.7    2023-06-29 [?] CRAN (R 4.2.3)
P  kableExtra        * 1.4.0    2024-01-24 [?] CRAN (R 4.2.3)
P  KernSmooth          2.23-20  2021-05-03 [?] CRAN (R 4.2.3)
P  knitr               1.45     2023-10-30 [?] CRAN (R 4.2.3)
P  labeling            0.4.3    2023-08-29 [?] CRAN (R 4.2.3)
P  labelled          * 2.12.0   2023-06-21 [?] CRAN (R 4.2.3)
P  later               1.3.1    2023-05-02 [?] CRAN (R 4.2.3)
P  lattice             0.20-45  2021-09-22 [?] CRAN (R 4.2.3)
P  lifecycle           1.0.4    2023-11-07 [?] CRAN (R 4.2.3)
P  lubridate         * 1.9.3    2023-09-27 [?] CRAN (R 4.2.3)
P  magick              2.8.2    2023-12-20 [?] CRAN (R 4.2.3)
```

```
P  magrittr         * 2.0.3    2022-03-30 [?] CRAN (R 4.2.3)
P  MASS               7.3-58.2 2023-01-23 [?] CRAN (R 4.2.3)
P  Matrix             1.6-0    2023-07-08 [?] RSPM (R 4.2.0)
VP MatrixModels       0.5-3    2023-07-10 [?] RSPM (R 4.2.0) (on disk 0.5.2)
P  matrixStats        1.2.0    2023-12-11 [?] CRAN (R 4.2.3)
P  mgcv               1.8-42   2023-03-02 [?] CRAN (R 4.2.3)
P  mime               0.12     2021-09-28 [?] CRAN (R 4.2.3)
P  munsell            0.5.0    2018-06-12 [?] CRAN (R 4.2.3)
P  nlme               3.1-162  2023-01-31 [?] CRAN (R 4.2.3)
P  officer            0.6.6    2024-05-05 [?] CRAN (R 4.2.3)
P  openssl            2.1.1    2023-09-25 [?] CRAN (R 4.2.3)
P  pander             0.6.5    2022-03-18 [?] CRAN (R 4.2.3)
P  pillar             1.9.0    2023-03-22 [?] CRAN (R 4.2.3)
P  pkgconfig          2.0.3    2019-09-22 [?] CRAN (R 4.2.3)
P  plyr               1.8.9    2023-10-02 [?] CRAN (R 4.2.3)
P  polyclip           1.10-6   2023-09-27 [?] CRAN (R 4.2.3)
P  promises           1.2.1    2023-08-10 [?] CRAN (R 4.2.3)
P  proxy              0.4-27   2022-06-09 [?] CRAN (R 4.2.3)
P  pryr               0.1.6    2023-01-17 [?] CRAN (R 4.2.3)
P  purrr            * 1.0.2    2023-08-10 [?] CRAN (R 4.2.3)
P  quantreg           5.96     2023-07-19 [?] RSPM (R 4.2.0)
P  R.cache            0.16.0   2022-07-21 [?] CRAN (R 4.2.3)
P  R.methodsS3        1.8.2    2022-06-13 [?] CRAN (R 4.2.3)
P  R.oo               1.26.0   2024-01-24 [?] CRAN (R 4.2.3)
P  R.utils            2.12.3   2023-11-18 [?] CRAN (R 4.2.3)
P  R6                 2.5.1    2021-08-19 [?] CRAN (R 4.2.3)
P  ragg               1.2.7    2023-12-11 [?] CRAN (R 4.2.3)
P  rapportools        1.1      2022-03-22 [?] CRAN (R 4.2.3)
P  RColorBrewer       1.1-3    2022-04-03 [?] CRAN (R 4.2.3)
P  Rcpp               1.0.11   2023-07-06 [?] CRAN (R 4.2.3)
P  readr            * 2.1.5    2024-01-10 [?] CRAN (R 4.2.3)
   renv               1.0.3    2023-09-19 [1] CRAN (R 4.2.3)
P  reshape2         * 1.4.4    2020-04-09 [?] CRAN (R 4.2.3)
P  rlang              1.1.2    2023-11-04 [?] CRAN (R 4.2.3)
P  rmarkdown          2.27     2024-05-17 [?] CRAN (R 4.2.3)
P  rstudioapi         0.15.0   2023-07-07 [?] CRAN (R 4.2.3)
P  scales             1.3.0    2023-11-28 [?] CRAN (R 4.2.3)
P  sessioninfo      * 1.2.2    2021-12-06 [?] CRAN (R 4.2.3)
P  shiny              1.7.5.1  2023-10-14 [?] CRAN (R 4.2.3)
P  snakecase          0.11.1   2023-08-27 [?] CRAN (R 4.2.3)
P  SparseM            1.81     2021-02-18 [?] CRAN (R 4.2.3)
P  stringi            1.8.1    2023-11-13 [?] RSPM (R 4.2.0)
P  stringr          * 1.5.1    2023-11-14 [?] CRAN (R 4.2.3)
```

```
 P  styler            * 1.10.2   2023-08-29 [?] CRAN (R 4.2.3)
 P  summarytools      * 1.0.1    2022-05-20 [?] CRAN (R 4.2.3)
 VP survival            3.6-4    2023-02-12 [?] CRAN (R 4.2.3) (on disk 3.5.3)
 P  svglite             2.1.3    2023-12-08 [?] CRAN (R 4.2.3)
 P  systemfonts         1.0.5    2023-10-09 [?] CRAN (R 4.2.3)
 P  textshaping         0.3.7    2023-10-09 [?] CRAN (R 4.2.3)
 P  tibble            * 3.2.1    2023-03-20 [?] CRAN (R 4.2.3)
 P  tidylog           * 1.0.2    2020-07-03 [?] CRAN (R 4.2.3)
 P  tidyr             * 1.3.1    2024-01-24 [?] CRAN (R 4.2.3)
 P  tidyselect        * 1.2.0    2022-10-10 [?] CRAN (R 4.2.3)
 P  tidyverse         * 2.0.0    2023-02-22 [?] CRAN (R 4.2.3)
 P  tidyvpc           * 1.5.1    2024-01-18 [?] CRAN (R 4.2.3)
 P  timechange          0.3.0    2024-01-18 [?] CRAN (R 4.2.3)
 P  tweenr              2.0.2    2022-09-06 [?] CRAN (R 4.2.3)
 P  tzdb                0.4.0    2023-05-12 [?] CRAN (R 4.2.3)
 P  utf8                1.2.4    2023-10-22 [?] CRAN (R 4.2.3)
 P  uuid                1.2-0    2024-01-14 [?] CRAN (R 4.2.3)
 P  vctrs               0.6.4    2023-10-12 [?] CRAN (R 4.2.3)
 P  viridisLite         0.4.2    2023-05-02 [?] CRAN (R 4.2.3)
 P  withr               2.5.2    2023-10-30 [?] CRAN (R 4.2.3)
 P  xfun                0.41     2023-11-01 [?] CRAN (R 4.2.3)
 P  xml2                1.3.6    2023-12-04 [?] CRAN (R 4.2.3)
 P  xtable              1.8-4    2019-04-21 [?] CRAN (R 4.2.3)
 P  yaml                2.3.7    2023-01-23 [?] CRAN (R 4.2.3)
 P  zip                 2.3.1    2024-01-27 [?] CRAN (R 4.2.3)
 P  zoo               * 1.8-12   2023-04-13 [?] CRAN (R 4.2.3)

 [1] /mnt/data/code/SSC/Emmes/Emmes-Dalbavancin-DOTS/renv/library/R-4.2/x86_64-pc-linux-gnu
 [2] /mnt/data/.cache/R/renv/sandbox/R-4.2/x86_64-pc-linux-gnu/e11edd0e

 V -- Loaded and on-disk version mismatch.
 P -- Loaded and on-disk path mismatch.

------------------------------------------------------------------------------
```

Task04: Dalbavancin Full popPK model - Results and Diagnostic plots

Final

Melanie Wilbaux Jessica Wojciechowski Daniel Selig

07-Jun-2024

Contents

1 BACKGROUND 3
1.1 Purpose 3
1.2 Executive Summary 3

2 INITIALIZE ANALYSIS 5
2.1 Initialize R Session 5
2.2 Load packages 5
2.3 Set plotting theme 5
2.4 Define functions 5

3 UNIVARIATE ANALYSIS 5

4 STEPWISE FORWARD SELECTION 7

5 RE-ASSESSMENT OF RESIDUAL AND BSV MODELS 8

6 RE-ASSESSMENT OF REMAINING TREND WITH COVARIATES 9

7 FULL MODEL 9

8 IMPORT FULL MODEL RESULTS 10

9 PARAMETER ESTIMATES 10

10 DIAGNOSTIC PLOTS 12
10.1 DV vs. PRED 13
10.1.1 Linear scale 13

10.1.2 Log scale 13
10.2 DV vs. IPRED 14
10.2.1 Linear scale 14
10.2.2 Log scale 15
10.3 RES vs. Time after first dose 16
10.3.1 CWRES vs. Time 16
10.3.2 CWRES vs. Time (log scale) 17
10.3.3 IWRES vs. Time 18
10.3.4 IWRES vs. Time (log scale) 19
10.4 RES vs. Time from previous dose 20
10.4.1 CWRES vs. Time 20
10.4.2 CWRES vs. Time (log scale) 21
10.4.3 IWRES vs. Time 22
10.4.4 iWRES vs. Time (log scale) 23
10.5 RES vs. PRED 24
10.5.1 CWRES vs. PRED 24
10.5.2 IWRES vs. IPRED 25
10.5.3 |IWRES| vs IPRED 26
10.6 RES Distribution 27
10.6.1 CWRES hist 27
10.6.2 CWRES qqplot 28
10.6.3 IWRES hist 29
10.6.4 IWRES qqplot 30
10.7 BSV Distribution 31
10.7.1 Hist 31
10.7.2 QQplots 32
10.8 ETAs Correlations 33
10.9 BSV vs. Cov 34
10.9.1 Continuous covariates 35
10.9.2 Continuous covariates (Log scale) 35
10.9.3 Categorical covariates 37

11 INDIVIDUAL FITS 37

12 INDIVIDUAL PARAMETERS VS COVARIATES 38
12.0.1 Continuous covariates 38
12.0.2 Categorical covariates 39

13 COVARIATE-EFFECT PLOTS 40
13.1 CL 40
13.2 V1 41
13.3 V2 42
13.4 V3 43

13.5 A 44

14 REPRODUCIBILITY **44**

1 BACKGROUND

1.1 Purpose

The purpose of this script is to summarize the results from the dalbavancin full popPK model and to generate diagnostic plots.

1.2 Executive Summary

The full popPK model was developed by forward inclusion of the covariate-parameter relationships selected for further consideration by the exploratory analysis.

Univariate analysis

Each pre-specified covariate-parameter relationship was incorporated into the base model to conduct univariate analysis (one model per covariate-parameter relationship). Key results, including OFV, p-values from LRT and BSV for each parameter, were presented in Section 3 (*Table 1 from ./results/04-full-model/*). Numerous covariate-parameter relationships were found to be statistically significant (LRT p<0.05). The most pronounced effects (reflected by a decrease in OFV from the base model) were observed for: CrCl on CL, age on V3, creatinine on CL and age on CL. The modeling process encountered convergence issues when incorporating time-varying albumin; therefore, baseline albumin (ALBBL) was examined, resulting in more stable models. All continuous covariates were initially assessed using a power model. Linear covariate-parameter relationships were also considered for the relevant covariates, but did not yield any improvement in model fit.

Stepwise Forward Inclusion of Covariate-Parameter Relationships

The full popPK model was constructed through stepwise forward inclusion of covariate-parameter relationships that exhibited statistical significance in univariate analysis (LRT p<0.05), starting with the most significant ones. Covariate-parameter relationships were retained in the full model if they resulted in a p-value < 0.01 from a LRT. Additional criteria for inclusion in the stepwise process were also considered, including model stability, a decrease in BSV for the parameter of interest and no compensatory increase in other BSV.

Due to the observed correlation between CrCl and creatinine, only CrCl-parameter relationships were evaluated for the full model. Among all the body size covariates, exhibiting correlation, baseline body weight was exclusively evaluated, as it showed the strongest effect on V1 and the largest reduction in its BSV.

Results from each step, including OFV, p-values from LRT and BSV for each parameter, were detailed in Section 4 (*Table 2 from ./results/04-full-model/*). Following completion of the forward selection, RUV, BSV and correlations were re-assessed, as summarized in Section 5 (*Table 3 from ./results/04-full-model/*). No changes were needed in the RUV model. However, exclusion of A from the correlation matrix resulted in a more parsimonious model. Of note, despite a decrease in the BIC (-2.2 points) upon the addition of a BSV on parameter k, the model was not retained due to issues with over-parameterization, including convergence and numerical instability. Remaining trends were noted between weight and CL and A, as well as between baseline albumin and CL, V1 and V3. These effects were re-evaluated, as summarized in Section 6 (*Table 4 from ./results/04-full-model/*). The results revealed statistically significant relationships between weight and CL and A, whereas baseline albumin effects on CL, V1 and V3 were not identifiable.

Full Model

To summarize, the full model is a model with three-compartments, zero-order input and first-order elimination that describes total plasma PK. unbound concentrations were assumed to be dependent on total concentrations with a power relationship. The RUV was modeled using a proportional error model for total concentrations and a combined error model for unbound concentrations. The BSV included log-normally distributed random effects on CL, V1, V2, V3 and A. The BSV on parameters, CL, V1, and V3 was modeled using a three-dimensional matrix taking into account correlations among the parameters.

The following covariate-parameter relationships were included in the full model:

- covariates on CL: CrCl, renal replacement therapy and baseline body weight
- covariates on V1: baseline body weight
- covariates on V2: baseline body weight, baseline albumin and renal replacement therapy
- covariates on V3: age and baseline body weight
- covariates on A: baseline albumin, sex and baseline body weight.

Parameter estimates from the full model were presented in Section 9 (*Table 5 from ./results/04-full-model/*). All parameters from the base model were well estimated, with acceptable RSE and consistent with the base model. Only the sex effect on A showed a high RSE.

Compared to the base model, reductions were observed in all BSV (CV%):

- BSV on CL was reduced from 30.2% to 19.7%;
- BSV on V1 was reduced from 26.4% to 19.6%;
- BSV on V2 was reduced from 33.5% to 28%;
- BSV on V3 was reduced from 38.9% to 28.9%;
- BSV on A was reduced from 38.3% to 29.8%.

The condition number, calculated as the ratio between the largest and smallest eigenvalues of the correlation matrix, was found to be 117, indicating that the full popPK model was stable and not over-parameterized (as the value is < 1000).

All the diagnostic plots from Section 10 suggested that the full popPK model described the total and unbound PK data adequately. The observed concentration vs. model predictions (population and individual) plots demonstrated a reasonable agreement between data and model predictions. The residual plots do not show any strong pattern with either time or concentration, and closely resembles a normal distribution. A slight trend in the residuals of unbound concentrations at later time points was observed; but it did not raise concerns as it only pertained to a few points, and all other diagnostic plots were acceptable.

2 INITIALIZE ANALYSIS

2.1 Initialize R Session

Initialize the global R environment and result directories.

```
[1] "/mnt/data/code/SSC/Emmes/Emmes-Dalbavancin-DOTS"
```

2.2 Load packages

```
[1] "/mnt/data/code/SSC/Emmes/Emmes-Dalbavancin-DOTS/renv/library/R-4.2/x86_64-pc-linux-gnu"
[2] "/mnt/data/.cache/R/renv/sandbox/R-4.2/x86_64-pc-linux-gnu/e11edd0e"
```

2.3 Set plotting theme

2.4 Define functions

3 UNIVARIATE ANALYSIS

> **Note**
>
> Univariate analysis was performed on the base model in Pumas (/programs/pumas/univ-CL.jl, univ-V1.jl, univ-V2.jl, univ-V3.jl and univ-A.jl).

Covariate	OFV	deltaOFV_fromBase	p_value	BSV.CL	BSV.V1	BSV.V2	BSV.V3	BSV.A
Base	3,434.518	0.000	1.0000	0.0913	0.0695	0.1121	0.1506	0.1465
CRCL_on_CL	3,385.755	-48.763	<0.001	0.0577	0.0711	0.1168	0.1489	0.1467
Age_on_V3	3,398.214	-36.304	<0.001	0.0877	0.0727	0.1067	0.1309	0.1450

Covariate	OFV	deltaOFV_fromBase	p_value	BSV.CL	BSV.V1	BSV.V2	BSV.V3	BSV.A
CREAT_on_CL	3,402.728	-31.790	<0.001	0.0759	0.0696	0.1092	0.1485	0.1469
Age_on_CL	3,416.103	-18.415	<0.001	0.0777	0.0707	0.1136	0.1520	0.1470
ALB_on_V2	3,419.400	-15.118	<0.001	0.0899	0.0684	0.1020	0.1472	0.1474
BSABL_on_V1	3,419.996	-14.522	<0.001	0.0909	0.0434	0.1118	0.1434	0.1467
WTBL_on_V1	3,420.820	-13.698	<0.001	0.0900	0.0424	0.1123	0.1450	0.1466
BMIBL_on_V2	3,421.776	-12.742	<0.001	0.0893	0.0679	0.0934	0.1483	0.1463
ALBBL_on_A	3,421.939	-12.579	<0.001	0.0908	0.0694	0.1171	0.1515	0.1145
LBWBL_on_CL	3,422.314	-12.204	<0.001	0.0702	0.0697	0.1097	0.1510	0.1460
ALBBL_on_V2	3,423.564	-10.954	<0.001	0.0905	0.0674	0.0989	0.1484	0.1479
Sex_on_A	3,424.487	-10.031	0.0015	0.0912	0.0674	0.1084	0.1480	0.1499
RRTFL_on_V2	3,425.806	-8.712	0.0032	0.0899	0.0696	0.1020	0.1524	0.1454
LBWBL_on_V3	3,425.861	-8.657	0.0033	0.0915	0.0661	0.1114	0.1162	0.1463
Sex_on_CL	3,426.014	-8.504	0.0035	0.0844	0.0675	0.1172	0.1520	0.1467
Sex_on_V3	3,426.038	-8.480	0.0036	0.0912	0.0669	0.1118	0.1373	0.1469
BSABL_on_V2	3,426.721	-7.797	0.0052	0.0891	0.0664	0.1186	0.1458	0.1484
BSABL_on_CL	3,426.848	-7.670	0.0056	0.0717	0.0714	0.1132	0.1508	0.1447
WTBL_on_V2	3,426.859	-7.659	0.0056	0.0886	0.0679	0.1033	0.1475	0.1461
BMIBL_on_V1	3,427.148	-7.370	0.0066	0.0897	0.0520	0.1030	0.1495	0.1417
IBWBL_on_CL	3,427.607	-6.911	0.0086	0.0815	0.0703	0.1134	0.1521	0.1464
LBWBL_on_V1	3,428.016	-6.502	0.0108	0.0895	0.0509	0.1027	0.1480	0.1415
RRTFL_on_CL	3,428.928	-5.590	0.0181	0.0885	0.0698	0.1106	0.1493	0.1468
WTBL_on_CL	3,429.475	-5.043	0.0247	0.0738	0.0695	0.1155	0.1508	0.1464
Age_on_V1	3,429.489	-5.029	0.0249	0.0917	0.0702	0.1119	0.1495	0.1455
LBWBL_on_V2	3,429.792	-4.726	0.0297	0.0902	0.0686	0.1100	0.1460	0.1480
IBWBL_on_V3	3,430.304	-4.214	0.0401	0.0915	0.0677	0.1124	0.1359	0.1466
ALB_on_A	3,430.591	-3.927	0.0475	0.0910	0.0696	0.1193	0.1525	0.1290
BSABL_on_V3	3,431.285	-3.233	0.0722	0.0903	0.0666	0.1169	0.1248	0.1464

Covariate	OFV	deltaOFV_fromBase	p_value	BSV.CL	BSV.V1	BSV.V2	BSV.V3	BSV.A
WTBL_on_V3	3,431.289	-3.229	0.0723	0.0905	0.0671	0.1150	0.1249	0.1466
RRTFL_on_V3	3,431.875	-2.643	0.1040	0.0895	0.0682	0.1100	0.1499	0.1462
ALBBL_on_V3	3,432.224	-2.294	0.1299	0.0909	0.0691	0.1118	0.1376	0.1472
IBWBL_on_V1	3,432.321	-2.197	0.1383	0.0914	0.0645	0.1112	0.1486	0.1468
Sex_on_V2	3,432.366	-2.152	0.1424	0.0905	0.0701	0.1143	0.1505	0.1472
BMIBL_on_CL	3,432.974	-1.544	0.2140	0.0851	0.0683	0.1084	0.1486	0.1476
RRTFL_on_V1	3,433.064	-1.454	0.2279	0.0903	0.0694	0.1151	0.1521	0.1467
BMIBL_on_V3	3,433.506	-1.012	0.3144	0.0906	0.0681	0.1174	0.1397	0.1465
Sex_on_V1	3,433.665	-0.853	0.3557	0.0906	0.0701	0.1165	0.1518	0.1467
CRCL_on_A	3,433.684	-0.834	0.3611	0.0905	0.0697	0.1150	0.1502	0.1505
IBWBL_on_V2	3,433.945	-0.573	0.4491	0.0910	0.0689	0.1144	0.1517	0.1470
Age_on_A	3,434.008	-0.510	0.4751	0.0914	0.0694	0.1113	0.1504	0.1475
ALBBL_on_CL	3,434.025	-0.493	0.4826	0.0886	0.0692	0.1113	0.1504	0.1465
ALB_on_V1	3,434.340	-0.178	0.6731	0.0902	0.0731	0.1031	0.1591	0.1409
CREAT_on_A	3,434.360	-0.158	0.6910	0.0913	0.0691	0.1109	0.1499	0.1468
Age_on_V2	3,434.502	-0.016	0.8993	0.0908	0.0684	0.1109	0.1502	0.1461
ALBBL_on_V1	3,434.509	-0.009	0.9244	0.0915	0.0694	0.1113	0.1504	0.1467
ALB_on_V3	3,434.904	0.386	1.0000	0.0914	0.0697	0.1139	0.1550	0.1470
ALB_on_CL	3,435.603	1.085	1.0000	0.0929	0.0843	0.0992	0.2045	0.2035

4 STEPWISE FORWARD SELECTION

! Important

- Stepwise forward inclusion of the covariate-parameter relationships that were found to be significant in univariate analysis (LRT p<0.05), started with the most significant ones.
- Covariate-parameter relationship was retained in the full model if they result in p-value < 0.01 from a LRT and in a stable model. Additional criteria for inclusion

in the stepwise process were also considered: decrease in BSV for the parameter of interest and no substantial compensatory increase in other BSV.

Note

Stepwise forward selection was performed on the base model in Pumas (/programs/pumas/forward-selection.jl).

Model	Ref_model	Added_covariate	OFV	deltaOFV_fromRef	p_value	BSV.CL	BSV.V1	BSV.V2	BSV.V3	BSV.A	Comments
0		Base	3,434.518		NA	0.0913	0.0695	0.1121	0.1506	0.1465	/
1	0	CrCl_on_CL	3,385.755	-48.763	<0.001	0.0577	0.0711	0.1168	0.1489	0.1467	Covariate added
2	1	Age_on_V3	3,345.722	-40.033	<0.001	0.0566	0.0745	0.1071	0.1304	0.1455	Covariate added
3	2	Age_on_CL	3,343.808	-1.914	0.1665	0.0550	0.0748	0.1073	0.1299	0.1451	Covariate rejected
4	2	ALB_on_V2	3,327.698	-18.024	<0.001	0.0545	0.0741	0.1088	0.1259	0.1449	Covariate rejected. Unstable model.
5	2	WTBL_on_V1	3,327.860	-17.862	<0.001	0.0548	0.0457	0.1200	0.1243	0.1438	Covariate added
6	5	WTBL_on_V2	3,318.622	-9.238	0.0024	0.0538	0.0454	0.1006	0.1218	0.1437	Covariate added
7	6	ALBBL_on_A	3,306.986	-11.636	<0.001	0.0549	0.0470	0.1180	0.1213	0.1113	Covariate added
8	7	WTBL_on_CL	3,306.275	-0.711	0.3991	0.0530	0.0457	0.1008	0.1227	0.1129	Covariate rejected
9	7	ALBBL_on_V2	3,293.285	-13.701	<0.001	0.0531	0.0451	0.0825	0.1221	0.1114	Covariate added
10	9	Sex_on_A	3,289.147	-4.138	0.0419	0.0538	0.0447	0.0801	0.1217	0.1140	Covariate added
11	10	RRTFL_on_V2	3,283.304	-5.843	0.0156	0.0543	0.0463	0.0760	0.1234	0.1122	Covariate added
12	11	WTBL_on_V3	3,273.624	-9.680	0.0019	0.0540	0.0429	0.1047	0.0910	0.1121	Covariate added
13	12	Sex_on_CL	3,269.187	-4.437	0.0352	0.0506	0.0417	0.0773	0.0906	0.1094	Covariate rejected
14	12	Sex_on_V3	3,270.344	-3.280	0.0701	0.0528	0.0418	0.0774	0.0893	0.1102	Covariate rejected
15	12	RRTFL_on_CL	3,264.686	-8.938	0.0028	0.0449	0.0420	0.0810	0.0946	0.1115	Covariate added
16	15	Age_on_V1	3,263.336	-1.350	0.2453	0.0457	0.0412	0.1102	0.0924	0.1128	Covariate rejected

5 RE-ASSESSMENT OF RESIDUAL AND BSV MODELS

Model	Description	Ref	nparam	AIC	AIC_ref	deltaAIC_fromRef
Full01	Full model	/	32	3,328.686		
Full02	Combined error Total	Full01	33	3,330.909	3,328.686	2.223
Full03	Proportional error unbound	Full01	31	3,333.175	3,328.686	4.489
Full04	Add BSV on k	Full01	33	3,326.460	3,328.686	-2.226
Full05	Corr CL-V1-V3	Full01	29	3,332.338	3,328.686	3.652
Full06	Corr V-V3	Full05	27	3,366.137	3,332.338	33.799
Full07	Corr CL-V1	Full05	27	3,373.735	3,332.338	41.397

6 RE-ASSESSMENT OF REMAINING TREND WITH COVARIATES

Model	Ref_model	Added_covariate	OFV	deltaOFV_fromRef	p_value	BSV.CL	BSV.V1	BSV.V2	BSV.V3	BSV.A
0		Full05	3,274.338		NA	0.0432	0.0395	0.0815	0.0876	0.1029
1	0	WTBL_on_A	3,265.125	-9.213	0.0024	0.0428	0.0411	0.0841	0.0881	0.0880
2	1	WTBL_on_CL	3,255.315	-9.810	0.0017	0.0387	0.0386	0.0785	0.0835	0.0886
3	2	ALBBL_on_CL	3,252.234	-3.081	0.0792	0.0339	0.0384	0.0777	0.0830	0.0884
4	2	ALBBL_on_V1	3,253.616	-1.699	0.1924	0.0381	0.0348	0.0774	0.0815	0.0885
5	2	ALBBL_on_V3	3,252.873	-2.442	0.1181	0.0389	0.0380	0.0772	0.0749	0.0885

7 FULL MODEL

! Important

- Full model:
  - 3-compartment model, zero-order input and first-order elimination;
  - unbound concentrations assumed to be dependent on total concentrations with a power relationship;
  - RUV modeled using a proportional error model for total concentrations and a combined error model for unbound concentrations;
  - BSV on CL, V1, V2, V3 and A;

  - Correlations between CL, V1 and V3.

- Covariates included in the Full Model:
  - CL ~ CrCl + RRTFL + WTBL
  - V1 ~ WTBL
  - V2 ~ WTBL + ALBBL + RRTFL
  - V3 ~ Age + WTBL
  - A ~ ALBBL + SEX + WTBL

8 IMPORT FULL MODEL RESULTS

Note

The dalbavancin full popPK model was executed in the Pumas script: ./programs/pumas/full/full-model.jl, to generate the output csv files.

9 PARAMETER ESTIMATES

Table 5: Parameter estimates - Full Model

Parameter	Description	Estim	RSE (%)	95% CI	CV (%)	Corr (%)	Shrinkage (%)
tvcl	Clearance (L/hr)	0.0646	2.6	[0.0612 ; 0.0679]	-	-	-
tvv1	Volume of Distribution in the central cmt (L)	5.67	2.7	[5.37 ; 5.96]	-	-	-
tvq2	Intercompartmental Clearance from cmt 1 to 2 (L/hr)	0.0262	8.5	[0.0218 ; 0.0306]	-	-	-
tvv2	Volume of Distribution of 2nd cmt (L)	9.12	5.7	[8.1 ; 10.1]	-	-	-
tvq3	Intercompartmental Clearance from cmt 1 to 3 (L/hr)	0.922	7.3	[0.791 ; 1.05]	-	-	-
tvv3	Volume of Distribution of 3rd cmt (L)	11.1	3.8	[10.2 ; 11.9]	-	-	-
tva	Intercept of power when ctot=1	0.00128	8.6	[0.00106 ; 0.0015]	-	-	-

Parameter	Description	Estim	RSE (%)	95% CI	CV (%)	Corr (%)	Shrinkage (%)
tvk	Exponent of the power function	1.32	1.4	[1.28 ; 1.36]	-	-	-
cl_crcl	CRCL effect on CL	0.241	15.8	[0.167 ; 0.316]	-	-	-
cl_rrtfl	RRTFL effect on CL	0.217	41.7	[0.0395 ; 0.394]	-	-	-
cl_wtbl	WTBL effect on CL	0.314	27.8	[0.143 ; 0.486]	-	-	-
v1_wtbl	WTBL effect on V1	0.74	15.1	[0.521 ; 0.958]	-	-	-
v2_wtbl	WTBL effect on V2	0.699	36	[0.205 ; 1.19]	-	-	-
v2_albbl	ALBBL effect on V2	-0.711	46.9	[-1.37 ; -0.0573]	-	-	-
v2_rrtfl	RRTFL effect on V2	-0.567	48	[-1.1 ; -0.0338]	-	-	-
v3_age	AGE effect on V3	0.617	16.1	[0.423 ; 0.812]	-	-	-
v3_wtbl	WTBL effect on V3	0.794	18.4	[0.509 ; 1.08]	-	-	-
a_albbl	ALBBL effect on A	-0.739	14	[-0.942 ; -0.537]	-	-	-
a_sex	SEX effect on A	0.139	64.5	[-0.0368 ; 0.315]	-	-	-
a_wtbl	WTBL effect on A	0.469	36.6	[0.133 ; 0.806]	-	-	-
Ω ,	BSV V2	0.0785	28.8	[0.0342 ; 0.123]	28	-	39.3
Ω ,	BSV A	0.0886	19.6	[0.0546 ; 0.123]	29.8	-	7.5
Ω_CLV1V3 ,	BSV CL	0.0387	15.9	[0.0266 ; 0.0508]	19.7	-	5.1
Ω_CLV1V3 ,	Corr CL-V1	0.022	27.5	[0.0102 ; 0.0339]	-	57	-
Ω_CLV1V3 ,	Corr CL-V3	0.0351	21.1	[0.0206 ; 0.0496]	-	61.7	-
Ω_CLV1V3 ,	BSV V1	0.0386	27.4	[0.0178 ; 0.0593]	19.6	-	11.2
Ω_CLV1V3 ,	Corr V1-V3	0.0467	14.6	[0.0334 ; 0.0601]	-	82.3	-

Parameter	Description	Estim	RSE (%)	95% CI	CV (%)	Corr (%)	Shrinkage (%)
Ω_CLV1V3 ,	BSV V3	0.0836	15	[0.0589 ; 0.108]	28.9	-	8.7
_proptot	Proportional Total RUV	0.126	6.4	[0.111 ; 0.142]	-	-	-
_propfree	Proportional Unbound RUV	0.198	6.2	[0.174 ; 0.222]	-	-	-
_addfree	Additive Unbound RUV	0.0128	18.1	[0.00829 ; 0.0174]	-	-	-
Epsilon shrinkage Total	-	-	-	-	-	-	17.6
Epsilon shrinkage Unbound	-	-	-	-	-	-	8.8

Abbreviations: BSV = between-subject variability; CI = confidence interval; CV = coefficient of variation; RSE = relative standard error; RUV = residual unexplained variability.

Notes:

- The condition number is equal to 117.
- RSE % was derived as: se/estimate*100.
- Estimates for BSV are the variances.
- The CV % was derived as: sqrt(var)*100.

10 DIAGNOSTIC PLOTS

ℹ Note

- The diagnostic plots are not stratified by dose group, as the number of patients are too limited in the groups different from Dalbavancin 1500 mg + 1500 mg.
- BLOQ values for unbound concentrations (<0.05 μg/mL) are removed for all the diagnostic plots.

10.1 DV vs. PRED

10.1.1 Linear scale

Observed vs. Population predicted dalbavancin concentrations - Full Model

Note: Solid black lines represent the lines of identity, and red lines the linear regression lines.

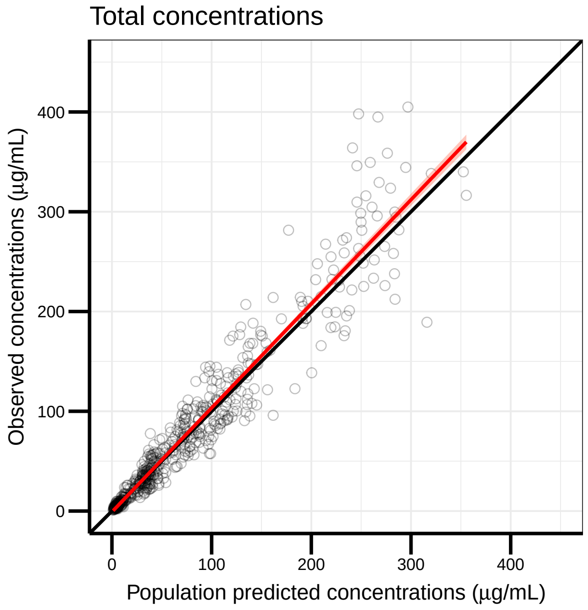

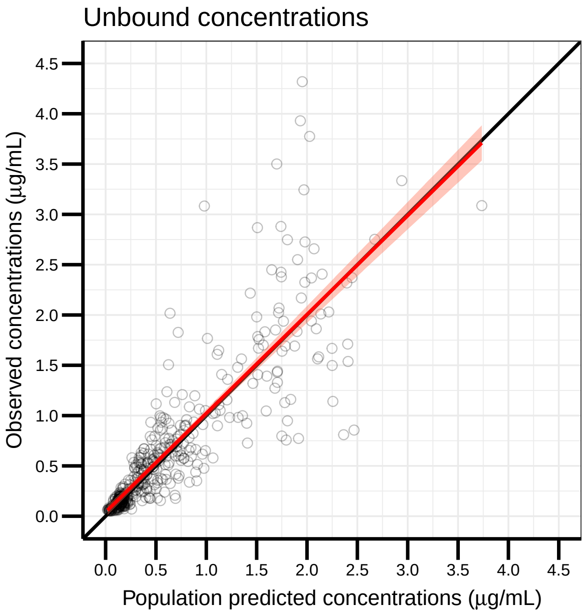

10.1.2 Log scale

Observed vs. Population predicted dalbavancin concentrations - Full Model

Note: Solid black lines represent the lines of identity, and red lines the linear regression lines.

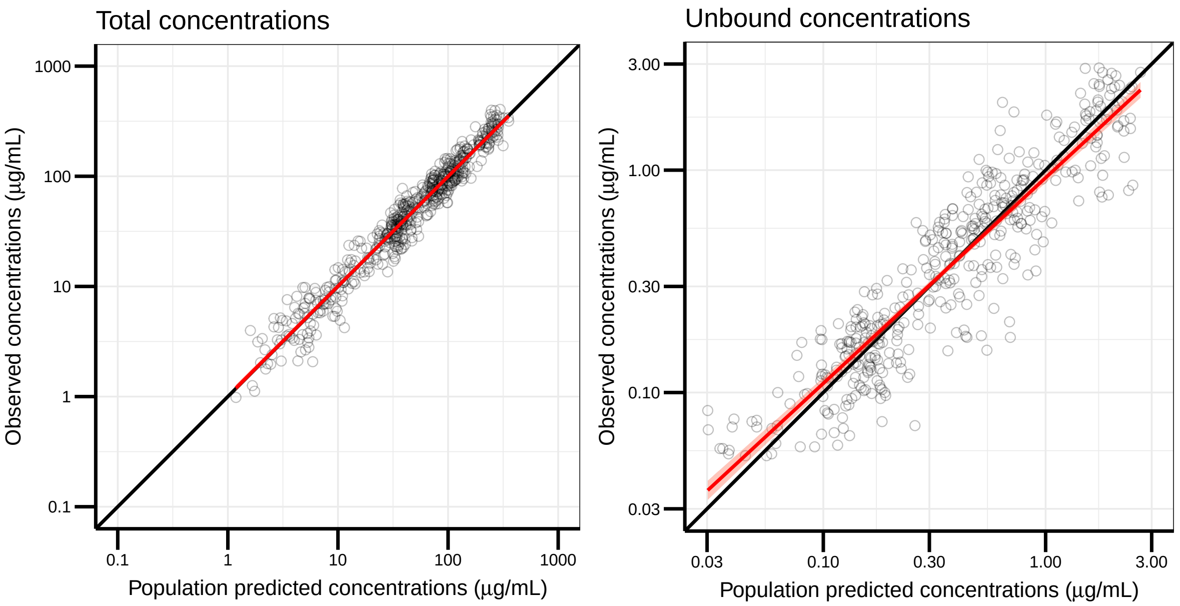

10.2 DV vs. IPRED

10.2.1 Linear scale

Observed vs. Individual predicted dalbavancin concentrations - Full Model

Note: Solid black lines represent the lines of identity, and red lines the linear regression lines (with 95% CI).

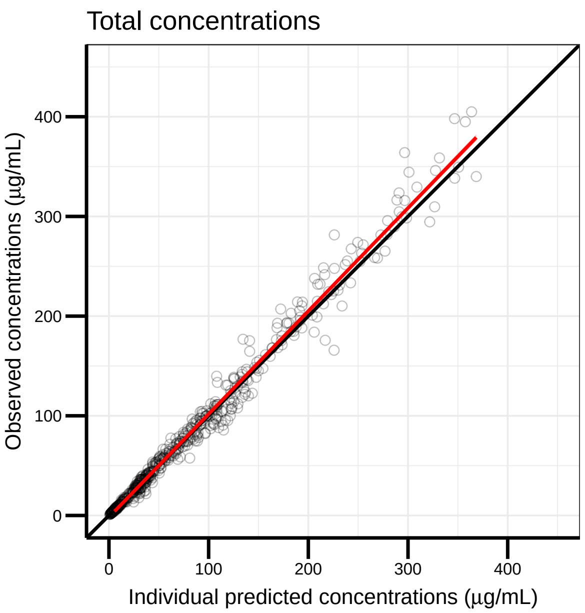

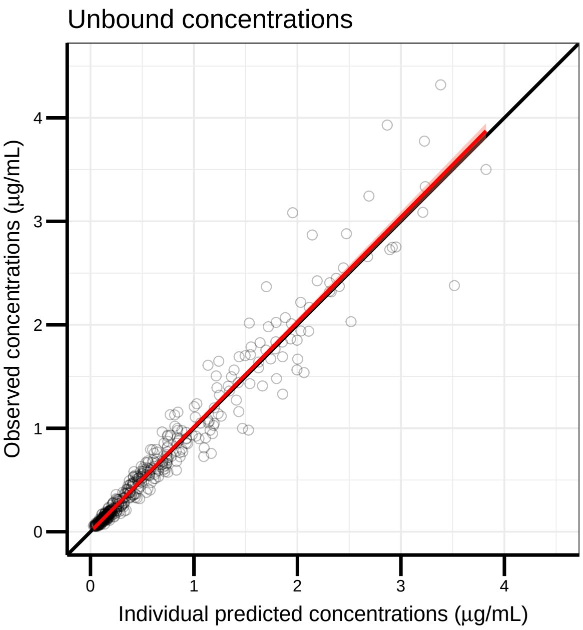

10.2.2 Log scale

Observed vs. Individual predicted dalbavancin concentrations - Full Model

Note: Solid black lines represent the lines of identity, and red lines the linear regression lines (with 95% CI).

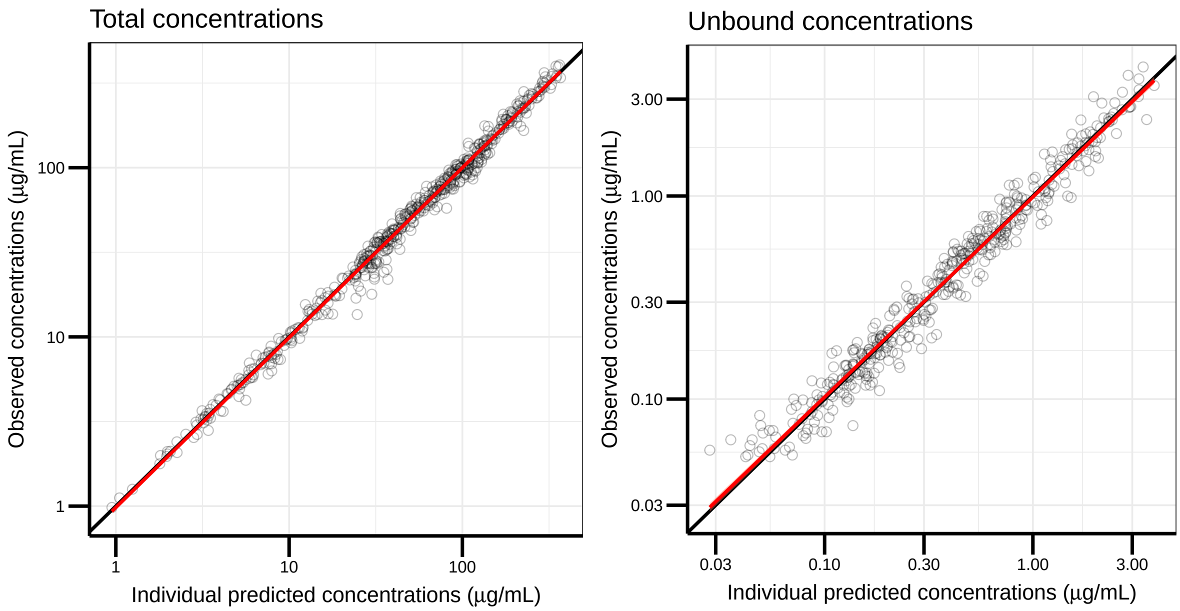

10.3 RES vs. Time after first dose

10.3.1 CWRES vs. Time

Conditional Weighted Residuals (CWRES) vs. Time after first dose - Full Model

Note: Solid black lines represent the lines of identity, and red curves the lowess smooth regression curves (with 95% CI).

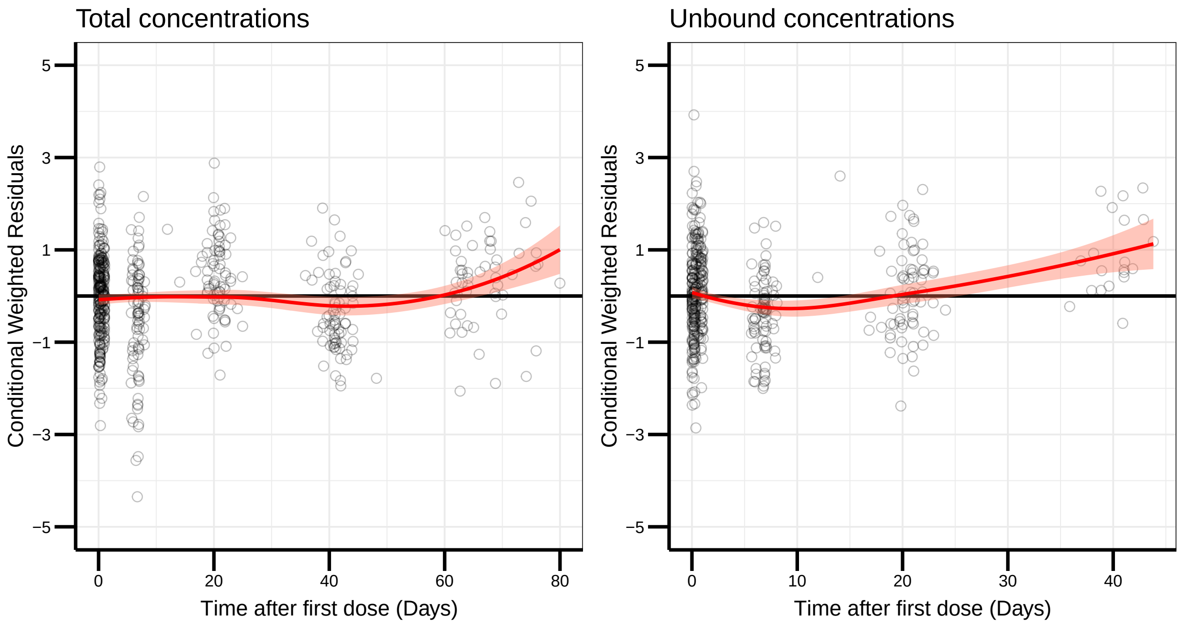

10.3.2 CWRES vs. Time (log scale)

Conditional Weighted Residuals (CWRES) vs. Time after first dose - Full Model

Note: Solid black lines represent the lines of identity, and red curves the lowess smooth regression curves (with 95% CI).

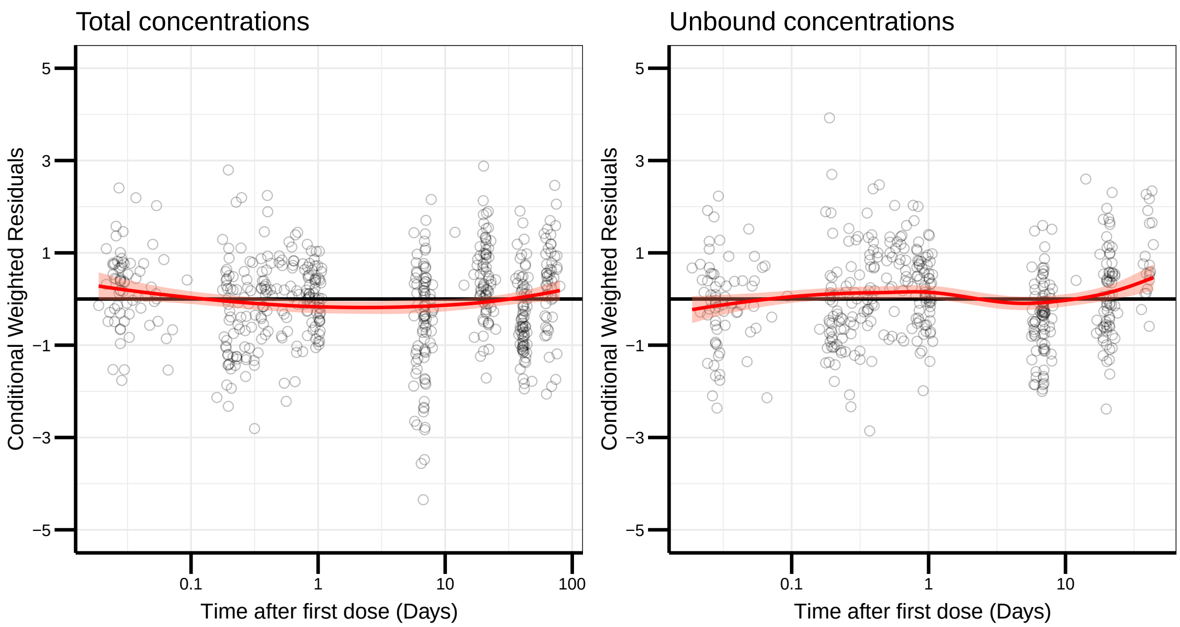

10.3.3 IWRES vs. Time

Individual Weighted Residuals (IWRES) vs. Time after first dose - Full Model

Note: Solid black lines represent the lines of identity, and red curves the lowess smooth regression curves (with 95% CI).

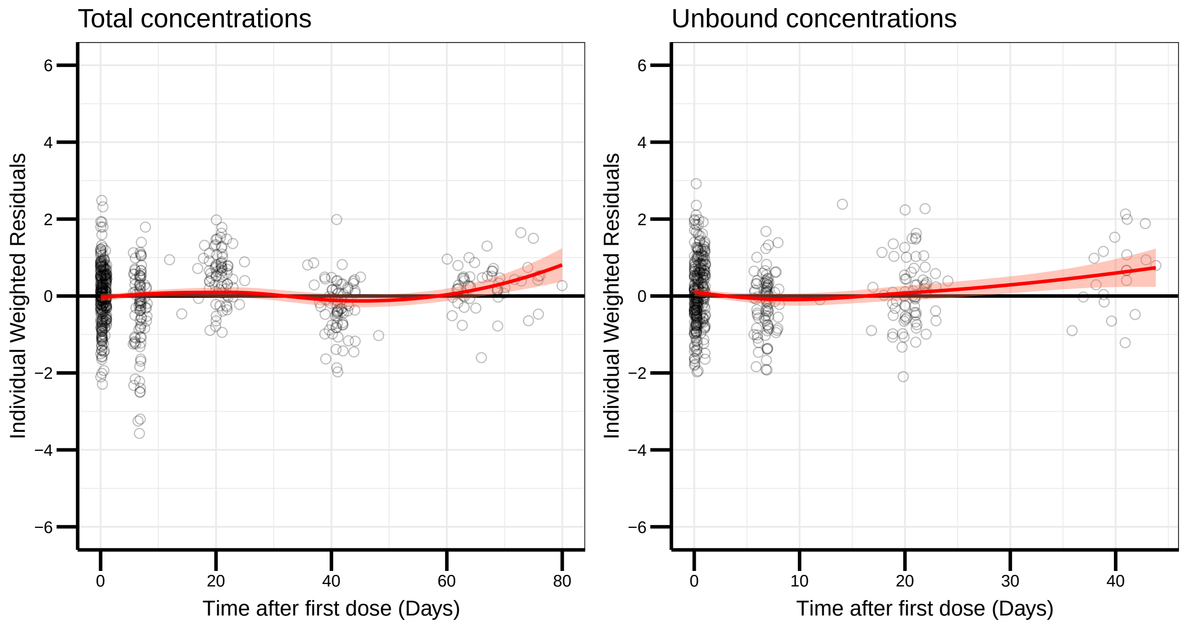

10.3.4 IWRES vs. Time (log scale)

Individual Weighted Residuals (IWRES) vs. Time after first dose - Full Model

Note: Solid black lines represent the lines of identity, and red curves the lowess smooth regression curves (with 95% CI).

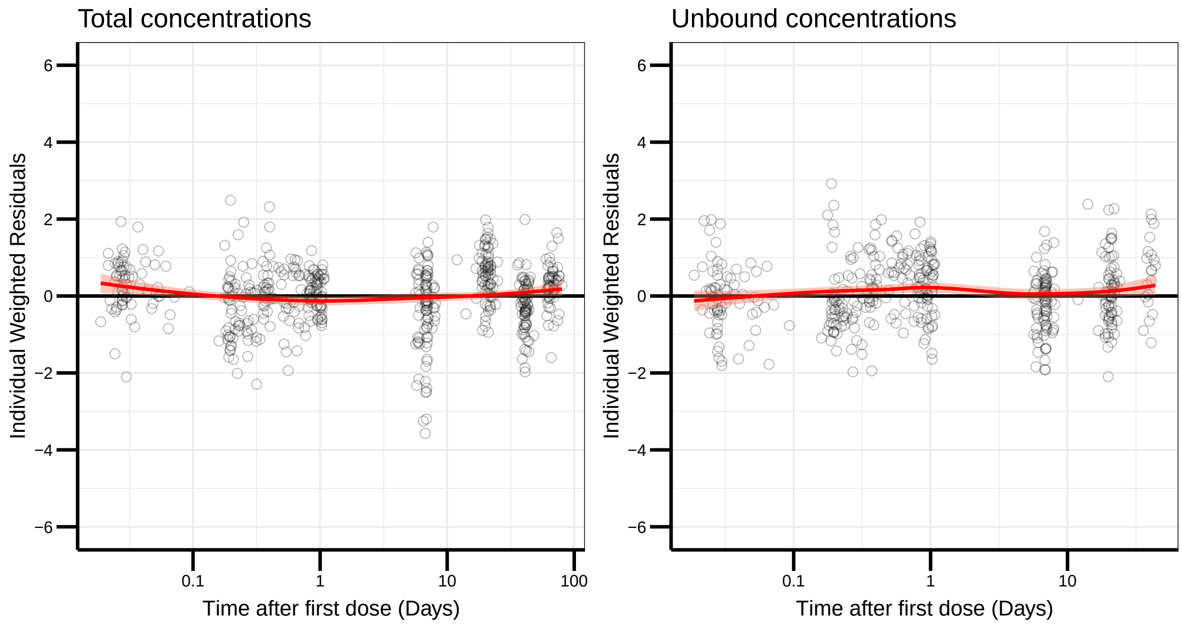

10.4 RES vs. Time from previous dose

10.4.1 CWRES vs. Time

Conditional Weighted Residuals (CWRES) vs. Time from previous dose - Full Model

Note: Solid black lines represent the lines of identity, and red curves the lowess smooth regression curves (with 95% CI).

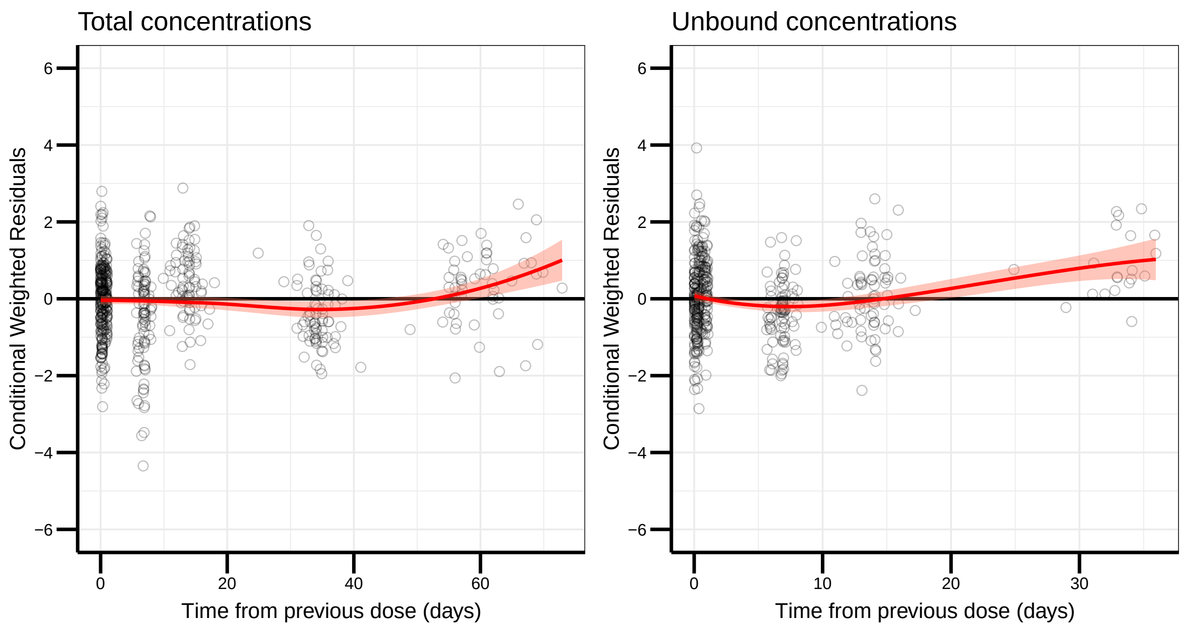

10.4.2 CWRES vs. Time (log scale)

Conditional Weighted Residuals (CWRES) vs. Time from previous dose - Full Model

Note: Solid black lines represent the lines of identity, and red curves the lowess smooth regression curves (with 95% CI).

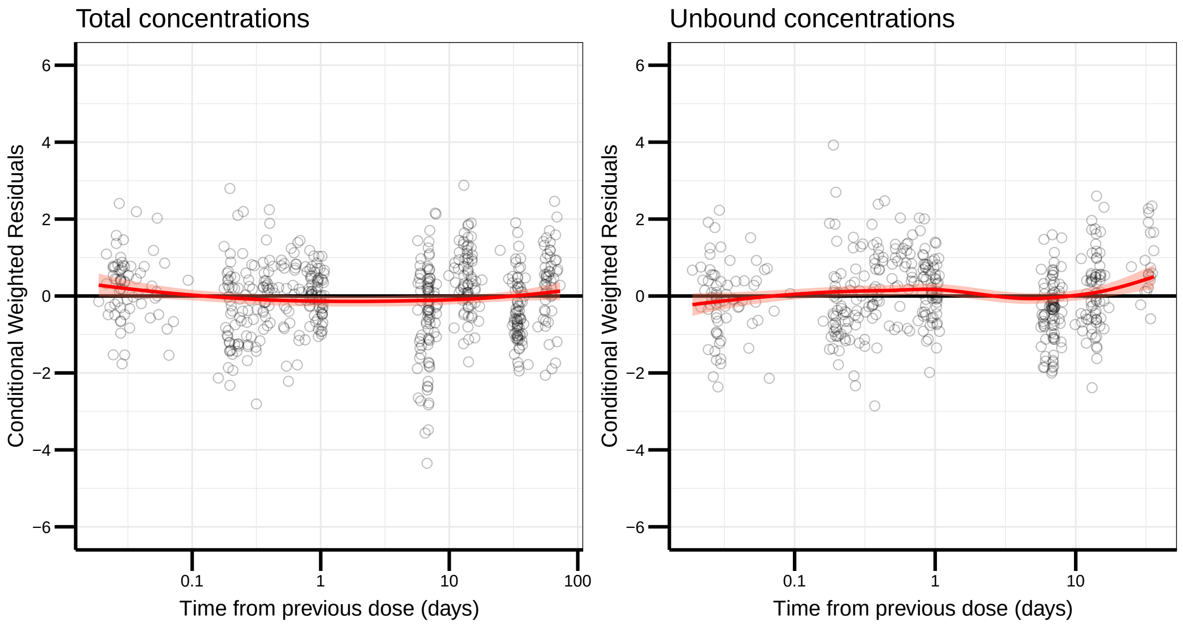

10.4.3 IWRES vs. Time

Individual Weighted Residuals (IWRES) vs. Time from previous dose - Full Model

Note: Solid black lines represent the lines of identity, and red curves the lowess smooth regression curves (with 95% CI).

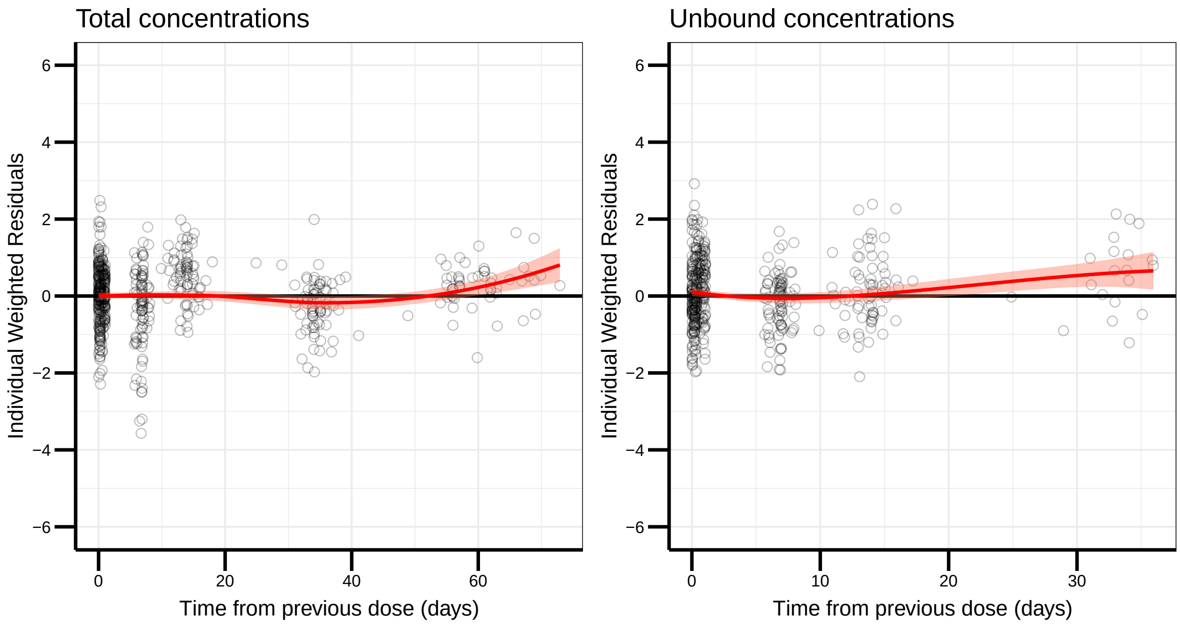

10.4.4 iWRES vs. Time (log scale)

Individual Weighted Residuals (IWRES) vs. Time from previous dose - Full Model

Note: Solid black lines represent the lines of identity, and red curves the lowess smooth regression curves (with 95% CI).

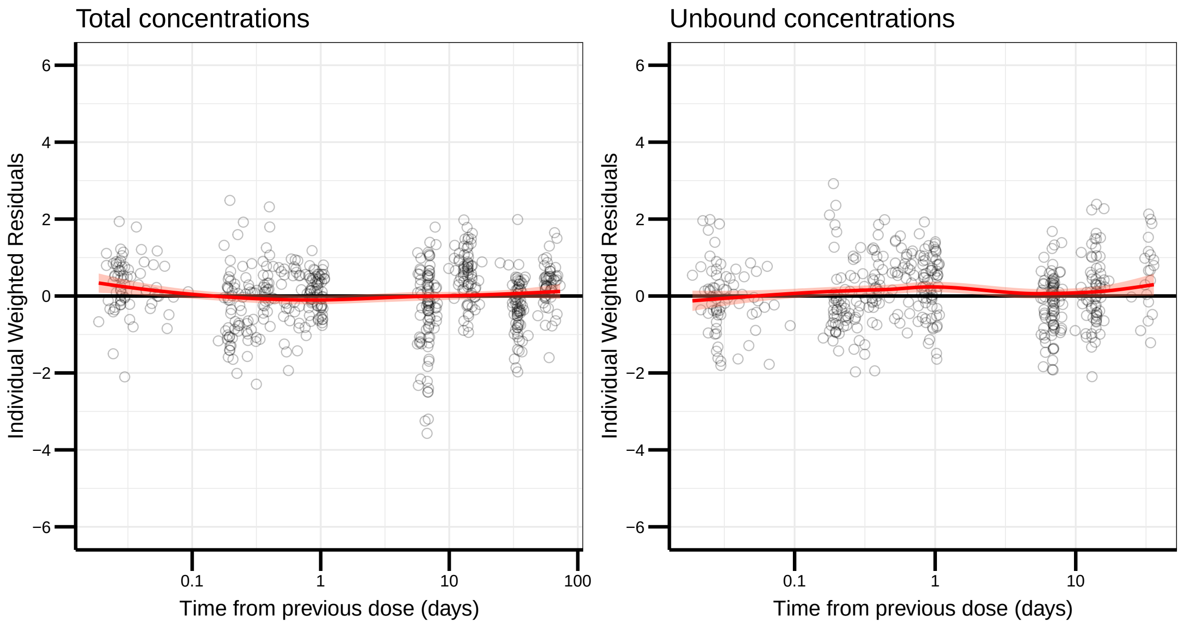

10.5 RES vs. PRED

10.5.1 CWRES vs. PRED

Conditional Weighted Residuals (CWRES) vs. Population predictions - Full Model

Note: Solid black lines represent the lines of identity, and red curves the lowess smooth regression curves (with 95% CI).

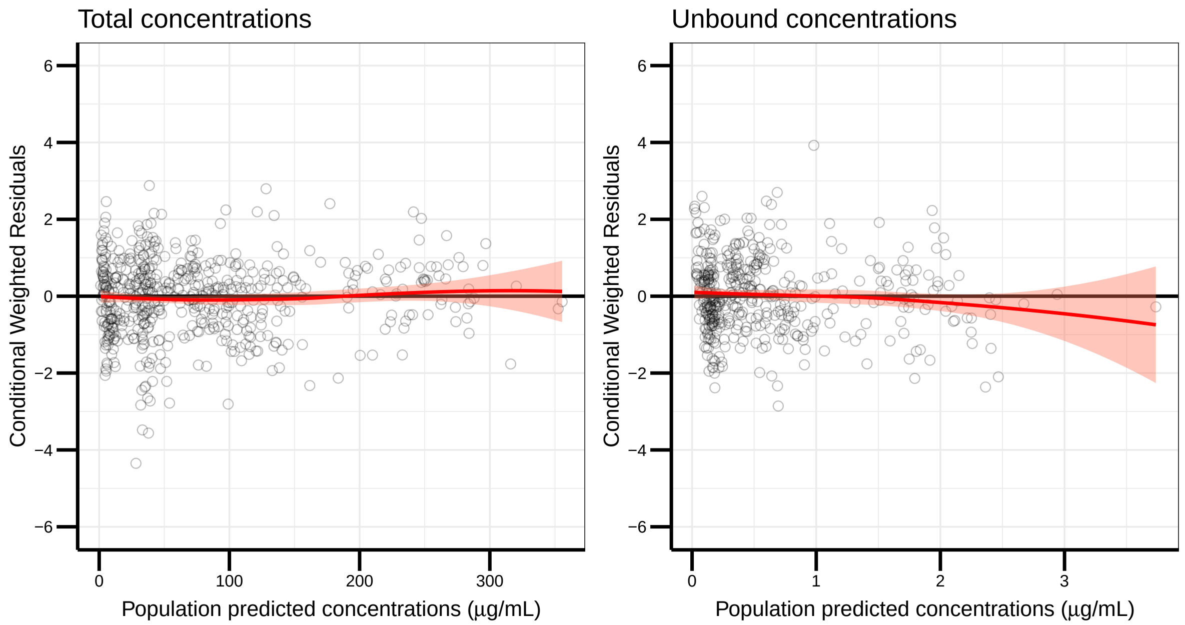

10.5.2 IWRES vs. IPRED

Individual Weighted Residuals (IWRES) vs. Individual predictions - Full Model

Note: Solid black lines represent the lines of identity, and red curves the lowess smooth regression curves (with 95% CI).

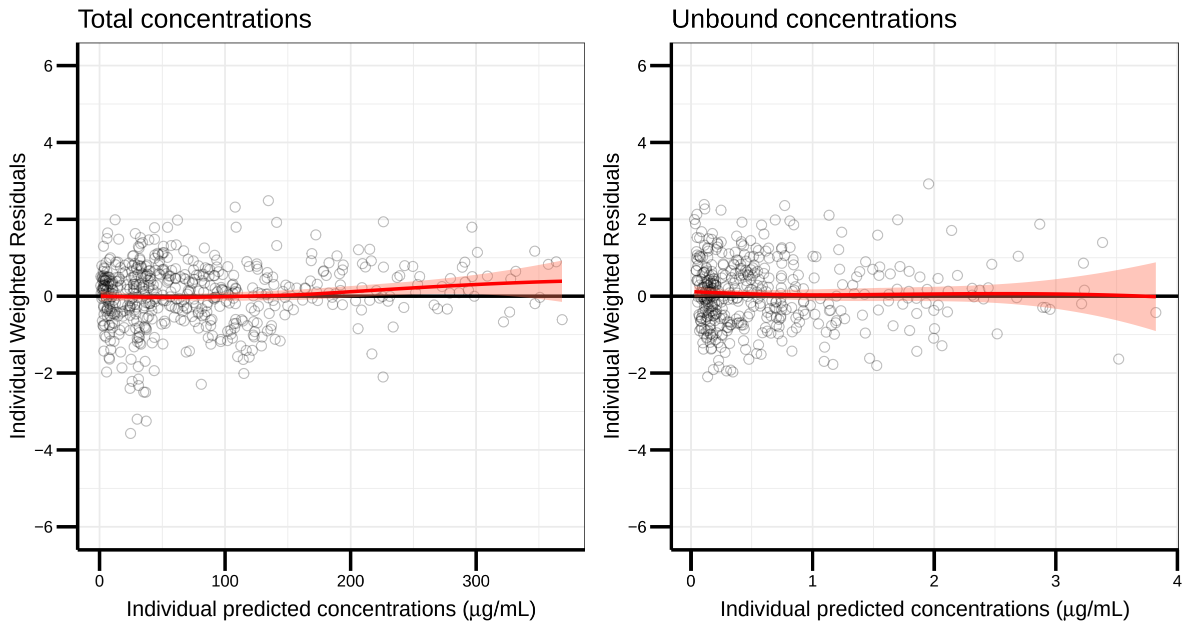

10.5.3 |IWRES| vs IPRED

|Individual Weighted Residuals| (|IWRES|) vs. Individual predictions - Full Model

Note: The red curves are the lowess smooth regression curves (with 95% CI).

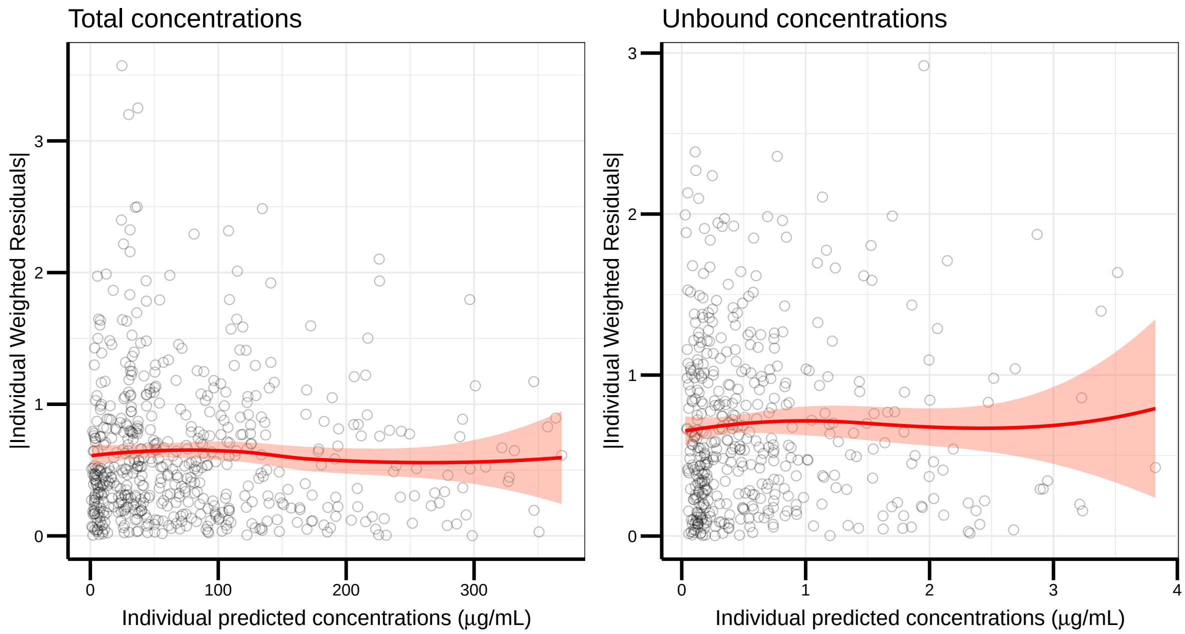

10.6 RES Distribution

10.6.1 CWRES hist

Distribution of Conditional Weighted Residuals (CWRES)

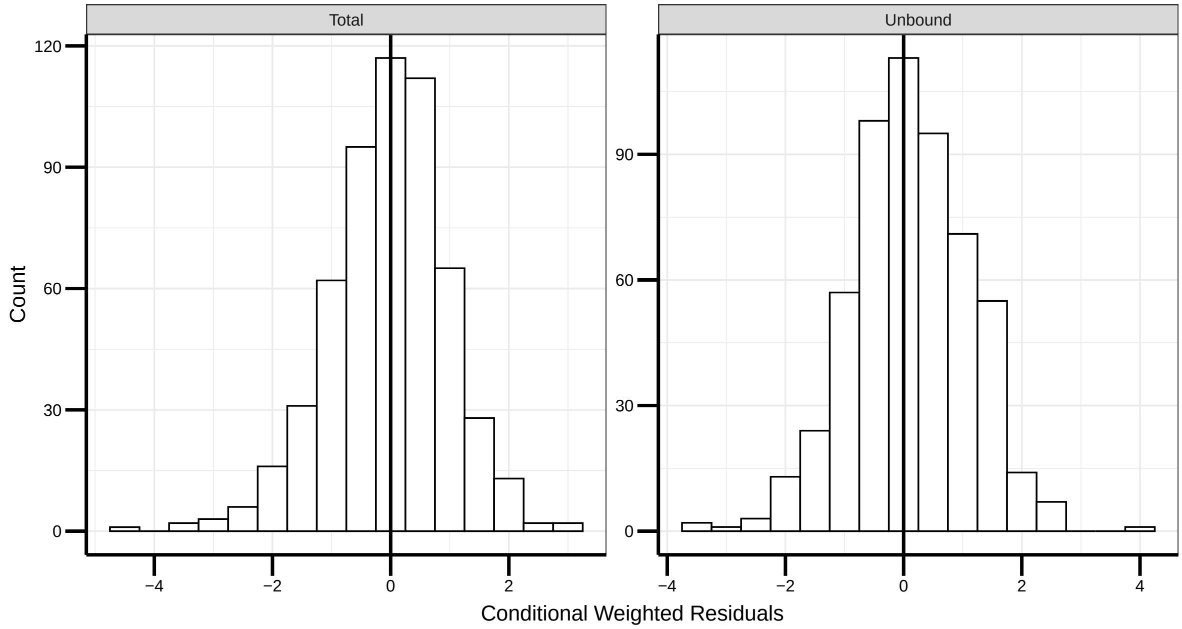

10.6.2 CWRES qqplot

QQplot of Conditional Weighted Residuals (CWRES)

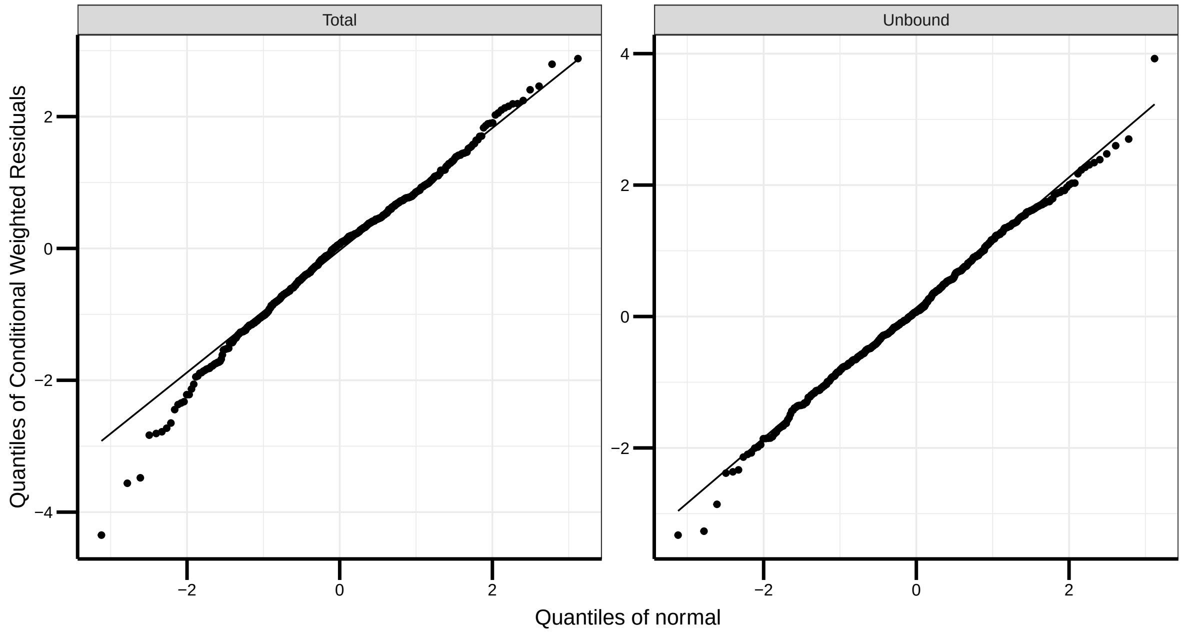

10.6.3 IWRES hist

Distribution of Individual Weighted Residuals (IWRES)

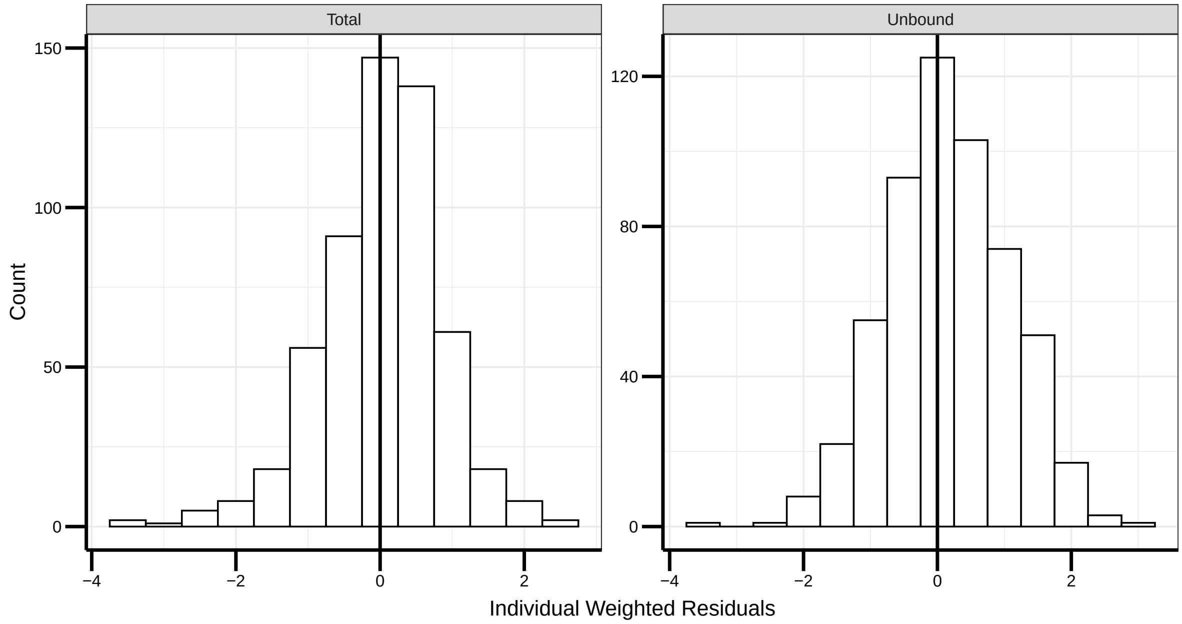

10.6.4 IWRES qqplot

QQplot of Individual Weighted Residuals (IWRES)

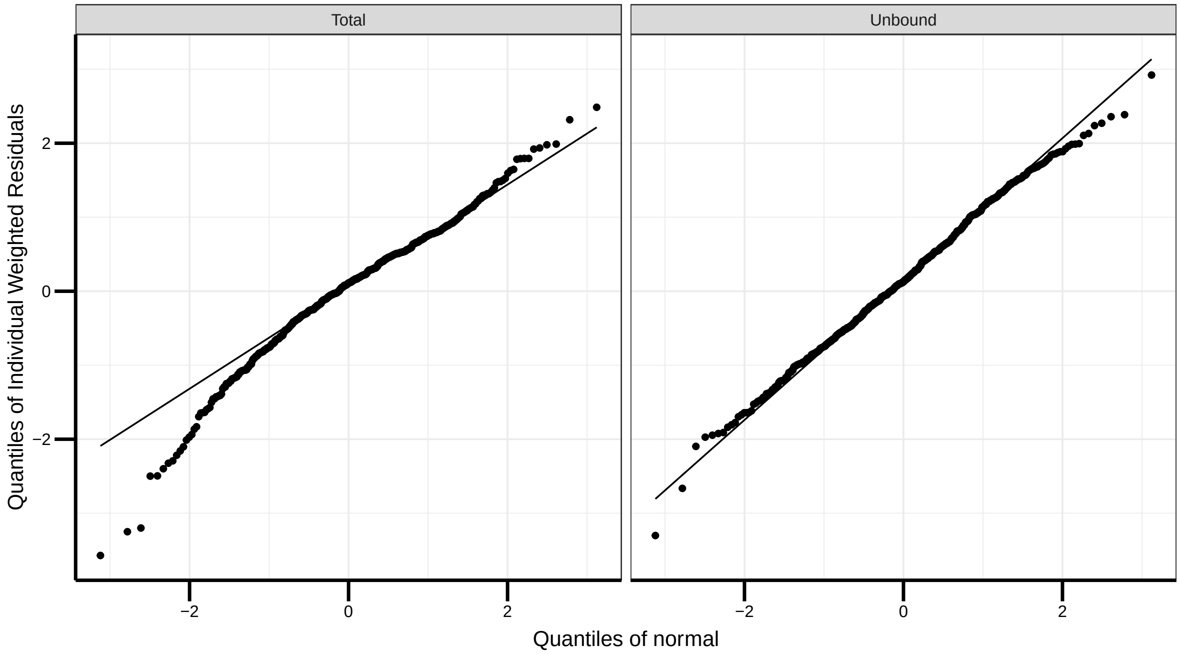

10.7 BSV Distribution

10.7.1 Hist

Distribution of between-subject variability (ETAs) - Full Model

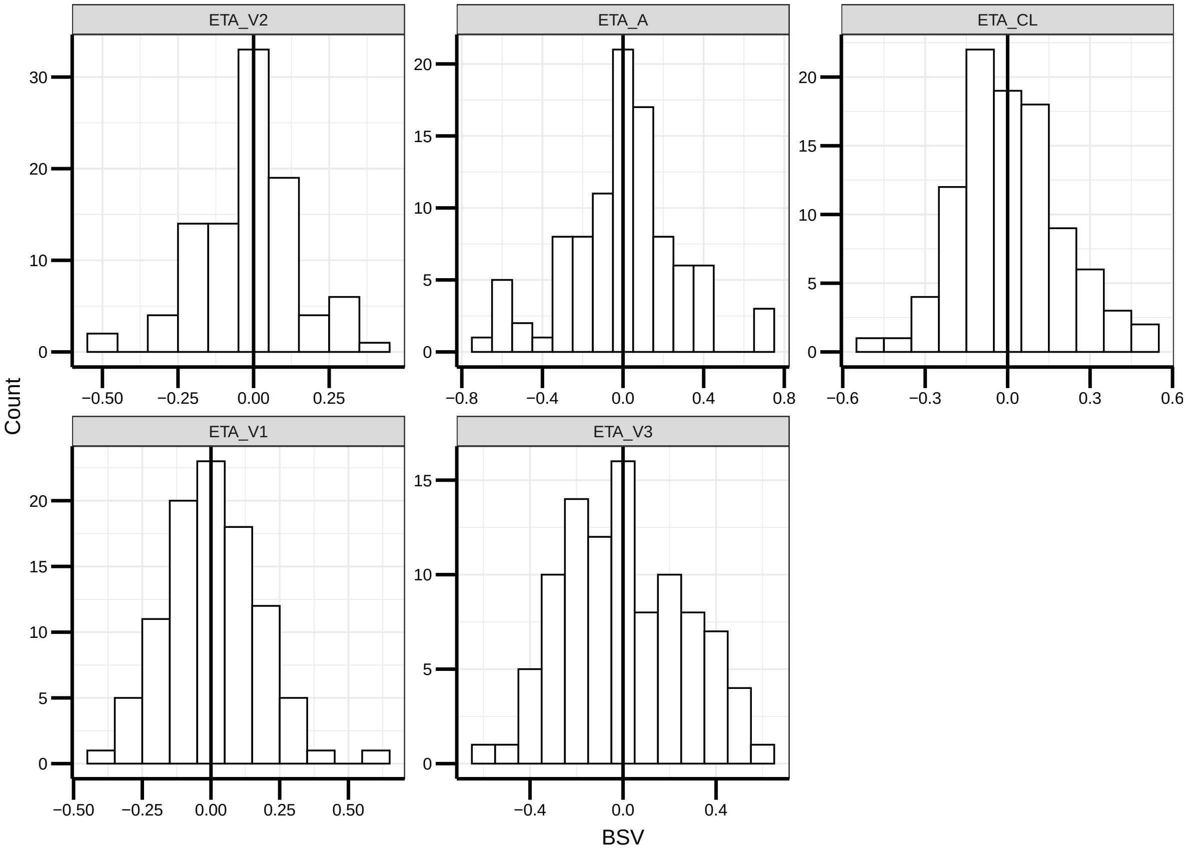

10.7.2 QQplots

QQplot of between-subject variability (ETAs) - Full Model

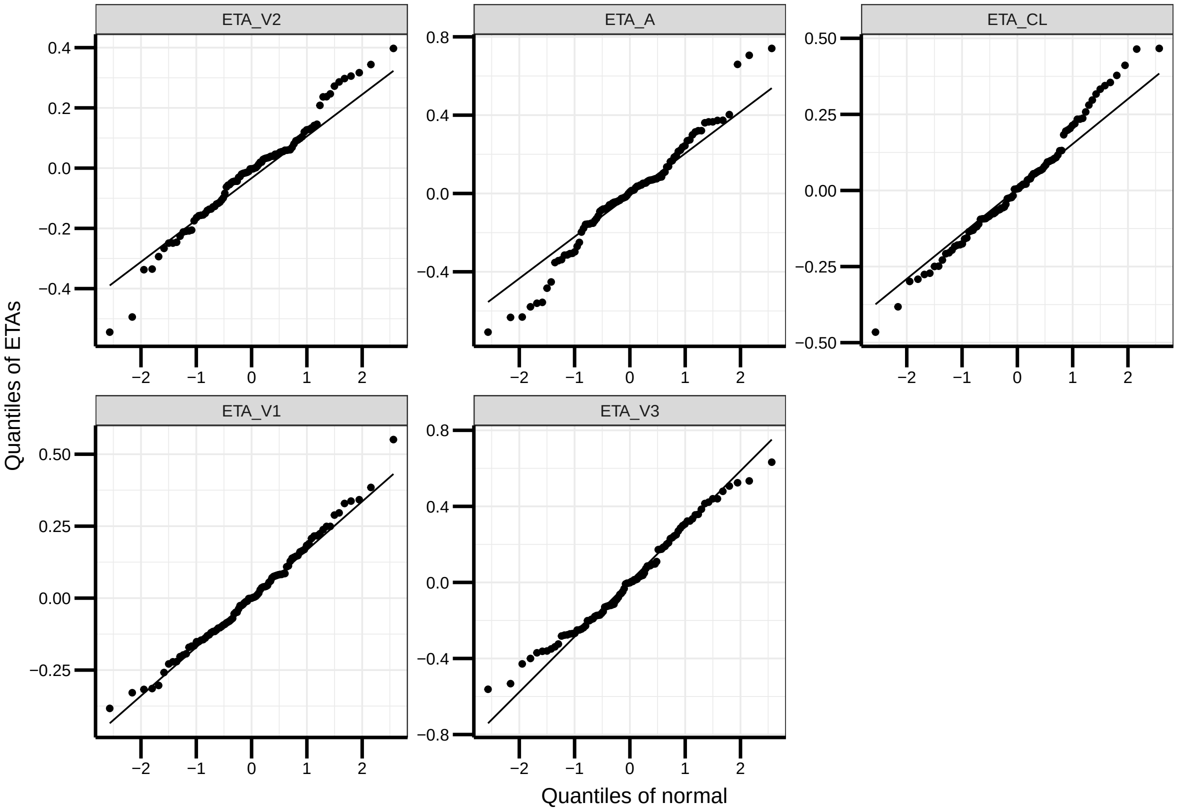

10.8 ETAs Correlations

Correlations of between-subject variability (ETAs) - Full Model

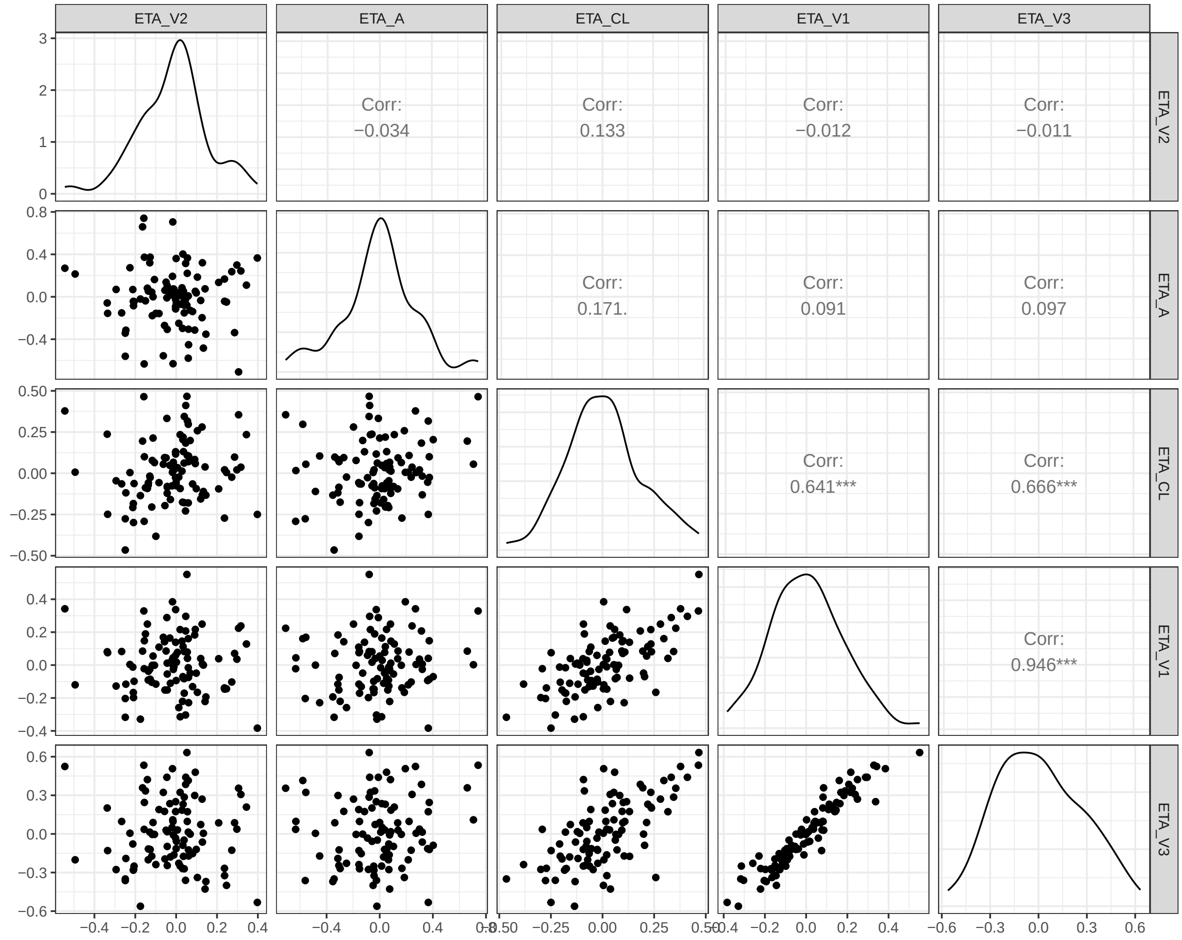

10.9 BSV vs. Cov

> **Note**
>
> - Lab values correspond to baseline values.

Between-subject variability (ETAs) vs. continuous and categorical covariates - Full Model

Note: Red curves correspond to the lowess smooth regression curves (with 95% CI).

10.9.1 Continuous covariates

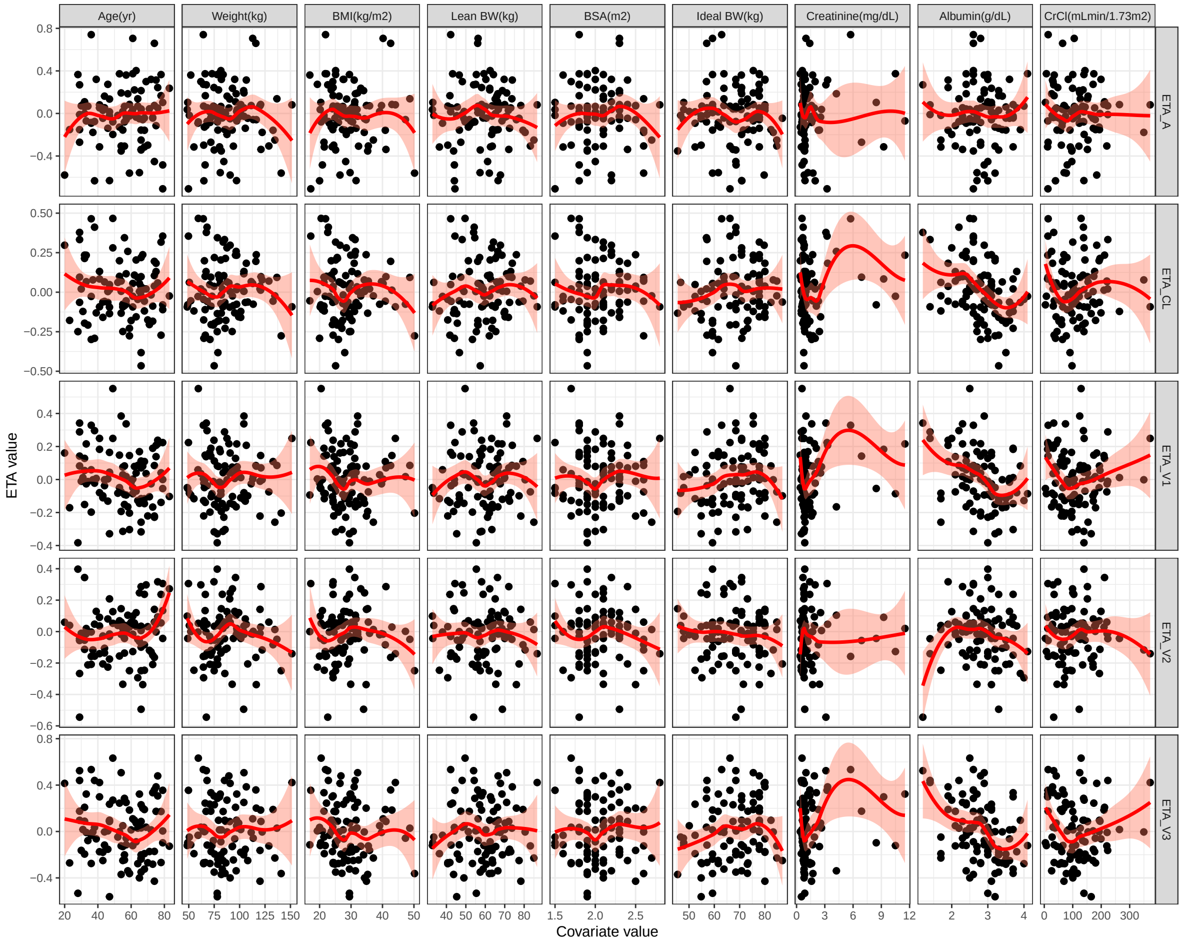

10.9.2 Continuous covariates (Log scale)

Covariate values in log scale

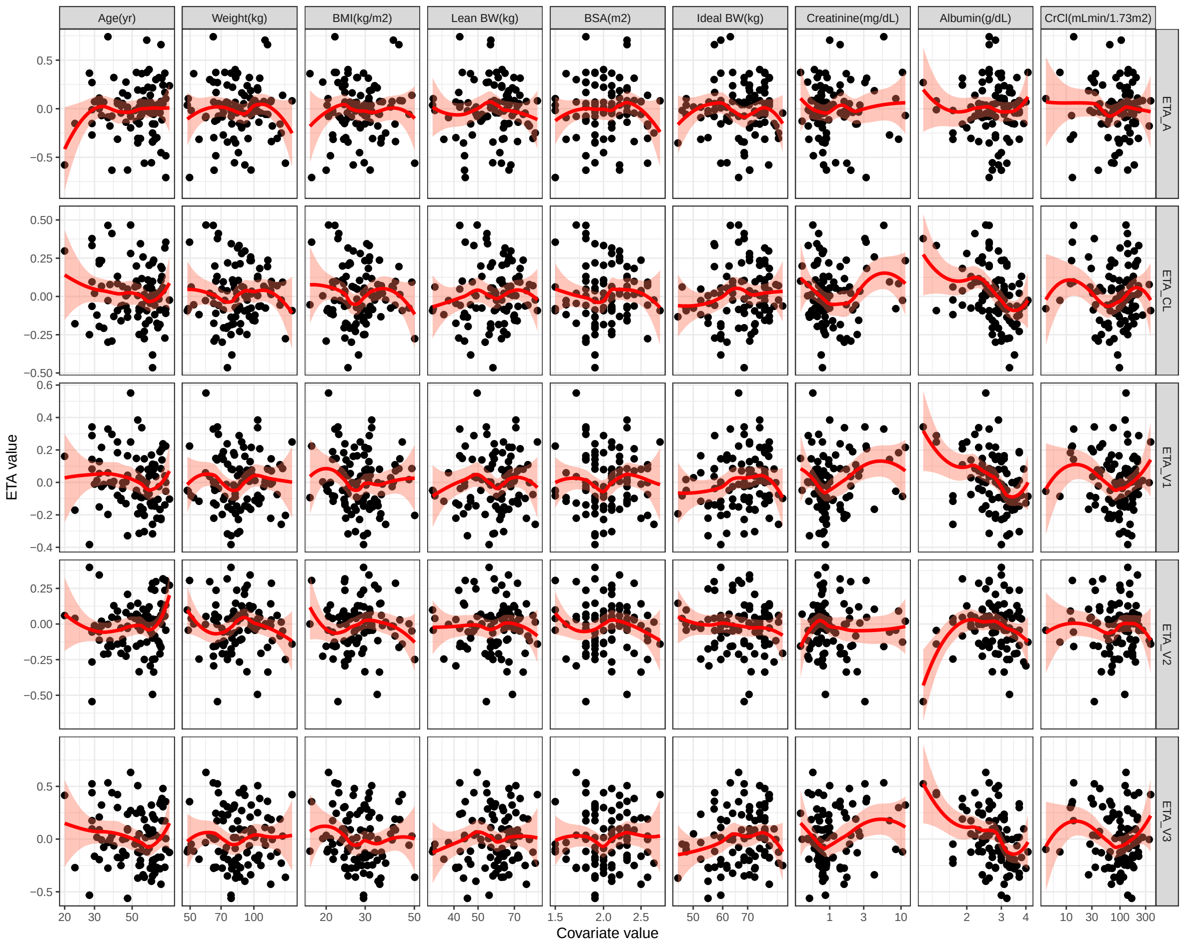
Age(yr)
Weight(kg)
BMI(kg/m2)
Lean BW(kg)
BSA(m2)
Ideal BW(kg)
Creatinine(mg/dL)
Albumin(g/dL)
CrCl(mLmin/1.73m2)
ETA_A
ETA_CL
ETA_V1
ETA_V2
ETA_V3
ETA value
Covariate value

10.9.3 Categorical covariates

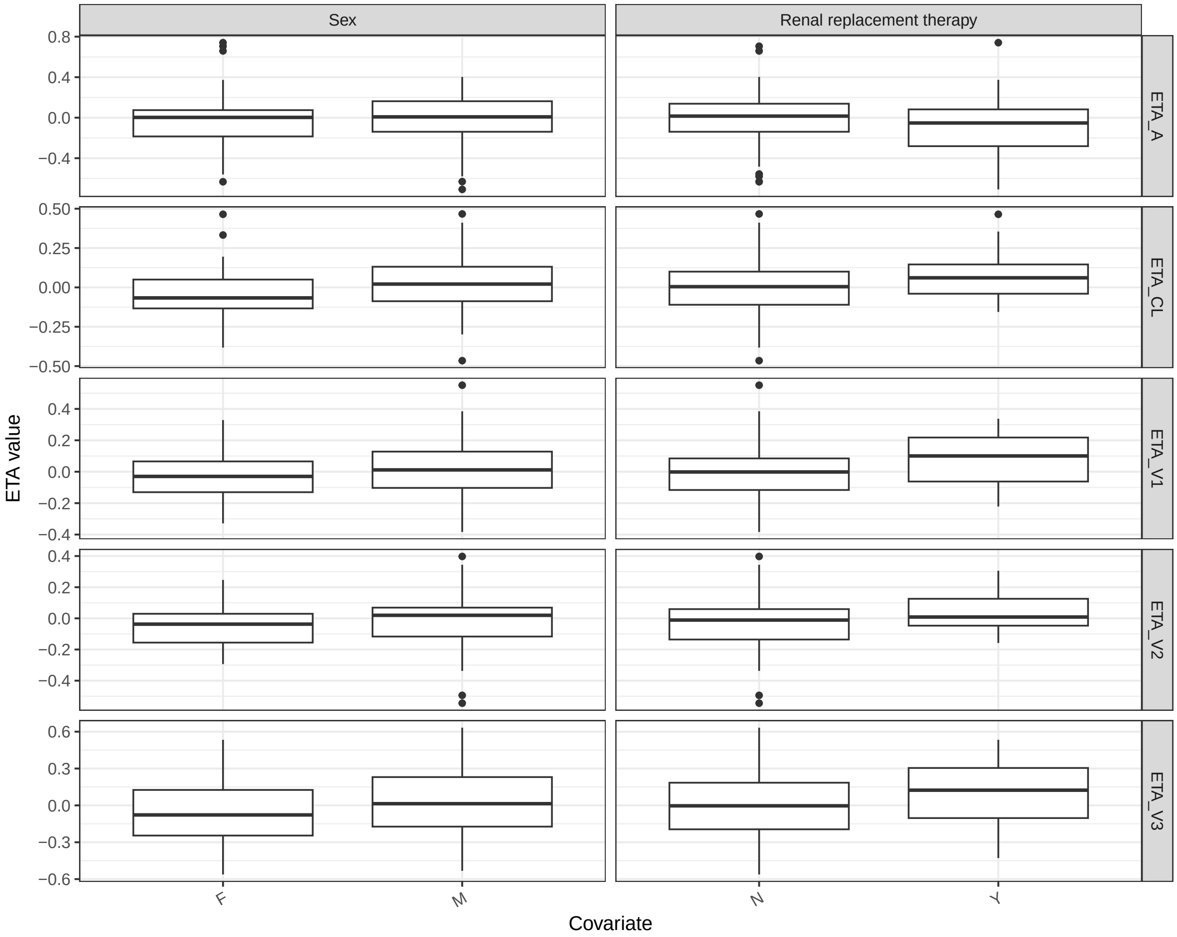

11 INDIVIDUAL FITS

Individual fits were generated in R and exported as a .pdf file in : ./results/04-full-model/indiv-fits-full.pdf

Individual fits with logged time axis in: ./results/04-full-model/indiv-fits-loggedtime-full.pdf

pdf
2

pdf
2

12 INDIVIDUAL PARAMETERS VS COVARIATES

> **Note**
>
> For covariates included in the full popPK model

12.0.1 Continuous covariates

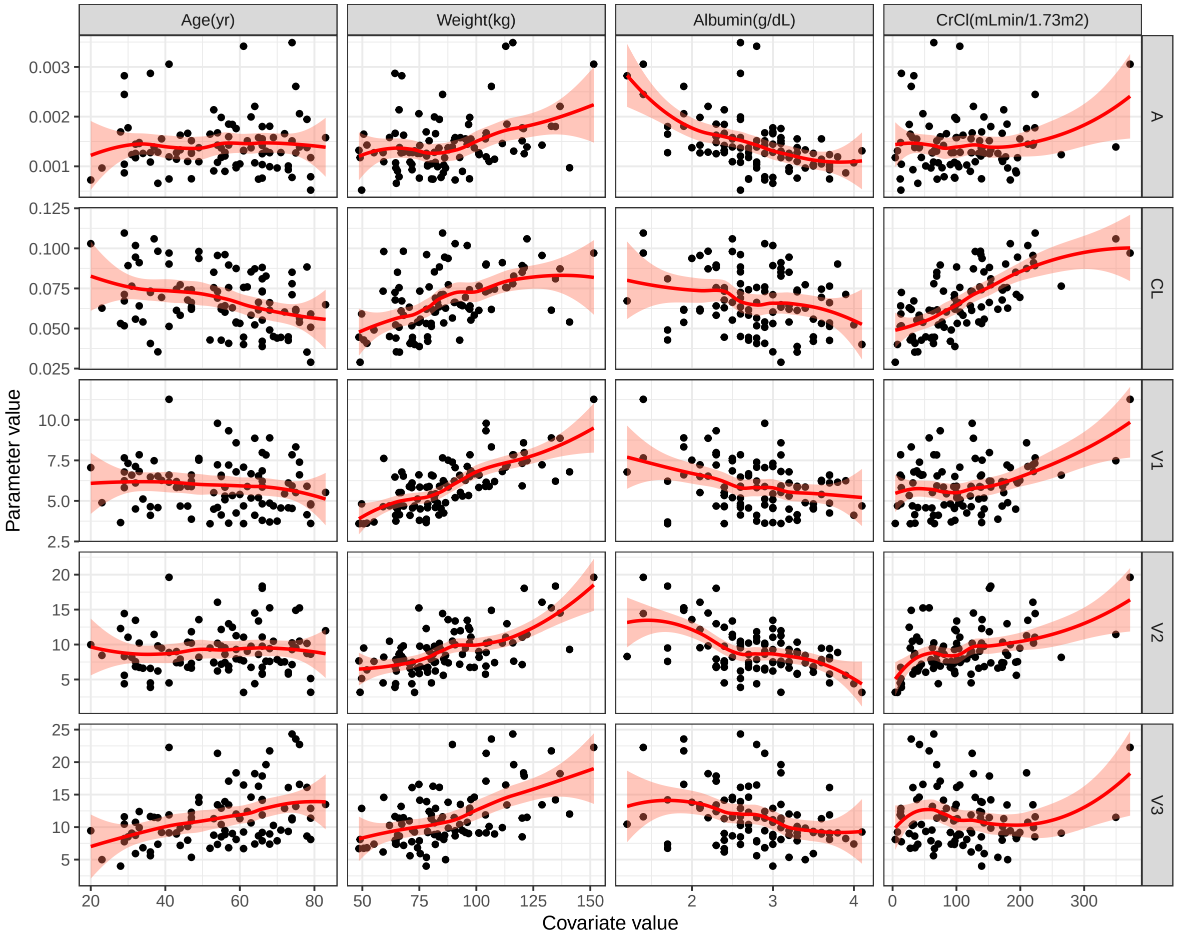

12.0.2 Categorical covariates

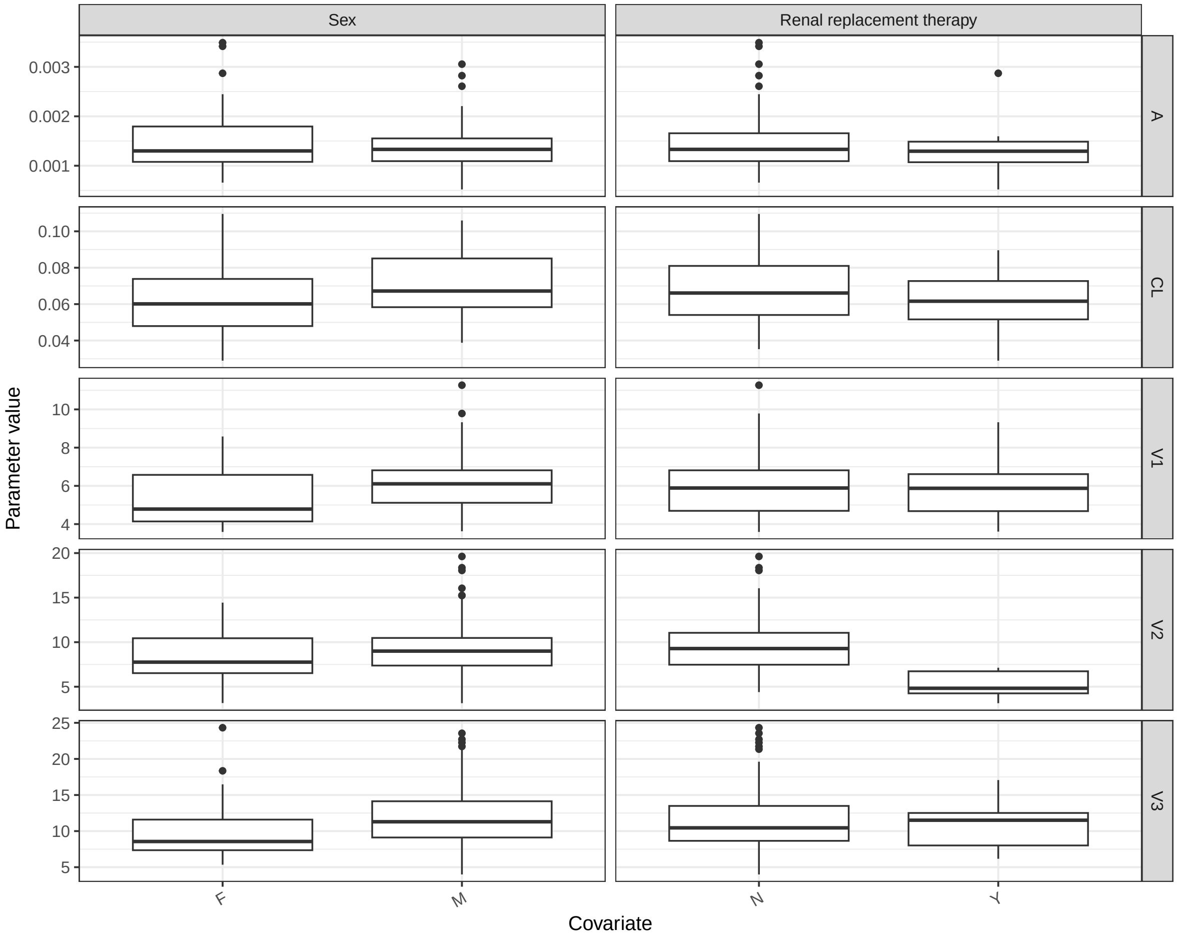

13 COVARIATE-EFFECT PLOTS

13.1 CL

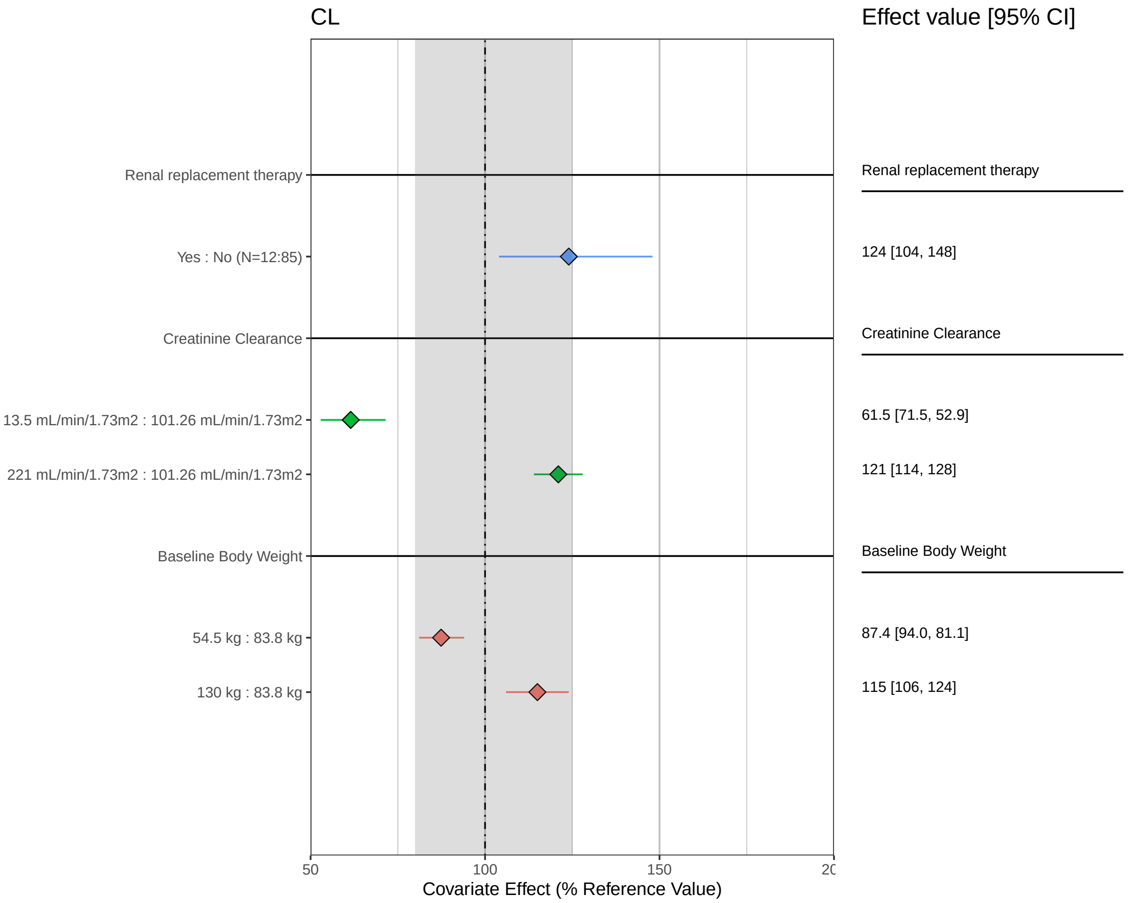

The shaded area corresponds to the interval [80, 125]. For continuous covariates, the 5th and 95th quantiles are compared to the median value.

13.2 V1

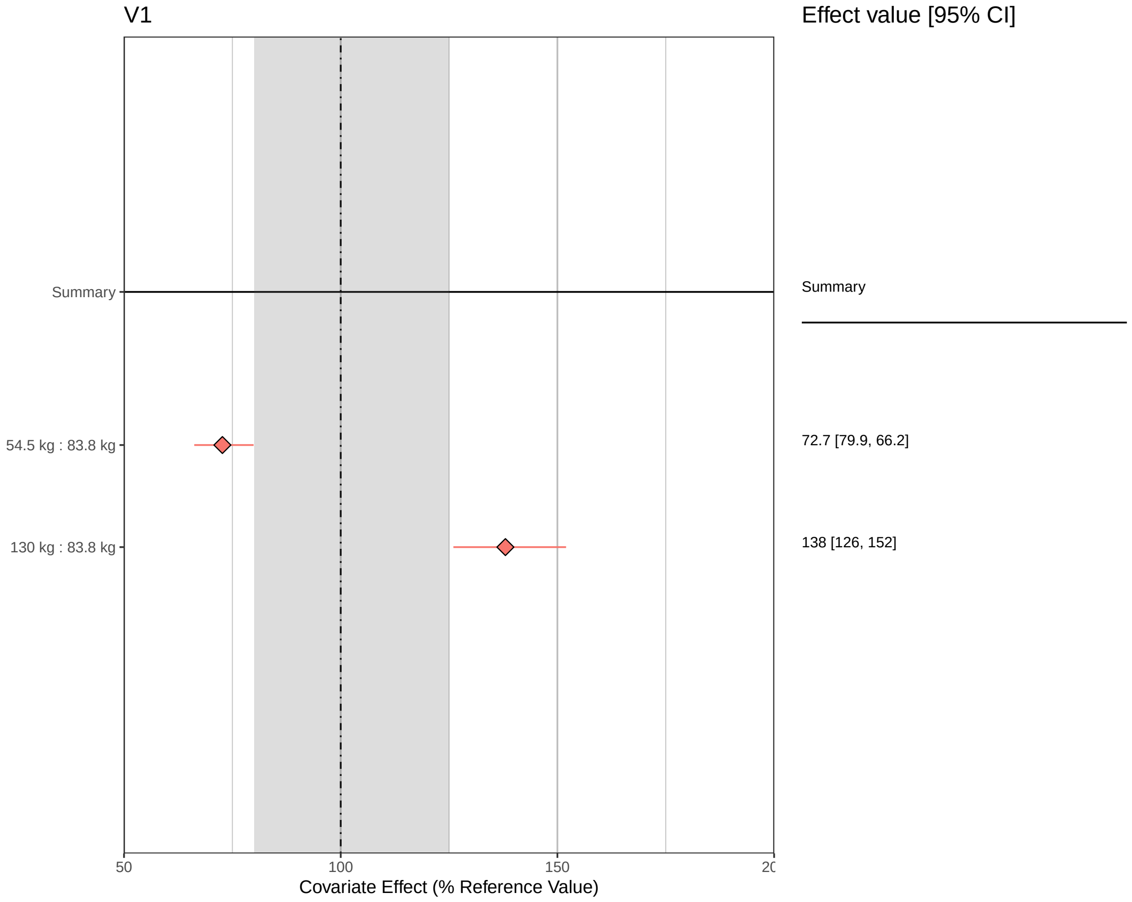

The shaded area corresponds to the interval [80, 125]. For continuous covariates, the 5th and 95th quantiles are compared to the median value.

13.3 V2

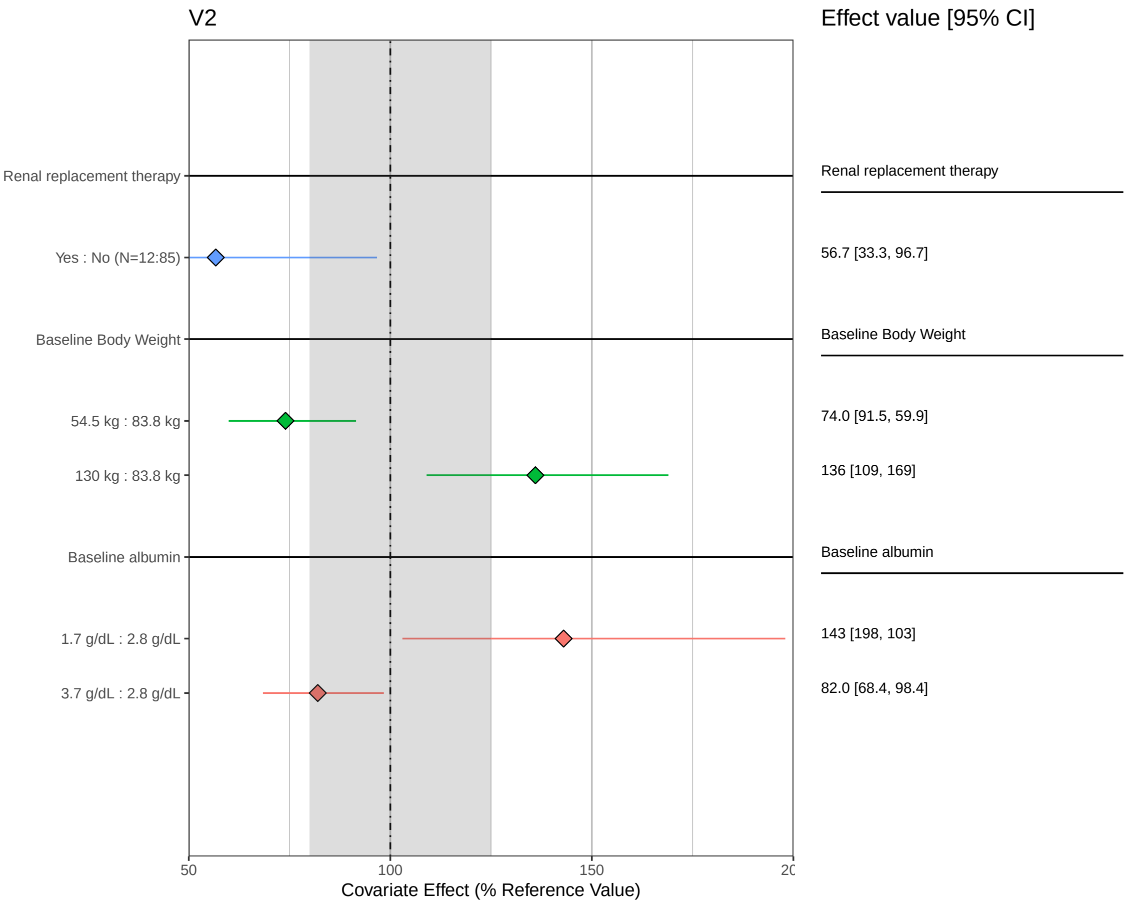

The shaded area corresponds to the interval [80, 125]. For continuous covariates, the 5th and 95th quantiles are compared to the median value.

13.4 V3

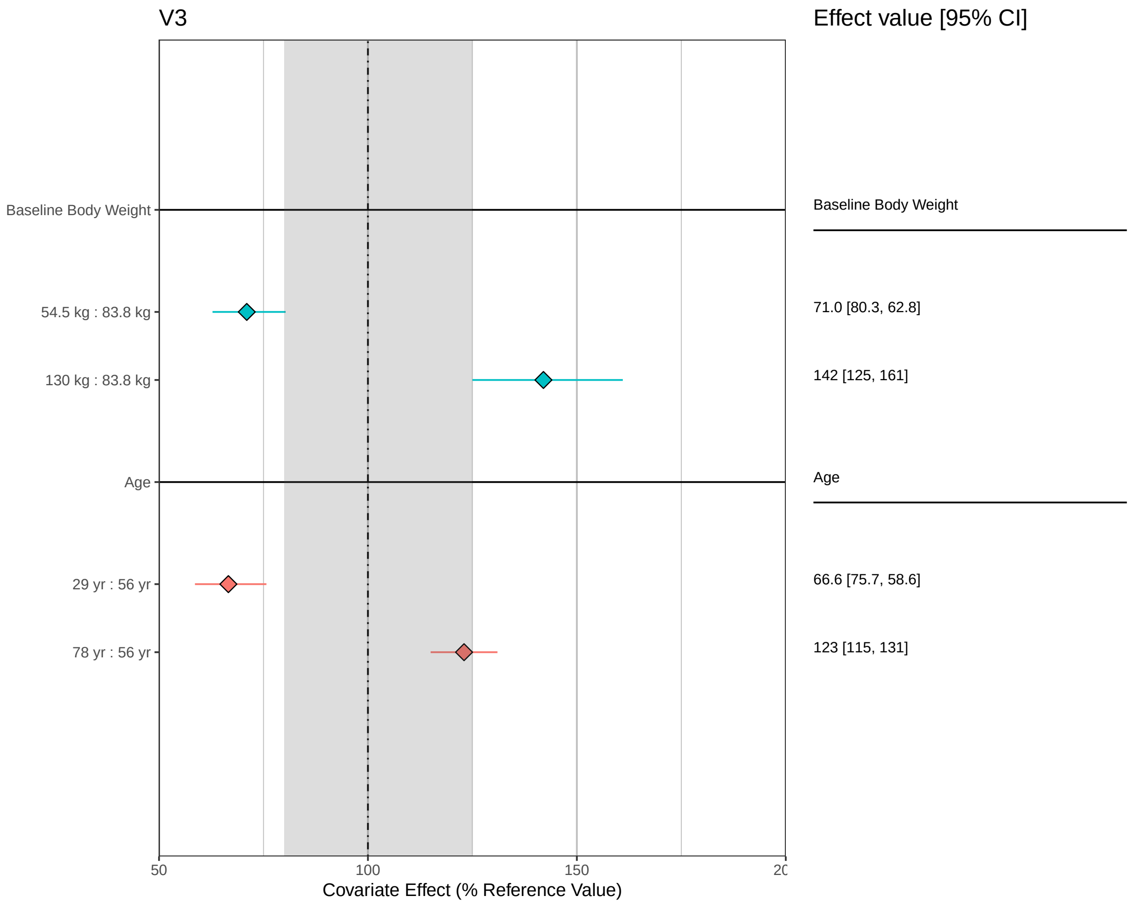

The shaded area corresponds to the interval [80, 125]. For continuous covariates, the 5th and 95th quantiles are compared to the median value.

13.5 A

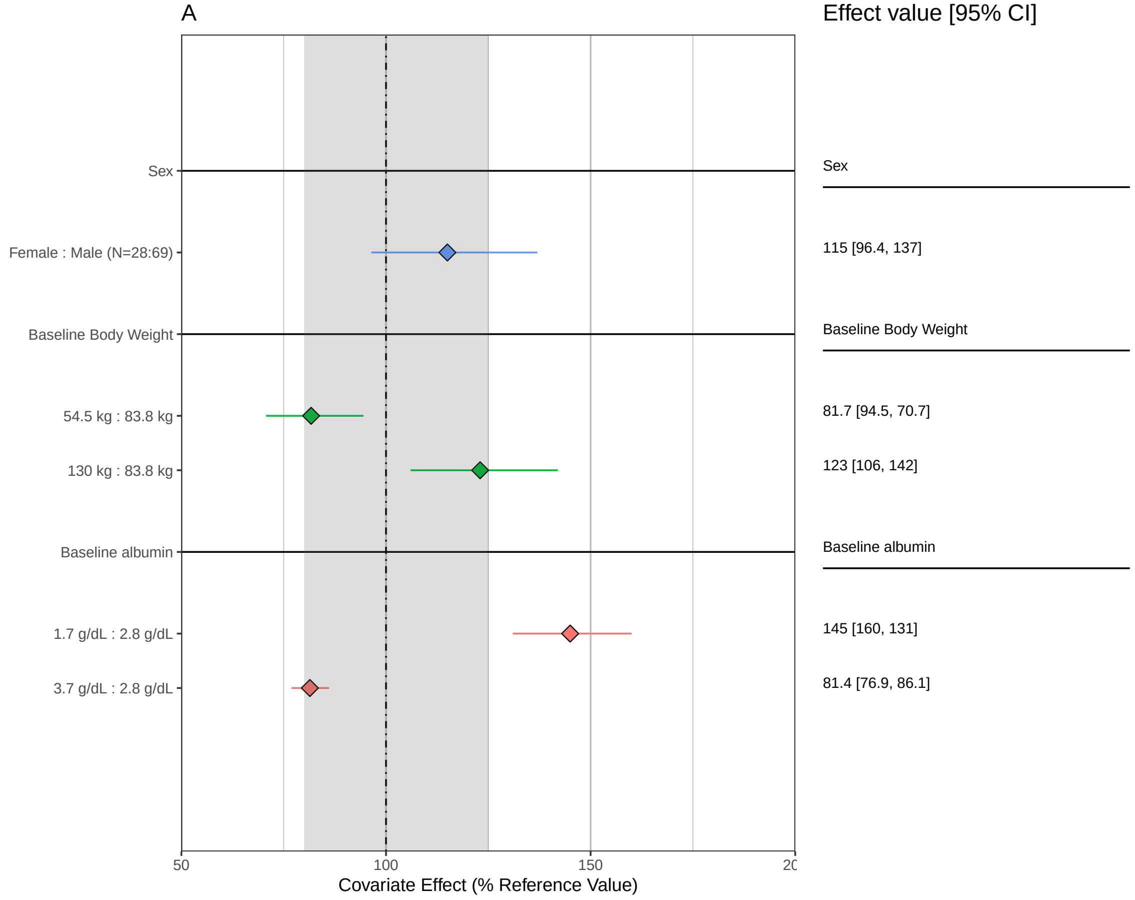

The shaded area corresponds to the interval [80, 125]. For continuous covariates, the 5th and 95th quantiles are compared to the median value.

14 REPRODUCIBILITY

The program code for this analysis is in the following file:

/mnt/data/code/SSC/Emmes/Emmes-Dalbavancin-DOTS/04-full-model.qmd

The packages used in this analysis are listed below:

```
- Session info ---------------------------------------------------------------
 setting  value
 version  R version 4.2.3 (2023-03-15)
 os       Ubuntu 20.04.6 LTS
 system   x86_64, linux-gnu
```

```
ui       X11
language (EN)
collate  en_US.UTF-8
ctype    en_US.UTF-8
tz       UTC
date     2024-06-07
pandoc   2.19.2 @ /usr/lib/rstudio-server/bin/quarto/bin/tools/ (via rmarkdown)

- Packages ------------------------------------------------------------------
! package           * version  date (UTC) lib source
P arsenal           * 3.6.3    2021-06-04 [?] CRAN (R 4.2.3)
P askpass             1.2.0    2023-09-03 [?] CRAN (R 4.2.3)
P assertthat          0.2.1    2019-03-21 [?] CRAN (R 4.2.3)
P backports           1.4.1    2021-12-13 [?] CRAN (R 4.2.3)
P base64enc           0.1-3    2015-07-28 [?] CRAN (R 4.2.3)
P broom.helpers       1.14.0   2023-08-07 [?] CRAN (R 4.2.3)
P checkmate           2.3.1    2023-12-04 [?] CRAN (R 4.2.3)
P class               7.3-21   2023-01-23 [?] CRAN (R 4.2.3)
P classInt            0.4-10   2023-09-05 [?] CRAN (R 4.2.3)
P cli                 3.6.1    2023-03-23 [?] CRAN (R 4.2.3)
P clisymbols          1.2.0    2017-05-21 [?] CRAN (R 4.2.3)
P codetools           0.2-19   2023-02-01 [?] CRAN (R 4.2.3)
P colorspace          2.1-0    2023-01-23 [?] CRAN (R 4.2.3)
P crayon              1.5.2    2022-09-29 [?] CRAN (R 4.2.3)
P crul                1.4.2    2024-04-09 [?] CRAN (R 4.2.3)
P curl                5.2.0    2023-12-08 [?] CRAN (R 4.2.3)
P data.table          1.14.8   2023-02-17 [?] CRAN (R 4.2.3)
P digest              0.6.33   2023-07-07 [?] CRAN (R 4.2.3)
P dplyr             * 1.1.4    2023-11-17 [?] CRAN (R 4.2.3)
P e1071               1.7-14   2023-12-06 [?] CRAN (R 4.2.3)
P ellipsis            0.3.2    2021-04-29 [?] CRAN (R 4.2.3)
P evaluate            0.23     2023-11-01 [?] CRAN (R 4.2.3)
P fansi               1.0.6    2023-12-08 [?] CRAN (R 4.2.3)
P farver              2.1.1    2022-07-06 [?] CRAN (R 4.2.3)
P fastmap             1.1.1    2023-02-24 [?] CRAN (R 4.2.3)
P flextable         * 0.9.6    2024-05-05 [?] CRAN (R 4.2.3)
P fontBitstreamVera   0.1.1    2017-02-01 [?] CRAN (R 4.2.3)
P fontLiberation      0.1.0    2016-10-15 [?] CRAN (R 4.2.3)
P fontquiver          0.2.1    2017-02-01 [?] CRAN (R 4.2.3)
P forcats           * 1.0.0    2023-01-29 [?] CRAN (R 4.2.3)
P fs                  1.6.3    2023-07-20 [?] CRAN (R 4.2.3)
P gdtools             0.3.7    2024-03-05 [?] CRAN (R 4.2.3)
P generics            0.1.3    2022-07-05 [?] CRAN (R 4.2.3)
```

```
P gfonts              0.2.0    2023-01-08 [?] CRAN (R 4.2.3)
P GGally            * 2.2.0    2023-11-22 [?] CRAN (R 4.2.3)
P ggforce           * 0.4.1    2022-10-04 [?] CRAN (R 4.2.3)
P ggplot2           * 3.4.4    2023-10-12 [?] CRAN (R 4.2.3)
P ggstats             0.5.1    2023-11-21 [?] CRAN (R 4.2.3)
P glue                1.6.2    2022-02-24 [?] CRAN (R 4.2.3)
P gridExtra         * 2.3      2017-09-09 [?] CRAN (R 4.2.3)
P gt                * 0.10.1   2024-01-17 [?] CRAN (R 4.2.3)
P gtable              0.3.4    2023-08-21 [?] CRAN (R 4.2.3)
P gtsummary         * 1.7.2    2023-07-15 [?] CRAN (R 4.2.3)
P haven             * 2.5.4    2023-11-30 [?] CRAN (R 4.2.3)
P here                1.0.1    2020-12-13 [?] CRAN (R 4.2.3)
P hms               * 1.1.3    2023-03-21 [?] CRAN (R 4.2.3)
P htmltools           0.5.7    2023-11-03 [?] CRAN (R 4.2.3)
P httpcode            0.3.0    2020-04-10 [?] CRAN (R 4.2.3)
P httpuv              1.6.12   2023-10-23 [?] CRAN (R 4.2.3)
P janitor           * 2.2.0    2023-02-02 [?] CRAN (R 4.2.3)
P jsonlite            1.8.7    2023-06-29 [?] CRAN (R 4.2.3)
P kableExtra        * 1.4.0    2024-01-24 [?] CRAN (R 4.2.3)
P KernSmooth          2.23-20  2021-05-03 [?] CRAN (R 4.2.3)
P knitr               1.45     2023-10-30 [?] CRAN (R 4.2.3)
P labeling            0.4.3    2023-08-29 [?] CRAN (R 4.2.3)
P labelled          * 2.12.0   2023-06-21 [?] CRAN (R 4.2.3)
P later               1.3.1    2023-05-02 [?] CRAN (R 4.2.3)
P lattice             0.20-45  2021-09-22 [?] CRAN (R 4.2.3)
P lifecycle           1.0.4    2023-11-07 [?] CRAN (R 4.2.3)
P lubridate         * 1.9.3    2023-09-27 [?] CRAN (R 4.2.3)
P magick              2.8.2    2023-12-20 [?] CRAN (R 4.2.3)
P magrittr          * 2.0.3    2022-03-30 [?] CRAN (R 4.2.3)
P MASS                7.3-58.2 2023-01-23 [?] CRAN (R 4.2.3)
P Matrix              1.6-0    2023-07-08 [?] RSPM (R 4.2.0)
P MatrixModels        0.5-2    2023-07-10 [?] RSPM (R 4.2.0)
P matrixStats         1.2.0    2023-12-11 [?] CRAN (R 4.2.3)
P mgcv                1.8-42   2023-03-02 [?] CRAN (R 4.2.3)
P mime                0.12     2021-09-28 [?] CRAN (R 4.2.3)
P munsell             0.5.0    2018-06-12 [?] CRAN (R 4.2.3)
P nlme                3.1-162  2023-01-31 [?] CRAN (R 4.2.3)
P officer             0.6.6    2024-05-05 [?] CRAN (R 4.2.3)
P openssl             2.1.1    2023-09-25 [?] CRAN (R 4.2.3)
P pander              0.6.5    2022-03-18 [?] CRAN (R 4.2.3)
P patchwork           1.2.0    2024-01-08 [?] CRAN (R 4.2.3)
P pillar              1.9.0    2023-03-22 [?] CRAN (R 4.2.3)
P pkgconfig           2.0.3    2019-09-22 [?] CRAN (R 4.2.3)
```

```
P plyr               1.8.9    2023-10-02 [?] CRAN (R 4.2.3)
P pmforest         * 0.2.0    2024-02-05 [?] MPNDEV (R 4.2.3)
P pmtables           0.6.0    2024-02-05 [?] MPNDEV (R 4.2.3)
P polyclip           1.10-6   2023-09-27 [?] CRAN (R 4.2.3)
P promises           1.2.1    2023-08-10 [?] CRAN (R 4.2.3)
P proxy              0.4-27   2022-06-09 [?] CRAN (R 4.2.3)
P pryr               0.1.6    2023-01-17 [?] CRAN (R 4.2.3)
P purrr            * 1.0.2    2023-08-10 [?] CRAN (R 4.2.3)
P quantreg           5.96     2023-07-19 [?] RSPM (R 4.2.0)
P R.cache            0.16.0   2022-07-21 [?] CRAN (R 4.2.3)
P R.methodsS3        1.8.2    2022-06-13 [?] CRAN (R 4.2.3)
P R.oo               1.26.0   2024-01-24 [?] CRAN (R 4.2.3)
P R.utils            2.12.3   2023-11-18 [?] CRAN (R 4.2.3)
P R6                 2.5.1    2021-08-19 [?] CRAN (R 4.2.3)
P ragg               1.2.7    2023-12-11 [?] CRAN (R 4.2.3)
P rapportools        1.1      2022-03-22 [?] CRAN (R 4.2.3)
P RColorBrewer       1.1-3    2022-04-03 [?] CRAN (R 4.2.3)
P Rcpp               1.0.11   2023-07-06 [?] CRAN (R 4.2.3)
P readr            * 2.1.5    2024-01-10 [?] CRAN (R 4.2.3)
  renv               1.0.3    2023-09-19 [1] CRAN (R 4.2.3)
P reshape2         * 1.4.4    2020-04-09 [?] CRAN (R 4.2.3)
P rlang              1.1.2    2023-11-04 [?] CRAN (R 4.2.3)
P rmarkdown          2.27     2024-05-17 [?] CRAN (R 4.2.3)
P rprojroot          2.0.4    2023-11-05 [?] CRAN (R 4.2.3)
P rstudioapi         0.15.0   2023-07-07 [?] CRAN (R 4.2.3)
P scales             1.3.0    2023-11-28 [?] CRAN (R 4.2.3)
P sessioninfo      * 1.2.2    2021-12-06 [?] CRAN (R 4.2.3)
P shiny              1.7.5.1  2023-10-14 [?] CRAN (R 4.2.3)
P snakecase          0.11.1   2023-08-27 [?] CRAN (R 4.2.3)
P SparseM            1.81     2021-02-18 [?] CRAN (R 4.2.3)
P stringi            1.8.1    2023-11-13 [?] RSPM (R 4.2.0)
P stringr          * 1.5.1    2023-11-14 [?] CRAN (R 4.2.3)
P styler           * 1.10.2   2023-08-29 [?] CRAN (R 4.2.3)
P summarytools     * 1.0.1    2022-05-20 [?] CRAN (R 4.2.3)
P survival           3.5-3    2023-02-12 [?] CRAN (R 4.2.3)
P svglite            2.1.3    2023-12-08 [?] CRAN (R 4.2.3)
P systemfonts        1.0.5    2023-10-09 [?] CRAN (R 4.2.3)
P textshaping        0.3.7    2023-10-09 [?] CRAN (R 4.2.3)
P tibble           * 3.2.1    2023-03-20 [?] CRAN (R 4.2.3)
P tidylog          * 1.0.2    2020-07-03 [?] CRAN (R 4.2.3)
P tidyr            * 1.3.1    2024-01-24 [?] CRAN (R 4.2.3)
P tidyselect       * 1.2.0    2022-10-10 [?] CRAN (R 4.2.3)
P tidyverse        * 2.0.0    2023-02-22 [?] CRAN (R 4.2.3)
```

```
 P tidyvpc          * 1.5.1    2024-01-18 [?] CRAN (R 4.2.3)
 P timechange         0.3.0    2024-01-18 [?] CRAN (R 4.2.3)
 P tweenr             2.0.2    2022-09-06 [?] CRAN (R 4.2.3)
 P tzdb               0.4.0    2023-05-12 [?] CRAN (R 4.2.3)
 P utf8               1.2.4    2023-10-22 [?] CRAN (R 4.2.3)
 P uuid               1.2-0    2024-01-14 [?] CRAN (R 4.2.3)
 P vctrs              0.6.4    2023-10-12 [?] CRAN (R 4.2.3)
 P viridisLite        0.4.2    2023-05-02 [?] CRAN (R 4.2.3)
 P withr              2.5.2    2023-10-30 [?] CRAN (R 4.2.3)
 P xfun               0.41     2023-11-01 [?] CRAN (R 4.2.3)
 P xml2               1.3.6    2023-12-04 [?] CRAN (R 4.2.3)
 P xtable             1.8-4    2019-04-21 [?] CRAN (R 4.2.3)
 P yaml               2.3.7    2023-01-23 [?] CRAN (R 4.2.3)
 P zip                2.3.1    2024-01-27 [?] CRAN (R 4.2.3)
 P zoo              * 1.8-12   2023-04-13 [?] CRAN (R 4.2.3)

 [1] /mnt/data/code/SSC/Emmes/Emmes-Dalbavancin-DOTS/renv/library/R-4.2/x86_64-pc-linux-gnu
 [2] /mnt/data/.cache/R/renv/sandbox/R-4.2/x86_64-pc-linux-gnu/e11edd0e

 P -- Loaded and on-disk path mismatch.

------------------------------------------------------------------------------
```

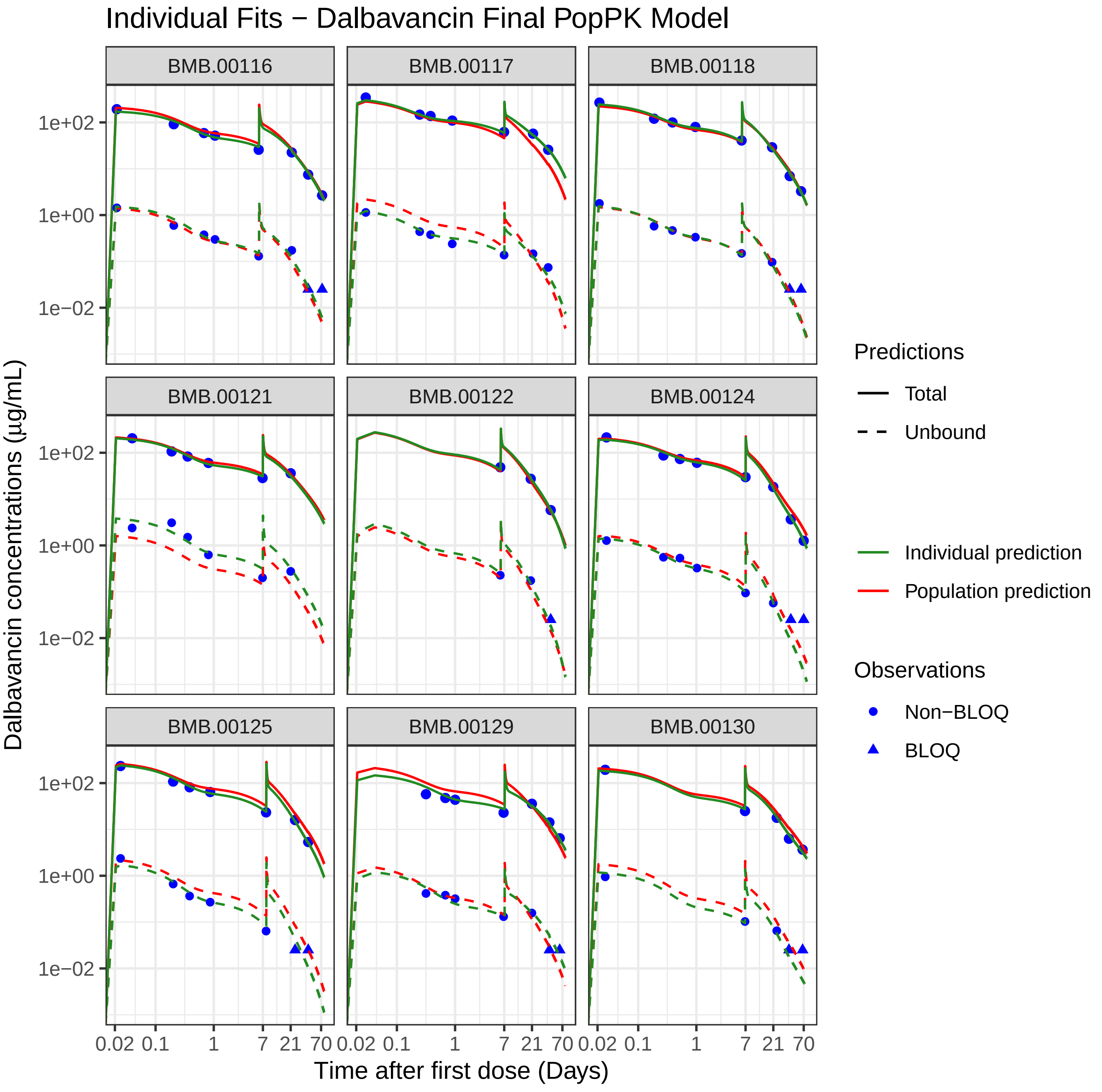
Individual Fits − Dalbavancin Final PopPK Model
BMB.00116
BMB.00117
BMB.00118
BMB.00121
BMB.00122
BMB.00124
BMB.00125
BMB.00129
BMB.00130
Dalbavancin concentrations (µg/mL)
1e+02
1e+00
1e−02
Time after first dose (Days)
0.02 0.1 1 7 21 70
Predictions
Total
Unbound
Individual prediction
Population prediction
Observations
Non−BLOQ
BLOQ

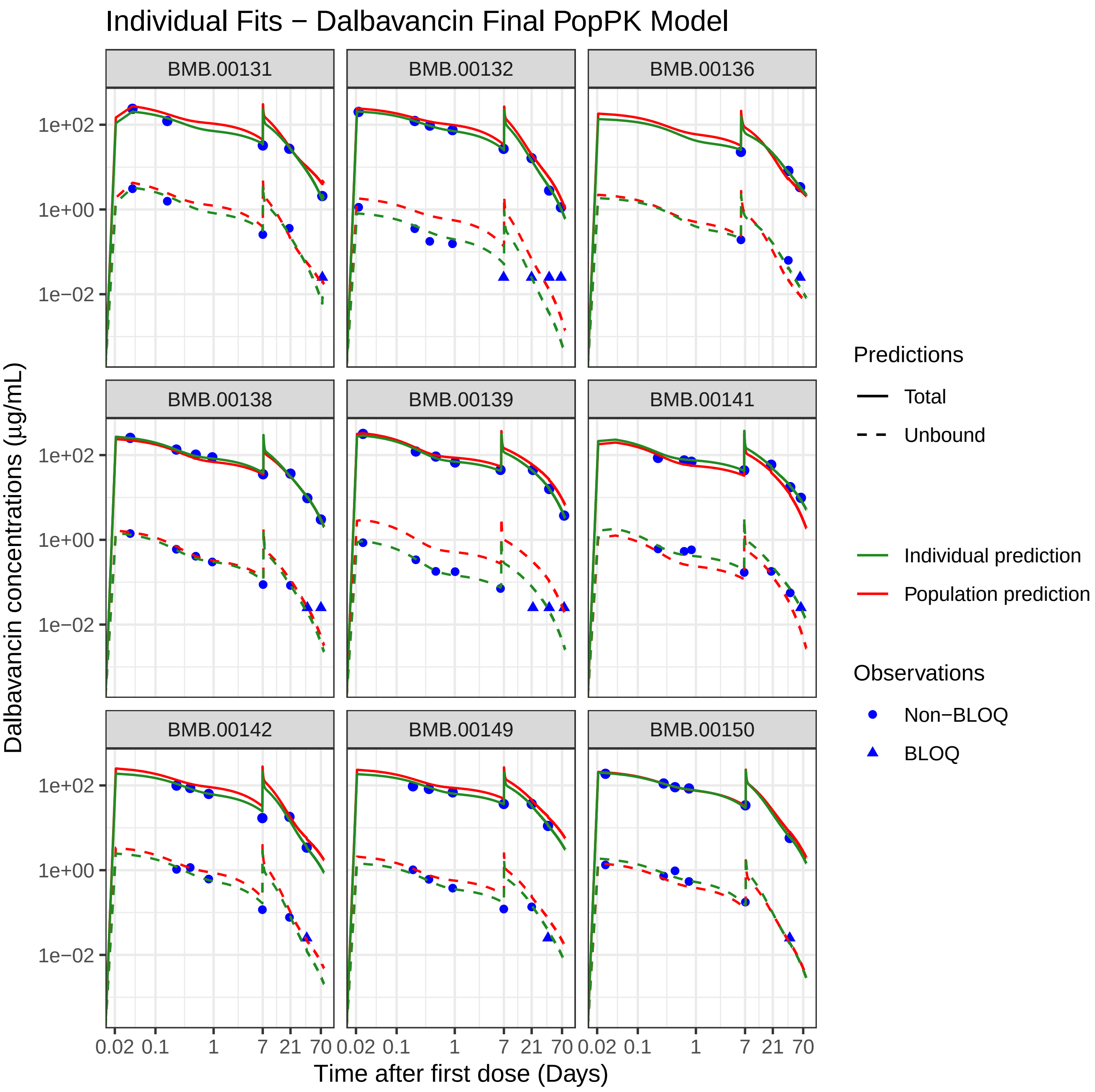
Individual Fits − Dalbavancin Final PopPK Model
BMB.00131
BMB.00132
BMB.00136
BMB.00138
BMB.00139
BMB.00141
BMB.00142
BMB.00149
BMB.00150
1e+02
1e+00
1e−02
0.02 0.1 1 7 21 70
Dalbavancin concentrations (µg/mL)
Time after first dose (Days)
Predictions
Total
Unbound
Individual prediction
Population prediction
Observations
Non−BLOQ
BLOQ

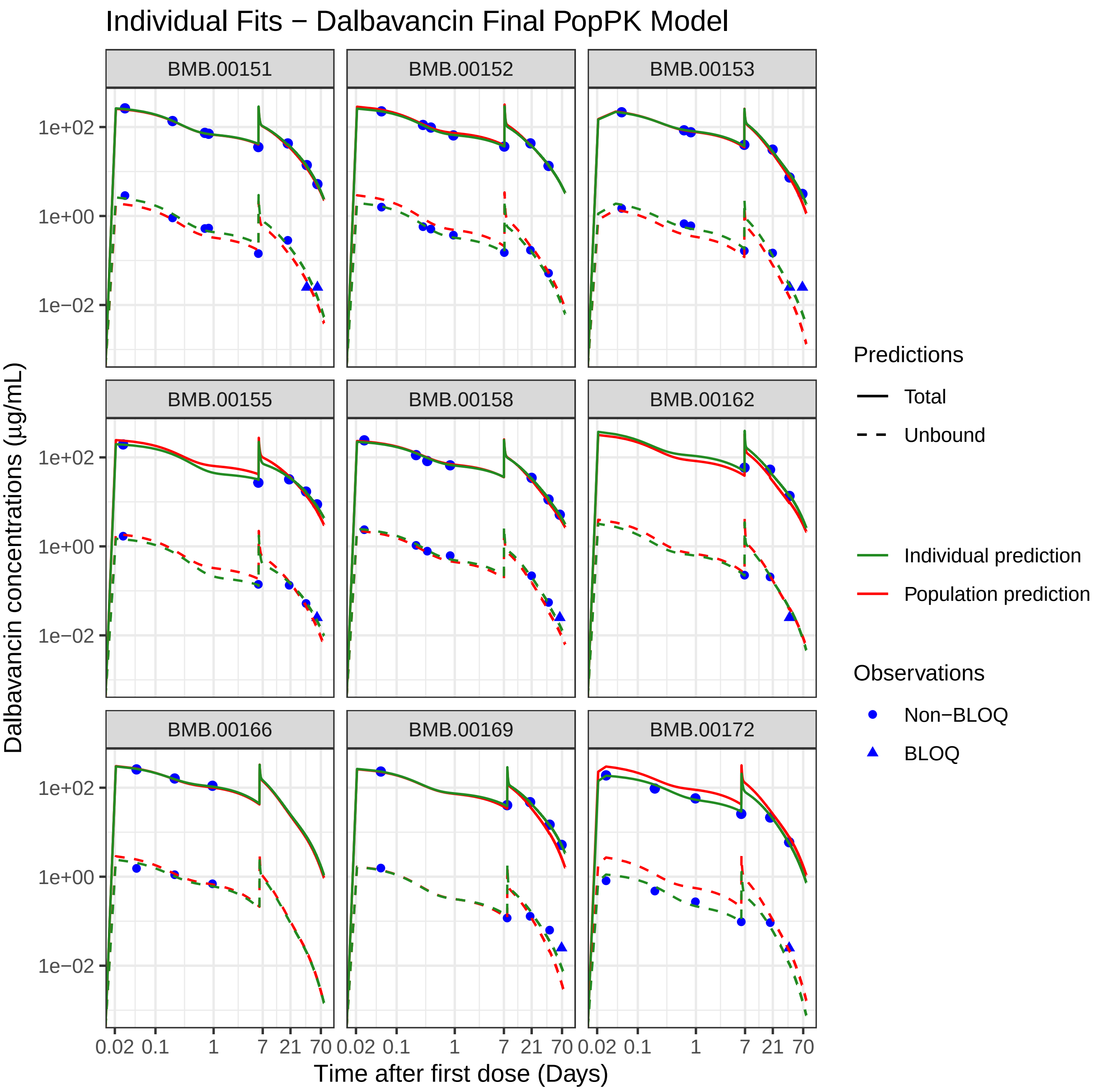
Individual Fits − Dalbavancin Final PopPK Model
BMB.00151
BMB.00152
BMB.00153
BMB.00155
BMB.00158
BMB.00162
BMB.00166
BMB.00169
BMB.00172
Dalbavancin concentrations (µg/mL)
1e+02
1e+00
1e−02
0.02
0.1
1
7
21
70
Time after first dose (Days)
Predictions
Total
Unbound
Individual prediction
Population prediction
Observations
Non−BLOQ
BLOQ

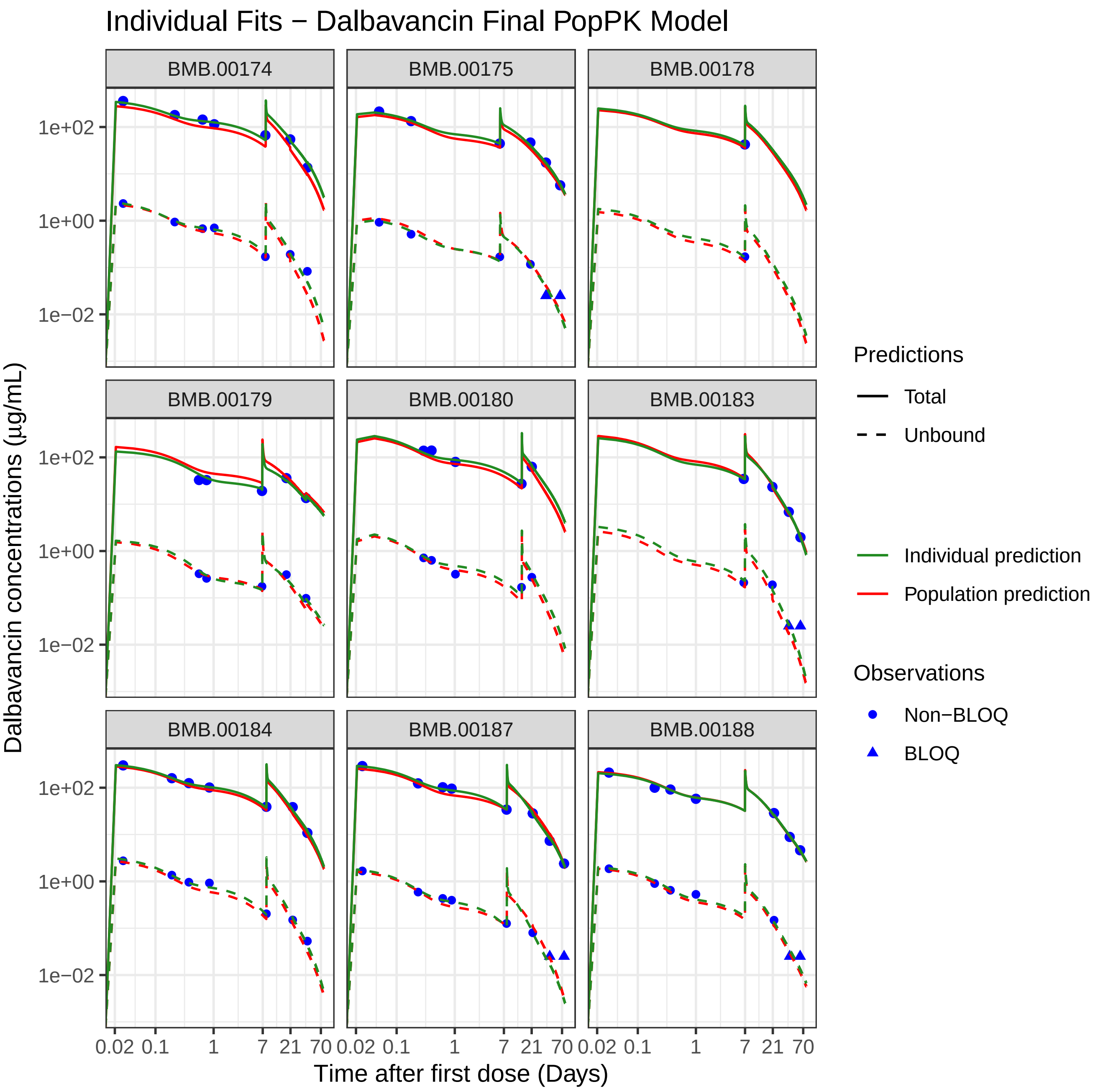
Individual Fits − Dalbavancin Final PopPK Model
BMB.00174
BMB.00175
BMB.00178
BMB.00179
BMB.00180
BMB.00183
BMB.00184
BMB.00187
BMB.00188
Dalbavancin concentrations (µg/mL)
1e+02
1e+00
1e−02
0.02 0.1 1 7 21 70
Time after first dose (Days)
Predictions
Total
Unbound
Individual prediction
Population prediction
Observations
Non−BLOQ
BLOQ

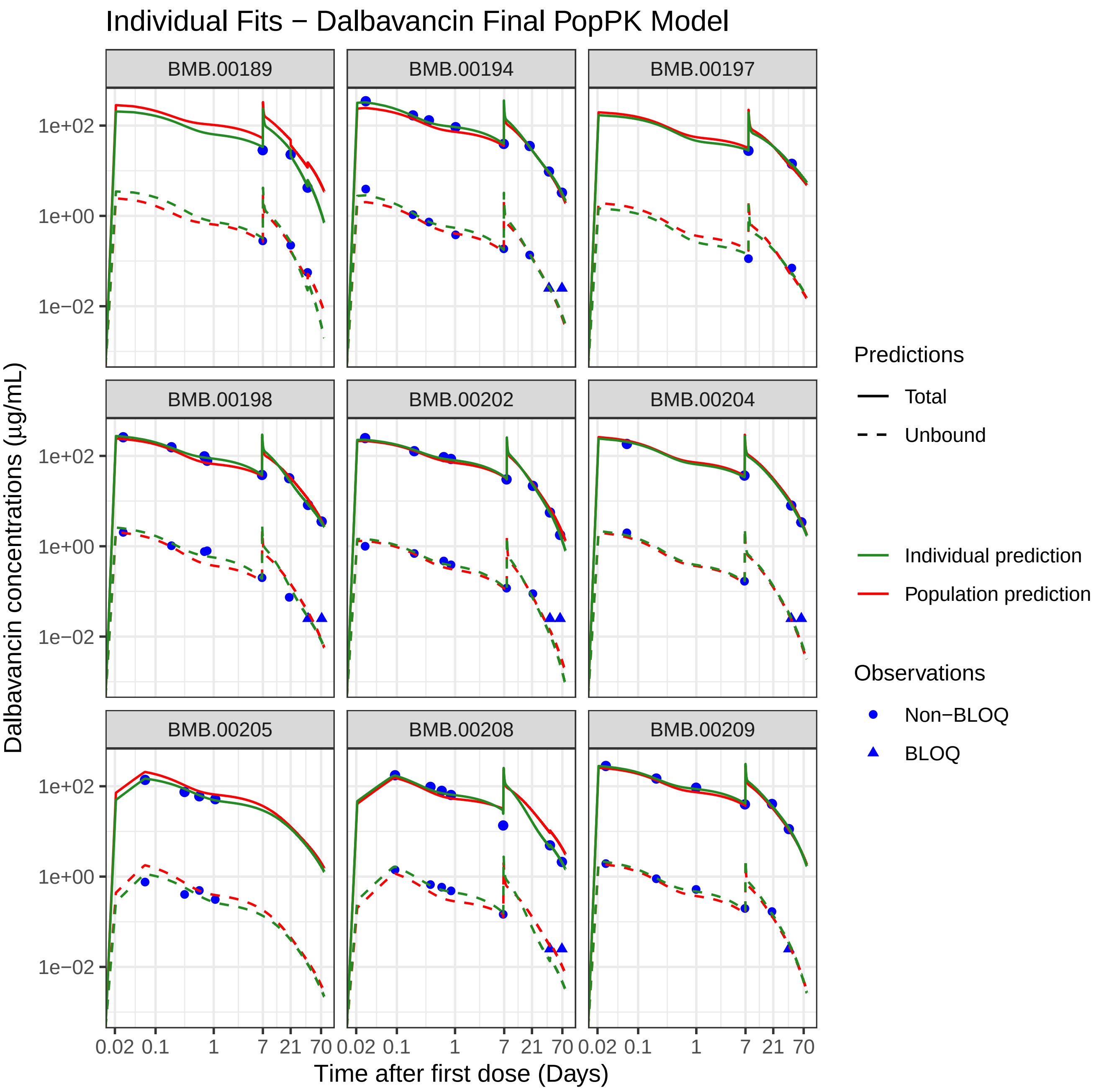
Individual Fits − Dalbavancin Final PopPK Model
BMB.00189
BMB.00194
BMB.00197
BMB.00198
BMB.00202
BMB.00204
BMB.00205
BMB.00208
BMB.00209
Dalbavancin concentrations (µg/mL)
1e+02
1e+00
1e−02
Time after first dose (Days)
0.02
0.1
1
7
21
70
Predictions
Total
Unbound
Individual prediction
Population prediction
Observations
Non−BLOQ
BLOQ

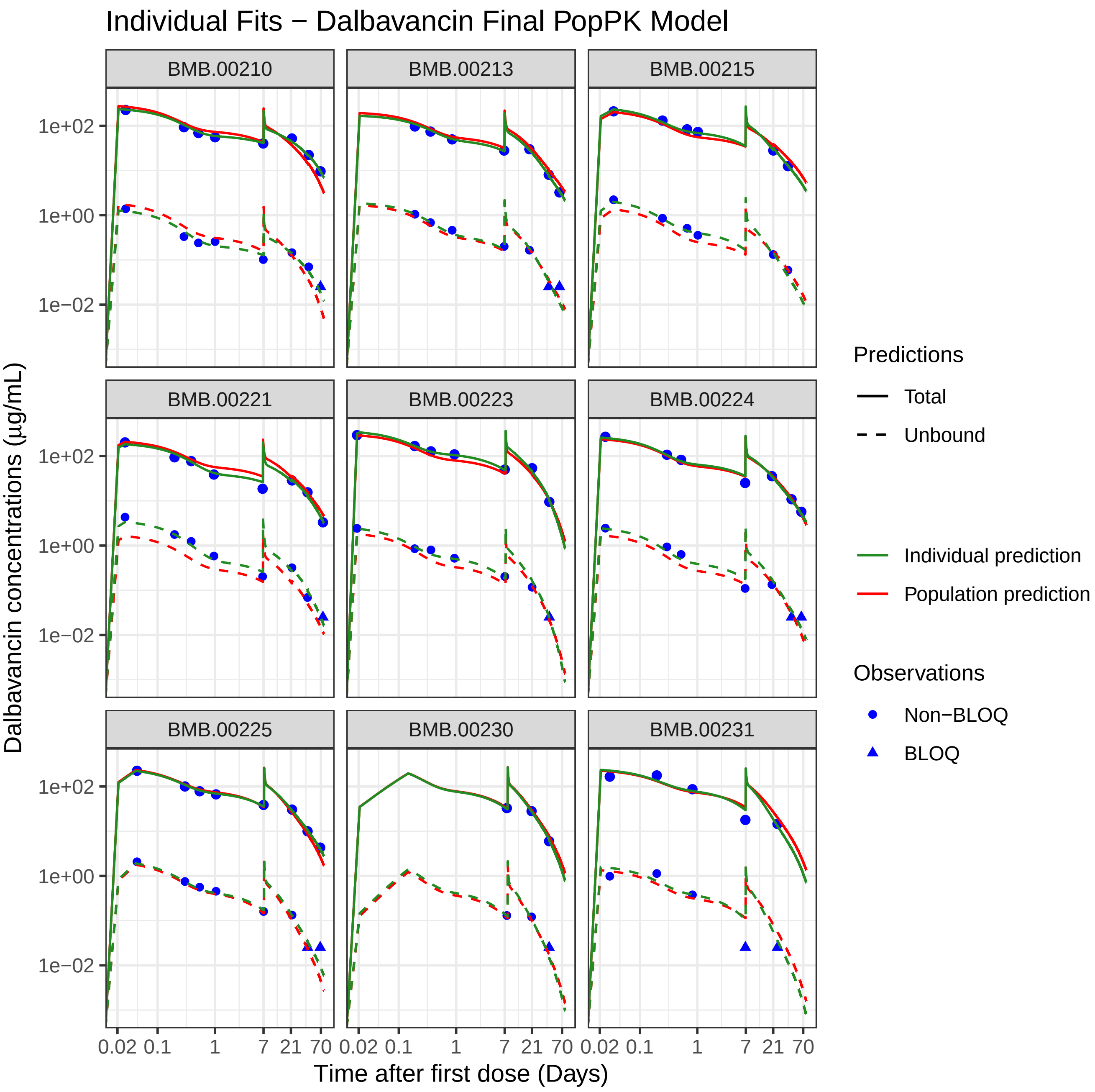
Individual Fits − Dalbavancin Final PopPK Model
BMB.00210
BMB.00213
BMB.00215
BMB.00221
BMB.00223
BMB.00224
BMB.00225
BMB.00230
BMB.00231
Dalbavancin concentrations (µg/mL)
1e+02
1e+00
1e−02
0.02 0.1 1 7 21 70
Time after first dose (Days)
Predictions
Total
Unbound
Individual prediction
Population prediction
Observations
Non−BLOQ
BLOQ

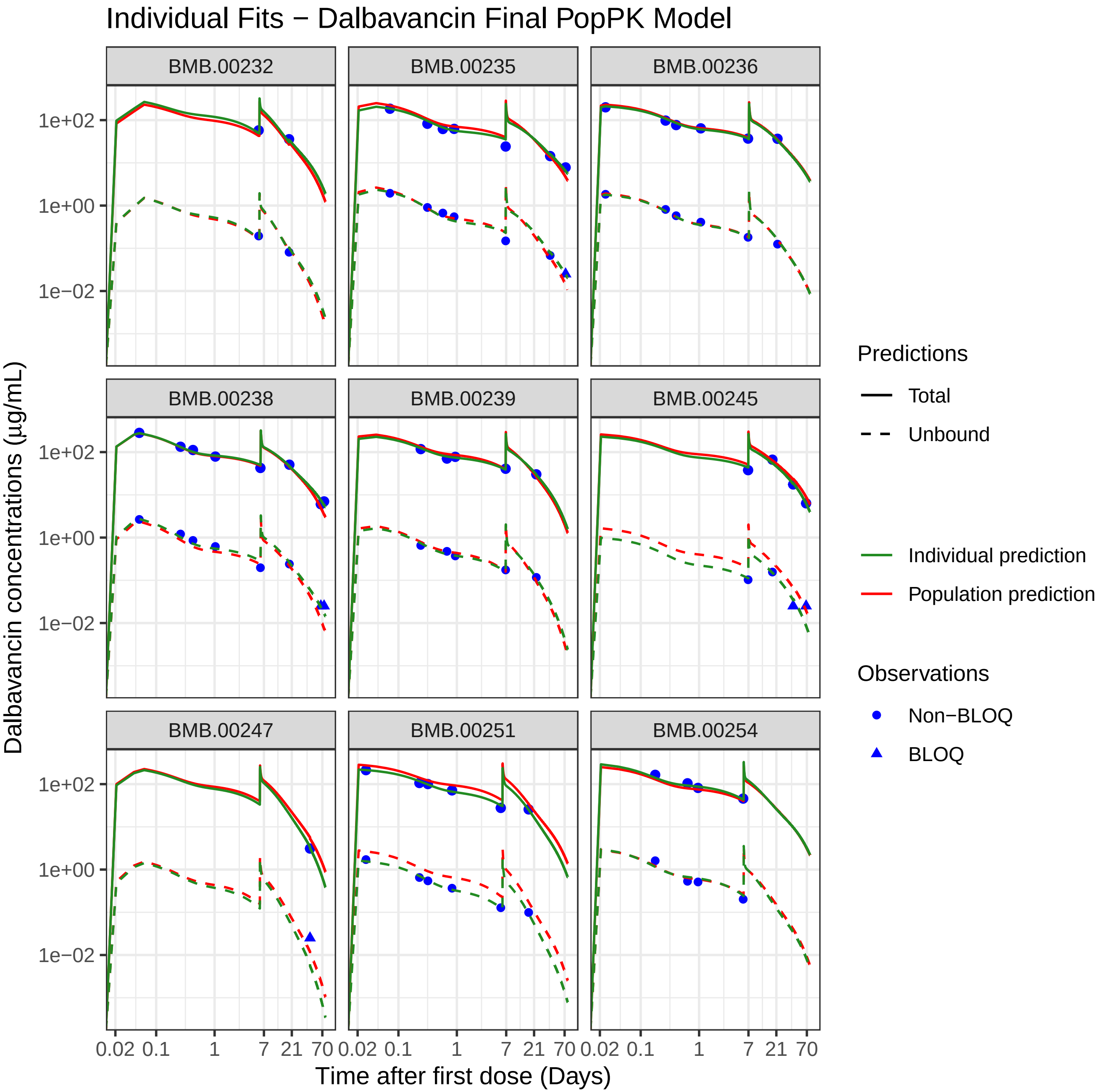
Individual Fits − Dalbavancin Final PopPK Model
BMB.00232
BMB.00235
BMB.00236
BMB.00238
BMB.00239
BMB.00245
BMB.00247
BMB.00251
BMB.00254
1e+02
1e+00
1e−02
0.02
0.1
1
7
21
70
Dalbavancin concentrations (µg/mL)
Time after first dose (Days)
Predictions
Total
Unbound
Individual prediction
Population prediction
Observations
Non−BLOQ
BLOQ

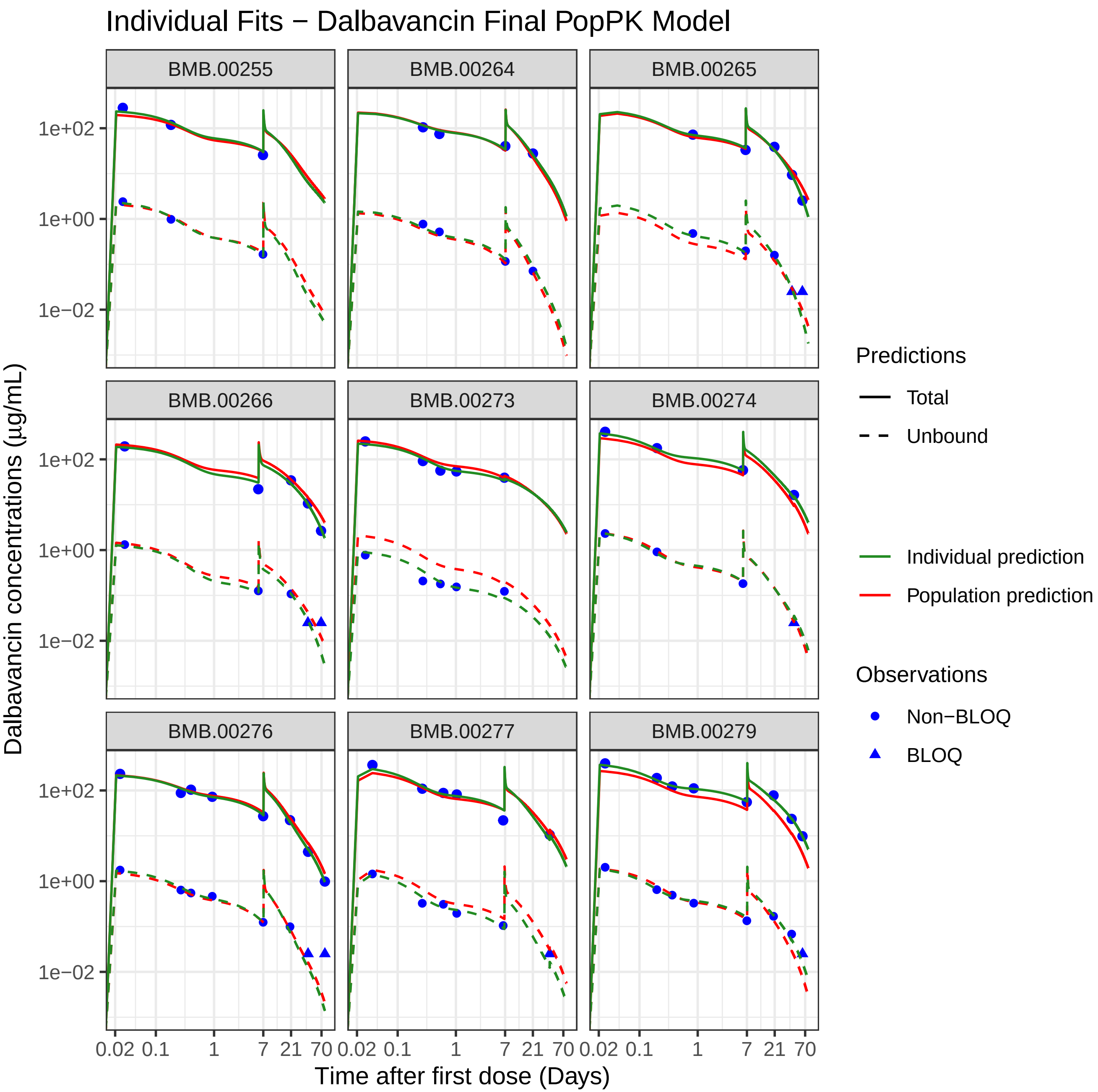
Individual Fits − Dalbavancin Final PopPK Model
BMB.00255
BMB.00264
BMB.00265
BMB.00266
BMB.00273
BMB.00274
BMB.00276
BMB.00277
BMB.00279
1e+02
1e+00
1e−02
0.02
0.1
1
7
21
70
Dalbavancin concentrations (µg/mL)
Time after first dose (Days)
Predictions
Total
Unbound
Individual prediction
Population prediction
Observations
Non−BLOQ
BLOQ

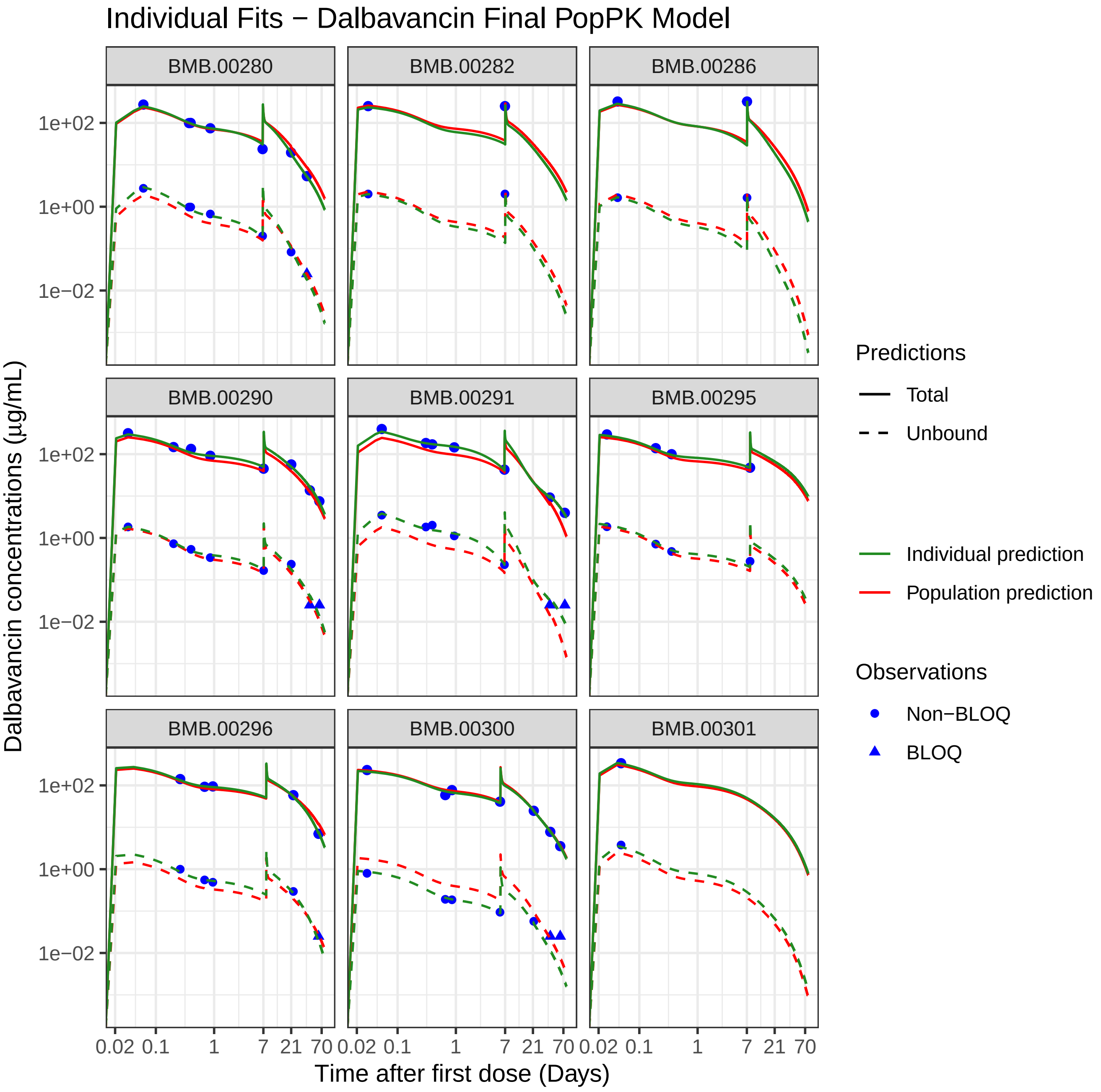
Individual Fits − Dalbavancin Final PopPK Model
BMB.00280
BMB.00282
BMB.00286
BMB.00290
BMB.00291
BMB.00295
BMB.00296
BMB.00300
BMB.00301
1e+02
1e+00
1e−02
0.02
0.1
1
7
21
70
Dalbavancin concentrations (µg/mL)
Time after first dose (Days)
Predictions
Total
Unbound
Individual prediction
Population prediction
Observations
Non−BLOQ
BLOQ

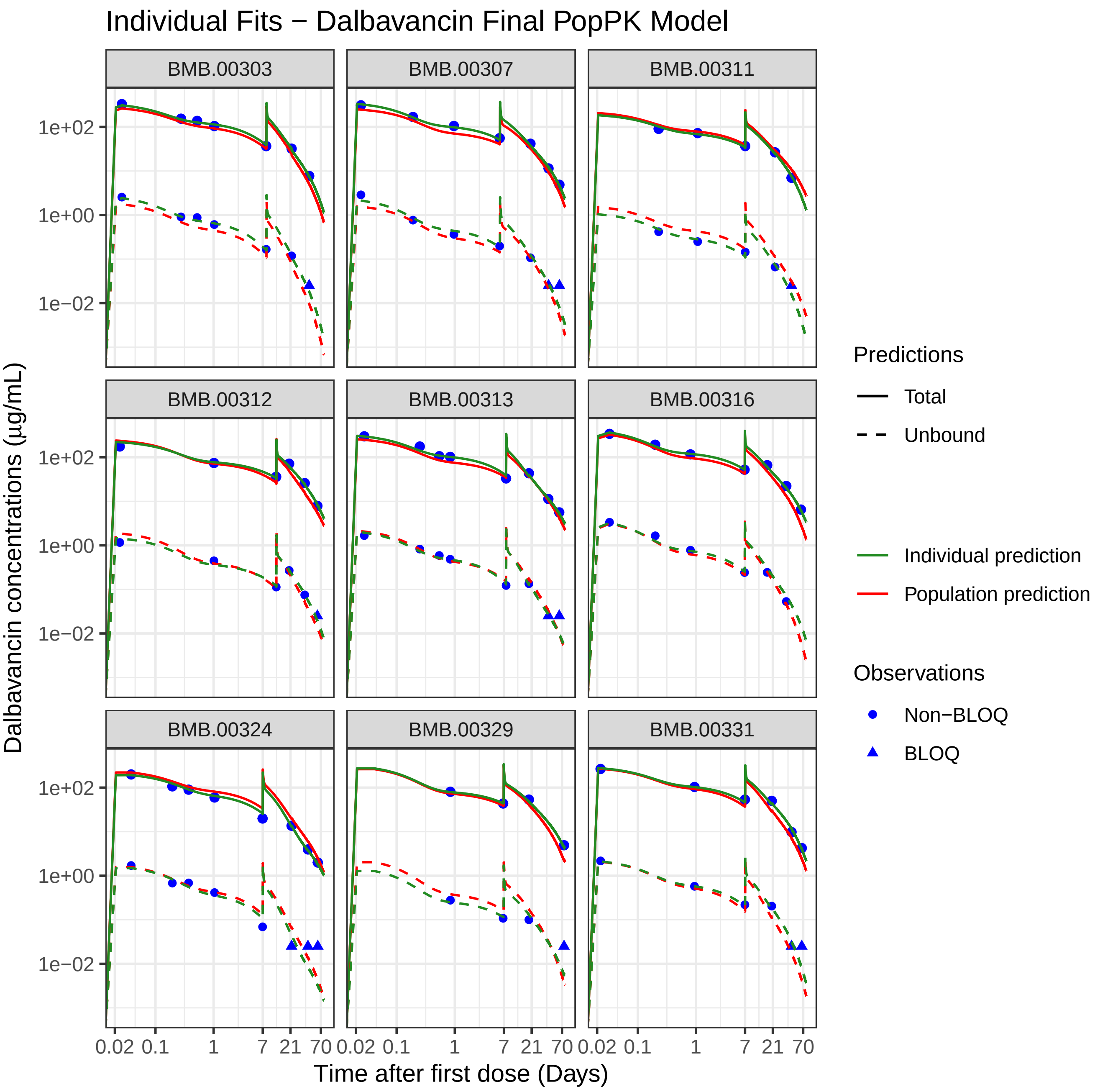
Individual Fits − Dalbavancin Final PopPK Model
BMB.00303
BMB.00307
BMB.00311
BMB.00312
BMB.00313
BMB.00316
BMB.00324
BMB.00329
BMB.00331
Dalbavancin concentrations (µg/mL)
1e+02
1e+00
1e−02
Time after first dose (Days)
0.02
0.1
1
7
21
70
Predictions
Total
Unbound
Individual prediction
Population prediction
Observations
Non−BLOQ
BLOQ

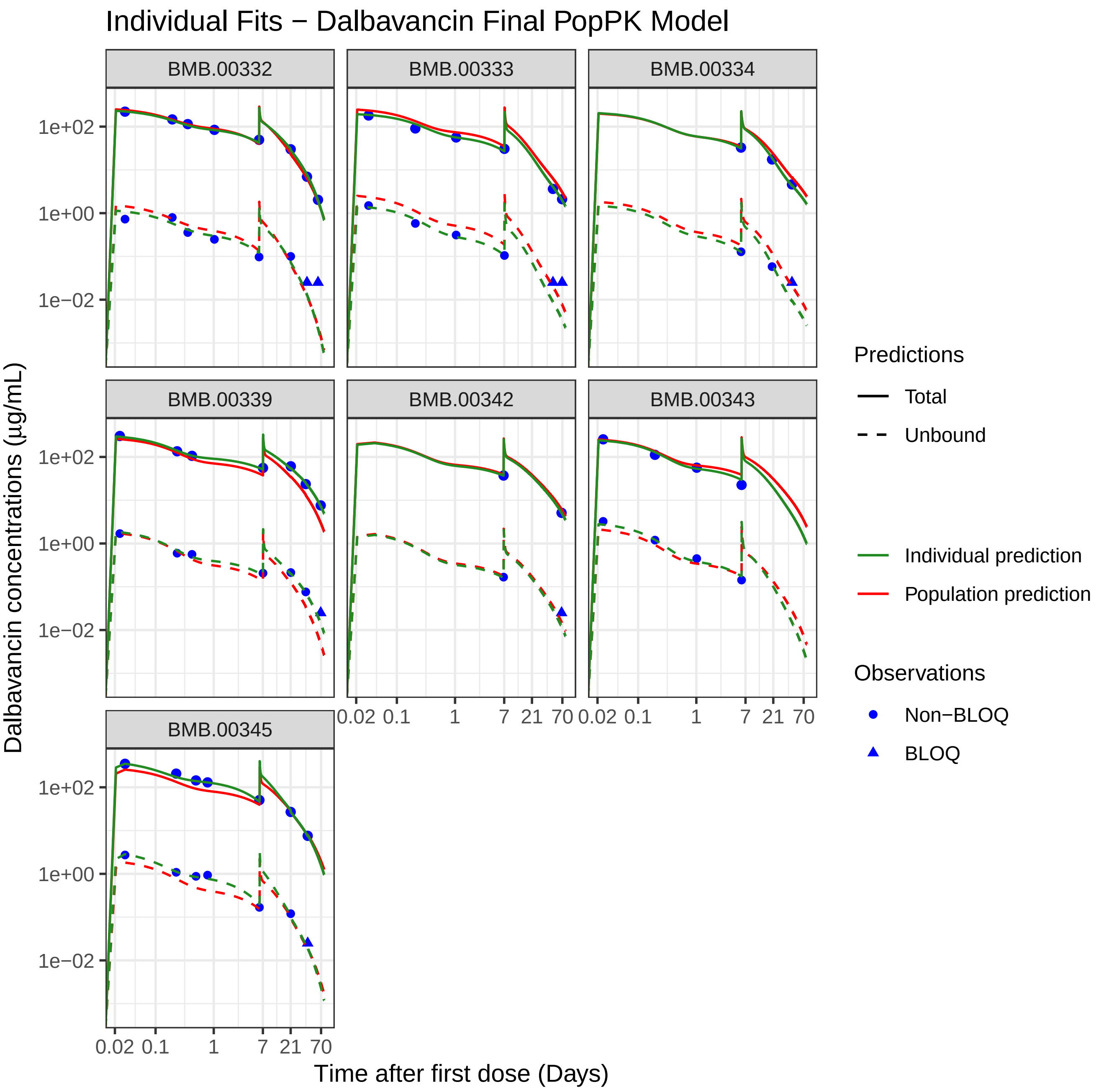
Individual Fits − Dalbavancin Final PopPK Model
BMB.00332
BMB.00333
BMB.00334
BMB.00339
BMB.00342
BMB.00343
BMB.00345
1e+02
1e+00
1e−02
0.02 0.1 1 7 21 70
Dalbavancin concentrations (µg/mL)
Time after first dose (Days)
Predictions
Total
Unbound
Individual prediction
Population prediction
Observations
Non−BLOQ
BLOQ

Task05: Dalbavancin Final PopPK Model - Results and Diagnostic plots

Final

Melanie Wilbaux Jessica Wojciechowski Daniel Selig

07-Jun-2024

Contents

1 BACKGROUND 3
1.1 Purpose . . . 3
1.2 Executive Summary . . . 3

2 INITIALIZE ANALYSIS 5
2.1 Initialize R Session . . . 5
2.2 Load packages . . . 5
2.3 Set plotting theme . . . 6
2.4 Define functions . . . 6

3 BACKWARD ELIMINATION 6

4 FINAL MODEL 12

5 IMPORT FINAL MODEL RESULTS 12

6 PARAMETER ESTIMATES 13

7 DIAGNOSTIC PLOTS 15
7.1 OBS vs. PRED . . . 15
7.1.1 Linear scale . . . 15
7.1.2 Log scale . . . 16
7.2 DV vs. IPRED . . . 17
7.2.1 Linear scale . . . 17
7.2.2 Log scale . . . 18

7.3 RES vs. Time after first dose . . . 19
7.3.1 CWRES vs. Time . . . 19
7.3.2 CWRES vs. Time (log scale) . . . 20
7.3.3 IWRES vs. Time . . . 21
7.3.4 IWRES vs. Time (log scale) . . . 22
7.4 RES vs. Time from previous dose . . . 23
7.4.1 CWRES vs. Time . . . 23
7.4.2 CWRES vs. Time (log scale) . . . 24
7.4.3 IWRES vs. Time . . . 25
7.4.4 iWRES vs. Time (log scale) . . . 26
7.5 RES vs. PRED . . . 27
7.5.1 CWRES vs. PRED . . . 27
7.5.2 IWRES vs. IPRED . . . 28
7.5.3 |IWRES| vs IPRED . . . 29
7.6 RES Distribution . . . 30
7.6.1 CWRES hist . . . 30
7.6.2 CWRES qqplot . . . 31
7.6.3 IWRES hist . . . 32
7.6.4 IWRES qqplot . . . 33
7.7 NPDE Distribution . . . 34
7.7.1 NPDE hist . . . 34
7.7.2 NPDE qqplot . . . 35
7.8 BSV Distribution . . . 36
7.8.1 Hist . . . 36
7.8.2 QQplots . . . 37
7.9 ETAs Correlations . . . 38
7.10 BSV vs. Cov . . . 39
7.10.1 Continuous covariates . . . 40
7.10.2 Continuous covariates (Log scale) . . . 40
7.10.3 Categorical covariates . . . 42

8 INDIVIDUAL FITS 42

9 INDIVIDUAL PARAMETERS VS COVARIATES 43
9.1 CL ~ CrCl . . . 43
9.2 V1 ~ WTB . . . 44
9.3 V2 ~ WTB . . . 44
9.4 V2 ~ ALBB . . . 45
9.5 V3 ~ AGE . . . 46
9.6 V3 ~ WTB . . . 47
9.7 A ~ ALBB . . . 48

10 COVARIATE-EFFECT PLOT **49**
10.1 CL 50
10.2 V1 51
10.3 V2 52
10.4 V3 53
10.5 A 54

11 VPC **54**
11.1 VPC Setup 55
11.2 pcVPC - Time After First Dose 55
11.2.1 Linear scale 55
11.2.2 Conc. in Log scale 56
11.2.3 Time in Log scale 58
11.2.4 Conc. and Time in Log scale 60
11.2.5 %BLOQ unbound 62
11.3 pcVPC - Time From Previous Dose 63
11.3.1 Linear scale 63
11.3.2 Conc. in Log scale 65
11.3.3 Time in Log scale 67
11.3.4 Conc. and Time in Log scale 69
11.3.5 %BLOQ unbound 71

12 REPRODUCIBILITY **72**

1 BACKGROUND

1.1 Purpose

The purpose of this script is to summarize the results from the dalbavancin final popPK model and to generate diagnostic plots.

1.2 Executive Summary

The final popPK model was developed by backward elimination of covariate-parameter relationships included in the full popPK model.

Backward Elimination of Covariate-Parameter Relationships

Univariate stepwise backward elimination was conducted on the full popPK model. A covariate-parameter relationship was considered statistically significant if its removal from the model resulted in an LRT p-value<0.001. In each step of backward elimination, the least significant covariate (with the highest p-value 0.001) was systematically removed from

the model until all remaining covariates in the model demonstrated statistical significance (p-value<0.001).

The results of the backward elimination process, including LRT criteria and changes from the final model, were presented in Section 3 (*Table 1 from ./results/05-final-model/*). The analysis resulted in the exclusion of the following covariates: renal replacement therapy effect on CL, V1 and V2; the effect of sex on A; and the effect of baseline body weight on CL and A.

Final PopPK Model

To summarize, the final dalbavancin popPK model comprises a three-compartment model, with zero-order input and first-order elimination. unbound concentrations were assumed to be dependent on total concentrations through a power relationship. The popPK model was coded with parameters CL (clearance), V1 (volume of distribution in the central compartment), Q2 (intercompartmental clearance from the first to the second compartment), V2 (volume of distribution in the second compartment), Q3 (intercompartmental clearance from the first to the third compartment), V3 (volume of distribution in the third compartment), A (scaling factor) and K (exponent of the power). The RUV was modeled using a proportional error model for total concentrations and a combined error model for unbound concentrations. The BSV included log-normally distributed random effects on CL, V1, V2, V3 and A. The BSV on parameters, CL, V1 and V3 was modeled using a three-dimensional matrix taking into account correlations among the parameters.

The final popPK model retained the following covariate effects:

- CrCL on CL
- baseline weight on V1, V2 and V3
- baseline albumin on V2 and A
- age on V3.

Parameter estimates from the final popPK model were presented in Section 6 (*Table 2 from ./results/05-final-model/*). All parameters from the final popPK model were well estimated, with acceptable RSE. Population clearance and central volume of distribution were estimated at 0.0658 L/hr and 5.67 L, respectively, with moderate variability (CV% = 22.5% and 19.6%, respectively). Q2 and Q3 were estimated at 0.0259 L/hr and 0.921 L/hr, respectively. V2 and V3 were estimated at 8.91 L and 11.1 L, respectively, with moderate variability (CV% = 29.9% and 29.3%, respectively). A was estimated at 0.00136 μg/mL with a moderate variability (CV% = 32.6%) and K at 1.32, indicating a curvature in the relationship between unbound and total concentrations. All eta and epsilon shrinkage were acceptable. The condition number, calculated as the ratio between the largest and smallest eigenvalues of the correlation matrix, was found to be 60, indicating that the final popPK model was stable and not over-parameterized (as the value is < 1000).

All the diagnostic plots from Section 7 confirmed that the final popPK model described the total and unbound PK data adequately. The observed concentration vs. model predictions (population and individual) plots demonstrated a reasonable agreement between data and

model predictions. The residual plots do not show any strong pattern with either time or concentration, and closely resembles a normal distribution. A slight trend in the residuals of unbound concentrations at later time points was observed; but it did not raise concerns as it only pertained to a few points, and all other diagnostic plots were acceptable.

The prediction-corrected Visual Predictive Checks (pcVPC) were presented in Section 11 , and showed good agreement between observed and simulated total and unbound dalbavancin PK, as well as for the BLOQ data from unbound concentrations.

Covariate effects

The forest plots presented in Section 10 quantified the covariate effects as followed:

- Dalbavancin CL was 35.1% lower at the 5th percentile of baseline CrCl (13.5 mL/min/1.73m2) compared to the median (101.26 mL/min/1.73m2).
- Dalbavancin V1 was 21.8% lower at the 5th percentile of baseline body weight (54.5 kg) compared to the median (83.8 kg).
- Dalbavancin V2 was 29.7% lower at the 5th percentile of baseline body weight (54.5 kg) compared to the median (83.8 kg).
- Dalbavancin V2 was 49% higher at the 5th percentile of baseline albumin (1.7 g/dL) compared to the median (2.8 g/dL).
- Dalbavancin V3 was 21.4 lower at the 5th percentile of baseline body weight (54.5 kg) compared to the median (83.8 kg).
- Dalbavancin V3 was 33.9% lower at the 5th percentile of age (29 yr) compared to the median (56 yr).
- Dalbavancin A was 48% higher at the 5th percentile of baseline albumin (1.7 g/dL) compared to the median (2.8 g/dL).

Additionally, individual model predicted popPK parameters were plotted against significant covariates from the final popPK model in Section 9 .

2 INITIALIZE ANALYSIS

2.1 Initialize R Session

Initialize the global R environment and result directories.

```
[1] "/mnt/data/code/SSC/Emmes/Emmes-Dalbavancin-DOTS"
```

2.2 Load packages

```
[1] "/mnt/data/code/SSC/Emmes/Emmes-Dalbavancin-DOTS/renv/library/R-4.2/x86_64-pc-linux-gnu"
[2] "/mnt/data/.cache/R/renv/sandbox/R-4.2/x86_64-pc-linux-gnu/e11edd0e"
```

2.3 Set plotting theme

2.4 Define functions

3 BACKWARD ELIMINATION

Note

- Backward elimination was applied on the full popPK model in Pumas (/programs/pumas/final/final-model.jl).
- Covariates included in the full popPK model:
  - CL ~ CrCl + RRTFL + WTBL
  - V1 ~ WTBL
  - V2 ~ WTBL + ALBBL + RRTFL
  - V3 ~ Age + WTBL
  - A ~ ALBBL + SEX + WTBL

Important

After backward elimination, the following covariate-parameter relationships were kept:

- CL ~ CrCl
- V1 ~ WTBL
- V2 ~ WTBL + ALBBL
- V3 ~ Age + WTBL
- A ~ ALBBL

The table below summarizes the results from each model from backward elimination, ordered from the lowest LRT criteria to the highest.

Table 1: Results from backward elimination

Included covariate-parameter relationships	Excluded covariate-parameter relationships	LRT_crit	delta_LRT_from_Final
cl_crcl, v1_wtbl, v2_wtbl, v2_albbl, v3_age, v3_wtbl, a_albbl	cl_rrtfl, cl_wtbl, v2_rrtfl, a_sex, a_wtbl	3569.31	0.00
cl_crcl, v1_wtbl, v2_albbl, v3_age, v3_wtbl, a_albbl	cl_rrtfl, cl_wtbl, v2_wtbl, v2_rrtfl, a_sex, a_wtbl	3570.69	1.39

Included covariate-parameter relationships	Excluded covariate-parameter relationships	LRT_crit	delta_LRT_from_Final
cl_crcl, v1_wtbl, v2_wtbl, v3_age, v3_wtbl, a_albbl	cl_rrtfl, cl_wtbl, v2_albbl, v2_rrtfl, a_sex, a_wtbl	3572.20	2.89
cl_crcl, cl_rrtfl, v1_wtbl, v2_wtbl, v2_albbl, v3_age, v3_wtbl, a_albbl	cl_wtbl, v2_rrtfl, a_sex, a_wtbl	3572.93	3.62
cl_crcl, cl_wtbl, v1_wtbl, v2_wtbl, v2_albbl, v3_age, v3_wtbl, a_albbl	cl_rrtfl, v2_rrtfl, a_sex, a_wtbl	3573.22	3.91
cl_crcl, cl_rrtfl, v1_wtbl, v2_albbl, v3_age, v3_wtbl, a_albbl	cl_wtbl, v2_wtbl, v2_rrtfl, a_sex, a_wtbl	3574.57	5.26
cl_crcl, v1_wtbl, v2_wtbl, v2_albbl, v3_age, a_albbl	cl_rrtfl, cl_wtbl, v2_rrtfl, v3_wtbl, a_sex, a_wtbl	3574.77	5.47
cl_crcl, cl_rrtfl, cl_wtbl, v1_wtbl, v2_albbl, v3_age, v3_wtbl, a_albbl	v2_wtbl, v2_rrtfl, a_sex, a_wtbl	3574.92	5.61
cl_crcl, cl_rrtfl, cl_wtbl, v1_wtbl, v2_wtbl, v2_albbl, v3_age, v3_wtbl, a_albbl	v2_rrtfl, a_sex, a_wtbl	3575.17	5.86
cl_crcl, cl_rrtfl, v1_wtbl, v2_wtbl, v3_age, v3_wtbl, a_albbl	cl_wtbl, v2_albbl, v2_rrtfl, a_sex, a_wtbl	3575.47	6.16
cl_crcl, cl_wtbl, v1_wtbl, v2_wtbl, v2_albbl, v3_age, v3_wtbl, a_albbl, a_wtbl	cl_rrtfl, v2_rrtfl, a_sex	3575.60	6.29
cl_crcl, cl_rrtfl, cl_wtbl, v1_wtbl, v2_wtbl, v3_age, v3_wtbl, a_albbl	v2_albbl, v2_rrtfl, a_sex, a_wtbl	3576.58	7.27
cl_crcl, cl_rrtfl, v1_wtbl, v2_wtbl, v2_albbl, v3_age, v3_wtbl, a_albbl, a_wtbl	cl_wtbl, v2_rrtfl, a_sex	3576.75	7.44
cl_crcl, cl_rrtfl, cl_wtbl, v1_wtbl, v2_wtbl, v2_albbl, v3_age, v3_wtbl, a_albbl, a_wtbl	v2_rrtfl, a_sex	3577.15	7.84

Included covariate-parameter relationships	Excluded covariate-parameter relationships	LRT__crit	delta__LRT__from__Final
cl__crcl, cl__rrtfl, cl__wtbl, v1__wtbl, v2__albbl, v3__age, v3__wtbl, a__albbl, a__wtbl	v2__wtbl, v2__rrtfl, a__sex	3578.06	8.75
cl__crcl, cl__wtbl, v1__wtbl, v2__wtbl, v2__albbl, v2__rrtfl, v3__age, v3__wtbl, a__albbl, a__wtbl	cl__rrtfl, a__sex	3578.07	8.76
cl__crcl, cl__rrtfl, cl__wtbl, v1__wtbl, v2__wtbl, v3__age, v3__wtbl, a__albbl, a__wtbl	v2__albbl, v2__rrtfl, a__sex	3578.09	8.78
cl__crcl, cl__rrtfl, v1__wtbl, v2__wtbl, v2__albbl, v3__age, a__albbl	cl__wtbl, v2__rrtfl, v3__wtbl, a__sex, a__wtbl	3578.16	8.85
cl__crcl, cl__rrtfl, cl__wtbl, v1__wtbl, v2__albbl, v2__rrtfl, v3__age, v3__wtbl, a__albbl, a__wtbl	v2__wtbl, a__sex	3578.91	9.61
cl__crcl, cl__rrtfl, cl__wtbl, v1__wtbl, v2__wtbl, v2__albbl, v2__rrtfl, v3__age, v3__wtbl, a__albbl	a__sex, a__wtbl	3580.98	11.67
cl__crcl, cl__rrtfl, v1__wtbl, v2__wtbl, v2__albbl, v2__rrtfl, v3__age, v3__wtbl, a__albbl, a__wtbl	cl__wtbl, a__sex	3582.22	12.91
cl__crcl, cl__rrtfl, cl__wtbl, v1__wtbl, v2__wtbl, v2__albbl, v2__rrtfl, v3__age, v3__wtbl, a__albbl, a__wtbl	a__sex,	3583.86	14.56
cl__crcl, v1__wtbl, v2__wtbl, v2__albbl, v3__age, v3__wtbl	cl__rrtfl, cl__wtbl, v2__rrtfl, a__albbl, a__sex, a__wtbl	3583.97	14.66
cl__crcl, cl__rrtfl, cl__wtbl, v1__wtbl, v2__wtbl, v2__albbl, v3__age, v3__wtbl, a__albbl, a__sex, a__wtbl	v2__rrtfl,	3584.44	15.13

Included covariate-parameter relationships	Excluded covariate-parameter relationships	LRT_crit	delta_LRT_from_Final
cl_crcl, cl_rrtfl, cl_wtbl, v1_wtbl, v2_wtbl, v2_rrtfl, v3_age, v3_wtbl, a_albbl, a_wtbl	v2_albbl, a_sex	3584.58	15.28
cl_crcl, cl_rrtfl, v1_wtbl, v2_wtbl, v2_albbl, v3_age, v3_wtbl	cl_wtbl, v2_rrtfl, a_albbl, a_sex, a_wtbl	3584.77	15.46
cl_crcl, cl_wtbl, v1_wtbl, v2_wtbl, v2_albbl, v2_rrtfl, v3_age, v3_wtbl, a_albbl, a_sex, a_wtbl	cl_rrtfl,	3585.81	16.51
cl_crcl, cl_rrtfl, cl_wtbl, v1_wtbl, v2_albbl, v2_rrtfl, v3_age, v3_wtbl, a_albbl, a_sex, a_wtbl	v2_wtbl,	3586.26	16.96
cl_crcl, cl_rrtfl, cl_wtbl, v1_wtbl, v2_wtbl, v2_albbl, v3_age, a_albbl	v2_rrtfl, v3_wtbl, a_sex, a_wtbl	3587.17	17.86
cl_crcl, cl_rrtfl, cl_wtbl, v1_wtbl, v2_wtbl, v2_albbl, v3_age, v3_wtbl	v2_rrtfl, a_albbl, a_sex, a_wtbl	3589.54	20.24
cl_crcl, cl_rrtfl, v1_wtbl, v2_wtbl, v2_albbl, v2_rrtfl, v3_age, v3_wtbl, a_albbl, a_sex, a_wtbl	cl_wtbl,	3589.95	20.65
cl_crcl, v2_wtbl, v2_albbl, v3_age, v3_wtbl, a_albbl	cl_rrtfl, cl_wtbl, v1_wtbl, v2_rrtfl, a_sex, a_wtbl	3589.96	20.65
cl_crcl, cl_rrtfl, cl_wtbl, v1_wtbl, v2_wtbl, v2_albbl, v2_rrtfl, v3_age, v3_wtbl, a_albbl, a_sex	a_wtbl,	3589.99	20.68
cl_crcl, cl_rrtfl, cl_wtbl, v1_wtbl, v2_wtbl, v2_albbl, v3_age, v3_wtbl, a_wtbl	v2_rrtfl, a_albbl, a_sex	3590.80	21.50
cl_crcl, cl_rrtfl, cl_wtbl, v1_wtbl, v2_wtbl, v2_albbl, v2_rrtfl, v3_age, v3_wtbl, a_albbl, a_sex, a_wtbl		3590.97	21.66

Included covariate-parameter relationships	Excluded covariate-parameter relationships	LRT__crit	delta__LRT__from__Final
cl__crcl, cl__rrtfl, cl__wtbl, v1__wtbl, v2__wtbl, v2__rrtfl, v3__age, v3__wtbl, a__albbl, a__sex, a__wtbl	v2__albbl,	3592.19	22.89
cl__crcl, cl__rrtfl, cl__wtbl, v1__wtbl, v2__wtbl, v2__albbl, v3__age, a__albbl, a__wtbl	v2__rrtfl, v3__wtbl, a__sex	3592.21	22.91
cl__crcl, cl__rrtfl, v2__wtbl, v2__albbl, v3__age, v3__wtbl, a__albbl	cl__wtbl, v1__wtbl, v2__rrtfl, a__sex, a__wtbl	3593.53	24.23
cl__crcl, cl__rrtfl, cl__wtbl, v1__wtbl, v2__wtbl, v2__albbl, v2__rrtfl, v3__age, a__albbl, a__wtbl	v3__wtbl, a__sex	3596.39	27.08
cl__crcl, cl__rrtfl, cl__wtbl, v1__wtbl, v2__wtbl, v2__albbl, v2__rrtfl, v3__age, v3__wtbl, a__wtbl	a__albbl, a__sex	3598.39	29.09
cl__crcl, cl__rrtfl, v1__wtbl, v2__wtbl, v2__albbl, v3__wtbl, a__albbl	cl__wtbl, v2__rrtfl, v3__age, a__sex, a__wtbl	3598.60	29.30
cl__crcl, v1__wtbl, v2__wtbl, v2__albbl, v3__wtbl, a__albbl	cl__rrtfl, cl__wtbl, v2__rrtfl, v3__age, a__sex, a__wtbl	3600.84	31.54
cl__crcl, cl__rrtfl, cl__wtbl, v1__wtbl, v2__wtbl, v2__albbl, v2__rrtfl, v3__age, v3__wtbl, a__sex, a__wtbl	a__albbl,	3603.24	33.93
cl__crcl, cl__rrtfl, cl__wtbl, v1__wtbl, v2__wtbl, v2__albbl, v3__wtbl, a__albbl	v2__rrtfl, v3__age, a__sex, a__wtbl	3603.49	34.18
cl__crcl, cl__rrtfl, cl__wtbl, v1__wtbl, v2__wtbl, v2__albbl, v3__wtbl, a__albbl, a__wtbl	v2__rrtfl, v3__age, a__sex	3603.86	34.55
cl__crcl, cl__rrtfl, cl__wtbl, v1__wtbl, v2__wtbl, v2__albbl, v2__rrtfl, v3__age, a__albbl, a__sex, a__wtbl	v3__wtbl,	3604.13	34.82

Included covariate-parameter relationships	Excluded covariate-parameter relationships	LRT_crit	delta_LRT_from_Final
cl_crcl, cl_rrtfl, cl_wtbl, v2_wtbl, v2_albbl, v3_age, v3_wtbl, a_albbl	v1_wtbl, v2_rrtfl, a_sex, a_wtbl	3606.31	37.00
cl_rrtfl, cl_wtbl, v1_wtbl, v2_wtbl, v2_albbl, v3_age, v3_wtbl, a_albbl	cl_crcl, v2_rrtfl, a_sex, a_wtbl	3610.23	40.92
cl_crcl, cl_rrtfl, cl_wtbl, v1_wtbl, v2_wtbl, v2_albbl, v2_rrtfl, v3_wtbl, a_albbl, a_wtbl	v3_age, a_sex	3610.28	40.97
cl_crcl, cl_rrtfl, cl_wtbl, v2_wtbl, v2_albbl, v3_age, v3_wtbl, a_albbl, a_wtbl	v1_wtbl, v2_rrtfl, a_sex	3610.92	41.62
cl_rrtfl, cl_wtbl, v1_wtbl, v2_wtbl, v2_albbl, v3_age, v3_wtbl, a_albbl, a_wtbl	cl_crcl, v2_rrtfl, a_sex	3613.78	44.48
cl_crcl, cl_rrtfl, cl_wtbl, v2_wtbl, v2_albbl, v2_rrtfl, v3_age, v3_wtbl, a_albbl, a_wtbl	v1_wtbl, a_sex	3615.56	46.26
cl_crcl, cl_rrtfl, cl_wtbl, v1_wtbl, v2_wtbl, v2_albbl, v2_rrtfl, v3_wtbl, a_albbl, a_sex, a_wtbl	v3_age,	3617.68	48.38
cl_rrtfl, v1_wtbl, v2_wtbl, v2_albbl, v3_age, v3_wtbl, a_albbl	cl_crcl, cl_wtbl, v2_rrtfl, a_sex, a_wtbl	3619.01	49.71
cl_rrtfl, cl_wtbl, v1_wtbl, v2_wtbl, v2_albbl, v2_rrtfl, v3_age, v3_wtbl, a_albbl, a_wtbl	cl_crcl, a_sex	3620.02	50.71
v1_wtbl, v2_wtbl, v2_albbl, v3_age, v3_wtbl, a_albbl	cl_crcl, cl_rrtfl, cl_wtbl, v2_rrtfl, a_sex, a_wtbl	3621.49	52.19
cl_crcl, cl_rrtfl, cl_wtbl, v2_wtbl, v2_albbl, v2_rrtfl, v3_age, v3_wtbl, a_albbl, a_sex, a_wtbl	v1_wtbl,	3624.50	55.19

Included covariate-parameter relationships	Excluded covariate-parameter relationships	LRT_crit	delta_LRT_from_Final
cl_rrtfl, cl_wtbl, v1_wtbl, v2_wtbl, v2_albbl, v2_rrtfl, v3_age, v3_wtbl, a_albbl, a_sex, a_wtbl	cl_crcl,	3627.20	57.89

4 FINAL MODEL

❗ Important

- Final model:
  - 3-compartment model, zero-order input and first-order elimination;
  - Unbound concentrations assumed to be dependent on total concentrations with a power relationship;
  - RUV modeled using a proportional error model for total concentrations and a combined error model for unbound concentrations;
  - BSV on CL, V1, V2, V3 and A;
  - Correlations between CL, V1 and V3.
- Covariates included in the final popPK model:
  - CL ~ CrCl + RRTFL + WTBL
  - V1 ~ WTBL
  - V2 ~ WTBL + ALBBL + RRTFL
  - V3 ~ Age + WTBL
  - A ~ ALBBL + SEX + WTBL

5 IMPORT FINAL MODEL RESULTS

ℹ Note

The dalbavancin final popPK model was executed in the Pumas script: ./programs/pumas/final/final-model.jl, to generate the output csv files.

6 PARAMETER ESTIMATES

Table 2: Parameter estimates - Final Model

Parameter	Description	Estim	RSE (%)	95% CI	CV (%)	Corr (%)	Shrinkage (%)
CL	Clearance (L/hr)	0.0658	2.6	[0.0624 ; 0.0692]	-	-	-
V1	Volume of distribution in the central compartment (L)	5.67	2.7	[5.37 ; 5.99]	-	-	-
Q2	Intercompartmental clearance from the 1st to the 2nd compartment (L/hr)	0.0259	7.9	[0.0224 ; 0.0302]	-	-	-
V2	Volume of distribution of 2nd compartment (L)	8.91	6.2	[8.04 ; 10.2]	-	-	-
Q3	Intercompartmental clearance from the 1st to the 3rd compartment (L/hr)	0.921	5.3	[0.83 ; 1.01]	-	-	-
V3	Volume of distribution of 3rd compartment (L)	11.1	3.8	[10.3 ; 12]	-	-	-
A	Scaling factor (ug/mL)	0.00136	8.1	[0.00115 ; 0.00159]	-	-	-
K	Exponent of the power function	1.32	1.4	[1.28 ; 1.35]	-	-	-
CL~CRCL	CRCL effect on CL	0.214	15.8	[0.16 ; 0.295]	-	-	-
V1~WTBL	WTBL effect on V1	0.57	21.1	[0.37 ; 0.855]	-	-	-
V2~WTBL	WTBL effect on V2	0.82	32.2	[0.374 ; 1.46]	-	-	-
V2~ALBBL	ALBBL effect on V2	-0.806	47.2	[-1.79 ; -0.318]	-	-	-
V3~AGE	AGE effect on V3	0.628	16.1	[0.44 ; 0.832]	-	-	-
V3~WTBL	WTBL effect on V3	0.559	24.5	[0.295 ; 0.816]	-	-	-
A~ALBBL	ALBBL effect on A	-0.782	14.3	[-0.984 ; -0.539]	-	-	-
Ω_CLV1V3 ,	BSV CL	0.0508	14.9	[0.0359 ; 0.0653]	22.5	-	4.2

Parameter	Description	Estim	RSE (%)	95% CI	CV (%)	Corr (%)	Shrinkage (%)
Ω_CLV1V3 ,	BSV V1	0.0386	29.5	[0.0191 ; 0.0623]	19.6	-	10.4
Ω ,	BSV V2	0.0897	27.6	[0.0239 ; 0.123]	29.9	-	39.4
Ω_CLV1V3 ,	BSV V3	0.0861	16.6	[0.0566 ; 0.113]	29.3	-	8.6
Ω ,	BSV A	0.106	21.5	[0.0616 ; 0.153]	32.6	-	6.8
Ω_CLV1V3 ,	Corr CL-V1	0.0264	29.6	[0.01 ; 0.0409]	-	59.7	-
Ω_CLV1V3 ,	Corr CL-V3	0.0416	22	[0.0238 ; 0.0578]	-	63	-
Ω_CLV1V3 ,	Corr V1-V3	0.0478	18.2	[0.0307 ; 0.0643]	-	82.9	-
_proptot	Proportional RUV for Total	0.127	6.1	[0.112 ; 0.142]	-	-	-
_propfree	Proportional RUV for Unbound	0.198	6.2	[0.173 ; 0.219]	-	-	-
_addfree	Additive RUV for Unbound	0.0124	21.9	[0.00521 ; 0.016]	-	-	-
Epsilon shrinkage Total	-	-	-	-	-	-	18
Epsilon shrinkage unbound	-	-	-	-	-	-	8.4

Abbreviations: ALBBL = baseline albumin ; BSV = between-subject variability; CI = confidence interval; CRCL = creatinine clearance; CV = coefficient of variation; RSE = relative standard error; RUV = residual unexplained variability; WTBL = baseline weight.

Notes:

- The condition number is equal to 60.
- Estimates for BSV are the variances.
- Estimates for RUV are the standard deviation.
- The CV % was derived as: sqrt(var)*100.
- RSE % was derived as: se/estimate*100.
- CI were generated by bootstrapping the original dataset (1000 replicates).

- A typical subject is a patient aged 56 years old, weighing 83.8 kg, with a baseline albumin value of 2.8 g/dL and a creatinine clearance value of 101.26 mL/min/1.73m2. These reference values represent the median values observed within the analysis population.

7 DIAGNOSTIC PLOTS

Note

- The diagnostic plots are not stratified by dose group, as the number of patients are too limited in the groups different from Dalbavancin 1500 mg + 1500 mg.
- BLOQ values for unbound concentrations (<0.05 μg/mL) are removed for all the diagnostic plots.

7.1 OBS vs. PRED

7.1.1 Linear scale

Observed vs. Population predicted dalbavancin concentrations - Final Model

Note: Solid black lines represent the lines of identity, and red lines the linear regression lines.

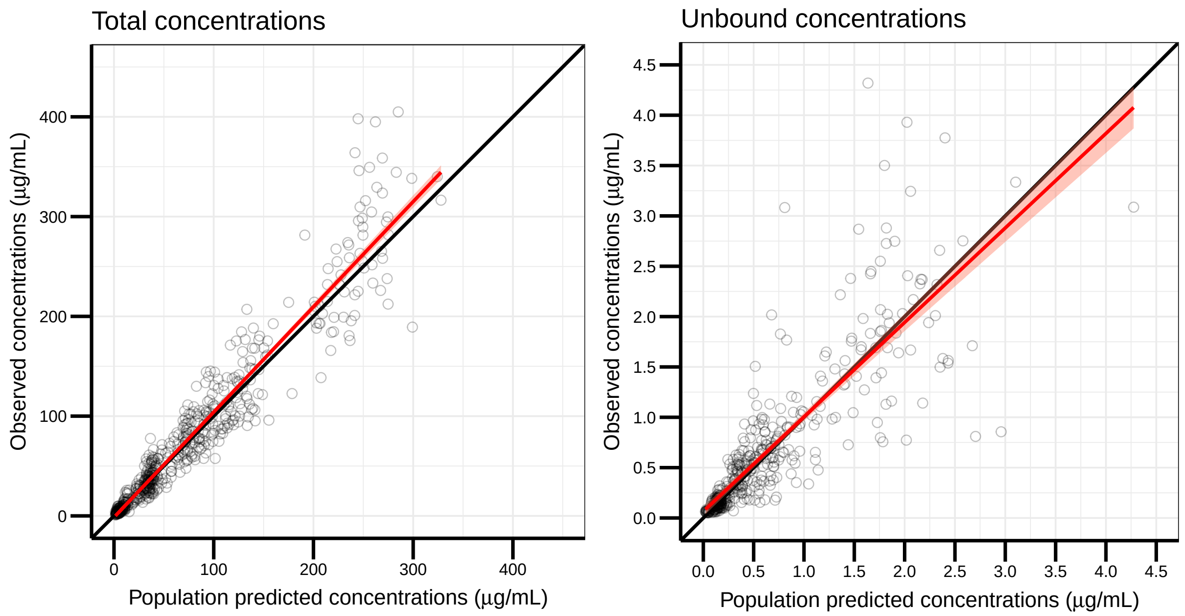

7.1.2 Log scale

Observed vs. Population predicted dalbavancin concentrations - Final Model

Note: Solid black lines represent the lines of identity, and red lines the linear regression lines.

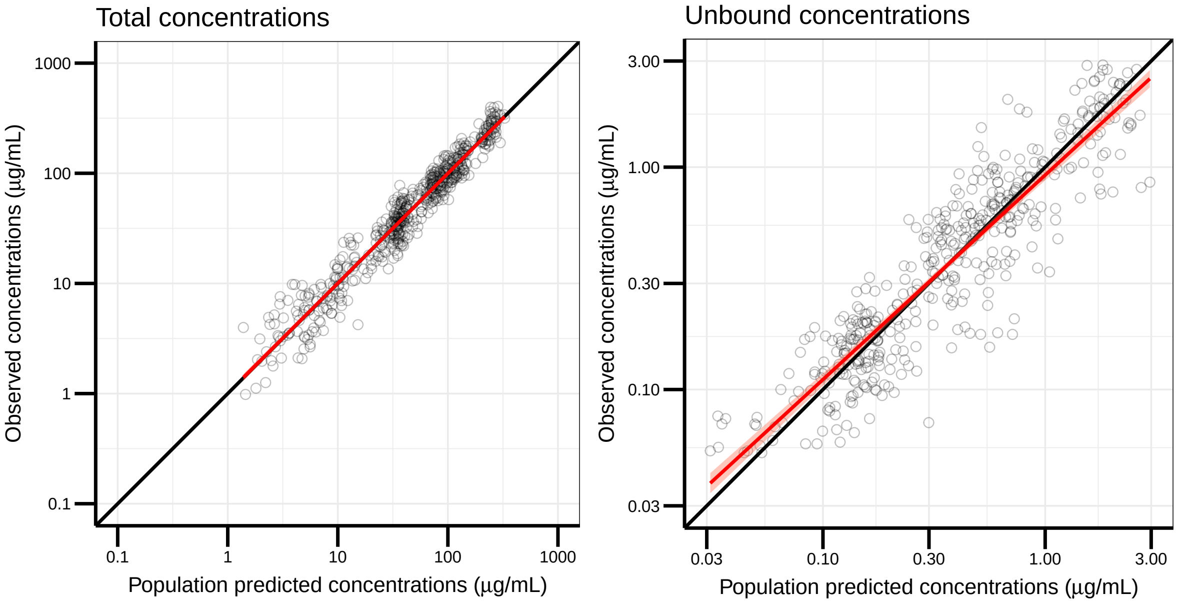

7.2 DV vs. IPRED

7.2.1 Linear scale

Observed vs. Individual predicted dalbavancin concentrations - Final Model

Note: Solid black lines represent the lines of identity, and red lines the linear regression lines (with 95% CI).

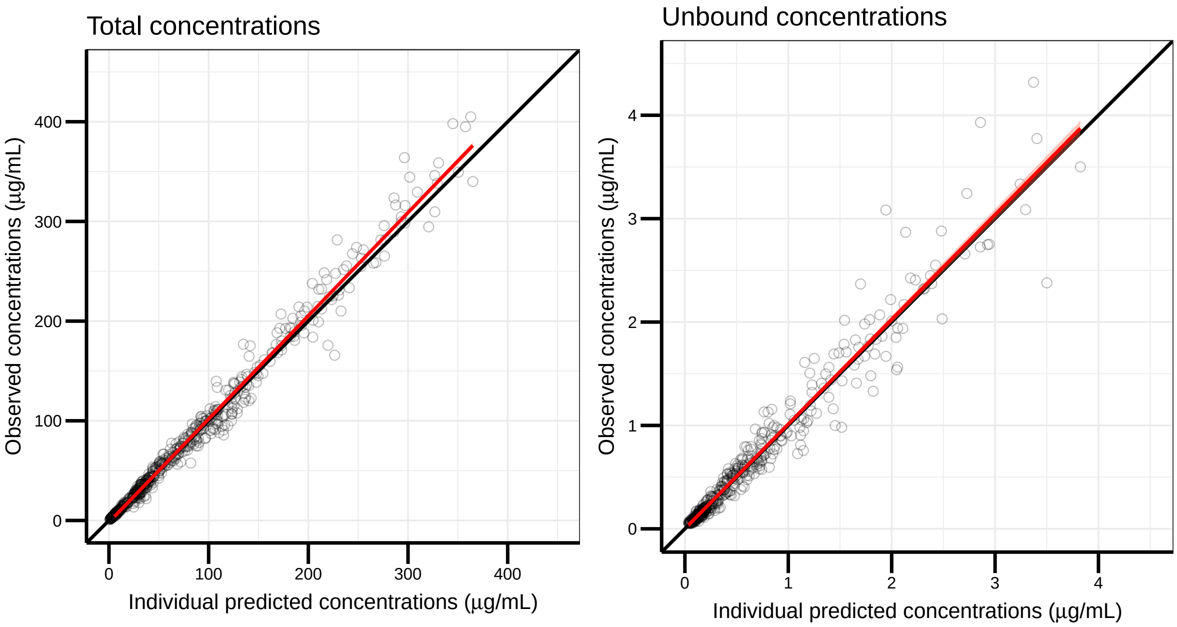

7.2.2 Log scale

Observed vs. Individual predicted dalbavancin concentrations - Final Model

Note: Solid black lines represent the lines of identity, and red lines the linear regression lines (with 95% CI).

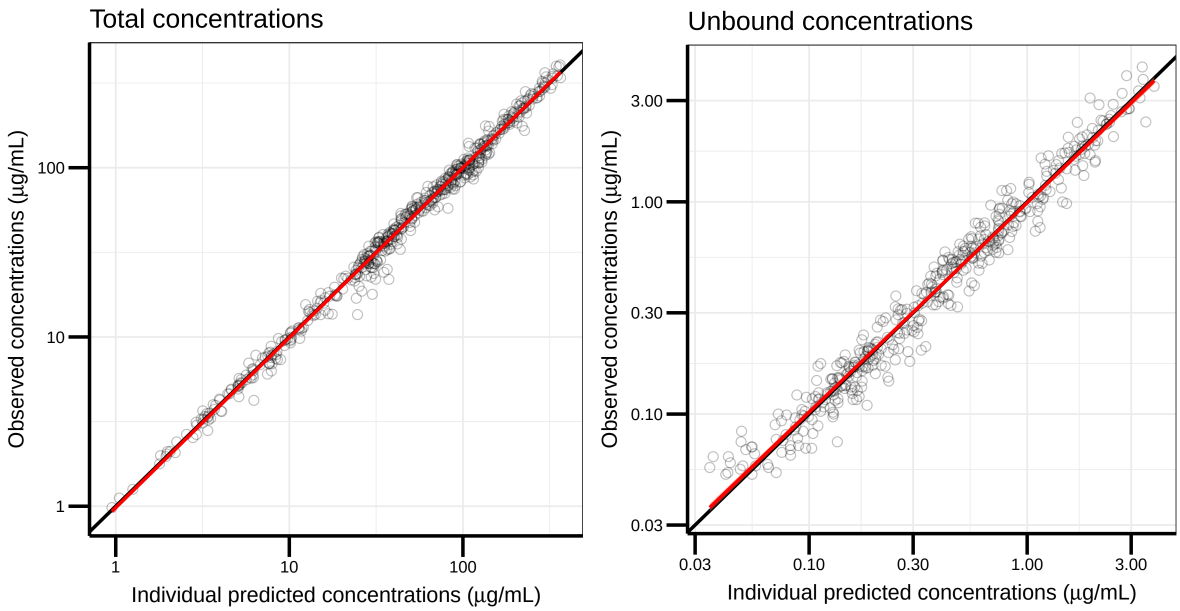

7.3 RES vs. Time after first dose

7.3.1 CWRES vs. Time

Conditional Weighted Residuals (CWRES) vs. Time after first dose - Final Model

Note: Solid black lines represent the lines of identity, and red curves the lowess smooth regression curves (with 95% CI).

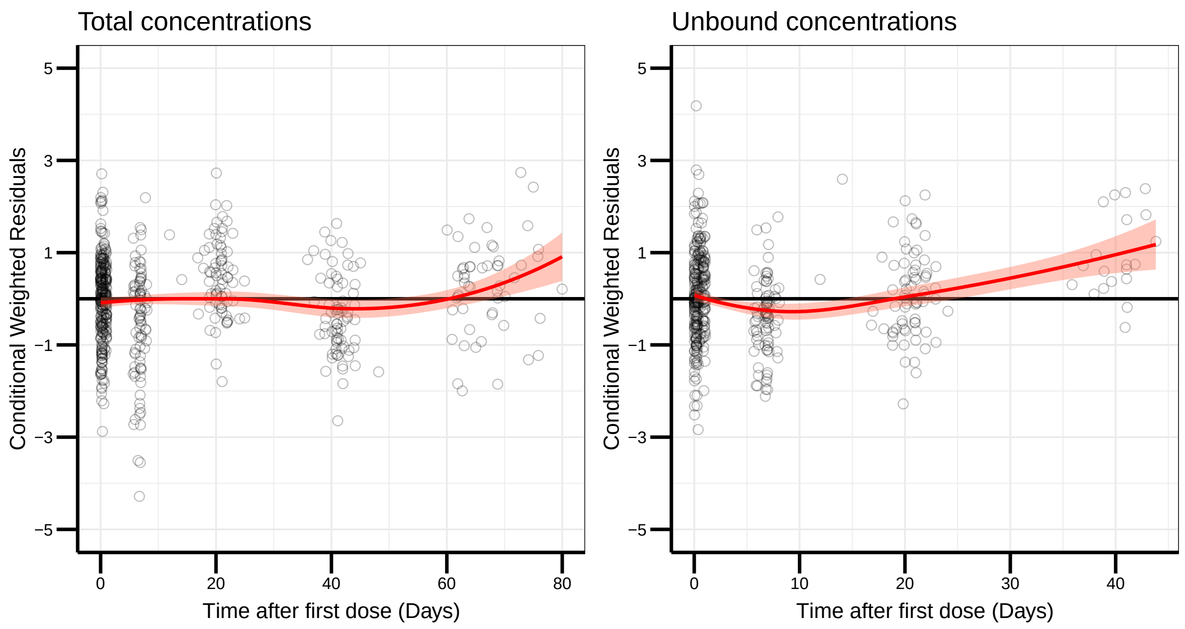

7.3.2 CWRES vs. Time (log scale)

Conditional Weighted Residuals (CWRES) vs. Time after first dose - Final Model

Note: Solid black lines represent the lines of identity, and red curves the lowess smooth regression curves (with 95% CI).

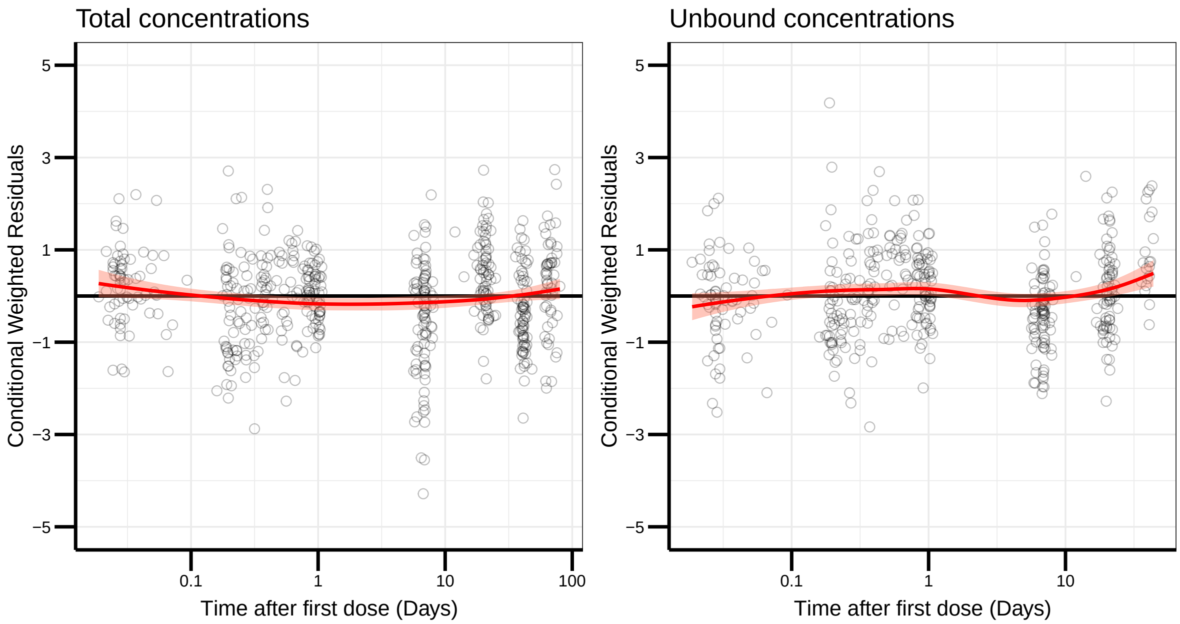

7.3.3 IWRES vs. Time

Individual Weighted Residuals (IWRES) vs. Time after first dose - Final Model

Note: Solid black lines represent the lines of identity, and red curves the lowess smooth regression curves (with 95% CI).

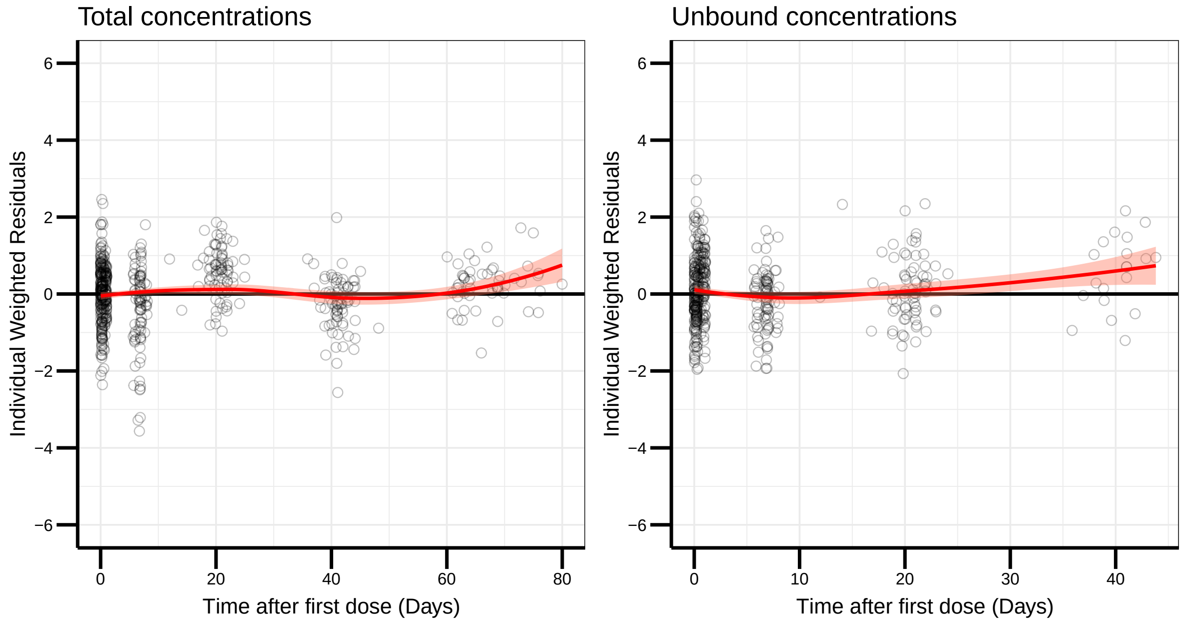

7.3.4 IWRES vs. Time (log scale)

Individual Weighted Residuals (IWRES) vs. Time after first dose - Final Model

Note: Solid black lines represent the lines of identity, and red curves the lowess smooth regression curves (with 95% CI).

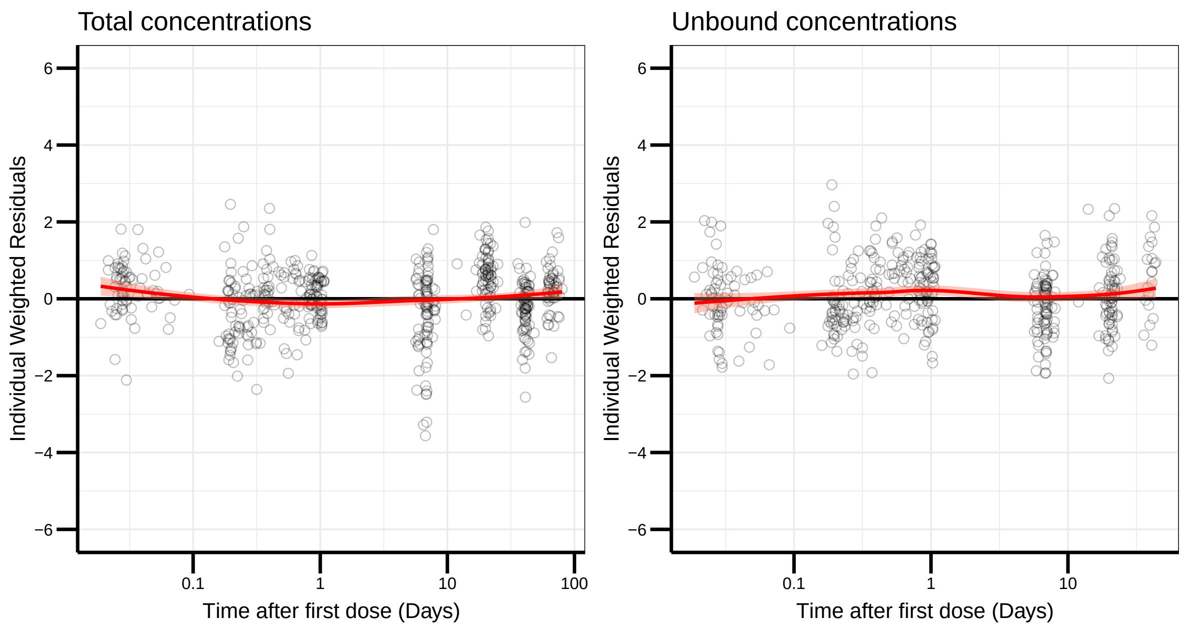

7.4 RES vs. Time from previous dose

7.4.1 CWRES vs. Time

Conditional Weighted Residuals (CWRES) vs. Time from previous dose - Final Model

Note: Solid black lines represent the lines of identity, and red curves the lowess smooth regression curves (with 95% CI).

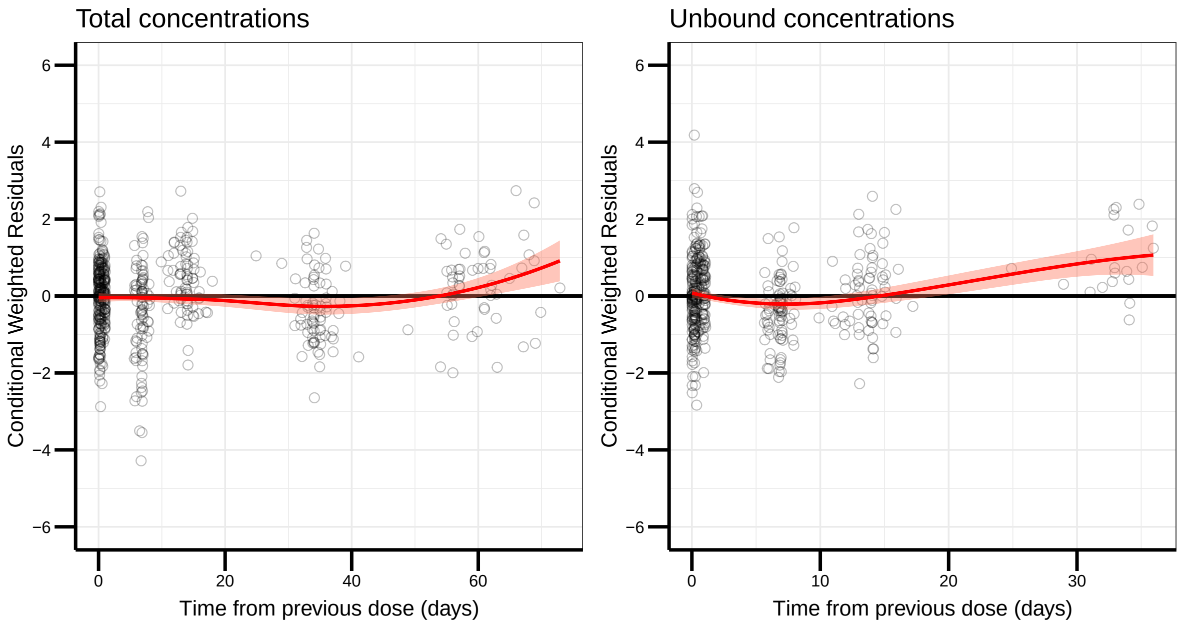

7.4.2 CWRES vs. Time (log scale)

Conditional Weighted Residuals (CWRES) vs. Time from previous dose - Final Model

Note: Solid black lines represent the lines of identity, and red curves the lowess smooth regression curves (with 95% CI).

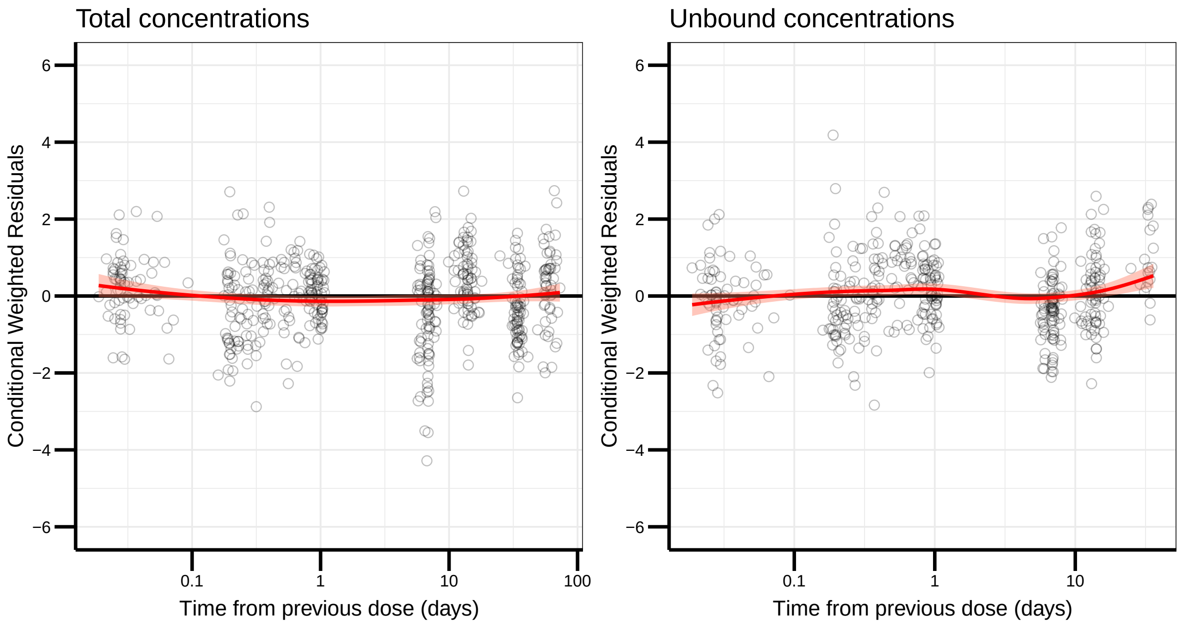

7.4.3 IWRES vs. Time

Individual Weighted Residuals (IWRES) vs. Time from previous dose - Final Model

Note: Solid black lines represent the lines of identity, and red curves the lowess smooth regression curves (with 95% CI).

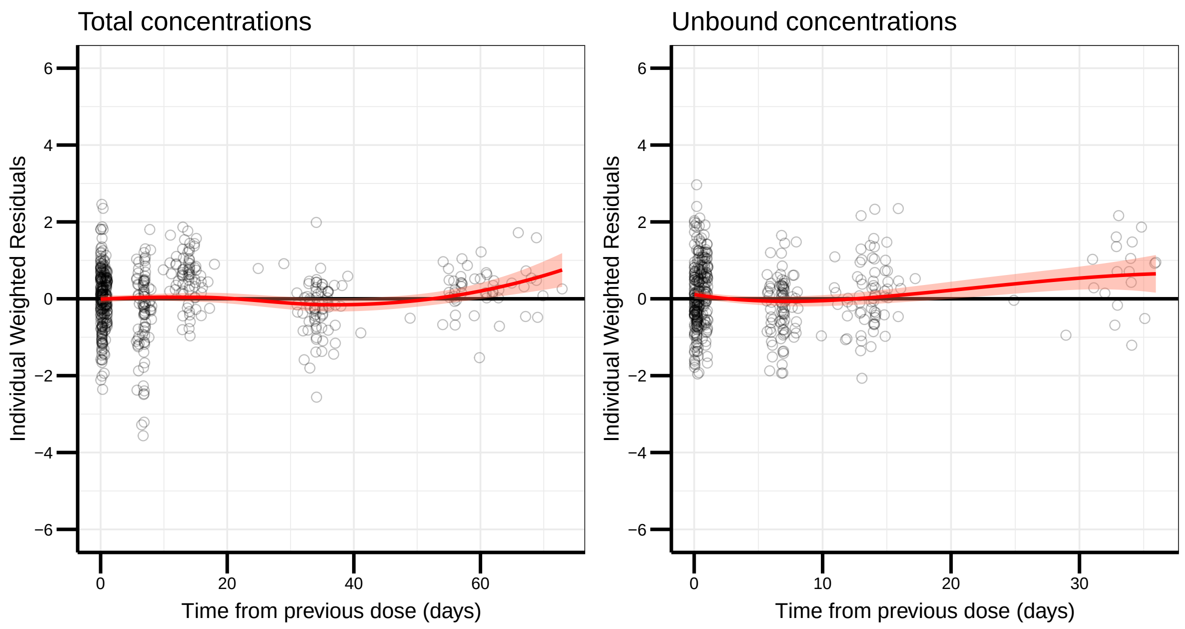

7.4.4 iWRES vs. Time (log scale)

Individual Weighted Residuals (IWRES) vs. Time from previous dose - Final Model

Note: Solid black lines represent the lines of identity, and red curves the lowess smooth regression curves (with 95% CI).

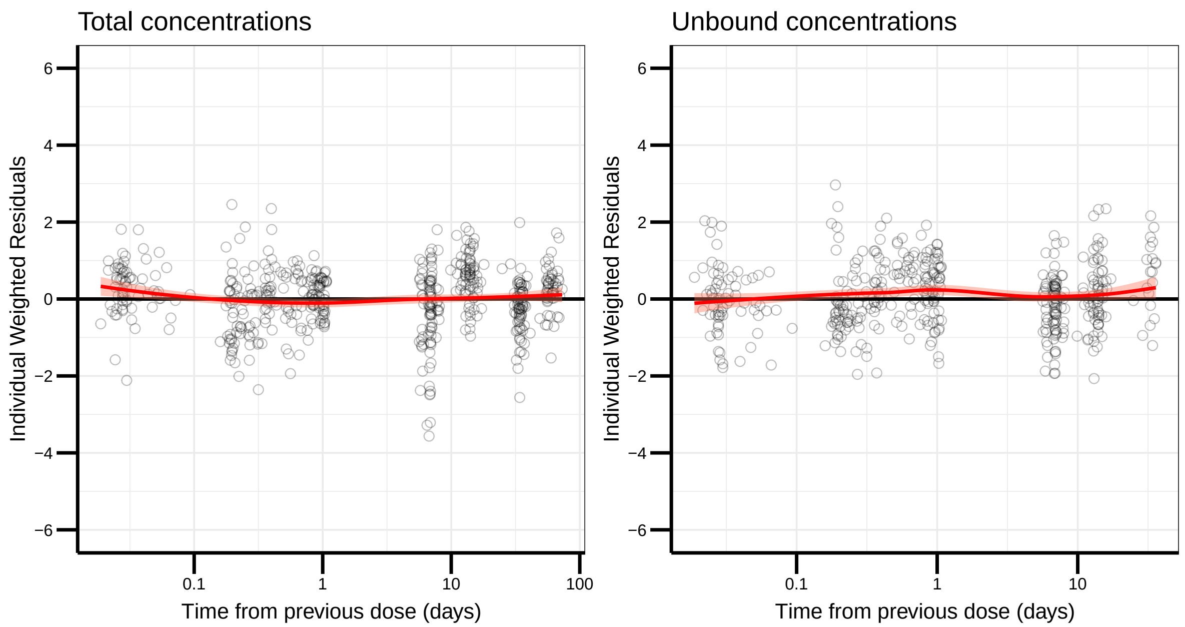

7.5 RES vs. PRED

7.5.1 CWRES vs. PRED

Conditional Weighted Residuals (CWRES) vs. Population predictions - Final Model

Note: Solid black lines represent the lines of identity, and red curves the lowess smooth regression curves (with 95% CI).

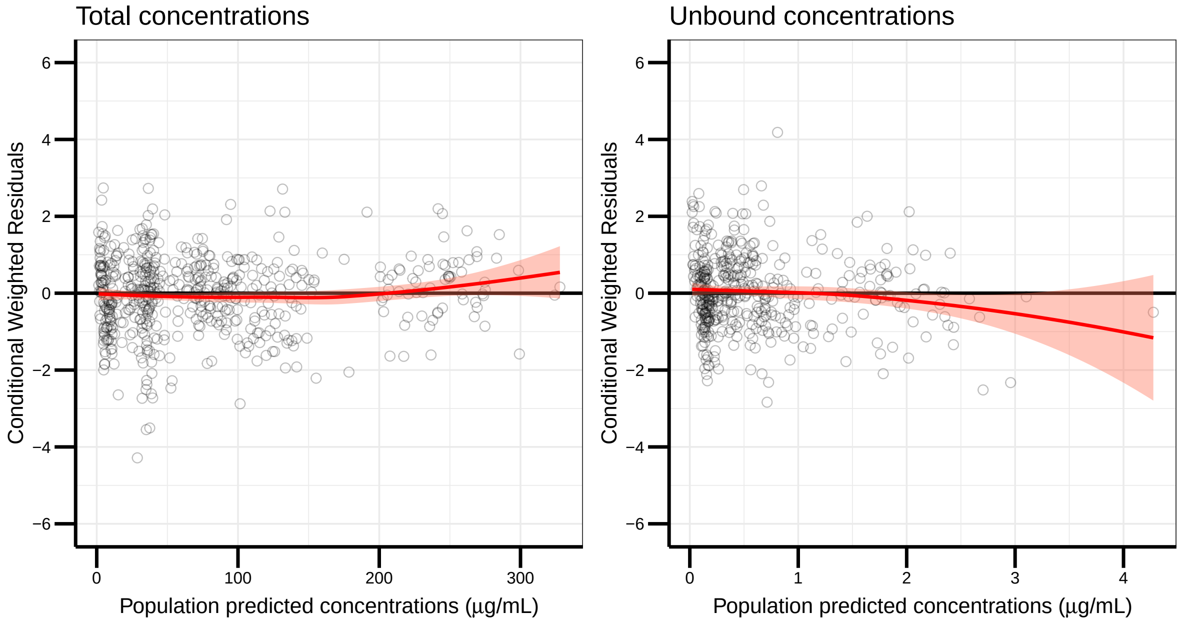

7.5.2 IWRES vs. IPRED

Individual Weighted Residuals (IWRES) vs. Individual predictions - Final Model

Note: Solid black lines represent the lines of identity, and red curves the lowess smooth regression curves (with 95% CI).

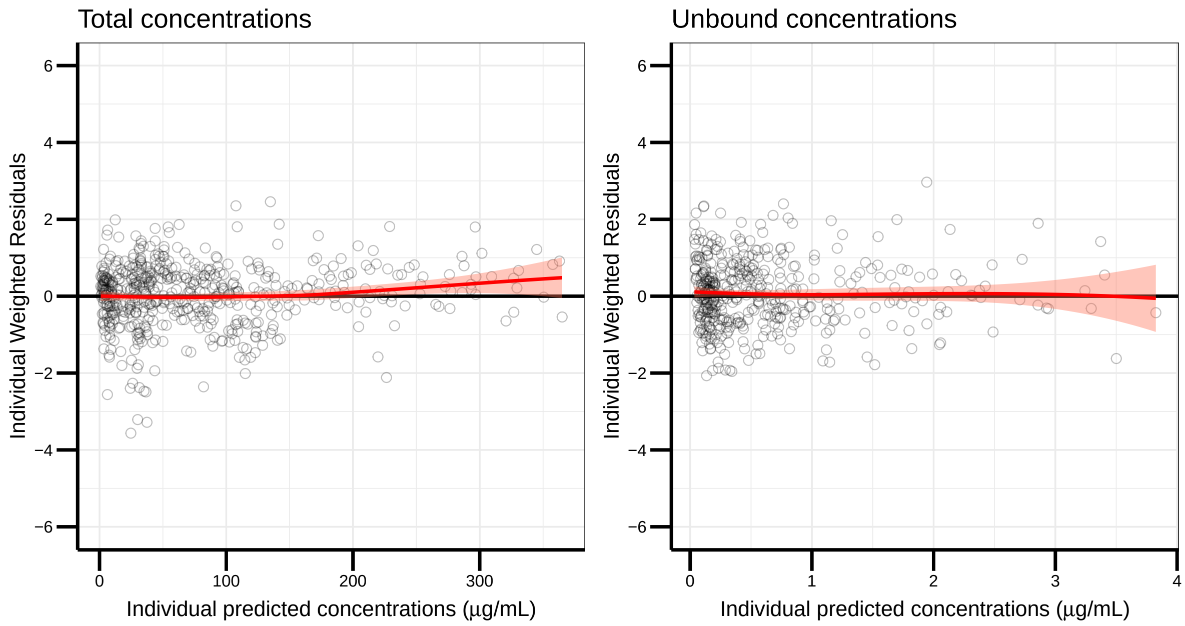

7.5.3 |IWRES| vs IPRED

|Individual Weighted Residuals| (|IWRES|) vs. Individual predictions - Final Model

Note: The red curves are the lowess smooth regression curves (with 95% CI).

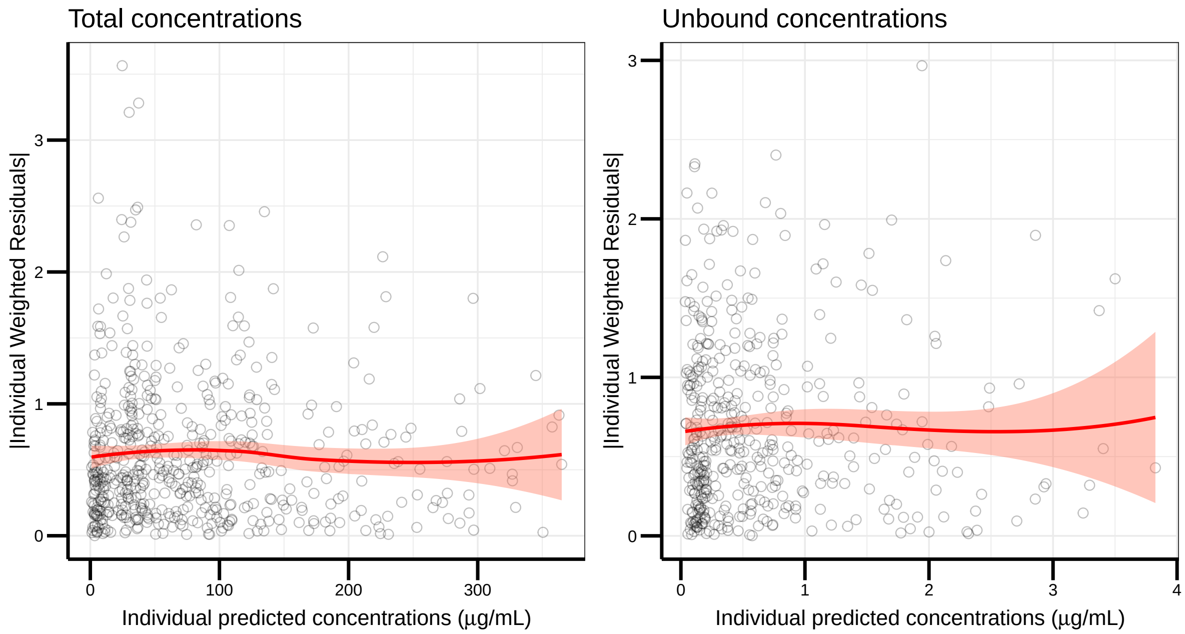

7.6 RES Distribution

7.6.1 CWRES hist

Distribution of Conditional Weighted Residuals (CWRES)

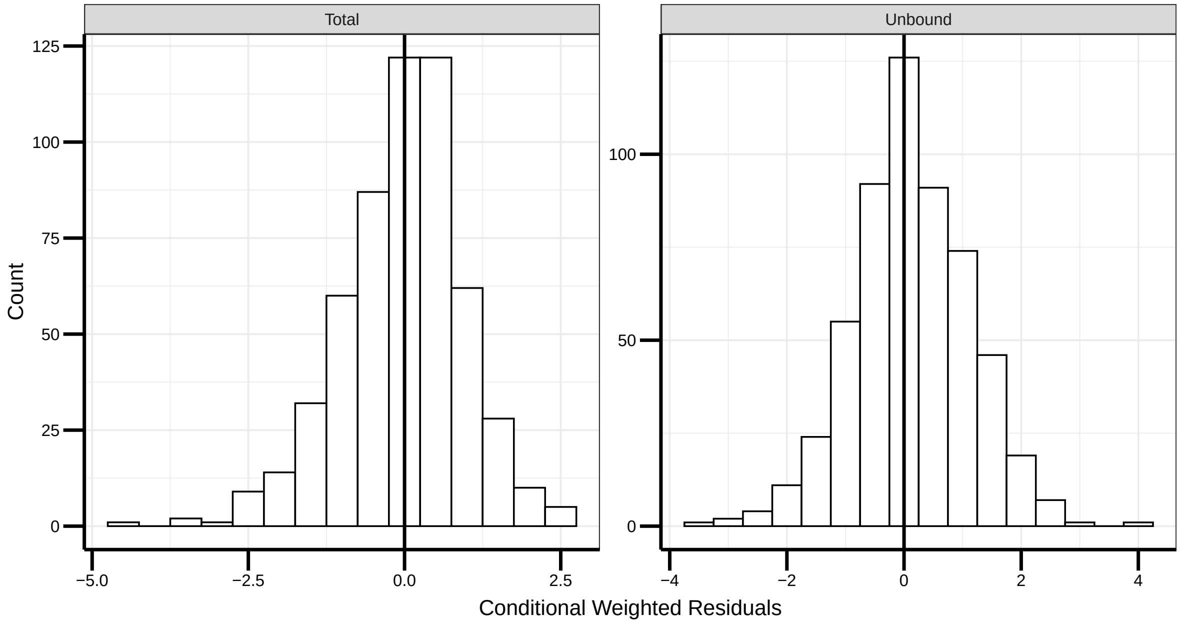

7.6.2 CWRES qqplot

QQplot of Conditional Weighted Residuals (CWRES)

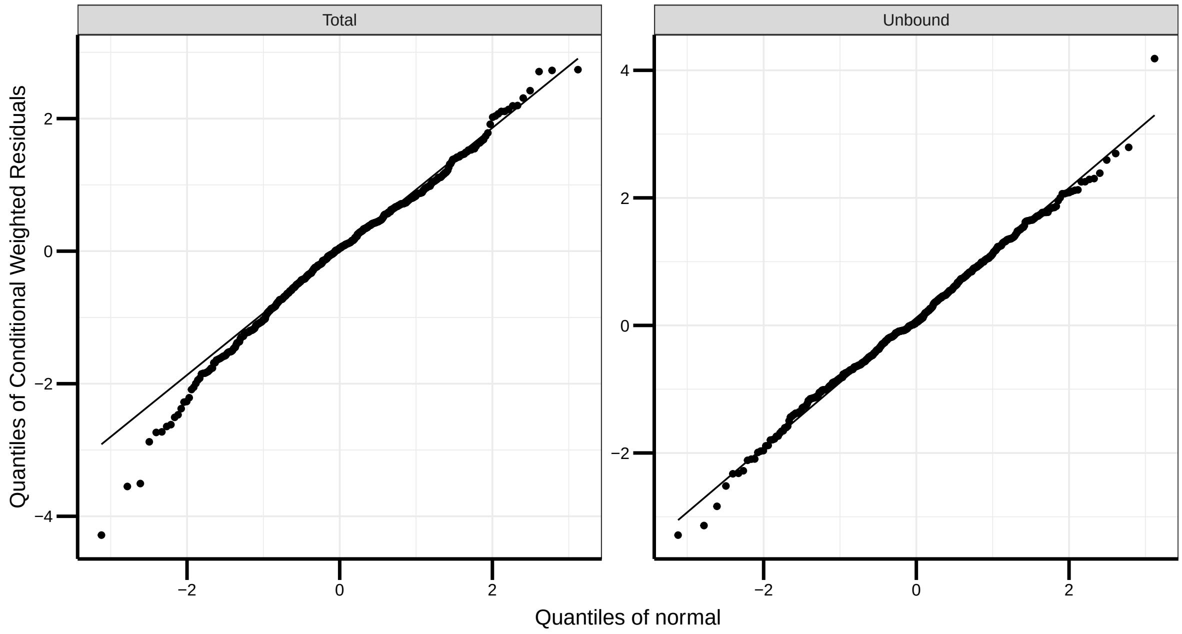

7.6.3 IWRES hist

Distribution of Individual Weighted Residuals (IWRES)

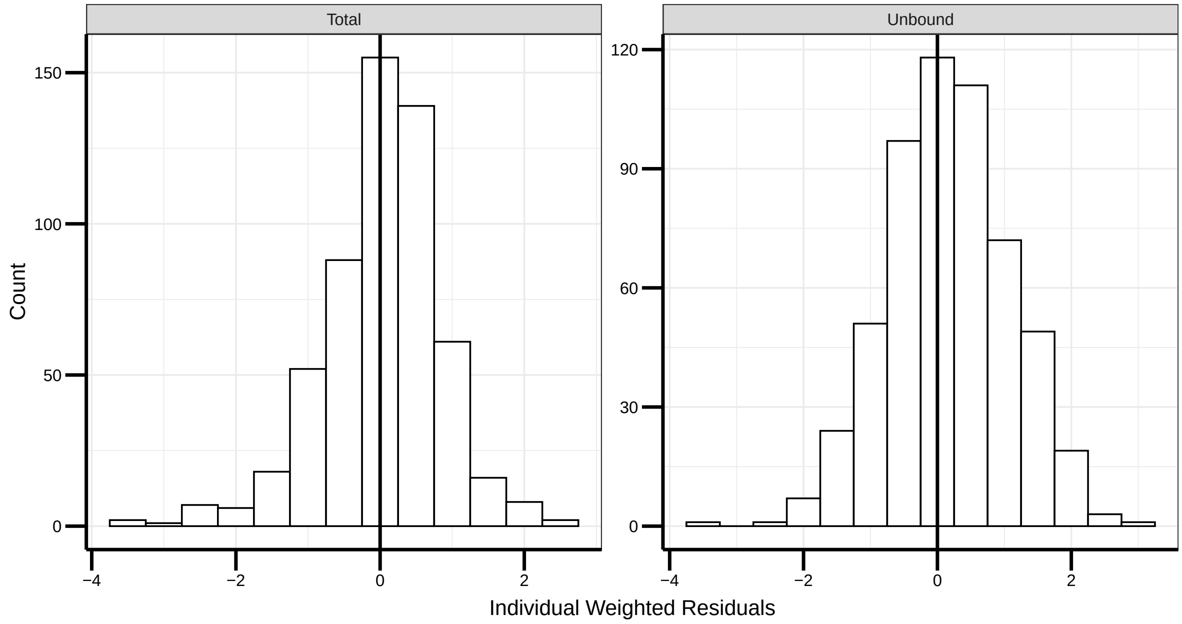

7.6.4 IWRES qqplot

QQplot of Individual Weighted Residuals (IWRES)

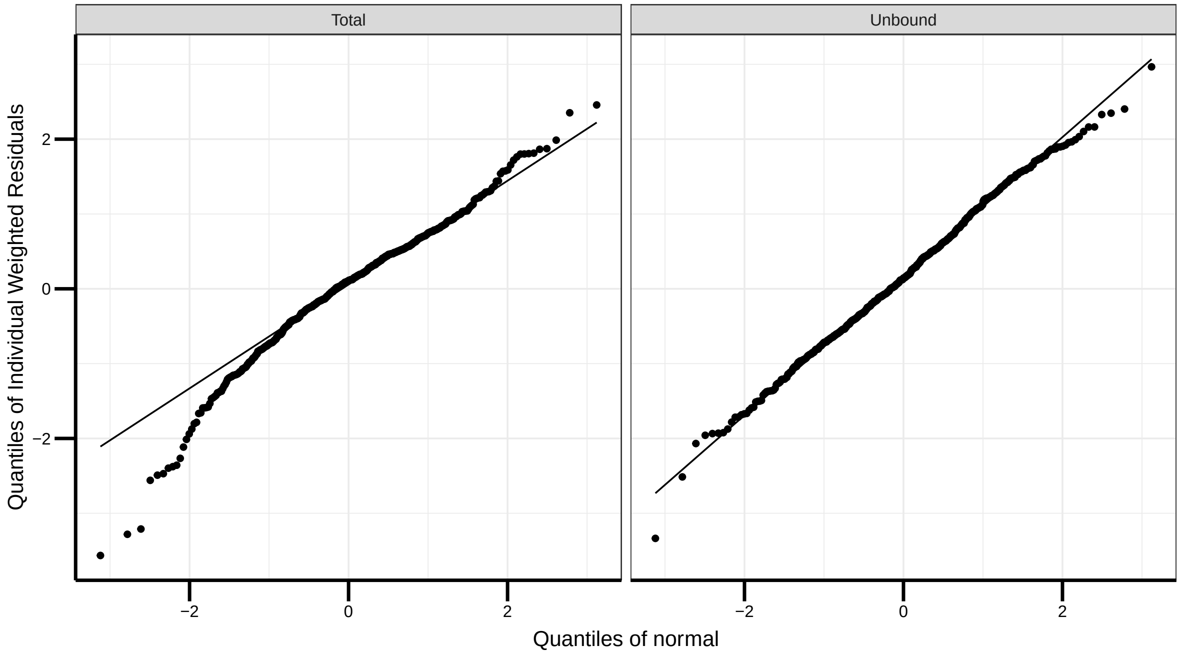

7.7 NPDE Distribution

7.7.1 NPDE hist

Distribution of Normalized Prediction Distribution Errors (NPDE)

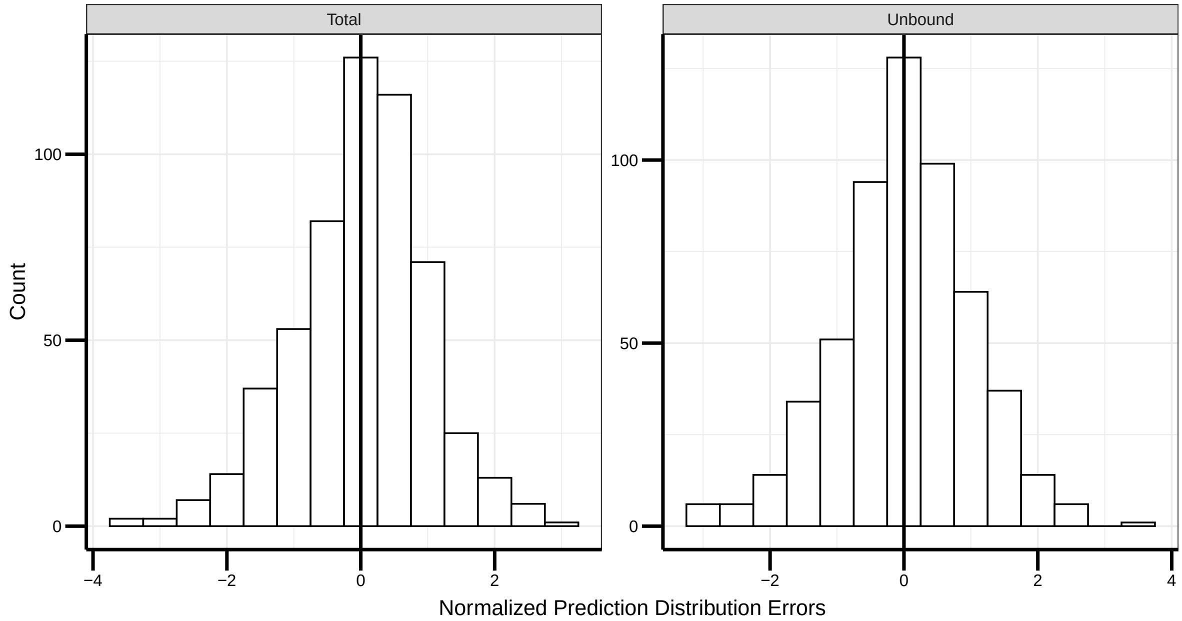

7.7.2 NPDE qqplot

QQplot of Normalized Prediction Distribution Errors (NPDE)

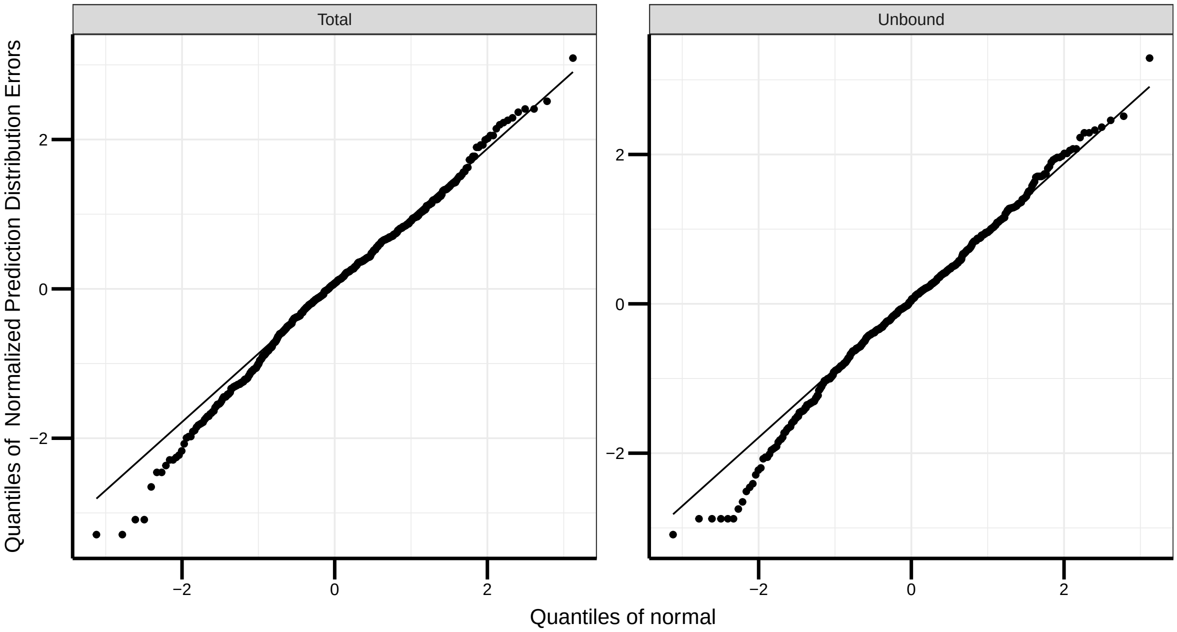

7.8 BSV Distribution

7.8.1 Hist

Distribution of between-subject variability (ETAs) - Final Model

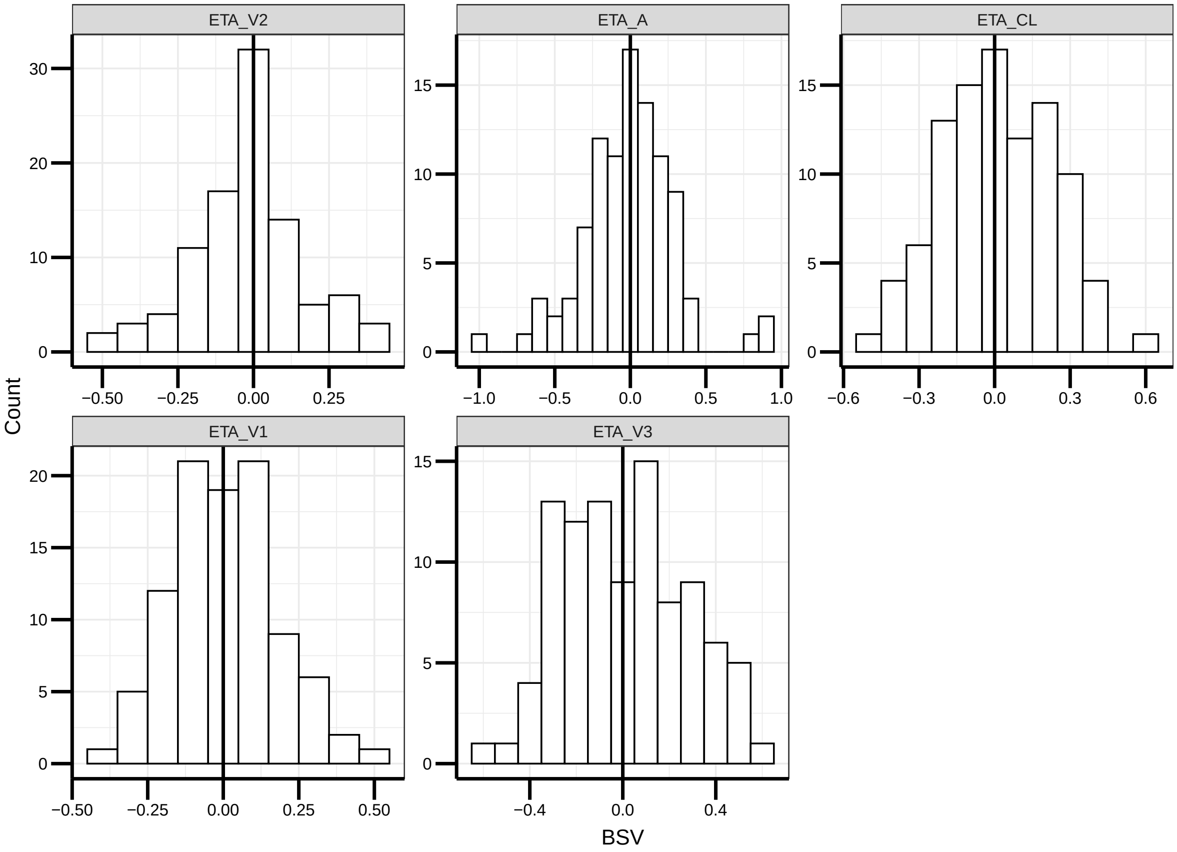

7.8.2 QQplots

Quantile-Quantile plot of between-subject variability (ETAs) - Final Model

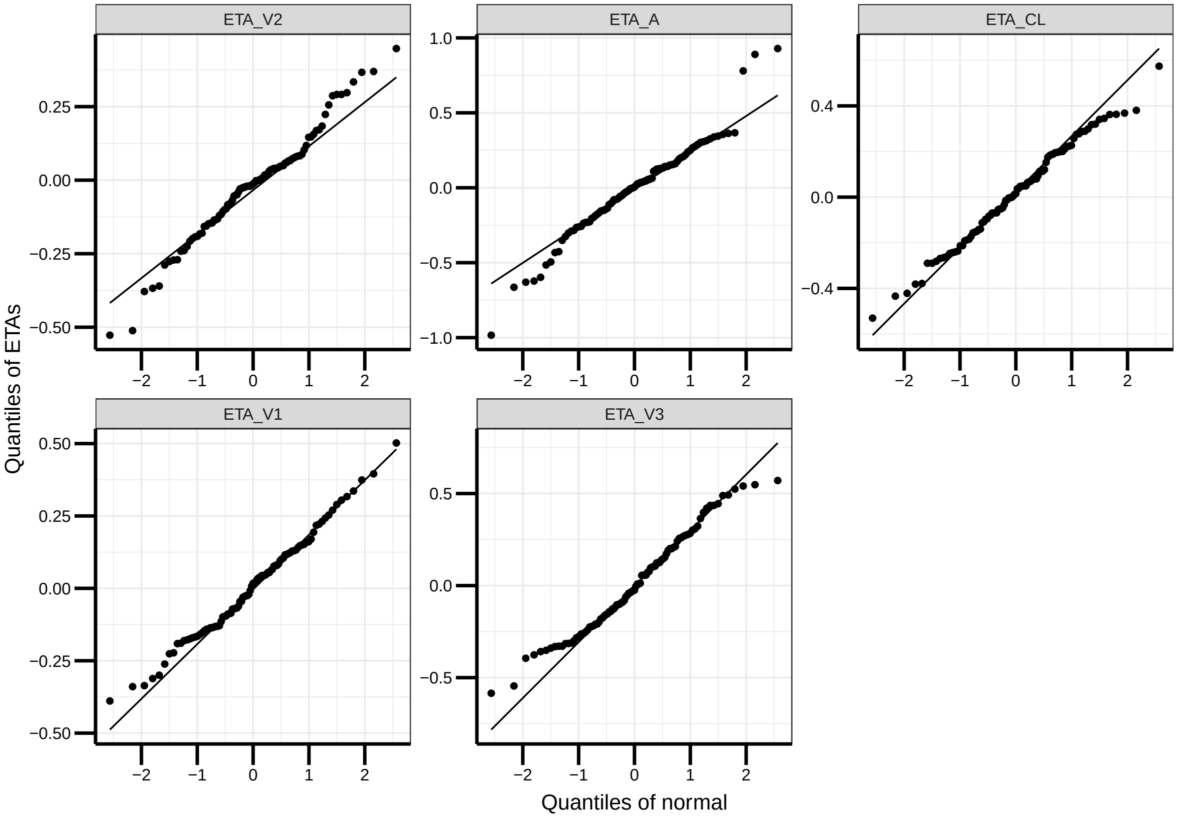

7.9 ETAs Correlations

Correlations of between-subject variability (ETAs) - Final Model

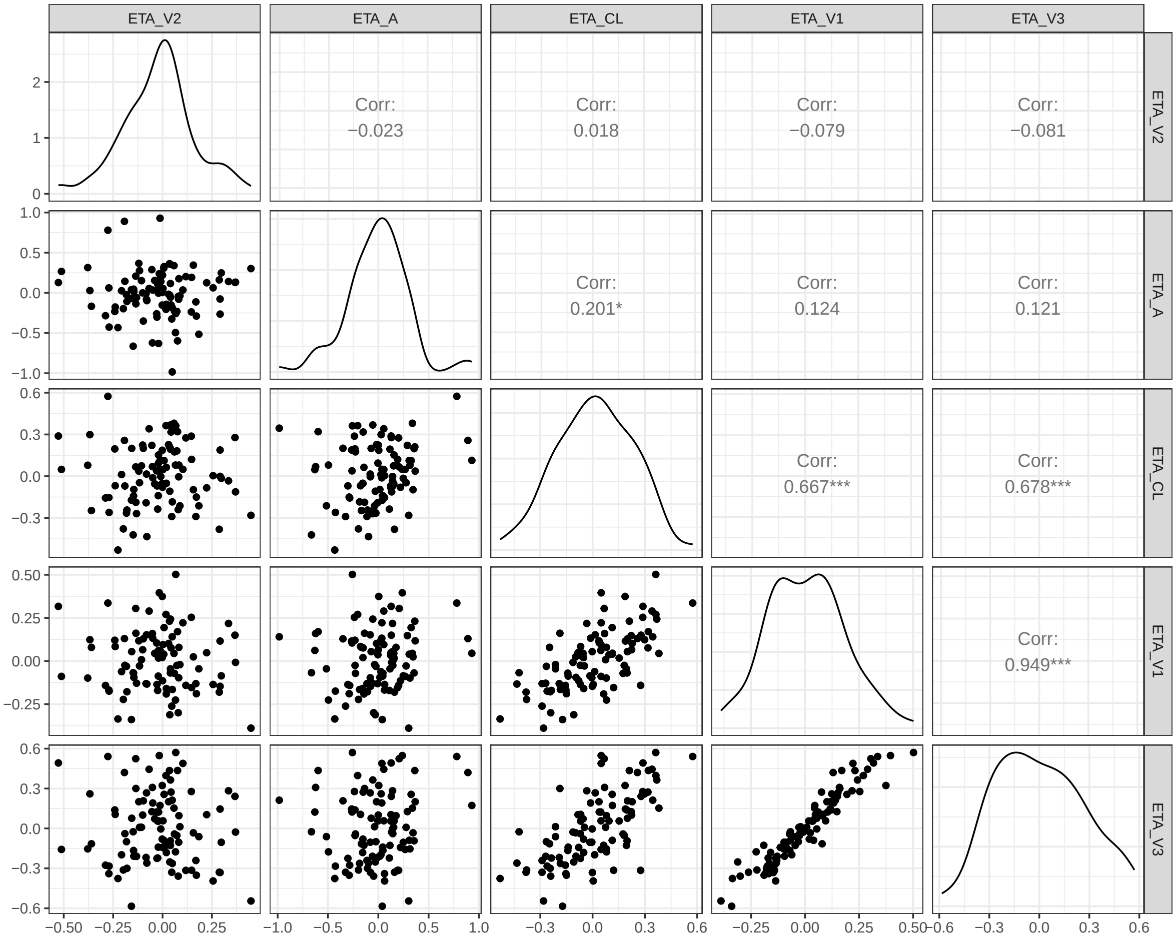

7.10 BSV vs. Cov

> **Note**
>
> - Lab values correspond to baseline values.

Between-subject variability (ETAs) vs. continuous and categorical covariates - Final Model

Note: Red curves correspond to the lowess smooth regression curves (with 95% CI).

7.10.1 Continuous covariates

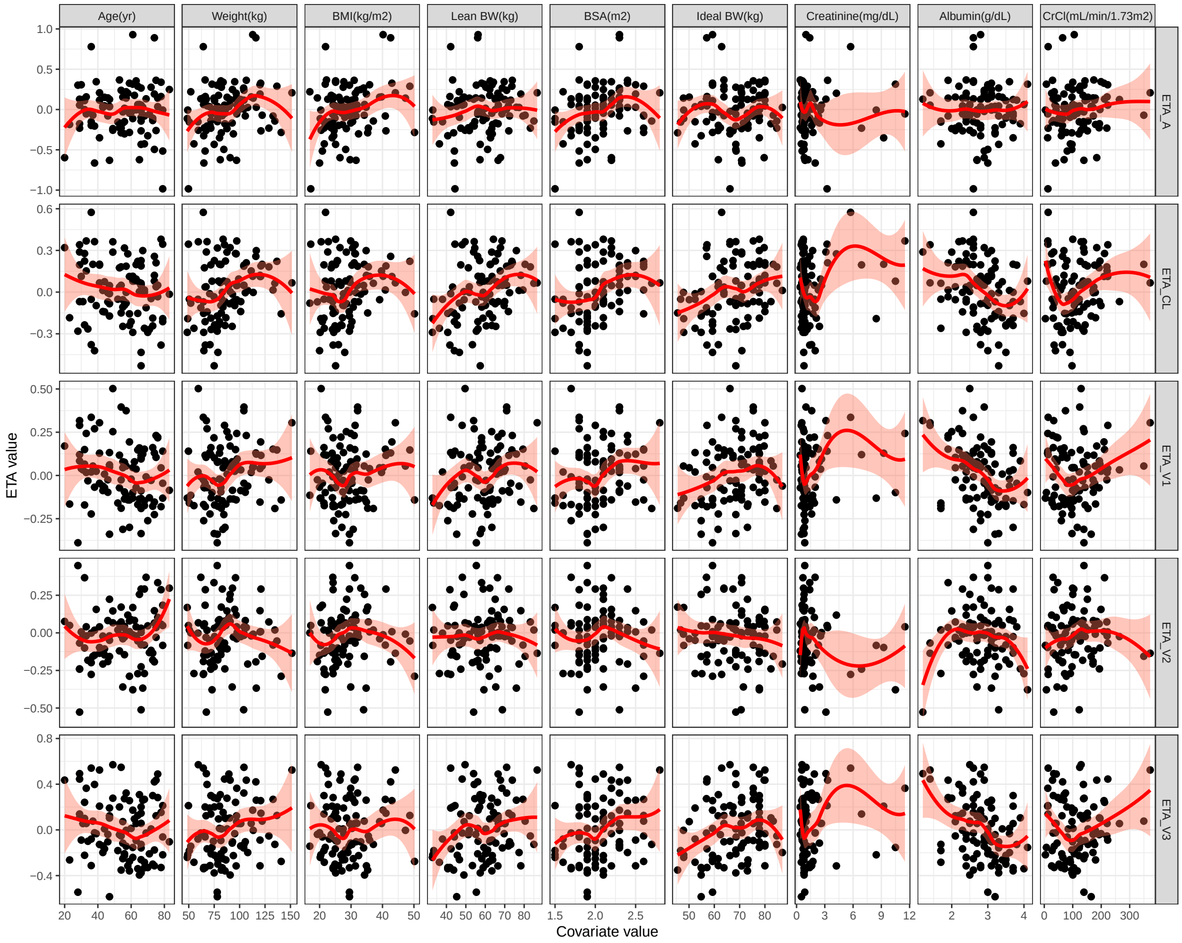

7.10.2 Continuous covariates (Log scale)

Covariate values in log scale

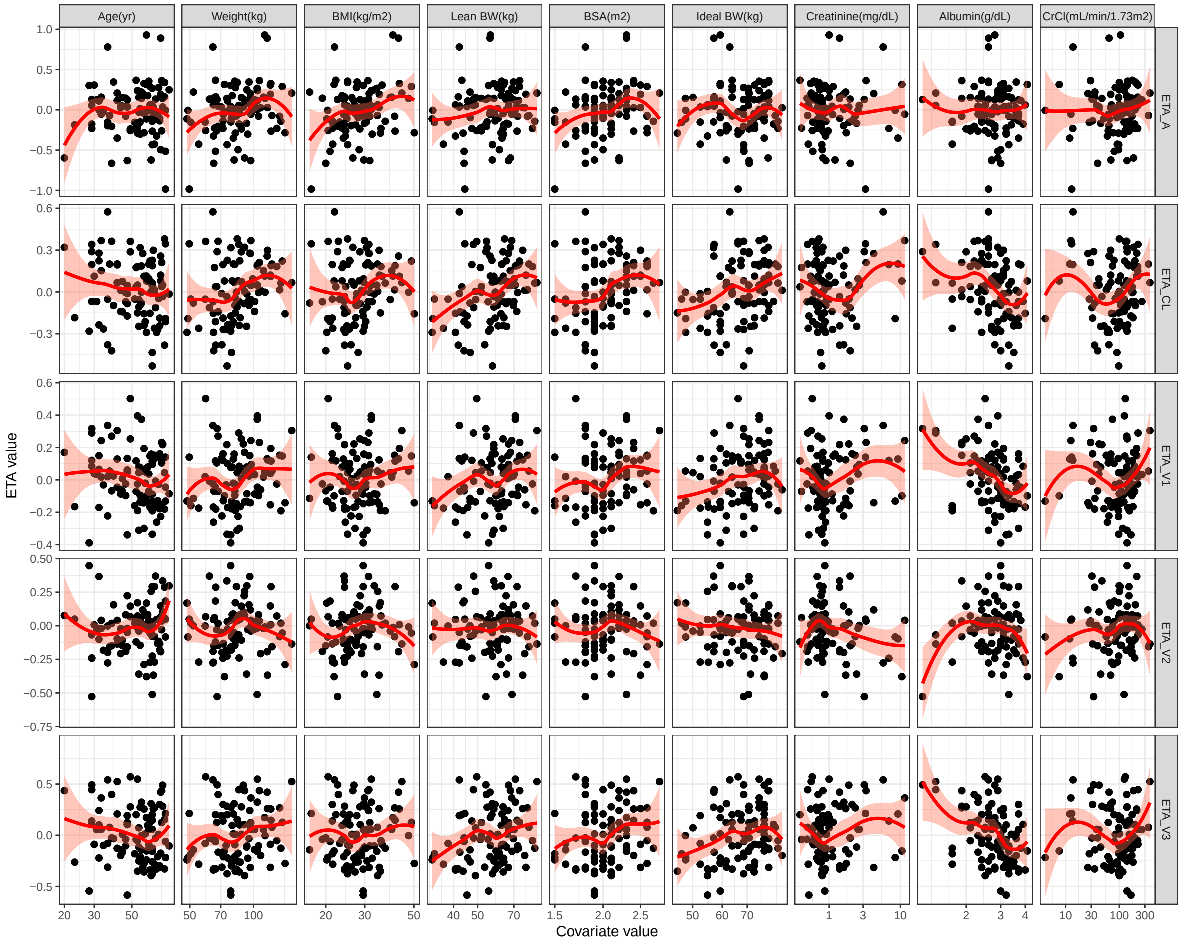
Age(yr)
Weight(kg)
BMI(kg/m2)
Lean BW(kg)
BSA(m2)
Ideal BW(kg)
Creatinine(mg/dL)
Albumin(g/dL)
CrCl(mL/min/1.73m2)
ETA_A
ETA_CL
ETA_V1
ETA_V2
ETA_V3
ETA value
Covariate value

7.10.3 Categorical covariates

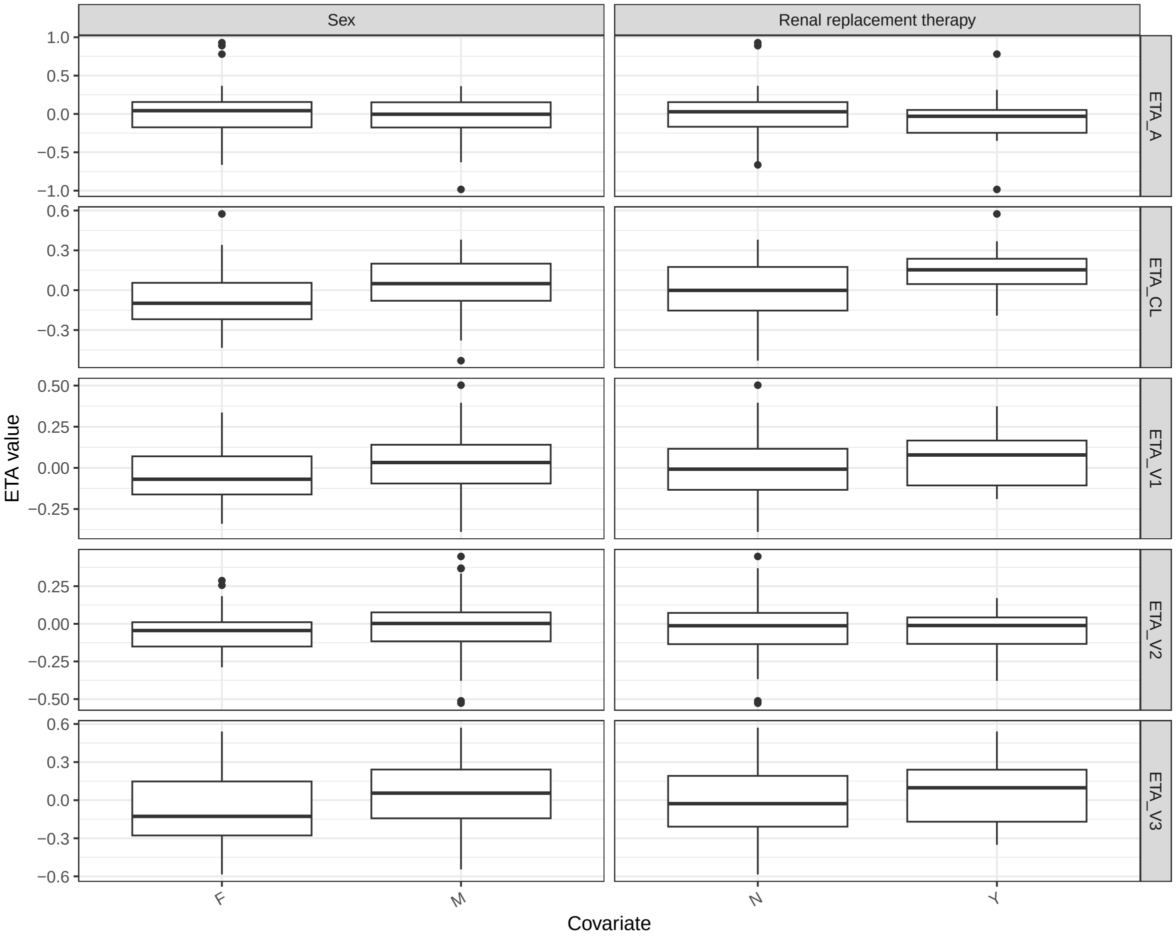

8 INDIVIDUAL FITS

Individual fits were generated in R and exported as a .pdf file in : ./results/05-final-model/indiv-fits-final.pdf

Individual fits with logged time axis in: ./results/05-final-model/indiv-fits-loggedtime-final.pdf

```
pdf
  2
```

```
pdf
  2
```

9 INDIVIDUAL PARAMETERS VS COVARIATES

> **Note**
>
> For covariate-parameters included in the final popPK model

9.1 CL ~ CrCl

Individual time-varying clearance vs. time-varying creatinine clearance - Final Model

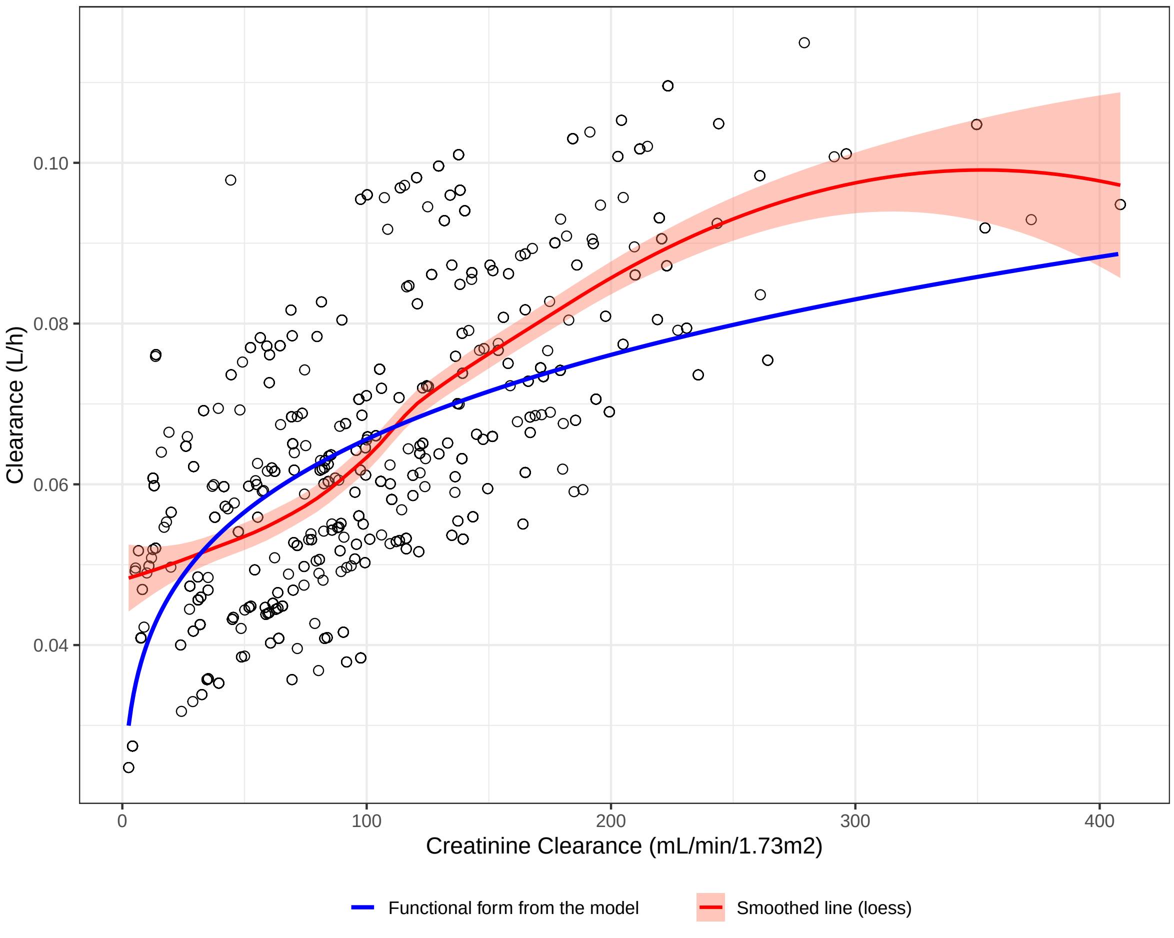

9.2 V1 ~ WTB

Individual volume of distribution in the central compartment vs. baseline weight - Final Model

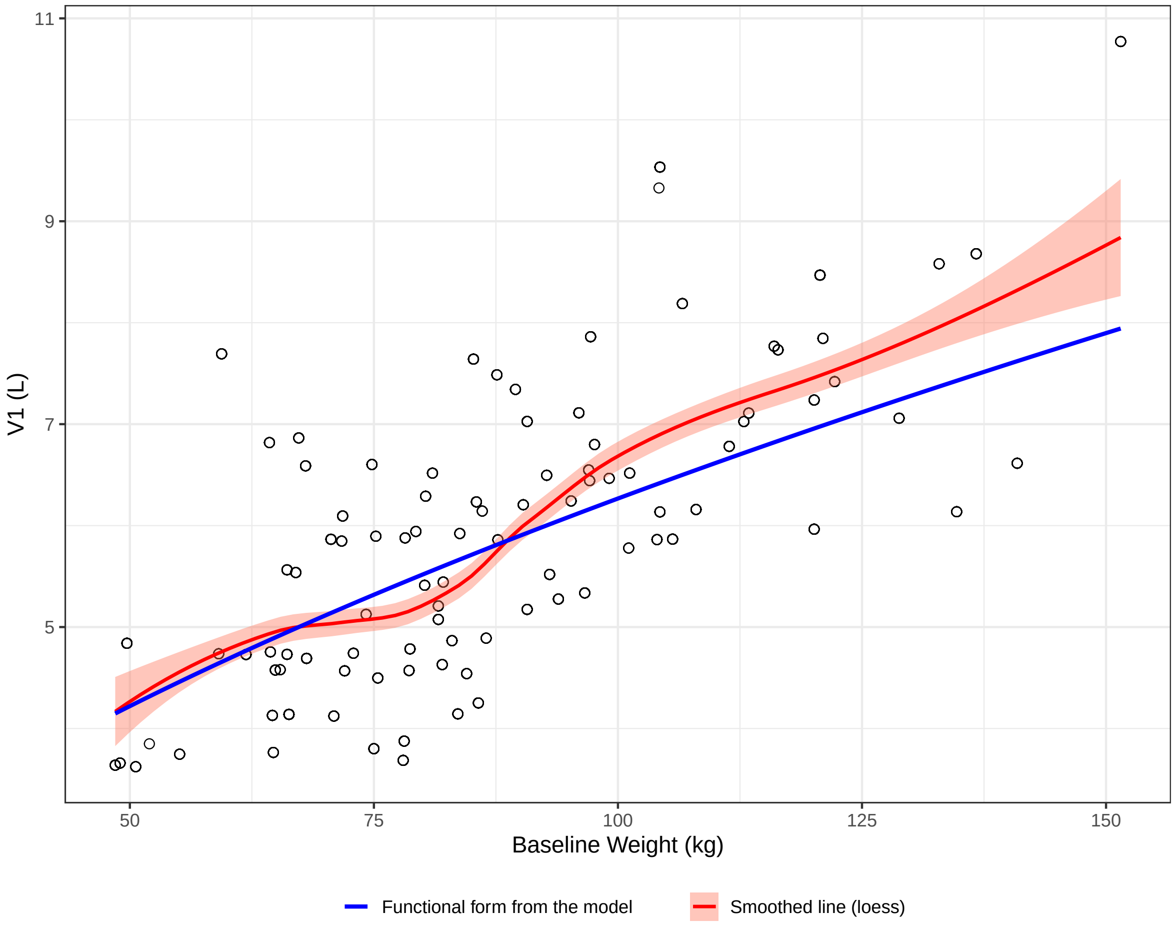

9.3 V2 ~ WTB

Individual volume of distribution of 2nd compartment vs. baseline weight - Final Model

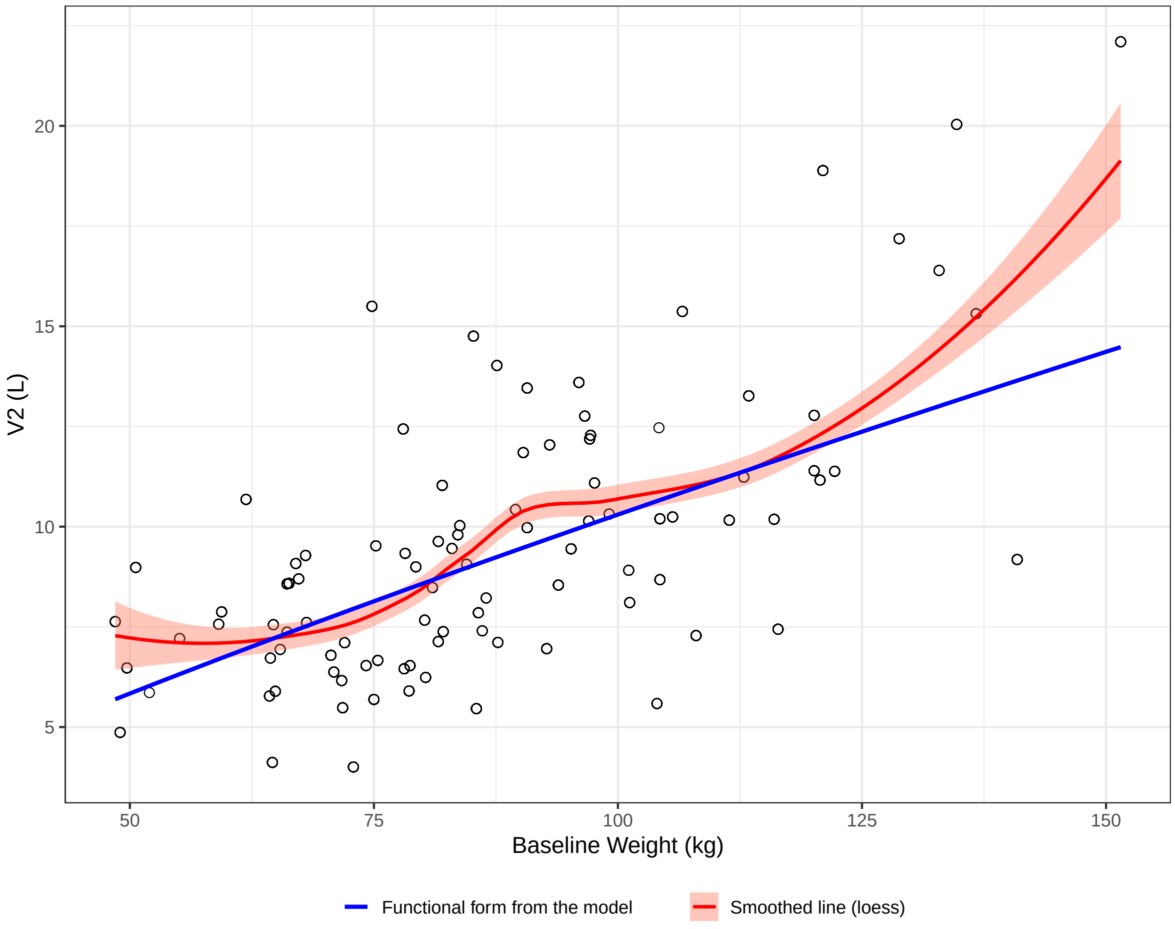

9.4 V2 ~ ALBB

Individual volume of distribution of 2nd compartment vs. baseline albumin - Final Model

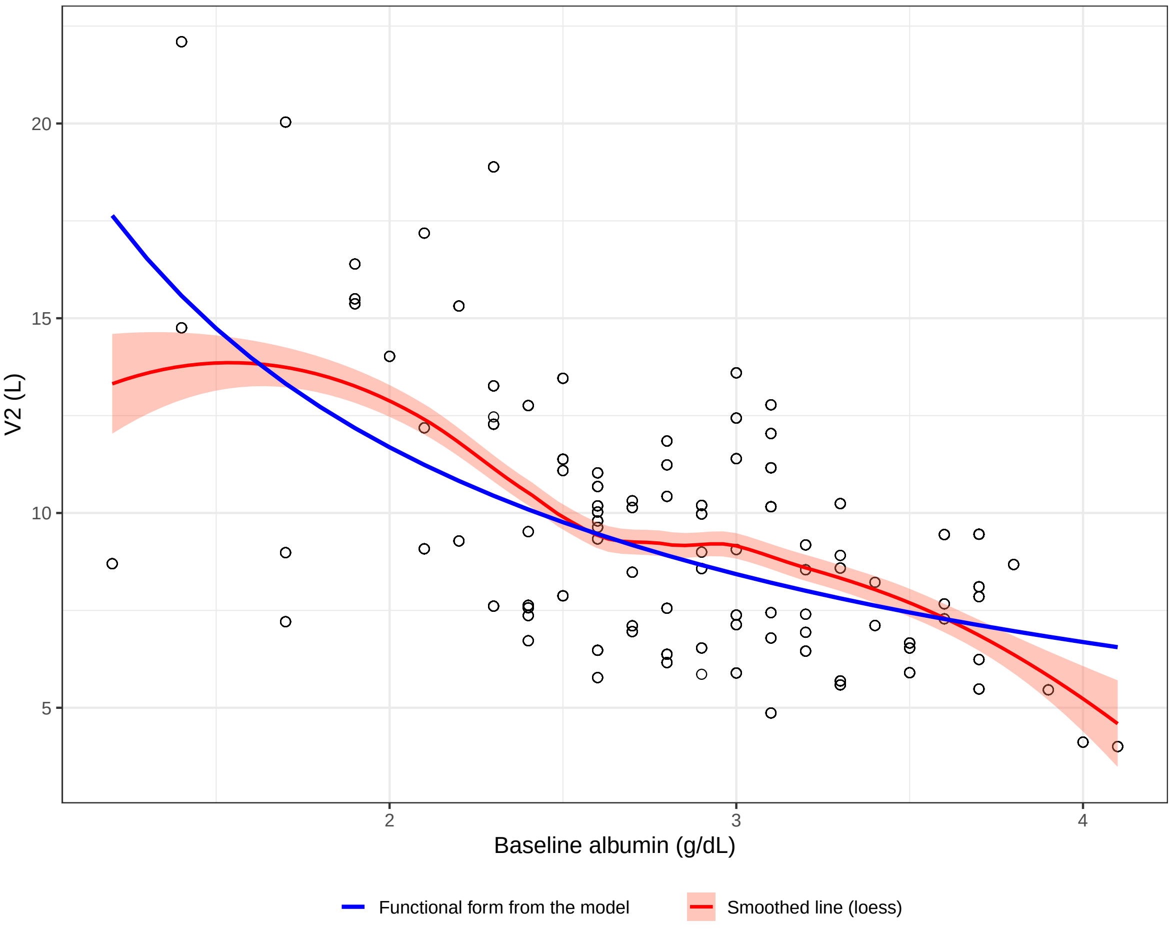

9.5 V3 ~ AGE

Individual volume of distribution of 3rd compartment vs. age - Final Model

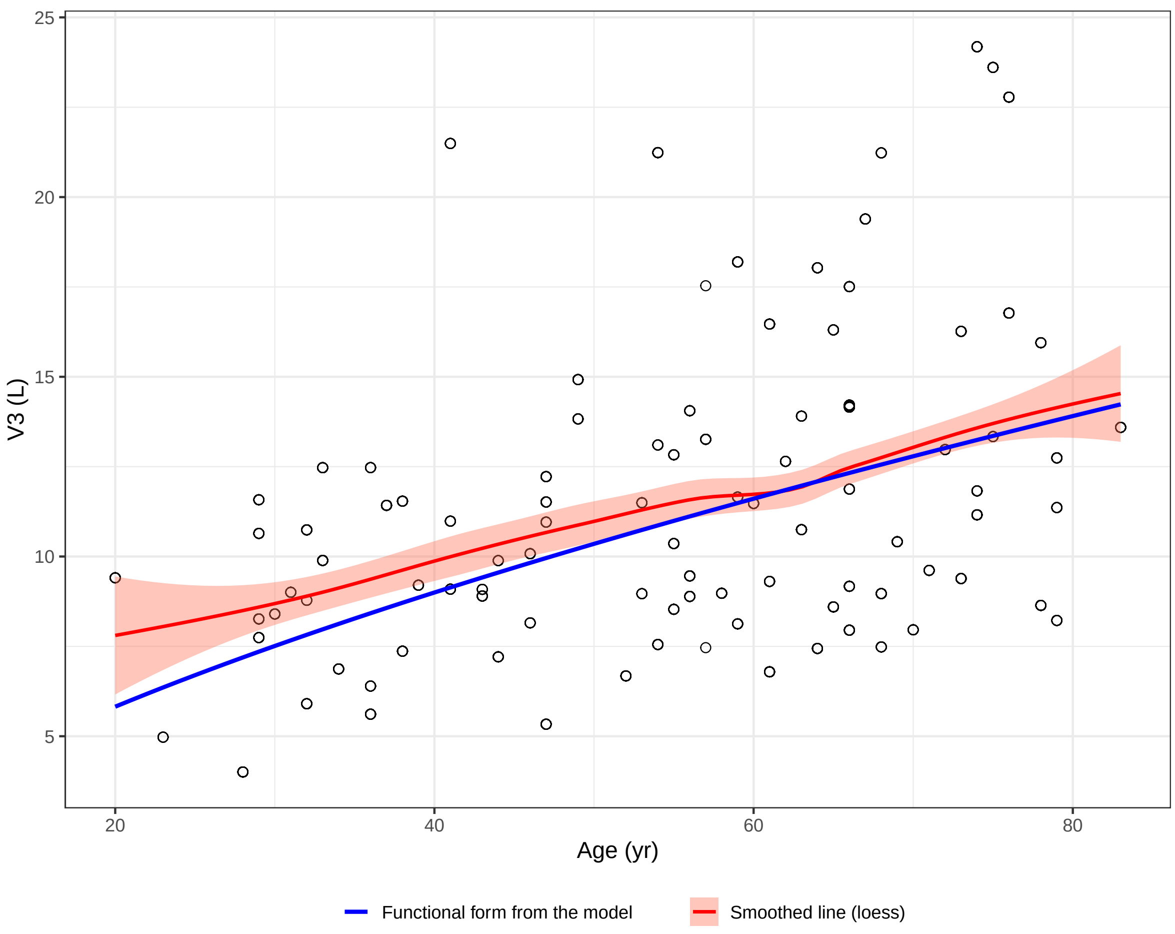

9.6 V3 ~ WTB

Individual volume of distribution of 3rd compartment vs. baseline weight - Final Model

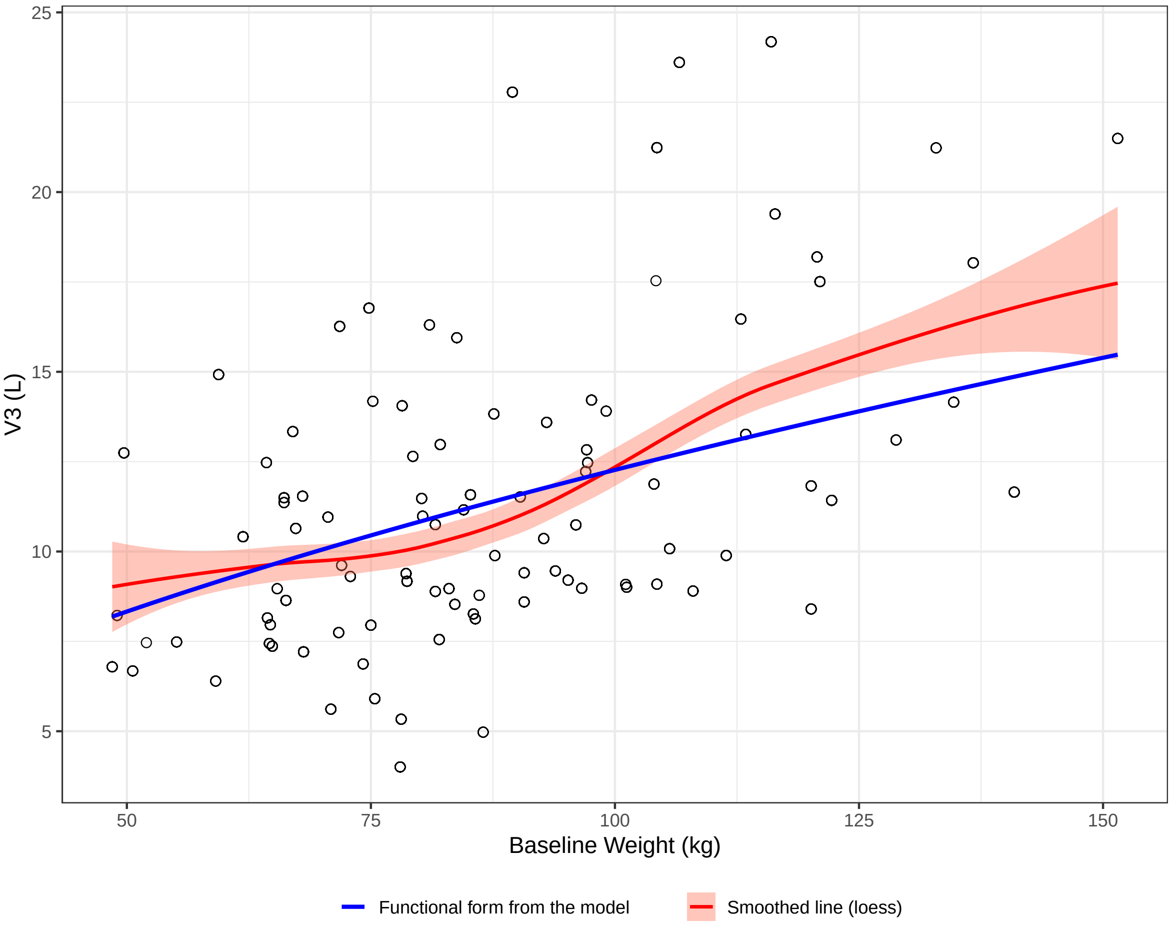

9.7 A ~ ALBB

Individual scaling factor vs. baseline albumin - Final Model

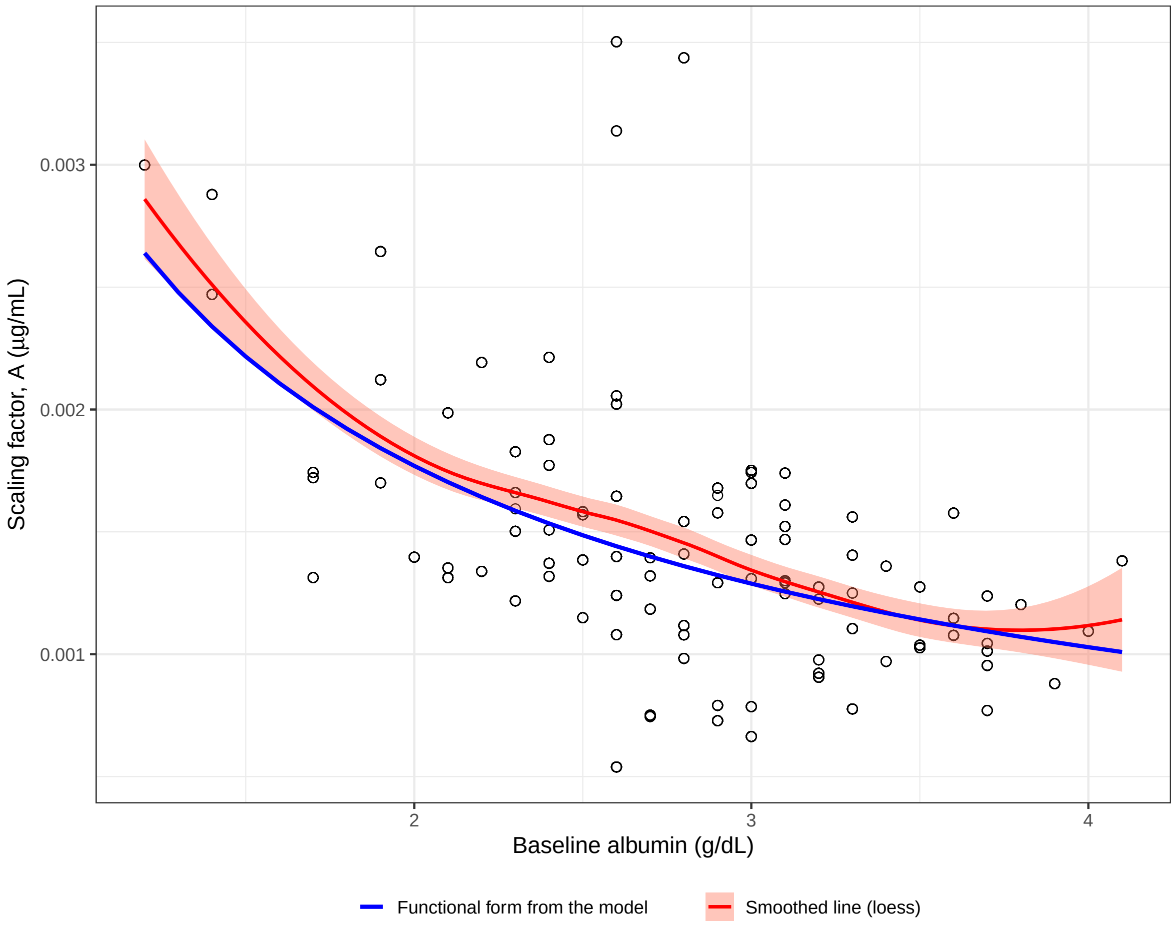

10 COVARIATE-EFFECT PLOT

! Important

- Dalbavancin CL was 35.1% lower at the 5th percentile of baseline CrCl (13.5 mL/min/1.73m2) compared to the median (101.26 mL/min/1.73m2).
- Dalbavancin V1 was 21.8% lower at the 5th percentile of baseline body weight (54.5 kg) compared to the median (83.8 kg).
- Dalbavancin V2 was 29.7% lower at the 5th percentile of baseline body weight (54.5 kg) compared to the median (83.8 kg).
- Dalbavancin V2 was 49% higher at the 5th percentile of baseline albumin (1.7 g/dL) compared to the median (2.8 g/dL).
- Dalbavancin V3 was 21.4 lower at the 5th percentile of baseline body weight (54.5

kg) compared to the median (83.8 kg).
- Dalbavancin V3 was 33.9% lower at the 5th percentile of age (29 yr) compared to the median (56 yr).
- Dalbavancin A was 48% higher at the 5th percentile of baseline albumin (1.7 g/dL) compared to the median (2.8 g/dL).

10.1 CL

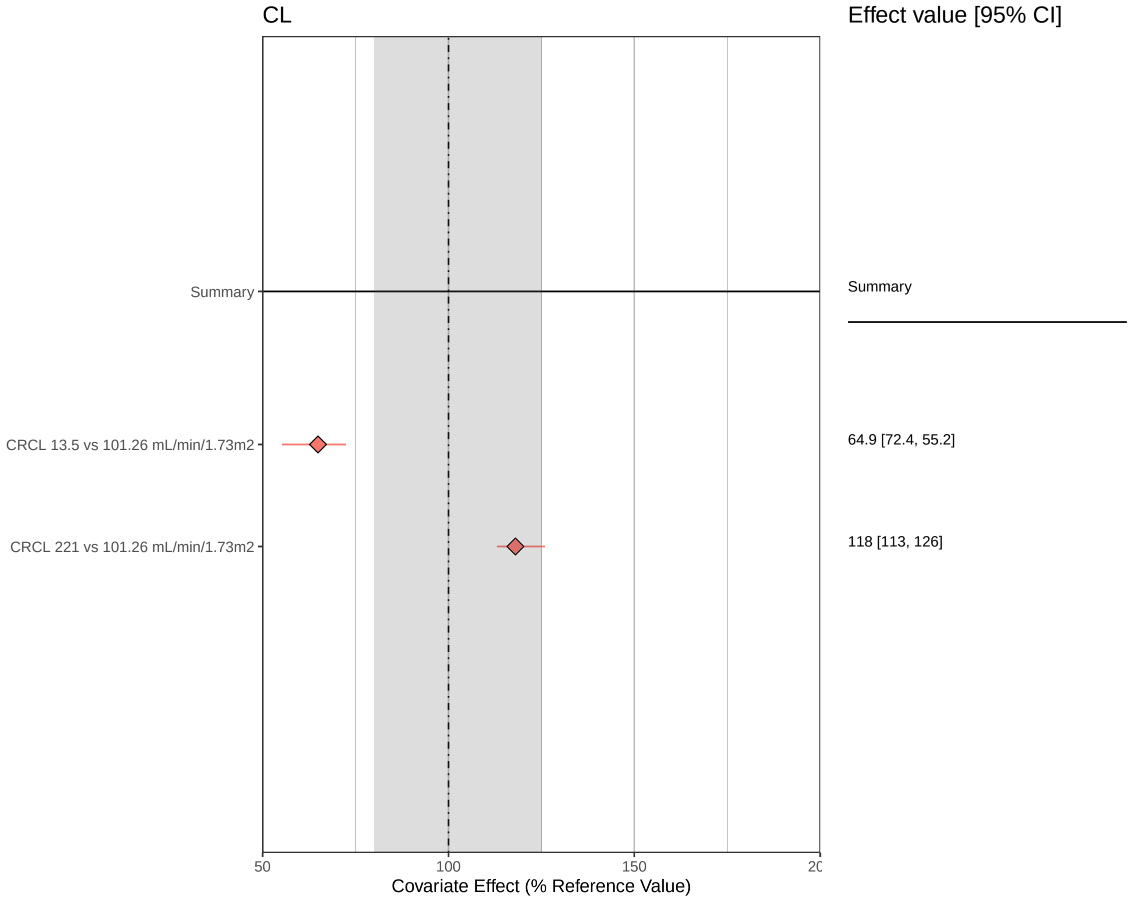

The shaded area corresponds to the interval [80, 125]. For continuous covariates, the 5th and 95th quantiles are compared to the median value.

10.2 V1

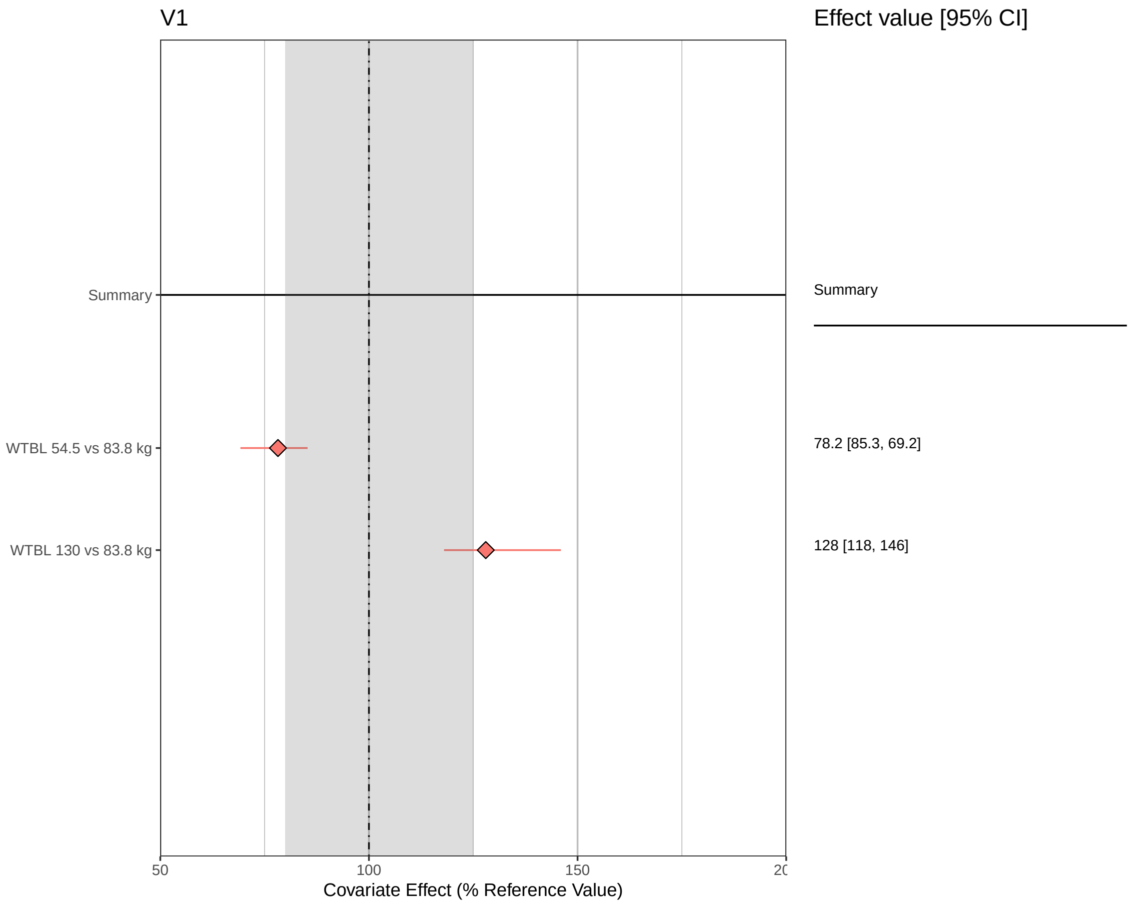

The shaded area corresponds to the interval [80, 125]. For continuous covariates, the 5th and 95th quantiles are compared to the median value.

10.3 V2

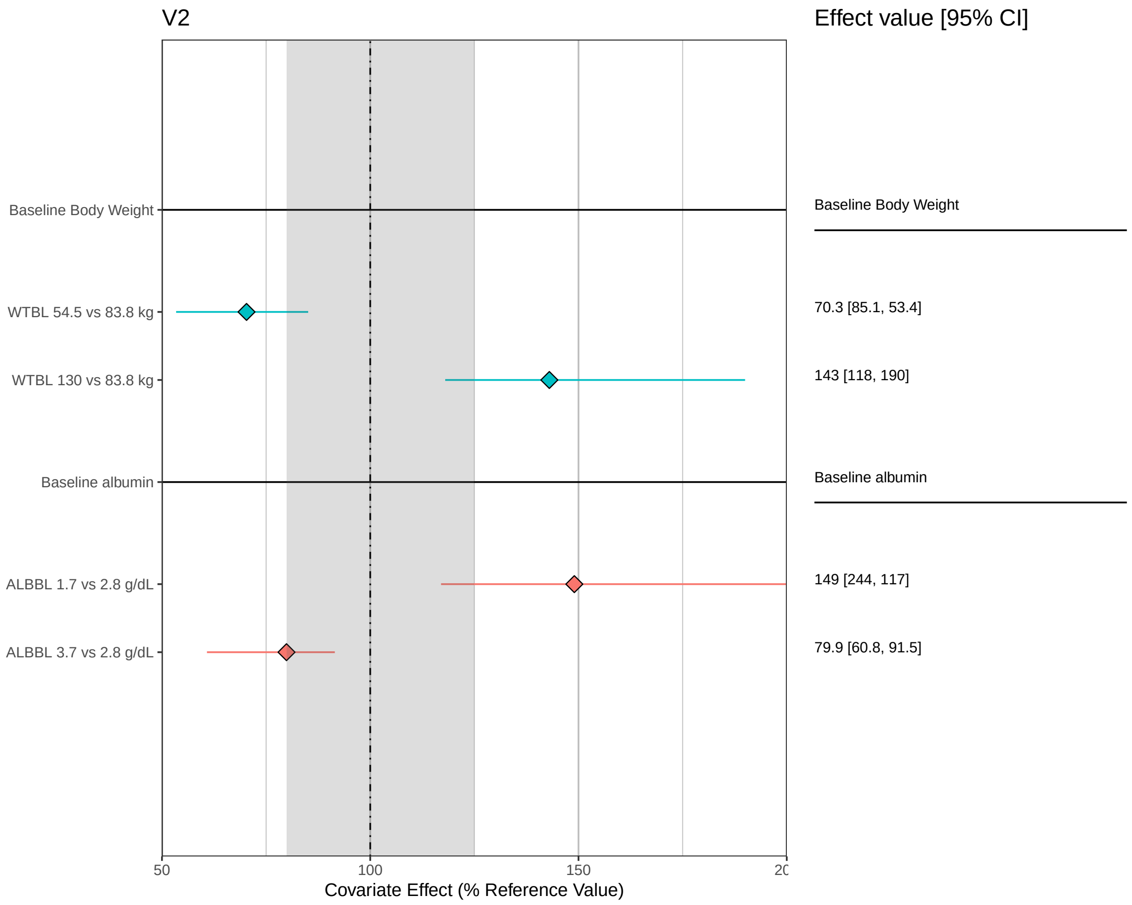

The shaded area corresponds to the interval [80, 125]. For continuous covariates, the 5th and 95th quantiles are compared to the median value.

10.4 V3

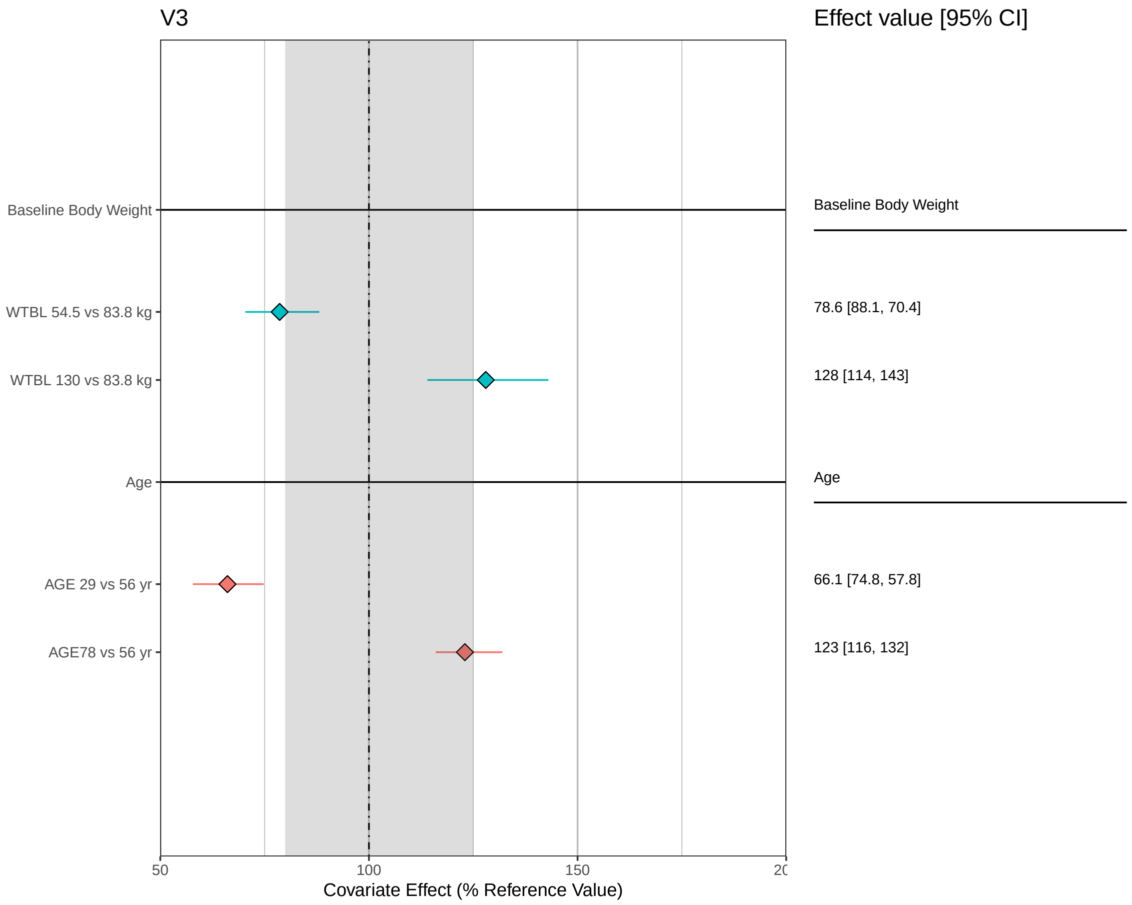

The shaded area corresponds to the interval [80, 125]. For continuous covariates, the 5th and 95th quantiles are compared to the median value.

10.5 A

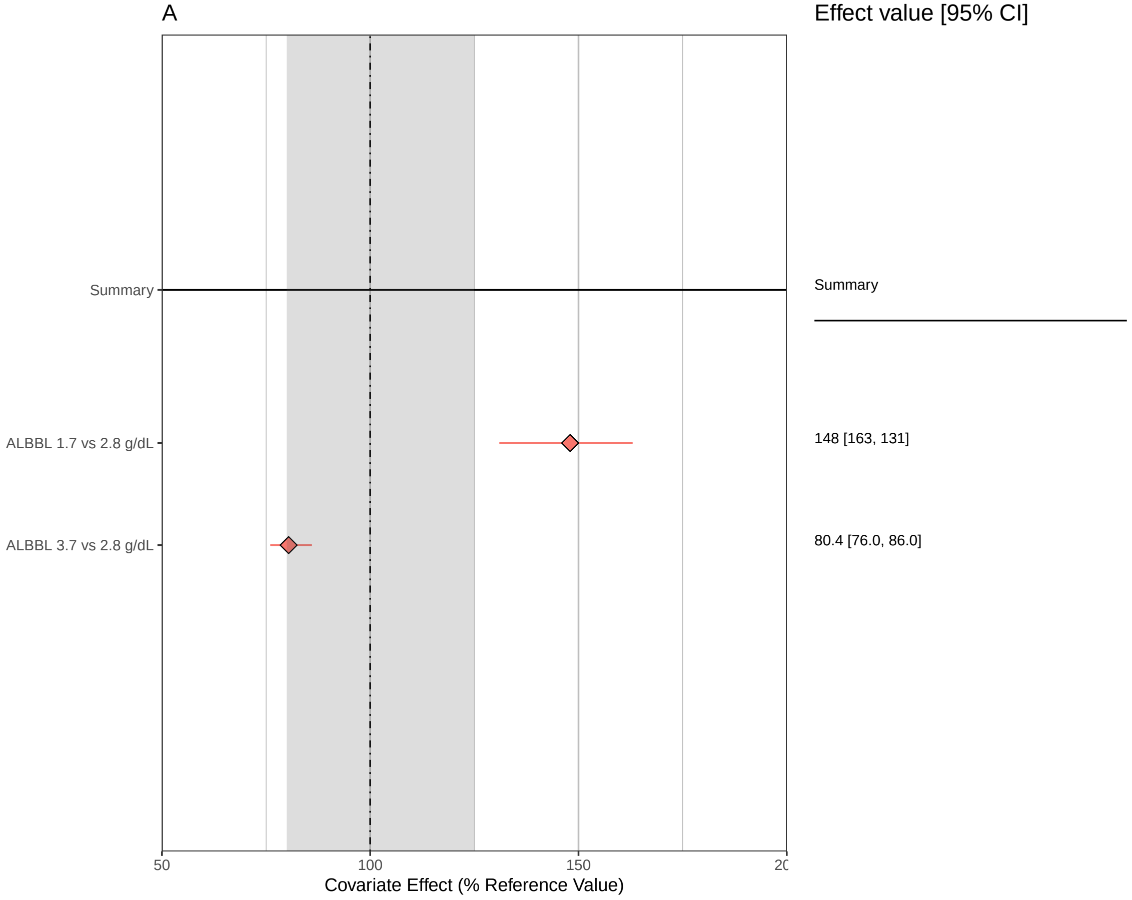

The shaded area corresponds to the interval [80, 125]. For continuous covariates, the 5th and 95th quantiles are compared to the median value.

11 VPC

> **Note**
>
> 1000 simulations of the popPK dataset were performed in Pumas (./programs/pumas/final/final-model.jl) and pcVPC generated with tidyvpc().

11.1 VPC Setup

11.2 pcVPC - Time After First Dose

11.2.1 Linear scale

Prediction-corrected Visual Predictive Check vs. time after first dose - Linear scale

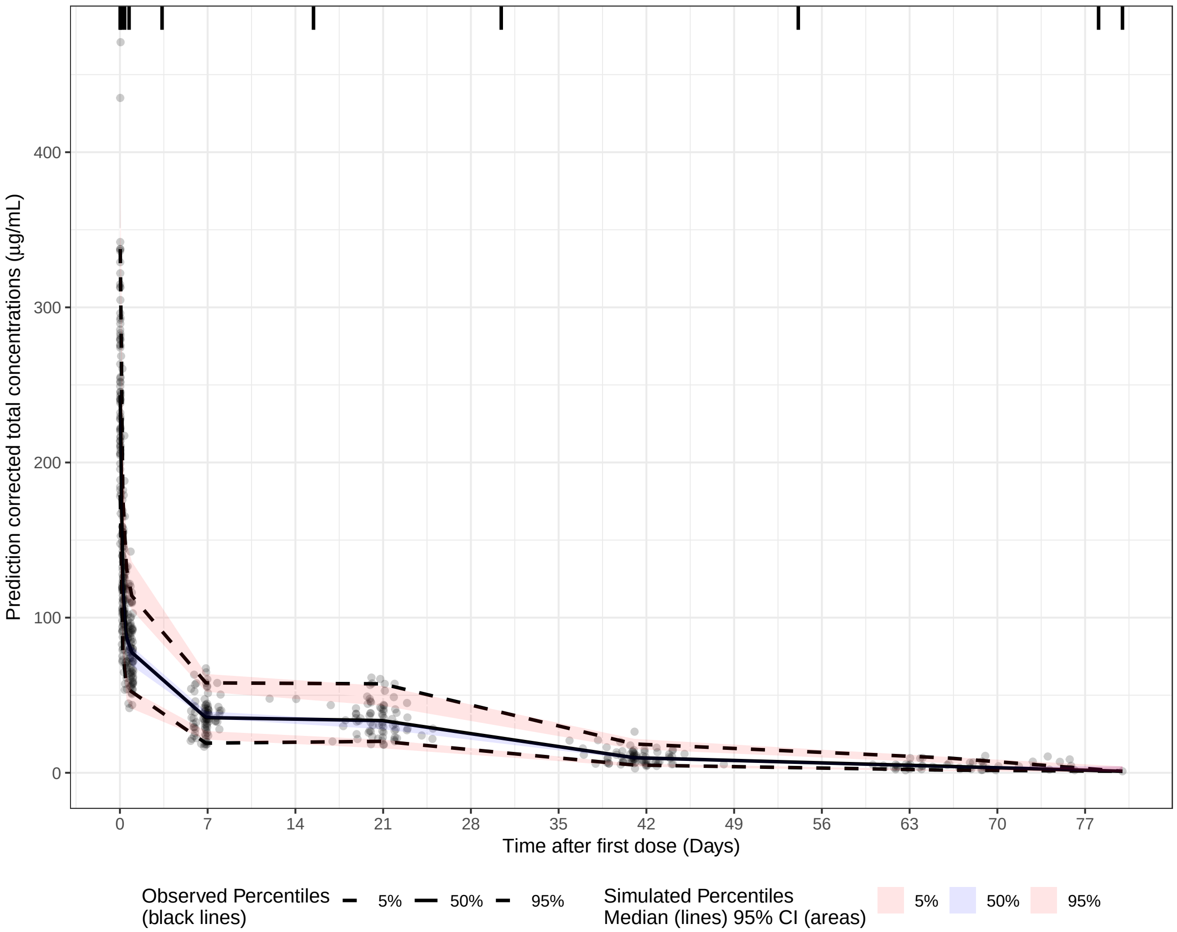

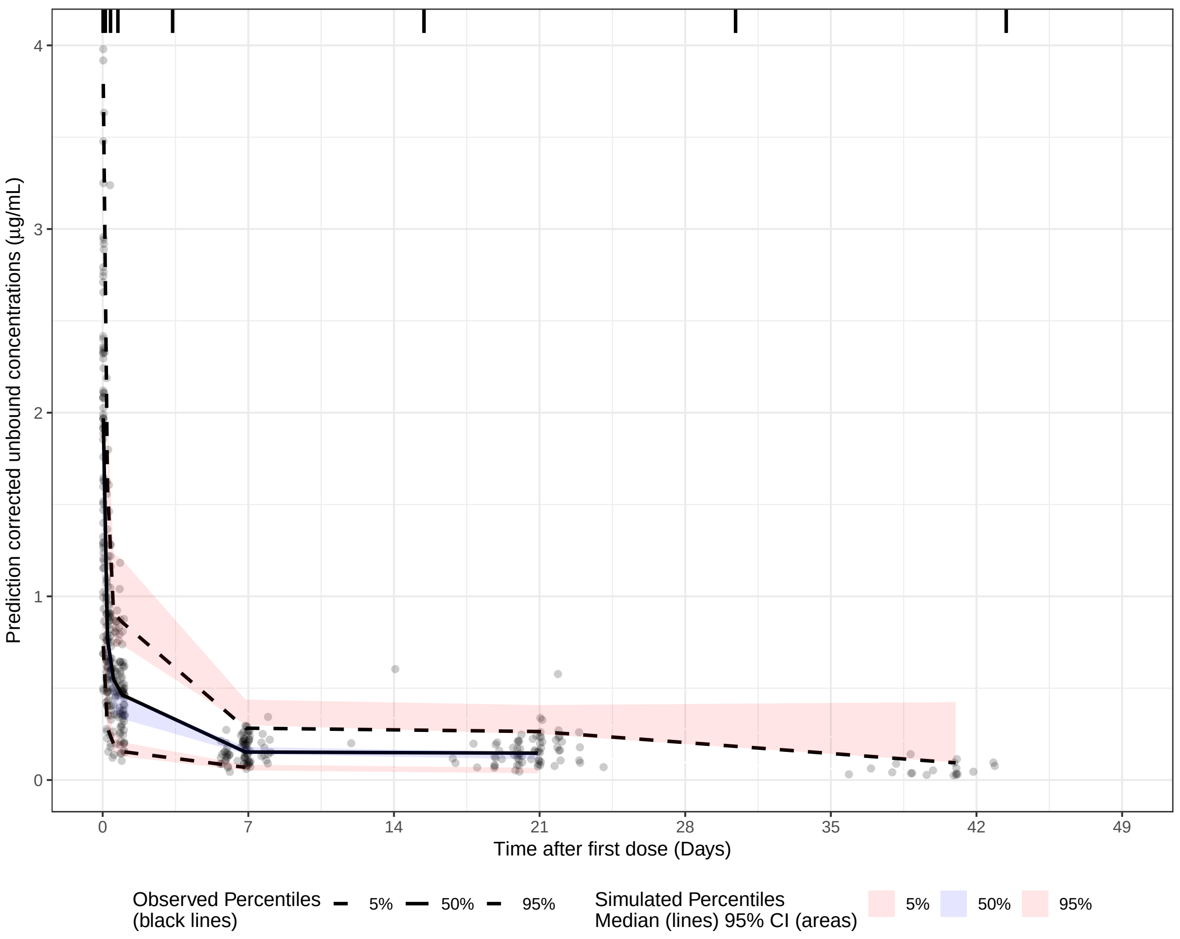

11.2.2 Conc. in Log scale

Prediction-corrected Visual Predictive Check vs. time after first dose - Concentrations in log scale

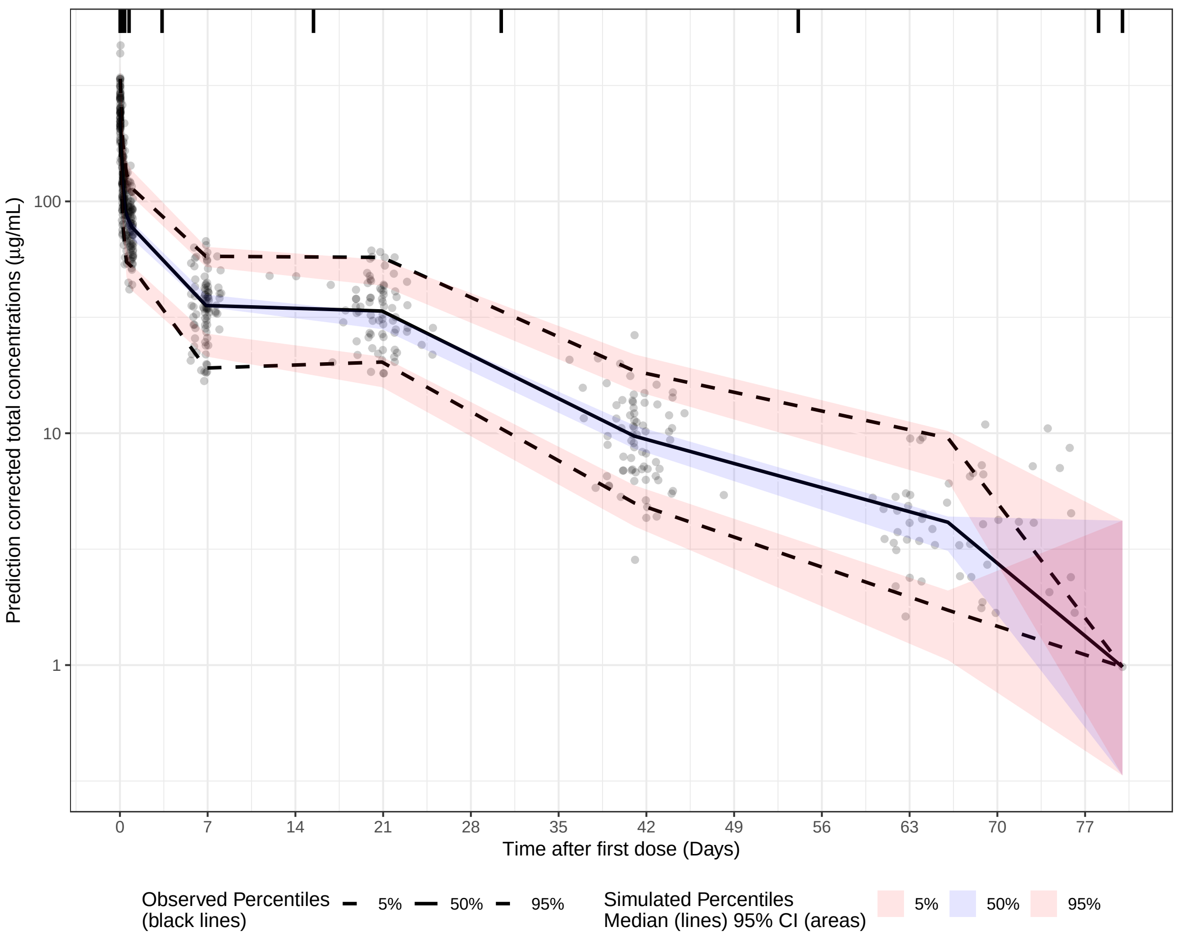
Prediction corrected total concentrations (µg/mL)
100
10
1
0
7
14
21
28
35
42
49
56
63
70
77
Time after first dose (Days)
Observed Percentiles
(black lines)
5%
50%
95%
Simulated Percentiles
Median (lines) 95% CI (areas)
5%
50%
95%

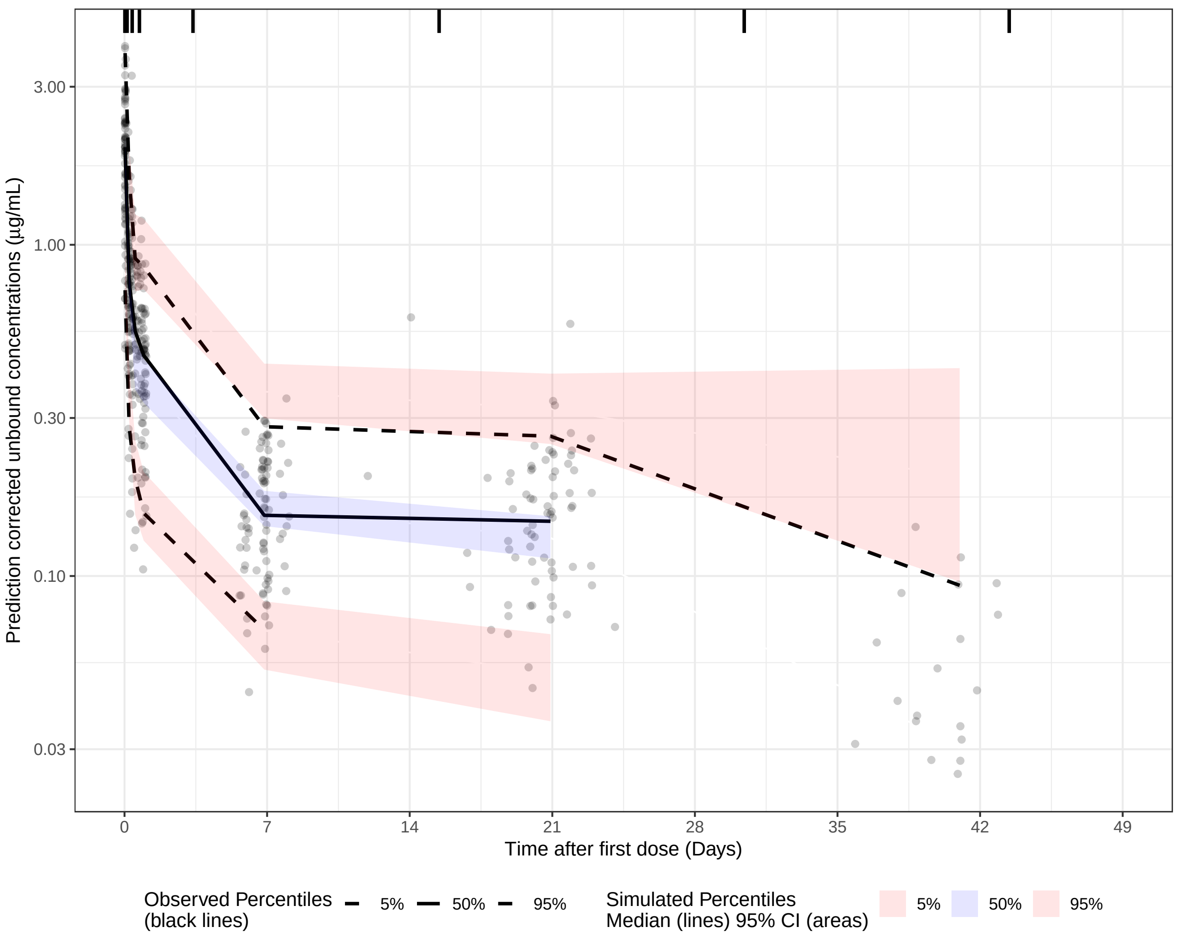

11.2.3 Time in Log scale

Prediction-corrected Visual Predictive Check vs. time after first dose - Time in log scale

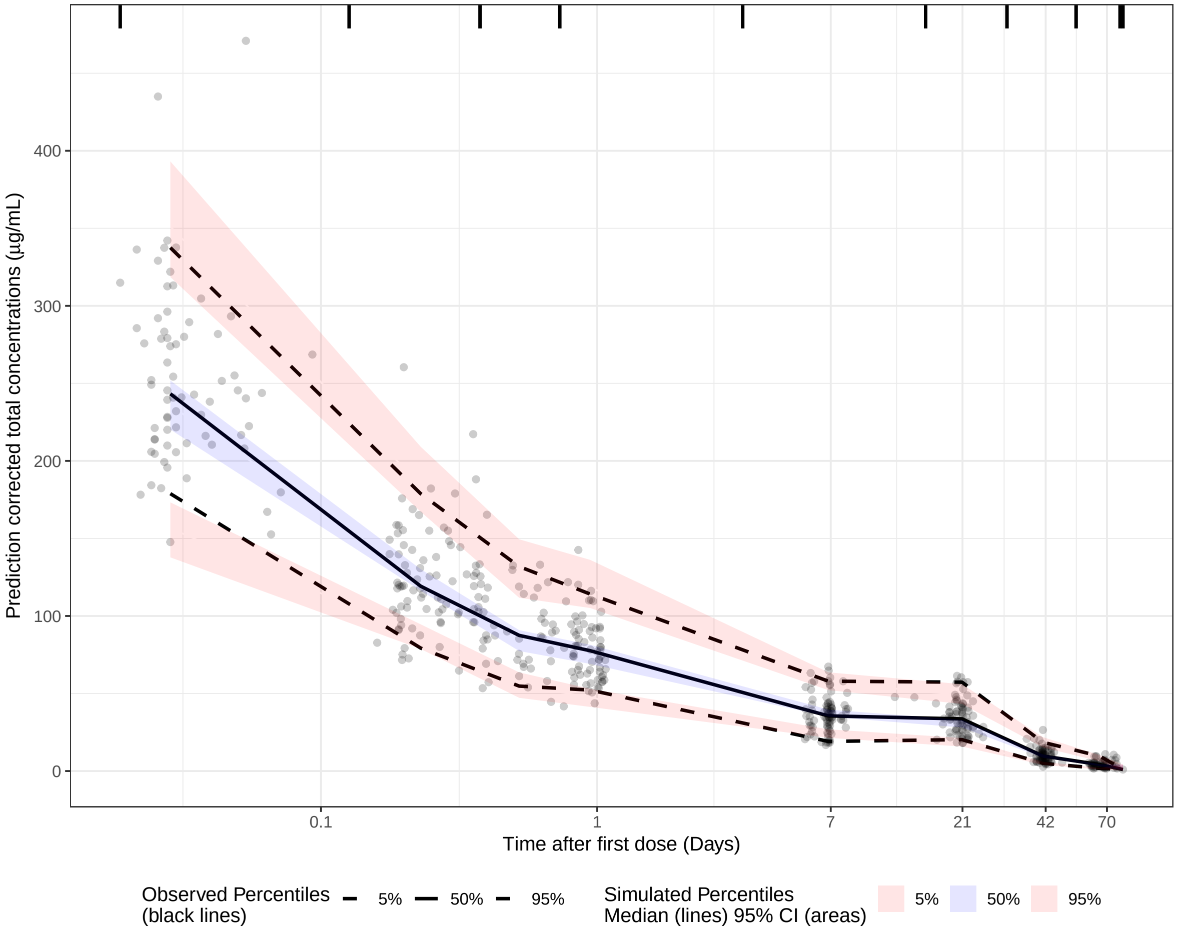
400
300
200
100
0
Prediction corrected total concentrations (µg/mL)
0.1
1
7
21
42
70
Time after first dose (Days)
Observed Percentiles (black lines)
5%
50%
95%
Simulated Percentiles Median (lines) 95% CI (areas)
5%
50%
95%

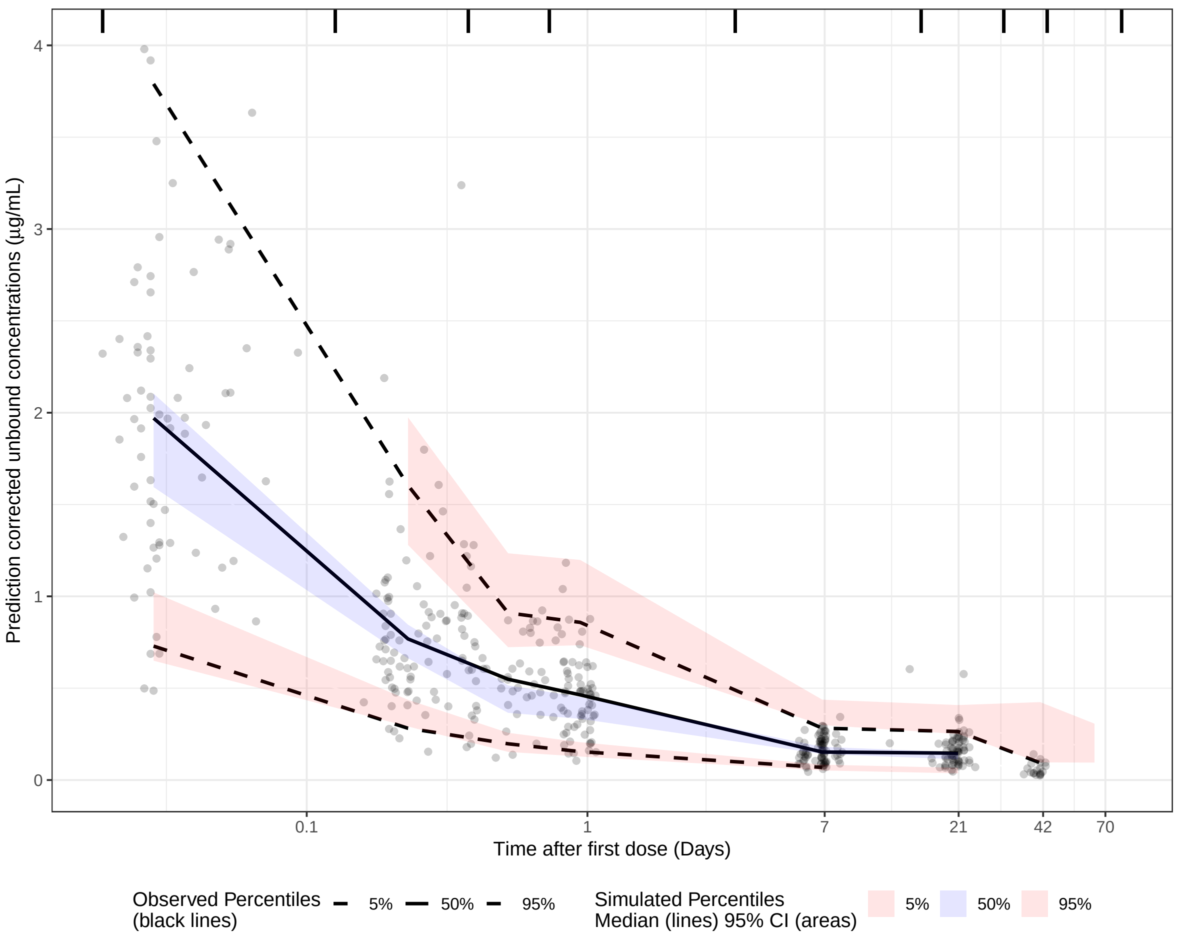

11.2.4 Conc. and Time in Log scale

Prediction-corrected Visual Predictive Check vs. time after first dose - Time and Concentrations in log scale

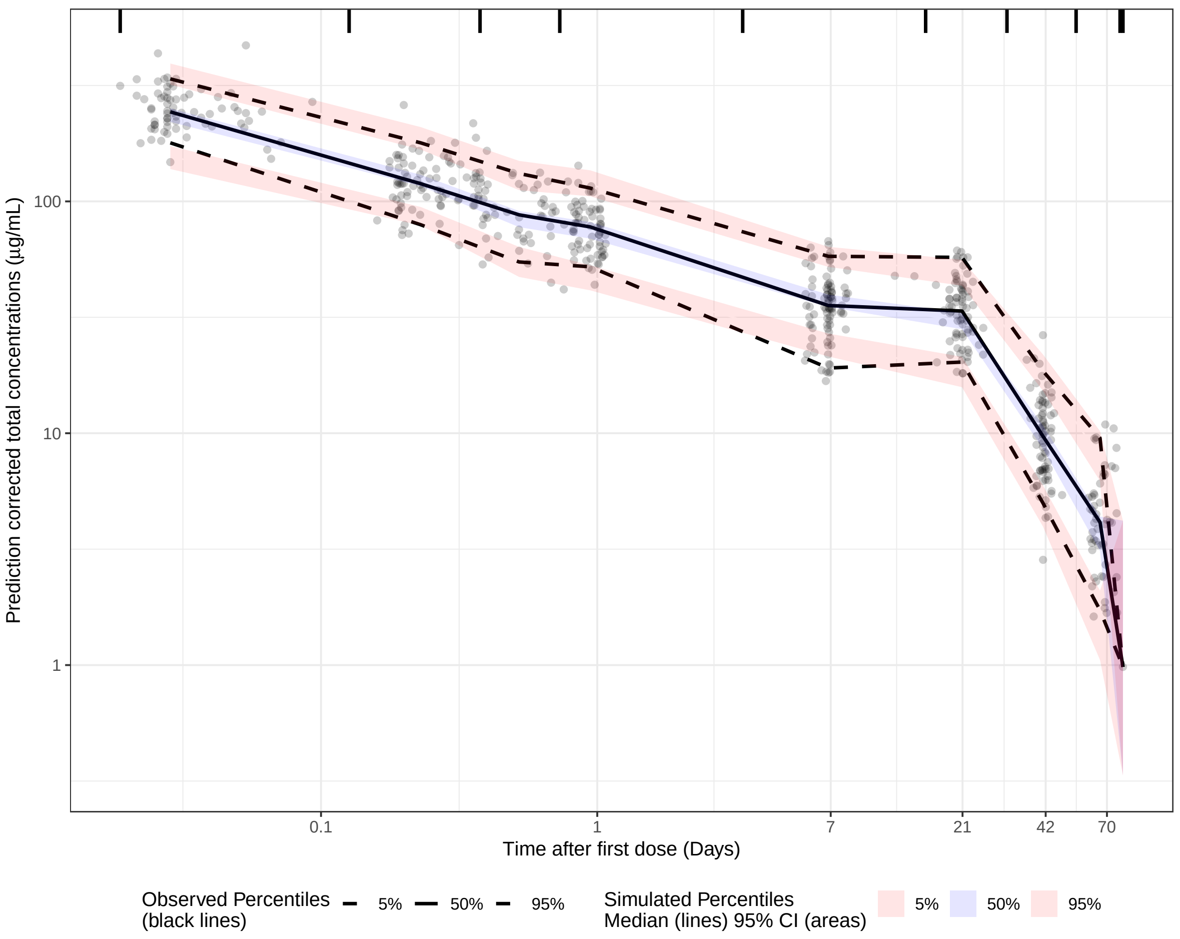
Prediction corrected total concentrations (µg/mL)
100
10
1
0.1
1
7
21
42
70
Time after first dose (Days)
Observed Percentiles (black lines)
5%
50%
95%
Simulated Percentiles Median (lines) 95% CI (areas)
5%
50%
95%

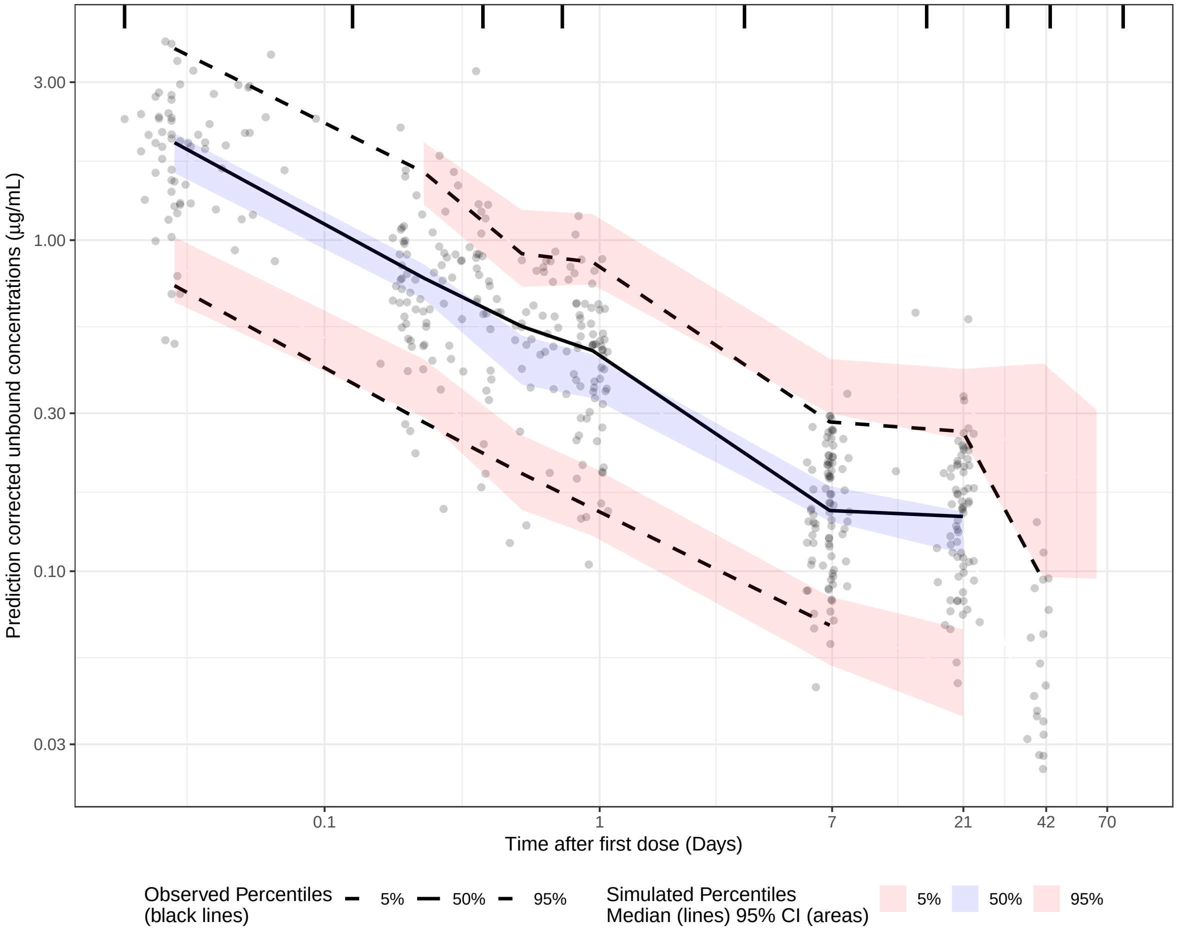

11.2.5 %BLOQ unbound

Visual Predictive Check of BLOQ percentage vs. time after first dose for unbound concentrations

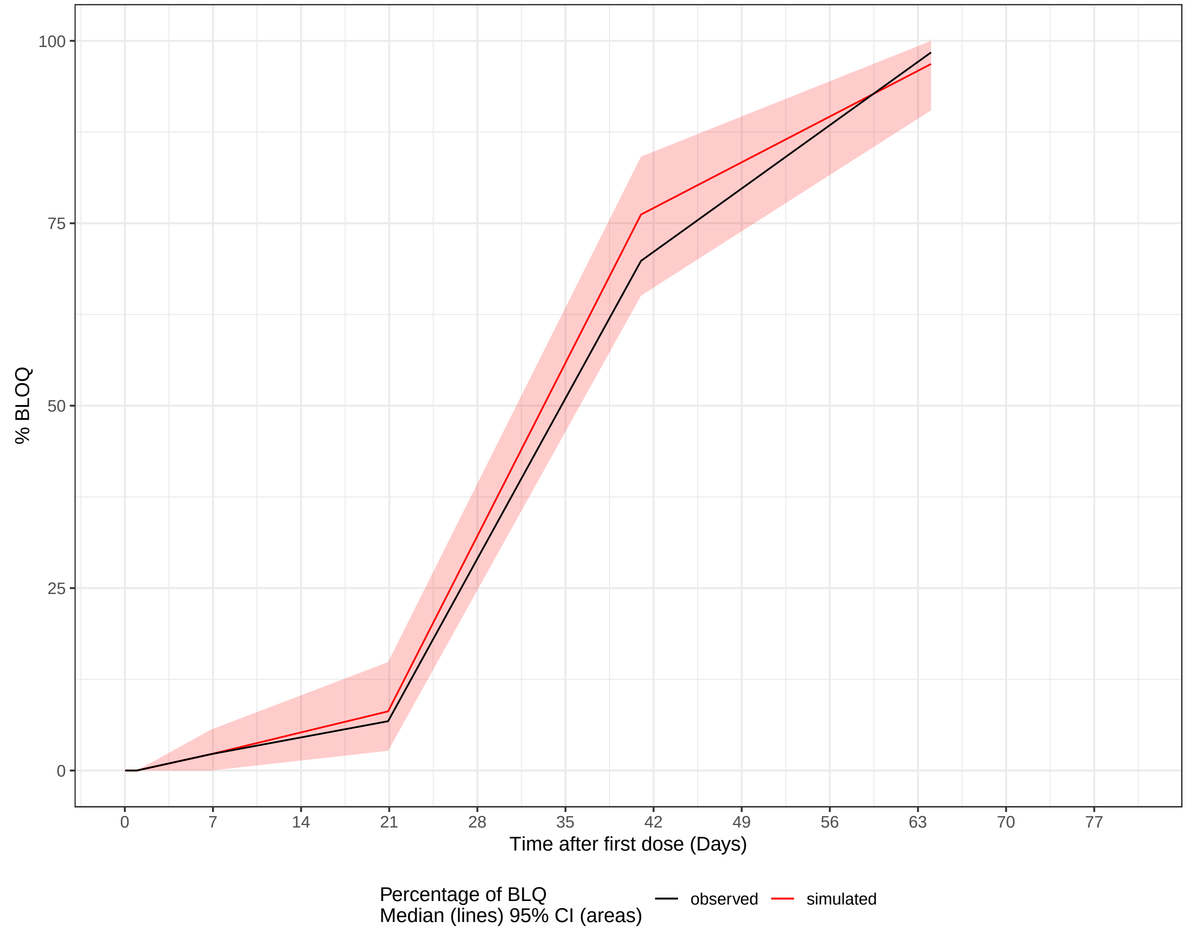

11.3 pcVPC - Time From Previous Dose

11.3.1 Linear scale

Prediction-corrected Visual Predictive Check vs. time from previous dose - Linear scale

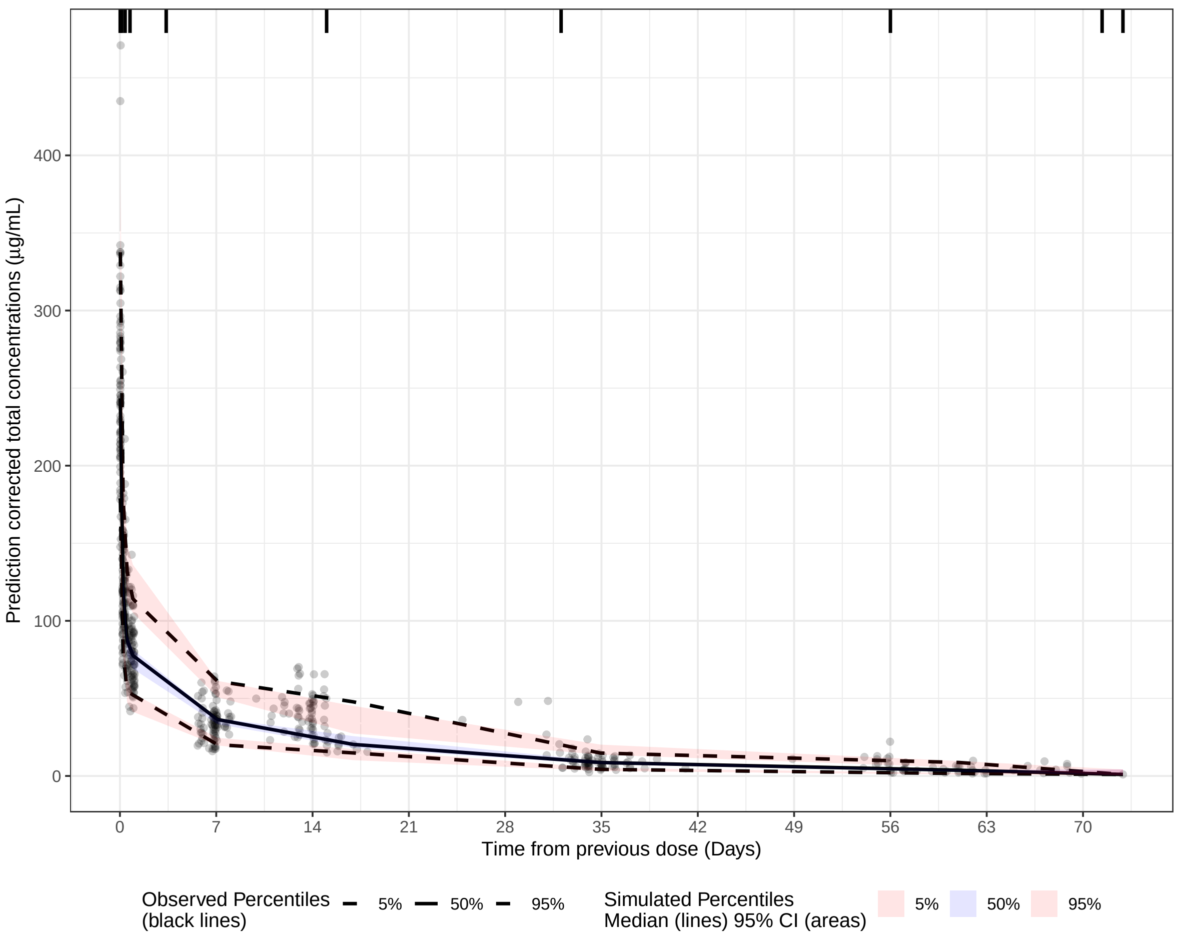
Prediction corrected total concentrations (µg/mL)
0
100
200
300
400
Time from previous dose (Days)
0
7
14
21
28
35
42
49
56
63
70
Observed Percentiles
(black lines)
5%
50%
95%
Simulated Percentiles
Median (lines) 95% CI (areas)
5%
50%
95%

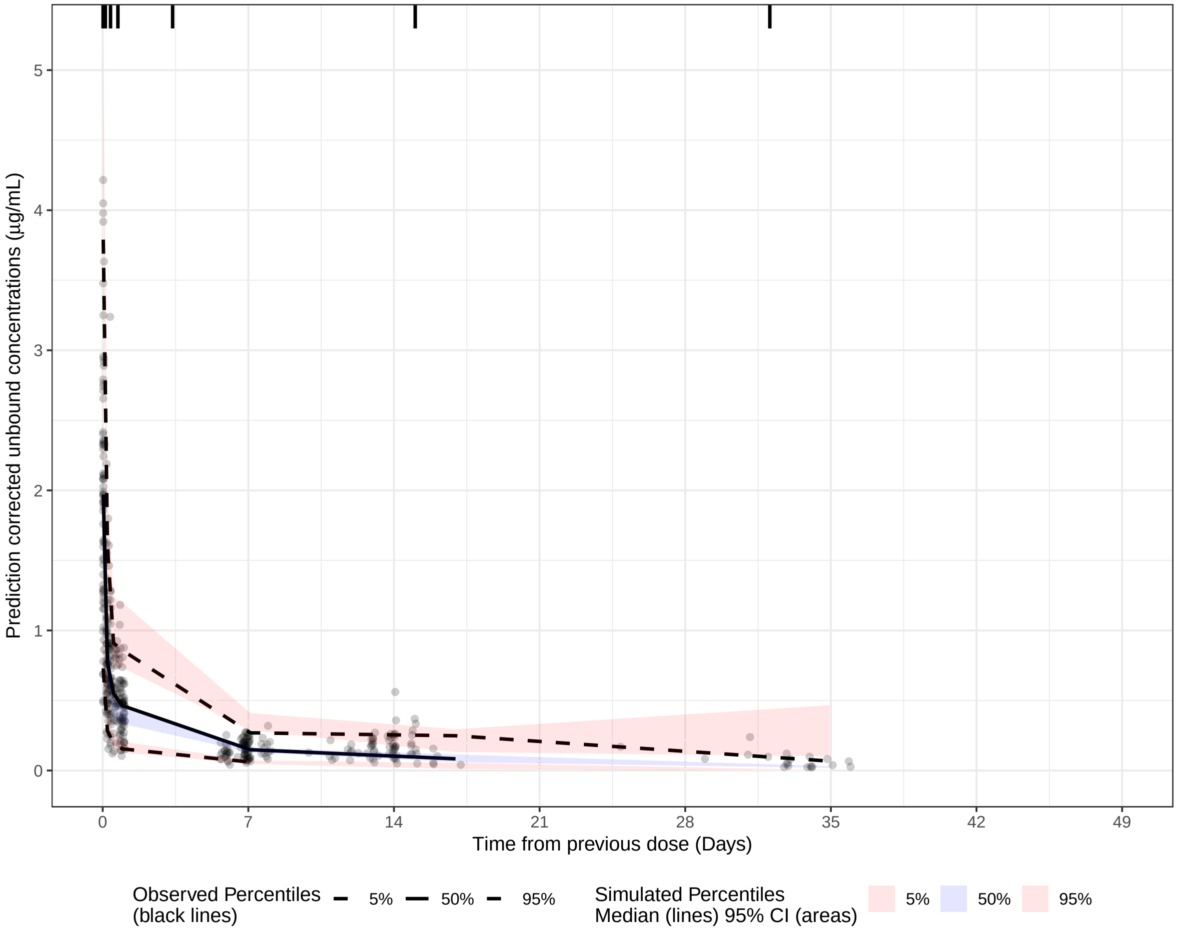

11.3.2 Conc. in Log scale

Prediction-corrected Visual Predictive Check vs. time from previous dose - Concentrations in log scale

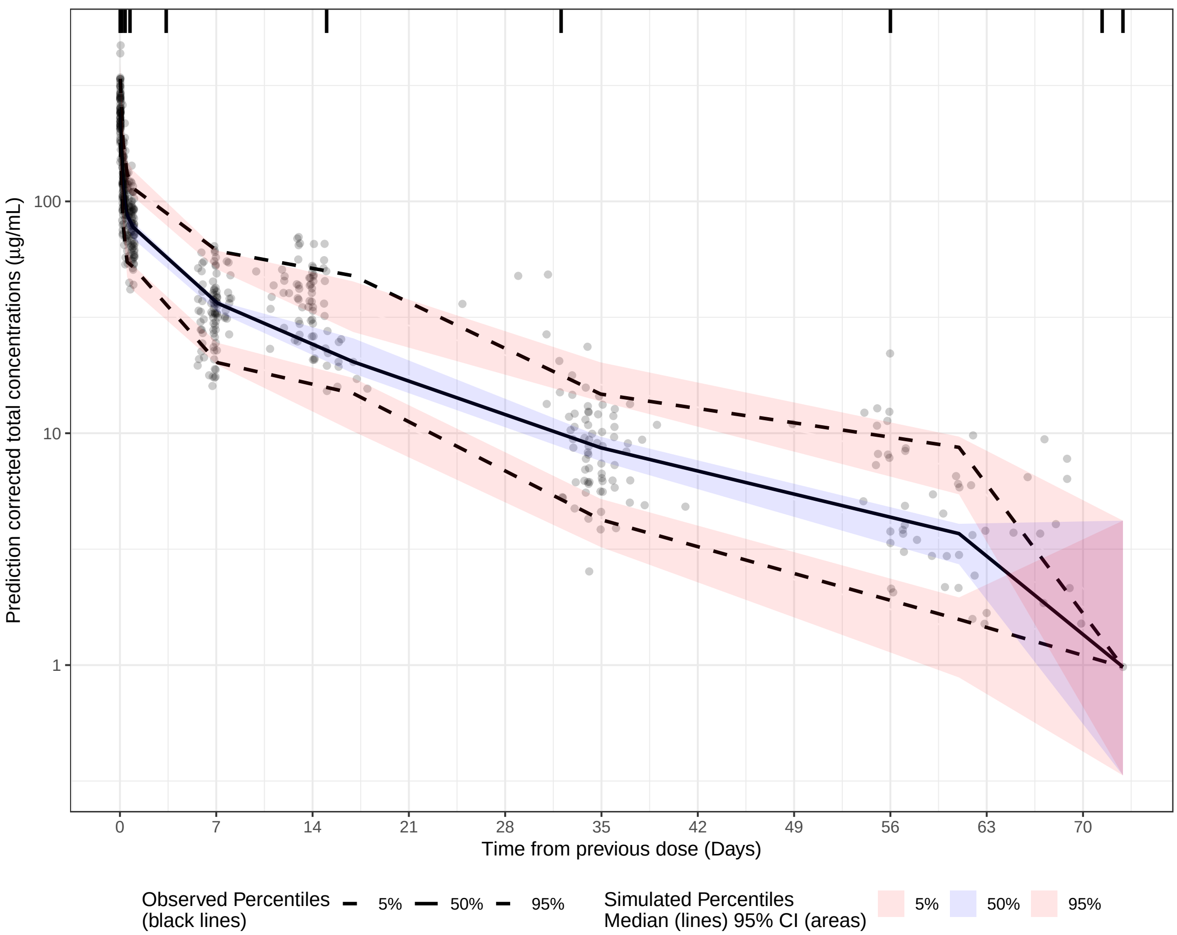
Prediction corrected total concentrations (µg/mL)
100
10
1
0
7
14
21
28
35
42
49
56
63
70
Time from previous dose (Days)
Observed Percentiles (black lines)
5%
50%
95%
Simulated Percentiles Median (lines) 95% CI (areas)
5%
50%
95%

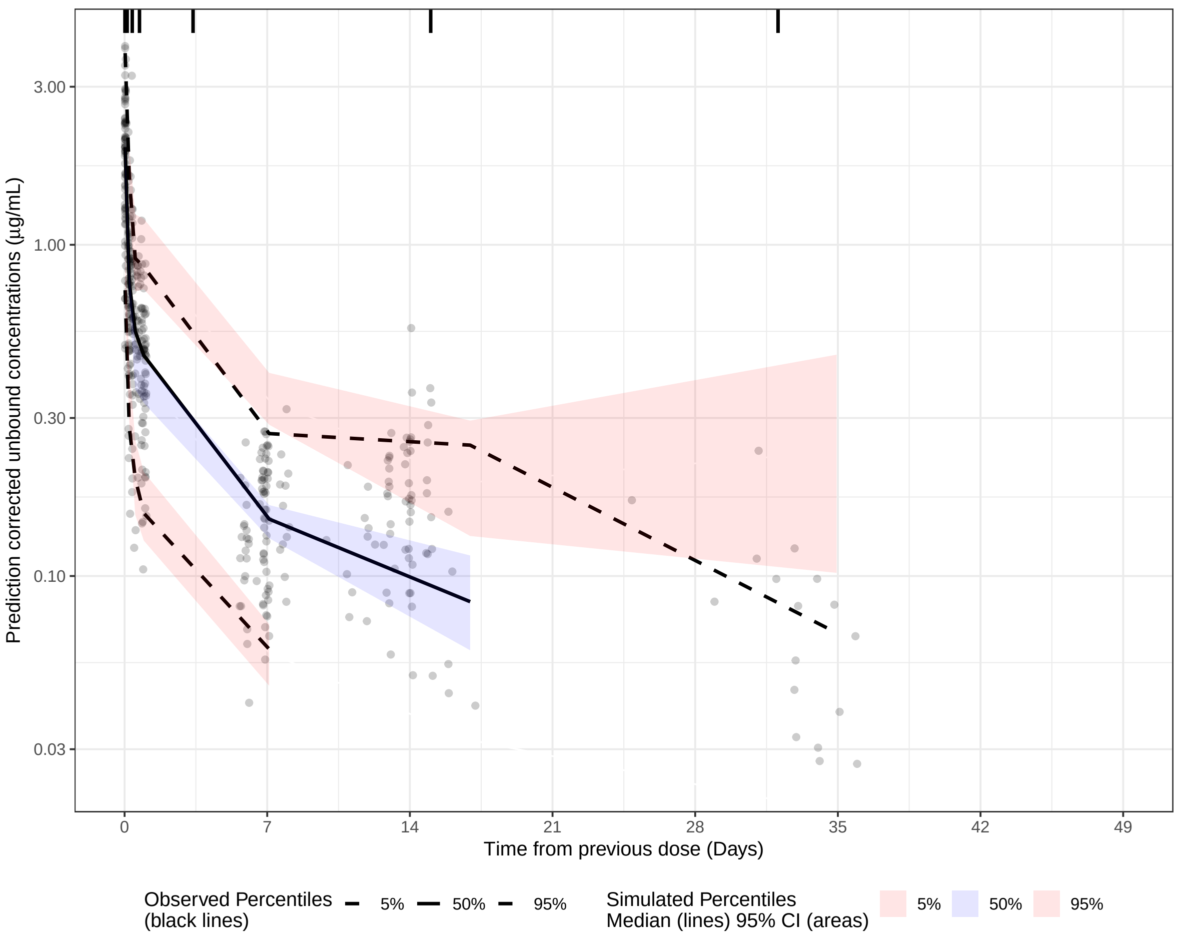

11.3.3 Time in Log scale

Prediction-corrected Visual Predictive Check vs. time from previous dose - Time in log scale

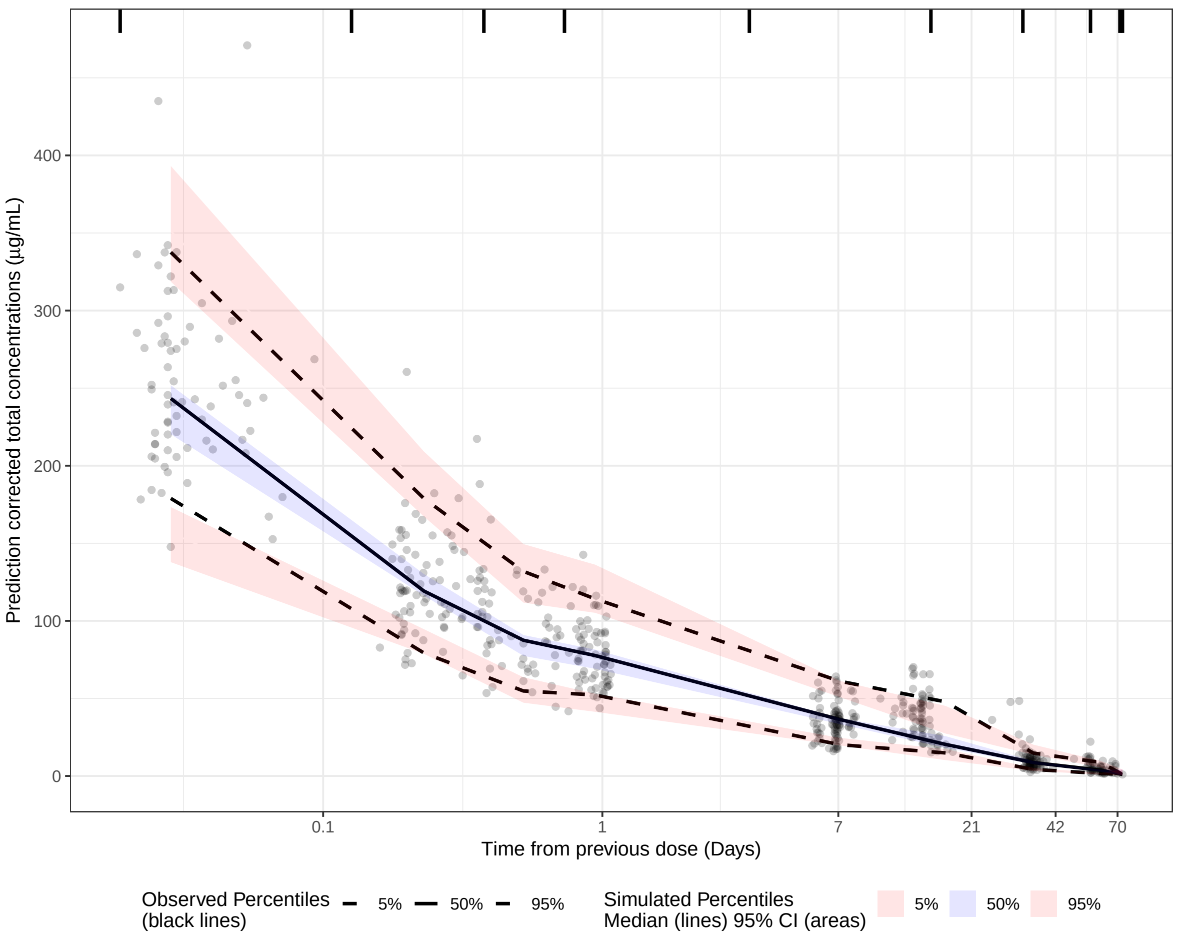
Prediction corrected total concentrations (µg/mL)
400
300
200
100
0
0.1
1
7
21
42
70
Time from previous dose (Days)
Observed Percentiles (black lines)
5%
50%
95%
Simulated Percentiles Median (lines) 95% CI (areas)
5%
50%
95%

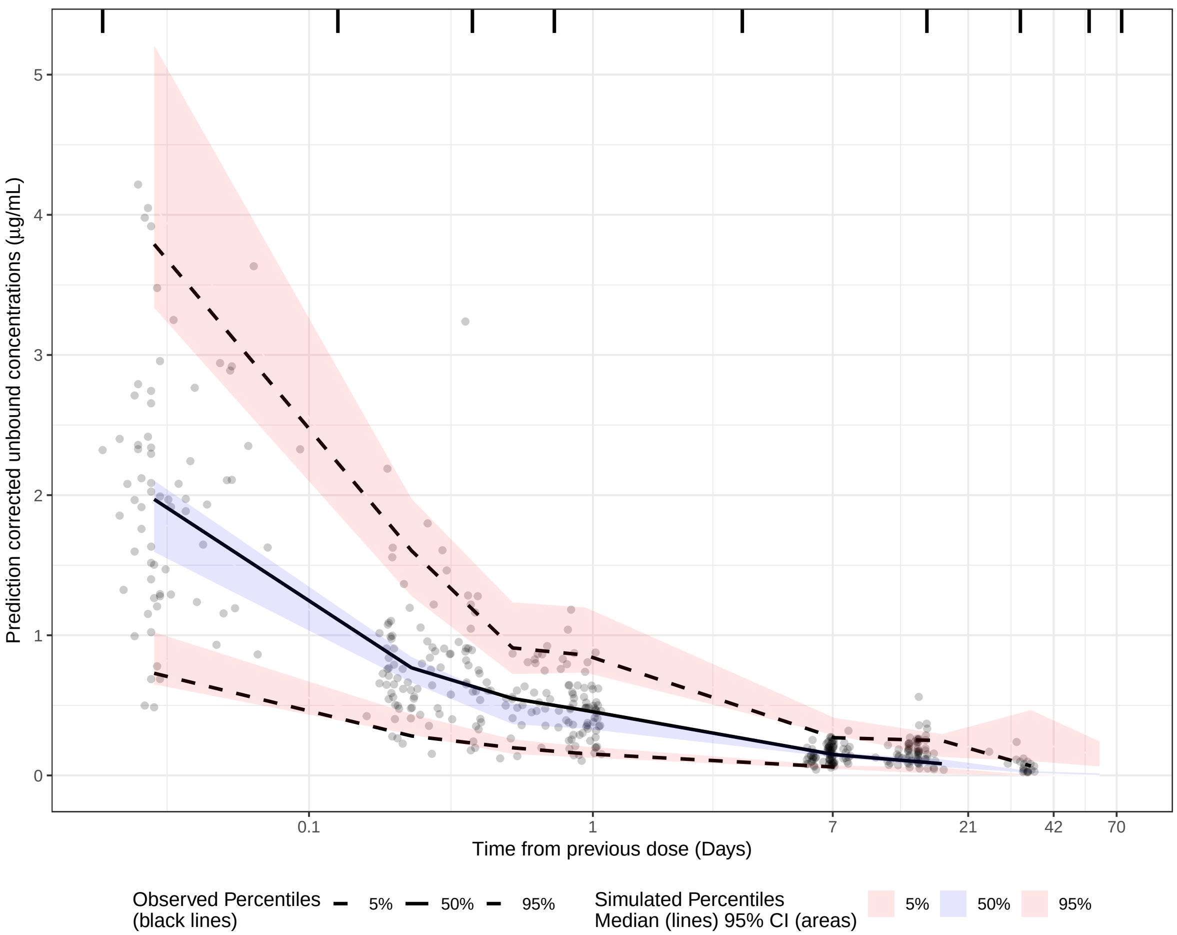

11.3.4 Conc. and Time in Log scale

Prediction-corrected Visual Predictive Check vs. time from previous dose - Time and Concentrations in log scale

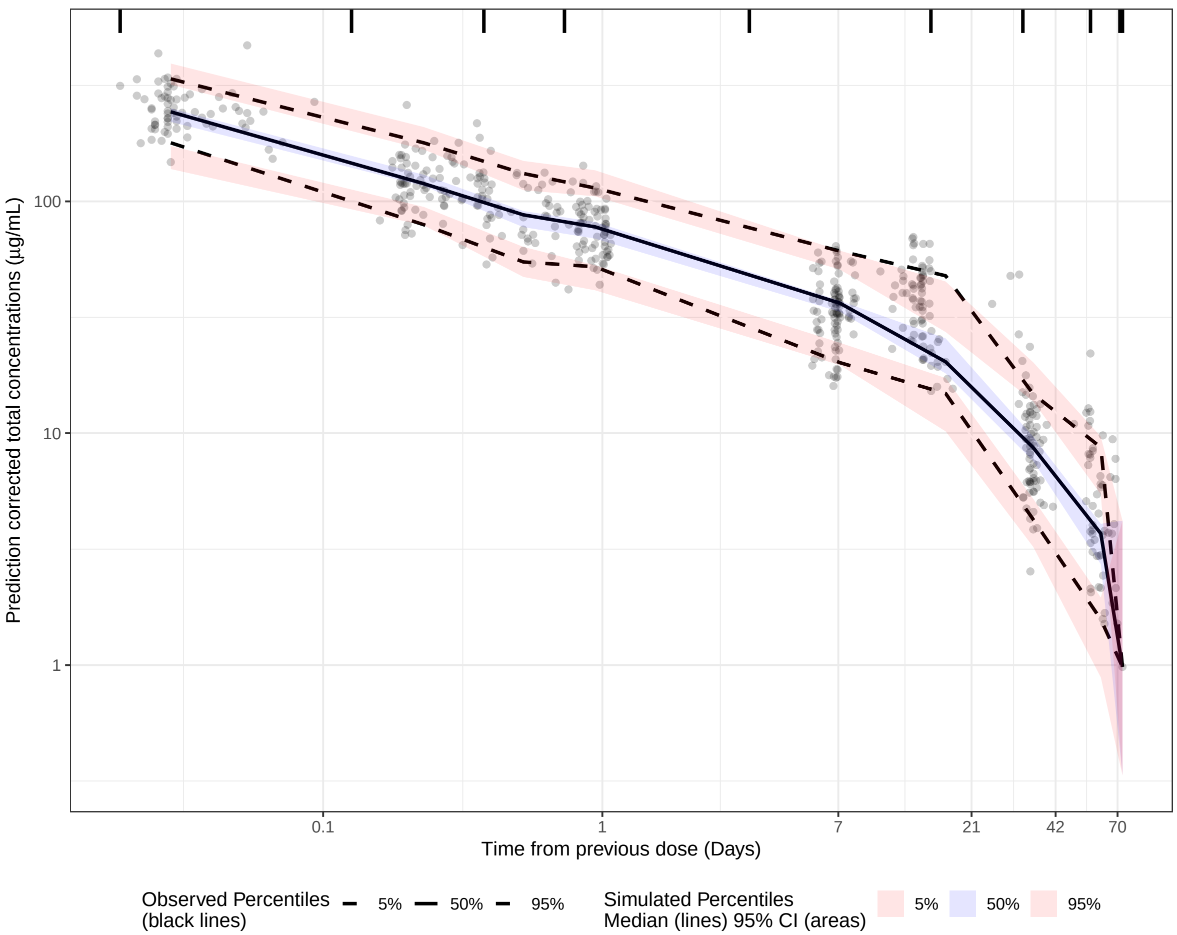
Prediction corrected total concentrations (µg/mL)
100
10
1
0.1
1
7
21
42
70
Time from previous dose (Days)
Observed Percentiles
(black lines)
5%
50%
95%
Simulated Percentiles
Median (lines) 95% CI (areas)
5%
50%
95%

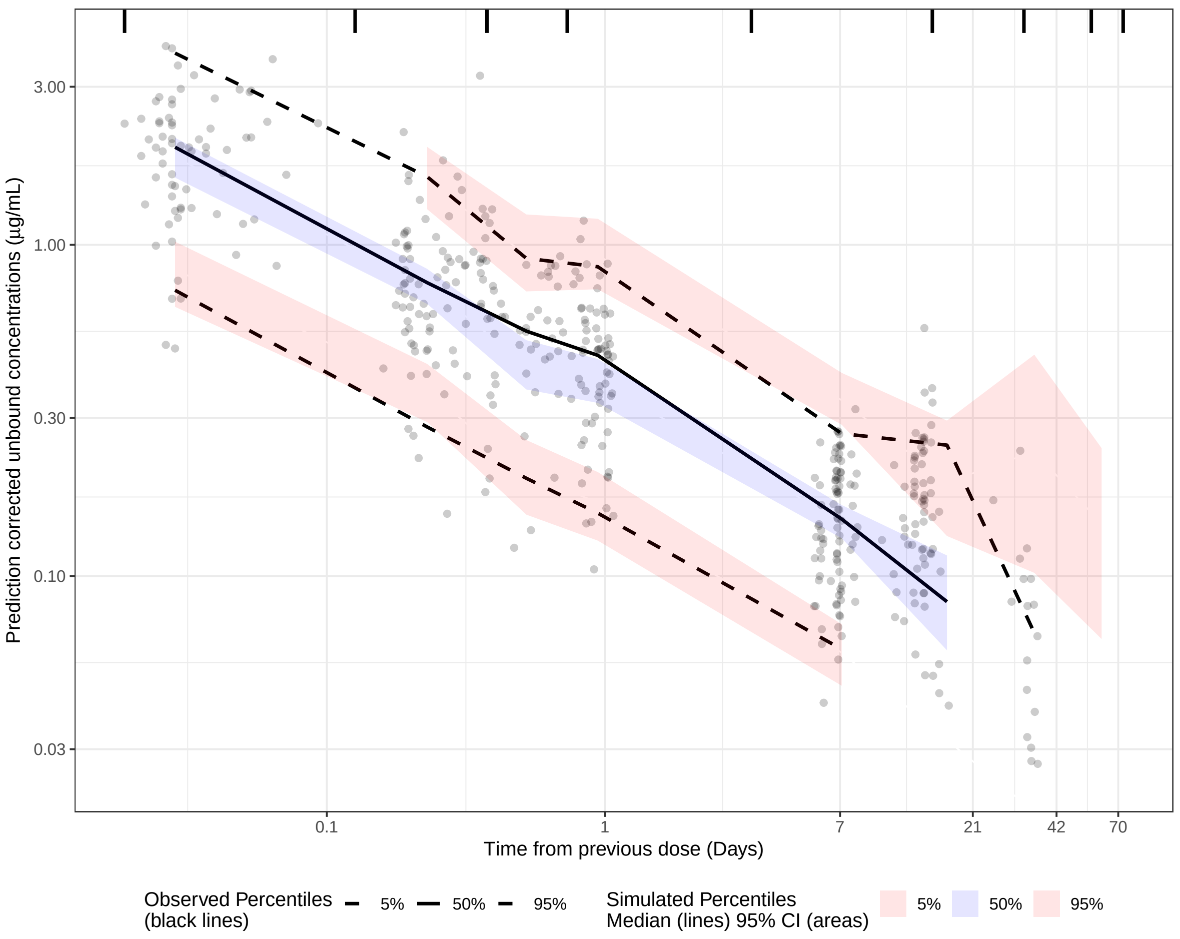

11.3.5 %BLOQ unbound

Visual Predictive Check of BLOQ percentage vs. time from previous dose for unbound concentrations

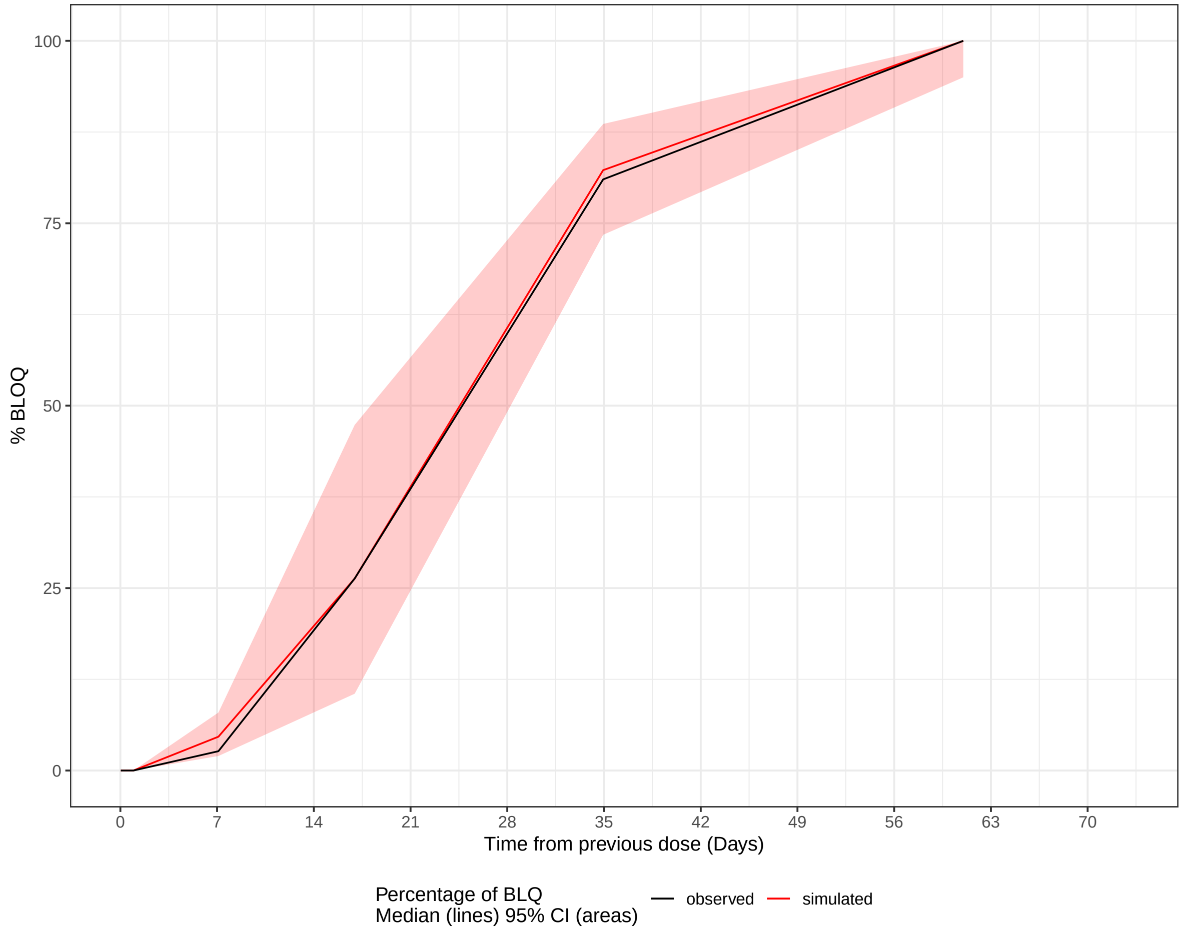

:::

12 REPRODUCIBILITY

The program code for this analysis is in the following file:

/mnt/data/code/SSC/Emmes/Emmes-Dalbavancin-DOTS/05-final-model.qmd

The packages used in this analysis are listed below:

```
- Session info ---------------------------------------------------------------
 setting  value
 version  R version 4.2.3 (2023-03-15)
 os       Ubuntu 20.04.6 LTS
 system   x86_64, linux-gnu
```

```
 ui       X11
 language (EN)
 collate  en_US.UTF-8
 ctype    en_US.UTF-8
 tz       UTC
 date     2024-06-07
 pandoc   2.19.2 @ /usr/lib/rstudio-server/bin/quarto/bin/tools/ (via rmarkdown)

- Packages -------------------------------------------------------------------
 ! package           * version  date (UTC) lib source
 P arsenal           * 3.6.3    2021-06-04 [?] CRAN (R 4.2.3)
 P askpass             1.2.0    2023-09-03 [?] CRAN (R 4.2.3)
 P assertthat          0.2.1    2019-03-21 [?] CRAN (R 4.2.3)
 P backports           1.4.1    2021-12-13 [?] CRAN (R 4.2.3)
 P base64enc           0.1-3    2015-07-28 [?] CRAN (R 4.2.3)
 P broom.helpers       1.14.0   2023-08-07 [?] CRAN (R 4.2.3)
 P checkmate           2.3.1    2023-12-04 [?] CRAN (R 4.2.3)
 P class               7.3-21   2023-01-23 [?] CRAN (R 4.2.3)
 P classInt            0.4-10   2023-09-05 [?] CRAN (R 4.2.3)
 P cli                 3.6.1    2023-03-23 [?] CRAN (R 4.2.3)
 P clisymbols          1.2.0    2017-05-21 [?] CRAN (R 4.2.3)
 P codetools           0.2-19   2023-02-01 [?] CRAN (R 4.2.3)
 P colorspace          2.1-0    2023-01-23 [?] CRAN (R 4.2.3)
 P crayon              1.5.2    2022-09-29 [?] CRAN (R 4.2.3)
 P crul                1.4.2    2024-04-09 [?] CRAN (R 4.2.3)
 P curl                5.2.0    2023-12-08 [?] CRAN (R 4.2.3)
 P data.table          1.14.8   2023-02-17 [?] CRAN (R 4.2.3)
 P digest              0.6.33   2023-07-07 [?] CRAN (R 4.2.3)
 P dplyr             * 1.1.4    2023-11-17 [?] CRAN (R 4.2.3)
 P e1071               1.7-14   2023-12-06 [?] CRAN (R 4.2.3)
 P ellipsis            0.3.2    2021-04-29 [?] CRAN (R 4.2.3)
 P evaluate            0.23     2023-11-01 [?] CRAN (R 4.2.3)
 P fansi               1.0.6    2023-12-08 [?] CRAN (R 4.2.3)
 P farver              2.1.1    2022-07-06 [?] CRAN (R 4.2.3)
 P fastmap             1.1.1    2023-02-24 [?] CRAN (R 4.2.3)
 P flextable         * 0.9.6    2024-05-05 [?] CRAN (R 4.2.3)
 P fontBitstreamVera   0.1.1    2017-02-01 [?] CRAN (R 4.2.3)
 P fontLiberation      0.1.0    2016-10-15 [?] CRAN (R 4.2.3)
 P fontquiver          0.2.1    2017-02-01 [?] CRAN (R 4.2.3)
 P forcats           * 1.0.0    2023-01-29 [?] CRAN (R 4.2.3)
 P fs                  1.6.3    2023-07-20 [?] CRAN (R 4.2.3)
 P gdtools             0.3.7    2024-03-05 [?] CRAN (R 4.2.3)
 P generics            0.1.3    2022-07-05 [?] CRAN (R 4.2.3)
```

```
P gfonts           0.2.0    2023-01-08 [?] CRAN (R 4.2.3)
P GGally         * 2.2.0    2023-11-22 [?] CRAN (R 4.2.3)
P ggforce        * 0.4.1    2022-10-04 [?] CRAN (R 4.2.3)
P ggplot2        * 3.4.4    2023-10-12 [?] CRAN (R 4.2.3)
P ggstats          0.5.1    2023-11-21 [?] CRAN (R 4.2.3)
P glue             1.6.2    2022-02-24 [?] CRAN (R 4.2.3)
P gridExtra      * 2.3      2017-09-09 [?] CRAN (R 4.2.3)
P gt             * 0.10.1   2024-01-17 [?] CRAN (R 4.2.3)
P gtable           0.3.4    2023-08-21 [?] CRAN (R 4.2.3)
P gtsummary      * 1.7.2    2023-07-15 [?] CRAN (R 4.2.3)
P haven          * 2.5.4    2023-11-30 [?] CRAN (R 4.2.3)
P here             1.0.1    2020-12-13 [?] CRAN (R 4.2.3)
P hms            * 1.1.3    2023-03-21 [?] CRAN (R 4.2.3)
P htmltools        0.5.7    2023-11-03 [?] CRAN (R 4.2.3)
P httpcode         0.3.0    2020-04-10 [?] CRAN (R 4.2.3)
P httpuv           1.6.12   2023-10-23 [?] CRAN (R 4.2.3)
P janitor        * 2.2.0    2023-02-02 [?] CRAN (R 4.2.3)
P jsonlite         1.8.7    2023-06-29 [?] CRAN (R 4.2.3)
P kableExtra     * 1.4.0    2024-01-24 [?] CRAN (R 4.2.3)
P KernSmooth       2.23-20  2021-05-03 [?] CRAN (R 4.2.3)
P knitr            1.45     2023-10-30 [?] CRAN (R 4.2.3)
P labeling         0.4.3    2023-08-29 [?] CRAN (R 4.2.3)
P labelled       * 2.12.0   2023-06-21 [?] CRAN (R 4.2.3)
P later            1.3.1    2023-05-02 [?] CRAN (R 4.2.3)
P lattice          0.20-45  2021-09-22 [?] CRAN (R 4.2.3)
P lifecycle        1.0.4    2023-11-07 [?] CRAN (R 4.2.3)
P lubridate      * 1.9.3    2023-09-27 [?] CRAN (R 4.2.3)
P magick           2.8.2    2023-12-20 [?] CRAN (R 4.2.3)
P magrittr       * 2.0.3    2022-03-30 [?] CRAN (R 4.2.3)
P MASS             7.3-58.2 2023-01-23 [?] CRAN (R 4.2.3)
P Matrix           1.6-0    2023-07-08 [?] RSPM (R 4.2.0)
P MatrixModels     0.5-2    2023-07-10 [?] RSPM (R 4.2.0)
P matrixStats      1.2.0    2023-12-11 [?] CRAN (R 4.2.3)
P mgcv             1.8-42   2023-03-02 [?] CRAN (R 4.2.3)
P mime             0.12     2021-09-28 [?] CRAN (R 4.2.3)
P munsell          0.5.0    2018-06-12 [?] CRAN (R 4.2.3)
P nlme             3.1-162  2023-01-31 [?] CRAN (R 4.2.3)
P officer          0.6.6    2024-05-05 [?] CRAN (R 4.2.3)
P openssl          2.1.1    2023-09-25 [?] CRAN (R 4.2.3)
P pander           0.6.5    2022-03-18 [?] CRAN (R 4.2.3)
P patchwork        1.2.0    2024-01-08 [?] CRAN (R 4.2.3)
P pillar           1.9.0    2023-03-22 [?] CRAN (R 4.2.3)
P pkgconfig        2.0.3    2019-09-22 [?] CRAN (R 4.2.3)
```

```
P plyr                 1.8.9    2023-10-02 [?] CRAN (R 4.2.3)
P pmforest           * 0.2.0    2024-02-05 [?] MPNDEV (R 4.2.3)
P pmtables             0.6.0    2024-02-05 [?] MPNDEV (R 4.2.3)
P polyclip             1.10-6   2023-09-27 [?] CRAN (R 4.2.3)
P promises             1.2.1    2023-08-10 [?] CRAN (R 4.2.3)
P proxy                0.4-27   2022-06-09 [?] CRAN (R 4.2.3)
P pryr                 0.1.6    2023-01-17 [?] CRAN (R 4.2.3)
P purrr              * 1.0.2    2023-08-10 [?] CRAN (R 4.2.3)
P quantreg             5.96     2023-07-19 [?] RSPM (R 4.2.0)
P R.cache              0.16.0   2022-07-21 [?] CRAN (R 4.2.3)
P R.methodsS3          1.8.2    2022-06-13 [?] CRAN (R 4.2.3)
P R.oo                 1.26.0   2024-01-24 [?] CRAN (R 4.2.3)
P R.utils              2.12.3   2023-11-18 [?] CRAN (R 4.2.3)
P R6                   2.5.1    2021-08-19 [?] CRAN (R 4.2.3)
P ragg                 1.2.7    2023-12-11 [?] CRAN (R 4.2.3)
P rapportools          1.1      2022-03-22 [?] CRAN (R 4.2.3)
P RColorBrewer         1.1-3    2022-04-03 [?] CRAN (R 4.2.3)
P Rcpp                 1.0.11   2023-07-06 [?] CRAN (R 4.2.3)
P readr              * 2.1.5    2024-01-10 [?] CRAN (R 4.2.3)
  renv                 1.0.3    2023-09-19 [1] CRAN (R 4.2.3)
P reshape2           * 1.4.4    2020-04-09 [?] CRAN (R 4.2.3)
P rlang                1.1.2    2023-11-04 [?] CRAN (R 4.2.3)
P rmarkdown            2.27     2024-05-17 [?] CRAN (R 4.2.3)
P rprojroot            2.0.4    2023-11-05 [?] CRAN (R 4.2.3)
P rstudioapi           0.15.0   2023-07-07 [?] CRAN (R 4.2.3)
P scales               1.3.0    2023-11-28 [?] CRAN (R 4.2.3)
P sessioninfo        * 1.2.2    2021-12-06 [?] CRAN (R 4.2.3)
P shiny                1.7.5.1  2023-10-14 [?] CRAN (R 4.2.3)
P snakecase            0.11.1   2023-08-27 [?] CRAN (R 4.2.3)
P SparseM              1.81     2021-02-18 [?] CRAN (R 4.2.3)
P stringi              1.8.1    2023-11-13 [?] RSPM (R 4.2.0)
P stringr            * 1.5.1    2023-11-14 [?] CRAN (R 4.2.3)
P styler             * 1.10.2   2023-08-29 [?] CRAN (R 4.2.3)
P summarytools       * 1.0.1    2022-05-20 [?] CRAN (R 4.2.3)
P survival             3.5-3    2023-02-12 [?] CRAN (R 4.2.3)
P svglite              2.1.3    2023-12-08 [?] CRAN (R 4.2.3)
P systemfonts          1.0.5    2023-10-09 [?] CRAN (R 4.2.3)
P textshaping          0.3.7    2023-10-09 [?] CRAN (R 4.2.3)
P tibble             * 3.2.1    2023-03-20 [?] CRAN (R 4.2.3)
P tidylog            * 1.0.2    2020-07-03 [?] CRAN (R 4.2.3)
P tidyr              * 1.3.1    2024-01-24 [?] CRAN (R 4.2.3)
P tidyselect         * 1.2.0    2022-10-10 [?] CRAN (R 4.2.3)
P tidyverse          * 2.0.0    2023-02-22 [?] CRAN (R 4.2.3)
```

```
P tidyvpc           * 1.5.1    2024-01-18 [?] CRAN (R 4.2.3)
P timechange          0.3.0    2024-01-18 [?] CRAN (R 4.2.3)
P tweenr              2.0.2    2022-09-06 [?] CRAN (R 4.2.3)
P tzdb                0.4.0    2023-05-12 [?] CRAN (R 4.2.3)
P utf8                1.2.4    2023-10-22 [?] CRAN (R 4.2.3)
P uuid                1.2-0    2024-01-14 [?] CRAN (R 4.2.3)
P vctrs               0.6.4    2023-10-12 [?] CRAN (R 4.2.3)
P viridisLite         0.4.2    2023-05-02 [?] CRAN (R 4.2.3)
P withr               2.5.2    2023-10-30 [?] CRAN (R 4.2.3)
P xfun                0.41     2023-11-01 [?] CRAN (R 4.2.3)
P xml2                1.3.6    2023-12-04 [?] CRAN (R 4.2.3)
P xtable              1.8-4    2019-04-21 [?] CRAN (R 4.2.3)
P yaml                2.3.7    2023-01-23 [?] CRAN (R 4.2.3)
P zip                 2.3.1    2024-01-27 [?] CRAN (R 4.2.3)
P zoo               * 1.8-12   2023-04-13 [?] CRAN (R 4.2.3)

 [1] /mnt/data/code/SSC/Emmes/Emmes-Dalbavancin-DOTS/renv/library/R-4.2/x86_64-pc-linux-gnu
 [2] /mnt/data/.cache/R/renv/sandbox/R-4.2/x86_64-pc-linux-gnu/e11edd0e

P -- Loaded and on-disk path mismatch.

------------------------------------------------------------------------------
```
