# Supplementary material for: Pharmacokinetics of Dalbavancin in Complicated Staphylococcus aureus Bacteremia: A Secondary Analysis of the DOTS Randomized Clinical Trial
Source: JAMA Netw Open. 2026 Apr 18;9(4):e2611652. doi: 10.1001/jamanetworkopen.2026.11652 (PMC13092111; doi:10.1001/jamanetworkopen.2026.11652)
Supplement: Supplement 3. — eFigure 1. Total and Unbound Plasma Dalbavancin Concentration-Time Profiles by Dosage Group eFigure 2: Association Between Observed Total and Unbound Dalbavancin Concentrations eFigure 3. Associations of Serum Albumin and Total Dalbavancin Concentration With Fraction Unbound eFigure 4. Association Between Creatinine Clearance and Fraction Unbound of Dalbavancin eFigure 5. Distribution of Observed Total and Unbound Dalbavancin Concentrations by Unbound Quantification Status eFigure 6: Association Between Total and Unbound Mean (SD) Exposure Metrics and Clinical Success at Day 42 eFigure 7. Association Between Tertiles of Total and Unbound Dalbavancin Exposure and Clinical Success at Day 42 eFigure 8. Unadjusted Risk Differences for Clinical Success Across Assumptions for Missing Day 70 Outcomes Using the Total Day 22 Concentration Cut Point eTable 1. Baseline Characteristics of Pharmacokinetic (PK) Analysis Population eTable 2. Day 70 Safety Outcomes by Exposure Groups Defined by Total and Unbound Dalbavancin Concentrations eTable 3: Baseline Characteristics by Clinical Outcome at Day 42 (Exposure-Efficacy Population) eTable 4. Association Between Total and Unbound Dichotomization Exposure Metrics and Clinical Efficacy at Day 42 eTable 5. Clinical Efficacy at Day 42 Across Exposure Thresholds by Clinical Subgroups eTable 6. Comparison of Baseline Characteristics Among Participants With Nonmissing vs Missing Day 70 Clinical Efficacy Data eTable 7. Exposure-Outcome Differences With Inverse Probability Weighting to Address Missing Outcome Data [file jamanetwopen-e2611652-s003.pdf]

Supplemental Online Content

Lodise TP, Turner NA, Hamasaki T, et al; for the Antibacterial Resistance Leadership Group. Pharmacokinetics of dalbavancin in complicated *Staphylococcus aureus* bacteremia: a secondary analysis of the dots randomized clinical trial. *JAMA Netw Open*. 2026;9(4):e2611652. doi:10.1001/jamanetworkopen.2026.11652

eFigure 1. Total and Unbound Plasma Dalbavancin Concentration-Time Profiles by Dosage Group

eFigure 2: Association Between Observed Total and Unbound Dalbavancin Concentrations

eFigure 3. Associations of Serum Albumin and Total Dalbavancin Concentration With Fraction Unbound

eFigure 4. Association Between Creatinine Clearance and Fraction Unbound of Dalbavancin

eFigure 5. Distribution of Observed Total and Unbound Dalbavancin Concentrations by Unbound Quantification Status

eFigure 6: Association Between Total and Unbound Mean (SD) Exposure Metrics and Clinical Success at Day 42

eFigure 7. Association Between Tertiles of Total and Unbound Dalbavancin Exposure and Clinical Success at Day 42

eFigure 8. Unadjusted Risk Differences for Clinical Success Across Assumptions for Missing Day 70 Outcomes Using the Total Day 22 Concentration Cut Point

eTable 1. Baseline Characteristics of Pharmacokinetic (PK) Analysis Population

eTable 2. Day 70 Safety Outcomes by Exposure Groups Defined by Total and Unbound Dalbavancin Concentrations

eTable 3: Baseline Characteristics by Clinical Outcome at Day 42 (Exposure-Efficacy Population)

eTable 4. Association Between Total and Unbound Dichotomization Exposure Metrics and Clinical Efficacy at Day 42

eTable 5. Clinical Efficacy at Day 42 Across Exposure Thresholds by Clinical Subgroups

eTable 6. Comparison of Baseline Characteristics Among Participants With Nonmissing vs Missing Day 70 Clinical Efficacy Data

eTable 7. Exposure-Outcome Differences With Inverse Probability Weighting to Address Missing Outcome Data

This supplemental material has been provided by the authors to give readers additional information about their work.

© 2026 Lodise TP et al. *JAMA Network Open*.

eFigure 1. Total and Unbound Plasma Dalbavancin Concentration–Time Profiles by Dosage Group

A. Total Dalbavancin Concentrations

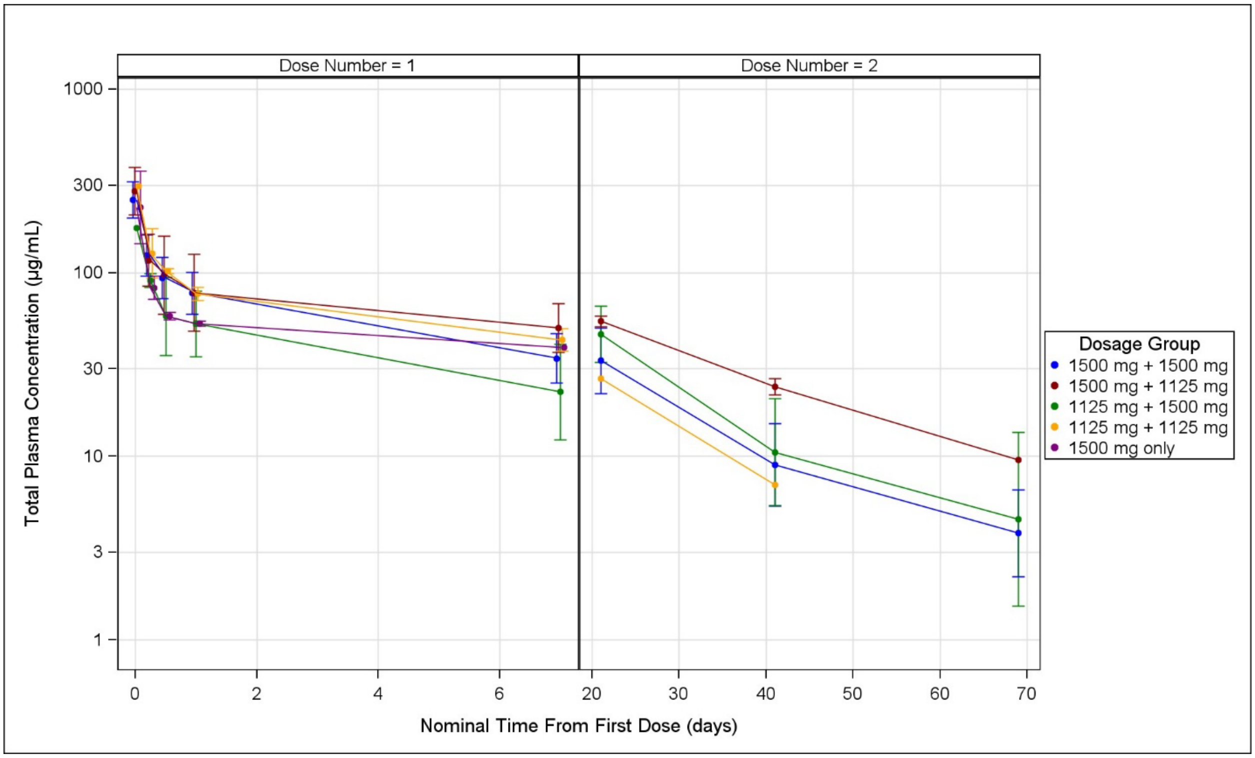

© 2026 Lodise TP et al. *JAMA Network Open*.

B. Unbound Dalbavancin Concentrations

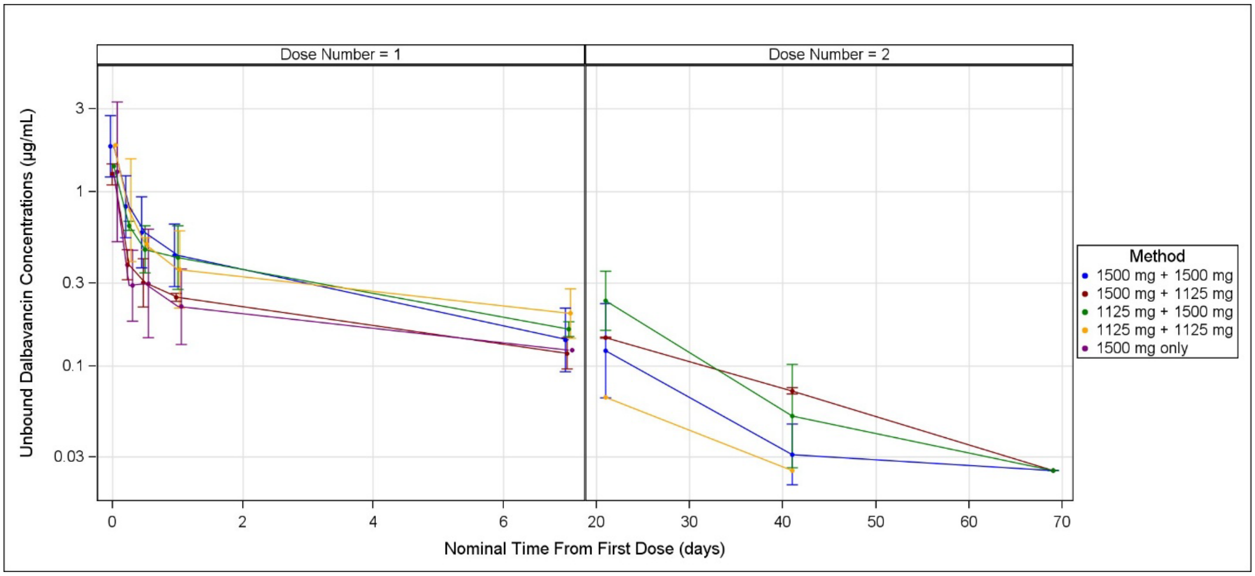

Footnote

Total and unbound dalbavancin plasma concentrations are shown as geometric means at each nominal sampling time by dosage group. Nominal time is referenced to the first dose (day 1) and the second dose (day 8). For the unbound concentration panel, observations below the lower limit of quantification (BQL) were imputed as one-half the lower limit of quantification for calculation of geometric means. Error bars represent geometric standard deviations.

© 2026 Lodise TP et al. *JAMA Network Open*.

eFigure 2: Association Between Observed Total and Unbound Dalbavancin Concentrations

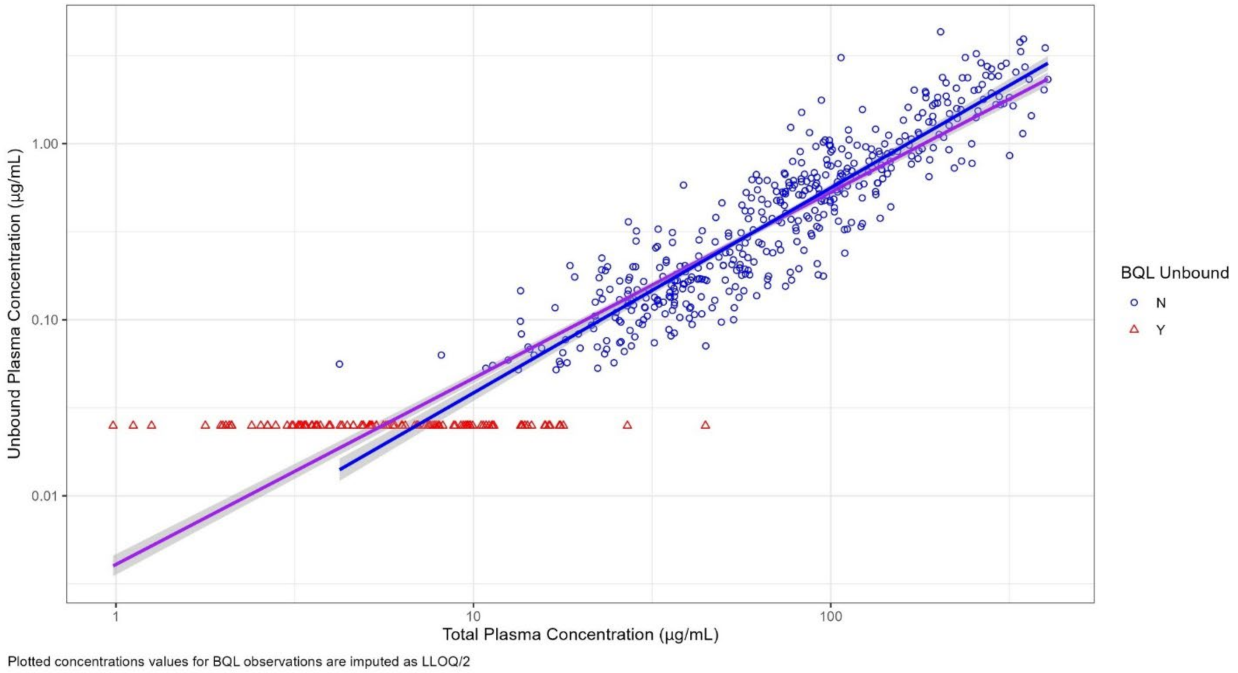

Footnote

Each point represents an observed paired total and unbound dalbavancin plasma concentration. Observations with unbound concentrations below the lower limit of quantification (BQL) are included and plotted at the BQL value for visualization. Both axes are displayed on a logarithmic scale.

© 2026 Lodise TP et al. *JAMA Network Open*.

eFigure 3. Associations of Serum Albumin and Total Dalbavancin Concentration With Fraction Unbound

A: Association Between Serum Albumin Concentration and Fraction Unbound of Dalbavancin

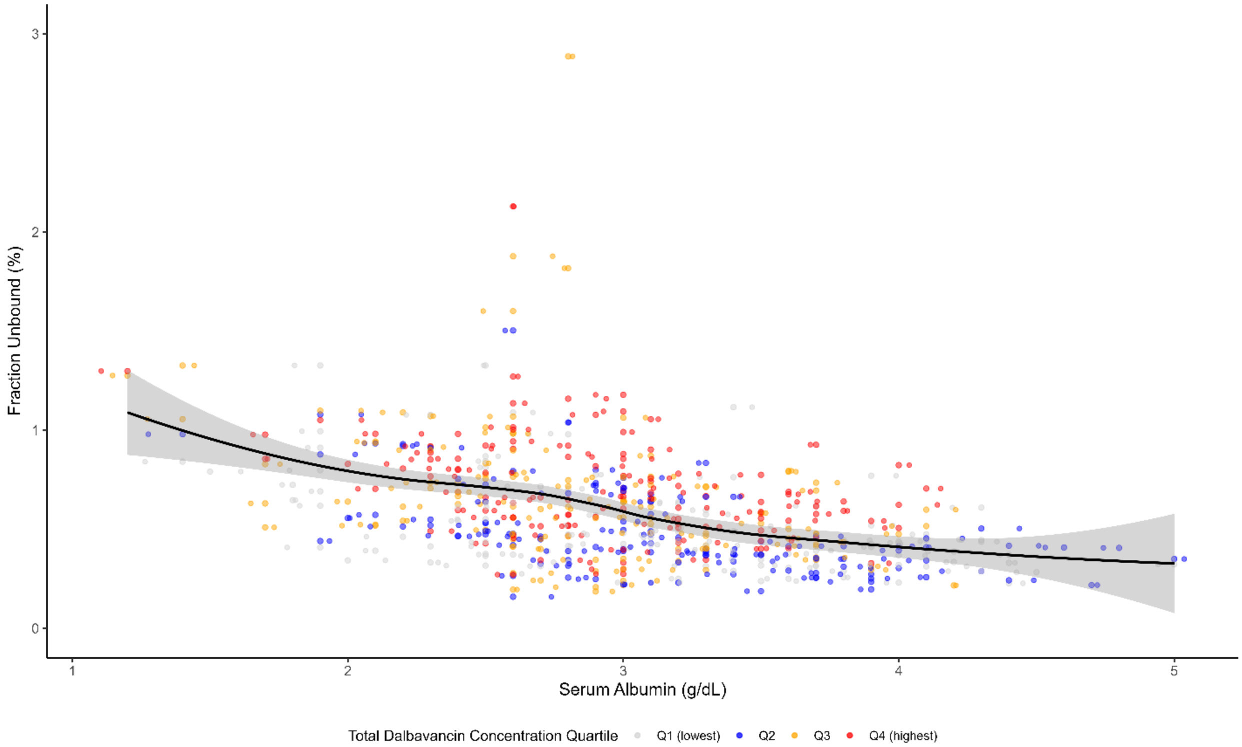

Spearman's ρ = -0.49 (95% CI: -0.56 to -0.42). CI was estimated by nonparametric bootstrap resampling (1,000 replicates).
Fraction unbound (%) was calculated only for samples with quantifiable unbound dalbavancin concentrations. Samples with unbound concentrations below the lower limit of quantitation were excluded from the analysis (n=112).

© 2026 Lodise TP et al. *JAMA Network Open*.

B. Fraction Unbound of Dalbavancin Across Total Concentrations Stratified by Serum Albumin

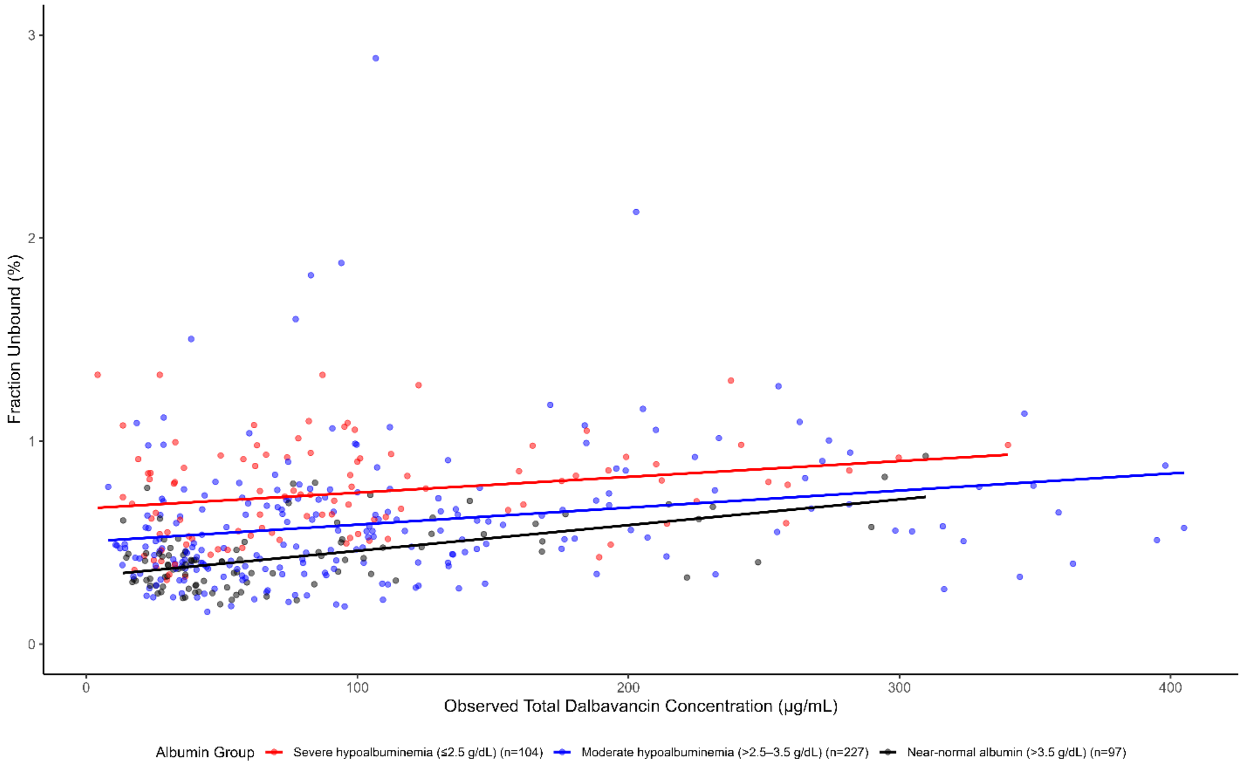

Fraction unbound (%) was calculated only for samples with quantifiable unbound dalbavancin concentrations. Samples with unbound concentrations below the lower limit of quantitation were excluded from the analysis (n=112).

Footnotes

Fraction unbound (%) was calculated as unbound concentration divided by total concentration ×100. Each point represents an observed paired pharmacokinetic sample with quantifiable unbound dalbavancin concentration. The albumin value used corresponds to the measurement closest to the pharmacokinetic sampling time. The solid line in Panel A represents locally weighted regression (LOESS) smoothing. Lines in Panel B represent fitted linear regression relationships within each albumin stratum. Observations with unbound concentrations below the lower limit of quantification were excluded from the fraction unbound calculation.

© 2026 Lodise TP et al. *JAMA Network Open*.

eFigure 4. Association Between Creatinine Clearance and Fraction Unbound of Dalbavancin

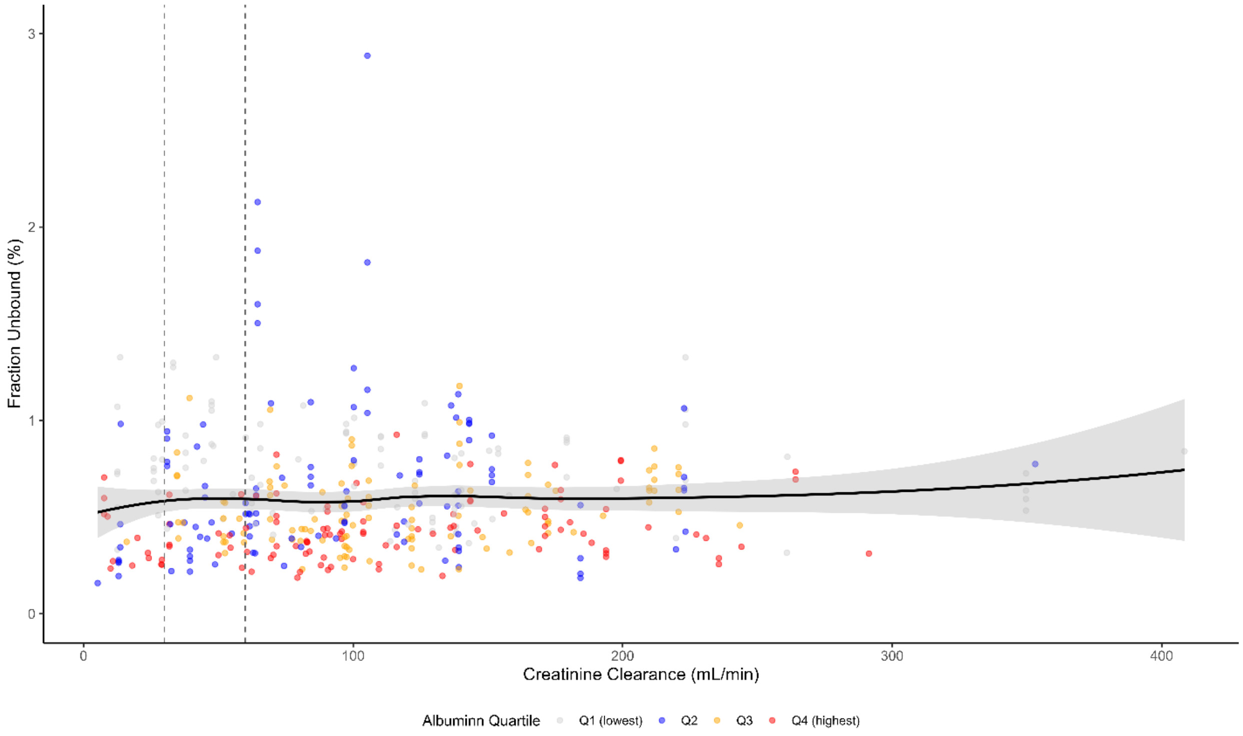

Spearman's ρ = 0.09 (95% CI: -0.01 to 0.18). CI was estimated by nonparametric bootstrap resampling (1,000 replicates).
Fraction unbound (%) was calculated only for samples with quantifiable unbound dalbavancin concentrations. Samples with unbound concentrations below the lower limit of quantitation were excluded from the analysis (n=112).
Vertical dashed lines at 30 and 60 mL/min indicate thresholds corresponding to severe renal impairment (<30 mL/min), moderate impairment (30–60 mL/min), and normal/mild renal function (>60 mL/min).

Footnote: Footnote

Each point represents an observed paired pharmacokinetic sample with quantifiable unbound dalbavancin concentration. Fraction unbound (%) was calculated as unbound concentration divided by total concentration ×100. Creatinine clearance (CL_{CR}) values correspond to the measurement closest to the pharmacokinetic sampling time within the same day when available. The solid line represents locally weighted regression (LOESS) smoothing with 95% confidence intervals. The full observed range of creatinine clearance values is displayed.

© 2026 Lodise TP et al. *JAMA Network Open*.

eFigure 5. Distribution of Observed Total and Unbound Dalbavancin Concentrations by Unbound Quantification Status

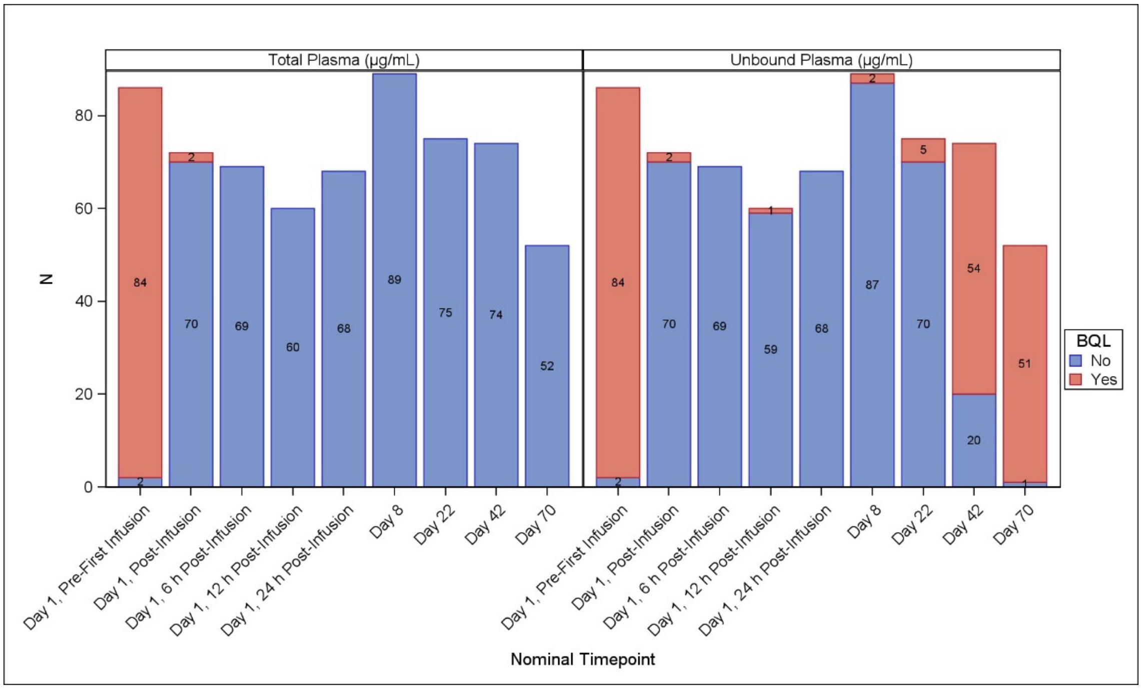

Footnote

Each point represents an observed paired total and unbound dalbavancin plasma concentration. Observations are stratified according to whether the unbound concentration was quantifiable or below the lower limit of quantification (BQL). Fraction unbound (%) was calculated as unbound concentration divided by total concentration ×100. Observations with unbound concentrations below the lower limit of quantification were identified but not used in fraction unbound calculations.

© 2026 Lodise TP et al. *JAMA Network Open*.

eFigure 6: Association between Total and Unbound Mean (Standard Deviation) Exposure Metrics and Clinical Success at Day 42

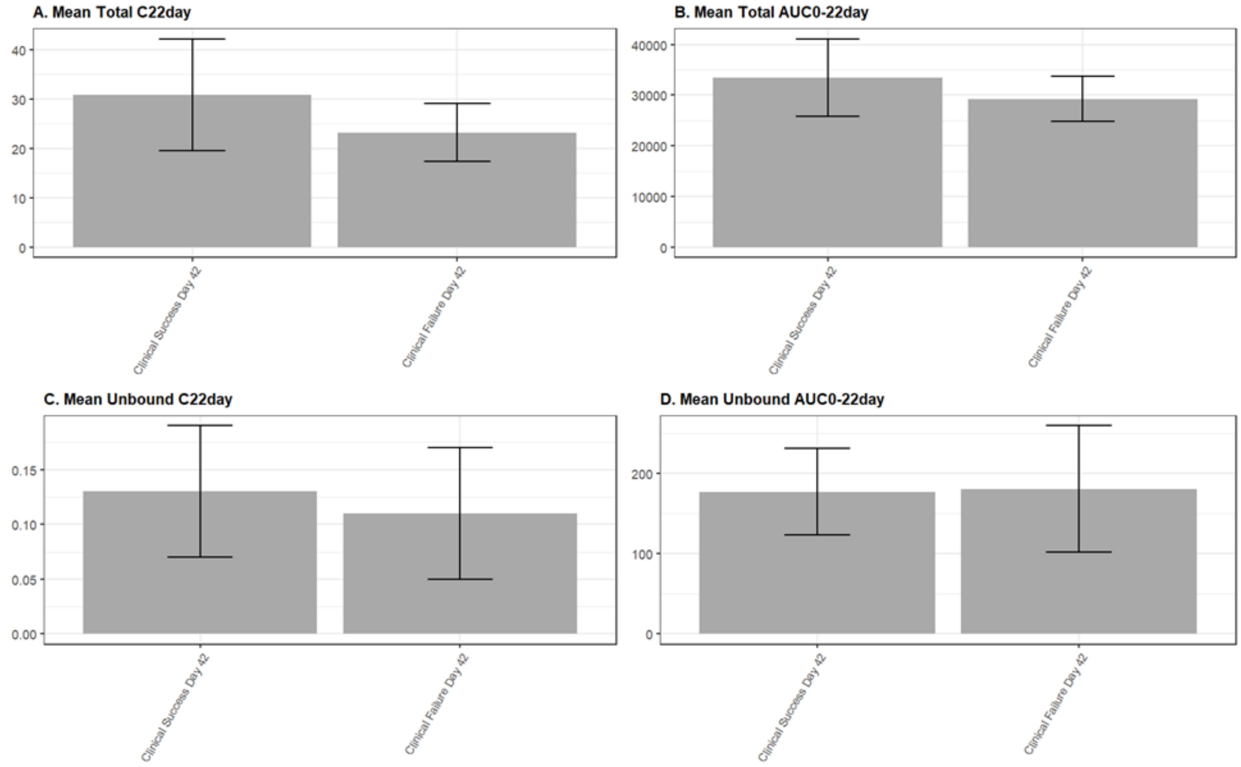

Footnotes: Values are presented as mean (standard deviation). Total and unbound dalbavancin concentrations at Day 22 (C22day) are expressed in μg/mL. Total and unbound area under the plasma concentration–time curve from Day 0 through Day 22 (AUC0–22days) are expressed in μg·h/mL.

© 2026 Lodise TP et al. *JAMA Network Open*.

eFigure 7. Association Between Tertiles of Total and Unbound Dalbavancin Exposure and Clinical Success at Day 42

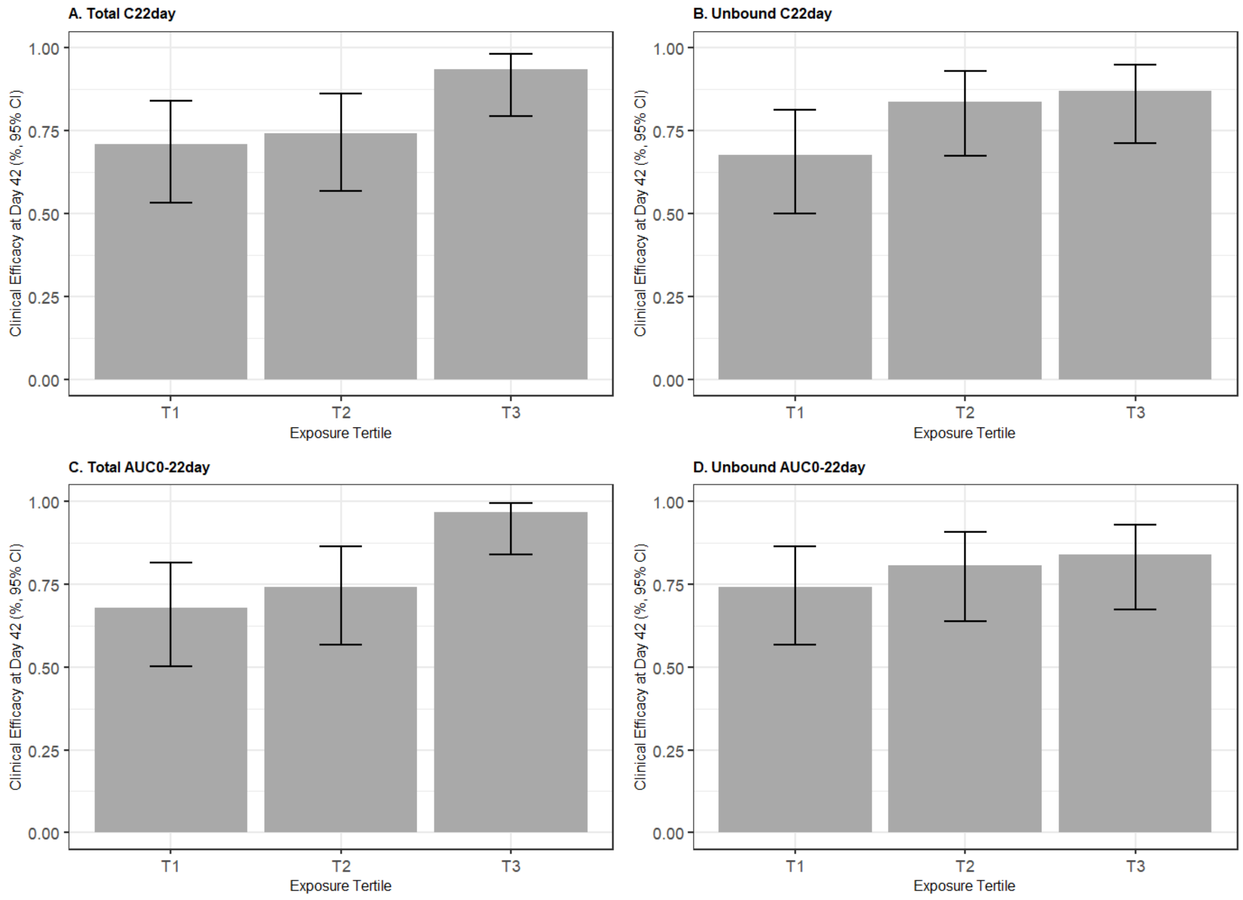

Footnotes: Total and unbound dalbavancin concentrations at Day 22 (C22day) are expressed in µg/mL. Total and unbound area under the plasma concentration–time curve from Day 0 through Day 22 (AUC0–22days) are expressed in µg·h/mL.

Tertiles of dalbavancin exposure were defined as follows:

Total C22day: T1, <23.7 µg/mL; T2, 23.7–<32 µg/mL; T3, 32–<61.5 µg/mL.

Unbound C22day: T1, <0.09 µg/mL; T2, 0.09–<0.138 µg/mL; T3, 0.138–<0.301 µg/mL.

Total AUC0–22days: T1, <28,806.3 µg·h/mL; T2, 28,806.3–<35,183.8 µg·h/mL; T3, 35,183.8–<51,488.9 µg·h/mL.Unbound AUC0–22days: T1, <146.2 µg·h/mL; T2, 146.2–<192.6 µg·h/mL; T3, 192.6–<342.8 µg·h/mL

© 2026 Lodise TP et al. *JAMA Network Open*.

eFigure 8. Unadjusted Risk Differences for Clinical Success Across Assumptions for Missing Day-70 Outcomes Using the Total C22day Cutpoint

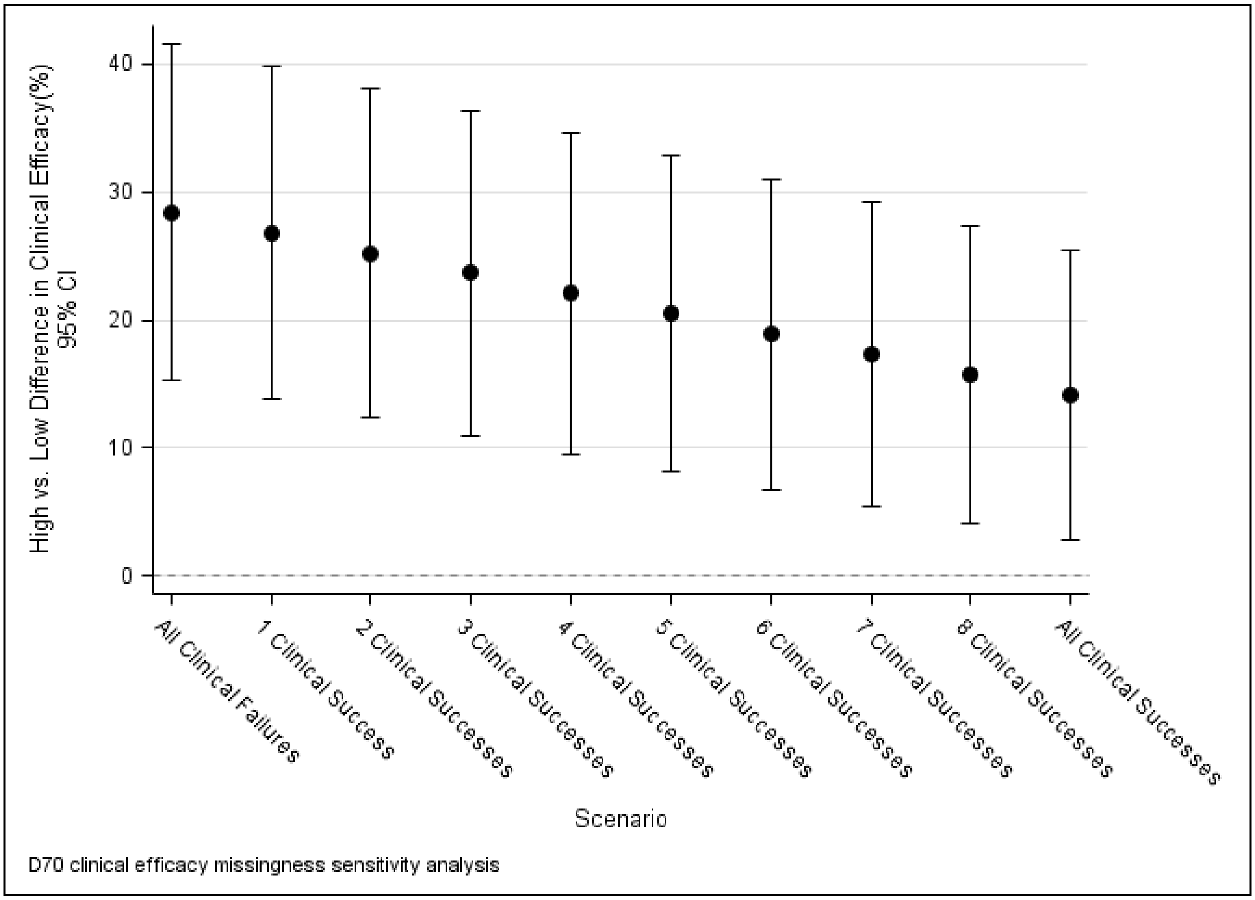

Footnote

The figure shows unadjusted risk differences comparing clinical success between participants with total dalbavancin concentrations above versus below the C22day exposure cutpoint under alternative assumptions for 9 participants with missing Day-70 efficacy outcomes. Each scenario represents a different assumption regarding the clinical outcome status of participants with missing data. Risk differences are presented with corresponding confidence intervals.

© 2026 Lodise TP et al. *JAMA Network Open*.

eTable 1. Baseline Characteristics of the Pharmacokinetic (PK) Analysis Population

Baseline characteristic	Patients, No. (%)
	**Overall (N = 97)**
Age, mean (SD), y	54.5 (15.8)
Sex	
Male	69 (71.1)
Female	28 (28.9)
Race	
Asian	5 (5.2)
American Indian or Alaska Native	1 (1.0)
Black or African American	20 (20.6)
White	67 (69.1)
Unknown	4 (4.1)
Ethnicity	
Hispanic or Latino	11 (11.3)
Not Hispanic or Latino	82 (84.5)
Not reported	2 (2)
Unknown	2 (2)
Weight, mean (SD), kg	87.4 (22.2)
BMI, mean (SD)	29.0 (6.9)
Creatinine clearance, mean (SD), mL/min	113.0 (70.8)
Albumin, mean (SD), g/dL	2.8 (0.6)
Qualifying pathogen	
MRSA	33 (34.0)
MSSA	64 (66.0)
Underlying site of infection	
Endovascular	28 (28.9)
Bone and joint	25 (25.8)
Skin	39 (40.2)
Pulmonary	11 (11.3)
Other or unknown	23 (23.7)
Persons who inject drugs status	14 (14.4)
Immunosuppression	35 (36.1)
Received dialysis	12 (12.4)
Pre randomization antibiotic	
β-Lactam	93 (95.9)
Vancomycin	82 (84.5)
Daptomycin	14 (14.4)
Other	14 (14.4)

© 2026 Lodise TP et al. *JAMA Network Open*.

Medical history	
Heart failure	21 (21.6)
Chronic kidney disease	20 (20.6)
Diabetes	43 (44.3)
Liver disease	13 (13.4)
Cancer	21 (21.6)
Duration of initial bacteremia, d	
<2	74 (76.3)
≥2	23 (23.7)

Footnote: Values are presented as mean (standard deviation) unless otherwise indicated. Percentages are calculated using the total number of participants in the pharmacokinetic analysis population (N = 97) as the denominator. Creatinine clearance was estimated using the Cockcroft–Gault equation and expressed as absolute mL/min. Body mass index (BMI) was calculated as weight in kilograms divided by height in meters squared. Baseline laboratory values correspond to the measurement closest to study enrollment. Underlying sites of infection are not mutually exclusive. Pre-randomization antibiotics reflect therapy received prior to study treatment initiation.

© 2026 Lodise TP et al. *JAMA Network Open*.

eTable 2. Day 70 Safety Outcomes by Exposure Groups Defined by Total and Unbound Dalbavancin Concentrations

Exposure Measure	Exposure Categorization	N	Safety Outcome, n (%)			Unadjusted Difference in SAE Proportions % (95% CI)
			SAE or AE leading to treatment discontinuation	AE leading to treatment discontinuation	SAE	
Total C_{22} (µg/mL)	≤ 32.0	63	27 (42.9%)	0	27 (42.9%)	-16.2 (-36.2, 3.8)
	> 32.0	30	8 (26.7%)	0	8 (26.7%)	
Unbound C_{22} (µg/mL)	≤ 0.137	61	23 (37.7%)	0	23 (37.7%)	-0.2 (-20.9, 20.5)
	> 0.137	32	12 (37.5%)	0	12 (37.5%)	
Total AUC_{0-22} (ug*h/mL)	≤ 37414	71	31 (43.7%)	0	31 (43.7%)	-25.5 (-45.3, -5.7)
	> 37414	22	4 (18.2%)	0	4 (18.2%)	
Unbound AUC_{0-22} (ug*h/mL)	≤ 174	47	17 (36.2%)	0	17 (36.2%)	3.0 (-16.7, 22.6)
	> 174	46	18 (39.1%)	0	18 (39.1%)	
Total PK		93	35 (37.6%)	0	35 (37.6%)	

Footnote: Total and unbound concentration at day 22 (C_{22day}) were expressed in µg/mL. Cumulative total and unbound area under the plasma concentration-time curves, were expressed as µg*h/mL, from days 0-22 ($AUC_{0-22days}$). All unadjusted differences were calculated using the lower-exposure group as reference.

Abbreviations: DICH, dichotomization; SAE, serious adverse event; AE, adverse event.

© 2026 Lodise TP et al. *JAMA Network Open*.

eTable 3: Baseline Characteristics by Clinical Outcome at Day 42 (Exposure–Efficacy Population)

Baseline characteristic	Patients, No. (%)			SMD
	Overall (N = 93)	Clinical success (n = 74)	Clinical failure[a] (n = 19)	
Age, mean (SD), y	54.1 (15.9)	55.1 (15.7)	50.3 (16.3)	0.30
Sex				
Male	66 (71.0)	51 (68.9)	15 (78.9)	0.23
Female	27 (29.0)	23 (31.1)	4 (21.1)	0.23
Race				
Asian	5 (5.4)	4 (5.4)	1 (5.3)	0.01
American Indian or Alaska Native	1 (1.1)	1 (1.4)	0 (0.0)	0.17
Black or African American	20 (21.5)	16 (21.6)	4 (21.1)	0.01
White	64 (68.8)	50 (67.6)	14 (73.7)	0.13
Unknown	3 (3.2)	3 (4.1)	0 (0.0)	0.29
Ethnicity				
Hispanic or Latino	9 (9.7)	8 (10.8)	1 (5.3)	0.21
Not Hispanic or Latino	81 (87.1)	64 (86.5)	17 (89.5)	0.09
Not reported	2 (2.2)	1 (1.4)	1 (5.3)	0.22
Unknown	1 (1.1)	1 (1.4)	0 (0.0)	0.17
Weight, mean (SD), kg	88.1 (22.0)	88.7 (22.5)	85.8 (20.1)	0.13
BMI, mean (SD)	29.2 (6.8)	29.5 (7.1)	28.1 (5.9)	0.21
Creatinine clearance, mean (SD), mL/min	115.9 (70.7)	116.4 (72.6)	114.2 (64.3)	0.03
Albumin, mean (SD), g/dL	2.8 (0.6)	2.8 (0.6)	2.8 (0.7)	0.07
Qualifying pathogen				
MRSA	32 (34.4)	25 (33.8)	7 (36.8)	0.06
MSSA	61 (65.6)	49 (66.2)	12 (63.2)	0.06
Underlying site of infection				
Endovascular	28 (30.1)	21 (28.4)	7 (36.8)	0.18
Bone and joint	25 (26.9)	18 (24.3)	7 (36.8)	0.27
Skin	37 (39.8)	30 (40.5)	7 (36.8)	0.08
Pulmonary	8 (8.6)	8 (10.8)	0 (0.0)	0.49
Other or unknown	21 (22.6)	18 (24.3)	3 (15.8)	0.21
Persons who inject drugs status	14 (15.1)	9 (12.2)	5 (26.3)	0.37
Immunosuppression	33 (35.5)	26 (35.1)	7 (36.8)	0.04
Received dialysis	10 (10.8)	9 (12.2)	1 (5.3)	0.25
Pre randomization antibiotic				
β-Lactam	89 (95.7)	70 (94.6)	19 (100.0)	0.34
Vancomycin	78 (83.9)	64 (86.5)	14 (73.7)	0.32

© 2026 Lodise TP et al. *JAMA Network Open*.

Daptomycin	14 (15.1)	13 (17.6)	1 (5.3)	0.39
Other	13 (14.0)	12 (16.2)	1 (5.3)	0.36
Medical history				
Heart failure	21 (22.6)	16 (21.6)	5 (26.3)	0.11
Chronic kidney disease	18 (19.4)	15 (20.3)	3 (15.8)	0.12
Diabetes	41 (44.1)	32 (43.2)	9 (47.4)	0.08
Liver disease	11 (11.8)	8 (10.8)	3 (15.8)	0.15
Cancer	19 (20.4)	15 (20.3)	4 (21.1)	0.02
Duration of initial bacteremia, d				
<2	70 (75.3)	56 (75.7)	14 (73.7)	0.05
≥2	23 (24.7)	18 (24.3)	5 (26.3)	0.05

Abbreviations: BMI, body mass index (calculated as weight in kilograms divided by height in meters squared); MRSA, methicillin resistant *Staphylococcus aureus*; MSSA, methicillin susceptible *Staphylococcus aureus*; SMD, standardized mean difference.

[a]By day 42, 19 participants were classified as having clinical failures: 5 had both absence of clinical efficacy and an infectious complication, and 14 had a single event (5 infectious complications and 9 lack of clinical efficacy). All infectious complications occurred on or after day 40.

© 2026 Lodise TP et al. *JAMA Network Open*.

eTable 4. Association between Total and Unbound Dichotomization (DICH) Exposure Metrics and Clinical Efficacy at Day 42

Exposure Measure	Exposure Categorization	N	Clinical Efficacy at Day 42, n (%)	Difference in Percentages % (95% CI)	
				Unadjusted	Adjusted
DICH Total C_{22day} (μg/mL) cutpoint	≤ 32.0	63	45 (71)	25.2 (12.4, 38.1)	26.5 (4.4-48.6)
	> 32.0	30	29 (97)		
DICH Unbound C_{22day} (μg/mL) cutpoint	≤ 0.125	53	38 (72)	18.3 (3.0-33.6)	18.9 (3.7-34.0)
	> 0.125	40	36 (90)		
DICH Total $AUC_{0\text{-}22days}$ (μg*h/mL) cutpoint	≤ 35184	63	45 (71)	25.2 (12.4-38.1)	26.5 (4.4-48.6)
	> 35184	30	29 (97)		
DICH Unbound $AUC_{0\text{-}22days}$ (μg*h/mL) cutpoint	≤ 185	55	41 (75)	12.3 (-3.5-28.1)	12.3 (-3.7-28.3)
	> 185	38	33 (87)		

Footnotes: Total and unbound dalbavancin concentrations at day 22 (C22day) are expressed in μg/mL. Total and unbound area under the plasma concentration–time curve from day 0 through day 22 (AUC0–22days) are expressed in μg·h/mL. Exposure groups were defined by dichotomization at the specified cutpoints. Adjusted models included baseline covariates evaluated for association with clinical efficacy. No baseline covariates were independently associated with clinical efficacy at day 42.

© 2026 Lodise TP et al. *JAMA Network Open*.

eTable 5. Clinical Efficacy at Day 42 Across Exposure Thresholds by Clinical Subgroups

DICH Exposure Metric	Timepoint	Subgroup	Below Cutpoint Efficacy % (n/N)	Above Cutpoint Efficacy % (n/N)	Difference in Percentages % (95% CI)
Total C_{22day} (32 μg/mL)	Day 42	Bacteremia ≤2 days	73% (35/48)	95% (21/22)	22.5 (7.3, 37.8)
		Bacteremia >2 days	67% (10/15)	100% (8/8)	33.3 (9.5, 57.2)
		No deep-seated infection	79% (19/24)	95% (18/19)	15.6 (−3.5, 34.7)
		Deep-seated infection	67% (26/39)	100% (11/11)	33.3 (18.5, 48.1)
		MSSA	72% (31/43)	100% (18/18)	27.9 (14.5, 41.3)
		MRSA	70% (14/20)	92% (11/12)	21.7 (-3.8, 47.1)
Unbound C_{22day} (0.125 μg/mL)	Day 42	Bacteremia ≤2 days	71% (30/42)	93% (26/28)	21.4 (4.8, 38.1)
		Bacteremia >2 days	73% (8/11)	83% (10/12)	10.6 (−23.1, 44.3)
		No deep-seated infection	80% (16/20)	91% (21/23)	11.3 (−9.7, 32.3)
		Deep-seated infection	67% (22/33)	88% (15/17)	21.6 (−0.6, 43.8)
		MSSA	75% (30/40)	90% (10/21)	15.5 (-0.3, 33.9)
		MRSA	62% (8/13)	89% (17/19)	27.9 (-1.9, 57.8)
Total $AUC_{0-22days}$ (35184 μg·h/mL)	Day 42	Bacteremia ≤2 days	72% (33/46)	96% (23/24)	24.1 (8.8, 39.4)
		Bacteremia >2 days	71% (12/17)	100% (6/6)	29.4 (7.8, 51.1)
		No deep-seated infection	78% (21/27)	100% (16/16)	22.2 (6.5, 37.9)

© 2026 Lodise TP et al. *JAMA Network Open*.

		Deep-seated infection	67% (24/36)	93% (13/14)	26.2 (5.7, 46.7)
		MSSA	72% (31/43)	100% (18/18)	27.9 (14.5, 41.3)
		MRSA	70% (14/20)	92% (11/12)	21.7 (-3.8, 47.1)
Unbound $AUC_{0–22days}$ (185 µg·h/mL)	Day 42	Bacteremia ≤2 days	74% (31/42)	89% (25/28)	15.5 (−2.1, 33.0)
		Bacteremia >2 days	77% (10/13)	80% (8/10)	3.1 (−30.7, 36.8)
		No deep-seated infection	85% (22/26)	88% (15/17)	3.6 (−17.0, 24.3)
		Deep-seated infection	66% (19/29)	86% (18/21)	20.2 (−2.7, 43.1)
		MSSA	78% (29/37)	83% (20/24)	5.0 (-15, 24.9)
		MRSA	67% (12/18)	93% (13/14)	26.2 (0.6, 51.8)

Footnote: Total and unbound dalbavancin concentrations at Day 22 (C22day) are expressed in µg/mL. Total and unbound area under the plasma concentration–time curve from Day 0 through Day 22 (AUC0–22days) are expressed in µg·h/mL. Exposure groups were defined by dichotomization at the specified cutpoints.

Deep-seated infection was defined as the presence of at least one of the following: endocarditis, osteoarticular infection, cardiac device infection, septic thrombophlebitis, or deep noncutaneous abscess.

Abbreviations: MRSA, methicillin-resistant *Staphylococcus aureus*; MSSA, methicillin-susceptible *Staphylococcus aureus*.

© 2026 Lodise TP et al. *JAMA Network Open*.

eTable 6. Comparison of Baseline Characteristics Among Participants With Non-Missing vs Missing Day 70 Clinical Efficacy Data

**Baseline Characteristics**	**Evaluable Population (N = 93)**	**Clinical Efficacy Non-missing (n = 84)**	**Clinical Efficacy Missing (n = 9)**
Age, mean (SD) years	54.1 (15.9)	55.0 (15.7)	45.3 (15.1)
Weight, mean (SD) kg	88.1 (22.0)	88.8 (22.4)	81.4 (17.5)
BMI, mean (SD) kg/m^2	29.2 (6.8)	29.5 (7.1)	26.9 (3.3)
Creatinine Clearance, mean (SD) mL/min	115.9 (70.7)	115.7 (70.6)	118.2 (75.9)
Albumin, mean (SD) g/dL	2.8 (0.6)	2.8 (0.6)	2.9 (0.7)
Qualifying Pathogen: MRSA, n (%)	32 (34)	30 (36)	2 (22)
Sex: Female, n (%)	27 (29)	24 (29)	3 (33)
Race: White, n (%)	64 (69)	58 (69)	6 (67)
Underlying Site of Infection: Endovascular, n (%)	28 (30)	23 (27)	5 (56)
Underlying Site of Infection: Bone and Joint, n (%)	25 (27)	21 (25)	4 (44)
Underlying Site of Infection: Skin, n (%)	37 (40)	31 (37)	6 (67)
Underlying Site of Infection: Pulmonary, n (%)	8 (9)	7 (8)	1 (11)
Underlying Site of Infection: Other/Unknown, n (%)	21 (23)	20 (24)	1 (11)
Persons who Inject Drugs, n (%)	14 (15)	11 (13)	3 (33)
Immunosuppression, n (%)	33 (35)	29 (35)	4 (44)
Received Dialysis, n (%)	10 (11)	9 (11)	1 (11)
Pre-Randomization Antibiotic: Beta-lactam, n (%)	89 (96)	80 (95)	9 (100)
Pre-Randomization Antibiotic: Vancomycin, n (%)	78 (84)	69 (82)	9 (100)
Pre-Randomization Antibiotic: Daptomycin, n (%)	14 (15)	14 (17)	0
Pre-Randomization Antibiotic: Other, n (%)	13 (14)	13 (15)	0
Medical History: Heart Failure, n (%)	21 (23)	19 (23)	2 (22)
Medical History: Chronic Kidney Disease, n (%)	18 (19)	16 (19)	2 (22)
Medical History: Diabetes, n (%)	41 (44)	37 (44)	4 (44)
Medical History: Liver Disease, n (%)	11 (12)	9 (11)	2 (22)

© 2026 Lodise TP et al. *JAMA Network Open*.

Medical History: Cancer, n (%)	19 (20)	19 (23)	0
Duration of Initial Bacteremia ≥2 days, n (%)	23 (25)	23 (27)	0

Footnote: Values are presented as mean (standard deviation) unless otherwise indicated. Percentages are calculated using the number of participants within each column as the denominator. One participant in the non-missing clinical efficacy group had missing creatinine clearance data.

© 2026 Lodise TP et al. *JAMA Network Open*.

eTable 7. Exposure–Outcome Differences With Inverse Probability Weighting to Address Missing Outcome Data

Exposure Measure	Exposure Categorization	N	Outcome Yes, n (%)	Unadjusted Difference % (95% CI)	IPW – adjusted difference % (95% CI) – Model 1	IPW – adjusted difference % (95% CI) – Model 2	IPW – adjusted difference % (95% CI) – Model 3	IPW – adjusted difference % (95% CI) – Model 4
DICH Total C_{22day} (μg/mL) cutpoint	≤ 32.0	63	43 (68)	28.4 (15.3–41.6)	18.0 (5.4–30.5)	17.0 (4.6–29.4)	17.5 (4.9–30.1)	17.5 (4.9–30.0)
	> 32.0	30	29 (97)					
DICH Unbound C_{22day} (μg/mL) cutpoint	≤ 0.137	61	43 (70)	20.1 (4.9–35.4)	6.6 (-8.3–21.5)	6.7 (-7.9–21.3)	8.0 (-6.4–22.5)	7.8 (-6.8–22.3)
	> 0.137	32	29 (91)					
DICH Total $AUC_{0\text{-}22days}$ (μg*h/mL) cutpoint	≤ 37414	71	51 (72)	23.6 (10.0–37.2)	20.0 (10.1–30.0)	19.1 (9.4–28.8)	19.4 (9.6–29.2)	19.5 (9.7–29.2)
	> 37414	22	21 (95)					
DICH Unbound $AUC_{0\text{-}22days}$ (μg*h/mL) cutpoint	≤ 174	47	34 (72)	10.3 (-6.6–27.1)	3.9 (-11.4–19.1)	4.4 (-10.6–19.4)	5.3 (-9.8–20.3)	5.4 (-9.8–20.5)
	> 174	46	38 (83)					

Footnote

Total and unbound dalbavancin concentrations at Day 22 (C22day) are expressed in μg/mL. Total and unbound area under the plasma concentration–time curve from Day 0 through Day 22 (AUC0–22days) are expressed in μg·h/mL. Exposure groups were defined by dichotomization at the specified cutpoints.

Confidence intervals were calculated using the Wald method.

Model 1: Inverse probability weights derived from logistic regression including age, body mass index (BMI), and a history of injection drug use.

Model 2: Inverse probability weights derived from logistic regression including age.

Model 3: Inverse probability weights derived from logistic regression including BMI.

Model 4: Inverse probability weights derived from logistic regression including a history of injection drug use.

© 2026 Lodise TP et al. *JAMA Network Open*.
