# Supplementary material for: Pharmacokinetics of Dalbavancin in Complicated Staphylococcus aureus Bacteremia: A Secondary Analysis of the DOTS Randomized Clinical Trial
Source: JAMA Netw Open. 2026 Apr 18;9(4):e2611652. doi: 10.1001/jamanetworkopen.2026.11652 (PMC13092111; doi:10.1001/jamanetworkopen.2026.11652)
Supplement: Supplement 4. — Nonauthor Collaborators. Antibacterial Resistance Leadership Group [file jamanetwopen-e2611652-s004.pdf]

***Group Name(s): Antibacterial Resistance Leadership Group (ARLG)**							
***First Name and Middle Initial(s)**	***Last Name**	***Suffix (eg, Jr, III)**	Academic Degrees	Institution	Location (city, state/province, country)	Role or Contribution, eg, chair, principal investigator	Group (if more than 1 Group listed in the byline) and/or Subgroup (eg, Steering Committee)
Henry F.	Chambers		MD	University of California - San Francisco	San Francisco, CA, USA	Chair	
Scott	Evans		PhD	George Washington University	Washington, DC, USA	Vice Chair	
Vance G.	Fowler	Jr	MD	Duke University	Durham, NC, USA		
Toshimitsu	Hamasaki		PhD	George Washington University	Washington, DC, USA		
Robin	Patel		MD	Mayo Clinic	Rochester, MN, USA		
Heather	Cross			Duke University	Durham, NC, USA		
Anthony	Harris		MD	University of Maryland	Baltimore, MD, USA		
Melinda	Pettigrew			University of Minnesota	MN, USA		
David	van Duin		MD PhD	University of North Carolina- Chapel Hill	Chapel Hill, NC, USA		
Helen	Boucher		MD	Tufts University	Boston, MA, USA		
Clayton	Huntley		PhD	NIH-Division of Microbiology and Infectious Diseases (DMID)	Bethesda, MD, USA		
Erica	Raterman		PhD	DMID	Bethesda, MD, USA		
Tamika	Samuel		PhD	DMID	College Park, MD, USA		
Kyung	Moon		PhD	DMID	Rockville, MD, USA		
Kim	Hanson		MD	University of Utah	Salt Lake City, UT, USA		
Yohei	Doi		MD PhD	University of Pittsburgh	Pittsburgh, PA, USA		
Thomas L.	Holland		MD	Duke University	Durham, NC, USA		
Tom	Lodise		PharmD, PhD	Albany College of Pharmacy and Health Sciences	Albany, NY, USA		
Ritu	Banerjee		MD PhD	Vanderbilt University	Nashville, TN, USA		
Sara	Cosgrove		MD	Johns Hopkins University	Baltimore, MD, USA		
David	Paterson		MBBS	National University of Singapore	Singapore		
Ebbing	Lautenbach		MD	University of Pennsylvania	Philadelphia, PA, USA		
Maureen	Mehigan			DMID	Rockville, MD, USA		
Sarah	Doernberg		MD	University of California - San Francisco	San Francisco, CA, USA		

***First Name and Middle Initial(s)**	***Last Name**	***Suffix (eg, Jr, III)**	Academic Degrees	Institution	Location (city, state/province, country)	Role or Contribution, eg, chair, principal investigator	Group (if more than 1 Group listed in the byline) and/or Subgroup (eg, Steering Committee)
Sam	Perdue		PhD	DMID	Rockville, MD, USA		
