# Supplementary material for: Pharmacokinetics of Dalbavancin in Complicated Staphylococcus aureus Bacteremia: A Secondary Analysis of the DOTS Randomized Clinical Trial
Source: JAMA Netw Open. 2026 Apr 18;9(4):e2611652. doi: 10.1001/jamanetworkopen.2026.11652 (PMC13092111; doi:10.1001/jamanetworkopen.2026.11652)
Supplement: Supplement 5. — Data Sharing Statement. [file jamanetwopen-e2611652-s005.pdf]

Data Sharing Statement

Lodise. Pharmacokinetics of Dalbavancin in Complicated *Staphylococcus aureus* Bacteremia: A Secondary Analysis of the DOTS Randomized Clinical Trial. *JAMA Netw Open*. Published online April 18, 2026. doi:10.1001/jamanetworkopen.2026.11652

Data
Additional Information: clinicaltrials.gov; NCT04775953 **Data available:** Yes
Data types: Deidentified participant data
How to access data: arlg_network@dm.duke.edu **When available:** With publication

Supporting Documents
Document types: None

Additional Information
Who can access the data: researchers whose proposed use of the data has been approved
Types of analyses: for the purpose(s) specified in their proposal
Mechanisms of data availability: with a signed data access agreement
